# Effects of Aging on Intrinsic Protein Disorder in Human Lenses and Zonules

**DOI:** 10.1007/s12013-024-01455-x

**Published:** 2024-08-08

**Authors:** Michael Antonietti, Colin K. Kim, Mak B. Djulbegovic, David J. Taylor Gonzalez, Jason A. Greenfield, Vladimir N. Uversky, Allister G. Gibbons, Carol L. Karp

**Affiliations:** 1 Bascom Palmer Eye Institute, University of Miami, Miami, FL USA; 2 Wills Eye Hospital, Thomas Jefferson University Hospital, Philadelphia, PA USA; 3 Department of Molecular Medicine and USF Health Byrd Alzheimer’s Research Institute, Morsani College of Medicine, University of South Florida, Tampa, FL USA

**Keywords:** Lens, Zonules, Intrinsically disordered proteins, bioinformatics, proteomics

## Abstract

This study aims to compare the levels of intrinsic protein disorder within the human lens and zonule proteomes and investigate the role of aging as a potential influencing factor on disorder levels. A cross-sectional proteomic analysis was employed, utilizing a dataset of 1466 proteins derived from the lens and zonule proteomes previously published by Wang et al. and De Maria et al. Bioinformatics tools, including a composition profiler and a rapid intrinsic disorder analysis online tool, were used to conduct a comparative analysis of protein disorder. Statistical tests such as ANOVA, Tukey’s HSD, and chi-squared tests were applied to evaluate differences between groups. The study revealed distinct amino acid compositions for each proteome, showing a direct correlation between aging and increased protein disorder in the zonular proteomes, whereas the lens proteomes exhibited the opposite trend. Findings suggest that age-related changes in intrinsic protein disorder within the lens and zonule proteomes may be linked to structural transformations in these tissues. Understanding how protein disorder evolves with age could enhance knowledge of the molecular basis for age-related conditions such as cataracts and pseudoexfoliation, potentially leading to better therapeutic strategies.

## Introduction

Intrinsically disordered proteins (IDP) are abundant in biological processes and have been previously described to be associated with ocular proteomes including the aqueous humor, tears, and vitreous [1–3]. Age-related conditions like cataracts and pseudoexfoliation are linked to structural changes in these tissues and are influenced by molecular processes [4–7]. However, the effect that age has on the disorder propensity of the lens and zonule proteomes remains unexplored. By analyzing proteomic data published by experimentalists [8, 9], we aim to understand how aging influences intrinsic protein disorder in these proteomes. We hypothesize that aging influences the intrinsic protein disorder in the lens and zonule proteomes. Our results show that age influences the overall propensity for intrinsic protein disorder in the lens and zonules in opposing ways. Age has a direct relationship with intrinsic protein disorder in the zonules and an inverse relationship with intrinsic protein disorder in the lens. These findings suggest that age-related changes in intrinsic protein disorder may play a crucial role in the structural and functional alterations observed in the lens and zonules, potentially contributing to the development of conditions such as cataracts and pseudoexfoliation.

The human eye lens is a biconvex, avascular, and transparent structure responsible for focusing light traversing the pupil onto the retina [5]. The lens is situated behind the iris and pupil in the posterior chamber and is held in place by suspensory ciliary zonules. The lens has a flexible composition formed by four distinct parts: the outermost lens capsule, a sub-capsular lens epithelium lining the anterior surface, lens fibers comprising the bulk of the lens, and a central nucleus with denser lens fibers [10]. Zonular fibers connect the lens capsule to the surrounding ciliary body, whereby contraction of the ciliary muscle allows for a structural change in the lens shape, a process known as accommodation [10]. Accommodation is a mechanical process in which the human eye alters its refractive power to optimally focus light on the retina. The optical clarity of the lens is maintained by a highly specialized population of lens fiber cells that, intriguingly, lack organelles such as mitochondria, endoplasmic reticulum, and nuclei, effectively reducing light scattering [11, 12]. These cells’ unique composition and arrangement are critical in maintaining lens transparency [11]. Previous studies have shown that loss of accommodation and lens clarity occur with increasing age [13, 14].

The lens is comprised of approximately 60% proteins, giving it the highest protein concentration of any tissue in the human body [15]. The lens is surrounded by a collagenous capsule that assists in allowing the lens to change shape through the process of accommodation. It is composed of type IV collagen, laminin, entactin, perlecan, type XVIII collagen, heparin sulfate proteoglycan, and fibronectin [16]. Crystallins comprise the major protein group found in the lens and are categorized into three major classes in humans: α-, β-, and γ-crystallins [17]. Crystallins comprise roughly 90% of the lens’s water-soluble proteins and are crucial in maintaining transparency [17]. These proteins are spatially regulated within the lens to create gradients of refractive indices and define the overall optical properties of the lens. Lens transparency relies on the conservation of the tertiary structure and solubility of these crystallin proteins, whereby protein misfolding and aggregation can result in cataract formation [6].

The associated zonular fibers are elastic structures primarily made up of microfibrils, with the main component being polymerized fibrillin [18]. Fibrillin-1 is the most abundant individual element of these fibers, which has been found to be significantly associated with Marfan syndrome [18]. Similar to changes observed in the structural protein environment seen in the lens with age, zonules experience changes to its protein foundation that manifest in increased fragility and potential for rupture during cataract surgery.

Intrinsically disordered proteins (IDPs) and intrinsically disordered protein regions (IDPRs) are distinguished from ordered proteins and domains/regions by the lack of a defined three-dimensional structure. Intrinsic disorder can be observed globally (e.g., lack of the overall three-dimensional structure of the protein) and at the local scale (e.g., lack of secondary structures like α-helices and β-sheets) [19]. Recent literature in the field corroborates that IDPs and IDPRs are acknowledged as some of the most versatile and often multifunctional proteins and protein sections playing a crucial role in various biological processes [20]. IDPs and IDPRs could also serve as promising leads for innovative drug development and biomarker identification due to their involvement in various diseases, such as cancers and neurodegenerative conditions [21, 22]. Therefore, the characterization of these protein elements provides a more profound understanding of the molecular dynamics governing the functionality of cells and other physiological systems [20]. To our knowledge, no previous research has characterized the presence of IDPs or IDPRs in the human lens or zonular fibers.

Our group has characterized the intrinsic disorder of the aqueous humor [1], the tear film [2], and the vitreous humor proteomes [3]. Using a similar methodology, this study aims to analyze the presence of IDPs and IDPRs in the fiber lens proteome. We focused on the 951 proteins previously identified in the human lens fiber cell membranes proteome by Wang et al., the most extensive set of proteins identified in the human lens proteome reported to date [9]. Furthermore, for the analysis of the zonular proteome, we utilized the data identified by De Maria et al., which found 279 distinct proteins in the zonules [8].

The primary aim of the study is to use bioinformatics tools and methods to measure and categorize the extent of intrinsic disorder in the human lens and zonular fiber proteomes. Secondly, we examined the effect that age may have on each tissue proteome’s propensity for protein disorder. As we have in prior studies for other ophthalmic-associated proteomes, we aim to utilize a similar computational methodology to identify the protein disorder level of the lens and zonular proteomes [1–3]. We specifically examined the intra and inter-group differences of intrinsic protein disorder profiles of the younger lens proteome, the older lens proteome, and the zonular proteome. By quantifying the levels of intrinsic disorder in these proteomes and measuring the observed differences in age groups, we aim to advance the understanding of the molecular interactions that underlie the anatomical function of these tissues. Our study could provide a foundation for additional proteome studies that could examine how the disorder propensity varies in many ocular diseases, including but not limited to cataracts, presbyopia, and pseudoexfoliation.

## Methods

### Proteome Protein Identification

Our study aimed to compare the propensity of intrinsic disorder of three distinct proteomes: lenses of three different ages and the proteome of human zonules. The proteome of the three different aged lenses was obtained from a study by Wang et al. In their study, they halved, decapsulated, and processed three human lenses (aged 25, 37, and 58 years) and identified 951 distinct human membrane proteins in these samples [9]. Importantly, the age 25 and 37 were reported in one group by Wang et al. and age 58 was reported as its own group. From this point forward, we will consider the “younger” lens proteome as proteins from the combined age 25 and age 37 protein set and the “older” lens proteome as the age 58 protein set. For the proteome of the human zonules, De Maria et al. obtained samples from 10 different patients (total of 16 eyes) and identified 357 proteins in the human zonule [8]. The 357 proteins included redundancies and contaminants removed from the data set, leaving 279 zonular proteins that we included in our study and defined as the “zonular” proteome.

To analyze these sets of proteins, we used their identifiers to retrieve amino acid sequences. The NCBI Reference Sequence accession identifiers were inputted into the Universal Protein Resource Knowledgebase (UniProt, available at: https://www.uniprot.org/) for mapping to UniProt IDs [23]. Proteomes of the 25- and 37-year-old lenses were reported together by Wang et al. and therefore were analyzed as one group in our study (referred to as “younger” lenses). Of the 951 identifiers for the 25- and 37-year-old lens proteome, 927 were successfully matched, resulting in 926 UniProt IDs. For the 58-year-old lens (referred to as “older” lens) patient, there were 260 identifiers, resulting in 261 UniProt IDs. For the zonules, the 279 identifiers resulted in 279 UniProt IDs. The corresponding protein sequences were downloaded from UniProt in FASTA format and were the primary input for our downstream analysis.

### Proteome Amino Acid Composition Analysis

We utilized the Composition Profiler (accessible at: http://www.cprofiler.org/) to analyze the proteomes of the younger lenses, the older lens, and the human zonules [24]. According to De Maria et al., the zonules were grouped into pools to control for number of eyes and sex, however these pools were not controlled for age. Taking advantage of this, we evaluated the mean age of each pool. We grouped Pool A and Pool D as the younger zonules they had a mean age of 38.5, while Pool B and Pool C were grouped as the older zonules as this pool had a mean age of 55.25. The Composition Profiler tool quantifies and visualizes individual amino acid abundance or scarcity within each proteome. The lens and zonule proteomes in FASTA format served as the query sets, with the ‘Protein Data Bank Select 25’ as the background set. To facilitate comparison, composition profiles were generated for the DisProt database, a repository of proteins validated to be disordered, and the SwissProt database, proteins that represent amino acid distribution in nature. Amino acids were evaluated based on their tendency to promote either order or disorder, with positive values indicating enrichment and negative values indicating depletion within the proteome. The ten order-promoting residues are C, W, I, Y, F, L, H, V, N, and M, and the ten disorder-promoting residues are R, T, D, G, A, K, Q, S, E, P. The normalized enrichment or depletion of a specific residue is calculated as (C_x_–C_order_)/C_order_, where C_x_ represents the content of the residue in the query protein, and C_order_ is the content of the same residue in the PDB Select 25.

### Analysis of Intrinsic Disorder in Different Aged Lenses and Zonule Proteomes

#### Prediction of Disorder Using Commonly Used Predictors

In the subsequent phase of our study, we assessed the degree of intrinsic disorder at the level of individual residues using various predictors specific to amino acid residues. These predictors were accessed through the Rapid Intrinsic Disorder Analysis Online (RIDAO) platform (available at https://RIDAO.app) [25]. Average Disorder Scores (ADS) and Percentages of Predicted Disordered Residues (PPDR) were computed for each protein, employing the Predictor of Natural Disordered Regions (PONDR^®^) VSL2, PONDR^®^ VL3, PONDR^®^ VLXT, PONDR^®^ FIT, and IUPred Short and Long. ADS measures the average disorder for a protein, and PPDR measures the proportion of amino acids within a protein that have a predicted disorder score above 0.5.

Next, we performed ANOVA (Analysis of Variance) statistical tests to assess the statistical significance of differences in ADS and PPDR across the three groups (younger lenses, older lenses, and zonules). These ANOVA tests helped determine whether the observed disparities in protein disorder among the younger lenses, the older lens, and the human zonules were statistically meaningful or merely random. It is important to note that in this initial analysis, we did not differentiate the zonules into separate age groups. This was primarily because, unlike the lens proteomes, the zonular samples were not initially reported as distinct sets categorized by age, necessitating a uniform approach in their comparative analysis. This methodological choice will be revisited and expanded upon in subsequent sections of our study as we further quantify age-related variations within the zonular proteomes.

#### Average Disorder vs Percent of Predicted Disorder Residues Analysis

Continuing our per-residue analysis, we adopted an established technique from our previous works, focusing on the PONDR^®^ VSL2 from the RIDAO output [25, 26]. PONDR^®^ VSL2 is designed to assess each amino acid for protein disorder, and its effectiveness has been proven in the Critical Assessment of Protein Intrinsic Disorder [27]. We used PONDR^®^ VSL2 outputs as the primary data, defining specific cut-off points to categorize each protein by its disorder status. We defined proteins as highly ordered if they had a PPDR of less than 10% or an ADS of less than 0.15. Proteins with 10% to less than 30% PPDR or 0.15 to less than 0.5 ADS were considered moderately disordered. Proteins with a PPDR of 30% or more and an ADS of 0.5 or more were labeled as highly disordered. These categorizations are consistent with the standards set in our previous publications [2, 3, 28, 29]. This stratification allows for a more detailed study of protein structures by clearly identifying varying levels of structural organization. To further clarify these metrics, ADS measures the average disorder score, a specific disorder predictor for each protein, while PPDR indicates the fraction of amino acids in a protein that is likely disordered (i.e., scores exceeding 0.5). It is important to acknowledge that ADS does not share a direct relationship with PPDR. As a result, we must consider ADS independently in evaluating proteins, categorizing them as highly ordered if ADS is below 0.15, as moderately disordered if ADS falls between 0.15 and 0.5, and as highly disordered if ADS reaches 0.5 or higher.

The process described uses the predefined thresholds of protein disorder measurements to plot each protein on a two-dimensional graph, where the y-axis represents ADS and the x-axis represents the PPDR. By applying these cut-offs, the graph is divided into quadrants that categorize proteins into highly ordered (PPDR < 10% or ADS < 0.15), moderately disordered (10% ≤PPDR < 30% or 0.15 ≤ ADS < 0.5), or highly disordered (PPDR ≥ 30% and ADS ≥ 0.5), allowing for visual stratification and easier comparison of proteins based on their structural organization. To perform a comparative analysis of the protein distribution across the different structural classes defined by the quadrant system on the graph, we employed a chi-squared test. This statistical test was utilized to assess the significance of the observed differences in protein categorization between the quadrants. By comparing the expected frequencies of protein classification under the assumption of no association with the observed frequencies in the data, the chi-squared test determines whether the deviations are due to random chance or indicate a significant pattern in protein disorder across the quadrants.

### CH-CDF Plot Analysis

For further analysis, we utilized two binary predictors of disorder: the charge-hydropathy (CH) and the cumulative distribution function (CDF) to assess the intrinsic disorder of the entire protein for the three different proteome groups. Rather than measuring disorder at the individual amino acid residue as in previous steps, we measure disorder as binary (i.e., yes or no; disordered or ordered; unstructured or structured). The CH factors in each protein’s net charge and hydrophobicity determine its level of disorder. Compared to ordered proteins, disordered proteins often have a lower hydropathy and higher net charge [30]. The CDF describes the cumulative frequency of disordered proteins along the length of a given protein. For this study, we integrated the CH and CDF metrics to create a CH-CDF plot on which every protein in the three proteome groups was graphed.

With this technique, we were able to define a structured vs an unstructured protein based on where it fell on the plot. The CH-CDF plot’s Cartesian coordinate system allows for qualitative classification and nuanced analysis of each protein’s structural attributes within a two-dimensional framework. Quadrant 1 (Q1, bottom right) encompasses proteins that are likely structured, characterized by a negative CH score and a positive CDF score indicating order. Quadrant 2 (Q2, bottom left) comprises proteins that are either molten globular or hybrid, with both CH and CDF scores being negative, suggesting these proteins are compact yet lack a distinctive 3D structure or contain noticeable levels of ordered and disordered residues. Quadrant 3 (Q3, top left) includes highly disordered proteins with positive CH scores and negative CDF values. Finally, Quadrant 4 (Q4, top right) captures proteins that are predicted to be disordered according to the CH plot yet ordered according to the CDF plot [30, 31]. Utilizing the chi-squared test once more, we assessed the distribution of proteins within this CH-CDF quadrant system to identify significant differences, operating under the same foundational assumptions as our prior chi-squared analyses.

### Normalization of Disorder Propensity Scores in Human Zonular Proteome Pools

In previous steps, we treated the zonules as one group when comparing the proteome of the zonules to both the proteomes of the younger and older lenses. In this prior analysis, we did not separate the zonule proteins into distinct age groups because they did not report separate protein for the various age groups. However, to delve into the age-related changes of the zonules, the next phase of our analysis incorporated protein expression data between groups of zonular proteomes. As per the data provided by De Maria et al., human zonular samples from 10 donors aged 23 to 66 years were categorized into four pools, as described in Table 1 of their manuscript [8]. Each pool was designed to encapsulate a broad demographic range, incorporating samples from both male and female donors to minimize individual variability. Pool A included samples from donors 1, 5, and 8. Pool B consisted of samples from donors 3 and 6. Pool C comprised samples from donors 4, 7, and 9. Pool D combined samples from donors 2 and 10. Each group consisted of four eyes, and the average ages of the donors for each pool were as follows: Pool A (39 years), Pool B (51.5 years), Pool C (60 years), and Pool D (38.5 years).

**Table 1:** Comparative analysis of disorder predictors across lens ages and zonules

	Younger lens	Older lens	Zonules	ANOVA F-statistic	ANOVA P value
PPDR-VLXT	29.66%	26.46%	30.95%	5.32	<0.01^a^
ADS-VLXT	0.33	0.3	0.35	7.21	<0.01^a^
PPDR-VSL2B	32.15%	28.51%	40.36%	24.09	<0.0001^a^
ADS-VSL2B	0.42	0.39	0.47	25.59	<0.0001^a^
PPDR-VL3	24.54%	19.6%	32.55%	20.81	<0.0001^a^
ADS-VL3	0.34	0.31	0.4	23.63	<0.0001^a^
PPDR-IUP-Short	13.85%	11.45%	16.3%	7.16	<0.01^a^
ADS-IUP-Short	0.25	0.24	0.28	13.22	<0.0001^a^
PPDR-IUP-Long	13.77%	11.25%	17.42%	7.69	<0.01^a^
ADS-IUP-Long	0.27	0.26	0.32	14.26	<0.0001^a^
PPDR-PFIT	20.46%	16.25%	23.41%	10.11	<0.0001^a^
ADS-PFIT	0.3	0.27	0.33	13.29	<0.0001^a^
PPDR-MDP	17.87%	13.81%	21.6%	9.18	<0.001^a^
ADS-MDP	0.3	0.27	0.34	17.11	<0.0001^a^

The table shows the Average Disorder Scores (ADS) and Percentages of Predicted Disordered Residues (PPDR) of each of the six disorder predictors and the statistical differences in these values among the younger lens, the older lens, and the human zonules proteomes. See methods for description of how the proteomes are defined

*PPDR* Percentages of Predicted Disordered Residues, *ADS* Average Disorder Scores, *MDP* Mean Disorder Profile

^a^Statistically significant, p value < 0.05

We calculated the normalized disorder propensity score for each pool by weighting the disorder score of each protein (Mean Disorder Profile Score) by its expression level and then normalizing these weighted scores by the total protein expression within the pool. This yielded a disorder score ranging from 0 to 1 for each pool, reflecting the relative disorder within the proteome of that particular sample pool. These normalized scores facilitate a comparison of the propensity for protein disorder across pools with varying levels of protein expression, offering insights into the structural characteristics of the zonular proteome associated with age and sex demographics within the sampled populations.

## Results

### Proteome Protein Identification

We analyzed 1466 proteins identified in the lens and zonules. We divided our analysis into three distinct groups, which we treated as separate entities: Group 1 comprised lenses from individuals aged 25 and 37, containing 926 proteins (referred to as younger lenses); Group 2 consisted of lenses from a 58-year-old, with 261 proteins (referred to as older lens); and Group 3 included zonules with 279 proteins (referred to as zonules). We utilized this nomenclature for our ensuing comparative studies.

### Proteome Amino Acid Composition Analysis

**Fig. 1:**
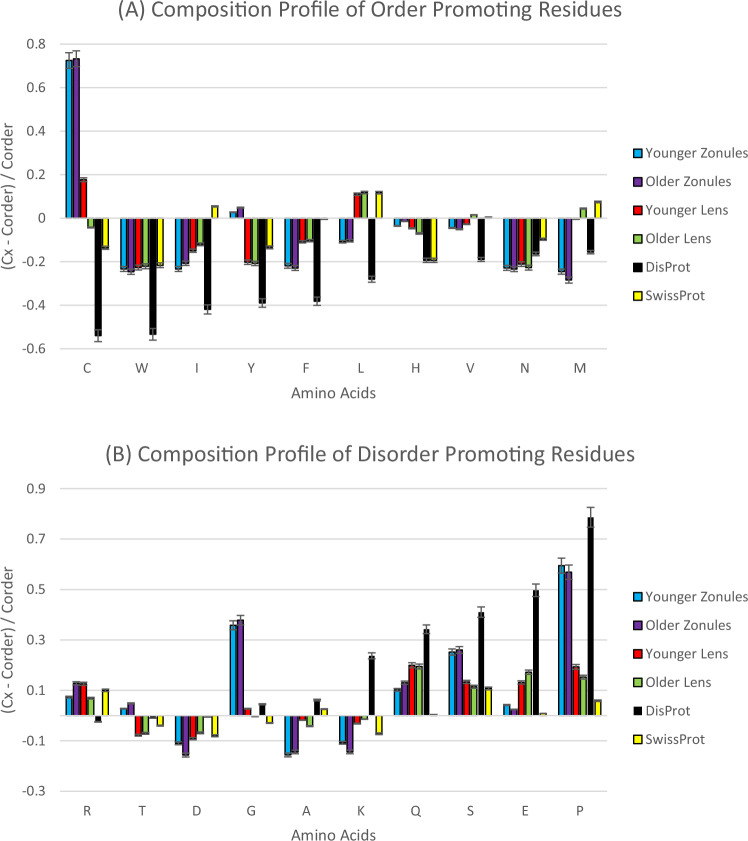
Amino acid composition profile of younger zonules (blue), older zonules (purple), younger lens (red bar), and older lens (green bar). The fractional difference is calculated as (C_x_ – C_order_)/C_order_, where C_x_ is the content of a given amino acid in the query set (243 younger zonules, 212 older zonules, 926 younger lens, and 261 older lenses of human lens proteins or proteins from the SwissProt database), and C_order_ is the content of a given amino acid in the background set (Protein Databank Select 25). The amino acid residues are ranked from most order-promoting residue to most disorder-promoting residue. Positive values indicate enrichment and negative values indicate depletion of a particular amino acid. The composition profile generated for experimentally validated disordered proteins from the DisProt database (black bars) and the distribution of amino acids in nature from the SwissProt database (yellow bars) is shown for comparison. The error bars correspond to standard deviations over 10,000 bootstrap iterations. The composition profile analysis showed 2/10 order-promoting residues (C and L) in the younger lenses enriched (p value < 0.05). The composition profile analysis showed 3/10 order-promoting residues (L, V, and M) in the older lens enriched (p value < 0.05). In the zonules, there was enrichment (p value < 0.05)t of one order promoting residue (C). The older lens and the zonules each showed 1/10 disorder-promoting amino acid residues (L and C), respectively, were enriched (p value < 0.05). The older lens showed enrichment of (R, Q, S, E, and P) with a (p value < 0.05). The younger lenses showed enrichment of six residues (R, G, Q, S, E, P) in the disorder-promoting amino acids (p value < 0.05). The zonules showed enrichment in the same residues of the younger lenses in addition to threonine, with seven total being (R, T, G, Q, S, E, and P) with a (p value < 0.05)

The amino acid composition of the 926 proteins of the younger lenses, 261 proteins of the older lens, and 279 proteins in the zonules were analyzed and compared through visualization with the experimentally validated protein composition profile of the DisProt database and the natural distribution of amino acids from the SwissProt database (Fig. 1). The amino acids were ranked according to their propensity to promote either order or disorder. Amino acids were ordered based on their likelihood to foster structure/order or lack of structure/disorder, where positive values denote an enrichment and negative values figures denote a depletion of an amino acid. Of the ten order-promoting residues in the younger lenses (C, W, I, Y, F, L, H, V, N, and M), only two (C and L) showed enrichment. However, in the older lens, three order-promoting amino acids were enriched (L, V, M). When analyzing the order-promoting residues, the zonule proteins showed enrichment only two (C and Y). There was agreement in enrichment and depletion across all order-promoting residues for the younger and older zonules. In the older lens, five (R, Q, S, E, and P) out of the ten disorder-promoting residues (R, T, D, G, A, K, Q, S, E, P) showed enrichment. The younger lenses show enrichment of six disorder-promoting amino acid residues (R, G, Q, S, E, P). The zonule had enrichment of seven amino acids (R, T, G, Q, S, E, and P) in the ten disorder-promoting residues. Furthermore, there was a consistent pattern of enrichment and depletion of disorder-promoting residues in both the younger and older zonules.

### Analysis of Intrinsic Disorder within Lens and Zonular Proteomes

#### Prediction of Disorder Using Commonly Used Predictors

The subsequent aim of our research was to measure and contrast the disorder tendencies at the amino acid level among the three categorized sets. These sets were defined according to proteins exclusive to and common between the lens proteins from the younger lenses, lens proteins from the older lens, and proteins from the zonules. For this portion of our analysis, the protein expression and comparison are considered one-to-one.

We employed the RIDAO tool to obtain measures of disorder for subsequent comparative evaluation of disorder profiles, utilizing two separate scoring metrics, the PPDR and ADS, for each type of disorder predictor, as noted in Table 1. The analysis was conducted on predictions of intrinsic disorder for two different lens age groups and the zonules by using an array of per-residue amino acid predictors. When the PONDR^®^ VLXT predictor was used, the PPDR for the younger lenses, older lenses, and zonules fluctuated between 26.46% and 30.95%. Simultaneously, the ADS spanned from 0.305 to 0.348. The ANOVA test produced F-statistics of 5.32 and 7.21 with P-values of < 0.01 for the PPDR and ADS metrics from PONDR^®^ VLXT, signifying a statistically significant variation.

The PONDR^®^ VSL2 algorithm reveals a range in PPDR values from about 28.51% to 40.35%, and ADS values fluctuate between 0.393 and 0.473. Statistically notable disparities are shown via ANOVA, where F-statistics stand at 24.09 and 25.59 with P values of <0.0001 for PPDR and ADS. The PONDR^®^ VL3 model produces PPDR figures ranging from 19.60% to 32.55%, and ADS from 0.306 to 0.398, accompanied by ANOVA F-statistics of 20.81 and 23.63, and P values of 1.23 × 10^−9^ and 7.95 × 10^−11^, indicating significant variances across different age groups of lenses and zonules in estimating protein disorder.

For IUPred Short (IUP_S), PPDR is reported between 11.45% and 16.29%, with ADS from 0.237 to 0.2800. ANOVA tests reveal significant differences for PPDR and ADS, with F-statistics of 7.16 and 13.22 and P-values of <0.01 and <0.0001, respectively.

IUPred Long (IUP_L) presents PPDR values from 11.25% to 17.42% and ADS between 0.258 and 0.316. ANOVA shows significant differences for PPDR with an F-statistic of 7.69 and a P value of 0.000475 and for ADS with an F-statistic of 14.26 and a P value of 7.39 × 10^−7^. The PONDR^®^ FIT model yields PPDR figures from 16.25% to 23.41% and ADS from 0.271 to 0.330, showing significant differences in ANOVA for both measures.

Finally, the PONDR^®^ MDP model indicates PPDR values from 13.81 to 21.60% and ADS between 0.273 and 0.341, with ANOVA highlighting significant differences as evidenced by F-statistics of 9.18 and 17.11, and P-values of <0.001 for PPDR, and <0.0001 for ADS.

The significance found via ANOVA led us to implement the Tukey HSD test to further investigate the specific differences between each pair of groups. For an extended statistical examination of these intrinsic disorder predictors across the three groups, see Table S1 in the Supplementary Files. The table contains data related to post-hoc analysis that utilizes Tukey’s HSD tests for pairwise comparisons and details the mean differences, confidence intervals, and the significance levels of these differences between lenses of different ages and zonules. The comparison between younger and older lens groups showed that 10 out of 14 predictors showed significant differences, rejecting the null hypothesis. Taken together with our analysis in Table 1, these findings suggest that the younger lens proteome is predicted to be more disordered then the older lens proteome. 11 out of 14 predictors supported rejecting the null hypothesis when comparing the younger lenses to the zonules. Again, when considering Table 1, the data suggest that the younger lens proteome is less disordered then the zonule proteome. All 14 predictors suggested rejecting the null hypothesis when comparing the older lens and the zonules. Ultimately, our data suggest that the older lens proteome is also less disordered then the zonule proteome. Taken together, these findings suggests that the significant variances highlighted by the ANOVA can be attributed to the pairwise differences between groups. In summary, the younger lens proteome is more disordered then the older lens proteome and both lens proteomes are less disordered then the zonule proteome.

#### Average Disorder vs Percent of Predicted Disorder Residues Analysis

**Fig. 2:**
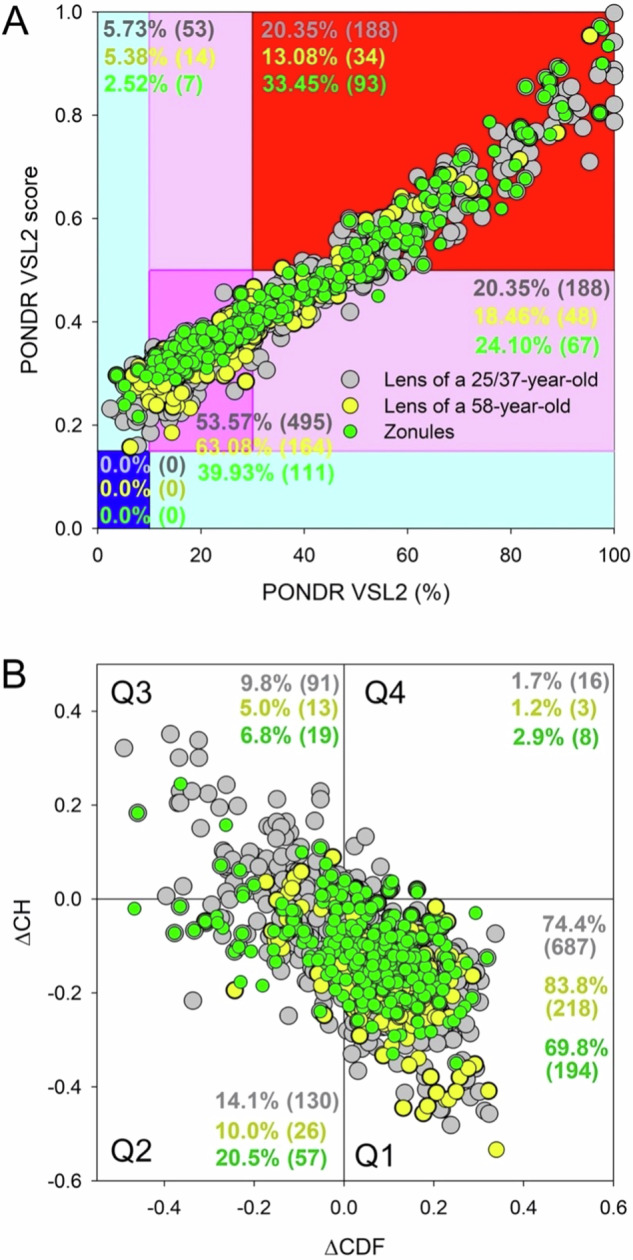
Multidimensional Protein Disorder Analysis in Lens and Zonular Proteins. **A** PONDR^®^ VSL2 Score vs. VSL2 PONDR^®^ (%) analysis showing younger lens, older lens, and zonular proteins with general agreement between each group. PONDR^®^ VSL2 (%) is a percent of predicted disordered residues (PPDR), i.e., residues with disorder scores above 0.5. Of the proteins analyzed, 188 (20.35%) younger lens proteins, 34 (13.08%) older lens proteins, and 93 (33.45%) zonular proteins are predicted to be highly disordered. PONDR^®^ VSL2 score is a protein’s average disorder score (ADS). Color blocks indicate regions in which proteins are mostly ordered (blue and light blue), moderately disordered (pink and light pink), or mostly disordered (red). If the two parameters agree, the corresponding part of the background is dark (blue or pink), whereas light blue and light pink reflect areas where the predictors disagree with each other. The boundaries of the colored regions represent arbitrary and accepted cutoffs for ADS (y-axis) and the percentage of predicted disordered residues (PPDR; x-axis). **B** Charge-Hydropathy and Cumulative Distribution Function (CH-CDF) analysis of three group proteins categorized by younger lens, older lens, and zonules. The CH-CDF plot is a two-dimensional representation that integrates both the CH plot, which correlates a protein’s net charge and hydrophobicity with its structural order, and the CDF, which accumulates disorder predictions from the N-terminus to the C-terminus of a protein, offering insight into the distribution of disorder residues. The Y-axis (ΔCH) represents the protein’s distance from the CH boundary, indicating the balance between charge and hydrophobicity, while the X-axis (ΔCDF) represents the deviation of a protein’s disorder frequency from the CDF boundary. Proteins are then stratified into four quadrants: Quadrant 1 (bottom right) indicates proteins likely to be structured; Quadrant 2 (bottom left) includes proteins that may be in a molten globule state or lack a unique 3D structure; Quadrant 3 (top left) consists of proteins predicted to be highly disordered; Quadrant 4 (top right) captures proteins that present a mixed prediction of being disordered according to CH but ordered according to CDF

Next, we utilized PONDR® VSL2 percents and scores to further our analysis of the propensity for intrinsic protein disorder in the younger and older lenses and zonules (Fig. 2). Of the younger lenses, 376 were classified as highly disordered (ADS > 0.5 or PPDR > 30%), making up a considerable proportion of the proteins (40.2%). For the older lens, 82 proteins (31.54%) were similarly categorized as highly disordered. The zonules were found to have a notable quantity of proteins with substantial disorder, with 160 proteins (57.55%) affected. Additionally, 111 proteins (39.93%) in the zonules were deemed as moderately disordered or somewhat flexible (ADS > 0.15 and 10% < PPDR < 30%). Moreover, a smaller subset of proteins—53 in the younger lenses (5.73%), 14 in the older lens (5.38%), and 7 in the zonules (2.52%)—were identified as moderately ordered or slightly flexible, signifying a lesser segment of each proteome.

Statistical analysis of these quadrant distributions using the chi-squared test showed significant variations in how proteins were categorized within the younger lenses, older lens, and zonular layers (Chi-squared value of 41.49, P value < 0.0001, with 4 degrees of freedom (DoF)). This underscores a non-random distribution of protein disorders that may be attributed to biological factors related to age or function. The DoF = (r-1) × (c-1), where the c represents columns and r represents rows. The three rows are the different groups of proteins: the younger lens, the older lens, and the zonules. The columns are the three distinct classifications: highly ordered (PPDR < 10% or ADS < 0.15), moderately disordered (10% ≤ PPDR < 30% or 0.15 ≤ ADS < 0.5), or highly disordered (PPDR ≥ 30% and ADS ≥ 0.5). Thus, the product of the differences in the calculation provided a final DoF value of 4.

### CH-CDF Plot Analysis

Using the CH-CDF analysis, which categorized ocular proteins by the younger lens, older lens, and zonules, a total of 1462 proteins were distributed into four quadrants according to their predicted disorder characteristics. The younger lens proteome analysis showed that 687 proteins (74.4%) had a structured state assigned to Quadrant 1 (Bottom Right). Quadrant 2 (Bottom Left), with 130 proteins (14.1%), corresponded to proteins in a molten globule state or lacking a distinct 3D structure. Quadrant 3 (Top Left) included 91 proteins (9.8%) associated with a high disorder or lack of structure, while Quadrant 4 (Top Right) had 16 proteins (1.7%) showing a mix of ordered and disordered predictions. For the older lens proteome, 218 proteins (83.8%) in Quadrant 1 (Bottom Right) indicative of a structured state. Quadrant 2 (Bottom Left) contained 26 proteins (10.0%) characterized as molten globules, and Quadrant 3 (Top Left), with 13 proteins (5.0%), signified a high level of disorder. Quadrant 4 (Top Right) had three proteins (1.2%) with mixed structural characteristics. In the zonule proteome, 194 proteins (69.8%) found in Quadrant 1 (Bottom Right) were suggestive of structure. Quadrant 2 (Bottom Left) included 57 proteins (20.5%) identified as molten globules. Quadrant 3 (Top Left) had 19 proteins (6.8%) with significant disorder, and Quadrant 4 (Top Right) had eight proteins (2.9%) indicating mixed structural predictions.

A chi-squared test was conducted to evaluate the distribution of proteins across the different structural classes in the CH-CDF plot. The results, with a chi-squared value of 23.392 and a P value < 0.001 for 6 degrees of freedom, demonstrated a significant discrepancy from the expected distribution in the quadrants. As mentioned above, the degrees of freedom were calculated utilizing DoF = (r-1) × (c-1), with r denoting rows on a table and c as columns on a table. The three rows are the different groups of proteins: the younger lens, older lens, and zonule proteins. The columns are the four distinct quadrants: Q1, Q2, Q3, and Q4. The product of the differences between these values when applying the formulas gives a final product of 6 degrees of freedom. This significant difference indicates that the protein distribution pattern across the quadrants is not due to random variation, suggesting distinctive differences in the structural states of the proteins within the younger lenses, older lens, and zonules compared to what would occur by chance.

### Normalization of Disorder Propensity Scores in Human Zonular Proteome Pools

In the study of human zonular samples, normalized disorder scores were calculated to incorporate protein expression while assessing the propensity for protein disorder within each sample pool, as shown in Table 2. The analysis yielded a normalized disorder score of 0.323 for Pool A, which contained samples from an average donor age of 39 years. Pool B, with a mean donor age of 51.5 years, had a normalized disorder score of 0.310. Pool C, which had the highest average age of donors at 60 years, showed a slightly higher propensity for protein disorder with a score of 0.353. Pool D, with a mean donor age of 38.5 years, equal to Pool A, also had a normalized disorder score of 0.310. Due to our methodology yielding a singular, composite normalized disorder score for each pool, we are presenting these values as fixed indicators rather than ranges. This approach reflects a summary measure of the average propensity for disorder in each pool’s proteome, distilled into a single metric. As such, our analysis focuses on direct comparisons of these aggregate scores to elucidate differences in structural characteristics across the pools, without the variability ranges typically associated with multiple independent observations.

**Table 2:** Age-related zonular disorder analysis

Zonular analysis	Pool A	Pool B	Pool C	Pool D
Mean Age of Pool (years)	38.5	50.5	60.0	38.5
Normalized Disorder Score	0.323	0.310	0.353	0.31

The table shows the mean age of each pool in years and the normalized disorder score of each of the four zonular pools. The normalized disorder scores take into account protein expression data as previously reported. See methods for more details

## Discussion

The research presented here provides a new perspective on the possible role of intrinsic protein disorder in three distinct human tissues: the younger lens, the older lens, and the zonules. Similar to our earlier investigations of the tear film [2], the aqueous humor [29], and the vitreous humor proteomes [3], this study leverages bioinformatics to map out the intricate landscape of protein disorder in the human lens and zonules. Our research goes beyond the conventional limits of proteomic studies that focus mainly on identifying and quantifying proteins. We evaluated 1466 proteins within these biological specimens and introduced an additional conceptual framework for comparatively analyzing the disorder profiles of lens and zonule proteomes. Our study found a unique amino composition profile between proteomes and significant differences in the intrinsic disorder profiles of these two tissues. High cysteine levels in young lens and zonular proteomes may contribute to the flexibility of these structures while maintaining their structural integrity, whereas proline enrichment of zonules may facilitate their elasticity. We found that the younger lens proteome had a higher propensity for intrinsic protein disorder compared to the older lens proteome. In contrast, we found that the zonular proteome had a higher propensity for intrinsic protein disorder in the older zonules when compared to the younger zonules. The varying degrees of protein disorder in the lens and zonular proteomes, influenced by aging, may be linked to the structural alterations observed in the lens and zonular fibers as they age. This variation in protein disorder could provide new perspectives on the molecular foundations of age-related eye conditions, including but not limited to cataracts, presbyopia, and pseudoexfoliation.

The amino acid composition analysis of lens and zonular proteomes indicates a distinctive, non-random arrangement that may be aligned with each proteome’s unique structural and functional demands. In the younger lenses and zonules, there is a notable presence of cysteine, which may have a role in preserving the structural integrity of these structures while providing them the ability to undergo anatomical structure changes involved in ocular biology and physiology. Conversely, the reduced presence of cysteine in the older lens proteome suggests a possible connection to age-related diseases such as cataracts and presbyopia. Moreover, the zonules are particularly rich in the disorder-promoting residue proline, indicating the highest concentration among all amino acids examined, which suggests a propensity for structural disorder. This may be attributable to fibrillin, a protein abundant in the zonules and known to be enriched with proline [32]. Fibrillin mutations are associated with various pathologies, especially Marfan syndrome. In such cases, the defective fibrillin cannot act as a scaffold for elastin, compromising the structural integrity of blood vessels and other tissues [33]. Furthermore, the proportion of disordered amino acids differed significantly across the younger lens, older lens, and zonules, potentially mirroring age-associated transformations within the lens and the distinct functional contributions these amino acids may have for the zonules.

An intriguing observation was the relative enrichment of the amino acid lysine in older lenses compared to younger ones. This increase in lysine levels suggests its potential involvement as a precursor residue for glycation reactions. Conversely, the relative levels of arginine exhibited a decreasing trend from younger to older lenses. Notably, studies have demonstrated arginine’s remarkable ability to block advanced glycation reactions, both in vitro and in vivo [34]. Arginine is the major crystallin residue susceptible to damage by glycation during the aging process of the lens. These findings further reinforce the understanding of the Maillard reaction, a type of glycation process. In this reaction, lysine serves as a precursor, leading to the formation of advanced glycation end products (AGEs) [35]. The accumulation of AGEs plays a crucial role in cataract formation by causing the lens to stiffen and brown over time [36]. In summary, while lysine levels increase and potentially contribute to glycation reactions, arginine levels, which potentially block these glycation reactions decrease with age. The increasing prevalence of cataracts with age may help explain our findings that lysine had greater enrichment in the older lenses than the younger lenses. In addition, arginine’s ability to inhibit advanced glycation reactions and its depletion with age positions it as a potential therapeutic target for preventing or delaying cataract development associated with lens stiffening and browning.

While the relationship with age-related variations in disorder profiles is contrary when comparing the association in the lens proteome to the zonule proteomes, these discrepancies could be attributable to changes in function and structure that the lens and zonules undergo as individuals grow older. The zonules had the most intrinsic protein disorder of the three proteomes, followed by the younger lens, and finally, the older lens, which had the least disorder. Amongst the zonular proteome pools analyzed, the oldest pool had the highest percent of intrinsic protein disorder. These results underscore the comparative differences in intrinsic disorder profiles among different proteomes of tissue types and how they may be affected by the aging process.

The PONDR® quadrant analysis suggests that there is a diverse array of protein structures across the young and old lens proteomes and the zonules, indicative of dynamic protein networks. By PONDR® analysis, the proteomes are predicted to consist of a spectrum of proteins that range from highly structured to mildly flexible to moderately disordered to somewhat flexible to highly disordered proteins. The CH-CDF quadrant analysis was in agreement with the PONDR analysis in that it predicts that the lens and zonular proteome consist of a spectrum of proteins. The CH-CDF analysis identified an array of proteins in each proteome that were either highly structured proteins, adaptable molten globules, or highly disordered proteins. The various distributions of these protein classifications within the lens and zonular proteomes may have evolved to meet the distinct mechanical and optical demands of the ocular system, thereby supporting the complex functions required for maintaining eye health and visual acuity.

The observed decline in intrinsic disorder within the younger lens as compared to the older lens can be indicative of the progressive age-associated changes occurring in the visual system. For example, presbyopia is a condition commonly associated with aging, where the lens of the eye becomes less flexible and its elasticity diminishes, leading to difficulties in focusing on close objects [37, 38]. This condition is a natural part of the aging process and can begin to affect people as early as their mid-40s. Furthermore, the considerable levels of intrinsic protein disorder noted in the younger lens might imply a preserved functional capacity for accommodation, which is the eye’s ability to adjust its focus on near and distant objects. This flexibility is critical for maintaining sharp vision across different distances and is affected by structural changes within the lens fiber cells that are likely regulated by its proteome [37]. While our research presents these relationships within the 25 to 60-year-olds, it is possible that similar age-related ocular changes could be observed beyond this range.

Furthermore, the increase in the proteome-wide disorder score could reflect the physiological or pathological processes affecting the eye’s zonular fibers with advancing age. These changes may be associated with alterations in the mechanical properties of the zonule that occur with age, such as pseudoexfoliation syndrome due to the accumulation of fibular material, thus leading to increased risks of lens dislocation and secondary glaucoma [7]. Another ocular phenomenon that may be implicated in the older zonule is age-related zonular weakening, leading to lens instability and complications during ocular surgery [39]. The zonules notably facilitate the lens’s accommodation, altering its curvature through the application or relaxation of tension at the lens’s equator [40]. They also secure the lens to the ciliary body and are affected by conditions such as pseudoexfoliation syndrome and retinitis pigmentosa, which can lead to zonular instability [41]. These fluctuations in intrinsic disorder, particularly the increased levels found in older zonules, may explain the observed conditions that lead to zonular instability and contribute to zonulopathy.

Ultimately, the change in the level of intrinsic protein disorder within the lens and zonular proteomes with age could be a consequence of an intricate interplay of factors. For example, the lens may stiffen over time and become less disordered due to the cumulative effects of metabolic byproducts that naturally increase with age [4]. In addition, the physiological stress endured by zonular fibers over time may result in adaptive responses at the molecular level, possibly leading to an increase in the disordered proteins that allow for flexibility while maintaining tensile strength. The zonules might develop compensatory mechanisms to maintain their function despite a less pliable lens, which could explain the inverse relationship observed: as the lens becomes more ordered and rigid, the zonules adapt by increasing their protein complexity and intrinsic protein disorder to preserve their elasticity and functionality.

In conclusion, our study quantifies and comparatively analyzes the intrinsic disorder profiles of zonular and lens proteins. Through our study, we show that there may be an inverse relationship between age and the amount of intrinsic protein disorder in the lens proteome. Our study also suggests that there may be a direct relationship between age and the amount of intrinsic protein disorder in the zonule proteome.

## Limitations

The lens proteome analyzed in our study was derived from previously published data by Wang et al., which primarily examined the human lens fiber cell membrane fraction. Their focus excluded more abundant soluble cytoplasmic proteins, such as lens crystallins. Nevertheless, the proteomic changes we observed, despite being based on a subset of lens proteins localized to the cell membrane, still has meaningful implications for the overall structure and function of the lens. Future research should incorporate both soluble and insoluble protein fractions to provide a more holistic view of age-related proteomic alterations in the lens.

Another limitation of our study is the disparity in the number of identified proteins between the younger lens and the older lens. Specifically, Wang et al. identified 951 proteins in the younger lens, while only 260 proteins were identified in the older lens. This discrepancy limits our understanding of age-related changes in lens proteomes. A future study evaluating more proteins in the older lens is needed to ensure a more balanced protein count which will allow for additional analysis of proteomic alterations with age.

In addition, as stated by De Maria et al., the primary limitation encountered in collecting human zonular samples was the often partially liquefied state of the vitreous in aged eyes. This condition precluded isolating a distinct hyaloid zonule sample, constraining our analysis to the equatorial zonule segment [8]. The implications of this limitation extend to the representativeness of the samples; certain proteins or structural features unique to the hyaloid zonule may not have been captured in our dataset. In addition, as per De Maria et al., to ensure a sufficient material volume for the proteomic analysis, zonular samples were pooled based on the availability of samples rather than stratified by specific age groups [8]. While each pool contained material from an equal number of eyes and was balanced for sex, the pool’s age distribution was not controlled. Consequently, the influence of age-related changes on protein disorder propensity could not be isolated and examined in a controlled manner. This grouping method may have masked age-specific trends and limit the granularity of our understanding of age-related changes in zonular protein composition and over propensity for intrinsic protein disorder. In a similar light, neither the Wang et al. nor De Maria et al. studies mentioned comorbidities or other confounding health conditions affecting the donors of the samples used. For example, underlying systemic or ocular inflammatory conditions may affect the expression of various proteins in the lens and zonules, potentially influencing the conclusions of our analysis. The sample size is limited by the proteins identified by Wang et al. We hope that future investigators will identify more proteins which we can then analyze. Additionally, Wang et al. and De Maria et al. studies utilized different digestion and fixation methods to examine the proteomes of their respective samples. The discrepancies in sample preparation may have affected the presence and relative levels of certain proteins in the final observed proteomes.

Another limitation of our study is that there may be variations in the disorder predictor software utilized. Disorder predictor models are developed with training and testing data sets, which are not standardized across predictors. When different data sets are used for training, performances among different disorder predictors may vary. However, we attempted to address this in two ways: by employing multiple disorder predictors and calculating the mean disorder profile for ADS and PPDR, which served as aggregate scores for all of the utilized disorder predictors.

The recognition of these limitations is critical for the interpretation of the study’s findings and for guiding future research directions. Addressing these limitations in subsequent studies could involve collecting and analyzing individual samples without pooling and instead stratifying sample groups by more refined age categories. Despite these limitations, our data highlights significant findings that suggest that the level of intrinsic disorder in both human lens and zonule proteomes are affected by age. Further investigation, including a more comprehensive age range and a larger sample size, may elucidate additional information regarding the biological significance of the observed variations of intrinsic protein disorder between lens and zonular proteomes.

## Conclusions

Our study quantifies the levels of intrinsically disordered proteins in the human lens and zonules. These biological tissues have significant variations in their levels of intrinsic protein disorder at a proteome level. In addition, our findings suggest that increases in age have an inverse relationship with intrinsic protein disorder in the lens proteome and a direct relationship with intrinsic protein disorder in the zonule proteome. These findings suggest that varying levels of intrinsic protein disorder in the lens and zonules may lead to age-related processes that affect these structures.

## Supplementary information

Supplementary S2 Zonule Fasta File

Supplementary S3 25-37 year old Lens Fasta

Supplementary S4 58 year old Lens fasta

Tukey HSD S1 Supplementary

## Data Availability

Data is provided within the manuscript or supplementary information files.

## Abbreviations

IDPs: Intrinsically Disordered Proteins
IDPRs: Intrinsically Disordered Protein Regions
RIDAO: Rapid Intrinsic Disorder Analysis Online
ADS: Average Disorder Score
PPDR: Percentages of Predicted Disordered Residues
PONDR®: Predictor of Natural Disordered Protein Regions
ANOVA: Analysis of Variance
Tukey’s HSD: Tukey Honestly Significant Difference
CH: Charge-Hydropathy
CDF: Cumulative Distribution Function
AGE: Advanced Glycation End Product

## References

[1] Djulbegovic, M., & Uversky, V. N. (2022). The aqueous humor proteome is intrinsically disordered. *Biochemistry and Biophysics Reports*, *29*, 101202. 10.1016/j.bbrep.2022.101202. 35128080 10.1016/j.bbrep.2022.101202 PMC8808082

[2] Taylor Gonzalez, D. J., Djulbegovic, M., Antonietti, M., Cordova, M., Dayhoff, G. W., Mattes, R., Galor, A., Uversky, V. N., & Karp, C. L. (2023). Intrinsic disorder in the human tear proteome. *Investigative Ophthalmology & Visual Science*, *64*(11), 14. 10.1167/iovs.64.11.14. 10.1167/iovs.64.11.14 PMC10424804 37561450

[3] Antonietti, M., Gonzalez, D. J. T., Djulbegovic, M. B., Gamerio, G. R., Uversky, V. N., Sridhar, J., & Karp, C. L. (2024). Intrinsic disorder in the human vitreous proteome. *International Journal of Biological Macromolecules*, *267*, 131274. 38569991 10.1016/j.ijbiomac.2024.131274 PMC11182622

[4] Hernandez-Zimbron, L. F., Gulias-Canizo, R., Golzarri, M. F., Martinez-Baez, B. E., Quiroz-Mercado, H. & Gonzalez-Salinas, R. (2017). Molecular age-related changes in the anterior segment of the eye. *British Journal of Ophthalmology*, *2017*, 1295132. 10.1155/2017/1295132. 10.1155/2017/1295132 PMC5632897 29147580

[5] Michael, R., & Bron, A. J. (2011). The ageing lens and cataract: a model of normal and pathological ageing. *Philosophical Transactions of the Royal Society B: Biological Sciences*, *366*(1568), 1278–1292. 10.1098/rstb.2010.0300. 10.1098/rstb.2010.0300 PMC3061107 21402586

[6] Moreau, K. L., & King, J. A. (2012). Protein misfolding and aggregation in cataract disease and prospects for prevention. *Trends in Molecular Medicine*, *18*(5), 273–282. 10.1016/j.molmed.2012.03.005. 22520268 10.1016/j.molmed.2012.03.005 PMC3621977

[7] Vazquez, L. E., & Lee, R. K. (2014). Genomic and proteomic pathophysiology of pseudoexfoliation glaucoma. *International Ophthalmology Clinics*, *54*(4), 1–13. 10.1097/IIO.0000000000000047. 25171640 10.1097/IIO.0000000000000047 PMC4182319

[8] De Maria, A., Wilmarth, P. A., David, L. L., & Bassnett, S. (2017). Proteomic analysis of the bovine and human ciliary zonule. *Investigative Ophthalmology & Visual Science*, *58*(1), 573–585. 10.1167/iovs.16-20866. 28125844 10.1167/iovs.16-20866 PMC5283081

[9] Wang, Z., Han, J., David, L. L., & Schey, K. L. (2013). Proteomics and phosphoproteomics analysis of human lens fiber cell membranes. *Investigative Ophthalmology & Visual Science*, *54*(2), 1135–1143. 10.1167/iovs.12-11168. 23349431 10.1167/iovs.12-11168 PMC3567755

[10] Ruan, X., Liu, Z., Luo, L., & Liu, Y. (2020). The structure of the lens and its associations with the visual quality. *BMJ Open Ophthalmology*, *5*(1), e000459. 10.1136/bmjophth-2020-000459. 33024825 10.1136/bmjophth-2020-000459 PMC7511618

[11] Bassnett, S.(2009). On the mechanism of organelle degradation in the vertebrate lens. *Experimental Eye Research*, *88*(2), 133–139. 10.1016/j.exer.2008.08.017. 18840431 10.1016/j.exer.2008.08.017 PMC2693198

[12] Muranov, K. O., & Ostrovsky, M. A. (2022). Biochemistry of eye lens in the norm and in cataractogenesis. *Biochemistry (Moscow)*, *87*(2), 106–120. 10.1134/S0006297922020031. 35508906 10.1134/S0006297922020031

[13] Glasser, A., & Campbell, M. C. (1999). Biometric, optical and physical changes in the isolated human crystalline lens with age in relation to presbyopia. *Vision Research*, *39*(11), 1991–2015. 10.1016/s0042-6989(98)00283-1. 10343784 10.1016/s0042-6989(98)00283-1

[14] Heys, K. R., Cram, S. L., & Truscott, R. J. (2004). Massive increase in the stiffness of the human lens nucleus with age: the basis for presbyopia?. *Molecular Vision*, *10*, 956–963. 15616482

[15] Wistow, G. J., & Piatigorsky, J. (1988). Lens crystallins: the evolution and expression of proteins for a highly specialized tissue. *Annual Review of Biochemistry*, *57*, 479–504. 10.1146/annurev.bi.57.070188.002403. 3052280 10.1146/annurev.bi.57.070188.002403

[16] Hejtmancik, J. F., & Shiels, A. (2015). Overview of the lens. *Progress in Molecular Biology and Translational Science*, *134*, 119–127. 10.1016/bs.pmbts.2015.04.006. 26310153 10.1016/bs.pmbts.2015.04.006 PMC5656279

[17] Andley, U. P.(2007). Crystallins in the eye: Function and pathology. *Progress in Retinal and Eye Research*, *26*(1), 78–98. 10.1016/j.preteyeres.2006.10.003. 17166758 10.1016/j.preteyeres.2006.10.003

[18] Bassnett, S.(2021). Zinn’s zonule. *Progress in Retinal and Eye Research*, *82*, 100902. 10.1016/j.preteyeres.2020.100902. 32980533 10.1016/j.preteyeres.2020.100902 PMC8139560

[19] Chakrabarti, P., & Chakravarty, D. (2022). Intrinsically disordered proteins/regions and insight into their biomolecular interactions. *Biophysical Chemistry*, *283*, 106769. 10.1016/j.bpc.2022.106769. 35139468 10.1016/j.bpc.2022.106769

[20] Uversky, V. N.(2013). A decade and a half of protein intrinsic disorder: biology still waits for physics. *Protein Science*, *22*(6), 693–724. 10.1002/pro.2261. 23553817 10.1002/pro.2261 PMC3690711

[21] Santofimia-Castano, P., Rizzuti, B., Xia, Y., Abian, O., Peng, L., Velazquez-Campoy, A., Neira, J. L., & Iovanna, J. (2020). Targeting intrinsically disordered proteins involved in cancer. *Cellular and Molecular Life Sciences*, *77*(9), 1695–1707. 10.1007/s00018-019-03347-3. 31667555 10.1007/s00018-019-03347-3 PMC7190594

[22] Ayyadevara, S., Ganne, A., Balasubramaniam, M., & Shmookler Reis, R. J. (2022). Intrinsically disordered proteins identified in the aggregate proteome serve as biomarkers of neurodegeneration. *Metabolic Brain Disease*, *37*(1), 147–152. 10.1007/s11011-021-00791-8. 34347206 10.1007/s11011-021-00791-8 PMC8748380

[23] Consortium, T. U. (2022). UniProt: the Universal Protein Knowledgebase in 2023. *Nucleic Acids Research*, *51*(D1), D523–D531. 10.1093/nar/gkac1052. (acccessed 4/14/2024). 10.1093/nar/gkac1052 PMC9825514 36408920

[24] Vacic, V., Uversky, V. N., Dunker, A. K., & Lonardi, S. (2007). Composition Profiler: a tool for discovery and visualization of amino acid composition differences. *BMC Bioinformatics*, *8*, 211 10.1186/1471-2105-8-211. 17578581 10.1186/1471-2105-8-211 PMC1914087

[25] Dayhoff, G. W., & Uversky, V. N. (2022). Rapid prediction and analysis of protein intrinsic disorder. *Protein Science*, *31*(12), e4496. 10.1002/pro.4496. 36334049 10.1002/pro.4496 PMC9679974

[26] Peng, K., Vucetic, S., Radivojac, P., Brown, C. J., Dunker, A. K., & Obradovic, Z. (2005). Optimizing long intrinsic disorder predictors with protein evolutionary information. *Journal of Bioinformatics and Computational Biology*, *3*(1), 35–60. 10.1142/s0219720005000886. 15751111 10.1142/s0219720005000886

[27] Necci, M., Piovesan, D., & Tosatto, S. C. (2021). Critical assessment of protein intrinsic disorder prediction. *Nature Methods*, *18*(5), 472–481. 33875885 10.1038/s41592-021-01117-3 PMC8105172

[28] Djulbegovic, M. B., Taylor, D. J., Antonietti, M., Cordova, M., Dayhoff, G., Uversky, V., Galor, A., & Karp, C. L. (2023). Intrinsic disorder and the human tear film proteome. *Investigative Ophthalmology & Visual Science*, *64*(8), 187–187. 10.1167/iovs.64.11.14 PMC10424804 37561450

[29] Djulbegovic, M., & Uversky, V. N. (2022). The aqueous humor proteome is intrinsically disordered. *Biochemistry and Biophysics Reports*, *29*, 101202. 35128080 10.1016/j.bbrep.2022.101202 PMC8808082

[30] Huang, F., Oldfield, C., Meng, J., Hsu, W. L., Xue, B., Uversky, V. N., Romero, P., & Dunker, A. K. (2012). Subclassifying disordered proteins by the CH-CDF plot method. The *Pacific Symposium* on *Biocomputing*, 128–139. 22174269

[31] Xue, B., Oldfield, C. J., Dunker, A. K., & Uversky, V. N. (2009). CDF it all: consensus prediction of intrinsically disordered proteins based on various cumulative distribution functions. *FEBS Letters*, *583*(9), 1469–1474. 10.1016/j.febslet.2009.03.070. 19351533 10.1016/j.febslet.2009.03.070 PMC2683465

[32] Piha-Gossack, A., Sossin, W., & Reinhardt, D. P. (2012). The evolution of extracellular fibrillins and their functional domains. *PLoS One*, *7*(3), e33560. 10.1371/journal.pone.0033560. 22438950 10.1371/journal.pone.0033560 PMC3306419

[33] Thomson, J., Singh, M., Eckersley, A., Cain, S. A., Sherratt, M. J., & Baldock, C. (2019). Fibrillin microfibrils and elastic fibre proteins: Functional interactions and extracellular regulation of growth factors. *Seminars in Cell and Developmental Biology*, *89*, 109–117. 10.1016/j.semcdb.2018.07.016. 30016650 10.1016/j.semcdb.2018.07.016 PMC6461133

[34] Fan, X., Xiaoqin, L., Potts, B., Strauch, C. M., Nemet, I., & Monnier, V. M. (2011). Topical application of L-arginine blocks advanced glycation by ascorbic acid in the lens of hSVCT2 transgenic mice. *Molecular Vision*, *17*, 2221–2227. 21897744 PMC3164690

[35] Ma, X. J., Gao, J. Y., Tong, P., Li, X., & Chen, H. B. (2017). Tracking the behavior of Maillard browning in lysine/arginine-sugar model systems under high hydrostatic pressure. *Journal of the Science of Food and Agriculture*, *97*(15), 5168–5175. 10.1002/jsfa.8398. 28436030 10.1002/jsfa.8398

[36] Rankenberg, J., Rakete, S., Wagner, B. D., Patnaik, J. L., Henning, C., Lynch, A., Glomb, M. A., & Nagaraj, R. H. (2021). Advanced glycation end products in human diabetic lens capsules. *Experimental Eye Research*, *210*, 108704. 10.1016/j.exer.2021.108704. 34302851 10.1016/j.exer.2021.108704 PMC8429260

[37] Katz, J. A., Karpecki, P. M., Dorca, A., Chiva-Razavi, S., Floyd, H., Barnes, E., Wuttke, M., & Donnenfeld, E. (2021). Presbyopia - A review of current treatment options and emerging therapies. *Clinical Ophthalmology*, *15*, 2167–2178. 10.2147/OPTH.S259011. 34079215 10.2147/OPTH.S259011 PMC8163965

[38] Garner, W. H., & Garner, M. H. (2016). Protein disulfide levels and lens elasticity modulation: Applications for presbyopia. *Investigative Ophthalmology & Visual Science*, *57*(6), 2851–2863. 10.1167/iovs.15-18413. 27233034 10.1167/iovs.15-18413 PMC5995025

[39] Shi, Y., Tu, Y., De Maria, A., Mecham, R. P., & Bassnett, S. (2013). Development, composition, and structural arrangements of the ciliary zonule of the mouse. *Investigative Ophthalmology & Visual Science*, *54*(4), 2504–2515. 10.1167/iovs.13-11619. 23493297 10.1167/iovs.13-11619 PMC3621578

[40] Nankivil, D., Maceo Heilman, B., Durkee, H., Manns, F., Ehrmann, K., Kelly, S., Arrieta-Quintero, E., & Parel, J. M. (2015). The zonules selectively alter the shape of the lens during accommodation based on the location of their anchorage points. *Investigative Ophthalmology & Visual Science*, *56*(3), 1751–1760. 10.1167/iovs.14-16082. 25698707 10.1167/iovs.14-16082 PMC4356199

[41] Dikopf, M. S., Chow, C. C., Mieler, W. F., & Tu, E. Y. (2013). Cataract extraction outcomes and the prevalence of zonular insufficiency in retinitis pigmentosa. *American Journal of Ophthalmology*, *156*(1), 82–88. 10.1016/j.ajo.2013.02.002. 23628349 10.1016/j.ajo.2013.02.002

