# Supplementary material for: Effects of Aging on Intrinsic Protein Disorder in Human Lenses and Zonules
Source: Cell Biochem Biophys. 2024 Aug 8;82(4):3667–79. doi: 10.1007/s12013-024-01455-x (PMC11576620; doi:10.1007/s12013-024-01455-x)
Supplement: Supplementary file 1 — Supplementary S2 Zonule Fasta File [file 12013_2024_1455_MOESM1_ESM.pdf]

>sp|P35555|FBN1_HUMAN Fibrillin-1 OS=Homo sapiens OX=9606 GN=FBN1 PE=1 SV=4
MRRGRLLEIALGFTVLLASYTSHGADANLEAGNVKETRASRAKRRGGGGHDALKGPNVCG
SRYNAYCCPGWKTLPGGNQCIVPICRHSCGDGFCSRPNMCTCPSGQIAPSCGSRSIQHCN
IRCMNGGSCSDDHCLCQKGYIGTHCGQPVCESGCLNGGRCVAPNRCACTYGFTGPQCERD
YRTGPCFTVISNQMCQGQLSGIVCTKTLCCATVGRAWGHPCEMCPAQPHPCRRGFIPNIR
TGACQDVDECQAIPGLCQGGNCINTVGSFECKCPAGHKLNEVSQKCEDIDECSTIPGICE
GGECTNTVSSYFCKCPPGFYTSPDGTRCIDVRPGYCYTALTNGRCSNQLPQSITKMQCCC
DAGRCWSPGVTVAPEMCPIRATEDFNKLCSVPMVIPGRPEYPPPPLGPIPPVLPVPPGFP
PGPQIPVPRPPVEYLYPSREPPRVLPVNVTDYCQLVRYLCQNGRCIPTPGSYRCECNKGF
QLDLRGECIDVDECEKNPCAGGECINNQGSYTCQCRAGYQSTLTRTECRDIDECLQNGRI
CNNGRCINTDGSFHCVCNAGFHVTRDGKNCEDMDECSIRNMCLNGMCINEDGSFKCICKP
GFQLASDGRYCKDINECETPGICMNGRCVNTDGSYRCECFPGLAVGLDGRVCVDTHMRST
CYGGYKRGQCIKPLFGAVTKSECCCASTEYAFGEPCQPCPAQNSAEYQALCSSGPGMTSA
GSDINECALDPDICPNGICENLRGTYKCICNSGYEVDSTGKNCVDINECVLNSLLCDNGQ
CRNTPGSFVCTCPKGFIYKPDLKTCEDIDECESSPCINGVCKNSPGSFICECSSESTLDP
TKTICIETIKGTCWQTVIDGRCEININGATLKSQCCSSLGAAWGSPCTLCQVDPICGKGY
SRIKGTQCEDIDECEVFPGVCKNGLCVNTRGSFKCQCPSGMTLDATGRICLDIRLETCFL
RYEDEECTLPIAGRHRMDACCCSVGAAWGTEECEECPMRNTPEYEELCPRGPGFATKEIT
NGKPFFKDINECKMIPSLCTHGKCRNTIGSFKCRCDSGFALDSEERNCTDIDECRISPDL
CGRGQCVNTPGDFECKCDEGYESGFMMMKNCMDIDECQRDPLLCRGGVCHNTEGSYRCEC
PPGHQLSPNISACIDINECELSAHLCPNGRCVNLIGKYQCACNPGYHSTPDRLFCVDIDE
CSIMNGGCETFCTNSEGSYECSCQPGFALMPDQRSCTDIDECEDNPNICDGGQCTNIPGE
YRCLCYDGFMASEDMKTCVDVNECDLNPNICLSGTCENTKGSFICHCDMGYSGKKGKTGC
TDINECEIGAHNCGKHAVCTNTAGSFKCSCSPGWIGDGIKCTDLDECSNGTHMCSQHADC
KNTMGSYRCLCKEGYTGDGFTCTDLDECSENLNLCGNGQCLNAPGGYRCECDMGFVPSAD
GKACEDIDECSLPNICVFGTCHNLPGLFRCECEIGYELDRSGGNCTDVNECLDPTTCISG
NCVNTPGSYICDCPPDFELNPTRVGCVDTRSGNCYLDIRPRGDNGDTACSNEIGVGVSKA
SCCCSLGKAWGTPCEMCPAVNTSEYKILCPGGEGFRPNPITVILEDIDECQELPGLCQGG
KCINTFGSFQCRCPTGYYLNEDTRVCDDVNECETPGICGPGTCYNTVGNYTCICPPDYMQ
VNGGNNCMDMRRSLCYRNYYADNQTCDGELLFNMTKKMCCCSYNIGRAWNKPCEQCPIPS
TDEFATLCGSQRPGFVIDIYTGLPVDIDECREIPGVCENGVCINMVGSFRCECPVGFFYN
DKLLVCEDIDECQNGPVCQRNAECINTAGSYRCDCKPGYRFTSTGQCNDRNECQEIPNIC
SHGQCIDTVGSFYCLCHTGFKTNDDQTMCLDINECERDACGNGTCRNTIGSFNCRCNHGF
ILSHNNDCIDVDECASGNGNLCRNGQCINTVGSFQCQCNEGYEVAPDGRTCVDINECLLE
PRKCAPGTCQNLDGSYRCICPPGYSLQNEKCEDIDECVEEPEICALGTCSNTEGSFKCLC
PEGFSLSSSGRRCQDLRMSYCYAKFEGGKCSSPKSRNHSKQECCCALKGEGWGDPCELCP
TEPDEAFRQICPYGSGIIVGPDDSAVDMDECKEPDVCKHGQCINTDGSYRCECPFGYILA
GNECVDTDECSVGNPCGNGTCKNVIGGFECTCEEGFEPGPMMTCEDINECAQNPLLCAFR
CVNTYGSYECKCPVGYVLREDRRMCKDEDECEEGKHDCTEKQMECKNLIGTYMCICGPGY
QRRPDGEGCVDENECQTKPGICENGRCLNTRGSYTCECNDGFTASPNQDECLDNREGYCF
TEVLQNMCQIGSSNRNPVTKSECCCDGGRGWGPHCEICPFQGTVAFKKLCPHGRGFMTNG
ADIDECKVIHDVCRNGECVNDRGSYHCICKTGYTPDITGTSCVDLNECNQAPKPCNFICK
NTEGSYQCSCPKGYILQEDGRSCKDLDECATKQHNCQFLCVNTIGGFTCKCPPGFTQHHT
SCIDNNECTSDINLCGSKGICQNTPGSFTCECQRGFSLDQTGSSCEDVDECEGNHRCQHG

CQNIIGGYRCSCPQGYLQHYQWNQCVDENECLSAHICGGASCHNTLGSYKCMCPAGFQYE
QFSGGCQDINECGSAQAPCSYGCSNTEGGYLCGCPPGYFRIGQGHCVSGMGMGRGNPEPP
VSGEMDDNSLSPEACYECKINGYPKRGRKRRSTNETDASNIEDQSETEANVSLASWDVEK
TAIFAFNISHVSNKVRILELLPALTTLTNHNRYLIESGNEDGFFKINQKEGISYLHFTKK
KPVAGTYSLQISSTPLYKKKELNQLEDKYDKDYLSGELGDNLKMKIQVLLH
>sp|Q14767|LTBP2_HUMAN Latent-transforming growth factor beta-binding protein 2 OS=Homo sapiens OX=9606 GN=LTBP2 PE=1 SV=3
MRPRTKARSPGRALRNPWRGFLPLTLALFVGAGHAQRDPVGRYEPAGGDANRLRRPGGSY
PAAAAAKVYSLFREQDAPVAGLQPVERAQPGWGSPRRPTEAEARRPSRAQQSRRVQPPAQ
TRRSTPLGQQQPAPRTRAAPALPRLGTPQRSGAAPPTPPRGRLTGRNVCGGQCCPGWTTA
NSTNHCIKPVCEPPCQNRGSCSRPQLCVCRSGFRGARCEEVIPDEEFDPQNSRLAPRRWA
ERSPNLRRSSAAGEGTLARAQPPAPQSPPAPQSPPAGTLSGLSQTHPSQQHVGLSRTVRL
HPTATASSQLSSNALPPGPGLEQRDGTQQAVPLEHPSSPWGLNLTEKIKKIKIVFTPTIC
KQTCARGHCANSCERGDTTTLYSQGGHGHDPKSGFRIYFCQIPCLNGGRCIGRDECWCPA
NSTGKFCHLPIPQPDREPPGRGSRPRALLEAPLKQSTFTLPLSNQLASVNPSLVKVHIHH
PPEASVQIHQVAQVRGGVEEALVENSVETRPPPWLPASPGHSLWDSNNIPARSGEPPRPL
PPAAPRPRGLLGRCYLNTVNGQCANPLLELTTQEDCCGSVGAFWGVTLCAPCPPRPASPV
IENGQLECPQGYKRLNLTHCQDINECLTLGLCKDAECVNTRGSYLCTCRPGLMLDPSRSR
CVSDKAISMLQGLCYRSLGPGTCTLPLAQRITKQICCCSRVGKAWGSECEKCPLPGTEAF
REICPAGHGYTYASSDIRLSMRKAEEEELARPPREQGQRSSGALPGPAERQPLRVVTDTW
LEAGTIPDKGDSQAGQVTTSVTHAPAWVTGNATTPPMPEQGIAEIQEEQVTPSTDVLVTL
STPGIDRCAAGATNVCGPGTCVNLPDGYRCVCSPGYQLHPSQAYCTDDNECLRDPCKGKG
RCINRVGSYSCFCYPGYTLATSGATQECQDINECEQPGVCSGGQCTNTEGSYHCECDQGY
IMVRKGHCQDINECRHPGTCPDGRCVNSPGSYTCLACEEGYRGQSGSCVDVNECLTPGVC
AHGKCTNLEGSFRCSCEQGYEVTSDEKGCQDVDECASRASCPTGLCLNTEGSFACSACEN
GYWVNEDGTACEDLDECAFPGVCPSGVCTNTAGSFSCKDCDGGYRPSPLGDSCEDVDECE
DPQSSCLGGECKNTVGSYQCLCPQGFQLANGTVCEDVNECMGEEHCAPHGECLNSHGSFF
CLCAPGFVSAEGGTSCQDVDECATTDPCVGGHCVNTEGSFNCLCETGFQPSPESGECVDI
DECEDYGDPVCGTWKCENSPGSYRCVLGCQPGFHMAPNGDCIDIDECANDTMCGSHGFCD
NTDGSFRCLCDQGFEISPSGWDCVDVNECELMLAVCGAALCENVEGSFLCLCASDLEEYD
AQEGHCRPRGAGGQSMSEAPTGDHAPAPTRMDCYSGQKGHAPCSSVLGRNTTQAECCCTQ
GASWGDACDLCPSEDSAEFSEICPSGKGYIPVEGAWTFGQTMYTDADECVIFGPGLCPNG
RCLNTVPGYVCLCNPGFHYDASHKKCEDHDECQDLACENGECVNTEGSFHCFCSPPLTLD
LSQQRCMNSTSSTEDLPDHDIHMDICWKKVTNDVCSEPLRGHRTTYTECCCQDGEAWSQQ
CALCPPRSSEVYAQLCNVARIEAEREAGVHFRPGYEYGPGPDDLHYSIYGPDGAPFYNYL
GPEDTVPEPAFPNTAGHSADRTPILESPLQPSELQPHYVASHPEPPAGFEGLQAEECGIL
NGCENGRCVRVREGYTCDCFEGFQLDAAHMACVDVNECDDLNGPAVLCVHGYCENTEGSY
RCHCSPGYVAEAGPPHCTAKE
>sp|Q96RW7|HMCN1_HUMAN Hemicentin-1 OS=Homo sapiens OX=9606 GN=HMCN1 PE=1 SV=2
MISWEVVHTVFLFALLYSSLAQDASPQSEIRAEEIPEGASTLAFVFDVTGSMYDDLVQVI
EGASKILETSLKRPKRPLFNFALVPFHDPEIGPVTITTDPKKFQYELRELYVQGGGDCPE
MSIGAIKIALEISLPGSFIYVFTDARSKDYRLTHEVLQLIQQKQSQVVFVLTGDCDDRTH
IGYKVYEEIASTSSGQVFHLDKKQVNEVLKWVEEAVQASKVHLLSTDHLEQAVNTWRIPF

DPSLKEVTVSLSGPSPMIEIRNPLGKLIKKGFGLHELLNIHNSAKVVNVKEPEAGMWTVK
TSSSGRHSVRITGLSTIDFRAGFSRKPTLDFKKTVSRPVQGIPTYVLLNTSGISTPARID
LLELLSISGSSLKTIPVKYYPHRKPYGIWNISDFVPPNEAFFLKVTGYDKDDYLFQRVSS
VSFSSIVPDAPKVTMPEKTPGYYLQPGQIPCSVDSLLPFTLSFVRNGVTLGVDQYLKESA
SVNLDIAKVTLSDEGFYECIAVSSAGTGRAQTFFDVSEPPPVIQVPNNVTVTPGERAVLT
CLIISAVDYNLTWQRNDRDVRLAEPARIRTLANLSLELKSVKFNDAGEYHCMVSSEGGSS
AASVFLTVQEPPKVTVMPKNQSFTGGSEVSIMCSATGYPKPKIAWTVNDMFIVGSHRYRM
TSDGTLFIKNAAPKDAGIYGCLASNSAGTDKQNSTLRYIEAPKLMVVQSELLVALGDITV
MECKTSGIPPPQVKWFKGDLELRPSTFLIIDPLLGLLKIQETQDLDAGDYTCVAINEAGR
ATGKITLDVGSPPVFIQEPADVSMEIGSNVTLPCYVQGYPEPTIKWRRLDNMPIFSRPFS
VSSISQLRTGALFILNLWASDKGTYICEAENQFGKIQSETTVTVTGLVAPLIGISPSVAN
VIEGQQLTLPCTLLAGNPIPERRWIKNSAMLLQNPYITVRSDGSLHIERVQLQDGGEYTC
VASNVAGTNNKTTSVVVHVLPTIQHGQQILSTIEGIPVTLPCKASGNPKPSVIWSKKGEL
ISTSSAKFSAGADGSLYVVSPGGEESGEYVCTATNTAGYAKRKVQLTVYVRPRVFGDQRG
LSQDKPVEISVLAGEEVTLPCEVKSLPPPIITWAKETQLISPFSPRHTFLPSGSMKITET
RTSDSGMYLCVATNIAGNVTQAVKLNVHVPPKIQRGPKHLKVQVGQRVDIPCNAQGTPLP
VITWSKGGSTMLVDGEHHVSNPDGTLSIDQATPSDAGIYTCVATNIAGTDETEITLHVQE
PPTVEDLEPPYNTTFQERVANQRIEFPCPAKGTPKPTIKWLHNGRELTGREPGISILEDG
TLLVIASVTPYDNGEYICVAVNEAGTTERKYNLKVHVPPVIKDKEQVTNVSVLLNQLTNL
FCEVEGTPSPIIMWYKDNVQVTESSTIQTVNNGKILKLFRATPEDAGRYSCKAINIAGTS
QKYFNIDVLVPPTIIGTNFPNEVSVVLNRDVALECQVKGTPFPDIHWFKDGKPLFLGDPN
VELLDRGQVLHLKNARRNDKGRYQCTVSNAAGKQAKDIKLTIYIPPSIKGGNVTTDISVL
INSLIKLECETRGLPMPAITWYKDGQPIMSSSQALYIDKGQYLHIPRAQVSDSATYTCHV
ANVAGTAEKSFHVDVYVPPMIEGNLATPLNKQVVIAHSLTLECKAAGNPSPILTWLKDGV
PVKANDNIRIEAGGKKLEIMSAQEIDRGQYICVATSVAGEKEIKYEVDVLVPPAIEGGDE
TSYFIVMVNNLLELDCHVTGSPPPTIMWLKDGQLIDERDGFKILLNGRKLVIAQAQVSNT
GLYRCMAANTAGDHKKEFEVTVHVPPTIKSSGLSERVVVKYKPVALQCIANGIPNPSITW
LKDDQPVNTAQGNLKIQSSGRVLQIAKTLLEDAGRYTCVATNAAGETQQHIQLHVHEPPS
LEDAGKMLNETVLVSNPVQLECKAAGNPVPVITWYKDNRLLSGSTSMTFLNRGQIIDIES
AQISDAGIYKCVAINSAGATELFYSLQVHVAPSISGSNNMVAVVVNNPVRLECEARGIPA
PSLTWLKDGSPVSSFSNGLQVLSGGRILALTSAQISDTGRYTCVAVNAAGEKQRDIDLRV
YVPPNIMGEEQNVSVLISQAVELLCQSDAIPPPTLTWLKDGHPLLKKPGLSISENRSVLK
IEDAQVQDTGRYTCEATNVAGKTEKNYNVNIWVPPNIGGSDELTQLTVIEGNLISLLCES
SGIPPPNLIWKKKGSPVLTDSMGRVRILSGGRQLQISIAEKSDAALYSCVASNVAGTAKK
EYNLQVYIRPTITNSGSHPTEIIVTRGKSISLECEVQGIPPPTVTWMKDGHPLIKAKGVE
ILDEGHILQLKNIHVSDTGRYVCVAVNVAGMTDKKYDLSVHAPPSIIGNHRSPENISVVE
KNSVSLTCEASGIPLPSITWFKDGWPVSLSNSVRILSGGRMLRLMQTTMEDAGQYTCVVR
NAAGEERKIFGLSVLVPPHIVGENTLEDVKVKEKQSVTLTCEVTGNPVPEITWHKDGQPL
QEDEAHHIISGGRFLQITNVQVPHTGRYTCLASSPAGHKSRSFSLNVFVSPTIAGVGSDG
NPEDVTVILNSPTSLVCEAYSYPPATITWFKDGTPLESNRNIRILPGGRTLQILNAQEDN
AGRYSCVATNEAGEMIKHYEVKVYIPPIINKGDLWGPGLSPKEVKIKVNNTLTLECEAYA
IPSASLSWYKDGQPLKSDDHVNIAANGHTLQIKEAQISDTGRYTCVASNIAGEDELDFDV
NIQVPPSFQKLWEIGNMLDTGRNGEAKDVIINNPISLYCETNAAPPPTLTWYKDGHPLTS
SDKVLILPGGRVLQIPRAKVEDAGRYTCVAVNEAGEDSLQYDVRVLVPPIIKGANSDLPE

EVTVLVNKSALIECLSSGSPAPRNSWQKDGQPLLEDDHHKFLSNGRILQILNTQITDIGR
YVCVAENTAGSAKKYFNLNVHVPPSVIGPKSENLTVVVNNFISLTCEVSGFPPPDLSWLK
NEQPIKLNTNTLIVPGGRTLQIIRAKVSDGGEYTCIAINQAGESKKKFSLTVYVPPSIKD
HDSESLSVVNVREGTSVSLECESNAVPPPVITWYKNGRMITESTHVEILADGQMLHIKKA
EVSDTGQYVCRAINVAGRDDKNFHLNVYVPPSIEGPEREVIVETISNPVTLTCDATGIPP
PTIAWLKNHKRIENSDSLEVRILSGGSKLQIARSQHSDSGNYTCIASNMEGKAQKYYFLS
IQVPPSVAGAEIPSDVSVLLGENVELVCNANGIPTPLIQWLKDGKPIASGETERIRVSAN
GSTLNIYGALTSDTGKYTCVATNPAGEEDRIFNLNVYVTPTIRGNKDEAEKLMTLVDTSI
NIECRATGTPPPQINWLKNGLPLPLSSHIRLLAAGQVIRIVRAQVSDVAVYTCVASNRAG
VDNKHYNLQVFAPPNMDNSMGTEEITVLKGSSTSMACITDGTPAPSMAWLRDGQPLGLDA
HLTVSTHGMVLQLLKAETEDSGKYTCIASNEAGEVSKHFILKVLEPPHINGSEEHEEISV
IVNNPLELTCIASGIPAPKMTWMKDGRPLPQTDQVQTLGGGEVLRISTAQVEDTGRYTCL
ASSPAGDDDKEYLVRVHVPPNIAGTDEPRDITVLRNRQVTLECKSDAVPPPVITWLRNGE
RLQATPRVRILSGGRYLQINNADLGDTANYTCVASNIAGKTTREFILTVNVPPNIKGGPQ
SLVILLNKSTVLECIAEGVPTPRITWRKDGAVLAGNHARYSILENGFLHIQSAHVTDTGR
YLCMATNAAGTDRRRIDLQVHVPPSIAPGPTNMTVIVNVQTTLACEATGIPKPSINWRKN
GHLLNVDQNQNSYRLLSSGSLVIISPSVDDTATYECTVTNGAGDDKRTVDLTVQVPPSIA
DEPTDFLVTKHAPAVITCTASGVPFPSIHWTKNGIRLLPRGDGYRILSSGAIEILATQLN
HAGRYTCVARNAAGSAHRHVTLHVHEPPVIQPQPSELHVILNNPILLPCEATGTPSPFIT
WQKEGINVNTSGRNHAVLPSGGLQISRAVREDAGTYMCVAQNPAGTALGKIKLNVQVPPV
ISPHLKEYVIAVDKPITLSCEADGLPPPDITWHKDGRAIVESIRQRVLSSGSLQIAFVQP
GDAGHYTCMAANVAGSSSTSTKLTVHVPPRIRSTEGHYTVNENSQAILPCVADGIPTPAI
NWKKDNVLLANLLGKYTAEPYGELILENVVLEDSGFYTCVANNAAGEDTHTVSLTVHVLP
TFTELPGDVSLNKGEQLRLSCKATGIPLPKLTWTFNNNIIPAHFDSVNGHSELVIERVSK
EDSGTYVCTAENSVGFVKAIGFVYVKEPPVFKGDYPSNWIEPLGGNAILNCEVKGDPTPT
IQWNRKGVDIEISHRIRQLGNGSLAIYGTVNEDAGDYTCVATNEAGVVERSMSLTLQSPP
IITLEPVETVINAGGKIILNCQATGEPQPTITWSRQGHSISWDDRVNVLSNNSLYIADAQ
KEDTSEFECVARNLMGSVLVRVPVIVQVHGGFSQWSAWRACSVTCGKGIQKRSRLCNQPL
PANGGKPCQGSDLEMRNCQNKPCPVDGSWSEWSLWEECTRSCGRGNQTRTRTCNNPSVQH
GGRPCEGNAVEIIMCNIRPCPVHGAWSAWQPWGTCSESCGKGTQTRARLCNNPPPAFGGS
YCDGAETQMQVCNERNCPIHGKWATWASWSACSVSCGGGARQRTRGCSDPVPQYGGRKCE
GSDVQSDFCNSDPCPTHGNWSPWSGWGTCSRTCNGGQMRRYRTCDNPPPSNGGRACGGPD
SQIQRCNTDMCPVDGSWGSWHSWSQCSASCGGGEKTRKRLCDHPVPVKGGRPCPGDTTQV
TRCNVQACPGGPQRARGSVIGNINDVEFGIAFLNATITDSPNSDTRIIRAKITNVPRSLG
SAMRKIVSILNPIYWTTAKEIGEAVNGFTLTNAVFKRETQVEFATGEILQMSHIARGLDS
DGSLLLDIVVSGYVLQLQSPAEVTVKDYTEDYIQTGPGQLYAYSTRLFTIDGISIPYTWN
HTVFYDQAQGRMPFLVETLHASSVESDYNQIEETLGFKIHASISKGDRSNQCPSGFTLDS
VGPFCADEDECAAGNPCSHSCHNAMGTYYCSCPKGLTIAADGRTCQDIDECALGRHTCHA
GQDCDNTIGSYRCVVRCGSGFRRTSDGLSCQDINECQESSPCHQRCFNAIGSFHCGCEPG
YQLKGRKCMDVNECRQNVCRPDQHCKNTRGGYKCIDLCPNGMTKAENGTCIDIDECKDGT
HQCRYNQICENTRGSYRCVCPRGYRSQGVGRPCMDINECEQVPKPCAHQCSNTPGSFKCI
CPPGQHLLGDGKSCAGLERLPNYGTQYSSYNLARFSPVRNNYQPQQHYRQYSHLYSSYSE
YRNSRTSLSRTRRTIRKTCPEGSEASHDTCVDIDECENTDACQHECKNTFGSYQCICPPG
YQLTHNGKTCQDIDECLEQNVHCGPNRMCFNMRGSYQCIDTPCPPNYQRDPVSGFCLKNC

PPNDLECALSPYALEYKLVSLPFGIATNQDLIRLVAYTQDGVMHPRTTFLMVDEEQTVPF
ALRDENLKGVVYTTRPLREAETYRMRVRASSYSANGTIEYQTTFIVYIAVSAYPY
>sp|P55001|MFAP2_HUMAN Microfibrillar-associated protein 2 OS=Homo sapiens OX=9606 GN=MFAP2 PE=1 SV=1
MRAAYLFLLFLPAGLLAQGQYDLDPLPPFPDHVQYTHYSDQIDNPDYYDYQEVTPRPSEE
QFQFQSQQQVQQEVIPAPTPEPGNAELEPTEPGPLDCREEQYPCTRLYSIHRPCKQCLNE
VCFYSLRRVYVINKEICVRTVCAHEELLRADLCRDKFSKCGVMASSGLCQSVAASCARSC
GSC
>sp|Q9Y6C2|EMIL1_HUMAN EMILIN-1 OS=Homo sapiens OX=9606 GN=EMILIN1 PE=1 SV=3
MAPRTLWSCYLCCLLTAAAGAASYPPRGFSLYTGSSGALSPGGPQAQIAPRPASRHRNWC
AYVVTRTVSCVLEDGVETYVKYQPCAWGQPQCPQSIMYRRFLRPRYRVAYKTVTDMEWRC
CQGYGGDDCAESPAPALGPASSTPRPLARPARPNLSGSSAGSPLSGLGGEGPGESEKVQQ
LEEQVQSLTKELQGLRGVLQGLSGRLAEDVQRAVETAFNGRQQPADAAARPGVHETLNEI
QHQLQLLDTRVSTHDQELGHLNNHHGGSSSSGGSRAPAPASAPPGPSEELLRQLEQRLQE
SCSVCLAGLDGFRRQQQEDRERLRAMEKLLASVEERQRHLAGLAVGRRPPQECCSPELGR
RLAELERRLDVVAGSVTVLSGRRGTELGGAAGQGGHPPGYTSLASRLSRLEDRFNSTLGP
SEEQEESWPGAPGGLSHWLPAARGRLEQLGGLLANVSGELGGRLDLLEEQVAGAMQACGQ
LCSGAPGEQDSQVSEILSALERRVLDSEGQLRLVGSGLHTVEAAGEARQATLEGLQEVVG
RLQDRVDAQDETAAEFTLRLNLTAARLGQLEGLLQAHGDEGCGACGGVQEELGRLRDGVE
RCSCPLLPPRGPGAGPGVGGPSRGPLDGFSVFGGSSGSALQALQGELSEVILSFSSLNDS
LNELQTTVEGQGADLADLGATKDRIISEINRLQQEATEHATESEERFRGLEEGQAQAGQC
PSLEGRLGRLEGVCERLDTVAGGLQGLREGLSRHVAGLWAGLRETNTTSQMQAALLEKLV
GGQAGLGRRLGALNSSLQLLEDRLHQLSLKDLTGPAGEAGPPGPPGLQGPPGPAGPPGSP
GKDGQEGPIGPPGPQGEQGVEGAPAAPVPQVAFSAALSLPRSEPGTVPFDRVLLNDGGYY
DPETGVFTAPLAGRYLLSAVLTGHRHEKVEAVLSRSNQGVARVDSGGYEPEGLENKPVAE
SQPSPGTLGVFSLILPLQAGDTVCVDLVMGQLAHSEEPLTIFSGALLYGDPELEHA
>sp|Q6ZMP0|THSD4_HUMAN Thrombospondin type-1 domain-containing protein 4 OS=Homo sapiens OX=9606 GN=THSD4 PE=1 SV=2
MVSHFMGSLSVLCFLLLLGFQFVCPQPSTQHRKVPQRMAAEGAPEDDGGGGAPGVWGAWG
PWSACSRSCSGGVMEQTRPCLPRSYRLRGGQRPGAPARAFADHVVSAVRTSVPLHRSRDE
TPALAGTDASRQGPTVLRGSRHPQPQGLEVTGDRRSRTRGTIGPGKYGYGKAPYILPLQT
DTAHTPQRLRRQKLSSRHSRSQGASSARHGYSSPAHQVPQHGPLYQSDSGPRSGLQAAEA
PIYQLPLTHDQGYPAASSLFHSPETSNNHGVGTHGATQSFSQPARSTAISCIGAYRQYKL
CNTNVCPESSRSIREVQCASYNNKPFMGRFYEWEPFAEVKGNRKCELNCQAMGYRFYVRQ
AEKVIDGTPCDQNGTAICVSGQCKSIGCDDYLGSDKVVDKCGVCGGDNTGCQVVSGVFKH
ALTSLGYHRVVEIPEGATKINITEMYKSNNYLALRSRSGRSIINGNWAIDRPGKYEGGGT
MFTYKRPNEISSTAGESFLAEGPTNEILDVYMIHQQPNPGVHYEYVIMGTNAISPQVPPH
RRPGEPFNGQMVTEGRSQEEGEQKGRNEEKEDLRGEAPEMFTSESAQTFPVRHPDRFSPH
RPDNLVPPAPQPPRRSRDHNWKQLGTTECSTTCGKGSQYPIFRCVHRSTHEEAPESYCDS
SMKPTPEEEPCNIFPCPAFWDIGEWSECSKTCGLGMQHRQVLCRQVYANRSLTVQPYRCQ
HLEKPETTSTCQLKICSEWQIRTDWTSCSVPCGVGQRTRDVKCVSNIGDVVDDEECNMKL
RPNDIENCDMGPCAKSWFLTEWSERCSAECGAGVRTRSVVCMTNHVSSLPLEGCGNNRPA
EATPCDNGPCTGKVEWFAGSWSQCSIECGSGTQQREVICVRKNADTFEVLDPSECSFLEK
PPSQQSCHLKPCGAKWFSTEWSMCSKSCQGGFRVREVRCLSDDMTLSNLCDPQLKPEERE

SCNPQDCVPEVDENCKDKYYNCNVVVQARLCVYNYYKTACCASCTRVANRQTGFLGSR
>sp|P35625|TIMP3_HUMAN Metalloproteinase inhibitor 3 OS=Homo sapiens OX=9606 GN=TIMP3 PE=1 SV=2
MTPWLGLIVLLGSWSLGDWGAEACTCSPSHPQDAFCNSDIVIRAKVVGKKLVKEGPFGTL
VYTIKQMKMYRGFTKMPHVQYIHTEASESLCGLKLEVNKYQYLLTGRVYDGKMYTGLCNF
VERWDQLTLSQRKGLNYRYHLGCNCKIKSCYYLPCFVTSKNECLWTDMLSNFGYPGYQSK
HYACIRQKGGYCSWYRGWAPPDKSIINATDP
>sp|P98160|PGBM_HUMAN Basement membrane-specific heparan sulfate proteoglycan core protein OS=Homo sapiens OX=9606 GN=HSPG2 PE=1 SV=4
MGWRAAGALLLALLLHGRLLAVTHGLRAYDGLSLPEDIETVTASQMRWTHSYLSDDEDML
ADSISGDDLGSGDLGSGDFQMVYFRALVNFTRSIEYSPQLEDAGSREFREVSEAVVDTLE
SEYLKIPGDQVVSVVFIKELDGWVFVELDVGSEGNADGAQIQEMLLRVISSGSVASYVTS
PQGFQFRRLGTVPQFPRACTEAEFACHSYNECVALEYRCDRRPDCRDMSDELNCEEPVLG
ISPTFSLLVETTSLPPRPETTIMRQPPVTHAPQPLLPGSVRPLPCGPQEAACRNGHCIPR
DYLCDGQEDCEDGSDELDCGPPPPCEPNEFPCGNGHCALKLWRCDGDFDCEDRTDEANCP
TKRPEEVCGPTQFRCVSTNMCIPASFHCDEESDCPDRSDEFGCMPPQVVTPPRESIQASR
GQTVTFTCVAIGVPTPIINWRLNWGHIPSHPRVTVTSEGGRGTLIIRDVKESDQGAYTCE
AMNARGMVFGIPDGVLELVPQRGPCPDGHFYLEHSAACLPCFCFGITSVCQSTRRFRDQI
RLRFDQPDDFKGVNVTMPAQPGTPPLSSTQLQIDPSLHEFQLVDLSRRFLVHDSFWALPE
QFLGNKVDSYGGSLRYNVRYELARGMLEPVQRPDVVLMGAGYRLLSRGHTPTQPGALNQR
QVQFSEEHWVHESGRPVQRAELLQVLQSLEAVLIQTVYNTKMASVGLSDIAMDTTVTHAT
SHGRAHSVEECRCPIGYSGLSCESCDAHFTRVPGGPYLGTCSGCNCNGHASSCDPVYGHC
LNCQHNTEGPQCNKCKAGFFGDAMKATATSCRPCPCPYIDASRRFSDTCFLDTDGQATCD
ACAPGYTGRRCESCAPGYEGNPIQPGGKCRPVNQEIVRCDERGSMGTSGEACRCKNNVVG
RLCNECADGSFHLSTRNPDGCLKCFCMGVSRHCTSSSWSRAQLHGASEEPGHFSLTNAAS
THTTNEGIFSPTPGELGFSSFHRLLSGPYFWSLPSRFLGDKVTSYGGELRFTVTQRSQPG
STPLHGQPLVVLQGNNIILEHHVAQEPSPGQPSTFIVPFREQAWQRPDGQPATREHLLMA
LAGIDTLLIRASYAQQPAESRVSGISMDVAVPEETGQDPALEVEQCSCPPGYRGPSCQDC
DTGYTRTPSGLYLGTCERCSCHGHSEACEPETGACQGCQHHTEGPRCEQCQPGYYGDAQR
GTPQDCQLCPCYGDPAAGQAAHTCFLDTDGHPTCDACSPGHSGRHCERCAPGYYGNPSQG
QPCQRDSQVPGPIGCNCDPQGSVSSQCDAAGQCQCKAQVEGLTCSHCRPHHFHLSASNPD
GCLPCFCMGITQQCASSAYTRHLISTHFAPGDFQGFALVNPQRNSRLTGEFTVEPVPEGA
QLSFGNFAQLGHESFYWQLPETYQGDKVAAYGGKLRYTLSYTAGPQGSPLSDPDVQITGN
NIMLVASQPALQGPERRSYEIMFREEFWRRPDGQPATREHLLMALADLDELLIRATFSSV
PLAASISAVSLEVAQPGPSNRPRALEVEECRCPPGYIGLSCQDCAPGYTRTGSGLYLGHC
ELCECNGHSDLCHPETGACSQCQHNAAGEFCELCAPGYYGDATAGTPEDCQPCACPLTNP
ENMFSRTCESLGAGGYRCTACEPGYTGQYCEQCGPGYVGNPSVQGGQCLPETNQAPLVVE
VHPARSIVPQGGSHSLRCQVSGSPPHYFYWSREDGRPVPSGTQQRHQGSELHFPSVQPSD
AGVYICTCRNLHQSNTSRAELLVTEAPSKPITVTVEEQRSQSVRPGADVTFICTAKSKSP
AYTLVWTRLHNGKLPTRAMDFNGILTIRNVQLSDAGTYVCTGSNMFAMDQGTATLHVQAS
GTLSAPVVSIHPPQLTVQPGQLAEFRCSATGSPTPTLEWTGGPGGQLPAKAQIHGGILRL
PAVEPTDQAQYLCRAHSSAGQQVARAVLHVHGGGGPRVQVSPERTQVHAGRTVRLYCRAA
GVPSATITWRKEGGSLPPQARSERTDIATLLIPAITTADAGFYLCVATSPAGTAQARIQV
VVLSASDASPPPVKIESSSPSVTEGQTLDLNCVVAGSAHAQVTWYRRGGSLPPHTQVHGS

RLRLPQVSPADSGEYVCRVENGSGPKEASITVSVLHGTHSGPSYTPVPGSTRPIRIEPSS
SHVAEGQTLDLNCVVPGQAHAQVTWHKRGGSLPARHQTHGSLLRLHQVTPADSGEYVCHV
VGTSGPLEASVLVTIEASVIPGPIPPVRIESSSSTVAEGQTLDLSCVVAGQAHAQVTWYK
RGGSLPARHQVRGSRLYIFQASPADAGQYVCRASNGMEASITVTVTGTQGANLAYPAGST
QPIRIEPSSSQVAEGQTLDLNCVVPGQSHAQVTWHKRGGSLPVRHQTHGSLLRLYQASPA
DSGEYVCRVLGSSVPLEASVLVTIEPAGSVPALGVTPTVRIESSSSQVAEGQTLDLNCLV
AGQAHAQVTWHKRGGSLPARHQVHGSRLRLLQVTPADSGEYVCRVVGSSGTQEASVLVTI
QQRLSGSHSQGVAYPVRIESSSASLANGHTLDLNCLVASQAPHTITWYKRGGSLPSRHQI
VGSRLRIPQVTPADSGEYVCHVSNGAGSRETSLIVTIQGSGSSHVPSVSPPIRIESSSPT
VVEGQTLDLNCVVARQPQAIITWYKRGGSLPSRHQTHGSHLRLHQMSVADSGEYVCRANN
NIDALEASIVISVSPSAGSPSAPGSSMPIRIESSSSHVAEGETLDLNCVVPGQAHAQVTW
HKRGGSLPSHHQTRGSRLRLHHVSPADSGEYVCRVMGSSGPLEASVLVTIEASGSSAVHV
PAPGGAPPIRIEPSSSRVAEGQTLDLKCVVPGQAHAQVTWHKRGGNLPARHQVHGPLLRL
NQVSPADSGEYSCQVTGSSGTLEASVLVTIEPSSPGPIPAPGLAQPIYIEASSSHVTEGQ
TLDLNCVVPGQAHAQVTWYKRGGSLPARHQTHGSQLRLHLVSPADSGEYVCRAASGPGPE
QEASFTVTVPPSEGSSYRLRSPVISIDPPSSTVQQGQDASFKCLIHDGAAPISLEWKTRN
QELEDNVHISPNGSIITIVGTRPSNHGTYRCVASNAYGVAQSVVNLSVHGPPTVSVLPEG
PVWVKVGKAVTLECVSAGEPRSSARWTRISSTPAKLEQRTYGLMDSHAVLQISSAKPSDA
GTYVCLAQNALGTAQKQVEVIVDTGAMAPGAPQVQAEEAELTVEAGHTATLRCSATGSPA
PTIHWSKLRSPLPWQHRLEGDTLIIPRVAQQDSGQYICNATSPAGHAEATIILHVESPPY
ATTVPEHASVQAGETVQLQCLAHGTPPLTFQWSRVGSSLPGRATARNELLHFERAAPEDS
GRYRCRVTNKVGSAEAFAQLLVQGPPGSLPATSIPAGSTPTVQVTPQLETKSIGASVEFH
CAVPSDRGTQLRWFKEGGQLPPGHSVQDGVLRIQNLDQSCQGTYICQAHGPWGKAQASAQ
LVIQALPSVLINIRTSVQTVVVGHAVEFECLALGDPKPQVTWSKVGGHLRPGIVQSGGVV
RIAHVELADAGQYRCTATNAAGTTQSHVLLLVQALPQISMPQEVRVPAGSAAVFPCIASG
YPTPDISWSKLDGSLPPDSRLENNMLMLPSVRPQDAGTYVCTATNRQGKVKAFAHLQVPE
RVVPYFTQTPYSFLPLPTIKDAYRKFEIKITFRPDSADGMLLYNGQKRVPGSPTNLANRQ
PDFISFGLVGGRPEFRFDAGSGMATIRHPTPLALGHFHTVTLLRSLTQGSLIVGDLAPVN
GTSQGKFQGLDLNEELYLGGYPDYGAIPKAGLSSGFIGCVRELRIQGEEIVFHDLNLTAH
GISHCPTCRDRPCQNGGQCHDSESSSYVCVCPAGFTGSRCEHSQALHCHPEACGPDATCV
NRPDGRGYTCRCHLGRSGLRCEEGVTVTTPSLSGAGSYLALPALTNTHHELRLDVEFKPL
APDGVLLFSGGKSGPVEDFVSLAMVGGHLEFRYELGSGLAVLRSAEPLALGRWHRVSAER
LNKDGSLRVNGGRPVLRSSPGKSQGLNLHTLLYLGGVEPSVPLSPATNMSAHFRGCVGEV
SVNGKRLDLTYSFLGSQGIGQCYDSSPCERQPCQHGATCMPAGEYEFQCLCRDGFKGDLC
EHEENPCQLREPCLHGGTCQGTRCLCLPGFSGPRCQQGSGHGIAESDWHLEGSGGNDAPG
QYGAYFHDDGFLAFPGHVFSRSLPEVPETIELEVRTSTASGLLLWQGVEVGEAGQGKDFI
SLGLQDGHLVFRYQLGSGEARLVSEDPINDGEWHRVTALREGRRGSIQVDGEELVSGRSP
GPNVAVNAKGSVYIGGAPDVATLTGGRFSSGITGCVKNLVLHSARPGAPPPQPLDLQHRA
QAGANTRPCPS
>sp|P07996|TSP1_HUMAN Thrombospondin-1 OS=Homo sapiens OX=9606 GN=THBS1 PE=1
SV=2
MGLAWGLGVLFLMHVCGTNRIPESGGDNSVFDIFELTGAARKGSGRRLVKGPDPSSPAFR
IEDANLIPPVPDDKFQDLVDAVRAEKGFLLLASLRQMKKTRGTLLALERKDHSGQVFSVV
SNGKAGTLDLSLTVQGKQHVVSVEEALLATGQWKSITLFVQEDRAQLYIDCEKMENAELD

VPIQSVFTRDLASIARLRIAKGGVNDNFQGVLQNVRFVFGTTPEDILRNKGCSSSTSVLL
TLDNNVVNGSSPAIRTNYIGHKTKDLQAICGISCDELSSMVLELRGLRTIVTTLQDSIRK
VTEENKELANELRRPPLCYHNGVQYRNNEEWTVDSCTECHCQNSVTICKKVSCPIMPCSN
ATVPDGECCPRCWPSDSADDGWSPWSEWTSCSTSCGNGIQQRGRSCDSLNNRCEGSSVQT
RTCHIQECDKRFKQDGGWSHWSPWSSCSVTCGDGVITRIRLCNSPSPQMNGKPCEGEARE
TKACKKDACPINGGWGPWSPWDICSVTCGGGVQKRSRLCNNPTPQFGGKDCVGDVTENQI
CNKQDCPIDGCLSNPCFAGVKCTSYPDGSWKCGACPPGYSGNGIQCTDVDECKEVPDACF
NHNGEHRCENTDPGYNCLPCPPRFTGSQPFGQGVEHATANKQVCKPRNPCTDGTHDCNKN
AKCNYLGHYSDPMYRCECKPGYAGNGIICGEDTDLDGWPNENLVCVANATYHCKKDNCPN
LPNSGQEDYDKDGIGDACDDDDDNDKIPDDRDNCPFHYNPAQYDYDRDDVGDRCDNCPYN
HNPDQADTDNNGEGDACAADIDGDGILNERDNCQYVYNVDQRDTDMDGVGDQCDNCPLEH
NPDQLDSDSDRIGDTCDNNQDIDEDGHQNNLDNCPYVPNANQADHDKDGKGDACDHDDDN
DGIPDDKDNCRLVPNPDQKDSDGDGRGDACKDDFDHDSVPDIDDICPENVDISETDFRRF
QMIPLDPKGTSQNDPNWVVRHQGKELVQTVNCDPGLAVGYDEFNAVDFSGTFFINTERDD
DYAGFVFGYQSSSRFYVVMWKQVTQSYWDTNPTRAQGYSGLSVKVVNSTTGPGEHLRNAL
WHTGNTPGQVRTLWHDPRHIGWKDFTAYRWRLSHRPKTGFIRVVMYEGKKIMADSGPIYD
KTYAGGRLGLFVFSQEMVFFSDLKYECRDP
>sp|Q13214|SEM3B_HUMAN Semaphorin-3B OS=Homo sapiens OX=9606 GN=SEMA3B PE=1 SV=1
MGRAGAAAVIPGLALLWAVGLGSAAPSPPRLRLSFQELQAWHGLQTFSLERTCCYQALLV
DEERGRLFVGAENHVASLNLDNISKRAKKLAWPAPVEWREECNWAGKDIGTECMNFVKLL
HAYNRTHLLACGTGAFHPTCAFVEVGHRAEEPVLRLDPGRIEDGKGKSPYDPRHRAASVL
VGEELYSGVAADLMGRDFTIFRSLGQRPSLRTEPHDSRWLNEPKFVKVFWIPESENPDDD
KIYFFFRETAVEAAPALGRLSVSRVGQICRNDVGGQRSLVNKWTTFLKARLVCSVPGVEG
DTHFDQLQDVFLLSSRDHRTPLLYAVFSTSSSIFQGSAVCVYSMNDVRRAFLGPFAHKEG
PMHQWVSYQGRVPYPRPGMCPSKTFGTFSSTKDFPDDVIQFARNHPLMYNSVLPTGGRPL
FLQVGANYTFTQIAADRVAAADGHYDVLFIGTDVGTVLKVISVPKGSRPSAEGLLLEELH
VFEDSAAVTSMQISSKRHQLYVASRSAVAQIALHRCAAHGRVCTECCLARDPYCAWDGVA
CTRFQPSAKRRFRRQDVRNGDPSTLCSGDSSRPALLEHKVFGVEGSSAFLECEPRSLQAR
VEWTFQRAGVTAHTQVLAEERTERTARGLLLRRLRRRDSGVYLCAAVEQGFTQPLRRLSL
HVLSATQAERLARAEEAAPAAPPGPKLWYRDFLQLVEPGGGGSANSLRMCRPQPALQSLP
LESRRKGRNRRTHAPEPRAERGPRSATHW
>sp|P35556|FBN2_HUMAN Fibrillin-2 OS=Homo sapiens OX=9606 GN=FBN2 PE=1 SV=3
MGRRRRLCLQLYFLWLGCVVLWAQGTAGQPQPPPPKPPRPQPPPQQVRSATAGSEGGFLA
PEYREEGAAVASRVRRRGQQDVLRGPNVCGSRFHSYCCPGWKTLPGGNQCIVPICRNSCG
DGFCSRPNMCTCSSGQISSTCGSKSIQQCSVRCMNGGTCADDHCQCQKGYIGTYCGQPVC
ENGCQNGGRCIGPNRCACVYGFTGPQCERDYRTGPCFTQVNNQMCQGQLTGIVCTKTLCC
ATIGRAWGHPCEMCPAQPQPCRRGFIPNIRTGACQDVDECQAIPGICQGGNCINTVGSFE
CRCPAGHKQSETTQKCEDIDECSIIPGICETGECSNTVGSYFCVCPRGYVTSTDGSRCID
QRTGMCFSGLVNGRCAQELPGRMTKMQCCCEPGRCWGIGTIPEACPVRGSEEYRRLCMDG
LPMGGIPGSAGSRPGGTGGNGFAPSGNGNGYGPGGTGFIPIPGGNGFSPGVGGAGVGAGG
QGPIITGLTILNQTIDICKHHANLCLNGRCIPTVSSYRCECNMGYKQDANGDCIDVDECT
SNPCTNGDCVNTPGSYYCKCHAGFQRTPTKQACIDIDECIQNGVLCKNGRCVNTDGSFQC
ICNAGFELTTDGKNCVDHDECTTTNMCLNGMCINEDGSFKCICKPGFVLAPNGRYCTDVD

ECQTPGICMNGHCINSEGSFRCDCPPGLAVGMDGRVCVDTHMRSTCYGGIKKGVCVRPFP
GAVTKSECCCANPDYGFGEPCQPCPAKNSAEFHGLCSSGVGITVDGRDINECALDPDICA
NGICENLRGSYRCNCNSGYEPDASGRNCIDIDECLVNRLLCDNGLCRNTPGSYSCTCPPG
YVFRTETETCEDINECESNPCVNGACRNNLGSFNCECSPGSKLSSTGLICIDSLKGTCWL
NIQDSRCEVNINGATLKSECCATLGAAWGSPCERCELDTACPRGLARIKGVTCEDVNECE
VFPGVCPNGRCVNSKGSFHCECPEGLTLDGTGRVCLDIRMEQCYLKWDEDECIHPVPGKF
RMDACCCAVGAAWGTECEECPKPGTKEYETLCPRGAGFANRGDVLTGRPFYKDINECKAF
PGMCTYGKCRNTIGSFKCRCNSGFALDMEERNCTDIDECRISPDLCGSGICVNTPGSFEC
ECFEGYESGFMMMKNCMDIDECERNPLLCRGGTCVNTEGSFQCDCPLGHELSPSREDCVD
INECSLSDNLCRNGKCVNMIGTYQCSCNPGYQATPDRQGCTDIDECMIMNGGCDTQCTNS
EGSYECSCSEGYALMPDGRSCADIDECENNPDICDGGQCTNIPGEYRCLCYDGFMASMDM
KTCIDVNECDLNSNICMFGECENTKGSFICHCQLGYSVKKGTTGCTDVDECEIGAHNCDM
HASCLNIPGSFKCSCREGWIGNGIKCIDLDECSNGTHQCSINAQCVNTPGSYRCACSEGF
TGDGFTCSDVDECAENINLCENGQCLNVPGAYRCECEMGFTPASDSRSCQDIDECSFQNI
CVFGTCNNLPGMFHCICDDGYELDRTGGNCTDIDECADPINCVNGLCVNTPGRYECNCPP
DFQLNPTGVGCVDNRVGNCYLKFGPRGDGSLSCNTEIGVGVSRSSCCCSLGKAWGNPCET
CPPVNSTEYYTLCPGGEGFRPNPITIILEDIDECQELPGLCQGGNCINTFGSFQCECPQG
YYLSEDTRICEDIDECFAHPGVCGPGTCYNTLGNYTCICPPEYMQVNGGHNCMDMRKSFC
YRSYNGTTCENELPFNVTKRMCCCTYNVGKAWNKPCEPCPTPGTADFKTICGNIPGFTFD
IHTGKAVDIDECKEIPGICANGVCINQIGSFRCECPTGFSYNDLLLVCEDIDECSNGDNL
CQRNADCINSPGSYRCECAAGFKLSPNGACVDRNECLEIPNVCSHGLCVDLQGSYQCICH
NGFKASQDQTMCMDVDECERHPCGNGTCKNTVGSYNCLCYPGFELTHNNDCLDIDECSSF
FGQVCRNGRCFNEIGSFKCLCNEGYELTPDGKNCIDTNECVALPGSCSPGTCQNLEGSFR
CICPPGYEVKSENCIDINECDEDPNICLFGSCTNTPGGFQCLCPPGFVLSDNGRRCFDTR
QSFCFTNFENGKCSVPKAFNTTKAKCCCSKMPGEGWGDPCELCPKDDEVAFQDLCPYGHG
TVPSLHDTREDVNECLESPGICSNGQCINTDGSFRCECPMGYNLDYTGVRCVDTDECSIG
NPCGNGTCTNVIGSFECNCNEGFEPGPMMNCEDINECAQNPLLCAFRCMNTFGSYECTCP
IGYALREDQKMCKDLDECAEGLHDCESRGMMCKNLIGTFMCICPPGMARRPDGEGCVDEN
ECRTKPGICENGRCVNIIGSYRCECNEGFQSSSSGTECLDNRQGLCFAEVLQTICQMASS
SRNLVTKSECCCDGGRGWGHQCELCPLPGTAQYKKICPHGPGYTTDGRDIDECKVMPNLC
TNGQCINTMGSFRCFCKVGYTTDISGTSCIDLDECSQSPKPCNYICKNTEGSYQCSCPRG
YVLQEDGKTCKDLDECQTKQHNCQFLCVNTLGGFTCKCPPGFTQHHTACIDNNECGSQPS
LCGAKGICQNTPGSFSCECQRGFSLDATGLNCEDVDECDGNHRCQHGCQNILGGYRCGCP
QGYIQHYQWNQCVDENECSNPNACGSASCYNTLGSYKCACPSGFSFDQFSSACHDVNECS
SSKNPCNYGCSNTEGGYLCGCPPGYYRVGQGHCVSGMGFNKGQYLSLDTEVDEENALSPE
ACYECKINGYSKKDSRQKRSIHEPDPTAVEQISLESVDMDSPVNMKFNLSHLGSKEHILE
LRPAIQPLNNHIRYVISQGNDDSVFRIHQRNGLSYLHTAKKKLMPGTYTLEITSIPLYKK
KELKKLEESNEDDYLLGELGEALRMRLQIQLY
>sp|P09382|LEG1_HUMAN Galectin-1 OS=Homo sapiens OX=9606 GN=LGALS1 PE=1 SV=2
MACGLVASNLNLKPGECLRVRGEVAPDAKSFVLNLGKDSNNLCLHFNPRFNAHGDANTIV
CNSKDGGAWGTEQREAVFPFQPGSVAEVCITFDQANLTVKLPDGYEFKFPNRLNLEAINY
MAADGDFKIKCVAFD
>sp|Q6NUI6|CHADL_HUMAN Chondroadherin-like protein OS=Homo sapiens OX=9606
GN=CHADL PE=1 SV=2

MEGPRSSTHVPLVLPLLVLLLLAPARQAAAQRCPQACICDNSRRHVACRYQNLTEVPDAI
PELTQRLDLQGNLLKVIPAAAFQGVPHLTHLDLRHCEVELVAEGAFRGLGRLLLLNLASN
HLRELPQEALDGLGSLRRLELEGNALEELRPGTFGALGALATLNLAHNALVYLPAMAFQG
LLRVRWLRLSHNALSVLAPEALAGLPALRRLSLHHNELQALPGPVLSQARGLARLELGHN
PLTYAGEEDGLALPGLRELLLDGGALQALGPRAFAHCPRLHTLDLRGNQLDTLPPLQGPG
QLRRLRLQGNPLWCGCQARPLLEWLARARVRSDGACQGPRRLRGEALDALRPWDLRCPGD
AAQEEEELEERAVAGPRAPPRGPPRGPGEERAVAPCPRACVCVPESRHSSCEGCGLQAVP
RGFPSDTQLLDLRRNHFPSVPRAAFPGLGHLVSLHLQHCGIAELEAGALAGLGRLIYLYL
SDNQLAGLSAAALEGAPRLGYLYLERNRFLQVPGAALRALPSLFSLHLQDNAVDRLAPGD
LGRTRALRWVYLSGNRITEVSLGALGPARELEKLHLDRNQLREVPTGALEGLPALLELQL
SGNPLRALRDGAFQPVGRSLQHLFLNSSGLEQICPGAFSGLGPGLQSLHLQKNQLRALPA
LPSLSQLELIDLSSNPFHCDCQLLPLHRWLTGLNLRVGATCATPPNARGQRVKAAAAVFE
DCPGWAARKAKRTPASRPSARRTPIKGRQCGADKVGKEKGRL
>sp|Q6UY14|ATL4_HUMAN ADAMTS-like protein 4 OS=Homo sapiens OX=9606 GN=ADAMTSL4
PE=1 SV=2
MENWTGRPWLYLLLLLSLPQLCLDQEVLSGHSLQTPTEEGQGPEGVWGPWVQWASCSQPC
GVGVQRRSRTCQLPTVQLHPSLPLPPRPPRHPEALLPRGQGPRPQTSPETLPLYRTQSRG
RGGPLRGPASHLGREETQEIRAARRSRLRDPIKPGMFGYGRVPFALPLHRNRRHPRSPPR
SELSLISSRGEEAIPSPTPRAEPFSANGSPQTELPPTELSVHTPSPQAEPLSPETAQTEV
APRTRPAPLRHHPRAQASGTEPPSPTHSLGEGGFFRASPQPRRPSSQGWASPQVAGRRPD
PFPSVPRGRGQQGQGPWGTGGTPHGPRLEPDPQHPGAWLPLLSNGPHASSLWSLFAPSSP
IPRCSGESEQLRACSQAPCPPEQPDPRALQCAAFNSQEFMGQLYQWEPFTEVQGSQRCEL
NCRPRGFRFYVRHTEKVQDGTLCQPGAPDICVAGRCLSPGCDGILGSGRRPDGCGVCGGD
DSTCRLVSGNLTDRGGPLGYQKILWIPAGALRLQIAQLRPSSNYLALRGPGGRSIINGNW
AVDPPGSYRAGGTVFRYNRPPREEGKGESLSAEGPTTQPVDVYMIFQEENPGVFYQYVIS
SPPPILENPTPEPPVPQLQPEILRVEPPLAPAPRPARTPGTLQRQVRIPQMPAPPHPRTP
LGSPAAYWKRVGHSACSASCGKGVWRPIFLCISRESGEELDERSCAAGARPPASPEPCHG
TPCPPYWEAGEWTSCSRSCGPGTQHRQLQCRQEFGGGGSSVPPERCGHLPRPNITQSCQL
RLCGHWEVGSPWSQCSVRCGRGQRSRQVRCVGNNGDEVSEQECASGPPQPPSREACDMGP
CTTAWFHSDWSSKCSAECGTGIQRRSVVCLGSGAALGPGQGEAGAGTGQSCPTGSRPPDM
RACSLGPCERTWRWYTGPWGECSSECGSGTQRRDIICVSKLGTEFNVTSPSNCSHLPRPP
ALQPCQGQACQDRWFSTPWSPCSRSCQGGTQTREVQCLSTNQTLSTRCPPQLRPSRKRPC
NSQPCSQRPDDQCKDSSPHCPLVVQARLCVYPYYTATCCRSCAHVLERSPQDPS
>sp|O95715|CXL14_HUMAN C-X-C motif chemokine 14 OS=Homo sapiens OX=9606
GN=CXCL14 PE=1 SV=2
MSLLPRRAPPVSMRLLAAALLLLLLALYTARVDGSKCKCSRKGPKIRYSDVKKLEMKPKY
PHCEEKMVIITTKSVSRYRGQEHCLHPKLQSTKRFIKWYNAWNEKRRVYEE
>sp|A1KZ92|PXDNL_HUMAN Probable oxidoreductase PXDNL OS=Homo sapiens OX=9606
GN=PXDNL PE=1 SV=3
MEPRLFCWTTLFLLAGWCLPGLPCPSRCLCFKSTVRCMHLMLDHIPQVPQQTTVLDLRFN
RIREIPGSAFKKLKNLNTLLLNNNHIRKISRNAFEGLENLLYLYLYKNEIHALDKQTFKG
LISLEHLYIHFNQLEMLQPETFGDLLRLERLFLHNNKLSKIPAGSFSNLDSLKRLRLDSN
ALVCDCDLMWLGELLQGFAQHGHTQAAATCEYPRRLHGRAVASVTVEEFNCQSPRITFEP
QDVEVPSGNTVYFTCRAEGNPKPEIIWIHNNHSLDLEDDTRLNVFDDGTLMIRNTRESDQ

GVYQCMARNSAGEAKTQSAMLRYSSLPAKPSFVIQPQDTEVLIGTSTTLECMATGHPHPL
ITWTRDNGLELDGSRHVATSSGLYLQNITQRDHGRFTCHANNSHGTVQAAANIIVQAPPQ
FTVTPKDQVVLEEHAVEWLCEADGNPPPVIVWTKTGGQLPVEGQHTVLSSGTLRIDRAAQ
HDQGQYECQAVSSLGVKKVSVQLTVKPKALAVFTQLPQDTSVEVGKNINISCHAQGEPQP
IITWNKEGVQITESGKFHVDDEGTLTIYDAGFPDQGRYECVARNSFGLAVTNMFLTVTAI
QGRQAGDDFVESSILDAVQRVDSAINSTRRHLFSQKPHTSSDLLAQFHYPRDPLIVEMAR
AGEIFEHTLQLIRERVKQGLTVDLEGKEFRYNDLVSPRSLSLIANLSGCTARRPLPNCSN
RCFHAKYRAHDGTCNNLQQPTWGAALTAFARLLQPAYRDGIRAPRGLGLPVGSRQPLPPP
RLVATVWARAAAVTPDHSYTRMLMHWGWFLEHDLDHTVPALSTARFSDGRPCSSVCTNDP
PCFPMNTRHADPRGTHAPCMLFARSSPACASGRPSATVDSVYAREQINQQTAYIDGSNVY
GSSERESQALRDPSVPRGLLKTGFPWPPSGKPLLPFSTGPPTECARQEQESPCFLAGDHR
ANEHLALAAMHTLWFREHNRMATELSALNPHWEGNTVYQEARKIVGAELQHITYSHWLPK
VLGDPGTRMLRGYRGYNPNVNAGIINSFATAAFRFGHTLINPILYRLNATLGEISEGHLP
FHKALFSPSRIIKEGGIDPVLRGLFGVAAKWRAPSYLLSPELTQRLFSAAYSAAVDSAAT
IIQRGRDHGIPPYVDFRVFCNLTSVKNFEDLQNEIKDSEIRQKLRKLYGSPGDIDLWPAL
MVEDLIPGTRVGPTLMCLFVTQFQRLRDGDRFWYENPGVFTPAQLTQLKQASLSRVLCDN
GDSIQQVQADVFVKAEYPQDYLNCSEIPKVDLRVWQDCCADCRSRGQFRAVTQESQKKRS
AQYSYPVDKDMELSHLRSRQQDKIYVGEDARNVTVLAKTKFSQDFSTFAAEIQETITALR
EQINKLEARLRQAGCTDVRGVPRKAEERWMKEDCTHCICESGQVTCVVEICPPAPCPSPE
LVKGTCCPVCRDRGMPSDSPEKR
>sp|P02675|FIBB_HUMAN Fibrinogen beta chain OS=Homo sapiens OX=9606 GN=FGB PE=1
SV=2
MKRMVSWSFHKLKTMKHLLLLLLCVFLVKSQGVNDNEEGFFSARGHRPLDKKREEAPSLR
PAPPPISGGGYRARPAKAAATQKKVERKAPDAGGCLHADPDLGVLCPTGCQLQEALLQQE
RPIRNSVDELNNNVEAVSQTSSSSFQYMYLLKDLWQKRQKQVKDNENVVNEYSSELEKHQ
LYIDETVNSNIPTNLRVLRSILENLRSKIQKLESDVSAQMEYCRTPCTVSCNIPVVSGKE
CEEIIRKGGETSEMYLIQPDSSVKPYRVYCDMNTENGGWTVIQNRQDGSVDFGRKWDPYK
QGFGNVATNTDGKNYCGLPGEYWLGNDKISQLTRMGPTELLIEMEDWKGDKVKAHYGGFT
VQNEANKYQISVNKYRGTAGNALMDGASQLMGENRTMTIHNGMFFSTYDRDNDGWLTSDP
RKQCSKEDGGGWWYNRCHAANPNGRYYWGGQYTWDMAKHGTDDGVVWMNWKGSWYSMRKM
SMKIRPFFPQQ
>sp|P02671|FIBA_HUMAN Fibrinogen alpha chain OS=Homo sapiens OX=9606 GN=FGA PE=1
SV=2
MFSMRIVCLVLSVVGTAWTADSGEGDFLAEGGGVRGPRVVERHQSACKDSDWPFCSDEDW
NYKCPSGCRMKGLIDEVNQDFTNRINKLKNSLFEYQKNNKDSHSLTTNIMEILRGDFSSA
NNRDNTYNRVSEDLRSRIEVLKRKVIEKVQHIQLLQKNVRAQLVDMKRLEVDIDIKIRSC
RGSCSRALAREVDLKDYEDQQKQLEQVIAKDLLPSRDRQHLPLIKMKPVPDLVPGNFKSQ
LQKVPPEWKALTDMPQMRMELERPGGNEITRGGSTSYGTGSETESPRNPSSAGSWNSGSS
GPGSTGNRNPGSSGTGGTATWKPGSSGPGSTGSWNSGSSGTGSTGNQNPGSPRPGSTGTW
NPGSSERGSAGHWTSESSVSGSTGQWHSESGSFRPDSPGSGNARPNNPDWGTFEEVSGNV
SPGTRREYHTEKLVTSKGDKELRTGKEKVTSGSTTTTRRSCSKTVTKTVIGPDGHKEVTK
EVVTSEDGSDCPEAMDLGTLSGIGTLDGFRHRHPDEAAFFDTASTGKTFPGFFSPMLGEF
VSETESRGSESGIFTNTKESSSHHPGIAEFPSRGKSSSYSKQFTSSTSYNRGDSTFESKS
YKMADEAGSEADHEGTHSTKRGHAKSRPVRDCDDVLQTHPSGTQSGIFNIKLPGSSKIFS

VYCDQETSLGGWLLIQQRMDGSLNFNRTWQDYKRGFGSLNDEGEGEFWLGNDYLHLLTQR
GSVLRVELEDWAGNEAYAEYHFRVGSEAEGYALQVSSYEGTAGDALIEGSVEEGAEYTSH
NNMQFSTFDRDADQWEENCAEVYGGGWWYNNCQAANLNGIYYPGGSYDPRNNSPYEIENG
VVWVSFRGADYSLRAVRMKIRPLVTQ
>sp|P02679|FIBG_HUMAN Fibrinogen gamma chain OS=Homo sapiens OX=9606 GN=FGG PE=1 SV=3
MSWSLHPRNLILYFYALLFLSSTCVAYVATRDNCCILDERFGSYCPTTCGIADFLSTYQT
KVDKDLQSLEDILHQVENKTSEVKQLIKAIQLTYNPDESSKPNMIDAATLKSRKMLEEIM
KYEASILTHDSSIRYLQEIYNSNNQKIVNLKEKVAQLEAQCQEPCKDTVQIHDITGKDCQ
DIANKGAKQSGLYFIKPLKANQQFLVYCEIDGSGNGWTVFQKRLDGSVDFKKNWIQYKEG
FGHLSPTGTTEFWLGNEKIHLISTQSAIPYALRVELEDWNGRTSTADYAMFKVGPEADKY
RLTYAYFAGGDAGDAFDGFDFGDDPSDKFFTSHNGMQFSTWDNDNDKFEGNCAEQDGSGW
WMNKCHAGHLNGVYYQGGTYSKASTPNGYDNGIIWATWKTRWYSMKKTTMKIIPFNRLTI
GEGQQHHLGGAKQVRPEHPAETEYDSLYPEDDL
>sp|P08572|CO4A2_HUMAN Collagen alpha-2(IV) chain OS=Homo sapiens OX=9606 GN=COL4A2 PE=1 SV=4
MGRDQRAVAGPALRRWLLLGTVTVGFLAQSVLAGVKKFDVPCGGRDCSGGCQCYPEKGGR
GQPGPVGPQGYNGPPGLQGFPGLQGRKGDKGERGAPGVTGPKGDVGARGVSGFPGADGIP
GHPGQGGPRGRPGYDGCNGTQGDSGPQGPPGSEGFTGPPGPQGPKGQKGEPYALPKEERD
RYRGEPGEPGLVGFQGPPGRPGHVGQMGPVGAPGRPGPPGPPGPKGQQGNRGLGFYGVKG
EKGDVGQPGPNGIPSDTLHPIIAPTGVTFHPDQYKGEKGSEGEPGIRGISLKGEEGIMGF
PGLRGYPGLSGEKGSPGQKGSRGLDGYQGPDGPRGPKGEAGDPGPPGLPAYSPHPSLAKG
ARGDPGFPGAQGEPGSQGEPGDPGLPGPPGLSIGDGDQRRGLPGEMGPKGFIGDPGIPAL
YGGPPGPDGKRGPPGPPGLPGPPGPDGFLFGLKGAKGRAGFPGLPGSPGARGPKGWKGDA
GECRCTEGDEAIKGLPGLPGPKGFAGINGEPGRKGDRGDPGQHGLPGFPGLKGVPGNIGA
PGPKGAKGDSRTITTKGERGQPGVPGVPGMKGDDGSPGRDGLDGFPGLPGPPGDGIKGPP
GDPGYPGIPGTKGTPGEMGPPGLGLPGLKGQRGFPGDAGLPGPPGFLGPPGPAGTPGQID
CDTDVKRAVGGDRQEAIQPGCIGGPKGLPGLPGPPGPTGAKGLRGIPGFAGADGGPGPRG
LPGDAGREGFPGPPGFIGPRGSKGAVGLPGPDGSPGPIGLPGPDGPPGERGLPGEVLGAQ
PGPRGDAGVPGQPGLKGLPGDRGPPGFRGSQGMPGMPGLKGQPGLPGPSGQPGLYGPPGL
HGFPGAPGQEGPLGLPGIPGREGLPGDRGDPGDTGAPGPVGMKGLSGDRGDAGFTGEQGH
PGSPGFKGIDGMPGTPGLKGDRGSPGMDGFQGMPGLKGRPGFPGSKGEAGFFGIPGLKGL
AGEPGFKGSRGDPGPPGPPPVILPGMKDIKGEKGDEGPMGLKGYLGAKGIQGMPGIPGLS
GIPGLPGRPGHIKGVKGDIGVPGIPGLPGFPGVAGPPGITGFPGFIGSRGDKGAPGRAGL
YGEIGATGDFGDIGDTINLPGRPGLKGERGTTGIPGLKGFFGEKGTEGDIGFPGITGVTG
VQGPPGLKGQTGFPGLTGPPGSQGELGRIGLPGGKGDDGWPGAPGLPGFPGLRGIRGLHG
LPGTKGFPGSPGSDIHGDPGFPGPPGERGDPGEANTLPGPVGVPGQKGDQGAPGERGPPG
SPGLQGFPGITPPSNISGAPGDKGAPGIFGLKGYRGPPGPPGSAALPGSKGDTGNPGAPG
TPGTKGWAGDSGPQGRPGVFGLPGEKGPRGEQGFMGNTGPTGAVGDRGPKGPKGDPGFPG
APGTVGAPGIAGIPQKIAVQPGTVGPQGRRGPPGAPGEMGPQGPPGEPGFRGAPGKAGPQ
GRGGVSAVPGFRGDEGPIGHQGPIGQEGAPGRPGSPGLPGMPGRSVSIGYLLVKHSQTDQ
EPMCPVGMNKLWSGYSLLYFEGQEKAHNQDLGLAGSCLARFSTMPFLYCNPGDVCYYASR
NDKSYWLSTTAPLPMMPVAEDEIKPYISRCSVCEAPAIAIAVHSQDVSIPHCPAGWRSLW
IGYSFLMHTAAGDEGGGQSLVSPGSCLEDFRATPFIECNGGRGTCHYYANKYSFWLTTIP

EQSFQGSPSADTLKAGLIRTHISRCQVCMKNL
>sp|O75095|MEGF6_HUMAN Multiple epidermal growth factor-like domains protein 6 OS=Homo sapiens OX=9606 GN=MEGF6 PE=1 SV=4
MSFLEEARAAGRAVVLALVLLLLPAVPVGASVPPRPLLPLQPGMPHVCAEQELTLVGRRQ
PCVQALSHTVPVWKAGCGWQAWCVGHERRTVYYMGYRQVYTTEARTVLRCCRGWMQQPDE
EGCLSAECSASLCFHGGRCVPGSAQPCHCPPGFQGPRCQYDVDECRTHNGGCQHRCVNTP
GSYLCECKPGFRLHTDSRTCLAINSCALGNGGCQHHCVQLTITRHRCQCRPGFQLQEDGR
HCVRRSPCANRNGSCMHRCQVVRGLARCECHVGYQLAADGKACEDVDECAAGLAQCAHGC
LNTQGSFKCVCHAGYELGADGRQCYRIEMEIVNSCEANNGGCSHGCSHTSAGPLCTCPRG
YELDTDQRTCIDVDDCADSPCCQQVCTNNPGGYECGCYAGYRLSADGCGCEDVDECASSR
GGCEHHCTNLAGSFQCSCEAGYRLHEDRRGCSPLEEPMVDLDGELPFVRPLPHIAVLQDE
LPQLFQDDDVGADEEEAELRGEHTLTEKFVCLDDSFGHDCSLTCDDCRNGGTCLLGLDGC
DCPEGWTGLICNETCPPDTFGKNCSFSCSCQNGGTCDSVTGACRCPPGVSGTNCEDGCPK
GYYGKHCRKKCNCANRGRCHRLYGACLCDPGLYGRFCHLTCPPWAFGPGCSEECQCVQPH
TQSCDKRDGSCSCKAGFRGERCQAECELGYFGPGCWQACTCPVGVACDSVSGECGKRCPA
GFQGEDCGQECPVGTFGVNCSSSCSCGGAPCHGVTGQCRCPPGRTGEDCEADCPEGRWGL
GCQEICPACQHAARCDPETGACLCLPGFVGSRCQDVCPAGWYGPSCQTRCSCANDGHCHP
ATGHCSCAPGWTGFSCQRACDTGHWGPDCSHPCNCSAGHGSCDAISGLCLCEAGYVGPRC
EQQCPQGHFGPGCEQRCQCQHGAACDHVSGACTCPAGWRGTFCEHACPAGFFGLDCRSAC
NCTAGAACDAVNGSCLCPAGRRGPRCAETCPAHTYGHNCSQACACFNGASCDPVHGQCHC
APGWMGPSCLQACPAGLYGDNCRHSCLCQNGGTCDPVSGHCACPEGWAGLACEKECLPRD
VRAGCRHSGGCLNGGLCDPHTGRCLCPAGWTGDKCQSPCLRGWFGEACAQRCSCPPGAAC
HHVTGACRCPPGFTGSGCEQACPPGSFGEDCAQMCQCPGENPACHPATGTCSCAAGYHGP
SCQQRCPPGRYGPGCEQLCGCLNGGSCDAATGACRCPTGFLGTDCNLTCPQGRFGPNCTH
VCGCGQGAACDPVTGTCLCPPGRAGVRCERGCPQNRFGVGCEHTCSCRNGGLCHASNGSC
SCGLGWTGRHCELACPPGRYGAACHLECSCHNNSTCEPATGTCRCGPGFYGQACEHPCPP
GFHGAGCQGLCWCQHGAPCDPISGRCLCPAGFHGHFCERGCEPGSFGEGCHQRCDCDGGA
PCDPVTGLCLCPPGRSGATCNLDCRRGQFGPSCTLHCDCGGGADCDPVSGQCHCVDGYMG
PTCREGGPLRLPENPSLAQGSAGTLPASSRPTSRSGGPARH
>sp|Q9NS15|LTBP3_HUMAN Latent-transforming growth factor beta-binding protein 3 OS=Homo sapiens OX=9606 GN=LTBP3 PE=1 SV=4
MPGPRGAAGGLAPEMRGAGAAGLLALLLLLLLLLLGLGGRVEGGPAGERGAGGGGALARE
RFKVVFAPVICKRTCLKGQCRDSCQQGSNMTLIGENGHSTDTLTGSGFRVVVCPLPCMNG
GQCSSRNQCLCPPDFTGRFCQVPAGGAGGGTGGSGPGLSRTGALSTGALPPLAPEGDSVA
SKHAIYAVQVIADPPGPGEGPPAQHAAFLVPLGPGQISAEVQAPPPVVNVRVHHPPEASV
QVHRIESSNAESAAPSQHLLPHPKPSHPRPPTQKPLGRCFQDTLPKQPCGSNPLPGLTKQ
EDCCGSIGTAWGQSKCHKCPQLQYTGVQKPGPVRGEVGADCPQGYKRLNSTHCQDINECA
MPGVCRHGDCLNNPGSYRCVCPPGHSLGPSRTQCIADKPEEKSLCFRLVSPEHQCQHPLT
TRLTRQLCCCSVGKAWGARCQRCPTDGTAAFKEICPAGKGYHILTSHQTLTIQGESDFSL
FLHPDGPPKPQQLPESPSQAPPPEDTEEERGVTTDSPVSEERSVQQSHPTATTTPARPYP
ELISRPSPPTMRWFLPDLPPSRSAVEIAPTQVTETDECRLNQNICGHGECVPGPPDYSCH
CNPGYRSHPQHRYCVDVNECEAEPCGPGRGICMNTGGSYNCHCNRGYRLHVGAGGRSCVD
LNECAKPHLCGDGGFCINFPGHYKCNCYPGYRLKASRPPVCEDIDECRDPSSCPDGKCEN
KPGSFKCIACQPGYRSQGGGACRDVNECAEGSPCSPGWCENLPGSFRCTCAQGYAPAPDG

RSCLDVDECEAGDVCDNGICSNTPGSFQCQCLSGYHLSRDRSHCEDIDECDFPAACIGGD
CINTNGSYRCLCPQGHRLVGGRKCQDIDECSQDPSLCLPHGACKNLQGSYVCVCDEGFTP
TQDQHGCEEVEQPHHKKECYLNFDDTVFCDSVLATNVTQQECCCSLGAGWGDHCEIYPCP
VYSSAEFHSLCPDGKGYTQDNNIVNYGIPAHRDIDECMLFGSEICKEGKCVNTQPGYECY
CKQGFYYDGNLLECVDVDECLDESNCRNGVCENTRGGYRCACTPPAEYSPAQRQCLSPEE
MDVDECQDPAACRPGRCVNLPGSYRCECRPPWVPGPSGRDCQLPESPAERAPERRDVCWS
QRGEDGMCAGPLAGPALTFDDCCCRQGRGWGAQCRPCPPRGAGSHCPTSQSESNSFWDTS
PLLLGKPPRDEDSSEEDSDECRCVSGRCVPRPGGAVCECPGGFQLDASRARCVDIDECRE
LNQRGLLCKSERCVNTSGSFRCVCKAGFARSRPHGACVPQRRR
>sp|P04004|VTNC_HUMAN Vitronectin OS=Homo sapiens OX=9606 GN=VTN PE=1 SV=1
MAPLRPLLILALLAWVALADQESCKGRCTEGFNVDKKCQCDELCSYYQSCCTDYTAECKP
QVTRGDVFTMPEDEYTVYDDGEEKNNATVHEQVGGPSLTSDLQAQSKGNPEQTPVLKPEE
EAPAPEVGASKPEGIDSRPETLHPGRPQPPAEEELCSGKPFDAFTDLKNGSLFAFRGQYC
YELDEKAVRPGYPKLIRDVWGIEGPIDAAFTRINCQGKTYLFKGSQYWRFEDGVLDPDYP
RNISDGFDGIPDNVDAALALPAHSYSGRERVYFFKGKQYWEYQFQHQPSQEECEGSSLSA
VFEHFAMMQRDSWEDIFELLFWGRTSAGTRQPQFISRDWHGVPGQVDAAMAGRIYISGMA
PRPSLAKKQRFRHRNRKGYRSQRGHSRGRNQNSRRPSRATWLSLFSSEESNLGANNYDDY
RMDWLVPATCEPIQSVFFFSGDKYYRVNLRTRRVDTVDPPYPRSIAQYWLGCPAPGHL
>sp|Q13361|MFAP5_HUMAN Microfibrillar-associated protein 5 OS=Homo sapiens OX=9606
GN=MFAP5 PE=1 SV=1
MSLLGPKVLLFLAAFIITSDWIPLGVNSQRGDDVTQATPETFTEDPNLVNDPATDETVLA
VLADIAPSTDDLASLSEKNTTAECWDEKFTCTRLYSVHRPVKQCIHQLCFTSLRRMYIVN
KEICSRLVCKEHEAMKDELCRQMAGLPPRRLRRSNYFRLPPCENVDLQRPNGL
>sp|Q9BU40|CRDL1_HUMAN Chordin-like protein 1 OS=Homo sapiens OX=9606 GN=CHRDL1
PE=1 SV=2
MRKKWKMGGMKYIFSLLFFLLLLEGGKTEQVKHSETYCMFQDKKYRVGERWHPYLEPYGLV
YCVNCICSENGNVLCSRVRCPNVHCLSPVHIPHLCCPRCPDSLPPVNNKVTSKSCEYNGT
TYQHGELFVAEGLFQNRQPNQCTQCSCSEGNVYCGLKTCPKLTCAFPVSVPDSCCRVCRG
DGELSWEHSDGDIFRQPANREARHSYHRSHYDPPPSRQAGGLSRFPGARSHRGALMDSQQ
ASGTIVQIVINNKHKHGQVCVSNGKTYSHGESWHPNLRAFGIVECVLCTCNVTKQECKKI
HCPNRYPCKYPQKIDGKCCKVCPGKKAKELPGQSFDNKGYFCGEETMPVYESVFMEDGET
TRKIALETERPPQVEVHVWTIRKGILQHFHIEKISKRMFEELPHFKLVTRTTLSQWKIFT
EGEAQISQMCSSRVCRTELEDLVKVLYLERSEKGHC
>sp|Q08397|LOXL1_HUMAN Lysyl oxidase homolog 1 OS=Homo sapiens OX=9606 GN=LOXL1
PE=1 SV=2
MALARGSRQLGALVWGACLCVLVHGQQAQPGQGSDPARWRQLIQWENNGQVYSLLNSGSE
YVPAGPQRSESSSRVLLAGAPQAQQRRSHGSPRRRQAPSLPLPGRVGSDTVRGQARHPFG
FGQVPDNWREVAVGDSTGMARARTSVSQQRHGGSASSVSASAFASTYRQQPSYPQQFPYP
QAPFVSQYENYDPASRTYDQGFVYYRPAGGGVGAGAAAVASAGVIYPYQPRARYEEYGGG
EELPEYPPQGFYPAPERPYVPPPPPPPDGLDRRYSHSLYSEGTPGFEQAYPDPGPEAAQA
HGGDPRLGWYPPYANPPPEAYGPPRALEPPYLPVRSSDTPPPGGERNGAQQGRLSVGSVY
RPNQNGRGLPDLVPDPNYVQASTYVQRAHLYSLRCAAEEKCLASTAYAPEATDYDVRVLL
RFPQRVKNQGTADFLPNRPRHTWEWHSCHQHYHSMDEFSHYDLLDAATGKKVAEGHKASF
CLEDSTCDFGNLKRYACTSHTQGLSPGCYDTYNADIDCQWIDITDVQPGNYILKVHVNPK

YIVLESDFTNNVVRCNIHYTGRYVSATNCKIVQS
>sp|Q9UHF1|EGFL7_HUMAN Epidermal growth factor-like protein 7 OS=Homo sapiens OX=9606 GN=EGFL7 PE=1 SV=3
MRGSQEVLLMWLLVLAVGGTEHAYRPGRRVCAVRAHGDPVSESFVQRVYQPFLTTCDGHR
ACSTYRTIYRTAYRRSPGLAPARPRYACCPGWKRTSGLPGACGAAICQPPCRNGGSCVQP
GRCRCPAGWRGDTCQSDVDECSARRGGCPQRCVNTAGSYWCQCWEGHSLSADGTLCVPKG
GPPRVAPNPTGVDSAMKEEVQRLQSRVDLLEEKLQLVLAPLHSLASQALEHGLPDPGSLL
VHSFQQLGRIDSLSEQISFLEEQLGSCSCKKDS
>sp|P07355|ANXA2_HUMAN Annexin A2 OS=Homo sapiens OX=9606 GN=ANXA2 PE=1 SV=2
MSTVHEILCKLSLEGDHSTPPSAYGSVKAYTNFDAERDALNIETAIKTKGVDEVTIVNIL
TNRSNAQRQDIAFAYQRRTKKELASALKSALSGHLETVILGLLKTPAQYDASELKASMKG
LGTDEDSLIEIICSRTNQELQEINRVYKEMYKTDLEKDIISDTSGDFRKLMVALAKGRRA
EDGSVIDYELIDQDARDLYDAGVKRKGTDVPKWISIMTERSVPHLQKVFDRYKSYSPYDM
LESIRKEVKGDLENAFLNLVQCIQNKPLYFADRLYDSMKGKGTRDKVLIRIMVSRSEVDM
LKIRSEFKRKYGKSLYYYIQQDTKGDYQKALLYLCGGDD
>sp|P39060|COIA1_HUMAN Collagen alpha-1(XVIII) chain OS=Homo sapiens OX=9606 GN=COL18A1 PE=1 SV=5
MAPYPCGCHILLLLLFCCLAAARANLLNLNWLWFNNEDTSHAATTIPEPQGPLPVQPTADT
TTHVTPRNGSTEPATAPGSPEPPSELLEDGQDTPTSAESPDAPEENIAGVGAEILNVAKG
IRSFVQLWNDTVPTESLARAETLVLETPVGPLALAGPSSTPQENGTTLWPSRGIPSSPGA
HTTEAGTLPAPTPSPPSLGRPWAPLTGPSVPPPSSGRASLSSLLGGAPPWGSLQDPDSQG
LSPAAAAPSQQLQRPDVRLRTPLLHPLVMGSLGKHAAPSAFSSGLPGALSQVAVTTLTRD
SGAWVSHVANSVGPGLANNSALLGADPEAPAGRCLPLPPSLPVCGHLGISRFWLPNHLHH
ESGEQVRAGARAWGGLLQTHCHPFLAWFFCLLLVPPCGSVPPPAPPPCCQFCEALQDACW
SRLGGGRLPVACASLPTQEDGYCVLIGPAAERISEEVGLLQLLGDPPPQQVTQTDDPDVG
LAYVFGPDANSGQVARYHFPSLFFRDFSLLFHIRPATEGPGVLFAITDSAQAMVLLGVKL
SGVQDGHQDISLLYTEPGAGQTHTAASFRLPAFVGQWTHLALSVAGGFVALYVDCEEFQR
MPLARSSRGLELEPGAGLFVAQAGGADPDKFQGVIAELKVRRDPQVSPMHCLDEEGDDSD
GASGDSGSGLGDARELLREETGAALKPRLPAPPPVTTPPLAGGSSTEDSRSEEVEEQTTV
ASLGAQTLPGSDSVSTWDGSVRTPGGRVKEGGLKGQKGEPGVPGPPGRAGPPGSPCLPGP
PGLPCPVSPLGPAGPALQTVPGPQGPPGPPGRDGTPGRDGEPGDPGEDGKPGDTGPQGFP
GTPGDVGPKGDKGDPGVGERGPPGPQGPPGPPGPSFRHDKLTFIDMEGSGFGGDLEALRG
PRGFPGPPGPPGVPGLPGEPGRFGVNSSDVPGPAGLPGVPGREGPPGFPGLPGPPGPPGR
EGPPGRTGQKGSLGEAGAPGHKGSKGAPGPAGARGESGLAGAPGPAGPPGPPGPPGPPGP
GLPAGFDDMEGSGGPFWSTARSADGPQGPPGLPGLKGDPGVPGLPGAKGEVGADGVPGFP
GLPGREGIAGPQGPKGDRGSRGEKGDPGKDGVGQPGLPGPPGPPGPVVYVSEQDGSVLSV
PGPEGRPGFAGFPGPAGPKGNLGSKGERGSPGPKGEKGEPGSIFSPDGGALGPAQKGAKG
EPGFRGPPGPYGRPGYKGEIGFPGRPGRPGMNGLKGEKGEPGDASLGFGMRGMPGPPGPP
GPPGPPGTPVYDSNVFAESSRPGPPGLPGNQGPPGPKGAKGEVGPPGPPGQFPFDFLQLE
AEMKGEKGDRGDAGQKGERGEPGGGGFFGSSLPGPPGPPGPPGPRGYPGIPGPKGESIRG
QPGPPGPQGPPGIGYEGRQGPPGPPGPPGPPSFPGPHRQTISVPGPPGPPGPPGPPGTMG
ASSGVRLWATRQAMLGQVHEVPEGWLIFVAEQEELYVRVQNGFRKVQLEARTPLPRGTDN
EVAALQPPVVQLHDSNPYPRREHPHPTARPWRADDILASPPRLPEPQPYPGAPHHSSYVH
LRPARPTSPPAHSHRDFQPVLHLVALNSPLSGGMRGIRGADFQCFQQARAVGLAGTFRAF

LSSRLQDLYSIVRRADRAAVPIVNLKDELLFPSWEALFSGSEGPLKPGARIFSFDGKDVL
RHPTWPQKSVWHGSDPNGRRLTESYCETWRTEAPSATGQASSLLGGRLLGQSAASCHHAY
IVLCIENSFMTASK
>sp|P61812|TGFB2_HUMAN Transforming growth factor beta-2 proprotein OS=Homo sapiens OX=9606 GN=TGFB2 PE=1 SV=1
MHYCVLSAFLILHLVTVALSLSTCSTLDMDQFMRKRIEAIRGQILSKLKLTSPPEDYPEP
EEVPPEVISIYNSTRDLLQEKASRRAAACERERSDEEYYAKEVYKIDMPPFFPSENAIPP
TFYRPYFRIVRFDVSAMEKNASNLVKAEFRVFRLQNPKARVPEQRIELYQILKSKDLTSP
TQRYIDSKVVKTRAEGEWLSFDVTDAVHEWLHHKDRNLGFKISLHCPCCTFVPSNNYIIP
NKSEELEARFAGIDGTSTYTSGDQKTIKSTRKKNSGKTPHLLLMLLPSYRLESQQTNRRK
KRALDAAYCFRNVQDNCCLRPLYIDFKRDLGWKWIHEPKGYNANFCAGACPYLWSSDTQH
SRVLSLYNTINPEASASPCCVSQDLEPLTILYYIGKTPKIEQLSNMIVKSCKCS
>sp|Q13201|MMRN1_HUMAN Multimerin-1 OS=Homo sapiens OX=9606 GN=MMRN1 PE=1 SV=3
MKGARLFVLLSSLWSGGIGLNNSKHSWTIPEDGNSQKTMPSASVPPNKIQSLQILPTTRV
MSAEIATTPEARTSEDSLLKSTLPPSETSAPAEGVRNQTLTSTEKAEGVVKLQNLTLPTN
ASIKFNPGAESVVLSNSTLKFLQSFARKSNEQATSLNTVGGTGGIGGVGGTGGVGNRAPR
ETYLSRGDSSSSQRTDYQKSNFETTRGKNWCAYVHTRLSPTVILDNQVTYVPGGKGPCGW
TGGSCPQRSQKISNPVYRMQHKIVTSLDWRCCPGYSGPKCQLRAQEQQSLIHTNQAESHT
AVGRGVAEQQQQQGCGDPEVMQKMTDQVNYQAMKLTLLQKKIDNISLTVNDVRNTYSSLE
GKVSEDKSREFQSLLKGLKSKSINVLIRDIVREQFKIFQNDMQETVAQLFKTVSSLSEDL
ESTRQIIQKVNESVVSIAAQQKFVLVQENRPTLTDIVELRNHIVNVRQEMTLTCEKPIKE
LEVKQTHLEGALEQEHSRSILYYESLNKTLSKLKEVHEQLLSTEQVSDQKNAPAAESVSN
NVTEYMSTLHENIKKQSLMMLQMFEDLHIQESKINNLTVSLEMEKESLRGECEDMLSKCR
NDFKFQLKDTEENLHVLNQTLAEVLFPMDNKMDKMSEQLNDLTYDMEILQPLLEQGASLR
QTMTYEQPKEAIVIRKKIENLTSAVNSLNFIIKELTKRHNLLRNEVQGRDDALERRINEY
ALEMEDGLNKTMTIINNAIDFIQDNYALKETLSTIKDNSEIHHKCTSDMETILTFIPQFH
RLNDSIQTLVNDNQRYNFVLQVAKTLAGIPRDEKLNQSNFQKMYQMFNETTSQVRKYQQN
MSHLEEKLLLTTKISKNFETRLQDIESKVTQTLIPYYISVKKGSVVTNERDQALQLQVLN
SRFKALEAKSIHLSINFFSLNKTLHEVLTMCHNASTSVSELNATIPKWIKHSLPDIQLLQ
KGLTEFVEPIIQIKTQAALSNLTCCIDRSLPGSLANVVKSQKQVKSLPKKINALKKPTVN
LTTVLIGRTQRNTDNIIYPEEYSSCSRHPCQNGGTCINGRTSFTCACRHPFTGDNCTIKL
VEENALAPDFSKGSYRYAPMVAFFASHTYGMTIPGPILFNNLDVNYGASYTPRTGKFRIP
YLGVYVFKYTIESFSAHISGFLVVDGIDKLAFESENINSEIHCDRVLTGDALLELNYGQE
VWLRLAKGTIPAKFPPVTTFSGYLLYRT
>sp|Q7Z7G0|TARSH_HUMAN Target of Nesh-SH3 OS=Homo sapiens OX=9606 GN=ABI3BP PE=1 SV=2
MLSSLGCLLLCGSITLALGNAQKLPKGKRPNLKVHINTTSDSILLKFLRPSPNVKLEGLL
LGYGSNVSPNQYFPLPAEGKFTEAIVDAEPKYLIVVRPAPPPSQKKSCSGKTRSRKPLQL
VVGTLTPSSVFLSWGFLINPHHDWTLPSHCPNDRFYTIRYREKDKEKKWIFQICPATETI
VENLKPNTVYEFGVKDNVEGGIWSKIFNHKTVVGSKKVNGKIQSTYDQDHTVPAYVPRKL
IPITIIKQVIQNVTHKDSAKSPEKAPLGGVILVHLIIPGLNETTVKLPASLMFEISDALK
TQLAKNETLALPAESKTPEVEKISARPTTVTPETVPRSTKPTTSSALDVSETTLASSEKP
WIVPTAKISEDSKVLQPQTATYDVFSSPTTSDEPEISDSYTATSDRILDSIPPKTSRTLE

QPRATLAPSETPFVPQKLEIFTSPEMQPTTPAPQQTTSIPSTPKRRPRPKPPRTKPERTT
SAGTITPKISKSPEPTWTTPAPGKTQFISLKPKIPLSPEVTHTKPAPKQTPRAPPKPKTS
PRPRIPQTQPVPKVPQRVTAKPKTSPSPEVSYTTPAPKDVLLPHKPYPEVSQSEPAPLET
RGIPFIPMISPSPSQEELQTTLEETDQSTQEPFTTKIPRTTELAKTTQAPHRFYTTVRPR
TSDKPHIRPGVKQAPRPSGADRNVSVDSTHPTKKPGTRRPPLPPRPTHPRRKPLPPNNVT
GKPGSAGIISSGPITTPPLRSTPRPTGTPLERIETDIKQPTVPASGEELENITDFSSSPT
RETDPLGKPRFKGPHVRYIQKPDNSPCSITDSVKRFPKEEATEGNATSPPQNPPTNLTVV
TVEGCPSFVILDWEKPLNDTVTEYEVISRENGSFSGKNKSIQMTNQTFSTVENLKPNTSY
EFQVKPKNPLGEGPVSNTVAFSTESADPRVSEPVSAGRDAIWTERPFNSDSYSECKGKQY
VKRTWYKKFVGVQLCNSLRYKIYLSDSLTGKFYNIGDQRGHGEDHCQFVDSFLDGRTGQQ
LTSDQLPIKEGYFRAVRQEPVQFGEIGGHTQINYVQWYECGTTIPGKW
>sp|Q9GZM7|TINAL_HUMAN Tubulointerstitial nephritis antigen-like OS=Homo sapiens OX=9606 GN=TINAGL1 PE=1 SV=1
MWRCPLGLLLLLPLAGHLALGAQQGRGRRELAPGLHLRGIRDAGGRYCQEQDLCCRGRAD
DCALPYLGAICYCDLFCNRTVSDCCPDFWDFCLGVPPPFPPIQGCMHGGRIYPVLGTYWD
NCNRCTCQENRQWQCDQEPCLVDPDMIKAINQGNYGWQAGNHSAFWGMTLDEGIRYRLGT
IRPSSSVMNMHEIYTVLNPGEVLPTAFEASEKWPNLIHEPLDQGNCAGSWAFSTAAVASD
RVSIHSLGHMTPVLSPQNLLSCDTHQQQGCRGGRLDGAWWFLRRRGVVSDHCYPFSGRER
DEAGPAPPCMMHSRAMGRGKRQATAHCPNSYVNNNDIYQVTPVYRLGSNDKEIMKELMEN
GPVQALMEVHEDFFLYKGGIYSHTPVSLGRPERYRRHGTHSVKITGWGEETLPDGRTLKY
WTAANSWGPAWGERGHFRIVRGVNECDIESFVLGVWGRVGMEDMGHH
>sp|P02462|CO4A1_HUMAN Collagen alpha-1(IV) chain OS=Homo sapiens OX=9606 GN=COL4A1 PE=1 SV=4
MGPRLSVWLLLLPAALLLHEEHSRAAAKGGCAGSGCGKCDCHGVKGQKGERGLPGLQGVI
GFPGMQGPEGPQGPPGQKGDTGEPGLPGTKGTRGPPGASGYPGNPGLPGIPGQDGPPGPP
GIPGCNGTKGERGPLGPPGLPGFAGNPGPPGLPGMKGDPGEILGHVPGMLLKGERGFPGI
PGTPGPPGLPGLQGPVGPPGFTGPPGPPGPPGPPGEKGQMGLSFQGPKGDKGDQGVSGPP
GVPGQAQVQEKGDFATKGEKGQKGEPGFQGMPGVGEKGEPGKPGPRGKPGKDGDKGEKGS
PGFPGEPGYPGLIGRQGPQGEKGEAGPPGPPGIVIGTGPLGEKGERGYPGTPGPRGEPGP
KGFPGLPGQPGPPGLPVPGQAGAPGFPGERGEKGDRGFPGTSLPGPSGRDGLPGPPGSPG
PPGQPGYTNGIVECQPGPPGDQGPPGIPGQPGFIGEIGEKGQKGESCLICDIDGYRGPPG
PQGPPGEIGFPGQPGAKGDRGLPGRDGVAGVPGPQGTPGLIGQPGAKGEPGEFYFDLRLK
GDKGDPGFPGQPGMPGRAGSPGRDGHPGLPGPKGSPGSVGLKGERGPPGGVGFPGSRGDT
GPPGPPGYGPAGPIGDKGQAGFPGGPGSPGLPGPKGEPGKIVPLPGPPGAEGLPGSPGFP
GPQGDRGFPGTPGRPGLPGEKGAVGQPGIGFPGPPGPKGVDGLPGDMGPPGTPGRPGFNG
LPGNPGVQGQKGEPGVGLPGLKGLPGLPGIPGTPGEKGSIGVPGVPGEHGAIGPPGLQGI
RGEPGPPGLPGSVGSPGVPGIGPPGARGPPGGQGPPGLSGPPGIKGEKGFPGFPGLDMPG
PKGDKGAQGLPGITGQSGLPGLPGQQGAPGIPGFPGSKGEMGVMGTPGQPGSPGPVGAPG
LPGEKGDHGFPGSSGPRGDPGLKGDKGDVGLPGKPGSMDKVDMGSMKGQKGDQGEKGQIG
PIGEKGSRGDPGTPGVPGKDGQAGQPGQPGPKGDPGISGTPGAPGLPGPKGSVGGMGLPG
TPGEKGVPGIPGPQGSPGLPGDKGAKGEKGQAGPPGIGIPGLRGEKGDQGIAGFPGSPGE
KGEKGSIGIPGMPGSPGLKGSPGSVGYPGSPGLPGEKGDKGLPGLDGIPGVKGEAGLPGT
PGPTGPAGQKGEPGSDGIPGSAGEKGEPGLPGRGFPGFPGAKGDKGSKGEVGFPGLAGSP
GIPGSKGEQGFMGPPGPQGQPGLPGSPGHATEGPKGDRGPQGQPGLPGLPGPMGPPGLPG

IDGVKGDKGNPGWPGAPGVPGPKGDPGFQGMPGIGGSPGITGSKGDMGPPGVPGFQGPKG
LPGLQGIKGDQGDQGVPGAKGLPGPPGPPGPYDIIKGEPGLPGPEGPPGLKGLQGLPGPK
GQQGVTGLVGIPGPPGIPGFDGAPGQKGEMGPAGPTGPRGFPGPPGPDGLPGSMGPPGTP
SVDHGFLVTRHSQTIDDPQCPSGTKILYHGYSLLYVQGNERAHGQDLGTAGSCLRKFSTM
PFLFCNINNVCNFASRNDYSYWLSTPEPMPMSMAPITGENIRPFISRCAVCEAPAMVMAV
HSQTIQIPPCPSGWSSLWIGYSFVMHTSAGAEGSGQALASPGSCLEEFRSAPFIECHGRG
TCNYYANAYSFWLATIERSEMFKKPTPSTLKAGELRTHVSRCQVCMRRT
>sp|P49765|VEGFB_HUMAN Vascular endothelial growth factor B OS=Homo sapiens OX=9606 GN=VEGFB PE=1 SV=2
MSPLLRRLLLAALLQLAPAQAPVSQPDAPGHQRKVVSWIDVYTRATCQPREVVVPLTVEL
MGTVAKQLVPSCVTVQRCGGCCPDDGLECVPTGQHQVRMQILMIRYPSSQLGEMSLEEHS
QCECRPKKKDSAVKPDRAATPHHRPQPRSVPGWDSAPGAPSPADITHPTPAPGPSAHAAP
STTSALTPGPAAAAADAAASSVAKGGA
>sp|O95967|FBLN4_HUMAN EGF-containing fibulin-like extracellular matrix protein 2 OS=Homo sapiens OX=9606 GN=EFEMP2 PE=1 SV=3
MLPCASCLPGSLLLWALLLLLLGSASPQDSEEPDSYTECTDGYEWDPDSQHCRDVNECLT
IPEACKGEMKCINHYGGYLCLPRSAAVINDLHGEGPPPPVPPAQHPNPCPPGYEPDDQDS
CVDVDECAQALHDCRPSQDCHNLPGSYQCTCPDGYRKIGPECVDIDECRYRYCQHRCVNL
PGSFRCQCEPGFQLGPNNRSCVDVNECDMGAPCEQRCFNSYGTFLCRCHQGYELHRDGFS
CSDIDECSYSSYLCQYRCINEPGRFSCHCPQGYQLLATRLCQDIDECESGAHQCSEAQTC
VNFHGGYRCVDTNRCVEPYIQVSENRCLCPASNPLCREQPSSIVHRYMTITSERSVPADV
FQIQATSVYPGAYNAFQIRAGNSQGDFYIRQINNVSAMLVLARPVTGPREYVLDLEMVTM
NSLMSYRASSVLRLTVFVGAYTF
>sp|Q93097|WNT2B_HUMAN Protein Wnt-2b OS=Homo sapiens OX=9606 GN=WNT2B PE=1 SV=2
MLRPGGAEEAAQLPLRRASAPVPVPSPAAPDGSRASARLGLACLLLLLLLTLPARVDTSW
WYIGALGARVICDNIPGLVSRQRQLCQRYPDIMRSVGEGAREWIRECQHQFRHHRWNCTT
LDRDHTVFGRVMLRSSREAAFVYAISSAGVVHAITRACSQGELSVCSCDPYTRGRHHDQR
GDFDWGGCSDNIHYGVRFAKAFVDAKEKRLKDARALMNLHNNRCGRTAVRRFLKLECKCH
GVSGSCTLRTCWRALSDFRRTGDYLRRRYDGAVQVMATQDGANFTAARQGYRRATRTDLV
YFDNSPDYCVLDKAAGSLGTAGRVCSKTSKGTDGCEIMCCGRGYDTTRVTRVTQCECKFH
WCCAVRCKECRNTVDVHTCKAPKKAEWLDQT
>sp|P02458|CO2A1_HUMAN Collagen alpha-1(II) chain OS=Homo sapiens OX=9606 GN=COL2A1 PE=1 SV=3
MIRLGAPQTLVLLTLLVAAVLRCQGQDVQEAGSCVQDGQRYNDKDVWKPEPCRICVCDTG
TVLCDDIICEDVKDCLSPEIPFGECCPICPTDLATASGQPGPKGQKGEPGDIKDIVGPKG
PPGPQGPAGEQGPRGDRGDKGEKGAPGPRGRDGEPGTPGNPGPPGPPGPPGPPGLGGNFA
AQMAGGFDEKAGGAQLGVMQGPMGPMGPRGPPGPAGAPGPQGFQGNPGEPGEPGVSGPMG
PRGPPGPPGKPGDDGEAGKPGKAGERGPPGPQGARGFPGTPGLPGVKGHRGYPGLDGAKG
EAGAPGVKGESGSPGENGSPGPMGPRGLPGERGRTGPAGAAGARGNDGQPGPAGPPGPVG
PAGGPGFPGAPGAKGEAGPTGARGPEGAQGPRGEPGTPGSPGPAGASGNPGTDGIPGAKG
SAGAPGIAGAPGFPGPRGPPGPQGATGPLGPKGQTGEPGIAGFKGEQGPKGEPGPAGPQG
APGPAGEEGKRGARGEPGGVGPIGPPGERGAPGNRGFPGQDGLAGPKGAPGERGPSGLAG
PKGANGDPGRPGEPGLPGARGLTGRPGDAGPQGKVGPSGAPGEDGRPGPPGPQGARGQPG

VMGFPGPKGANGEPGKAGEKGLPGAPGLRGLPGKDGETGAAGPPGPAGPAGERGEQGAPG
PSGFQGLPGPPGPPGEGGKPGDQGVPGEAGAPGLVGPRGERGFPGERGSPGAQGLQGPRG
LPGTPGTDGPKGASGPAGPPGAQGPPGLQGMPGERGAAGIAGPKGDRGDVGEKGPEGAPG
KDGGRGLTGPIGPPGPAGANGEKGEVGPPGPAGSAGARGAPGERGETGPPGPAGFAGPPG
ADGQPGAKGEQGEAGQKGDAGAPGPQGPSGAPGPQGPTGVTGPKGARGAQGPPGATGFPG
AAGRVGPPGSNGNPGPPGPPGPSGKDGPKGARGDSGPPGRAGEPGLQGPAGPPGEKGEPG
DDGPSGAEGPPGPQGLAGQRGIVGLPGQRGERGFPGLPGPSGEPGKQGAPGASGDRGPPG
PVGPPGLTGPAGEPGREGSPGADGPPGRDGAAGVKGDRGETGAVGAPGAPGPPGSPGPAG
PTGKQGDRGEAGAQGPMGPSGPAGARGIQGPQGPRGDKGEAGEPGERGLKGHRGFTGLQG
LPGPPGPSGDQGASGPAGPSGPRGPPGPVGPSGKDGANGIPGPIGPPGPRGRSGETGPAG
PPGNPGPPGPPGPPGPGIDMSAFAGLGPREKGPDPLQYMRADQAAGGLRQHDAEVDATLK
SLNNQIESIRSPEGSRKNPARTCRDLKLCHPEWKSGDYWIDPNQGCTLDAMKVFCNMETG
ETCVYPNPANVPKKNWWSSKSKEKKHIWFGETINGGFHFSYGDDNLAPNTANVQMTFLRL
LSTEGSQNITYHCKNSIAYLDEAAGNLKKALLIQGSNDVEIRAEGNSRFTYTALKDGCTK
HTGKWGKTVIEYRSQKTSRLPIIDIAPMDIGGPEQEFGVDIGPVCFL
>sp|Q14055|CO9A2_HUMAN Collagen alpha-2(IX) chain OS=Homo sapiens OX=9606
GN=COL9A2 PE=1 SV=2
MAAATASPRSLLVLLQVVVLALAQIRGPPGERGPPGPPGPPGVPGSDGIDGDNGPPGKAG
PPGPKGEPGKAGPDGPDGKPGIDGLTGAKGEPGPMGIPGVKGQPGLPGPPGLPGPGFAGP
PGPPGPVGLPGEIGIRGPKGDPGPDGPSGPPGPPGKPGRPGTIQGLEGSADFLCPTNCPP
GMKGPPGLQGVKGHAGKRGILGDPGHQGKPGPKGDVGASGEQGIPGPPGPQGIRGYPGMA
GPKGETGPHGYKGMVGAIGATGPPGEEGPRGPPGRAGEKGDEGSPGIRGPQGITGPKGAT
GPPGINGKDGTPGTPGMKGSAGQAGQPGSPGHQGLAGVPGQPGTKGGPGDQGEPGPQGLP
GFSGPPGKEGEPGPRGEIGPQGIMGQKGDQGERGPVGQPGPQGRQGPKGEQGPPGIPGPQ
GLPGVKGDKGSPGKTGPRGKVGDPGVAGLPGEKGEKGESGEPGPKGQQGVRGEPGYPGPS
GDAGAPGVQGYPGPPGPRGLAGNRGVPGQPGRQGVEGRDATDQHIVDVALKMLQEQLAEV
AVSAKREALGAVGMMGPPGPPGPPGYPGKQGPHGHPGPRGVPGIVGAVGQIGNTGPKGKR
GEKGDPGEVGRGHPGMPGPPGIPGLPGRPGQAINGKDGDRGSPGAPGEAGRPGLPGPVGL
PGFCEPAACLGASAYASARLTEPGSIKGP
>sp|P04083|ANXA1_HUMAN Annexin A1 OS=Homo sapiens OX=9606 GN=ANXA1 PE=1 SV=2
MAMVSEFLKQAWFIENEEQEYVQTVKSSKGGPGSAVSPYPTFNPSSDVAALHKAIMVKGV
DEATIIDILTKRNNAQRQQIKAAYLQETGKPLDETLKKALTGHLEEVVLALLKTPAQFDA
DELRAAMKGLGTDEDTLIEILASRTNKEIRDINRVYREELKRDLAKDITSDTSGDFRNAL
LSLAKGDRSEDFGVNEDLADSDARALYEAGERRKGTDVNVFNTILTTRSYPQLRRVFQKY
TKYSKHDMNKVLDLELKGDIEKCLTAIVKCATSKPAFFAEKLHQAMKGVGTRHKALIRIM
VSRSEIDMNDIKAFYQKMYGISLCQAILDETKGDYEKILVALCGGN
>sp|P21980|TGM2_HUMAN Protein-glutamine gamma-glutamyltransferase 2 OS=Homo
sapiens OX=9606 GN=TGM2 PE=1 SV=2
MAEELVLERCDLELETNGRDHHTADLCREKLVVRRGQPFWLTLHFEGRNYEASVDSLTFS
VVTGPAPSQEAGTKARFPLRDAVEEGDWTATVVDQQDCTLSLQLTTPANAPIGLYRLSLE
ASTGYQGSSFVLGHFILLFNAWCPADAVYLDSEEERQEYVLTQQGFIYQGSAKFIKNIPW
NFGQFEDGILDICLILLDVNPKFLKNAGRDCSRRSSPVYVGRVVSGMVNCNDDQGVLLGR
WDNNYGDGVSPMSWIGSVDILRRWKNHGCQRVKYGQCWVFAAVACTVLRCLGIPTRVVTN
YNSAHDQNSNLLIEYFRNEFGEIQGDKSEMIWNFHCWVESWMTRPDLQPGYEGWQALDPT

PQEKSEGTYCCGPVPVRAIKEGDLSTKYDAPFVFAEVNADVVDWIQQDDGSVHKSINRSL
IVGLKISTKSVGRDEREDITHTYKYPEGSSEEREAFTRANHLNKLAEKEETGMAMRIRVG
QSMNMGSDFDVFAHITNNTAEEYVCRLLLCARTVSYNGILGPECGTKYLLNLNLEPFSEK
SVPLCILYEKYRDCLTESNLIKVRALLVEPVINSYLLAERDLYLENPEIKIRILGEPKQK
RKLVAEVSLQNPLPVALEGCTFTVEGAGLTEEQKTVEIPDPVEAGEEVKVRMDLLPLHMG
LHKLVVNFESDKLKAVKGFRNVIIGPA
>sp|P01023|A2MG_HUMAN Alpha-2-macroglobulin OS=Homo sapiens OX=9606 GN=A2M PE=1 SV=3
MGKNKLLHPSLVLLLLVLLPTDASVSGKPQYMVLVPSLLHTETTEKGCVLLSYLNETVTV
SASLESVRGNRSLFTDLEAENDVLHCVAFAVPKSSSNEEVMFLTVQVKGPTQEFKKRTTV
MVKNEDSLVFVQTDKSIYKPGQTVKFRVVSMDENFHPLNELIPLVYIQDPKGNRIAQWQS
FQLEGGLKQFSFPLSSEPFQGSYKVVVQKKSGGRTEHPFTVEEFVLPKFEVQVTVPKIIT
ILEEEMNVSVCGLYTYGKPVPGHVTVSICRKYSDASDCHGEDSQAFCEKFSGQLNSHGCF
YQQVKTKVFQLKRKEYEMKLHTEAQIQEEGTVVELTGRQSSEITRTITKLSFVKVDSHFR
QGIPFFGQVRLVDGKGVPIPNKVIFIRGNEANYYSNATTDEHGLVQFSINTTNVMGTSLT
VRVNYKDRSPCYGYQWVSEEHEEAHHTAYLVFSPSKSFVHLEPMSHELPCGHTQTVQAHY
ILNGGTLLGLKKLSFYYLIMAKGGIVRTGTHGLLVKQEDMKGHFSISIPVKSDIAPVARL
LIYAVLPTGDVIGDSAKYDVENCLANKVDLSFSPSQSLPASHAHLRVTAAPQSVCALRAV
DQSVLLMKPDAELSASSVYNLLPEKDLTGFPGPLNDQDNEDCINRHNVYINGITYTPVSS
TNEKDMYSFLEDMGLKAFTNSKIRKPKMCPQLQQYEMHGPEGLRVGFYESDVMGRGHARL
VHVEEPHTETVRKYFPETWIWDLVVVNSAGVAEVGVTVPDTITEWKAGAFCLSEDAGLGI
SSTASLRAFQPFFVELTMPYSVIRGEAFTLKATVLNYLPKCIRVSVQLEASPAFLAVPVE
KEQAPHCICANGRQTVSWAVTPKSLGNVNFTVSAEALESQELCGTEVPSVPEHGRKDTVI
KPLLVEPEGLEKETTFNSLLCPSGGEVSEELSLKLPPNVVEESARASVSVLGDILGSAMQ
NTQNLLQMPYGCGEQNMVLFAPNIYVLDYLNETQQLTPEIKSKAIGYLNTGYQRQLNYKH
YDGSYSTFGERYGRNQGNTWLTAFVLKTFAQARAYIFIDEAHITQALIWLSQRQKDNGCF
RSSGSLLNNAIKGGVEDEVTLSAYITIALLEIPLTVTHPVVRNALFCLESAWKTAQEGDH
GSHVYTKALLAYAFALAGNQDKRKEVLKSLNEEAVKKDNSVHWERPQKPKAPVGHFYEPQ
APSAEVEMTSYVLLAYLTAQPAPTSEDLTSATNIVKWITKQQNAQGGFSSTQDTVVALHA
LSKYGAATFTRTGKAAQVTIQSSGTFSSKFQVDNNNRLLLQQVSLPELPGEYSMKVTGEG
CVYLQTSLKYNILPEKEEFPFALGVQTLPQTCDEPKAHTSFQISLSVSYTGSRSASNMAI
VDVKMVSGFIPLKPTVKMLERSNHVSRTEVSSNHVLIYLDKVSNQTLSLFFTVLQDVPVR
DLKPAIVKVYDYYETDEFAIAEYNAPCSKDLGNA
>sp|P29279|CCN2_HUMAN CCN family member 2 OS=Homo sapiens OX=9606 GN=CCN2 PE=1 SV=2
MTAASMGPVRVAFVVLLALCSRPAVGQNCSGPCRCPDEPAPRCPAGVSLVLDGCGCCRVC
AKQLGELCTERDPCDPHKGLFCHFGSPANRKIGVCTAKDGAPCIFGGTVYRSGESFQSSC
KYQCTCLDGAVGCMPLCSMDVRLPSPDCPFPRRVKLPGKCCEEWVCDEPKDQTVVGPALA
AYRLEDTFGPDPTMIRANCLVQTTEWSACSKTCGMGISTRVTNDNASCRLEKQSRLCMVR
PCEADLEENIKKGKKCIRTPKISKPIKFELSGCTSMKTYRAKFCGVCTDGRCCTPHRTTT
LPVEFKCPDGEVMKKNMMFIKTCACHYNCPGDNDIFESLYYRKMYGDMA
>sp|Q12805|FBLN3_HUMAN EGF-containing fibulin-like extracellular matrix protein 1 OS=Homo sapiens OX=9606 GN=EFEMP1 PE=1 SV=2
MLKALFLTMLTLALVKSQDTEETITYTQCTDGYEWDPVRQQCKDIDECDIVPDACKGGMK

CVNHYGGYLCLPKTAQIIVNNEQPQQETQPAEGTSGATTGVVAASSMATSGVLPGGGFVA
SAAAVAGPEMQTGRNNFVIRRNPADPQRIPSNPSHRIQCAAGYEQSEHNVCQDIDECTAG
THNCRADQVCINLRGSFACQCPPGYQKRGEQCVDIDECTIPPYCHQRCVNTPGSFYCQCS
PGFQLAANNYTCVDINECDASNQCAQQCYNILGSFICQCNQGYELSSDRLNCEDIDECRT
SSYLCQYQCVNEPGKFSCMCPQGYQVVRSRTCQDINECETTNECREDEMCWNYHGGFRCY
PRNPCQDPYILTPENRCVCPVSNAMCRELPQSIVYKYMSIRSDRSVPSDIFQIQATTIYA
NTINTFRIKSGNENGEFYLRQTSPVSAMLVLVKSLSGPREHIVDLEMLTVSSIGTFRTSS
VLRLTIIVGPFSF
>sp|P04196|HRG_HUMAN Histidine-rich glycoprotein OS=Homo sapiens OX=9606 GN=HRG PE=1 SV=1
MKALIAALLLITLQYSCAVSPTDCSAVEPEAEKALDLINKRRRDGYLFQLLRIADAHLDR
VENTTVYYLVLDVQESDCSVLSRKYWNDCEPPDSRRPSEIVIGQCKVIATRHSHESQDLR
VIDFNCTTSSVSSALANTKDSPVLIDFFEDTERYRKQANKALEKYKEENDDFASFRVDRI
ERVARVRGGEGTGYFVDFSVRNCPRHHFPRHPNVFGFCRADLFYDVEALDLESPKNLVIN
CEVFDPQEHENINGVPPHLGHPFHWGGHERSSTTKPPFKPHGSRDHHHPHKPHEHGPPPP
PDERDHSHGPPLPQGPPPLLPMSCSSCQHATFGTNGAQRHSHNNNSSDLHPHKHHSHEQH
PHGHHPHAHHPHEHDTHRQHPHGHHPHGHHPHGHHPHGHHPHGHHPHCHDFQDYGPCDPP
PHNQGHCCHGHGPPPGHLRRRGPGKGPRPFHCRQIGSVYRLPPLRKGEVLPLPEANFPSF
PLPHHKHPLKPDNQPFPQSVSESCPGKFKSGFPQVSMFFTHTFPK
>sp|P08758|ANXA5_HUMAN Annexin A5 OS=Homo sapiens OX=9606 GN=ANXA5 PE=1 SV=2
MAQVLRGTVTDFPGFDERADAETLRKAMKGLGTDEESILTLLTSRSNAQRQEISAAFKTL
FGRDLLDDLKSELTGKFEKLIVALMKPSRLYDAYELKHALKGAGTNEKVLTEIIASRTPE
ELRAIKQVYEEEYGSSLEDDVVGDTSGYYQRMLVVLLQANRDPDAGIDEAQVEQDAQALF
QAGELKWGTDEEKFITIFGTRSVSHLRKVFDKYMTISGFQIEETIDRETSGNLEQLLLAV
VKSIRSIPAYLAETLYYAMKGAGTDDHTLIRVMVSRSEIDLFNIRKEFRKNFATSLYSMI
KGDTSGDYKKALLLLCGEDD
>sp|Q9HCB6|SPON1_HUMAN Spondin-1 OS=Homo sapiens OX=9606 GN=SPON1 PE=1 SV=2
MRLSPAPLKLSRTPALLALALPLAAALAFSDETLDKVPKSEGYCSRILRAQGTRREGYTE
FSLRVEGDPDFYKPGTSYRVTLSAAPPSYFRGFTLIALRENREGDKEEDHAGTFQIIDEE
ETQFMSNCPVAVTESTPRRRTRIQVFWIAPPAGTGCVILKASIVQKRIIYFQDEGSLTKK
LCEQDSTFDGVTDKPILDCCACGTAKYRLTFYGNWSEKTHPKDYPRRANHWSAIIGGSHS
KNYVLWEYGGYASEGVKQVAELGSPVKMEEEIRQQSDEVLTVIKAKAQWPAWQPLNVRAA
PSAEFSVDRTRHLMSFLTMMGPSPDWNVGLSAEDLCTKECGWVQKVVQDLIPWDAGTDSG
VTYESPNKPTIPQEKIRPLTSLDHPQSPFYDPEGGSITQVARVVIERIARKGEQCNIVPD
NVDDIVADLAPEEKDEDDTPETCIYSNWSPWSACSSSTCDKGKRMRQRMLKAQLDLSVPC
PDTQDFQPCMGPGCSDEDGSTCTMSEWITWSPCSISCGMGMRSRERYVKQFPEDGSVCTL
PTEETEKCTVNEECSPSSCLMTEWGEWDECSATCGMGMKKRHRMIKMNPADGSMCKAETS
QAEKCMMPECHTIPCLLSPWSEWSDCSVTCGKGMRTRQRMLKSLAELGDCNEDLEQVEKC
MLPECPIDCELTEWSQWSECNKSCGKGHVIRTRMIQMEPQFGGAPCPETVQRKKCRIRKC
LRNPSIQKLRWREARESRRSEQLKEESEGEQFPGCRMRPWTAWSECTKLCGGGIQERYMT
VKKRFKSSQFTSCKDKKEIRACNVHPC
>sp|Q86YZ3|HORN_HUMAN Hornerin OS=Homo sapiens OX=9606 GN=HRNR PE=1 SV=2
MPKLLQGVITVIDVFYQYATQHGEYDTLNKAELKELLENEFHQILKNPNDPDTVDIILQS
LDRDHNKKVDFTEYLLMIFKLVQARNKIIGKDYCQVSGSKLRDDTHQHQEEQEETEKEEN

KRQESSFSHSSWSAGENDSYSRNVRGSLKPGTESISRRLSFQRDFSGQHNSYSGQSSSYG
EQNSDSHQSSGRGQCGSGSGQSPNYGQHGSGSGQSSSNDTHGSGSGQSSGFSQHKSSSGQ
SSGYSQHGSGSGHSSGYGQHGSRSGQSSRGERHRSSSGSSSSYGQHGSGSRQSLGHGRQG
SGSRQSPSHVRHGSGSGHSSSHGQHGSGSSYSYSRGHYESGSGQTSGFGQHESGSGQSSG
YSKHGSGSGHSSSQGQHGSTSGQASSSGQHGSSSRQSSSYGQHESASRHSSGRGQHSSGS
GQSPGHGQRGSGSGQSPSSGQHGTGFGRSSSSGPYVSGSGYSSGFGHHESSSEHSSGYTQ
HGSGSGHSSGHGQHGSRSGQSSRGERQGSSAGSSSSYGQHGSGSRQSLGHSRHGSGSGQS
PSPSRGRHESGSRQSSSYGPHGYGSGRSSSRGPYESGSGHSSGLGHQESRSGQSSGYGQH
GSSSGHSSTHGQHGSTSGQSSSCGQHGATSGQSSSHGQHGSGSSQSSRYGQQGSGSGQSP
SRGRHGSDFGHSSSYGQHGSGSGWSSSNGPHGSVSGQSSGFGHKSGSGQSSGYSQHGSGS
SHSSGYRKHGSRSGQSSRSEQHGSSSGLSSSYGQHGSGSHQSSGHGRQGSGSGHSPSRVR
HGSSSGHSSSHGQHGSGTSCSSSCGHYESGSGQASGFGQHESGSGQGYSQHGSASGHFSS
QGRHGSTSGQSSSSGQHDSSSGQSSSYGQHESASHHASGRGRHGSGSGQSPGHGQRGSGS
GQSPSYGRHGSGSGRSSSSGRHGSGSGQSSGFGHKSSSGQSSGYTQHGSGSGHSSSYEQH
GSRSGQSSRSEQHGSSSGSSSSYGQHGSGSRQSLGHGQHGSGSGQSPSPSRGRHGSGSGQ
SSSYGPYRSGSGWSSSRGPYESGSGHSSGLGHRESRSGQSSGYGQHGSSSGHSSTHGQHG
STSGQSSSCGQHGASSGQSSSHGQHGSGSSQSSGYGRQGSGSGQSPGHGQRGSGSRQSPS
YGRHGSGSGRSSSSGQHGSGLGESSGFGHHESSSGQSSSYSQHGSGSGHSSGYGQHGSRS
GQSSRGERHGSSSGSSSHYGQHGSGSRQSSGHGRQGSGSGHSPSRGRHGSGLGHSSSHGQ
HGSGSGRSSSRGPYESRSGHSSVFGQHESGSGHSSAYSQHGSGSGHFCSQGQHGSTSGQS
STFDQEGSSTGQSSSYGHRGSGSSQSSGYGRHGAGSGQSPSRGRHGSGSGHSSSYGQHGS
GSGWSSSSGRHGSGSGQSSGFGHHESSSWQSSGCTQHGSGSGHSSSYEQHGSRSGQSSRG
ERHGSSSGSSSSYGQHGSGSRQSLGHGQHGSGSGQSPSPSRGRHGSGSGQSSSYSPYGSG
SGWSSSRGPYESGSSHSSGLGHRESRSGQSSGYGQHGSSSGHSSTHGQHGSTSGQSSSCG
QHGASSGQSSSHGQHGSGSSQSSGYGRQGSGSGQSPGHGQRGSGSRQSPSYGRHGSGSGR
SSSSGQHGSGLGESSGFGHHESSSGQSSSYSQHGSGSGHSSGYGQHGSRSGQSSRGERHG
SSSRSSSRYGQHGSGSRQSSGHGRQGSGSGQSPSRGRHGSGLGHSSSHGQHGSGSGRSSS
RGPYESRSGHSSVFGQHESGSGHSSAYSQHGSGSGHFCSQGQHGSTSGQSSTFDQEGSST
GQSSSHGQHGSGSSQSSSYGQQGSGSGQSPSRGRHGSGSGHSSSYGQHGSGSGWSSSSGR
HGSGSGQSSGFGHHESSSWQSSGYTQHGSGSGHSSSYEQHGSRSGQSSRGEQHGSSSGSS
SSYGQHGSGSRQSLGHGQHGSGSGQSPSPSRGRHGSGSGQSSSYGPYGSGSGWSSSRGPY
ESGSGHSSGLGHRESRSGQSSGYGQHGSSSGHSSTHGQHGSASGQSSSCGQHGASSGQSS
SHGQHGSGSSQSSGYGRQGSGSGQSPGHGQRGSGSRQSPSYGRHGSGSGRSSSSGQHGPG
LGESSGFGHHESSSGQSSSYSQHGSGSGHSSGYGQHGSRSGQSSRGERHGSSSGSSSRYG
QHGSGSRQSSGHGRQGSGSGHSPSRGRHGSGSGHSSSHGQHGSGSGRSSSRGPYESRSGH
SSVFGQHESGSGHSSAYSQHGSGSGHFCSQGQHGSTSGQSSTFDQEGSSTGQSSSHGQHG
SGSSQSSSYGQQGSGSGQSPSRGRHGSGSGHSSSYGQHGSGSGWSSSSGRHGSGSGQSSG
FGHHESSSWQSSGYTQHGSGSGHSSSYEQHGSRSGQSSRGERHGSSSGSSSSYGQHGSGS
RQSLGHGQHGSGSGQSPSPSRGRHGSGSGQSSSYSPYGSGSGWSSSRGPYESGSGHSSGL
GHRESRSGQSSGYGQHGSSSGHSSTHGQHGSTSGQSSSCGQHGASSGQSSSHGQHGSGSS
QSSGYGRQGSGSGQSPGHGQRGSGSRQSPSYGRHGSGSGRSSSSGQHGSGLGESSGFGHH
ESSSGQSSSYSQHGSGSGHSSGYGQHGSRSGQSSRGERHGSSSGSSSHYGQHGSGSRQSS
GHGRQGSGSGQSPSRGRHGSGLGHSSSHGQHGSGSGRSSSRGPYESRLGHSSVFGQHESG
SGHSSAYSQHGSGSGHFCSQGQHGSTSGQSSTFDQEGSSTGQSSSYGHRGSGSSQSSGYG

RHGAGSGQSLSHGRHGSGSGQSSSYGQHGSGSGQSSGYSQHGSGSGQDGYSYCKGGSNHD
GGSSGSYFLSFPSSTSPYEYVQEQRCYFYQ
>sp|Q92765|SFRP3_HUMAN Secreted frizzled-related protein 3 OS=Homo sapiens OX=9606 GN=FRZB PE=1 SV=2
MVCGSPGGMLLLRAGLLALAALCLLRVPGARAAACEPVRIPLCKSLPWNMTKMPNHLHHS
TQANAILAIEQFEGLLGTHCSPDLLFFLCAMYAPICTIDFQHEPIKPCKSVCERARQGCE
PILIKYRHSWPENLACEELPVYDRGVCISPEAIVTADGADFPMDSSNGNCRGASSERCKC
KPIRATQKTYFRNNYNYVIRAKVKEIKTKCHDVTAVVEVKEILKSSLVNIPRDTVNLYTS
SGCLCPPLNVNEEYIIMGYEDEERSRLLLVEGSIAEKWKDRLGKKVKRWDMKLRHLGLSK
SDSSNSDSTQSQKSGRNSNPRQARN
>sp|P26447|S10A4_HUMAN Protein S100-A4 OS=Homo sapiens OX=9606 GN=S100A4 PE=1 SV=1
MACPLEKALDVMVSTFHKYSGKEGDKFKLNKSELKELLTRELPSFLGKRTDEAAFQKLMS
NLDSNRDNEVDFQEYCVFLSCIAMMCNEFFEGFPDKQPRKK
>sp|Q14031|CO4A6_HUMAN Collagen alpha-6(IV) chain OS=Homo sapiens OX=9606 GN=COL4A6 PE=1 SV=3
MLINKLWLLLVTLCLTEELAAAGEKSYGKPCGGQDCSGSCQCFPEKGARGRPGPIGIQGP
TGPQGFTGSTGLSGLKGERGFPGLLGPYGPKGDKGPMGVPGFLGINGIPGHPGQPGPRGP
PGLDGCNGTQGAVGFPGPDGYPGLLGPPGLPGQKGSKGDPVLAPGSFKGMKGDPGLPGLD
GITGPQGAPGFPGAVGPAGPPGLQGPPGPPGPLGPDGNMGLGFQGEKGVKGDVGLPGPAG
PPPSTGELEFMGFPKGKKGSKGEPGPKGFPGISGPPGFPGLGTTGEKGEKGEKGIPGLPG
PRGPMGSEGVQGPPGQQGKKGTLGFPGLNGFQGIEGQKGDIGLPGPDVFIDIDGAVISGN
PGDPGVPGLPGLKGDEGIQGLRGPSGVPGLPALSGVPGALGPQGFPGLKGDQGNPGRTTI
GAAGLPGRDGLPGPPGPPGPPSPEFETETLHNKESGFPGLRGEQGPKGNLGLKGIKGDSG
FCACDGGVPNTGPPGEPGPPGPWGLIGLPGLKGARGDRGSGGAQGPAGAPGLVGPLGPSG
PKGKKGEPILSTIQGMPGDRGDSGSQGFRGVIGEPGKDGVPGLPGLPGLPGDGGQGFPGE
KGLPGLPGEKGHPGPPGLPGNGLPGLPGPRGLPGDKGKDGLPGQQGLPGSKGITLPCIIP
GSYGPSGFPGTPGFPGPKGSRGLPGTPGQPGSSGSKGEPGSPGLVHLPELPGFPGPRGEK
GLPGFPGLPGKDGLPGMIGSPGLPGSKGATGDIFGAENGAPGEQGLQGLTGHKGFLGDSG
LPGLKGVHGKPGLLGPKGERGSPGTPGQVGQPGTPGSSGPYGIKGKSGLPGAPGFPGISG
HPGKKGTRGKKGPPGSIVKKGLPGLKGLPGNPGLVGLKGSPGSPGVAGLPALSGPKGEKG
SVGFVGFPGIPGLPGIPGTRGLKGIPGSTGKMGPSGRAGTPGEKGDRGNPGPVGIPSPRR
PMSNLWLKGDKGSQGSAGSNGFPGPRGDKGEAGRPGPPGLPGAPGLPGIIKGVSGKPGPP
GFMGIRGLPGLKGSSGITGFPGMPGESGSQGIRGSPGLPGASGLPGLKGDNGQTVEISGS
PGPKGQPGESGFKGTKGRDGLIGNIGFPGNKGEDGKVGVSGDVGLPGAPGFPGVAGMRGE
PGLPGSSGHQGAIGPLGSPGLIGPKGFPGFPGLHGLNGLPGTKGTHGTPGPSITGVPGPA
GLPGPKGEKGYPGIGIGAPGKPGLRGQKGDRGFPGLQGPAGLPGAPGISLPSLIAGQPGD
PGRPGLDGERGRPGPAGPPGPPGPSSNQGDTGDPGFPGIPGPKGPKGDQGIPGFSGLPGE
LGLKGMRGEPGFMGTPGKVGPPGDPGFPGMKGKAGPRGSSGLQGDPGQTPTAEAVQVPPG
PLGLPGIDGIPGLTGDPGAQGPVGLQGSKGLPGIPGKDGPSGLPGPPGALGDPGLPGLQG
PPGFEGAPGQQGPFGMPGMPGQSMRVGYTLVKHSQSEQVPPCPIGMSQLWVGYSLLFVEG
QEKAHNQDLGFAGSCLPRFSTMPFIYCNINEVCHYARRNDKSYWLSTTAPIPMMPVSQTQ
IPQYISRCSVCEAPSQAIAVHSQDITIPQCPLGWRSLWIGYSFLMHTAAGAEGGGQSLVS
PGSCLEDFRATPFIECSGARGTCHYFANKYSFWLTTVEERQQFGELPVSETLKAGQLHTR

VSRCQVCMKSL
>sp|Q6ZMM2|ATL5_HUMAN ADAMTS-like protein 5 OS=Homo sapiens OX=9606
GN=ADAMTSL5 PE=1 SV=3
MGKLRPGRVEWLASGHTERPHLFQNLLLFLWALLNCGLGVSAQGPGEWTPWVSWTRCSSS
CGRGVSVRSRRCLRLPGEEPCWGDSHEYRLCQLPDCPPGAVPFRDLQCALYNGRPVLGTQ
KTYQWVPFHGAPNQCDLNCLAEGHAFYHSFGRVLDGTACSPGAQGVCVAGRCLSAGCDGL
LGSGALEDRCGRCGGANDSCLFVQRVFRDAGAFAGYWNVTLIPEGARHIRVEHRSRNHLA
LMGGDGRYVLNGHWVVSPPGTYEAAGTHVVYTRDTGPQETLQAAGPTSHDLLLQVLLQEP
NPGIEFEFWLPRERYSPFQARVQALGWPLRQPQPRGVEPQPPAAPAVTPAQTPTLAPDPC
PPCPDTRGRAHRLLHYCGSDFVFQARVLGHHHQAQETRYEVRIQLVYKNRSPLRAREYVW
APGHCPCPMLAPHRDYLMAVQRLVSPDGTQDQLLLPHAGYARPWSPAEDSRIRLTARRCP
G
>sp|Q8TAD2|IL17D_HUMAN Interleukin-17D OS=Homo sapiens OX=9606 GN=IL17D PE=2 SV=1
MLVAGFLLALPPSWAAGAPRAGRRPARPRGCADRPEELLEQLYGRLAAGVLSAFHHTLQL
GPREQARNASCPAGGRPADRRFRPPTNLRSVSPWAYRISYDPARYPRYLPEAYCLCRGCL
TGLFGEEDVRFRSAPVYMPTVVLRRTPACAGGRSVYTEAYVTIPVGCTCVPEPEKDADSI
NSSIDKQGAKLLLGPNDAPAGP
>sp|P00488|F13A_HUMAN Coagulation factor XIII A chain OS=Homo sapiens OX=9606
GN=F13A1 PE=1 SV=5
MSETSRTAFGGRRAVPPNNSNAAEDDLPTVELQGVVPRGVNLQEFLNVTSVHLFKERWDT
NKVDHHTDKYENNKLIVRRGQSFYVQIDFSRPYDPRRDLFRVEYVIGRYPQENKGTYIPV
PIVSELQSGKWGAKIVMREDRSVRLSIQSSPKCIVGKFRMYVAVWTPYGVLRTSRNPETD
TYILFNPWCEDDAVYLDNEKEREEYVLNDIGVIFYGEVNDIKTRSWSYGQFEDGILDTCL
YVMDRAQMDLSGRGNPIKVSRVGSAMVNAKDDEGVLVGSWDNIYAYGVPPSAWTGSVDIL
LEYRSSENPVRYGQCWVFAGVFNTFLRCLGIPARIVTNYFSAHDNDANLQMDIFLEEDGN
VNSKLTKDSVWNYHCWNEAWMTRPDLPVGFGGWQAVDSTPQENSDGMYRCGPASVQAIKH
GHVCFQFDAPFVFAEVNSDLIYITAKKDGTHVVENVDATHIGKLIVTKQIGGDGMMDITD
TYKFQEGQEEERLALETALMYGAKKPLNTEGVMKSRSNVDMDFEVENAVLGKDFKLSITF
RNNSHNRYTITAYLSANITFYTGVPKAEFKKETFDVTLEPLSFKKEAVLIQAGEYMGQLL
EQASLHFFVTARINETRDVLAKQKSTVLTIPEIIIKVRGTQVVGSDMTVTVEFTNPLKET
LRNVWVHLDGPGVTRPMKKMFREIRPNSTVQWEEVCRPWVSGHRKLIASMSSDSLRHVYG
ELDVQIQRRPSM
>sp|A5D8T8|CL18A_HUMAN C-type lectin domain family 18 member A OS=Homo sapiens
OX=9606 GN=CLEC18A PE=1 SV=3
MLHPETSPGRGHLLAVLLALLGTAWAEVWPPQLQEQAPMAGALNRKESFLLLSLHNRLRS
WVQPPAADMRRLDWSDSLAQLAQARAALCGTPTPSLASGLWRTLQVGWNMQLLPAGLVSF
VEVVSLWFAEGQRYSHAAGECARNATCTHYTQLVWATSSQLGCGRHLCSAGQAAIEAFVC
AYSPRGNWEVNGKTIVPYKKGAWCSLCTASVSGCFKAWDHAGGLCEVPRNPCRMSCQNHG
RLNISTCHCHCPPGYTGRYCQVRCSLQCVHGRFREEECSCVCDIGYGGAQCATKVHFPFH
TCDLRIDGDCFMVSSEADTYYRARMKCQRKGGVLAQIKSQKVQDILAFYLGRLETTNEVI
DSDFETRNFWIGLTYKTAKDSFRWATGEHQAFTSFAFGQPDNHGFGNCVELQASAAFNWN
DQRCKTRNRYICQFAQEHISRWGPGS
>sp|P51888|PRELP_HUMAN Prolargin OS=Homo sapiens OX=9606 GN=PRELP PE=1 SV=1
MRSPLCWLLPLLILASVAQGQPTRRPRPGTGPGRRPRPRPRPTPSFPQPDEPAEPTDLPP

PLPPGPPSIFPDCPRECYCPPDFPSALYCDSRNLRKVPVIPPRIHYLYLQNNFITELPVE
SFQNATGLRWINLDNNRIRKIDQRVLEKLPGLVFLYMEKNQLEEVPSALPRNLEQLRLSQ
NHISRIPPGVFSKLENLLLLDLQHNRLSDGVFKPDTFHGLKNLMQLNLAHNILRKMPPRV
PTAIHQLYLDSNKIETIPNGYFKSFPNLAFIRLNYNKLTDRGLPKNSFNISNLLVLHLSH
NRISSVPAINNRLEHLYLNNNSIEKINGTQICPNDLVAFHDFSSDLENVPHLRYLRLDGN
YLKPPIPLDLMMCFRLLQSVVI
>sp|Q14766|LTBP1_HUMAN Latent-transforming growth factor beta-binding protein 1 OS=Homo sapiens OX=9606 GN=LTBP1 PE=1 SV=4
MAGAWLRWGLLLWAGLLASSAHGRLRRITYVVHPGPGLAAGALPLSGPPRSRTFNVALNA
RYSRSSAAAGAPSRASPGVPSERTRRTSKPGGAALQGLRPPPPPPPEPARPAVPGGQLHP
NPGGHPAAAPFTKQGRQVVRSKVPQETQSGGGSRLQVHQKQQLQGVNVCGGRCCHGWSKA
PGSQRCTKPSCVPPCQNGGMCLRPQLCVCKPGTKGKACETIAAQDTSSPVFGGQSPGAAS
SWGPPEQAAKHTSSKKADTLPRVSPVAQMTLTLKPKPSVGLPQQIHSQVTPLSSQSVVIH
HGQTQEYVLKPKYFPAQKGISGEQSTEGSFPLRYVQDQVAAPFQLSNHTGRIKVVFTPSI
CKVTCTKGSCQNSCEKGNTTTLISENGHAADTLTATNFRVVICHLPCMNGGQCSSRDKCQ
CPPNFTGKLCQIPVHGASVPKLYQHSQQPGKALGTHVIHSTHTLPLTVTSQQGVKVKFPP
NIVNIHVKHPPEASVQIHQVSRIDGPTGQKTKEAQPGQSQVSYQGLPVQKTQTIHSTYSH
QQVIPHVYPVAAKTQLGRCFQETIGSQCGKALPGLSKQEDCCGTVGTSWGFNKCQKCPKK
PSYHGYNQMMECLPGYKRVNNTFCQDINECQLQGVCPNGECLNTMGSYRCTCKIGFGPDP
TFSSCVPDPPVISEEKGPCYRLVSSGRQCMHPLSVHLTKQLCCCSVGKAWGPHCEKCPLP
GTAAFKEICPGGMGYTVSGVHRRRPIHHHVGKGPVFVKPKNTQPVAKSTHPPPLPAKEEP
VEALTFSREHGPGVAEPEVATAPPEKEIPSLDQEKTKLEPGQPQLSPGISTIHLHPQFPV
VIEKTSPPVPVEVAPEASTSSASQVIAPTQVTEINECTVNPDICGAGHCINLPVRYTCIC
YEGYRFSEQQRKCVDIDECTQVQHLCSQGRCENTEGSFLCICPAGFMASEEGTNCIDVDE
CLRPDVCGEGHCVNTVGAFRCEYCDSGYRMTQRGRCEDIDECLNPSTCPDEQCVNSPGSY
QCVPCTEGFRGWNGQCLDVDECLEPNVCANGDCSNLEGSYMCSCHKGYTRTPDHKHCRDI
DECQQGNLCVNGQCKNTEGSFRCTCGQGYQLSAAKDQCEDIDECQHRHLCAHGQCRNTEG
SFQCVCDQGYRASGLGDHCEDINECLEDKSVCQRGDCINTAGSYDCTCPDGFQLDDNKTC
QDINECEHPGLCGPQGECLNTEGSFHCVCQQGFSISADGRTCEDIDECVNNTVCDSHGFC
DNTAGSFRCLCYQGFQAPQDGQGCVDVNECELLSGVCGEAFCENVEGSFLCVCADENQEY
SPMTGQCRSRTSTDLDVDVDQPKEEKKECYYNLNDASLCDNVLAPNVTKQECCCTSGVGW
GDNCEIFPCPVLGTAEFTEMCPKGKGFVPAGESSSEAGGENYKDADECLLFGQEICKNGF
CLNTRPGYECYCKQGTYYDPVKLQCFDMDECQDPSSCIDGQCVNTEGSYNCFCTHPMVLD
ASEKRCIRPAESNEQIEETDVYQDLCWEHLSDEYVCSRPLVGKQTTYTECCCLYGEAWGM
QCALCPLKDSDDYAQLCNIPVTGRRQPYGRDALVDFSEQYTPEADPYFIQDRFLNSFEEL
QAEECGILNGCENGRCVRVQEGYTCDCFDGYHLDTAKMTCVDVNECDELNNRMSLCKNAK
CINTDGSYKCLCLPGYVPSDKPNYCTPLNTALNLEKDSDLE
>sp|P13611|CSPG2_HUMAN Versican core protein OS=Homo sapiens OX=9606 GN=VCAN PE=1 SV=3
MFINIKSILWMCSTLIVTHALHKVKVGKSPPVRGSLSGKVSLPCHFSTMPTLPPSYNTSE
FLRIKWSKIEVDKNGKDLKETTVLVAQNGNIKIGQDYKGRVSVPTHPEAVGDASLTVVKL
LASDAGLYRCDVMYGIEDTQDTVSLTVDGVVFHYRAATSRYTLNFEAAQKACLDVGAVIA
TPEQLFAAYEDGFEQCDAGWLADQTVRYPIRAPRVGCYGDKMGKAGVRTYGFRSPQETYD
VYCYVDHLDGDVFHLTVPSKFTFEEAAKECENQDARLATVGELQAAWRNGFDQCDYGWLS

DASVRHPVTVARAQCGGGLLGVRTLYRFENQTGFPPPDSRFDAYCFKPKEATTIDLSILA
ETASPSLSKEPQMVSDRTTPIIPLVDELPVIPTEFPPVGNIVSFEQKATVQPQAITDSLA
TKLPTPTGSTKKPWDMDDYSPSASGPLGKLDISEIKEEVLQSTTGVSHYATDSWDGVVED
KQTQESVTQIEQIEVGPLVTSMEILKHIPSKEFPVTETPLVTARMILESKTEKKMVSTVS
ELVTTGHYGFTLGEEDDEDRTLTVGSDESTLIFDQIPEVITVSKTSEDTIHTHLEDLESV
SASTTVSPLIMPDNNGSSMDDWEERQTSGRITEEFLGKYLSTTPFPSQHRTEIELFPYSG
DKILVEGISTVIYPSLQTEMTHRRERTETLIPEMRTDTYTDEIQEEITKSPFMGKTEEEV
FSGMKLSTSLSEPIHVTESSVEMTKSFDFPTLITKLSAEPTEVRDMEEDFTATPGTTKYD
ENITTVLLAHGTLSVEAATVSKWSWDEDNTTSKPLESTEPSASSKLPPALLTTVGMNGKD
KDIPSFTEDGADEFTLIPDSTQKQLEEVTDEDIAAHGKFTIRFQPTTSTGIAEKSTLRDS
TTEEKVPPITSTEGQVYATMEGSALGEVEDVDLSKPVSTVPQFAHTSEVEGLAFVSYSST
QEPTTYVDSSHTIPLSVIPKTDWGVLVPSVPSEDEVLGEPSQDILVIDQTRLEATISPET
MRTTKITEGTTQEEFPWKEQTAEKPVPALSSTAWTPKEAVTPLDEQEGDGSAYTVSEDEL
LTGSERVPVLETTPVGKIDHSVSYPPGAVTEHKVKTDEVVTLTPRIGPKVSLSPGPEQKY
ETEGSSTTGFTSSLSPFSTHITQLMEETTTEKTSLEDIDLGSGLFEKPKATELIEFSTIK
VTVPSDITTAFSSVDRLHTTSAFKPSSAITKKPPLIDREPGEETTSDMVIIGESTSHVPP
TTLEDIVAKETETDIDREYFTTSSPPATQPTRPPTVEDKEAFGPQALSTPQPPASTKFHP
DINVYIIEVRENKTGRMSDLSVIGHPIDSESKEDEPCSEETDPVHDLMAEILPEFPDIIE
IDLYHSEENEEEEEECANATDVTTTPSVQYINGKHLVTTVPKDPEAAEARRGQFESVAPS
QNFSDSSESDTHPFVIAKTELSTAVQPNESTETTESLEVTWKPETYPETSEHFSGGEPDV
FPTVPFHEEFESGTAKKGAESVTERDTEVGHQAHEHTEPVSLFPEESSGEIAIDQESQKI
AFARATEVTFGEEVEKSTSVTYTPTIVPSSASAYVSEEEAVTLIGNPWPDDLLSTKESWV
EATPRQVVELSGSSSIPITEGSGEAEEDEDTMFTMVTDLSQRNTTDTLITLDTSRIITES
FFEVPATTIYPVSEQPSAKVVPTKFVSETDTSEWISSTTVEEKKRKEEEGTTGTASTFEV
YSSTQRSDQLILPFELESPNVATSSDSGTRKSFMSLTTPTQSEREMTDSTPVFTETNTLE
NLGAQTTEHSSIHQPGVQEGLTTLPRSPASVFMEQGSGEAAADPETTTVSSFSLNVEYAI
QAEKEVAGTLSPHVETTFSTEPTGLVLSTVMDRVVAENITQTSREIVISERLGEPNYGAE
IRGFSTGFPLEEDFSGDFREYSTVSHPIAKEETVMMEGSGDAAFRDTQTSPSTVPTSVHI
SHISDSEGPSSTMVSTSAFPWEEFTSSAEGSGEQLVTVSSSVVPVLPSAVQKFSGTASSI
IDEGLGEVGTVNEIDRRSTILPTAEVEGTKAPVEKEEVKVSGTVSTNFPQTIEPAKLWSR
QEVNPVRQEIESETTSEEQIQEEKSFESPQNSPATEQTIFDSQTFTETELKTTDYSVLTT
KKTYSDDKEMKEEDTSLVNMSTPDPDANGLESYTTLPEATEKSHFFLATALVTESIPAEH
VVTDSPIKKEESTKHFPKGMRPTIQESDTELLFSGLGSGEEVLPTLPTESVNFTEVEQIN
NTLYPHTSQVESTSSDKIEDFNRMENVAKEVGPLVSQTDIFEGSGSVTSTTLIEILSDTG
AEGPTVAPLPFSTDIGHPQNQTVRWAEEIQTSRPQTITEQDSNKNSSTAEINETTTSSTD
FLARAYGFEMAKEFVTSAPKPSDLYYEPSGEGSGEVDIVDSFHTSATTQATRQESSTTFV
SDGSLEKHPEVPSAKAVTADGFPTVSVMLPLHSEQNKSSPDPTSTLSNTVSYERSTDGSF
QDRFREFEDSTLKPNRKKPTENIIIDLDKEDKDLILTITESTILEILPELTSDKNTIIDI
DHTKPVYEDILGMQTDIDTEVPSEPHDSNDESNDDSTQVQEIYEAAVNLSLTEETFEGSA
DVLASYTQATHDESMTYEDRSQLDHMGFHFTTGIPAPSTETELDVLLPTATSLPIPRKSA
TVIPEIEGIKAEAKALDDMFESSTLSDGQAIADQSEIIPTLGQFERTQEEYEDKKHAGPS
FQPEFSSGAEEALVDHTPYLSIATTHLMDQSVTEVPDVMEGSNPPYYTDTTLAVSTFAKL
SSQTPSSPLTIYSGSEASGHTEIPQPSALPGIDVGSSVMSPQDSFKEIHVNIEATFKPSS
EEYLHITEPPSLSPDTKLEPSEDDGKPELLEEMEASPTELIAVEGTEILQDFQNKTDGQV

SGEAIKMFPTIKTPEAGTVITTADEIELEGATQWPHSTSASATYGVEAGVVPWLSPQTSE
RPTLSSSPEINPETQAALIRGQDSTIAASEQQVAARILDSNDQATVNPVEFNTEVATPPF
SLLETSNETDFLIGINEESVEGTAIYLPGPDRCKMNPCLNGGTCYPTETSYVCTCVPGYS
GDQCELDFDECHSNPCRNGATCVDGFNTFRCLCLPSYVGALCEQDTETCDYGWHKFQGQC
YKYFAHRRTWDAAERECRLQGAHLTSILSHEEQMFVNRVGHDYQWIGLNDKMFEHDFRWT
DGSTLQYENWRPNQPDSFFSAGEDCVVIIWHENGQWNDVPCNYHLTYTCKKGTVACGQPP
VVENAKTFGKMKPRYEINSLIRYHCKDGFIQRHLPTIRCLGNGRWAIPKITCMNPSAYQR
TYSMKYFKNSSSAKDNSINTSKHDHRWSRRWQESRR
>sp|Q08188|TGM3_HUMAN Protein-glutamine gamma-glutamyltransferase E OS=Homo sapiens OX=9606 GN=TGM3 PE=1 SV=4
MAALGVQSINWQTAFNRQAHHTDKFSSQELILRRGQNFQVLMIMNKGLGSNERLEFIVST
GPYPSESAMTKAVFPLSNGSSGGWSAVLQASNGNTLTISISSPASAPIGRYTMALQIFSQ
GGISSVKLGTFILLFNPWLNVDSVFMGNHAEREEYVQEDAGIIFVGSTNRIGMIGWNFGQ
FEEDILSICLSILDRSLNFRRDAATDVASRNDPKYVGRVLSAMINSNDDNGVLAGNWSGT
YTGGRDPRSWNGSVEILKNWKKSGFSPVRYGQCWVFAGTLNTALRSLGIPSRVITNFNSA
HDTDRNLSVDVYYDPMGNPLDKGSDSVWNFHVWNEGWFVRSDLGPSYGGWQVLDATPQER
SQGVFQCGPASVIGVREGDVQLNFDMPFIFAEVNADRITWLYDNTTGKQWKNSVNSHTIG
RYISTKAVGSNARMDVTDKYKYPEGSDQERQVFQKALGKLKPNTPFAATSSMGLETEEQE
PSIIGKLKVAGMLAVGKEVNLVLLLKNLSRDTKTVTVNMTAWTIIYNGTLVHEVWKDSAT
MSLDPEEEAEHPIKISYAQYEKYLKSDNMIRITAVCKVPDESEVVVERDIILDNPTLTLE
VLNEARVRKPVNVQMLFSNPLDEPVRDCVLMVEGSGLLLGNLKIDVPTLGPKEGSRVRFD
ILPSRSGTKQLLADFSCNKFPAIKAMLSIDVAE
>sp|P02760|AMBP_HUMAN Protein AMBP OS=Homo sapiens OX=9606 GN=AMBP PE=1 SV=1
MRSLGALLLLLSACLAVSAGPVPTPPDNIQVQENFNISRIYGKWYNLAIGSTCPWLKKIM
DRMTVSTLVLGEGATEAEISMTSTRWRKGVCEETSGAYEKTDTDGKFLYHKSKWNITMES
YVVHTNYDEYAIFLTKKFSRHHGPTITAKLYGRAPQLRETLLQDFRVVAQGVGIPEDSIF
TMADRGECVPGEQEPEPILIPRVRRAVLPQEEEGSGGGQLVTEVTKKEDSCQLGYSAGPC
MGMTSRYFYNGTSMACETFQYGGCMGNGNNFVTEKECLQTCRTVAACNLPIVRGPCRAFI
QLWAFDAVKGKCVLFPYGGCQGNGNKFYSEKECREYCGVPGDGDEELLRFSN
>sp|P07339|CATD_HUMAN Cathepsin D OS=Homo sapiens OX=9606 GN=CTSD PE=1 SV=1
MQPSSLLPLALCLLAAPASALVRIPLHKFTSIRRTMSEVGGSVEDLIAKGPVSKYSQAVP
AVTEGPIPEVLKNYMDAQYYGEIGIGTPPQCFTVVFDTGSSNLWVPSIHCKLLDIACWIH
HKYNSDKSSTYVKNGTSFDIHYGSGSLSGYLSQDTVSVPCQSASSASALGGVKVERQVFG
EATKQPGITFIAAKFDGILGMAYPRISVNNVLPVFDNLMQQKLVDQNIFSFYLSRDPDAQ
PGGELMLGGTDSKYYKGSLSYLNVTRKAYWQVHLDQVEVASGLTLCKEGCEAIVDTGTSL
MVGPVDEVRELQKAIGAVPLIQGEYMIPCEKVSTLPAITLKLGGKGYKLSPEDYTLKVSQ
AGKTLCLSGFMGMDIPPPSGPLWILGDVFIGRYYTVFDRDNNRVGFAEAARL
>sp|P20849|CO9A1_HUMAN Collagen alpha-1(IX) chain OS=Homo sapiens OX=9606 GN=COL9A1 PE=1 SV=3
MKTCWKIPVFFFVCSFLEPWASAAVKRRPRFPVNSNSNGGNELCPKIRIGQDDLPGFDLI
SQFQVDKAASRRAIQRVVGSATLQVAYKLGNNVDFRIPTRNLYPSGLPEEYSFLTTFRMT
GSTLKKNWNIWQIQDSSGKEQVGIKINGQTQSVVFSYKGLDGSLQTAAFSNLSSLFDSQW
HKIMIGVERSSATLFVDCNRIESLPIKPRGPIDIDGFAVLGKLADNPQVSVPFELQWMLI
HCDPLRPRRETCHELPARITPSQTTDERGPPGEQGPPGPPGPPGVPGIDGIDGDRGPKGP

PGPPGPAGEPGKPGAPGKPGTPGADGLTGPDGSPGSIGSKGQKGEPGVPGSRGFPGRGIP
GPPGPPGTAGLPGELGRVGPVGDPGRRGPPGPPGPPGPRGTIGFHDGDPLCPNACPPGRS
GYPGLPGMRGHKGAKGEIGEPGRQGHKGEEGDQGELGEVGAQGPPGAQGLRGITGIVGDK
GEKGARGLDGEPGPQGLPGAPGDQGQRGPPGEAGPKGDRGAEGARGIPGLPGPKGDTGLP
GVDGRDGIPGMPGTKGEPGKPGPPGDAGLQGLPGVPGIPGAKGVAGEKGSTGAPGKPGQM
GNSGKPGQQGPPGEVGPRGPQGLPGSRGELGPVGSPGLPGKLGSLGSPGLPGLPGPPGLP
GMKGDRGVVGEPGPKGEQGASGEEGEAGERGELGDIGLPGPKGSAGNPGEPGLRGPEGSR
GLPGVEGPRGPPGPRGVQGEQGATGLPGVQGPPGRAPTDQHIKQVCMRVIQEHFAEMAAS
LKRPDSGATGLPGRPGPPGPPGPPGENGFPGQMGIRGLPGIKGPPGALGLRGPKGDLGEK
GERGPPGRGPNGLPGAIGLPGDPGPASYGRNGRDGERGPPGVAGIPGVPGPPGPPGLPGF
CEPASCTMQAGQRAFNKGPDP
>sp|P36955|PEDF_HUMAN Pigment epithelium-derived factor OS=Homo sapiens OX=9606
GN=SERPINF1 PE=1 SV=4
MQALVLLLCIGALLGHSSCQNPASPPEEGSPDPDSTGALVEEEDPFFKVPVNKLAAAVSN
FGYDLYRVRSSTSPTTNVLLSPLSVATALSALSLGAEQRTESIIHRALYYDLISSPDIHG
TYKELLDTVTAPQKNLKSASRIVFEKKLRIKSSFVAPLEKSYGTRPRVLTGNPRLDLQEI
NNWVQAQMKGKLARSTKEIPDEISILLLGVAHFKGQWVTKFDSRKTSLEDFYLDEERTVR
VPMMSDPKAVLRYGLDSDLSCKIAQLPLTGSMSIIFFLPLKVTQNLTLIEESLTSEFIHD
IDRELKTVQAVLTVPKLKLSYEGEVTKSLQEMKLQSLFDSPDFSKITGKPIKLTQVEHRA
GFEWNEDGAGTTPSPGLQPAHLTFPLDYHLNQPFIFVLRDTDTGALLFIGKILDPRGP
>sp|Q16610|ECM1_HUMAN Extracellular matrix protein 1 OS=Homo sapiens OX=9606
GN=ECM1 PE=1 SV=2
MGTTARAALVLTYLAVASAASEGGFTATGQRQLRPEHFQEVGYAAPPSPPLSRSLPMDHP
DSSQHGPPFEGQSQVQPPPSQEATPLQQEKLLPAQLPAEKEVGPPLPQEAVPLQKELPSL
QHPNEQKEGTPAPFGDQSHPEPESWNAAQHCQQDRSQGGWGHRLDGFPPGRPSPDNLNQI
CLPNRQHVVYGPWNLPQSSYSHLTRQGETLNFLEIGYSRCCHCRSHTNRLECAKLVWEEA
MSRFCEAEFSVKTRPHWCCTRQGEARFSCFQEEAPQPHYQLRACPSHQPDISSGLELPFP
PGVPTLDNIKNICHLRRFRSVPRNLPATDPLQRELLALIQLEREFQRCCRQGNNHTCTWK
AWEDTLDKYCDREYAVKTHHHLCCRHPPSPTRDECFARRAPYPNYDRDILTIDIGRVTPN
LMGHLCGNQRVLTKHKHIPGLIHNMTARCCDLPFPEQACCAEEEKLTFINDLCGPRRNIW
RDPALCCYLSPGDEQVNCFNINYLRNVALVSGDTENAKGQGEQGSTGGTNISSTSEPKEE
>sp|Q96P63|SPB12_HUMAN Serpin B12 OS=Homo sapiens OX=9606 GN=SERPINB12 PE=1 SV=1
MDSLVTANTKFCFDLFQEIGKDDRHKNIFFSPLSLSAALGMVRLGARSDSAHQIDEVLHF
NEFSQNESKEPDPCLKSNKQKAGSLNNESGLVSCYFGQLLSKLDRIKTDYTLSIANRLYG
EQEFPICQEYLDGVIQFYHTTIESVDFQKNPEKSRQEINFWVECQSQGKIKELFSKDAIN
AETVLVLVNAVYFKAKWETYFDHENTVDAPFCLNANENKSVKMMTQKGLYRIGFIEEVKA
QILEMRYTKGKLSMFVLLPSHSKDNLKGLEELERKITYEKMVAWSSSENMSEESVVLSFP
RFTLEDSYDLNSILQDMGITDIFDETRADLTGISPSPNLYLSKIIHKTFVEVDENGTQAA
AATGAVVSERSLRSWVEFNANHPFLFFIRHNKTQTILFYGRVCSP
>sp|P08133|ANXA6_HUMAN Annexin A6 OS=Homo sapiens OX=9606 GN=ANXA6 PE=1 SV=3
MAKPAQGAKYRGSIHDFPGFDPNQDAEALYTAMKGFGSDKEAILDIITSRSNRQRQEVCQ
SYKSLYGKDLIADLKYELTGKFERLIVGLMRPPAYCDAKEIKDAISGIGTDEKCLIEILA
SRTNEQMHQLVAAYKDAYERDLEADIIGDTSGHFQKMLVVLLQGTREEDDVVSEDLVQQD
VQDLYEAGELKWGTDEAQFIYILGNRSKQHLRLVFDEYLKTTGKPIEASIRGELSGDFEK

LMLAVVKCIRSTPEYFAERLFKAMKGLGTRDNTLIRIMVSRSELDMLDIREIFRTKYEKS
LYSMIKNDTSGEYKKTLLKLSGGDDDAAGQFFPEAAQVAYQMWELSAVARVELKGTVRPA
NDFNPDADAKALRKAMKGLGTDEDTIIDIITHRSNVQRQQIRQTFKSHFGRDLMTDLKSE
ISGDLARLILGLMMPPAHYDAKQLKKAMEGAGTDEKALIEILATRTNAEIRAINEAYKED
YHKSLEDALSSDTSGHFRRILISLATGHREEGGENLDQAREDAQVAAEILEIADTPSGDK
TSLETRFMTILCTRSYPHLRRVFQEFIKMTNYDVEHTIKKEMSGDVRDAFVAIVQSVKNK
PLFFADKLYKSMKGAGTDEKTLTRIMVSRSEIDLLNIRREFIEKYDKSLHQAIEGDTSGD
FLKALLALCGGED
>sp|P53420|CO4A4_HUMAN Collagen alpha-4(IV) chain OS=Homo sapiens OX=9606
GN=COL4A4 PE=1 SV=3
MWSLHIVLMRCSFRLTKSLATGPWSLILILFSVQYVYGSGKKYIGPCGGRDCSVCHCVPE
KGSRGPPGPPGPQGPIGPLGAPGPIGLSGEKGMRGDRGPPGAAGDKGDKGPTGVPGFPGL
DGIPGHPGPPGPRGKPGMSGHNGSRGDPGFPGGRGALGPGGPLGHPGEKGEKGNSVFILG
AVKGIQGDRGDPGLPGLPGSWGAGGPAGPTGYPGEPGLVGPPGQPGRPGLKGNPGVGVKG
QMGDPGEVGQQGSPGPTLLVEPPDFCLYKGEKGIKGIPGMVGLPGPPGRKGESGIGAKGE
KGIPGFPGPRGDPGSYGSPGFPGLKGELGLVGDPGLFGLIGPKGDPGNRGHPGPPGVLVT
PPLPLKGPPGDPGFPGRYGETGDVGPPGPPGLLGRPGEACAGMIGPPGPQGFPGLPGLPG
EAGIPGRPDSAPGKPGKPGSPGLPGAPGLQGLPGSSVIYCSVGNPGPQGIKGKVGPPGGR
GPKGEKGNEGLCACEPGPMGPPGPPGLPGRQGSKGDLGLPGWLGTKGDPGPPGAEGPPGL
PGKHGASGPPGNKGAKGDMVVSRVKGHKGERGPDGPPGFPGQPGSHGRDGHAGEKGDPGP
PGDHEDATPGGKGFPGPLGPPGKAGPVGPPGLGFPGPPGERGHPGVPGHPGVRGPDGLKG
QKGDTISCNVTYPGRHGPPGFDGPPGPKGFPGPQGAPGLSGSDGHKGRPGTPGTAEIPGP
PGFRGDMGDPGFGGEKGSSPVGPPGPPGSPGVNGQKGIPGDPAFGHLGPPGKRGLSGVPG
IKGPRGDPGCPGAEGPAGIPGFLGLKGPKGREGHAGFPGVPGPPGHSCERGAPGIPGQPG
LPGYPGSPGAPGGKGQPGDVGPPGPAGMKGLPGLPGRPGAHGPPGLPGIPGPFGDDGLPG
PPGPKGPRGLPGFPGFPGERGKPGAEGCPGAKGEPGEKGMSGLPGDRGLRGAKGAIGPPG
DEGEMAIISQKGTPGEPGPPGDDGFPGERGDKGTPGMQGRRGEPGRYGPPGFHRGEPGEK
GQPGPPGPPGPPGSTGLRGFIGFPGLPGDQGEPGSPGPPGFSGIDGARGPKGNKGDPASH
FGPPGPKGEPGSPGCPGHFGASGEQGLPGIQGPRGSPGRPGPPGSSGPPGCPGDHGMPGL
RGQPGEMGDPGPRGLQGDPGIPGPPGIKGPSGSPGLNGLHGLKGQKGTKGASGLHDVGPP
GPVGIPGLKGERGDPGSPGISPPGPRGKKGPPGPPGSSGPPGPAGATGRAPKDIPDPGPP
GDQGPPGPDGPRGAPGPPGLPGSVDLLRGEPGDCGLPGPPGPPGPPGPPGYKGFPGCDGK
DGQKGPVGFPGPQGPHGFPGPPGEKGLPGPPGRKGPTGLPGPRGEPGPPADVDDCPRIPG
LPGAPGMRGPEGAMGLPGMRGPSGPGCKGEPGLDGRRGVDGVPGSPGPPGRKGDTGEDGY
PGGPGPPGPIGDPGPKGFGPGYLGGFLLVLHSQTDQEPTCPLGMPRLWTGYSLLYLEGQE
KAHNQDLGLAGSCLPVFSTLPFAYCNIHQVCHYAQRNDRSYWLASAAPLPMMPLSEEAIR
PYVSRCAVCEAPAQAVAVHSQDQSIPPCPQTWRSLWIGYSFLMHTGAGDQGGGQALMSPG
SCLEDFRAAPFLECQGRQGTCHFFANKYSFWLTTVKADLQFSSAPAPDTLKESQAQRQKI
SRCQVCVKYS
>sp|Q01955|CO4A3_HUMAN Collagen alpha-3(IV) chain OS=Homo sapiens OX=9606
GN=COL4A3 PE=1 SV=3
MSARTAPRPQVLLLPLLLVLLAAAPAASKGCVCKDKGQCFCDGAKGEKGEKGFPGPPGSP
GQKGFTGPEGLPGPQGPKGFPGLPGLTGSKGVRGISGLPGFSGSPGLPGTPGNTGPYGLV
GVPGCSGSKGEQGFPGLPGTLGYPGIPGAAGLKGQKGAPAKEEDIELDAKGDPGLPGAPG

PQGLPGPPGFPGPVGPPGPPGFFGFPGAMGPRGPKGHMGERVIGHKGERGVKGLTGPPGP
PGTVIVTLTGPDNRTDLKGEKGDKGAMGEPGPPGPSGLPGESYGSEKGAPGDPGLQGKPG
KDGVPGFPGSEGVKGNRGFPGLMGEDGIKGQKGDIGPPGFRGPTEYYDTYQEKGDEGTPG
PPGPRGARGPQGPSGPPGVPGSPGSSRPGLRGAPGWPGLKGSKGERGRPGKDAMGTPGSP
GCAGSPGLPGSPGPPGPPGDIVFRKGPPGDHGLPGYLGSPGIPGVDGPKGEPGLLCTQCP
YIPGPPGLPGLPGLHGVKGIPGRQGAAGLKGSPGSPGNTGLPGFPGFPGAQGDPGLKGEK
GETLQPEGQVGVPGDPGLRGQPGRKGLDGIPGTPGVKGLPGPKGELALSGEKGDQGPPGD
PGSPGSPGPAGPAGPPGYGPQGEPGLQGTQGVPGAPGPPGEAGPRGELSVSTPVPGPPGP
PGPPGHPGPQGPPGIPGSLGKCGDPGLPGPDGEPGIPGIGFPGPPGPKGDQGFPGTKGSL
GCPGKMGEPGLPGKPGLPGAKGEPAVAMPGGPGTPGFPGERGNSGEHGEIGLPGLPGLPG
TPGNEGLDGPRGDPGQPGPPGEQGPPGRCIEGPRGAQGLPGLNGLKGQQGRRGKTGPKGD
PGIPGLDRSGFPGETGSPGIPGHQGEMGPLGQRGYPGNPGILGPPGEDGVIGMMGFPGAI
GPPGPPGNPGTPGQRGSPGIPGVKGQRGTPGAKGEQGDKGNPGPSEISHVIGDKGEPGLK
GFAGNPGEKGNRGVPGMPGLKGLKGLPGPAGPPGPRGDLGSTGNPGEPGLRGIPGSMGNM
GMPGSKGKRGTLGFPGRAGRPGLPGIHGLQGDKGEPGYSEGTRPGPPGPTGDPGLPGDMG
KKGEMGQPGPPGHLGPAGPEGAPGSPGSPGLPGKPGPHGDLGFKGIKGLLGPPGIRGPPG
LPGFPGSPGPMGIRGDQGRDGIPGPAGEKGETGLLRAPPGPRGNPGAQGAKGDRGAPGFP
GLPGRKGAMGDAGPRGPTGIEGFPGPPGLPGAIIPGQTGNRGPPGSRGSPGAPGPPGPPG
SHVIGIKGDKGSMGHPGPKGPPGTAGDMGPPGRLGAPGTPGLPGPRGDPGFQGFPGVKGE
KGNPGFLGSIGPPGPIGPKGPPGVRGDPGTLKIISLPGSPGPPGTPGEPGMQGEPGPPGP
PGNLGPCGPRGKPGKDGKPGTPGPAGEKGNKGSKGEPGPAGSDGLPGLKGKRGDSGSPAT
WTTRGFVFTRHSQTTAIPSCPEGTVPLYSGFSFLFVQGNQRAHGQDLGTLGSCLQRFTTM
PFLFCNVNDVCNFASRNDYSYWLSTPALMPMNMAPITGRALEPYISRCTVCEGPAIAIAV
HSQTTDIPPCPHGWISLWKGFSFIMFTSAGSEGTGQALASPGSCLEEFRASPFLECHGRG
TCNYYSNSYSFWLASLNPERMFRKPIPSTVKAGELEKIISRCQVCMKKRH
>sp|Q14112|NID2_HUMAN Nidogen-2 OS=Homo sapiens OX=9606 GN=NID2 PE=1 SV=3
MEGDRVAGRPVLSSLPVLLLLPLLMLRAAALHPDELFPHGESWGDQLLQEGDDESSAVVK
LANPLHFYEARFSNLYVGTNGIISTQDFPRETQYVDYDFPTDFPAIAPFLADIDTSHGRG
RVLYREDTSPAVLGLAARYVRAGFPRSARFTPTHAFLATWEQVGAYEEVKRGALPSGELN
TFQAVLASDGSDSYALFLYPANGLQFLGTRPKESYNVQLQLPARVGFCRGEADDLKSEGP
YFSLTSTEQSVKNLYQLSNLGIPGVWAFHIGSTSPLDNVRPAAVGDLSAAHSSVPLGRSF
SHATALESDYNEDNLDYYDVNEEEAEYLPGEPEEALNGHSSIDVSFQSKVDTKPLEESST
LDPHTKEGTSLGEVGGPDLKGQVEPWDERETRSPAPPEVDRDSLAPSWETPPPYPENGSI
QPYPDGGPVPSEMDVPPAHPEEEIVLRSYPASGHTTPLSRGTYEVGLEDNIGSNTEVFTY
NAANKETCEHNHRQCSRHAFCTDYATGFCCHCQSKFYGNGKHCLPEGAPHRVNGKVSGHL
HVGHTPVHFTDVDLHAYIVGNDGRAYTAISHIPQPAAQALLPLTPIGGLFGWLFALEKPG
SENGFSLAGAAFTHDMEVTFYPGEETVRITQTAEGLDPENYLSIKTNIQGQVPYVSANFT
AHISPYKELYHYSDSTVTSTSSRDYSLTFGAINQTWSYRIHQNITYQVCRHAPRHPSFPT
TQQLNVDRVFALYNDEERVLRFAVTNQIGPVKEDSDPTPGNPCYDGSHMCDTTARCHPGT
GVDYTCECASGYQGDGRNCVDENECATGFHRCGPNSVCINLPGSYRCECRSGYEFADDRH
TCILITPPANPCEDGSHTCAPAGQARCVHHGGSTFSCACLPGYAGDGHQCTDVDECSENR
CHPAATCYNTPGSFSCRCQPGYYGDGFQCIPDSTSSLTPCEQQQRHAQAQYAYPGARFHI
PQCDEQGNFLPLQCHGSTGFCWCVDPDGHEVPGTQTPPGSTPPHCGPSPEPTQRPPTICE
RWRENLLEHYGGTPRDDQYVPQCDDLGHFIPLQCHGKSDFCWCVDKDGREVQGTRSQPGT

TPACIPTVAPPMVRPTPRPDVTPPSVGTFLLYTQGQQIGYLPLNGTRLQKDAAKTLLSLH
GSIIVGIDYDCRERMVYWTDVAGRTISRAGLELGAEPETIVNSGLISPEGLAIDHIRRTM
YWTDSVLDKIESALLDGSERKVLFYTDLVNPRAIAVDPIRGNLYWTDWNREAPKIETSSL
DGENRRILINTDIGLPNGLTFDPFSKLLCWADAGTKKLECTLPDGTGRRVIQNNLKYPFS
IVSYADHFYHTDWRRDGVVSVNKHSGQFTDEYLPEQRSHLYGITAVYPYCPTGRK
>sp|Q14624|ITIH4_HUMAN Inter-alpha-trypsin inhibitor heavy chain H4 OS=Homo sapiens
OX=9606 GN=ITIH4 PE=1 SV=4
MKPPRPVRTCSKVLVLLSLLAIHQTTTAEKNGIDIYSLTVDSRVSSRFAHTVVTSRVVNR
ANTVQEATFQMELPKKAFITNFSMIIDGMTYPGIIKEKAEAQAQYSAAVAKGKSAGLVKA
TGRNMEQFQVSVSVAPNAKITFELVYEELLKRRLGVYELLLKVRPQQLVKHLQMDIHIFE
PQGISFLETESTFMTNQLVDALTTWQNKTKAHIRFKPTLSQQQKSPEQQETVLDGNLIIR
YDVDRAISGGSIQIENGYFVHYFAPEGLTTMPKNVVFVIDKSGSMSGRKIQQTREALIKI
LDDLSPRDQFNLIVFSTEATQWRPSLVPASAENVNKARSFAAGIQALGGTNINDAMLMAV
QLLDSSNQEERLPEGSVSLIILLTDGDPTVGETNPRSIQNNVREAVSGRYSLFCLGFGFD
VSYAFLEKLALDNGGLARRIHEDSDSALQLQDFYQEVANPLLTAVTFEYPSNAVEEVTQN
NFRLLFKGSEMVVAGKLQDRGPDVLTATVSGKLPTQNITFQTESSVAEQEAEFQSPKYIF
HNFMERLWAYLTIQQLLEQTVSASDADQQALRNQALNLSLAYSFVTPLTSMVVTKPDDQE
QSQVAEKPMEGESRNRNVHSGSTFFKYYLQGAKIPKPEASFSPRRGWNRQAGAAGSRMNF
RPGVLSSRQLGLPGPPDVPDHAAYHPFRRLAILPASAPPATSNPDPAVSRVMNMKIEETT
MTTQTPAPIQAPSAILPLPGQSVERLCVDPRHRQGPVNLLSDPEQGVEVTGQYEREKAGF
SWIEVTFKNPLVWVHASPEHVVVTRNRRSSAYKWKETLFSVMPGLKMTMDKTGLLLLSDP
DKVTIGLLFWDGRGEGLRLLLRDTDRFSSHVGGTLGQFYQEVLWGSPAASDDGRRTLRVQ
GNDHSATRERRLDYQEGPPGVEISCWSVEL
>sp|Q86UX2|ITIH5_HUMAN Inter-alpha-trypsin inhibitor heavy chain H5 OS=Homo sapiens
OX=9606 GN=ITIH5 PE=2 SV=2
MLLLLGLCLGLSLCVGSQEEAQSWGHSSEQDGLRVPRQVRLLQRLKTKPLMTEFSVKSTI
ISRYAFTTVSCRMLNRASEDQDIEFQMQIPAAAFITNFTMLIGDKVYQGEITEREKKSGD
RVKEKRNKTTEENGEKGTEIFRASAVIPSKDKAAFFLSYEELLQRRLGKYEHSISVRPQQ
LSGRLSVDVNILESAGIASLEVLPLHNSRQRGSGRGEDDSGPPPSTVINQNETFANIIFK
PTVVQQARIAQNGILGDFIIRYDVNREQSIGDIQVLNGYFVHYFAPKDLPPLPKNVVFVL
DSSASMVGTKLRQTKDALFTILHDLRPQDRFSIIGFSNRIKVWKDHLISVTPDSIRDGKV
YIHHMSPTGGTDINGALQRAIRLLNKYVAHSGIGDRSVSLIVFLTDGKPTVGETHTLKIL
NNTREAARGQVCIFTIGIGNDVDFRLLEKLSLENCGLTRRVHEEEDAGSQLIGFYDEIRT
PLLSDIRIDYPPSSVVQATKTLFPNYFNGSEIIIAGKLVDRKLDHLHVEVTASNSKKFII
LKTDVPVRPQKAGKDVTGSPRPGGDGEGDTNHIERLWSYLTTKELLSSWLQSDDEPEKER
LRQRAQALAVSYRFLTPFTSMKLRGPVPRMDGLEEAHGMSAAMGPEPVVQSVRGAGTQPG
PLLKKPYQPRIKISKTSVDGDPHFVVDFPLSRLTVCFNIDGQPGDILRLVSDHRDSGVTV
NGELIGAPAPPNGHKKQRTYLRTITILINKPERSYLEITPSRVILDGGDRLVLPCNQSVV
VGSWGLEVSVSANANVTVTIQGSIAFVILIHLYKKPAPFQRHHLGFYIANSEGLSSNCHG
LLGQFLNQDARLTEDPAGPSQNLTHPLLLQVGEGPEAVLTVKGHQVPVVWKQRKIYNGEE
QIDCWFARNNAAKLIDGEYKDYLAFHPFDTGMTLGQGMSREL
>sp|Q8N6G6|ATL1_HUMAN ADAMTS-like protein 1 OS=Homo sapiens OX=9606
GN=ADAMTSL1 PE=1 SV=4
MECCRRATPGTLLLFLAFLLLSSRTARSEEDRDGLWDAWGPWSECSRTCGGGASYSLRRC

LSSKSCEGRNIRYRTCSNVDCPPEAGDFRAQQCSAHNDVKHHGQFYEWLPVSNDPDNPCS
LKCQAKGTTLVVELAPKVLDGTRCYTESLDMCISGLCQIVGCDHQLGSTVKEDNCGVCNG
DGSTCRLVRGQYKSQLSATKSDDTVVAIPYGSRHIRLVLKGPDHLYLETKTLQGTKGENS
LSSTGTFLVDNSSVDFQKFPDKEILRMAGPLTADFIVKIRNSGSADSTVQFIFYQPIIHR
WRETDFFPCSATCGGGYQLTSAECYDLRSNRVVADQYCHYYPENIKPKPKLQECNLDPCP
ASDGYKQIMPYDLYHPLPRWEATPWTACSSSCGGGIQSRAVSCVEEDIQGHVTSVEEWKC
MYTPKMPIAQPCNIFDCPKWLAQEWSPCTVTCGQGLRYRVVLCIDHRGMHTGGCSPKTKP
HIKEECIVPTPCYKPKEKLPVEAKLPWFKQAQELEEGAAVSEEPSFIPEAWSACTVTCGV
GTQVRIVRCQVLLSFSQSVADLPIDECEGPKPASQRACYAGPCSGEIPEFNPDETDGLFG
GLQDFDELYDWEYEGFTKCSESCGGGVQEAVVSCLNKQTREPAEENLCVTSRRPPQLLKS
CNLDPCPARWEIGKWSPCSLTCGVGLQTRDVFCSHLLSREMNETVILADELCRQPKPSTV
QACNRFNCPPAWYPAQWQPCSRTCGGGVQKREVLCKQRMADGSFLELPETFCSASKPACQ
QACKKDDCPSEWLLSDWTECSTSCGEGTQTRSAICRKMLKTGLSTVVNSTLCPPLPFSSS
IRPCMLATCARPGRPSTKHSPHIAAARKVYIQTRRQRKLHFVVGGFAYLLPKTAVVLRCP
ARRVRKPLITWEKDGQHLISSTHVTVAPFGYLKIHRLKPSDAGVYTCSAGPAREHFVIKL
IGGNRKLVARPLSPRSEEEVLAGRKGGPKEALQTHKHQNGIFSNGSKAEKRGLAANPGSR
YDDLVSRLLEQGGWPGELLASWEAQDSAERNTTSEEDPGAEQVLLHLPFTMVTEQRRLDD
ILGNLSQQPEELRDLYSKHLVAQLAQEIFRSHLEHQDTLLKPSERRTSPVTLSPHKHVSG
FSSSLRTSSTGDAGGGSRRPHRKPTILRKISAAQQLSASEVVTHLGQTVALASGTLSVLL
HCEAIGHPRPTISWARNGEEVQFSDRILLQPDDSLQILAPVEADVGFYTCNATNALGYDS
VSIAVTLAGKPLVKTSRMTVINTEKPAVTVDIGSTIKTVQGVNVTINCQVAGVPEAEVTW
FRNKSKLGSPHHLHEGSLLLTNVSSSDQGLYSCRAANLHGELTESTQLLILDPPQVPTQL
EDIRALLAATGPNLPSVLTSPLGTQLVLDPGNSALLGCPIKGHPVPNITWFHGGQPIVTA
TGLTHHILAAGQILQVANLSGGSQGEFSCLAQNEAGVLMQKASLVIQDYWWSVDRLATCS
ASCGNRGVQQPRLRCLLNSTEVNPAHCAGKVRPAVQPIACNRRDCPSRWMVTSWSACTRS
CGGGVQTRRVTCQKLKASGISTPVSNDMCTQVAKRPVDTQACNQQLCVEWAFSSWGQCNG
PCIGPHLAVQHRQVFCQTRDGITLPSEQCSALPRPVSTQNCWSEACSVHWRVSLWTLCTA
TCGNYGFQSRRVECVHARTNKAVPEHLCSWGPRPANWQRCNITPCENMECRDTTRYCEKV
KQLKLCQLSQFKSRCCGTCGKA
>sp|P29400|CO4A5_HUMAN Collagen alpha-5(IV) chain OS=Homo sapiens OX=9606
GN=COL4A5 PE=1 SV=2
MKLRGVSLAAGLFLLALSLWGQPAEAAACYGCSPGSKCDCSGIKGEKGERGFPGLEGHPG
LPGFPGPEGPPGPRGQKGDDGIPGPPGPKGIRGPPGLPGFPGTPGLPGMPGHDGAPGPQG
IPGCNGTKGERGFPGSPGFPGLQGPPGPPGIPGMKGEPGSIIMSSLPGPKGNPGYPGPPG
IQGLPGPTGIPGPIGPPGPPGLMGPPGPPGLPGPKGNMGLNFQGPKGEKGEQGLQGPPGP
PGQISEQKRPIDVEFQKGDQGLPGDRGPPGPPGIRGPPGPPGGEKGEKGEQGEPGKRGKP
GKDGENGQPGIPGLPGDPGYPGEPGRDGEKGQKGDTGPPGPPGLVIPRPGTGITIGEKGN
IGLPGLPGEKGERGFPGIQGPPGLPGPPGAAVMGPPGPPGFPGERGQKGDEGPPGISIPG
PPGLDGQPGAPGLPGPPGPAGPHIPPSDEICEPGPPGPPGSPGDKGLQGEQGVKGDKGDT
CFNCIGTGISGPPGQPGLPGLPGPPGSLGFPGQKGEKGQAGATGPKGLPGIPGAPGAPGF
PGSKGEPGDILTFPGMKGDKGELGSPGAPGLPGLPGTPGQDGLPGLPGPKGEPGGITFKG
ERGPPGNPGLPGLPGNIGPMGPPGFGPPGPVGEKGIQGVAGNPGQPGIPGPKGDPGQTIT
QPGKPGLPGNPGRDGDVGLPGDPGLPGQPGLPGIPGSKGEPGIPGIGLPGPPGPKGFPGI
PGPPGAPGTPGRIGLEGPPGPPGFPGPKGEPGFALPGPPGPPGLPGFKGALGPKGDRGFP

GPPGPPGRTGLDGLPGPKGDVGPNGQPGPMGPPGLPGIGVQGPPGPPGIPGPIGQPGLHG
IPGEKGDPGPPGLDVPGPPGERGSPGIPGAPGPIGPPGSPGLPGKAGASGFPGTKGEMGM
MGPPGPPGPLGIPGRSGVPGLKGDDGLQGQPGLPGPTGEKGSKGEPGLPGPPGPMDPNLL
GSKGEKGEPGLPGIPGVSGPKGYQGLPGDPGQPGLSGQPGLPGPPGPKGNPGLPGQPGLI
GPPGLKGTIGDMGFPGPQGVEGPPGPSGVPGQPGSPGLPGQKGDKGDPGISSIGLPGLPG
PKGEPGLPGYPGNPGIKGSVGDPGLPGLPGTPGAKGQPGLPGFPGTPGPPGPKGISGPPG
NPGLPGEPGPVGGGGHPGQPGPPGEKGKPGQDGIPGPAGQKGEPGQPGFGNPGPPGLPGL
SGQKGDGGLPGIPGNPGLPGPKGEPGFHGFPGVQGPPGPPGSPGPALEGPKGNPGPQGPP
GRPGLPGPEGPPGLPGNGGIKGEKGNPGQPGLPGLPGLKGDQGPPGLQGNPGRPGLNGMK
GDPGLPGVPGFPGMKGPSGVPGSAGPEGEPGLIGPPGPPGLPGPSGQSIIIKGDAGPPGI
PGQPGLKGLPGPQGPQGLPGPTGPPGDPGRNGLPGFDGAGGRKGDPGLPGQPGTRGLDGP
PGPDGLQGPPGPPGTSSVAHGFLITRHSQTTDAPQCPQGTLQVYEGFSLLYVQGNKRAHG
QDLGTAGSCLRRFSTMPFMFCNINNVCNFASRNDYSYWLSTPEPMPMSMQPLKGQSIQPF
ISRCAVCEAPAVVIAVHSQTIQIPHCPQGWDSLWIGYSFMMHTSAGAEGSGQALASPGSC
LEEFRSAPFIECHGRGTCNYYANSYSFWLATVDVSDMFSKPQSETLKAGDLRTRISRCQV
CMKRT
>sp|P00747|PLMN_HUMAN Plasminogen OS=Homo sapiens OX=9606 GN=PLG PE=1 SV=2
MEHKEVVLLLLLFLKSGQGEPLDDYVNTQGASLFSVTKKQLGAGSIEECAAKCEEDEEFT
CRAFQYHSKEQQCVIMAENRKSSIIIRMRDVVLFEKKVYLSECKTGNGKNYRGTMSKTKN
GITCQKWSSTSPHRPRFSPATHPSEGLEENYCRNPDNDPQGPWCYTTDPEKRYDYCDILE
CEEECMHCSGENYDGKISKTMSGLECQAWDSQSPHAHGYIPSKFPNKNLKKNYCRNPDRE
LRPWCFTTDPNKRWELCDIPRCTTPPPSSGPTYQCLKGTGENYRGNVAVTVSGHTCQHWS
AQTPHTHNRTPENFPCKNLDENYCRNPDGKRAPWCHTTNSQVRWEYCKIPSCDSSPVSTE
QLAPTAPPELTPVVQDCYHGDGQSYRGTSSTTTTGKKCQSWSSMTPHRHQKTPENYPNAG
LTMNYCRNPDADKGPWCFTTDPSVRWEYCNLKKCSGTEASVVAPPPVVLLPDVETPSEED
CMFGNGKGYRGKRATTVTGTPCQDWAAQEPHRHSIFTPETNPRAGLEKNYCRNPDGDVGG
PWCYTTNPRKLYDYCDVPQCAAPSFDCGKPQVEPKKCPGRVVGGCVAHPHSWPWQVSLRT
RFGMHFCGGTLISPEWVLTAAHCLEKSPRPSSYKVILGAHQEVNLEPHVQEIEVSRLFLE
PTRKDIALLKLSSPAVITDKVIPACLPSPNYVVADRTECFITGWGETQGTFGAGLLKEAQ
LPVIENKVCNRYEFLNGRVQSTELCAGHLAGGTDSCQGDSGGPLVCFEKDKYILQGVTSW
GLGCARPNKPGVYVRVSRFVTWIEGVMRNN
>sp|P01009|A1AT_HUMAN Alpha-1-antitrypsin OS=Homo sapiens OX=9606 GN=SERPINA1
PE=1 SV=3
MPSSVSWGILLLAGLCCLVPVSLAEDPQGDAAQKTDTSHHDQDHPTFNKITPNLAEFAFS
LYRQLAHQSNSTNIFFSPVSIATAFAMLSLGTKADTHDEILEGLNFNLTEIPEAQIHEGF
QELLRTLNQPDSQLQLTTGNGLFLSEGLKLVDKFLEDVKKLYHSEAFTVNFGDTEEAKKQ
INDYVEKGTQGKIVDLVKELDRDTVFALVNYIFFKGKWERPFEVKDTEEEDFHVDQVTTV
KVPMMKRLGMFNIQHCKKLSSWVLLMKYLGNATAIFFLPDEGKLQHLENELTHDIITKFL
ENEDRRSASLHLPKLSITGTYDLKSVLGQLGITKVFSNGADLSGVTEEAPLKLSKAVHKA
VLTIDEKGTEAAGAMFLEAIPMSIPPEVKFNKPFVFLMIEQNTKSPLFMGKVVNPTQK
>sp|P01040|CYTA_HUMAN Cystatin-A OS=Homo sapiens OX=9606 GN=CSTA PE=1 SV=1
MIPGGLSEAKPATPEIQEIVDKVKPQLEEKTNETYGKLEAVQYKTQVVAGTNYYIKVRAG
DNKYMHLKVFKSLPGQNEDLVLTGYQVDKNKDDELTGF
>sp|P04271|S100B_HUMAN Protein S100-B OS=Homo sapiens OX=9606 GN=S100B PE=1 SV=2

MSELEKAMVALIDVFHQYSGREGDKHKLKKSELKELINNELSHFLEEIKEQEVVDKVMET
LDNDGDGECDFQEFMAFVAMVTTACHEFFEHE
>sp|P10600|TGFB3_HUMAN Transforming growth factor beta-3 proprotein OS=Homo sapiens OX=9606 GN=TGFB3 PE=1 SV=1
MKMHLQRALVVLALLNFATVSLSLSTCTTLDFGHIKKKRVEAIRGQILSKLRLTSPPEPT
VMTHVPYQVLALYNSTRELLEEMHGEREEGCTQENTESEYYAKEIHKFDMIQGLAEHNEL
AVCPKGITSKVFRFNVSSVEKNRTNLFRAEFRVLRVPNPSSKRNEQRIELFQILRPDEHI
AKQRYIGGKNLPTRGTAEWLSFDVTDTVREWLLRRESNLGLEISIHCPCHTFQPNGDILE
NIHEVMEIKFKGVDNEDDHGRGDLGRLKKQKDHHNPHLILMMIPPHRLDNPGQGGQRKKR
ALDTNYCFRNLEENCCVRPLYIDFRQDLGWKWVHEPKGYYANFCSGPCPYLRSADTTHST
VLGLYNTLNPEASASPCCVPQDLEPLTILYYVGRTPKVEQLSNMVVKSCKCS
>sp|Q13275|SEM3F_HUMAN Semaphorin-3F OS=Homo sapiens OX=9606 GN=SEMA3F PE=2 SV=2
MLVAGLLLWASLLTGAWPSFPTQDHLPATPRVRLSFKELKATGTAHFFNFLLNTTDYRIL
LKDEDHDRMYVGSKDYVLSLDLHDINREPLIIHWAASPQRIEECVLSGKDVNGECGNFVR
LIQPWNRTHLYVCGTGAYNPMCTYVNRGRRAQATPWTQTQAVRGRGSRATDGALRPMPTA
PRQDYIFYLEPERLESGKGKCPYDPKLDTASALINEELYAGVYIDFMGTDAAIFRTLGKQ
TAMRTDQYNSRWLNDPSFIHAELIPDSAERNDDKLYFFFRERSAEAPQSPAVYARIGRIC
LNDDGGHCCLVNKWSTFLKARLVCSVPGEDGIETHFDELQDVFVQQTQDVRNPVIYAVFT
SSGSVFRGSAVCVYSMADIRMVFNGPFAHKEGPNYQWMPFSGKMPYPRPGTCPGGTFTPS
MKSTKDYPDEVINFMRSHPLMYQAVYPLQRRPLVVRTGAPYRLTTIAVDQVDAADGRYEV
LFLGTDRGTVQKVIVLPKDDQELEELMLEEVEVFKDPAPVKTMTISSKRQQLYVASAVGV
THLSLHRCQAYGAACADCCLARDPYCAWDGQACSRYTASSKRRSRRQDVRHGNPIRQCRG
FNSNANKNAVESVQYGVAGSAAFLECQPRSPQATVKWLFQRDPGDRRREIRAEDRFLRTE
QGLLLRALQLSDRGLYSCTATENNFKHVVTRVQLHVLGRDAVHAALFPPLSMSAPPPPGA
GPPTPPYQELAQLLAQPEVGLIHQYCQGYWRHVPPSPREAPGAPRSPEPQDQKKPRNRRH
HPPDT
>sp|Q5D862|FILA2_HUMAN Filaggrin-2 OS=Homo sapiens OX=9606 GN=FLG2 PE=1 SV=1
MTDLLRSVVTVIDVFYKYTKQDGECGTLSKGELKELLEKELHPVLKNPDDPDTVDVIMHM
LDRDHDRRLDFTEFLLMIFKLTMACNKVLSKEYCKASGSKKHRRGHRHQEEESETEEDEE
DTPGHKSGYRHSSWSEGEEHGYSSGHSRGTVKCRHGSNSRRLGRQGNLSSSGNQEGSQKR
YHRSSCGHSWSGGKDRHGSSSVELRERINKSHISPSRESGEEYESGSGSNSWERKGHGGL
SCGLETSGHESNSTQSRIREQKLGSSCSGSGDSGRRSHACGYSNSSGCGRPQNASSSCQS
HRFGGQGNQFSYIQSGCQSGIKGGQGHGCVSGGQPSGCGQPESNPCSQSYSQRGYGAREN
GQPQNCGGQWRTGSSQSSCCGQYGSGGSQSCSNGQHEYGSCGRFSNSSSSNEFSKCDQYG
SGSSQSTSFEQHGTGLSQSSGFEQHVCGSGQTCGQHESTSSQSLGYDQHGSSSGKTSGFG
QHGSGSGQSSGFGQCGSGSGQSSGFGQHGSVSGQSSGFGQHGSVSGQSSGFGQHESRSRQ
SSYGQHGSGSSQSSGYGQYGSRETSGFGQHGLGSGQSTGFGQYGSGSGQSSGFGQHGSGS
GQSSGFGQHESRSGQSSYGQHSSGSSQSSGYGQHGSRQTSGFGQHGSGSSQSTGFGQYGS
GSGQSSGFGQHVSGSGQSSGFGQHESRSGHSSYGQHGFGSSQSSGYGQHGSSSGQTSGFG
QHELSSGQSSSFGQHGSGSGQSSGFGQHGSGSGQSSGFGQHESRSGQSSYGQHSSGSSQS
SGYGQHGSRQTSGFGQHGSGSSQSTGFGQYGSGSGQSAGFGQHGSGSGQSSGFGQHESRS
HQSSYGQHGSGSSQSSGYGQHGSSSGQTSGFGQHRSSSGQYSGFGQHGSGSGQSSGFGQH
GTGSGQYSGFGQHESRSHQSSYGQHGSGSSQSSGYGQHGSSSGQTFGFGQHRSGSGQSSG

FGQHGSGSGQSSGFGQHESGSGKSSGFGQHESRSSQSNYGQHGSGSSQSSGYGQHGSSSG
QTTGFGQHRSSSGQYSGFGQHGSGSDQSSGFGQHGTGSGQSSGFGQYESRSRQSSYGQHG
SGSSQSSGYGQHGSNSGQTSGFGQHRPGSGQSSGFGQYGSGSGQSSGFGQHGSGTGKSSG
FAQHEYRSGQSSYGQHGTGSSQSSGCGQHESGSGPTTSFGQHVSGSDNFSSSGQHISDSG
QSTGFGQYGSGSGQSTGLGQGESQQVESGSTVHGRQETTHGQTINTTRHSQSGQGQSTQT
GSRVTRRRRSSQSENSDSEVHSKVSHRHSEHIHTQAGSHYPKSGSTVRRRQGTTHGQRGD
TTRHGHSGHGQSTQTGSRTSGRQRFSHSDATDSEVHSGVSHRPHSQEQTHSQAGSQHGES
ESTVHERHETTYGQTGEATGHGHSGHGQSTQRGSRTTGRRGSGHSESSDSEVHSGGSHRP
QSQEQTHGQAGSQHGESGSTVHGRHGTTHGQTGDTTRHAHYHHGKSTQRGSSTTGRRGSG
HSESSDSEVHSGGSHTHSGHTHGQSGSQHGESESIIHDRHRITHGQTGDTTRHSYSGHEQ
TTQTGSRTTGRQRTSHSESTDSEVHSGGSHRPHSREHTYGQAGSQHEEPEFTVHERHGTT
HGQIGDTTGHSHSGHGQSTQRGSRTTGRQRSSHSESSDSEVHSGVSHTHTGHTHGQAGSQ
HGQSESIVPERHGTTHGQTGDTTRHAHYHHGLTTQTGSRTTGRRGSGHSEYSDSEGYSGV
SHTHSGHTHGQARSQHGESESIVHERHGTIHGQTGDTTRHAHSGHGQSTQTGSRTTGRRS
SGHSEYSDSEGHSGFSQRPHSRGHTHGQAGSQHGESESIVDERHGTTHGQTGDTSGHSQS
GHGQSTQSGSSTTGRRRSGHSESSDSEVHSGGSHTHSGHTHSQARSQHGESESTVHKRHQ
TTHGQTGDTTEHGHPSHGQTIQTGSRTTGRRGSGHSEYSDSEGPSGVSHTHSGHTHGQAG
SHYPESGSSVHERHGTTHGQTADTTRHGHSGHGQSTQRGSRTTGRRASGHSEYSDSEGHS
GVSHTHSGHAHGQAGSQHGESGSSVHERHGTTHGQTGDTTRHAHSGHGQSTQRGSRTAGR
RGSGHSESSDSEVHSGVSHTHSGHTYGQARSQHGESGSAIHGRQGTIHGQTGDTTRHGQS
GHGQSTQTGSRTTGRQRSSHSESSDSEVHSEASPTHSGHTHSQAGSRHGQSGSSGHGRQG
TTHGQTGDTTRHAHYGYGQSTQRGSRTTGRRGSGHSESSDSEVHSWGSHTHSGHIQGQAG
SQQRQPGSTVHGRLETTHGQTGDTTRHGHSGYGQSTQTGSRSSRASHFQSHSSERQRHGS
SQVWKHGSYGPAEYDYGHTGYGPSGGSRKSISNSHLSWSTDSTANKQLSRH
>sp|O00468|AGRIN_HUMAN Agrin OS=Homo sapiens OX=9606 GN=AGRN PE=1 SV=6
MAGRSHPGPLRPLLPLLVVAACVLPGAGGTCPERALERREEEANVVLTGTVEEILNVDPV
QHTYSCKVRVWRYLKGKDLVARESLLDGGNKVVISGFGDPLICDNQVSTGDTRIFFVNPA
PPYLWPAHKNELMLNSSLMRITLRNLEEVEFCVEDKPGTHFTPVPPTPPDACRGMLCGFG
AVCEPNAEGPGRASCVCKKSPCPSVVAPVCGSDASTYSNECELQRAQCSQQRRIRLLSRG
PCGSRDPCSNVTCSFGSTCARSADGLTASCLCPATCRGAPEGTVCGSDGADYPGECQLLR
RACARQENVFKKFDGPCDPCQGALPDPSRSCRVNPRTRRPEMLLRPESCPARQAPVCGDD
GVTYENDCVMGRSGAARGLLLQKVRSGQCQGRDQCPEPCRFNAVCLSRRGRPRCSCDRVT
CDGAYRPVCAQDGRTYDSDCWRQQAECRQQRAIPSKHQGPCDQAPSPCLGVQCAFGATCA
VKNGQAACECLQACSSLYDPVCGSDGVTYGSACELEATACTLGREIQVARKGPCDRCGQC
RFGALCEAETGRCVCPSECVALAQPVCGSDGHTYPSECMLHVHACTHQISLHVASAGPCE
TCGDAVCAFGAVCSAGQCVCPRCEHPPPGPVCGSDGVTYGSACELREAACLQQTQIEEAR
AGPCEQAECGSGGSGSGEDGDCEQELCRQRGGIWDEDSEDGPCVCDFSCQSVPGSPVCGS
DGVTYSTECELKKARCESQRGLYVAAQGACRGPTFAPLPPVAPLHCAQTPYGCCQDNITA
ARGVGLAGCPSACQCNPHGSYGGTCDPATGQCSCRPGVGGLRCDRCEPGFWNFRGIVTDG
RSGCTPCSCDPQGAVRDDCEQMTGLCSCKPGVAGPKCGQCPDGRALGPAGCEADASAPAT
CAEMRCEFGARCVEESGSAHCVCPMLTCPEANATKVCGSDGVTYGNECQLKTIACRQGLQ
ISIQSLGPCQEAVAPSTHPTSASVTVTTPGLLLSQALPAPPGALPLAPSSTAHSQTTPPP
SSRPRTTASVPRTTVWPVLTVPPTAPSPAPSLVASAFGESGSTDGSSDEELSGDQEASGG
GSGGLEPLEGSSVATPGPPVERASCYNSALGCCSDGKTPSLDAEGSNCPATKVFQGVLEL

EGVEGQELFYTPEMADPKSELFGETARSIESTLDDLFRNSDVKKDFRSVRLRDLGPGKSV
RAIVDVHFDPTTAFRAPDVARALLRQIQVSRRRSLGVRRPLQEHVRFMDFDWFPAFITGA
TSGAIAAGATARATTASRLPSSAVTPRAPHPSHTSQPVAKTTAAPTTRRPPTTAPSRVPG
RRPPAPQQPPKPCDSQPCFHGGTCQDWALGGGFTCSCPAGRGGAVCEKVLGAPVPAFEGR
SFLAFPTLRAYHTLRLALEFRALEPQGLLLYNGNARGKDFLALALLDGRVQLRFDTGSGP
AVLTSAVPVEPGQWHRLELSRHWRRGTLSVDGETPVLGESPSGTDGLNLDTDLFVGGVPE
DQAAVALERTFVGAGLRGCIRLLDVNNQRLELGIGPGAATRGSGVGECGDHPCLPNPCHG
GAPCQNLEAGRFHCQCPPGRVGPTCADEKSPCQPNPCHGAAPCRVLPEGGAQCECPLGRE
GTFCQTASGQDGSGPFLADFNGFSHLELRGLHTFARDLGEKMALEVVFLARGPSGLLLYN
GQKTDGKGDFVSLALRDRRLEFRYDLGKGAAVIRSREPVTLGAWTRVSLERNGRKGALRV
GDGPRVLGESPKSRKVPHTVLNLKEPLYVGGAPDFSKLARAAAVSSGFDGAIQLVSLGGR
QLLTPEHVLRQVDVTSFAGHPCTRASGHPCLNGASCVPREAAYVCLCPGGFSGPHCEKGL
VEKSAGDVDTLAFDGRTFVEYLNAVTESELANEIPVPETLDSGALHSEKALQSNHFELSL
RTEATQGLVLWSGKATERADYVALAIVDGHLQLSYNLGSQPVVLRSTVPVNTNRWLRVVA
HREQREGSLQVGNEAPVTGSSPLGATQLDTDGALWLGGLPELPVGPALPKAYGTGFVGCL
RDVVVGRHPLHLLEDAVTKPELRPCPTP
>sp|P14543|NID1_HUMAN Nidogen-1 OS=Homo sapiens OX=9606 GN=NID1 PE=1 SV=3
MLASSSRIRAAWTRALLLPLLLAGPVGCLSRQELFPFGPGQGDLELEDGDDFVSPALELS
GALRFYDRSDIDAVYVTTNGIIATSEPPAKESHPGLFPPTFGAVAPFLADLDTTDGLGKV
YYREDLSPSITQRAAECVHRGFPEISFQPSSAVVVTWESVAPYQGPSRDPDQKGKRNTFQ
AVLASSDSSSYAIFLYPEDGLQFHTTFSKKENNQVPAVVAFSQGSVGFLWKSNGAYNIFA
NDRESVENLAKSSNSGQQGVWVFEIGSPATTNGVVPADVILGTEDGAEYDDEDEDYDLAT
TRLGLEDVGTTPFSYKALRRGGADTYSVPSVLSPRRAATERPLGPPTERTRSFQLAVETF
HQQHPQVIDVDEVEETGVVFSYNTDSRQTCANNRHQCSVHAECRDYATGFCCSCVAGYTG
NGRQCVAEGSPQRVNGKVKGRIFVGSSQVPIVFENTDLHSYVVMNHGRSYTAISTIPETV
GYSLLPLAPVGGIIGWMFAVEQDGFKNGFSITGGEFTRQAEVTFVGHPGNLVIKQRFSGI
DEHGHLTIDTELEGRVPQIPFGSSVHIEPYTELYHYSTSVITSSSTREYTVTEPERDGAS
PSRIYTYQWRQTITFQECVHDDSRPALPSTQQLSVDSVFVLYNQEEKILRYALSNSIGPV
REGSPDALQNPCYIGTHGCDTNAACRPGPRTQFTCECSIGFRGDGRTCYDIDECSEQPSV
CGSHTICNNHPGTFRCECVEGYQFSDEGTCVAVVDQRPINYCETGLHNCDIPQRAQCIYT
GGSSYTCSCLPGFSGDGQACQDVDECQPSRCHPDAFCYNTPGSFTCQCKPGYQGDGFRCV
PGEVEKTRCQHEREHILGAAGATDPQRPIPPGLFVPECDAHGHYAPTQCHGSTGYCWCVD
RDGREVEGTRTRPGMTPPCLSTVAPPIHQGPAVPTAVIPLPPGTHLLFAQTGKIERLPLE
GNTMRKTEAKAFLHVPAKVIIGLAFDCVDKMVYWTDITEPSIGRASLHGGEPTTIIRQDL
GSPEGIAVDHLGRNIFWTDSNLDRIEVAKLDGTQRRVLFETDLVNPRGIVTDSVRGNLYW
TDWNRDNPKIETSYMDGTNRRILVQDDLGLPNGLTFDAFSSQLCWVDAGTNRAECLNPSQ
PSRRKALEGLQYPFAVTSYGKNLYFTDWKMNSVVALDLAISKETDAFQPHKQTRLYGITT
ALSQCPQGHNYCSVNNGGCTHLCLATPGSRTCRCPDNTLGVDCIEQK
>sp|Q8TE56|ATS17_HUMAN A disintegrin and metalloproteinase with thrombospondin motifs 17 OS=Homo sapiens OX=9606 GN=ADAMTS17 PE=2 SV=2
MCDGALLPPLVLPVLLLLVWGLDPGTAVGDAAADVEVVLPWRVRPDDVHLPPLPAAPGPR
RRRRPRTPPAAPRARPGERALLLHLPAFGRDLYLQLRRDLRFLSRGFEVEEAGAARRRGR
PAELCFYSGRVLGHPGSLVSLSACGAAGGLVGLIQLGQEQVLIQPLNNSQGPFSGREHLI
RRKWSLTPSPSAEAQRPEQLCKVLTEKKKPTWGRPSRDWRERRNAIRLTSEHTVETLVVA

DADMVQYHGAEAAQRFILTVMNMVYNMFQHQSLGIKINIQVTKLVLLRQRPAKLSIGHHG
ERSLESFCHWQNEEYGGARYLGNNQVPGGKDDPPLVDAAVFVTRTDFCVHKDEPCDTVGI
AYLGGVCSAKRKCVLAEDNGLNLAFTIAHELGHNLGMNHDDDHSSCAGRSHIMSGEWVKG
RNPSDLSWSSCSRDDLENFLKSKVSTCLLVTDPRSQHTVRLPHKLPGMHYSANEQCQILF
GMNATFCRNMEHLMCAGLWCLVEGDTSCKTKLDPPLDGTECGADKWCRAGECVSKTPIPE
HVDGDWSPWGAWSMCSRTCGTGARFRQRKCDNPPPGPGGTHCPGASVEHAVCENLPCPKG
LPSFRDQQCQAHDRLSPKKKGLLTAVVVDDKPCELYCSPLGKESPLLVADRVLDGTPCGP
YETDLCVHGKCQKIGCDGIIGSAAKEDRCGVCSGDGKTCHLVKGDFSHARGTALKDSGKG
SINSDWKIELPGEFQIAGTTVRYVRRGLWEKISAKGPTKLPLHLMVLLFHDQDYGIHYEY
TVPVNRTAENQSEPEKPQDSLFIWTHSGWEGCSVQCGGGERRTIVSCTRIVNKTTTLVND
SDCPQASRPEPQVRRCNLHPCQSRWVAGPWSPCSATCEKGFQHREVTCVYQLQNGTHVAT
RPLYCPGPRPAAVQSCEGQDCLSIWEASEWSQCSASCGKGVWKRTVACTNSQGKCDASTR
PRAEEACEDYSGCYEWKTGDWSTCSSTCGKGLQSRVVQCMHKVTGRHGSECPALSKPAPY
RQCYQEVCNDRINANTITSPRLAALTYKCTRDQWTVYCRVIREKNLCQDMRWYQRCCQTC
RDFYANKMRQPPPNS
>sp|P00734|THRB_HUMAN Prothrombin OS=Homo sapiens OX=9606 GN=F2 PE=1 SV=2
MAHVRGLQLPGCLALAALCSLVHSQHVFLAPQQARSLLQRVRRANTFLEEVRKGNLEREC
VEETCSYEEAFEALESSTATDVFWAKYTACETARTPRDKLAACLEGNCAEGLGTNYRGHV
NITRSGIECQLWRSRYPHKPEINSTTHPGADLQENFCRNPDSSTTGPWCYTTDPTVRRQE
CSIPVCGQDQVTVAMTPRSEGSSVNLSPPLEQCVPDRGQQYQGRLAVTTHGLPCLAWASA
QAKALSKHQDFNSAVQLVENFCRNPDGDEEGVWCYVAGKPGDFGYCDLNYCEEAVEEETG
DGLDEDSDRAIEGRTATSEYQTFFNPRTFGSGEADCGLRPLFEKKSLEDKTERELLESYI
DGRIVEGSDAEIGMSPWQVMLFRKSPQELLCGASLISDRWVLTAAHCLLYPPWDKNFTEN
DLLVRIGKHSRTRYERNIEKISMLEKIYIHPRYNWRENLDRDIALMKLKKPVAFSDYIHP
VCLPDRETAASLLQAGYKGRVTGWGNLKETWTANVGKGQPSVLQVVNLPIVERPVCKDST
RIRITDNMFCAGYKPDEGKRGDACEGDSGGPFVMKSPFNNRWYQMGIVSWGEGCDRDGKY
GFYTHVFRLKKWIQKVIDQFGE
>sp|P01011|AACT_HUMAN Alpha-1-antichymotrypsin OS=Homo sapiens OX=9606
GN=SERPINA3 PE=1 SV=2
MERMLPLLALGLLAAGFCPAVLCHPNSPLDEENLTQENQDRGTHVDLGLASANVDFAFSL
YKQLVLKAPDKNVIFSPLSISTALAFLSLGAHNTTLTEILKGLKFNLTETSEAEIHQSFQ
HLLRTLNQSSDELQLSMGNAMFVKEQLSLLDRFTEDAKRLYGSEAFATDFQDSAAAKKLI
NDYVKNGTRGKITDLIKDLDSQTMMVLVNYIFFKAKWEMPFDPQDTHQSRFYLSKKKWVM
VPMMSLHHLTIPYFRDEELSCTVVELKYTGNASALFILPDQDKMEEVEAMLLPETLKRWR
DSLEFREIGELYLPKFSISRDYNLNDILLQLGIEEAFTSKADLSGITGARNLAVSQVVHK
AVLDVFEEGTEASAATAVKITLLSALVETRTIVRFNRPFLMIIVPTDTQNIFFMSKVTNP
KQA
>sp|P02747|C1QC_HUMAN Complement C1q subcomponent subunit C OS=Homo sapiens
OX=9606 GN=C1QC PE=1 SV=3
MDVGPSSLPHLGLKLLLLLLLLPLRGQANTGCYGIPGMPGLPGAPGKDGYDGLPGPKGEP
GIPAIPGIRGPKGQKGEPGLPGHPGKNGPMGPPGMPGVPGPMGIPGEPGEEGRYKQKFQS
VFTVTRQTHQPPAPNSLIRFNAVLTNPQGDYDTSTGKFTCKVPGLYYFVYHASHTANLCV
LLYRSGVKVVTFCGHTSKTNQVNSGGVLLRLQVGEEVWLAVNDYYDMVGIQGSDSVFSGF
LLFPD

>sp|P02776|PLF4_HUMAN Platelet factor 4 OS=Homo sapiens OX=9606 GN=PF4 PE=1 SV=2
MSSAAGFCASRPGLLFLGLLLLPLVVAFASAEAEEDGDLQCLCVKTTSQVRPRHITSLEV
IKAGPHCPTAQLIATLKNGRKICLDLQAPLYKKIIKKLLES
>sp|P02790|HEMO_HUMAN Hemopexin OS=Homo sapiens OX=9606 GN=HPX PE=1 SV=2
MARVLGAPVALGLWSLCWSLAIATPLPPTSAHGNVAEGETKPDPDVTERCSDGWSFDATT
LDDNGTMLFFKGEFVWKSHKWDRELISERWKNFPSPVDAAFRQGHNSVFLIKGDKVWVYP
PEKKEKGYPKLLQDEFPGIPSPLDAAVECHRGECQAEGVLFFQGDREWFWDLATGTMKER
SWPAVGNCSSALRWLGRYYCFQGNQFLRFDPVRGEVPPRYPRDVRDYFMPCPGRGHGHRN
GTGHGNSTHHGPEYMRCSPHLVLSALTSDNHGATYAFSGTHYWRLDTSRDGWHSWPIAHQ
WPQGPSAVDAAFSWEEKLYLVQGTQVYVFLTKGGYTLVSGYPKRLEKEVGTPHGIILDSV
DAAFICPGSSRLHIMAGRRLWWLDLKSGAQATWTELPWPHEKVDGALCMEKSLGPNSCSA
NGPGLYLIHGPNLYCYSDVEKLNAAKALPQPQNVTSLLGCTH
>sp|P27216|ANX13_HUMAN Annexin A13 OS=Homo sapiens OX=9606 GN=ANXA13 PE=1 SV=3
MGNRHAKASSPQGFDVDRDAKKLNKACKGMGTNEAAIIEILSGRTSDERQQIKQKYKATY
GKELEEVLKSELSGNFEKTALALLDRPSEYAARQLQKAMKGLGTDESVLIEVLCTRTNKE
IIAIKEAYQRLFDRSLESDVKGDTSGNLKKILVSLLQANRNEGDDVDKDLAGQDAKDLYD
AGEGRWGTDELAFNEVLAKRSYKQLRATFQAYQILIGKDIEEAIEEETSGDLQKAYLTLV
RCAQDCEDYFAERLYKSMKGAGTDEETLIRIVVTRAEVDLQGIKAKFQEKYQKSLSDMVR
SDTSGDFRKLLVALLH
>sp|P31151|S10A7_HUMAN Protein S100-A7 OS=Homo sapiens OX=9606 GN=S100A7 PE=1
SV=4
MSNTQAERSIIGMIDMFHKYTRRDDKIEKPSLLTMMKENFPNFLSACDKKGTNYLADVFE
KKDKNEDKKIDFSEFLSLLGDIATDYHKQSHGAAPCSGGSQ
>sp|P35237|SPB6_HUMAN Serpin B6 OS=Homo sapiens OX=9606 GN=SERPINB6 PE=1 SV=3
MDVLAEANGTFALNLLKTLGKDNSKNVFFSPMSMSCALAMVYMGAKGNTAAQMAQILSFN
KSGGGGDIHQGFQSLLTEVNKTGTQYLLRMANRLFGEKSCDFLSSFRDSCQKFYQAEMEE
LDFISAVEKSRKHINTWVAEKTEGKIAELLSPGSVDPLTRLVLVNAVYFRGNWDEQFDKE
NTEERLFKVSKNEEKPVQMMFKQSTFKKTYIGEIFTQILVLPYVGKELNMIIMLPDETTD
LRTVEKELTYEKFVEWTRLDMMDEEEVEVSLPRFKLEESYDMESVLRNLGMTDAFELGKA
DFSGMSQTDLSLSKVVHKSFVEVNEEGTEAAAATAAIMMMRCARFVPRFCADHPFLFFIQ
HSKTNGILFCGRFSSP
>sp|Q75N90|FBN3_HUMAN Fibrillin-3 OS=Homo sapiens OX=9606 GN=FBN3 PE=2 SV=3
MTLEGLYLARGPLARLLLAWSALLCMAGGQGRWDGALEAAGPGRVRRRGSPGILQGPNVC
GSRFHAYCCPGWRTFPGRSQCVVPICRRACGEGFCSQPNLCTCADGTLAPSCGVSRGSGC
SVSCMNGGTCRGASCLCQKGYTGTVCGQPICDRGCHNGGRCIGPNRCACVYGFMGPQCER
DYRTGPCFGQVGPEGCQHQLTGLVCTKALCCATVGRAWGLPCELCPAQPHPCRRGFIPNI
HTGACQDVDECQAVPGLCQGGSCVNMVGSFHCRCPVGHRLSDSSAACEDYRAGACFSVLF
GGRCAGDLAGHYTRRQCCCDRGRCWAAGPVPELCPPRGSNEFQQLCAQRLPLLPGHPGLF
PGLLGFGSNGMGPPLGPARLNPHGSDARGIPSLGPGNSNIGTATLNQTIDICRHFTNLCL
NGRCLPTPSSYRCECNVGYTQDVRGECIDVDECTSSPCHHGDCVNIPGTYHCRCYPGFQA
TPTRQACVDVDECIVSGGLCHLGRCVNTEGSFQCVCNAGFELSPDGKNCVDHNECATSTM
CVNGVCLNEDGSFSCLCKPGFLLAPGGHYCMDIDECQTPGICVNGHCTNTEGSFRCQCLG
GLAVGTDGRVCVDTHVRSTCYGAIEKGSCARPFPGTVTKSECCCANPDHGFGEPCQLCPA
KDSAEFQALCSSGLGITTDGRDINECALDPEVCANGVCENLRGSYRCVCNLGYEAGASGK

DCTDVDECALNSLLCDNGWCQNSPGSYSCSCPPGFHFWQDTEICKDVDECLSSPCVSGVC
RNLAGSYTCKCGPGSRLDPSGTFCLDSTKGTCWLKIQESRCEVNLQGASLRSECCATLGA
AWGSPCERCEIDPACARGFARMTGVTCDDVNECESFPGVCPNGRCVNTAGSFRCECPEGL
MLDASGRLCVDVRLEPCFLRWDEDECGVTLPGKYRMDVCCCSIGAVWGVECEACPDPESL
EFASLCPRGLGFASRDFLSGRPFYKDVNECKVFPGLCTHGTCRNTVGSFHCACAGGFALD
AQERNCTDIDECRISPDLCGQGTCVNTPGSFECECFPGYESGFMLMKNCMDVDECARDPL
LCRGGTCTNTDGSYKCQCPPGHELTAKGTACEDIDECSLSDGLCPHGQCVNVIGAFQCSC
HAGFQSTPDRQGCVDINECRVQNGGCDVHCINTEGSYRCSCGQGYSLMPDGRACADVDEC
EENPRVCDQGHCTNMPGGHRCLCYDGFMATPDMRTCVDVDECDLNPHICLHGDCENTKGS
FVCHCQLGYMVRKGATGCSDVDECEVGGHNCDSHASCLNIPGSFSCRCLPGWVGDGFECH
DLDECVSQEHRCSPRGDCLNVPGSYRCTCRQGFAGDGFFCEDRDECAENVDLCDNGQCLN
APGGYRCECEMGFDPTEDHRACQDVDECAQGNLCAFGSCENLPGMFRCICNGGYELDRGG
GNCTDINECADPVNCINGVCINTPGSYLCSCPQDFELNPSGVGCVDTRAGNCFLETHDRG
DSGISCSAEIGVGVTRASCCCSLGRAWGNPCELCPMANTTEYRTLCPGGEGFQPNRITVI
LEDIDECQELPGLCQGGDCVNTFGSFQCECPPGYHLSEHTRICEDIDECSTHSGICGPGT
CYNTLGNYTCVCPAEYLQVNGGNNCMDMRKSVCFRHYNGTCQNELAFNVTRKMCCCSYNI
GQAWNRPCEACPTPISPDYQILCGNQAPGFLTDIHTGKPLDIDECGEIPAICANGICINQ
IGSFRCECPAGFNYNSILLACEDVDECGSRESPCQQNADCINIPGSYRCKCTRGYKLSPG
GACVGRNECREIPNVCSHGDCMDTEGSYMCLCHRGFQASADQTLCMDIDECDRQPCGNGT
CKNIIGSYNCLCFPGFVVTHNGDCVDFDECTTLVGQVCRFGHCLNTAGSFHCLCQDGFEL
TADGKNCVDTNECLSLAGTCLPGTCQNLEGSFRCICPPGFQVQSDHCIDIDECSEEPNLC
LFGTCTNSPGSFQCLCPPGFVLSDNGHRCFDTRQSFCFTRFEAGKCSVPKAFNTTKTRCC
CSKRPGEGWGDPCELCPQEGSAAFQELCPFGHGAVPGPDDSREDVNECAENPGVCTNGVC
VNTDGSFRCECPFGYSLDFTGINCVDTDECSVGHPCGQGTCTNVIGGFECACADGFEPGL
MMTCEDIDECSLNPLLCAFRCHNTEGSYLCTCPAGYTLREDGAMCRDVDECADGQQDCHA
RGMECKNLIGTFACVCPPGMRPLPGSGEGCTDDNECHAQPDLCVNGRCVNTAGSFRCDCD
EGFQPSPTLTECHDIRQGPCFAEVLQTMCRSLSSSSEAVTRAECCCGGGRGWGPRCELCP
LPGTSAYRKLCPHGSGYTAEGRDVDECRMLAHLCAHGECINSLGSFRCHCQAGYTPDATA
TTCLDMDECSQVPKPCTFLCKNTKGSFLCSCPRGYLLEEDGRTCKDLDECTSRQHNCQFL
CVNTVGAFTCRCPPGFTQHHQACFDNDECSAQPGPCGAHGHCHNTPGSFRCECHQGFTLV
SSGHGCEDVNECDGPHRCQHGCQNQLGGYRCSCPQGFTQHSQWAQCVDENECALSPPTCG
SASCRNTLGGFRCVCPSGFDFDQALGGCQEVDECAGRRGPCSYSCANTPGGFLCGCPQGY
FRAGQGHCVSGLGFSPGPQDTPDKEELLSSEACYECKINGLSPRDRPRRSAHRDHQVNLA
TLDSEALLTLGLNLSHLGRAERILELRPALEGLEGRIRYVIVRGNEQGFFRMHHLRGVSS
LQLGRRRPGPGTYRLEVVSHMAGPWGVQPEGQPGPWGQALRLKVQLQLL
>sp|A0M8Q6|IGLC7_HUMAN Immunoglobulin lambda constant 7 OS=Homo sapiens OX=9606 GN=IGLC7 PE=1 SV=3
GQPKAAPSVTLFPPSSEELQANKATLVCLVSDFNPGAVTVAWKADGSPVKVGVETTKPSK
QSNNKYAASSYLSLTPEQWKSHRSYSCRVTHEGSTVEKTVAPAECS
>sp|O14498|ISLR_HUMAN Immunoglobulin superfamily containing leucine-rich repeat protein OS=Homo sapiens OX=9606 GN=ISLR PE=2 SV=1
MQELHLLWWALLLGLAQACPEPCDCGEKYGFQIADCAYRDLESVPPGFPANVTTLSLSAN
RLPGLPEGAFREVPLLQSLWLAHNEIRTVAAGALASLSHLKSLDLSHNLISDFAWSDLHN
LSALQLLKMDSNELTFIPRDAFRSLRALRSLQLNHNRLHTLAEGTFTPLTALSHLQINEN

PFDCTCGIVWLKTWALTTAVSIPEQDNIACTSPHVLKGTPLSRLPPLPCSAPSVQLSYQP
SQDGAELRPGFVLALHCDVDGQPAPQLHWHIQIPSGIVEITSPNVGTDGRALPGTPVASS
QPRFQAFANGSLLIPDFGKLEEGTYSCLATNELGSAESSVDVALATPGEGGEDTLGRRFH
GKAVEGKGCYTVDNEVQPSGPEDNVVIIYLSRAGNPEAAVAEGVPGQLPPGLLLLGQSLL
LFFFLTSF
>sp|O15230|LAMA5_HUMAN Laminin subunit alpha-5 OS=Homo sapiens OX=9606 GN=LAMA5 PE=1 SV=8
MAKRLCAGSALCVRGPRGPAPLLLVGLALLGAARAREEAGGGFSLHPPYFNLAEGARIAA
SATCGEEAPARGSPRPTEDLYCKLVGGPVAGGDPNQTIRGQYCDICTAANSNKAHPASNA
IDGTERWWQSPPLSRGLEYNEVNVTLDLGQVFHVAYVLIKFANSPRPDLWVLERSMDFGR
TYQPWQFFASSKRDCLERFGPQTLERITRDDAAICTTEYSRIVPLENGEIVVSLVNGRPG
AMNFSYSPLLREFTKATNVRLRFLRTNTLLGHLMGKALRDPTVTRRYYYSIKDISIGGRC
VCHGHADACDAKDPTDPFRLQCTCQHNTCGGTCDRCCPGFNQQPWKPATANSANECQSCN
CYGHATDCYYDPEVDRRRASQSLDGTYQGGGVCIDCQHHTTGVNCERCLPGFYRSPNHPL
DSPHVCRRCNCESDFTDGTCEDLTGRCYCRPNFSGERCDVCAEGFTGFPSCYPTPSSSND
TREQVLPAGQIVNCDCSAAGTQGNACRKDPRVGRCLCKPNFQGTHCELCAPGFYGPGCQP
CQCSSPGVADDRCDPDTGQCRCRVGFEGATCDRCAPGYFHFPLCQLCGCSPAGTLPEGCD
EAGRCLCQPEFAGPHCDRCRPGYHGFPNCQACTCDPRGALDQLCGAGGLCRCRPGYTGTA
CQECSPGFHGFPSCVPCHCSAEGSLHAACDPRSGQCSCRPRVTGLRCDTCVPGAYNFPYC
EAGSCHPAGLAPVDPALPEAQVPCMCRAHVEGPSCDRCKPGFWGLSPSNPEGCTRCSCDL
RGTLGGVAECQPGTGQCFCKPHVCGQACASCKDGFFGLDQADYFGCRSCRCDIGGALGQS
CEPRTGVCRCRPNTQGPTCSEPARDHYLPDLHHLRLELEEAATPEGHAVRFGFNPLEFEN
FSWRGYAQMAPVQPRIVARLNLTSPDLFWLVFRYVNRGAMSVSGRVSVREEGRSATCANC
TAQSQPVAFPPSTEPAFITVPQRGFGEPFVLNPGTWALRVEAEGVLLDYVVLLPSAYYEA
ALLQLRVTEACTYRPSAQQSGDNCLLYTHLPLDGFPSAAGLEALCRQDNSLPRPCPTEQL
SPSHPPLITCTGSDVDVQLQVAVPQPGRYALVVEYANEDARQEVGVAVHTPQRAPQQGLL
SLHPCLYSTLCRGTARDTQDHLAVFHLDSEASVRLTAEQARFFLHGVTLVPIEEFSPEFV
EPRVSCISSHGAFGPNSAACLPSRFPKPPQPIILRDCQVIPLPPGLPLTHAQDLTPAMSP
AGPRPRPPTAVDPDAEPTLLREPQATVVFTTHVPTLGRYAFLLHGYQPAHPTFPVEVLIN
AGRVWQGHANASFCPHGYGCRTLVVCEGQALLDVTHSELTVTVRVPKGRWLWLDYVLVVP
ENVYSFGYLREEPLDKSYDFISHCAAQGYHISPSSSSLFCRNAAASLSLFYNNGARPCGC
HEVGATGPTCEPFGGQCPCHAHVIGRDCSRCATGYWGFPNCRPCDCGARLCDELTGQCIC
PPRTIPPDCLLCQPQTFGCHPLVGCEECNCSGPGIQELTDPTCDTDSGQCKCRPNVTGRR
CDTCSPGFHGYPRCRPCDCHEAGTAPGVCDPLTGQCYCKENVQGPKCDQCSLGTFSLDAA
NPKGCTRCFCFGATERCRSSSYTRQEFVDMEGWVLLSTDRQVVPHERQPGTEMLRADLRH
VPEAVPEAFPELYWQAPPSYLGDRVSSYGGTLRYELHSETQRGDVFVPMESRPDVVLQGN
QMSITFLEPAYPTPGHVHRGQLQLVEGNFRHTETRNTVSREELMMVLASLEQLQIRALFS
QISSAVFLRRVALEVASPAGQGALASNVELCLCPASYRGDSCQECAPGFYRDVKGLFLGR
CVPCQCHGHSDRCLPGSGVCVDCQHNTEGAHCERCQAGFVSSRDDPSAPCVSCPCPLSVP
SNNFAEGCVLRGGRTQCLCKPGYAGASCERCAPGFFGNPLVLGSSCQPCDCSGNGDPNLL
FSDCDPLTGACRGCLRHTTGPRCEICAPGFYGNALLPGNCTRCDCTPCGTEACDPHSGHC
LCKAGVTGRRCDRCQEGHFGFDGCGGCRPCACGPAAEGSECHPQSGQCHCRPGTMGPQCR
ECAPGYWGLPEQGCRRCQCPGGRCDPHTGRCNCPPGLSGERCDTCSQQHQVPVPGGPVGH
SIHCEVCDHCVVLLLDDLERAGALLPAIHEQLRGINASSMAWARLHRLNASIADLQSQLR

SPLGPRHETAQQLEVLEQQSTSLGQDARRLGGQAVGTRDQASQLLAGTEATLGHAKTLLA
AIRAVDRTLSELMSQTGHLGLANASAPSGEQLLRTLAEVERLLWEMRARDLGAPQAAAEA
ELAAAQRLLARVQEQLSSLWEENQALATQTRDRLAQHEAGLMDLREALNRAVDATREAQE
LNSRNQERLEEALQRKQELSRDNATLQATLHAARDTLASVFRLLHSLDQAKEELERLAAS
LDGARTPLLQRMQTFSPAGSKLRLVEAAEAHAQQLGQLALNLSSIILDVNQDRLTQRAIE
ASNAYSRILQAVQAAEDAAGQALQQADHTWATVVRQGLVDRAQQLLANSTALEEAMLQEQ
QRLGLVWAALQGARTQLRDVRAKKDQLEAHIQAAQAMLAMDTDETSKKIAHAKAVAAEAQ
DTATRVQSQLQAMQENVERWQGQYEGLRGQDLGQAVLDAGHSVSTLEKTLPQLLAKLSIL
ENRGVHNASLALSASIGRVRELIAQARGAASKVKVPMKFNGRSGVQLRTPRDLADLAAYT
ALKFYLQGPEPEPGQGTEDRFVMYMGSRQATGDYMGVSLRDKKVHWVYQLGEAGPAVLSI
DEDIGEQFAAVSLDRTLQFGHMSVTVERQMIQETKGDTVAPGAEGLLNLRPDDFVFYVGG
YPSTFTPPPLLRFPGYRGCIEMDTLNEEVVSLYNFERTFQLDTAVDRPCARSKSTGDPWL
TDGSYLDGTGFARISFDSQISTTKRFEQELRLVSYSGVLFFLKQQSQFLCLAVQEGSLVL
LYDFGAGLKKAVPLQPPPPLTSASKAIQVFLLGGSRKRVLVRVERATVYSVEQDNDLELA
DAYYLGGVPPDQLPPSLRRLFPTGGSVRGCVKGIKALGKYVDLKRLNTTGVSAGCTADLL
VGRAMTFHGHGFLRLALSNVAPLTGNVYSGFGFHSAQDSALLYYRASPDGLCQVSLQQGR
VSLQLLRTEVKTQAGFADGAPHYVAFYSNATGVWLYVDDQLQQMKPHRGPPPELQPQPEG
PPRLLLGGLPESGTIYNFSGCISNVFVQRLLGPQRVFDLQQNLGSVNVSTGCAPALQAQT
PGLGPRGLQATARKASRRSRQPARHPACMLPPHLRTTRDSYQFGGSLSSHLEFVGILARH
RNWPSLSMHVLPRSSRGLLLFTARLRPGSPSLALFLSNGHFVAQMEGLGTRLRAQSRQRS
RPGRWHKVSVRWEKNRILLVTDGARAWSQEGPHRQHQGAEHPQPHTLFVGGLPASSHSSK
LPVTVGFSGCVKRLRLHGRPLGAPTRMAGVTPCILGPLEAGLFFPGSGGVITLDLPGATL
PDVGLELEVRPLAVTGLIFHLGQARTPPYLQLQVTEKQVLLRADDGAGEFSTSVTRPSVL
CDGQWHRLAVMKSGNVLRLEVDAQSNHTVGPLLAAAAGAPAPLYLGGLPEPMAVQPWPPA
YCGCMRRLAVNRSPVAMTRSVEVHGAVGASGCPAA
>sp|O15417|TNC18_HUMAN Trinucleotide repeat-containing gene 18 protein OS=Homo sapiens OX=9606 GN=TNRC18 PE=1 SV=3
MDGRDFGPQRSVHGPPPPLLSGLAMDSHRVGAATAGRLPASGLPGPLPPGKYMAGLNLHP
HPGEAFLGSFVASGMGPSASSHGSPVPLPSDLSFRSPTPSNLPMVQLWAAHAHEGFSHLP
SGLYPSYLHLNHLEPPSSGSPLLSQLGQPSIFDTQKGQGPGGDGFYLPTAGAPGSLHSHA
PSARTPGGGHSSGAPAKGSSSRDGPAKERAGRGGEPPPLFGKKDPRARGEEASGPRGVVD
LTQEARAEGRQDRGPPRLAERLSPFLAESKTKNAALQPSVLTMCNGGAGDVGLPALVAEA
GRGGAKEAARQDEGARLLRRTETLLPGPRPCPSPLPPPPAPPKGPPAPPAATPAGVYTVF
REQGREHRVVAPTFVPSVEAFDERPGPIQIASQARDARAREREAGRPGVLQAPPGSPRPL
DRPEGLREKNSVIRSLKRPPPADAPTVRATRASPDPRAYVPAKELLKPEADPRPCERAPR
GPAGPAAQQAAKLFGLEPGRPPPTGPEHKWKPFELGNFAATQMAVLAAQHHHSRAEEEAA
VVAASSSKKAYLDPGAVLPRSAATCGRPVADMHSAAHGSGEASAMQSLIKYSGSFARDAV
AVRPGGCGKKSPFGGLGTMKPEPAPTSAGASRAQARLPHSGGPAAGGGRQLKRDPERPES
AKAFGREGSGAQGEAEVRHPPVGIAVAVARQKDSGGSGRLGPGLVDQERSLSLSNVKGHG
RADEDCVDDRARHREERLLGARLDRDQEKLLRESKELADLARLHPTSCAPNGLNPNLMVT
GGPALAGSGRWSADPAAHLATHPWLPRSGNASMWLAGHPYGLGPPSLHQGMAPAFPPGLG
GSLPSAYQFVRDPQSGQLVVIPSDHLPHFAELMERATVPPLWPALYPPGRSPLHHAQQLQ
LFSQQHFLRQQEFLYLQQQAAQALELQRSAQLVQERLKAQEHRAEMEEKGSKRGLEAAGK
AGLATAGPGLLPRKPPGLAAGPAGTYGKAVSPPPSPRASPVAALKAKVIQKLEDVSKPPA

YAYPATPSSHPTSPPPASPPPTPGITRKEEAPENVVEKKDLELEKEAPSPFQALFSDIPP
RYPFQALPPHYGRPYPFLLQPTAAADADGLAPDVPLPADGPERLALSPEDKPIRLSPSKI
TEPLREGPEEEPLAEREVKAEVEDMDEGPTELPPLESPLPLPAAEAMATPSPAGGCGGGL
LEAQALSATGQSCAEPSECPDFVEGPEPRVDSPGRTEPCTAALDLGVQLTPETLVEAKEE
PVEVPVAVPVVEAVPEEGLAQVAPSESQPTLEMSDCDVPAGEGQCPSLEPQEAVPVLGST
CFLEEASSDQFLPSLEDPLAGMNALAAAAELPQARPLPSPGAAGAQALEKLEAAESLVLE
QSFLHGITLLSEIAELELERRSQEMGGAERALVARPSLESLLAAGSHMLREVLDGPVVDP
LKNLRLPRELKPNKKYSWMRKKEERMYAMKSSLEDMDALELDFRMRLAEVQRQYKEKQRE
LVKLQRRRDSEDRREEPHRSLARRGPGRPRKRTHAPSALSPPRKRGKSGHSSGKLSSKSL
LTSDDYELGAGIRKRHKGSEEEHDALIGMGKARGRNQTWDEHEASSDFISQLKIKKKKMA
SDQEQLASKLDKALSLTKQDKLKSPFKFSDSAGGKSKTSGGCGRYLTPYDSLLGKNRKAL
AKGLGLSLKSSREGKHKRAAKTRKMEVGFKARGQPKSAHSPFASEVSSYSYNTDSEEDEE
FLKDEWPAQGPSSSKLTPSLLCSMVAKNSKAAGGPKLTKRGLAAPRTLKPKPATSRKQPF
CLLLREAEARSSFSDSSEESFDQDESSEEEDEEEELEEEDEASGGGYRLGARERALSPGL
EESGLGLLARFAASALPSPTVGPSLSVVQLEAKQKARKKEERQSLLGTEFEYTDSESEVK
VRKRSPAGLLRPKKGLGEPGPSLAAPTPGARGPDPSSPDKAKLAVEKGRKARKLRGPKEP
GFEAGPEASDDDLWTRRRSERIFLHDASAAAPAPVSTAPATKTSRCAKGGPLSPRKDAGR
AKDRKDPRKKKKGKEAGPGAGLPPPRAPALPSEARAPHASSLTAAKRSKAKAKGKEVKKE
NRGKGGAVSKLMESMAAEEDFEPNQDSSFSEDEHLPRGGAVERPLTPAPRSCIIDKDELK
DGLRVLIPMDDKLLYAGHVQTVHSPDIYRVVVEGERGNRPHIYCLEQLLQEAIIDVRPAS
TRFLPQGTRIAAYWSQQYRCLYPGTVVRGLLDLEDDGDLITVEFDDGDTGRIPLSHIRLL
PPDYKIQCAEPSPALLVPSAKRRSRKTSKDTGEGKDGGTAGSEEPGAKARGRGRKPSAKA
KGDRAATLEEGNPTDEVPSTPLALEPSSTPGSKKSPPEPVDKRAKAPKARPAPPQPSPAP
PAFTSCPAPEPFAELPAPATSLAPAPLITMPATRPKPKKARAAEESGAKGPRRPGEEAEL
LVKLDHEGVTSPKSKKAKEALLLREDPGAGGWQEPKSLLSLGSYPPAAGSSEPKAPWPKA
TDGDLAQEPGPGLTFEDSGNPKSPDKAQAEQDGAEESESSSSSSSGSSSSSSSSSSSGSE
TEGEEEGDKNGDGGCGTGGRNCSAASSRAASPASSSSSSSSSSSSSSSSSSSSSSSSSSS
SSSSSSSSSSSSSSSSSSSSSSSSSSSSSSSTTDEDSSCSSDDEAAPAPTAGPSAQAALP
TKATKQAGKARPSAHSPGKKTPAPQPQAPPPQPTQPLQPKAQAGAKSRPKKREGVHLPTT
KELAKRQRLPSVENRPKIAAFLPARQLWKWFGKPTQRRGMKGKARKLFYKAIVRGKEMIR
IGDCAVFLSAGRPNLPYIGRIQSMWESWGNNMVVRVKWFYHPEETSPGKQFHQGQHWDQK
SSRSLPAALRVSSQRKDFMERALYQSSHVDENDVQTVSHKCLVVGLEQYEQMLKTKKYQD
SEGLYYLAGTYEPTTGMIFSTDGVPVLC
>sp|O15537|XLRS1_HUMAN Retinoschisin OS=Homo sapiens OX=9606 GN=RS1 PE=1 SV=2
MSRKIEGFLLLLLFGYEATLGLSSTEDEGEDPWYQKACKCDCQGGPNALWSAGATSLDCI
PECPYHKPLGFESGEVTPDQITCSNPEQYVGWYSSWTANKARLNSQGFGCAWLSKFQDSS
QWLQIDLKEIKVISGILTQGRCDIDEWMTKYSVQYRTDERLNWIYYKDQTGNNRVFYGNS
DRTSTVQNLLRPPIISRFIRLIPLGWHVRIAIRMELLECVSKCA
>sp|O60814|H2B1K_HUMAN Histone H2B type 1-K OS=Homo sapiens OX=9606 GN=H2BC12 PE=1 SV=3
MPEPAKSAPAPKKGSKKAVTKAQKKDGKKRKRSRKESYSVYVYKVLKQVHPDTGISSKAM
GIMNSFVNDIFERIAGEASRLAHYNKRSTITSREIQTAVRLLLPGELAKHAVSEGTKAVT
KYTSAK

>sp|O75340|PDCD6_HUMAN Programmed cell death protein 6 OS=Homo sapiens OX=9606 GN=PDCD6 PE=1 SV=1
MAAYSYRPGPGAGPGPAAGAALPDQSFLWNVFQRVDKDRSGVISDTELQQALSNGTWTPF
NPVTVRSIISMFDRENKAGVNFSEFTGVWKYITDWQNVFRTYDRDNSGMIDKNELKQALS
GFGYRLSDQFHDILIRKFDRQGRGQIAFDDFIQGCIVLQRLTDIFRRYDTDQDGWIQVSY
EQYLSMVFSIV
>sp|O75828|CBR3_HUMAN Carbonyl reductase [NADPH] 3 OS=Homo sapiens OX=9606 GN=CBR3 PE=1 SV=3
MSSCSRVALVTGANRGIGLAIARELCRQFSGDVVLTARDVARGQAAVQQLQAEGLSPRFH
QLDIDDLQSIRALRDFLRKEYGGLNVLVNNAAVAFKSDDPMPFDIKAEMTLKTNFFATRN
MCNELLPIMKPHGRVVNISSLQCLRAFENCSEDLQERFHSETLTEGDLVDLMKKFVEDTK
NEVHEREGWPNSPYGVSKLGVTVLSRILARRLDEKRKADRILVNACCPGPVKTDMDGKDS
IRTVEEGAETPVYLALLPPDATEPQGQLVHDKVVQNW
>sp|O75829|CNMD_HUMAN Leukocyte cell-derived chemotaxin 1 OS=Homo sapiens OX=9606 GN=CNMD PE=1 SV=1
MTENSDKVPIALVGPDDVEFCSPPAYATLTVKPSSPARLLKVGAVVLISGAVLLLFGAIG
AFYFWKGSDSHIYNVHYTMSINGKLQDGSMEIDAGNNLETFKMGSGAEEAIAVNDFQNGI
TGIRFAGGEKCYIKAQVKARIPEVGAVTKQSISSKLEGKIMPVKYEENSLIWVAVDQPVK
DNSFLSSKVLELCGDLPIFWLKPTYPKEIQRERREVVRKIVPTTTKRPHSGPRSNPGAGR
LNNETRPSVQEDSQAFNPDNPYHQQEGESMTFDPRLDHEGICCIECRRSYTHCQKICEPL
GGYYPWPYNYQGCRSACRVIMPCSWWVARILGMV
>sp|O95428|PPN_HUMAN Papilin OS=Homo sapiens OX=9606 GN=PAPLN PE=2 SV=4
MRLLLLVPLLLAPAPGSSAPKVRRQSDTWGPWSQWSPCSRTCGGGVSFRERPCYSQRRDG
GSSCVGPARSHRSCRTESCPDGARDFRAEQCAEFDGAEFQGRRYRWLPYYSAPNKCELNC
IPKGENFYYKHREAVVDGTPCEPGKRDVCVDGSCRVVGCDHELDSSKQEDKCLRCGGDGT
TCYPVAGTFDANDLSRGYNQILIVPMGATSILIDEAAASRNFLAVKNVRGEYYLNGHWTI
EAARALPAASTILHYERGAEGDLAPERLHARGPTSEPLVIELISQEPNPGVHYEYHLPLR
RPSPGFSWSHGSWSDCSAECGGGHQSRLVFCTIDHEAYPDHMCQRQPRPADRRSCNLHPC
PETKRWKAGPWAPCSASCGGGSQSRSVYCISSDGAGIQEAVEEAECAGLPGKPPAIQACN
LQRCAAWSPEPWGECSVSCGVGVRKRSVTCRGERGSLLHTAACSLEDRPPLTEPCVHEDC
PLLSDQAWHVGTWGLCSKSCSSGTRRRQVICAIGPPSHCGSLQHSKPVDVEPCNTQPCHL
PQEVPSMQDVHTPASNPWMPLGPQESPASDSRGQWWAAQEHPSARGDHRGERGDPRGDQG
THLSALGPAPSLQQPPYQQPLRSGSGPHDCRHSPHGCCPDGHTASLGPQWQGCPGAPCQQ
SRYGCCPDRVSVAEGPHHAGCTKSYGGDSTGGMPRSRAVASTVHNTHQPQAQQNEPSECR
GSQFGCCYDNVATAAGPLGEGCVGQPSHAYPVRCLLPSAHGSCADWAARWYFVASVGQCN
RFWYGGCHGNANNFASEQECMSSCQGSLHGPRRPQPGASGRSTHTDGGGSSPAGEQEPSQ
HRTGAAVQRKPWPSGGLWRQDQQPGPGEAPHTQAFGEWPWGQELGSRAPGLGGDAGSPAP
PFHSSSYRISLAGVEPSLVQAALGQLVRLSCSDDTAPESQAAWQKDGQPISSDRHRLQFD
GSLIIHPLQAEDAGTYSCGSTRPGRDSQKIQLRIIGGDMAVLSEAELSRFPQPRDPAQDF
GQAGAAGPLGAIPSSHPQPANRLRLDQNQPRVVDASPGQRIRMTCRAEGFPPPAIEWQRD
GQPVSSPRHQLQPDGSLVISRVAVEDGGFYTCVAFNGQDRDQRWVQLRVLGELTISGLPP
TVTVPEGDTARLLCVVAGESVNIRWSRNGLPVQADGHRVHQSPDGTLLIYNLRARDEGSY
TCSAYQGSQAVSRSTEVKVVSPAPTAQPRDPGRDCVDQPELANCDLILQAQLCGNEYYSS
FCCASCSRFQPHAQPIWQ

>sp|O95678|K2C75_HUMAN Keratin, type II cytoskeletal 75 OS=Homo sapiens OX=9606 GN=KRT75 PE=1 SV=2
MSRQSSITFQSGSRRGFSTTSAITPAAGRSRFSSVSVARSAAGSGGLGRISSAGASFGSR
SLYNLGGAKRVSINGCGSSCRSGFGGRASNRFGVNSGFGYGGGVGGGFSGPSFPVCPPGG
IQEVTVNQSLLTPLHLQIDPTIQRVRAEEREQIKTLNNKFASFIDKVRFLEQQNKVLETK
WALLQEQGSRTVRQNLEPLFDSYTSELRRQLESITTERGRLEAELRNMQDVVEDFKVRYE
DEINKRTAAENEFVALKKDVDAAYMNKVELEAKVKSLPEEINFIHSVFDAELSQLQTQVG
DTSVVLSMDNNRNLDLDSIIAEVKAQYEDIANRSRAEAESWYQTKYEELQVTAGRHGDDL
RNTKQEISEMNRMIQRLRAEIDSVKKQCSSLQTAIADAEQRGELALKDARAKLVDLEEAL
QKAKQDMARLLREYQELMNIKLALDVEIATYRKLLEGEECRLSGEGVSPVNISVVTSTLS
SGYGSGSSIGGGNLGLGGGSGYSFTTSGGHSLGAGLGGSGFSATSNRGLGGSGSSVKFVS
TTSSSQKSYTH
>sp|P00352|AL1A1_HUMAN Aldehyde dehydrogenase 1A1 OS=Homo sapiens OX=9606 GN=ALDH1A1 PE=1 SV=2
MSSSGTPDLPVLLTDLKIQYTKIFINNEWHDSVSGKKFPVFNPATEEELCQVEEGDKEDV
DKAVKAARQAFQIGSPWRTMDASERGRLLYKLADLIERDRLLLATMESMNGGKLYSNAYL
NDLAGCIKTLRYCAGWADKIQGRTIPIDGNFFTYTRHEPIGVCGQIIPWNFPLVMLIWKI
GPALSCGNTVVVKPAEQTPLTALHVASLIKEAGFPPGVVNIVPGYGPTAGAAISSHMDID
KVAFTGSTEVGKLIKEAAGKSNLKRVTLELGGKSPCIVLADADLDNAVEFAHHGVFYHQG
QCCIAASRIFVEESIYDEFVRRSVERAKKYILGNPLTPGVTQGPQIDKEQYDKILDLIES
GKKEGAKLECGGGPWGNKGYFVQPTVFSNVTDEMRIAKEEIFGPVQQIMKFKSLDDVIKR
ANNTFYGLSAGVFTKDIDKAITISSALQAGTVWVNCYGVVSAQCPFGGFKMSGNGRELGE
YGFHEYTEVKTVTVKISQKNS
>sp|P00403|COX2_HUMAN Cytochrome c oxidase subunit 2 OS=Homo sapiens OX=9606 GN=MT-CO2 PE=1 SV=1
MAHAAQVGLQDATSPIMEELITFHDHALMIIFLICFLVLYALFLTLTTKLTNTNISDAQE
METVWTILPAIILVLIALPSLRILYMTDEVNDPSLTIKSIGHQWYWTYEYTDYGGLIFNS
YMLPPLFLEPGDLRLLDVDNRVVLPIEAPIRMMITSQDVLHSWAVPTLGLKTDAIPGRLN
QTTFTATRPGVYYGQCSEICGANHSFMPIVLELIPLKIFEMGPVFTL
>sp|P00450|CERU_HUMAN Ceruloplasmin OS=Homo sapiens OX=9606 GN=CP PE=1 SV=2
MKILILGIFLFLCSTPAWAKEKHYYIGIIETTWDYASDHGEKKLISVDTEHSNIYLQNGP
DRIGRLYKKALYLQYTDETFRTTIEKPVWLGFLGPIIKAETGDKVYVHLKNLASRPYTFH
SHGITYYKEHEGAIYPDNTTDFQRADDKVYPGEQYTYMLLATEEQSPGEGDGNCVTRIYH
SHIDAPKDIASGLIGPLIICKKDSLDKEKEKHIDREFVVMFSVVDENFSWYLEDNIKTYC
SEPEKVDKDNEDFQESNRMYSVNGYTFGSLPGLSMCAEDRVKWYLFGMGNEVDVHAAFFH
GQALTNKNYRIDTINLFPATLFDAYMVAQNPGEWMLSCQNLNHLKAGLQAFFQVQECNKS
SSKDNIRGKHVRHYYIAAEEIIWNYAPSGIDIFTKENLTAPGSDSAVFFEQGTTRIGGSY
KKLVYREYTDASFTNRKERGPEEEHLGILGPVIWAEVGDTIRVTFHNKGAYPLSIEPIGV
RFNKNNEGTYYSPNYNPQSRSVPPSASHVAPTETFTYEWTVPKEVGPTNADPVCLAKMYY
SAVEPTKDIFTGLIGPMKICKKGSLHANGRQKDVDKEFYLFPTVFDENESLLLEDNIRMF
TTAPDQVDKEDEDFQESNKMHSMNGFMYGNQPGLTMCKGDSVVWYLFSAGNEADVHGIYF
SGNTYLWRGERRDTANLFPQTSLTLHMWPDTEGTFNVECLTTDHYTGGMKQKYTVNQCRR
QSEDSTFYLGERTYYIAAVEVEWDYSPQREWEKELHHLQEQNVSNAFLDKGEFYIGSKYK
KVVYRQYTDSTFRVPVERKAEEEHLGILGPQLHADVGDKVKIIFKNMATRPYSIHAHGVQ

TESSTVTPTLPGETLTYVWKIPERSGAGTEDSACIPWAYYSTVDQVKDLYSGLIGPLIVC
RRPYLKVFNPRRKLEFALLFLVFDENESWYLDDNIKTYSDHPEKVNKDDEEFIESNKMHA
INGRMFGNLQGLTMHVGDEVNWYLMGMGNEIDLHTVHFHGHSFQYKHRGVYSSDVFDIFP
GTYQTLEMFPRTPGIWLLHCHVTDHIHAGMETTYTVLQNEDTKSG
>sp|P00505|AATM_HUMAN Aspartate aminotransferase, mitochondrial OS=Homo sapiens OX=9606 GN=GOT2 PE=1 SV=3
MALLHSGRVLPGIAAAFHPGLAAAASARASSWWTHVEMGPPDPILGVTEAFKRDTNSKKM
NLGVGAYRDDNGKPYVLPSVRKAEAQIAAKNLDKEYLPIGGLAEFCKASAELALGENSEV
LKSGRFVTVQTISGTGALRIGASFLQRFFKFSRDVFLPKPTWGNHTPIFRDAGMQLQGYR
YYDPKTCGFDFTGAVEDISKIPEQSVLLLHACAHNPTGVDPRPEQWKEIATVVKKRNLFA
FFDMAYQGFASGDGDKDAWAVRHFIEQGINVCLCQSYAKNMGLYGERVGAFTMVCKDADE
AKRVESQLKILIRPMYSNPPLNGARIAAAILNTPDLRKQWLQEVKVMADRIIGMRTQLVS
NLKKEGSTHNWQHITDQIGMFCFTGLKPEQVERLIKEFSIYMTKDGRISVAGVTSSNVGY
LAHAIHQVTK
>sp|P00558|PGK1_HUMAN Phosphoglycerate kinase 1 OS=Homo sapiens OX=9606 GN=PGK1 PE=1 SV=3
MSLSNKLTLDKLDVKGKRVVMRVDFNVPMKNNQITNNQRIKAAVPSIKFCLDNGAKSVVL
MSHLGRPDGVPMPDKYSLEPVAVELKSLLGKDVLFLKDCVGPEVEKACANPAAGSVILLE
NLRFHVEEEGKGKDASGNKVKAEPAKIEAFRASLSKLGDVYVNDAFGTAHRAHSSMVGVN
LPQKAGGFLMKKELNYFAKALESPERPFLAILGGAKVADKIQLINNMLDKVNEMIIGGGM
AFTFLKVLNNMEIGTSLFDEEGAKIVKDLMSKAEKNGVKITLPVDFVTADKFDENAKTGQ
ATVASGIPAGWMGLDCGPESSKKYAEAVTRAKQIVWNGPVGVFEWEAFARGTKALMDEVV
KATSRGCITIIGGGDTATCCAKWNTEDKVSHVSTGGGASLELLEGKVLPGVDALSNI
>sp|P00918|CAH2_HUMAN Carbonic anhydrase 2 OS=Homo sapiens OX=9606 GN=CA2 PE=1 SV=2
MSHHWGYGKHNGPEHWHKDFPIAKGERQSPVDIDTHTAKYDPSLKPLSVSYDQATSLRIL
NNGHAFNVEFDDSQDKAVLKGGPLDGTYRLIQFHFHWGSLDGQGSEHTVDKKKYAAELHL
VHWNTKYGDFGKAVQQPDGLAVLGIFLKVGSAKPGLQKVVDVLDSIKTKGKSADFTNFDP
RGLLPESLDYWTYPGSLTTPPLLECVTWIVLKEPISVSSEQVLKFRKLNFNGEGEPEELM
VDNWRPAQPLKNRQIKASFK
>sp|P01024|CO3_HUMAN Complement C3 OS=Homo sapiens OX=9606 GN=C3 PE=1 SV=2
MGPTSGPSLLLLLLTHLPLALGSPMYSIITPNILRLESEETMVLEAHDAQGDVPVTVTVH
DFPGKKLVLSSEKTVLTPATNHMGNVTFTIPANREFKSEKGRNKFVTVQATFGTQVVEKV
VLVSLQSGYLFIQTDKTIYTPGSTVLYRIFTVNHKLLPVGRTVMVNIENPEGIPVKQDSL
SSQNQLGVLPLSWDIPELVNMGQWKIRAYYENSPQQVFSTEFEVKEYVLPSFEVIVEPTE
KFYYIYNEKGLEVTITARFLYGKKVEGTAFVIFGIQDGEQRISLPESLKRIPIEDGSGEV
VLSRKVLLDGVQNPRAEDLVGKSLYVSATVILHSGSDMVQAERSGIPIVTSPYQIHFTKT
PKYFKPGMPFDLMVFVTNPDGSPAYRVPVAVQGEDTVQSLTQGDGVAKLSINTHPSQKPL
SITVRTKKQELSEAEQATRTMQALPYSTVGNSNNYLHLSVLRTELRPGETLNVNFLLRMD
RAHEAKIRYYTYLIMNKGRLLKAGRQVREPGQDLVVLPLSITTDFIPSFRLVAYYTLIGA
SGQREVVADSVWVDVKDSCVGSLVVKSGQSEDRQPVPGQQMTLKIEGDHGARVVLVAVDK
GVFVLNKKNKLTQSKIWDVVEKADIGCTPGSGKDYAGVFSDAGLTFTSSSGQQTAQRAEL
QCPQPAARRRRSVQLTEKRMDKVGKYPKELRKCCEDGMRENPMRFSCQRRTRFISLGEAC
KKVFLDCCNYITELRRQHARASHLGLARSNLDEDIIAEENIVSRSEFPESWLWNVEDLKE

PPKNGISTKLMNIFLKDSITTWEILAVSMSDKKGICVADPFEVTVMQDFFIDLRLPYSVV
RNEQVEIRAVLYNYRQNQELKVRVELLHNPAFCSLATTKRRHQQTVTIPPKSSLSVPYVI
VPLKTGLQEVEVKAAVYHHFISDGVRKSLKVVPEGIRMNKTVAVRTLDPERLGREGVQKE
DIPPADLSDQVPDTESETRILLQGTPVAQMTEDAVDAERLKHLIVTPSGCGEQNMIGMTP
TVIAVHYLDETEQWEKFGLEKRQGALELIKKGYTQQLAFRQPSSAFAAFVKRAPSTWLTA
YVVKVFSLAVNLIAIDSQVLCGAVKWLILEKQKPDGVFQEDAPVIHQEMIGGLRNNNEKD
MALTAFVLISLQEAKDICEEQVNSLPGSITKAGDFLEANYMNLQRSYTVAIAGYALAQMG
RLKGPLLNKFLTTAKDKNRWEDPGKQLYNVEATSYALLALLQLKDFDFVPPVVRWLNEQR
YYGGGYGSTQATFMVFQALAQYQKDAPDHQELNLDVSLQLPSRSSKITHRIHWESASLLR
SEETKENEGFTVTAEGKGQGTLSVVTMYHAKAKDQLTCNKFDLKVTIKPAPETEKRPQDA
KNTMILEICTRYRGDQDATMSILDISMMTGFAPDTDDLKQLANGVDRYISKYELDKAFSD
RNTLIIYLDKVSHSEDDCLAFKVHQYFNVELIQPGAVKVYAYYNLEESCTRFYHPEKEDG
KLNKLCRDELCRCAEENCFIQKSDDKVTLEERLDKACEPGVDYVYKTRLVKVQLSNDFDE
YIMAIEQTIKSGSDEVQVGQQRTFISPIKCREALKLEEKKHYLMWGLSSDFWGEKPNLSY
IIGKDTWVEHWPEEDECQDEENQKQCQDLGAFTESMVVFGCPN
>sp|P01766|HV313_HUMAN Immunoglobulin heavy variable 3-13 OS=Homo sapiens OX=9606
GN=IGHV3-13 PE=1 SV=2
MELGLSWVFLVAILEGVQCEVQLVESGGGLVQPGGSLRLSCAASGFTFSSYDMHWVRQAT
GKGLEWVSAIGTAGDPYYPGSVKGRFTISRENAKNSLYLQMNSLRAGDTAVYYCAR
>sp|P01834|IGKC_HUMAN Immunoglobulin kappa constant OS=Homo sapiens OX=9606
GN=IGKC PE=1 SV=2
RTVAAPSVFIFPPSDEQLKSGTASVVCLLNNFYPREAKVQWKVDNALQSGNSQESVTEQD
SKDSTYSLSSTLTLSKADYEKHKVYACEVTHQGLSSPVTKSFNRGEC
>sp|P01857|IGHG1_HUMAN Immunoglobulin heavy constant gamma 1 OS=Homo sapiens
OX=9606 GN=IGHG1 PE=1 SV=2
ASTKGPSVFPLAPSSKSTSGGTAALGCLVKDYFPEPVTVSWNSGALTSGVHTFPAVLQSS
GLYSLSSVVTVPSSSLGTQTYICNVNHKPSNTKVDKKVEPKSCDKTHTCPPCPAPELLGG
PSVFLFPPKPKDTLMISRTPEVTCVVVDVSHEDPEVKFNWYVDGVEVHNAKTKPREEQYN
STYRVVSVLTVLHQDWLNGKEYKCKVSNKALPAPIEKTISKAKGQPREPQVYTLPPSRDE
LTKNQVSLTCLVKGFYPSDIAVEWESNGQPENNYKTTPPVLDSDGSFFLYSKLTVDKSRW
QQGNVFSCSVMHEALHNHYTQKSLSLSPELQLEESCAEAQDGELDGLWTTITIFITLFLL
SVCYSATVTFFKVKWIFSSVVDLKQTIIPDYRNMIGQGA
>sp|P01859|IGHG2_HUMAN Immunoglobulin heavy constant gamma 2 OS=Homo sapiens
OX=9606 GN=IGHG2 PE=1 SV=3
ASTKGPSVFPLAPCSRSTSESTAALGCLVKDYFPEPVTVSWNSGALTSGVHTFPAVLQSS
GLYSLSSVVTVPSSNFGTQTYTCNVDHKPSNTKVDKTVERKCCVECPPCPAPPVAGPSVF
LFPPKPKDTLMISRTPEVTCVVVDVSHEDPEVQFNWYVDGVEVHNAKTKPREEQFNSTFR
VVSVLTVVHQDWLNGKEYKCKVSNKGLPAPIEKTISKTKGQPREPQVYTLPPSREEMTKN
QVSLTCLVKGFYPSDISVEWESNGQPENNYKTTPPMLDSDGSFFLYSKLTVDKSRWQQGN
VFSCSVMHEALHNHYTQKSLSLSPELQLEESCAEAQDGELDGLWTTITIFITLFLLSVCY
SATITFFKVKWIFSSVVDLKQTIVPDYRNMIRQGA
>sp|P01860|IGHG3_HUMAN Immunoglobulin heavy constant gamma 3 OS=Homo sapiens
OX=9606 GN=IGHG3 PE=1 SV=3
ASTKGPSVFPLAPCSRSTSGGTAALGCLVKDYFPEPVTVSWNSGALTSGVHTFPAVLQSS

GLYSLSSVVTVPSSSLGTQTYTCNVNHKPSNTKVDKRVELKTPLGDTTHTCPRCPEPKSC
DTPPPCPRCPEPKSCDTPPPCPRCPEPKSCDTPPPCPRCPAPELLGGPSVFLFPPKPKDT
LMISRTPEVTCVVVDVSHEDPEVQFKWYVDGVEVHNAKTKPREEQYNSTFRVVSVLTVLH
QDWLNGKEYKCKVSNKALPAPIEKTISKTKGQPREPQVYTLPPSREEMTKNQVSLTCLVK
GFYPSDIAVEWESSGQPENNYNTTPPMLDSDGSFFLYSKLTVDKSRWQQGNIFSCSVMHE
ALHNRFTQKSLSLSPELQLEESCAEAQDGELDGLWTTITIFITLFLLSVCYSATVTFFKV
KWIFSSVVDLKQTIIPDYRNMIGQGA
>sp|P01861|IGHG4_HUMAN Immunoglobulin heavy constant gamma 4 OS=Homo sapiens OX=9606 GN=IGHG4 PE=1 SV=2
ASTKGPSVFPLAPCSRSTSESTAALGCLVKDYFPEPVTVSWNSGALTSGVHTFPAVLQSS
GLYSLSSVVTVPSSSLGTKTYTCNVDHKPSNTKVDKRVESKYGPPCPSCPAPEFLGGPSV
FLFPPKPKDTLMISRTPEVTCVVVDVSQEDPEVQFNWYVDGVEVHNAKTKPREEQFNSTY
RVVSVLTVLHQDWLNGKEYKCKVSNKGLPSSIEKTISKAKGQPREPQVYTLPPSQEEMTK
NQVSLTCLVKGFYPSDIAVEWESNGQPENNYKTTPPVLDSDGSFFLYSRLTVDKSRWQEG
NVFSCSVMHEALHNHYTQKSLSLSLELQLEESCAEAQDGELDGLWTTITIFITLFLLSVC
YSATVTFFKVKWIFSSVVDLKQTIVPDYRNMIRQGA
>sp|P01871|IGHM_HUMAN Immunoglobulin heavy constant mu OS=Homo sapiens OX=9606 GN=IGHM PE=1 SV=5
GSASAPTLFPLVSCENSPSDTSSVAVGCLAQDFLPDSITFSWKYKNNSDISSTRGFPSVL
RGGKYAATSQVLLPSKDVMQGTDEHVVCKVQHPNGNKEKNVPLPVIAELPPKVSVFVPPR
DGFFGNPRKSKLICQATGFSPRQIQVSWLREGKQVGSGVTTDQVQAEAKESGPTTYKVTS
TLTIKESDWLGQSMFTCRVDHRGLTFQQNASSMCVPDQDTAIRVFAIPPSFASIFLTKST
KLTCLVTDLTTYDSVTISWTRQNGEAVKTHTNISESHPNATFSAVGEASICEDDWNSGER
FTCTVTHTDLPSPLKQTISRPKGVALHRPDVYLLPPAREQLNLRESATITCLVTGFSPAD
VFVQWMQRGQPLSPEKYVTSAPMPEPQAPGRYFAHSILTVSEEEWNTGETYTCVVAHEAL
PNRVTERTVDKSTEGEVSADEEGFENLWATASTFIVLFLLSLFYSTTVTLFKVK
>sp|P01876|IGHA1_HUMAN Immunoglobulin heavy constant alpha 1 OS=Homo sapiens OX=9606 GN=IGHA1 PE=1 SV=3
ASPTSPKVFPLSLCSTQPDGNVVIACLVQGFFPQEPLSVTWSESGQGVTARNFPPSQDAS
GDLYTTSSQLTLPATQCLAGKSVTCHVKHYTNPSQDVTVPCPVPSTPPTPSPSTPPTPSP
SCCHPRLSLHRPALEDLLLGSEANLTCTLTGLRDASGVTFTWTPSSGKSAVQGPPERDLC
GCYSVSSVLPGCAEPWNHGKTFTCTAAYPESKTPLTATLSKSGNTFRPEVHLLPPPSEEL
ALNELVTLTCLARGFSPKDVLVRWLQGSQELPREKYLTWASRQEPSQGTTTFAVTSILRV
AAEDWKKGDTFSCMVGHEALPLAFTQKTIDRLADWQMPPPYVVLDLPQETLEEETPGANL
WPTTITFLTLFLLSLFYSTALTVTSVRGPSGNREGPQY
>sp|P02042|HBD_HUMAN Hemoglobin subunit delta OS=Homo sapiens OX=9606 GN=HBD PE=1 SV=2
MVHLTPEEKTAVNALWGKVNVDAVGGEALGRLLVVYPWTQRFFESFGDLSSPDAVMGNPK
VKAHGKKVLGAFSDGLAHLDNLKGTFSQLSELHCDKLHVDPENFRLLGNVLVCVLARNFG
KEFTPQMQAAYQKVVAGVANALAHKYH
>sp|P02489|CRYAA_HUMAN Alpha-crystallin A chain OS=Homo sapiens OX=9606 GN=CRYAA PE=1 SV=2
MDVTIQHPWFKRTLGPFYPSRLFDQFFGEGLFEYDLLPFLSSTISPYYRQSLFRTVLDSG
ISEVRSDRDKFVIFLDVKHFSPEDLTVKVQDDFVEIHGKHNERQDDHGYISREFHRRYRL

PSNVDQSALSCSLSADGMLTFCGPKIQTGLDATHAERAIPVSREEKPTSAPSS
>sp|P02511|CRYAB_HUMAN Alpha-crystallin B chain OS=Homo sapiens OX=9606 GN=CRYAB PE=1 SV=2
MDIAIHHPWIRRPFFPFHSPSRLFDQFFGEHLLESDLFPTSTSLSPFYLRPPSFLRAPSW
FDTGLSEMRLEKDRFSVNLDVKHFSPEELKVKVLGDVIEVHGKHEERQDEHGFISREFHR
KYRIPADVDPLTITSSLSSDGVLTVNGPRKQVSGPERTIPITREEKPAVTAAPKK
>sp|P02533|K1C14_HUMAN Keratin, type I cytoskeletal 14 OS=Homo sapiens OX=9606 GN=KRT14 PE=1 SV=4
MTTCSRQFTSSSSMKGSCGIGGGIGGGSSRISSVLAGGSCRAPSTYGGGLSVSSSRFSSG
GACGLGGGYGGGFSSSSSSFGSGFGGGYGGGLGAGLGGGFGGGFAGGDGLLVGSEKVTMQ
NLNDRLASYLDKVRALEEANADLEVKIRDWYQRQRPAEIKDYSPYFKTIEDLRNKILTAT
VDNANVLLQIDNARLAADDFRTKYETELNLRMSVEADINGLRRVLDELTLARADLEMQIE
SLKEELAYLKKNHEEEMNALRGQVGGDVNVEMDAAPGVDLSRILNEMRDQYEKMAEKNRK
DAEEWFFTKTEELNREVATNSELVQSGKSEISELRRTMQNLEIELQSQLSMKASLENSLE
ETKGRYCMQLAQIQEMIGSVEEQLAQLRCEMEQQNQEYKILLDVKTRLEQEIATYRRLLE
GEDAHLSSSQFSSGSQSSRDVTSSSRQIRTKVMDVHDGKVVSTHEQVLRTKN
>sp|P02545|LMNA_HUMAN Prelamin-A/C OS=Homo sapiens OX=9606 GN=LMNA PE=1 SV=1
METPSQRRATRSGAQASSTPLSPTRITRLQEKEDLQELNDRLAVYIDRVRSLETENAGLR
LRITESEEVVSREVSGIKAAYEAELGDARKTLDSVAKERARLQLELSKVREEFKELKARN
TKKEGDLIAAQARLKDLEALLNSKEAALSTALSEKRTLEGELHDLRGQVAKLEAALGEAK
KQLQDEMLRRVDAENRLQTMKEELDFQKNIYSEELRETKRRHETRLVEIDNGKQREFESR
LADALQELRAQHEDQVEQYKKELEKTYSAKLDNARQSAERNSNLVGAAHEELQQSRIRID
SLSAQLSQLQKQLAAKEAKLRDLEDSLARERDTSRRLLAEKEREMAEMRARMQQQLDEYQ
ELLDIKLALDMEIHAYRKLLEGEEERLRLSPSPTSQRSRGRASSHSSQTQGGGSVTKKRK
LESTESRSSFSQHARTSGRVAVEEVDEEGKFVRLRNKSNEDQSMGNWQIKRQNGDDPLLT
YRFPPKFTLKAGQVVTIWAAGAGATHSPPTDLVWKAQNTWGCGNSLRTALINSTGEEVAM
RKLVRSVTVVEDDEDEDGDDLLHHHHGSHCSSSGDPAEYNLRSRTVLCGTCGQPADKASA
SGSGAQVGGPISSGSSASSVTVTRSYRSVGGSGGGSFGDNLVTRSYLLGNSSPRTQSPQN
CSIM
>sp|P02647|APOA1_HUMAN Apolipoprotein A-I OS=Homo sapiens OX=9606 GN=APOA1 PE=1 SV=1
MKAAVLTLAVLFLTGSQARHFWQQDEPPQSPWDRVKDLATVYVDVLKDSGRDYVSQFEGS
ALGKQLNLKLLDNWDSVTSTFSKLREQLGPVTQEFWDNLEKETEGLRQEMSKDLEEVKAK
VQPYLDDFQKKWQEEMELYRQKVEPLRAELQEGARQKLHELQEKLSPLGEEMRDRARAHV
DALRTHLAPYSDELRQRLAARLEALKENGGARLAEYHAKATEHLSTLSEKAKPALEDLRQ
GLLPVLESFKVSFLSALEEYTKKLNTQ
>sp|P02649|APOE_HUMAN Apolipoprotein E OS=Homo sapiens OX=9606 GN=APOE PE=1 SV=1
MKVLWAALLVTFLAGCQAKVEQAVETEPEPELRQQTEWQSGQRWELALGRFWDYLRWVQT
LSEQVQEELLSSQVTQELRALMDETMKELKAYKSELEEQLTPVAEETRARLSKELQAAQA
RLGADMEDVCGRLVQYRGEVQAMLGQSTEELRVRLASHLRKLRKRLLRDADDLQKRLAVY
QAGAREGAERGLSAIRERLGPLVEQGRVRAATVGSLAGQPLQERAQAWGERLRARMEEMG
SRTRDRLDEVKEQVAEVRAKLEEQAQQIRLQAEAFQARLKSWFEPLVEDMQRQWAGLVEK
VQAAVGTSAAPVPSDNH

>sp|P02730|B3AT_HUMAN Band 3 anion transport protein OS=Homo sapiens OX=9606 GN=SLC4A1 PE=1 SV=3
MEELQDDYEDMMEENLEQEEYEDPDIPESQMEEPAAHDTEATATDYHTTSHPGTHKVYVE
LQELVMDEKNQELRWMEAARWVQLEENLGENGAWGRPHLSHLTFWSLLELRRVFTKGTVL
LDLQETSLAGVANQLLDRFIFEDQIRPQDREELLRALLLKHSHAGELEALGGVKPAVLTR
SGDPSQPLLPQHSSLETQLFCEQGDGGTEGHSPSGILEKIPPDSEATLVLVGRADFLEQP
VLGFVRLQEAAELEAVELPVPIRFLFVLLGPEAPHIDYTQLGRAAATLMSERVFRIDAYM
AQSRGELLHSLEGFLDCSLVLPPTDAPSEQALLSLVPVQRELLRRRYQSSPAKPDSSFYK
GLDLNGGPDDPLQQTGQLFGGLVRDIRRRYPYYLSDITDAFSPQVLAAVIFIYFAALSPA
ITFGGLLGEKTRNQMGVSELLISTAVQGILFALLGAQPLLVVGFSGPLLVFEEAFFSFCE
TNGLEYIVGRVWIGFWLILLVVLVVAFEGSFLVRFISRYTQEIFSFLISLIFIYETFSKL
IKIFQDHPLQKTYNYNVLMVPKPQGPLPNTALLSLVLMAGTFFFAMMLRKFKNSSYFPGK
LRRVIGDFGVPISILIMVLVDFFIQDTYTQKLSVPDGFKVSNSSARGWVIHPLGLRSEFP
IWMMFASALPALLVFILIFLESQITTLIVSKPERKMVKGSGFHLDLLLVVGMGGVAALFG
MPWLSATTVRSVTHANALTVMGKASTPGAAAQIQEVKEQRISGLLVAVLVGLSILMEPIL
SRIPLAVLFGIFLYMGVTSLSGIQLFDRILLLFKPPKYHPDVPYVKRVKTWRMHLFTGIQ
IICLAVLWVVKSTPASLALPFVLILTVPLRRVLLPLIFRNVELQCLDADDAKATFDEEEG
RDEYDEVAMPV
>sp|P02743|SAMP_HUMAN Serum amyloid P-component OS=Homo sapiens OX=9606 GN=APCS PE=1 SV=2
MNKPLLWISVLTSLLEAFAHTDLSGKVFVFPRESVTDHVNLITPLEKPLQNFTLCFRAYS
DLSRAYSLFSYNTQGRDNELLVYKERVGEYSLYIGRHKVTSKVIEKFPAPVHICVSWESS
SGIAEFWINGTPLVKKGLRQGYFVEAQPKIVLGQEQDSYGGKFDRSQSFVGEIGDLYMWD
SVLPPENILSAYQGTPLPANILDWQALNYEIRGYVIIKPLVWV
>sp|P02751|FINC_HUMAN Fibronectin OS=Homo sapiens OX=9606 GN=FN1 PE=1 SV=5
MLRGPGPGLLLLAVQCLGTAVPSTGASKSKRQAQQMVQPQSPVAVSQSKPGCYDNGKHYQ
INQQWERTYLGNALVCTCYGGSRGFNCESKPEAEETCFDKYTGNTYRVGDTYERPKDSMI
WDCTCIGAGRGRISCTIANRCHEGGQSYKIGDTWRRPHETGGYMLECVCLGNGKGEWTCK
PIAEKCFDHAAGTSYVVGETWEKPYQGWMMVDCTCLGEGSGRITCTSRNRCNDQDTRTSY
RIGDTWSKKDNRGNLLQCICTGNGRGEWKCERHTSVQTTSSGSGPFTDVRAAVYQPQPHP
QPPPYGHCVTDSGVVYSVGMQWLKTQGNKQMLCTCLGNGVSCQETAVTQTYGGNSNGEPC
VLPFTYNGRTFYSCTTEGRQDGHLWCSTTSNYEQDQKYSFCTDHTVLVQTRGGNSNGALC
HFPFLYNNHNYTDCTSEGRRDNMKWCGTTQNYDADQKFGFCPMAAHEEICTTNEGVMYRI
GDQWDKQHDMGHMMRCTCVGNGRGEWTCIAYSQLRDQCIVDDITYNVNDTFHKRHEEGHM
LNCTCFGQGRGRWKCDPVDQCQDSETGTFYQIGDSWEKYVHGVRYQCYCYGRGIGEWHCQ
PLQTYPSSSGPVEVFITETPSQPNSHPIQWNAPQPSHISKYILRWRPKNSVGRWKEATIP
GHLNSYTIKGLKPGVVYEGQLISIQQYGHQEVTRFDFTTTSTSTPVTSNTVTGETTPFSP
LVATSESVTEITASSFVVSWVSASDTVSGFRVEYELSEEGDEPQYLDLPSTATSVNIPDL
LPGRKYIVNVYQISEDGEQSLILSTSQTTAPDAPPDTTVDQVDDTSIVVRWSRPQAPITG
YRIVYSPSVEGSSTELNLPETANSVTLSDLQPGVQYNITIYAVEENQESTPVVIQQETTG
TPRSDTVPSPRDLQFVEVTDVKVTIMWTPPESAVTGYRVDVIPVNLPGEHGQRLPISRNT
FAEVTGLSPGVTYYFKVFAVSHGRESKPLTAQQTTKLDAPTNLQFVNETDSTVLVRWTPP
RAQITGYRLTVGLTRRGQPRQYNVGPSVSKYPLRNLQPASEYTVSLVAIKGNQESPKATG
VFTTLQPGSSIPPYNTEVTETTIVITWTPAPRIGFKLGVRPSQGGEAPREVTSDSGSIVV

SGLTPGVEYVVYTIQVLRDGQERDAPIVNKVVTPLSPPTNLHLEANPDTGVLTVSWERSTT
PDITGYRITTTPTNGQQGNSLEEVVHADQSSCTFDNLSPGLEYNVSVYTVKDDKESVPIS
DTIIPEVPQLTDLSFVDITDSSIGLRWTPLNSSTIIGYRITVVAAGEGIPIFEDFVDSSV
GYYTVTGLEPGIDYDISVITLINGGESAPTTLTQQTAVPPPTDLRFTNIGPDTMRVTWAP
PPSIDLTNFLVRYSPVKNEEDVAELSISPSDNAVVLTNLLPGTEYVVSVSSVYEQHESTP
LRGRQKTGLDSPTGIDFSDITANSFTVHWIAPRATITGYRIRHHPEHFSGRPREDRVPHS
RNSITLTNLTPGTEYVVSIVALNGREESPLLIGQQSTVSDVPRDLEVVAATPTSLLISWD
APAVTVRYYRITYGETGGNSPVQEFTVPGSKSTATISGLKPGVDYTITVYAVTGRGDSPA
SSKPISINYRTEIDKPSQMQVTDVQDNSISVKWLPSSSPVTGYRVTTTPKNGPGPTKTKT
AGPDQTEMTIEGLQPTVEYVVSVYAQNPSGESQPLVQTAVTNIDRPKGLAFTDVDVDSIK
IAWESPQGQVSRYRVTYSSPEDGIHELFPAPDGEEDTAELQGLRPGSEYTVSVVALHDDM
ESQPLIGTQSTAIPAPTDLKFTQVTPTSLSAQWTPPNVQLTGYRVRVTPKEKTGPMKEIN
LAPDSSSVVVSGLMVATKYEVSVYALKDTLTSRPAQGVVTTLENVSPPRRARVTDATETT
ITISWRTKTETITGFQVDAVPANGQTPIQRTIKPDVRSYTITGLQPGTDYKIYLYTLNDN
ARSSPVVIDASTAIDAPSNLRFLATTPNSLLVSWQPPRARITGYIIKYEKPGSPPREVVP
RPRPGVTEATITGLEPGTEYTIYVIALKNNQKSEPLIGRKKTDELPQLVTLPHPNLHGPE
ILDVPSTVQKTPFVTHPGYDTGNGIQLPGTSGQQPSVGQQMIFEEHGFRRTTPPTTATPI
RHRPRPYPPNVGEEIQIGHIPREDVDYHLYPHGPGLNPNASTGQEALSQTTISWAPFQDT
SEYIISCHPVGTDEEPLQFRVPGTSTSATLTGLTRGATYNVIVEALKDQQRHKVREEVVT
VGNSVNEGLNQPTDDSCFDPYTVSHYAVGDEWERMSESGFKLLCQCLGFGSGHFRCDSSR
WCHDNGVNYKIGEKWDRQGENGQMMSCTCLGNGKGEFKCDPHEATCYDDGKTYHVGEQWQ
KEYLGAICSCTCFGGQRGWRCDNCRRPGGEPSPEGTTGQSYNQYSQRYHQRTNTNVNCPI
ECFMPLDVQADREDSRE
>sp|P02763|A1AG1_HUMAN Alpha-1-acid glycoprotein 1 OS=Homo sapiens OX=9606 GN=ORM1 PE=1 SV=2
MALSWVLTVLSLLPLLEAQIPLCANLVPVPITNATLDRITGKWFYIASAFRNEEYNKSVQ
EIQATFFYFTPNKTEDTIFLREYQTRQDQCIYNTTYLNVQRENGTISRYVGGQEHFAHLL
ILRDTKTYMLAFDVNDEKNWGLSVYADKPETTKEQLGEFYEALDCLRIPKSDVVYTDWKK
DKCEPLEKQHEKERKQEEGES
>sp|P02766|TTHY_HUMAN Transthyretin OS=Homo sapiens OX=9606 GN=TTR PE=1 SV=1
MASHRLLLLCLAGLVFVSEAGPTGTGESKCPLMVKVLDAVRGSPAINVAVHVFRKAADDT
WEPFASGKTSESGELHGLTTEEEFVEGIYKVEIDTKSYWKALGISPFHEHAEVVFTANDS
GPRRYTIAALLSPYSYSTTAVVTNPKE
>sp|P02787|TRFE_HUMAN Serotransferrin OS=Homo sapiens OX=9606 GN=TF PE=1 SV=4
MRLAVGALLVCAVLGLCLAVPDKTVRWCAVSEHEATKCQSFRDHMKSVIPSDGPSVACVK
KASYLDCIRAIAANEADAVTLDAGLVYDAYLAPNNLKPVVAEFYGSKEDPQTFYYAVAVV
KKDSGFQMNQLRGKKSCHTGLGRSAGWNIPIGLLYCDLPEPRKPLEKAVANFFSGSCAPC
ADGTDFPQLCQLCPGCGCSTLNQYFGYSGAFKCLKDGAGDVAFVKHSTIFENLANKADRD
QYELLCLDNTRKPVDEYKDCHLAQVPSHTVVARSMGGKEDLIWELLNQAQEHFGKDKSKE
FQLFSSPHGKDLLFKDSAHGFLKVPPRMDAKMYLGYEYVTAIRNLREGTCPEAPTDECKP
VKWCALSHHERLKCDEWSVNSVGKIECVSAETTEDCIAKIMNGEADAMSLDGGFVYIAGK
CGLVPVLAENYNKSDNCEDTPEAGYFAVAVVKKSASDLTWDNLKGKKSCHTAVGRTAGWN
IPMGLLYNKINHCRFDEFFSEGCAPGSKKDSSLCKLCMGSGLNLCEPNNKEGYYGYTGAF
RCLVEKGDVAFVKHQTVPQNTGGKNPDPWAKNLNEKDYELLCLDGTRKPVEEYANCHLAR

APNHAVVTRKDKEACVHKILRQQQHLFGSNVTDCSGNFCLFRSETKDLLFRDDTVCLAKL
HDRNTYEKYLGEEYVKAVGNLRKCSTSSLLEACTFRRP
>sp|P02788|TRFL_HUMAN Lactotransferrin OS=Homo sapiens OX=9606 GN=LTF PE=1 SV=6
MKLVFLVLLFLGALGLCLAGRRRSVQWCAVSQPEATKCFQWQRNMRKVRGPPVSCIKRDS
PIQCIQAIAENRADAVTLDGGFIYEAGLAPYKLRPVAAEVYGTERQPRTHYYAVAVVKKG
GSFQLNELQGLKSCHTGLRRTAGWNVPIGTLRPFLNWTGPPEPIEAAVARFFSASCVPGA
DKGQFPNLCRLCAGTGENKCAFSSQEPYFSYSGAFKCLRDGAGDVAFIRESTVFEDLSDE
AERDEYELLCPDNTRKPVDKFKDCHLARVPSHAVVARSVNGKEDAIWNLLRQAQEKFGKD
KSPKFQLFGSPSGQKDLLFKDSAIGFSRVPPRIDSGLYLGSGYFTAIQNLRKSEEEVAAR
RARVVWCAVGEQELRKCNQWSGLSEGSVTCSSASTTEDCIALVLKGEADAMSLDGGYVYT
AGKCGLVPVLAENYKSQQSSDPDPNCVDRPVEGYLAVAVVRRSDTSLTWNSVKGKKSCHT
AVDRTAGWNIPMGLLFNQTGSCKFDEYFSQSCAPGSDPRSNLCALCIGDEQGENKCVPNS
NERYYGYTGAFRCLAENAGDVAFVKDVTVLQNTDGNNNEAWAKDLKLADFALLCLDGKRK
PVTEARSCHLAMAPNHAVVSRMDKVERLKQVLLHQQAKFGRNGSDCPDKFCLFQSETKNL
LFNDNTECLARLHGKTTYEKYLGPQYVAGITNLKKCSTSPLLEACEFLRK
>sp|P02792|FRIL_HUMAN Ferritin light chain OS=Homo sapiens OX=9606 GN=FTL PE=1 SV=2
MSSQIRQNYSTDVEAAVNSLVNLYLQASYTYLSLGFYFDRDDVALEGVSHFFRELAEEKR
EGYERLLKMQNQRGGRALFQDIKKPAEDEWGKTPDAMKAAMALEKKLNQALLDLHALGSA
RTDPHLCDFLETHFLDEEVKLIKKMGDHLTNLHRLGGPEAGLGEYLFERLTLKHD
>sp|P02794|FRIH_HUMAN Ferritin heavy chain OS=Homo sapiens OX=9606 GN=FTH1 PE=1
SV=2
MTTASTSQVRQNYHQDSEAAINRQINLELYASYVYLSMSYYFDRDDVALKNFAKYFLHQS
HEEREHAEKLMKLQNQRGGRIFLQDIKKPDCDDWESGLNAMECALHLEKNVNQSLLELHK
LATDKNDPHLCDFIETHYLNEQVKAIKELGDHVTNLRKMGAPESGLAEYLFDKHTLGDSD
NES
>sp|P04003|C4BPA_HUMAN C4b-binding protein alpha chain OS=Homo sapiens OX=9606
GN=C4BPA PE=1 SV=2
MHPPKTPSGALHRKRKMAAWPFSRLWKVSDPILFQMTLIAALLPAVLGNCGPPPTLSFAA
PMDITLTETRFKTGTTLKYTCLPGYVRSHSTQTLTCNSDGEWVYNTFCIYKRCRHPGELR
NGQVEIKTDLSFGSQIEFSCSEGFFLIGSTTSRCEVQDRGVGWSHPLPQCEIVKCKPPPD
IRNGRHSGEENFYAYGFSVTYSCDPRFSLLGHASISCTVENETIGVWRPSPPTCEKITCR
KPDVSHGEMVSGFGPIYNYKDTIVFKCQKGFVLRGSSVIHCDADSKWNPSPPACEPNSCI
NLPDIPHASWETYPRPTKEDVYVVGTVLRYRCHPGYKPTTDEPTTVICQKNLRWTPYQGC
EALCCPEPKLNNGEITQHRKSRPANHCVYFYGDEISFSCHETSRFSAICQGDGTWSPRTP
SCGDICNFPPKIAHGHYKQSSSYSFFKEEIIYECDKGYILVGQAKLSCSYSHWSAPAPQC
KALCRKPELVNGRLSVDKDQYVEPENVTIQCDSGYGVVGPQSITCSGNRTWYPEVPKCEW
ETPEGCEQVLTGKRLMQCLPNPEDVKMALEVYKLSLEIEQLELQRDSARQSTLDKEL
>sp|P04075|ALDOA_HUMAN Fructose-bisphosphate aldolase A OS=Homo sapiens OX=9606
GN=ALDOA PE=1 SV=2
MPYQYPALTPEQKKELSDIAHRIVAPGKGILAADESTGSIAKRLQSIGTENTEENRRFYR
QLLLTADDRVNPCIGGVILFHETLYQKADDGRPFPQVIKSKGGVVGIKVDKGVVPLAGTN
GETTTQGLDGLSERCAQYKKDGADFAKWRCVLKIGEHTPSALAIMENANVLARYASICQQ
NGIVPIVEPEILPDGDHDLKRCQYVTEKVLAAVYKALSDHHIYLEGTLLKPNMVTPGHAC
TQKFSHEEIAMATVTALRRTVPPAVTGITFLSGGQSEEEASINLNAINKCPLLKPWALTF

SYGRALQASALKAWGGKKENLKAAQEEYVKRALANSLACQGKYTPSGQAGAAASESLFVS
NHAY
>sp|P04264|K2C1_HUMAN Keratin, type II cytoskeletal 1 OS=Homo sapiens OX=9606 GN=KRT1 PE=1 SV=6
MSRQFSSRSGYRSGGGFSSGSAGIINYQRRTTSSSTRRSGGGGGRFSSCGGGGGSFGAGG
GFGSRSLVNLGGSKSISISVARGGGRGSGFGGGYGGGGFGGGGFGGGGFGGGGIGGGGFG
GFGSGGGGFGGGGFGGGGYGGGYGPVCPPGGIQEVTINQSLLQPLNVEIDPEIQKVKSRE
REQIKSLNNQFASFIDKVRFLEQQNQVLQTKWELLQQVDTSTRTHNLEPYFESFINNLRR
RVDQLKSDQSRLDSELKNMQDMVEDYRNKYEDEINKRTNAENEFVTIKKDVDGAYMTKVD
LQAKLDNLQQEIDFLTALYQAELSQMQTQISETNVILSMDNNRSLDLDSIIAEVKAQYED
IAQKSKAEAESLYQSKYEELQITAGRHGDSVRNSKIEISELNRVIQRLRSEIDNVKKQIS
NLQQSISDAEQRGENALKDAKNKLNDLEDALQQAKEDLARLLRDYQELMNTKLALDLEIA
TYRTLLEGEESRMSGECAPNVSVSVSTSHTTISGGGSRGGGGGGYGSGGSSYGSGGGSYG
SGGGGGGGRGSYGSGGSSYGSGGGSYGSGGGGGGHGSYGSGSSSGGYRGGSGGGGGGSSG
GRGSGGGSSGGSIGGRGSSSGGVKSSGGSSSVKFVSTTYSGVTR
>sp|P04406|G3P_HUMAN Glyceraldehyde-3-phosphate dehydrogenase OS=Homo sapiens OX=9606 GN=GAPDH PE=1 SV=3
MGKVKVGVNGFGRIGRLVTRAAFNSGKVDIVAINDPFIDLNYMVYMFQYDSTHGKFHGTV
KAENGKLVINGNPITIFQERDPSKIKWGDAGAEYVVESTGVFTTMEKAGAHLQGGAKRVI
ISAPSADAPMFVMGVNHEKYDNSLKIISNASCTTNCLAPLAKVIHDNFGIVEGLMTTVHA
ITATQKTVDGPSGKLWRDGRGALQNIIPASTGAAKAVGKVIPELNGKLTGMAFRVPTANV
SVVDLTCRLEKPAKYDDIKKVVKQASEGPLKGILGYTEHQVVSSDFNSDTHSSTFDAGAG
IALNDHFVKLISWYDNEFGYSNRVVDLMAHMASKE
>sp|P04792|HSPB1_HUMAN Heat shock protein beta-1 OS=Homo sapiens OX=9606 GN=HSPB1 PE=1 SV=2
MTERRVPFSLLRGPSWDPFRDWYPHSRLFDQAFGLPRLPEEWSQWLGGSSWPGYVRPLPP
AAIESPAVAAPAYSRALSRQLSSGVSEIRHTADRWRVSLDVNHFAPDELTVKTKDGVVEI
TGKHEERQDEHGYISRCFTRKYTLPPGVDPTQVSSSLSPEGTLTVEAPMPKLATQSNEIT
IPVTFESRAQLGGPEAAKSDETAAK
>sp|P04908|H2A1B_HUMAN Histone H2A type 1-B/E OS=Homo sapiens OX=9606 GN=H2AC4 PE=1 SV=2
MSGRGKQGGKARAKAKTRSSRAGLQFPVGRVHRLLRKGNYSERVGAGAPVYLAAVLEYLT
AEILELAGNAARDNKKTRIIPRHLQLAIRNDEELNKLLGRVTIAQGGVLPNIQAVLLPKK
TESHHKAKGK
>sp|P05023|AT1A1_HUMAN Sodium/potassium-transporting ATPase subunit alpha-1 OS=Homo sapiens OX=9606 GN=ATP1A1 PE=1 SV=1
MGKGVGRDKYEPAAVSEQGDKKGKKGKKDRDMDELKKEVSMDDHKLSLDELHRKYGTDLS
RGLTSARAAEILARDGPNALTPPPTTPEWIKFCRQLFGGFSMLLWIGAILCFLAYSIQAA
TEEEPQNDNLYLGVVLSAVVIITGCFSYYQEAKSSKIMESFKNMVPQQALVIRNGEKMSI
NAEEVVVGDLVEVKGGDRIPADLRIISANGCKVDNSSLTGESEPQTRSPDFTNENPLETR
NIAFFSTNCVEGTARGIVVYTGDRTVMGRIATLASGLEGGQTPIAAEIEHFIHIITGVAV
FLGVSFFILSLILEYTWLEAVIFLIGIIVANVPEGLLATVTVCLTLTAKRMARKNCLVKN
LEAVETLGSTSTICSDKTGTLTQNRMTVAHMWFDNQIHEADTTENQSGVSFDKTSATWLA
LSRIAGLCNRAVFQANQENLPILKRAVAGDASESALLKCIELCCGSVKEMRERYAKIVEI

PFNSTNKYQLSIHKNPNTSEPQHLLVMKGAPERILDRCSSILLHGKEQPLDEELKDAFQN
AYLELGGLGERVLGFCHLFLPDEQFPEGFQFDTDDVNFPIDNLCFVGLISMIDPPRAAVP
DAVGKCRSAGIKVIMVTGDHPITAKAIAKGVGIISEGNETVEDIAARLNIPVSQVNPRDA
KACVVHGSDLKDMTSEQLDDILKYHTEIVFARTSPQQKLIIVEGCQRQGAIVAVTGDGVN
DSPALKKADIGVAMGIAGSDVSKQAADMILLDDNFASIVTGVEEGRLIFDNLKKSIAYTL
TSNIPEITPFLIFIIANIPLPLGTVTILCIDLGTDMVPAISLAYEQAESDIMKRQPRNPK
TDKLVNERLISMAYGQIGMIQALGGFFTYFVILAENGFLPIHLLGLRVDWDDRWINDVED
SYGQQWTYEQRKIVEFTCHTAFFVSIVVVQWADLVICKTRRNSVFQQGMKNKILIFGLFE
ETALAAFLSYCPGMGVALRMYPLKPTWWFCAFPYSLLIFVYDEVRKLIIRRRPGGWVEKE
TYY
>sp|P05089|ARGI1_HUMAN Arginase-1 OS=Homo sapiens OX=9606 GN=ARG1 PE=1 SV=2
MSAKSRTIGIIGAPFSKGQPRGGVEEGPTVLRKAGLLEKLKEQECDVKDYGDLPFADIPN
DSPFQIVKNPRSVGKASEQLAGKVAEVKKNGRISLVLGGDHSLAIGSISGHARVHPDLGV
IWVDAHTDINTPLTTTSGNLHGQPVSFLLKELKGKIPDVPGFSWVTPCISAKDIVYIGLR
DVDPGEHYILKTLGIKYFSMTEVDRLGIGKVMEETLSYLLGRKKRPIHLSFDVDGLDPSF
TPATGTPVVGGLTYREGLYITEEIYKTGLLSGLDIMEVNPSLGKTPEEVTRTVNTAVAIT
LACFGLAREGNHKPIDYLNPPK
>sp|P05091|ALDH2_HUMAN Aldehyde dehydrogenase, mitochondrial OS=Homo sapiens
OX=9606 GN=ALDH2 PE=1 SV=2
MLRAAARFGPRLGRRLLSAAATQAVPAPNQQPEVFCNQIFINNEWHDAVSRKTFPTVNPS
TGEVICQVAEGDKEDVDKAVKAARAAFQLGSPWRRMDASHRGRLLNRLADLIERDRTYLA
ALETLDNGKPYVISYLVDLDMVLKCLRYYAGWADKYHGKTIPIDGDFFSYTRHEPVGVCG
QIIPWNFPLLMQAWKLGPALATGNVVVMKVAEQTPLTALYVANLIKEAGFPPGVVNIVPG
FGPTAGAAIASHEDVDKVAFTGSTEIGRVIQVAAGSSNLKRVTLELGGKSPNIIMSDADM
DWAVEQAHFALFFNQGQCCCAGSRTFVQEDIYDEFVERSVARAKSRVVGNPFDSKTEQGP
QVDETQFKKILGYINTGKQEGAKLLCGGGIAADRGYFIQPTVFGDVQDGMTIAKEEIFGP
VMQILKFKTIEEVVGRANNSTYGLAAAVFTKDLDKANYLSQALQAGTVWVNCYDVFGAQS
PFGGYKMSGSGRELGEYGLQAYTEVKTVTVKVPQKNS
>sp|P05387|RLA2_HUMAN Large ribosomal subunit protein P2 OS=Homo sapiens OX=9606
GN=RPLP2 PE=1 SV=1
MRYVASYLLAALGGNSSPSAKDIKKILDSVGIEADDDRLNKVISELNGKNIEDVIAQGIG
KLASVPAGGAVAVSAAPGSAAPAAGSAPAAAEEKKDEKKEESEESDDDMGFGLFD
>sp|P05813|CRBA1_HUMAN Beta-crystallin A3 OS=Homo sapiens OX=9606 GN=CRYBA1 PE=1
SV=4
METQAEQQELETLPTTKMAQTNPTPGSLGPWKITIYDQENFQGKRMEFTSSCPNVSERSF
DNVRSLKVESGAWIGYEHTSFCGQQFILERGEYPRWDAWSGSNAYHIERLMSFRPICSAN
HKESKMTIFEKENFIGRQWEISDDYPSLQAMGWFNNEVGSMKIQSGAWVCYQYPGYRGYQ
YILECDHHGGDYKHWREWGSHAQTSQIQSIRRIQQ
>sp|P06396|GELS_HUMAN Gelsolin OS=Homo sapiens OX=9606 GN=GSN PE=1 SV=1
MAPHRPAPALLCALSLALCALSLPVRAATASRGASQAGAPQGRVPEARPNSMVVEHPEFL
KAGKEPGLQIWRVEKFDLVPVPTNLYGDFFTGDAYVILKTVQLRNGNLQYDLHYWLGNEC
SQDESGAAAIFTVQLDDYLNGRAVQHREVQGFESATFLGYFKSGLKYKKGGVASGFKHVV
PNEVVVQRLFQVKGRRVVRATEVPVSWESFNNGDCFILDLGNNIHQWCGSNSNRYERLKA
TQVSKGIRDNERSGRARVHVSEEGTEPEAMLQVLGPKPALPAGTEDTAKEDAANRKLAKL

YKVSNGAGTMSVSLVADENPFAQGALKSEDCFILDHGKDGKIFVWKGKQANTEERKAALK
TASDFITKMDYPKQTQVSVLPEGGETPLFKQFFKNWRDPDQTDGLGLSYLSSHIANVERV
PFDAATLHTSTAMAAQHGMDDDGTGQKQIWRIEGSNKVPVDPATYGQFYGGDSYIILYNY
RHGGRQGQIIYNWQGAQSTQDEVAASAILTAQLDEELGGTPVQSRVVQGKEPAHLMSLFG
GKPMIIYKGGTSREGGQTAPASTRLFQVRANSAGATRAVEVLPKAGALNSNDAFVLKTPS
AAYLWVGTGASEAEKTGAQELLRVLRAQPVQVAEGSEPDGFWEALGGKAAYRTSPRLKDK
KMDAHPPRLFACSNKIGRFVIEEVPGELMQEDLATDDVMLLDTWDQVFVWVGKDSQEEEK
TEALTSAKRYIETDPANRDRRTPITVVKQGFEPPSFVGWFLGWDDDYWSVDPLDRAMAEL
AA
>sp|P06576|ATPB_HUMAN ATP synthase subunit beta, mitochondrial OS=Homo sapiens OX=9606 GN=ATP5F1B PE=1 SV=3
MLGFVGRVAAAPASGALRRLTPSASLPPAQLLLRAAPTAVHPVRDYAAQTSPSPKAGAAT
GRIVAVIGAVVDVQFDEGLPPILNALEVQGRETRLVLEVAQHLGESTVRTIAMDGTEGLV
RGQKVLDSGAPIKIPVGPETLGRIMNVIGEPIDERGPIKTKQFAPIHAEAPEFMEMSVEQ
EILVTGIKVVDLLAPYAKGGKIGLFGGAGVGKTVLIMELINNVAKAHGGYSVFAGVGERT
REGNDLYHEMIESGVINLKDATSKVALVYGQMNEPPGARARVALTGLTVAEYFRDQEGQD
VLLFIDNIFRFTQAGSEVSALLGRIPSAVGYQPTLATDMGTMQERITTTKKGSITSVQAI
YVPADDLTDPAPATTFAHLDATTVLSRAIAELGIYPAVDPLDSTSRIMDPNIVGSEHYDV
ARGVQKILQDYKSLQDIIAILGMDELSEEDKLTVSRARKIQRFLSQPFQVAEVFTGHMGK
LVPLKETIKGFQQILAGEYDHLPEQAFYMVGPIEEAVAKADKLAEEHSS
>sp|P06733|ENOA_HUMAN Alpha-enolase OS=Homo sapiens OX=9606 GN=ENO1 PE=1 SV=2
MSILKIHAREIFDSRGNPTVEVDLFTSKGLFRAAVPSGASTGIYEALELRDNDKTRYMGK
GVSKAVEHINKTIAPALVSKKLNVTEQEKIDKLMIEMDGTENKSKFGANAILGVSLAVCK
AGAVEKGVPLYRHIADLAGNSEVILPVPAFNVINGGSHAGNKLAMQEFMILPVGAANFRE
AMRIGAEVYHNLKNVIKEKYGKDATNVGDEGGFAPNILENKEGLELLKTAIGKAGYTDKV
VIGMDVAASEFFRSGKYDLDFKSPDDPSRYISPDQLADLYKSFIKDYPVVSIEDPFDQDD
WGAWQKFTASAGIQVVGDDLTVTNPKRIAKAVNEKSCNCLLLKVNQIGSVTESLQACKLA
QANGWGVMVSHRSGETEDTFIADLVVGLCTGQIKTGAPCRSERLAKYNQLLRIEEELGSK
AKFAGRNFRNPLAK
>sp|P06899|H2B1J_HUMAN Histone H2B type 1-J OS=Homo sapiens OX=9606 GN=H2BC11 PE=1 SV=3
MPEPAKSAPAPKKGSKKAVTKAQKKDGKKRKRSRKESYSIYVYKVLKQVHPDTGISSKAM
GIMNSFVNDIFERIAGEASRLAHYNKRSTITSREIQTAVRLLLPGELAKHAVSEGTKAVT
KYTSAK
>sp|P07195|LDHB_HUMAN L-lactate dehydrogenase B chain OS=Homo sapiens OX=9606 GN=LDHB PE=1 SV=2
MATLKEKLIAPVAEEEATVPNNKITVVGVGQVGMACAISILGKSLADELALVDVLEDKLK
GEMMDLQHGSLFLQTPKIVADKDYSVTANSKIVVVTAGVRQQEGESRLNLVQRNVNVFKF
IIPQIVKYSPDCIIIVVSNPVDILTYVTWKLSGLPKHRVIGSGCNLDSARFRYLMAEKLG
IHPSSCHGWILGEHGDSSVAVWSGVNVAGVSLQELNPEMGTDNDSENWKEVHKMVVESAY
EVIKLKGYTNWAIGLSVADLIESMLKNLSRIHPVSTMVKGMYGIENEVFLSLPCILNARG
LTSVINQKLKDDEVAQLKKSADTLWDIQKDLKDL
>sp|P07315|CRGC_HUMAN Gamma-crystallin C OS=Homo sapiens OX=9606 GN=CRYGC PE=1 SV=2

MGKITFYEDRAFQGRSYETTTDCPNLQPYFSRCNSIRVESGCWMLYERPNYQGQQYLLRR
GEYPDYQQWMGLSDSIRSCCLIPQTVSHRLRLYEREDHKGLMMELSEDCPSIQDRFHLSE
IRSLHVLEGCWVLYELPNYRGRQYLLRPQEYRRCQDWGAMDAKAGSLRRVVDLY
>sp|P07437|TBB5_HUMAN Tubulin beta chain OS=Homo sapiens OX=9606 GN=TUBB PE=1
SV=2
MREIVHIQAGQCGNQIGAKFWEVISDEHGIDPTGTYHGDSDLQLDRISVYYNEATGGKYV
PRAILVDLEPGTMDSVRSGPFGQIFRPDNFVFGQSGAGNNWAKGHYTEGAELVDSVLDVV
RKEAESCDCLQGFQLTHSLGGGTGSGMGTLLISKIREEYPDRIMNTFSVVPSPKVSDTVV
EPYNATLSVHQLVENTDETYCIDNEALYDICFRTLKLTTPTYGDLNHLVSATMSGVTTCL
RFPGQLNADLRKLAVNMVPFPRLHFFMPGFAPLTSRGSQQYRALTVPELTQQVFDAKNMM
AACDPRHGRYLTVAAVFRGRMSMKEVDEQMLNVQNKNSSYFVEWIPNNVKTAVCDIPPRG
LKMAVTFIGNSTAIQELFKRISEQFTAMFRRKAFLHWYTGEGMDEMEFTEAESNMNDLVS
EYQQYQDATAEEEEDFGEEAEEEA
>sp|P08174|DAF_HUMAN Complement decay-accelerating factor OS=Homo sapiens OX=9606
GN=CD55 PE=1 SV=4
MTVARPSVPAALPLLGELPRLLLLVLLCLPAVWGDCGLPPDVPNAQPALEGRTSFPEDTV
ITYKCEESFVKIPGEKDSVICLKGSQWSDIEEFCNRSCEVPTRLNSASLKQPYITQNYFP
VGTVVEYECRPGYRREPSLSPKLTCLQNLKWSTAVEFCKKKSCPNPGEIRNGQIDVPGGI
LFGATISFSCNTGYKLFGSTSSFCLISGSSVQWSDPLPECREIYCPAPPQIDNGIIQGER
DHYGYRQSVTYACNKGFTMIGEHSIYCTVNNDEGEWSGPPPECRGKSLTSKVPPTVQKPT
TVNVPTTEVSPTSQKTTTKTTTPNAQATRSTPVSRTTKHFHETTPNKGSGTTSGTTRLLS
GHTCFTLTGLLGTLVTMGLLT
>sp|P08238|HS90B_HUMAN Heat shock protein HSP 90-beta OS=Homo sapiens OX=9606
GN=HSP90AB1 PE=1 SV=4
MPEEVHHGEEEVETFAFQAEIAQLMSLIINTFYSNKEIFLRELISNASDALDKIRYESLT
DPSKLDSGKELKIDIIPNPQERTLTLVDTGIGMTKADLINNLGTIAKSGTKAFMEALQAG
ADISMIGQFGVGFYSAYLVAEKVVVITKHNDDEQYAWESSAGGSFTVRADHGEPIGRGTK
VILHLKEDQTEYLEERRVKEVVKKHSQFIGYPITLYLEKEREKEISDDEAEEEKGEKEEE
DKDDEEKPKIEDVGSDEEDDSGKDKKKKTKKIKEKYIDQEELNKTKPIWTRNPDDITQEE
YGEFYKSLTNDWEDHLAVKHFSVEGQLEFRALLFIPRRAPFDLFENKKKKNNIKLYVRRV
FIMDSCDELIPEYLNFIRGVVDSEDLPLNISREMLQQSKILKVIRKNIVKKCLELFSELA
EDKENYKKFYEAFSKNLKLGIHEDSTNRRRLSELLRYHTSQSGDEMTSLSEYVSRMKETQ
KSIYYITGESKEQVANSAFVERVRKRGFEVVYMTEPIDEYCVQQLKEFDGKSLVSVTKEG
LELPEDEEEKKKMEESKAKFENLCKLMKEILDKKVEKVTISNRLVSSPCCIVTSTYGWTA
NMERIMKAQALRDNSTMGYMMAKKHLEINPDHPIVETLRQKAEADKNDKAVKDLVVLLFE
TALLSSGFSLEDPQTHSNRIYRMIKLGLGIDEDEVAAEEPNAAVPDEIPPLEGDEDASRM
EEVD
>sp|P08670|VIME_HUMAN Vimentin OS=Homo sapiens OX=9606 GN=VIM PE=1 SV=4
MSTRSVSSSSYRRMFGGPGTASRPSSSRSYVTTSTRTYSLGSALRPSTSRSLYASSPGGV
YATRSSAVRLRSSVPGVRLLQDSVDFSLADAINTEFKNTRTNEKVELQELNDRFANYIDK
VRFLEQQNKILLAELEQLKGQGKSRLGDLYEEEMRELRRQVDQLTNDKARVEVERDNLAE
DIMRLREKLQEEMLQREEAENTLQSFRQDVDNASLARLDLERKVESLQEEIAFLKKLHEE
EIQELQAQIQEQHVQIDVDVSKPDLTAALRDVRQQYESVAAKNLQEAEEWYKSKFADLSE
AANRNNDALRQAKQESTEYRRQVQSLTCEVDALKGTNESLERQMREMEENFAVEAANYQD

TIGRLQDEIQNMKEEMARHLREYQDLLNVKMALDIEIATYRKLLEGEESRISLPLPNFSS
LNLRETNLDSLPLVDTHSKRTLLIKTVETRDGQVINETSQHHDDLE
>sp|P08865|RSSA_HUMAN Small ribosomal subunit protein uS2 OS=Homo sapiens OX=9606
GN=RPSA PE=1 SV=4
MSGALDVLQMKEEDVLKFLAAGTHLGGTNLDFQMEQYIYKRKSDGIYIINLKRTWEKLLL
AARAIVAIENPADVSVISSRNTGQRAVLKFAAATGATPIAGRFTPGTFTNQIQAAFREPR
LLVVTDPRADHQPLTEASYVNLPTIALCNTDSPLRYVDIAIPCNNKGAHSVGLMWWMLAR
EVLRMRGTISREHPWEVMPDLYFYRDPEEIEKEEQAAAEKAVTKEEFQGEWTAPAPEFTA
TQPEVADWSEGVQVPSVPIQQFPTEDWSAQPATEDWSAAPTAQATEWVGATTDWS
>sp|P09211|GSTP1_HUMAN Glutathione S-transferase P OS=Homo sapiens OX=9606
GN=GSTP1 PE=1 SV=2
MPPYTVVYFPVRGRCAALRMLLADQGQSWKEEVVTVETWQEGSLKASCLYGQLPKFQDGD
LTLYQSNTILRHLGRTLGLYGKDQQEAALVDMVNDGVEDLRCKYISLIYTNYEAGKDDYV
KALPGQLKPFETLLSQNQGGKTFIVGDQISFADYNLLDLLLIHEVLAPGCLDAFPLLSAY
VGRLSARPKLKAFLASPEYVNLPINGNGKQ
>sp|P0C0L4|CO4A_HUMAN Complement C4-A OS=Homo sapiens OX=9606 GN=C4A PE=1 SV=2
MRLLWGLIWASSFFTLSLQKPRLLLFSPSVVHLGVPLSVGVQLQDVPRGQVVKGSVFLRN
PSRNNVPCSPKVDFTLSSERDFALLSLQVPLKDAKSCGLHQLLRGPEVQLVAHSPWLKDS
LSRTTNIQGINLLFSSRRGHLFLQTDQPIYNPGQRVRYRVFALDQKMRPSTDTITVMVEN
SHGLRVRKKEVYMPSSIFQDDFVIPDISEPGTWKISARFSDGLESNSSTQFEVKKYVLPN
FEVKITPGKPYILTVPGHLDEMQLDIQARYIYGKPVQGVAYVRFGLLDEDGKKTFFRGLE
SQTKLVNGQSHISLSKAEFQDALEKLNMGITDLQGLRLYVAAAIIESPGGEMEEAELTSW
YFVSSPFSLDLSKTKRHLVPGAPFLLQALVREMSGSPASGIPVKVSATVSSPGSVPEVQD
IQQNTDGSGQVSIPIIIPQTISELQLSVSAGSPHPAIARLTVAAPPSGGPGFLSIERPDS
RPPRVGDTLNLNLRAVGSGATFSHYYYMILSRGQIVFMNREPKRTLTSVSVFVDHHLAPS
FYFVAFYYHGDHPVANSLRVDVQAGACEGKLELSVDGAKQYRNGESVKLHLETDSLALVA
LGALDTALYAAGSKSHKPLNMGKVFEAMNSYDLGCGPGGGDSALQVFQAAGLAFSDGDQW
TLSRKRLSCPKEKTTRKKRNVNFQKAINEKLGQYASPTAKRCCQDGVTRLPMMRSCEQRA
ARVQQPDCREPFLSCCQFAESLRKKSRDKGQAGLQRALEILQEEDLIDEDDIPVRSFFPE
NWLWRVETVDRFQILTLWLPDSLTTWEIHGLSLSKTKGLCVATPVQLRVFREFHLHLRLP
MSVRRFEQLELRPVLYNYLDKNLTVSVHVSPVEGLCLAGGGGLAQQVLVPAGSARPVAFS
VVPTAAAAVSLKVVARGSFEFPVGDAVSKVLQIEKEGAIHREELVYELNPLDHRGRTLEI
PGNSDPNMIPDGDFNSYVRVTASDPLDTLGSEGALSPGGVASLLRLPRGCGEQTMIYLAP
TLAASRYLDKTEQWSTLPPETKDHAVDLIQKGYMRIQQFRKADGSYAAWLSRDSSTWLTA
FVLKVLSLAQEQVGGSPEKLQETSNWLLSQQQADGSFQDPCPVLDRSMQGGLVGNDETVA
LTAFVTIALHHGLAVFQDEGAEPLKQRVEASISKANSFLGEKASAGLLGAHAAAITAYAL
TLTKAPVDLLGVAHNNLMAMAQETGDNLYWGSVTGSQSNAVSPTPAPRNPSDPMPQAPAL
WIETTAYALLHLLLHEGKAEMADQASAWLTRQGSFQGGFRSTQDTVIALDALSAYWIASH
TTEERGLNVTLSSTGRNGFKSHALQLNNRQIRGLEEELQFSLGSKINVKVGGNSKGTLKV
LRTYNVLDMKNTTCQDLQIEVTVKGHVEYTMEANEDYEDYEYDELPAKDDPDAPLQPVTP
LQLFEGRRNRRRREAPKVVEEQESRVHYTVCIWRNGKVGLSGMAIADVTLLSGFHALRAD
LEKLTSLSDRYVSHFETEGPHVLLYFDSVPTSRECVGFEAVQEVPVGLVQPASATLYDYY
NPERRCSVFYGAPSKSRLLATLCSAEVCQCAEGKCPRQRRALERGLQDEDGYRMKFACYY
PRVEYGFQVKVLREDSRAAFRLFETKITQVLHFTKDVKAAANQMRNFLVRASCRLRLEPG

KEYLIMGLDGATYDLEGHPQYLLDSNSWIEEMPSERLCRSTRQRAACAQLNDFLQEYGTQ
GCQV
>sp|P0CG47|UBB_HUMAN Polyubiquitin-B OS=Homo sapiens OX=9606 GN=UBB PE=1 SV=1
MQIFVKTLTGKTITLEVEPSDTIENVKAKIQDKEGIPPDQQRLIFAGKQLEDGRTLSDYN
IQKESTLHLVLRLRGGMQIFVKTLTGKTITLEVEPSDTIENVKAKIQDKEGIPPDQQRLI
FAGKQLEDGRTLSDYNIQKESTLHLVLRLRGGMQIFVKTLTGKTITLEVEPSDTIENVKA
KIQDKEGIPPDQQRLIFAGKQLEDGRTLSDYNIQKESTLHLVLRLRGGC
>sp|P0DMV8|HS71A_HUMAN Heat shock 70 kDa protein 1A OS=Homo sapiens OX=9606
GN=HSPA1A PE=1 SV=1
MAKAAAIGIDLGTTYSCVGVFQHGKVEIIANDQGNRTTPSYVAFTDTERLIGDAAKNQVA
LNPQNTVFDAKRLIGRKFGDPVVQSDMKHWPFQVINDGDKPKVQVSYKGETKAFYPEEIS
SMVLTKMKEIAEAYLGYPVTNAVITVPAYFNDSQRQATKDAGVIAGLNVLRIINEPTAAA
IAYGLDRTGKGERNVLIFDLGGGTFDVSILTIDDGIFEVKATAGDTHLGGEDFDNRLVNH
FVEEFKRKHKKDISQNKRAVRRLRTACERAKRTLSSSTQASLEIDSLFEGIDFYTSITRA
RFEELCSDLFRSTLEPVEKALRDAKLDKAQIHDLVLVGGSTRIPKVQKLLQDFFNGRDLN
KSINPDEAVAYGAAVQAAILMGDKSENVQDLLLLDVAPLSLGLETAGGVMTALIKRNSTI
PTKQTQIFTTYSDNQPGVLIQVYEGERAMTKDNNLLGRFELSGIPPAPRGVPQIEVTFDI
DANGILNVTATDKSTGKANKITITNDKGRLSKEEIERMVQEAEKYKAEDEVQRERVSAKN
ALESYAFNMKSAVEDEGLKGKISEADKKKVLDKCQEVISWLDANTLAEKDEFEHKRKELE
QVCNPIISGLYQGAGGPGPGGFGAQGPKGGSGSGPTIEEVD
>sp|P10412|H14_HUMAN Histone H1.4 OS=Homo sapiens OX=9606 GN=H1-4 PE=1 SV=2
MSETAPAAPAAPAPAEKTPVKKKARKSAGAAKRKASGPPVSELITKAVAASKERSGVSLA
ALKKALAAAGYDVEKNNSRIKLGLKSLVSKGTLVQTKGTGASGSFKLNKKAASGEAKPKA
KKAGAAKAKKPAGAAKKPKKATGAATPKKSAKKTPKKAKKPAAAAGAKKAKSPKKAKAAK
PKKAPKSPAKAKAVKPKAAKPKTAKPKAAKPKKAAAKKK
>sp|P10745|RET3_HUMAN Retinol-binding protein 3 OS=Homo sapiens OX=9606 GN=RBP3
PE=1 SV=2
MMREWVLLMSVLLCGLAGPTHLFQPSLVLDMAKVLLDNYCFPENLLGMQEAIQQAIKSHE
ILSISDPQTLASVLTAGVQSSLNDPRLVISYEPSTPEPPPQVPALTSLSEEELLAWLQRG
LRHEVLEGNVGYLRVDSVPGQEVLSMMGEFLVAHVWGNLMGTSALVLDLRHCTGGQVSGI
PYIISYLHPGNTILHVDTIYNRPSNTTTEIWTLPQVLGERYGADKDVVVLTSSQTRGVAE
DIAHILKQMRRAIVVGERTGGGALDLRKLRIGESDFFFTVPVSRSLGPLGGGSQTWEGSG
VLPCVGTPAEQALEKALAILTLRSALPGVVHCLQEVLKDYYTLVDRVPTLLQHLASMDFS
TVVSEEDLVTKLNAGLQAASEDPRLLVRAIGPTETPSWPAPDAAAEDSPGVAPELPEDEA
IRQALVDSVFQVSVLPGNVGYLRFDSFADASVLGVLAPYVLRQVWEPLQDTEHLIMDLRH
NPGGPSSAVPLLLSYFQGPEAGPVHLFTTYDRRTNITQEHFSHMELPGPRYSTQRGVYLL
TSHRTATAAEEFAFLMQSLGWATLVGEITAGNLLHTRTVPLLDTPEGSLALTVPVLTFID
NHGEAWLGGGVVPDAIVLAEEALDKAQEVLEFHQSLGALVEGTGHLLEAHYARPEVVGQT
SALLRAKLAQGAYRTAVDLESLASQLTADLQEVSGDHRLLVFHSPGELVVEEAPPPPPAV
PSPEELTYLIEALFKTEVLPGQLGYLRFDAMAELETVKAVGPQLVRLVWQQLVDTAALVI
DLRYNPGSYSTAIPLLCSYFFEAEPRQHLYSVFDRATSKVTEVWTLPQVAGQRYGSHKDL
YILMSHTSGSAAEAFAHTMQDLQRATVIGEPTAGGALSVGIYQVGSSPLYASMPTQMAMS
ATTGKAWDLAGVEPDITVPMSEALSIAQDIVALRAKVPTVLQTAGKLVADNYASAELGAK
MATKLSGLQSRYSRVTSEVALAEILGADLQMLSGDPHLKAAHIPENAKDRIPGIVPMQIP

SPEVFEELIKFSFHTNVLEDNIGYLRFDMFGDGELLTQVSRLLVEHIWKKIMHTDAMIID
MRFNIGGPTSSIPILCSYFFDEGPPVLLDKIYSRPDDSVSELWTHAQVVGERYGSKKSMV
ILTSSVTAGTAEEFTYIMKRLGRALVIGEVTSGGCQPPQTYHVDDTNLYLTIPTARSVGA
SDGSSWEGVGVTPHVVVPAEEALARAKEMLQHNQLRVKRSPGLQDHL
>sp|P10909|CLUS_HUMAN Clusterin OS=Homo sapiens OX=9606 GN=CLU PE=1 SV=1
MMKTLLLFVGLLLTWESGQVLGDQTVSDNELQEMSNQGSKYVNKEIQNAVNGVKQIKTLI
EKTNEERKTLLSNLEEAKKKKEDALNETRESETKLKELPGVCNETMMALWEECKPCLKQT
CMKFYARVCRSGSGLVGRQLEEFLNQSSPFYFWMNGDRIDSLLENDRQQTHMLDVMQDHF
SRASSIIDELFQDRFFTREPQDTYHYLPFSLPHRRPHFFFPKSRIVRSLMPFSPYEPLNF
HAMFQPFLEMIHEAQQAMDIHFHSPAFQHPPTEFIREGDDDRTVCREIRHNSTGCLRMKD
QCDKCREILSVDCSTNNPSQAKLRRELDESLQVAERLTRKYNELLKSYQWKMLNTSSLLE
QLNEQFNWVSRLANLTQGEDQYYLRVTTVASHTSDSDVPSGVTEVVVKLFDSDPITVTVP
VEVSRKNPKFMETVAEKALQEYRKKHREE
>sp|P11021|BIP_HUMAN Endoplasmic reticulum chaperone BiP OS=Homo sapiens OX=9606
GN=HSPA5 PE=1 SV=2
MKLSLVAAMLLLLSAARAEEEDKKEDVGTVVGIDLGTTYSCVGVFKNGRVEIIANDQGNR
ITPSYVAFTPEGERLIGDAAKNQLTSNPENTVFDAKRLIGRTWNDPSVQQDIKFLPFKVV
EKKTKPYIQVDIGGGQTKTFAPEEISAMVLTKMKETAEAYLGKKVTHAVVTVPAYFNDAQ
RQATKDAGTIAGLNVMRIINEPTAAAIAYGLDKREGEKNILVFDLGGGTFDVSLLTIDNG
VFEVVATNGDTHLGGEDFDQRVMEHFIKLYKKKTGKDVRKDNRAVQKLRREVEKAKRALS
SQHQARIEIESFYEGEDFSETLTRAKFEELNMDLFRSTMKPVQKVLEDSDLKKSDIDEIV
LVGGSTRIPKIQQLVKEFFNGKEPSRGINPDEAVAYGAAVQAGVLSGDQDTGDLVLLDVC
PLTLGIETVGGVMTKLIPRNTVVPTKKSQIFSTASDNQPTVTIKVYEGERPLTKDNHLLG
TFDLTGIPPAPRGVPQIEVTFEIDVNGILRVTAEDKGTGNKNKITITNDQNRLTPEEIER
MVNDAEKFAEEDKKLKERIDTRNELESYAYSLKNQIGDKEKLGGKLSSEDKETMEKAVEE
KIEWLESHQDADIEDFKAKKKELEEIVQPIISKLYGSAGPPPTGEEDTAEKDEL
>sp|P11047|LAMC1_HUMAN Laminin subunit gamma-1 OS=Homo sapiens OX=9606
GN=LAMC1 PE=1 SV=3
MRGSHRAAPALRPRGRLWPVLAVLAAAAAAGCAQAAMDECTDEGGRPQRCMPEFVNAAFN
VTVVATNTCGTPPEEYCVQTGVTGVTKSCHLCDAGQPHLQHGAAFLTDYNNQADTTWWQS
QTMLAGVQYPSSINLTLHLGKAFDITYVRLKFHTSRPESFAIYKRTREDGPWIPYQYYSG
SCENTYSKANRGFIRTGGDEQQALCTDEFSDISPLTGGNVAFSTLEGRPSAYNFDNSPVL
QEWVTATDIRVTLNRLNTFGDEVFNDPKVLKSYYYAISDFAVGGRCKCNGHASECMKNEF
DKLVCNCKHNTYGVDCEKCLPFFNDRPWRRATAESASECLPCDCNGRSQECYFDPELYRS
TGHGGHCTNCQDNTDGAHCERCRENFFRLGNNEACSSCHCSPVGSLSTQCDSYGRCSCKP
GVMGDKCDRCQPGFHSLTEAGCRPCSCDPSGSIDECNIETGRCVCKDNVEGFNCERCKPG
FFNLESSNPRGCTPCFCFGHSSVCTNAVGYSVYSISSTFQIDEDGWRAEQRDGSEASLEW
SSERQDIAVISDSYFPRYFIAPAKFLGKQVLSYGQNLSFSFRVDRRDTRLSAEDLVLEGA
GLRVSVPLIAQGNSYPSETTVKYVFRLHEATDYPWRPALTPFEFQKLLNNLTSIKIRGTY
SERSAGYLDDVTLASARPGPGVPATWVESCTCPVGYGGQFCEMCLSGYRRETPNLGPYSP
CVLCACNGHSETCDPETGVCNCRDNTAGPHCEKCSDGYYGDSTAGTSSDCQPCPCPGGSS
CAVVPKTKEVVCTNCPTGTTGKRCELCDDGYFGDPLGRNGPVRLCRLCQCSDNIDPNAVG
NCNRLTGECLKCIYNTAGFYCDRCKDGFFGNPLAPNPADKCKACNCNLYGTMKQQSSCNP
VTGQCECLPHVTGQDCGACDPGFYNLQSGQGCERCDCHALGSTNGQCDIRTGQCECQPGI

TGQHCERCEVNHFGFGPEGCKPCDCHPEGSLSLQCKDDGRCECREGFVGNRCDQCEENYF
YNRSWPGCQECPACYRLVKDKVADHRVKLQELESLIANLGTGDEMVTDQAFEDRLKEAER
EVMDLLREAQDVKDVDQNLMDRLQRVNNTLSSQISRLQNIRNTIEETGNLAEQARAHVEN
TERLIEIASRELEKAKVAAANVSVTQPESTGDPNNMTLLAEEARKLAERHKQEADDIVRV
AKTANDTSTEAYNLLLRTLAGENQTAFEIEELNRKYEQAKNISQDLEKQAARVHEEAKRA
GDKAVEIYASVAQLSPLDSETLENEANNIKMEAENLEQLIDQKLKDYEDLREDMRGKELE
VKNLLEKGKTEQQTADQLLARADAAKALAEEAAKKGRDTLQEANDILNNLKDFDRRVNDN
KTAAEEALRKIPAINQTITEANEKTREAQQALGSAAADATEAKNKAHEAERIASAVQKNA
TSTKAEAERTFAEVTDLDNEVNNMLKQLQEAEKELKRKQDDADQDMMMAGMASQAAQEAE
INARKAKNSVTSLLSIINDLLEQLGQLDTVDLNKLNEIEGTLNKAKDEMKVSDLDRKVSD
LENEAKKQEAAIMDYNRDIEEIMKDIRNLEDIRKTLPSGCFNTPSIEKP
>sp|P11142|HSP7C_HUMAN Heat shock cognate 71 kDa protein OS=Homo sapiens OX=9606 GN=HSPA8 PE=1 SV=1
MSKGPAVGIDLGTTYSCVGVFQHGKVEIIANDQGNRTTPSYVAFTDTERLIGDAAKNQVA
MNPTNTVFDAKRLIGRRFDDAVVQSDMKHWPFMVVNDAGRPKVQVEYKGETKSFYPEEVS
SMVLTKMKEIAEAYLGKTVTNAVVTVPAYFNDSQRQATKDAGTIAGLNVLRIINEPTAAA
IAYGLDKKVGAERNVLIFDLGGGTFDVSILTIEDGIFEVKSTAGDTHLGGEDFDNRMVNH
FIAEFKRKHKKDISENKRAVRRLRTACERAKRTLSSSTQASIEIDSLYEGIDFYTSITRA
RFEELNADLFRGTLDPVEKALRDAKLDKSQIHDIVLVGGSTRIPKIQKLLQDFFNGKELN
KSINPDEAVAYGAAVQAAILSGDKSENVQDLLLLDVTPLSLGIETAGGVMTVLIKRNTTI
PTKQTQTFTTYSDNQPGVLIQVYEGERAMTKDNNLLGKFELTGIPPAPRGVPQIEVTFDI
DANGILNVSAVDKSTGKENKITITNDKGRLSKEDIERMVQEAEKYKAEDEKQRDKVSSKN
SLESYAFNMKATVEDEKLQGKINDEDKQKILDKCNEIINWLDKNQTAEKEEFEHQQKELE
KVCNPIITKLYQSAGGMPGGMPGGFPGGGAPPSGGASSGPTIEEVD
>sp|P11150|LIPC_HUMAN Hepatic triacylglycerol lipase OS=Homo sapiens OX=9606 GN=LIPC PE=1 SV=3
MDTSPLCFSILLVLCIFIQSSALGQSLKPEPFGRRAQAVETNKTLHEMKTRFLLFGETNQ
GCQIRINHPDTLQECGFNSSLPLVMIIHGWSVDGVLENWIWQMVAALKSQPAQPVNVGLV
DWITLAHDHYTIAVRNTRLVGKEVAALLRWLEESVQLSRSHVHLIGYSLGAHVSGFAGSS
IGGTHKIGRITGLDAAGPLFEGSAPSNRLSPDDANFVDAIHTFTREHMGLSVGIKQPIGH
YDFYPNGGSFQPGCHFLELYRHIAQHGFNAITQTIKCSHERSVHLFIDSLLHAGTQSMAY
PCGDMNSFSQGLCLSCKKGRCNTLGYHVRQEPRSKSKRLFLVTRAQSPFKVYHYQFKIQF
INQTETPIQTTFTMSLLGTKEKMQKIPITLGKGIASNKTYSFLITLDVDIGELIMIKFKW
ENSAVWANVWDTVQTIIPWSTGPRHSGLVLKTIRVKAGETQQRMTFCSENTDDLLLRPTQ
EKIFVKCEIKSKTSKRKIR
>sp|P12259|FA5_HUMAN Coagulation factor V OS=Homo sapiens OX=9606 GN=F5 PE=1 SV=4
MFPGCPRLWVLVVLGTSWVGWGSQGTEAAQLRQFYVAAQGISWSYRPEPTNSSLNLSVTS
FKKIVYREYEPYFKKEKPQSTISGLLGPTLYAEVGDIIKVHFKNKADKPLSIHPQGIRYS
KLSEGASYLDHTFPAEKMDDAVAPGREYTYEWSISEDSGPTHDDPPCLTHIYYSHENLIE
DFNSGLIGPLLICKKGTLTEGGTQKTFDKQIVLLFAVFDESKSWSQSSSLMYTVNGYVNG
TMPDITVCAHDHISWHLLGMSSGPELFSIHFNGQVLEQNHHKVSAITLVSATSTTANMTV
GPEGKWIISSLTPKHLQAGMQAYIDIKNCPKKTRNLKKITREQRRHMKRWEYFIAAEEVI
WDYAPVIPANMDKKYRSQHLDNFSNQIGKHYKKVMYTQYEDESFTKHTVNPNMKEDGILG
PIIRAQVRDTLKIVFKNMASRPYSIYPHGVTFSPYEDEVNSSFTSGRNNTMIRAVQPGET

YTYKWNILEFDEPTENDAQCLTRPYYSDVDIMRDIASGLIGLLLICKSRSLDRRGIQRAA
DIEQQAVFAVFDENKSWYLEDNINKFCENPDEVKRDDPKFYESNIMSTINGYVPESITTL
GFCFDDTVQWHFCSVGTQNEILTIHFTGHSFIYGKRHEDTLTLFPMRGESVTVTMDNVGT
WMLTSMNSSPRSKKLRLKFRDVKCIPDDDEDSYEIFEPPESTVMATRKMHDRLEPEDEES
DADYDYQNRLAAALGIRSFRNSSLNQEEEEFNLTALALENGTEFVSSNTDIIVGSNYSSP
SNISKFTVNNLAEPQKAPSHQQATTAGSPLRHLIGKNSVLNSSTAEHSSPYSEDPIEDPL
QPDVTGIRLLSLGAGEFKSQEHAKHKGPKVERDQAAKHRFSWMKLLAHKVGRHLSQDTGS
PSGMRPWEDLPSQDTGSPSRMRPWKDPPSDLLLLKQSNSSKILVGRWHLASEKGSYEIIQ
DTDEDTAVNNWLISPQNASRAWGESTPLANKPGKQSGHPKFPRVRHKSLQVRQDGGKSRL
KKSQFLIKTRKKKKEKHTHHAPLSPRTFHPLRSEAYNTFSERRLKHSLVLHKSNETSLPT
DLNQTLPSMDFGWIASLPDHNQNSSNDTGQASCPPGLYQTVPPEEHYQTFPIQDPDQMHS
TSDPSHRSSSPELSEMLEYDRSHKSFPTDISQMSPSSEHEVWQTVISPDLSQVTLSPELS
QTNLSPDLSHTTLSPELIQRNLSPALGQMPISPDLSHTTLSPDLSHTTLSLDLSQTNLSP
ELSQTNLSPALGQMPLSPDLSHTTLSLDFSQTNLSPELSHMTLSPELSQTNLSPALGQMP
ISPDLSHTTLSLDFSQTNLSPELSQTNLSPALGQMPLSPDPSHTTLSLDLSQTNLSPELS
QTNLSPDLSEMPLFADLSQIPLTPDLDQMTLSPDLGETDLSPNFGQMSLSPDLSQVTLSP
DISDTTLLPDLSQISPPPDLDQIFYPSESSQSLLLQEFNESFPYPDLGQMPSPSSPTLND
TFLSKEFNPLVIVGLSKDGTDYIEIIPKEEVQSSEDDYAEIDYVPYDDPYKTDVRTNINS
SRDPDNIAAWYLRSNNGNRRNYYIAAEEISWDYSEFVQRETDIEDSDDIPEDTTYKKVVF
RKYLDSTFTKRDPRGEYEEHLGILGPIIRAEVDDVIQVRFKNLASRPYSLHAHGLSYEKS
SEGKTYEDDSPEWFKEDNAVQPNSSYTYVWHATERSGPESPGSACRAWAYYSAVNPEKDI
HSGLIGPLLICQKGILHKDSNMPMDMREFVLLFMTFDEKKSWYYEKKSRSSWRLTSSEMK
KSHEFHAINGMIYSLPGLKMYEQEWVRLHLLNIGGSQDIHVVHFHGQTLLENGNKQHQLG
VWPLLPGSFKTLEMKASKPGWWLLNTEVGENQRAGMQTPFLIMDRDCRMPMGLSTGIISD
SQIKASEFLGYWEPRLARLNNGGSYNAWSVEKLAAEFASKPWIQVDMQKEVIITGIQTQG
AKHYLKSCYTTEFYVAYSSNQINWQIFKGNSTRNVMYFNGNSDASTIKENQFDPPIVARY
IRISPTRAYNRPTLRLELQGCEVNGCSTPLGMENGKIENKQITASSFKKSWWGDYWEPFR
ARLNAQGRVNAWQAKANNNKQWLEIDLLKIKKITAIITQGCKSLSSEMYVKSYTIHYSEQ
GVEWKPYRLKSSMVDKIFEGNTNTKGHVKNFFNPPIISRFIRVIPKTWNQSIALRLELFG
CDIY
>sp|P13533|MYH6_HUMAN Myosin-6 OS=Homo sapiens OX=9606 GN=MYH6 PE=1 SV=5
MTDAQMADFGAAAQYLRKSEKERLEAQTRPFDIRTECFVPDDKEEFVKAKILSREGGKVI
AETENGKTVTVKEDQVLQQNPPKFDKIEDMAMLTFLHEPAVLFNLKERYAAWMIYTYSGL
FCVTVNPYKWLPVYNAEVVAAYRGKKRSEAPPHIFSISDNAYQYMLTDRENQSILITGES
GAGKTVNTKRVIQYFASIAAIGDRGKKDNANANKGTLEDQIIQANPALEAFGNAKTVRND
NSSRFGKFIRIHFGATGKLASADIETYLLEKSRVIFQLKAERNYHIFYQILSNKKPELLD
MLLVTNNPYDYAFVSQGEVSVASIDDSEELMATDSAFDVLGFTSEEKAGVYKLTGAIMHY
GNMKFKQKQREEQAEPDGTEDADKSAYLMGLNSADLLKGLCHPRVKVGNEYVTKGQSVQQ
VYYSIGALAKAVYEKMFNWMVTRINATLETKQPRQYFIGVLDIAGFEIFDFNSFEQLCIN
FTNEKLQQFFNHHMFVLEQEEYKKEGIEWTFIDFGMDLQACIDLIEKPMGIMSILEEECM
FPKATDMTFKAKLYDNHLGKSNNFQKPRNIKGKQEAHFSLIHYAGTVDYNILGWLEKNKD
PLNETVVALYQKSSLKLMATLFSSYATADTGDSGKSKGGKKKGSSFQTVSALHRENLNKL
MTNLRTTHPHFVRCIIPNERKAPGVMDNPLVMHQLRCNGVLEGIRICRKGFPNRILYGDF
RQRYRILNPVAIPEGQFIDSRKGTEKLLSSLDIDHNQYKFGHTKVFFKAGLLGLLEEMRD

ERLSRIITRMQAQARGQLMRIEFKKIVERRDALLVIQWNIRAFMGVKNWPWMKLYFKIKP
LLKSAETEKEMATMKEEFGRIKETLEKSEARRKELEEKMVSLLQEKNDLQLQVQAEQDNL
NDAEERCDQLIKNKIQLEAKVKEMNERLEDEEEMNAELTAKKRKLEDECSELKKDIDDLE
LTLAKVEKEKHATENKVKNLTEEMAGLDEIIAKLTKEKKALQEAHQQALDDLQVEEDKVN
SLSKSKVKLEQQVDDLEGSLEQEKKVRMDLERAKRKLEGDLKLTQESIMDLENDKLQLEE
KLKKKEFDINQQNSKIEDEQVLALQLQKKLKENQARIEELEEELEAERTARAKVEKLRSD
LSRELEEISERLEEAGGATSVQIEMNKKREAEFQKMRRDLEEATLQHEATAAALRKKHAD
SVAELGEQIDNLQRVKQKLEKEKSEFKLELDDVTSNMEQIIKAKANLEKVSRTLEDQANE
YRVKLEEAQRSLNDFTTQRAKLQTENGELARQLEEKEALISQLTRGKLSYTQQMEDLKRQ
LEEEGKAKNALAHALQSARHDCDLLREQYEEETEAKAELQRVLSKANSEVAQWRTKYETD
AIQRTEELEEAKKKLAQRLQDAEEAVEAVNAKCSSLEKTKHRLQNEIEDLMVDVERSNAA
AAALDKKQRNFDKILAEWKQKYEESQSELESSQKEARSLSTELFKLKNAYEESLEHLETF
KRENKNLQEEISDLTEQLGEGGKNVHELEKVRKQLEVEKLELQSALEEAEASLEHEEGKI
LRAQLEFNQIKAEIERKLAEKDEEMEQAKRNHQRVVDSLQTSLDAETRSRNEVLRVKKKM
EGDLNEMEIQLSHANRMAAEAQKQVKSLQSLLKDTQIQLDDAVRANDDLKENIAIVERRN
NLLQAELEELRAVVEQTERSRKLAEQELIETSERVQLLHSQNTSLINQKKKMESDLTQLQ
SEVEEAVQECRNAEEKAKKAITDAAMMAEELKKEQDTSAHLERMKKNMEQTIKDLQHRLD
EAEQIALKGGKKQLQKLEARVRELEGELEAEQKRNAESVKGMRKSERRIKELTYQTEEDK
KNLLRLQDLVDKLQLKVKAYKRQAEEAEEQANTNLSKFRKVQHELDEAEERADIAESQVN
KLRAKSRDIGAKQKMHDEE
>sp|P13639|EF2_HUMAN Elongation factor 2 OS=Homo sapiens OX=9606 GN=EEF2 PE=1 SV=4
MVNFTVDQIRAIMDKKANIRNMSVIAHVDHGKSTLTDSLVCKAGIIASARAGETRFTDTR
KDEQERCITIKSTAISLFYELSENDLNFIKQSKDGAGFLINLIDSPGHVDFSSEVTAALR
VTDGALVVVDCVSGVCVQTETVLRQAIAERIKPVLMMNKMDRALLELQLEPEELYQTFQR
IVENVNVIISTYGEGESGPMGNIMIDPVLGTVGFGSGLHGWAFTLKQFAEMYVAKFAAKG
EGQLGPAERAKKVEDMMKKLWGDRYFDPANGKFSKSATSPEGKKLPRTFCQLILDPIFKV
FDAIMNFKKEETAKLIEKLDIKLDSEDKDKEGKPLLKAVMRRWLPAGDALLQMITIHLPS
PVTAQKYRCELLYEGPPDDEAAMGIKSCDPKGPLMMYISKMVPTSDKGRFYAFGRVFSGL
VSTGLKVRIMGPNYTPGKKEDLYLKPIQRTILMMGRYVEPIEDVPCGNIVGLVGVDQFLV
KTGTITTFEHAHNMRVMKFSVSPVVRVAVEAKNPADLPKLVEGLKRLAKSDPMVQCIIEE
SGEHIIAGAGELHLEICLKDLEEDHACIPIKKSDPVVSYRETVSEESNVLCLSKSPNKHN
RLYMKARPFPDGLAEDIDKGEVSARQELKQRARYLAEKYEWDVAEARKIWCFGPDGTGPN
ILTDITKGVQYLNEIKDSVVAGFQWATKEGALCEENMRGVRFDVHDVTLHADAIHRGGGQ
IIPTARRCLYASVLTAQPRLMEPIYLVEIQCPEQVVGGIYGVLNRKRGHVFEESQVAGTP
MFVVKAYLPVNESFGFTADLRSNTGGQAFPQCVFDHWQILPGDPFDNSSRPSQVVAETRK
RKGLKEGIPALDNFLDKL
>sp|P13645|K1C10_HUMAN Keratin, type I cytoskeletal 10 OS=Homo sapiens OX=9606
GN=KRT10 PE=1 SV=6
MSVRYSSSKHYSSSRSGGGGGGGGCGGGGGVSSLRISSSKGSLGGGFSSGGFSGGSFSRG
SSGGGCFGGSSGGYGGLGGFGGGSFRGSYGSSSFGGSYGGIFGGGSFGGGSFGGGSFGGG
GFGGGGFGGGFGGGFGGDGGLLSGNEKVTMQNLNDRLASYLDKVRALEESNYELEGKIKE
WYEKHGNSHQGEPRDYSKYYKTIDDLKNQILNLTTDNANILLQIDNARLAADDFRLKYEN
EVALRQSVEADINGLRRVLDELTLTKADLEMQIESLTEELAYLKKNHEEEMKDLRNVSTG
DVNVEMNAAPGVDLTQLLNNMRSQYEQLAEQNRKDAEAWFNEKSKELTTEIDNNIEQISS

YKSEITELRRNVQALEIELQSQLALKQSLEASLAETEGRYCVQLSQIQAQISALEEQLQQ
IRAETECQNTEYQQLLDIKIRLENEIQTYRSLLEGEGSSGGGGRGGGSFGGGYGGGSSGG
GSSGGGHGGGHGGSSGGGYGGGSSGGGSSGGGYGGGSSSGGHGGSSSGGYGGGSSGGGGG
GYGGGSSGGGSSSGGGYGGGSSSGGHKSSSSGSVGESSSKGPRY
>sp|P13646|K1C13_HUMAN Keratin, type I cytoskeletal 13 OS=Homo sapiens OX=9606
GN=KRT13 PE=1 SV=4
MSLRLQSSSASYGGGFGGGSCQLGGGRGVSTCSTRFVSGGSAGGYGGGVSCGFGGGAGSG
FGGGYGGGLGGGYGGGLGGGFGGGFAGGFVDFGACDGGLLTGNEKITMQNLNDRLASYLE
KVRALEEANADLEVKIRDWHLKQSPASPERDYSPYYKTIEELRDKILTATIENNRVILEI
DNARLAADDFRLKYENELALRQSVEADINGLRRVLDELTLSKTDLEMQIESLNEELAYMK
KNHEEEMKEFSNQVVGQVNVEMDATPGIDLTRVLAEMREQYEAMAERNRRDAEEWFHTKS
AELNKEVSTNTAMIQTSKTEITELRRTLQGLEIELQSQLSMKAGLENTVAETECRYALQL
QQIQGLISSIEAQLSELRSEMECQNQEYKMLLDIKTRLEQEIATYRSLLEGQDAKMIGFP
SSAGSVSPRSTSVTTTSSASVTTTSNASGRRTSDVRRP
>sp|P13647|K2C5_HUMAN Keratin, type II cytoskeletal 5 OS=Homo sapiens OX=9606 GN=KRT5
PE=1 SV=3
MSRQSSVSFRSGGSRSFSTASAITPSVSRTSFTSVSRSGGGGGGGFGRVSLAGACGVGGY
GSRSLYNLGGSKRISISTSGGSFRNRFGAGAGGGYGFGGGAGSGFGFGGGAGGGFGLGGG
AGFGGGFGGPGFPVCPPGGIQEVTVNQSLLTPLNLQIDPSIQRVRTEEREQIKTLNNKFA
SFIDKVRFLEQQNKVLDTKWTLLQEQGTKTVRQNLEPLFEQYINNLRRQLDSIVGERGRL
DSELRNMQDLVEDFKNKYEDEINKRTTAENEFVMLKKDVDAAYMNKVELEAKVDALMDEI
NFMKMFFDAELSQMQTHVSDTSVVLSMDNNRNLDLDSIIAEVKAQYEEIANRSRTEAESW
YQTKYEELQQTAGRHGDDLRNTKHEISEMNRMIQRLRAEIDNVKKQCANLQNAIADAEQR
GELALKDARNKLAELEEALQKAKQDMARLLREYQELMNTKLALDVEIATYRKLLEGEECR
LSGEGVGPVNISVVTSSVSSGYGSGSGYGGGLGGGLGGGLGGGLAGGSSGSYYSSSSGGV
GLGGGLSVGGSGFSASSGRGLGVGFGSGGGSSSSVKFVSTTSSSRKSFKS
>sp|P13929|ENOB_HUMAN Beta-enolase OS=Homo sapiens OX=9606 GN=ENO3 PE=1 SV=5
MAMQKIFAREILDSRGNPTVEVDLHTAKGRFRAAVPSGASTGIYEALELRDGDKGRYLGK
GVLKAVENINNTLGPALLQKKLSVVDQEKVDKFMIELDGTENKSKFGANAILGVSLAVCK
AGAAEKGVPLYRHIADLAGNPDLILPVPAFNVINGGSHAGNKLAMQEFMILPVGASSFKE
AMRIGAEVYHHLKGVIKAKYGKDATNVGDEGGFAPNILENNEALELLKTAIQAAGYPDKV
VIGMDVAASEFYRNGKYDLDFKSPDDPARHITGEKLGELYKSFIKNYPVVSIEDPFDQDD
WATWTSFLSGVNIQIVGDDLTVTNPKRIAQAVEKKACNCLLLKVNQIGSVTESIQACKLA
QSNGWGVMVSHRSGETEDTFIADLVVGLCTGQIKTGAPCRSERLAKYNQLMRIEEALGDK
AIFAGRKFRNPKAK
>sp|P14555|PA2GA_HUMAN Phospholipase A2, membrane associated OS=Homo sapiens
OX=9606 GN=PLA2G2A PE=1 SV=2
MKTLLLLAVIMIFGLLQAHGNLVNFHRMIKLTTGKEAALSYGFYGCHCGVGGRGSPKDAT
DRCCVTHDCCYKRLEKRGCGTKFLSYKFSNSGSRITCAKQDSCRSQLCECDKAAATCFAR
NKTTYNKKYQYYSNKHCRGSTPRC
>sp|P14618|KPYM_HUMAN Pyruvate kinase PKM OS=Homo sapiens OX=9606 GN=PKM PE=1
SV=4
MSKPHSEAGTAFIQTQQLHAAMADTFLEHMCRLDIDSPPITARNTGIICTIGPASRSVET
LKEMIKSGMNVARLNFSHGTHEYHAETIKNVRTATESFASDPILYRPVAVALDTKGPEIR

TGLIKGSGTAEVELKKGATLKITLDNAYMEKCDENILWLDYKNICKVVEVGSKIYVDDGL
ISLQVKQKGADFLVTEVENGGSLGSKKGVNLPGAAVDLPAVSEKDIQDLKFGVEQDVDMV
FASFIRKASDVHEVRKVLGEKGKNIKIISKIENHEGVRRFDEILEASDGIMVARGDLGIE
IPAEKVFLAQKMMIGRCNRAGKPVICATQMLESMIKKPRPTRAEGSDVANAVLDGADCIM
LSGETAKGDYPLEAVRMQHLIAREAEAAIYHLQLFEELRRLAPITSDPTEATAVGAVEAS
FKCCSGAIIVLTKSGRSAHQVARYRPRAPIIAVTRNPQTARQAHLYRGIFPVLCKDPVQE
AWAEDVDLRVNFAMNVGKARGFFKKGDVVIVLTGWRPGSGFTNTMRVVPVP
>sp|P14625|ENPL_HUMAN Endoplasmin OS=Homo sapiens OX=9606 GN=HSP90B1 PE=1 SV=1
MRALWVLGLCCVLLTFGSVRADDEVDVDGTVEEDLGKSREGSRTDDEVVQREEEAIQLDG
LNASQIRELREKSEKFAFQAEVNRMMKLIINSLYKNKEIFLRELISNASDALDKIRLISL
TDENALSGNEELTVKIKCDKEKNLLHVTDTGVGMTREELVKNLGTIAKSGTSEFLNKMTE
AQEDGQSTSELIGQFGVGFYSAFLVADKVIVTSKHNNDTQHIWESDSNEFSVIADPRGNT
LGRGTTITLVLKEEASDYLELDTIKNLVKKYSQFINFPIYVWSSKTETVEEPMEEEEAAK
EEKEESDDEAAVEEEEEEKKPKTKKVEKTVWDWELMNDIKPIWQRPSKEVEEDEYKAFYK
SFSKESDDPMAYIHFTAEGEVTFKSILFVPTSAPRGLFDEYGSKKSDYIKLYVRRVFITD
DFHDMMPKYLNFVKGVVDSDDLPLNVSRETLQQHKLLKVIRKKLVRKTLDMIKKIADDKY
NDTFWKEFGTNIKLGVIEDHSNRTRLAKLLRFQSSHHPTDITSLDQYVERMKEKQDKIYF
MAGSSRKEAESSPFVERLLKKGYEVIYLTEPVDEYCIQALPEFDGKRFQNVAKEGVKFDE
SEKTKESREAVEKEFEPLLNWMKDKALKDKIEKAVVSQRLTESPCALVASQYGWSGNMER
IMKAQAYQTGKDISTNYYASQKKTFEINPRHPLIRDMLRRIKEDEDDKTVLDLAVVLFET
ATLRSGYLLPDTKAYGDRIERMLRLSLNIDPDAKVEEEPEEEPEETAEDTTEDTEQDEDE
EMDVGTDEEEETAKESTAEKDEL
>sp|P14923|PLAK_HUMAN Junction plakoglobin OS=Homo sapiens OX=9606 GN=JUP PE=1
SV=3
MEVMNLMEQPIKVTEWQQTYTYDSGIHSGANTCVPSVSSKGIMEEDEACGRQYTLKKTTT
YTQGVPPSQGDLEYQMSTTARAKRVREAMCPGVSGEDSSLLLATQVEGQATNLQRLAEPS
QLLKSAIVHLINYQDDAELATRALPELTKLLNDEDPVVVTKAAMIVNQLSKKEASRRALM
GSPQLVAAVVRTMQNTSDLDTARCTTSILHNLSHHREGLLAIFKSGGIPALVRMLSSPVE
SVLFYAITTLHNLLLYQEGAKMAVRLADGLQKMVPLLNKNNPKFLAITTDCLQLLAYGNQ
ESKLIILANGGPQALVQIMRNYSYEKLLWTTSRVLKVLSVCPSNKPAIVEAGGMQALGKH
LTSNSPRLVQNCLWTLRNLSDVATKQEGLESVLKILVNQLSVDDVNVLTCATGTLSNLTC
NNSKNKTLVTQNSGVEALIHAILRAGDKDDITEPAVCALRHLTSRHPEAEMAQNSVRLNY
GIPAIVKLLNQPNQWPLVKATIGLIRNLALCPANHAPLQEAAVIPRLVQLLVKAHQDAQR
HVAAGTQQPYTDGVRMEEIVEGCTGALHILARDPMNRMEIFRLNTIPLFVQLLYSSVENI
QRVAAGVLCELAQDKEAADAIDAEGASAPLMELLHSRNEGTATYAAAVLFRISEDKNPDY
RKRVSVELTNSLFKHDPAAWEAAQSMIPINEPYGDDMDATYRPMYSSDVPLDPLEMHMDM
DGDYPIDTYSDGLRPPYPTADHMLA
>sp|P15531|NDKA_HUMAN Nucleoside diphosphate kinase A OS=Homo sapiens OX=9606
GN=NME1 PE=1 SV=1
MANCERTFIAIKPDGVQRGLVGEIIKRFEQKGFRLVGLKFMQASEDLLKEHYVDLKDRPF
FAGLVKYMHSGPVVAMVWEGLNVVKTGRVMLGETNPADSKPGTIRGDFCIQVGRNIIHGS
DSVESAEKEIGLWFHPEELVDYTSCAQNWIYE
>sp|P15923|TFE2_HUMAN Transcription factor E2-alpha OS=Homo sapiens OX=9606 GN=TCF3
PE=1 SV=1

MNQPQRMAPVGTDKELSDLLDFSMMFPLPVTNGKGRPASLAGAQFGGSGLEDRPSSGSWG
SGDQSSSSFDPSRTFSEGTHFTESHSSLSSSTFLGPGLGGKSGERGAYASFGRDAGVGGL
TQAGFLSGELALNSPGPLSPSGMKGTSQYYPSYSGSSRRRAADGSLDTQPKKVRKVPPGL
PSSVYPPSSGEDYGRDATAYPSAKTPSSTYPAPFYVADGSLHPSAELWSPPGQAGFGPML
GGGSSPLPLPPGSGPVGSSGSSSTFGGLHQHERMGYQLHGAEVNGGLPSASSFSSAPGAT
YGGVSSHTPPVSGADSLLGSRGTTAGSSGDALGKALASIYSPDHSSNNFSSSPSTPVGSP
QGLAGTSQWPRAGAPGALSPSYDGGLHGLQSKIEDHLDEAIHVLRSHAVGTAGDMHTLLP
GHGALASGFTGPMSLGGRHAGLVGGSHPEDGLAGSTSLMHNHAALPSQPGTLPDLSRPPD
SYSGLGRAGATAAASEIKREEKEDEENTSAADHSEEEKKELKAPRARTSPDEDEDDLLPP
EQKAEREKERRVANNARERLRVRDINEAFKELGRMCQLHLNSEKPQTKLLILHQAVSVIL
NLEQQVRERNLNPKAACLKRREEEKVSGVVGDPQMVLSAPHPGLSEAHNPAGHM
>sp|P15924|DESP_HUMAN Desmoplakin OS=Homo sapiens OX=9606 GN=DSP PE=1 SV=3
MSCNGGSHPRINTLGRMIRAESGPDLRYEVTSGGGGTSRMYYSRRGVITDQNSDGYCQTG
TMSRHQNQNTIQELLQNCSDCLMRAELIVQPELKYGDGIQLTRSRELDECFAQANDQMEI
LDSLIREMRQMGQPCDAYQKRLLQLQEQMRALYKAISVPRVRRASSKGGGGYTCQSGSGW
DEFTKHVTSECLGWMRQQRAEMDMVAWGVDLASVEQHINSHRGIHNSIGDYRWQLDKIKA
DLREKSAIYQLEEEYENLLKASFERMDHLRQLQNIIQATSREIMWINDCEEEELLYDWSD
KNTNIAQKQEAFSIRMSQLEVKEKELNKLKQESDQLVLNQHPASDKIEAYMDTLQTQWSW
ILQITKCIDVHLKENAAYFQFFEEAQSTEAYLKGLQDSIRKKYPCDKNMPLQHLLEQIKE
LEKEREKILEYKRQVQNLVNKSKKIVQLKPRNPDYRSNKPIILRALCDYKQDQKIVHKGD
ECILKDNNERSKWYVTGPGGVDMLVPSVGLIIPPPNPLAVDLSCKIEQYYEAILALWNQL
YINMKSLVSWHYCMIDIEKIRAMTIAKLKTMRQEDYMKTIADLELHYQEFIRNSQGSEMF
GDDDKRKIQSQFTDAQKHYQTLVIQLPGYPQHQTVTTTEITHHGTCQDVNHNKVIETNRE
NDKQETWMLMELQKIRRQIEHCEGRMTLKNLPLADQGSSHHITVKINELKSVQNDSQAIA
EVLNQLKDMLANFRGSEKYCYLQNEVFGLFQKLENINGVTDGYLNSLCTVRALLQAILQT
EDMLKVYEARLTEEETVCLDLDKVEAYRCGLKKIKNDLNLKKSLLATMKTELQKAQQIHS
QTSQQYPLYDLDLGKFGEKVTQLTDRWQRIDKQIDFRLWDLEKQIKQLRNYRDNYQAFCK
WLYDAKRRQDSLESMKFGDSNTVMRFLNEQKNLHSEISGKRDKSEEVQKIAELCANSIKD
YELQLASYTSGLETLLNIPIKRTMIQSPSGVILQEAADVHARYIELLTRSGDYYRFLSEM
LKSLEDLKLKNTKIEVLEEELRLARDANSENCNKNKFLDQNLQKYQAECSQFKAKLASLE
ELKRQAELDGKSAKQNLDKCYGQIKELNEKITRLTYEIEDEKRRRKSVEDRFDQQKNDYD
QLQKARQCEKENLGWQKLESEKAIKEKEYEIERLRVLLQEEGTRKREYENELAKVRNHYN
EEMSNLRNKYETEINITKTTIKEISMQKEDDSKNLRNQLDRLSRENRDLKDEIVRLNDSI
LQATEQRRRAEENALQQKACGSEIMQKKQHLEIELKQVMQQRSEDNARHKQSLEEAAKTI
QDKNKEIERLKAEFQEEAKRRWEYENELSKVRNNYDEEIISLKNQFETEINITKTTIHQL
TMQKEEDTSGYRAQIDNLTRENRSLSEEIKRLKNTLTQTTENLRRVEEDIQQQKATGSEV
SQRKQQLEVELRQVTQMRTEESVRYKQSLDDAAKTIQDKNKEIERLKQLIDKETNDRKCL
EDENARLQRVQYDLQKANSSATETINKLKVQEQELTRLRIDYERVSQERTVKDQDITRFQ
NSLKELQLQKQKVEEELNRLKRTASEDSCKRKKLEEELEGMRRSLKEQAIKITNLTQQLE
QASIVKKRSEDDLRQQRDVLDGHLREKQRTQEELRRLSSEVEALRRQLLQEQESVKQAHL
RNEHFQKAIEDKSRSLNESKIEIERLQSLTENLTKEHLMLEEELRNLRLEYDDLRRGRSE
ADSDKNATILELRSQLQISNNRTLELQGLINDLQRERENLRQEIEKFQKQALEASNRIQE
SKNQCTQVVQERESLLVKIKVLEQDKARLQRLEDELNRAKSTLEAETRVKQRLECEKQQI
QNDLNQWKTQYSRKEEAIRKIESEREKSEREKNSLRSEIERLQAEIKRIEERCRRKLEDS

TRETQSQLETERSRYQREIDKLRQRPYGSHRETQTECEWTVDTSKLVFDGLRKKVTAMQL
YECQLIDKTTLDKLLKGKKSVEEVASEIQPFLRGAGSIAGASASPKEKYSLVEAKRKKLI
SPESTVMLLEAQAATGGIIDPHRNEKLTVDSAIARDLIDFDDRQQIYAAEKAITGFDDPF
SGKTVSVSEAIKKNLIDRETGMRLLEAQIASGGVVDPVNSVFLPKDVALARGLIDRDLYR
SLNDPRDSQKNFVDPVTKKKVSYVQLKERCRIEPHTGLLLLSVQKRSMSFQGIRQPVTVT
ELVDSGILRPSTVNELESGQISYDEVGERIKDFLQGSSCIAGIYNETTKQKLGIYEAMKI
GLVRPGTALELLEAQAATGFIVDPVSNLRLPVEEAYKRGLVGIEFKEKLLSAERAVTGYN
DPETGNIISLFQAMNKELIEKGHGIRLLEAQIATGGIIDPKESHRLPVDIAYKRGYFNEE
LSEILSDPSDDTKGFFDPNTEENLTYLQLKERCIKDEETGLCLLPLKEKKKQVQTSQKNT
LRKRRVVIVDPETNKEMSVQEAYKKGLIDYETFKELCEQECEWEEITITGSDGSTRVVLV
DRKTGSQYDIQDAIDKGLVDRKFFDQYRSGSLSLTQFADMISLKNGVGTSSSMGSGVSDD
VFSSSRHESVSKISTISSVRNLTIRSSSFSDTLEESSPIAAIFDTENLEKISITEGIERG
IVDSITGQRLLEAQACTGGIIHPTTGQKLSLQDAVSQGVIDQDMATRLKPAQKAFIGFEG
VKGKKKMSAAEAVKEKWLPYEAGQRFLEFQYLTGGLVDPEVHGRISTEEAIRKGFIDGRA
AQRLQDTSSYAKILTCPKTKLKISYKDAINRSMVEDITGLRLLEAASVSSKGLPSPYNMS
SAPGSRSGSRSGSRSGSRSGSRSGSRRGSFDATGNSSYSYSYSFSSSSIGH
>sp|P16152|CBR1_HUMAN Carbonyl reductase [NADPH] 1 OS=Homo sapiens OX=9606
GN=CBR1 PE=1 SV=3
MSSGIHVALVTGGNKGIGLAIVRDLCRLFSGDVVLTARDVTRGQAAVQQLQAEGLSPRFH
QLDIDDLQSIRALRDFLRKEYGGLDVLVNNAGIAFKVADPTPFHIQAEVTMKTNFFGTRD
VCTELLPLIKPQGRVVNVSSIMSVRALKSCSPELQQKFRSETITEEELVGLMNKFVEDTK
KGVHQKEGWPSSAYGVTKIGVTVLSRIHARKLSEQRKGDKILLNACCPGWVRTDMAGPKA
TKSPEEGAETPVYLALLPPDAEGPHGQFVSEKRVEQW
>sp|P17066|HSP76_HUMAN Heat shock 70 kDa protein 6 OS=Homo sapiens OX=9606
GN=HSPA6 PE=1 SV=2
MQAPRELAVGIDLGTTYSCVGVFQQGRVEILANDQGNRTTPSYVAFTDTERLVGDAAKSQ
AALNPHNTVFDAKRLIGRKFADTTVQSDMKHWPFRVVSEGGKPKVRVCYRGEDKTFYPEE
ISSMVLSKMKETAEAYLGQPVKHAVITVPAYFNDSQRQATKDAGAIAGLNVLRIINEPTA
AAIAYGLDRRGAGERNVLIFDLGGGTFDVSVLSIDAGVFEVKATAGDTHLGGEDFDNRLV
NHFMEEFRRKHGKDLSGNKRALRRLRTACERAKRTLSSSTQATLEIDSLFEGVDFYTSIT
RARFEELCSDLFRSTLEPVEKALRDAKLDKAQIHDVVLVGGSTRIPKVQKLLQDFFNGKE
LNKSINPDEAVAYGAAVQAAVLMGDKCEKVQDLLLLDVAPLSLGLETAGGVMTTLIQRNA
TIPTKQTQTFTTYSDNQPGVFIQVYEGERAMTKDNNLLGRFELSGIPPAPRGVPQIEVTF
DIDANGILSVTATDRSTGKANKITITNDKGRLSKEEVERMVHEAEQYKAEDEAQRDRVAA
KNSLEAHVFHVKGSLQEESLRDKIPEEDRRKMQDKCREVLAWLEHNQLAEKEEYEHQKRE
LEQICRPIFSRLYGGPGVPGGSSCGTQARQGDPSTGPIIEEVD
>sp|P17931|LEG3_HUMAN Galectin-3 OS=Homo sapiens OX=9606 GN=LGALS3 PE=1 SV=5
MADNFSLHDALSGSGNPNPQGWPGAWGNQPAGAGGYPGASYPGAYPGQAPPGAYPGQAPP
GAYPGAPGAYPGAPAPGVYPGPPSGPGAYPSSGQPSATGAYPATGPYGAPAGPLIVPYNL
PLPGGVVPRMLITILGTVKPNANRIALDFQRGNDVAFHFNPRFNENNRRVIVCNTKLDNN
WGREERQSVFPFESGKPFKIQVLVEPDHFKVAVNDAHLLQYNHRVKKLNEISKLGISGDI
DLTSASYTMI
>sp|P21333|FLNA_HUMAN Filamin-A OS=Homo sapiens OX=9606 GN=FLNA PE=1 SV=4
MSSSHSRAGQSAAGAAPGGGVDTRDAEMPATEKDLAEDAPWKKIQQNTFTRWCNEHLKCV

SKRIANLQTDLSDGLRLIALLEVLSQKKMHRKHNQRPTFRQMQLENVSVALEFLDRESIK
LVSIDSKAIVDGNLKLILGLIWTLILHYSISMPMWDEEEDEEAKKQTPKQRLLGWIQNKL
PQLPITNFSRDWQSGRALGALVDSCAPGLCPDWDSWDASKPVTNAREAMQQADDWLGIPQ
VITPEEIVDPNVDEHSVMTYLSQFPKAKLKPGAPLRPKLNPKKARAYGPGIEPTGNMVKK
RAEFTVETRSAGQGEVLVYVEDPAGHQEEAKVTANNDKNRTFSVWYVPEVTGTHKVTVLF
AGQHIAKSPFEVYVDKSQGDASKVTAQGPGLEPSGNIANKTTYFEIFTAGAGTGEVEVVI
QDPMGQKGTVEPQLEARGDSTYRCSYQPTMEGVHTVHVTFAGVPIPRSPYTVTVGQACNP
SACRAVGRGLQPKGVRVKETADFKVYTKGAGSGELKVTVKGPKGEERVKQKDLGDGVYGF
EYYPMVPGTYIVTITWGGQNIGRSPFEVKVGTECGNQKVRAWGPGLEGGVVGKSADFVVE
AIGDDVGTLGFSVEGPSQAKIECDDKGDGSCDVRYWPQEAGEYAVHVLCNSEDIRLSPFM
ADIRDAPQDFHPDRVKARGPGLEKTGVAVNKPAEFTVDAKHGGKAPLRVQVQDNEGCPVE
ALVKDNGNGTYSCSYVPRKPVKHTAMVSWGGVSIPNSPFRVNVGAGSHPNKVKVYGPGVA
KTGLKAHEPTYFTVDCAEAGQGDVSIGIKCAPGVVGPAEADIDFDIIRNDNDTFTVKYTP
RGAGSYTIMVLFADQATPTSPIRVKVEPSHDASKVKAEGPGLSRTGVELGKPTHFTVNAK
AAGKGKLDVQFSGLTKGDAVRDVDIIDHHDNTYTVKYTPVQQGPVGVNVTYGGDPIPKSP
FSVAVSPSLDLSKIKVSGLGEKVDVGKDQEFTVKSKGAGGQGKVASKIVGPSGAAVPCKV
EPGLGADNSVVRFLPREEGPYEVEVTYDGVPVPGSPFPLEAVAPTKPSKVKAFGPGLQGG
SAGSPARFTIDTKGAGTGGLGLTVEGPCEAQLECLDNGDGTCSVSYVPTEPGDYNINILF
ADTHIPGSPFKAHVVPCFDASKVKCSGPGLERATAGEVGQFQVDCSSAGSAELTIEICSE
AGLPAEVYIQDHGDGTHTITYIPLCPGAYTVTIKYGGQPVPNFPSKLQVEPAVDTSGVQC
YGPGIEGQGVFREATTEFSVDARALTQTGGPHVKARVANPSGNLTETYVQDRGDGMYKVE
YTPYEEGLHSVDVTYDGSPVPSSPFQVPVTEGCDPSRVRVHGPGIQSGTTNKPNKFTVET
RGAGTGGLGLAVEGPSEAKMSCMDNKDGSCSVEYIPYEAGTYSLNVTYGGHQVPGSPFKV
PVHDVTDASKVKCSGPGLSPGMVRANLPQSFQVDTSKAGVAPLQVKVQGPKGLVEPVDVV
DNADGTQTVNYVPSREGPYSISVLYGDEEVPRSPFKVKVLPTHDASKVKASGPGLNTTGV
PASLPVEFTIDAKDAGEGLLAVQITDPEGKPKKTHIQDNHDGTYTVAYVPDVTGRYTILI
KYGGDEIPFSPYRVRAVPTGDASKCTVTVSIGGHGLGAGIGPTIQIGEETVITVDTKAAG
KGKVTCTVCTPDGSEVDVDVVENEDGTFDIFYTAPQPGKYVICVRFGGEHVPNSPFQVTA
LAGDQPSVQPPLRSQQLAPQYTYAQGGQQTWAPERPLVGVNGLDVTSLRPFDLVIPFTIK
KGEITGEVRMPSGKVAQPTITDNKDGTVTVRYAPSEAGLHEMDIRYDNMHIPGSPLQFYV
DYVNCGHVTAYGPGLTHGVVNKPATFTVNTKDAGEGGLSLAIEGPSKAEISCTDNQDGTC
SVSYLPVLPGDYSILVKYNEQHVPGSPFTARVTGDDSMRMSHLKVGSAADIPINISETDL
SLLTATVVPPSGREEPCLLKRLRNGHVGISFVPKETGEHLVHVKKNGQHVASSPIPVVIS
QSEIGDASRVRVSGQGLHEGHTFEPAEFIIDTRDAGYGGLSLSIEGPSKVDINTEDLEDG
TCRVTYCPTEPGNYIINIKFADQHVPGSPFSVKVTGEGRVKESITRRRRAPSVANVGSHC
DLSLKIPEISIQDMTAQVTSPSGKTHEAEIVEGENHTYCIRFVPAEMGTHTVSVKYKGQH
VPGSPFQFTVGPLGEGGAHKVRAGGPGLERAEAGVPAEFSIWTREAGAGGLAIAVEGPSK
AEISFEDRKDGSCGVAYVVQEPGDYEVSVKFNEEHIPDSPFVVPVASPSGDARRLTVSSL
QESGLKVNQPASFAVSLNGAKGAIDAKVHSPSGALEECYVTEIDQDKYAVRFIPRENGVY
LIDVKFNGTHIPGSPFKIRVGEPGHGGDPGLVSAYGAGLEGGVTGNPAEFVVNTSNAGAG
ALSVTIDGPSKVKMDCQECPEGYRVTYTPMAPGSYLISIKYGGPYHIGGSPFKAKVTGPR
LVSNHSLHETSSVFVDSLTKATCAPQHGAPGPGPADASKVVAKGLGLSKAYVGQKSSFTV
DCSKAGNNMLLVGVHGPRTPCEEILVKHVGSRLYSVSYLLKDKGEYTLVVKWGDEHIPGS
PYRVVVP

>sp|P21796|VDAC1_HUMAN Voltage-dependent anion-selective channel protein 1 OS=Homo sapiens OX=9606 GN=VDAC1 PE=1 SV=2
MAVPPTYADLGKSARDVFTKGYGFGLIKLDLKTKSENGLEFTSSGSANTETTKVTGSLET
KYRWTEYGLTFTEKWNTDNTLGTEITVEDQLARGLKLTFDSSFSPNTGKKNAKIKTGYKR
EHINLGCDMDFDIAGPSIRGALVLGYEGWLAGYQMNFETAKSRVTQSNFAVGYKTDEFQL
HTNVNDGTEFGGSIYQKVNKKLETAVNLAWTAGNSNTRFGIAAKYQIDPDACFSAKVNNS
SLIGLGYTQTLKPGIKLTLSALLDGKNVNAGGHKLGLGLEFQA
>sp|P22061|PIMT_HUMAN Protein-L-isoaspartate(D-aspartate) O-methyltransferase OS=Homo sapiens OX=9606 GN=PCMT1 PE=1 SV=4
MAWKSGGASHSELIHNLRKNGIIKTDKVFEVMLATDRSHYAKCNPYMDSPQSIGFQATIS
APHMHAYALELLFDQLHEGAKALDVGSGSGILTACFARMVGCTGKVIGIDHIKELVDDSV
NNVRKDDPTLLSSGRVQLVVGDGRMGYAEEAPYDAIHVGAAAPVVPQALIDQLKPGGRLI
LPVGPAGGNQMLEQYDKLQDGSIKMKPLMGVIYVPLTDKEKQWSRWK
>sp|P22352|GPX3_HUMAN Glutathione peroxidase 3 OS=Homo sapiens OX=9606 GN=GPX3 PE=1 SV=2
MARLLQASCLLSLLLAGFVSQSRGQEKSKMDCHGGISGTIYEYGALTIDGEEYIPFKQYA
GKYVLFVNVASYUGLTGQYIELNALQEELAPFGLVILGFPCNQFGKQEPGENSEILPTLK
YVRPGGGFVPNFQLFEKGDVNGEKEQKFYTFLKNSCPPTSELLGTSDRLFWEPMKVHDIR
WNFEKFLVGPDGIPIMRWHHRTTVSNVKMDILSYMRRQAALGVKRK
>sp|P22626|ROA2_HUMAN Heterogeneous nuclear ribonucleoproteins A2/B1 OS=Homo sapiens OX=9606 GN=HNRNPA2B1 PE=1 SV=2
MEKTLETVPLERKKREKEQFRKLFIGGLSFETTEESLRNYYEQWGKLTDCVVMRDPASKR
SRGFGFVTFSSMAEVDAAMAARPHSIDGRVVEPKRAVAREESGKPGAHVTVKKLFVGGIK
EDTEEHHLRDYFEEYGKIDTIEIITDRQSGKKRGFGFVTFDDHDPVDKIVLQKYHTINGH
NAEVRKALSRQEMQEVQSSRSGRGGNFGFGDSRGGGGNFGPGPGSNFRGGSDGYGSGRGF
GDGYNGYGGGPGGGNFGGSPGYGGGRGGYGGGGPGYGNQGGGYGGGYDNYGGGNYGSGNY
NDFGNYNQQPSNYGPMKSGNFGGSRNMGGPYGGGNYGPGGSGGSGGYGGRSRY
>sp|P22914|CRYGS_HUMAN Gamma-crystallin S OS=Homo sapiens OX=9606 GN=CRYGS PE=1 SV=4
MSKTGTKITFYEDKNFQGRRYDCDCDCADFHTYLSRCNSIKVEGGTWAVYERPNFAGYMY
ILPQGEYPEYQRWMGLNDRLSSCRAVHLPSGGQYKIQIFEKGDFSGQMYETTEDCPSIME
QFHMREIHSCKVLEGVWIFYELPNYRGRQYLLDKKEYRKPIDWGAASPAVQSFRRIVE
>sp|P23284|PPIB_HUMAN Peptidyl-prolyl cis-trans isomerase B OS=Homo sapiens OX=9606 GN=PPIB PE=1 SV=2
MLRLSERNMKVLLAAALIAGSVFFLLLPGPSAADEKKKGPKVTVKVYFDLRIGDEDVGRV
IFGLFGKTVPKTVDNFVALATGEKGFGYKNSKFHRVIKDFMIQGGDFTRGDGTGGKSIYG
ERFPDENFKLKHYGPGWVSMANAGKDTNGSQFFITTVKTAWLDGKHVVFGKVLEGMEVVR
KVESTKTDSRDKPLKDVIIADCGKIEVEKPFAIAKE
>sp|P23528|COF1_HUMAN Cofilin-1 OS=Homo sapiens OX=9606 GN=CFL1 PE=1 SV=3
MASGVAVSDGVIKVFNDMKVRKSSTPEEVKKRKKAVLFCLSEDKKNIILEEGKEILVGDV
GQTVDDPYATFVKMLPDKDCRYALYDATYETKESKKEDLVFIFWAPESAPLKSKMIYASS
KDAIKKKLTGIKHELQANCYEEVKDRCTLAEKLGGSAVISLEGKPL
>sp|P24752|THIL_HUMAN Acetyl-CoA acetyltransferase, mitochondrial OS=Homo sapiens OX=9606 GN=ACAT1 PE=1 SV=1

MAVLAALLRSGARSRSPLLRRLVQEIRYVERSYVSKPTLKEVVIVSATRTPIGSFLGSLS
LLPATKLGSIAIQGAIEKAGIPKEEVKEAYMGNVLQGGEGQAPTRQAVLGAGLPISTPCT
TINKVCASGMKAIMMASQSLMCGHQDVMVAGGMESMSNVPYVMNRGSTPYGGVKLEDLIV
KDGLTDVYNKIHMGSCAENTAKKLNIARNEQDAYAINSYTRSKAAWEAGKFGNEVIPVTV
TVKGQPDVVVKEDEEYKRVDFSKVPKLKTVFQKENGTVTAANASTLNDGAAALVLMTADA
AKRLNVTPLARIVAFADAAVEPIDFPIAPVYAASMVLKDVGLKKEDIAMWEVNEAFSLVV
LANIKMLEIDPQKVNINGGAVSLGHPIGMSGARIVGHLTHALKQGEYGLASICNGGGGAS
AMLIQKL
>sp|P25705|ATPA_HUMAN ATP synthase subunit alpha, mitochondrial OS=Homo sapiens OX=9606 GN=ATP5F1A PE=1 SV=1
MLSVRVAAAVVRALPRRAGLVSRNALGSSFIAARNFHASNTHLQKTGTAEMSSILEERIL
GADTSVDLEETGRVLSIGDGIARVHGLRNVQAEEMVEFSSGLKGMSLNLEPDNVGVVVFG
NDKLIKEGDIVKRTGAIVDVPVGEELLGRVVDALGNAIDGKGPIGSKTRRRVGLKAPGII
PRISVREPMQTGIKAVDSLVPIGRGQRELIIGDRQTGKTSIAIDTIINQKRFNDGSDEKK
KLYCIYVAIGQKRSTVAQLVKRLTDADAMKYTIVVSATASDAAPLQYLAPYSGCSMGEYF
RDNGKHALIIYDDLSKQAVAYRQMSLLLRRPPGREAYPGDVFYLHSRLLERAAKMNDAFG
GGSLTALPVIETQAGDVSAYIPTNVISITDGQIFLETELFYKGIRPAINVGLSVSRVGSA
AQTRAMKQVAGTMKLELAQYREVAAFAQFGSDLDAATQQLLSRGVRLTELLKQGQYSPMA
IEEQVAVIYAGVRGYLDKLEPSKITKFENAFLSHVVSQHQALLGTIRADGKISEQSDAKL
KEIVTNFLAGFEA
>sp|P26998|CRBB3_HUMAN Beta-crystallin B3 OS=Homo sapiens OX=9606 GN=CRYBB3 PE=1 SV=4
MAEQHGAPEQAAAGKSHGDLGGSYKVILYELENFQGKRCELSAECPSLTDSLLEKVGSIQ
VESGPWLAFESRAFRGEQFVLEKGDYPRWDAWSNSRDSDSLLSLRPLNIDSPHHKLHLFE
NPAFSGRKMEIVDDDVPSLWAHGFQDRVASVRAINGTWVGYEFPGYRGRQYVFERGEYRH
WNEWDASQPQLQSVRRIRDQKWHKRGRFPSS
>sp|P27824|CALX_HUMAN Calnexin OS=Homo sapiens OX=9606 GN=CANX PE=1 SV=2
MEGKWLLCMLLVLGTAIVEAHDGHDDDVIDIEDDLDDVIEEVEDSKPDTTAPPSSPKVTY
KAPVPTGEVYFADSFDRGTLSGWILSKAKKDDTDDEIAKYDGKWEVEEMKESKLPGDKGL
VLMSRAKHHAISAKLNKPFLFDTKPLIVQYEVNFQNGIECGGAYVKLLSKTPELNLDQFH
DKTPYTIMFGPDKCGEDYKLHFIFRHKNPKTGIYEEKHAKRPDADLKTYFTDKKTHLYTL
ILNPDNSFEILVDQSVVNSGNLLNDMTPPVNPSREIEDPEDRKPEDWDERPKIPDPEAVK
PDDWDEDAPAKIPDEEATKPEGWLDDEPEYVPDPDAEKPEDWDEDMDGEWEAPQIANPRC
ESAPGCGVWQRPVIDNPNYKGKWKPPMIDNPSYQGIWKPRKIPNPDFFEDLEPFRMTPFS
AIGLELWSMTSDIFFDNFIICADRRIVDDWANDGWGLKKAADGAAEPGVVGQMIEAAEER
PWLWVVYILTVALPVFLVILFCCSGKKQTSGMEYKKTDAPQPDVKEEEEEKEEEKDKGDE
EEEGEEKLEEKQKSDAEEDGGTVSQEEEDRKPKAEEDEILNRSPRNRKPRRE
>sp|P29401|TKT_HUMAN Transketolase OS=Homo sapiens OX=9606 GN=TKT PE=1 SV=3
MESYHKPDQQKLQALKDTANRLRISSIQATTAAGSGHPTSCCSAAEIMAVLFFHTMRYKS
QDPRNPHNDRFVLSKGHAAPILYAVWAEAGFLAEAELLNLRKISSDLDGHPVPKQAFTDV
ATGSLGQGLGAACGMAYTGKYFDKASYRVYCLLGDGELSEGSVWEAMAFASIYKLDNLVA
ILDINRLGQSDPAPLQHQMDIYQKRCEAFGWHAIIVDGHSVEELCKAFGQAKHQPTAIIA
KTFKGRGITGVEDKESWHGKPLPKNMAEQIIQEIYSQIQSKKKILATPPQEDAPSVDIAN
IRMPSLPSYKVGDKIATRKAYGQALAKLGHASDRIIALDGDTKNSTFSEIFKKEHPDRFI

ECYIAEQNMVSIAVGCATRNRTVPFCSTFAAFFTRAFDQIRMAAISESNINLCGSHCGVS
IGEDGPSQMALEDLAMFRSVPTSTVFYPSDGVATEKAVELAANTKGICFIRTSRPENAII
YNNNEDFQVGQAKVVLKSKDDQVTVIGAGVTLHEALAAAELLKKEKINIRVLDPFTIKPL
DRKLILDSARATKGRILTVEDHYYEGGIGEAVSSAVVGEPGITVTHLAVNRVPRSGKPAE
LLKMFGIDRDAIAQAVRGLITKA
>sp|P30044|PRDX5_HUMAN Peroxiredoxin-5, mitochondrial OS=Homo sapiens OX=9606
GN=PRDX5 PE=1 SV=4
MGLAGVCALRRSAGYILVGGAGGQSAAAAARRYSEGEWASGGVRSFSRAAAAMAPIKVGD
AIPAVEVFEGEPGNKVNLAELFKGKKGVLFGVPGAFTPGCSKTHLPGFVEQAEALKAKGV
QVVACLSVNDAFVTGEWGRAHKAEGKVRLLADPTGAFGKETDLLLDDSLVSIFGNRRLKR
FSMVVQDGIVKALNVEPDGTGLTCSLAPNIISQL
>sp|P30086|PEBP1_HUMAN Phosphatidylethanolamine-binding protein 1 OS=Homo sapiens
OX=9606 GN=PEBP1 PE=1 SV=3
MPVDLSKWSGPLSLQEVDEQPQHPLHVTYAGAAVDELGKVLTPTQVKNRPTSISWDGLDS
GKLYTLVLTDPDAPSRKDPKYREWHHFLVVNMKGNDISSGTVLSDYVGSGPPKGTGLHRY
VWLVYEQDRPLKCDEPILSNRSGDHRGKFKVASFRKKYELRAPVAGTCYQAEWDDYVPKL
YEQLSGK
>sp|P30101|PDIA3_HUMAN Protein disulfide-isomerase A3 OS=Homo sapiens OX=9606
GN=PDIA3 PE=1 SV=4
MRLRRLALFPGVALLLAAARLAAASDVLELTDDNFESRISDTGSAGLMLVEFFAPWCGHC
KRLAPEYEAAATRLKGIVPLAKVDCTANTNTCNKYGVSGYPTLKIFRDGEEAGAYDGPRT
ADGIVSHLKKQAGPASVPLRTEEEFKKFISDKDASIVGFFDDSFSEAHSEFLKAASNLRD
NYRFAHTNVESLVNEYDDNGEGIILFRPSHLTNKFEDKTVAYTEQKMTSGKIKKFIQENI
FGICPHMTEDNKDLIQGKDLLIAYYDVDYEKNAKGSNYWRNRVMMVAKKFLDAGHKLNFA
VASRKTFSHELSDFGLESTAGEIPVVAIRTAKGEKFVMQEEFSRDGKALERFLQDYFDGN
LKRYLKSEPIPESNDGPVKVVVAENFDEIVNNENKDVLIEFYAPWCGHCKNLEPKYKELG
EKLSKDPNIVIAKMDATANDVPSPYEVRGFPTIYFSPANKKLNPKKYEGGRELSDFISYL
QREATNPPVIQEEKPKKKKKAQEDL
>sp|P30626|SORCN_HUMAN Sorcin OS=Homo sapiens OX=9606 GN=SRI PE=1 SV=1
MAYPGHPGAGGGYYPGGYGGAPGGPAFPGQTQDPLYGYFAAVAGQDGQIDADELQRCLTQ
SGIAGGYKPFNLETCRLMVSMLDRDMSGTMGFNEFKELWAVLNGWRQHFISFDTDRSGTV
DPQELQKALTTMGFRLSPQAVNSIAKRYSTNGKITFDDYIACCVKLRALTDSFRRRDTAQ
QGVVNFPYDDFIQCVMSV
>sp|P31944|CASPE_HUMAN Caspase-14 OS=Homo sapiens OX=9606 GN=CASP14 PE=1 SV=2
MSNPRSLEEEKYDMSGARLALILCVTKAREGSEEDLDALEHMFRQLRFESTMKRDPTAEQ
FQEELEKFQQAIDSREDPVSCAFVVLMAHGREGFLKGEDGEMVKLENLFEALNNKNCQAL
RAKPKVYIIQACRGEQRDPGETVGGDEIVMVIKDSPQTIPTYTDALHVYSTVEGYIAYRH
DQKGSCFIQTLVDVFTKRKGHILELLTEVTRRMAEAELVQEGKARKTNPEIQSTLRKRLY
LQ
>sp|P31946|1433B_HUMAN 14-3-3 protein beta/alpha OS=Homo sapiens OX=9606
GN=YWHAB PE=1 SV=3
MTMDKSELVQKAKLAEQAERYDDMAAAMKAVTEQGHELSNEERNLLSVAYKNVVGARRSS
WRVISSIEQKTERNEKKQQMGKEYREKIEAELQDICNDVLELLDKYLIPNATQPESKVFY
LKMKGDYFRYLSEVASGDNKQTTVSNSQQAYQEAFEISKKEMQPTHPIRLGLALNFSVFY

YEILNSPEKACSLAKTAFDEAIAELDTLNEESYKDSTLIMQLLRDNLTLWTSENQGDEGD
AGEGEN
>sp|P32119|PRDX2_HUMAN Peroxiredoxin-2 OS=Homo sapiens OX=9606 GN=PRDX2 PE=1 SV=5
MASGNARIGKPAPDFKATAVVDGAFKEVKLSDYKGKYVVLFFYPLDFTFVCPTEIIAFSN
RAEDFRKLGCEVLGVSVDSQFTHLAWINTPRKEGGLGPLNIPLLADVTRRLSEDYGVLKT
DEGIAYRGLFIIDGKGVLRQITVNDLPVGRSVDEALRLVQAFQYTDEHGEVCPAGWKPGS
DTIKPNVDDSKEYFSKHN
>sp|P34931|HS71L_HUMAN Heat shock 70 kDa protein 1-like OS=Homo sapiens OX=9606 GN=HSPA1L PE=1 SV=2
MATAKGIAIGIDLGTTYSCVGVFQHGKVEIIANDQGNRTTPSYVAFTDTERLIGDAAKNQ
VAMNPQNTVFDAKRLIGRKFNDPVVQADMKLWPFQVINEGGKPKVLVSYKGENKAFYPEE
ISSMVLTKLKETAEAFLGHPVTNAVITVPAYFNDSQRQATKDAGVIAGLNVLRIINEPTA
AAIAYGLDKGGQGERHVLIFDLGGGTFDVSILTIDDGIFEVKATAGDTHLGGEDFDNRLV
SHFVEEFKRKHKKDISQNKRAVRRLRTACERAKRTLSSSTQANLEIDSLYEGIDFYTSIT
RARFEELCADLFRGTLEPVEKALRDAKMDKAKIHDIVLVGGSTRIPKVQRLLQDYFNGRD
LNKSINPDEAVAYGAAVQAAILMGDKSEKVQDLLLLDVAPLSLGLETAGGVMTALIKRNS
TIPTKQTQIFTTYSDNQPGVLIQVYEGERAMTKDNNLLGRFDLTGIPPAPRGVPQIEVTF
DIDANGILNVTATDKSTGKVNKITITNDKGRLSKEEIERMVLDAEKYKAEDEVQREKIAA
KNALESYAFNMKSVVSDEGLKGKISESDKNKILDKCNELLSWLEVNQLAEKDEFDHKRKE
LEQMCNPIITKLYQGGCTGPACGTGYVPGRPATGPTIEEVD
>sp|P35268|RL22_HUMAN Large ribosomal subunit protein eL22 OS=Homo sapiens OX=9606 GN=RPL22 PE=1 SV=2
MAPVKKLVVKGGKKKKQVLKFTLDCTHPVEDGIMDAANFEQFLQERIKVNGKAGNLGGGV
VTIERSKSKITVTSEVPFSKRYLKYLTKKYLKKNNLRDWLRVVANSKESYELRYFQINQD
EEEEEDED
>sp|P35527|K1C9_HUMAN Keratin, type I cytoskeletal 9 OS=Homo sapiens OX=9606 GN=KRT9 PE=1 SV=3
MSCRQFSSSYLSRSGGGGGGGLGSGGSIRSSYSRFSSSGGGGGGGRFSSSSGYGGGSSRV
CGRGGGGSFGYSYGGGSGGGFSASSLGGGFGGGSRGFGGASGGGYSSSGGFGGGFGGGSG
GGFGGGYGSGFGGFGGFGGGAGGGDGGILTANEKSTMQELNSRLASYLDKVQALEEANND
LENKIQDWYDKKGPAAIQKNYSPYYNTIDDLKDQIVDLTVGNNKTLLDIDNTRMTLDDFR
IKFEMEQNLRQGVDADINGLRQVLDNLTMEKSDLEMQYETLQEELMALKKNHKEEMSQLT
GQNSGDVNVEINVAPGKDLTKTLNDMRQEYEQLIAKNRKDIENQYETQITQIEHEVSSSG
QEVQSSAKEVTQLRHGVQELEIELQSQLSKKAALEKSLEDTKNRYCGQLQMIQEQISNLE
AQITDVRQEIECQNQEYSLLLSIKMRLEKEIETYHNLLEGGQEDFESSGAGKIGLGGRGG
SGGSYGRGSRGGSGGSYGGGGSGGGYGGGSGSRGGSGGSYGGGSGSGGGSGGGYGGGSGG
GHSGGSGGGHSGGSGGNYGGGSGSGGGSGGGYGGGSGSRGGSGGSHGGGSGFGGESGGSY
GGGEEASGSGGGYGGGSGKSSHS
>sp|P35908|K22E_HUMAN Keratin, type II cytoskeletal 2 epidermal OS=Homo sapiens OX=9606 GN=KRT2 PE=1 SV=2
MSCQISCKSRGRGGGGGGFRGFSSGSAVVSGGSRRSTSSFSCLSRHGGGGGGFGGGGFGS
RSLVGLGGTKSISISVAGGGGGFGAAGGFGGRGGGFGGGSSFGGGSGFSGGGFGGGGFGG
GRFGGFGGPGGVGGLGGPGGFGPGGYPGGIHEVSVNQSLLQPLNVKVDPEIQNVKAQERE

QIKTLNNKFASFIDKVRFLEQQNQVLQTKWELLQQMNVGTRPINLEPIFQGYIDSLKRYL
DGLTAERTSQNSELNNMQDLVEDYKKKYEDEINKRTAAENDFVTLKKDVDNAYMIKVELQ
SKVDLLNQEIEFLKVLYDAEISQIHQSVTDTNVILSMDNSRNLDLDSIIAEVKAQYEEIA
QRSKEEAEALYHSKYEELQVTVGRHGDSLKEIKIEISELNRVIQRLQGEIAHVKKQCKNV
QDAIADAEQRGEHALKDARNKLNDLEEALQQAKEDLARLLRDYQELMNVKLALDVEIATY
RKLLEGEECRMSGDLSSNVTVSVTSSTISSNVASKAAFGGSGGRGSSSGGGYSSGSSSYG
SGGRQSGSRGGSGGGGSISGGGYGSGGGSGGRYGSGGGSKGGSISGGGYGSGGGKHSSGG
GSRGGSSSGGGYGSGGGGSSSVKGSSGEAFGSSVTFSFR
>sp|P40926|MDHM_HUMAN Malate dehydrogenase, mitochondrial OS=Homo sapiens OX=9606 GN=MDH2 PE=1 SV=3
MLSALARPASAALRRSFSTSAQNNAKVAVLGASGGIGQPLSLLLKNSPLVSRLTLYDIAH
TPGVAADLSHIETKAAVKGYLGPEQLPDCLKGCDVVVIPAGVPRKPGMTRDDLFNTNATI
VATLTAACAQHCPEAMICVIANPVNSTIPITAEVFKKHGVYNPNKIFGVTTLDIVRANTF
VAELKGLDPARVNVPVIGGHAGKTIIPLISQCTPKVDFPQDQLTALTGRIQEAGTEVVKA
KAGAGSATLSMAYAGARFVFSLVDAMNGKEGVVECSFVKSQETECTYFSTPLLLGKKGIE
KNLGIGKVSSFEEKMISDAIPELKASIKKGEDFVKTLK
>sp|P40939|ECHA_HUMAN Trifunctional enzyme subunit alpha, mitochondrial OS=Homo sapiens OX=9606 GN=HADHA PE=1 SV=2
MVACRAIGILSRFSAFRILRSRGYICRNFTGSSALLTRTHINYGVKGDVAVVRINSPNSK
VNTLSKELHSEFSEVMNEIWASDQIRSAVLISSKPGCFIAGADINMLAACKTLQEVTQLS
QEAQRIVEKLEKSTKPIVAAINGSCLGGGLEVAISCQYRIATKDRKTVLGTPEVLLGALP
GAGGTQRLPKMVGVPAALDMMLTGRSIRADRAKKMGLVDQLVEPLGPGLKPPEERTIEYL
EEVAITFAKGLADKKISPKRDKGLVEKLTAYAMTIPFVRQQVYKKVEEKVRKQTKGLYPA
PLKIIDVVKTGIEQGSDAGYLCESQKFGELVMTKESKALMGLYHGQVLCKKNKFGAPQKD
VKHLAILGAGLMGAGIAQVSVDKGLKTILKDATLTALDRGQQQVFKGLNDKVKKKALTSF
ERDSIFSNLTGQLDYQGFEKADMVIEAVFEDLSLKHRVLKEVEAVIPDHCIFASNTSALP
ISEIAAVSKRPEKVIGMHYFSPVDKMQLLEIITTEKTSKDTSASAVAVGLKQGKVIIVVK
DGPGFYTTRCLAPMMSEVIRILQEGVDPKKLDSLTTSFGFPVGAATLVDEVGVDVAKHVA
EDLGKVFGERFGGGNPELLTQMVSKGFLGRKSGKGFYIYQEGVKRKDLNSDMDSILASLK
LPPKSEVSSDEDIQFRLVTRFVNEAVMCLQEGILATPAEGDIGAVFGLGFPPCLGGPFRF
VDLYGAQKIVDRLKKYEAAYGKQFTPCQLLADHANSPNKKFYQ
>sp|P41222|PTGDS_HUMAN Prostaglandin-H2 D-isomerase OS=Homo sapiens OX=9606 GN=PTGDS PE=1 SV=1
MATHHTLWMGLALLGVLGDLQAAPEAQVSVQPNFQQDKFLGRWFSAGLASNSSWLREKKA
ALSMCKSVVAPATDGGLNLTSTFLRKNQCETRTMLLQPAGSLGSYSYRSPHWGSTYSVSV
VETDYDQYALLYSQGSKGPGEDFRMATLYSRTQTPRAELKEKFTAFCKAQGFTEDTIVFL
PQTDKCMTEQ
>sp|P43320|CRBB2_HUMAN Beta-crystallin B2 OS=Homo sapiens OX=9606 GN=CRYBB2 PE=1 SV=2
MASDHQTQAGKPQSLNPKIIIFEQENFQGHSHELNGPCPNLKETGVEKAGSVLVQAGPWV
GYEQANCKGEQFVFEKGEYPRWDSWTSSRRTDSLSSLRPIKVDSQEHKIILYENPNFTGK
KMEIIDDDVPSFHAHGYQEKVSSVRVQSGTWVGYQYPGYRGLQYLLEKGDYKDSSDFGAP
HPQVQSVRRIRDMQWHQRGAFHPSN

>sp|P45880|VDAC2_HUMAN Voltage-dependent anion-selective channel protein 2 OS=Homo sapiens OX=9606 GN=VDAC2 PE=1 SV=2
MATHGQTCARPMCIPPSYADLGKAARDIFNKGFGFGLVKLDVKTKSCSGVEFSTSGSSNT
DTGKVTGTLETKYKWCEYGLTFTEKWNTDNTLGTEIAIEDQICQGLKLTFDTTFSPNTGK
KSGKIKSSYKRECINLGCDVDFDFAGPAIHGSAVFGYEGWLAGYQMTFDSAKSKLTRNNF
AVGYRTGDFQLHTNVNDGTEFGGSIYQKVCEDLDTSVNLAWTSGTNCTRFGIAAKYQLDP
TASISAKVNNSSLIGVGYTQTLRPGVKLTLSALVDGKSINAGGHKVGLALELEA
>sp|P48307|TFPI2_HUMAN Tissue factor pathway inhibitor 2 OS=Homo sapiens OX=9606 GN=TFPI2 PE=1 SV=1
MDPARPLGLSILLLFLTEAALGDAAQEPTGNNAEICLLPLDYGPCRALLLRYYYDRYTQS
CRQFLYGGCEGNANNFYTWEACDDACWRIEKVPKVCRLQVSVDDQCEGSTEKYFFNLSSM
TCEKFFSGGCHRNRIENRFPDEATCMGFCAPKKIPSFCYSPKDEGLCSANVTRYYFNPRY
RTCDAFTYTGCGGNDNNFVSREDCKRACAKALKKKKKMPKLRFASRIRKIRKKQF
>sp|P48668|K2C6C_HUMAN Keratin, type II cytoskeletal 6C OS=Homo sapiens OX=9606 GN=KRT6C PE=1 SV=3
MASTSTTIRSHSSSRRGFSANSARLPGVSRSGFSSISVSRSRGSGGLGGACGGAGFGSRS
LYGLGGSKRISIGGGSCAISGGYGSRAGGSYGFGGAGSGFGFGGGAGIGFGLGGGAGLAG
GFGGPGFPVCPPGGIQEVTVNQSLLTPLNLQIDPAIQRVRAEEREQIKTLNNKFASFIDK
VRFLEQQNKVLDTKWTLLQEQGTKTVRQNLEPLFEQYINNLRRQLDSIVGERGRLDSELR
NMQDLVEDLKNKYEDEINKRTAAENEFVTLKKDVDAAYMNKVELQAKADTLTDEINFLRA
LYDAELSQMQTHISDTSVVLSMDNNRNLDLDSIIAEVKAQYEEIAQRSRAEAESWYQTKY
EELQVTAGRHGDDLRNTKQEIAEINRMIQRLRSEIDHVKKQCASLQAAIADAEQRGEMAL
KDAKNKLEGLEDALQKAKQDLARLLKEYQELMNVKLALDVEIATYRKLLEGEECRLNGEG
VGQVNVSVVQSTISSGYGGASGVGSGLGLGGGSSYSYGSGLGIGGGFSSSSGRAIGGGLS
SVGGGSSTIKYTTTSSSSRKSYKH
>sp|P50993|AT1A2_HUMAN Sodium/potassium-transporting ATPase subunit alpha-2 OS=Homo sapiens OX=9606 GN=ATP1A2 PE=1 SV=1
MGRGAGREYSPAATTAENGGGKKKQKEKELDELKKEVAMDDHKLSLDELGRKYQVDLSKG
LTNQRAQDVLARDGPNALTPPPTTPEWVKFCRQLFGGFSILLWIGAILCFLAYGIQAAME
DEPSNDNLYLGVVLAAVVIVTGCFSYYQEAKSSKIMDSFKNMVPQQALVIREGEKMQINA
EEVVVGDLVEVKGGDRVPADLRIISSHGCKVDNSSLTGESEPQTRSPEFTHENPLETRNI
CFFSTNCVEGTARGIVIATGDRTVMGRIATLASGLEVGRTPIAMEIEHFIQLITGVAVFL
GVSFFVLSLILGYSWLEAVIFLIGIIVANVPEGLLATVTVCLTLTAKRMARKNCLVKNLE
AVETLGSTSTICSDKTGTLTQNRMTVAHMWFDNQIHEADTTEDQSGATFDKRSPTWTALS
RIAGLCNRAVFKAGQENISVSKRDTAGDASESALLKCIELSCGSVRKMRDRNPKVAEIPF
NSTNKYQLSIHEREDSPQSHVLVMKGAPERILDRCSTILVQGKEIPLDKEMQDAFQNAYM
ELGGLGERVLGFCQLNLPSGKFPRGFKFDTDELNFPTEKLCFVGLMSMIDPPRAAVPDAV
GKCRSAGIKVIMVTGDHPITAKAIAKGVGIISEGNETVEDIAARLNIPMSQVNPREAKAC
VVHGSDLKDMTSEQLDEILKNHTEIVFARTSPQQKLIIVEGCQRQGAIVAVTGDGVNDSP
ALKKADIGIAMGISGSDVSKQAADMILLDDNFASIVTGVEEGRLIFDNLKKSIAYTLTSN
IPEITPFLLFIIANIPLPLGTVTILCIDLGTDMVPAISLAYEAAESDIMKRQPRNSQTDK
LVNERLISMAYGQIGMIQALGGFFTYFVILAENGFLPSRLLGIRLDWDDRTMNDLEDSYG
QEWTYEQRKVVEFTCHTAFFASIVVVQWADLIICKTRRNSVFQQGMKNKILIFGLLEETA
LAAFLSYCPGMGVALRMYPLKVTWWFCAFPYSLLIFIYDEVRKLILRRYPGGWVEKETYY

>sp|P51153|RAB13_HUMAN Ras-related protein Rab-13 OS=Homo sapiens OX=9606
GN=RAB13 PE=1 SV=1
MAKAYDHLFKLLLIGDSGVGKTCLIIRFAEDNFNNTYISTIGIDFKIRTVDIEGKKIKLQ
VWDTAGQERFKTITTAYYRGAMGIILVYDITDEKSFENIQNWMKSIKENASAGVERLLLG
NKCDMEAKRKVQKEQADKLAREHGIRFFETSAKSSMNVDEAFSSLARDILLKSGGRRSGN
GNKPPSTDLKTCDKKNTNKCSLG
>sp|P52209|6PGD_HUMAN 6-phosphogluconate dehydrogenase, decarboxylating OS=Homo
sapiens OX=9606 GN=PGD PE=1 SV=3
MAQADIALIGLAVMGQNLILNMNDHGFVVCAFNRTVSKVDDFLANEAKGTKVVGAQSLKE
MVSKLKKPRRIILLVKAGQAVDDFIEKLVPLLDTGDIIIDGGNSEYRDTTRRCRDLKAKG
ILFVGSGVSGGEEGARYGPSLMPGGNKEAWPHIKTIFQGIAAKVGTGEPCCDWVGDEGAG
HFVKMVHNGIEYGDMQLICEAYHLMKDVLGMAQDEMAQAFEDWNKTELDSFLIEITANIL
KFQDTDGKHLLPKIRDSAGQKGTGKWTAISALEYGVPVTLIGEAVFARCLSSLKDERIQA
SKKLKGPQKFQFDGDKKSFLEDIRKALYASKIISYAQGFMLLRQAATEFGWTLNYGGIAL
MWRGGCIIRSVFLGKIKDAFDRNPELQNLLLDDFFKSAVENCQDSWRRAVSTGVQAGIPM
PCFTTALSFYDGYRHEMLPASLIQAQRDYFGAHTYELLAKPGQFIHTNWTGHGGTVSSSS
YNA
>sp|P53672|CRBA2_HUMAN Beta-crystallin A2 OS=Homo sapiens OX=9606 GN=CRYBA2 PE=1
SV=3
MSSAPAPGPAPASLTLWDEEDFQGRRCRLLSDCANVCERGGLPRVRSVKVENGVWVAFEY
PDFQGQQFILEKGDYPRWSAWSGSSSHNSNQLLSFRPVLCANHNDSRVTLFEGDNFQGCK
FDLVDDYPSLPSMGWASKDVGSLKVSSGAWVAYQYPGYRGYQYVLERDRHSGEFCTYGEL
GTQAHTGQLQSIRRVQH
>sp|P53673|CRBA4_HUMAN Beta-crystallin A4 OS=Homo sapiens OX=9606 GN=CRYBA4 PE=1
SV=3
MTLQCTKSAGPWKMVVWDEDGFQGRRHEFTAECPSVLELGFETVRSLKVLSGAWVGFEHA
GFQGQQYILERGEYPSWDAWGGNTAYPAERLTSFRPAACANHRDSRLTIFEQENFLGKKG
ELSDDYPSLQAMGWEGNEVGSFHVHSGAWVCSQFPGYRGFQYVLECDHHSGDYKHFREWG
SHAPTFQVQSIRRIQQ
>sp|P53674|CRBB1_HUMAN Beta-crystallin B1 OS=Homo sapiens OX=9606 GN=CRYBB1 PE=1
SV=2
MSQAAKASASATVAVNPGPDTKGKGAPPAGTSPSPGTTLAPTTVPITSAKAAELPPGNYR
LVVFELENFQGRRAEFSGECSNLADRGFDRVRSIIVSAGPWVAFEQSNFRGEMFILEKGE
YPRWNTWSSSYRSDRLMSFRPIKMDAQEHKISLFEGANFKGNTIEIQGDDAPSLWVYGFS
DRVGSVKVSSGTWVGYQYPGYRGYQYLLEPGDFRHWNEWGAFQPQMQSLRRLRDKQWHLE
GSFPVLATEPPK
>sp|P55268|LAMB2_HUMAN Laminin subunit beta-2 OS=Homo sapiens OX=9606 GN=LAMB2
PE=1 SV=2
MELTSRERGRGQPLPWELRLGLLLSVLAATLAQAPAPDVPGCSRGSCYPATGDLLVGRAD
RLTASSTCGLNGPQPYCIVSHLQDEKKCFLCDSRRPFSARDNPHSHRIQNVVTSFAPQRR
AAWWQSENGIPAVTIQLDLEAEFHFTHLIMTFKTFRPAAMLVERSADFGRTWHVYRYFSY
DCGADFPGVPLAPPRHWDDVVCESRYSEIEPSTEGEVIYRVLDPAIPIPDPYSSRIQNLL
KITNLRVNLTRLHTLGDNLLDPRREIREKYYYALYELVVRGNCFCYGHASECAPAPGAPA
HAEGMVHGACICKHNTRGLNCEQCQDFYRDLPWRPAEDGHSHACRKCECHGHTHSCHFDM

AVYLASGNVSGGVCDGCQHNTAGRHCELCRPFFYRDPTKDLRDPAVCRSCDCDPMGSQDG
GRCDSHDDPALGLVSGQCRCKEHVVGTRCQQCRDGFFGLSISDRLGCRRCQCNARGTVPG
STPCDPNSGSCYCKRLVTGRGCDRCLPGHWGLSHDLLGCRPCDCDVGGALDPQCDEGTGQ
CHCRQHMVGRRCEQVQPGYFRPFLDHLIWEAEDTRGQVLDVVERLVTPGETPSWTGSGFV
RLQEGQTLEFLVASVPKAMDYDLLLRLEPQVPEQWAELELIVQRPGPVPAHSLCGHLVPK
DDRIQGTLQPHARYLIFPNPVCLEPGISYKLHLKLVRTGGSAQPETPYSGPGLLIDSLVL
LPRVLVLEMFSGGDAAALERQATFERYQCHEEGLVPSKTSPSEACAPLLISLSTLIYNGA
LPCQCNPQGSLSSECNPHGGQCLCKPGVVGRRCDLCAPGYYGFGPTGCQACQCSHEGALS
SLCEKTSGQCLCRTGAFGLRCDRCQRGQWGFPSCRPCVCNGHADECNTHTGACLGCRDHT
GGEHCERCIAGFHGDPRLPYGGQCRPCPCPEGPGSQRHFATSCHQDEYSQQIVCHCRAGY
TGLRCEACAPGHFGDPSRPGGRCQLCECSGNIDPMDPDACDPHTGQCLRCLHHTEGPHCA
HCKPGFHGQAARQSCHRCTCNLLGTNPQQCPSPDQCHCDPSSGQCPCLPNVQGPSCDRCA
PNFWNLTSGHGCQPCACHPSRARGPTCNEFTGQCHCRAGFGGRTCSECQELHWGDPGLQC
HACDCDSRGIDTPQCHRFTGHCSCRPGVSGVRCDQCARGFSGIFPACHPCHACFGDWDRV
VQDLAARTQRLEQRAQELQQTGVLGAFESSFWHMQEKLGIVQGIVGARNTSAASTAQLVE
ATEELRREIGEATEHLTQLEADLTDVQDENFNANHALSGLERDRLALNLTLRQLDQHLDL
LKHSNFLGAYDSIRHAHSQSAEAERRANTSALAVPSPVSNSASARHRTEALMDAQKEDFN
SKHMANQRALGKLSAHTHTLSLTDINELVCGAPGDAPCATSPCGGAGCRDEDGQPRCGGL
SCNGAAATADLALGRARHTQAELQRALAEGGSILSRVAETRRQASEAQQRAQAALDKANA
SRGQVEQANQELQELIQSVKDFLNQEGADPDSIEMVATRVLELSIPASAEQIQHLAGAIA
ERVRSLADVDAILARTVGDVRRAEQLLQDARRARSWAEDEKQKAETVQAALEEAQRAQGI
AQGAIRGAVADTRDTEQTLYQVQERMAGAERALSSAGERARQLDALLEALKLKRAGNSLA
ASTAEETAGSAQGRAQEAEQLLRGPLGDQYQTVKALAERKAQGVLAAQARAEQLRDEARD
LLQAAQDKLQRLQELEGTYEENERALESKAAQLDGLEARMRSVLQAINLQVQIYNTCQ
>sp|P58546|MTPN_HUMAN Myotrophin OS=Homo sapiens OX=9606 GN=MTPN PE=1 SV=2
MCDKEFMWALKNGDLDEVKDYVAKGEDVNRTLEGGRKPLHYAADCGQLEILEFLLLKGAD
INAPDKHHITPLLSAVYEGHVSCVKLLLSKGADKTVKGPDGLTAFEATDNQAIKALLQ
>sp|P60174|TPIS_HUMAN Triosephosphate isomerase OS=Homo sapiens OX=9606 GN=TPI1 PE=1 SV=4
MAPSRKFFVGGNWKMNGRKQSLGELIGTLNAAKVPADTEVVCAPPTAYIDFARQKLDPKI
AVAAQNCYKVTNGAFTGEISPGMIKDCGATWVVLGHSERRHVFGESDELIGQKVAHALAE
GLGVIACIGEKLDEREAGITEKVVFEQTKVIADNVKDWSKVVLAYEPVWAIGTGKTATPQ
QAQEVHEKLRGWLKSNVSDAVAQSTRIIYGGSVTGATCKELASQPDVDGFLVGGASLKPE
FVDIINAKQ
>sp|P60709|ACTB_HUMAN Actin, cytoplasmic 1 OS=Homo sapiens OX=9606 GN=ACTB PE=1 SV=1
MDDDIAALVVDNGSGMCKAGFAGDDAPRAVFPSIVGRPRHQGVMVGMGQKDSYVGDEAQS
KRGILTLKYPIEHGIVTNWDDMEKIWHHTFYNELRVAPEEHPVLLTEAPLNPKANREKMT
QIMFETFNTPAMYVAIQAVLSLYASGRTTGIVMDSGDGVTHTVPIYEGYALPHAILRLDL
AGRDLTDYLMKILTERGYSFTTTAEREIVRDIKEKLCYVALDFEQEMATAASSSSLEKSY
ELPDGQVITIGNERFRCPEALFQPSFLGMESCGIHETTFNSIMKCDVDIRKDLYANTVLS
GGTTMYPGIADRMQKEITALAPSTMKIKIIAPPERKYSVWIGGSILASLSTFQQMWISKQ
EYDESGPSIVHRKCF

>sp|P61978|HNRPK_HUMAN Heterogeneous nuclear ribonucleoprotein K OS=Homo sapiens OX=9606 GN=HNRNPK PE=1 SV=1
METEQPEETFPNTETNGEFGKRPAEDMEEEQAFKRSRNTDEMVELRILLQSKNAGAVIGK
GGKNIKALRTDYNASVSVPDSSGPERILSISADIETIGEILKKIIPTLEEGLQLPSPTAT
SQLPLESDAVECLNYQHYKGSDFDCELRLLIHQSLAGGIIGVKGAKIKELRENTQTTIKL
FQECCPHSTDRVVLIGGKPDRVVECIKIILDLISESPIKGRAQPYDPNFYDETYDYGGFT
MMFDDRRGRPVGFPMRGRGGFDRMPPGRGGRPMPPSRRDYDDMSPRRGPPPPPPGRGGRG
GSRARNLPLPPPPPPRGGDLMAYDRRGRPGDRYDGMVGFSADETWDSAIDTWSPSEWQMA
YEPQGGSGYDYSYAGGRGSYGDLGGPIITTQVTIPKDLAGSIIGKGGQRIKQIRHESGAS
IKIDEPLEGSEDRIITITGTQDQIQNAQYLLQNSVKQYSGKFF
>sp|P62258|1433E_HUMAN 14-3-3 protein epsilon OS=Homo sapiens OX=9606 GN=YWHAE PE=1 SV=1
MDDREDLVYQAKLAEQAERYDEMVESMKKVAGMDVELTVEERNLLSVAYKNVIGARRASW
RIISSIEQKEENKGGEDKLKMIREYRQMVETELKLICCDILDVLDKHLIPAANTGESKVF
YYKMKGDYHRYLAEFATGNDRKEAAENSLVAYKAASDIAMTELPPTHPIRLGLALNFSVF
YYEILNSPDRACRLAKAAFDDAIAELDTLSEESYKDSTLIMQLLRDNLTLWTSDMQGDGE
EQNKEALQDVEDENQ
>sp|P62736|ACTA_HUMAN Actin, aortic smooth muscle OS=Homo sapiens OX=9606 GN=ACTA2 PE=1 SV=1
MCEEEDSTALVCDNGSGLCKAGFAGDDAPRAVFPSIVGRPRHQGVMVGMGQKDSYVGDEA
QSKRGILTLKYPIEHGIITNWDDMEKIWHHSFYNELRVAPEEHPTLLTEAPLNPKANREK
MTQIMFETFNVPAMYVAIQAVLSLYASGRTTGIVLDSGDGVTHNVPIYEGYALPHAIMRL
DLAGRDLTDYLMKILTERGYSFVTTAEREIVRDIKEKLCYVALDFENEMATAASSSSLEK
SYELPDGQVITIGNERFRCPETLFQPSFIGMESAGIHETTYNSIMKCDIDIRKDLYANNV
LSGGTTMYPGIADRMQKEITALAPSTMKIKIIAPPERKYSVWIGGSILASLSTFQQMWIS
KQEYDEAGPSIVHRKCF
>sp|P62805|H4_HUMAN Histone H4 OS=Homo sapiens OX=9606 GN=H4C1 PE=1 SV=2
MSGRGKGGKGLGKGGAKRHRKVLRDNIQGITKPAIRRLARRGGVKRISGLIYEETRGVLK
VFLENVIRDAVTYTEHAKRKTVTAMDVVYALKRQGRTLYGFGG
>sp|P62937|PPIA_HUMAN Peptidyl-prolyl cis-trans isomerase A OS=Homo sapiens OX=9606 GN=PPIA PE=1 SV=2
MVNPTVFFDIAVDGEPLGRVSFELFADKVPKTAENFRALSTGEKGFGYKGSCFHRIIPGF
MCQGGDFTRHNGTGGKSIYGEKFEDENFILKHTGPGILSMANAGPNTNGSQFFICTAKTE
WLDGKHVVFGKVKEGMNIVEAMERFGSRNGKTSKKITIADCGQLE
>sp|P63104|1433Z_HUMAN 14-3-3 protein zeta/delta OS=Homo sapiens OX=9606 GN=YWHAZ PE=1 SV=1
MDKNELVQKAKLAEQAERYDDMAACMKSVTEQGAELSNEERNLLSVAYKNVVGARRSSWR
VVSSIEQKTEGAEKKQQMAREYREKIETELRDICNDVLSLLEKFLIPNASQAESKVFYLK
MKGDYYRYLAEVAAGDDKKGIVDQSQQAYQEAFEISKKEMQPTHPIRLGLALNFSVFYYE
ILNSPEKACSLAKTAFDEAIAELDTLSEESYKDSTLIMQLLRDNLTLWTSDTQGDEAEAG
EGGEN
>sp|P68104|EF1A1_HUMAN Elongation factor 1-alpha 1 OS=Homo sapiens OX=9606 GN=EEF1A1 PE=1 SV=1
MGKEKTHINIVVIGHVDSGKSTTTGHLIYKCGGIDKRTIEKFEKEAAEMGKGSFKYAWVL

DKLKAERERGITIDISLWKFETSKYYVTIIDAPGHRDFIKNMITGTSQADCAVLIVAAGV
GEFEAGISKNGQTREHALLAYTLGVKQLIVGVNKMDSTEPPYSQKRYEEIVKEVSTYIKK
IGYNPDTVAFVPISGWNGDNMLEPSANMPWFKGWKVTRKDGNASGTTLLEALDCILPPTR
PTDKPLRLPLQDVYKIGGIGTVPVGRVETGVLKPGMVVTFAPVNVTTEVKSVEMHHEALS
EALPGDNVGFNVKNVSVKDVRRGNVAGDSKNDPPMEAAGFTAQVIILNHPGQISAGYAPV
LDCHTAHIACKFAELKEKIDRRSGKKLEDGPKFLKSGDAAIVDMVPGKPMCVESFSDYPP
LGRFAVRDMRQTVAVGVIKAVDKKAAGAGKVTKSAQKAQKAK
>sp|P68363|TBA1B_HUMAN Tubulin alpha-1B chain OS=Homo sapiens OX=9606 GN=TUBA1B PE=1 SV=1
MRECISIHVGQAGVQIGNACWELYCLEHGIQPDGQMPSDKTIGGGDDSFNTFFSETGAGK
HVPRAVFVDLEPTVIDEVRTGTYRQLFHPEQLITGKEDAANNYARGHYTIGKEIIDLVLD
RIRKLADQCTGLQGFLVFHSFGGGTGSGFTSLLMERLSVDYGKKSKLEFSIYPAPQVSTA
VVEPYNSILTTHTTLEHSDCAFMVDNEAIYDICRRNLDIERPTYTNLNRLISQIVSSITA
SLRFDGALNVDLTEFQTNLVPYPRIHFPLATYAPVISAEKAYHEQLSVAEITNACFEPAN
QMVKCDPRHGKYMACCLLYRGDVVPKDVNAAIATIKTKRSIQFVDWCPTGFKVGINYQPP
TVVPGGDLAKVQRAVCMLSNTTAIAEAWARLDHKFDLMYAKRAFVHWYVGEGMEEGEFSE
AREDMAALEKDYEEVGVDSVEGEGEEEGEEY
>sp|P68366|TBA4A_HUMAN Tubulin alpha-4A chain OS=Homo sapiens OX=9606 GN=TUBA4A PE=1 SV=1
MRECISVHVGQAGVQMGNACWELYCLEHGIQPDGQMPSDKTIGGGDDSFTTFFCETGAGK
HVPRAVFVDLEPTVIDEIRNGPYRQLFHPEQLITGKEDAANNYARGHYTIGKEIIDPVLD
RIRKLSDQCTGLQGFLVFHSFGGGTGSGFTSLLMERLSVDYGKKSKLEFSIYPAPQVSTA
VVEPYNSILTTHTTLEHSDCAFMVDNEAIYDICRRNLDIERPTYTNLNRLISQIVSSITA
SLRFDGALNVDLTEFQTNLVPYPRIHFPLATYAPVISAEKAYHEQLSVAEITNACFEPAN
QMVKCDPRHGKYMACCLLYRGDVVPKDVNAAIAAIKTKRSIQFVDWCPTGFKVGINYQPP
TVVPGGDLAKVQRAVCMLSNTTAIAEAWARLDHKFDLMYAKRAFVHWYVGEGMEEGEFSE
AREDMAALEKDYEEVGIDSYEDEDEGEE
>sp|P68371|TBB4B_HUMAN Tubulin beta-4B chain OS=Homo sapiens OX=9606 GN=TUBB4B PE=1 SV=1
MREIVHLQAGQCGNQIGAKFWEVISDEHGIDPTGTYHGDSDLQLERINVYYNEATGGKYV
PRAVLVDLEPGTMDSVRSGPFGQIFRPDNFVFGQSGAGNNWAKGHYTEGAELVDSVLDVV
RKEAESCDCLQGFQLTHSLGGGTGSGMGTLLISKIREEYPDRIMNTFSVVPSPKVSDTVV
EPYNATLSVHQLVENTDETYCIDNEALYDICFRTLKLTTPTYGDLNHLVSATMSGVTTCL
RFPGQLNADLRKLAVNMVPFPRLHFFMPGFAPLTSRGSQQYRALTVPELTQQMFDAKNMM
AACDPRHGRYLTVAAVFRGRMSMKEVDEQMLNVQNKNSSYFVEWIPNNVKTAVCDIPPRG
LKMSATFIGNSTAIQELFKRISEQFTAMFRRKAFLHWYTGEGMDEMEFTEAESNMNDLVS
EYQQYQDATAEEEGEFEEEAEEEVA
>sp|P68431|H31_HUMAN Histone H3.1 OS=Homo sapiens OX=9606 GN=H3C1 PE=1 SV=2
MARTKQTARKSTGGKAPRKQLATKAARKSAPATGGVKKPHRYRPGTVALREIRRYQKSTE
LLIRKLPFQRLVREIAQDFKTDLRFQSSAVMALQEACEAYLVGLFEDTNLCAIHAKRVTI
MPKDIQLARRIRGERA
>sp|P68871|HBB_HUMAN Hemoglobin subunit beta OS=Homo sapiens OX=9606 GN=HBB PE=1 SV=2
MVHLTPEEKSAVTALWGKVNVDEVGGEALGRLLVVYPWTQRFFESFGDLSTPDAVMGNPK

VKAHGKKVLGAFSDGLAHLDNLKGTFATLSELHCDKLHVDPENFRLLGNVLVCVLAHHFG
KEFTPPVQAAYQKVVAGVANALAHKYH
>sp|P69905|HBA_HUMAN Hemoglobin subunit alpha OS=Homo sapiens OX=9606 GN=HBA1 PE=1 SV=2
MVLSPADKTNVKAAWGKVGAHAGEYGAEALERMFLSFPTTKTYFPHFDLSHGSAQVKGHG
KKVADALTNAVAHVDDMPNALSALSDLHAHKLRVDPVNFKLLSHCLLVTLAAHLPAEFTP
AVHASLDKFLASVSTVLTSKYR
>sp|P81605|DCD_HUMAN Dermcidin OS=Homo sapiens OX=9606 GN=DCD PE=1 SV=2
MRFMTLLFLTALAGALVCAYDPEAASAPGSGNPCHEASAAQKENAGEDPGLARQAPKPRK
QRSSLLEKGLDGAKKAVGGLGKLGKDAVEDLESVGKGAVHDVKDVLDSVL
>sp|Q00604|NDP_HUMAN Norrin OS=Homo sapiens OX=9606 GN=NDP PE=1 SV=1
MRKHVLAASFSMLSLLVIMGDTDSKTDSSFIMDSDPRRCMRHHYVDSISHPLYKCSSKMV
LLARCEGHCSQASRSEPLVSFSTVLKQPFRSSCHCCRPQTSKLKALRLRCSGGMRLTATY
RYILSCHCEECNS
>sp|Q01469|FABP5_HUMAN Fatty acid-binding protein 5 OS=Homo sapiens OX=9606 GN=FABP5 PE=1 SV=3
MATVQQLEGRWRLVDSKGFDEYMKELGVGIALRKMGAMAKPDCIITCDGKNLTIKTESTL
KTTQFSCTLGEKFEETTADGRKTQTVCNFTDGALVQHQEWDGKESTITRKLKDGKLVVEC
VMNNVTCTRIYEKVE
>sp|Q01546|K22O_HUMAN Keratin, type II cytoskeletal 2 oral OS=Homo sapiens OX=9606 GN=KRT76 PE=1 SV=2
MNRQVCKKSFSGRSQGFSGRSAVVSGSSRMSCVARSGGAGGGACGFRSGAGSFGSRSLYN
LGSNKSISISVAAGSSRAGGFGGGRSSCGFAGGYGGGFGGSYGGGFGGGRGVGSGFGGAG
GFGGAGGFGGPGVFGGPGSFGGPGGFGPGGFPGGIQEVIVNQSLLQPLNVEIDPQIGQVK
AQEREQIKTLNNKFASFIDKVRFLEQQNKVLETKWELLQQQTTGSGPSSLEPCFESYISF
LCKQLDSLLGERGNLEGELKSMQDLVEDFKKKYEDEINKRTAAENEFVGLKKDVDAAFMN
KVELQAKVDSLTDEVSFLRTLYEMELSQMQSHASDTSVVLSMDNNRCLDLGSIIAEVRAQ
YEEIAQRSKSEAEALYQTKLGELQTTAGRHGDDLRNTKSEIMELNRMIQRLRAEIENVKK
QNANLQTAIAEAEQRGEMALKDANAKLQDLQTALQKAKDDLARLLRDYQELMNVKLALDV
EIATYRKLLEGEECRMSGECQSAVCISVVSNVTSTSGSSGSSRGVFGGVSGSGSGGYKGG
SSSSSSSGYGVSGGSGSGYGGVSSGSTGGRGSSGSYQSSSSGSRLGGAGSISVSHSGMGS
SSGSIQTSGGSGYKSGGGGSTSIRFSQTTSSSQHSSTK
>sp|Q02413|DSG1_HUMAN Desmoglein-1 OS=Homo sapiens OX=9606 GN=DSG1 PE=1 SV=2
MDWSFFRVVAMLFIFLVVVEVNSEFRIQVRDYNTKNGTIKWHSIRRQKREWIKFAAACRE
GEDNSKRNPIAKIHSDCAANQQVTYRISGVGIDQPPYGIFVINQKTGEINITSIVDREVT
PFFIIYCRALNSMGQDLERPLELRVRVLDINDNPPVFSMATFAGQIEENSNANTLVMILN
ATDADEPNNLNSKIAFKIIRQEPSDSPMFIINRNTGEIRTMNNFLDREQYGQYALAVRGS
DRDGGADGMSAECECNIKILDVNDNIPYMEQSSYTIEIQENTLNSNLLEIRVIDLDEEFS
ANWMAVIFFISGNEGNWFEIEMNERTNVGILKVVKPLDYEAMQSLQLSIGVRNKAEFHHS
IMSQYKLKASAISVTVLNVIEGPVFRPGSKTYVVTGNMGSNDKVGDFVATDLDTGRPSTT
VRYVMGNNPADLLAVDSRTGKLTLKNKVTKEQYNMLGGKYQGTILSIDDNLQRTCTGTIN
INIQSFGNDDRTNTEPNTKITTNTGRQESTSSTNYDTSTTSTDSSQVYSSEPGNGAKDLL
SDNVHFGPAGIGLLIMGFLVLGLVPFLMICCDCGGAPRSAAGFEPVPECSDGAIHSWAVE
GPQPEPRDITTVIPQIPPDNANIIECIDNSGVYTNEYGGREMQDLGGGERMTGFELTEGV

KTSGMPEICQEYSGTLRRNSMRECREGGLNMNFMESYFCQKAYAYADEDEGRPSNDCLLI
YDIEGVGSPAGSVGCCSFIGEDLDDSFLDTLGPKFKKLADISLGKESYPDLDPSWPPQST
EPVCLPQETEPVVSGHPPISPHFGTTTVISESTYPSGPGVLHPKPILDPLGYGNVTVTES
YTTSDTLKPSVHVHDNRPASNVVVTERVVGPISGADLHGMLEMPDLRDGSNVIVTERVIA
PSSSLPTSLTIHHPRESSNVVVTERVIQPTSGMIGSLSMHPELANAHNVIVTERVVSGAG
VTGISGTTGISGGIGSSGLVGTSMGAGSGALSGAGISGGGIGLSSLGGTASIGHMRSSSD
HHFNQTIGSASPSTARSRITKYSTVQYSK
>sp|Q06830|PRDX1_HUMAN Peroxiredoxin-1 OS=Homo sapiens OX=9606 GN=PRDX1 PE=1 SV=1
MSSGNAKIGHPAPNFKATAVMPDGQFKDISLSDYKGKYVVFFFYPLDFTFVCPTEIIAFS
DRAEEFKKLNCQVIGASVDSHFCHLAWVNTPKKQGGLGPMNIPLVSDPKRTIAQDYGVLK
ADEGISFRGLFIIDDKGILRQITVNDLPVGRSVDETLRLVQAFQFTDKHGEVCPAGWKPG
SDTIKPDVQKSKEYFSKQK
>sp|Q08554|DSC1_HUMAN Desmocollin-1 OS=Homo sapiens OX=9606 GN=DSC1 PE=1 SV=2
MALASAAPGSIFCKQLLFSLLVLTLLCDACQKVYLRVPSHLQAETLVGKVNLEECLKSAS
LIRSSDPAFRILEDGSIYTTHDLILSSERKSFSIFLSDGQRREQQEIKVVLSARENKSPK
KRHTKDTALKRSKRRWAPIPASLMENSLGPFPQHVQQIQSDAAQNYTIFYSISGPGVDKE
PFNLFYIEKDTGDIFCTRSIDREKYEQFALYGYATTADGYAPEYPLPLIIKIEDDNDNAP
YFEHRVTIFTVPENCRSGTSVGKVTATDLDEPDTLHTRLKYKILQQIPDHPKHFSIHPDT
GVITTTTPFLDREKCDTYQLIMEVRDMGGQPFGLFNTGTITISLEDENDNPPSFTETSYV
TEVEENRIDVEILRMKVQDQDLPNTPHSKAVYKILQGNENGNFIISTDPNTNEGVLCVVK
PLNYEVNRQVILQVGVINEAQFSKAASSQTPTMCTTTVTVKIIDSDEGPECHPPVKVIQS
QDGFPAGQELLGYKALDPEISSGEGLRYQKLGDEDNWFEINQHTGDLRTLKVLDRESKFV
KNNQYNISVVAVDAVGRSCTGTLVVHLDDYNDHAPQIDKEVTICQNNEDFAVLKPVDPDG
PENGPPFQFFLDNSASKNWNIEEKDGKTAILRQRQNLDYNYYSVPIQIKDRHGLVATHML
TVRVCDCSTPSECRMKDKSTRDVRPNVILGRWAILAMVLGSVLLLCILFTCFCVTAKRTV
KKCFPEDIAQQNLIVSNTEGPGEEVTEANIRLPMQTSNICDTSMSVGTVGGQGIKTQQSF
EMVKGGYTLDSNKGGGHQTLESVKGVGQGDTGRYAYTDWQSFTQPRLGEKVYLCGQDEEH
KHCEDYVCSYNYEGKGSLAGSVGCCSDRQEEEGLEFLDHLEPKFRTLAKTCIKK
>sp|Q09666|AHNK_HUMAN Neuroblast differentiation-associated protein AHNAK OS=Homo sapiens OX=9606 GN=AHNAK PE=1 SV=2
MEKEETTRELLLPNWQGSGSHGLTIAQRDDGVFVQEVTQNSPAARTGVVKEGDQIVGATI
YFDNLQSGEVTQLLNTMGHHTVGLKLHRKGDRSPEPGQTWTREVFSSCSSEVVLSGDDEE
YQRIYTTKIKPRLKSEDGVEGDLGETQSRTITVTRRVTAYTVDVTGREGAKDIDISSPEF
KIKIPRHELTEISNVDVETQSGKTVIRLPSGSGAASPTGSAVDIRAGAISASGPELQGAG
HSKLQVTMPGIKVGGSGVNVNAKGLDLGGRGGVQVPAVDISSSLGGRAVEVQGPSLESGD
HGKIKFPTMKVPKFGVSTGREGQTPKAGLRVSAPEVSVGHKGGKPGLTIQAPQLEVSVPS
ANIEGLEGKLKGPQITGPSLEGDLGLKGAKPQGHIGVDASAPQIGGSITGPSVEVQAPDI
DVQGPGSKLNVPKMKVPKFSVSGAKGEETGIDVTLPTGEVTVPGVSGDVSLPEIATGGLE
GKMKGTKVKTPEMIIQKPKISMQDVDLSLGSPKLKGDIKVSAPGVQGDVKGPQVALKGSR
VDIETPNLEGTLTGPRLGSPSGKTGTCRISMSEVDLNVAAPKVKGGVDVTLPRVEGKVKV
PEVDVRGPKVDVSAPDVEAHGPEWNLKMPKMKMPTFSTPGAKGEGPDVHMTLPKGDISIS
GPKVNVEAPDVNLEGLGGKLKGPDVKLPDMSVKTPKISMPDVDLHVKGTKVKGEYDVTVP
KLEGELKGPKVDIDAPDVDVHGPDWHLKMPKMKMPKFSVPGFKAEGPEVDVNLPKADVDI

SGPKIDVTAPDVSIEEPEGKLKGPKFKMPEMNIKVPKISMPDVDLHLKGPNVKGEYDVTM
PKVESEIKVPDVELKSAKMDIDVPDVEVQGPDWHLKMPKMKMPKFSMPGFKAEGPEVDVN
LPKADVDISGPKVGVEVPDVNIEGPEGKLKGPKFKMPEMNIKAPKISMPDVDLHMKGPKV
KGEYDMTVPKLEGDLKGPKVDVSAPDVEMQGPDWNLKMPKIKMPKFSMPSLKGEGPEFDV
NLSKANVDISAPKVDTNAPDLSLEGPEGKLKGPKFKMPEMHFRAPKMSLPDVDLDLKGPK
MKGNVDISAPKIEGEMQVPDVDIRGPKVDIKAPDVEGQGLDWSLKIPKMKMPKFSMPSLK
GEGPEVDVNLPKADVVVSGPKVDIEAPDVSLEGPEGKLKGPKFKMPEMHFKTPKISMPDV
DLHLKGPKVKGDVDVSVPKVEGEMKVPDVEIKGPKMDIDAPDVEVQGPDWHLKMPKMKMP
KFSMPGFKGEGREVDVNLPKADIDVSGPKVDVEVPDVSLEGPEGKLKGPKFKMPEMHFKA
PKISMPDVDLNLKGPKLKGDVDVSLPEVEGEMKVPDVDIKGPKVDISAPDVDVHGPDWHL
KMPKVKMPKFSMPGFKGEGPEVDVKLPKADVDVSGPKMDAEVPDVNIEGPDAKLKGPKFK
MPEMSIKPQKISIPDVGLHLKGPKMKGDYDVTVPKVEGEIKAPDVDIKGPKVDINAPDVE
VHGPDWHLKMPKVKMPKFSMPGFKGEGPEVDMNLPKADLGVSGPKVDIDVPDVNLEAPEG
KLKGPKFKMPSMNIQTHKISMPDVGLNLKAPKLKTDVDVSLPKVEGDLKGPEIDVKAPKM
DVNVGDIDIEGPEGKLKGPKFKMPEMHFKAPKISMPDVDLHLKGPKVKGDMDVSVPKVEG
EMKVPDVDIKGPKVDIDAPDVEVHDPDWHLKMPKMKMPKFSMPGFKAEGPEVDVNLPKAD
IDVSGPSVDTDAPDLDIEGPEGKLKGSKFKMPKLNIKAPKVSMPDVDLNLKGPKLKGEID
ASVPELEGDLRGPQVDVKGPFVEAEVPDVDLECPDAKLKGPKFKMPEMHFKAPKISMPDV
DLHLKGPKVKGDADVSVPKLEGDLTGPSVGVEVPDVELECPDAKLKGPKFKMPDMHFKAP
KISMPDVDLHLKGPKVKGDVDVSVPKLEGDLTGPSVGVEVPDVELECPDAKLKGPKFKMP
EMHFKTPKISMPDVDLHLKGPKVKGDMDVSVPKVEGEMKVPDVDIKGPKMDIDAPDVDVH
GPDWHLKMPKMKMPKFSMPGFKAEGPEVDVNLPKADVVVSGPKVDVEVPDVSLEGPEGKL
KGPKLKMPEMHFKAPKISMPDVDLHLKGPKVKGDVDVSLPKLEGDLTGPSVDVEVPDVEL
ECPDAKLKGPKFKMPEMHFKTPKISMPDVNLNLKGPKVKGDMDVSVPKVEGEMKVPDVDI
RGPKVDIDAPDVDVHGPDWHLKMPKMKMPKFSMPGFKGEGPEVDVNLPKADVDVSGPKVD
VEVPDVSLEGPEGKLKGPKFKMPEMHFKTPKISMPDVDFNLKGPKIKGDVDVSAPKLEGE
LKGPELDVKGPKLDADMPEVAVEGPNGKWKTPKFKMPDMHFKAPKISMPDLDLHLKSPKA
KGEVDVDVPKLEGDLKGPHVDVSGPDIDIEGPEGKLKGPKFKMPDMHFKAPNISMPDVDL
NLKGPKIKGDVDVSVPEVEGKLEVPDMNIRGPKVDVNAPDVQAPDWHLKMPKMKMPKFSM
PGFKAEGPEVDVNLPKADVDISGPKVDIEGPDVNIEGPEGKLKGPKLKMPEMNIKAPKIS
MPDFDLHLKGPKVKGDVDVSLPKVEGDLKGPEVDIKGPKVDINAPDVGVQGPDWHLKMPK
VKMPKFSMPGFKGEGPDGDVKLPKADIDVSGPKVDIEGPDVNIEGPEGKLKGPKFKMPEM
NIKAPKISMPDIDLNLKGPKVKGDVDVSLPKVEGDLKGPEVDIKGPKVDIDAPDVDVHGP
DWHLKMPKIKMPKISMPGFKGEGPDVDVNLPKADIDVSGPKVDVECPDVNIEGPEGKWKS
PKFKMPEMHFKTPKISMPDIDLNLTGPKIKGDVDVTGPKVEGDLKGPEVDLKGPKVDIDV
PDVNVQGPDWHLKMPKMKMPKFSMPGFKAEGPEVDVNLPKADVDVSGPKVDVEGPDVNIE
GPEGKLKGPKFKMPEMNIKAPKIPMPDFDLHLKGPKVKGDVDISLPKVEGDLKGPEVDIR
GPQVDIDVPDVGVQGPDWHLKMPKVKMPKFSMPGFKGEGPDVDVNLPKADLDVSGPKVDI
DVPDVNIEGPEGKLKGPKFKMPEMNIKAPKISMPDIDLNLKGPKVKGDMDVSLPKVEGDM
KVPDVDIKGPKVDINAPDVDVQGPDWHLKMPKIKMPKISMPGFKGEGPEVDVNLPKADLD
VSGPKVDVDVPDVNIEGPDAKLKGPKFKMPEMNIKAPKISMPDLDLNLKGPKMKGEVDVS
LANVEGDLKGPALDIKGPKIDVDAPDIDIHGPDAKLKGPKLKMPDMHVNMPKISMPEIDL
NLKGSKLKGDVDVSGPKLEGDIKAPSLDIKGPEVDVSGPKLNIEGKSKKSRFKLPKFNFS
GSKVQTPEVDVKGKKPDIDITGPKVDINAPDVEVQGKVKGSKFKMPFLSISSPKVSMPDV

ELNLKSPKVKGDLDIAGPNLEGDFKGPKVDIKAPEVNLNAPDVDVHGPDWNLKMPKMKMP
KFSVSGLKAEGPDVAVDLPKGDINIEGPSMNIEGPDLNVEGPEGGLKGPKFKMPDMNIKA
PKISMPDIDLNLKGPKVKGDVDISLPKLEGDLKGPEVDIKGPKVDINAPDVDVHGPDWHL
KMPKVKMPKFSMPGFKGEGPEVDVTLPKADIDISGPNVDVDVPDVNIEGPDAKLKGPKFK
MPEMNIKAPKISMPDFDLNLKGPKMKGDVVVSLPKVEGDLKGPEVDIKGPKVDIDTPDIN
IEGSEGKFKGPKFKIPEMHLKAPKISMPDIDLNLKGPKVKGDVDVSLPKMEGDLKGPEVD
IKGPKVDINAPDVDVQGPDWHLKMPKVKMPKFSMPGFKGEGPDVDVNLPKADLDVSGPKV
DIDVPDVNIEGPEGKLKGPKFKMPEMNIKAPKISMPDIDLNLKGPKVKGDMDVSLPKVEG
DMQVPDLDIKGPKVDINAPDVDVRGPDWHLKMPKIKMPKISMPGFKGEGPEVDVNLPKAD
LDVSGPKVDVDVPDVNIEGPDAKLKGPKFKMPEMNIKAPKISMPDFDLHLKGPKVKGDVD
VSLPKMEGDLKAPEVDIKGPKVDIDAPDVDVHGPDWHLKMPKVKMPKFSMPGFKGEGPEV
DVNLPKADIDVSGPKVDIDTPDIDIHGPEGKLKGPKFKMPDLHLKAPKISMPEVDLNLKG
PKMKGDVDVSLPKVEGDLKGPEVDIKGPKVDIDVPDVDVQGPDWHLKMPKVKMPKFSMPG
FKGEGPDVDVNLPKADLDVSGPKVDIDVPDVNIEGPDAKLKGPKFKMPEMNIKAPKISMP
DFDLHLKGPKVKGDVDVSLPKVEGDLKGPEVDIKGPKVDIDAPDVDVHGPDWHLKMPKVK
MPKFSMPGFKGEGPDVDVTLPKADIEISGPKVDIDAPDVSIEGPDAKLKGPKFKMPEMNI
KAPKISMPDIDFNLKGPKVKGDVDVSLPKVEGDLKGPEIDIKGPSLDIDTPDVNIEGPEG
KLKGPKFKMPEMNIKAPKISMPDFDLHLKGPKVKGDVDVSLPKVESDLKGPEVDIEGPEG
KLKGPKFKMPDVHFKSPQISMSDIDLNLKGPKIKGDMDISVPKLEGDLKGPKVDVKGPKV
GIDTPDIDIHGPEGKLKGPKFKMPDLHLKAPKISMPEVDLNLKGPKVKGDMDISLPKVEG
DLKGPEVDIRDPKVDIDVPDVDVQGPDWHLKMPKVKMPKFSMPGFKGEGPDVDVNLPKAD
IDVSGPKVDVDVPDVNIEGPDAKLKGPKFKMPEMSIKAPKISMPDIDLNLKGPKVKGDVD
VTLPKVEGDLKGPEADIKGPKVDINTPDVDVHGPDWHLKMPKVKMPKFSMPGFKGEGPDV
DVSLPKADIDVSGPKVDVDIPDVNIEGPDAKLKGPKFKMPEINIKAPKISIPDVDLDLKG
PKVKGDFDVSVPKVEGTLKGPEVDLKGPRLDFEGPDAKLSGPSLKMPSLEISAPKVTAPD
VDLHLKAPKIGFSGPKLEGGEVDLKGPKVEAPSLDVHMDSPDINIEGPDVKIPKFKKPKF
GFGAKSPKADIKSPSLDVTVPEAELNLETPEISVGGKGKKSKFKMPKIHMSGPKIKAKKQ
GFDLNVPGGEIDASLKAPDVDVNIAGPDAALKVDVKSPKTKKTMFGKMYFPDVEFDIKSP
KFKAEAPLPSPKLEGELQAPDLELSLPAIHVEGLDIKAKAPKVKMPDVDISVPKIEGDLK
GPKVQANLGAPDINIEGLDAKVKTPSFGISAPQVSIPDVNVNLKGPKIKGDVPSVGLEGP
DVDLQGPEAKIKFPKFSMPKIGIPGVKMEGGGAEVHAQLPSLEGDLRGPDVKLEGPDVSL
KGPGVDLPSVNLSMPKVSGPDLDLNLKGPSLKGDLDASVPSMKVHAPGLNLSGVGGKMQV
GGDGVKVPGIDATTKLNVGAPDVTLRGPSLQGDLAVSGDIKCPKVSVGAPDLSLEASEGS
IKLPKMKLPQFGISTPGSDLHVNAKGPQVSGELKGPGVDVNLKGPRISAPNVDFNLEGPK
VKGSLGATGEIKGPTVGGGLPGIGVQGLEGNLQMPGIKSSGCDVNLPGVNVKLPTGQISG
PEIKGGLKGSEVGFHGAAPDISVKGPAFNMASPESDFGINLKGPKIKGGADVSGGVSAPD
ISLGEGHLSVKGSGGEWKGPQVSSALNLDTSKFAGGLHFSGPKVEGGVKGGQIGLQAPGL
SVSGPQGHLESGSGKVTFPKMKIPKFTFSGRELVGREMGVDVHFPKAEASIQAGAGDGEW
EESEVKLKKSKIKMPKFNFSKPKGKGGVTGSPEASISGSKGDLKSSKASLGSLEGEAEAE
ASSPKGKFSLFKSKKPRHRSNSFSDEREFSGPSTPTGTLEFEGGEVSLEGGKVKGKHGKL
KFGTFGGLGSKSKGHYEVTGSDDETGKLQGSGVSLASKKSRLSSSSSNDSGNKVGIQLPE
VELSVSTKKE
>sp|Q13509|TBB3_HUMAN Tubulin beta-3 chain OS=Homo sapiens OX=9606 GN=TUBB3 PE=1
SV=2

MREIVHIQAGQCGNQIGAKFWEVISDEHGIDPSGNYVGDSDLQLERISVYYNEASSHKYV
PRAILVDLEPGTMDSVRSGAFGHLFRPDNFIFGQSGAGNNWAKGHYTEGAELVDSVLDVV
RKECENCDCLQGFQLTHSLGGGTGSGMGTLLISKVREEYPDRIMNTFSVVPSPKVSDTVV
EPYNATLSIHQLVENTDETYCIDNEALYDICFRTLKLATPTYGDLNHLVSATMSGVTTSL
RFPGQLNADLRKLAVNMVPFPRLHFFMPGFAPLTARGSQQYRALTVPELTQQMFDAKNMM
AACDPRHGRYLTVATVFRGRMSMKEVDEQMLAIQSKNSSYFVEWIPNNVKVAVCDIPPRG
LKMSSTFIGNSTAIQELFKRISEQFTAMFRRKAFLHWYTGEGMDEMEFTEAESNMNDLVS
EYQQYQDATAEEEGEMYEDDEEESEAQGPK
>sp|Q13813|SPTN1_HUMAN Spectrin alpha chain, non-erythrocytic 1 OS=Homo sapiens
OX=9606 GN=SPTAN1 PE=1 SV=3
MDPSGVKVLETAEDIQERRQQVLDRYHRFKELSTLRRQKLEDSYRFQFFQRDAEELEKWI
QEKLQIASDENYKDPTNLQGKLQKHQAFEAEVQANSGAIVKLDETGNLMISEGHFASETI
RTRLMELHRQWELLLEKMREKGIKLLQAQKLVQYLRECEDVMDWINDKEAIVTSEELGQD
LEHVEVLQKKFEEFQTDMAAHEERVNEVNQFAAKLIQEQHPEEELIKTKQDEVNAAWQRL
KGLALQRQGKLFGAAEVQRFNRDVDETISWIKEKEQLMASDDFGRDLASVQALLRKHEGL
ERDLAALEDKVKALCAEADRLQQSHPLSATQIQVKREELITNWEQIRTLAAERHARLNDS
YRLQRFLADFRDLTSWVTEMKALINADELASDVAGAEALLDRHQEHKGEIDAHEDSFKSA
DESGQALLAAGHYASDEVREKLTVLSEERAALLELWELRRQQYEQCMDLQLFYRDTEQVD
NWMSKQEAFLLNEDLGDSLDSVEALLKKHEDFEKSLSAQEEKITALDEFATKLIQNNHYA
MEDVATRRDALLSRRNALHERAMRRRAQLADSFHLQQFFRDSDELKSWVNEKMKTATDEA
YKDPSNLQGKVQKHQAFEAELSANQSRIDALEKAGQKLIDVNHYAKDEVAARMNEVISLW
KKLLEATELKGIKLREANQQQQFNRNVEDIELWLYEVEGHLASDDYGKDLTNVQNLQKKH
ALLEADVAAHQDRIDGITIQARQFQDAGHFDAENIKKKQEALVARYEALKEPMVARKQKL
ADSLRLQQLFRDVEDEETWIREKEPIAASTNRGKDLIGVQNLLKKHQALQAEIAGHEPRI
KAVTQKGNAMVEEGHFAAEDVKAKLHELNQKWEALKAKASQRRQDLEDSLQAQQYFADAN
EAESWMREKEPIVGSTDYGKDEDSAEALLKKHEALMSDLSAYGSSIQALREQAQSCRQQV
APTDDETGKELVLALYDYQEKSPREVTMKKGDILTLLNSTNKDWWKVEVNDRQGFVPAAY
VKKLDPAQSASRENLLEEQGSIALRQEQIDNQTRITKEAGSVSLRMKQVEELYHSLLELG
EKRKGMLEKSCKKFMLFREANELQQWINEKEAALTSEEVGADLEQVEVLQKKFDDFQKDL
KANESRLKDINKVAEDLESEGLMAEEVQAVQQQEVYGMMPRDETDSKTASPWKSARLMVH
TVATFNSIKELNERWRSLQQLAEERSQLLGSAHEVQRFHRDADETKEWIEEKNQALNTDN
YGHDLASVQALQRKHEGFERDLAALGDKVNSLGETAERLIQSHPESAEDLQEKCTELNQA
WSSLGKRADQRKAKLGDSHDLQRFLSDFRDLMSWINGIRGLVSSDELAKDVTGAEALLER
HQEHRTEIDARAGTFQAFEQFGQQLLAHGHYASPEIKQKLDILDQERADLEKAWVQRRMM
LDQCLELQLFHRDCEQAENWMAAREAFLNTEDKGDSLDSVEALIKKHEDFDKAINVQEEK
IAALQAFADQLIAAGHYAKGDISSRRNEVLDRWRRLKAQMIEKRSKLGESQTLQQFSRDV
DEIEAWISEKLQTASDESYKDPTNIQSKHQKHQAFEAELHANADRIRGVIDMGNSLIERG
ACAGSEDAVKARLAALADQWQFLVQKSAEKSQKLKEANKQQNFNTGIKDFDFWLSEVEAL
LASEDYGKDLASVNNLLKKHQLLEADISAHEDRLKDLNSQADSLMTSSAFDTSQVKDKRD
TINGRFQKIKSMAASRRAKLNESHRLHQFFRDMDDEESWIKEKKLLVGSEDYGRDLTGVQ
NLRKKHKRLEAELAAHEPAIQGVLDTGKKLSDDNTIGKEEIQQRLAQFVEHWKELKQLAA
ARGQRLEESLEYQQFVANVEEEEAWINEKMTLVASEDYGDTLAAIQGLLKKHEAFETDFT
VHKDRVNDVCTNGQDLIKKNNHHEENISSKMKGLNGKVSDLEKAAAQRKAKLDENSAFLQ
FNWKADVVESWIGEKENSLKTDDYGRDLSSVQTLLTKQETFDAGLQAFQQEGIANITALK

DQLLAAKHVQSKAIEARHASLMKRWSQLLANSAARKKKLLEAQSHFRKVEDLFLTFAKKA
SAFNSWFENAEEDLTDPVRCNSLEEIKALREAHDAFRSSLSSAQADFNQLAELDRQIKSF
RVASNPYTWFTMEALEETWRNLQKIIKERELELQKEQRRQEENDKLRQEFAQHANAFHQW
IQETRTYLLDGSCMVEESGTLESQLEATKRKHQEIRAMRSQLKKIEDLGAAMEEALILDN
KYTEHSTVGLAQQWDQLDQLGMRMQHNLEQQIQARNTTGVTEEALKEFSMMFKHFDKDKS
GRLNHQEFKSCLRSLGYDLPMVEEGEPDPEFEAILDTVDPNRDGHVSLQEYMAFMISRET
ENVKSSEEIESAFRALSSEGKPYVTKEELYQNLTREQADYCVSHMKPYVDGKGRELPTAF
DYVEFTRSLFVN
>sp|Q14574|DSC3_HUMAN Desmocollin-3 OS=Homo sapiens OX=9606 GN=DSC3 PE=1 SV=3
MAAAGPRRSVRGAVCLHLLLTLVIFSRAGEACKKVILNVPSKLEADKIIGRVNLEECFRS
ADLIRSSDPDFRVLNDGSVYTARAVALSDKKRSFTIWLSDKRKQTQKEVTVLLEHQKKVS
KTRHTRETVLRRAKRRWAPIPCSMQENSLGPFPLFLQQVESDAAQNYTVFYSISGRGVDK
EPLNLFYIERDTGNLFCTRPVDREEYDVFDLIAYASTADGYSADLPLPLPIRVEDENDNH
PVFTEAIYNFEVLESSRPGTTVGVVCATDRDEPDTMHTRLKYSILQQTPRSPGLFSVHPS
TGVITTVSHYLDREVVDKYSLIMKVQDMDGQFFGLIGTSTCIITVTDSNDNAPTFRQNAY
EAFVEENAFNVEILRIPIEDKDLINTANWRVNFTILKGNENGHFKISTDKETNEGVLSVV
KPLNYEENRQVNLEIGVNNEAPFARDIPRVTALNRALVTVHVRDLDEGPECTPAAQYVRI
KENLAVGSKINGYKAYDPENRNGNGLRYKKLHDPKGWITIDEISGSIITSKILDREVETP
KNELYNITVLAIDKDDRSCTGTLAVNIEDVNDNPPEILQEYVVICKPKMGYTDILAVDPD
EPVHGAPFYFSLPNTSPEISRLWSLTKVNDTAARLSYQKNAGFQEYTIPITVKDRAGQAA
TKLLRVNLCECTHPTQCRATSRSTGVILGKWAILAILLGIALLFSVLLTLVCGVFGATKG
KRFPEDLAQQNLIISNTEAPGDDRVCSANGFMTQTTNNSSQGFCGTMGSGMKNGGQETIE
MMKGGNQTLESCRGAGHHHTLDSCRGGHTEVDNCRYTYSEWHSFTQPRLGEKLHRCNQNE
DRMPSQDYVLTYNYEGRGSPAGSVGCCSEKQEEDGLDFLNNLEPKFITLAEACTKR
>sp|Q16555|DPYL2_HUMAN Dihydropyrimidinase-related protein 2 OS=Homo sapiens
OX=9606 GN=DPYSL2 PE=1 SV=1
MSYQGKKNIPRITSDRLLIKGGKIVNDDQSFYADIYMEDGLIKQIGENLIVPGGVKTIEA
HSRMVIPGGIDVHTRFQMPDQGMTSADDFFQGTKAALAGGTTMIIDHVVPEPGTSLLAAF
DQWREWADSKSCCDYSLHVDISEWHKGIQEEMEALVKDHGVNSFLVYMAFKDRFQLTDCQ
IYEVLSVIRDIGAIAQVHAENGDIIAEEQQRILDLGITGPEGHVLSRPEEVEAEAVNRAI
TIANQTNCPLYITKVMSKSSAEVIAQARKKGTVVYGEPITASLGTDGSHYWSKNWAKAAA
FVTSPPLSPDPTTPDFLNSLLSCGDLQVTGSAHCTFNTAQKAVGKDNFTLIPEGTNGTEE
RMSVIWDKAVVTGKMDENQFVAVTSTNAAKVFNLYPRKGRIAVGSDADLVIWDPDSVKTI
SAKTHNSSLEYNIFEGMECRGSPLVVISQGKIVLEDGTLHVTEGSGRYIPRKPFPDFVYK
RIKARSRLAELRGVPRGLYDGPVCEVSVTPKTVTPASSAKTSPAKQQAPPVRNLHQSGFS
LSGAQIDDNIPRRTTQRIVAPPGGRANITSLG
>sp|Q5XKE5|K2C79_HUMAN Keratin, type II cytoskeletal 79 OS=Homo sapiens OX=9606
GN=KRT79 PE=1 SV=2
MRSSVSRQTYSTKGGFSSNSASGGSGSQARTSFSSVTVSRSSGSGGGAHCGPGTGGFGSR
SLYNLGGHKSISVSVAGGALLGRALGGFGFGSRAFMGQGAGRQTFGPACPPGGIQEVTVN
QSLLTPLHVEIDPEIQRVRTQEREQIKTLNNKFASFIDKVRFLEQQNKVLETKWALLQEQ
GQNLGVTRNNLEPLFEAYLGSMRSTLDRLQSERGRLDSELRNVQDLVEDFKNKYEDEINK
HTAAENEFVVLKKDVDAAYMGRMDLHGKVGTLTQEIDFLQQLYEMELSQVQTHVSNTNVV
LSMDNNRNLDLDSIIAEVKAQYELIAQRSRAEAEAWYQTKYEELQVTAGKHGDNLRDTKN

EIAELTRTIQRLQGEADAAKKQCQQLQTAIAEAEQRGELALKDAQKKLGDLDVALHQAKE
DLTRLLRDYQELMNVKLALDVEIATYRKLLESEESRMSGECPSAVSISVTGNSTTVCGGG
AASFGGGISLGGSGGATKGGFSTNVGYSTVKGGPVSAGTSILRKTTTVKTSSQRY
>sp|Q6KB66|K2C80_HUMAN Keratin, type II cytoskeletal 80 OS=Homo sapiens OX=9606 GN=KRT80 PE=1 SV=2
MACRSCVVGFSSLSSCEVTPVGSPRPGTSGWDSCRAPGPGFSSRSLTGCWSAGTISKVTV
NPGLLVPLDVKLDPAVQQLKNQEKEEMKALNDKFASLIGKVQALEQRNQLLETRWSFLQG
QDSAIFDLGHLYEEYQGRLQEELRKVSQERGQLEANLLQVLEKVEEFRIRYEDEISKRTD
MEFTFVQLKKDLDAECLHRTELETKLKSLESFVELMKTIYEQELKDLAAQVKDVSVTVGM
DSRCHIDLSGIVEEVKAQYDAVAARSLEEAEAYSRSQLEEQAARSAEYGSSLQSSRSEIA
DLNVRIQKLRSQILSVKSHCLKLEENIKTAEEQGELAFQDAKTKLAQLEAALQQAKQDMA
RQLRKYQELMNVKLALDIEIATYRKLVEGEEGRMDSPSATVVSAVQSRCKTAASRSGLSK
APSRKKKGSKGPVIKITEMSEKYFSQESEVSE
>sp|Q71U36|TBA1A_HUMAN Tubulin alpha-1A chain OS=Homo sapiens OX=9606 GN=TUBA1A PE=1 SV=1
MRECISIHVGQAGVQIGNACWELYCLEHGIQPDGQMPSDKTIGGGDDSFNTFFSETGAGK
HVPRAVFVDLEPTVIDEVRTGTYRQLFHPEQLITGKEDAANNYARGHYTIGKEIIDLVLD
RIRKLADQCTGLQGFLVFHSFGGGTGSGFTSLLMERLSVDYGKKSKLEFSIYPAPQVSTA
VVEPYNSILTTHTTLEHSDCAFMVDNEAIYDICRRNLDIERPTYTNLNRLIGQIVSSITA
SLRFDGALNVDLTEFQTNLVPYPRIHFPLATYAPVISAEKAYHEQLSVAEITNACFEPAN
QMVKCDPRHGKYMACCLLYRGDVVPKDVNAAIATIKTKRTIQFVDWCPTGFKVGINYQPP
TVVPGGDLAKVQRAVCMLSNTTAIAEAWARLDHKFDLMYAKRAFVHWYVGEGMEEGEFSE
AREDMAALEKDYEEVGVDSVEGEGEEEGEEY
>sp|Q7RTS7|K2C74_HUMAN Keratin, type II cytoskeletal 74 OS=Homo sapiens OX=9606 GN=KRT74 PE=1 SV=2
MSRQLNIKSSGDKGNFSVHSAVVPRKAVGSLASYCAAGRGAGAGFGSRSLYSLGGNRRIS
FNVAGGGVRAGGYGFRPGSGYGGGRASGFAGSMFGSVALGPACLSVCPPGGIHQVTVNKS
LLAPLNVELDPEIQKVRAQEREQIKVLNDKFASFIDKVRFLEQQNQVLETKWELLQQLDL
NNCKKNLEPILEGYISNLRKQLETLSGDRVRLDSELRSMRDLVEDYKKRYEVEINRRTTA
ENEFVVLKKDADAAYAVKVELQAKVDSLDKEIKFLKCLYDAEIAQIQTHASETSVILSMD
NNRDLDLDSIIAEVRMHYEEIALKSKAEAEALYQTKIQELQLAASRHGDDLKHTRSEMVE
LNRLIQRIRCEIGNVKKQRASLETAIADAEQRGDNALKDAQAKLDELEGALHQAKEELAR
MLREYQELMSLKLALDMEIATYRKLLEGEECRMSGENPSSVSISVISSSSYSYHHPSSAG
VDLGASAVAGSSGSTQSGQTKTTEARGGDLKDTQGKSTPASIPARKATR
>sp|Q7Z794|K2C1B_HUMAN Keratin, type II cytoskeletal 1b OS=Homo sapiens OX=9606 GN=KRT77 PE=1 SV=3
MSHQFSSQSAFSSMSRRVYSTSSSAGSGGGSPAVGSVCYARGRCGGGGYGIHGRGFGSRS
LYNLGGSRSISINLMGRSTSGFCQGGGVGGFGGGRGFGVGSTGAGGFGGGGFGGAGFGTS
NFGLGGFGPYCPPGGIQEVTINQSLLEPLHLEVDPEIQRIKTQEREQIMVLNNKFASFID
KVRFLEQQNQVLQTKWELLQQVNTSTGTNNLEPLLENYIGDLRRQVDLLSAEQMRQNAEV
RSMQDVVEDYKSKYEDEINKRTGSENDFVVLKKDVDAAYVSKVDLESRVDTLTGEVNFLK
YLFLTELSQVQTHISDTNVILSMDNNRSLDLDSIIDAVRTQYELIAQRSKDEAEALYQTK
YQELQITAGRHGDDLKNSKMEIAELNRTVQRLQAEISNVKKQIEQMQSLISDAEERGEQA
LQDAWQKLQDLEEALQQSKEELARLLRDYQAMLGVKLSLDVEIATYRQLLEGEESRMSGE

LQSHVSISVQNSQVSVNGGAGGGGSYGSGGYGGGSGGGYGGGRSYRGGGARGRSGGGYGS
GCGGGGGSYGGSGRSGRGSSRVQIIQTSTNTSHRRILE
>sp|Q86TH1|ATL2_HUMAN ADAMTS-like protein 2 OS=Homo sapiens OX=9606 GN=ADAMTSL2 PE=1 SV=1
MDGRWQCSCWAWFLLVLAVVAGDTVSTGSTDNSPTSNSLEGGTDATAFWWGEWTKWTACS
RSCGGGVTSQERHCLQQRRKSVPGPGNRTCTGTSKRYQLCRVQECPPDGRSFREEQCVSF
NSHVYNGRTHQWKPLYPDDYVHISSKPCDLHCTTVDGQRQLMVPARDGTSCKLTDLRGVC
VSGKCEPIGCDGVLFSTHTLDKCGICQGDGSSCTHVTGNYRKGNAHLGYSLVTHIPAGAR
DIQIVERKKSADVLALADEAGYYFFNGNYKVDSPKNFNIAGTVVKYRRPMDVYETGIEYI
VAQGPTNQGLNVMVWNQNGKSPSITFEYTLLQPPHESRPQPIYYGFSESAESQGLDGAGL
MGFVPHNGSLYGQASSERLGLDNRLFGHPGLDMELGPSQGQETNEVCEQAGGGACEGPPR
GKGFRDRNVTGTPLTGDKDDEEVDTHFASQEFFSANAISDQLLGAGSDLKDFTLNETVNS
IFAQGAPRSSLAESFFVDYEENEGAGPYLLNGSYLELSSDRVANSSSEAPFPNVSTSLLT
SAGNRTHKARTRPKARKQGVSPADMYRWKLSSHEPCSATCTTGVMSAYAMCVRYDGVEVD
DSYCDALTRPEPVHEFCAGRECQPRWETSSWSECSRTCGEGYQFRVVRCWKMLSPGFDSS
VYSDLCEAAEAVRPEERKTCRNPACGPQWEMSEWSECTAKCGERSVVTRDIRCSEDEKLC
DPNTRPVGEKNCTGPPCDRQWTVSDWGPCSGSCGQGRTIRHVYCKTSDGRVVPESQCQME
TKPLAIHPCGDKNCPAHWLAQDWERCNTTCGRGVKKRLVLCMELANGKPQTRSGPECGLA
KKPPEESTCFERPCFKWYTSPWSECTKTCGVGVRMRDVKCYQGTDIVRGCDPLVKPVGRQ
ACDLQPCPTEPPDDSCQDQPGTNCALAIKVNLCGHWYYSKACCRSCRPPHS
>sp|Q8IZT6|ASPM_HUMAN Abnormal spindle-like microcephaly-associated protein OS=Homo sapiens OX=9606 GN=ASPM PE=1 SV=2
MANRRVGRGCWEVSPTERRPPAGLRGPAAEEEASSPPVLSLSHFCRSPFLCFGDVLLGAS
RTLSLALDNPNEEVAEVKISHFPAADLGFSVSQRCFVLQPKEKIVISVNWTPLKEGRVRE
IMTFLVNDVLKHQAILLGNAEEQKKKKRSLWDTIKKKKISASTSHNRRVSNIQNVNKTFS
VSQKVDRVRSPLQACENLAMNEGGPPTENNSLILEENKIPISPISPAFNECHGATCLPLS
VRRSTTYSSLHASENRELLNVHSANVSKVSFNEKAVTETSFNSVNVNGQRGENSKLSLTP
NCSSTLNITQSQIHFLSPDSFVNNSHGANNELELVTCLSSDMFMKDNSQPVHLESTIAHE
IYQKILSPDSFIKDNYGLNQDLESESVNPILSPNQFLKDNMAYMCTSQQTCKVPLSNENS
QVPQSPEDWRKSEVSPRIPECQGSKSPKAIFEELVEMKSNYYSFIKQNNPKFSAVQDISS
HSHNKQPKRRPILSATVTKRKATCTRENQTEINKPKAKRCLNSAVGEHEKVINNQKEKED
FHSYLPIIDPILSKSKSYKNEVTPSSTTASVARKRKSDGSMEDANVRVAITEHTEVREIK
RIHFSPSEPKTSAVKKTKNVTTPISKRISNREKLNLKKKTDLSIFRTPISKTNKRTKPII
AVAQSSLTFIKPLKTDIPRHPMPFAAKNMFYDERWKEKQEQGFTWWLNFILTPDDFTVKT
NISEVNAATLLLGIENQHKISVPRAPTKEEMSLRAYTARCRLNRLRRAACRLFTSEKMVK
AIKKLEIEIEARRLIVRKDRHLWKDVGERQKVLNWLLSYNPLWLRIGLETTYGELISLED
NSDVTGLAMFILNRLLWNPDIAAEYRHPTVPHLYRDGHEEALSKFTLKKLLLLVCFLDYA
KISRLIDHDPCLFCKDAEFKASKEILLAFSRDFLSGEGDLSRHLGLLGLPVNHVQTPFDE
FDFAVTNLAVDLQCGVRLVRTMELLTQNWDLSKKLRIPAISRLQKMHNVDIVLQVLKSRG
IELSDEHGNTILSKDIVDRHREKTLRLLWKIAFAFQVDISLNLDQLKEEIAFLKHTKSIK
KTISLLSCHSDDLINKKKGKRDSGSFEQYSENIKLLMDWVNAVCAFYNKKVENFTVSFSD
GRVLCYLIHHYHPCYVPFDAICQRTTQTVECTQTGSVVLNSSSESDDSSLDMSLKAFDHE
NTSELYKELLENEKKNFHLVRSAVRDLGGIPAMINHSDMSNTIPDEKVVITYLSFLCARL
LDLRKEIRAARLIQTTWRKYKLKTDLKRHQEREKAARIIQLAVINFLAKQRLRKRVNAAL

VIQKYWRRVLAQRKLLMLKKEKLEKVQNKAASLIQGYWRRYSTRQRFLKLKYYSIILQSR
IRMIIAVTSYKRYLWATVTIQRHWRAYLRRKQDQQRYEMLKSSTLIIQSMFRKWKQRKMQ
SQVKATVILQRAFREWHLRKQAKEENSAIIIQSWYRMHKELRKYIYIRSCVVIIQKRFRC
FQAQKLYKRRKESILTIQKYYKAYLKGKIERTNYLQKRAAAIQLQAAFRRLKAHNLCRQI
RAACVIQSYWRMRQDRVRFLNLKKTIIKFQAHVRKHQQRQKYKKMKKAAVIIQTHFRAYI
FAMKVLASYQKTRSAVIVLQSAYRGMQARKMYIHILTSVIKIQSYYRAYVSKKEFLSLKN
ATIKLQSTVKMKQTRKQYLHLRAAALFIQQCYRSKKIAAQKREEYMQMRESCIKLQAFVR
GYLVRKQMRLQRKAVISLQSYFRMRKARQYYLKMYKAIIVIQNYYHAYKAQVNQRKNFLQ
VKKAATCLQAAYRGYKVRQLIKQQSIAALKIQSAFRGYNKRVKYQSVLQSIIKIQRWYRA
YKTLHDTRTHFLKTKAAVISLQSAYRGWKVRKQIRREHQAALKIQSAFRMAKAQKQFRLF
KTAALVIQQNFRAWTAGRKQCMEYIELRHAVLVLQSMWKGKTLRRQLQRQHKCAIIIQSY
YRMHVQQKKWKIMKKAALLIQKYYRAYSIGREQNHLYLKTKAAVVTLQSAYRGMKVRKRI
KDCNKAAVTIQSKYRAYKTKKKYATYRASAIIIQRWYRGIKITNHQHKEYLNLKKTAIKI
QSVYRGIRVRRHIQHMHRAATFIKAMFKMHQSRISYHTMRKAAIVIQVRCRAYYQGKMQR
EKYLTILKAVKVLQASFRGVRVRRTLRKMQTAATLIQSNYRRYRQQTYFNKLKKITKTVQ
QRYWAMKERNIQFQRYNKLRHSVIYIQAIFRGKKARRHLKMMHIAATLIQRRFRTLMMRR
RFLSLKKTAILIQRKYRAHLCTKHHLQFLQVQNAVIKIQSSYRRWMIRKRMREMHRAATF
IQSTFRMHRLHMRYQALKQASVVIQQQYQANRAAKLQRQHYLRQRHSAVILQAAFRGMKT
RRHLKSMHSSATLIQSRFRSLLVRRRFISLKKATIFVQRKYRATICAKHKLYQFLHLRKA
AITIQSSYRRLMVKKKLQEMQRAAVLIQATFRMYRTYITFQTWKHASILIQQHYRTYRAA
KLQRENYIRQWHSAVVIQAAYKGMKARQLLREKHKASIVIQSTYRMYRQYCFYQKLQWAT
KIIQEKYRANKKKQKVFQHNELKKETCVQAGFQDMNIKKQIQEQHQAAIIIQKHCKAFKI
RKHYLHLRATVVSIQRRYRKLTAVRTQAVICIQSYYRGFKVRKDIQNMHRAATLIQSFYR
MHRAKVDYETKKTAIVVIQNYYRLYVRVKTERKNFLAVQKSVRTIQAAFRGMKVRQKLKN
VSEEKMAAIVNQSALCCYRSKTQYEAVQSEGVMIQEWYKASGLACSQEAEYHSQSRAAVT
IQKAFCRMVTRKLETQKCAALRIQFFLQMAVYRRRFVQQKRAAITLQHYFRTWQTRKQFL
LYRKAAVVLQNHYRAFLSAKHQRQVYLQIRSSVIIIQARSKGFIQKRKFQEIKNSTIKIQ
AMWRRYRAKKYLCKVKAACKIQAWYRCWRAHKEYLAILKAVKIIQGCFYTKLERTRFLNV
RASAIIIQRKWRAILPAKIAHEHFLMIKRHRAACLIQAHYRGYKGRQVFLRQKSAALIIQ
KYIRAREAGKHERIKYIEFKKSTVILQALVRGWLVRKRFLEQRAKIRLLHFTAAAYYHLN
AVRIQRAYKLYLAVKNANKQVNSVICIQRWFRARLQEKRFIQKYHSIKKIEHEGQECLSQ
RNRAASVIQKAVRHFLLRKKQEKFTSGIIKIQALWRGYSWRKKNDCTKIKAIRLSLQVVN
REIREENKLYKRTALALHYLLTYKHLSAILEALKHLEVVTRLSPLCCENMAQSGAISKIF
VLIRSCNRSIPCMEVIRYAVQVLLNVSKYEKTTSAVYDVENCIDILLELLQIYREKPGNK
VADKGGSIFTKTCCLLAILLKTTNRASDVRSRSKVVDRIYSLYKLTAHKHKMNTERILYK
QKKNSSISIPFIPETPVRTRIVSRLKPDWVLRRDNMEEITNPLQAIQMVMDTLGIPY
>sp|Q8N1N4|K2C78_HUMAN Keratin, type II cytoskeletal 78 OS=Homo sapiens OX=9606 GN=KRT78 PE=1 SV=2
MSLSPCRAQRGFSARSACSARSRGRSRGGFSSRGGFSSRSLNSFGGCLEGSRGSTWGSGG
RLGVRFGEWSGGPGLSLCPPGGIQEVTINQNLLTPLKIEIDPQFQVVRTQETQEIRTLNN
QFASFIDKVRFLEQQNKVLETKWHLLQQQGLSGSQQGLEPVFEACLDQLRKQLEQLQGER
GALDAELKACRDQEEEYKSKYEEEAHRRATLENDFVVLKKDVDGVFLSKMELEGKLEALR
EYLYFLKHLNEEELGQLQTQASDTSVVLSMDNNRYLDFSSIITEVRARYEEIARSSKAEA
EALYQTKYQELQVSAQLHGDRMQETKVQISQLHQEIQRLQSQTENLKKQNASLQAAITDA

EQRGELALKDAQAKVDELEAALRMAKQNLARLLCEYQELTSTKLSLDVEIATYRRLLEGE
ECRMSGECTSQVTISSVGGSAVMSGGVGGGLGSTCGLGSGKGSPGSCCTSIVTGGSNIIL
GSGKDPVLDSCSVSGSSAGSSCHTILKKTVESSLKTSITY
>sp|Q8N4T0|CBPA6_HUMAN Carboxypeptidase A6 OS=Homo sapiens OX=9606 GN=CPA6 PE=1 SV=2
MKCLGKRRGQAAAFLPLCWLFLKILQPGHSHLYNNRYAGDKVIRFIPKTEEEAYALKKIS
YQLKVDLWQPSSISYVSEGTVTDVHIPQNGSRALLAFLQEANIQYKVLIEDLQKTLEKGS
SLHTQRNRRSLSGYNYEVYHSLEEIQNWMHHLNKTHSGLIHMFSIGRSYEGRSLFILKLG
RRSRLKRAVWIDCGIHAREWIGPAFCQWFVKEALLTYKSDPAMRKMLNHLYFYIMPVFNV
DGYHFSWTNDRFWRKTRSRNSRFRCRGVDANRNWKVKWCDEGASMHPCDDTYCGPFPESE
PEVKAVANFLRKHRKHIRAYLSFHAYAQMLLYPYSYKYATIPNFRCVESAAYKAVNALQS
VYGVRYRYGPASTTLYVSSGSSMDWAYKNGIPYAFAFELRDTGYFGFLLPEMLIKPTCTE
TMLAVKNITMHLLKKCP
>sp|Q8NCH0|CHSTE_HUMAN Carbohydrate sulfotransferase 14 OS=Homo sapiens OX=9606 GN=CHST14 PE=1 SV=2
MFPRPLTPLAAPNGAEPLGRALRRAPLGRARAGLGGPPLLLPSMLMFAVIVASSGLLLMI
ERGILAEMKPLPLHPPGREGTAWRGKAPKPGGLSLRAGDADLQVRQDVRNRTLRAVCGQP
GMPRDPWDLPVGQRRTLLRHILVSDRYRFLYCYVPKVACSNWKRVMKVLAGVLDSVDVRL
KMDHRSDLVFLADLRPEEIRYRLQHYFKFLFVREPLERLLSAYRNKFGEIREYQQRYGAE
IVRRYRAGAGPSPAGDDVTFPEFLRYLVDEDPERMNEHWMPVYHLCQPCAVHYDFVGSYE
RLEADANQVLEWVRAPPHVRFPARQAWYRPASPESLHYHLCSAPRALLQDVLPKYILDFS
LFAYPLPNVTKEACQQ
>sp|Q8NDZ4|DIK2A_HUMAN Divergent protein kinase domain 2A OS=Homo sapiens OX=9606 GN=DIPK2A PE=1 SV=1
MWRLVPPKLGRLSRSLKLAALGSLLVLMVLHSPSLLASWQRNELTDRRFLQLNKCPACFG
TSWCRRFLNGQVVFEAWGRLRLLDFLNVKNVYFAQYGEPREGGRRRVVLKRLGSQRELAQ
LDQSICKRATGRPRCDLLQAMPRTEFARLNGDVRLLTPEAVEGWSDLVHCPSQRLLDRLV
RRYAETKDSGSFLLRNLKDSERMQLLLTLAFNPEPLVLQSFPSDEGWPFAKYLGACGRMV
AVNYVGEELWSYFNAPWEKRVDLAWQLMEIAEQLTNNDFEFALYLLDVSFDNFAVGPRDG
KVIIVDAENVLVADKRLIRQNKPENWDVWYESKFDDCDKEACLSFSKEILCARATVDHNY
YAVCQNLLSRHATWRGTSGGLLHDPPSEIAKDGRLEALLDECANPKKRYGRFQAAKELRE
YLAQLSNNVR
>sp|Q8TER0|SNED1_HUMAN Sushi, nidogen and EGF-like domain-containing protein 1 OS=Homo sapiens OX=9606 GN=SNED1 PE=1 SV=2
MRHGVAWALLVAAALGLGARGVRGAVALADFYPFGAERGDAVTPKQDDGGSGLRPLSVPF
PFFGAEHSGLYVNNNGIISFLKEVSQFTPVAFPIAKDRCVVAAFWADVDNRRAGDVYYRE
ATDPAMLRRATEDVRHYFPELLDFNATWVFVATWYRVTFFGGSSSSPVNTFQTVLITDGK
LSFTIFNYESIVWTTGTHASSGGNATGLGGIAAQAGFNAGDGQRYFSIPGSRTADMAEVE
TTTNVGVPGRWAFRIDDAQVRVGGCGHTTSVCLALRPCLNGGKCIDDCVTGNPSYTCSCL
SGFTGRRCHLDVNECASQPCQNGGTCTHGINSFRCQCPAGFGGPTCETAQSPCDTKECQH
GGQCQVENGSAVCVCQAGYTGAACEMDVDDCSPDPCLNGGSCVDLVGNYTCLCAEPFKGL
RCETGDHPVPDACLSAPCHNGGTCVDADQGYVCECPEGFMGLDCRERVPDDCECRNGGRC
LGANTTLCQCPLGFFGLLCEFEITAMPCNMNTQCPDGGYCMEHGGSYLCVCHTDHNASHS
LPSPCDSDPCFNGGSCDAHDDSYTCECPRGFHGKHCEKARPHLCSSGPCRNGGTCKEAGG

EYHCSCPYRFTGRHCEIGKPDSCASGPCHNGGTCFHYIGKYKCDCPPGFSGRHCEIAPSP
CFRSPCVNGGTCEDRDTDFFCHCQAGYMGRRCQAEVDCGPPEEVKHATLRFNGTRLGAVA
LYACDRGYSLSAPSRIRVCQPHGVWSEPPQCLEIDECRSQPCLHGGSCQDRVAGYLCLCS
TGYEGAHCELERDECRAHPCRNGGSCRNLPGAYVCRCPAGFVGVHCETEVDACDSSPCQH
GGRCESGGGAYLCVCPESFFGYHCETVSDPCFSSPCGGRGYCLASNGSHSCTCKVGYTGE
DCAKELFPPTALKMERVEESGVSISWNPPNGPAARQMLDGYAVTYVSSDGSYRRTDFVDR
TRSSHQLQALAAGRAYNISVFSVKRNSNNKNDISRPAVLLARTRPRPVEGFEVTNVTAST
ISVQWALHRIRHATVSGVRVSIRHPEALRDQATDVDRSVDRFTFRALLPGKRYTIQLTTL
SGLRGEEHPTESLATAPTHVWTRPLPPANLTAARVTATSAHVVWDAPTPGSLLEAYVINV
TTSQSTKSRYVPNGKLASYTVRDLLPGRRYQLSVIAVQSTELGPQHSEPAHLYIITSPRD
GADRRWHQGGHHPRVLKNRPPPARLPELRLLNDHSAPETPTQPPRFSELVDGRGRVSARF
GGSPSKAATVRSQPTASAQLENMEEAPKRVSLALQLPEHGSKDIGNVPGNCSENPCQNGG
TCVPGADAHSCDCGPGFKGRRCELACIKVSRPCTRLFSETKAFPVWEGGVCHHVYKRVYR
VHQDICFKESCESTSLKKTPNRKQSKSQTLEKS
>sp|Q8WZ42|TITIN_HUMAN Titin OS=Homo sapiens OX=9606 GN=TTN PE=1 SV=4
MTTQAPTFTQPLQSVVVLEGSTATFEAHISGFPVPEVSWFRDGQVISTSTLPGVQISFSD
GRAKLTIPAVTKANSGRYSLKATNGSGQATSTAELLVKAETAPPNFVQRLQSMTVRQGSQ
VRLQVRVTGIPTPVVKFYRDGAEIQSSLDFQISQEGDLYSLLIAEAYPEDSGTYSVNATN
SVGRATSTAELLVQGEEEVPAKKTKTIVSTAQISESRQTRIEKKIEAHFDARSIATVEMV
IDGAAGQQLPHKTPPRIPPKPKSRSPTPPSIAAKAQLARQQSPSPIRHSPSPVRHVRAPT
PSPVRSVSPAARISTSPIRSVRSPLLMRKTQASTVATGPEVPPPWKQEGYVASSSEAEMR
ETTLTTSTQIRTEERWEGRYGVQEQVTISGAAGAAASVSASASYAAEAVATGAKEVKQDA
DKSAAVATVVAAVDMARVREPVISAVEQTAQRTTTTAVHIQPAQEQVRKEAEKTAVTKVV
VAADKAKEQELKSRTKEVITTKQEQMHVTHEQIRKETEKTFVPKVVISAAKAKEQETRIS
EEITKKQKQVTQEAIRQETEITAASMVVVATAKSTKLETVPGAQEETTTQQDQMHLSYEK
IMKETRKTVVPKVIVATPKVKEQDLVSRGREGITTKREQVQITQEKMRKEAEKTALSTIA
VATAKAKEQETILRTRETMATRQEQIQVTHGKVDVGKKAEAVATVVAAVDQARVREPREP
GHLEESYAQQTTLEYGYKERISAAKVAEPPQRPASEPHVVPKAVKPRVIQAPSETHIKTT
DQKGMHISSQIKKTTDLTTERLVHVDKRPRTASPHFTVSKISVPKTEHGYEASIAGSAIA
TLQKELSATSSAQKITKSVKAPTVKPSETRVRAEPTPLPQFPFADTPDTYKSEAGVEVKK
EVGVSITGTTVREERFEVLHGREAKVTETARVPAPVEIPVTPPTLVSGLKNVTVIEGESV
TLECHISGYPSPTVTWYREDYQIESSIDFQITFQSGIARLMIREAFAEDSGRFTCSAVNE
AGTVSTSCYLAVQVSEEFEKETTAVTEKFTTEEKRFVESRDVVMTDTSLTEEQAGPGEPA
APYFITKPVVQKLVEGGSVVFGCQVGGNPKPHVYWKKSGVPLTTGYRYKVSYNKQTGECK
LVISMTFADDAGEYTIVVRNKHGETSASASLLEEADYELLMKSQQEMLYQTQVTAFVQEP
KVGETAPGFVYSEYEKEYEKEQALIRKKMAKDTVVVRTYVEDQEFHISSFEERLIKEIEY
RIIKTTLEELLEEDGEEKMAVDISESEAVESGFDSRIKNYRILEGMGVTFHCKMSGYPLP
KIAWYKDGKRIKHGERYQMDFLQDGRASLRIPVVLPEDEGIYTAFASNIKGNAICSGKLY
VEPAAPLGAPTYIPTLEPVSRIRSLSPRSVSRSPIRMSPARMSPARMSPARMSPARMSPG
RRLEETDESQLERLYKPVFVLKPVSFKCLEGQTARFDLKVVGRPMPETFWFHDGQQIVND
YTHKVVIKEDGTQSLIIVPATPSDSGEWTVVAQNRAGRSSISVILTVEAVEHQVKPMFVE
KLKNVNIKEGSRLEMKVRATGNPNPDIVWLKNSDIIVPHKYPKIRIEGTKGEAALKIDST
VSQDSAWYTATAINKAGRDTTRCKVNVEVEFAEPEPERKLIIPRGTYRAKEIAAPELEPL
HLRYGQEQWEEGDLYDKEKQQKPFFKKKLTSLRLKRFGPAHFECRLTPIGDPTMVVEWLH

DGKPLEAANRLRMINEFGYCSLDYGVAYSRDSGIITCRATNKYGTDHTSATLIVKDEKSL
VEESQLPEGRKGLQRIEELERMAHEGALTGVTTDQKEKQKPDIVLYPEPVRVLEGETARF
RCRVTGYPQPKVNWYLNGQLIRKSKRFRVRYDGIHYLDIVDCKSYDTGEVKVTAENPEGV
IEHKVKLEIQQREDFRSVLRRAPEPRPEFHVHEPGKLQFEVQKVDRPVDTTETKEVVKLK
RAERITHEKVPEESEELRSKFKRRTEEGYYEAITAVELKSRKKDESYEELLRKTKDELLH
WTKELTEEEKKALAEEGKITIPTFKPDKIELSPSMEAPKIFERIQSQTVGQGSDAHFRVR
VVGKPDPECEWYKNGVKIERSDRIYWYWPEDNVCELVIRDVTAEDSASIMVKAINIAGET
SSHAFLLVQAKQLITFTQELQDVVAKEKDTMATFECETSEPFVKVKWYKDGMEVHEGDKY
RMHSDRKVHFLSILTIDTSDAEDYSCVLVEDENVKTTAKLIVEGAVVEFVKELQDIEVPE
SYSGELECIVSPENIEGKWYHNDVELKSNGKYTITSRRGRQNLTVKDVTKEDQGEYSFVI
DGKKTTCKLKMKPRPIAILQGLSDQKVCEGDIVQLEVKVSLESVEGVWMKDGQEVQPSDR
VHIVIDKQSHMLLIEDMTKEDAGNYSFTIPALGLSTSGRVSVYSVDVITPLKDVNVIEGT
KAVLECKVSVPDVTSVKWYLNDEQIKPDDRVQAIVKGTKQRLVINRTHASDEGPYKLIVG
RVETNCNLSVEKIKIIRGLRDLTCTETQNVVFEVELSHSGIDVLWNFKDKEIKPSSKYKI
EAHGKIYKLTVLNMMKDDEGKYTFYAGENMTSGKLTVAGGAISKPLTDQTVAESQEAVFE
CEVANPDSKGEWLRDGKHLPLTNNIRSESDGHKRRLIIAATKLDDIGEYTYKVATSKTSA
KLKVEAVKIKKTLKNLTVTETQDAVFTVELTHPNVKGVQWIKNGVVLESNEKYAISVKGT
IYSLRIKNCAIVDESVYGFRLGRLGASARLHVETVKIIKKPKDVTALENATVAFEVSVSH
DTVPVKWFHKSVEIKPSDKHRLVSERKVHKLMLQNISPSDAGEYTAVVGQLECKAKLFVE
TLHITKTMKNIEVPETKTASFECEVSHFNVPSMWLKNGVEIEMSEKFKIVVQGKLHQLII
MNTSTEDSAEYTFVCGNDQVSATLTVTPIMITSMLKDINAEEKDTITFEVTVNYEGISYK
WLKNGVEIKSTDKCQMRTKKLTHSLNIRNVHFGDAADYTFVAGKATSTATLYVEARHIEF
RKHIKDIKVLEKKRAMFECEVSEPDITVQWMKDDQELQITDRIKIQKEKYVHRLLIPSTR
MSDAGKYTVVAGGNVSTAKLFVEGRDVRIRSIKKEVQVIEKQRAVVEFEVNEDDVDAHWY
KDGIEINFQVQERHKYVVERRIHRMFISETRQSDAGEYTFVAGRNRSSVTLYVNAPEPPQ
VLQELQPVTVQSGKPARFCAVISGRPQPKISWYKEEQLLSTGFKCKFLHDGQEYTLLLIE
AFPEDAAVYTCEAKNDYGVATTSASLSVEVPEVVSPDQEMPVYPPAIITPLQDTVTSEGQ
PARFQCRVSGTDLKVSWYSKDKKIKPSRFFRMTQFEDTYQLEIAEAYPEDEGTYTFVASN
AVGQVSSTANLSLEAPESILHERIEQEIEMEMKEFSSSFLSAEEEGLHSAELQLSKINET
LELLSESPVYPTKFDSEKEGTGPIFIKEVSNADISMGDVATLSVTVIGIPKPKIQWFFNG
VLLTPSADYKFVFDGDDHSLIILFTKLEDEGEYTCMASNDYGKTICSAYLKINSKGEGHK
DTETESAVAKSLEKLGGPCPPHFLKELKPIRCAQGLPAIFEYTVVGEPAPTVTWFKENKQ
LCTSVYYTIIHNPNGSGTFIVNDPQREDSGLYICKAENMLGESTCAAELLVLLEDTDMTD
TPCKAKSTPEAPEDFPQTPLKGPAVEALDSEQEIATFVKDTILKAALITEENQQLSYEHI
AKANELSSQLPLGAQELQSILEQDKLTPESTREFLCINGSIHFQPLKEPSPNLQLQIVQS
QKTFSKEGILMPEEPETQAVLSDTEKIFPSAMSIEQINSLTVEPLKTLLAEPEGNYPQSS
IEPPMHSYLTSVAEEVLSPKEKTVSDTNREQRVTLQKQEAQSALILSQSLAEGHVESLQS
PDVMISQVNYEPLVPSEHSCTEGGKILIESANPLENAGQDSAVRIEEGKSLRFPLALEEK
QVLLKEEHSDNVVMPPDQIIESKREPVAIKKVQEVQGRDLLSKESLLSGIPEEQRLNLKI
QICRALQAAVASEQPGLFSEWLRNIEKVEVEAVNITQEPRHIMCMYLVTSAKSVTEEVTI
IIEDVDPQMANLKMELRDALCAIIYEEIDILTAEGPRIQQGAKTSLQEEMDSFSGSQKVE
PITEPEVESKYLISTEEVSYFNVQSRVKYLDATPVTKGVASAVVSDEKQDESLKPSEEKE
ESSSESGTEEVATVKIQEAEGGLIKEDGPMIHTPLVDTVSEEGDIVHLTTSITNAKEVNW
YFENKLVPSDEKFKCLQDQNTYTLVIDKVNTEDHQGEYVCEALNDSGKTATSAKLTVVKR

AAPVIKRKIEPLEVALGHLAKFTCEIQSAPNVRFQWFKAGREIYESDKCSIRSSKYISSL
EILRTQVVDCGEYTCKASNEYGSVSCTATLTVTEAYPPTFLSRPKSLTTFVGKAAKFICT
VTGTPVIETIWQKDGAALSPSPNWRISDAENKHILELSNLTIQDRGVYSCKASNKFGADI
CQAELIIIDKPHFIKELEPVQSAINKKVHLECQVDEDRKVTVTWSKDGQKLPPGKDYKIC
FEDKIATLEIPLAKLKDSGTYVCTASNEAGSSSCSATVTVREPPSFVKKVDPSYLMLPGE
SARLHCKLKGSPVIQVTWFKNNKELSESNTVRMYFVNSEAILDITDVKVEDSGSYSCEAV
NDVGSDSCSTEIVIKEPPSFIKTLEPADIVRGTNALLQCEVSGTGPFEISWFKDKKQIRS
SKKYRLFSQKSLVCLEIFSFNSADVGEYECVVANEVGKCGCMATHLLKEPPTFVKKVDDL
IALGGQTVTLQAAVRGSEPISVTWMKGQEVIREDGKIKMSFSNGVAVLIIPDVQISFGGK
YTCLAENEAGSQTSVGELIVKEPAKIIERAELIQVTAGDPATLEYTVAGTPELKPKWYKD
GRPLVASKKYRISFKNNVAQLKFYSAELHDSGQYTFEISNEVGSSSCETTFTVLDRDIAP
FFTKPLRNVDSVVNGTCRLDCKIAGSLPMRVSWFKDGKEIAASDRYRIAFVEGTASLEII
RVDMNDAGNFTCRATNSVGSKDSSGALIVQEPPSFVTKPGSKDVLPGSAVCLKSTFQGST
PLTIRWFKGNKELVSGGSCYITKEALESSLELYLVKTSDSGTYTCKVSNVAGGVECSANL
FVKEPATFVEKLEPSQLLKKGDATQLACKVTGTPPIKITWFANDREIKESSKHRMSFVES
TAVLRLTDVGIEDSGEYMCEAQNEAGSDHCSSIVIVKESPYFTKEFKPIEVLKEYDVMLL
AEVAGTPPFEITWFKDNTILRSGRKYKTFIQDHLVSLQILKFVAADAGEYQCRVTNEVGS
SICSARVTLREPPSFIKKIESTSSLRGGTAAFQATLKGSLPITVTWLKDSDEITEDDNIR
MTFENNVASLYLSGIEVKHDGKYVCQAKNDAGIQRCSALLSVKEPATITEEAVSIDVTQG
DPATLQVKFSGTKEITAKWFKDGQELTLGSKYKISVTDTVSILKIISTEKKDSGEYTFEV
QNDVGRSSCKARINVLDLIIPPSFTKKLKKMDSIKGSFIDLECIVAGSHPISIQWFKDDQ
EISASEKYKFSFHDNTAFLEISQLEGTDSGTYTCSATNKAGHNQCSGHLTVKEPPYFVEK
PQSQDVNPNTRVQLKALVGGTAPMTIKWFKDNKELHSGAARSVWKDDTSTSLELFAAKAT
DSGTYICQLSNDVGTATSKATLFVKEPPQFIKKPSPVLVLRNGQSTTFECQITGTPKIRV
SWYLDGNEITAIQKHGISFIDGLATFQISGARVENSGTYVCEARNDAGTASCSIELKVKE
PPTFIRELKPVEVVKYSDVELECEVTGTPPFEVTWLKNNREIRSSKKYTLTDRVSVFNLH
ITKCDPSDTGEYQCIVSNEGGSCSCSTRVALKEPPSFIKKIENTTTVLKSSATFQSTVAG
SPPISITWLKDDQILDEDDNVYISFVDSVATLQIRSVDNGHSGRYTCQAKNESGVERCYA
FLLVQEPAQIVEKAKSVDVTEKDPMTLECVVAGTPELKVKWLKDGKQIVPSRYFSMSFEN
NVASFRIQSVMKQDSGQYTFKVENDFGSSSCDAYLRVLDQNIPPSFTKKLTKMDKVLGSS
IHMECKVSGSLPISAQWFKDGKEISTSAKYRLVCHERSVSLEVNNLELEDTANYTCKVSN
VAGDDACSGILTVKEPPSFLVKPGRQQAIPDSTVEFKAILKGTPPFKIKWFKDDVELVSG
PKCFIGLEGSTSFLNLYSVDASKTGQYTCHVTNDVGSDSCTTMLLVTEPPKFVKKLEASK
IVKAGDSSRLECKIAGSPEIRVVWFRNEHELPASDKYRMTFIDSVAVIQMNNLSTEDSGD
FICEAQNPAGSTSCSTKVIVKEPPVFSSFPPIVETLKNAEVSLECELSGTPPFEVVWYKD
KRQLRSSKKYKIASKNFHTSIHILNVDTSDIGEYHCKAQNEVGSDTCVCTVKLKEPPRFV
SKLNSLTVVAGEPAELQASIEGAQPIFVQWLKEKEEVIRESENIRITFVENVATLQFAKA
EPANAGKYICQIKNDGGMRENMATLMVLEPAVIVEKAGPMTVTVGETCTLECKVAGTPEL
SVEWYKDGKLLTSSQKHKFSFYNKISSLRILSVERQDAGTYTFQVQNNVGKSSCTAVVDV
SDRAVPPSFTRRLKNTGGVLGASCILECKVAGSSPISVAWFHEKTKIVSGAKYQTTFSDN
VCTLQLNSLDSSDMGNYTCVAANVAGSDECRAVLTVQEPPSFVKEPEPLEVLPGKNVTFT
SVIRGTPPFKVNWFRGARELVKGDRCNIYFEDTVAELELFNIDISQSGEYTCVVSNNAGQ
ASCTTRLFVKEPAAFLKRLSDHSVEPGKSIILESTYTGTLPISVTWKKDGFNITTSEKCN
IVTTEKTCILEILNSTKRDAGQYSCEIENEAGRDVCGALVSTLEPPYFVTELEPLEAAVG

DSVSLQCQVAGTPEITVSWYKGDTKLRPTPEYRTYFTNNVATLVFNKVNINDSGEYTCKA
ENSIGTASSKTVFRIQERQLPPSFARQLKDIEQTVGLPVTLTCRLNGSAPIQVCWYRDGV
LLRDDENLQTSFVDNVATLKILQTDLSHSGQYSCSASNPLGTASSSARLTAREPKKSPFF
DIKPVSIDVIAGESADFECHVTGAQPMRITWSKDNKEIRPGGNYTITCVGNTPHLRILKV
GKGDSGQYTCQATNDVGKDMCSAQLSVKEPPKFVKKLEASKVAKQGESIQLECKISGSPE
IKVSWFRNDSELHESWKYNMSFINSVALLTINEASAEDSGDYICEAHNGVGDASCSTALT
VKAPPVFTQKPSPVGALKGSDVILQCEISGTPPFEVVWVKDRKQVRNSKKFKITSKHFDT
SLHILNLEASDVGEYHCKATNEVGSDTCSCSVKFKEPPRFVKKLSDTSTLIGDAVELRAI
VEGFQPISVVWLKDRGEVIRESENTRISFIDNIATLQLGSPEASNSGKYICQIKNDAGMR
ECSAVLTVLEPARIIEKPEPMTVTTGNPFALECVVTGTPELSAKWFKDGRELSADSKHHI
TFINKVASLKIPCAEMSDKGLYSFEVKNSVGKSNCTVSVHVSDRIVPPSFIRKLKDVNAI
LGASVVLECRVSGSAPISVGWFQDGNEIVSGPKCQSSFSENVCTLNLSLLEPSDTGIYTC
VAANVAGSDECSAVLTVQEPPSFEQTPDSVEVLPGMSLTFTSVIRGTPPFKVKWFKGSRE
LVPGESCNISLEDFVTELELFEVQPLESGDYSCLVTNDAGSASCTTHLFVKEPATFVKRL
ADFSVETGSPIVLEATYTGTPPISVSWIKDEYLISQSERCSITMTEKSTILEILESTIED
YAQYSCLIENEAGQDICEALVSVLEPPYFIEPLEHVEAVIGEPATLQCKVDGTPEIRISW
YKEHTKLRSAPAYKMQFKNNVASLVINKVDHSDVGEYSCKADNSVGAVASSAVLVIKARK
LPPFFARKLKDVHETLGFPVAFECRINGSEPLQVSWYKDGVLLKDDANLQTSFVHNVATL
QILQTDQSHIGQYNCSASNPLGTASSSAKLILSEHEVPPFFDLKPVSVDLALGESGTFKC
HVTGTAPIKITWAKDNREIRPGGNYKMTLVENTATLTVLKVGKGDAGQYTCYASNIAGKD
SCSAQLGVQEPPRFIKKLEPSRIVKQDEFTRYECKIGGSPEIKVLWYKDETEIQESSKFR
MSFVDSVAVLEMHNLSVEDSGDYTCEAHNAAGSASSSTSLKVKEPPIFRKKPHPIETLKG
ADVHLECELQGTPPFHVSWYKDKRELRSGKKYKIMSENFLTSIHILNVDAADIGEYQCKA
TNDVGSDTCVGSIALKAPPRFVKKLSDISTVVGKEVQLQTTIEGAEPISVVWFKDKGEIV
RESDNIWISYSENIATLQFSRVEPANAGKYTCQIKNDAGMQECFATLSVLEPATIVEKPE
SIKVTTGDTCTLECTVAGTPELSTKWFKDGKELTSDNKYKISFFNKVSGLKIINVAPSDS
GVYSFEVQNPVGKDSCTASLQVSDRTVPPSFTRKLKETNGLSGSSVVMECKVYGSPPISV
SWFHEGNEISSGRKYQTTLTDNTCALTVNMLEESDSGDYTCIATNMAGSDECSAPLTVRE
PPSFVQKPDPMDVLTGTNVTFTSIVKGTPPFSVSWFKGSSELVPGDRCNVSLEDSVAELE
LFDVDTSQSGEYTCIVSNEAGKASCTTHLYIKAPAKFVKRLNDYSIEKGKPLILEGTFTG
TPPISVTWKKNGINVTPSQRCNITTTEKSAILEIPSSTVEDAGQYNCYIENASGKDSCSA
QILILEPPYFVKQLEPVKVSVGDSASLQCQLAGTPEIGVSWYKGDTKLRPTTTYKMHFRN
NVATLVFNQVDINDSGEYICKAENSVGEVSASTFLTVQEQKLPPSFSRQLRDVQETVGLP
VVFDCAISGSEPISVSWYKDGKPLKDSPNVQTSFLDNTATLNIFKTDRSLAGQYSCTATN
PIGSASSSARLILTEGKNPPFFDIRLAPVDAVVGESADFECHVTGTQPIKVSWAKDSREI
RSGGKYQISYLENSAHLTVLKVDKGDSGQYTCYAVNEVGKDSCTAQLNIKERLIPPSFTK
RLSETVEETEGNSFKLEGRVAGSQPITVAWYKNNIEIQPTSNCEITFKNNTLVLQVRKAG
MNDAGLYTCKVSNDAGSALCTSSIVIKEPKKPPVFDQHLTPVTVSEGEYVQLSCHVQGSE
PIRIQWLKAGREIKPSDRCSFSFASGTAVLELRDVAKADSGDYVCKASNVAGSDTTKSKV
TIKDKPAVAPATKKAAVDGRLFFVSEPQSIRVVEKTTATFIAKVGGDPIPNVKWTKGKWR
QLNQGGRVFIHQKGDEAKLEIRDTTKTDSGLYRCVAFNEHGEIESNVNLQVDERKKQEKI
EGDLRAMLKKTPILKKGAGEEEEIDIMELLKNVDPKEYEKYARMYGITDFRGLLQAFELL
KQSQEEETHRLEIEEIERSERDEKEFEELVSFIQQRLSQTEPVTLIKDIENQTVLKDNDA
VFEIDIKINYPEIKLSWYKGTEKLEPSDKFEISIDGDRHTLRVKNCQLKDQGNYRLVCGP

HIASAKLTVIEPAWERHLQDVTLKEGQTCTMTCQFSVPNVKSEWFRNGRILKPQGRHKTE
VEHKVHKLTIADVRAEDQGQYTCKYEDLETSAELRIEAEPIQFTKRIQNIVVSEHQSATF
ECEVSFDDAIVTWYKGPTELTESQKYNFRNDGRCHYMTIHNVTPDDEGVYSVIARLEPRG
EARSTAELYLTTKEIKLELKPPDIPDSRVPIPTMPIRAVPPEEIPPVVAPPIPLLLPTPE
EKKPPPKRIEVTKKAVKKDAKKVVAKPKEMTPREEIVKKPPPPTTLIPAKAPEIIDVSSK
AEEVKIMTITRKKEVQKEKEAVYEKKQAVHKEKRVFIESFEEPYDELEVEPYTEPFEQPY
YEEPDEDYEEIKVEAKKEVHEEWEEDFEEGQEYYEREEGYDEGEEEWEEAYQEREVIQVQ
KEVYEESHERKVPAKVPEKKAPPPPKVIKKPVIEKIEKTSRRMEEEKVQVTKVPEVSKKI
VPQKPSRTPVQEEVIEVKVPAVHTKKMVISEEKMFFASHTEEEVSVTVPEVQKEIVTEEK
IHVAISKRVEPPPKVPELPEKPAPEEVAPVPIPKKVEPPAPKVPEVPKKPVPEEKKPVPV
PKKEPAAPPKVPEVPKKPVPEEKIPVPVAKKKEAPPAKVPEVQKGVVTEEKITIVTQREE
SPPPAVPEIPKKKVPEERKPVPRKEEEVPPPPKVPALPKKPVPEEKVAVPVPVAKKAPPP
RAEVSKKTVVEEKRFVAEEKLSFAVPQRVEVTRHEVSAEEEWSYSEEEEGVSISVYREEE
REEEEEAEVTEYEVMEEPEEYVVEEKLHIISKRVEAEPAEVTERQEKKIVLKPKIPAKIE
EPPPAKVPEAPKKIVPEKKVPAPVPKKEKVPPPKVPEEPKKPVPEKKVPPKVIKMEEPLP
AKVTERHMQITQEEKVLVAVTKKEAPPKARVPEEPKRAVPEEKVLKLKPKREEEPPAKVT
EFRKRVVKEEKVSIEAPKREPQPIKEVTIMEEKERAYTLEEEAVSVQREEEYEEYEEYDY
KEFEEYEPTEEYDQYEEYEEREYERYEEHEEYITEPEKPIPVKPVPEEPVPTKPKAPPAK
VLKKAVPEEKVPVPIPKKLKPPPPKVPEEPKKVFEEKIRISITKREKEQVTEPAAKVPMK
PKRVVAEEKVPVPRKEVAPPVRVPEVPKELEPEEVAFEEEVVTHVEEYLVEEEEEYIHEE
EEFITEEEVVPVIPVKVPEVPRKPVPEEKKPVPVPKKKEAPPAKVPEVPKKPEEKVPVLI
PKKEKPPPAKVPEVPKKPVPEEKVPVPVPKKVEAPPAKVPEVPKKPVPEKKVPVPAPKKV
EAPPAKVPEVPKKLIPEEKKPTPVPKKVEAPPPKVPKKREPVPVPVALPQEEEVLFEEEI
VPEEEVLPEEEEVLPEEEEVLPEEEEVLPEEEEIPPEEEEVPPEEEYVPEEEEFVPEEEV
LPEVKPKVPVPAPVPEIKKKVTEKKVVIPKKEEAPPAKVPEVPKKVEEKRIILPKEEEVL
PVEVTEEPEEEPISEEEIPEEPPSIEEVEEVAPPRVPEVIKKAVPEAPTPVPKKVEAPPA
KVSKKIPEEKVPVPVQKKEAPPAKVPEVPKKVPEKKVLVPKKEAVPPAKGRTVLEEKVSV
AFRQEVVVKERLELEVVEAEVEEIPEEEEFHEVEEYFEEGEFHEVEEFIKLEQHRVEEEH
RVEKVHRVIEVFEAEEVEVFEKPKAPPKGPEISEKIIPPKKPPTKVVPRKEPPAKVPEVP
KKIVVEEKVRVPEEPRVPPTKVPEVLPPKEVVPEKKVPVPPAKKPEAPPPKVPEAPKEVV
PEKKVPVPPPKKPEVPPTKVPEVPKAAVPEKKVPEAIPPKPESPPPEVPEAPKEVVPEKK
VPAAPPKKPEVTPVKVPEAPKEVVPEKKVPVPPPKKPEVPPTKVPEVPKVAVPEKKVPEA
IPPKPESPPPEVFEEPEEVALEEPPAEVVEEPEPAAPPQVTVPPKKPVPEKKAPAVVAKK
PELPPVKVPEVPKEVVPEKKVPLVVPKKPEAPPAKVPEVPKEVVPEKKVAVPKKPEVPPA
KVPEVPKKPVLEEKPAVPVPERAESPPPEVYEEPEEIAPEEEIAPEEEKPVPVAEEEEPE
VPPPAVPEEPKKIIPEKKVPVIKKPEAPPPKEPEPEKVIEKPKLKPRPPPPPPAPPKEDV
KEKIFQLKAIPKKKVPEKPQVPEKVELTPLKVPGGEKKVRKLLPERKPEPKEEVVLKSVL
RKRPEEEEPKVEPKKLEKVKKPAVPEPPPPKPVEEVEVPTVTKRERKIPEPTKVPEIKPA
IPLPAPEPKPKPEAEVKTIKPPPVEPEPTPIAAPVTVPVVGKKAEAKAPKEEAAKPKGPI
KGVPKKTPSPIEAERRKLRPGSGGEKPPDEAPFTYQLKAVPLKFVKEIKDIILTESEFVG
SSAIFECLVSPSTAITTWMKDGSNIRESPKHRFIADGKDRKLHIIDVQLSDAGEYTCVLR
LGNKEKTSTAKLVVEELPVRFVKTLEEEVTVVKGQPLYLSCELNKERDVVWRKDGKIVVE
KPGRIVPGVIGLMRALTINDADDTDAGTYTVTVENANNLECSSCVKVVEVIRDWLVKPIR
DQHVKPKGTAIFACDIAKDTPNIKWFKGYDEIPAEPNDKTEILRDGNHLYLKIKNAMPED

IAEYAVEIEGKRYPAKLTLGEREVELLKPIEDVTIYEKESASFDAEISEADIPGQWKLKG
ELLRPSPTCEIKAEGGKRFLTLHKVKLDQAGEVLYQALNAITTAILTVKEIELDFAVPLK
DVTVPERRQARFECVLTREANVIWSKGPDIIKSSDKFDIIADGKKHILVINDSQFDDEGV
YTAEVEGKKTSARLFVTGIRLKFMSPLEDQTVKEGETATFVCELSHEKMHVVWFKNDAKL
HTSRTVLISSEGKTHKLEMKEVTLDDISQIKAQVKELSSTAQLKVLEADPYFTVKLHDKT
AVEKDEITLKCEVSKDVPVKWFKDGEEIVPSPKYSIKADGLRRILKIKKADLKDKGEYVC
DCGTDKTKANVTVEARLIKVEKPLYGVEVFVGETAHFEIELSEPDVHGQWKLKGQPLTAS
PDCEIIEDGKKHILILHNCQLGMTGEVSFQAANAKSAANLKVKELPLIFITPLSDVKVFE
KDEAKFECEVSREPKTFRWLKGTQEITGDDRFELIKDGTKHSMVIKSAAFEDEAKYMFEA
EDKHTSGKLIIEGIRLKFLTPLKDVTAKEKESAVFTVELSHDNIRVKWFKNDQRLHTTRS
VSMQDEGKTHSITFKDLSIDDTSQIRVEAMGMSSEAKLTVLEGDPYFTGKLQDYTGVEKD
EVILQCEISKADAPVKWFKDGKEIKPSKNAVIKADGKKRMLILKKALKSDIGQYTCDCGT
DKTSGKLDIEDREIKLVRPLHSVEVMETETARFETEISEDDIHANWKLKGEALLQTPDCE
IKEEGKIHSLVLHNCRLDQTGGVDFQAANVKSSAHLRVKPRVIGLLRPLKDVTVTAGETA
TFDCELSYEDIPVEWYLKGKKLEPSDKVVPRSEGKVHTLTLRDVKLEDAGEVQLTAKDFK
THANLFVKEPPVEFTKPLEDQTVEEGATAVLECEVSRENAKVKWFKNGTEILKSKKYEIV
ADGRVRKLVIHDCTPEDIKTYTCDAKDFKTSCNLNVVPPHVEFLRPLTDLQVREKEMARF
ECELSRENAKVKWFKDGAEIKKGKKYDIISKGAVRILVINKCLLDDEAEYSCEVRTARTS
GMLTVLEEEAVFTKNLANIEVSETDTIKLVCEVSKPGAEVIWYKGDEEIIETGRYEILTE
GRKRILVIQNAHLEDAGNYNCRLPSSRTDGKVKVHELAAEFISKPQNLEILEGEKAEFVC
SISKESFPVQWKRDDKTLESGDKYDVIADGKKRVLVVKDATLQDMGTYVVMVGAARAAAH
LTVIEKLRIVVPLKDTRVKEQQEVVFNCEVNTEGAKAKWFRNEEAIFDSSKYIILQKDLV
YTLRIRDAHLDDQANYNVSLTNHRGENVKSAANLIVEEEDLRIVEPLKDIETMEKKSVTF
WCKVNRLNVTLKWTKNGEEVPFDNRVSYRVDKYKHMLTIKDCGFPDEGEYIVTAGQDKSV
AELLIIEAPTEFVEHLEDQTVTEFDDAVFSCQLSREKANVKWYRNGREIKEGKKYKFEKD
GSIHRLIIKDCRLDDECEYACGVEDRKSRARLFVEEIPVEIIRPPQDILEAPGADVVFLA
ELNKDKVEVQWLRNNMVVVQGDKHQMMSEGKIHRLQICDIKPRDQGEYRFIAKDKEARAK
LELAAAPKIKTADQDLVVDVGKPLTMVVPYDAYPKAEAEWFKENEPLSTKTIDTTAEQTS
FRILEAKKGDKGRYKIVLQNKHGKAEGFINLKVIDVPGPVRNLEVTETFDGEVSLAWEEP
LTDGGSKIIGYVVERRDIKRKTWVLATDRAESCEFTVTGLQKGGVEYLFRVSARNRVGTG
EPVETDNPVEARSKYDVPGPPLNVTITDVNRFGVSLTWEPPEYDGGAEITNYVIELRDKT
SIRWDTAMTVRAEDLSATVTDVVEGQEYSFRVRAQNRIGVGKPSAATPFVKVADPIERPS
PPVNLTSSDQTQSSVQLKWEPPLKDGGSPILGYIIERCEEGKDNWIRCNMKLVPELTYKV
TGLEKGNKYLYRVSAENKAGVSDPSEILGPLTADDAFVEPTMDLSAFKDGLEVIVPNPIT
ILVPSTGYPRPTATWCFGDKVLETGDRVKMKTLSAYAELVISPSERSDKGIYTLKLENRV
KTISGEIDVNVIARPSAPKELKFGDITKDSVHLTWEPPDDDGGSPLTGYVVEKREVSRKT
WTKVMDFVTDLEFTVPDLVQGKEYLFKVCARNKCGPGEPAYVDEPVNMSTPATVPDPPEN
VKWRDRTANSIFLTWDPPKNDGGSRIKGYIVERCPRGSDKWVACGEPVAETKMEVTGLEE
GKWYAYRVKALNRQGASKPSRPTEEIQAVDTQEAPEIFLDVKLLAGLTVKAGTKIELPAT
VTGKPEPKITWTKADMILKQDKRITIENVPKKSTVTIVDSKRSDTGTYIIEAVNVCGRAT
AVVEVNVLDKPGPPAAFDITDVTNESCLLTWNPPRDDGGSKITNYVVERRATDSEVWHKL
SSTVKDTNFKATKLIPNKEYIFRVAAENMYGVGEPVQASPITAKYQFDPPGPPTRLEPSD
ITKDAVTLTWCEPDDDGGSPITGYWVERLDPDTDKWVRCNKMPVKDTTYRVKGLTNKKKY
RFRVLAENLAGPGKPSKSTEPILIKDPIDPPWPPGKPTVKDVGKTSVRLNWTKPEHDGGA

KIESYVIEMLKTGTDEWVRVAEGVPTTQHLLPGLMEGQEYSFRVRAVNKAGESEPSEPSD
PVLCREKLYPPSPPRWLEVINITKNTADLKWTVPEKDGGSPITNYIVEKRDVRRKGWQTV
DTTVKDTKCTVTPLTEGSLYVFRVAAENAIGQSDYTEIEDSVLAKDTFTTPGPPYALAVV
DVTKRHVDLKWEPPKNDGGRPIQRYVIEKKERLGTRWVKAGKTAGPDCNFRVTDVIEGTE
VQFQVRAENEAGVGHPSEPTEILSIEDPTSPPSPPLDLHVTDAGRKHIAIAWKPPEKNGG
SPIIGYHVEMCPVGTEKWMRVNSRPIKDLKFKVEEGVVPDKEYVLRVRAVNAIGVSEPSE
ISENVVAKDPDCKPTIDLETHDIIVIEGEKLSIPVPFRAVPVPTVSWHKDGKEVKASDRL
TMKNDHISAHLEVPKSVRADAGIYTITLENKLGSATASINVKVIGLPGPCKDIKASDITK
SSCKLTWEPPEFDGGTPILHYVLERREAGRRTYIPVMSGENKLSWTVKDLIPNGEYFFRV
KAVNKVGGGEYIELKNPVIAQDPKQPPDPPVDVEVHNPTAEAMTITWKPPLYDGGSKIMG
YIIEKIAKGEERWKRCNEHLVPILTYTAKGLEEGKEYQFRVRAENAAGISEPSRATPPTK
AVDPIDAPKVILRTSLEVKRGDEIALDASISGSPYPTITWIKDENVIVPEEIKKRAAPLV
RRRKGEVQEEEPFVLPLTQRLSIDNSKKGESQLRVRDSLRPDHGLYMIKVENDHGIAKAP
CTVSVLDTPGPPINFVFEDIRKTSVLCKWEPPLDDGGSEIINYTLEKKDKTKPDSEWIVV
TSTLRHCKYSVTKLIEGKEYLFRVRAENRFGPGPPCVSKPLVAKDPFGPPDAPDKPIVED
VTSNSMLVKWNEPKDNGSPILGYWLEKREVNSTHWSRVNKSLLNALKANVDGLLEGLTYV
FRVCAENAAGPGKFSPPSDPKTAHDPISPPGPPIPRVTDTSSTTIELEWEPPAFNGGGEI
VGYFVDKQLVGTNEWSRCTEKMIKVRQYTVKEIREGADYKLRVSAVNAAGEGPPGETQPV
TVAEPQEPPAVELDVSVKGGIQIMAGKTLRIPAVVTGRPVPTKVWTKEEGELDKDRVVID
NVGTKSELIIKDALRKDHGRYVITATNSCGSKFAAARVEVFDVPGPVLDLKPVVTNRKMC
LLNWSDPEDDGGSEITGFIIERKDAKMHTWRQPIETERSKCDITGLLEGQEYKFRVIAKN
KFGCGPPVEIGPILAVDPLGPPTSPERLTYTERTKSTITLDWKEPRSNGGSPIQGYIIEK
RRHDKPDFERVNKRLCPTTSFLVENLDEHQMYEFRVKAVNEIGESEPSLPLNVVIQDDEV
PPTIKLRLSVRGDTIKVKAGEPVHIPADVTGLPMPKIEWSKNETVIEKPTDALQITKEEV
SRSEAKTELSIPKAVREDKGTYTVTASNRLGSVFRNVHVEVYDRPSPPRNLAVTDIKAES
CYLTWDAPLDNGGSEITHYVIDKRDASRKKAEWEEVTNTAVEKRYGIWKLIPNGQYEFRV
RAVNKYGISDECKSDKVVIQDPYRLPGPPGKPKVLARTKGSMLVSWTPPLDNGGSPITGY
WLEKREEGSPYWSRVSRAPITKVGLKGVEFNVPRLLEGVKYQFRAMAINAAGIGPPSEPS
DPEVAGDPIFPPGPPSCPEVKDKTKSSISLGWKPPAKDGGSPIKGYIVEMQEEGTTDWKR
VNEPDKLITTCECVVPNLKELRKYRFRVKAVNEAGESEPSDTTGEIPATDIQEEPEVFID
IGAQDCLVCKAGSQIRIPAVIKGRPTPKSSWEFDGKAKKAMKDGVHDIPEDAQLETAENS
SVIIIPECKRSHTGKYSITAKNKAGQKTANCRVKVMDVPGPPKDLKVSDITRGSCRLSWK
MPDDDGGDRIKGYVIEKRTIDGKAWTKVNPDCGSTTFVVPDLLSEQQYFFRVRAENRFGI
GPPVETIQRTTARDPIYPPDPPIKLKIGLITKNTVHLSWKPPKNDGGSPVTHYIVECLAW
DPTGTKKEAWRQCNKRDVEELQFTVEDLVEGGEYEFRVKAVNAAGVSKPSATVGPVTVKD
QTCPPSIDLKEFMEVEEGTNVNIVAKIKGVPFPTLTWFKAPPKKPDNKEPVLYDTHVNKL
VVDDTCTLVIPQSRRSDTGLYTITAVNNLGTASKEMRLNVLGRPGPPVGPIKFESVSADQ
MTLSWFPPKDDGGSKITNYVIEKREANRKTWVHVSSEPKECTYTIPKLLEGHEYVFRIMA
QNKYGIGEPLDSEPETARNLFSVPGAPDKPTVSSVTRNSMTVNWEEPEYDGGSPVTGYWL
EMKDTTSKRWKRVNRDPIKAMTLGVSYKVTGLIEGSDYQFRVYAINAAGVGPASLPSDPA
TARDPIAPPGPPFPKVTDWTKSSADLEWSPPLKDGGSKVTGYIVEYKEEGKEEWEKGKDK
EVRGTKLVVTGLKEGAFYKFRVRAVNIAGIGEPGEVTDVIEMKDRLVSPDLQLDASVRDR
IVVHAGGVIRIIAYVSGKPPPTVTWNMNERTLPQEATIETTAISSSMVIKNCQRSHQGVY
SLLAKNEAGERKKTIIVDVLDVPGPVGTPFLAHNLTNESCKLTWFSPEDDGGSPITNYVI

EKRESDRRAWTPVTYTVTRQNATVQGLIQGKAYFFRIAAENSIGMGPFVETSEALVIREP
ITVPERPEDLEVKEVTKNTVTLTWNPPKYDGGSEIINYVLESRLIGTEKFHKVTNDNLLS
RKYTVKGLKEGDTYEYRVSAVNIVGQGKPSFCTKPITCKDELAPPTLHLDFRDKLTIRVG
EAFALTGRYSGKPKPKVSWFKDEADVLEDDRTHIKTTPATLALEKIKAKRSDSGKYCVVV
ENSTGSRKGFCQVNVVDRPGPPVGPVSFDEVTKDYMVISWKPPLDDGGSKITNYIIEKKE
VGKDVWMPVTSASAKTTCKVSKLLEGKDYIFRIHAENLYGISDPLVSDSMKAKDRFRVPD
APDQPIVTEVTKDSALVTWNKPHDGGKPITNYILEKRETMSKRWARVTKDPIHPYTKFRV
PDLLEGCQYEFRVSAENEIGIGDPSPPSKPVFAKDPIAKPSPPVNPEAIDTTCNSVDLTW
QPPRHDGGSKILGYIVEYQKVGDEEWRRANHTPESCPETKYKVTGLRDGQTYKFRVLAVN
AAGESDPAHVPEPVLVKDRLEPPELILDANMAREQHIKVGDTLRLSAIIKGVPFPKVTWK
KEDRDAPTKARIDVTPVGSKLEIRNAAHEDGGIYSLTVENPAGSKTVSVKVLVLDKPGPP
RDLEVSEIRKDSCYLTWKEPLDDGGSVITNYVVERRDVASAQWSPLSATSKKKSHFAKHL
NEGNQYLFRVAAENQYGRGPFVETPKPIKALDPLHPPGPPKDLHHVDVDKTEVSLVWNKP
DRDGGSPITGYLVEYQEEGTQDWIKFKTVTNLECVVTGLQQGKTYRFRVKAENIVGLGLP
DTTIPIECQEKLVPPSVELDVKLIEGLVVKAGTTVRFPAIIRGVPVPTAKWTTDGSEIKT
DEHYTVETDNFSSVLTIKNCLRRDTGEYQITVSNAAGSKTVAVHLTVLDVPGPPTGPINI
LDVTPEHMTISWQPPKDDGGSPVINYIVEKQDTRKDTWGVVSSGSSKTKLKIPHLQKGCE
YVFRVRAENKIGVGPPLDSTPTVAKHKFSPPSPPGKPVVTDITENAATVSWTLPKSDGGS
PITGYYMERREVTGKWVRVNKTPIADLKFRVTGLYEGNTYEFRVFAENLAGLSKPSPSSD
PIKACRPIKPPGPPINPKLKDKSRETADLVWTKPLSDGGSPILGYVVECQKPGTAQWNRI
NKDELIRQCAFRVPGLIEGNEYRFRIKAANIVGEGEPRELAESVIAKDILHPPEVELDVT
CRDVITVRVGQTIRILARVKGRPEPDITWTKEGKVLVREKRVDLIQDLPRVELQIKEAVR
ADHGKYIISAKNSSGHAQGSAIVNVLDRPGPCQNLKVTNVTKENCTISWENPLDNGGSEI
TNFIVEYRKPNQKGWSIVASDVTKRLIKANLLANNEYYFRVCAENKVGVGPTIETKTPIL
AINPIDRPGEPENLHIADKGKTFVYLKWRRPDYDGGSPNLSYHVERRLKGSDDWERVHKG
SIKETHYMVDRCVENQIYEFRVQTKNEGGESDWVKTEEVVVKEDLQKPVLDLKLSGVLTV
KAGDTIRLEAGVRGKPFPEVAWTKDKDATDLTRSPRVKIDTRADSSKFSLTKAKRSDGGK
YVVTATNTAGSFVAYATVNVLDKPGPVRNLKIVDVSSDRCTVCWDPPEDDGGCEIQNYIL
EKCETKRMVWSTYSATVLTPGTTVTRLIEGNEYIFRVRAENKIGTGPPTESKPVIAKTKY
DKPGRPDPPEVTKVSKEEMTVVWNPPEYDGGKSITGYFLEKKEKHSTRWVPVNKSAIPER
RMKVQNLLPDHEYQFRVKAENEIGIGEPSLPSRPVVAKDPIEPPGPPTNFRVVDTTKHSI
TLGWGKPVYDGGAPIIGYVVEMRPKIADASPDEGWKRCNAAAQLVRKEFTVTSLDENQEY
EFRVCAQNQVGIGRPAELKEAIKPKEILEPPEIDLDASMRKLVIVRAGCPIRLFAIVRGR
PAPKVTWRKVGIDNVVRKGQVDLVDTMAFLVIPNSTRDDSGKYSLTLVNPAGEKAVFVNV
RVLDTPGPVSDLKVSDVTKTSCHVSWAPPENDGGSQVTHYIVEKREADRKTWSTVTPEVK
KTSFHVTNLVPGNEYYFRVTAVNEYGPGVPTDVPKPVLASDPLSEPDPPRKLEVTEMTKN
SATLAWLPPLRDGGAKIDGYITSYREEEQPADRWTEYSVVKDLSLVVTGLKEGKKYKFRV
AARNAVGVSLPREAEGVYEAKEQLLPPKILMPEQITIKAGKKLRIEAHVYGKPHPTCKWK
KGEDEVVTSSHLAVHKADSSSILIIKDVTRKDSGYYSLTAENSSGTDTQKIKVVVMDAPG
PPQPPFDISDIDADACSLSWHIPLEDGGSNITNYIVEKCDVSRGDWVTALASVTKTSCRV
GKLIPGQEYIFRVRAENRFGISEPLTSPKMVAQFPFGVPSEPKNARVTKVNKDCIFVAWD
RPDSDGGSPIIGYLIERKERNSLLWVKANDTLVRSTEYPCAGLVEGLEYSFRIYALNKAG
SSPPSKPTEYVTARMPVDPPGKPEVIDVTKSTVSLIWARPKHDGGSKIIGYFVEACKLPG
DKWVRCNTAPHQIPQEEYTATGLEEKAQYQFRAIARTAVNISPPSEPSDPVTILAENVPP

RIDLSVAMKSLLTVKAGTNVCLDATVFGKPMPTVSWKKDGTLLKPAEGIKMAMQRNLCTL
ELFSVNRKDSGDYTITAENSSGSKSATIKLKVLDKPGPPASVKINKMYSDRAMLSWEPPL
EDGGSEITNYIVDKRETSRPNWAQVSATVPITSCSVEKLIEGHEYQFRICAENKYGVGDP
VFTEPAIAKNPYDPPGRCDPPVISNITKDHMTVSWKPPADDGGSPITGYLLEKRETQAVN
WTKVNRKPIIERTLKATGLQEGTEYEFRVTAINKAGPGKPSDASKAAYARDPQYPPGPPA
FPKVYDTTRSSVSLSWGKPAYDGGSPIIGYLVEVKRADSDNWVRCNLPQNLQKTRFEVTG
LMEDTQYQFRVYAVNKIGYSDPSDVPDKHYPKDILIPPEGELDADLRKTLILRAGVTMRL
YVPVKGRPPPKITWSKPNVNLRDRIGLDIKSTDFDTFLRCENVNKYDAGKYILTLENSCG
KKEYTIVVKVLDTPGPPVNVTVKEISKDSAYVTWEPPIIDGGSPIINYVVQKRDAERKSW
STVTTECSKTSFRVANLEEGKSYFFRVFAENEYGIGDPGETRDAVKASQTPGPVVDLKVR
SVSKSSCSIGWKKPHSDGGSRIIGYVVDFLTEENKWQRVMKSLSLQYSAKDLTEGKEYTF
RVSAENENGEGTPSEITVVARDDVVAPDLDLKGLPDLCYLAKENSNFRLKIPIKGKPAPS
VSWKKGEDPLATDTRVSVESSAVNTTLIVYDCQKSDAGKYTITLKNVAGTKEGTISIKVV
GKPGIPTGPIKFDEVTAEAMTLKWAPPKDDGGSEITNYILEKRDSVNNKWVTCASAVQKT
TFRVTRLHEGMEYTFRVSAENKYGVGEGLKSEPIVARHPFDVPDAPPPPNIVDVRHDSVS
LTWTDPKKTGGSPITGYHLEFKERNSLLWKRANKTPIRMRDFKVTGLTEGLEYEFRVMAI
NLAGVGKPSLPSEPVVALDPIDPPGKPEVINITRNSVTLIWTEPKYDGGHKLTGYIVEKR
DLPSKSWMKANHVNVPECAFTVTDLVEGGKYEFRIRAKNTAGAISAPSESTETIICKDEY
EAPTIVLDPTIKDGLTIKAGDTIVLNAISILGKPLPKSSWSKAGKDIRPSDITQITSTPT
SSMLTIKYATRKDAGEYTITATNPFGTKVEHVKVTVLDVPGPPGPVEISNVSAEKATLTW
TPPLEDGGSPIKSYILEKRETSRLLWTVVSEDIQSCRHVATKLIQGNEYIFRVSAVNHYG
KGEPVQSEPVKMVDRFGPPGPPEKPEVSNVTKNTATVSWKRPVDDGGSEITGYHVERREK
KSLRWVRAIKTPVSDLRCKVTGLQEGSTYEFRVSAENRAGIGPPSEASDSVLMKDAAYPP
GPPSNPHVTDTTKKSASLAWGKPHYDGGLEITGYVVEHQKVGDEAWIKDTTGTALRITQF
VVPDLQTKEKYNFRISAINDAGVGEPAVIPDVEIVEREMAPDFELDAELRRTLVVRAGLS
IRIFVPIKGRPAPEVTWTKDNINLKNRANIENTESFTLLIIPECNRYDTGKFVMTIENPA
GKKSGFVNVRVLDTPGPVLNLRPTDITKDSVTLHWDLPLIDGGSRITNYIVEKREATRKS
YSTATTKCHKCTYKVTGLSEGCEYFFRVMAENEYGIGEPTETTEPVKASEAPSPPDSLNI
MDITKSTVSLAWPKPKHDGGSKITGYVIEAQRKGSDQWTHITTVKGLECVVRNLTEGEEY
TFQVMAVNSAGRSAPRESRPVIVKEQTMLPELDLRGIYQKLVIAKAGDNIKVEIPVLGRP
KPTVTWKKGDQILKQTQRVNFETTATSTILNINECVRSDSGPYPLTARNIVGEVGDVITI
QVHDIPGPPTGPIKFDEVSSDFVTFSWDPPENDGGVPISNYVVEMRQTDSTTWVELATTV
IRTTYKATRLTTGLEYQFRVKAQNRYGVGPGITSACIVANYPFKVPGPPGTPQVTAVTKD
SMTISWHEPLSDGGSPILGYHVERKERNGILWQTVSKALVPGNIFKSSGLTDGIAYEFRV
IAENMAGKSKPSKPSEPMLALDPIDPPGKPVPLNITRHTVTLKWAKPEYTGGFKITSYIV
EKRDLPNGRWLKANFSNILENEFTVSGLTEDAAYEFRVIAKNAAGAISPPSEPSDAITCR
DDVEAPKIKVDVKFKDTVILKAGEAFRLEADVSGRPPPTMEWSKDGKELEGTAKLEIKIA
DFSTNLVNKDSTRRDSGAYTLTATNPGGFAKHIFNVKVLDRPGPPEGPLAVTEVTSEKCV
LSWFPPLDDGGAKIDHYIVQKRETSRLAWTNVASEVQVTKLKVTKLLKGNEYIFRVMAVN
KYGVGEPLESEPVLAVNPYGPPDPPKNPEVTTITKDSMVVCWGHPDSDGGSEIINYIVER
RDKAGQRWIKCNKKTLTDLRYKVSGLTEGHEYEFRIMAENAAGISAPSPTSPFYKACDTV
FKPGPPGNPRVLDTSRSSISIAWNKPIYDGGSEITGYMVEIALPEEDEWQIVTPPAGLKA
TSYTITGLTENQEYKIRIYAMNSEGLGEPALVPGTPKAEDRMLPPEIELDADLRKVVTIR
ACCTLRLFVPIKGRPAPEVKWARDHGESLDKASIESTSSYTLLIVGNVNRFDSGKYILTV

ENSSGSKSAFVNVRVLDTPGPPQDLKVKEVTKTSVTLTWDPPLLDGGSKIKNYIVEKRES
TRKAYSTVATNCHKTSWKVDQLQEGCSYYFRVLAENEYGIGLPAETAESVKASERPLPPG
KITLMDVTRNSVSLSWEKPEHDGGSRILGYIVEMQTKGSDKWATCATVKVTEATITGLIQ
GEEYSFRVSAQNEKGISDPRQLSVPVIAKDLVIPPAFKLLFNTFTVLAGEDLKVDVPFIG
RPTPAVTWHKDNVPLKQTTRVNAESTENNSLLTIKDACREDVGHYVVKLTNSAGEAIETL
NVIVLDKPGPPTGPVKMDEVTADSITLSWGPPKYDGGSSINNYIVEKRDTSTTTWQIVSA
TVARTTIKACRLKTGCEYQFRIAAENRYGKSTYLNSEPTVAQYPFKVPGPPGTPVVTLSS
RDSMEVQWNEPISDGGSRVIGYHLERKERNSILWVKLNKTPIPQTKFKTTGLEEGVEYEF
RVSAENIVGIGKPSKVSECYVARDPCDPPGRPEAIIVTRNSVTLQWKKPTYDGGSKITGY
IVEKKELPEGRWMKASFTNIIDTHFEVTGLVEDHRYEFRVIARNAAGVFSEPSESTGAIT
ARDEVDPPRISMDPKYKDTIVVHAGESFKVDADIYGKPIPTIQWIKGDQELSNTARLEIK
STDFATSLSVKDAVRVDSGNYILKAKNVAGERSVTVNVKVLDRPGPPEGPVVISGVTAEK
CTLAWKPPLQDGGSDIINYIVERRETSRLVWTVVDANVQTLSCKVTKLLEGNEYTFRIMA
VNKYGVGEPLESEPVVAKNPFVVPDAPKAPEVTTVTKDSMIVVWERPASDGGSEILGYVL
EKRDKEGIRWTRCHKRLIGELRLRVTGLIENHDYEFRVSAENAAGLSEPSPPSAYQKACD
PIYKPGPPNNPKVIDITRSSVFLSWSKPIYDGGCEIQGYIVEKCDVSVGEWTMCTPPTGI
NKTNIEVEKLLEKHEYNFRICAINKAGVGEHADVPGPIIVEEKLEAPDIDLDLELRKIIN
IRAGGSLRLFVPIKGRPTPEVKWGKVDGEIRDAAIIDVTSSFTSLVLDNVNRYDSGKYTL
TLENSSGTKSAFVTVRVLDTPSPPVNLKVTEITKDSVSITWEPPLLDGGSKIKNYIVEKR
EATRKSYAAVVTNCHKNSWKIDQLQEGCSYYFRVTAENEYGIGLPAQTADPIKVAEVPQP
PGKITVDDVTRNSVSLSWTKPEHDGGSKIIQYIVEMQAKHSEKWSECARVKSLQAVITNL
TQGEEYLFRVVAVNEKGRSDPRSLAVPIVAKDLVIEPDVKPAFSSYSVQVGQDLKIEVPI
SGRPKPTITWTKDGLPLKQTTRINVTDSLDLTTLSIKETHKDDGGQYGITVANVVGQKTA
SIEIVTLDKPDPPKGPVKFDDVSAESITLSWNPPLYTGGCQITNYIVQKRDTTTTVWDVV
SATVARTTLKVTKLKTGTEYQFRIFAENRYGQSFALESDPIVAQYPYKEPGPPGTPFATA
ISKDSMVIQWHEPVNNGGSPVIGYHLERKERNSILWTKVNKTIIHDTQFKAQNLEEGIEY
EFRVYAENIVGVGKASKNSECYVARDPCDPPGTPEPIMVKRNEITLQWTKPVYDGGSMIT
GYIVEKRDLPDGRWMKASFTNVIETQFTVSGLTEDQRYEFRVIAKNAAGAISKPSDSTGP
ITAKDEVELPRISMDPKFRDTIVVNAGETFRLEADVHGKPLPTIEWLRGDKEIEESARCE
IKNTDFKALLIVKDAIRIDGGQYILRASNVAGSKSFPVNVKVLDRPGPPEGPVQVTGVTS
EKCSLTWSPPLQDGGSDISHYVVEKRETSRLAWTVVASEVVTNSLKVTKLLEGNEYVFRI
MAVNKYGVGEPLESAPVLMKNPFVLPGPPKSLEVTNIAKDSMTVCWNRPDSDGGSEIIGY
IVEKRDRSGIRWIKCNKRRITDLRLRVTGLTEDHEYEFRVSAENAAGVGEPSPATVYYKA
CDPVFKPGPPTNAHIVDTTKNSITLAWGKPIYDGGSEILGYVVEICKADEEEWQIVTPQT
GLRVTRFEISKLTEHQEYKIRVCALNKVGLGEATSVPGTVKPEDKLEAPELDLDSELRKG
IVVRAGGSARIHIPFKGRPTPEITWSREEGEFTDKVQIEKGVNYTQLSIDNCDRNDAGKY
ILKLENSSGSKSAFVTVKVLDTPGPPQNLAVKEVRKDSAFLVWEPPIIDGGAKVKNYVID
KRESTRKAYANVSSKCSKTSFKVENLTEGAIYYFRVMAENEFGVGVPVETVDAVKAAEPP
SPPGKVTLTDVSQTSASLMWEKPEHDGGSRVLGYVVEMQPKGTEKWSIVAESKVCNAVVT
GLSSGQEYQFRVKAYNEKGKSDPRVLGVPVIAKDLTIQPSLKLPFNTYSIQAGEDLKIEI
PVIGRPRPNISWVKDGEPLKQTTRVNVEETATSTVLHIKEGNKDDFGKYTVTATNSAGTA
TENLSVIVLEKPGPPVGPVRFDEVSADFVVISWEPPAYTGGCQISNYIVEKRDTTTTTWH
MVSATVARTTIKITKLKTGTEYQFRIFAENRYGKSAPLDSKAVIVQYPFKEPGPPGTPFV
TSISKDQMLVQWHEPVNDGGTKIIGYHLEQKEKNSILWVKLNKTPIQDTKFKTTGLDEGL

EYEFKVSAENIVGIGKPSKVSECFVARDPCDPPGRPEAIVITRNNVTLKWKKPAYDGGSK
ITGYIVEKKDLPDGRWMKASFTNVLETEFTVSGLVEDQRYEFRVIARNAAGNFSEPSDSS
GAITARDEIDAPNASLDPKYKDVIVVHAGETFVLEADIRGKPIPDVVWSKDGKELEETAA
RMEIKSTIQKTTLVVKDCIRTDGGQYILKLSNVGGTKSIPITVKVLDRPGPPEGPLKVTG
VTAEKCYLAWNPPLQDGGANISHYIIEKRETSRLSWTQVSTEVQALNYKVTKLLPGNEYI
FRVMAVNKYGIGEPLESGPVTACNPYKPPGPPSTPEVSAITKDSMVVTWARPVDDGGTEI
EGYILEKRDKEGVRWTKCNKKTLTDLRLRVTGLTEGHSYEFRVAAENAAGVGEPSEPSVF
YRACDALYPPGPPSNPKVTDTSRSSVSLAWSKPIYDGGAPVKGYVVEVKEAAADEWTTCT
PPTGLQGKQFTVTKLKENTEYNFRICAINSEGVGEPATLPGSVVAQERIEPPEIELDADL
RKVVVLRASATLRLFVTIKGRPEPEVKWEKAEGILTDRAQIEVTSSFTMLVIDNVTRFDS
GRYNLTLENNSGSKTAFVNVRVLDSPSAPVNLTIREVKKDSVTLSWEPPLIDGGAKITNY
IVEKRETTRKAYATITNNCTKTTFRIENLQEGCSYYFRVLASNEYGIGLPAETTEPVKVS
EPPLPPGRVTLVDVTRNTATIKWEKPESDGGSKITGYVVEMQTKGSEKWSTCTQVKTLEA
TISGLTAGEEYVFRVAAVNEKGRSDPRQLGVPVIARDIEIKPSVELPFHTFNVKAREQLK
IDVPFKGRPQATVNWRKDGQTLKETTRVNVSSSKTVTSLSIKEASKEDVGTYELCVSNSA
GSITVPITIIVLDRPGPPGPIRIDEVSCDSITISWNPPEYDGGCQISNYIVEKKETTSTT
WHIVSQAVARTSIKIVRLTTGSEYQFRVCAENRYGKSSYSESSAVVAEYPFSPPGPPGTP
KVVHATKSTMLVTWQVPVNDGGSRVIGYHLEYKERSSILWSKANKILIADTQMKVSGLDE
GLMYEYRVYAENIAGIGKCSKSCEPVPARDPCDPPGQPEVTNITRKSVSLKWSKPHYDGG
AKITGYIVERRELPDGRWLKCNYTNIQETYFEVTELTEDQRYEFRVFARNAADSVSEPSE
STGPIIVKDDVEPPRVMMDVKFRDVIVVKAGEVLKINADIAGRPLPVISWAKDGIEIEER
ARTEIISTDNHTLLTVKDCIRRDTGQYVLTLKNVAGTRSVAVNCKVLDKPGPPAGPLEIN
GLTAEKCSLSWGRPQEDGGADIDYYIVEKRETSHLAWTICEGELQMTSCKVTKLLKGNEY
IFRVTGVNKYGVGEPLESVAIKALDPFTVPSPPTSLEITSVTKESMTLCWSRPESDGGSE
ISGYIIERREKNSLRWVRVNKKPVYDLRVKSTGLREGCEYEYRVYAENAAGLSLPSETSP
LIRAEDPVFLPSPPSKPKIVDSGKTTITIAWVKPLFDGGAPITGYTVEYKKSDDTDWKTS
IQSLRGTEYTISGLTTGAEYVFRVKSVNKVGASDPSDSSDPQIAKEREEEPLFDIDSEMR
KTLIVKAGASFTMTVPFRGRPVPNVLWSKPDTDLRTRAYVDTTDSRTSLTIENANRNDSG
KYTLTIQNVLSAASLTLVVKVLDTPGPPTNITVQDVTKESAVLSWDVPENDGGAPVKNYH
IEKREASKKAWVSVTNNCNRLSYKVTNLQEGAIYYFRVSGENEFGVGIPAETKEGVKITE
KPSPPEKLGVTSISKDSVSLTWLKPEHDGGSRIVHYVVEALEKGQKNWVKCAVAKSTHHV
VSGLRENSEYFFRVFAENQAGLSDPRELLLPVLIKEQLEPPEIDMKNFPSHTVYVRAGSN
LKVDIPISGKPLPKVTLSRDGVPLKATMRFNTEITAENLTINLKESVTADAGRYEITAAN
SSGTTKAFINIVVLDRPGPPTGPVVISDITEESVTLKWEPPKYDGGSQVTNYILLKRETS
TAVWTEVSATVARTMMKVMKLTTGEEYQFRIKAENRFGISDHIDSACVTVKLPYTTPGPP
STPWVTNVTRESITVGWHEPVSNGGSAVVGYHLEMKDRNSILWQKANKLVIRTTHFKVTT
ISAGLIYEFRVYAENAAGVGKPSHPSEPVLAIDACEPPRNVRITDISKNSVSLSWQQPAF
DGGSKITGYIVERRDLPDGRWTKASFTNVTETQFIISGLTQNSQYEFRVFARNAVGSISN
PSEVVGPITCIDSYGGPVIDLPLEYTEVVKYRAGTSVKLRAGISGKPAPTIEWYKDDKEL
QTNALVCVENTTDLASILIKDADRLNSGCYELKLRNAMGSASATIRVQILDKPGPPGGPI
EFKTVTAEKITLLWRPPADDGGAKITHYIVEKRETSRVVWSMVSEHLEECIITTTKIIKG
NEYIFRVRAVNKYGIGEPLESDSVVAKNAFVTPGPPGIPEVTKITKNSMTVVWSRPIADG
GSDISGYFLEKRDKKSLGWFKVLKETIRDTRQKVTGLTENSDYQYRVCAVNAAGQGPFSE
PSEFYKAADPIDPPGPPAKIRIADSTKSSITLGWSKPVYDGGSAVTGYVVEIRQGEEEEW

TTVSTKGEVRTTEYVVSNLKPGVNYYFRVSAVNCAGQGEPIEMNEPVQAKDILEAPEIDL
DVALRTSVIAKAGEDVQVLIPFKGRPPPTVTWRKDEKNLGSDARYSIENTDSSSLLTIPQ
VTRNDTGKYILTIENGVGEPKSSTVSVKVLDTPAACQKLQVKHVSRGTVTLLWDPPLIDG
GSPIINYVIEKRDATKRTWSVVSHKCSSTSFKLIDLSEKTPFFFRVLAENEIGIGEPCET
TEPVKAAEVPAPIRDLSMKDSTKTSVILSWTKPDFDGGSVITEYVVERKGKGEQTWSHAG
ISKTCEIEVSQLKEQSVLEFRVFAKNEKGLSDPVTIGPITVKELIITPEVDLSDIPGAQV
TVRIGHNVHLELPYKGKPKPSISWLKDGLPLKESEFVRFSKTENKITLSIKNAKKEHGGK
YTVILDNAVCRIAVPITVITLGPPSKPKGPIRFDEIKADSVILSWDVPEDNGGGEITCYS
IEKRETSQTNWKMVCSSVARTTFKVPNLVKDAEYQFRVRAENRYGVSQPLVSSIIVAKHQ
FRIPGPPGKPVIYNVTSDGMSLTWDAPVYDGGSEVTGFHVEKKERNSILWQKVNTSPISG
REYRATGLVEGLDYQFRVYAENSAGLSSPSDPSKFTLAVSPVDPPGTPDYIDVTRETITL
KWNPPLRDGGSKIVGYSIEKRQGNERWVRCNFTDVSECQYTVTGLSPGDRYEFRIIARNA
VGTISPPSQSSGIIMTRDENVPPIVEFGPEYFDGLIIKSGESLRIKALVQGRPVPRVTWF
KDGVEIEKRMNMEITDVLGSTSLFVRDATRDHRGVYTVEAKNASGSAKAEIKVKVQDTPG
KVVGPIRFTNITGEKMTLWWDAPLNDGCAPITHYIIEKRETSRLAWALIEDKCEAQSYTA
IKLINGNEYQFRVSAVNKFGVGRPLDSDPVVAQIQYTVPDAPGIPEPSNITGNSITLTWA
RPESDGGSEIQQYILERREKKSTRWVKVISKRPISETRFKVTGLTEGNEYEFHVMAENAA
GVGPASGISRLIKCREPVNPPGPPTVVKVTDTSKTTVSLEWSKPVFDGGMEIIGYIIEMC
KADLGDWHKVNAEACVKTRYTVTDLQAGEEYKFRVSAINGAGKGDSCEVTGTIKAVDRLT
APELDIDANFKQTHVVRAGASIRLFIAYQGRPTPTAVWSKPDSNLSLRADIHTTDSFSTL
TVENCNRNDAGKYTLTVENNSGSKSITFTVKVLDTPGPPGPITFKDVTRGSATLMWDAPL
LDGGARIHHYVVEKREASRRSWQVISEKCTRQIFKVNDLAEGVPYYFRVSAVNEYGVGEP
YEMPEPIVATEQPAPPRRLDVVDTSKSSAVLAWLKPDHDGGSRITGYLLEMRQKGSDFWV
EAGHTKQLTFTVERLVEKTEYEFRVKAKNDAGYSEPREAFSSVIIKEPQIEPTADLTGIT
NQLITCKAGSPFTIDVPISGRPAPKVTWKLEEMRLKETDRVSITTTKDRTTLTVKDSMRG
DSGRYFLTLENTAGVKTFSVTVVVIGRPGPVTGPIEVSSVSAESCVLSWGEPKDGGGTEI
TNYIVEKRESGTTAWQLVNSSVKRTQIKVTHLTKYMEYSFRVSSENRFGVSKPLESAPII
AEHPFVPPSAPTRPEVYHVSANAMSIRWEEPYHDGGSKIIGYWVEKKERNTILWVKENKV
PCLECNYKVTGLVEGLEYQFRTYALNAAGVSKASEASRPIMAQNPVDAPGRPEVTDVTRS
TVSLIWSAPAYDGGSKVVGYIIERKPVSEVGDGRWLKCNYTIVSDNFFTVTALSEGDTYE
FRVLAKNAAGVISKGSESTGPVTCRDEYAPPKAELDARLHGDLVTIRAGSDLVLDAAVGG
KPEPKIIWTKGDKELDLCEKVSLQYTGKRATAVIKFCDRSDSGKYTLTVKNASGTKAVSV
MVKVLDSPGPCGKLTVSRVTQEKCTLAWSLPQEDGGAEITHYIVERRETSRLNWVIVEGE
CPTLSYVVTRLIKNNEYIFRVRAVNKYGPGVPVESEPIVARNSFTIPSPPGIPEEVGTGK
EHIIIQWTKPESDGGNEISNYLVDKREKKSLRWTRVNKDYVVYDTRLKVTSLMEGCDYQF
RVTAVNAAGNSEPSEASNFISCREPSYTPGPPSAPRVVDTTKHSISLAWTKPMYDGGTDI
VGYVLEMQEKDTDQWYRVHTNATIRNTEFTVPDLKMGQKYSFRVAAVNVKGMSEYSESIA
EIEPVERIEIPDLELADDLKKTVTIRAGASLRLMVSVSGRPPPVITWSKQGIDLASRAII
DTTESYSLLIVDKVNRYDAGKYTIEAENQSGKKSATVLVKVYDTPGPCPSVKVKEVSRDS
VTITWEIPTIDGGAPVNNYIVEKREAAMRAFKTVTTKCSKTLYRISGLVEGTMYYFRVLP
ENIYGIGEPCETSDAVLVSEVPLVPAKLEVVDVTKSTVTLAWEKPLYDGGSRLTGYVLEA
CKAGTERWMKVVTLKPTVLEHTVTSLNEGEQYLFRIRAQNEKGVSEPRETVTAVTVQDLR
VLPTIDLSTMPQKTIHVPAGRPVELVIPIAGRPPPAASWFFAGSKLRESERVTVETHTKV
AKLTIRETTIRDTGEYTLELKNVTGTTSETIKVIILDKPGPPTGPIKIDEIDATSITISW

EPPELDGGAPLSGYVVEQRDAHRPGWLPVSESVTRSTFKFTRLTEGNEYVFRVAATNRFG
IGSYLQSEVIECRSSIRIPGPPETLQIFDVSRDGMTLTWYPPEDDGGSQVTGYIVERKEV
RADRWVRVNKVPVTMTRYRSTGLTEGLEYEHRVTAINARGSGKPSRPSKPIVAMDPIAPP
GKPQNPRVTDTTRTSVSLAWSVPEDEGGSKVTGYLIEMQKVDQHEWTKCNTTPTKIREYT
LTHLPQGAEYRFRVLACNAGGPGEPAEVPGTVKVTEMLEYPDYELDERYQEGIFVRQGGV
IRLTIPIKGKPFPICKWTKEGQDISKRAMIATSETHTELVIKEADRGDSGTYDLVLENKC
GKKAVYIKVRVIGSPNSPEGPLEYDDIQVRSVRVSWRPPADDGGADILGYILERREVPKA
AWYTIDSRVRGTSLVVKGLKENVEYHFRVSAENQFGISKPLKSEEPVTPKTPLNPPEPPS
NPPEVLDVTKSSVSLSWSRPKDDGGSRVTGYYIERKETSTDKWVRHNKTQITTTMYTVTG
LVPDAEYQFRIIAQNDVGLSETSPASEPVVCKDPFDKPSQPGELEILSISKDSVTLQWEK
PECDGGKEILGYWVEYRQSGDSAWKKSNKERIKDKQFTIGGLLEATEYEFRVFAENETGL
SRPRRTAMSIKTKLTSGEAPGIRKEMKDVTTKLGEAAQLSCQIVGRPLPDIKWYRFGKEL
IQSRKYKMSSDGRTHTLTVMTEEQEDEGVYTCIATNEVGEVETSSKLLLQATPQFHPGYP
LKEKYYGAVGSTLRLHVMYIGRPVPAMTWFHGQKLLQNSENITIENTEHYTHLVMKNVQR
KTHAGKYKVQLSNVFGTVDAILDVEIQDKPDKPTGPIVIEALLKNSAVISWKPPADDGGS
WITNYVVEKCEAKEGAEWQLVSSAISVTTCRIVNLTENAGYYFRVSAQNTFGISDPLEVS
SVVIIKSPFEKPGAPGKPTITAVTKDSCVVAWKPPASDGGAKIRNYYLEKREKKQNKWIS
VTTEEIRETVFSVKNLIEGLEYEFRVKCENLGGESEWSEISEPITPKSDVPIQAPHFKEE
LRNLNVRYQSNATLVCKVTGHPKPIVKWYRQGKEIIADGLKYRIQEFKGGYHQLIIASVT
DDDATVYQVRATNQGGSVSGTASLEVEVPAKIHLPKTLEGMGAVHALRGEVVSIKIPFSG
KPDPVITWQKGQDLIDNNGHYQVIVTRSFTSLVFPNGVERKDAGFYVVCAKNRFGIDQKT
VELDVADVPDPPRGVKVSDVSRDSVNLTWTEPASDGGSKITNYIVEKCATTAERWLRVGQ
ARETRYTVINLFGKTSYQFRVIAENKFGLSKPSEPSEPTITKEDKTRAMNYDEEVDETRE
VSMTKASHSSTKELYEKYMIAEDLGRGEFGIVHRCVETSSKKTYMAKFVKVKGTDQVLVK
KEISILNIARHRNILHLHESFESMEELVMIFEFISGLDIFERINTSAFELNEREIVSYVH
QVCEALQFLHSHNIGHFDIRPENIIYQTRRSSTIKIIEFGQARQLKPGDNFRLLFTAPEY
YAPEVHQHDVVSTATDMWSLGTLVYVLLSGINPFLAETNQQIIENIMNAEYTFDEEAFKE
ISIEAMDFVDRLLVKERKSRMTASEALQHPWLKQKIERVSTKVIRTLKHRRYYHTLIKKD
LNMVVSAARISCGGAIRSQKGVSVAKVKVASIEIGPVSGQIMHAVGEEGGHVKYVCKIEN
YDQSTQVTWYFGVRQLENSEKYEITYEDGVAILYVKDITKLDDGTYRCKVVNDYGEDSSY
AELFVKGVREVYDYYCRRTMKKIKRRTDTMRLLERPPEFTLPLYNKTAYVGENVRFGVTI
TVHPEPHVTWYKSGQKIKPGDNDKKYTFESDKGLYQLTINSVTTDDDAEYTVVARNKYGE
DSCKAKLTVTLHPPPTDSTLRPMFKRLLANAECQEGQSVCFEIRVSGIPPPTLKWEKDGQ
PLSLGPNIEIIHEGLDYYALHIRDTLPEDTGYYRVTATNTAGSTSCQAHLQVERLRYKKQ
EFKSKEEHERHVQKQIDKTLRMAEILSGTESVPLTQVAKEALREAAVLYKPAVSTKTVKG
EFRLEIEEKKEERKLRMPYDVPEPRKYKQTTIEEDQRIKQFVPMSDMKWYKKIRDQYEMP
GKLDRVVQKRPKRIRLSRWEQFYVMPLPRITDQYRPKWRIPKLSQDDLEIVRPARRRTPS
PDYDFYYRPRRRSLGDISDEELLLPIDDYLAMKRTEEERLRLEEELELGFSASPPSRSPP
HFELSSLRYSSPQAHVKVEETRKDFRYSTYHIPTKAEASTSYAELRERHAQAAYRQPKQR
QRIMAEREDEELLRPVTTTQHLSEYKSELDFMSKEEKSRKKSRRQREVTEITEIEEEYEI
SKHAQRESSSSASRLLRRRRSLSPTYIELMRPVSELIRSRPQPAEEYEDDTERRSPTPER
TRPRSPSPVSSERSLSRFERSARFDIFSRYESMKAALKTQKTSERKYEVLSQQPFTLDHA
PRITLRMRSHRVPCGQNTRFILNVQSKPTAEVKWYHNGVELQESSKIHYTNTSGVLTLEI
LDCHTDDSGTYRAVCTNYKGEASDYATLDVTGGDYTTYASQRRDEEVPRSVFPELTRTEA

YAVSSFKKTSEMEASSSVREVKSQMTETRESLSSYEHSASAEMKSAALEEKSLEEKSTTR
KIKTTLAARILTKPRSMTVYEGESARFSCDTDGEPVPTVTWLRKGQVLSTSARHQVTTTK
YKSTFEISSVQASDEGNYSVVVENSEGKQEAEFTLTIQKARVTEKAVTSPPRVKSPEPRV
KSPEAVKSPKRVKSPEPSHPKAVSPTETKPTPTEKVQHLPVSAPPKITQFLKAEASKEIA
KLTCVVESSVLRAKEVTWYKDGKKLKENGHFQFHYSADGTYELKINNLTESDQGEYVCEI
SGEGGTSKTNLQFMGQAFKSIHEKVSKISETKKSDQKTTESTVTRKTEPKAPEPISSKPV
IVTGLQDTTVSSDSVAKFAVKATGEPRPTAIWTKDGKAITQGGKYKLSEDKGGFFLEIHK
TDTSDSGLYTCTVKNSAGSVSSSCKLTIKAIKDTEAQKVSTQKTSEITPQKKAVVQEEIS
QKALRSEEIKMSEAKSQEKLALKEEASKVLISEEVKKSAATSLEKSIVHEEITKTSQASE
EVRTHAEIKAFSTQMSINEGQRLVLKANIAGATDVKWVLNGVELTNSEEYRYGVSGSDQT
LTIKQASHRDEGILTCISKTKEGIVKCQYDLTLSKELSDAPAFISQPRSQNINEGQNVLF
TCEISGEPSPEIEWFKNNLPISISSNVSISRSRNVYSLEIRNASVSDSGKYTIKAKNFRG
QCSATASLMVLPLVEEPSREVVLRTSGDTSLQGSFSSQSVQMSASKQEASFSSFSSSSAS
SMTEMKFASMSAQSMSSMQESFVEMSSSSFMGISNMTQLESSTSKMLKAGIRGIPPKIEA
LPSDISIDEGKVLTVACAFTGEPTPEVTWSCGGRKIHSQEQGRFHIENTDDLTTLIIMDV
QKQDGGLYTLSLGNEFGSDSATVNIHIRSI
>sp|Q92626|PXDN_HUMAN Peroxidasin homolog OS=Homo sapiens OX=9606 GN=PXDN PE=1 SV=2
MAKRSRGPGRRCLLALVLFCAWGTLAVVAQKPGAGCPSRCLCFRTTVRCMHLLLEAVPAV
APQTSILDLRFNRIREIQPGAFRRLRNLNTLLLNNNQIKRIPSGAFEDLENLKYLYLYKN
EIQSIDRQAFKGLASLEQLYLHFNQIETLDPDSFQHLPKLERLFLHNNRITHLVPGTFNH
LESMKRLRLDSNTLHCDCEILWLADLLKTYAESGNAQAAAICEYPRRIQGRSVATITPEE
LNCERPRITSEPQDADVTSGNTVYFTCRAEGNPKPEIIWLRNNNELSMKTDSRLNLLDDG
TLMIQNTQETDQGIYQCMAKNVAGEVKTQEVTLRYFGSPARPTFVIQPQNTEVLVGESVT
LECSATGHPPPRISWTRGDRTPLPVDPRVNITPSGGLYIQNVVQGDSGEYACSATNNIDS
VHATAFIIVQALPQFTVTPQDRVVIEGQTVDFQCEAKGNPPPVIAWTKGGSQLSVDRRHL
VLSSGTLRISGVALHDQGQYECQAVNIIGSQKVVAHLTVQPRVTPVFASIPSDTTVEVGA
NVQLPCSSQGEPEPAITWNKDGVQVTESGKFHISPEGFLTINDVGPADAGRYECVARNTI
GSASVSMVLSVNVPDVSRNGDPFVATSIVEAIATVDRAINSTRTHLFDSRPRSPNDLLAL
FRYPRDPYTVEQARAGEIFERTLQLIQEHVQHGLMVDLNGTSYHYNDLVSPQYLNLIANL
SGCTAHRRVNNCSDMCFHQKYRTHDGTCNNLQHPMWGASLTAFERLLKSVYENGFNTPRG
INPHRLYNGHALPMPRLVSTTLIGTETVTPDEQFTHMLMQWGQFLDHDLDSTVVALSQAR
FSDGQHCSNVCSNDPPCFSVMIPPNDSRARSGARCMFFVRSSPVCGSGMTSLLMNSVYPR
EQINQLTSYIDASNVYGSTEHEARSIRDLASHRGLLRQGIVQRSGKPLLPFATGPPTECM
RDENESPIPCFLAGDHRANEQLGLTSMHTLWFREHNRIATELLKLNPHWDGDTIYYETRK
IVGAEIQHITYQHWLPKILGEVGMRTLGEYHGYDPGINAGIFNAFATAAFRFGHTLVNPL
LYRLDENFQPIAQDHLPLHKAFFSPFRIVNEGGIDPLLRGLFGVAGKMRVPSQLLNTELT
ERLFSMAHTVALDLAAINIQRGRDHGIPPYHDYRVYCNLSAAHTFEDLKNEIKNPEIREK
LKRLYGSTLNIDLFPALVVEDLVPGSRLGPTLMCLLSTQFKRLRDGDRLWYENPGVFSPA
QLTQIKQTSLARILCDNADNITRVQSDVFRVAEFPHGYGSCDEIPRVDLRVWQDCCEDCR
TRGQFNAFSYHFRGRRSLEFSYQEDKPTKKTRPRKIPSVGRQGEHLSNSTSAFSTRSDAS
GTNDFREFVLEMQKTITDLRTQIKKLESRLSTTECVDAGGESHANNTKWKKDACTICECK
DGQVTCFVEACPPATCAVPVNIPGACCPVCLQKRAEEKP

>sp|Q96A11|G3ST3_HUMAN Galactose-3-O-sulfotransferase 3 OS=Homo sapiens OX=9606 GN=GAL3ST3 PE=1 SV=1
MPPILQRLQQATKMMSRRKILLLVLGCSTVSLLIHQGAQLSWYPKLFPLSCPPLRNSPPR
PKHMTVAFLKTHKTAGTTVQNILFRFAERHNLTVALPHPSCEHQFCYPRNFSAHFVHPAT
RPPHVLASHLRFDRAELERLMPPSTVYVTILREPAAMFESLFSYYNQYCPAFRRVPNASL
EAFLRAPEAYYRAGEHFAMFAHNTLAYDLGGDNERSPRDDAAYLAGLIRQVEEVFSLVMI
AEYFDESLVLLRRLLAWDLDDVLYAKLNARAASSRLAAIPAALARAARTWNALDAGLYDH
FNATFWRHVARAGRACVEREARELREARQRLLRRCFGDEPLLRPAAQIRTKQLQPWQPSR
KVDIMGYDLPGGGAGPATEACLKLAMPEVQYSNYLLRKQKRRGGARARPEPVLDNPPPRP
IRVLPRGPQGP
>sp|Q96DE0|NUD16_HUMAN U8 snoRNA-decapping enzyme OS=Homo sapiens OX=9606 GN=NUDT16 PE=1 SV=2
MAGARRLELGEALALGSGWRHACHALLYAPDPGMLFGRIPLRYAILMQMRFDGRLGFPGG
FVDTQDRSLEDGLNRELREELGEAAAAFRVERTDYRSSHVGSGPRVVAHFYAKRLTLEEL
LAVEAGATRAKDHGLEVLGLVRVPLYTLRDGVGGLPTFLENSFIGSAREQLLEALQDLGL
LQSGSISGLKIPAHH
>sp|Q96HF1|SFRP2_HUMAN Secreted frizzled-related protein 2 OS=Homo sapiens OX=9606 GN=SFRP2 PE=1 SV=2
MLQGPGSLLLLFLASHCCLGSARGLFLFGQPDFSYKRSNCKPIPANLQLCHGIEYQNMRL
PNLLGHETMKEVLEQAGAWIPLVMKQCHPDTKKFLCSLFAPVCLDDLDETIQPCHSLCVQ
VKDRCAPVMSAFGFPWPDMLECDRFPQDNDLCIPLASSDHLLPATEEAPKVCEACKNKND
DDNDIMETLCKNDFALKIKVKEITYINRDTKIILETKSKTIYKLNGVSERDLKKSVLWLK
DSLQCTCEEMNDINAPYLVMGQKQGGELVITSVKRWQKGQREFKRISRSIRKLQC
>sp|Q99497|PARK7_HUMAN Parkinson disease protein 7 OS=Homo sapiens OX=9606 GN=PARK7 PE=1 SV=2
MASKRALVILAKGAEEMETVIPVDVMRRAGIKVTVAGLAGKDPVQCSRDVVICPDASLED
AKKEGPYDVVVLPGGNLGAQNLSESAAVKEILKEQENRKGLIAAICAGPTALLAHEIGFG
SKVTTHPLAKDKMMNGGHYTYSENRVEKDGLILTSRGPGTSFEFALAIVEALNGKEVAAQ
VKAPLVLKD
>sp|Q99733|NP1L4_HUMAN Nucleosome assembly protein 1-like 4 OS=Homo sapiens OX=9606 GN=NAP1L4 PE=1 SV=1
MADHSFSDGVPSDSVEAAKNASNTEKLTDQVMQNPRVLAALQERLDNVPHTPSSYIETLP
KAVKRRINALKQLQVRCAHIEAKFYEEVHDLERKYAALYQPLFDKRREFITGDVEPTDAE
SEWHSENEEEEKLAGDMKSKVVVTEKAAATAEEPDPKGIPEFWFTIFRNVDMLSELVQEY
DEPILKHLQDIKVKFSDPGQPMSFVLEFHFEPNDYFTNSVLTKTYKMKSEPDKADPFSFE
GPEIVDCDGCTIDWKKGKNVTVKTIKKKQKHKGRGTVRTITKQVPNESFFNFFNPLKASG
DGESLDEDSEFTLASDFEIGHFFRERIVPRAVLYFTGEAIEDDDNFEEGEEGEEEELEGD
EEGEDEDDAEINPKV
>sp|Q99969|RARR2_HUMAN Retinoic acid receptor responder protein 2 OS=Homo sapiens OX=9606 GN=RARRES2 PE=1 SV=1
MRRLLIPLALWLGAVGVGVAELTEAQRRGLQVALEEFHKHPPVQWAFQETSVESAVDTPF
PAGIFVRLEFKLQQTSCRKRDWKKPECKVRPNGRKRKCLACIKLGSEDKVLGRLVHCPIE
TQVLREAEEHQETQCLRVQRAGEDPHSFYFPGQFAFSKALPRS
>sp|Q9BSG5|RTBDN_HUMAN Retbindin OS=Homo sapiens OX=9606 GN=RTBDN PE=1 SV=2

MDCRVHMRPIGLTWVLQLTLAWILLEACGGSRPLQARSQQHHGLAADLGKGKLHLAGPCC
PSEMDTTETSGPGNHPERCGVPSPECESFLEHLQRALRSRFRLRLLGVRQAQPLCEELCQ
AWFANCEDDITCGPTWLPLSEKRGCEPSCLTYGQTFADGTDLCRSALGHALPVAAPGARH
CFNISISAVPRPRPGRRGREAPSRRSRSPRTSILDAAGSGSGSGSGSGP
>sp|Q9BY67|CADM1_HUMAN Cell adhesion molecule 1 OS=Homo sapiens OX=9606
GN=CADM1 PE=1 SV=2
MASVVLPSGSQCAAAAAAAAPPGLRLRLLLLLFSAAALIPTGDGQNLFTKDVTVIEGEVA
TISCQVNKSDDSVIQLLNPNRQTIYFRDFRPLKDSRFQLLNFSSSELKVSLTNVSISDEG
RYFCQLYTDPPQESYTTITVLVPPRNLMIDIQKDTAVEGEEIEVNCTAMASKPATTIRWF
KGNTELKGKSEVEEWSDMYTVTSQLMLKVHKEDDGVPVICQVEHPAVTGNLQTQRYLEVQ
YKPQVHIQMTYPLQGLTREGDALELTCEAIGKPQPVMVTWVRVDDEMPQHAVLSGPNLFI
NNLNKTDNGTYRCEASNIVGKAHSDYMLYVYDPPTTIPPPTTTTTTTTTTTTILTIITD
SRAGEEGSIRAVDHAVIGGVVAVVVFAMLCLLIILGRYFARHKGTYFTHEAKGADDAADA
DTAIINAEGGQNNSEEKKEYFI
>sp|Q9BYX7|ACTBM_HUMAN Putative beta-actin-like protein 3 OS=Homo sapiens OX=9606
GN=POTEKP PE=5 SV=1
MDDDTAVLVIDNGSGMCKAGFAGDDAPQAVFPSIVGRPRHQGMMEGMHQKESYVGKEAQS
KRGMLTLKYPMEHGIITNWDDMEKIWHHTFYNELRVAPEEHPILLTEAPLNPKANREKMT
QIMFETFNTPAMYVAIQAVLSLYTSGRTTGIVMDSGDGFTHTVPIYEGNALPHATLRLDL
AGRELTDYLMKILTERGYRFTTTAEQEIVRDIKEKLCYVALDSEQEMAMAASSSSVEKSY
ELPDGQVITIGNERFRCPEALFQPCFLGMESCGIHKTTFNSIVKSDVDIRKDLYTNTVLS
GGTTMYPGIAHRMQKEITALAPSIMKIKIIAPPKRKYSVWVGGSILASLSTFQQMWISKQ
EYDESGPSIVHRKCF
>sp|Q9NQ79|CRAC1_HUMAN Cartilage acidic protein 1 OS=Homo sapiens OX=9606
GN=CRTAC1 PE=1 SV=2
MAPSADPGMSRMLPFLLLLWFLPITEGSQRAEPMFTAVTNSVLPPDYDSNPTQLNYGVAV
TDVDHDGDFEIVVAGYNGPNLVLKYDRAQKRLVNIAVDERSSPYYALRDRQGNAIGVTAC
DIDGDGREEIYFLNTNNAFSGVATYTDKLFKFRNNRWEDILSDEVNVARGVASLFAGRSV
ACVDRKGSGRYSIYIANYAYGNVGPDALIEMDPEASDLSRGILALRDVAAEAGVSKYTGG
RGVSVGPILSSSASDIFCDNENGPNFLFHNRGDGTFVDAAASAGVDDPHQHGRGVALADF
NRDGKVDIVYGNWNGPHRLYLQMSTHGKVRFRDIASPKFSMPSPVRTVITADFDNDQELE
IFFNNIAYRSSSANRLFRVIRREHGDPLIEELNPGDALEPEGRGTGGVVTDFDGDGMLDL
ILSHGESMAQPLSVFRGNQGFNNNWLRVVPRTRFGAFARGAKVVLYTKKSGAHLRIIDGG
SGYLCEMEPVAHFGLGKDEASSVEVTWPDGKMVSRNVASGEMNSVLEILYPRDEDTLQDP
APLECGQGFSQQENGHCMDTNECIQFPFVCPRDKPVCVNTYGSYRCRTNKKCSRGYEPNE
DGTACVGTLGQSPGPRPTTPTAAAATAAAAAAAGAATAAPVLVDGDLNLGSVVKESCEPS
C
>sp|Q9NR99|MXRA5_HUMAN Matrix-remodeling-associated protein 5 OS=Homo sapiens
OX=9606 GN=MXRA5 PE=1 SV=3
MPKRAHWGALSVVLILLWGHPRVALACPHPCACYVPSEVHCTFRSLASVPAGIAKHVERI
NLGFNSIQALSETSFAGLTKLELLMIHGNEIPSIPDGALRDLSSLQVFKFSYNKLRVITG
QTLQGLSNLMRLHIDHNKIEFIHPQAFNGLTSLRLLHLEGNLLHQLHPSTFSTFTFLDYF
RLSTIRHLYLAENMVRTLPASMLRNMPLLENLYLQGNPWTCDCEMRWFLEWDAKSRGILK
CKKDKAYEGGQLCAMCFSPKKLYKHEIHKLKDMTCLKPSIESPLRQNRSRSIEEEQEQEE

DGGSQLILEKFQLPQWSISLNMTDEHGNMVNLVCDIKKPMDVYKIHLNQTDPPDIDINAT
VALDFECPMTRENYEKLWKLIAYYSEVPVKLHRELMLSKDPRVSYQYRQDADEEALYYTG
VRAQILAEPEWVMQPSIDIQLNRRQSTAKKVLLSYYTQYSQTISTKDTRQARGRSWVMIE
PSGAVQRDQTVLEGGPCQLSCNVKASESPSIFWVLPDGSILKAPMDDPDSKFSILSSGWL
RIKSMEPSDSGLYQCIAQVRDEMDRMVYRVLVQSPSTQPAEKDTVTIGKNPGESVTLPCN
ALAIPEAHLSWILPNRRIINDLANTSHVYMLPNGTLSIPKVQVSDSGYYRCVAVNQQGAD
HFTVGITVTKKGSGLPSKRGRRPGAKALSRVREDIVEDEGGSGMGDEENTSRRLLHPKDQ
EVFLKTKDDAINGDKKAKKGRRKLKLWKHSEKEPETNVAEGRRVFESRRRINMANKQINP
ERWADILAKVRGKNLPKGTEVPPLIKTTSPPSLSLEVTPPFPAISPPSASPVQTVTSAEE
SSADVPLLGEEEHVLGTISSASMGLEHNHNGVILVEPEVTSTPLEEVVDDLSEKTEEITS
TEGDLKGTAAPTLISEPYEPSPTLHTLDTVYEKPTHEETATEGWSAADVGSSPEPTSSEY
EPPLDAVSLAESEPMQYFDPDLETKSQPDEDKMKEDTFAHLTPTPTIWVNDSSTSQLFED
STIGEPGVPGQSHLQGLTDNIHLVKSSLSTQDTLLIKKGMKEMSQTLQGGNMLEGDPTHS
RSSESEGQESKSITLPDSTLGIMSSMSPVKKPAETTVGTLLDKDTTTATTTPRQKVAPSS
TMSTHPSRRRPNGRRRLRPNKFRHRHKQTPPTTFAPSETFSTQPTQAPDIKISSQVESSL
VPTAWVDNTVNTPKQLEMEKNAEPTSKGTPRRKHGKRPNKHRYTPSTVSSRASGSKPSPS
PENKHRNIVTPSSETILLPRTVSLKTEGPYDSLDYMTTTRKIYSSYPKVQETLPVTYKPT
SDGKEIKDDVATNVDKHKSDILVTGESITNAIPTSRSLVSTMGEFKEESSPVGFPGTPTW
NPSRTAQPGRLQTGIPVTTSGENLTDPPLLKELEDVDFTSEFLSSLTVSTPFHQEEAGSS
TTLSSIKVEVASSQAETTTLDQDHLETTVAILLSETRPQNHTPTAARMKEPASSSPSTIL
MSLGQTTTTKPALPSPRISQASRDSKENVFLNYVGNPETEATPVNNEGTQHMSGPNELST
PSSDQDAFNLSTKLELEKQVFGSRSLPRGPDSQRQDGRVHASHQLTRVPAKPILPTATVR
LPEMSTQSASRYFVTSQSPRHWTNKPEITTYPSGALPENKQFTTPRLSSTTIPLPLHMSK
PSIPSKFTDRRTDQFNGYSKVFGNNNIPEARNPVGKPPSPRIPHYSNGRLPFFTNKTLSF
PQLGVTRRPQIPTSPAPVMRERKVIPGSYNRIHSHSTFHLDFGPPAPPLLHTPQTTGSPS
TNLQNIPMVSSTQSSISFITSSVQSSGSFHQSSSKFFAGGPPASKFWSLGEKPQILTKSP
QTVSVTAETDTVFPCEATGKPKPFVTWTKVSTGALMTPNTRIQRFEVLKNGTLVIRKVQV
QDRGQYMCTASNLHGLDRMVVLLSVTVQQPQILASHYQDVTVYLGDTIAMECLAKGTPAP
QISWIFPDRRVWQTVSPVEGRITLHENRTLSIKEASFSDRGVYKCVASNAAGADSLAIRL
HVAALPPVIHQEKLENISLPPGLSIHIHCTAKAAPLPSVRWVLGDGTQIRPSQFLHGNLF
VFPNGTLYIRNLAPKDSGRYECVAANLVGSARRTVQLNVQRAAANARITGTSPRRTDVRY
GGTLKLDCSASGDPWPRILWRLPSKRMIDALFSFDSRIKVFANGTLVVKSVTDKDAGDYL
CVARNKVGDDYVVLKVDVVMKPAKIEHKEENDHKVFYGGDLKVDCVATGLPNPEISWSLP
DGSLVNSFMQSDDSGGRTKRYVVFNNGTLYFNEVGMREEGDYTCFAENQVGKDEMRVRVK
VVTAPATIRNKTYLAVQVPYGDVVTVACEAKGEPMPKVTWLSPTNKVIPTSSEKYQIYQD
GTLLIQKAQRSDSGNYTCLVRNSAGEDRKTVWIHVNVQPPKINGNPNPITTVREIAAGGS
RKLIDCKAEGIPTPRVLWAFPEGVVLPAPYYGNRITVHGNGSLDIRSLRKSDSVQLVCMA
RNEGGEARLILQLTVLEPMEKPIFHDPISEKITAMAGHTISLNCSAAGTPTPSLVWVLPN
GTDLQSGQQLQRFYHKADGMLHISGLSSVDAGAYRCVARNAAGHTERLVSLKVGLKPEAN
KQYHNLVSIINGETLKLPCTPPGAGQGRFSWTLPNGMHLEGPQTLGRVSLLDNGTLTVRE
ASVFDRGTYVCRMETEYGPSVTSIPVIVIAYPPRITSEPTPVIYTRPGNTVKLNCMAMGI
PKADITWELPDKSHLKAGVQARLYGNRFLHPQGSLTIQHATQRDAGFYKCMAKNILGSDS
KTTYIHVF

>sp|Q9P2V4|LRIT1_HUMAN Leucine-rich repeat, immunoglobulin-like domain and transmembrane domain-containing protein 1 OS=Homo sapiens OX=9606 GN=LRIT1 PE=2 SV=1
MRVALGMLWLLALAWPPQARGFCPSQCSCSLHIMGDGSKARTVVCNDPDMTLPPASIPPD
TSRLRLERTAIRRVPGEAFRPLGRLEQLWLPYNALSELNALMLRGLRRLRELRLPGNRLA
AFPWAALRDAPKLRLLDLQANRLSAVPAEAARFLENLTFLDLSSNQLMRLPQELIVSWAH
LETGIFPPGHHPRRVLGLQDNPWACDCRLYDLVHLLDGWAPNLAFIETELRCASPRSLAG
VAFSQLELRKCQGPELHPGVASIRSLLGGTALLRCGATGVPGPEMSWRRANGRPLNGTVH
QEVSSDGTSWTLLGLPAVSHLDSGDYICQAKNFLGASETVISLIVTEPPTSTEHSGSPGA
LWARTGGGGEAAAYNNKLVARHVPQIPKPAVLATGPSVPSTKEELTLEHFQMDALGELSD
GRAGPSEARMVRSVKVVGDTYHSVSLVWKAPQAKNTTAFSVLYAVFGQHSMRRVIVQPGK
TRVTITGLLPKTKYVACVCVQGLVPRKEQCVIFSTNEVVDAENTQQLINVVVISVAIVIA
LPLTLLVCCSALQKRCRKCFNKDSTEATVTYVNLERLGYSEDGLEELSRHSVSEADRLLS
ARSSVDFQAFGVKGGRRINEYFC
>sp|Q9UBM4|OPT_HUMAN Opticin OS=Homo sapiens OX=9606 GN=OPTC PE=1 SV=1
MRLLAFLSLLALVLQETGTASLPRKERKRREEQMPREGDSFEVLPLRNDVLNPDNYGEVI
DLSNYEELTDYGDQLPEVKVTSLAPATSISPAKSTTAPGTPSSNPTMTRPTTAGLLLSSQ
PNHGLPTCLVCVCLGSSVYCDDIDLEDIPPLPRRTAYLYARFNRISRIRAEDFKGLTKLK
RIDLSNNLISSIDNDAFRLLHALQDLILPENQLEALPVLPSGIEFLDVRLNRLQSSGIQP
AAFRAMEKLQFLYLSDNLLDSIPGPLPLSLRSVHLQNNLIETMQRDVFCDPEEHKHTRRQ
LEDIRLDGNPINLSLFPSAYFCLPRLPIGRFT
>sp|Q9UKZ1|CNO11_HUMAN CCR4-NOT transcription complex subunit 11 OS=Homo sapiens OX=9606 GN=CNOT11 PE=1 SV=1
MPGGGASAASGRLLTAAEQRGSREAAGSASRSGFGGSGGGRGGASGPGSGSGGPGGPAGR
MSLTPKELSSLLSIISEEAGGGSTFEGLSTAFHHYFSKADHFRLGSVLVMLLQQPDLLPS
AAQRLTALYLLWEMYRTEPLAANPFAASFAHLLNPAPPARGGQEPDRPPLSGFLPPITPP
EKFFLSQLMLAPPRELFKKTPRQIALMDVGNMGQSVDISGLQLALAERQSELPTQSKASF
PSILSDPDPDSSNSGFDSSVASQITEALVSGPKPPIESHFRPEFIRPPPPLHICEDELAW
LNPTEPDHAIQWDKSMCVKNSTGVEIKRIMAKAFKSPLSSPQQTQLLGELEKDPKLVYHI
GLTPAKLPDLVENNPLVAIEMLLKLMQSSQITEYFSVLVNMDMSLHSMEVVNRLTTAVDL
PPEFIHLYISNCISTCEQIKDKYMQNRLVRLVCVFLQSLIRNKIINVQDLFIEVQAFCIE
FSRIREAAGLFRLLKTLDTGETPSETKMSK
>sp|Q9UPZ6|THS7A_HUMAN Thrombospondin type-1 domain-containing protein 7A OS=Homo sapiens OX=9606 GN=THSD7A PE=1 SV=4
MGLQARRWASGSRGAAGPRRGVLQLLPLPLPLPLLLLLLLRPGAGRAAAQGEAEAPTLYL
WKTGPWGRCMGDECGPGGIQTRAVWCAHVEGWTTLHTNCKQAERPNNQQNCFKVCDWHKE
LYDWRLGPWNQCQPVISKSLEKPLECIKGEEGIQVREIACIQKDKDIPAEDIICEYFEPK
PLLEQACLIPCQQDCIVSEFSAWSECSKTCGSGLQHRTRHVVAPPQFGGSGCPNLTEFQV
CQSSPCEAEELRYSLHVGPWSTCSMPHSRQVRQARRRGKNKEREKDRSKGVKDPEARELI
KKKRNRNRQNRQENKYWDIQIGYQTREVMCINKTGKAADLSFCQQEKLPMTFQSCVITKE
CQVSEWSEWSPCSKTCHDMVSPAGTRVRTRTIRQFPIGSEKECPEFEEKEPCLSQGDGVV
PCATYGWRTTEWTECRVDPLLSQQDKRRGNQTALCGGGIQTREVYCVQANENLLSQLSTH
KNKEASKPMDLKLCTGPIPNTTQLCHIPCPTECEVSPWSAWGPCTYENCNDQQGKKGFKL
RKRRITNEPTGGSGVTGNCPHLLEAIPCEEPACYDWKAVRLGNCEPDNGKECGPGTQVQE
VVCINSDGEEVDRQLCRDAIFPIPVACDAPCPKDCVLSTWSTWSSCSHTCSGKTTEGKQI

RARSILAYAGEEGGIRCPNSSALQEVRSCNEHPCTVYHWQTGPWGQCIEDTSVSSFNTTT
TWNGEASCSVGMQTRKVICVRVNVGQVGPKKCPESLRPETVRPCLLPCKKDCIVTPYSDW
TSCPSSCKEGDSSIRKQSRHRVIIQLPANGGRDCTDPLYEEKACEAPQACQSYRWKTHKW
RRCQLVPWSVQQDSPGAQEGCGPGRQARAITCRKQDGGQAGIHECLQYAGPVPALTQACQ
IPCQDDCQLTSWSKFSSCNGDCGAVRTRKRTLVGKSKKKEKCKNSHLYPLIETQYCPCDK
YNAQPVGNWSDCILPEGKVEVLLGMKVQGDIKECGQGYRYQAMACYDQNGRLVETSRCNS
HGYIEEACIIPCPSDCKLSEWSNWSRCSKSCGSGVKVRSKWLREKPYNGGRPCPKLDHVN
QAQVYEVVPCHSDCNQYLWVTEPWSICKVTFVNMRENCGEGVQTRKVRCMQNTADGPSEH
VEDYLCDPEEMPLGSRVCKLPCPEDCVISEWGPWTQCVLPCNQSSFRQRSADPIRQPADE
GRSCPNAVEKEPCNLNKNCYHYDYNVTDWSTCQLSEKAVCGNGIKTRMLDCVRSDGKSVD
LKYCEALGLEKNWQMNTSCMVECPVNCQLSDWSPWSECSQTCGLTGKMIRRRTVTQPFQG
DGRPCPSLMDQSKPCPVKPCYRWQYGQWSPCQVQEAQCGEGTRTRNISCVVSDGSADDFS
KVVDEEFCADIELIIDGNKNMVLEESCSQPCPGDCYLKDWSSWSLCQLTCVNGEDLGFGG
IQVRSRPVIIQELENQHLCPEQMLETKSCYDGQCYEYKWMASAWKGSSRTVWCQRSDGIN
VTGGCLVMSQPDADRSCNPPCSQPHSYCSETKTCHCEEGYTEVMSSNSTLEQCTLIPVVV
LPTMEDKRGDVKTSRAVHPTQPSSNPAGRGRTWFLQPFGPDGRLKTWVYGVAAGAFVLLI
FIVSMIYLACKKPKKPQRRQNNRLKPLTLAYDGDADM
