# Supplementary material for: Effects of Aging on Intrinsic Protein Disorder in Human Lenses and Zonules
Source: Cell Biochem Biophys. 2024 Aug 8;82(4):3667–79. doi: 10.1007/s12013-024-01455-x (PMC11576620; doi:10.1007/s12013-024-01455-x)
Supplement: Supplementary file 2 — Supplementary S3 25-37 year old Lens Fasta [file 12013_2024_1455_MOESM2_ESM.pdf]

>sp|A0AVT1|UBA6_HUMAN Ubiquitin-like modifier-activating enzyme 6 OS=Homo sapiens OX=9606 GN=UBA6 PE=1 SV=1
MEGSEPVAAHQGEEASCSSWGTGSTNKNLPIMSTASVEIDDALYSRQRYVLGDTAMQKMA
KSHVFLSGMGGLGLEIAKNLVLAGIKAVTIHDTEKCQAWDLGTNFFLSEDDVVNKRNRAE
AVLKHIAELNPYVHVTSSSVPFNETTDLSFLDKYQCVVLTEMKLPLQKKINDFCRSQCPP
IKFISADVHGIWSRLFCDFGDEFEVLDTTGEEPKEIFISNITQANPGIVTCLENHPHKLE
TGQFLTFREINGMTGLNGSIQQITVISPFSFSIGDTTELEPYLHGGIAVQVKTPKTVFFE
SLERQLKHPKCLIVDFSNPEAPLEIHTAMLALDQFQEKYSRKPNVGCQQDSEELLKLATS
ISETLEEKPDVNADIVHWLSWTAQGFLSPLAAAVGGVASQEVLKAVTGKFSPLCQWLYLE
AADIVESLGKPECEEFLPRGDRYDALRACIGDTLCQKLQNLNIFLVGCGAIGCEMLKNFA
LLGVGTSKEKGMITVTDPDLIEKSNLNRQFLFRPHHIQKPKSYTAADATLKINSQIKIDA
HLNKVCPTTETIYNDEFYTKQDVIITALDNVEARRYVDSRCLANLRPLLDSGTMGTKGHT
EVIVPHLTESYNSHRDPPEEEIPFCTLKSFPAAIEHTIQWARDKFESSFSHKPSLFNKFW
QTYSSAEEVLQKIQSGHSLEGCFQVIKLLSRRPRNWSQCVELARLKFEKYFNHKALQLLH
CFPLDIRLKDGSLFWQSPKRPPSPIKFDLNEPLHLSFLQNAAKLYATVYCIPFAEEDLSA
DALLNILSEVKIQEFKPSNKVVQTDETARKPDHVPISSEDERNAIFQLEKAILSNEATKS
DLQMAVLSFEKDDDHNGHIDFITAASNLRAKMYSIEPADRFKTKRIAGKIIPAIATTTAT
VSGLVALEMIKVTGGYPFEAYKNCFLNLAIPIVVFTETTEVRKTKIRNGISFTIWDRWTV
HGKEDFTLLDFINAVKEKYGIEPTMVVQGVKMLYVPVMPGHAKRLKLTMHKLVKPTTEKK
YVDLTVSFAPDIDGDEDLPGPPVRYYFSHDTD
>tr|A2J423|A2J423_HUMAN Anti-Mpl scFv (Fragment) OS=Homo sapiens OX=9606 PE=2 SV=1
QVQLVQSGGGLVRPGGSLSLSCAVSGITLRTYGMHWVRQAPGKGLEWVAGISFDGRSEYY
ADSVQGRFTISRDSSKNTLYLQMNSLRAEDTAVYYCARGAHYGFDIWGQGTMVTVSSGGG
GTGGGGSGGGGSDIQMTQSPSTLSASIGDRVTITCRASEGIYHWLAWYQQKPGKAPKLLI
YKASSLASGAPSRFSGSGSGTDFTLTISSLQPDDFATYYCQQYSNYPLTFGGGTKLEIK
>sp|A2RU48|SMCO3_HUMAN Single-pass membrane and coiled-coil domain-containing protein 3 OS=Homo sapiens OX=9606 GN=SMCO3 PE=1 SV=1
MAQSDFLYPENPKRREEVNRLHQQLLDCLSDSFDVTNKLTEVLNMHLGCRLASIEMKRDG
TIKENCDLIIQAIMKIQKELQKVDEALKDKLEPTLYRKLQDIKEKETDKIAIVQKVISVI
LGEATSAASAVAVKLVGSNVTTGIINKLVTVLAQIGASLLGSIGVAVLGLGIDMIVRAIL
GAVEKTQLQAAIKSYEKHLVEFKSASEKYNHAITEVINTVKHQMK
>sp|A4D1U4|DEN11_HUMAN DENN domain-containing protein 11 OS=Homo sapiens OX=9606 GN=DENND11 PE=1 SV=1
MVEQGDAAPLLRWAEGPAVSLPQAPQPQAGGWGRGGGGGARPAAEPPRRREPEEPAAPEV
LLQPGRLELGDVEEDQVVAVFVVTFDPRSGNMVEWCLPQDIDLEGVEFKSMASGSHKIQS
DFIYFRKGPFFGLACFANMPVESELERGARMKSVGILSPSYTLLYRYMHFLENQVRHQLE
MPGHYSHLAAFYEDKKGVLHAGPGRGSSLPPVYWLPSIHRYMYPEMKITHPAGCMSQFIK
FFGEQILILWKFALLRKRILIFSPPPVGVVCYRVYCCCCLANVSLPGIGGTIPESKPFFY
VNVADIESLEVEVSYVACTTEKIFEEKRELYDVYVDNQNVKTHHDHLQPLLKINSADREK
YRRLNEQRQMLLYSQEVEEDYNPCEEDLFVLFFLEQNNRIFQTLLEVSASQDKTLTAEHA
RGMGLDPQGDRSFLLDLLEAYGIDVMLVIDNPCCP
>tr|B2RDW1|B2RDW1_HUMAN Ubiquitin-ribosomal protein eS31 fusion protein OS=Homo sapiens OX=9606 GN=RPS27A PE=2 SV=1
MQIFVKTLTGKTITLEVEPSDTIENVKAKIQDKEGIPPDQQRLIFAGKQLEDGRTLSDYN

IQKESTLHLVLRLRGGAKKRKKKSYTTPKKNKHKRKKVKLAVLKYYKVDENGKISRLRRE
CPSDECGAGVFMASHFDRHYCGKCCLTYCFNKPEDK
>sp|B3SHH9|TM114_HUMAN Transmembrane protein 114 OS=Homo sapiens OX=9606
GN=TMEM114 PE=1 SV=2
MRVHLGGLAGAAALTGALSFVLLAAAIGTDFWYIIDTERLERTGPGAQDLLGSINRSQPE
PLSSHSGLWRTCRVQSPCTPLMNPFRLENVTVSESSRQLLTMHGTFVILLPLSLILMVFG
GMTGFLSFLLQAYLLLLLTGILFLFGAMVTLAGISVYIAYSAAAFREALCLLEEKALLDQ
VDISFGWSLALGWISFIAELLTGAAFLAAARELSLRRRQDQAI
>tr|B4DL86|B4DL86_HUMAN 6-phosphogluconate dehydrogenase, decarboxylating OS=Homo
sapiens OX=9606 PE=2 SV=1
MTTALWSVLLIGLSPKLMISWPMRQAVDDFIEKLVPLLDTGDIIIDGGNSEYRDTTRRCR
DLKAKGILFVGSGVSGGEEGARYGPSLMPGGNKEAWPHIKTIFQGIAAKVGTGEPCCDWV
GDEGAGHFVKMVHNGIEYGDMQLICEAYHLMKDVLGMAQDEMAQAFEDWNKTELDSFLIE
ITANILKFQDTDGKHLLPKIRDSAGQKGTGKWTAISALEYGVPVTLIGEAVFARCLSSLK
DERIQASKKLKGPQKFQFDGDKKSFLEDIRKALYASKIISYAQGFMLLRQAATEFGWTLN
YGGIALMWRGGCIIRSVFLGKIKDAFDRNPELQDLLLDDFFKSAVENCQDSWRRAVSTGV
QAGIPMPCFTTALSFYDGYRHEMLPASLIQAQRDYFGAHTYELLAKPGQFIHTNWTGHGG
TVSSSSYNA
>tr|B4DRT3|B4DRT3_HUMAN Pyruvate kinase OS=Homo sapiens OX=9606 PE=2 SV=1
MSKPHSEAGTAFIQTQQLHAAMADTFLEHMCRLDIDSPPITARNTGIICTIGPASRSVET
LKEMIKSGMNVARLNFSHGTHEYHAETIKNVRTATESFASDPIIKGSGTAEVELKKGSTL
KITLDNAYMEKCDENILWLDYKNICKVVEVGSKIYVDDGLISLQVKQKGADFLVTEVENG
GSLGSKKGVNLPGAAVDLPAVSEKDIQDLKFGVEQDVDMVFASFIRKASDVHEVRKVLGE
KGKNIKIISKIENHEGVRRFDEILEASDGIMVARGDLGIEIPAEKVFLAQKMMIGRCNRA
GKPVICATQMLESMIKKPRPTRAEGSDVANAVLDGADCIMLSGETAKGDYPLEAVRMQHL
IAREAEAAMFHRKLFEELVRASSHSTDLMEAMAMGSVEASYKCLAAALIVLTESGRSAHQ
VARYRPRAPIIAVTRNPQTARQAHLYRGIFPVLCKDPVQEAWAEDVDLRVNFAMNVGKAR
GFFKKGDVVIVLTGWRPGSGFTNTMRVVPVP
>tr|B4DRV9|B4DRV9_HUMAN glyceraldehyde-3-phosphate dehydrogenase (phosphorylating)
OS=Homo sapiens OX=9606 PE=2 SV=1
MGKVKVGVNGFGRIGRLVTRAAFNSGKVDIVAINDPFIDLNYMVYMFQYDSTHGKFHGTV
KAENGKLVINGNPITIFQERDPSKIKWGDAGAEYVVESTGVFTTMEKAGAHLQGGAKRVI
ISAPSADAPMFVMGVNHEKYDNSLKIISNASCTTNCLAPLAKVIHDNFGIVEGLMTTVHA
ITATLREAHWHGLPCPHCQRVSGGPDLPSRKTCQI
>tr|B4DVQ0|B4DVQ0_HUMAN cDNA FLJ58286, highly similar to Actin, cytoplasmic 2 OS=Homo
sapiens OX=9606 PE=2 SV=1
MEEEIAALVIDNGSGMCKAGFAGDDAPRAVFPSIVGRPRHQGVMVGMGQKDSYVGDEAQS
KRGILTLKYPIEHGIVTNWDDMEKIWHHTFYNELRVAPEEHPVLLTEAPLNPKANREKMT
QIMFETYALPHAILRLDLAGRGLTDYLMKILTERGYSFTTTAEREIVRDIKEKLCYVALD
FEQEMATAASSSSLEKSYELPDGQVITIGNERFRCPEALFQPSFLGMESCGIHETTFNSI
MKCDVDIRKDLYANTVLSGGTTMYPGIADRMQKEITALAPSTMKIKIIAPPERKYSVWIG
GSILASLSTFQQMWISKQEYDESGPSIVHRKCF
>tr|B4DYU3|B4DYU3_HUMAN cDNA FLJ57235, highly similar to Aldehyde dehydrogenase 1A3
OS=Homo sapiens OX=9606 PE=2 SV=1

MATANGAVENGQPDRKPPALPRPIRNLEVKFTKIFINNEWHESKSGKKFATCNPSTREQI
CEVEEGDKPDVDKAVEAAQVAFQRGSPWRRLDALSRGRLLHQLADLVERDRATLAALETM
DTGKPFLHAFFIDLEGCIRTLRYFAGWADTSLIKEAGFPPGVVNIVPGFGPIVGAAISSH
PQINKIAFTGSTEVGKLVKEAASRSNLKRVTLELGGKNPCIVCADADLDLAVECAHQGVF
FNQGQCCTAASRVFVEEQVYSEFVRRSVEYAKKRPVGDPFDVKTEQGPQIDQKQFDKILE
LIESGKKEGAKLECGGSAMEDKGLFIKPTVFSEVTDNMRIAKEEIFGPVQPILKFKSIEE
VIKRANSTDYGLTAAVFTKNLDKALKLASALESGTVWINCYNALYAQAPFGGFKMSGNGR
ELGEYALAEYTEVKTVTIKLGDKNP
>tr|B4E1T1|B4E1T1_HUMAN Keratin, type II cytoskeletal 5 OS=Homo sapiens OX=9606 PE=2 SV=1
MSRQSSVSFRSGGSRSFSTASAITPSVSRTSFTSVSRSGGGGGGGFGRVSLAGACGVGGY
GFGGGAGSGFGFGGGAGGGFGLGGGAGFGGGFGGPGFPVCPPGGIQEVTVNQSLLTPLNL
QIDPSIQRVRTEEREQIKTLNNKFASFIDKVRFLEQQNKVLETKWTLLQEQGTKTVRQNL
EPLFEQYINNLRRQLDSIVGERGRLDSELRNMQDLVEDLKNKYEDEINKRTTAENEFVML
KKDVDAAYMNKVELEAKVDALMDEINFMKMFFDAELSQMQTHVSDTSVVLSMDNNRNLDL
DSIIAEVKAQYEEIANRSRTEAESWYQTKYEELQQTAGRHGDDLRNTKHEISEMNRMIQR
LRAEIDNVKKQCANLQNAIADAEQRGELALKDARNKLAELEEALQKAKQDMARLLREYQE
LMNTKLALDVEIATYRKLLEGEECRLSGEGVGPVNISVVTSSVSSGYGSGGGYGGGLGGG
LGGGLGGGLAGGSSGSYYSSSSGGVGLGGGLSVGGSGFSASSGRGLGVGFGSGGGSSSSV
KFVSTTSSSRKSFKS
>tr|B7Z6W1|B7Z6W1_HUMAN Thromboxane-A synthase OS=Homo sapiens OX=9606 PE=2 SV=1
MMEALGFLKLEVNGPMVTVALSVALLALLKWYSTSAFSRLEKLGLRHPKPSPFIGNLTFF
RQGFWESQMELRKLYGPLCGYYLGRRMFIVISEPDMIKQVLVENFSNFTNRMPQSIMPNS
RHHVASRGMWPGGRRLACYLHPFRMAEGIAEV
>tr|B7Z705|B7Z705_HUMAN Chitinase domain-containing protein 1 OS=Homo sapiens OX=9606 PE=2 SV=1
MSVRGILRLGTQEYPSPLDPEVGPYCDTPTMRTLFNLLWLALACSPVHTTLSKSDAKKAA
SKTLLEKSQFSDKPVQDRGLVVTDLKAESVVLEHRSYCSAKARDRHFAGDVLGYVTPWNS
HGYDVTKVFGSKFTQISPVWLQLKRRGREMFEVTGLHDVDQGWMRAVRKHAKGLHIVPRL
LFEDWTYDDFRNVLDSEDEIEELSKTVVQVAKNQHFDGFVVEVWNQLLSQKRVGLIHMLT
HLAEALHQARLLALLVIPPAITPGTDQLGMFTHKEFEQLAPVLDGFSLMTYDYSTAHQPG
PNAPLSWVRACVQVLDPKSKWRSKILLGLNFYGMDYVTSKDAREPVVGARYIQTLKDHRP
RMVWDSQASEHFFEYEKSRSGRHVVFYPTLKSLQVRLELARELGVGVSIWELGQGLDYFY
DLP
>tr|B7Z972|B7Z972_HUMAN Protein-L-isoaspartate O-methyltransferase OS=Homo sapiens OX=9606 PE=2 SV=1
MAWKSGGASHSELIHNLRSFQATISAPHMHAYALELLFDQLHEGAKALDVGSGSGILTAC
FARMVGCTGKVIGIDHIKELVDDSVNNVRKDDPTLLSSGRVQLVVGDGRMGYAEEAPYDA
IHVGAAAPVVPQALIDQLKPGGRLILPVGPAGGNQMLEQYDKLQDGSIKMKPLMGVIYVP
LTDKEKQWSRWK
>tr|B9EH95|B9EH95_HUMAN Armadillo repeat gene deletes in velocardiofacial syndrome OS=Homo sapiens OX=9606 GN=ARVCF PE=2 SV=1
MEDCNVHSAASILASVKEQEARFERLTRALEQERRHVALQLERAQQPGMVSGGMGSGQPL

PMAWQQLVLQEQSPGSQASLATMPEAPDVLEETVTVEEDPGTPTSHVSIVTSEDGTTRRT
ETKVTKTVKTVTTRTVRQVPVGPDGLPLLDGGPPLGPFADGALDRHFLLRGGGPAATLSR
AYLSSGGGFPEGPEPRDSPSYGSLSRGLGMRPPRAGPLGLGPGDGCFTLPGHREAFPVGP
EPGPPGGRSLPERFQAEPYGLEDDTRSLAADDEGGPELEPDYGTATRRRPECGRGLHTRA
YEDTADDGGELADERPAFPMVTAPLAQPERGSMGSLDRLVRRSPSVDSARKEPRWDPEL
PEVLAMLRHPVDPVKANAAAYLQHLCFENEGVKRRVRQLRGLPLLVALLDHPRAEVRRRA
CGALRNLSYGRDTDNKAAIRDCGGVPALVRLLRAARDNEVRELVTGTLWNLSSYEPLKMV
IIDHGLQTLTHEVIVPHSGWEREPNEDSKPRDAEWTTVFKNTSGCLRNVSSDGAEARRRL
RECEGLVDALLHALQSAVGRKDTDNKSVENCVCIMRNLSYHVHKEVPGADRYQEAEPGPL
GSAVGSQRRRRDDASCFGGKKAKEEWFHQGKKDGEMDRNFDTLDLPKRTEAAKGFELLYQ
PEVVRLYLSLLTESRNFNTLEAAAGALQNLSAGNWMWATYIRATVRKERGLPVLVELLQS
ETDKVVRAVAIALRNLSLDRRNKDLIGSYAMAELVRNVRNAQAPPRPGACLEEDTVVAVL
NTIHEIVSDSLDNARSLLQARGVPALVALVASSQSVREAKAASHVLQTVWSYKELRGTLQ
KDGWTKARFQSAAATAKGPKGALSPGGFDDSTLPLVDKSLEGEKTGSRDVIPMDALGPDG
YSTVDQRERRPRGASSAGEASEKEPLKLDPSRKAPPPGPSRPAVRLVDAVGDAKPQPVDS
WV
>sp|O00159|MYO1C_HUMAN Unconventional myosin-Ic OS=Homo sapiens OX=9606 GN=MYO1C PE=1 SV=4
MALQVELVPTGEIIRVVHPHRPCKLALGSDGVRVTMESALTARDRVGVQDFVLLENFTSE
AAFIENLRRRFRENLIYTYIGPVLVSVNPYRDLQIYSRQHMERYRGVSFYEVPPHLFAVA
DTVYRALRTERRDQAVMISGESGAGKTEATKRLLQFYAETCPAPERGGAVRDRLLQSNPV
LEAFGNAKTLRNDNSSRFGKYMDVQFDFKGAPVGGHILSYLLEKSRVVHQNHGERNFHIF
YQLLEGGEEETLRRLGLERNPQSYLYLVKGQCAKVSSINDKSDWKVVRKALTVIDFTEDE
VEDLLSIVASVLHLGNIHFAANEESNAQVTTENQLKYLTRLLSVEGSTLREALTHRKIIA
KGEELLSPLNLEQAAYARDALAKAVYSRTFTWLVGKINRSLASKDVESPSWRSTTVLGLL
DIYGFEVFQHNSFEQFCINYCNEKLQQLFIELTLKSEQEEYEAEGIAWEPVQYFNNKIIC
DLVEEKFKGIISILDEECLRPGEATDLTFLEKLEDTVKHHPHFLTHKLADQRTRKSLGRG
EFRLLHYAGEVTYSVTGFLDKNNDLLFRNLKETMCSSKNPIMSQCFDRSELSDKKRPETV
ATQFKMSLLQLVEILQSKEPAYVRCIKPNDAKQPGRFDEVLIRHQVKYLGLLENLRVRRA
GFAYRRKYEAFLQRYKSLCPETWPTWAGRPQDGVAVLVRHLGYKPEEYKMGRTKIFIRFP
KTLFATEDALEVRRQSLATKIQAAWRGFHWRQKFLRVKRSAICIQSWWRGTLGRRKAAKR
KWAAQTIRRLIRGFVLRHAPRCPENAFFLDHVRTSFLLNLRRQLPQNVLDTSWPTPPPAL
REASELLRELCIKNMVWKYCRSISPEWKQQLQQKAVASEIFKGKKDNYPQSVPRLFISTR
LGTDEISPRVLQALGSEPIQYAVPVVKYDRKGYKPRSRQLLLTPNAVVIVEDAKVKQRID
YANLTGISVSSLSDSLFVLHVQRADNKQKGDVVLQSDHVIETLTKTALSANRVNSININQ
GSITFAGGPGRDGTIDFTPGSELLITKAKNGHLAVVAPRLNSR
>sp|O00192|ARVC_HUMAN Splicing regulator ARVCF OS=Homo sapiens OX=9606 GN=ARVCF PE=1 SV=1
MEDCNVHSAASILASVKEQEARFERLTRALEQERRHVALQLERAQQPGMVSGGMGSGQPL
PMAWQQLVLQEQSPGSQASLATMPEAPDVLEETVTVEEDPGTPTSHVSIVTSEDGTTRRT
ETKVTKTVKTVTTRTVRQVPVGPDGLPLLDGGPPLGPFADGALDRHFLLRGGGPVATLSR
AYLSSGGGFPEGPEPRDSPSYGSLSRGLGMRPPRAGPLGPGPGDGCFTLPGHREAFPVGP
EPGPPGGRSLPERFQAEPYGLEDDTRSLAADDEGGPELEPDYGTATRRRPECGRGLHTRA
YEDTADDGGELADERPAFPMVTAPLAQPERGSMGSLDRLVRRSPSVDSARKEPRWDPEL

PEVLAMLRHPVDPVKANAAAYLQHLCFENEGVKRRVRQLRGLPLLVALLDHPRAEVRRRA
CGALRNLSYGRDTDNKAAIRDCGGVPALVRLLRAARDNEVRELVTGTLWNLSSYEPLKMV
IIDHGLQTLTHEVIVPHSGWEREPNEDSKPRDAEWTTVFKNTSGCLRNVSSDGAEARRRL
RECEGLVDALLHALQSAVGRKDTDNKSVENCVCIMRNLSYHVHKEVPGADRYQEAEPGPL
GSAVGSQRRRRDDASCFGGKKAKEEWFHQGKKDGEMDRNFDTLDLPKRTEAAKGFELLYQ
PEVVRLYLSLLTESRNFNTLEAAAGALQNLSAGNWMWATYIRATVRKERGLPVLVELLQS
ETDKVVRAVAIALRNLSLDRRNKDLIGSYAMAELVRNVRNAQAPPRPGACLEEDTVVAVL
NTIHEIVSDSLDNARSLLQARGVPALVALVASSQSVREAKAASHVLQTVWSYKELRGTLQ
KDGWTKARFQSAAATAKGPKGALSPGGFDDSTLPLVDKSLEGEKTGSRDVIPMDALGPDG
YSTVDRRERRPRGASSAGEASEKEPLKLDPSRKAPPPGPSRPAVRLVDAVGDAKPQPVDS
WV
>sp|O00231|PSD11_HUMAN 26S proteasome non-ATPase regulatory subunit 11 OS=Homo sapiens OX=9606 GN=PSMD11 PE=1 SV=3
MAAAAVVEFQRAQSLLSTDREASIDILHSIVKRDIQENDEEAVQVKEQSILELGSLLAKT
GQAAELGGLLKYVRPFLNSISKAKAARLVRSLLDLFLDMEAATGQEVELCLECIEWAKSE
KRTFLRQALEARLVSLYFDTKRYQEALHLGSQLLRELKKMDDKALLVEVQLLESKTYHAL
SNLPKARAALTSARTTANAIYCPPKLQATLDMQSGIIHAAEEKDWKTAYSYFYEAFEGYD
SIDSPKAITSLKYMLLCKIMLNTPEDVQALVSGKLALRYAGRQTEALKCVAQASKNRSLA
DFEKALTDYRAELRDDPIISTHLAKLYDNLLEQNLIRVIEPFSRVQIEHISSLIKLSKAD
VERKLSQMILDKKFHGILDQGEGVLIIFDEPPVDKTYEAALETIQNMSKVVDSLYNKAKK
LT
>sp|O00303|EIF3F_HUMAN Eukaryotic translation initiation factor 3 subunit F OS=Homo sapiens OX=9606 GN=EIF3F PE=1 SV=1
MATPAVPVSAPPATPTPVPAAAPASVPAPTPAPAAAPVPAAAPASSSDPAAAAAATAAPG
QTPASAQAPAQTPAPALPGPALPGPFPGGRVVRLHPVILASIVDSYERRNEGAARVIGTL
LGTVDKHSVEVTNCFSVPHNESEDEVAVDMEFAKNMYELHKKVSPNELILGWYATGHDIT
EHSVLIHEYYSREAPNPIHLTVDTSLQNGRMSIKAYVSTLMGVPGRTMGVMFTPLTVKYA
YYDTERIGVDLIMKTCFSPNRVIGLSSDLQQVGGASARIQDALSTVLQYAEDVLSGKVSA
DNTVGRFLMSLVNQVPKIVPDDFETMLNSNINDLLMVTYLANLTQSQIALNEKLVNL
>sp|O00391|QSOX1_HUMAN Sulfhydryl oxidase 1 OS=Homo sapiens OX=9606 GN=QSOX1 PE=1 SV=3
MRRCNSGSGPPPSLLLLLLWLLAVPGANAAPRSALYSPSDPLTLLQADTVRGAVLGSRSA
WAVEFFASWCGHCIAFAPTWKALAEDVKAWRPALYLAALDCAEETNSAVCRDFNIPGFPT
VRFFKAFTKNGSGAVFPVAGADVQTLRERLIDALESHHDTWPPACPPLEPAKLEEIDGFF
ARNNEEYLALIFEKGGSYLGREVALDLSQHKGVAVRRVLNTEANVVRKFGVTDFPSCYLL
FRNGSVSRVPVLMESRSFYTAYLQRLSGLTREAAQTTVAPTTANKIAPTVWKLADRSKIY
MADLESALHYILRIEVGRFPVLEGQRLVALKKFVAVLAKYFPGRPLVQNFLHSVNEWLKR
QKRNKIPYSFFKTALDDRKEGAVLAKKVNWIGCQGSEPHFRGFPCSLWVLFHFLTVQAAR
QNVDHSQEAAKAKEVLPAIRGYVHYFFGCRDCASHFEQMAAASMHRVGSPNAAVLWLWSS
HNRVNARLAGAPSEDPQFPKVQWPPRELCSACHNERLDVPVWDVEATLNFLKAHFSPSNI
ILDFPAAGSAARRDVQNVAAAPELAMGALELESRNSTLDPGKPEMMKSPTNTTPHVPAEG
PEASRPPKLHPGLRAAPGQEPPEHMAELQRNEQEQPLGQWHLSKRDTGAALLAESRAEKN
RLWGPLEVRRVGRSSKQLVDIPEGQLEARAGRGRGQWLQVLGGGFSYLDISLCVGLYSLS
FMGLLAMYTYFQAKIRALKGHAGHPAA

>sp|O00410|IPO5_HUMAN Importin-5 OS=Homo sapiens OX=9606 GN=IPO5 PE=1 SV=4
MAAAAAEQQQFYLLLGNLLSPDNVVRKQAEETYENIPGQSKITFLLQAIRNTTAAEEARQ
MAAVLLRRLLSSAFDEVYPALPSDVQTAIKSELLMIIQMETQSSMRKKVCDIAAELARNL
IDEDGNNQWPEGLKFLFDSVSSQNVGLREAALHIFWNFPGIFGNQQQHYLDVIKRMLVQC
MQDQEHPSIRTLSARATAAFILANEHNVALFKHFADLLPGFLQAVNDSCYQNDDSVLKSL
VEIADTVPKYLRPHLEATLQLSLKLCGDTSLNNMQRQLALEVIVTLSETAAAMLRKHTNI
VAQTIPQMLAMMVDLEEDEDWANADELEDDDFDSNAVAGESALDRMACGLGGKLVLPMIK
EHIMQMLQNPDWKYRHAGLMALSAIGEGCHQQMEGILNEIVNFVLLFLQDPHPRVRYAAC
NAVGQMATDFAPGFQKKFHEKVIAALLQTMEDQGNQRVQAHAAAALINFTEDCPKSLLIP
YLDNLVKHLHSIMVLKLQELIQKGTKLVLEQVVTSIASVADTAEEKFVPYYDLFMPSLKH
IVENAVQKELRLLRGKTIECISLIGLAVGKEKFMQDASDVMQLLLKTQTDFNDMEDDDPQ
ISYMISAWARMCKILGKEFQQYLPVVMGPLMKTASIKPEVALLDTQDMENMSDDDGWEFV
NLGDQQSFGIKTAGLEEKSTACQMLVCYAKELKEGFVEYTEQVVKLMVPLLKFYFHDGVR
VAAAESMPLLLECARVRGPEYLTQMWHFMCDALIKAIGTEPDSDVLSEIMHSFAKCIEVM
GDGCLNNEHFEELGGILKAKLEEHFKNQELRQVKRQDEDYDEQVEESLQDEDDNDVYILT
KVSDILHSIFSSYKEKVLPWFEQLLPLIVNLICPHRPWPDRQWGLCIFDDVIEHCSPASF
KYAEYFLRPMLQYVCDNSPEVRQAAAYGLGVMAQYGGDNYRPFCTEALPLLVRVIQSADS
KTKENVNATENCISAVGKIMKFKPDCVNVEEVLPHWLSWLPLHEDKEEAVQTFNYLCDLI
ESNHPIVLGPNNTNLPKIFSIIAEGEMHEAIKHEDPCAKRLANVVRQVQTSGGLWTECIA
QLSPEQQAAIQELLNSA
>sp|O00442|RTCA_HUMAN RNA 3'-terminal phosphate cyclase OS=Homo sapiens OX=9606
GN=RTCA PE=1 SV=1
MAGPRVEVDGSIMEGGGQILRVSTALSCLLGLPLRVQKIRAGRSTPGLRPQHLSGLEMIR
DLCDGQLEGAEIGSTEITFTPEKIKGGIHTADTKTAGSVCLLMQVSMPCVLFAASPSELH
LKGGTNAEMAPQIDYTVMVFKPIVEKFGFIFNCDIKTRGYYPKGGGEVIVRMSPVKQLNP
INLTERGCVTKIYGRAFVAGVLPFKVAKDMAAAAVRCIRKEIRDLYVNIQPVQEPKDQAF
GNGNGIIIIAETSTGCLFAGSSLGKRGVNADKVGIEAAEMLLANLRHGGTVDEYLQDQLI
VFMALANGVSRIKTGPVTLHTQTAIHFAEQIAKAKFIVKKSEDEEDAAKDTYIIECQGIG
MTNPNL
>sp|O00519|FAAH1_HUMAN Fatty-acid amide hydrolase 1 OS=Homo sapiens OX=9606
GN=FAAH PE=1 SV=2
MVQYELWAALPGASGVALACCFVAAAVALRWSGRRTARGAVVRARQRQRAGLENMDRAAQ
RFRLQNPDLDSEALLALPLPQLVQKLHSRELAPEAVLFTYVGKAWEVNKGTNCVTSYLAD
CETQLSQAPRQGLLYGVPVSLKECFTYKGQDSTLGLSLNEGVPAECDSVVVHVLKLQGAV
PFVHTNVPQSMFSYDCSNPLFGQTVNPWKSSKSPGGSSGGEGALIGSGGSPLGLGTDIGG
SIRFPSSFCGICGLKPTGNRLSKSGLKGCVYGQEAVRLSVGPMARDVESLALCLRALLCE
DMFRLDPTVPPLPFREEVYTSSQPLRVGYYETDNYTMPSPAMRRAVLETKQSLEAAGHTL
VPFLPSNIPHALETLSTGGLFSDGGHTFLQNFKGDFVDPCLGDLVSILKLPQWLKGLLAF
LVKPLLPRLSAFLSNMKSRSAGKLWELQHEIEVYRKTVIAQWRALDLDVVLTPMLAPALD
LNAPGRATGAVSYTMLYNCLDFPAGVVPVTTVTAEDEAQMEHYRGYFGDIWDKMLQKGMK
KSVGLPVAVQCVALPWQEELCLRFMREVERLMTPEKQSS
>sp|O00534|VMA5A_HUMAN von Willebrand factor A domain-containing protein 5A
OS=Homo sapiens OX=9606 GN=VWA5A PE=1 SV=2
MVHFCGLLTLHREPVPLKSISVSVNIYEFVAGVSATLNYENEEKVPLEAFFVFPMDEDSA

VYSFEALVDGKKIVAELQDKMKARTNYEKAISQGHQAFLLEGDSSSRDVFSCNVGNLQPG
SKAAVTLKYVQELPLEADGALRFVLPAVLNPRYQFSGSSKDSCLNVKTPIVPVEDLPYTL
SMVATIDSQHGIEKVQSNCPLSPTEYLGEDKTSAQVSLAAGHKFDRDVELLIYYNEVHTP
SVVLEMGMPNMKPGHLMGDPSAMVSFYPNIPEDQPSNTCGEFIFLMDRSGSMQSPMSSQD
TSQLRIQAAKETLILLLKSLPIGCYFNIYGFGSSYEACFPESVKYTQQTMEEALGRVKLM
QADLGGTEILAPLQNIYRGPSIPGHPLQLFVFTDGEVTDTFSVIKEVRINRQKHRCFSFG
IGEGTSTSLIKGIARASGGTSEFITGKDRMQSKALRTLKRSLQPVVEDVSLSWHLPPGLS
AKMLSPEQTVIFRGQRLISYAQLTGRMPAAETTGEVCLKYTLQGKTFEDKVTFPLQPKPD
VNLTIHRLAAKSLLQTKDMGLRETPASDKKDALNLSLESGVISSFTAFIAINKELNKPVQ
GPLAHRDVPRPILLGASAPLKIKCQSGFRKALHSDRPPSASQPRGELMCYKAKTFQMDDY
SLCGLISHKDQHSPGFGENHLVQLIYHQNANGSWDLNEDLAKILGMSLEEIMAAQPAELV
DSSGWATILAVIWLHSNGKDLKCEWELLERKAVAWMRAHAGSTMPSVVKAAITFLKSSVD
PAIFAF
>sp|O00743|PPP6_HUMAN Serine/threonine-protein phosphatase 6 catalytic subunit
OS=Homo sapiens OX=9606 GN=PPP6C PE=1 SV=1
MAPLDLDKYVEIARLCKYLPENDLKRLCDYVCDLLLEESNVQPVSTPVTVCGDIHGQFYD
LCELFRTGGQVPDTNYIFMGDFVDRGYYSLETFTYLLALKAKWPDRITLLRGNHESRQIT
QVYGFYDECQTKYGNANAWRYCTKVFDMLTVAALIDEQILCVHGGLSPDIKTLDQIRTIE
RNQEIPHKGAFCDLVWSDPEDVDTWAISPRGAGWLFGAKVTNEFVHINNLKLICRAHQLV
HEGYKFMFDEKLVTVWSAPNYCYRCGNIASIMVFKDVNTREPKLFRAVPDSERVIPPRTT
TPYFL
>sp|O14556|G3PT_HUMAN Glyceraldehyde-3-phosphate dehydrogenase, testis-specific
OS=Homo sapiens OX=9606 GN=GAPDHS PE=1 SV=2
MSKRDIVLTNVTVVQLLRQPCPVTRAPPPPEPKAEVEPQPQPEPTPVREEIKPPPPPLPP
HPATPPPKMVSVARELTVGINGFGRIGRLVLRACMEKGVKVVAVNDPFIDPEYMVYMFKY
DSTHGRYKGSVEFRNGQLVVDNHEISVYQCKEPKQIPWRAVGSPYVVESTGVYLSIQAAS
DHISAGAQRVVISAPSPDAPMFVMGVNENDYNPGSMNIVSNASCTTNCLAPLAKVIHERF
GIVEGLMTTVHSYTATQKTVDGPSRKAWRDGRGAHQNIIPASTGAAKAVTKVIPELKGKL
TGMAFRVPTPDVSVVDLTCRLAQPAPYSAIKEAVKAAAKGPMAGILAYTEDEVVSTDFLG
DTHSSIFDAKAGIALNDNFVKLISWYDNEYGYSHRVVDLLRYMFSRDK
>sp|O14672|ADA10_HUMAN Disintegrin and metalloproteinase domain-containing protein 10
OS=Homo sapiens OX=9606 GN=ADAM10 PE=1 SV=1
MVLLRVLILLLSWAAGMGGQYGNPLNKYIRHYEGLSYNVDSLHQKHQRAKRAVSHEDQFL
RLDFHAHGRHFNLRMKRDTSLFSDEFKVETSNKVLDYDTSHIYTGHIYGEEGSFSHGSVI
DGRFEGFIQTRGGTFYVEPAERYIKDRTLPFHSVIYHEDDINYPHKYGPQGGCADHSVFE
RMRKYQMTGVEEVTQIPQEEHAANGPELLRKKRTTSAEKNTCQLYIQTDHLFFKYYGTRE
AVIAQISSHVKAIDTIYQTTDFSGIRNISFMVKRIRINTTADEKDPTNPFRFPNIGVEKF
LELNSEQNHDDYCLAYVFTDRDFDDGVLGLAWVGAPSGSSGGICEKSKLYSDGKKKSLNT
GIITVQNYGSHVPPKVSHITFAHEVGHNFGSPHDSGTECTPGESKNLGQKENGNYIMYAR
ATSGDKLNNNKFSLCSIRNISQVLEKKRNNCFVESGQPICGNGMVEQGEECDCGYSDQCK
DECCFDANQPEGRKCKLKPGKQCSPSQGPCCTAQCAFKSKSEKCRDDSDCAREGICNGFT
ALCPASDPKPNFTDCNRHTQVCINGQCAGSICEKYGLEECTCASSDGKDDKELCHVCCMK
KMDPSTCASTGSVQWSRHFSGRTITLQPGSPCNDFRGYCDVFMRCRLVDADGPLARLKKA
IFSPELYENIAEWIVAHWWAVLLMGIALIMLMAGFIKICSVHTPSSNPKLPPPKPLPGTL

KRRRPPQPIQQPQRQRPRESYQMGHMRR
>sp|O14727|APAF_HUMAN Apoptotic protease-activating factor 1 OS=Homo sapiens OX=9606 GN=APAF1 PE=1 SV=2
MDAKARNCLLQHREALEKDIKTSYIMDHMISDGFLTISEEEKVRNEPTQQQRAAMLIKMI
LKKDNDSYVSFYNALLHEGYKDLAALLHDGIPVVSSSSGKDSVSGITSYVRTVLCEGGVP
QRPVVFVTRKKLVNAIQQKLSKLKGEPGWVTIHGMAGCGKSVLAAEAVRDHSLLEGCFPG
GVHWVSVGKQDKSGLLMKLQNLCTRLDQDESFSQRLPLNIEEAKDRLRILMLRKHPRSLL
ILDDVWDSWVLKAFDSQCQILLTTRDKSVTDSVMGPKYVVPVESSLGKEKGLEILSLFVN
MKKADLPEQAHSIIKECKGSPLVVSLIGALLRDFPNRWEYYLKQLQNKQFKRIRKSSSYD
YEALDEAMSISVEMLREDIKDYYTDLSILQKDVKVPTKVLCILWDMETEEVEDILQEFVN
KSLLFCDRNGKSFRYYLHDLQVDFLTEKNCSQLQDLHKKIITQFQRYHQPHTLSPDQEDC
MYWYNFLAYHMASAKMHKELCALMFSLDWIKAKTELVGPAHLIHEFVEYRHILDEKDCAV
SENFQEFLSLNGHLLGRQPFPNIVQLGLCEPETSEVYQQAKLQAKQEVDNGMLYLEWINK
KNITNLSRLVVRPHTDAVYHACFSEDGQRIASCGADKTLQVFKAETGEKLLEIKAHEDEV
LCCAFSTDDRFIATCSVDKKVKIWNSMTGELVHTYDEHSEQVNCCHFTNSSHHLLLATGS
SDCFLKLWDLNQKECRNTMFGHTNSVNHCRFSPDDKLLASCSADGTLKLWDATSANERKS
INVKQFFLNLEDPQEDMEVIVKCCSWSADGARIMVAAKNKIFLFDIHTSGLLGEIHTGHH
STIQYCDFSPQNHLAVVALSQYCVELWNTDSRSKVADCRGHLSWVHGVMFSPDGSSFLTS
SDDQTIRLWETKKVCKNSAVMLKQEVDVVFQENEVMVLAVDHIRRLQLINGRTGQIDYLT
EAQVSCCCLSPHLQYIAFGDENGAIEILELVNNRIFQSRFQHKKTVWHIQFTADEKTLIS
SSDDAEIQVWNWQLDKCIFLRGHQETVKDFRLLKNSRLLSWSFDGTVKVWNIITGNKEKD
FVCHQGTVLSCDISHDATKFSSTSADKTAKIWSFDLLLPLHELRGHNGCVRCSAFSVDST
LLATGDDNGEIRIWNVSNGELLHLCAPLSEEGAATHGGWVTDLCFSPDGKMLISAGGYIK
WWNVVTGESSQTFYTNGTNLKKIHVSPDFKTYVTVDNLGILYILQTLE
>sp|O14744|ANM5_HUMAN Protein arginine N-methyltransferase 5 OS=Homo sapiens OX=9606 GN=PRMT5 PE=1 SV=4
MAAMAVGGAGGSRVSSGRDLNCVPEIADTLGAVAKQGFDFLCMPVFHPRFKREFIQEPAK
NRPGPQTRSDLLLSGRDWNTLIVGKLSPWIRPDSKVEKIRRNSEAAMLQELNFGAYLGLP
AFLLPLNQEDNTNLARVLTNHIHTGHHSSMFWMRVPLVAPEDLRDDIIENAPTTHTEEYS
GEEKTWMWWHNFRTLCDYSKRIAVALEIGADLPSNHVIDRWLGEPIKAAILPTSIFLTNK
KGFPVLSKMHQRLIFRLLKLEVQFIITGTNHHSEKEFCSYLQYLEYLSQNRPPPNAYELF
AKGYEDYLQSPLQPLMDNLESQTYEVFEKDPIKYSQYQQAIYKCLLDRVPEEEKDTNVQV
LMVLGAGRGPLVNASLRAAKQADRRIKLYAVEKNPNAVVTLENWQFEEWGSQVTVVSSDM
REWVAPEKADIIVSELLGSFADNELSPECLDGAQHFLKDDGVSIPGEYTSFLAPISSSKL
YNEVRACREKDRDPEAQFEMPYVVRLHNFHQLSAPQPCFTFSHPNRDPMIDNNRYCTLEF
PVEVNTVLHGFAGYFETVLYQDITLSIRPETHSPGMFSWFPILFPIKQPITVREGQTICV
RFWRCSNSKKVWYEWAVTAPVCSAIHNPTGRSYTIGL
>sp|O14787|TNPO2_HUMAN Transportin-2 OS=Homo sapiens OX=9606 GN=TNPO2 PE=1 SV=3
MDWQPDEQGLQQVLQLLKDSQSPNTATQRIVQDKLKQLNQFPDFNNYLIFVLTRLKSEDE
PTRSLSGLILKNNVKAHYQSFPPPVADFIKQECLNNIGDASSLIRATIGILITTIASKGE
LQMWPELLPQLCNLLNSEDYNTCEGAFGALQKICEDSSELLDSDALNRPLNIMIPKFLQF
FKHCSPKIRSHAIACVNQFIMDRAQALMDNIDTFIEHLFALAVDDDPEVRKNVCRALVML
LEVRIDRLIPHMHSIIQYMLQRTQDHDENVALEACEFWLTLAEQPICKEVLASHLVQLIP
ILVNGMKYSEIDIILLKGDVEEDEAVPDSEQDIKPRFHKSRTVTLPHEAERPDGSEDAED

DDDDDDALSDWNLRKCSAAALDVLANVFREELLPHLLPLLKGLLFHPEWVVKESGILVLGA
IAEGCMQGMVPYLPELIPHLIQCLSDKKALVRSIACWTLSRYAHWVVSQPPDMHLKPLMT
ELLKRILDGNKRVQEAACSAFATLEEEACTELVPYLSYILDTLVFAFGKYQHKNLLILYD
AIGTLADSVGHHLNQPEYIQKLMPPLIQKWNELKDEDKDLFPLLECLSSVATALQSGFLP
YCEPVYQRCVTLVQKTLAQAMMYTQHPEQYEAPDKDFMIVALDLLSGLAEGLGGHVEQLV
ARSNIMTLLFQCMQDSMPEVRQSSFALLGDLTKACFIHVKPCIAEFMPILGTNLNPEFIS
VCNNATWAIGEICMQMGAEMQPYVQMVLNNLVEIINRPNTPKTLLENTGRLTSPSAIPAI
TIGRLGYVCPQEVAPMLQQFIRPWCTSLRNIRDNEEKDSAFRGICMMIGVNPGGVVQDFI
FFCDAVASWVSPKDDLRDMFYKILHGFKDQVGEDNWQQFSEQFPPLLKERLAAFYGV
>sp|O14925|TIM23_HUMAN Mitochondrial import inner membrane translocase subunit Tim23 OS=Homo sapiens OX=9606 GN=TIMM23 PE=1 SV=1
MEGGGGSGNKTTGGLAGFFGAGGAGYSHADLAGVPLTGMNPLSPYLNVDPRYLVQDTDEF
ILPTGANKTRGRFELAFFTIGGCCMTGAAFGAMNGLRLGLKETQNMAWSKPRNVQILNMV
TRQGALWANTLGSLALLYSAFGVIIEKTRGAEDDLNTVAAGTMTGMLYKCTGGLRGIARG
GLTGLTLTSLYALYNNWEHMKGSLLQQSL
>sp|O14960|LECT2_HUMAN Leukocyte cell-derived chemotaxin-2 OS=Homo sapiens OX=9606 GN=LECT2 PE=1 SV=2
MFSTKALLLAGLISTALAGPWANICAGKSSNEIRTCDRHGCGQYSAQRSQRPHQGVDILC
SAGSTVYAPFTGMIVGQEKPYQNKNAINNGVRISGRGFCVKMFYIKPIKYKGPIKKGEKL
GTLLPLQKVYPGIQSHVHIENCDSSDPTAYL
>sp|O14980|XPO1_HUMAN Exportin-1 OS=Homo sapiens OX=9606 GN=XPO1 PE=1 SV=1
MPAIMTMLADHAARQLLDFSQKLDINLLDNVVNCLYHGEGAQQRMAQEVLTHLKEHPDAW
TRVDTILEFSQNMNTKYYGLQILENVIKTRWKILPRNQCEGIKKYVVGLIIKTSSDPTCV
EKEKVYIGKLNMILVQILKQEWPKHWPTFISDIVGASRTSESLCQNNMVILKLLSEEVFD
FSSGQITQVKSKHLKDSMCNEFSQIFQLCQFVMENSQNAPLVHATLETLLRFLNWIPLGY
IFETKLISTLIYKFLNVPMFRNVSLKCLTEIAGVSVSQYEEQFVTLFTLTMMQLKQMLPL
NTNIRLAYSNGKDDEQNFIQNLSLFLCTFLKEHDQLIEKRLNLRETLMEALHYMLLVSEV
EETEIFKICLEYWNHLAAELYRESPFSTSASPLLSGSQHFDVPPRRQLYLPMLFKVRLLM
VSRMAKPEEVLVVENDQGEVVREFMKDTDSINLYKNMRETLVYLTHLDYVDTERIMTEKL
HNQVNGTEWSWKNLNTLCWAIGSISGAMHEEDEKRFLVTVIKDLLGLCEQKRGKDNKAII
ASNIMYIVGQYPRFLRAHWKFLKTVVNKLFEFMHETHDGVQDMACDTFIKIAQKCRRHFV
QVQVGEVMPFIDEILNNINTIICDLQPQQVHTFYEAVGYMIGAQTDQTVQEHLIEKYMLL
PNQVWDSIIQQATKNVDILKDPETVKQLGSILKTNVRACKAVGHPFVIQLGRIYLDMLNV
YKCLSENISAAIQANGEMVTKQPLIRSMRTVKRETLKLISGWVSRSNDPQMVAENFVPPL
LDAVLIDYQRNVPAAREPEVLSTMAIIVNKLGGHITAEIPQIFDAVFECTLNMINKDFEE
YPEHRTNFFLLLQAVNSHCFPAFLAIPPTQFKLVLDSIIWAFKHTMRNVADTGLQILFTL
LQNVAQEEAAAQSFYQTYFCDILQHIFSVVTDTSHTAGLTMHASILAYMFNLVEEGKIST
SLNPGNPVNNQIFLQEYVANLLKSAFPHLQDAQVKLFVTGLFSLNQDIPAFKEHLRDFLV
QIKEFAGEDTSDLFLEEREIALRQADEEKHKRQMSVPGIFNPHEIPEEMCD
>sp|O15020|SPTN2_HUMAN Spectrin beta chain, non-erythrocytic 2 OS=Homo sapiens OX=9606 GN=SPTBN2 PE=1 SV=3
MSSTLSPTDFDSLEIQGQYSDINNRWDLPDSDWDNDSSSARLFERSRIKALADEREAVQK
KTFTKWVNSHLARVTCRVGDLYSDLRDGRNLLRLLEVLSGEILPKPTKGRMRIHCLENVD
KALQFLKEQKVHLENMGSHDIVDGNHRLTLGLVWTIILRFQIQDISVETEDNKEKKSAKD

ALLLWCQMKTAGYPNVNVHNFTTSWRDGLAFNAIVHKHRPDLLDFESLKKCNAHYNLQNA
FNLAEKELGLTKLLDPEDVNVDQPDEKSIITYVATYYHYFSKMKALAVEGKRIGKVLDHA
MEAERLVEKYESLASELLQWIEQTIVTLNDRQLANSLSGVQNQLQSFNSYRTVEKPPKFT
EKGNLEVLLFTIQSKLRANNQKVYTPREGRLISDINKAWERLEKAEHERELALRTELIRQ
EKLEQLAARFDRKAAMRETWLSENQRLVSQDNFGLELAAVEAAVRKHEAIETDIVAYSGR
VQAVDAVAAELAAERYHDIKRIAARQHNVARLWDFLRQMVAARRERLLLNLELQKVFQDL
LYLMDWMEEMKGRLQSQDLGRHLAGVEDLLQLHELVEADIAVQAERVRAVSASALRFCNP
GKEYRPCDPQLVSERVAKLEQSYEALCELAAARRARLEESRRLWRFLWEVGEAEAWVREQ
QHLLASADTGRDLTGALRLLNKHTALRGEMSGRLGPLKLTLEQGQQLVAEGHPGASQASA
RAAELQAQWERLEALAEERAQRLAQAASLYQFQADANDMEAWLVDALRLVSSPELGHDEF
STQALARQHRALEEEIRSHRPTLDALREQAAALPPTLSRTPEVQSRVPTLERHYEELQAR
AGERARALEAALALYTMLSEAGACGLWVEEKEQWLNGLALPERLEDLEVVQQRFETLEPE
MNTLAAQITAVNDIAEQLLKANPPGKDRIVNTQEQLNHRWQQFRRLADGKKAALTSALSI
QNYHLECTETQAWMREKTKVIESTQGLGNDLAGVLALQRKLAGTERDLEAIAARVGELTR
EANALAAGHPAQAVAINARLREVQTGWEDLRATMRRREESLGEARRLQDFLRSLDDFQAW
LGRTQTAVASEEGPATLPEAEALLAQHAALRGEVERAQSEYSRLRALGEEVTRDQADPQC
LFLRQRLEALGTGWEELGRMWESRQGRLAQAHGFQGFLRDARQAEGVLSSQEYVLSHTEM
PGTLQAADAAIKKLEDFMSTMDANGERIHGLLEAGRQLVSEGNIHADKIREKADSIERRH
KKNQDAAQQFLGRLRDNREQQHFLQDCHELKLWIDEKMLTAQDVSYDEARNLHTKWQKHQ
AFMAELAANKDWLDKVDKEGRELTLEKPELKALVSEKLRDLHRRWDELETTTQAKARSLF
DANRAELFAQSCCALESWLESLQAQLHSDDYGKDLTSVNILLKKQQMLEWEMAVREKEVE
AIQAQAKALAQEDQGAGEVERTSRAVEEKFRALCQPMRERCRRLQASREQHQFHRDVEDE
ILWVTERLPMASSMEHGKDLPSVQLLMKKNQTLQKEIQGHEPRIADLRERQRALGAAAAG
PELAELQEMWKRLGHELELRGKRLEDALRAQQFYRDAAEAEAWMGEQELHMMGQEKAKDE
LSAQAEVKKHQVLEQALADYAQTIHQLAASSQDMIDHEHPESTRISIRQAQVDKLYAGLK
ELAGERRERLQEHLRLCQLRRELDDLEQWIQEREVVAASHELGQDYEHVTMLRDKFREFS
RDTSTIGQERVDSANALANGLIAGGHAARATVAEWKDSLNEAWADLLELLDTRGQVLAAA
YELQRFLHGARQALARVQHKQQQLPDGTGRDLNAAEALQRRHCAYEHDIQALSPQVQQVQ
DDGHRLQKAYAGDKAEEIGRHMQAVAEAWAQLQGSSAARRQLLLDTTDKFRFFKAVRELM
LWMDEVNLQMDAQERPRDVSSADLVIKNQQGIKAEIEARADRFSSCIDMGKELLARSHYA
AEEISEKLSQLQARRQETAEKWQEKMDWLQLVLEVLVFGRDAGMAEAWLCSQEPLVRSAE
LGCTVDEVESLIKRHEAFQKSAVAWEERFCALEKLTALEEREKERKRKREEEERRKQPPA
PEPTASVPPGDLVGGQTASDTTWDGTQPRPPPSTQAPSVNGVCTDGEPSQPLLGQQRLEH
SSFPEGPGPGSGDEANGPRGERQTRTRGPAPSAMPQSRSTESAHAATLPPRGPEPSAQEQ
MEGMLCRKQEMEAFGKKAANRSWQNVYCVLRRGSLGFYKDAKAASAGVPYHGEVPVSLAR
AQGSVAFDYRKRKHVFKLGLQDGKEYLFQAKDEAEMSSWLRVVNAAIATASSASGEPEEP
VVPSTTRGMTRAMTMPPVSPVGAEGPVVLRSKDGREREREKRFSFFKKNK
>sp|O15031|PLXB2_HUMAN Plexin-B2 OS=Homo sapiens OX=9606 GN=PLXNB2 PE=1 SV=3
MALQLWALTLLGLLGAGASLRPRKLDFFRSEKELNHLAVDEASGVVYLGAVNALYQLDAK
LQLEQQVATGPALDNKKCTPPIEASQCHEAEMTDNVNQLLLLDPPRKRLVECGSLFKGIC
ALRALSNISLRLFYEDGSGEKSFVASNDEGVATVGLVSSTGPGGDRVLFVGKGNGPHDNG
IIVSTRLLDRTDSREAFEAYTDHATYKAGYLSTNTQQFVAAFEDGPYVFFVFNQQDKHPA
RNRTLLARMCREDPNYYSYLEMDLQCRDPDIHAAAFGTCLAASVAAPGSGRVLYAVFSRD
SRSSGGPGAGLCLFPLDKVHAKMEANRNACYTGTREARDIFYKPFHGDIQCGGHAPGSSK

SFPCGSEHLPYPLGSRDGLRGTAVLQRGGLNLTAVTVAAENNHTVAFLGTSDGRILKVYL
TPDGTSSEYDSILVEINKRVKRDLVLSGDLGSLYAMTQDKVFRLPVQECLSYPTCTQCRD
SQDPYCGWCVVEGRCTRKAECPRAEEASHWLWSRSKSCVAVTSAQPQNMSRRAQGEVQLT
VSPLPALSEEDELLCLFGESPPHPARVEGEAVICNSPSSIPVTPPGQDHVAVTIQLLLRR
GNIFLTSYQYPFYDCRQAMSLEENLPCISCVSNRWTCQWDLRYHECREASPNPEDGIVRA
HMEDSCPQFLGPSPLVIPMNHETDVNFQGKNLDTVKGSSLHVGSDLLKFMEPVTMQESGT
FAFRTPKLSHDANETLPLHLYVKSYGKNIDSKLHVTLYNCSFGRSDCSLCRAANPDYRCA
WCGGQSRCVYEALCNTTSECPPPVITRIQPETGPLGGGIRITILGSNLGVQAGDIQRISV
AGRNCSFQPERYSVSTRIVCVIEAAETPFTGGVEVDVFGKLGRSPPNVQFTFQQPKPLSV
EPQQGPQAGGTTLTIHGTHLDTGSQEDVRVTLNGVPCKVTKFGAQLQCVTGPQATRGQML
LEVSYGGSPVPNPGIFFTYRENPVLRAFEPLRSFASGGRSINVTGQGFSLIQRFAMVVIA
EPLQSWQPPREAESLQPMTVVGTDYVFHNDTKVVFLSPAVPEEPEAYNLTVLIEMDGHRA
LLRTEAGAFEYVPDPTFENFTGGVKKQVNKLIHARGTNLNKAMTLQEAEAFVGAERCTMK
TLTETDLYCEPPEVQPPPKRRQKRDTTHNLPEFIVKFGSREWVLGRVEYDTRVSDVPLSL
ILPLVIVPMVVVIAVSVYCYWRKSQQAEREYEKIKSQLEGLEESVRDRCKKEFTDLMIEM
EDQTNDVHEAGIPVLDYKTYTDRVFFLPSKDGDKDVMITGKLDIPEPRRPVVEQALYQFS
NLLNSKSFLINFIHTLENQREFSARAKVYFASLLTVALHGKLEYYTDIMHTLFLELLEQY
VVAKNPKLMLRRSETVVERMLSNWMSICLYQYLKDSAGEPLYKLFKAIKHQVEKGPVDAV
QKKAKYTLNDTGLLGDDVEYAPLTVSVIVQDEGVDAIPVKVLNCDTISQVKEKIIDQVYR
GQPCSCWPRPDSVVLEWRPGSTAQILSDLDLTSQREGRWKRVNTLMHYNVRDGATLILSK
VGVSQQPEDSQQDLPGERHALLEEENRVWHLVRPTDEVDEGKSKRGSVKEKERTKAITEI
YLTRLLSVKGTLQQFVDNFFQSVLAPGHAVPPAVKYFFDFLDEQAEKHNIQDEDTIHIWK
TNSLPLRFWVNILKNPHFIFDVHVHEVVDASLSVIAQTFMDACTRTEHKLSRDSPSNKLL
YAKEISTYKKMVEDYYKGIRQMVQVSDQDMNTHLAEISRAHTDSLNTLVALHQLYQYTQK
YYDEIINALEEDPAAQKMQLAFRLQQIAAALENKVTDL
>sp|O15144|ARPC2_HUMAN Actin-related protein 2/3 complex subunit 2 OS=Homo sapiens OX=9606 GN=ARPC2 PE=1 SV=1
MILLEVNNRIIEETLALKFENAAAGNKPEAVEVTFADFDGVLYHISNPNGDKTKVMVSIS
LKFYKELQAHGADELLKRVYGSFLVNPESGYNVSLLYDLENLPASKDSIVHQAGMLKRNC
FASVFEKYFQFQEEGKEGENRAVIHYRDDETMYVESKKDRVTVVFSTVFKDDDDVVIGKV
FMQEFKEGRRASHTAPQVLFSHREPPLELKDTDAAVGDNIGYITFVLFPRHTNASARDNT
INLIHTFRDYLHYHIKCSKAYIHTRMRAKTSDFLKVLNRARPDAEKKEMKTITGKTFSSR
>sp|O15230|LAMA5_HUMAN Laminin subunit alpha-5 OS=Homo sapiens OX=9606 GN=LAMA5 PE=1 SV=8
MAKRLCAGSALCVRGPRGPAPLLLVGLALLGAARAREEAGGGFSLHPPYFNLAEGARIAA
SATCGEEAPARGSPRPTEDLYCKLVGGPVAGGDPNQTIRGQYCDICTAANSNKAHPASNA
IDGTERWWQSPPLSRGLEYNEVNVTLDLGQVFHVAYVLIKFANSPRPDLWVLERSMDFGR
TYQPWQFFASSKRDCLERFGPQTLERITRDDAAICTTEYSRIVPLENGEIVVSLVNGRPG
AMNFSYSPLLREFTKATNVRLRFLRTNTLLGHLMGKALRDPTVTRRYYYSIKDISIGGRC
VCHGHADACDAKDPTDPFRLQCTCQHNTCGGTCDRCCPGFNQQPWKPATANSANECQSCN
CYGHATDCYYDPEVDRRRASQSLDGTYQGGGVCIDCQHHTTGVNCERCLPGFYRSPNHPL
DSPHVCRRCNCESDFTDGTCEDLTGRCYCRPNFSGERCDVCAEGFTGFPSCYPTPSSSND
TREQVLPAGQIVNCDCSAAGTQGNACRKDPRVGRCLCKPNFQGTHCELCAPGFYGPGCQP
CQCSSPGVADDRCDPDTGQCRCRVGFEGATCDRCAPGYFHFPLCQLCGCSPAGTLPEGCD

EAGRCLCQPEFAGPHCDRCRPGYHGFPNCQACTCDPRGALDQLCGAGGLCRCRPGYTGTA
CQECSPGFHGFPSCVPCHCSAEGSLHAACDPRSGQCSCRPRVTGLRCDTCVPGAYNFPYC
EAGSCHPAGLAPVDPALPEAQVPCMCRAHVEGPSCDRCKPGFWGLSPSNPEGCTRCSCDL
RGTLGGVAECQPGTGQCFCKPHVCGQACASCKDGFFGLDQADYFGCRSCRCDIGGALGQS
CEPRTGVCRCRPNTQGPTCSEPARDHYLPDLHHLRLELEEAATPEGHAVRFGFNPLEFEN
FSWRGYAQMAPVQPRIVARLNLTSPDLFWLVFRYVNRGAMSVSGRVSVREEGRSATCANC
TAQSQPVAFPPSTEPAFITVPQRGFGEPFVLNPGTWALRVEAEGVLLDYVVLLPSAYYEA
ALLQLRVTEACTYRPSAQQSGDNCLLYTHLPLDGFPSAAGLEALCRQDNSLPRPCPTEQL
SPSHPPLITCTGSDVDVQLQVAVPQPGRYALVVEYANEDARQEVGVAVHTPQRAPQQGLL
SLHPCLYSTLCRGTARDTQDHLAVFHLDSEASVRLTAEQARFFLHGVTLVPIEEFSPEFV
EPRVSCISSHGAFGPNSAACLPSRFPKPPQPIILRDCQVIPLPPGLPLTHAQDLTPAMSP
AGPRPRPPTAVDPDAEPTLLREPQATVVFTTHVPTLGRYAFLLHGYQPAHPTFPVEVLIN
AGRVWQGHANASFCPHGYGCRTLVVCEGQALLDVTHSELTVTVRVPKGRWLWLDYVLVVP
ENVYSFGYLREEPLDKSYDFISHCAAQGYHISPSSSSLFCRNAAASLSLFYNNGARPCGC
HEVGATGPTCEPFGGQCPCHAHVIGRDCSRCATGYWGFPNCRPCDCGARLCDELTGQCIC
PPRTIPPDCLLCQPQTFGCHPLVGCEECNCSGPGIQELTDPTCDTDSGQCKCRPNVTGRR
CDTCSPGFHGYPRCRPCDCHEAGTAPGVCDPLTGQCYCKENVQGPKCDQCSLGTFSLDAA
NPKGCTRCFCFGATERCRSSSYTRQEFVDMEGWVLLSTDRQVVPHERQPGTEMLRADLRH
VPEAVPEAFPELYWQAPPSYLGDRVSSYGGTLRYELHSETQRGDVFVPMESRPDVVLQGN
QMSITFLEPAYPTPGHVHRGQLQLVEGNFRHTETRNTVSREELMMVLASLEQLQIRALFS
QISSAVFLRRVALEVASPAGQGALASNVELCLCPASYRGDSCQECAPGFYRDVKGLFLGR
CVPCQCHGHSDRCLPGSGVCVDCQHNTEGAHCERCQAGFVSSRDDPSAPCVSCPCPLSVP
SNNFAEGCVLRGGRTQCLCKPGYAGASCERCAPGFFGNPLVLGSSCQPCDCSGNGDPNLL
FSDCDPLTGACRGCLRHTTGPRCEICAPGFYGNALLPGNCTRCDCTPCGTEACDPHSGHC
LCKAGVTGRRCDRCQEGHFGFDGCGGCRPCACGPAAEGSECHPQSGQCHCRPGTMGPQCR
ECAPGYWGLPEQGCRRCQCPGGRCDPHTGRCNCPPGLSGERCDTCSQQHQVPVPGGPVGH
SIHCEVCDHCVVLLLDDLERAGALLPAIHEQLRGINASSMAWARLHRLNASIADLQSQLR
SPLGPRHETAQQLEVLEQQSTSLGQDARRLGGQAVGTRDQASQLLAGTEATLGHAKTLLA
AIRAVDRTLSELMSQTGHLGLANASAPSGEQLLRTLAEVERLLWEMRARDLGAPQAAAEA
ELAAAQRLLARVQEQLSSLWEENQALATQTRDRLAQHEAGLMDLREALNRAVDATREAQE
LNSRNQERLEEALQRKQELSRDNATLQATLHAARDTLASVFRLLHSLDQAKEELERLAAS
LDGARTPLLQRMQTFSPAGSKLRLVEAAEAHAQQLGQLALNLSSIILDVNQDRLTQRAIE
ASNAYSRILQAVQAAEDAAGQALQQADHTWATVVRQGLVDRAQQLLANSTALEEAMLQEQ
QRLGLVWAALQGARTQLRDVRAKKDQLEAHIQAAQAMLAMDTDETSKKIAHAKAVAAEAQ
DTATRVQSQLQAMQENVERWQGQYEGLRGQDLGQAVLDAGHSVSTLEKTLPQLLAKLSIL
ENRGVHNASLALSASIGRVRELIAQARGAASKVKVPMKFNGRSGVQLRTPRDLADLAAYT
ALKFYLQGPEPEPGQGTEDRFVMYMGSRQATGDYMGVSLRDKKVHWVYQLGEAGPAVLSI
DEDIGEQFAAVSLDRTLQFGHMSVTVERQMIQETKGDTVAPGAEGLLNLRPDDFVFYVGG
YPSTFTPPPLLRFPGYRGCIEMDTLNEEVVSLYNFERTFQLDTAVDRPCARSKSTGDPWL
TDGSYLDGTGFARISFDSQISTTKRFEQELRLVSYSGVLFFLKQQSQFLCLAVQEGSLVL
LYDFGAGLKKAVPLQPPPPLTSASKAIQVFLLGGSRKRVLVRVERATVYSVEQDNDLELA
DAYYLGGVPPDQLPPSLRRLFPTGGSVRGCVKGIKALGKYVDLKRLNTTGVSAGCTADLL
VGRAMTFHGHGFLRLALSNVAPLTGNVYSGFGFHSAQDSALLYYRASPDGLCQVSLQQGR
VSLQLLRTEVKTQAGFADGAPHYVAFYSNATGVWLYVDDQLQQMKPHRGPPPELQPQPEG

PPRLLLGGLPESGTIYNFSGCISNVFVQRLLGPQRVFDLQQNLGSVNVSTGCAPALQAQT
PGLGPRGLQATARKASRRSRQPARHPACMLPPHLRTTRDSYQFGGSLSSHLEFVGILARH
RNWPSLSMHVLPRSSRGLLLFTARLRPGSPSLALFLSNGHFVAQMEGLGTRLRAQSRQRS
RPGRWHKVSVRWEKNRILLVTDGARAWSQEGPHRQHQGAEHPQPHTLFVGGLPASSHSSK
LPVTVGFSGCVKRLRLHGRPLGAPTRMAGVTPCILGPLEAGLFFPGSGGVITLDLPGATL
PDVGLELEVRPLAVTGLIFHLGQARTPPYLQLQVTEKQVLLRADDGAGEFSTSVTRPSVL
CDGQWHRLAVMKSGNVLRLEVDAQSNHTVGPLLAAAAGAPAPLYLGGLPEPMAVQPWPPA
YCGCMRRLAVNRSPVAMTRSVEVHGAVGASGCPAA
>sp|O15498|YKT6_HUMAN Synaptobrevin homolog YKT6 OS=Homo sapiens OX=9606
GN=YKT6 PE=1 SV=1
MKLYSLSVLYKGEAKVVLLKAAYDVSSFSFFQRSSVQEFMTFTSQLIVERSSKGTRASVK
EQDYLCHVYVRNDSLAGVVIADNEYPSRVAFTLLEKVLDEFSKQVDRIDWPVGSPATIHY
PALDGHLSRYQNPREADPMTKVQAELDETKIILHNTMESLLERGEKLDDLVSKSEVLGTQ
SKAFYKTARKQNSCCAIM
>sp|O15530|PDPK1_HUMAN 3-phosphoinositide-dependent protein kinase 1 OS=Homo
sapiens OX=9606 GN=PDPK1 PE=1 SV=1
MARTTSQLYDAVPIQSSVVLCSCPSPSMVRTQTESSTPPGIPGGSRQGPAMDGTAAEPRP
GAGSLQHAQPPPQPRKKRPEDFKFGKILGEGSFSTVVLARELATSREYAIKILEKRHIIK
ENKVPYVTRERDVMSRLDHPFFVKLYFTFQDDEKLYFGLSYAKNGELLKYIRKIGSFDET
CTRFYTAEIVSALEYLHGKGIIHRDLKPENILLNEDMHIQITDFGTAKVLSPESKQARAN
SFVGTAQYVSPELLTEKSACKSSDLWALGCIIYQLVAGLPPFRAGNEYLIFQKIIKLEYD
FPEKFFPKARDLVEKLLVLDATKRLGCEEMEGYGPLKAHPFFESVTWENLHQQTPPKLTA
YLPAMSEDDEDCYGNYDNLLSQFGCMQVSSSSSSHSLSASDTGLPQRSGSNIEQYIHDLD
SNSFELDLQFSEDEKRLLLEKQAGGNPWHQFVENNLILKMGPVDKRKGLFARRRQLLLTE
GPHLYYVDPVNKVLKGEIPWSQELRPEAKNFKTFFVHTPNRTYYLMDPSGNAHKWCRKIQ
EVWRQRYQSHPDAAVQ
>sp|O43242|PSMD3_HUMAN 26S proteasome non-ATPase regulatory subunit 3 OS=Homo
sapiens OX=9606 GN=PSMD3 PE=1 SV=2
MKQEGSARRRGADKAKPPPGGGEQEPPPPPAPQDVEMKEEAATGGGSTGEADGKTAAAAA
EHSQRELDTVTLEDIKEHVKQLEKAVSGKEPRFVLRALRMLPSTSRRLNHYVLYKAVQGF
FTSNNATRDFLLPFLEEPMDTEADLQFRPRTGKAASTPLLPEVEAYLQLLVVIFMMNSKR
YKEAQKISDDLMQKISTQNRRALDLVAAKCYYYHARVYEFLDKLDVVRSFLHARLRTATL
RHDADGQATLLNLLLRNYLHYSLYDQAEKLVSKSVFPEQANNNEWARYLYYTGRIKAIQL
EYSEARRTMTNALRKAPQHTAVGFKQTVHKLLIVVELLLGEIPDRLQFRQPSLKRSLMPY
FLLTQAVRTGNLAKFNQVLDQFGEKFQADGTYTLIIRLRHNVIKTGVRMISLSYSRISLA
DIAQKLQLDSPEDAEFIVAKAIRDGVIEASINHEKGYVQSKEMIDIYSTREPQLAFHQRI
SFCLDIHNMSVKAMRFPPKSYNKDLESAEERREREQQDLEFAKEMAEDDDDSFP
>sp|O43491|E41L2_HUMAN Band 4.1-like protein 2 OS=Homo sapiens OX=9606 GN=EPB41L2
PE=1 SV=1
MTTEVGSVSEVKKDSSQLGTDATKEKPKEVAENQQNQSSDPEEEKGSQPPPAAESQSSLR
RQKREKETSESRGISRFIPPWLKKQKSYTLVVAKDGGDKKEPTQAVVEEQVLDKEEPLPE
EQRQAKGDAEEMAQKKQEIKVEVKEEKPSVSKEEKPSVSKVEMQPTELVSKEREEKVKET
QEDKLEGGAAKRETKEVQTNELKAEKASQKVTKKTKTVQCKVTLLDGTEYSCDLEKHAKG
QVLFDKVCEHLNLLEKDYFGLLFQESPEQKNWLDPAKEIKRQLRNLPWLFTFNVKFYPPD

PSQLTEDITRYFLCLQLRQDIASGRLPCSFVTHALLGSYTLQAELGDYDPEEHGSIDLSE
FQFAPTQTKELEEKVAELHKTHRGLSPAQADSQFLENAKRLSMYGVDLHHAKDSEGVDIK
LGVCANGLLIYKDRLRINRFAWPKILKISYKRSNFYIKVRPAELEQFESTIGFKLPNHRA
AKRLWKVCVEHHTFYRLVSPEQPPKAKFLTLGSKFRYSGRTQAQTRQASTLIDRPAPHFE
RTSSKRVSRSLDGAPIGVMDQSLMKDFPGAAGEISAYGPGLVSIAVVQDGDGRREVRSPT
KAPHLQLIEGKKNSLRVEGDNIYVRHSNLMLEELDKAQEDILKHQASISELKRNFMESTP
EPRPNEWEKRRITPLSLQTQGSSHETLNIVEEKKRAEVGKDERVITEEMNGKEISPGSGP
GEIRKVEPVTQKDSTSLSSESSSSSSESEEEDVGEYRPHHRVTEGTIREEQEYEEEVEEE
PRPAAKVVEREEAVPEASPVTQAGASVITVETVIQENVGAQKIPGEKSVHEGALKQDMGE
EAEEEPQKVNGEVSHVDIDVLPQIICCSEPPVVKTEMVTISDASQRTEISTKEVPIVQTE
TKTITYESPQIDGGAGGDSGTLLTAQTITSESVSTTTTTHITKTVKGGISETRIEKRIVI
TGDGDIDHDQALAQAIREAREQHPDMSVTRVVVHKETELAEEGED
>sp|O43592|XPOT_HUMAN Exportin-T OS=Homo sapiens OX=9606 GN=XPOT PE=1 SV=2
MDEQALLGLNPNADSDFRQRALAYFEQLKISPDAWQVCAEALAQRTYSDDHVKFFCFQVL
EHQVKYKYSELTTVQQQLIRETLISWLQAQMLNPQPEKTFIRNKAAQVFALLFVTEYLTK
WPKFFFDILSVVDLNPRGVDLYLRILMAIDSELVDRDVVHTSEEARRNTLIKDTMREQCI
PNLVESWYQILQNYQFTNSEVTCQCLEVVGAYVSWIDLSLIANDRFINMLLGHMSIEVLR
EEACDCLFEVVNKGMDPVDKMKLVESLCQVLQSAGFFSIDQEEDVDFLARFSKLVNGMGQ
SLIVSWSKLIKNGDIKNAQEALQAIETKVALMLQLLIHEDDDISSNIIGFCYDYLHILKQ
LTVLSDQQKANVEAIMLAVMKKLTYDEEYNFENEGEDEAMFVEYRKQLKLLLDRLAQVSP
ELLLASVRRVFSSTLQNWQTTRFMEVEVAIRLLYMLAEALPVSHGAHFSGDVSKASALQD
MMRTLVTSGVSSYQHTSVTLEFFETVVRYEKFFTVEPQHIPCVLMAFLDHRGLRHSSAKV
RSRTAYLFSRFVKSLNKQMNPFIEDILNRIQDLLELSPPENGHQSLLSSDDQLFIYETAG
VLIVNSEYPAERKQALMRNLLTPLMEKFKILLEKLMLAQDEERQASLADCLNHAVGFASR
TSKAFSNKQTVKQCGCSEVYLDCLQTFLPALSCPLQKDILRSGVRTFLHRMIICLEEEVL
PFIPSASEHMLKDCEAKDLQEFIPLINQITAKFKIQVSPFLQQMFMPLLHAIFEVLLRPA
EENDQSAALEKQMLRRSYFAFLQTVTGSGMSEVIANQGAENVERVLVTVIQGAVEYPDPI
AQKTCFIILSKLVELWGGKDGPVGFADFVYKHIVPACFLAPLKQTFDLADAQTVLALSEC
AVTLKTIHLKRGPECVQYLQQEYLPSLQVAPEIIQEFCQALQQPDAKVFKNYLKVFFQRA
KP
>sp|O43674|NDUB5_HUMAN NADH dehydrogenase [ubiquinone] 1 beta subcomplex subunit 5, mitochondrial OS=Homo sapiens OX=9606 GN=NDUFB5 PE=1 SV=1
MAAMSLLRRVSVTAVAALSGRPLGTRLGFGGFLTRGFPKAAAPVRHSGDHGKRLFVIRPS
RFYDRRFLKLLRFYIALTGIPVAIFITLVNVFIGQAELAEIPEGYVPEHWEYYKHPISRW
IARNFYDSPEKIYERTMAVLQIEAEKAELRVKELEVRKLMHVRGDGPWYYYETIDKELID
HSPKATPDN
>sp|O43707|ACTN4_HUMAN Alpha-actinin-4 OS=Homo sapiens OX=9606 GN=ACTN4 PE=1 SV=2
MVDYHAANQSYQYGPSSAGNGAGGGGSMGDYMAQEDDWDRDLLLDPAWEKQQRKTFTAWC
NSHLRKAGTQIENIDEDFRDGLKLMLLLEVISGERLPKPERGKMRVHKINNVNKALDFIA
SKGVKLVSIGAEEIVDGNAKMTLGMIWTIILRFAIQDISVEETSAKEGLLLWCQRKTAPY
KNVNVQNFHISWKDGLAFNALIHRHRPELIEYDKLRKDDPVTNLNNAFEVAEKYLDIPKM
LDAEDIVNTARPDEKAIMTYVSSFYHAFSGAQKAETAANRICKVLAVNQENEHLMEDYEK
LASDLLEWIRRTIPWLEDRVPQKTIQEMQQKLEDFRDYRRVHKPPKVQEKCQLEINFNTL

QTKLRLSNRPAFMPSEGKMVSDINNGWQHLEQAEKGYEEWLLNEIRRLERLDHLAEKFRQ
KASIHEAWTDGKEAMLKHRDYETATLSDIKALIRKHEAFESDLAAHQDRVEQIAAIAQEL
NELDYYDSHNVNTRCQKICDQWDALGSLTHSRREALEKTEKQLEAIDQLHLEYAKRAAPF
NNWMESAMEDLQDMFIVHTIEEIEGLISAHDQFKSTLPDADREREAILAIHKEAQRIAES
NHIKLSGSNPYTTVTPQIINSKWEKVQQLVPKRDHALLEEQSKQQSNEHLRRQFASQANV
VGPWIQTKMEEIGRISIEMNGTLEDQLSHLKQYERSIVDYKPNLDLLEQQHQLIQEALIF
DNKHTNYTMEHIRVGWEQLLTTIARTINEVENQILTRDAKGISQEQMQEFRASFNHFDKD
HGGALGPEEFKACLISLGYDVENDRQGEAEFNRIMSLVDPNHSGLVTFQAFIDFMSRETT
DTDTADQVIASFKVLAGDKNFITAEELRRELPPDQAEYCIARMAPYQGPDAVPGALDYKS
FSTALYGESDL
>sp|O43708|MAAI_HUMAN Maleylacetoacetate isomerase OS=Homo sapiens OX=9606
GN=GSTZ1 PE=1 SV=3
MQAGKPILYSYFRSSCSWRVRIALALKGIDYKTVPINLIKDRGQQFSKDFQALNPMKQVP
TLKIDGITIHQSLAIIEYLEEMRPTPRLLPQDPKKRASVRMISDLIAGGIQPLQNLSVLK
QVGEEMQLTWAQNAITCGFNALEQILQSTAGIYCVGDEVTMADLCLVPQVANAERFKVDL
TPYPTISSINKRLLVLEAFQVSHPCRQPDTPTELRA
>sp|O43776|SYNC_HUMAN Asparagine--tRNA ligase, cytoplasmic OS=Homo sapiens OX=9606
GN=NARS1 PE=1 SV=1
MVLAELYVSDREGSDATGDGTKEKPFKTGLKALMTVGKEPFPTIYVDSQKENERWNVISK
SQLKNIKKMWHREQMKSESREKKEAEDSLRREKNLEEAKKITIKNDPSLPEPKCVKIGAL
EGYRGQRVKVFGWVHRLRRQGKNLMFLVLRDGTGYLQCVLADELCQCYNGVLLSTESSVA
VYGMLNLTPKGKQAPGGHELSCDFWELIGLAPAGGADNLINEESDVDVQLNNRHMMIRGE
NMSKILKARSMVTRCFRDHFFDRGYYEVTPPTLVQTQVEGGATLFKLDYFGEEAFLTQSS
QLYLETCLPALGDVFCIAQSYRAEQSRTRRHLAEYTHVEAECPFLTFDDLLNRLEDLVCD
VVDRILKSPAGSIVHELNPNFQPPKRPFKRMNYSDAIVWLKEHDVKKEDGTFYEFGEDIP
EAPERLMTDTINEPILLCRFPVEIKSFYMQRCPEDSRLTESVDVLMPNVGEIVGGSMRIF
DSEEILAGYKREGIDPTPYYWYTDQRKYGTCPHGGYGLGLERFLTWILNRYHIRDVCLYP
RFVQRCTP
>sp|O43795|MYO1B_HUMAN Unconventional myosin-Ib OS=Homo sapiens OX=9606
GN=MYO1B PE=1 SV=3
MAKMEVKTSLLDNMIGVGDMVLLEPLNEETFINNLKKRFDHSEIYTYIGSVVISVNPYRS
LPIYSPEKVEEYRNRNFYELSPHIFALSDEAYRSLRDQDKDQCILITGESGAGKTEASKL
VMSYVAAVCGKGAEVNQVKEQLLQSNPVLEAFGNAKTVRNDNSSRFGKYMDIEFDFKGDP
LGGVISNYLLEKSRVVKQPRGERNFHVFYQLLSGASEELLNKLKLERDFSRYNYLSLDSA
KVNGVDDAANFRTVRNAMQIVGFMDHEAESVLAVVAAVLKLGNIEFKPESRVNGLDESKI
KDKNELKEICELTGIDQSVLERAFSFRTVEAKQEKVSTTLNVAQAYYARDALAKNLYSRL
FSWLVNRINESIKAQTKVRKKVMGVLDIYGFEIFEDNSFEQFIINYCNEKLQQIFIELTL
KEEQEEYIREDIEWTHIDYFNNAIICDLIENNTNGILAMLDEECLRPGTVTDETFLEKLN
QVCATHQHFESRMSKCSRFLNDTSLPHSCFRIQHYAGKVLYQVEGFVDKNNDLLYRDLSQ
AMWKASHALIKSLFPEGNPAKINLKRPPTAGSQFKASVATLMKNLQTKNPNYIRCIKPND
KKAAHIFNEALVCHQIRYLGLLENVRVRRAGYAFRQAYEPCLERYKMLCKQTWPHWKGPA
RSGVEVLFNELEIPVEEYSFGRSKIFIRNPRTLFKLEDLRKQRLEDLATLIQKIYRGWKC
RTHFLLMKKSQIVIAAWYRRYAQQKRYQQTKSSALVIQSYIRGWKARKILRELKHQKRCK
EAVTTIAAYWHGTQARRELRRLKEEARNKHAIAVIWAYWLGSKARRELKRLKEEARRKHA

VAVIWAYWLGLKVRREYRKFFRANAGKKIYEFTLQRIVQKYFLEMKNKMPSLSPIDKNWP
SRPYLFLDSTHKELKRIFHLWRCKKYRDQFTDQQKLIYEEKLEASELFKDKKALYPSSVG
QPFQGAYLEINKNPKYKKLKDAIEEKIIIAEVVNKINRANGKSTSRIFLLTNNNLLLADQ
KSGQIKSEVPLVDVTKVSMSSQNDGFFAVHLKEGSEAASKGDFLFSSDHLIEMATKLYRT
TLSQTKQKLNIEISDEFLVQFRQDKVCVKFIQGNQKNGSVPTCKRKNNRLLEVAVP
>sp|O43813|LANC1_HUMAN Glutathione S-transferase LANCL1 OS=Homo sapiens OX=9606
GN=LANCL1 PE=1 SV=1
MAQRAFPNPYADYNKSLAEGYFDAAGRLTPEFSQRLTNKIRELLQQMERGLKSADPRDGT
GYTGWAGIAVLYLHLYDVFGDPAYLQLAHGYVKQSLNCLTKRSITFLCGDAGPLAVAAVL
YHKMNNEKQAEDCITRLIHLNKIDPHAPNEMLYGRIGYIYALLFVNKNFGVEKIPQSHIQ
QICETILTSGENLARKRNFTAKSPLMYEWYQEYYVGAAHGLAGIYYYLMQPSLQVSQGKL
HSLVKPSVDYVCQLKFPSGNYPPCIGDNRDLLVHWCHGAPGVIYMLIQAYKVFREEKYLC
DAYQCADVIWQYGLLKKGYGLCHGSAGNAYAFLTLYNLTQDMKYLYRACKFAEWCLEYGE
HGCRTPDTPFSLFEGMAGTIYFLADLLVPTKARFPAFEL
>sp|O60256|KPRB_HUMAN Phosphoribosyl pyrophosphate synthase-associated protein 2
OS=Homo sapiens OX=9606 GN=PRPSAP2 PE=1 SV=1
MFCVTPPELETKMNITKGGLVLFSANSNSSCMELSKKIAERLGVEMGKVQVYQEPNRETR
VQIQESVRGKDVFIIQTVSKDVNTTIMELLIMVYACKTSCAKSIIGVIPYFPYSKQCKMR
KRGSIVSKLLASMMCKAGLTHLITMDLHQKEIQGFFNIPVDNLRASPFLLQYIQEEIPDY
RNAVIVAKSPASAKRAQSFAERLRLGIAVIHGEAQDAESDLVDGRHSPPMVRSVAAIHPS
LEIPMLIPKEKPPITVVGDVGGRIAIIVDDIIDDVDSFLAAAETLKERGAYKIFVMATHG
LLSSDAPRRIEESAIDEVVVTNTIPHEVQKLQCPKIKTVDISMILSEAIRRIHNGESMSY
LFRNIGLDD
>sp|O60262|GBG7_HUMAN Guanine nucleotide-binding protein G(I)/G(S)/G(O) subunit
gamma-7 OS=Homo sapiens OX=9606 GN=GNG7 PE=1 SV=1
MSATNNIAQARKLVEQLRIEAGIERIKVSKAASDLMSYCEQHARNDPLLVGVPASENPFK
DKKPCIIL
>sp|O60437|PEPL_HUMAN Periplakin OS=Homo sapiens OX=9606 GN=PPL PE=1 SV=4
MNSLFRKRNKGKYSPTVQTRSISNKELSELIEQLQKNADQVEKNIVDTEAKMQSDLARLQ
EGRQPEHRDVTLQKVLDSEKLLYVLEADAAIAKHMKHPQGDMIAEDIRQLKERVTNLRGK
HKQIYRLAVKEVDPQVNWAALVEEKLDKLNNQSFGTDLPLVDHQVEEHNIFHNEVKAIGP
HLAKDGDKEQNSELRAKYQKLLAASQARQQHLSSLQDYMQRCTNELYWLDQQAKGRMQYD
WSDRNLDYPSRRRQYENFINRNLEAKEERINKLHSEGDQLLAAEHPGRNSIEAHMEAVHA
DWKEYLNLLICEESHLKYMEDYHQFHEDVKDAQELLRKVDSDLNQKYGPDFKDRYQIELL
LRELDDQEKVLDKYEDVVQGLQKRGQQVVPLKYRRETPLKPIPVEALCDFEGEQGLISRG
YSYTLQKNNGESWELMDSAGNKLIAPAVCFVIPPTDPEALALADSLGSQYRSVRQKAAGS
KRTLQQRYEVLKTENPGDASDLQGRQLLAGLDKVASDLDRQEKAITGILRPPLEQGRAVQ
DSAERAKDLKNITNELLRIEPEKTRSTAEGEAFIQALPGSGTTPLLRTRVEDTNRKYEHL
LQLLDLAQEKVDVANRLEKSLQQSWELLATHENHLNQDDTVPESSRVLDSKGQELAAMAC
ELQAQKSLLGEVEQNLQAAKQCSSTLASRFQEHCPDLERQEAEVHKLGQRFNNLRQQVER
RAQSLQSAKAAYEHFHRGHDHVLQFLVSIPSYEPQETDSLSQMETKLKNQKNLLDEIASR
EQEVQKICANSQQYQQAVKDYELEAEKLRSLLDLENGRRSHVSKRARLQSPATKVKEEEA
ALAAKFTEVYAINRQRLQNLEFALNLLRQQPEVEVTHETLQRNRPDSGVEEAWKIRKELD
EETERRRQLENEVKSTQEEIWTLRNQGPQESVVRKEVLKKVPDPVLEESFQQLQRTLAEE

QHKNQLLQEELEALQLQLRALEQETRDGGQEYVVKEVLRIEPDRAQADEVLQLREELEAL
RRQKGAREAEVLLLQQRVAALAEEKSRAQEKVTEKEVVKLQNDPQLEAEYQQLQEDHQRQ
DQLREKQEEELSFLQDKLKRLEKERAMAEGKITVKEVLKVEKDAATEREVSDLTRQYEDE
AAKARASQREKTELLRKIWALEEENAKVVVQEKVREIVRPDPKAESEVANLRLELVEQER
KYRGAEEQLRSYQSELEALRRRGPQVEVKEVTKEVIKYKTDPEMEKELQRLREEIVDKTR
LIERCDLEIYQLKKEIQALKDTKPQVQTKEVVQEILQFQEDPQTKEEVASLRAKLSEEQK
KQVDLERERASQEEQIARKEEELSRVKERVVQQEVVRYEEEPGLRAEASAFAESIDVELR
QIDKLRAELRRLQRRRTELERQLEELERERQARREAEREVQRLQQRLAALEQEEAEAREK
VTHTQKVVLQQDPQQAREHALLRLQLEEEQHRRQLLEGELETLRRKLAALEKAEVKEKVV
LSESVQVEKGDTEQEIQRLKSSLEEESRSKRELDVEVSRLEARLSELEFHNSKSSKELDF
LREENHKLQLERQNLQLETRRLQSEINMAATETRDLRNMTVADSGTNHDSRLWSLERELD
DLKRLSKDKDLEIDELQKRLGSVAVKREQRENHLRRSIVVIHPDTGRELSPEEAHRAGLI
DWNMFVKLRSQECDWEEISVKGPNGESSVIHDRKSGKKFSIEEALQSGRLTPAQYDRYVN
KDMSIQELAVLVSGQK
>sp|O60513|B4GT4_HUMAN Beta-1,4-galactosyltransferase 4 OS=Homo sapiens OX=9606 GN=B4GALT4 PE=1 SV=1
MGFNLTFHLSYKFRLLLLLTLCLTVVGWATSNYFVGAIQEIPKAKEFMANFHKTLILGKG
KTLTNEASTKKVELDNCPSVSPYLRGQSKLIFKPDLTLEEVQAENPKVSRGRYRPQECKA
LQRVAILVPHRNREKHLMYLLEHLHPFLQRQQLDYGIYVIHQAEGKKFNRAKLLNVGYLE
ALKEENWDCFIFHDVDLVPENDFNLYKCEEHPKHLVVGRNSTGYRLRYSGYFGGVTALSR
EQFFKVNGFSNNYWGWGGEDDDLRLRVELQRMKISRPLPEVGKYTMVFHTRDKGNEVNAE
RMKLLHQVSRVWRTDGLSSCSYKLVSVEHNPLYINITVDFWFGA
>sp|O60664|PLIN3_HUMAN Perilipin-3 OS=Homo sapiens OX=9606 GN=PLIN3 PE=1 SV=3
MSADGAEADGSTQVTVEEPVQQPSVVDRVASMPLISSTCDMVSAAYASTKESYPHIKTVC
DAAEKGVRTLTAAAVSGAQPILSKLEPQIASASEYAHRGLDKLEENLPILQQPTEKVLAD
TKELVSSKVSGAQEMVSSAKDTVATQLSEAVDATRGAVQSGVDKTKSVVTGGVQSVMGSR
LGQMVLSGVDTVLGKSEEWADNHLPLTDAELARIATSLDGFDVASVQQQRQEQSYFVRLG
SLSERLRQHAYEHSLGKLRATKQRAQEALLQLSQVLSLMETVKQGVDQKLVEGQEKLHQM
WLSWNQKQLQGPEKEPPKPEQVESRALTMFRDIAQQLQATCTSLGSSIQGLPTNVKDQVQ
QARRQVEDLQATFSSIHSFQDLSSSILAQSRERVASAREALDHMVEYVAQNTPVTWLVGP
FAPGITEKAPEEKK
>sp|O60733|PLPL9_HUMAN 85/88 kDa calcium-independent phospholipase A2 OS=Homo sapiens OX=9606 GN=PLA2G6 PE=1 SV=2
MQFFGRLVNTFSGVTNLFSNPFRVKEVAVADYTSSDRVREEGQLILFQNTPNRTWDCVLV
NPRNSQSGFRLFQLELEADALVNFHQYSSQLLPFYESSPQVLHTEVLQHLTDLIRNHPSW
SVAHLAVELGIRECFHHSRIISCANCAENEEGCTPLHLACRKGDGEILVELVQYCHTQMD
VTDYKGETVFHYAVQGDNSQVLQLLGRNAVAGLNQVNNQGLTPLHLACQLGKQEMVRVLL
LCNARCNIMGPNGYPIHSAMKFSQKGCAEMIISMDSSQIHSKDPRYGASPLHWAKNAEMA
RMLLKRGCNVNSTSSAGNTALHVAVMRNRFDCAIVLLTHGANADARGEHGNTPLHLAMSK
DNVEMIKALIVFGAEVDTPNDFGETPTFLASKIGRLVTRKAILTLLRTVGAEYCFPPIHG
VPAEQGSAAPHHPFSLERAQPPPISLNNLELQDLMHISRARKPAFILGSMRDEKRTHDHL
LCLDGGGVKGLIIIQLLIAIEKASGVATKDLFDWVAGTSTGGILALAILHSKSMAYMRGM
YFRMKDEVFRGSRPYESGPLEEFLKREFGEHTKMTDVRKPKVMLTGTLSDRQPAELHLFR
NYDAPETVREPRFNQNVNLRPPAQPSDQLVWRAARSSGAAPTYFRPNGRFLDGGLLANNP

TLDAMTEIHEYNQDLIRKGQANKVKKLSIVVSLGTGRSPQVPVTCVDVFRPSNPWELAKT
VFGAKELGKMVVDCCTDPDGRAVDRARAWCEMVGIQYFRLNPQLGTDIMLDEVSDTVLVN
ALWETEVYIYEHREEFQKLIQLLLSP
>sp|O60830|TI17B_HUMAN Mitochondrial import inner membrane translocase subunit Tim17-B OS=Homo sapiens OX=9606 GN=TIMM17B PE=1 SV=1
MEEYAREPCPWRIVDDCGGAFTMGVIGGGVFQAIKGFRNAPVGIRHRLRGSANAVRIRAP
QIGGSFAVWGGLFSTIDCGLVRLRGKEDPWNSITSGALTGAVLAARSGPLAMVGSAMMGG
ILLALIEGVGILLTRYTAQQFRNAPPFLEDPSQLPPKDGTPAPGYPSYQQYH
>sp|O60888|CUTA_HUMAN Protein CutA OS=Homo sapiens OX=9606 GN=CUTA PE=1 SV=2
MSGGRAPAVLLGGVASLLLSFVWMPALLPVASRLLLLPRVLLTMASGSPPTQPSPASDSG
SGYVPGSVSAAFVTCPNEKVAKEIARAVVEKRLAACVNLIPQITSIYEWKGKIEEDSEVL
MMIKTQSSLVPALTDFVRSVHPYEVAEVIALPVEQGNFPYLQWVRQVTESVSDSITVLP
>sp|O75083|WDR1_HUMAN WD repeat-containing protein 1 OS=Homo sapiens OX=9606 GN=WDR1 PE=1 SV=4
MPYEIKKVFASLPQVERGVSKIIGGDPKGNNFLYTNGKCVILRNIDNPALADIYTEHAHQ
VVVAKYAPSGFYIASGDVSGKLRIWDTTQKEHLLKYEYQPFAGKIKDIAWTEDSKRIAVV
GEGREKFGAVFLWDSGSSVGEITGHNKVINSVDIKQSRPYRLATGSDDNCAAFFEGPPFK
FKFTIGDHSRFVNCVRFSPDGNRFATASADGQIYIYDGKTGEKVCALGGSKAHDGGIYAI
SWSPDSTHLLSASGDKTSKIWDVSVNSVVSTFPMGSTVLDQQLGCLWQKDHLLSVSLSGY
INYLDRNNPSKPLHVIKGHSKSIQCLTVHKNGGKSYIYSGSHDGHINYWDSETGENDSFA
GKGHTNQVSRMTVDESGQLISCSMDDTVRYTSLMLRDYSGQGVVKLDVQPKCVAVGPGGY
AVVVCIGQIVLLKDQRKCFSIDNPGYEPEVVAVHPGGDTVAIGGVDGNVRLYSILGTTLK
DEGKLLEAKGPVTDVAYSHDGAFLAVCDASKVVTVFSVADGYSENNVFYGHHAKIVCLAW
SPDNEHFASGGMDMMVYVWTLSDPETRVKIQDAHRLHHVSSLAWLDEHTLVTTSHDASVK
EWTITY
>sp|O75095|MEGF6_HUMAN Multiple epidermal growth factor-like domains protein 6 OS=Homo sapiens OX=9606 GN=MEGF6 PE=1 SV=4
MSFLEEARAAGRAVVLALVLLLLPAVPVGASVPPRPLLPLQPGMPHVCAEQELTLVGRRQ
PCVQALSHTVPVWKAGCGWQAWCVGHERRTVYYMGYRQVYTTEARTVLRCCRGWMQQPDE
EGCLSAECSASLCFHGGRCVPGSAQPCHCPPGFQGPRCQYDVDECRTHNGGCQHRCVNTP
GSYLCECKPGFRLHTDSRTCLAINSCALGNGGCQHHCVQLTITRHRCQCRPGFQLQEDGR
HCVRRSPCANRNGSCMHRCQVVRGLARCECHVGYQLAADGKACEDVDECAAGLAQCAHGC
LNTQGSFKCVCHAGYELGADGRQCYRIEMEIVNSCEANNGGCSHGCSHTSAGPLCTCPRG
YELDTDQRTCIDVDDCADSPCCQQVCTNNPGGYECGCYAGYRLSADGCGCEDVDECASSR
GGCEHHCTNLAGSFQCSCEAGYRLHEDRRGCSPLEEPMVDLDGELPFVRPLPHIAVLQDE
LPQLFQDDDVGADEEEAELRGEHTLTEKFVCLDDSFGHDCSLTCDDCRNGGTCLLGLDGC
DCPEGWTGLICNETCPPDTFGKNCSFSCSCQNGGTCDSVTGACRCPPGVSGTNCEDGCPK
GYYGKHCRKKCNCANRGRCHRLYGACLCDPGLYGRFCHLTCPPWAFGPGCSEECQCVQPH
TQSCDKRDGSCSCKAGFRGERCQAECELGYFGPGCWQACTCPVGVACDSVSGECGKRCPA
GFQGEDCGQECPVGTFGVNCSSSCSCGGAPCHGVTGQCRCPPGRTGEDCEADCPEGRWGL
GCQEICPACQHAARCDPETGACLCLPGFVGSRCQDVCPAGWYGPSCQTRCSCANDGHCHP
ATGHCSCAPGWTGFSCQRACDTGHWGPDCSHPCNCSAGHGSCDAISGLCLCEAGYVGPRC
EQQCPQGHFGPGCEQRCQCQHGAACDHVSGACTCPAGWRGTFCEHACPAGFFGLDCRSAC
NCTAGAACDAVNGSCLCPAGRRGPRCAETCPAHTYGHNCSQACACFNGASCDPVHGQCHC

APGWMGPSCLQACPAGLYGDNCRHSCLCQNGGTCDPVSGHCACPEGWAGLACEKECLPRD
VRAGCRHSGGCLNGGLCDPHTGRCLCPAGWTGDKCQSPCLRGWFGEACAQRCSCPPGAAC
HHVTGACRCPPGFTGSGCEQACPPGSFGEDCAQMCQCPGENPACHPATGTCSCAAGYHGP
SCQQRCPPGRYGPGCEQLCGCLNGGSCDAATGACRCPTGFLGTDCNLTCPQGRFGPNCTH
VCGCGQGAACDPVTGTCLCPPGRAGVRCERGCPQNRFGVGCEHTCSCRNGGLCHASNGSC
SCGLGWTGRHCELACPPGRYGAACHLECSCHNNSTCEPATGTCRCGPGFYGQACEHPCPP
GFHGAGCQGLCWCQHGAPCDPISGRCLCPAGFHGHFCERGCEPGSFGEGCHQRCDCDGGA
PCDPVTGLCLCPPGRSGATCNLDCRRGQFGPSCTLHCDCGGGADCDPVSGQCHCVDGYMG
PTCREGGPLRLPENPSLAQGSAGTLPASSRPTSRSGGPARH
>sp|O75144|ICOSL_HUMAN ICOS ligand OS=Homo sapiens OX=9606 GN=ICOSLG PE=1 SV=2
MRLGSPGLLFLLFSSLRADTQEKEVRAMVGSDVELSCACPEGSRFDLNDVYVYWQTSESK
TVVTYHIPQNSSLENVDSRYRNRALMSPAGMLRGDFSLRLFNVTPQDEQKFHCLVLSQSL
GFQEVLSVEVTLHVAANFSVPVVSAPHSPSQDELTFTCTSINGYPRPNVYWINKTDNSLL
DQALQNDTVFLNMRGLYDVVSVLRIARTPSVNIGCCIENVLLQQNLTVGSQTGNDIGERD
KITENPVSTGEKNAATWSILAVLCLLVVVAVAIGWVCRDRCLQHSYAGAWAVSPETELTG
HV
>sp|O75182|SIN3B_HUMAN Paired amphipathic helix protein Sin3b OS=Homo sapiens OX=9606 GN=SIN3B PE=1 SV=2
MAHAGGGSGGSGAGGPAGRGLSGARWGRSGSAGHEKLPVHVEDALTYLDQVKIRFGSDPA
TYNGFLEIMKEFKSQSIDTPGVIRRVSQLFHEHPDLIVGFNAFLPLGYRIDIPKNGKLNI
QSPLTSQENSHNHGDGAEDFKQQVPYKEDKPQVPLESDSVEFNNAISYVNKIKTRFLDHP
EIYRSFLEILHTYQKEQLNTRGRPFRGMSEEEVFTEVANLFRGQEDLLSEFGQFLPEAKR
SLFTGNGPCEMHSVQKNEHDKTPEHSRKRSRPSLLRPVSAPAKKKMKLRGTKDLSIAAVG
KYGTLQEFSFFDKVRRVLKSQEVYENFLRCIALFNQELVSGSELLQLVSPFLGKFPELFA
QFKSFLGVKELSFAPPMSDRSGDGISREIDYASCKRIGSSYRALPKTYQQPKCSGRTAIC
KELDHWTLLQGSWTDDYCMSKFKNTCWIPGYSAGVLNDTWVSFPSWSEDSTFVSSKKTPY
EEQLHRCEDERFELDVVLETNLATIRVLESVQKKLSRMAPEDQEKFRLDDSLGGTSEVIQ
RRAIYRIYGDKAPEIIESLKKNPVTAVPVVLKRLKAKEEEWREAQQGFNKIWREQYEKAY
LKSLDHQAVNFKQNDTKALRSKSLLNEIESVYDEHQEQHSEGRSAPSSEPHLIFVYEDRQ
ILEDAAALISYYVKRQPAIQKEDQGTIHQLLHQFVPSLFFSQQLDLGASEESADEDRDSP
QGQTTDPSERKKPAPGPHSSPPEEKGAFGDAPATEQPPLPPPAPHKPLDDVYSLFFANNN
WYFFLRLHQTLCSRLLKIYRQAQKQLLEYRTEKEREKLLCEGRREKGSDPAMELRLKQPS
EVELEEYYPAFLDMVRSLLEGSIDPTQYEDTLREMFTIHAYVGFTMDKLVQNIARQLHHL
VSDDVCLKVVELYLNEKKRGAAGGNLSSRCVRAARETSYQWKAERCMADENCFKVMFLQR
KGQVIMTIELLDTEEAQTEDPVEVQHLARYVEQYVGTEGASSSPTEGFLLKPVFLQRNLK
KFRRRWQSEQARALRGEARSSWKRLVGVESACDVDCRFKLSTHKMVFIVNSEDYMYRRGT
LCRAKQVQPLVLLRHHQHFEEWHSRWLEDNVTVEAASLVQDWLMGEEDEDMVPCKTLCET
VHVHGLPVTRYRVQYSRRPASP
>sp|O75367|H2AY_HUMAN Core histone macro-H2A.1 OS=Homo sapiens OX=9606 GN=MACROH2A1 PE=1 SV=5
MSSRGGKKKSTKTSRSAKAGVIFPVGRMLRYIKKGHPKYRIGVGAPVYMAAVLEYLTAEI
LELAGNAARDNKKGRVTPRHILLAVANDEELNQLLKGVTIASGGVLPNIHPELLAKKRGS
KGKLEAIITPPPAKKAKSPSQKKPVSKKAGGKKGARKSKKKQGEVSKAASADSTTEGTPA
DGFTVLSTKSLFLGQKLQVVQADIASIDSDAVVHPTNTDFYIGGEVGNTLEKKGGKEFVE

AVLELRKKNGPLEVAGAAVSAGHGLPAKFVIHCNSPVWGADKCEELLEKTVKNCLALADD
KKLKSIAFPSIGSGRNGFPKQTAAQLILKAISSYFVSTMSSSIKTVYFVLFDSESIGIYV
QEMAKLDAN
>sp|O75369|FLNB_HUMAN Filamin-B OS=Homo sapiens OX=9606 GN=FLNB PE=1 SV=2
MPVTEKDLAEDAPWKKIQQNTFTRWCNEHLKCVNKRIGNLQTDLSDGLRLIALLEVLSQK
RMYRKYHQRPTFRQMQLENVSVALEFLDRESIKLVSIDSKAIVDGNLKLILGLVWTLILH
YSISMPVWEDEGDDDAKKQTPKQRLLGWIQNKIPYLPITNFNQNWQDGKALGALVDSCAP
GLCPDWESWDPQKPVDNAREAMQQADDWLGVPQVITPEEIIHPDVDEHSVMTYLSQFPKA
KLKPGAPLKPKLNPKKARAYGRGIEPTGNMVKQPAKFTVDTISAGQGDVMVFVEDPEGNK
EEAQVTPDSDKNKTYSVEYLPKVTGLHKVTVLFAGQHISKSPFEVSVDKAQGDASKVTAK
GPGLEAVGNIANKPTYFDIYTAGAGVGDIGVEVEDPQGKNTVELLVEDKGNQVYRCVYKP
MQPGPHVVKIFFAGDTIPKSPFVVQVGEACNPNACRASGRGLQPKGVRIRETTDFKVDTK
AAGSGELGVTMKGPKGLEELVKQKDFLDGVYAFEYYPSTPGRYSIAITWGGHHIPKSPFE
VQVGPEAGMQKVRAWGPGLHGGIVGRSADFVVESIGSEVGSLGFAIEGPSQAKIEYNDQN
DGSCDVKYWPKEPGEYAVHIMCDDEDIKDSPYMAFIHPATGGYNPDLVRAYGPGLEKSGC
IVNNLAEFTVDPKDAGKAPLKIFAQDGEGQRIDIQMKNRMDGTYACSYTPVKAIKHTIAV
VWGGVNIPHSPYRVNIGQGSHPQKVKVFGPGVERSGLKANEPTHFTVDCTEAGEGDVSVG
IKCDARVLSEDEEDVDFDIIHNANDTFTVKYVPPAAGRYTIKVLFASQEIPASPFRVKVD
PSHDASKVKAEGPGLSKAGVENGKPTHFTVYTKGAGKAPLNVQFNSPLPGDAVKDLDIID
NYDYSHTVKYTPTQQGNMQVLVTYGGDPIPKSPFTVGVAAPLDLSKIKLNGLENRVEVGK
DQEFTVDTRGAGGQGKLDVTILSPSRKVVPCLVTPVTGRENSTAKFIPREEGLYAVDVTY
DGHPVPGSPYTVEASLPPDPSKVKAHGPGLEGGLVGKPAEFTIDTKGAGTGGLGLTVEGP
CEAKIECSDNGDGTCSVSYLPTKPGEYFVNILFEEVHIPGSPFKADIEMPFDPSKVVASG
PGLEHGKVGEAGLLSVDCSEAGPGALGLEAVSDSGTKAEVSIQNNKDGTYAVTYVPLTAG
MYTLTMKYGGELVPHFPARVKVEPAVDTSRIKVFGPGIEGKDVFREATTDFTVDSRPLTQ
VGGDHIKAHIANPSGASTECFVTDNADGTYQVEYTPFEKGLHVVEVTYDDVPIPNSPFKV
AVTEGCQPSRVQAQGPGLKEAFTNKPNVFTVVTRGAGIGGLGITVEGPSESKINCRDNKD
GSCSAEYIPFAPGDYDVNITYGGAHIPGSPFRVPVKDVVDPSKVKIAGPGLGSGVRARVL
QSFTVDSSKAGLAPLEVRVLGPRGLVEPVNVVDNGDGTHTVTYTPSQEGPYMVSVKYADE
EIPRSPFKVKVLPTYDASKVTASGPGLSSYGVPASLPVDFAIDARDAGEGLLAVQITDQE
GKPKRAIVHDNKDGTYAVTYIPDKTGRYMIGVTYGGDDIPLSPYRIRATQTGDASKCLAT
GPGIASTVKTGEEVGFVVDAKTAGKGKVTCTVLTPDGTEAEADVIENEDGTYDIFYTAAK
PGTYVIYVRFGGVDIPNSPFTVMATDGEVTAVEEAPVNACPPGFRPWVTEEAYVPVSDMN
GLGFKPFDLVIPFAVRKGEITGEVHMPSGKTATPEIVDNKDGTVTVRYAPTEVGLHEMHI
KYMGSHIPESPLQFYVNYPNSGSVSAYGPGLVYGVANKTATFTIVTEDAGEGGLDLAIEG
PSKAEISCIDNKDGTCTVTYLPTLPGDYSILVKYNDKHIPGSPFTAKITDDSRRCSQVKL
GSAADFLLDISETDLSSLTASIKAPSGRDEPCLLKRLPNNHIGISFIPREVGEHLVSIKK
NGNHVANSPVSIMVVQSEIGDARRAKVYGRGLSEGRTFEMSDFIVDTRDAGYGGISLAVE
GPSKVDIQTEDLEDGTCKVSYFPTVPGVYIVSTKFADEHVPGSPFTVKISGEGRVKESIT
RTSRAPSVATVGSICDLNLKIPEINSSDMSAHVTSPSGRVTEAEIVPMGKNSHCVRFVPQ
EMGVHTVSVKYRGQHVTGSPFQFTVGPLGEGGAHKVRAGGPGLERGEAGVPAEFSIWTRE
AGAGGLSIAVEGPSKAEITFDDHKNGSCGVSYIAQEPGNYEVSIKFNDEHIPESPYLVPV
IAPSDDARRLTVMSLQESGLKVNQPASFAIRLNGAKGKIDAKVHSPSGAVEECHVSELEP
DKYAVRFIPHENGVHTIDVKFNGSHVVGSPFKVRVGEPGQAGNPALVSAYGTGLEGGTTG

IQSEFFINTTRAGPGTLSVTIEGPSKVKMDCQETPEGYKVMYTPMAPGNYLISVKYGGPN
HIVGSPFKAKVTGQRLVSPGSANETSSILVESVTRSSTETCYSAIPKASSDASKVTSKGA
GLSKAFVGQKSSFLVDCSKAGSNMLLIGVHGPTTPCEEVSMKHVGNQQYNVTYVVKERGD
YVLAVKWGEEHIPGSPFHVTVP
>sp|O75663|TIPRL_HUMAN TIP41-like protein OS=Homo sapiens OX=9606 GN=TIPRL PE=1 SV=2
MMIHGFQSSHRDFCFGPWKLTASKTHIMKSADVEKLADELHMPSLPEMMFGDNVLRIQHG
SGFGIEFNATDALRCVNNYQGMLKVACAEEWQESRTEGEHSKEVIKPYDWTYTTDYKGTL
LGESLKLKVVPTTDHIDTEKLKAREQIKFFEEVLLFEDELHDHGVSSLSVKIRVMPSSFF
LLLRFFLRIDGVLIRMNDTRLYHEADKTYMLREYTSRESKISSLMHVPPSLFTEPNEISQ
YLPIKEAVCEKLIFPERIDPNPADSQKSTQVE
>sp|O75695|XRP2_HUMAN Protein XRP2 OS=Homo sapiens OX=9606 GN=RP2 PE=1 SV=4
MGCFFSKRRKADKESRPENEEERPKQYSWDQREKVDPKDYMFSGLKDETVGRLPGTVAGQ
QFLIQDCENCNIYIFDHSATVTIDDCTNCIIFLGPVKGSVFFRNCRDCKCTLACQQFRVR
DCRKLEVFLCCATQPIIESSSNIKFGCFQWYYPELAFQFKDAGLSIFNNTWSNIHDFTPV
SGELNWSLLPEDAVVQDYVPIPTTEELKAVRVSTEANRSIVPISRGQRQKSSDESCLVVL
FAGDYTIANARKLIDEMVGKGFFLVQTKEVSMKAEDAQRVFREKAPDFLPLLNKGPVIAL
EFNGDGAVEVCQLIVNEIFNGTKMFVSESKETASGDVDSFYNFADIQMGI
>sp|O75781|PALM_HUMAN Paralemmin-1 OS=Homo sapiens OX=9606 GN=PALM PE=1 SV=2
MEVLAAETTSQQERLQAIAEKRKRQAEIENKRRQLEDERRQLQHLKSKALRERWLLEGTP
SSASEGDEDLRRQMQDDEQKTRLLEDSVSRLEKEIEVLERGDSAPATAKENAAAPSPVRA
PAPSPAKEERKTEVVMNSQQTPVGTPKDKRVSNTPLRTVDGSPMMKAAMYSVEITVEKDK
VTGETRVLSSTTLLPRQPLPLGIKVYEDETKVVHAVDGTAENGIHPLSSSEVDELIHKAD
EVTLSEAGSTAGAAETRGAVEGAARTTPSRREITGVQAQPGEATSGPPGIQPGQEPPVTM
IFMGYQNVEDEAETKKVLGLQDTITAELVVIEDAAEPKEPAPPNGSAAEPPTEAASREEN
QAGPEATTSDPQDLDMKKHRCKCCSIM
>sp|O75828|CBR3_HUMAN Carbonyl reductase [NADPH] 3 OS=Homo sapiens OX=9606 GN=CBR3 PE=1 SV=3
MSSCSRVALVTGANRGIGLAIARELCRQFSGDVVLTARDVARGQAAVQQLQAEGLSPRFH
QLDIDDLQSIRALRDFLRKEYGGLNVLVNNAAVAFKSDDPMPFDIKAEMTLKTNFFATRN
MCNELLPIMKPHGRVVNISSLQCLRAFENCSEDLQERFHSETLTEGDLVDLMKKFVEDTK
NEVHEREGWPNSPYGVSKLGVTVLSRILARRLDEKRKADRILVNACCPGPVKTDMDGKDS
IRTVEEGAETPVYLALLPPDATEPQGQLVHDKVVQNW
>sp|O75874|IDHC_HUMAN Isocitrate dehydrogenase [NADP] cytoplasmic OS=Homo sapiens OX=9606 GN=IDH1 PE=1 SV=2
MSKKISGGSVVEMQGDEMTRIIWELIKEKLIFPYVELDLHSYDLGIENRDATNDQVTKDA
AEAIKKHNVGVKCATITPDEKRVEEFKLKQMWKSPNGTIRNILGGTVFREAIICKNIPRL
VSGWVKPIIIGRHAYGDQYRATDFVVPGPGKVEITYTPSDGTQKVTYLVHNFEEGGGVAM
GMYNQDKSIEDFAHSSFQMALSKGWPLYLSTKNTILKKYDGRFKDIFQEIYDKQYKSQFE
AQKIWYEHRLIDDMVAQAMKSEGGFIWACKNYDGDVQSDSVAQGYGSLGMMTSVLVCPDG
KTVEAEAAHGTVTRHYRMYQKGQETSTNPIASIFAWTRGLAHRAKLDNNKELAFFANALE
EVSIETIEAGFMTKDLAACIKGLPNVQRSDYLNTFEFMDKLGENLKIKLAQAKL
>sp|O75923|DYSF_HUMAN Dysferlin OS=Homo sapiens OX=9606 GN=DYSF PE=1 SV=1
MLRVFILYAENVHTPDTDISDAYCSAVFAGVKKRTKVIKNSVNPVWNEGFEWDLKGIPLD

QGSELHVVVKDHETMGRNRFLGEAKVPLREVLATPSLSASFNAPLLDTKKQPTGASLVLQ
VSYTPLPGAVPLFPPPTPLEPSPTLPDLDVVADTGGEEDTEDQGLTGDEAEPFLDQSGGP
GAPTTPRKLPSRPPPHYPGIKRKRSAPTSRKLLSDKPQDFQIRVQVIEGRQLPGVNIKPV
VKVTAAGQTKRTRIHKGNSPLFNETLFFNLFDSPGELFDEPIFITVVDSRSLRTDALLGE
FRMDVGTIYREPRHAYLRKWLLLSDPDDFSAGARGYLKTSLCVLGPGDEAPLERKDPSED
KEDIESNLLRPTGVALRGAHFCLKVFRAEDLPQMDDAVMDNVKQIFGFESNKKNLVDPFV
EVSFAGKMLCSKILEKTANPQWNQNITLPAMFPSMCEKMRIRIIDWDRLTHNDIVATTYL
SMSKISAPGGEIEEEPAGAVKPSKASDLDDYLGFLPTFGPCYINLYGSPREFTGFPDPYT
ELNTGKGEGVAYRGRLLLSLETKLVEHSEQKVEDLPADDILRVEKYLRRRKYSLFAAFYS
ATMLQDVDDAIQFEVSIGNYGNKFDMTCLPLASTTQYSRAVFDGCHYYYLPWGNVKPVVV
LSSYWEDISHRIETQNQLLGIADRLEAGLEQVHLALKAQCSTEDVDSLVAQLTDELIAGC
SQPLGDIHETPSATHLDQYLYQLRTHHLSQITEAALALKLGHSELPAALEQAEDWLLRLR
ALAEEPQNSLPDIVIWMLQGDKRVAYQRVPAHQVLFSRRGANYCGKNCGKLQTIFLKYPM
EKVPGARMPVQIRVKLWFGLSVDEKEFNQFAEGKLSVFAETYENETKLALVGNWGTTGLT
YPKFSDVTGKIKLPKDSFRPSAGWTWAGDWFVCPEKTLLHDMDAGHLSFVEEVFENQTRL
PGGQWIYMSDNYTDVNGEKVLPKDDIECPLGWKWEDEEWSTDLNRAVDEQGWEYSITIPP
ERKPKHWVPAEKMYYTHRRRRWVRLRRRDLSQMEALKRHRQAEAEGEGWEYASLFGWKFH
LEYRKTDAFRRRRWRRRMEPLEKTGPAAVFALEGALGGVMDDKSEDSMSVSTLSFGVNRP
TISCIFDYGNRYHLRCYMYQARDLAAMDKDSFSDPYAIVSFLHQSQKTVVVKNTLNPTWD
QTLIFYEIEIFGEPATVAEQPPSIVVELYDHDTYGADEFMGRCICQPSLERMPRLAWFPL
TRGSQPSGELLASFELIQREKPAIHHIPGFEVQETSRILDESEDTDLPYPPPQREANIYM
VPQNIKPALQRTAIEILAWGLRNMKSYQLANISSPSLVVECGGQTVQSCVIRNLRKNPNF
DICTLFMEVMLPREELYCPPITVKVIDNRQFGRRPVVGQCTIRSLESFLCDPYSAESPSP
QGGPDDVSLLSPGEDVLIDIDDKEPLIPIQEEEFIDWWSKFFASIGEREKCGSYLEKDFD
TLKVYDTQLENVEAFEGLSDFCNTFKLYRGKTQEETEDPSVIGEFKGLFKIYPLPEDPAI
PMPPRQFHQLAAQGPQECLVRIYIVRAFGLQPKDPNGKCDPYIKISIGKKSVSDQDNYIP
CTLEPVFGKMFELTCTLPLEKDLKITLYDYDLLSKDEKIGETVVDLENRLLSKFGARCGL
PQTYCVSGPNQWRDQLRPSQLLHLFCQQHRVKAPVYRTDRVMFQDKEYSIEEIEAGRIPN
PHLGPVEERLALHVLQQQGLVPEHVESRPLYSPLQPDIEQGKLQMWVDLFPKALGRPGPP
FNITPRRARRFFLRCIIWNTRDVILDDLSLTGEKMSDIYVKGWMIGFEEHKQKTDVHYRS
LGGEGNFNWRFIFPFDYLPAEQVCTIAKKDAFWRLDKTESKIPARVVFQIWDNDKFSFDD
FLGSLQLDLNRMPKPAKTAKKCSLDQLDDAFHPEWFVSLFEQKTVKGWWPCVAEEGEKKI
LAGKLEMTLEIVAESEHEERPAGQGRDEPNMNPKLEDPRRPDTSFLWFTSPYKTMKFILW
RRFRWAIILFIILFILLLFLAIFIYAFPNYAAMKLVKPFS
>tr|O75942|O75942_HUMAN Major prion protein OS=Homo sapiens OX=9606 GN=PRNP PE=3 SV=2
MANLGCWMLVLFVATWSDLGLCKKRPKPGGWNTGGSRYPGQGSPGGNRYPPQGGGGWGQP
HGGGWGQPHGGGWGQPHGGGWGQPHGGGWGQPHGGGWGQPHGGGWGQPHGGGWGQPHGG
G
WGQGGGTHSQWNKPSKPKTNMKHMAGAAAAGAVVGGLGGYMLGSAMSRPIIHFGSDYEDR
YYRENMHRYPNQVYYRPMDEYSNQNNFVHDCVNITIKQHTVTTTTKGENFTETDVKMMER
VVEQMCITQYERESQAYYQRGSSMVLFSSPPVILLISFLIFLIVG
>sp|O75955|FLOT1_HUMAN Flotillin-1 OS=Homo sapiens OX=9606 GN=FLOT1 PE=1 SV=3
MFFTCGPNEAMVVSGFCRSPPVMVAGGRVFVLPCIQQIQRISLNTLTLNVKSEKVYTRHG

VPISVTGIAQVKIQGQNKEMLAAACQMFLGKTEAEIAHIALETLEGHQRAIMAHMTVEEI
YKDRQKFSEQVFKVASSDLVNMGISVVSYTLKDIHDDQDYLHSLGKARTAQVQKDARIGE
AEAKRDAGIREAKAKQEKVSAQYLSEIEMAKAQRDYELKKAAYDIEVNTRRAQADLAYQL
QVAKTKQQIEEQRVQVQVVERAQQVAVQEQEIARREKELEARVRKPAEAERYKLERLAEA
EKSQLIMQAEAEAASVRMRGEAEAFAIGARARAEAEQMAKKAEAFQLYQEAAQLDMLLEK
LPQVAEEISGPLTSANKITLVSSGSGTMGAAKVTGEVLDILTRLPESVERLTGVSISQVN
HKPLRTA
>sp|O76024|WFS1_HUMAN Wolframin OS=Homo sapiens OX=9606 GN=WFS1 PE=1 SV=2
MDSNTAPLGPSCPQPPPAPQPQARSRLNATASLEQERSERPRAPGPQAGPGPGVRDAAAP
AEPQAQHTRSRERADGTGPTKGDMEIPFEEVLERAKAGDPKAQTEVGKHYLQLAGDTDEE
LNSCTAVDWLVLAAKQGRREAVKLLRRCLADRRGITSENEREVRQLSSETDLERAVRKAA
LVMYWKLNPKKKKQVAVAELLENVGQVNEHDGGAQPGPVPKSLQKQRRMLERLVSSESKN
YIALDDFVEITKKYAKGVIPSSLFLQDDEDDDELAGKSPEDLPLRLKVVKYPLHAIMEIK
EYLIDMASRAGMHWLSTIIPTHHINALIFFFIVSNLTIDFFAFFIPLVIFYLSFISMVIC
TLKVFQDSKAWENFRTLTDLLLRFEPNLDVEQAEVNFGWNHLEPYAHFLLSVFFVIFSFP
IASKDCIPCSELAVITGFFTVTSYLSLSTHAEPYTRRALATEVTAGLLSLLPSMPLNWPY
LKVLGQTFITVPVGHLVVLNVSVPCLLYVYLLYLFFRMAQLRNFKGTYCYLVPYLVCFMW
CELSVVILLESTGLGLLRASIGYFLFLFALPILVAGLALVGVLQFARWFTSLELTKIAVT
VAVCSVPLLLRWWTKASFSVVGMVKSLTRSSMVKLILVWLTAIVLFCWFYVYRSEGMKVY
NSTLTWQQYGALCGPRAWKETNMARTQILCSHLEGHRVTWTGRFKYVRVTDIDNSAESAI
NMLPFFIGDWMRCLYGEAYPACSPGNTSTAEEELCRLKLLAKHPCHIKKFDRYKFEITVG
MPFSSGADGSRSREEDDVTKDIVLRASSEFKSVLLSLRQGSLIEFSTILEGRLGSKWPVF
ELKAISCLNCMAQLSPTRRHVKIEHDWRSTVHGAVKFAFDFFFFPFLSAA
>sp|O76054|S14L2_HUMAN SEC14-like protein 2 OS=Homo sapiens OX=9606 GN=SEC14L2
PE=1 SV=1
MSGRVGDLSPRQKEALAKFRENVQDVLPALPNPDDYFLLRWLRARSFDLQKSEAMLRKHV
EFRKQKDIDNIISWQPPEVIQQYLSGGMCGYDLDGCPVWYDIIGPLDAKGLLFSASKQDL
LRTKMRECELLLQECAHQTTKLGRKVETITIIYDCEGLGLKHLWKPAVEAYGEFLCMFEE
NYPETLKRLFVVKAPKLFPVAYNLIKPFLSEDTRKKIMVLGANWKEVLLKHISPDQVPVE
YGGTMTDPDGNPKCKSKINYGGDIPRKYYVRDQVKQQYEHSVQISRGSSHQVEYEILFPG
CVLRWQFMSDGADVGFGIFLKTKMGERQRAGEMTEVLPNQRYNSHLVPEDGTLTCSDPGI
YVLRFDNTYSFIHAKKVNFTVEVLLPDKASEEKMKQLGAGTPK
>sp|O94760|DDAH1_HUMAN N(G),N(G)-dimethylarginine dimethylaminohydrolase 1
OS=Homo sapiens OX=9606 GN=DDAH1 PE=1 SV=3
MAGLGHPAAFGRATHAVVRALPESLGQHALRSAKGEEVDVARAERQHQLYVGVLGSKLGL
QVVELPADESLPDCVFVEDVAVVCEETALITRPGAPSRRKEVDMMKEALEKLQLNIVEMK
DENATLDGGDVLFTGREFFVGLSKRTNQRGAEILADTFKDYAVSTVPVADGLHLKSFCSM
AGPNLIAIGSSESAQKALKIMQQMSDHRYDKLTVPDDIAANCIYLNIPNKGHVLLHRTPE
EYPESAKVYEKLKDHMLIPVSMSELEKVDGLLTCCSVLINKKVDS
>sp|O94856|NFASC_HUMAN Neurofascin OS=Homo sapiens OX=9606 GN=NFASC PE=1 SV=4
MARQPPPPWVHAAFLLCLLSLGGAIEIPMDPSIQNELTQPPTITKQSAKDHIVDPRDNIL
IECEAKGNPAPSFHWTRNSRFFNIAKDPRVSMRRRSGTLVIDFRSGGRPEEYEGEYQCFA
RNKFGTALSNRIRLQVSKSPLWPKENLDPVVVQEGAPLTLQCNPPPGLPSPVIFWMSSSM
EPITQDKRVSQGHNGDLYFSNVMLQDMQTDYSCNARFHFTHTIQQKNPFTLKVLTTRGVA

ERTPSFMYPQGTASSQMVLRGMDLLLECIASGVPTPDIAWYKKGGDLPSDKAKFENFNKA
LRITNVSEEDSGEYFCLASNKMGSIRHTISVRVKAAPYWLDEPKNLILAPGEDGRLVCRA
NGNPKPTVQWMVNGEPLQSAPPNPNREVAGDTIIFRDTQISSRAVYQCNTSNEHGYLLAN
AFVSVLDVPPRMLSPRNQLIRVILYNRTRLDCPFFGSPIPTLRWFKNGQGSNLDGGNYHV
YENGSLEIKMIRKEDQGIYTCVATNILGKAENQVRLEVKDPTRIYRMPEDQVARRGTTVQ
LECRVKHDPSLKLTVSWLKDDEPLYIGNRMKKEDDSLTIFGVAERDQGSYTCVASTELDQ
DLAKAYLTVLADQATPTNRLAALPKGRPDRPRDLELTDLAERSVRLTWIPGDANNSPITD
YVVQFEEDQFQPGVWHDHSKYPGSVNSAVLRLSPYVNYQFRVIAINEVGSSHPSLPSERY
RTSGAPPESNPGDVKGEGTRKNNMEITWTPMNATSAFGPNLRYIVKWRRRETREAWNNVT
VWGSRYVVGQTPVYVPYEIRVQAENDFGKGPEPESVIGYSGEDYPRAAPTEVKVRVMNST
AISLQWNRVYSDTVQGQLREYRAYYWRESSLLKNLWVSQKRQQASFPGDRLRGVVSRLFP
YSNYKLEMVVVNGRGDGPRSETKEFTTPEGVPSAPRRFRVRQPNLETINLEWDHPEHPNG
IMIGYTLKYVAFNGTKVGKQIVENFSPNQTKFTVQRTDPVSRYRFTLSARTQVGSGEAVT
EESPAPPNEATPTAAPPTLPPTTVGATGAVSSTDATAIAATTEATTVPIIPTVAPTTIAT
TTTVATTTTTTAAATTTTESPPTTTSGTKIHESAPDEQSIWNVTVLPNSKWANITWKHNF
GPGTDFVVEYIDSNHTKKTVPVKAQAQPIQLTDLYPGMTYTLRVYSRDNEGISSTVITFM
TSTAYTNNQADIATQGWFIGLMCAIALLVLILLIVCFIKRSRGGKYPVREKKDVPLGPED
PKEEDGSFDYSDEDNKPLQGSQTSLDGTIKQQESDDSLVDYGEGGEGQFNEDGSFIGQYT
VKKDKEETEGNESSEATSPVNAIYSLA
>sp|O94905|ERLN2_HUMAN Erlin-2 OS=Homo sapiens OX=9606 GN=ERLIN2 PE=1 SV=1
MAQLGAVVAVASSFFCASLFSAVHKIEEGHIGVYYRGGALLTSTSGPGFHLMLPFITSYK
SVQTTLQTDEVKNVPCGTSGGVMIYFDRIEVVNFLVPNAVYDIVKNYTADYDKALIFNKI
HHELNQFCSVHTLQEVYIELFDQIDENLKLALQQDLTSMAPGLVIQAVRVTKPNIPEAIR
RNYELMESEKTKLLIAAQKQKVVEKEAETERKKALIEAEKVAQVAEITYGQKVMEKETEK
KISEIEDAAFLAREKAKADAECYTAMKIAEANKLKLTPEYLQLMKYKAIASNSKIYFGKD
IPNMFMDSAGSVSKQFEGLADKLSFGLEDEPLETATKEN
>sp|O94973|AP2A2_HUMAN AP-2 complex subunit alpha-2 OS=Homo sapiens OX=9606
GN=AP2A2 PE=1 SV=2
MPAVSKGDGMRGLAVFISDIRNCKSKEAEIKRINKELANIRSKFKGDKALDGYSKKKYVC
KLLFIFLLGHDIDFGHMEAVNLLSSNRYTEKQIGYLFISVLVNSNSELIRLINNAIKNDL
ASRNPTFMGLALHCIASVGSREMAEAFAGEIPKVLVAGDTMDSVKQSAALCLLRLYRTSP
DLVPMGDWTSRVVHLLNDQHLGVVTAATSLITTLAQKNPEEFKTSVSLAVSRLSRIVTSA
STDLQDYTYYFVPAPWLSVKLLRLLQCYPPPDPAVRGRLTECLETILNKAQEPPKSKKVQ
HSNAKNAVLFEAISLIIHHDSEPNLLVRACNQLGQFLQHRETNLRYLALESMCTLASSEF
SHEAVKTHIETVINALKTERDVSVRQRAVDLLYAMCDRSNAPQIVAEMLSYLETADYSIR
EEIVLKVAILAEKYAVDYTWYVDTILNLIRIAGDYVSEEVWYRVIQIVINRDDVQGYAAK
TVFEALQAPACHENLVKVGGYILGEFGNLIAGDPRSSPLIQFHLLHSKFHLCSVPTRALL
LSTYIKFVNLFPEVKPTIQDVLRSDSQLRNADVELQQRAVEYLRLSTVASTDILATVLEE
MPPFPERESSILAKLKKKKGPSTVTDLEDTKRDRSVDVNGGPEPAPASTSAVSTPSPSAD
LLGLGAAPPAPAGPPPSSGGSGLLVDVFSDSASVVAPLAPGSEDNFARFVCKNNGVLFEN
QLLQIGLKSEFRQNLGRMFIFYGNKTSTQFLNFTPTLICSDDLQPNLNLQTKPVDPTVEG
GAQVQQVVNIECVSDFTEAPVLNIQFRYGGTFQNVSVQLPITLNKFFQPTEMASQDFFQR
WKQLSNPQQEVQNIFKAKHPMDTEVTKAKIIGFGSALLEEVDPNPANFVGAGIIHTKTTQ
IGCLLRLEPNLQAQMYRLTLRTSKEAVSQRLCELLSAQF

>sp|O95168|NDUB4_HUMAN NADH dehydrogenase [ubiquinone] 1 beta subcomplex subunit 4 OS=Homo sapiens OX=9606 GN=NDUFB4 PE=1 SV=3
MSFPKYKPSSLRTLPETLDPAEYNISPETRRAQAERLAIRAQLKREYLLQYNDPNRRGLI
ENPALLRWAYARTINVYPNFRPTPKNSLMGALCGFGPLIFIYYIIKTERDRKEKLIQEGK
LDRTFHLSY
>sp|O95336|6PGL_HUMAN 6-phosphogluconolactonase OS=Homo sapiens OX=9606 GN=PGLS PE=1 SV=2
MAAPAPGLISVFSSSQELGAALAQLVAQRAACCLAGARARFALGLSGGSLVSMLARELPA
AVAPAGPASLARWTLGFCDERLVPFDHAESTYGLYRTHLLSRLPIPESQVITINPELPVE
EAAEDYAKKLRQAFQGDSIPVFDLLILGVGPDGHTCSLFPDHPLLQEREKIVAPISDSPK
PPPQRVTLTLPVLNAARTVIFVATGEGKAAVLKRILEDQEENPLPAALVQPHTGKLCWFL
DEAAARLLTVPFEKHSTL
>sp|O95373|IPO7_HUMAN Importin-7 OS=Homo sapiens OX=9606 GN=IPO7 PE=1 SV=1
MDPNTIIEALRGTMDPALREAAERQLNEAHKSLNFVSTLLQITMSEQLDLPVRQAGVIYL
KNMITQYWPDRETAPGDISPYTIPEEDRHCIRENIVEAIIHSPELIRVQLTTCIHHIIKH
DYPSRWTAIVDKIGFYLQSDNSACWLGILLCLYQLVKNYEYKKPEERSPLVAAMQHFLPV
LKDRFIQLLSDQSDQSVLIQKQIFKIFYALVQYTLPLELINQQNLTEWIEILKTVVNRDV
PNETLQVEEDDRPELPWWKCKKWALHILARLFERYGSPGNVSKEYNEFAEVFLKAFAVGV
QQVLLKVLYQYKEKQYMAPRVLQQTLNYINQGVSHALTWKNLKPHIQGIIQDVIFPLMCY
TDADEELWQEDPYEYIRMKFDVFEDFISPTTAAQTLLFTACSKRKEVLQKTMGFCYQILT
EPNADPRKKDGALHMIGSLAEILLKKKIYKDQMEYMLQNHVFPLFSSELGYMRARACWVL
HYFCEVKFKSDQNLQTALELTRRCLIDDREMPVKVEAAIALQVLISNQEKAKEYITPFIR
PVMQALLHIIRETENDDLTNVIQKMICEYSEEVTPIAVEMTQHLAMTFNQVIQTGPDEEG
SDDKAVTAMGILNTIDTLLSVVEDHKEITQQLEGICLQVIGTVLQQHVLEFYEEIFSLAH
SLTCQQVSPQMWQLLPLVFEVFQQDGFDYFTDMMPLLHNYVTVDTDTLLSDTKYLEMIYS
MCKKVLTGVAGEDAECHAAKLLEVIILQCKGRGIDQCIPLFVEAALERLTREVKTSELRT
MCLQVAIAALYYNPHLLLNTLENLRFPNNVEPVTNHFITQWLNDVDCFLGLHDRKMCVLG
LCALIDMEQIPQVLNQVSGQILPAFILLFNGLKRAYACHAEHENDSDDDDEAEDDDETEE
LGSDEDDIDEDGQEYLEILAKQAGEDGDDEDWEEDDAEETALEGYSTIIDDEDNPVDEYQ
IFKAIFQTIQNRNPVWYQALTHGLNEEQRKQLQDIATLADQRRAAHESKMIEKHGGYKFS
APVVPSSFNFGGPAPGMN
>sp|O95428|PPN_HUMAN Papilin OS=Homo sapiens OX=9606 GN=PAPLN PE=2 SV=4
MRLLLLVPLLLAPAPGSSAPKVRRQSDTWGPWSQWSPCSRTCGGGVSFRERPCYSQRRDG
GSSCVGPARSHRSCRTESCPDGARDFRAEQCAEFDGAEFQGRRYRWLPYYSAPNKCELNC
IPKGENFYYKHREAVVDGTPCEPGKRDVCVDGSCRVVGCDHELDSSKQEDKCLRCGGDGT
TCYPVAGTFDANDLSRGYNQILIVPMGATSILIDEAAASRNFLAVKNVRGEYYLNGHWTI
EAARALPAASTILHYERGAEGDLAPERLHARGPTSEPLVIELISQEPNPGVHYEYHLPLR
RPSPGFSWSHGSWSDCSAECGGGHQSRLVFCTIDHEAYPDHMCQRQPRPADRRSCNLHPC
PETKRWKAGPWAPCSASCGGGSQSRSVYCISSDGAGIQEAVEEAECAGLPGKPPAIQACN
LQRCAAWSPEPWGECSVSCGVGVRKRSVTCRGERGSLLHTAACSLEDRPPLTEPCVHEDC
PLLSDQAWHVGTWGLCSKSCSSGTRRRQVICAIGPPSHCGSLQHSKPVDVEPCNTQPCHL
PQEVPSMQDVHTPASNPWMPLGPQESPASDSRGQWWAAQEHPSARGDHRGERGDPRGDQG
THLSALGPAPSLQQPPYQQPLRSGSGPHDCRHSPHGCCPDGHTASLGPQWQGCPGAPCQQ
SRYGCCPDRVSVAEGPHHAGCTKSYGGDSTGGMPRSRAVASTVHNTHQPQAQQNEPSECR

GSQFGCCYDNVATAAGPLGEGCVGQPSHAYPVRCLLPSAHGSCADWAARWYFVASVGQCN
RFWYGGCHGNANNFASEQECMSSCQGSLHGPRRPQPGASGRSTHTDGGGSSPAGEQEPSQ
HRTGAAVQRKPWPSGGLWRQDQQPGPGEAPHTQAFGEWPWGQELGSRAPGLGGDAGSPAP
PFHSSSYRISLAGVEPSLVQAALGQLVRLSCSDDTAPESQAAWQKDGQPISSDRHRLQFD
GSLIIHPLQAEDAGTYSCGSTRPGRDSQKIQLRIIGGDMAVLSEAELSRFPQPRDPAQDF
GQAGAAGPLGAIPSSHPQPANRLRLDQNQPRVVDASPGQRIRMTCRAEGFPPPAIEWQRD
GQPVSSPRHQLQPDGSLVISRVAVEDGGFYTCVAFNGQDRDQRWVQLRVLGELTISGLPP
TVTVPEGDTARLLCVVAGESVNIRWSRNGLPVQADGHRVHQSPDGTLLIYNLRARDEGSY
TCSAYQGSQAVSRSTEVKVVSPAPTAQPRDPGRDCVDQPELANCDLILQAQLCGNEYYSS
FCCASCSRFQPHAQPIWQ
>sp|O95433|AHSA1_HUMAN Activator of 90 kDa heat shock protein ATPase homolog 1
OS=Homo sapiens OX=9606 GN=AHSA1 PE=1 SV=1
MAKWGEGDPRWIVEERADATNVNNWHWTERDASNWSTDKLKTLFLAVQVQNEEGKCEVTE
VSKLDGEASINNRKGKLIFFYEWSVKLNWTGTSKSGVQYKGHVEIPNLSDENSVDEVEIS
VSLAKDEPDTNLVALMKEEGVKLLREAMGIYISTLKTEFTQGMILPTMNGESVDPVGQPA
LKTEERKAKPAPSKTQARPVGVKIPTCKITLKETFLTSPEELYRVFTTQELVQAFTHAPA
TLEADRGGKFHMVDGNVSGEFTDLVPEKHIVMKWRFKSWPEGHFATITLTFIDKNGETEL
CMEGRGIPAPEEERTRQGWQRYYFEGIKQTFGYGARLF
>sp|O95490|AGRL2_HUMAN Adhesion G protein-coupled receptor L2 OS=Homo sapiens
OX=9606 GN=ADGRL2 PE=1 SV=2
MVSSGCRMRSLWFIIVISFLPNTEGFSRAALPFGLVRRELSCEGYSIDLRCPGSDVIMIE
SANYGRTDDKICDADPFQMENTDCYLPDAFKIMTQRCNNRTQCIVVTGSDVFPDPCPGTY
KYLEVQYECVPYIFVCPGTLKAIVDSPCIYEAEQKAGAWCKDPLQAADKIYFMPWTPYRT
DTLIEYASLEDFQNSRQTTTYKLPNRVDGTGFVVYDGAVFFNKERTRNIVKFDLRTRIKS
GEAIINYANYHDTSPYRWGGKTDIDLAVDENGLWVIYATEQNNGMIVISQLNPYTLRFEA
TWETVYDKRAASNAFMICGVLYVVRSVYQDNESETGKNSIDYIYNTRLNRGEYVDVPFPN
QYQYIAAVDYNPRDNQLYVWNNNFILRYSLEFGPPDPAQVPTTAVTITSSAELFKTIIST
TSTTSQKGPMSTTVAGSQEGSKGTKPPPAVSTTKIPPITNIFPLPERFCEALDSKGIKWP
QTQRGMMVERPCPKGTRGTASYLCMISTGTWNPKGPDLSNCTSHWVNQLAQKIRSGENAA
SLANELAKHTKGPVFAGDVSSSVRLMEQLVDILDAQLQELKPSEKDSAGRSYNKLQKREK
TCRAYLKAIVDTVDNLLRPEALESWKHMNSSEQAHTATMLLDTLEEGAFVLADNLLEPTR
VSMPTENIVLEVAVLSTEGQIQDFKFPLGIKGAGSSIQLSANTVKQNSRNGLAKLVFIIY
RSLGQFLSTENATIKLGADFIGRNSTIAVNSHVISVSINKESSRVYLTDPVLFTLPHIDP
DNYFNANCSFWNYSERTMMGYWSTQGCKLVDTNKTRTTCACSHLTNFAILMAHREIAYKD
GVHELLLTVITWVGIVISLVCLAICIFTFCFFRGLQSDRNTIHKNLCINLFIAEFIFLIG
IDKTKYAIACPIFAGLLHFFFLAAFAWMCLEGVQLYLMLVEVFESEYSRKKYYYVAGYLF
PATVVGVSAAIDYKSYGTEKACWLHVDNYFIWSFIGPVTFIILLNIIFLVITLCKMVKHS
NTLKPDSSRLENIKSWVLGAFALLCLLGLTWSFGLLFINEETIVMAYLFTIFNAFQGVFI
FIFHCALQKKVRKEYGKCFRHSYCCGGLPTESPHSSVKASTTRTSARYSSGTQSRIRRMW
NDTVRKQSESSFISGDINSTSTLNQGMTGNYLLTNPLLRPHGTNNPYNTLLAETVVCNAP
SAPVFNSPGHSLNNARDTSAMDTLPLNGNFNNSYSLHKGDYNDSVQVVDCGLSLNDTAFE
KMIISELVHNNLRGSSKTHNLELTLPVKPVIGGSSSEDDAIVADASSLMHSDNPGLELHH
KELEAPLIPQRTHSLLYQPQKKVKSEGTDSYVSQLTAEAEDHLQSPNRDSLYTSMPNLRD
SPYPESSPDMEEDLSPSRRSENEDIYYKSMPNLGAGHQLQMCYQISRGNSDGYIIPINKE

GCIPEGDVREGQMQLVTSL
>sp|O95563|MPC2_HUMAN Mitochondrial pyruvate carrier 2 OS=Homo sapiens OX=9606 GN=MPC2 PE=1 SV=1
MSAAGARGLRATYHRLLDKVELMLPEKLRPLYNHPAGPRTVFFWAPIMKWGLVCAGLADM
ARPAEKLSTAQSAVLMATGFIWSRYSLVIIPKNWSLFAVNFFVGAAGASQLFRIWRYNQE
LKAKAHK
>sp|O95573|ACSL3_HUMAN Fatty acid CoA ligase Acsl3 OS=Homo sapiens OX=9606 GN=ACSL3 PE=1 SV=3
MNNHVSSKPSTMKLKHTINPILLYFIHFLISLYTILTYIPFYFFSESRQEKSNRIKAKPV
NSKPDSAYRSVNSLDGLASVLYPGCDTLDKVFTYAKNKFKNKRLLGTREVLNEEDEVQPN
GKIFKKVILGQYNWLSYEDVFVRAFNFGNGLQMLGQKPKTNIAIFCETRAEWMIAAQACF
MYNFQLVTLYATLGGPAIVHALNETEVTNIITSKELLQTKLKDIVSLVPRLRHIITVDGK
PPTWSEFPKGIIVHTMAAVEALGAKASMENQPHSKPLPSDIAVIMYTSGSTGLPKGVMIS
HSNIIAGITGMAERIPELGEEDVYIGYLPLAHVLELSAELVCLSHGCRIGYSSPQTLADQ
SSKIKKGSKGDTSMLKPTLMAAVPEIMDRIYKNVMNKVSEMSSFQRNLFILAYNYKMEQI
SKGRNTPLCDSFVFRKVRSLLGGNIRLLLCGGAPLSATTQRFMNICFCCPVGQGYGLTES
AGAGTISEVWDYNTGRVGAPLVCCEIKLKNWEEGGYFNTDKPHPRGEILIGGQSVTMGYY
KNEAKTKADFFEDENGQRWLCTGDIGEFEPDGCLKIIDRKKDLVKLQAGEYVSLGKVEAA
LKNLPLVDNICAYANSYHSYVIGFVVPNQKELTELARKKGLKGTWEELCNSCEMENEVLK
VLSEAAISASLEKFEIPVKIRLSPEPWTPETGLVTDAFKLKRKELKTHYQADIERMYGRK
>sp|O95671|ASML_HUMAN Probable bifunctional dTTP/UTP pyrophosphatase/methyltransferase protein OS=Homo sapiens OX=9606 GN=ASMTL PE=1 SV=3
MVLCPVIGKLLHKRVVLASASPRRQEILSNAGLRFEVVPSKFKEKLDKASFATPYGYAME
TAKQKALEVANRLYQKDLRAPDVVIGADTIVTVGGLILEKPVDKQDAYRMLSRLSGREHS
VFTGVAIVHCSSKDHQLDTRVSEFYEETKVKFSELSEELLWEYVHSGEPMDKAGGYGIQA
LGGMLVESVHGDFLNVVGFPLNHFCKQLVKLYYPPRPEDLRRSVKHDSIPAADTFEDLSD
VEGGGSEPTQRDAGSRDEKAEAGEAGQATAEAECHRTRETLPPFPTRLLELIEGFMLSKG
LLTACKLKVFDLLKDEAPQKAADIASKVDASACGMERLLDICAAMGLLEKTEQGYSNTET
ANVYLASDGEYSLHGFIMHNNDLTWNLFTYLEFAIREGTNQHHRALGKKAEDLFQDAYYQ
SPETRLRFMRAMHGMTKLTACQVATAFNLSRFSSACDVGGCTGALARELAREYPRMQVTV
FDLPDIIELAAHFQPPGPQAVQIHFAAGDFFRDPLPSAELYVLCRILHDWPDDKVHKLLS
RVAESCKPGAGLLLVETLLDEEKRVAQRALMQSLNMLVQTEGKERSLGEYQCLLELHGFH
QVQVVHLGGVLDAILATKVAP
>sp|O95715|CXL14_HUMAN C-X-C motif chemokine 14 OS=Homo sapiens OX=9606 GN=CXCL14 PE=1 SV=2
MSLLPRRAPPVSMRLLAAALLLLLLALYTARVDGSKCKCSRKGPKIRYSDVKKLEMKPKY
PHCEEKMVIITTKSVSRYRGQEHCLHPKLQSTKRFIKWYNAWNEKRRVYEE
>sp|O95834|EMAL2_HUMAN Echinoderm microtubule-associated protein-like 2 OS=Homo sapiens OX=9606 GN=EML2 PE=1 SV=1
MSSFGAGKTKEVIFSVEDGSVKMFLRGRPVPMMIPDELAPTYSLDTRSELPSCRLKLEWV
YGYRGRDCRANLYLLPTGEIVYFVASVAVLYSVEEQRQRHYLGHNDDIKCLAIHPDMVTI
ATGQVAGTTKEGKPLPPHVRIWDSVSLSTLHVLGLGVFDRAVCCVGFSKSNGGNLLCAVD
ESNDHMLSVWDWAKETKVVDVKCSNEAVLVATFHPTDPTVLITCGKSHIYFWTLEGGSLS
KRQGLFEKHEKPKYVLCVTFLEGGDVVTGDSGGNLYVWGKGGNRITQAVLGAHDGGVFGL

CALRDGTLVSGGGRDRRVVLWGSDYSKLQEVEVPEDFGPVRTVAEGHGDTLYVGTTRNSI
LQGSVHTGFSLLVQGHVEELWGLATHPSRAQFVTCGQDKLVHLWSSDSHQPLWSRIIEDP
ARSAGFHPSGSVLAVGTVTGRWLLLDTETHDLVAIHTDGNEQISVVSFSPDGAYLAVGSH
DNLVYVYTVDQGGRKVSRLGKCSGHSSFITHLDWAQDSSCFVTNSGDYEILYWDPATCKQ
ITSADAVRNMEWATATCVLGFGVFGIWSEGADGTDINAVARSHDGKLLASADDFGKVHLF
SYPCCQPRALSHKYGGHSSHVTNVAFLWDDSMALTTGGKDTSVLQWRVV
>sp|O95861|BPNT1_HUMAN 3'(2'),5'-bisphosphate nucleotidase 1 OS=Homo sapiens OX=9606
GN=BPNT1 PE=1 SV=1
MASSNTVLMRLVASAYSIAQKAGMIVRRVIAEGDLGIVEKTCATDLQTKADRLAQMSICS
SLARKFPKLTIIGEEDLPSEEVDQELIEDSQWEEILKQPCPSQYSAIKEEDLVVWVDPLD
GTKEYTEGLLDNVTVLIGIAYEGKAIAGVINQPYYNYEAGPDAVLGRTIWGVLGLGAFGF
QLKEVPAGKHIITTTRSHSNKLVTDCVAAMNPDAVLRVGGAGNKIIQLIEGKASAYVFAS
PGCKKWDTCAPEVILHAVGGKLTDIHGNVLQYHKDVKHMNSAGVLATLRNYDYYASRVPE
SIKNALVP
>sp|P00338|LDHA_HUMAN L-lactate dehydrogenase A chain OS=Homo sapiens OX=9606
GN=LDHA PE=1 SV=2
MATLKDQLIYNLLKEEQTPQNKITVVGVGAVGMACAISILMKDLADELALVDVIEDKLKG
EMMDLQHGSLFLRTPKIVSGKDYNVTANSKLVIITAGARQQEGESRLNLVQRNVNIFKFI
IPNVVKYSPNCKLLIVSNPVDILTYVAWKISGFPKNRVIGSGCNLDSARFRYLMGERLGV
HPLSCHGWVLGEHGDSSVPVWSGMNVAGVSLKTLHPDLGTDKDKEQWKEVHKQVVESAYE
VIKLKGYTSWAIGLSVADLAESIMKNLRRVHPVSTMIKGLYGIKDDVFLSVPCILGQNGI
SDLVKVTLTSEEEARLKKSADTLWGIQKELQF
>sp|P00352|AL1A1_HUMAN Aldehyde dehydrogenase 1A1 OS=Homo sapiens OX=9606
GN=ALDH1A1 PE=1 SV=2
MSSSGTPDLPVLLTDLKIQYTKIFINNEWHDSVSGKKFPVFNPATEEELCQVEEGDKEDV
DKAVKAARQAFQIGSPWRTMDASERGRLLYKLADLIERDRLLLATMESMNGGKLYSNAYL
NDLAGCIKTLRYCAGWADKIQGRTIPIDGNFFTYTRHEPIGVCGQIIPWNFPLVMLIWKI
GPALSCGNTVVVKPAEQTPLTALHVASLIKEAGFPPGVVNIVPGYGPTAGAAISSHMDID
KVAFTGSTEVGKLIKEAAGKSNLKRVTLELGGKSPCIVLADADLDNAVEFAHHGVFYHQG
QCCIAASRIFVEESIYDEFVRRSVERAKKYILGNPLTPGVTQGPQIDKEQYDKILDLIES
GKKEGAKLECGGGPWGNKGYFVQPTVFSNVTDEMRIAKEEIFGPVQQIMKFKSLDDVIKR
ANNTFYGLSAGVFTKDIDKAITISSALQAGTVWVNCYGVVSAQCPFGGFKMSGNGRELGE
YGFHEYTEVKTVTVKISQKNS
>sp|P00387|NB5R3_HUMAN NADH-cytochrome b5 reductase 3 OS=Homo sapiens OX=9606
GN=CYB5R3 PE=1 SV=3
MGAQLSTLGHMVLFPVWFLYSLLMKLFQRSTPAITLESPDIKYPLRLIDREIISHDTRRF
RFALPSPQHILGLPVGQHIYLSARIDGNLVVRPYTPISSDDDKGFVDLVIKVYFKDTHPK
FPAGGKMSQYLESMQIGDTIEFRGPSGLLVYQGKGKFAIRPDKKSNPIIRTVKSVGMIAG
GTGITPMLQVIRAIMKDPDDHTVCHLLFANQTEKDILLRPELEELRNKHSARFKLWYTLD
RAPEAWDYGQGFVNEEMIRDHLPPPEEEPLVLMCGPPPMIQYACLPNLDHVGHPTERCFV
F
>sp|P00390|GSHR_HUMAN Glutathione reductase, mitochondrial OS=Homo sapiens OX=9606
GN=GSR PE=1 SV=2
MALLPRALSAGAGPSWRRAARAFRGFLLLLPEPAALTRALSRAMACRQEPQPQGPPPAAG

AVASYDYLVIGGGSGGLASARRAAELGARAAVVESHKLGGTCVNVGCVPKKVMWNTAVHS
EFMHDHADYGFPSCEGKFNWRVIKEKRDAYVSRLNAIYQNNLTKSHIEIIRGHAAFTSDP
KPTIEVSGKKYTAPHILIATGGMPSTPHESQIPGASLGITSDGFFQLEELPGRSVIVGAG
YIAVEMAGILSALGSKTSLMIRHDKVLRSFDSMISTNCTEELENAGVEVLKFSQVKEVKK
TLSGLEVSMVTAVPGRLPVMTMIPDVDCLLWAIGRVPNTKDLSLNKLGIQTDDKGHIIVD
EFQNTNVKGIYAVGDVCGKALLTPVAIAAGRKLAHRLFEYKEDSKLDYNNIPTVVFSHPP
IGTVGLTEDEAIHKYGIENVKTYSTSFTPMYHAVTKRKTKCVMKMVCANKEEKVVGIHMQ
GLGCDEMLQGFAVAVKMGATKADFDNTVAIHPTSSEELVTLR
>sp|P00403|COX2_HUMAN Cytochrome c oxidase subunit 2 OS=Homo sapiens OX=9606
GN=MT-CO2 PE=1 SV=1
MAHAAQVGLQDATSPIMEELITFHDHALMIIFLICFLVLYALFLTLTTKLTNTNISDAQE
METVWTILPAIILVLIALPSLRILYMTDEVNDPSLTIKSIGHQWYWTYEYTDYGGLIFNS
YMLPPLFLEPGDLRLLDVDNRVVLPIEAPIRMMITSQDVLHSWAVPTLGLKTDAIPGRLN
QTTFTATRPGVYYGQCSEICGANHSFMPIVLELIPLKIFEMGPVFTL
>sp|P00441|SODC_HUMAN Superoxide dismutase [Cu-Zn] OS=Homo sapiens OX=9606
GN=SOD1 PE=1 SV=2
MATKAVCVLKGDGPVQGIINFEQKESNGPVKVWGSIKGLTEGLHGFHVHEFGDNTAGCTS
AGPHFNPLSRKHGGPKDEERHVGDLGNVTADKDGVADVSIEDSVISLSGDHCIIGRTLVV
HEKADDLGKGGNEESTKTGNAGSRLACGVIGIAQ
>sp|P00450|CERU_HUMAN Ceruloplasmin OS=Homo sapiens OX=9606 GN=CP PE=1 SV=2
MKILILGIFLFLCSTPAWAKEKHYYIGIIETTWDYASDHGEKKLISVDTEHSNIYLQNGP
DRIGRLYKKALYLQYTDETFRTTIEKPVWLGFLGPIIKAETGDKVYVHLKNLASRPYTFH
SHGITYYKEHEGAIYPDNTTDFQRADDKVYPGEQYTYMLLATEEQSPGEGDGNCVTRIYH
SHIDAPKDIASGLIGPLIICKKDSLDKEKEKHIDREFVVMFSVVDENFSWYLEDNIKTYC
SEPEKVDKDNEDFQESNRMYSVNGYTFGSLPGLSMCAEDRVKWYLFGMGNEVDVHAAFFH
GQALTNKNYRIDTINLFPATLFDAYMVAQNPGEWMLSCQNLNHLKAGLQAFFQVQECNKS
SSKDNIRGKHVRHYYIAAEEIIWNYAPSGIDIFTKENLTAPGSDSAVFFEQGTTRIGGSY
KKLVYREYTDASFTNRKERGPEEEHLGILGPVIWAEVGDTIRVTFHNKGAYPLSIEPIGV
RFNKNNEGTYYSPNYNPQSRSVPPSASHVAPTETFTYEWTVPKEVGPTNADPVCLAKMYY
SAVEPTKDIFTGLIGPMKICKKGSLHANGRQKDVDKEFYLFPTVFDENESLLLEDNIRMF
TTAPDQVDKEDEDFQESNKMHSMNGFMYGNQPGLTMCKGDSVVWYLFSAGNEADVHGIYF
SGNTYLWRGERRDTANLFPQTSLTLHMWPDTEGTFNVECLTTDHYTGGMKQKYTVNQCRR
QSEDSTFYLGERTYYIAAVEVEWDYSPQREWEKELHHLQEQNVSNAFLDKGEFYIGSKYK
KVVYRQYTDSTFRVPVERKAEEEHLGILGPQLHADVGDKVKIIFKNMATRPYSIHAHGVQ
TESSTVTPTLPGETLTYVWKIPERSGAGTEDSACIPWAYYSTVDQVKDLYSGLIGPLIVC
RRPYLKVFNPRRKLEFALLFLVFDENESWYLDDNIKTYSDHPEKVNKDDEEFIESNKMHA
INGRMFGNLQGLTMHVGDEVNWYLMGMGNEIDLHTVHFHGHSFQYKHRGVYSSDVFDIFP
GTYQTLEMFPRTPGIWLLHCHVTDHIHAGMETTYTVLQNEDTKSG
>sp|P00491|PNPH_HUMAN Purine nucleoside phosphorylase OS=Homo sapiens OX=9606
GN=PNP PE=1 SV=2
MENGYTYEDYKNTAEWLLSHTKHRPQVAIICGSGLGGLTDKLTQAQIFDYGEIPNFPRST
VPGHAGRLVFGFLNGRACVMMQGRFHMYEGYPLWKVTFPVRVFHLLGVDTLVVTNAAGGL
NPKFEVGDIMLIRDHINLPGFSGQNPLRGPNDERFGDRFPAMSDAYDRTMRQRALSTWKQ
MGEQRELQEGTYVMVAGPSFETVAECRVLQKLGADAVGMSTVPEVIVARHCGLRVFGFSL

ITNKVIMDYESLEKANHEEVLAAGKQAAQKLEQFVSILMASIPLPDKAS
>sp|P00505|AATM_HUMAN Aspartate aminotransferase, mitochondrial OS=Homo sapiens OX=9606 GN=GOT2 PE=1 SV=3
MALLHSGRVLPGIAAAFHPGLAAAASARASSWWTHVEMGPPDPILGVTEAFKRDTNSKKM
NLGVGAYRDDNGKPYVLPSVRKAEAQIAAKNLDKEYLPIGGLAEFCKASAELALGENSEV
LKSGRFVTVQTISGTGALRIGASFLQRFFKFSRDVFLPKPTWGNHTPIFRDAGMQLQGYR
YYDPKTCGFDFTGAVEDISKIPEQSVLLLHACAHNPTGVDPRPEQWKEIATVVKKRNLFA
FFDMAYQGFASGDGDKDAWAVRHFIEQGINVCLCQSYAKNMGLYGERVGAFTMVCKDADE
AKRVESQLKILIRPMYSNPPLNGARIAAAILNTPDLRKQWLQEVKVMADRIIGMRTQLVS
NLKKEGSTHNWQHITDQIGMFCFTGLKPEQVERLIKEFSIYMTKDGRISVAGVTSSNVGY
LAHAIHQVTK
>sp|P00533|EGFR_HUMAN Epidermal growth factor receptor OS=Homo sapiens OX=9606 GN=EGFR PE=1 SV=2
MRPSGTAGAALLALLAALCPASRALEEKKVCQGTSNKLTQLGTFEDHFLSLQRMFNNCEV
VLGNLEITYVQRNYDLSFLKTIQEVAGYVLIALNTVERIPLENLQIIRGNMYYENSYALA
VLSNYDANKTGLKELPMRNLQEILHGAVRFSNNPALCNVESIQWRDIVSSDFLSNMSMDF
QNHLGSCQKCDPSCPNGSCWGAGEENCQKLTKIICAQQCSGRCRGKSPSDCCHNQCAAGC
TGPRESDCLVCRKFRDEATCKDTCPPLMLYNPTTYQMDVNPEGKYSFGATCVKKCPRNYV
VTDHGSCVRACGADSYEMEEDGVRKCKKCEGPCRKVCNGIGIGEFKDSLSINATNIKHFK
NCTSISGDLHILPVAFRGDSFTHTPPLDPQELDILKTVKEITGFLLIQAWPENRTDLHAF
ENLEIIRGRTKQHGQFSLAVVSLNITSLGLRSLKEISDGDVIISGNKNLCYANTINWKKL
FGTSGQKTKIISNRGENSCKATGQVCHALCSPEGCWGPEPRDCVSCRNVSRGRECVDKCN
LLEGEPREFVENSECIQCHPECLPQAMNITCTGRGPDNCIQCAHYIDGPHCVKTCPAGVM
GENNTLVWKYADAGHVCHLCHPNCTYGCTGPGLEGCPTNGPKIPSIATGMVGALLLLLVV
ALGIGLFMRRRHIVRKRTLRRLLQERELVEPLTPSGEAPNQALLRILKETEFKKIKVLGS
GAFGTVYKGLWIPEGEKVKIPVAIKELREATSPKANKEILDEAYVMASVDNPHVCRLLGI
CLTSTVQLITQLMPFGCLLDYVREHKDNIGSQYLLNWCVQIAKGMNYLEDRRLVHRDLAA
RNVLVKTPQHVKITDFGLAKLLGAEEKEYHAEGGKVPIKWMALESILHRIYTHQSDVWSY
GVTVWELMTFGSKPYDGIPASEISSILEKGERLPQPPICTIDVYMIMVKCWMIDADSRPK
FRELIIEFSKMARDPQRYLVIQGDERMHLPSPTDSNFYRALMDEEDMDDVVDADEYLIPQ
QGFFSSPSTSRTPLLSSLSATSNNSTVACIDRNGLQSCPIKEDSFLQRYSSDPTGALTED
SIDDTFLPVPEYINQSVPKRPAGSVQNPVYHNQPLNPAPSRDPHYQDPHSTAVGNPEYLN
TVQPTCVNSTFDSPAHWAQKGSHQISLDNPDYQQDFFPKEAKPNGIFKGSTAENAEYLRV
APQSSEFIGA
>sp|P00558|PGK1_HUMAN Phosphoglycerate kinase 1 OS=Homo sapiens OX=9606 GN=PGK1 PE=1 SV=3
MSLSNKLTLDKLDVKGKRVVMRVDFNVPMKNNQITNNQRIKAAVPSIKFCLDNGAKSVVL
MSHLGRPDGVPMPDKYSLEPVAVELKSLLGKDVLFLKDCVGPEVEKACANPAAGSVILLE
NLRFHVEEEGKGKDASGNKVKAEPAKIEAFRASLSKLGDVYVNDAFGTAHRAHSSMVGVN
LPQKAGGFLMKKELNYFAKALESPERPFLAILGGAKVADKIQLINNMLDKVNEMIIGGGM
AFTFLKVLNNMEIGTSLFDEEGAKIVKDLMSKAEKNGVKITLPVDFVTADKFDENAKTGQ
ATVASGIPAGWMGLDCGPESSKKYAEAVTRAKQIVWNGPVGVFEWEAFARGTKALMDEVV
KATSRGCITIIGGGDTATCCAKWNTEDKVSHVSTGGGASLELLEGKVLPGVDALSNI

>sp|P00918|CAH2_HUMAN Carbonic anhydrase 2 OS=Homo sapiens OX=9606 GN=CA2 PE=1 SV=2
MSHHWGYGKHNGPEHWHKDFPIAKGERQSPVDIDTHTAKYDPSLKPLSVSYDQATSLRIL
NNGHAFNVEFDDSQDKAVLKGGPLDGTYRLIQFHFHWGSLDGQGSEHTVDKKKYAAELHL
VHWNTKYGDFGKAVQQPDGLAVLGIFLKVGSAKPGLQKVVDVLDSIKTKGKSADFTNFDP
RGLLPESLDYWTYPGSLTTPPLLECVTWIVLKEPISVSSEQVLKFRKLNFNGEGEPEELM
VDNWRPAQPLKNRQIKASFK
>sp|P01024|CO3_HUMAN Complement C3 OS=Homo sapiens OX=9606 GN=C3 PE=1 SV=2
MGPTSGPSLLLLLLTHLPLALGSPMYSIITPNILRLESEETMVLEAHDAQGDVPVTVTVH
DFPGKKLVLSSEKTVLTPATNHMGNVTFTIPANREFKSEKGRNKFVTVQATFGTQVVEKV
VLVSLQSGYLFIQTDKTIYTPGSTVLYRIFTVNHKLLPVGRTVMVNIENPEGIPVKQDSL
SSQNQLGVLPLSWDIPELVNMGQWKIRAYYENSPQQVFSTEFEVKEYVLPSFEVIVEPTE
KFYYIYNEKGLEVTITARFLYGKKVEGTAFVIFGIQDGEQRISLPESLKRIPIEDGSGEV
VLSRKVLLDGVQNPRAEDLVGKSLYVSATVILHSGSDMVQAERSGIPIVTSPYQIHFTKT
PKYFKPGMPFDLMVFVTNPDGSPAYRVPVAVQGEDTVQSLTQGDGVAKLSINTHPSQKPL
SITVRTKKQELSEAEQATRTMQALPYSTVGNSNNYLHLSVLRTELRPGETLNVNFLLRMD
RAHEAKIRYYTYLIMNKGRLLKAGRQVREPGQDLVVLPLSITTDFIPSFRLVAYYTLIGA
SGQREVVADSVWVDVKDSCVGSLVVKSGQSEDRQPVPGQQMTLKIEGDHGARVVLVAVDK
GVFVLNKKNKLTQSKIWDVVEKADIGCTPGSGKDYAGVFSDAGLTFTSSSGQQTAQRAEL
QCPQPAARRRRSVQLTEKRMDKVGKYPKELRKCCEDGMRENPMRFSCQRRTRFISLGEAC
KKVFLDCCNYITELRRQHARASHLGLARSNLDEDIIAEENIVSRSEFPESWLWNVEDLKE
PPKNGISTKLMNIFLKDSITTWEILAVSMSDKKGICVADPFEVTVMQDFFIDLRLPYSVV
RNEQVEIRAVLYNYRQNQELKVRVELLHNPAFCSLATTKRRHQQTVTIPPKSSLSVPYVI
VPLKTGLQEVEVKAAVYHHFISDGVRKSLKVVPEGIRMNKTVAVRTLDPERLGREGVQKE
DIPPADLSDQVPDTESETRILLQGTPVAQMTEDAVDAERLKHLIVTPSGCGEQNMIGMTP
TVIAVHYLDETEQWEKFGLEKRQGALELIKKGYTQQLAFRQPSSAFAAFVKRAPSTWLTA
YVVKVFSLAVNLIAIDSQVLCGAVKWLILEKQKPDGVFQEDAPVIHQEMIGGLRNNNEKD
MALTAFVLISLQEAKDICEEQVNSLPGSITKAGDFLEANYMNLQRSYTVAIAGYALAQMG
RLKGPLLNKFLTTAKDKNRWEDPGKQLYNVEATSYALLALLQLKDFDFVPPVVRWLNEQR
YYGGGYGSTQATFMVFQALAQYQKDAPDHQELNLDVSLQLPSRSSKITHRIHWESASLLR
SEETKENEGFTVTAEGKGQGTLSVVTMYHAKAKDQLTCNKFDLKVTIKPAPETEKRPQDA
KNTMILEICTRYRGDQDATMSILDISMMTGFAPDTDDLKQLANGVDRYISKYELDKAFSD
RNTLIIYLDKVSHSEDDCLAFKVHQYFNVELIQPGAVKVYAYYNLEESCTRFYHPEKEDG
KLNKLCRDELCRCAEENCFIQKSDDKVTLEERLDKACEPGVDYVYKTRLVKVQLSNDFDE
YIMAIEQTIKSGSDEVQVGQQRTFISPIKCREALKLEEKKHYLMWGLSSDFWGEKPNLSY
IIGKDTWVEHWPEEDECQDEENQKQCQDLGAFTESMVVFGCPN
>sp|P01034|CYTC_HUMAN Cystatin-C OS=Homo sapiens OX=9606 GN=CST3 PE=1 SV=1
MAGPLRAPLLLLAILAVALAVSPAAGSSPGKPPRLVGGPMDASVEEEGVRRALDFAVGEY
NKASNDMYHSRALQVVRARKQIVAGVNYFLDVELGRTTCTKTQPNLDNCPFHDQPHLKRK
AFCSFQIYAVPWQGTMTLSKSTCQDA
>sp|P01112|RASH_HUMAN GTPase HRas OS=Homo sapiens OX=9606 GN=HRAS PE=1 SV=1
MTEYKLVVVGAGGVGKSALTIQLIQNHFVDEYDPTIEDSYRKQVVIDGETCLLDILDTAG
QEEYSAMRDQYMRTGEGFLCVFAINNTKSFEDIHQYREQIKRVKDSDDVPMVLVGNKCDL
AARTVESRQAQDLARSYGIPYIETSAKTRQGVEDAFYTLVREIRQHKLRKLNPPDESGPG

CMSCKCVLS
>sp|P01116|RASK_HUMAN GTPase KRas OS=Homo sapiens OX=9606 GN=KRAS PE=1 SV=1
MTEYKLVVVGAGGVGKSALTIQLIQNHFVDEYDPTIEDSYRKQVVIDGETCLLDILDTAG
QEEYSAMRDQYMRTGEGFLCVFAINNTKSFEDIHHYREQIKRVKDSEDVPMVLVGNKCDL
PSRTVDTKQAQDLARSYGIPFIETSAKTRQRVEDAFYTLVREIRQYRLKKISKEEKTPGC
VKIKKCIIM
>sp|P01834|IGKC_HUMAN Immunoglobulin kappa constant OS=Homo sapiens OX=9606
GN=IGKC PE=1 SV=2
RTVAAPSVFIFPPSDEQLKSGTASVVCLLNNFYPREAKVQWKVDNALQSGNSQESVTEQD
SKDSTYSLSSTLTLSKADYEKHKVYACEVTHQGLSSPVTKSFNRGEC
>sp|P01857|IGHG1_HUMAN Immunoglobulin heavy constant gamma 1 OS=Homo sapiens
OX=9606 GN=IGHG1 PE=1 SV=2
ASTKGPSVFPLAPSSKSTSGGTAALGCLVKDYFPEPVTVSWNSGALTSGVHTFPAVLQSS
GLYSLSSVVTVPSSSLGTQTYICNVNHKPSNTKVDKKVEPKSCDKTHTCPPCPAPELLGG
PSVFLFPPKPKDTLMISRTPEVTCVVVDVSHEDPEVKFNWYVDGVEVHNAKTKPREEQYN
STYRVVSVLTVLHQDWLNGKEYKCKVSNKALPAPIEKTISKAKGQPREPQVYTLPPSRDE
LTKNQVSLTCLVKGFYPSDIAVEWESNGQPENNYKTTPPVLDSDGSFFLYSKLTVDKSRW
QQGNVFSCSVMHEALHNHYTQKSLSLSPELQLEESCAEAQDGELDGLWTTITIFITLFLL
SVCYSATVTFFKVKWIFSSVVDLKQTIIPDYRNMIGQGA
>sp|P02458|CO2A1_HUMAN Collagen alpha-1(II) chain OS=Homo sapiens OX=9606
GN=COL2A1 PE=1 SV=3
MIRLGAPQTLVLLTLLVAAVLRCQGQDVQEAGSCVQDGQRYNDKDVWKPEPCRICVCDTG
TVLCDDIICEDVKDCLSPEIPFGECCPICPTDLATASGQPGPKGQKGEPGDIKDIVGPKG
PPGPQGPAGEQGPRGDRGDKGEKGAPGPRGRDGEPGTPGNPGPPGPPGPPGPPGLGGNFA
AQMAGGFDEKAGGAQLGVMQGPMGPMGPRGPPGPAGAPGPQGFQGNPGEPGEPGVSGPMG
PRGPPGPPGKPGDDGEAGKPGKAGERGPPGPQGARGFPGTPGLPGVKGHRGYPGLDGAKG
EAGAPGVKGESGSPGENGSPGPMGPRGLPGERGRTGPAGAAGARGNDGQPGPAGPPGPVG
PAGGPGFPGAPGAKGEAGPTGARGPEGAQGPRGEPGTPGSPGPAGASGNPGTDGIPGAKG
SAGAPGIAGAPGFPGPRGPPGPQGATGPLGPKGQTGEPGIAGFKGEQGPKGEPGPAGPQG
APGPAGEEGKRGARGEPGGVGPIGPPGERGAPGNRGFPGQDGLAGPKGAPGERGPSGLAG
PKGANGDPGRPGEPGLPGARGLTGRPGDAGPQGKVGPSGAPGEDGRPGPPGPQGARGQPG
VMGFPGPKGANGEPGKAGEKGLPGAPGLRGLPGKDGETGAAGPPGPAGPAGERGEQGAPG
PSGFQGLPGPPGPPGEGGKPGDQGVPGEAGAPGLVGPRGERGFPGERGSPGAQGLQGPRG
LPGTPGTDGPKGASGPAGPPGAQGPPGLQGMPGERGAAGIAGPKGDRGDVGEKGPEGAPG
KDGGRGLTGPIGPPGPAGANGEKGEVGPPGPAGSAGARGAPGERGETGPPGPAGFAGPPG
ADGQPGAKGEQGEAGQKGDAGAPGPQGPSGAPGPQGPTGVTGPKGARGAQGPPGATGFPG
AAGRVGPPGSNGNPGPPGPPGPSGKDGPKGARGDSGPPGRAGEPGLQGPAGPPGEKGEPG
DDGPSGAEGPPGPQGLAGQRGIVGLPGQRGERGFPGLPGPSGEPGKQGAPGASGDRGPPG
PVGPPGLTGPAGEPGREGSPGADGPPGRDGAAGVKGDRGETGAVGAPGAPGPPGSPGPAG
PTGKQGDRGEAGAQGPMGPSGPAGARGIQGPQGPRGDKGEAGEPGERGLKGHRGFTGLQG
LPGPPGPSGDQGASGPAGPSGPRGPPGPVGPSGKDGANGIPGPIGPPGPRGRSGETGPAG
PPGNPGPPGPPGPPGPGIDMSAFAGLGPREKGPDPLQYMRADQAAGGLRQHDAEVDATLK
SLNNQIESIRSPEGSRKNPARTCRDLKLCHPEWKSGDYWIDPNQGCTLDAMKVFCNMETG
ETCVYPNPANVPKKNWWSSKSKEKKHIWFGETINGGFHFSYGDDNLAPNTANVQMTFLRL

LSTEGSQNITYHCKNSIAYLDEAAGNLKKALLIQGSNDVEIRAEGNSRFTYTALKDGCTK
HTGKWGKTVIEYRSQKTSRLPIIDIAPMDIGGPEQEFGVDIGPVCFL
>sp|P02462|CO4A1_HUMAN Collagen alpha-1(IV) chain OS=Homo sapiens OX=9606
GN=COL4A1 PE=1 SV=4
MGPRLSVWLLLLPAALLLHEEHSRAAAKGGCAGSGCGKCDCHGVKGQKGERGLPGLQGVI
GFPGMQGPEGPQGPPGQKGDTGEPGLPGTKGTRGPPGASGYPGNPGLPGIPGQDGPPGPP
GIPGCNGTKGERGPLGPPGLPGFAGNPGPPGLPGMKGDPGEILGHVPGMLLKGERGFPGI
PGTPGPPGLPGLQGPVGPPGFTGPPGPPGPPGPPGEKGQMGLSFQGPKGDKGDQGVSGPP
GVPGQAQVQEKGDFATKGEKGQKGEPGFQGMPGVGEKGEPGKPGPRGKPGKDGDKGEKGS
PGFPGEPGYPGLIGRQGPQGEKGEAGPPGPPGIVIGTGPLGEKGERGYPGTPGPRGEPGP
KGFPGLPGQPGPPGLPVPGQAGAPGFPGERGEKGDRGFPGTSLPGPSGRDGLPGPPGSPG
PPGQPGYTNGIVECQPGPPGDQGPPGIPGQPGFIGEIGEKGQKGESCLICDIDGYRGPPG
PQGPPGEIGFPGQPGAKGDRGLPGRDGVAGVPGPQGTPGLIGQPGAKGEPGEFYFDLRLK
GDKGDPGFPGQPGMPGRAGSPGRDGHPGLPGPKGSPGSVGLKGERGPPGGVGFPGSRGDT
GPPGPPGYGPAGPIGDKGQAGFPGGPGSPGLPGPKGEPGKIVPLPGPPGAEGLPGSPGFP
GPQGDRGFPGTPGRPGLPGEKGAVGQPGIGFPGPPGPKGVDGLPGDMGPPGTPGRPGFNG
LPGNPGVQGQKGEPGVGLPGLKGLPGLPGIPGTPGEKGSIGVPGVPGEHGAIGPPGLQGI
RGEPGPPGLPGSVGSPGVPGIGPPGARGPPGGQGPPGLSGPPGIKGEKGFPGFPGLDMPG
PKGDKGAQGLPGITGQSGLPGLPGQQGAPGIPGFPGSKGEMGVMGTPGQPGSPGPVGAPG
LPGEKGDHGFPGSSGPRGDPGLKGDKGDVGLPGKPGSMDKVDMGSMKGQKGDQGEKGQIG
PIGEKGSRGDPGTPGVPGKDGQAGQPGQPGPKGDPGISGTPGAPGLPGPKGSVGGMGLPG
TPGEKGVPGIPGPQGSPGLPGDKGAKGEKGQAGPPGIGIPGLRGEKGDQGIAGFPGSPGE
KGEKGSIGIPGMPGSPGLKGSPGSVGYPGSPGLPGEKGDKGLPGLDGIPGVKGEAGLPGT
PGPTGPAGQKGEPGSDGIPGSAGEKGEPGLPGRGFPGFPGAKGDKGSKGEVGFPGLAGSP
GIPGSKGEQGFMGPPGPQGQPGLPGSPGHATEGPKGDRGPQGQPGLPGLPGPMGPPGLPG
IDGVKGDKGNPGWPGAPGVPGPKGDPGFQGMPGIGGSPGITGSKGDMGPPGVPGFQGPKG
LPGLQGIKGDQGDQGVPGAKGLPGPPGPPGPYDIIKGEPGLPGPEGPPGLKGLQGLPGPK
GQQGVTGLVGIPGPPGIPGFDGAPGQKGEMGPAGPTGPRGFPGPPGPDGLPGSMGPPGTP
SVDHGFLVTRHSQTIDDPQCPSGTKILYHGYSLLYVQGNERAHGQDLGTAGSCLRKFSTM
PFLFCNINNVCNFASRNDYSYWLSTPEPMPMSMAPITGENIRPFISRCAVCEAPAMVMAV
HSQTIQIPPCPSGWSSLWIGYSFVMHTSAGAEGSGQALASPGSCLEEFRSAPFIECHGRG
TCNYYANAYSFWLATIERSEMFKKPTPSTLKAGELRTHVSRCQVCMRRT
>sp|P02489|CRYAA_HUMAN Alpha-crystallin A chain OS=Homo sapiens OX=9606 GN=CRYAA
PE=1 SV=2
MDVTIQHPWFKRTLGPFYPSRLFDQFFGEGLFEYDLLPFLSSTISPYYRQSLFRTVLDSG
ISEVRSDRDKFVIFLDVKHFSPEDLTVKVQDDFVEIHGKHNERQDDHGYISREFHRRYRL
PSNVDQSALSCSLSADGMLTFCGPKIQTGLDATHAERAIPVSREEKPTSAPSS
>sp|P02511|CRYAB_HUMAN Alpha-crystallin B chain OS=Homo sapiens OX=9606 GN=CRYAB
PE=1 SV=2
MDIAIHHPWIRRPFFPFHSPSRLFDQFFGEHLLESDLFPTSTSLSPFYLRPPSFLRAPSW
FDTGLSEMRLEKDRFSVNLDVKHFSPEELKVKVLGDVIEVHGKHEERQDEHGFISREFHR
KYRIPADVDPLTITSSLSSDGVLTVNGPRKQVSGPERTIPITREEKPAVTAAPKK
>sp|P02533|K1C14_HUMAN Keratin, type I cytoskeletal 14 OS=Homo sapiens OX=9606
GN=KRT14 PE=1 SV=4

MTTCSRQFTSSSSMKGSCGIGGGIGGGSSRISSVLAGGSCRAPSTYGGGLSVSSSRFSSG
GACGLGGGYGGGFSSSSSFGSGFGGGYGGGLGAGLGGGFGGGFAGGDGLLVGSEKVTMQ
NLNDRLASYLDKVRALEEANADLEVKIRDWYQRQRPAEIKDYSPYFKTIEDLRNKILTAT
VDNANVLLQIDNARLAADDFRTKYETELNLRMSVEADINGLRRVLDELTLARADLEMQIE
SLKEELAYLKKNHEEEMNALRGQVGGDVNVEMDAAPGVDLSRILNEMRDQYEKMAEKNRK
DAEEWFFTKTEELNREVATNSELVQSGKSEISELRRTMQNLEIELQSQLSMKASLENSLE
ETKGRYCMQLAQIQEMIGSVEEQLAQLRCEMEQQNQEYKILLDVKTRLEQEIATYRRLLE
GEDAHLSSSQFSSGSQSSRDVTSSSRQIRTKVMDVHDGKVVSTHEQVLRTKN
>sp|P02538|K2C6A_HUMAN Keratin, type II cytoskeletal 6A OS=Homo sapiens OX=9606
GN=KRT6A PE=1 SV=3
MASTSTTIRSHSSSRRGFSANSARLPGVSRSGFSSVSVSRSRGSGGLGGACGGAGFGSRS
LYGLGGSKRISIGGGSCAISGGYGSRAGGSYGFGGAGSGFGFGGGAGIGFGLGGGAGLAG
GFGGPGFPVCPPGGIQEVTVNQSLLTPLNLQIDPTIQRVRAEEREQIKTLNNKFASFIDK
VRFLEQQNKVLETKWTLLQEQGTKTVRQNLEPLFEQYINNLRRQLDSIVGERGRLDSELR
GMQDLVEDFKNKYEDEINKRTAAENEFVTLKKDVDAAYMNKVELQAKADTLTDEINFLRA
LYDAELSQMQTHISDTSVVLSMDNNRNLDLDSIIAEVKAQYEEIAQRSRAEAESWYQTKY
EELQVTAGRHGDDLRNTKQEIAEINRMIQRLRSEIDHVKKQCANLQAAIADAEQRGEMAL
KDAKNKLEGLEDALQKAKQDLARLLKEYQELMNVKLALDVEIATYRKLLEGEECRLNGEG
VGQVNISVVQSTVSSGYGGASGVGSGLGLGGGSSYSYGSGLGVGGGFSSSSGRAIGGGLS
SVGGGSSTIKYTTTSSSSRKSYKH
>sp|P02686|MBP_HUMAN Myelin basic protein OS=Homo sapiens OX=9606 GN=MBP PE=1
SV=3
MGNHAGKRELNAEKASTNSETNRGESEKKRNLGELSRTTSEDNEVFGEADANQNNGTSSQ
DTAVTDSKRTADPKNAWQDAHPADPGSRPHLIRLFSRDAPGREDNTFKDRPSESDELQTI
QEDSAATSESLDVMASQKRPSQRHGSKYLATASTMDHARHGFLPRHRDTGILDSIGRFFG
GDRGAPKRGSGKDSHHPARTAHYGSLPQKSHGRTQDENPVVHFFKNIVTPRTPPPSQGKG
RGLSLSRFSWGAEGQRPGFGYGGRASDYKSAHKGFKGVDAQGTLSKIFKLGGRDSRSGSP
MARR
>sp|P02768|ALBU_HUMAN Albumin OS=Homo sapiens OX=9606 GN=ALB PE=1 SV=2
MKWVTFISLLFLFSSAYSRGVFRRDAHKSEVAHRFKDLGEENFKALVLIAFAQYLQQCPF
EDHVKLVNEVTEFAKTCVADESAENCDKSLHTLFGDKLCTVATLRETYGEMADCCAKQEP
ERNECFLQHKDDNPNLPRLVRPEVDVMCTAFHDNEETFLKKYLYEIARRHPYFYAPELLF
FAKRYKAAFTECCQAADKAACLLPKLDELRDEGKASSAKQRLKCASLQKFGERAFKAWAV
ARLSQRFPKAEFAEVSKLVTDLTKVHTECCHGDLLECADDRADLAKYICENQDSISSKLK
ECCEKPLLEKSHCIAEVENDEMPADLPSLAADFVESKDVCKNYAEAKDVFLGMFLYEYAR
RHPDYSVVLLLRLAKTYETTLEKCCAAADPHECYAKVFDEFKPLVEEPQNLIKQNCELFE
QLGEYKFQNALLVRYTKKVPQVSTPTLVEVSRNLGKVGSKCCKHPEAKRMPCAEDYLSVV
LNQLCVLHEKTPVSDRVTKCCTESLVNRRPCFSALEVDETYVPKEFNAETFTFHADICTL
SEKERQIKKQTALVELVKHKPKATKEQLKAVMDDFAAFVEKCCKADDKETCFAEEGKKLV
AASQAALGL
>sp|P02786|TFR1_HUMAN Transferrin receptor protein 1 OS=Homo sapiens OX=9606
GN=TFRC PE=1 SV=2
MMDQARSAFSNLFGGEPLSYTRFSLARQVDGDNSHVEMKLAVDEEENADNNTKANVTKPK
RCSGSICYGTIAVIVFFLIGFMIGYLGYCKGVEPKTECERLAGTESPVREEPGEDFPAAR

RLYWDDLKRKLSEKLDSTDFTGTIKLLNENSYVPREAGSQKDENLALYVENQFREFKLSK
VWRDQHFVKIQVKDSAQNSVIIVDKNGRLVYLVENPGGYVAYSKAATVTGKLVHANFGTK
KDFEDLYTPVNGSIVIVRAGKITFAEKVANAESLNAIGVLIYMDQTKFPIVNAELSFFGH
AHLGTGDPYTPGFPSFNHTQFPPSRSSGLPNIPVQTISRAAAEKLFGNMEGDCPSDWKTD
STCRMVTSESKNVKLTVSNVLKEIKILNIFGVIKGFVEPDHYVVVGAQRDAWGPGAAKSG
VGTALLLKLAQMFSDMVLKDGFQPSRSIIFASWSAGDFGSVGATEWLEGYLSSLHLKAFT
YINLDKAVLGTSNFKVSASPLLYTLIEKTMQNVKHPVTGQFLYQDSNWASKVEKLTLDNA
AFPFLAYSGIPAVSFCFCEDTDYPYLGTTMDTYKELIERIPELNKVARAAAEVAGQFVIK
LTHDVELNLDYERYNSQLLSFVRDLNQYRADIKEMGLSLQWLYSARGDFFRATSRLTTDF
GNAEKTDRFVMKKLNDRVMRVEYHFLSPYVSPKESPFRHVFWGSGSHTLPALLENLKLRK
QNNGAFNETLFRNQLALATWTIQGAANALSGDVWDIDNEF
>sp|P02787|TRFE_HUMAN Serotransferrin OS=Homo sapiens OX=9606 GN=TF PE=1 SV=4
MRLAVGALLVCAVLGLCLAVPDKTVRWCAVSEHEATKCQSFRDHMKSVIPSDGPSVACVK
KASYLDCIRAIAANEADAVTLDAGLVYDAYLAPNNLKPVVAEFYGSKEDPQTFYYAVAVV
KKDSGFQMNQLRGKKSCHTGLGRSAGWNIPIGLLYCDLPEPRKPLEKAVANFFSGSCAPC
ADGTDFPQLCQLCPGCGCSTLNQYFGYSGAFKCLKDGAGDVAFVKHSTIFENLANKADRD
QYELLCLDNTRKPVDEYKDCHLAQVPSHTVVARSMGGKEDLIWELLNQAQEHFGKDKSKE
FQLFSSPHGKDLLFKDSAHGFLKVPPRMDAKMYLGYEYVTAIRNLREGTCPEAPTDECKP
VKWCALSHHERLKCDEWSVNSVGKIECVSAETTEDCIAKIMNGEADAMSLDGGFVYIAGK
CGLVPVLAENYNKSDNCEDTPEAGYFAVAVVKKSASDLTWDNLKGKKSCHTAVGRTAGWN
IPMGLLYNKINHCRFDEFFSEGCAPGSKKDSSLCKLCMGSGLNLCEPNNKEGYYGYTGAF
RCLVEKGDVAFVKHQTVPQNTGGKNPDPWAKNLNEKDYELLCLDGTRKPVEEYANCHLAR
APNHAVVTRKDKEACVHKILRQQQHLFGSNVTDCSGNFCLFRSETKDLLFRDDTVCLAKL
HDRNTYEKYLGEEYVKAVGNLRKCSTSSLLEACTFRRP
>sp|P02792|FRIL_HUMAN Ferritin light chain OS=Homo sapiens OX=9606 GN=FTL PE=1 SV=2
MSSQIRQNYSTDVEAAVNSLVNLYLQASYTYLSLGFYFDRDDVALEGVSHFFRELAEEKR
EGYERLLKMQNQRGGRALFQDIKKPAEDEWGKTPDAMKAAMALEKKLNQALLDLHALGSA
RTDPHLCDFLETHFLDEEVKLIKKMGDHLTNLHRLGGPEAGLGEYLFERLTLKHD
>sp|P02794|FRIH_HUMAN Ferritin heavy chain OS=Homo sapiens OX=9606 GN=FTH1 PE=1 SV=2
MTTASTSQVRQNYHQDSEAAINRQINLELYASYVYLSMSYYFDRDDVALKNFAKYFLHQS
HEEREHAEKLMKLQNQRGGRIFLQDIKKPDCDDWESGLNAMECALHLEKNVNQSLLELHK
LATDKNDPHLCDFIETHYLNEQVKAIKELGDHVTNLRKMGAPESGLAEYLFDKHTLGDSD
NES
>sp|P03950|ANGI_HUMAN Angiogenin OS=Homo sapiens OX=9606 GN=ANG PE=1 SV=1
MVMGLGVLLLVFVLGLGLTPPTLAQDNSRYTHFLTQHYDAKPQGRDDRYCESIMRRRGLT
SPCKDINTFIHGNKRSIKAICENKNGNPHRENLRISKSSFQVTTCKLHGGSPWPPCQYRA
TAGFRNVVVACENGLPVHLDQSIFRRP
>sp|P04075|ALDOA_HUMAN Fructose-bisphosphate aldolase A OS=Homo sapiens OX=9606 GN=ALDOA PE=1 SV=2
MPYQYPALTPEQKKELSDIAHRIVAPGKGILAADESTGSIAKRLQSIGTENTEENRRFYR
QLLLTADDRVNPCIGGVILFHETLYQKADDGRPFPQVIKSKGGVVGIKVDKGVVPLAGTN
GETTTQGLDGLSERCAQYKKDGADFAKWRCVLKIGEHTPSALAIMENANVLARYASICQQ
NGIVPIVEPEILPDGDHDLKRCQYVTEKVLAAVYKALSDHHIYLEGTLLKPNMVTPGHAC

TQKFSHEEIAMATVTALRRTVPPAVTGITFLSGGQSEEEASINLNAINKCPLLKPWALTF
SYGRALQASALKAWGGKKENLKAAQEEYVKRALANSLACQGKYTPSGQAGAAASESLFVS
NHAY
>sp|P04080|CYTB_HUMAN Cystatin-B OS=Homo sapiens OX=9606 GN=CSTB PE=1 SV=2
MMCGAPSATQPATAETQHIADQVRSQLEEKENKKFPVFKAVSFKSQVVAGTNYFIKVHVG
DEDFVHLRVFQSLPHENKPLTLSNYQTNKAKHDELTYF
>sp|P04083|ANXA1_HUMAN Annexin A1 OS=Homo sapiens OX=9606 GN=ANXA1 PE=1 SV=2
MAMVSEFLKQAWFIENEEQEYVQTVKSSKGGPGSAVSPYPTFNPSSDVAALHKAIMVKGV
DEATIIDILTKRNNAQRQQIKAAYLQETGKPLDETLKKALTGHLEEVVLALLKTPAQFDA
DELRAAMKGLGTDEDTLIEILASRTNKEIRDINRVYREELKRDLAKDITSDTSGDFRNAL
LSLAKGDRSEDFGVNEDLADSDARALYEAGERRKGTDVNVFNTILTTRSYPQLRRVFQKY
TKYSKHDMNKVLDLELKGDIEKCLTAIVKCATSKPAFFAEKLHQAMKGVGTRHKALIRIM
VSRSEIDMNDIKAFYQKMYGISLCQAILDETKGDYEKILVALCGGN
>sp|P04216|THY1_HUMAN Thy-1 membrane glycoprotein OS=Homo sapiens OX=9606
GN=THY1 PE=1 SV=2
MNLAISIALLLTVLQVSRGQKVTSLTACLVDQSLRLDCRHENTSSSPIQYEFSLTRETKK
HVLFGTVGVPEHTYRSRTNFTSKYNMKVLYLSAFTSKDEGTYTCALHHSGHSPPISSQNV
TVLRDKLVKCEGISLLAQNTSWLLLLLLSLSLLQATDFMSL
>sp|P04264|K2C1_HUMAN Keratin, type II cytoskeletal 1 OS=Homo sapiens OX=9606 GN=KRT1
PE=1 SV=6
MSRQFSSRSGYRSGGGFSSGSAGIINYQRRTTSSSTRRSGGGGGRFSSCGGGGGSFGAGG
GFGSRSLVNLGGSKSISISVARGGGRGSGFGGGYGGGGFGGGGFGGGGFGGGGIGGGGFG
GFGSGGGGFGGGGFGGGGYGGGYGPVCPPGGIQEVTINQSLLQPLNVEIDPEIQKVKSRE
REQIKSLNNQFASFIDKVRFLEQQNQVLQTKWELLQQVDTSTRTHNLEPYFESFINNLRR
RVDQLKSDQSRLDSELKNMQDMVEDYRNKYEDEINKRTNAENEFVTIKKDVDGAYMTKVD
LQAKLDNLQQEIDFLTALYQAELSQMQTQISETNVILSMDNNRSLDLDSIIAEVKAQYED
IAQKSKAEAESLYQSKYEELQITAGRHGDSVRNSKIEISELNRVIQRLRSEIDNVKKQIS
NLQQSISDAEQRGENALKDAKNKLNDLEDALQQAKEDLARLLRDYQELMNTKLALDLEIA
TYRTLLEGEESRMSGECAPNVSVSVSTSHTTISGGGSRGGGGGGYGSGGSSYGSGGGSYG
SGGGGGGGRGSYGSGGSSYGSGGGSYGSGGGGGGHGSYGSGSSSGGYRGGSGGGGGGSSG
GRGSGGGSSGGSIGGRGSSSGGVKSSGGSSSVKFVSTTYSGVTR
>sp|P04271|S100B_HUMAN Protein S100-B OS=Homo sapiens OX=9606 GN=S100B PE=1 SV=2
MSELEKAMVALIDVFHQYSGREGDKHKLKKSELKELINNELSHFLEEIKEQEVVDKVMET
LDNDGDGECDFQEFMAFVAMVTTACHEFFEHE
>sp|P04350|TBB4A_HUMAN Tubulin beta-4A chain OS=Homo sapiens OX=9606 GN=TUBB4A
PE=1 SV=2
MREIVHLQAGQCGNQIGAKFWEVISDEHGIDPTGTYHGDSDLQLERINVYYNEATGGNYV
PRAVLVDLEPGTMDSVRSGPFGQIFRPDNFVFGQSGAGNNWAKGHYTEGAELVDAVLDVV
RKEAESCDCLQGFQLTHSLGGGTGSGMGTLLISKIREEFPDRIMNTFSVVPSPKVSDTVV
EPYNATLSVHQLVENTDETYCIDNEALYDICFRTLKLTTPTYGDLNHLVSATMSGVTTCL
RFPGQLNADLRKLAVNMVPFPRLHFFMPGFAPLTSRGSQQYRALTVPELTQQMFDAKNMM
AACDPRHGRYLTVAAVFRGRMSMKEVDEQMLSVQSKNSSYFVEWIPNNVKTAVCDIPPRG
LKMAATFIGNSTAIQELFKRISEQFTAMFRRKAFLHWYTGEGMDEMEFTEAESNMNDLVS
EYQQYQDATAEEGEFEEEAEEEVA

>sp|P04406|G3P_HUMAN Glyceraldehyde-3-phosphate dehydrogenase OS=Homo sapiens OX=9606 GN=GAPDH PE=1 SV=3
MGKVKVGVNGFGRIGRLVTRAAFNSGKVDIVAINDPFIDLNYMVYMFQYDSTHGKFHGTV
KAENGKLVINGNPITIFQERDPSKIKWGDAGAEYVVESTGVFTTMEKAGAHLQGGAKRVI
ISAPSADAPMFVMGVNHEKYDNSLKIISNASCTTNCLAPLAKVIHDNFGIVEGLMTTVHA
ITATQKTVDGPSGKLWRDGRGALQNIIPASTGAAKAVGKVIPELNGKLTGMAFRVPTANV
SVVDLTCRLEKPAKYDDIKKVVKQASEGPLKGILGYTEHQVVSSDFNSDTHSSTFDAGAG
IALNDHFVKLISWYDNEFGYSNRVVDLMAHMASKE
>sp|P04792|HSPB1_HUMAN Heat shock protein beta-1 OS=Homo sapiens OX=9606 GN=HSPB1 PE=1 SV=2
MTERRVPFSLLRGPSWDPFRDWYPHSRLFDQAFGLPRLPEEWSQWLGGSSWPGYVRPLPP
AAIESPAVAAPAYSRALSRQLSSGVSEIRHTADRWRVSLDVNHFAPDELTVKTKDGVVEI
TGKHEERQDEHGYISRCFTRKYTLPPGVDPTQVSSSLSPEGTLTVEAPMPKLATQSNEIT
IPVTFESRAQLGGPEAAKSDETAAK
>sp|P04843|RPN1_HUMAN Dolichyl-diphosphooligosaccharide--protein glycosyltransferase subunit 1 OS=Homo sapiens OX=9606 GN=RPN1 PE=1 SV=1
MEAPAAGLFLLLLLGTWAPAPGSASSEAPPLINEDVKRTVDLSSHLAKVTAEVVLAHLGG
GSTSRATSFLLALEPELEARLAHLGVQVKGEDEEENNLEVRETKIKGKSGRFFTVKLPVA
LDPGAKISVIVETVYTHVLHPYPTQITQSEKQFVVFEGNHYFYSPYPTKTQTMRVKLASR
NVESYTKLGNPTRSEDLLDYGPFRDVPAYSQDTFKVHYENNSPFLTITSMTRVIEVSHWG
NIAVEENVDLKHTGAVLKGPFSRYDYQRQPDSGISSIRSFKTILPAAAQDVYYRDEIGNV
STSHLLILDDSVEMEIRPRFPLFGGWKTHYIVGYNLPSYEYLYNLGDQYALKMRFVDHVF
DEQVIDSLTVKIILPEGAKNIEIDSPYEISRAPDELHYTYLDTFGRPVIVAYKKNLVEQH
IQDIVVHYTFNKVLMLQEPLLVVAAFYILFFTVIIYVRLDFSITKDPAAEARMKVACITE
QVLTLVNKRIGLYRHFDETVNRYKQSRDISTLNSGKKSLETEHKALTSEIALLQSRLKTE
GSDLCDRVSEMQKLDAQVKELVLKSAVEAERLVAGKLKKDTYIENEKLISGKRQELVTKI
DHILDAL
>sp|P04844|RPN2_HUMAN Dolichyl-diphosphooligosaccharide--protein glycosyltransferase subunit 2 OS=Homo sapiens OX=9606 GN=RPN2 PE=1 SV=3
MAPPGSSTVFLLALTIIASTWALTPTHYLTKHDVERLKASLDRPFTNLESAFYSIVGLSS
LGAQVPDAKKACTYIRSNLDPSNVDSLFYAAQASQALSGCEISISNETKDLLLAAVSEDS
SVTQIYHAVAALSGFGLPLASQEALSALTARLSKEETVLATVQALQTASHLSQQADLRSI
VEEIEDLVARLDELGGVYLQFEEGLETTALFVAATYKLMDHVGTEPSIKEDQVIQLMNAI
FSKKNFESLSEAFSVASAAAVLSHNRYHVPVVVVPEGSASDTHEQAILRLQVTNVLSQPL
TQATVKLEHAKSVASRATVLQKTSFTPVGDVFELNFMNVKFSSGYYDFLVEVEGDNRYIA
NTVELRVKISTEVGITNVDLSTVDKDQSIAPKTTRVTYPAKAKGTFIADSHQNFALFFQL
VDVNTGAELTPHQTFVRLHNQKTGQEVVFVAEPDNKNVYKFELDTSERKIEFDSASGTYT
LYLIIGDATLKNPILWNVADVVIKFPEEEAPSTVLSQNLFTPKQEIQHLFREPEKRPPTV
VSNTFTALILSPLLLLFALWIRIGANVSNFTFAPSTIIFHLGHAAMLGLMYVYWTQLNMF
QTLKYLAILGSVTFLAGNRMLAQQAVKRTAH
>sp|P04899|GNAI2_HUMAN Guanine nucleotide-binding protein G(i) subunit alpha-2 OS=Homo sapiens OX=9606 GN=GNAI2 PE=1 SV=3
MGCTVSAEDKAAAERSKMIDKNLREDGEKAAREVKLLLLGAGESGKSTIVKQMKIIHEDG
YSEEECRQYRAVVYSNTIQSIMAIVKAMGNLQIDFADPSRADDARQLFALSCTAEEQGVL

PDDLSGVIRRLWADHGVQACFGRSREYQLNDSAAYYLNDLERIAQSDYIPTQQDVLRTRV
KTTGIVETHFTFKDLHFKMFDVGGQRSERKKWIHCFEGVTAIIFCVALSAYDLVLAEDEE
MNRMHESMKLFDSICNNKWFTDTSIILFLNKKDLFEEKITHSPLTICFPEYTGANKYDEA
ASYIQSKFEDLNKRKDTKEIYTHFTCATDTKNVQFVFDAVTDVIIKNNLKDCGLF
>sp|P04908|H2A1B_HUMAN Histone H2A type 1-B/E OS=Homo sapiens OX=9606 GN=H2AC4
PE=1 SV=2
MSGRGKQGGKARAKAKTRSSRAGLQFPVGRVHRLLRKGNYSERVGAGAPVYLAAVLEYLT
AEILELAGNAARDNKKTRIIPRHLQLAIRNDEELNKLLGRVTIAQGGVLPNIQAVLLPKK
TESHHKAKGK
>sp|P04920|B3A2_HUMAN Anion exchange protein 2 OS=Homo sapiens OX=9606 GN=SLC4A2
PE=1 SV=4
MSSAPRRPAKGADSFCTPEPESLGPGTPGFPEQEEDELHRTLGVERFEEILQEAGSRGGE
EPGRSYGEEDFEYHRQSSHHIHHPLSTHLPPDARRRKTPQGPGRKPRRRPGASPTGETPT
IEEGEEDEDEASEAEGARALTQPSPVSTPSSVQFFLQEDDSADRKAERTSPSSPAPLPHQ
EATPRASKGAQAGTQVEEAEAEAVAVASGTAGGDDGGASGRPLPKAQPGHRSYNLQERRR
IGSMTGAEQALLPRVPTDEIEAQTLATADLDLMKSHRFEDVPGVRRHLVRKNAKGSTQSG
REGREPGPTPRARPRAPHKPHEVFVELNELLLDKNQEPQWRETARWIKFEEDVEEETERW
GKPHVASLSFRSLLELRRTLAHGAVLLDLDQQTLPGVAHQVVEQMVISDQIKAEDRANVL
RALLLKHSHPSDEKDFSFPRNISAGSLGSLLGHHHGQGAESDPHVTEPLMGGVPETRLEV
ERERELPPPAPPAGITRSKSKHELKLLEKIPENAEATVVLVGCVEFLSRPTMAFVRLREA
VELDAVLEVPVPVRFLFLLLGPSSANMDYHEIGRSISTLMSDKQFHEAAYLADEREDLLT
AINAFLDCSVVLPPSEVQGEELLRSVAHFQRQMLKKREEQGRLLPTGAGLEPKSAQDKAL
LQMVEAAGAAEDDPLRRTGRPFGGLIRDVRRRYPHYLSDFRDALDPQCLAAVIFIYFAAL
SPAITFGGLLGEKTQDLIGVSELIMSTALQGVVFCLLGAQPLLVIGFSGPLLVFEEAFFS
FCSSNHLEYLVGRVWIGFWLVFLALLMVALEGSFLVRFVSRFTQEIFAFLISLIFIYETF
YKLVKIFQEHPLHGCSASNSSEVDGGENMTWAGARPTLGPGNRSLAGQSGQGKPRGQPNT
ALLSLVLMAGTFFIAFFLRKFKNSRFFPGRIRRVIGDFGVPIAILIMVLVDYSIEDTYTQ
KLSVPSGFSVTAPEKRGWVINPLGEKSPFPVWMMVASLLPAILVFILIFMETQITTLIIS
KKERMLQKGSGFHLDLLLIVAMGGICALFGLPWLAAATVRSVTHANALTVMSKAVAPGDK
PKIQEVKEQRVTGLLVALLVGLSIVIGDLLRQIPLAVLFGIFLYMGVTSLNGIQFYERLH
LLLMPPKHHPDVTYVKKVRTLRMHLFTALQLLCLALLWAVMSTAASLAFPFILILTVPLR
MVVLTRIFTDREMKCLDANEAEPVFDEREGVDEYNEMPMPV
>sp|P05023|AT1A1_HUMAN Sodium/potassium-transporting ATPase subunit alpha-1
OS=Homo sapiens OX=9606 GN=ATP1A1 PE=1 SV=1
MGKGVGRDKYEPAAVSEQGDKKGKKGKKDRDMDELKKEVSMDDHKLSLDELHRKYGTDLS
RGLTSARAAEILARDGPNALTPPPTTPEWIKFCRQLFGGFSMLLWIGAILCFLAYSIQAA
TEEEPQNDNLYLGVVLSAVVIITGCFSYYQEAKSSKIMESFKNMVPQQALVIRNGEKMSI
NAEEVVVGDLVEVKGGDRIPADLRIISANGCKVDNSSLTGESEPQTRSPDFTNENPLETR
NIAFFSTNCVEGTARGIVVYTGDRTVMGRIATLASGLEGGQTPIAAEIEHFIHIITGVAV
FLGVSFFILSLILEYTWLEAVIFLIGIIVANVPEGLLATVTVCLTLTAKRMARKNCLVKN
LEAVETLGSTSTICSDKTGTLTQNRMTVAHMWFDNQIHEADTTENQSGVSFDKTSATWLA
LSRIAGLCNRAVFQANQENLPILKRAVAGDASESALLKCIELCCGSVKEMRERYAKIVEI
PFNSTNKYQLSIHKNPNTSEPQHLLVMKGAPERILDRCSSILLHGKEQPLDEELKDAFQN
AYLELGGLGERVLGFCHLFLPDEQFPEGFQFDTDDVNFPIDNLCFVGLISMIDPPRAAVP

DAVGKCRSAGIKVIMVTGDHPITAKAIAKGVGIISEGNETVEDIAARLNIPVSQVNPRDA
KACVVHGSDLKDMTSEQLDDILKYHTEIVFARTSPQQKLIIVEGCQRQGAIVAVTGDGVN
DSPALKKADIGVAMGIAGSDVSKQAADMILLDDNFASIVTGVEEGRLIFDNLKKSIAYTL
TSNIPEITPFLIFIIANIPLPLGTVTILCIDLGTDMVPAISLAYEQAESDIMKRQPRNPK
TDKLVNERLISMAYGQIGMIQALGGFFTYFVILAENGFLPIHLLGLRVDWDDRWINDVED
SYGQQWTYEQRKIVEFTCHTAFFVSIVVVQWADLVICKTRRNSVFQQGMKNKILIFGLFE
ETALAAFLSYCPGMGVALRMYPLKPTWWFCAFPYSLLIFVYDEVRKLIIRRRPGGWVEKE
TYY
>sp|P05026|AT1B1_HUMAN Sodium/potassium-transporting ATPase subunit beta-1 OS=Homo sapiens OX=9606 GN=ATP1B1 PE=1 SV=1
MARGKAKEEGSWKKFIWNSEKKEFLGRTGGSWFKILLFYVIFYGCLAGIFIGTIQVMLLT
ISEFKPTYQDRVAPPGLTQIPQIQKTEISFRPNDPKSYEAYVLNIVRFLEKYKDSAQRDD
MIFEDCGDVPSEPKERGDFNHERGERKVCRFKLEWLGNCSGLNDETYGYKEGKPCIIIKL
NRVLGFKPKPPKNESLETYPVMKYNPNVLPVQCTGKRDEDKDKVGNVEYFGLGNSPGFPL
QYYPYYGKLLQPKYLQPLLAVQFTNLTMDTEIRIECKAYGENIGYSEKDRFQGRFDVKIE
VKS
>sp|P05062|ALDOB_HUMAN Fructose-bisphosphate aldolase B OS=Homo sapiens OX=9606 GN=ALDOB PE=1 SV=2
MAHRFPALTQEQKKELSEIAQSIVANGKGILAADESVGTMGNRLQRIKVENTEENRRQFR
EILFSVDSSINQSIGGVILFHETLYQKDSQGKLFRNILKEKGIVVGIKLDQGGAPLAGTN
KETTIQGLDGLSERCAQYKKDGVDFGKWRAVLRIADQCPSSLAIQENANALARYASICQQ
NGLVPIVEPEVIPDGDHDLEHCQYVTEKVLAAVYKALNDHHVYLEGTLLKPNMVTAGHAC
TKKYTPEQVAMATVTALHRTVPAAVPGICFLSGGMSEEDATLNLNAINLCPLPKPWKLSF
SYGRALQASALAAWGGKAANKEATQEAFMKRAMANCQAAKGQYVHTGSSGAASTQSLFTA
CYTY
>sp|P05091|ALDH2_HUMAN Aldehyde dehydrogenase, mitochondrial OS=Homo sapiens OX=9606 GN=ALDH2 PE=1 SV=2
MLRAAARFGPRLGRRLLSAAATQAVPAPNQQPEVFCNQIFINNEWHDAVSRKTFPTVNPS
TGEVICQVAEGDKEDVDKAVKAARAAFQLGSPWRRMDASHRGRLLNRLADLIERDRTYLA
ALETLDNGKPYVISYLVDLDMVLKCLRYYAGWADKYHGKTIPIDGDFFSYTRHEPVGVCG
QIIPWNFPLLMQAWKLGPALATGNVVVMKVAEQTPLTALYVANLIKEAGFPPGVVNIVPG
FGPTAGAAIASHEDVDKVAFTGSTEIGRVIQVAAGSSNLKRVTLELGGKSPNIIMSDADM
DWAVEQAHFALFFNQGQCCCAGSRTFVQEDIYDEFVERSVARAKSRVVGNPFDSKTEQGP
QVDETQFKKILGYINTGKQEGAKLLCGGGIAADRGYFIQPTVFGDVQDGMTIAKEEIFGP
VMQILKFKTIEEVVGRANNSTYGLAAAVFTKDLDKANYLSQALQAGTVWVNCYDVFGAQS
PFGGYKMSGSGRELGEYGLQAYTEVKTVTVKVPQKNS
>sp|P05198|IF2A_HUMAN Eukaryotic translation initiation factor 2 subunit 1 OS=Homo sapiens OX=9606 GN=EIF2S1 PE=1 SV=3
MPGLSCRFYQHKFPEVEDVVMVNVRSIAEMGAYVSLLEYNNIEGMILLSELSRRRIRSIN
KLIRIGRNECVVVIRVDKEKGYIDLSKRRVSPEEAIKCEDKFTKSKTVYSILRHVAEVLE
YTKDEQLESLFQRTAWVFDDKYKRPGYGAYDAFKHAVSDPSILDSLDLNEDEREVLINNI
NRRLTPQAVKIRADIEVACYGYEGIDAVKEALRAGLNCSTENMPIKINLIAPPRYVMTTT
TLERTEGLSVLSQAMAVIKEKIEEKRGVFNVQMEPKVVTDTDETELARQMERLERENAEV
DGDDDAEEMEAKAED

>sp|P05388|RLA0_HUMAN Large ribosomal subunit protein uL10 OS=Homo sapiens OX=9606 GN=RPLP0 PE=1 SV=1
MPREDRATWKSNYFLKIIQLLDDYPKCFIVGADNVGSKQMQQIRMSLRGKAVVLMGKNTM
MRKAIRGHLENNPALEKLLPHIRGNVGFVFTKEDLTEIRDMLLANKVPAAARAGAIAPCE
VTVPAQNTGLGPEKTSFFQALGITTKISRGTIEILSDVQLIKTGDKVGASEATLLNMLNI
SPFSFGLVIQQVFDNGSIYNPEVLDITEETLHSRFLEGVRNVASVCLQIGYPTVASVPHS
IINGYKRVLALSVETDYTFPLAEKVKAFLADPSAFVAAAPVAAATTAAPAAAAAPAKVEA
KEESEESDEDMGFGLFD
>sp|P05556|ITB1_HUMAN Integrin beta-1 OS=Homo sapiens OX=9606 GN=ITGB1 PE=1 SV=2
MNLQPIFWIGLISSVCCVFAQTDENRCLKANAKSCGECIQAGPNCGWCTNSTFLQEGMPT
SARCDDLEALKKKGCPPDDIENPRGSKDIKKNKNVTNRSKGTAEKLKPEDITQIQPQQLV
LRLRSGEPQTFTLKFKRAEDYPIDLYYLMDLSYSMKDDLENVKSLGTDLMNEMRRITSDF
RIGFGSFVEKTVMPYISTTPAKLRNPCTSEQNCTSPFSYKNVLSLTNKGEVFNELVGKQR
ISGNLDSPEGGFDAIMQVAVCGSLIGWRNVTRLLVFSTDAGFHFAGDGKLGGIVLPNDGQ
CHLENNMYTMSHYYDYPSIAHLVQKLSENNIQTIFAVTEEFQPVYKELKNLIPKSAVGTL
SANSSNVIQLIIDAYNSLSSEVILENGKLSEGVTISYKSYCKNGVNGTGENGRKCSNISI
GDEVQFEISITSNKCPKKDSDSFKIRPLGFTEEVEVILQYICECECQSEGIPESPKCHEG
NGTFECGACRCNEGRVGRHCECSTDEVNSEDMDAYCRKENSSEICSNNGECVCGQCVCRK
RDNTNEIYSGKFCECDNFNCDRSNGLICGGNGVCKCRVCECNPNYTGSACDCSLDTSTCE
ASNGQICNGRGICECGVCKCTDPKFQGQTCEMCQTCLGVCAEHKECVQCRAFNKGEKKDT
CTQECSYFNITKVESRDKLPQPVQPDPVSHCKEKDVDDCWFYFTYSVNGNNEVMVHVVEN
PECPTGPDIIPIVAGVVAGIVLIGLALLLIWKLLMIIHDRREFAKFEKEKMNAKWDTGEN
PIYKSAVTTVVNPKYEGK
>sp|P05813|CRBA1_HUMAN Beta-crystallin A3 OS=Homo sapiens OX=9606 GN=CRYBA1 PE=1 SV=4
METQAEQQELETLPTTKMAQTNPTPGSLGPWKITIYDQENFQGKRMEFTSSCPNVSERSF
DNVRSLKVESGAWIGYEHTSFCGQQFILERGEYPRWDAWSGSNAYHIERLMSFRPICSAN
HKESKMTIFEKENFIGRQWEISDDYPSLQAMGWFNNEVGSMKIQSGAWVCYQYPGYRGYQ
YILECDHHGGDYKHWREWGSHAQTSQIQSIRRIQQ
>sp|P06396|GELS_HUMAN Gelsolin OS=Homo sapiens OX=9606 GN=GSN PE=1 SV=1
MAPHRPAPALLCALSLALCALSLPVRAATASRGASQAGAPQGRVPEARPNSMVVEHPEFL
KAGKEPGLQIWRVEKFDLVPVPTNLYGDFFTGDAYVILKTVQLRNGNLQYDLHYWLGNEC
SQDESGAAAIFTVQLDDYLNGRAVQHREVQGFESATFLGYFKSGLKYKKGGVASGFKHVV
PNEVVVQRLFQVKGRRVVRATEVPVSWESFNNGDCFILDLGNNIHQWCGSNSNRYERLKA
TQVSKGIRDNERSGRARVHVSEEGTEPEAMLQVLGPKPALPAGTEDTAKEDAANRKLAKL
YKVSNGAGTMSVSLVADENPFAQGALKSEDCFILDHGKDGKIFVWKGKQANTEERKAALK
TASDFITKMDYPKQTQVSVLPEGGETPLFKQFFKNWRDPDQTDGLGLSYLSSHIANVERV
PFDAATLHTSTAMAAQHGMDDDGTGQKQIWRIEGSNKVPVDPATYGQFYGGDSYIILYNY
RHGGRQGQIIYNWQGAQSTQDEVAASAILTAQLDEELGGTPVQSRVVQGKEPAHLMSLFG
GKPMIIYKGGTSREGGQTAPASTRLFQVRANSAGATRAVEVLPKAGALNSNDAFVLKTPS
AAYLWVGTGASEAEKTGAQELLRVLRAQPVQVAEGSEPDGFWEALGGKAAYRTSPRLKDK
KMDAHPPRLFACSNKIGRFVIEEVPGELMQEDLATDDVMLLDTWDQVFVWVGKDSQEEEK
TEALTSAKRYIETDPANRDRRTPITVVKQGFEPPSFVGWFLGWDDDYWSVDPLDRAMAEL
AA

>sp|P06576|ATPB_HUMAN ATP synthase subunit beta, mitochondrial OS=Homo sapiens OX=9606 GN=ATP5F1B PE=1 SV=3
MLGFVGRVAAAPASGALRRLTPSASLPPAQLLLRAAPTAVHPVRDYAAQTSPSPKAGAAT
GRIVAVIGAVVDVQFDEGLPPILNALEVQGRETRLVLEVAQHLGESTVRTIAMDGTEGLV
RGQKVLDSGAPIKIPVGPETLGRIMNVIGEPIDERGPIKTKQFAPIHAEAPEFMEMSVEQ
EILVTGIKVVDLLAPYAKGGKIGLFGGAGVGKTVLIMELINNVAKAHGGYSVFAGVGERT
REGNDLYHEMIESGVINLKDATSKVALVYGQMNEPPGARARVALTGLTVAEYFRDQEGQD
VLLFIDNIFRFTQAGSEVSALLGRIPSAVGYQPTLATDMGTMQERITTTKKGSITSVQAI
YVPADDLTDPAPATTFAHLDATTVLSRAIAELGIYPAVDPLDSTSRIMDPNIVGSEHYDV
ARGVQKILQDYKSLQDIIAILGMDELSEEDKLTVSRARKIQRFLSQPFQVAEVFTGHMGK
LVPLKETIKGFQQILAGEYDHLPEQAFYMVGPIEEAVAKADKLAEEHSS
>sp|P06703|S10A6_HUMAN Protein S100-A6 OS=Homo sapiens OX=9606 GN=S100A6 PE=1 SV=1
MACPLDQAIGLLVAIFHKYSGREGDKHTLSKKELKELIQKELTIGSKLQDAEIARLMEDL
DRNKDQEVNFQEYVTFLGALALIYNEALKG
>sp|P06733|ENOA_HUMAN Alpha-enolase OS=Homo sapiens OX=9606 GN=ENO1 PE=1 SV=2
MSILKIHAREIFDSRGNPTVEVDLFTSKGLFRAAVPSGASTGIYEALELRDNDKTRYMGK
GVSKAVEHINKTIAPALVSKKLNVTEQEKIDKLMIEMDGTENKSKFGANAILGVSLAVCK
AGAVEKGVPLYRHIADLAGNSEVILPVPAFNVINGGSHAGNKLAMQEFMILPVGAANFRE
AMRIGAEVYHNLKNVIKEKYGKDATNVGDEGGFAPNILENKEGLELLKTAIGKAGYTDKV
VIGMDVAASEFFRSGKYDLDFKSPDDPSRYISPDQLADLYKSFIKDYPVVSIEDPFDQDD
WGAWQKFTASAGIQVVGDDLTVTNPKRIAKAVNEKSCNCLLLKVNQIGSVTESLQACKLA
QANGWGVMVSHRSGETEDTFIADLVVGLCTGQIKTGAPCRSERLAKYNQLLRIEEELGSK
AKFAGRNFRNPLAK
>sp|P06737|PYGL_HUMAN Glycogen phosphorylase, liver form OS=Homo sapiens OX=9606 GN=PYGL PE=1 SV=4
MAKPLTDQEKRRQISIRGIVGVENVAELKKSFNRHLHFTLVKDRNVATTRDYYFALAHTV
RDHLVGRWIRTQQHYYDKCPKRVYYLSLEFYMGRTLQNTMINLGLQNACDEAIYQLGLDI
EELEEIEEDAGLGNGGLGRLAACFLDSMATLGLAAYGYGIRYEYGIFNQKIRDGWQVEEA
DDWLRYGNPWEKSRPEFMLPVHFYGKVEHTNTGTKWIDTQVVLALPYDTPVPGYMNNTVN
TMRLWSARAPNDFNLRDFNVGDYIQAVLDRNLAENISRVLYPNDNFFEGKELRLKQEYFV
VAATLQDIIRRFKASKFGSTRGAGTVFDAFPDQVAIQLNDTHPALAIPELMRIFVDIEKL
PWSKAWELTQKTFAYTNHTVLPEALERWPVDLVEKLLPRHLEIIYEINQKHLDRIVALFP
KDVDRLRRMSLIEEEGSKRINMAHLCIVGSHAVNGVAKIHSDIVKTKVFKDFSELEPDKF
QNKTNGITPRRWLLLCNPGLAELIAEKIGEDYVKDLSQLTKLHSFLGDDVFLRELAKVKQ
ENKLKFSQFLETEYKVKINPSSMFDVQVKRIHEYKRQLLNCLHVITMYNRIKKDPKKLFV
PRTVIIGGKAAPGYHMAKMIIKLITSVADVVNNDPMVGSKLKVIFLENYRVSLAEKVIPA
TDLSEQISTAGTEASGTGNMKFMLNGALTIGTMDGANVEMAEEAGEENLFIFGMRIDDVA
ALDKKGYEAKEYYEALPELKLVIDQIDNGFFSPKQPDLFKDIINMLFYHDRFKVFADYEA
YVKCQDKVSQLYMNPKAWNTMVLKNIAASGKFSSDRTIKEYAQNIWNVEPSDLKISLSNE
SNKVNGN
>sp|P06744|G6PI_HUMAN Glucose-6-phosphate isomerase OS=Homo sapiens OX=9606 GN=GPI PE=1 SV=4
MAALTRDPQFQKLQQWYREHRSELNLRRLFDANKDRFNHFSLTLNTNHGHILVDYSKNLV

TEDVMRMLVDLAKSRGVEAARERMFNGEKINYTEGRAVLHVALRNRSNTPILVDGKDVMP
EVNKVLDKMKSFCQRVRSGDWKGYTGKTITDVINIGIGGSDLGPLMVTEALKPYSSGGPR
VWYVSNIDGTHIAKTLAQLNPESSLFIIASKTFTTQETITNAETAKEWFLQAAKDPSAVA
KHFVALSTNTTKVKEFGIDPQNMFEFWDWVGGRYSLWSAIGLSIALHVGFDNFEQLLSGA
HWMDQHFRTTPLEKNAPVLLALLGIWYINCFGCETHAMLPYDQYLHRFAAYFQQGDMESN
GKYITKSGTRVDHQTGPIVWGEPGTNGQHAFYQLIHQGTKMIPCDFLIPVQTQHPIRKGL
HHKILLANFLAQTEALMRGKSTEEARKELQAAGKSPEDLERLLPHKVFEGNRPTNSIVFT
KLTPFMLGALVAMYEHKIFVQGIIWDINSFDQWGVELGKQLAKKIEPELDGSAQVTSHDA
STNGLINFIKQQREARVQ
>sp|P06753|TPM3_HUMAN Tropomyosin alpha-3 chain OS=Homo sapiens OX=9606 GN=TPM3 PE=1 SV=2
MMEAIKKKMQMLKLDKENALDRAEQAEAEQKQAEERSKQLEDELAAMQKKLKGTEDELDK
YSEALKDAQEKLELAEKKAADAEAEVASLNRRIQLVEEELDRAQERLATALQKLEEAEKA
ADESERGMKVIENRALKDEEKMELQEIQLKEAKHIAEEADRKYEEVARKLVIIEGDLERT
EERAELAESKCSELEEELKNVTNNLKSLEAQAEKYSQKEDKYEEEIKILTDKLKEAETRA
EFAERSVAKLEKTIDDLEDELYAQKLKYKAISEELDHALNDMTSI
>sp|P07195|LDHB_HUMAN L-lactate dehydrogenase B chain OS=Homo sapiens OX=9606 GN=LDHB PE=1 SV=2
MATLKEKLIAPVAEEEATVPNNKITVVGVGQVGMACAISILGKSLADELALVDVLEDKLK
GEMMDLQHGSLFLQTPKIVADKDYSVTANSKIVVVTAGVRQQEGESRLNLVQRNVNVFKF
IIPQIVKYSPDCIIIVVSNPVDILTYVTWKLSGLPKHRVIGSGCNLDSARFRYLMAEKLG
IHPSSCHGWILGEHGDSSVAVWSGVNVAGVSLQELNPEMGTDNDSENWKEVHKMVVESAY
EVIKLKGYTNWAIGLSVADLIESMLKNLSRIHPVSTMVKGMYGIENEVFLSLPCILNARG
LTSVINQKLKDDEVAQLKKSADTLWDIQKDLKDL
>sp|P07203|GPX1_HUMAN Glutathione peroxidase 1 OS=Homo sapiens OX=9606 GN=GPX1 PE=1 SV=4
MCAARLAAAAAAAQSVYAFSARPLAGGEPVSLGSLRGKVLLIENVASLUGTTVRDYTQMN
ELQRRLGPRGLVVLGFPCNQFGHQENAKNEEILNSLKYVRPGGGFEPNFMLFEKCEVNGA
GAHPLFAFLREALPAPSDDATALMTDPKLITWSPVCRNDVAWNFEKFLVGPDGVPLRRYS
RRFQTIDIEPDIEALLSQGPSCA
>sp|P07305|H10_HUMAN Histone H1.0 OS=Homo sapiens OX=9606 GN=H1-0 PE=1 SV=3
MTENSTSAPAAKPKRAKASKKSTDHPKYSDMIVAAIQAEKNRAGSSRQSIQKYIKSHYKV
GENADSQIKLSIKRLVTTGVLKQTKGVGASGSFRLAKSDEPKKSVAFKKTKKEIKKVATP
KKASKPKKAASKAPTKKPKATPVKKAKKKLAATPKKAKKPKTVKAKPVKASKPKKAKPVK
PKAKSSAKRAGKKK
>sp|P07315|CRGC_HUMAN Gamma-crystallin C OS=Homo sapiens OX=9606 GN=CRYGC PE=1 SV=2
MGKITFYEDRAFQGRSYETTTDCPNLQPYFSRCNSIRVESGCWMLYERPNYQGQQYLLRR
GEYPDYQQWMGLSDSIRSCCLIPQTVSHRLRLYEREDHKGLMMELSEDCPSIQDRFHLSE
IRSLHVLEGCWVLYELPNYRGRQYLLRPQEYRRCQDWGAMDAKAGSLRRVVDLY
>sp|P07316|CRGB_HUMAN Gamma-crystallin B OS=Homo sapiens OX=9606 GN=CRYGB PE=1 SV=3
MGKITFYEDRAFQGRSYECTTDCPNLQPYFSRCNSIRVESGCWMIYERPNYQGHQYFLRR
GEYPDYQQWMGLSDSIRSCCLIPPHSGAYRMKIYDRDELRGQMSELTDDCISVQDRFHLT

EIHSLNVLEGSWILYEMPNYRGRQYLLRPGEYRRFLDWGAPNAKVGSLRRVMDLY
>sp|P07320|CRGD_HUMAN Gamma-crystallin D OS=Homo sapiens OX=9606 GN=CRYGD PE=1 SV=3
MGKITLYEDRGFQGRHYECSSDHPNLQPYLSRCNSARVDSGCWMLYEQPNYSGLQYFLRR
GDYADHQQWMGLSDSVRSCRLIPHSGSHRIRLYEREDYRGQMIEFTEDCSCLQDRFRFNE
IHSLNVLEGSWVLYELSNYRGRQYLLMPGDYRRYQDWGATNARVGSLRRVIDFS
>sp|P07355|ANXA2_HUMAN Annexin A2 OS=Homo sapiens OX=9606 GN=ANXA2 PE=1 SV=2
MSTVHEILCKLSLEGDHSTPPSAYGSVKAYTNFDAERDALNIETAIKTKGVDEVTIVNIL
TNRSNAQRQDIAFAYQRRTKKELASALKSALSGHLETVILGLLKTPAQYDASELKASMKG
LGTDEDSLIEIICSRTNQELQEINRVYKEMYKTDLEKDIISDTSGDFRKLMVALAKGRRA
EDGSVIDYELIDQDARDLYDAGVKRKGTDVPKWISIMTERSVPHLQKVFDRYKSYSPYDM
LESIRKEVKGDLENAFLNLVQCIQNKPLYFADRLYDSMKGKGTRDKVLIRIMVSRSEVDM
LKIRSEFKRKYGKSLYYYIQQDTKGDYQKALLYLCGGDD
>sp|P07437|TBB5_HUMAN Tubulin beta chain OS=Homo sapiens OX=9606 GN=TUBB PE=1 SV=2
MREIVHIQAGQCGNQIGAKFWEVISDEHGIDPTGTYHGDSDLQLDRISVYYNEATGGKYV
PRAILVDLEPGTMDSVRSGPFGQIFRPDNFVFGQSGAGNNWAKGHYTEGAELVDSVLDVV
RKEAESCDCLQGFQLTHSLGGGTGSGMGTLLISKIREEYPDRIMNTFSVVPSPKVSDTVV
EPYNATLSVHQLVENTDETYCIDNEALYDICFRTLKLTTPTYGDLNHLVSATMSGVTTCL
RFPGQLNADLRKLAVNMVPFPRLHFFMPGFAPLTSRGSQQYRALTVPELTQQVFDAKNMM
AACDPRHGRYLTVAAVFRGRMSMKEVDEQMLNVQNKNSSYFVEWIPNNVKTAVCDIPPRG
LKMAVTFIGNSTAIQELFKRISEQFTAMFRRKAFLHWYTGEGMDEMEFTEAESNMNDLVS
EYQQYQDATAEEEEDFGEEAEEEA
>sp|P07738|PMGE_HUMAN Bisphosphoglycerate mutase OS=Homo sapiens OX=9606 GN=BPGM PE=1 SV=2
MSKYKLIMLRHGEGAWNKENRFCSWVDQKLNSEGMEEARNCGKQLKALNFEFDLVFTSVL
NRSIHTAWLILEELGQEWVPVESSWRLNERHYGALIGLNREQMALNHGEEQVRLWRRSYN
VTPPPIEESHPYYQEIYNDRRYKVCDVPLDQLPRSESLKDVLERLLPYWNERIAPEVLRG
KTILISAHGNSSRALLKHLEGISDEDIINITLPTGVPILLELDENLRAVGPHQFLGDQEA
IQAAIKKVEDQGKVKQAKK
>sp|P07900|HS90A_HUMAN Heat shock protein HSP 90-alpha OS=Homo sapiens OX=9606 GN=HSP90AA1 PE=1 SV=5
MPEETQTQDQPMEEEEVETFAFQAEIAQLMSLIINTFYSNKEIFLRELISNSSDALDKIR
YESLTDPSKLDSGKELHINLIPNKQDRTLTIVDTGIGMTKADLINNLGTIAKSGTKAFME
ALQAGADISMIGQFGVGFYSAYLVAEKVTVITKHNDDEQYAWESSAGGSFTVRTDTGEPM
GRGTKVILHLKEDQTEYLEERRIKEIVKKHSQFIGYPITLFVEKERDKEVSDDEAEEKED
KEEEKEKEEKESEDKPEIEDVGSDEEEEKKDGDKKKKKKIKEKYIDQEELNKTKPIWTRN
PDDITNEEYGEFYKSLTNDWEDHLAVKHFSVEGQLEFRALLFVPRRAPFDLFENRKKKNN
IKLYVRRVFIMDNCEELIPEYLNFIRGVVDSEDLPLNISREMLQQSKILKVIRKNLVKKC
LELFTELAEDKENYKKFYEQFSKNIKLGIHEDSQNRKKLSELLRYYTSASGDEMVSLKDY
CTRMKENQKHIYYITGETKDQVANSAFVERLRKHGLEVIYMIEPIDEYCVQQLKEFEGKT
LVSVTKEGLELPEDEEEKKKQEEKKTKFENLCKIMKDILEKKVEKVVVSNRLVTSPCCIV
TSTYGWTANMERIMKAQALRDNSTMGYMAAKKHLEINPDHSIIETLRQKAEADKNDKSVK
DLVILLYETALLSSGFSLEDPQTHANRIYRMIKLGLGIDEDDPTADDTSAAVTEEMPPLE

GDDDTSRMEEVD
>sp|P07942|LAMB1_HUMAN Laminin subunit beta-1 OS=Homo sapiens OX=9606 GN=LAMB1 PE=1 SV=2
MGLLQLLAFSFLALCRARVRAQEPEFSYGCAEGSCYPATGDLLIGRAQKLSVTSTCGLHK
PEPYCIVSHLQEDKKCFICNSQDPYHETLNPDSHLIENVVTTFAPNRLKIWWQSENGVEN
VTIQLDLEAEFHFTHLIMTFKTFRPAAMLIERSSDFGKTWGVYRYFAYDCEASFPGISTG
PMKKVDDIICDSRYSDIEPSTEGEVIFRALDPAFKIEDPYSPRIQNLLKITNLRIKFVKL
HTLGDNLLDSRMEIREKYYYAVYDMVVRGNCFCYGHASECAPVDGFNEEVEGMVHGHCMC
RHNTKGLNCELCMDFYHDLPWRPAEGRNSNACKKCNCNEHSISCHFDMAVYLATGNVSGG
VCDDCQHNTMGRNCEQCKPFYYQHPERDIRDPNFCERCTCDPAGSQNEGICDSYTDFSTG
LIAGQCRCKLNVEGEHCDVCKEGFYDLSSEDPFGCKSCACNPLGTIPGGNPCDSETGHCY
CKRLVTGQHCDQCLPEHWGLSNDLDGCRPCDCDLGGALNNSCFAESGQCSCRPHMIGRQC
NEVEPGYYFATLDHYLYEAEEANLGPGVSIVERQYIQDRIPSWTGAGFVRVPEGAYLEFF
IDNIPYSMEYDILIRYEPQLPDHWEKAVITVQRPGRIPTSSRCGNTIPDDDNQVVSLSPG
SRYVVLPRPVCFEKGTNYTVRLELPQYTSSDSDVESPYTLIDSLVLMPYCKSLDIFTVGG
SGDGVVTNSAWETFQRYRCLENSRSVVKTPMTDVCRNIIFSISALLHQTGLACECDPQGS
LSSVCDPNGGQCQCRPNVVGRTCNRCAPGTFGFGPSGCKPCECHLQGSVNAFCNPVTGQC
HCFQGVYARQCDRCLPGHWGFPSCQPCQCNGHADDCDPVTGECLNCQDYTMGHNCERCLA
GYYGDPIIGSGDHCRPCPCPDGPDSGRQFARSCYQDPVTLQLACVCDPGYIGSRCDDCAS
GYFGNPSEVGGSCQPCQCHNNIDTTDPEACDKETGRCLKCLYHTEGEHCQFCRFGYYGDA
LQQDCRKCVCNYLGTVQEHCNGSDCQCDKATGQCLCLPNVIGQNCDRCAPNTWQLASGTG
CDPCNCNAAHSFGPSCNEFTGQCQCMPGFGGRTCSECQELFWGDPDVECRACDCDPRGIE
TPQCDQSTGQCVCVEGVEGPRCDKCTRGYSGVFPDCTPCHQCFALWDVIIAELTNRTHRF
LEKAKALKISGVIGPYRETVDSVERKVSEIKDILAQSPAAEPLKNIGNLFEEAEKLIKDV
TEMMAQVEVKLSDTTSQSNSTAKELDSLQTEAESLDNTVKELAEQLEFIKNSDIRGALDS
ITKYFQMSLEAEERVNASTTEPNSTVEQSALMRDRVEDVMMERESQFKEKQEEQARLLDE
LAGKLQSLDLSAAAEMTCGTPPGASCSETECGGPNCRTDEGERKCGGPGCGGLVTVAHNA
WQKAMDLDQDVLSALAEVEQLSKMVSEAKLRADEAKQSAEDILLKTNATKEKMDKSNEEL
RNLIKQIRNFLTQDSADLDSIEAVANEVLKMEMPSTPQQLQNLTEDIRERVESLSQVEVI
LQHSAADIARAEMLLEEAKRASKSATDVKVTADMVKEALEEAEKAQVAAEKAIKQADEDI
QGTQNLLTSIESETAASEETLFNASQRISELERNVEELKRKAAQNSGEAEYIEKVVYTVK
QSAEDVKKTLDGELDEKYKKVENLIAKKTEESADARRKAEMLQNEAKTLLAQANSKLQLL
KDLERKYEDNQRYLEDKAQELARLEGEVRSLLKDISQKVAVYSTCL
>sp|P08134|RHOC_HUMAN Rho-related GTP-binding protein RhoC OS=Homo sapiens OX=9606 GN=RHOC PE=1 SV=1
MAAIRKKLVIVGDGACGKTCLLIVFSKDQFPEVYVPTVFENYIADIEVDGKQVELALWDT
AGQEDYDRLRPLSYPDTDVILMCFSIDSPDSLENIPEKWTPEVKHFCPNVPIILVGNKKD
LRQDEHTRRELAKMKQEPVRSEEGRDMANRISAFGYLECSAKTKEGVREVFEMATRAGLQ
VRKNKRRRGCPIL
>sp|P08195|4F2_HUMAN Amino acid transporter heavy chain SLC3A2 OS=Homo sapiens OX=9606 GN=SLC3A2 PE=1 SV=3
MELQPPEASIAVVSIPRQLPGSHSEAGVQGLSAGDDSELGSHCVAQTGLELLASGDPLPS
ASQNAEMIETGSDCVTQAGLQLLASSDPPALASKNAEVTGTMSQDTEVDMKEVELNELEP
EKQPMNAASGAAMSLAGAEKNGLVKIKVAEDEAEAAAAAKFTGLSKEELLKVAGSPGWVR

TRWALLLLFWLGWLGMLAGAVVIIVRAPRCRELPAQKWWHTGALYRIGDLQAFQGHGAGN
LAGLKGRLDYLSSLKVKGLVLGPIHKNQKDDVAQTDLLQIDPNFGSKEDFDSLLQSAKKK
SIRVILDLTPNYRGENSWFSTQVDTVATKVKDALEFWLQAGVDGFQVRDIENLKDASSFL
AEWQNITKGFSEDRLLIAGTNSSDLQQILSLLESNKDLLLTSSYLSDSGSTGEHTKSLVT
QYLNATGNRWCSWSLSQARLLTSFLPAQLLRLYQLMLFTLPGTPVFSYGDEIGLDAAALP
GQPMEAPVMLWDESSFPDIPGAVSANMTVKGQSEDPGSLLSLFRRLSDQRSKERSLLHGD
FHAFSAGPGLFSYIRHWDQNERFLVVLNFGDVGLSAGLQASDLPASASLPAKADLLLSTQ
PGREEGSPLELERLKLEPHEGLLLRFPYAA
>sp|P08237|PFKAM_HUMAN ATP-dependent 6-phosphofructokinase, muscle type OS=Homo sapiens OX=9606 GN=PFKM PE=1 SV=2
MTHEEHHAAKTLGIGKAIAVLTSGGDAQGMNAAVRAVVRVGIFTGARVFFVHEGYQGLVD
GGDHIKEATWESVSMMLQLGGTVIGSARCKDFREREGRLRAAYNLVKRGITNLCVIGGDG
SLTGADTFRSEWSDLLSDLQKAGKITDEEATKSSYLNIVGLVGSIDNDFCGTDMTIGTDS
ALHRIMEIVDAITTTAQSHQRTFVLEVMGRHCGYLALVTSLSCGADWVFIPECPPDDDWE
EHLCRRLSETRTRGSRLNIIIVAEGAIDKNGKPITSEDIKNLVVKRLGYDTRVTVLGHVQ
RGGTPSAFDRILGSRMGVEAVMALLEGTPDTPACVVSLSGNQAVRLPLMECVQVTKDVTK
AMDEKKFDEALKLRGRSFMNNWEVYKLLAHVRPPVSKSGSHTVAVMNVGAPAAGMNAAVR
STVRIGLIQGNRVLVVHDGFEGLAKGQIEEAGWSYVGGWTGQGGSKLGTKRTLPKKSFEQ
ISANITKFNIQGLVIIGGFEAYTGGLELMEGRKQFDELCIPFVVIPATVSNNVPGSDFSV
GADTALNTICTTCDRIKQSAAGTKRRVFIIETMGGYCGYLATMAGLAAGADAAYIFEEPF
TIRDLQANVEHLVQKMKTTVKRGLVLRNEKCNENYTTDFIFNLYSEEGKGIFDSRKNVLG
HMQQGGSPTPFDRNFATKMGAKAMNWMSGKIKESYRNGRIFANTPDSGCVLGMRKRALVF
QPVAELKDQTDFEHRIPKEQWWLKLRPILKILAKYEIDLDTSDHAHLEHITRKRSGEAAV
>sp|P08238|HS90B_HUMAN Heat shock protein HSP 90-beta OS=Homo sapiens OX=9606 GN=HSP90AB1 PE=1 SV=4
MPEEVHHGEEEVETFAFQAEIAQLMSLIINTFYSNKEIFLRELISNASDALDKIRYESLT
DPSKLDSGKELKIDIIPNPQERTLTLVDTGIGMTKADLINNLGTIAKSGTKAFMEALQAG
ADISMIGQFGVGFYSAYLVAEKVVVITKHNDDEQYAWESSAGGSFTVRADHGEPIGRGTK
VILHLKEDQTEYLEERRVKEVVKKHSQFIGYPITLYLEKEREKEISDDEAEEEKGEKEEE
DKDDEEKPKIEDVGSDEEDDSGKDKKKKTKKIKEKYIDQEELNKTKPIWTRNPDDITQEE
YGEFYKSLTNDWEDHLAVKHFSVEGQLEFRALLFIPRRAPFDLFENKKKKNNIKLYVRRV
FIMDSCDELIPEYLNFIRGVVDSEDLPLNISREMLQQSKILKVIRKNIVKKCLELFSELA
EDKENYKKFYEAFSKNLKLGIHEDSTNRRRLSELLRYHTSQSGDEMTSLSEYVSRMKETQ
KSIYYITGESKEQVANSAFVERVRKRGFEVVYMTEPIDEYCVQQLKEFDGKSLVSVTKEG
LELPEDEEEKKKMEESKAKFENLCKLMKEILDKKVEKVTISNRLVSSPCCIVTSTYGWTA
NMERIMKAQALRDNSTMGYMMAKKHLEINPDHPIVETLRQKAEADKNDKAVKDLVVLLFE
TALLSSGFSLEDPQTHSNRIYRMIKLGLGIDEDEVAAEEPNAAVPDEIPPLEGDEDASRM
EEVD
>sp|P08572|CO4A2_HUMAN Collagen alpha-2(IV) chain OS=Homo sapiens OX=9606 GN=COL4A2 PE=1 SV=4
MGRDQRAVAGPALRRWLLLGTVTVGFLAQSVLAGVKKFDVPCGGRDCSGGCQCYPEKGGR
GQPGPVGPQGYNGPPGLQGFPGLQGRKGDKGERGAPGVTGPKGDVGARGVSGFPGADGIP
GHPGQGGPRGRPGYDGCNGTQGDSGPQGPPGSEGFTGPPGPQGPKGQKGEPYALPKEERD
RYRGEPGEPGLVGFQGPPGRPGHVGQMGPVGAPGRPGPPGPPGPKGQQGNRGLGFYGVKG

EKGDVGQPGPNGIPSDTLHPIIAPTGVTFHPDQYKGEKGSEGEPGIRGISLKGEEGIMGF
PGLRGYPGLSGEKGSPGQKGSRGLDGYQGPDGPRGPKGEAGDPGPPGLPAYSPHPSLAKG
ARGDPGFPGAQGEPGSQGEPGDPGLPGPPGLSIGDGDQRRGLPGEMGPKGFIGDPGIPAL
YGGPPGPDGKRGPPGPPGLPGPPGPDGFLFGLKGAKGRAGFPGLPGSPGARGPKGWKGDA
GECRCTEGDEAIKGLPGLPGPKGFAGINGEPGRKGDRGDPGQHGLPGFPGLKGVPGNIGA
PGPKGAKGDSRTITTKGERGQPGVPGVPGMKGDDGSPGRDGLDGFPGLPGPPGDGIKGPP
GDPGYPGIPGTKGTPGEMGPPGLGLPGLKGQRGFPGDAGLPGPPGFLGPPGPAGTPGQID
CDTDVKRAVGGDRQEAIQPGCIGGPKGLPGLPGPPGPTGAKGLRGIPGFAGADGGPGPRG
LPGDAGREGFPGPPGFIGPRGSKGAVGLPGPDGSPGPIGLPGPDGPPGERGLPGEVLGAQ
PGPRGDAGVPGQPGLKGLPGDRGPPGFRGSQGMPGMPGLKGQPGLPGPSGQPGLYGPPGL
HGFPGAPGQEGPLGLPGIPGREGLPGDRGDPGDTGAPGPVGMKGLSGDRGDAGFTGEQGH
PGSPGFKGIDGMPGTPGLKGDRGSPGMDGFQGMPGLKGRPGFPGSKGEAGFFGIPGLKGL
AGEPGFKGSRGDPGPPGPPPVILPGMKDIKGEKGDEGPMGLKGYLGAKGIQGMPGIPGLS
GIPGLPGRPGHIKGVKGDIGVPGIPGLPGFPGVAGPPGITGFPGFIGSRGDKGAPGRAGL
YGEIGATGDFGDIGDTINLPGRPGLKGERGTTGIPGLKGFFGEKGTEGDIGFPGITGVTG
VQGPPGLKGQTGFPGLTGPPGSQGELGRIGLPGGKGDDGWPGAPGLPGFPGLRGIRGLHG
LPGTKGFPGSPGSDIHGDPGFPGPPGERGDPGEANTLPGPVGVPGQKGDQGAPGERGPPG
SPGLQGFPGITPPSNISGAPGDKGAPGIFGLKGYRGPPGPPGSAALPGSKGDTGNPGAPG
TPGTKGWAGDSGPQGRPGVFGLPGEKGPRGEQGFMGNTGPTGAVGDRGPKGPKGDPGFPG
APGTVGAPGIAGIPQKIAVQPGTVGPQGRRGPPGAPGEMGPQGPPGEPGFRGAPGKAGPQ
GRGGVSAVPGFRGDEGPIGHQGPIGQEGAPGRPGSPGLPGMPGRSVSIGYLLVKHSQTDQ
EPMCPVGMNKLWSGYSLLYFEGQEKAHNQDLGLAGSCLARFSTMPFLYCNPGDVCYYASR
NDKSYWLSTTAPLPMMPVAEDEIKPYISRCSVCEAPAIAIAVHSQDVSIPHCPAGWRSLW
IGYSFLMHTAAGDEGGGQSLVSPGSCLEDFRATPFIECNGGRGTCHYYANKYSFWLTTIP
EQSFQGSPSADTLKAGLIRTHISRCQVCMKNL
>sp|P08581|MET_HUMAN Hepatocyte growth factor receptor OS=Homo sapiens OX=9606
GN=MET PE=1 SV=4
MKAPAVLAPGILVLLFTLVQRSNGECKEALAKSEMNVNMKYQLPNFTAETPIQNVILHEH
HIFLGATNYIYVLNEEDLQKVAEYKTGPVLEHPDCFPCQDCSSKANLSGGVWKDNINMAL
VVDTYYDDQLISCGSVNRGTCQRHVFPHNHTADIQSEVHCIFSPQIEEPSQCPDCVVSAL
GAKVLSSVKDRFINFFVGNTINSSYFPDHPLHSISVRRLKETKDGFMFLTDQSYIDVLPE
FRDSYPIKYVHAFESNNFIYFLTVQRETLDAQTFHTRIIRFCSINSGLHSYMEMPLECIL
TEKRKKRSTKKEVFNILQAAYVSKPGAQLARQIGASLNDDILFGVFAQSKPDSAEPMDRS
AMCAFPIKYVNDFFNKIVNKNNVRCLQHFYGPNHEHCFNRTLLRNSSGCEARRDEYRTEF
TTALQRVDLFMGQFSEVLLTSISTFIKGDLTIANLGTSEGRFMQVVVSRSGPSTPHVNFL
LDSHPVSPEVIVEHTLNQNGYTLVITGKKITKIPLNGLGCRHFQSCSQCLSAPPFVQCGW
CHDKCVRSEECLSGTWTQQICLPAIYKVFPNSAPLEGGTRLTICGWDFGFRRNNKFDLKK
TRVLLGNESCTLTLSESTMNTLKCTVGPAMNKHFNMSIIISNGHGTTQYSTFSYVDPVIT
SISPKYGPMAGGTLLTLTGNYLNSGNSRHISIGGKTCTLKSVSNSILECYTPAQTISTEF
AVKLKIDLANRETSIFSYREDPIVYEIHPTKSFISGGSTITGVGKNLNSVSVPRMVINVH
EAGRNFTVACQHRSNSEIICCTTPSLQQLNLQLPLKTKAFFMLDGILSKYFDLIYVHNPV
FKPFEKPVMISMGNENVLEIKGNDIDPEAVKGEVLKVGNKSCENIHLHSEAVLCTVPNDL
LKLNSELNIEWKQAISSTVLGKVIVQPDQNFTGLIAGVVSISTALLLLLGFFLWLKKRKQ
IKDLGSELVRYDARVHTPHLDRLVSARSVSPTTEMVSNESVDYRATFPEDQFPNSSQNGS

CRQVQYPLTDMSPILTSGDSDISSPLLQNTVHIDLSALNPELVQAVQHVVIGPSSLIVHF
NEVIGRGHFGCVYHGTLLDNDGKKIHCAVKSLNRITDIGEVSQFLTEGIIMKDFSHPNVL
SLLGICLRSEGSPLVVLPYMKHGDLRNFIRNETHNPTVKDLIGFGLQVAKGMKYLASKKF
VHRDLAARNCMLDEKFTVKVADFGLARDMYDKEYYSVHNKTGAKLPVKWMALESLQTQKF
TTKSDVWSFGVLLWELMTRGAPPYPDVNTFDITVYLLQGRRLLQPEYCPDPLYEVMLKCW
HPKAEMRPSFSELVSRISAIFSTFIGEHYVHVNATYVNVKCVAPYPSLLSSEDNADDEVD
TRPASFWETS
>sp|P08670|VIME_HUMAN Vimentin OS=Homo sapiens OX=9606 GN=VIM PE=1 SV=4
MSTRSVSSSSYRRMFGGPGTASRPSSSRSYVTTSTRTYSLGSALRPSTSRSLYASSPGGV
YATRSSAVRLRSSVPGVRLLQDSVDFSLADAINTEFKNTRTNEKVELQELNDRFANYIDK
VRFLEQQNKILLAELEQLKGQGKSRLGDLYEEEMRELRRQVDQLTNDKARVEVERDNLAE
DIMRLREKLQEEMLQREEAENTLQSFRQDVDNASLARLDLERKVESLQEEIAFLKKLHEE
EIQELQAQIQEQHVQIDVDVSKPDLTAALRDVRQQYESVAAKNLQEAEEWYKSKFADLSE
AANRNNDALRQAKQESTEYRRQVQSLTCEVDALKGTNESLERQMREMEENFAVEAANYQD
TIGRLQDEIQNMKEEMARHLREYQDLLNVKMALDIEIATYRKLLEGEESRISLPLPNFSS
LNLRETNLDSLPLVDTHSKRTLLIKTVETRDGQVINETSQHHDDLE
>sp|P08708|RS17_HUMAN Small ribosomal subunit protein eS17 OS=Homo sapiens OX=9606 GN=RPS17 PE=1 SV=2
MGRVRTKTVKKAARVIIEKYYTRLGNDFHTNKRVCEEIAIIPSKKLRNKIAGYVTHLMKR
IQRGPVRGISIKLQEEERERRDNYVPEVSALDQEIIEVDPDTKEMLKLLDFGSLSNLQVT
QPTVGMNFKTPRGPV
>sp|P08754|GNAI3_HUMAN Guanine nucleotide-binding protein G(i) subunit alpha-3 OS=Homo sapiens OX=9606 GN=GNAI3 PE=1 SV=3
MGCTLSAEDKAAVERSKMIDRNLREDGEKAAKEVKLLLLGAGESGKSTIVKQMKIIHEDG
YSEDECKQYKVVVYSNTIQSIIAIIRAMGRLKIDFGEAARADDARQLFVLAGSAEEGVMT
PELAGVIKRLWRDGGVQACFSRSREYQLNDSASYYLNDLDRISQSNYIPTQQDVLRTRVK
TTGIVETHFTFKDLYFKMFDVGGQRSERKKWIHCFEGVTAIIFCVALSDYDLVLAEDEEM
NRMHESMKLFDSICNNKWFTETSIILFLNKKDLFEEKIKRSPLTICYPEYTGSNTYEEAA
AYIQCQFEDLNRRKDTKEIYTHFTCATDTKNVQFVFDAVTDVIIKNNLKECGLY
>sp|P08758|ANXA5_HUMAN Annexin A5 OS=Homo sapiens OX=9606 GN=ANXA5 PE=1 SV=2
MAQVLRGTVTDFPGFDERADAETLRKAMKGLGTDEESILTLLTSRSNAQRQEISAAFKTL
FGRDLLDDLKSELTGKFEKLIVALMKPSRLYDAYELKHALKGAGTNEKVLTEIIASRTPE
ELRAIKQVYEEEYGSSLEDDVVGDTSGYYQRMLVVLLQANRDPDAGIDEAQVEQDAQALF
QAGELKWGTDEEKFITIFGTRSVSHLRKVFDKYMTISGFQIEETIDRETSGNLEQLLLAV
VKSIRSIPAYLAETLYYAMKGAGTDDHTLIRVMVSRSEIDLFNIRKEFRKNFATSLYSMI
KGDTSGDYKKALLLLCGEDD
>sp|P08779|K1C16_HUMAN Keratin, type I cytoskeletal 16 OS=Homo sapiens OX=9606 GN=KRT16 PE=1 SV=4
MTTCSRQFTSSSSMKGSCGIGGGIGGGSSRISSVLAGGSCRAPSTYGGGLSVSSRFSSGG
ACGLGGGYGGGFSSSSSFGSGFGGGYGGGLGAGFGGGLGAGFGGGFAGGDGLLVGSEKVT
MQNLNDRLASYLDKVRALEEANADLEVKIRDWYQRQRPSEIKDYSPYFKTIEDLRNKIIA
ATIENAQPILQIDNARLAADDFRTKYEHELALRQTVEADVNGLRRVLDELTLARTDLEMQ
IEGLKEELAYLRKNHEEEMLALRGQTGGDVNVEMDAAPGVDLSRILNEMRDQYEQMAEKN
RRDAETWFLSKTEELNKEVASNSELVQSSRSEVTELRRVLQGLEIELQSQLSMKASLENS

LEETKGRYCMQLSQIQGLIGSVEEQLAQLRCEMEQQSQEYQILLDVKTRLEQEIATYRRL
LEGEDAHLSSQQASGQSYSSREVFTSSSSSSSRQTRPILKEQSSSSFSQGQSS
>sp|P09104|ENOG_HUMAN Gamma-enolase OS=Homo sapiens OX=9606 GN=ENO2 PE=1 SV=3
MSIEKIWAREILDSRGNPTVEVDLYTAKGLFRAAVPSGASTGIYEALELRDGDKQRYLGK
GVLKAVDHINSTIAPALISSGLSVVEQEKLDNLMLELDGTENKSKFGANAILGVSLAVCK
AGAAERELPLYRHIAQLAGNSDLILPVPAFNVINGGSHAGNKLAMQEFMILPVGAESFRD
AMRLGAEVYHTLKGVIKDKYGKDATNVGDEGGFAPNILENSEALELVKEAIDKAGYTEKI
VIGMDVAASEFYRDGKYDLDFKSPTDPSRYITGDQLGALYQDFVRDYPVVSIEDPFDQDD
WAAWSKFTANVGIQIVGDDLTVTNPKRIERAVEEKACNCLLLKVNQIGSVTEAIQACKLA
QENGWGVMVSHRSGETEDTFIADLVVGLCTGQIKTGAPCRSERLAKYNQLMRIEEELGDE
ARFAGHNFRNPSVL
>sp|P09211|GSTP1_HUMAN Glutathione S-transferase P OS=Homo sapiens OX=9606
GN=GSTP1 PE=1 SV=2
MPPYTVVYFPVRGRCAALRMLLADQGQSWKEEVVTVETWQEGSLKASCLYGQLPKFQDGD
LTLYQSNTILRHLGRTLGLYGKDQQEAALVDMVNDGVEDLRCKYISLIYTNYEAGKDDYV
KALPGQLKPFETLLSQNQGGKTFIVGDQISFADYNLLDLLLIHEVLAPGCLDAFPLLSAY
VGRLSARPKLKAFLASPEYVNLPINGNGKQ
>sp|P09382|LEG1_HUMAN Galectin-1 OS=Homo sapiens OX=9606 GN=LGALS1 PE=1 SV=2
MACGLVASNLNLKPGECLRVRGEVAPDAKSFVLNLGKDSNNLCLHFNPRFNAHGDANTIV
CNSKDGGAWGTEQREAVFPFQPGSVAEVCITFDQANLTVKLPDGYEFKFPNRLNLEAINY
MAADGDFKIKCVAFD
>sp|P09417|DHPR_HUMAN Dihydropteridine reductase OS=Homo sapiens OX=9606 GN=QDPR
PE=1 SV=2
MAAAAAAGEARRVLVYGGRGALGSRCVQAFRARNWWVASVDVVENEEASASIIVKMTDSF
TEQADQVTAEVGKLLGEEKVDAILCVAGGWAGGNAKSKSLFKNCDLMWKQSIWTSTISSH
LATKHLKEGGLLTLAGAKAALDGTPGMIGYGMAKGAVHQLCQSLAGKNSGMPPGAAAIAV
LPVTLDTPMNRKSMPEADFSSWTPLEFLVETFHDWITGKNRPSSGSLIQVVTTEGRTELT
PAYF
>sp|P09488|GSTM1_HUMAN Glutathione S-transferase Mu 1 OS=Homo sapiens OX=9606
GN=GSTM1 PE=1 SV=3
MPMILGYWDIRGLAHAIRLLLEYTDSSYEEKKYTMGDAPDYDRSQWLNEKFKLGLDFPNL
PYLIDGAHKITQSNAILCYIARKHNLCGETEEEKIRVDILENQTMDNHMQLGMICYNPEF
EKLKPKYLEELPEKLKLYSEFLGKRPWFAGNKITFVDFLVYDVLDLHRIFEPKCLDAFPN
LKDFISRFEGLEKISAYMKSSRFLPRPVFSKMAVWGNK
>sp|P09543|CN37_HUMAN 2',3'-cyclic-nucleotide 3'-phosphodiesterase OS=Homo sapiens
OX=9606 GN=CNP PE=1 SV=2
MNRGFSRKSHTFLPKIFFRKMSSSGAKDKPELQFPFLQDEDTVATLLECKTLFILRGLPG
SGKSTLARVIVDKYRDGTKMVSADAYKITPGARGAFSEEYKRLDEDLAAYCRRRDIRILV
LDDTNHERERLEQLFEMADQYQYQVVLVEPKTAWRLDCAQLKEKNQWQLSADDLKKLKPG
LEKDFLPLYFGWFLTKKSSETLRKAGQVFLEELGNHKAFKKELRQFVPGDEPREKMDLVT
YFGKRPPGVLHCTTKFCDYGKAPGAEEYAQQDVLKKSYSKAFTLTISALFVTPKTTGARV
ELSEQQLQLWPSDVDKLSPTDNLPRGSRAHITLGCAADVEAVQTGLDLLEILRQEKGGSR
GEEVGELSRGKLYSLGNGRWMLTLAKNMEVRAIFTGYYGKGKPVPTQGSRKGGALQSCTI
I

>sp|P09936|UCHL1_HUMAN Ubiquitin carboxyl-terminal hydrolase isozyme L1 OS=Homo sapiens OX=9606 GN=UCHL1 PE=1 SV=2
MQLKPMEINPEMLNKVLSRLGVAGQWRFVDVLGLEEESLGSVPAPACALLLLFPLTAQHE
NFRKKQIEELKGQEVSPKVYFMKQTIGNSCGTIGLIHAVANNQDKLGFEDGSVLKQFLSE
TEKMSPEDRAKCFEKNEAIQAAHDAVAQEGQCRVDDKVNFHFILFNNVDGHLYELDGRMP
FPVNHGASSEDTLLKDAAKVCREFTEREQGEVRFSAVALCKAA
>sp|P09960|LKHA4_HUMAN Leukotriene A-4 hydrolase OS=Homo sapiens OX=9606 GN=LTA4H PE=1 SV=2
MPEIVDTCSLASPASVCRTKHLHLRCSVDFTRRTLTGTAALTVQSQEDNLRSLVLDTKDL
TIEKVVINGQEVKYALGERQSYKGSPMEISLPIALSKNQEIVIEISFETSPKSSALQWLT
PEQTSGKEHPYLFSQCQAIHCRAILPCQDTPSVKLTYTAEVSVPKELVALMSAIRDGETP
DPEDPSRKIYKFIQKVPIPCYLIALVVGALESRQIGPRTLVWSEKEQVEKSAYEFSETES
MLKIAEDLGGPYVWGQYDLLVLPPSFPYGGMENPCLTFVTPTLLAGDKSLSNVIAHEISH
SWTGNLVTNKTWDHFWLNEGHTVYLERHICGRLFGEKFRHFNALGGWGELQNSVKTFGET
HPFTKLVVDLTDIDPDVAYSSVPYEKGFALLFYLEQLLGGPEIFLGFLKAYVEKFSYKSI
TTDDWKDFLYSYFKDKVDVLNQVDWNAWLYSPGLPPIKPNYDMTLTNACIALSQRWITAK
EDDLNSFNATDLKDLSSHQLNEFLAQTLQRAPLPLGHIKRMQEVYNFNAINNSEIRFRWL
RLCIQSKWEDAIPLALKMATEQGRMKFTRPLFKDLAAFDKSHDQAVRTYQEHKASMHPVT
AMLVGKDLKVD
>sp|P09972|ALDOC_HUMAN Fructose-bisphosphate aldolase C OS=Homo sapiens OX=9606 GN=ALDOC PE=1 SV=2
MPHSYPALSAEQKKELSDIALRIVAPGKGILAADESVGSMAKRLSQIGVENTEENRRLYR
QVLFSADDRVKKCIGGVIFFHETLYQKDDNGVPFVRTIQDKGIVVGIKVDKGVVPLAGTD
GETTTQGLDGLSERCAQYKKDGADFAKWRCVLKISERTPSALAILENANVLARYASICQQ
NGIVPIVEPEILPDGDHDLKRCQYVTEKVLAAVYKALSDHHVYLEGTLLKPNMVTPGHAC
PIKYTPEEIAMATVTALRRTVPPAVPGVTFLSGGQSEEEASFNLNAINRCPLPRPWALTF
SYGRALQASALNAWRGQRDNAGAATEEFIKRAEVNGLAAQGKYEGSGEDGGAAAQSLYIA
NHAY
>sp|P0C870|JMJD7_HUMAN Bifunctional peptidase and (3S)-lysyl hydroxylase JMJD7 OS=Homo sapiens OX=9606 GN=JMJD7 PE=1 SV=1
MAEAALEAVRSELREFPAAARELCVPLAVPYLDKPPTPLHFYRDWVCPNRPCIIRNALQH
WPALQKWSLPYFRATVGSTEVSVAVTPDGYADAVRGDRFMMPAERRLPLSFVLDVLEGRA
QHPGVLYVQKQCSNLPSELPQLLPDLESHVPWASEALGKMPDAVNFWLGEAAAVTSLHKD
HYENLYCVVSGEKHFLFHPPSDRPFIPYELYTPATYQLTEEGTFKVVDEEAMEKVPWIPL
DPLAPDLARYPSYSQAQALRCTVRAGEMLYLPALWFHHVQQSQGCIAVNFWYDMEYDLKY
SYFQLLDSLTKASGLD
>sp|P0CG47|UBB_HUMAN Polyubiquitin-B OS=Homo sapiens OX=9606 GN=UBB PE=1 SV=1
MQIFVKTLTGKTITLEVEPSDTIENVKAKIQDKEGIPPDQQRLIFAGKQLEDGRTLSDYN
IQKESTLHLVLRLRGGMQIFVKTLTGKTITLEVEPSDTIENVKAKIQDKEGIPPDQQRLI
FAGKQLEDGRTLSDYNIQKESTLHLVLRLRGGMQIFVKTLTGKTITLEVEPSDTIENVKA
KIQDKEGIPPDQQRLIFAGKQLEDGRTLSDYNIQKESTLHLVLRLRGGC
>sp|P0CG48|UBC_HUMAN Polyubiquitin-C OS=Homo sapiens OX=9606 GN=UBC PE=1 SV=3
MQIFVKTLTGKTITLEVEPSDTIENVKAKIQDKEGIPPDQQRLIFAGKQLEDGRTLSDYN
IQKESTLHLVLRLRGGMQIFVKTLTGKTITLEVEPSDTIENVKAKIQDKEGIPPDQQRLI

FAGKQLEDGRTLSDYNIQKESTLHLVLRLRGGMQIFVKTLTGKTITLEVEPSDTIENVKA
KIQDKEGIPPDQQRLIFAGKQLEDGRTLSDYNIQKESTLHLVLRLRGGMQIFVKTLTGKT
ITLEVEPSDTIENVKAKIQDKEGIPPDQQRLIFAGKQLEDGRTLSDYNIQKESTLHLVLR
LRGGMQIFVKTLTGKTITLEVEPSDTIENVKAKIQDKEGIPPDQQRLIFAGKQLEDGRTL
SDYNIQKESTLHLVLRLRGGMQIFVKTLTGKTITLEVEPSDTIENVKAKIQDKEGIPPDQ
QRLIFAGKQLEDGRTLSDYNIQKESTLHLVLRLRGGMQIFVKTLTGKTITLEVEPSDTIE
NVKAKIQDKEGIPPDQQRLIFAGKQLEDGRTLSDYNIQKESTLHLVLRLRGGMQIFVKTL
TGKTITLEVEPSDTIENVKAKIQDKEGIPPDQQRLIFAGKQLEDGRTLSDYNIQKESTLH
LVLRLRGGMQIFVKTLTGKTITLEVEPSDTIENVKAKIQDKEGIPPDQQRLIFAGKQLED
GRTLSDYNIQKESTLHLVLRLRGGV
>sp|P0DP57|SLUR2_HUMAN Secreted Ly-6/uPAR domain-containing protein 2 OS=Homo sapiens OX=9606 GN=SLURP2 PE=1 SV=1
MQLGTGLLLAAVLSLQLAAAEAIWCHQCTGFGGCSHGSRCLRDSTHCVTTATRVLSNTED
LPLVTKMCHIGCPDIPSLGLGPYVSIACCQTSLCNHD
>sp|P0DP58|LYNX1_HUMAN Ly-6/neurotoxin-like protein 1 OS=Homo sapiens OX=9606 GN=LYNX1 PE=1 SV=1
MTPLLTLILVVLMGLPLAQALDCHVCAYNGDNCFNPMRCPAMVAYCMTTRTYYTPTRMKV
SKSCVPRCFETVYDGYSKHASTTSCCQYDLCNGTGLATPATLALAPILLATLWGLL
>sp|P10114|RAP2A_HUMAN Ras-related protein Rap-2a OS=Homo sapiens OX=9606 GN=RAP2A PE=1 SV=1
MREYKVVVLGSGGVGKSALTVQFVTGTFIEKYDPTIEDFYRKEIEVDSSPSVLEILDTAG
TEQFASMRDLYIKNGQGFILVYSLVNQQSFQDIKPMRDQIIRVKRYEKVPVILVGNKVDL
ESEREVSSSEGRALAEEWGCPFMETSAKSKTMVDELFAEIVRQMNYAAQPDKDDPCCSAC
NIQ
>sp|P10155|RO60_HUMAN RNA-binding protein RO60 OS=Homo sapiens OX=9606 GN=RO60 PE=1 SV=2
MEESVNQMQPLNEKQIANSQDGYVWQVTDMNRLHRFLCFGSEGGTYYIKEQKLGLENAEA
LIRLIEDGRGCEVIQEIKSFSQEGRTTKQEPMLFALAICSQCSDISTKQAAFKAVSEVCR
IPTHLFTFIQFKKDLKESMKCGMWGRALRKAIADWYNEKGGMALALAVTKYKQRNGWSHK
DLLRLSHLKPSSEGLAIVTKYITKGWKEVHELYKEKALSVETEKLLKYLEAVEKVKRTRD
ELEVIHLIEEHRLVREHLLTNHLKSKEVWKALLQEMPLTALLRNLGKMTANSVLEPGNSE
VSLVCEKLCNEKLLKKARIHPFHILIALETYKTGHGLRGKLKWRPDEEILKALDAAFYKT
FKTVEPTGKRFLLAVDVSASMNQRVLGSILNASTVAAAMCMVVTRTEKDSYVVAFSDEMV
PCPVTTDMTLQQVLMAMSQIPAGGTDCSLPMIWAQKTNTPADVFIVFTDNETFAGGVHPA
IALREYRKKMDIPAKLIVCGMTSNGFTIADPDDRGMLDMCGFDTGALDVIRNFTLDMI
>sp|P10644|KAP0_HUMAN cAMP-dependent protein kinase type I-alpha regulatory subunit OS=Homo sapiens OX=9606 GN=PRKAR1A PE=1 SV=1
MESGSTAASEEARSLRECELYVQKHNIQALLKDSIVQLCTARPERPMAFLREYFERLEKE
EAKQIQNLQKAGTRTDSREDEISPPPPNPVVKGRRRRGAISAEVYTEEDAASYVRKVIPK
DYKTMAALAKAIEKNVLFSHLDDNERSDIFDAMFSVSFIAGETVIQQGDEGDNFYVIDQG
ETDVYVNNEWATSVGEGGSFGELALIYGTPRAATVKAKTNVKLWGIDRDSYRRILMGSTL
RKRKMYEEFLSKVSILESLDKWERLTVADALEPVQFEDGQKIVVQGEPGDEFFIILEGSA
AVLQRRSENEEFVEVGRLGPSDYFGEIALLMNRPRAATVVARGPLKCVKLDRPRFERVLG
PCSDILKRNIQQYNSFVSLSV

>sp|P10768|ESTD_HUMAN S-formylglutathione hydrolase OS=Homo sapiens OX=9606 GN=ESD PE=1 SV=2
MALKQISSNKCFGGLQKVFEHDSVELNCKMKFAVYLPPKAETGKCPALYWLSGLTCTEQN
FISKSGYHQSASEHGLVVIAPDTSPRGCNIKGEDESWDFGTGAGFYVDATEDPWKTNYRM
YSYVTEELPQLINANFPVDPQRMSIFGHSMGGHGALICALKNPGKYKSVSAFAPICNPVL
CPWGKKAFSGYLGTDQSKWKAYDATHLVKSYPGSQLDILIDQGKDDQFLLDGQLLPDNFI
AACTEKKIPVVFRLQEGYDHSYYFIATFITDHIRHHAKYLNA
>sp|P10909|CLUS_HUMAN Clusterin OS=Homo sapiens OX=9606 GN=CLU PE=1 SV=1
MMKTLLLFVGLLLTWESGQVLGDQTVSDNELQEMSNQGSKYVNKEIQNAVNGVKQIKTLI
EKTNEERKTLLSNLEEAKKKKEDALNETRESETKLKELPGVCNETMMALWEECKPCLKQT
CMKFYARVCRSGSGLVGRQLEEFLNQSSPFYFWMNGDRIDSLLENDRQQTHMLDVMQDHF
SRASSIIDELFQDRFFTREPQDTYHYLPFSLPHRRPHFFFPKSRIVRSLMPFSPYEPLNF
HAMFQPFLEMIHEAQQAMDIHFHSPAFQHPPTEFIREGDDDRTVCREIRHNSTGCLRMKD
QCDKCREILSVDCSTNNPSQAKLRRELDESLQVAERLTRKYNELLKSYQWKMLNTSSLLE
QLNEQFNWVSRLANLTQGEDQYYLRVTTVASHTSDSDVPSGVTEVVVKLFDSDPITVTVP
VEVSRKNPKFMETVAEKALQEYRKKHREE
>sp|P11047|LAMC1_HUMAN Laminin subunit gamma-1 OS=Homo sapiens OX=9606 GN=LAMC1 PE=1 SV=3
MRGSHRAAPALRPRGRLWPVLAVLAAAAAAGCAQAAMDECTDEGGRPQRCMPEFVNAAFN
VTVVATNTCGTPPEEYCVQTGVTGVTKSCHLCDAGQPHLQHGAAFLTDYNNQADTTWWQS
QTMLAGVQYPSSINLTLHLGKAFDITYVRLKFHTSRPESFAIYKRTREDGPWIPYQYYSG
SCENTYSKANRGFIRTGGDEQQALCTDEFSDISPLTGGNVAFSTLEGRPSAYNFDNSPVL
QEWVTATDIRVTLNRLNTFGDEVFNDPKVLKSYYYAISDFAVGGRCKCNGHASECMKNEF
DKLVCNCKHNTYGVDCEKCLPFFNDRPWRRATAESASECLPCDCNGRSQECYFDPELYRS
TGHGGHCTNCQDNTDGAHCERCRENFFRLGNNEACSSCHCSPVGSLSTQCDSYGRCSCKP
GVMGDKCDRCQPGFHSLTEAGCRPCSCDPSGSIDECNIETGRCVCKDNVEGFNCERCKPG
FFNLESSNPRGCTPCFCFGHSSVCTNAVGYSVYSISSTFQIDEDGWRAEQRDGSEASLEW
SSERQDIAVISDSYFPRYFIAPAKFLGKQVLSYGQNLSFSFRVDRRDTRLSAEDLVLEGA
GLRVSVPLIAQGNSYPSETTVKYVFRLHEATDYPWRPALTPFEFQKLLNNLTSIKIRGTY
SERSAGYLDDVTLASARPGPGVPATWVESCTCPVGYGGQFCEMCLSGYRRETPNLGPYSP
CVLCACNGHSETCDPETGVCNCRDNTAGPHCEKCSDGYYGDSTAGTSSDCQPCPCPGGSS
CAVVPKTKEVVCTNCPTGTTGKRCELCDDGYFGDPLGRNGPVRLCRLCQCSDNIDPNAVG
NCNRLTGECLKCIYNTAGFYCDRCKDGFFGNPLAPNPADKCKACNCNLYGTMKQQSSCNP
VTGQCECLPHVTGQDCGACDPGFYNLQSGQGCERCDCHALGSTNGQCDIRTGQCECQPGI
TGQHCERCEVNHFGFGPEGCKPCDCHPEGSLSLQCKDDGRCECREGFVGNRCDQCEENYF
YNRSWPGCQECPACYRLVKDKVADHRVKLQELESLIANLGTGDEMVTDQAFEDRLKEAER
EVMDLLREAQDVKDVDQNLMDRLQRVNNTLSSQISRLQNIRNTIEETGNLAEQARAHVEN
TERLIEIASRELEKAKVAAANVSVTQPESTGDPNNMTLLAEEARKLAERHKQEADDIVRV
AKTANDTSTEAYNLLLRTLAGENQTAFEIEELNRKYEQAKNISQDLEKQAARVHEEAKRA
GDKAVEIYASVAQLSPLDSETLENEANNIKMEAENLEQLIDQKLKDYEDLREDMRGKELE
VKNLLEKGKTEQQTADQLLARADAAKALAEEAAKKGRDTLQEANDILNNLKDFDRRVNDN
KTAAEEALRKIPAINQTITEANEKTREAQQALGSAAADATEAKNKAHEAERIASAVQKNA
TSTKAEAERTFAEVTDLDNEVNNMLKQLQEAEKELKRKQDDADQDMMMAGMASQAAQEAE
INARKAKNSVTSLLSIINDLLEQLGQLDTVDLNKLNEIEGTLNKAKDEMKVSDLDRKVSD

LENEAKKQEAAIMDYNRDIEEIMKDIRNLEDIRKTLPSGCFNTPSIEKP
>sp|P11142|HSP7C_HUMAN Heat shock cognate 71 kDa protein OS=Homo sapiens OX=9606 GN=HSPA8 PE=1 SV=1
MSKGPAVGIDLGTTYSCVGVFQHGKVEIIANDQGNRTTPSYVAFTDTERLIGDAAKNQVA
MNPTNTVFDAKRLIGRRFDDAVVQSDMKHWPFMVVNDAGRPKVQVEYKGETKSFYPEEVS
SMVLTKMKEIAEAYLGKTVTNAVVTVPAYFNDSQRQATKDAGTIAGLNVLRIINEPTAAA
IAYGLDKKVGAERNVLIFDLGGGTFDVSILTIEDGIFEVKSTAGDTHLGGEDFDNRMVNH
FIAEFKRKHKKDISENKRAVRRLRTACERAKRTLSSSTQASIEIDSLYEGIDFYTSITRA
RFEELNADLFRGTLDPVEKALRDAKLDKSQIHDIVLVGGSTRIPKIQKLLQDFFNGKELN
KSINPDEAVAYGAAVQAAILSGDKSENVQDLLLLDVTPLSLGIETAGGVMTVLIKRNTTI
PTKQTQTFTTYSDNQPGVLIQVYEGERAMTKDNNLLGKFELTGIPPAPRGVPQIEVTFDI
DANGILNVSAVDKSTGKENKITITNDKGRLSKEDIERMVQEAEKYKAEDEKQRDKVSSKN
SLESYAFNMKATVEDEKLQGKINDEDKQKILDKCNEIINWLDKNQTAEKEEFEHQQKELE
KVCNPIITKLYQSAGGMPGGMPGGFPGGGAPPSGGASSGPTIEEVD
>sp|P11166|GTR1_HUMAN Solute carrier family 2, facilitated glucose transporter member 1 OS=Homo sapiens OX=9606 GN=SLC2A1 PE=1 SV=2
MEPSSKKLTGRLMLAVGGAVLGSLQFGYNTGVINAPQKVIEEFYNQTWVHRYGESILPTT
LTTLWSLSVAIFSVGGMIGSFSVGLFVNRFGRRNSMLMMNLLAFVSAVLMGFSKLGKSFE
MLILGRFIIGVYCGLTTGFVPMYVGEVSPTALRGALGTLHQLGIVVGILIAQVFGLDSIM
GNKDLWPLLLSIIFIPALLQCIVLPFCPESPRFLLINRNEENRAKSVLKKLRGTADVTHD
LQEMKEESRQMMREKKVTILELFRSPAYRQPILIAVVLQLSQQLSGINAVFYYSTSIFEK
AGVQQPVYATIGSGIVNTAFTVVSLFVVERAGRRTLHLIGLAGMAGCAILMTIALALLEQ
LPWMSYLSIVAIFGFVAFFEVGPGPIPWFIVAELFSQGPRPAAIAVAGFSNWTSNFIVGM
CFQYVEQLCGPYVFIIFTVLLVLFFIFTYFKVPETKGRTFDEIASGFRQGGASQSDKTPE
ELFHPLGADSQV
>sp|P11171|EPB41_HUMAN Protein 4.1 OS=Homo sapiens OX=9606 GN=EPB41 PE=1 SV=4
MTTEKSLVTEAENSQHQQKEEGEEAINSGQQEPQQEESCQTAAEGDNWCEQKLKASNGDT
PTHEDLTKNKERTSESRGLSRLFSSFLKRPKSQVSEEEGKEVESDKEKGEGGQKEIEFGT
SLDEEIILKAPIAAPEPELKTDPSLDLHSLSSAETQPAQEELREDPDFEIKEGEGLEECS
KIEVKEESPQSKAETELKASQKPIRKHRNMHCKVSLLDDTVYECVVEKHAKGQDLLKRVC
EHLNLLEEDYFGLAIWDNATSKTWLDSAKEIKKQVRGVPWNFTFNVKFYPPDPAQLTEDI
TRYYLCLQLRQDIVAGRLPCSFATLALLGSYTIQSELGDYDPELHGVDYVSDFKLAPNQT
KELEEKVMELHKSYRSMTPAQADLEFLENAKKLSMYGVDLHKAKDLEGVDIILGVCSSGL
LVYKDKLRINRFPWPKVLKISYKRSSFFIKIRPGEQEQYESTIGFKLPSYRAAKKLWKVC
VEHHTFFRLTSTDTIPKSKFLALGSKFRYSGRTQAQTRQASALIDRPAPHFERTASKRAS
RSLDGAAAVDSADRSPRPTSAPAITQGQVAEGGVLDASAKKTVVPKAQKETVKAEVKKED
EPPEQAEPEPTEAWKVEKTHIEVTVPTSNGDQTQKLAEKTEDLIRMRKKKRERLDGENIY
IRHSNLMLEDLDKSQEEIKKHHASISELKKNFMESVPEPRPSEWDKRLSTHSPFRTLNIN
GQIPTGEGPPLVKTQTVTISDNANAVKSEIPTKDVPIVHTETKTITYEAAQTDDNSGDLD
PGVLLTAQTITSETPSSTTTTQITKTVKGGISETRIEKRIVITGDADIDHDQVLVQAIKE
AKEQHPDMSVTKVVVHQETEIADE
>sp|P11172|UMPS_HUMAN Uridine 5'-monophosphate synthase OS=Homo sapiens OX=9606 GN=UMPS PE=1 SV=1
MAVARAALGPLVTGLYDVQAFKFGDFVLKSGLSSPIYIDLRGIVSRPRLLSQVADILFQT

AQNAGISFDTVCGVPYTALPLATVICSTNQIPMLIRRKETKDYGTKRLVEGTINPGETCL
IIEDVVTSGSSVLETVEVLQKEGLKVTDAIVLLDREQGGKDKLQAHGIRLHSVCTLSKML
EILEQQKKVDAETVGRVKRFIQENVFVAANHNGSPLSIKEAPKELSFGARAELPRIHPVA
SKLLRLMQKKETNLCLSADVSLARELLQLADALGPSICMLKTHVDILNDFTLDVMKELIT
LAKCHEFLIFEDRKFADIGNTVKKQYEGGIFKIASWADLVNAHVVPGSGVVKGLQEVGLP
LHRGCLLIAEMSSTGSLATGDYTRAAVRMAEEHSEFVVGFISGSRVSMKPEFLHLTPGVQ
LEAGGDNLGQQYNSPQEVIGKRGSDIIIVGRGIISAADRLEAAEMYRKAAWEAYLSRLGV
>sp|P11216|PYGB_HUMAN Glycogen phosphorylase, brain form OS=Homo sapiens OX=9606 GN=PYGB PE=1 SV=5
MAKPLTDSEKRKQISVRGLAGLGDVAEVRKSFNRHLHFTLVKDRNVATPRDYFFALAHTV
RDHLVGRWIRTQQHYYERDPKRIYYLSLEFYMGRTLQNTMVNLGLQNACDEAIYQLGLDL
EELEEIEEDAGLGNGGLGRLAACFLDSMATLGLAAYGYGIRYEFGIFNQKIVNGWQVEEA
DDWLRYGNPWEKARPEYMLPVHFYGRVEHTPDGVKWLDTQVVLAMPYDTPVPGYKNNTVN
TMRLWSAKAPNDFKLQDFNVGDYIEAVLDRNLAENISRVLYPNDNFFEGKELRLKQEYFV
VAATLQDIIRRFKSSKFGCRDPVRTCFETFPDKVAIQLNDTHPALSIPELMRILVDVEKV
DWDKAWEITKKTCAYTNHTVLPEALERWPVSMFEKLLPRHLEIIYAINQRHLDHVAALFP
GDVDRLRRMSVIEEGDCKRINMAHLCVIGSHAVNGVARIHSEIVKQSVFKDFYELEPEKF
QNKTNGITPRRWLLLCNPGLADTIVEKIGEEFLTDLSQLKKLLPLVSDEVFIRDVAKVKQ
ENKLKFSAFLEKEYKVKINPSSMFDVHVKRIHEYKRQLLNCLHVVTLYNRIKRDPAKAFV
PRTVMIGGKAAPGYHMAKLIIKLVTSIGDVVNHDPVVGDRLKVIFLENYRVSLAEKVIPA
ADLSQQISTAGTEASGTGNMKFMLNGALTIGTMDGANVEMAEEAGAENLFIFGLRVEDVE
ALDRKGYNAREYYDHLPELKQAVDQISSGFFSPKEPDCFKDIVNMLMHHDRFKVFADYEA
YMQCQAQVDQLYRNPKEWTKKVIRNIACSGKFSSDRTITEYAREIWGVEPSDLQIPPPNI
PRD
>sp|P11217|PYGM_HUMAN Glycogen phosphorylase, muscle form OS=Homo sapiens OX=9606 GN=PYGM PE=1 SV=6
MSRPLSDQEKRKQISVRGLAGVENVTELKKNFNRHLHFTLVKDRNVATPRDYYFALAHTV
RDHLVGRWIRTQQHYYEKDPKRIYYLSLEFYMGRTLQNTMVNLALENACDEATYQLGLDM
EELEEIEEDAGLGNGGLGRLAACFLDSMATLGLAAYGYGIRYEFGIFNQKISGGWQMEEA
DDWLRYGNPWEKARPEFTLPVHFYGHVEHTSQGAKWVDTQVVLAMPYDTPVPGYRNNVVN
TMRLWSAKAPNDFNLKDFNVGGYIQAVLDRNLAENISRVLYPNDNFFEGKELRLKQEYFV
VAATLQDIIRRFKSSKFGCRDPVRTNFDAFPDKVAIQLNDTHPSLAIPELMRILVDLERM
DWDKAWDVTVRTCAYTNHTVLPEALERWPVHLLETLLPRHLQIIYEINQRFLNRVAAAFP
GDVDRLRRMSLVEEGAVKRINMAHLCIAGSHAVNGVARIHSEILKKTIFKDFYELEPHKF
QNKTNGITPRRWLVLCNPGLAEVIAERIGEDFISDLDQLRKLLSFVDDEAFIRDVAKVKQ
ENKLKFAAYLEREYKVHINPNSLFDIQVKRIHEYKRQLLNCLHVITLYNRIKREPNKFFV
PRTVMIGGKAAPGYHMAKMIIRLVTAIGDVVNHDPAVGDRLRVIFLENYRVSLAEKVIPA
ADLSEQISTAGTEASGTGNMKFMLNGALTIGTMDGANVEMAEEAGEENFFIFGMRVEDVD
KLDQRGYNAQEYYDRIPELRQVIEQLSSGFFSPKQPDLFKDIVNMLMHHDRFKVFADYED
YIKCQEKVSALYKNPREWTRMVIRNIATSGKFSSDRTIAQYAREIWGVEPSRQRLPAPDE
AI
>sp|P11233|RALA_HUMAN Ras-related protein Ral-A OS=Homo sapiens OX=9606 GN=RALA PE=1 SV=1
MAANKPKGQNSLALHKVIMVGSGGVGKSALTLQFMYDEFVEDYEPTKADSYRKKVVLDGE

EVQIDILDTAGQEDYAAIRDNYFRSGEGFLCVFSITEMESFAATADFREQILRVKEDENV
PFLLVGNKSDLEDKRQVSVEEAKNRAEQWNVNYVETSAKTRANVDKVFFDLMREIRARKM
EDSKEKNGKKKRKSLAKRIRERCCIL
>sp|P11234|RALB_HUMAN Ras-related protein Ral-B OS=Homo sapiens OX=9606 GN=RALB PE=1 SV=1
MAANKSKGQSSLALHKVIMVGSGGVGKSALTLQFMYDEFVEDYEPTKADSYRKKVVLDGE
EVQIDILDTAGQEDYAAIRDNYFRSGEGFLLVFSITEHESFTATAEFREQILRVKAEEDK
IPLLVVGNKSDLEERRQVPVEEARSKAEEWGVQYVETSAKTRANVDKVFFDLMREIRTKK
MSENKDKNGKKSSKNKKSFKERCCLL
>sp|P11277|SPTB1_HUMAN Spectrin beta chain, erythrocytic OS=Homo sapiens OX=9606 GN=SPTB PE=1 SV=5
MTSATEFENVGNQPPYSRINARWDAPDDELDNDNSSARLFERSRIKALADEREVVQKKTF
TKWVNSHLARVSCRITDLYKDLRDGRMLIKLLEVLSGEMLPKPTKGKMRIHCLENVDKAL
QFLKEQRVHLENMGSHDIVDGNHRLVLGLIWTIILRFQIQDIVVQTQEGRETRSAKDALL
LWCQMKTAGYPHVNVTNFTSSWKDGLAFNALIHKHRPDLIDFDKLKDSNARHNLEHAFNV
AERQLGIIPLLDPEDVFTENPDEKSIITYVVAFYHYFSKMKVLAVEGKRVGKVIDHAIET
EKMIEKYSGLASDLLTWIEQTITVLNSRKFANSLTGVQQQLQAFSTYRTVEKPPKFQEKG
NLEVLLFTIQSRMRANNQKVYTPHDGKLVSDINRAWESLEEAEYRRELALRNELIRQEKL
EQLARRFDRKAAMRETWLSENQRLVAQDNFGYDLAAVEAAKKKHEAIETDTAAYEERVRA
LEDLAQELEKENYHDQKRITARKDNILRLWSYLQELLQSRRQRLETTLALQKLFQDMLHS
IDWMDEIKAHLLSAEFGKHLLEVEDLLQKHKLMEADIAIQGDKVKAITAATLKFTEGKGY
QPCDPQVIQDRISHLEQCFEELSNMAAGRKAQLEQSKRLWKFFWEMDEAESWIKEKEQIY
SSLDYGKDLTSVLILQRKHKAFEDELRGLDAHLEQIFQEAHGMVARKQFGHPQIEARIKE
VSAQWDQLKDLAAFCKKNLQDAENFFQFQGDADDLKAWLQDAHRLLSGEDVGQDEGATRA
LGKKHKDFLEELEESRGVMEHLEQQAQGFPEEFRDSPDVTHRLQALRELYQQVVAQADLR
QQRLQEALDLYTVFGETDACELWMGEKEKWLAEMEMPDTLEDLEVVQHRFDILDQEMKTL
MTQIDGVNLAANSLVESGHPRSREVKQYQDHLNTRWQAFQTLVSERREAVDSALRVHNYC
VDCEETSKWITDKTKVVESTKDLGRDLAGIIAIQRKLSGLERDVAAIQARVDALERESQQ
LMDSHPEQKEDIGQRQKHLEELWQGLQQSLQGQEDLLGEVSQLQAFLQDLDDFQAWLSIT
QKAVASEDMPESLPEAEQLLQQHAGIKDEIDGHQDSYQRVKESGEKVIQGQTDPEYLLLG
QRLEGLDTGWNALGRMWESRSHTLAQCLGFQEFQKDAKQAEAILSNQEYTLAHLEPPDSL
EAAEAGIRKFEDFLGSMENNRDKVLSPVDSGNKLVAEGNLYSDKIKEKVQLIEDRHRKNN
EKAQEASVLLRDNLELQNFLQNCQELTLWINDKLLTSQDVSYDEARNLHNKWLKHQAFVA
ELASHEGWLENIDAEGKQLMDEKPQFTALVSQKLEALHRLWDELQATTKEKTQHLSAARS
SDLRLQTHADLNKWISAMEDQLRSDDPGKDLTSVNRMLAKLKRVEDQVNVRKEELGELFA
QVPSMGEEGGDADLSIEKRFLDLLEPLGRRKKQLESSRAKLQISRDLEDETLWVEERLPL
AQSADYGTNLQTVQLFMKKNQTLQNEILGHTPRVEDVLQRGQQLVEAAEIDCQDLEERLG
HLQSSWDRLREAAAGRLQRLRDANEAQQYYLDADEAEAWIGEQELYVISDEIPKDEEGAI
VMLKRHLRQQRAVEDYGRNIKQLASRAQGLLSAGHPEGEQIIRLQGQVDKHYAGLKDVAE
ERKRKLENMYHLFQLKRETDDLEQWISEKELVASSPEMGQDFDHVTLLRDKFRDFARETG
AIGQERVDNVNAFIERLIDAGHSEAATIAEWKDGLNEMWADLLELIDTRMQLLAASYDLH
RYFYTGAEILGLIDEKHRELPEDVGLDASTAESFHRVHTAFERELHLLGVQVQQFQDVAT
RLQTAYAGEKAEAIQNKEQEVSAAWQALLDACAGRRTQLVDTADKFRFFSMARDLLSWME
SIIRQIETQERPRDVSSVELLMKYHQGINAEIETRSKNFSACLELGESLLQRQHQASEEI

REKLQQVMSRRKEMNEKWEARWERLRMLLEVCQFSRDASVAEAWLIAQEPYLASGDFGHT
VDSVEKLIKRHEAFEKSTASWAERFAALEKPTTLELKERQIAERPAEETGPQEEEGETAG
EAPVSHHAATERTSPVSLWSRLSSSWESLQPEPSHPY
>sp|P11413|G6PD_HUMAN Glucose-6-phosphate 1-dehydrogenase OS=Homo sapiens OX=9606 GN=G6PD PE=1 SV=4
MAEQVALSRTQVCGILREELFQGDAFHQSDTHIFIIMGASGDLAKKKIYPTIWWLFRDGL
LPENTFIVGYARSRLTVADIRKQSEPFFKATPEEKLKLEDFFARNSYVAGQYDDAASYQR
LNSHMNALHLGSQANRLFYLALPPTVYEAVTKNIHESCMSQIGWNRIIVEKPFGRDLQSS
DRLSNHISSLFREDQIYRIDHYLGKEMVQNLMVLRFANRIFGPIWNRDNIACVILTFKEP
FGTEGRGGYFDEFGIIRDVMQNHLLQMLCLVAMEKPASTNSDDVRDEKVKVLKCISEVQA
NNVVLGQYVGNPDGEGEATKGYLDDPTVPRGSTTATFAAVVLYVENERWDGVPFILRCGK
ALNERKAEVRLQFHDVAGDIFHQQCKRNELVIRVQPNEAVYTKMMTKKPGMFFNPEESEL
DLTYGNRYKNVKLPDAYERLILDVFCGSQMHFVRSDELREAWRIFTPLLHQIELEKPKPI
PYIYGSRGPTEADELMKRVGFQYEGTYKWVNPHKL
>sp|P11766|ADHX_HUMAN Alcohol dehydrogenase class-3 OS=Homo sapiens OX=9606 GN=ADH5 PE=1 SV=4
MANEVIKCKAAVAWEAGKPLSIEEIEVAPPKAHEVRIKIIATAVCHTDAYTLSGADPEGC
FPVILGHEGAGIVESVGEGVTKLKAGDTVIPLYIPQCGECKFCLNPKTNLCQKIRVTQGK
GLMPDGTSRFTCKGKTILHYMGTSTFSEYTVVADISVAKIDPLAPLDKVCLLGCGISTGY
GAAVNTAKLEPGSVCAVFGLGGVGLAVIMGCKVAGASRIIGVDINKDKFARAKEFGATEC
INPQDFSKPIQEVLIEMTDGGVDYSFECIGNVKVMRAALEACHKGWGVSVVVGVAASGEE
IATRPFQLVTGRTWKGTAFGGWKSVESVPKLVSEYMSKKIKVDEFVTHNLSFDEINKAFE
LMHSGKSIRTVVKI
>sp|P11844|CRGA_HUMAN Gamma-crystallin A OS=Homo sapiens OX=9606 GN=CRYGA PE=2 SV=3
MGKITFYEDRDFQGRCYNCISDCPNLRVYFSRCNSIRVDSGCWMLYERPNYQGHQYFLRR
GKYPDYQHWMGLSDSVQSCRIIPHTSSHKLRLYERDDYRGLMSELTDDCACVPELFRLPE
IYSLHVLEGCWVLYEMPNYRGRQYLLRPGDYRRYHDWGGADAKVGSLRRVTDLY
>sp|P12235|ADT1_HUMAN ADP/ATP translocase 1 OS=Homo sapiens OX=9606 GN=SLC25A4 PE=1 SV=4
MGDHAWSFLKDFLAGGVAAAVSKTAVAPIERVKLLLQVQHASKQISAEKQYKGIIDCVVR
IPKEQGFLSFWRGNLANVIRYFPTQALNFAFKDKYKQLFLGGVDRHKQFWRYFAGNLASG
GAAGATSLCFVYPLDFARTRLAADVGKGAAQREFHGLGDCIIKIFKSDGLRGLYQGFNVS
VQGIIIYRAAYFGVYDTAKGMLPDPKNVHIFVSWMIAQSVTAVAGLVSYPFDTVRRRMMM
QSGRKGADIMYTGTVDCWRKIAKDEGAKAFFKGAWSNVLRGMGGAFVLVLYDEIKKYV
>sp|P12268|IMDH2_HUMAN Inosine-5'-monophosphate dehydrogenase 2 OS=Homo sapiens OX=9606 GN=IMPDH2 PE=1 SV=2
MADYLISGGTSYVPDDGLTAQQLFNCGDGLTYNDFLILPGYIDFTADQVDLTSALTKKIT
LKTPLVSSPMDTVTEAGMAIAMALTGGIGFIHHNCTPEFQANEVRKVKKYEQGFITDPVV
LSPKDRVRDVFEAKARHGFCGIPITDTGRMGSRLVGIISSRDIDFLKEEEHDCFLEEIMT
KREDLVVAPAGITLKEANEILQRSKKGKLPIVNEDDELVAIIARTDLKKNRDYPLASKDA
KKQLLCGAAIGTHEDDKYRLDLLAQAGVDVVVLDSSQGNSIFQINMIKYIKDKYPNLQVI
GGNVVTAAQAKNLIDAGVDALRVGMGSGSICITQEVLACGRPQATAVYKVSEYARRFGVP
VIADGGIQNVGHIAKALALGASTVMMGSLLAATTEAPGEYFFSDGIRLKKYRGMGSLDAM

DKHLSSQNRYFSEADKIKVAQGVSGAVQDKGSIHKFVPYLIAGIQHSCQDIGAKSLTQVR
AMMYSGELKFEKRTSSAQVEGGVHSLHSYEKRLF
>sp|P12814|ACTN1_HUMAN Alpha-actinin-1 OS=Homo sapiens OX=9606 GN=ACTN1 PE=1 SV=2
MDHYDSQQTNDYMQPEEDWDRDLLLDPAWEKQQRKTFTAWCNSHLRKAGTQIENIEEDFR
DGLKLMLLLEVISGERLAKPERGKMRVHKISNVNKALDFIASKGVKLVSIGAEEIVDGNV
KMTLGMIWTIILRFAIQDISVEETSAKEGLLLWCQRKTAPYKNVNIQNFHISWKDGLGFC
ALIHRHRPELIDYGKLRKDDPLTNLNTAFDVAEKYLDIPKMLDAEDIVGTARPDEKAIMT
YVSSFYHAFSGAQKAETAANRICKVLAVNQENEQLMEDYEKLASDLLEWIRRTIPWLENR
VPENTMHAMQQKLEDFRDYRRLHKPPKVQEKCQLEINFNTLQTKLRLSNRPAFMPSEGRM
VSDINNAWGCLEQVEKGYEEWLLNEIRRLERLDHLAEKFRQKASIHEAWTDGKEAMLRQK
DYETATLSEIKALLKKHEAFESDLAAHQDRVEQIAAIAQELNELDYYDSPSVNARCQKIC
DQWDNLGALTQKRREALERTEKLLETIDQLYLEYAKRAAPFNNWMEGAMEDLQDTFIVHT
IEEIQGLTTAHEQFKATLPDADKERLAILGIHNEVSKIVQTYHVNMAGTNPYTTITPQEI
NGKWDHVRQLVPRRDQALTEEHARQQHNERLRKQFGAQANVIGPWIQTKMEEIGRISIEM
HGTLEDQLSHLRQYEKSIVNYKPKIDQLEGDHQLIQEALIFDNKHTNYTMEHIRVGWEQL
LTTIARTINEVENQILTRDAKGISQEQMNEFRASFNHFDRDHSGTLGPEEFKACLISLGY
DIGNDPQGEAEFARIMSIVDPNRLGVVTFQAFIDFMSRETADTDTADQVMASFKILAGDK
NYITMDELRRELPPDQAEYCIARMAPYTGPDSVPGALDYMSFSTALYGESDL
>sp|P12955|PEPD_HUMAN Xaa-Pro dipeptidase OS=Homo sapiens OX=9606 GN=PEPD PE=1 SV=3
MAAATGPSFWLGNETLKVPLALFALNRQRLCERLRKNPAVQAGSIVVLQGGEETQRYCTD
TGVLFRQESFFHWAFGVTEPGCYGVIDVDTGKSTLFVPRLPASHATWMGKIHSKEHFKEK
YAVDDVQYVDEIASVLTSQKPSVLLTLRGVNTDSGSVCREASFDGISKFEVNNTILHPEI
VECRVFKTDMELEVLRYTNKISSEAHREVMKAVKVGMKEYELESLFEHYCYSRGGMRHSS
YTCICGSGENSAVLHYGHAGAPNDRTIQNGDMCLFDMGGEYYCFASDITCSFPANGKFTA
DQKAVYEAVLRSSRAVMGAMKPGVWWPDMHRLADRIHLEELAHMGILSGSVDAMVQAHLG
AVFMPHGLGHFLGIDVHDVGGYPEGVERIDEPGLRSLRTARHLQPGMVLTVEPGIYFIDH
LLDEALADPARASFLNREVLQRFRGFGGVRIEEDVVVTDSGIELLTCVPRTVEEIEACMA
GCDKAFTPFSGPK
>sp|P13073|COX41_HUMAN Cytochrome c oxidase subunit 4 isoform 1, mitochondrial OS=Homo sapiens OX=9606 GN=COX4I1 PE=1 SV=1
MLATRVFSLVGKRAISTSVCVRAHESVVKSEDFSLPAYMDRRDHPLPEVAHVKHLSASQK
ALKEKEKASWSSLSMDEKVELYRIKFKESFAEMNRGSNEWKTVVGGAMFFIGFTALVIMW
QKHYVYGPLPQSFDKEWVAKQTKRMLDMKVNPIQGLASKWDYEKNEWKK
>sp|P13489|RINI_HUMAN Ribonuclease inhibitor OS=Homo sapiens OX=9606 GN=RNH1 PE=1 SV=2
MSLDIQSLDIQCEELSDARWAELLPLLQQCQVVRLDDCGLTEARCKDISSALRVNPALAE
LNLRSNELGDVGVHCVLQGLQTPSCKIQKLSLQNCCLTGAGCGVLSSTLRTLPTLQELHL
SDNLLGDAGLQLLCEGLLDPQCRLEKLQLEYCSLSAASCEPLASVLRAKPDFKELTVSNN
DINEAGVRVLCQGLKDSPCQLEALKLESCGVTSDNCRDLCGIVASKASLRELALGSNKLG
DVGMAELCPGLLHPSSRLRTLWIWECGITAKGCGDLCRVLRAKESLKELSLAGNELGDEG
ARLLCETLLEPGCQLESLWVKSCSFTAACCSHFSSVLAQNRFLLELQISNNRLEDAGVRE
LCQGLGQPGSVLRVLWLADCDVSDSSCSSLAATLLANHSLRELDLSNNCLGDAGILQLVE

SVRQPGCLLEQLVLYDIYWSEEMEDRLQALEKDKPSLRVIS
>sp|P13591|NCAM1_HUMAN Neural cell adhesion molecule 1 OS=Homo sapiens OX=9606 GN=NCAM1 PE=1 SV=3
MLQTKDLIWTLFFLGTAVSLQVDIVPSQGEISVGESKFFLCQVAGDAKDKDISWFSPNGE
KLTPNQQRISVVWNDDSSSTLTIYNANIDDAGIYKCVVTGEDGSESEATVNVKIFQKLMF
KNAPTPQEFREGEDAVIVCDVVSSLPPTIIWKHKGRDVILKKDVRFIVLSNNYLQIRGIK
KTDEGTYRCEGRILARGEINFKDIQVIVNVPPTIQARQNIVNATANLGQSVTLVCDAEGF
PEPTMSWTKDGEQIEQEEDDEKYIFSDDSSQLTIKKVDKNDEAEYICIAENKAGEQDATI
HLKVFAKPKITYVENQTAMELEEQVTLTCEASGDPIPSITWRTSTRNISSEEKASWTRPE
KQETLDGHMVVRSHARVSSLTLKSIQYTDAGEYICTASNTIGQDSQSMYLEVQYAPKLQG
PVAVYTWEGNQVNITCEVFAYPSATISWFRDGQLLPSSNYSNIKIYNTPSASYLEVTPDS
ENDFGNYNCTAVNRIGQESLEFILVQADTPSSPSIDQVEPYSSTAQVQFDEPEATGGVPI
LKYKAEWRAVGEEVWHSKWYDAKEASMEGIVTIVGLKPETTYAVRLAALNGKGLGEISAA
SEFKTQPVQGEPSAPKLEGQMGEDGNSIKVNLIKQDDGGSPIRHYLVRYRALSSEWKPEI
RLPSGSDHVMLKSLDWNAEYEVYVVAENQQGKSKAAHFVFRTSAQPTAIPANGSPTSGLS
TGAIVGILIVIFVLLLVVVDITCYFLNKCGLFMCIAVNLCGKAGPGAKGKDMEEGKAAFS
KDESKEPIVEVRTEEERTPNHDGGKHTEPNETTPLTEPEKGPVEAKPECQETETKPAPAE
VKTVPNDATQTKENESKA
>sp|P13637|AT1A3_HUMAN Sodium/potassium-transporting ATPase subunit alpha-3 OS=Homo sapiens OX=9606 GN=ATP1A3 PE=1 SV=3
MGDKKDDKDSPKKNKGKERRDLDDLKKEVAMTEHKMSVEEVCRKYNTDCVQGLTHSKAQE
ILARDGPNALTPPPTTPEWVKFCRQLFGGFSILLWIGAILCFLAYGIQAGTEDDPSGDNL
YLGIVLAAVVIITGCFSYYQEAKSSKIMESFKNMVPQQALVIREGEKMQVNAEEVVVGDL
VEIKGGDRVPADLRIISAHGCKVDNSSLTGESEPQTRSPDCTHDNPLETRNITFFSTNCV
EGTARGVVVATGDRTVMGRIATLASGLEVGKTPIAIEIEHFIQLITGVAVFLGVSFFILS
LILGYTWLEAVIFLIGIIVANVPEGLLATVTVCLTLTAKRMARKNCLVKNLEAVETLGST
STICSDKTGTLTQNRMTVAHMWFDNQIHEADTTEDQSGTSFDKSSHTWVALSHIAGLCNR
AVFKGGQDNIPVLKRDVAGDASESALLKCIELSSGSVKLMRERNKKVAEIPFNSTNKYQL
SIHETEDPNDNRYLLVMKGAPERILDRCSTILLQGKEQPLDEEMKEAFQNAYLELGGLGE
RVLGFCHYYLPEEQFPKGFAFDCDDVNFTTDNLCFVGLMSMIDPPRAAVPDAVGKCRSAG
IKVIMVTGDHPITAKAIAKGVGIISEGNETVEDIAARLNIPVSQVNPRDAKACVIHGTDL
KDFTSEQIDEILQNHTEIVFARTSPQQKLIIVEGCQRQGAIVAVTGDGVNDSPALKKADI
GVAMGIAGSDVSKQAADMILLDDNFASIVTGVEEGRLIFDNLKKSIAYTLTSNIPEITPF
LLFIMANIPLPLGTITILCIDLGTDMVPAISLAYEAAESDIMKRQPRNPRTDKLVNERLI
SMAYGQIGMIQALGGFFSYFVILAENGFLPGNLVGIRLNWDDRTVNDLEDSYGQQWTYEQ
RKVVEFTCHTAFFVSIVVVQWADLIICKTRRNSVFQQGMKNKILIFGLFEETALAAFLSY
CPGMDVALRMYPLKPSWWFCAFPYSFLIFVYDEIRKLILRRNPGGWVEKETYY
>sp|P13639|EF2_HUMAN Elongation factor 2 OS=Homo sapiens OX=9606 GN=EEF2 PE=1 SV=4
MVNFTVDQIRAIMDKKANIRNMSVIAHVDHGKSTLTDSLVCKAGIIASARAGETRFTDTR
KDEQERCITIKSTAISLFYELSENDLNFIKQSKDGAGFLINLIDSPGHVDFSSEVTAALR
VTDGALVVVDCVSGVCVQTETVLRQAIAERIKPVLMMNKMDRALLELQLEPEELYQTFQR
IVENVNVIISTYGEGESGPMGNIMIDPVLGTVGFGSGLHGWAFTLKQFAEMYVAKFAAKG
EGQLGPAERAKKVEDMMKKLWGDRYFDPANGKFSKSATSPEGKKLPRTFCQLILDPIFKV
FDAIMNFKKEETAKLIEKLDIKLDSEDKDKEGKPLLKAVMRRWLPAGDALLQMITIHLPS

PVTAQKYRCELLYEGPPDDEAAMGIKSCDPKGPLMMYISKMVPTSDKGRFYAFGRVFSGL
VSTGLKVRIMGPNYTPGKKEDLYLKPIQRTILMMGRYVEPIEDVPCGNIVGLVGVDQFLV
KTGTITTFEHAHNMRVMKFSVSPVVRVAVEAKNPADLPKLVEGLKRLAKSDPMVQCIIEE
SGEHIIAGAGELHLEICLKDLEEDHACIPIKKSDPVVSYRETVSEESNVLCLSKSPNKHN
RLYMKARPFPDGLAEDIDKGEVSARQELKQRARYLAEKYEWDVAEARKIWCFGPDGTGPN
ILTDITKGVQYLNEIKDSVVAGFQWATKEGALCEENMRGVRFDVHDVTLHADAIHRGGGQ
IIPTARRCLYASVLTAQPRLMEPIYLVEIQCPEQVVGGIYGVLNRKRGHVFEESQVAGTP
MFVVKAYLPVNESFGFTADLRSNTGGQAFPQCVFDHWQILPGDPFDNSSRPSQVVAETRK
RKGLKEGIPALDNFLDKL
>sp|P13645|K1C10_HUMAN Keratin, type I cytoskeletal 10 OS=Homo sapiens OX=9606
GN=KRT10 PE=1 SV=6
MSVRYSSSKHYSSSRSGGGGGGGGCGGGGGVSSLRISSSKGSLGGGFSSGGFSGGSFSRG
SSGGGCFGGSSGGYGGLGGFGGGSFRGSYGSSSFGGSYGGIFGGGSFGGGSFGGGSFGGG
GFGGGGFGGGFGGGFGGDGGLLSGNEKVTMQNLNDRLASYLDKVRALEESNYELEGKIKE
WYEKHGNSHQGEPRDYSKYYKTIDDLKNQILNLTTDNANILLQIDNARLAADDFRLKYEN
EVALRQSVEADINGLRRVLDELTLTKADLEMQIESLTEELAYLKKNHEEEMKDLRNVSTG
DVNVEMNAAPGVDLTQLLNNMRSQYEQLAEQNRKDAEAWFNEKSKELTTEIDNNIEQISS
YKSEITELRRNVQALEIELQSQLALKQSLEASLAETEGRYCVQLSQIQAQISALEEQLQQ
IRAETECQNTEYQQLLDIKIRLENEIQTYRSLLEGEGSSGGGGRGGGSFGGGYGGGSSGG
GSSGGGHGGGHGGSSGGGYGGGSSGGGSSGGGYGGGSSSGGHGGSSSGGYGGGSSGGGGG
GYGGGSSGGGSSSGGGYGGGSSSGGHKSSSSGSVGESSSKGPRY
>sp|P13716|HEM2_HUMAN Delta-aminolevulinic acid dehydratase OS=Homo sapiens OX=9606
GN=ALAD PE=1 SV=1
MQPQSVLHSGYFHPLLRAWQTATTTLNASNLIYPIFVTDVPDDIQPITSLPGVARYGVKR
LEEMLRPLVEEGLRCVLIFGVPSRVPKDERGSAADSEESPAIEAIHLLRKTFPNLLVACD
VCLCPYTSHGHCGLLSENGAFRAEESRQRLAEVALAYAKAGCQVVAPSDMMDGRVEAIKE
ALMAHGLGNRVSVMSYSAKFASCFYGPFRDAAKSSPAFGDRRCYQLPPGARGLALRAVDR
DVREGADMLMVKPGMPYLDIVREVKDKHPDLPLAVYHVSGEFAMLWHGAQAGAFDLKAAV
LEAMTAFRRAGADIIITYYTPQLLQWLKEE
>sp|P13797|PLST_HUMAN Plastin-3 OS=Homo sapiens OX=9606 GN=PLS3 PE=1 SV=4
MDEMATTQISKDELDELKEAFAKVDLNSNGFICDYELHELFKEANMPLPGYKVREIIQKL
MLDGDRNKDGKISFDEFVYIFQEVKSSDIAKTFRKAINRKEGICALGGTSELSSEGTQHS
YSEEEKYAFVNWINKALENDPDCRHVIPMNPNTDDLFKAVGDGIVLCKMINLSVPDTIDE
RAINKKKLTPFIIQENLNLALNSASAIGCHVVNIGAEDLRAGKPHLVLGLLWQIIKIGLF
ADIELSRNEALAALLRDGETLEELMKLSPEELLLRWANFHLENSGWQKINNFSADIKDSK
AYFHLLNQIAPKGQKEGEPRIDINMSGFNETDDLKRAESMLQQADKLGCRQFVTPADVVS
GNPKLNLAFVANLFNKYPALTKPENQDIDWTLLEGETREERTFRNWMNSLGVNPHVNHLY
ADLQDALVILQLYERIKVPVDWSKVNKPPYPKLGANMKKLENCNYAVELGKHPAKFSLVG
IGGQDLNDGNQTLTLALVWQLMRRYTLNVLEDLGDGQKANDDIIVNWVNRTLSEAGKSTS
IQSFKDKTISSSLAVVDLIDAIQPGCINYDLVKSGNLTEDDKHNNAKYAVSMARRIGARV
YALPEDLVEVKPKMVMTVFACLMGRGMKRV
>sp|P13798|ACPH_HUMAN Acylamino-acid-releasing enzyme OS=Homo sapiens OX=9606
GN=APEH PE=1 SV=4
MERQVLLSEPEEAAALYRGLSRQPALSAACLGPEVTTQYGGQYRTVHTEWTQRDLERMEN

IRFCRQYLVFHDGDSVVFAGPAGNSVETRGELLSRESPSGTMKAVLRKAGGTGPGEEKQF
LEVWEKNRKLKSFNLSALEKHGPVYEDDCFGCLSWSHSETHLLYVAEKKRPKAESFFQTK
ALDVSASDDEIARLKKPDQAIKGDQFVFYEDWGENMVSKSIPVLCVLDVESGNISVLEGV
PENVSPGQAFWAPGDAGVVFVGWWHEPFRLGIRFCTNRRSALYYVDLIGGKCELLSDDSL
AVSSPRLSPDQCRIVYLQYPSLIPHHQCSQLCLYDWYTKVTSVVVDVVPRQLGENFSGIY
CSLLPLGCWSADSQRVVFDSAQRSRQDLFAVDTQVGTVTSLTAGGSGGSWKLLTIDQDLM
VAQFSTPSLPPTLKVGFLPSAGKEQSVLWVSLEEAEPIPDIHWGIRVLQPPPEQENVQYA
GLDFEAILLQPGSPPDKTQVPMVVMPHGGPHSSFVTAWMLFPAMLCKMGFAVLLVNYRGS
TGFGQDSILSLPGNVGHQDVKDVQFAVEQVLQEEHFDASHVALMGGSHGGFISCHLIGQY
PETYRACVARNPVINIASMLGSTDIPDWCVVEAGFPFSSDCLPDLSVWAEMLDKSPIRYI
PQVKTPLLLMLGQEDRRVPFKQGMEYYRALKTRNVPVRLLLYPKSTHALSEVEVESDSFM
NAVLWLRTHLGS
>sp|P13861|KAP2_HUMAN cAMP-dependent protein kinase type II-alpha regulatory subunit OS=Homo sapiens OX=9606 GN=PRKAR2A PE=1 SV=2
MSHIQIPPGLTELLQGYTVEVLRQQPPDLVEFAVEYFTRLREARAPASVLPAATPRQSLG
HPPPEPGPDRVADAKGDSESEEDEDLEVPVPSRFNRRVSVCAETYNPDEEEEDTDPRVIH
PKTDEQRCRLQEACKDILLFKNLDQEQLSQVLDAMFERIVKADEHVIDQGDDGDNFYVIE
RGTYDILVTKDNQTRSVGQYDNRGSFGELALMYNTPRAATIVATSEGSLWGLDRVTFRRI
IVKNNAKKRKMFESFIESVPLLKSLEVSERMKIVDVIGEKIYKDGERIITQGEKADSFYI
IESGEVSILIRSRTKSNKDGGNQEVEIARCHKGQYFGELALVTNKPRAASAYAVGDVKCL
VMDVQAFERLLGPCMDIMKRNISHYEEQLVKMFGSSVDLGNLGQ
>sp|P13929|ENOB_HUMAN Beta-enolase OS=Homo sapiens OX=9606 GN=ENO3 PE=1 SV=5
MAMQKIFAREILDSRGNPTVEVDLHTAKGRFRAAVPSGASTGIYEALELRDGDKGRYLGK
GVLKAVENINNTLGPALLQKKLSVVDQEKVDKFMIELDGTENKSKFGANAILGVSLAVCK
AGAAEKGVPLYRHIADLAGNPDLILPVPAFNVINGGSHAGNKLAMQEFMILPVGASSFKE
AMRIGAEVYHHLKGVIKAKYGKDATNVGDEGGFAPNILENNEALELLKTAIQAAGYPDKV
VIGMDVAASEFYRNGKYDLDFKSPDDPARHITGEKLGELYKSFIKNYPVVSIEDPFDQDD
WATWTSFLSGVNIQIVGDDLTVTNPKRIAQAVEKKACNCLLLKVNQIGSVTESIQACKLA
QSNGWGVMVSHRSGETEDTFIADLVVGLCTGQIKTGAPCRSERLAKYNQLMRIEEALGDK
AIFAGRKFRNPKAK
>sp|P13987|CD59_HUMAN CD59 glycoprotein OS=Homo sapiens OX=9606 GN=CD59 PE=1 SV=1
MGIQGGSVLFGLLLVLAVFCHSGHSLQCYNCPNPTADCKTAVNCSSDFDACLITKAGLQV
YNKCWKFEHCNFNDVTTRLRENELTYYCCKKDLCNFNEQLENGGTSLSEKTVLLLVTPFL
AAAWSLHP
>sp|P14174|MIF_HUMAN Macrophage migration inhibitory factor OS=Homo sapiens OX=9606 GN=MIF PE=1 SV=4
MPMFIVNTNVPRASVPDGFLSELTQQLAQATGKPPQYIAVHVVPDQLMAFGGSSEPCALC
SLHSIGKIGGAQNRSYSKLLCGLLAERLRISPDRVYINYYDMNAANVGWNNSTFA
>sp|P14543|NID1_HUMAN Nidogen-1 OS=Homo sapiens OX=9606 GN=NID1 PE=1 SV=3
MLASSSRIRAAWTRALLLPLLLAGPVGCLSRQELFPFGPGQGDLELEDGDDFVSPALELS
GALRFYDRSDIDAVYVTTNGIIATSEPPAKESHPGLFPPTFGAVAPFLADLDTTDGLGKV
YYREDLSPSITQRAAECVHRGFPEISFQPSSAVVVTWESVAPYQGPSRDPDQKGKRNTFQ
AVLASSDSSSYAIFLYPEDGLQFHTTFSKKENNQVPAVVAFSQGSVGFLWKSNGAYNIFA

NDRESVENLAKSSNSGQQGVWVFEIGSPATTNGVVPADVILGTEDGAEYDDEDEDYDLAT
TRLGLEDVGTTPFSYKALRRGGADTYSVPSVLSPRRAATERPLGPPTERTRSFQLAVETF
HQQHPQVIDVDEVEETGVVFSYNTDSRQTCANNRHQCSVHAECRDYATGFCCSCVAGYTG
NGRQCVAEGSPQRVNGKVKGRIFVGSSQVPIVFENTDLHSYVVMNHGRSYTAISTIPETV
GYSLLPLAPVGGIIGWMFAVEQDGFKNGFSITGGEFTRQAEVTFVGHPGNLVIKQRFSGI
DEHGHLTIDTELEGRVPQIPFGSSVHIEPYTELYHYSTSVITSSSTREYTVTEPERDGAS
PSRIYTYQWRQTITFQECVHDDSRPALPSTQQLSVDSVFVLYNQEEKILRYALSNSIGPV
REGSPDALQNPCYIGTHGCDTNAACRPGPRTQFTCECSIGFRGDGRTCYDIDECSEQPSV
CGSHTICNNHPGTFRCECVEGYQFSDEGTCVAVVDQRPINYCETGLHNCDIPQRAQCIYT
GGSSYTCSCLPGFSGDGQACQDVDECQPSRCHPDAFCYNTPGSFTCQCKPGYQGDGFRCV
PGEVEKTRCQHEREHILGAAGATDPQRPIPPGLFVPECDAHGHYAPTQCHGSTGYCWCVD
RDGREVEGTRTRPGMTPPCLSTVAPPIHQGPAVPTAVIPLPPGTHLLFAQTGKIERLPLE
GNTMRKTEAKAFLHVPAKVIIGLAFDCVDKMVYWTDITEPSIGRASLHGGEPTTIIRQDL
GSPEGIAVDHLGRNIFWTDSNLDRIEVAKLDGTQRRVLFETDLVNPRGIVTDSVRGNLYW
TDWNRDNPKIETSYMDGTNRRILVQDDLGLPNGLTFDAFSSQLCWVDAGTNRAECLNPSQ
PSRRKALEGLQYPFAVTSYGKNLYFTDWKMNSVVALDLAISKETDAFQPHKQTRLYGITT
ALSQCPQGHNYCSVNNGGCTHLCLATPGSRTCRCPDNTLGVDCIEQK
>sp|P14550|AK1A1_HUMAN Aldo-keto reductase family 1 member A1 OS=Homo sapiens OX=9606 GN=AKR1A1 PE=1 SV=3
MAASCVLLHTGQKMPLIGLGTWKSEPGQVKAAVKYALSVGYRHIDCAAIYGNEPEIGEAL
KEDVGPGKAVPREELFVTSKLWNTKHHPEDVEPALRKTLADLQLEYLDLYLMHWPYAFER
GDNPFPKNADGTICYDSTHYKETWKALEALVAKGLVQALGLSNFNSRQIDDILSVASVRP
AVLQVECHPYLAQNELIAHCQARGLEVTAYSPLGSSDRAWRDPDEPVLLEEPVVLALAEK
YGRSPAQILLRWQVQRKVICIPKSITPSRILQNIKVFDFTFSPEEMKQLNALNKNWRYIV
PMLTVDGKRVPRDAGHPLYPFNDPY
>sp|P14618|KPYM_HUMAN Pyruvate kinase PKM OS=Homo sapiens OX=9606 GN=PKM PE=1 SV=4
MSKPHSEAGTAFIQTQQLHAAMADTFLEHMCRLDIDSPPITARNTGIICTIGPASRSVET
LKEMIKSGMNVARLNFSHGTHEYHAETIKNVRTATESFASDPILYRPVAVALDTKGPEIR
TGLIKGSGTAEVELKKGATLKITLDNAYMEKCDENILWLDYKNICKVVEVGSKIYVDDGL
ISLQVKQKGADFLVTEVENGGSLGSKKGVNLPGAAVDLPAVSEKDIQDLKFGVEQDVDMV
FASFIRKASDVHEVRKVLGEKGKNIKIISKIENHEGVRRFDEILEASDGIMVARGDLGIE
IPAEKVFLAQKMMIGRCNRAGKPVICATQMLESMIKKPRPTRAEGSDVANAVLDGADCIM
LSGETAKGDYPLEAVRMQHLIAREAEAAIYHLQLFEELRRLAPITSDPTEATAVGAVEAS
FKCCSGAIIVLTKSGRSAHQVARYRPRAPIIAVTRNPQTARQAHLYRGIFPVLCKDPVQE
AWAEDVDLRVNFAMNVGKARGFFKKGDVVIVLTGWRPGSGFTNTMRVVPVP
>sp|P14625|ENPL_HUMAN Endoplasmin OS=Homo sapiens OX=9606 GN=HSP90B1 PE=1 SV=1
MRALWVLGLCCVLLTFGSVRADDEVDVDGTVEEDLGKSREGSRTDDEVVQREEEAIQLDG
LNASQIRELREKSEKFAFQAEVNRMMKLIINSLYKNKEIFLRELISNASDALDKIRLISL
TDENALSGNEELTVKIKCDKEKNLLHVTDTGVGMTREELVKNLGTIAKSGTSEFLNKMTE
AQEDGQSTSELIGQFGVGFYSAFLVADKVIVTSKHNNDTQHIWESDSNEFSVIADPRGNT
LGRGTTITLVLKEEASDYLELDTIKNLVKKYSQFINFPIYVWSSKTETVEEPMEEEEAAK
EEKEESDDEAAVEEEEEEKKPKTKKVEKTVWDWELMNDIKPIWQRPSKEVEEDEYKAFYK
SFSKESDDPMAYIHFTAEGEVTFKSILFVPTSAPRGLFDEYGSKKSDYIKLYVRRVFITD

DFHDMMPKYLNFVKGVVDSDDLPLNVSRETLQQHKLLKVIRKKLVRKTLDMIKKIADDKY
NDTFWKEFGTNIKLGVIEDHSNRTRLAKLLRFQSSHHPTDITSLDQYVERMKEKQDKIYF
MAGSSRKEAESSPFVERLLKKGYEVIYLTEPVDEYCIQALPEFDGKRFQNVAKEGVKFDE
SEKTKESREAVEKEFEPLLNWMKDKALKDKIEKAVVSQRLTESPCALVASQYGWSGNMER
IMKAQAYQTGKDISTNYYASQKKTFEINPRHPLIRDMLRRIKEDEDDKTVLDLAVVLFET
ATLRSGYLLPDTKAYGDRIERMLRLSLNIDPDAKVEEEPEEEPEETAEDTTEDTEQDEDE
EMDVGTDEEEETAKESTAEKDEL
>sp|P14868|SYDC_HUMAN Aspartate--tRNA ligase, cytoplasmic OS=Homo sapiens OX=9606 GN=DARS1 PE=1 SV=2
MPSASASRKSQEKPREIMDAAEDYAKERYGISSMIQSQEKPDRVLVRVRDLTIQKADEVV
WVRARVHTSRAKGKQCFLVLRQQQFNVQALVAVGDHASKQMVKFAANINKESIVDVEGVV
RKVNQKIGSCTQQDVELHVQKIYVISLAEPRLPLQLDDAVRPEAEGEEEGRATVNQDTRL
DNRVIDLRTSTSQAVFRLQSGICHLFRETLINKGFVEIQTPKIISAASEGGANVFTVSYF
KNNAYLAQSPQLYKQMCICADFEKVFSIGPVFRAEDSNTHRHLTEFVGLDIEMAFNYHYH
EVMEEIADTMVQIFKGLQERFQTEIQTVNKQFPCEPFKFLEPTLRLEYCEALAMLREAGV
EMGDEDDLSTPNEKLLGHLVKEKYDTDFYILDKYPLAVRPFYTMPDPRNPKQSNSYDMFM
RGEEILSGAQRIHDPQLLTERALHHGIDLEKIKAYIDSFRFGAPPHAGGGIGLERVTMLF
LGLHNVRQTSMFPRDPKRLTP
>sp|P14923|PLAK_HUMAN Junction plakoglobin OS=Homo sapiens OX=9606 GN=JUP PE=1 SV=3
MEVMNLMEQPIKVTEWQQTYTYDSGIHSGANTCVPSVSSKGIMEEDEACGRQYTLKKTTT
YTQGVPPSQGDLEYQMSTTARAKRVREAMCPGVSGEDSSLLLATQVEGQATNLQRLAEPS
QLLKSAIVHLINYQDDAELATRALPELTKLLNDEDPVVVTKAAMIVNQLSKKEASRRALM
GSPQLVAAVVRTMQNTSDLDTARCTTSILHNLSHHREGLLAIFKSGGIPALVRMLSSPVE
SVLFYAITTLHNLLLYQEGAKMAVRLADGLQKMVPLLNKNNPKFLAITTDCLQLLAYGNQ
ESKLIILANGGPQALVQIMRNYSYEKLLWTTSRVLKVLSVCPSNKPAIVEAGGMQALGKH
LTSNSPRLVQNCLWTLRNLSDVATKQEGLESVLKILVNQLSVDDVNVLTCATGTLSNLTC
NNSKNKTLVTQNSGVEALIHAILRAGDKDDITEPAVCALRHLTSRHPEAEMAQNSVRLNY
GIPAIVKLLNQPNQWPLVKATIGLIRNLALCPANHAPLQEAAVIPRLVQLLVKAHQDAQR
HVAAGTQQPYTDGVRMEEIVEGCTGALHILARDPMNRMEIFRLNTIPLFVQLLYSSVENI
QRVAAGVLCELAQDKEAADAIDAEGASAPLMELLHSRNEGTATYAAAVLFRISEDKNPDY
RKRVSVELTNSLFKHDPAAWEAAQSMIPINEPYGDDMDATYRPMYSSDVPLDPLEMHMDM
DGDYPIDTYSDGLRPPYPTADHMLA
>sp|P15121|ALDR_HUMAN Aldo-keto reductase family 1 member B1 OS=Homo sapiens OX=9606 GN=AKR1B1 PE=1 SV=3
MASRLLLNNGAKMPILGLGTWKSPPGQVTEAVKVAIDVGYRHIDCAHVYQNENEVGVAIQ
EKLREQVVKREELFIVSKLWCTYHEKGLVKGACQKTLSDLKLDYLDLYLIHWPTGFKPGK
EFFPLDESGNVVPSDTNILDTWAAMEELVDEGLVKAIGISNFNHLQVEMILNKPGLKYKP
AVNQIECHPYLTQEKLIQYCQSKGIVVTAYSPLGSPDRPWAKPEDPSLLEDPRIKAIAAK
HNKTTAQVLIRFPMQRNLVVIPKSVTPERIAENFKVFDFELSSQDMTTLLSYNRNWRVCA
LLSCTSHKDYPFHEEF
>sp|P15151|PVR_HUMAN Poliovirus receptor OS=Homo sapiens OX=9606 GN=PVR PE=1 SV=2
MARAMAAAWPLLLVALLVLSWPPPGTGDVVVQAPTQVPGFLGDSVTLPCYLQVPNMEVTH
VSQLTWARHGESGSMAVFHQTQGPSYSESKRLEFVAARLGAELRNASLRMFGLRVEDEGN

YTCLFVTFPQGSRSVDIWLRVLAKPQNTAEVQKVQLTGEPVPMARCVSTGGRPPAQITWH
SDLGGMPNTSQVPGFLSGTVTVTSLWILVPSSQVDGKNVTCKVEHESFEKPQLLTVNLTV
YYPPEVSISGYDNNWYLGQNEATLTCDARSNPEPTGYNWSTTMGPLPPFAVAQGAQLLIR
PVDKPINTTLICNVTNALGARQAELTVQVKEGPPSEHSGISRNAIIFLVLGILVFLILLG
IGIYFYWSKCSREVLWHCHLCPSSTEHASASANGHVSYSAVSRENSSSQDPQTEGTR
>sp|P15170|ERF3A_HUMAN Eukaryotic peptide chain release factor GTP-binding subunit ERF3A OS=Homo sapiens OX=9606 GN=GSPT1 PE=1 SV=1
MELSEPIVENGETEMSPEESWEHKEEISEAEPGGGSLGDGRPPEESAHEMMEEEEEIPKP
KSVVAPPGAPKKEHVNVVFIGHVDAGKSTIGGQIMYLTGMVDKRTLEKYEREAKEKNRET
WYLSWALDTNQEERDKGKTVEVGRAYFETEKKHFTILDAPGHKSFVPNMIGGASQADLAV
LVISARKGEFETGFEKGGQTREHAMLAKTAGVKHLIVLINKMDDPTVNWSNERYEECKEK
LVPFLKKVGFNPKKDIHFMPCSGLTGANLKEQSDFCPWYIGLPFIPYLDNLPNFNRSVDG
PIRLPIVDKYKDMGTVVLGKLESGSICKGQQLVMMPNKHNVEVLGILSDDVETDTVAPGE
NLKIRLKGIEEEEILPGFILCDPNNLCHSGRTFDAQIVIIEHKSIICPGYNAVLHIHTCI
EEVEITALICLVDKKSGEKSKTRPRFVKQDQVCIARLRTAGTICLETFKDFPQMGRFTLR
DEGKTIAIGKVLKLVPEKD
>sp|P15259|PGAM2_HUMAN Phosphoglycerate mutase 2 OS=Homo sapiens OX=9606 GN=PGAM2 PE=1 SV=3
MATHRLVMVRHGESTWNQENRFCGWFDAELSEKGTEEAKRGAKAIKDAKMEFDICYTSVL
KRAIRTLWAILDGTDQMWLPVVRTWRLNERHYGGLTGLNKAETAAKHGEEQVKIWRRSFD
IPPPPMDEKHPYYNSISKERRYAGLKPGELPTCESLKDTIARALPFWNEEIVPQIKAGKR
VLIAAHGNSLRGIVKHLEGMSDQAIMELNLPTGIPIVYELNKELKPTKPMQFLGDEETVR
KAMEAVAAQGKAK
>sp|P15311|EZRI_HUMAN Ezrin OS=Homo sapiens OX=9606 GN=EZR PE=1 SV=4
MPKPINVRVTTMDAELEFAIQPNTTGKQLFDQVVKTIGLREVWYFGLHYVDNKGFPTWLK
LDKKVSAQEVRKENPLQFKFRAKFYPEDVAEELIQDITQKLFFLQVKEGILSDEIYCPPE
TAVLLGSYAVQAKFGDYNKEVHKSGYLSSERLIPQRVMDQHKLTRDQWEDRIQVWHAEHR
GMLKDNAMLEYLKIAQDLEMYGINYFEIKNKKGTDLWLGVDALGLNIYEKDDKLTPKIGF
PWSEIRNISFNDKKFVIKPIDKKAPDFVFYAPRLRINKRILQLCMGNHELYMRRRKPDTI
EVQQMKAQAREEKHQKQLERQQLETEKKRRETVEREKEQMMREKEELMLRLQDYEEKTKK
AERELSEQIQRALQLEEERKRAQEEAERLEADRMAALRAKEELERQAVDQIKSQEQLAAE
LAEYTAKIALLEEARRRKEDEVEEWQHRAKEAQDDLVKTKEELHLVMTAPPPPPPPVYEP
VSYHVQESLQDEGAEPTGYSAELSSEGIRDDRNEEKRITEAEKNERVQRQLLTLSSELSQ
ARDENKRTHNDIIHNENMRQGRDKYKTLRQIRQGNTKQRIDEFEAL
>sp|P15924|DESP_HUMAN Desmoplakin OS=Homo sapiens OX=9606 GN=DSP PE=1 SV=3
MSCNGGSHPRINTLGRMIRAESGPDLRYEVTSGGGGTSRMYYSRRGVITDQNSDGYCQTG
TMSRHQNQNTIQELLQNCSDCLMRAELIVQPELKYGDGIQLTRSRELDECFAQANDQMEI
LDSLIREMRQMGQPCDAYQKRLLQLQEQMRALYKAISVPRVRRASSKGGGGYTCQSGSGW
DEFTKHVTSECLGWMRQQRAEMDMVAWGVDLASVEQHINSHRGIHNSIGDYRWQLDKIKA
DLREKSAIYQLEEEYENLLKASFERMDHLRQLQNIIQATSREIMWINDCEEEELLYDWSD
KNTNIAQKQEAFSIRMSQLEVKEKELNKLKQESDQLVLNQHPASDKIEAYMDTLQTQWSW
ILQITKCIDVHLKENAAYFQFFEEAQSTEAYLKGLQDSIRKKYPCDKNMPLQHLLEQIKE
LEKEREKILEYKRQVQNLVNKSKKIVQLKPRNPDYRSNKPIILRALCDYKQDQKIVHKGD
ECILKDNNERSKWYVTGPGGVDMLVPSVGLIIPPPNPLAVDLSCKIEQYYEAILALWNQL

YINMKSLVSWHYCMIDIEKIRAMTIAKLKTMRQEDYMKTIADLELHYQEFIRNSQGSEMF
GDDDKRKIQSQFTDAQKHYQTLVIQLPGYPQHQTVTTTEITHHGTCQDVNHNKVIETNRE
NDKQETWMLMELQKIRRQIEHCEGRMTLKNLPLADQGSSHHITVKINELKSVQNDSQAIA
EVLNQLKDMLANFRGSEKYCYLQNEVFGLFQKLENINGVTDGYLNSLCTVRALLQAILQT
EDMLKVYEARLTEEETVCLDLDKVEAYRCGLKKIKNDLNLKKSLLATMKTELQKAQQIHS
QTSQQYPLYDLDLGKFGEKVTQLTDRWQRIDKQIDFRLWDLEKQIKQLRNYRDNYQAFCK
WLYDAKRRQDSLESMKFGDSNTVMRFLNEQKNLHSEISGKRDKSEEVQKIAELCANSIKD
YELQLASYTSGLETLLNIPIKRTMIQSPSGVILQEAADVHARYIELLTRSGDYYRFLSEM
LKSLEDLKLKNTKIEVLEEELRLARDANSENCNKNKFLDQNLQKYQAECSQFKAKLASLE
ELKRQAELDGKSAKQNLDKCYGQIKELNEKITRLTYEIEDEKRRRKSVEDRFDQQKNDYD
QLQKARQCEKENLGWQKLESEKAIKEKEYEIERLRVLLQEEGTRKREYENELAKVRNHYN
EEMSNLRNKYETEINITKTTIKEISMQKEDDSKNLRNQLDRLSRENRDLKDEIVRLNDSI
LQATEQRRRAEENALQQKACGSEIMQKKQHLEIELKQVMQQRSEDNARHKQSLEEAAKTI
QDKNKEIERLKAEFQEEAKRRWEYENELSKVRNNYDEEIISLKNQFETEINITKTTIHQL
TMQKEEDTSGYRAQIDNLTRENRSLSEEIKRLKNTLTQTTENLRRVEEDIQQQKATGSEV
SQRKQQLEVELRQVTQMRTEESVRYKQSLDDAAKTIQDKNKEIERLKQLIDKETNDRKCL
EDENARLQRVQYDLQKANSSATETINKLKVQEQELTRLRIDYERVSQERTVKDQDITRFQ
NSLKELQLQKQKVEEELNRLKRTASEDSCKRKKLEEELEGMRRSLKEQAIKITNLTQQLE
QASIVKKRSEDDLRQQRDVLDGHLREKQRTQEELRRLSSEVEALRRQLLQEQESVKQAHL
RNEHFQKAIEDKSRSLNESKIEIERLQSLTENLTKEHLMLEEELRNLRLEYDDLRRGRSE
ADSDKNATILELRSQLQISNNRTLELQGLINDLQRERENLRQEIEKFQKQALEASNRIQE
SKNQCTQVVQERESLLVKIKVLEQDKARLQRLEDELNRAKSTLEAETRVKQRLECEKQQI
QNDLNQWKTQYSRKEEAIRKIESEREKSEREKNSLRSEIERLQAEIKRIEERCRRKLEDS
TRETQSQLETERSRYQREIDKLRQRPYGSHRETQTECEWTVDTSKLVFDGLRKKVTAMQL
YECQLIDKTTLDKLLKGKKSVEEVASEIQPFLRGAGSIAGASASPKEKYSLVEAKRKKLI
SPESTVMLLEAQAATGGIIDPHRNEKLTVDSAIARDLIDFDDRQQIYAAEKAITGFDDPF
SGKTVSVSEAIKKNLIDRETGMRLLEAQIASGGVVDPVNSVFLPKDVALARGLIDRDLYR
SLNDPRDSQKNFVDPVTKKKVSYVQLKERCRIEPHTGLLLLSVQKRSMSFQGIRQPVTVT
ELVDSGILRPSTVNELESGQISYDEVGERIKDFLQGSSCIAGIYNETTKQKLGIYEAMKI
GLVRPGTALELLEAQAATGFIVDPVSNLRLPVEEAYKRGLVGIEFKEKLLSAERAVTGYN
DPETGNIISLFQAMNKELIEKGHGIRLLEAQIATGGIIDPKESHRLPVDIAYKRGYFNEE
LSEILSDPSDDTKGFFDPNTEENLTYLQLKERCIKDEETGLCLLPLKEKKKQVQTSQKNT
LRKRRVVIVDPETNKEMSVQEAYKKGLIDYETFKELCEQECEWEEITITGSDGSTRVVLV
DRKTGSQYDIQDAIDKGLVDRKFFDQYRSGSLSLTQFADMISLKNGVGTSSSMGSGVSDD
VFSSSRHESVSKISTISSVRNLTIRSSSFSDTLEESSPIAAIFDTENLEKISITEGIERG
IVDSITGQRLLEAQACTGGIIHPTTGQKLSLQDAVSQGVIDQDMATRLKPAQKAFIGFEG
VKGKKKMSAAEAVKEKWLPYEAGQRFLEFQYLTGGLVDPEVHGRISTEEAIRKGFIDGRA
AQRLQDTSSYAKILTCPKTKLKISYKDAINRSMVEDITGLRLLEAASVSSKGLPSPYNMS
SAPGSRSGSRSGSRSGSRSGSRSGSRRGSFDATGNSSYSYSYSFSSSSIGH
>sp|P16035|TIMP2_HUMAN Metalloproteinase inhibitor 2 OS=Homo sapiens OX=9606
GN=TIMP2 PE=1 SV=2
MGAAARTLRLALGLLLLATLLRPADACSCSPVHPQQAFCNADVVIRAKAVSEKEVDSGND
IYGNPIKRIQYEIKQIKMFKGPEKDIEFIYTAPSSAVCGVSLDVGGKKEYLIAGKAEGDG
KMHITLCDFIVPWDTLSTTQKKSLNHRYQMGCECKITRCPMIPCYISSPDECLWMDWVTE

KNINGHQAKFFACIKRSDGSCAWYRGAAPPKQEFLDIEDP
>sp|P16104|H2AX_HUMAN Histone H2AX OS=Homo sapiens OX=9606 GN=H2AX PE=1 SV=2
MSGRGKTGGKARAKAKSRSSRAGLQFPVGRVHRLLRKGHYAERVGAGAPVYLAAVLEYLT
AEILELAGNAARDNKKTRIIPRHLQLAIRNDEELNKLLGGVTIAQGGVLPNIQAVLLPKK
TSATVGPKAPSGGKKATQASQEY
>sp|P16152|CBR1_HUMAN Carbonyl reductase [NADPH] 1 OS=Homo sapiens OX=9606
GN=CBR1 PE=1 SV=3
MSSGIHVALVTGGNKGIGLAIVRDLCRLFSGDVVLTARDVTRGQAAVQQLQAEGLSPRFH
QLDIDDLQSIRALRDFLRKEYGGLDVLVNNAGIAFKVADPTPFHIQAEVTMKTNFFGTRD
VCTELLPLIKPQGRVVNVSSIMSVRALKSCSPELQQKFRSETITEEELVGLMNKFVEDTK
KGVHQKEGWPSSAYGVTKIGVTVLSRIHARKLSEQRKGDKILLNACCPGWVRTDMAGPKA
TKSPEEGAETPVYLALLPPDAEGPHGQFVSEKRVEQW
>sp|P16401|H15_HUMAN Histone H1.5 OS=Homo sapiens OX=9606 GN=H1-5 PE=1 SV=3
MSETAPAETATPAPVEKSPAKKKATKKAAGAGAAKRKATGPPVSELITKAVAASKERNGL
SLAALKKALAAGGYDVEKNNSRIKLGLKSLVSKGTLVQTKGTGASGSFKLNKKAASGEAK
PKAKKAGAAKAKKPAGATPKKAKKAAGAKKAVKKTPKKAKKPAAAGVKKVAKSPKKAKAA
AKPKKATKSPAKPKAVKPKAAKPKAAKPKAAKPKAAKAKKAAAKKK
>sp|P16403|H12_HUMAN Histone H1.2 OS=Homo sapiens OX=9606 GN=H1-2 PE=1 SV=2
MSETAPAAPAAAPPAEKAPVKKKAAKKAGGTPRKASGPPVSELITKAVAASKERSGVSLA
ALKKALAAAGYDVEKNNSRIKLGLKSLVSKGTLVQTKGTGASGSFKLNKKAASGEAKPKV
KKAGGTKPKKPVGAAKKPKKAAGGATPKKSAKKTPKKAKKPAAATVTKKVAKSPKKAKVA
KPKKAAKSAAKAVKPKAAKPKVVKPKKAAPKKK
>sp|P16435|NCPR_HUMAN NADPH--cytochrome P450 reductase OS=Homo sapiens OX=9606
GN=POR PE=1 SV=2
MGDSHVDTSSTVSEAVAEEVSLFSMTDMILFSLIVGLLTYWFLFRKKKEEVPEFTKIQTL
TSSVRESSFVEKMKKTGRNIIVFYGSQTGTAEEFANRLSKDAHRYGMRGMSADPEEYDLA
DLSSLPEIDNALVVFCMATYGEGDPTDNAQDFYDWLQETDVDLSGVKFAVFGLGNKTYEH
FNAMGKYVDKRLEQLGAQRIFELGLGDDDGNLEEDFITWREQFWPAVCEHFGVEATGEES
SIRQYELVVHTDIDAAKVYMGEMGRLKSYENQKPPFDAKNPFLAAVTTNRKLNQGTERHL
MHLELDISDSKIRYESGDHVAVYPANDSALVNQLGKILGADLDVVMSLNNLDEESNKKHP
FPCPTSYRTALTYYLDITNPPRTNVLYELAQYASEPSEQELLRKMASSSGEGKELYLSWV
VEARRHILAILQDCPSLRPPIDHLCELLPRLQARYYSIASSSKVHPNSVHICAVVVEYET
KAGRINKGVATNWLRAKEPAGENGGRALVPMFVRKSQFRLPFKATTPVIMVGPGTGVAPF
IGFIQERAWLRQQGKEVGETLLYYGCRRSDEDYLYREELAQFHRDGALTQLNVAFSREQS
HKVYVQHLLKQDREHLWKLIEGGAHIYVCGDARNMARDVQNTFYDIVAELGAMEHAQAVD
YIKKLMTKGRYSLDVWS
>sp|P16930|FAAA_HUMAN Fumarylacetoacetase OS=Homo sapiens OX=9606 GN=FAH PE=1
SV=2
MSFIPVAEDSDFPIHNLPYGVFSTRGDPRPRIGVAIGDQILDLSIIKHLFTGPVLSKHQD
VFNQPTLNSFMGLGQAAWKEARVFLQNLLSVSQARLRDDTELRKCAFISQASATMHLPAT
IGDYTDFYSSRQHATNVGIMFRDKENALMPNWLHLPVGYHGRASSVVVSGTPIRRPMGQM
KPDDSKPPVYGACKLLDMELEMAFFVGPGNRLGEPIPISKAHEHIFGMVLMNDWSARDIQ
KWEYVPLGPFLGKSFGTTVSPWVVPMDALMPFAVPNPKQDPRPLPYLCHDEPYTFDINLS
VNLKGEGMSQAATICKSNFKYMYWTMLQQLTHHSVNGCNLRPGDLLASGTISGPEPENFG

SMLELSWKGTKPIDLGNGQTRKFLLDGDEVIITGYCQGDGYRIGFGQCAGKVLPALLPS
>sp|P17301|ITA2_HUMAN Integrin alpha-2 OS=Homo sapiens OX=9606 GN=ITGA2 PE=1 SV=2
MGPERTGAAPLPLLLVLALSQGILNCCLAYNVGLPEAKIFSGPSSEQFGYAVQQFINPKG
NWLLVGSPWSGFPENRMGDVYKCPVDLSTATCEKLNLQTSTSIPNVTEMKTNMSLGLILT
RNMGTGGFLTCGPLWAQQCGNQYYTTGVCSDISPDFQLSASFSPATQPCPSLIDVVVVCD
ESNSIYPWDAVKNFLEKFVQGLDIGPTKTQVGLIQYANNPRVVFNLNTYKTKEEMIVATS
QTSQYGGDLTNTFGAIQYARKYAYSAASGGRRSATKVMVVVTDGESHDGSMLKAVIDQCN
HDNILRFGIAVLGYLNRNALDTKNLIKEIKAIASIPTERYFFNVSDEAALLEKAGTLGEQ
IFSIEGTVQGGDNFQMEMSQVGFSADYSSQNDILMLGAVGAFGWSGTIVQKTSHGHLIFP
KQAFDQILQDRNHSSYLGYSVAAISTGESTHFVAGAPRANYTGQIVLYSVNENGNITVIQ
AHRGDQIGSYFGSVLCSVDVDKDTITDVLLVGAPMYMSDLKKEEGRVYLFTIKEGILGQH
QFLEGPEGIENTRFGSAIAALSDINMDGFNDVIVGSPLENQNSGAVYIYNGHQGTIRTKY
SQKILGSDGAFRSHLQYFGRSLDGYGDLNGDSITDVSIGAFGQVVQLWSQSIADVAIEAS
FTPEKITLVNKNAQIILKLCFSAKFRPTKQNNQVAIVYNITLDADGFSSRVTSRGLFKEN
NERCLQKNMVVNQAQSCPEHIIYIQEPSDVVNSLDLRVDISLENPGTSPALEAYSETAKV
FSIPFHKDCGEDGLCISDLVLDVRQIPAAQEQPFIVSNQNKRLTFSVTLKNKRESAYNTG
IVVDFSENLFFASFSLPVDGTEVTCQVAASQKSVACDVGYPALKREQQVTFTINFDFNLQ
NLQNQASLSFQALSESQEENKADNLVNLKIPLLYDAEIHLTRSTNINFYEISSDGNVPSI
VHSFEDVGPKFIFSLKVTTGSVPVSMATVIIHIPQYTKEKNPLMYLTGVQTDKAGDISCN
ADINPLKIGQTSSSVSFKSENFRHTKELNCRTASCSNVTCWLKDVHMKGEYFVNVTTRIW
NGTFASSTFQTVQLTAAAEINTYNPEIYVIEDNTVTIPLMIMKPDEKAEVPTGVIIGSII
AGILLLLALVAILWKLGFFKRKYEKMTKNPDEIDETTELSS
>sp|P17612|KAPCA_HUMAN cAMP-dependent protein kinase catalytic subunit alpha OS=Homo sapiens OX=9606 GN=PRKACA PE=1 SV=2
MGNAAAAKKGSEQESVKEFLAKAKEDFLKKWESPAQNTAHLDQFERIKTLGTGSFGRVML
VKHKETGNHYAMKILDKQKVVKLKQIEHTLNEKRILQAVNFPFLVKLEFSFKDNSNLYMV
MEYVPGGEMFSHLRRIGRFSEPHARFYAAQIVLTFEYLHSLDLIYRDLKPENLLIDQQGY
IQVTDFGFAKRVKGRTWTLCGTPEYLAPEIILSKGYNKAVDWWALGVLIYEMAAGYPPFF
ADQPIQIYEKIVSGKVRFPSHFSSDLKDLLRNLLQVDLTKRFGNLKNGVNDIKNHKWFAT
TDWIAIYQRKVEAPFIPKFKGPGDTSNFDDYEEEEIRVSINEKCGKEFSEF
>sp|P17858|PFKAL_HUMAN ATP-dependent 6-phosphofructokinase, liver type OS=Homo sapiens OX=9606 GN=PFKL PE=1 SV=6
MAAVDLEKLRASGAGKAIGVLTSGGDAQGMNAAVRAVTRMGIYVGAKVFLIYEGYEGLVE
GGENIKQANWLSVSNIIQLGGTIIGSARCKAFTTREGRRAAAYNLVQHGITNLCVIGGDG
SLTGANIFRSEWGSLLEELVAEGKISETTARTYSHLNIAGLVGSIDNDFCGTDMTIGTDS
ALHRIMEVIDAITTTAQSHQRTFVLEVMGRHCGYLALVSALASGADWLFIPEAPPEDGWE
NFMCERLGETRSRGSRLNIIIIAEGAIDRNGKPISSSYVKDLVVQRLGFDTRVTVLGHVQ
RGGTPSAFDRILSSKMGMEAVMALLEATPDTPACVVTLSGNQSVRLPLMECVQMTKEVQK
AMDDKRFDEATQLRGGSFENNWNIYKLLAHQKPPKEKSNFSLAILNVGAPAAGMNAAVRS
AVRTGISHGHTVYVVHDGFEGLAKGQVQEVGWHDVAGWLGRGGSMLGTKRTLPKGQLESI
VENIRIYGIHALLVVGGFEAYEGVLQLVEARGRYEELCIVMCVIPATISNNVPGTDFSLG
SDTAVNAAMESCDRIKQSASGTKRRVFIVETMGGYCGYLATVTGIAVGADAAYVFEDPFN
IHDLKVNVEHMTEKMKTDIQRGLVLRNEKCHDYYTTEFLYNLYSSEGKGVFDCRTNVLGH
LQQGGAPTPFDRNYGTKLGVKAMLWLSEKLREVYRKGRVFANAPDSACVIGLKKKAVAFS

PVTELKKDTDFEHRMPREQWWLSLRLMLKMLAQYRISMAAYVSGELEHVTRRTLSMDKGF
>sp|P17931|LEG3_HUMAN Galectin-3 OS=Homo sapiens OX=9606 GN=LGALS3 PE=1 SV=5
MADNFSLHDALSGSGNPNPQGWPGAWGNQPAGAGGYPGASYPGAYPGQAPPGAYPGQAPP
GAYPGAPGAYPGAPAPGVYPGPPSGPGAYPSSGQPSATGAYPATGPYGAPAGPLIVPYNL
PLPGGVVPRMLITILGTVKPNANRIALDFQRGNDVAFHFNPRFNENNRRVIVCNTKLDNN
WGREERQSVFPFESGKPFKIQVLVEPDHFKVAVNDAHLLQYNHRVKKLNEISKLGISGDI
DLTSASYTMI
>sp|P17987|TCPA_HUMAN T-complex protein 1 subunit alpha OS=Homo sapiens OX=9606 GN=TCP1 PE=1 SV=1
MEGPLSVFGDRSTGETIRSQNVMAAASIANIVKSSLGPVGLDKMLVDDIGDVTITNDGAT
ILKLLEVEHPAAKVLCELADLQDKEVGDGTTSVVIIAAELLKNADELVKQKIHPTSVISG
YRLACKEAVRYINENLIVNTDELGRDCLINAAKTSMSSKIIGINGDFFANMVVDAVLAIK
YTDIRGQPRYPVNSVNILKAHGRSQMESMLISGYALNCVVGSQGMPKRIVNAKIACLDFS
LQKTKMKLGVQVVITDPEKLDQIRQRESDITKERIQKILATGANVILTTGGIDDMCLKYF
VEAGAMAVRRVLKRDLKRIAKASGATILSTLANLEGEETFEAAMLGQAEEVVQERICDDE
LILIKNTKARTSASIILRGANDFMCDEMERSLHDALCVVKRVLESKSVVPGGGAVEAALS
IYLENYATSMGSREQLAIAEFARSLLVIPNTLAVNAAQDSTDLVAKLRAFHNEAQVNPER
KNLKWIGLDLSNGKPRDNKQAGVFEPTIVKVKSLKFATEAAITILRIDDLIKLHPESKDD
KHGSYEDAVHSGALND
>sp|P18085|ARF4_HUMAN ADP-ribosylation factor 4 OS=Homo sapiens OX=9606 GN=ARF4 PE=1 SV=3
MGLTISSLFSRLFGKKQMRILMVGLDAAGKTTILYKLKLGEIVTTIPTIGFNVETVEYKN
ICFTVWDVGGQDRIRPLWKHYFQNTQGLIFVVDSNDRERIQEVADELQKMLLVDELRDAV
LLLFANKQDLPNAMAISEMTDKLGLQSLRNRTWYVQATCATQGTGLYEGLDWLSNELSKR
>sp|P18124|RL7_HUMAN Large ribosomal subunit protein uL30 OS=Homo sapiens OX=9606 GN=RPL7 PE=1 SV=1
MEGVEEKKKEVPAVPETLKKKRRNFAELKIKRLRKKFAQKMLRKARRKLIYEKAKHYHKE
YRQMYRTEIRMARMARKAGNFYVPAEPKLAFVIRIRGINGVSPKVRKVLQLLRLRQIFNG
TFVKLNKASINMLRIVEPYIAWGYPNLKSVNELIYKRGYGKINKKRIALTDNALIARSLG
KYGIICMEDLIHEIYTVGKRFKEANNFLWPFKLSSPRGGMKKKTTHFVEGGDAGNREDQI
NRLIRRMN
>sp|P18206|VINC_HUMAN Vinculin OS=Homo sapiens OX=9606 GN=VCL PE=1 SV=4
MPVFHTRTIESILEPVAQQISHLVIMHEEGEVDGKAIPDLTAPVAAVQAAVSNLVRVGKE
TVQTTEDQILKRDMPPAFIKVENACTKLVQAAQMLQSDPYSVPARDYLIDGSRGILSGTS
DLLLTFDEAEVRKIIRVCKGILEYLTVAEVVETMEDLVTYTKNLGPGMTKMAKMIDERQQ
ELTHQEHRVMLVNSMNTVKELLPVLISAMKIFVTTKNSKNQGIEEALKNRNFTVEKMSAE
INEIIRVLQLTSWDEDAWASKDTEAMKRALASIDSKLNQAKGWLRDPSASPGDAGEQAIR
QILDEAGKVGELCAGKERREILGTCKMLGQMTDQVADLRARGQGSSPVAMQKAQQVSQGL
DVLTAKVENAARKLEAMTNSKQSIAKKIDAAQNWLADPNGGPEGEEQIRGALAEARKIAE
LCDDPKERDDILRSLGEISALTSKLADLRRQGKGDSPEARALAKQVATALQNLQTKTNRA
VANSRPAKAAVHLEGKIEQAQRWIDNPTVDDRGVGQAAIRGLVAEGHRLANVMMGPYRQD
LLAKCDRVDQLTAQLADLAARGEGESPQARALASQLQDSLKDLKARMQEAMTQEVSDVFS
DTTTPIKLLAVAATAPPDAPNREEVFDERAANFENHSGKLGATAEKAAAVGTANKSTVEG
IQASVKTARELTPQVVSAARILLRNPGNQAAYEHFETMKNQWIDNVEKMTGLVDEAIDTK

SLLDASEEAIKKDLDKCKVAMANIQPQMLVAGATSIARRANRILLVAKREVENSEDPKFR
EAVKAASDELSKTISPMVMDAKAVAGNISDPGLQKSFLDSGYRILGAVAKVREAFQPQEP
DFPPPPPDLEQLRLTDELAPPKPPLPEGEVPPPRPPPPEEKDEEFPEQKAGEVINQPMMM
AARQLHDEARKWSSKPGIPAAEVGIGVVAEADAADAAGFPVPPDMEDDYEPELLLMPSNQ
PVNQPILAAAQSLHREATKWSSKGNDIIAAAKRMALLMAEMSRLVRGGSGTKRALIQCAK
DIAKASDEVTRLAKEVAKQCTDKRIRTNLLQVCERIPTISTQLKILSTVKATMLGRTNIS
DEESEQATEMLVHNAQNLMQSVKETVREAEAASIKIRTDAGFTLRWVRKTPWYQ
>sp|P18433|PTPRA_HUMAN Receptor-type tyrosine-protein phosphatase alpha OS=Homo sapiens OX=9606 GN=PTPRA PE=1 SV=3
MDSWFILVLLGSGLICVSANNATTVAPSVGITRLINSSTAEPVKEEAKTSNPTSSLTSLS
VAPTFSPNITLGPTYLTTVNSSDSDNGTTRTASTNSIGITISPNGTWLPDNQFTDARTEP
WEGNSSTAATTPETFPPSGNSDSKDRRDETPIIAVMVALSSLLVIVFIIIVLYMLRFKKY
KQAGSHSNSFRLSNGRTEDVEPQSVPLLARSPSTNRKYPPLPVDKLEEEINRRMADDNKL
FREEFNALPACPIQATCEAASKEENKEKNRYVNILPYDHSRVHLTPVEGVPDSDYINASF
INGYQEKNKFIAAQGPKEETVNDFWRMIWEQNTATIVMVTNLKERKECKCAQYWPDQGCW
TYGNIRVSVEDVTVLVDYTVRKFCIQQVGDMTNRKPQRLITQFHFTSWPDFGVPFTPIGM
LKFLKKVKACNPQYAGAIVVHCSAGVGRTGTFVVIDAMLDMMHTERKVDVYGFVSRIRAQ
RCQMVQTDMQYVFIYQALLEHYLYGDTELEVTSLETHLQKIYNKIPGTSNNGLEEEFKKL
TSIKIQNDKMRTGNLPANMKKNRVLQIIPYEFNRVIIPVKRGEENTDYVNASFIDGYRQK
DSYIASQGPLLHTIEDFWRMIWEWKSCSIVMLTELEERGQEKCAQYWPSDGLVSYGDITV
ELKKEEECESYTVRDLLVTNTRENKSRQIRQFHFHGWPEVGIPSDGKGMISIIAAVQKQQ
QQSGNHPITVHCSAGAGRTGTFCALSTVLERVKAEGILDVFQTVKSLRLQRPHMVQTLEQ
YEFCYKVVQEYIDAFSDYANFK
>sp|P18669|PGAM1_HUMAN Phosphoglycerate mutase 1 OS=Homo sapiens OX=9606 GN=PGAM1 PE=1 SV=2
MAAYKLVLIRHGESAWNLENRFSGWYDADLSPAGHEEAKRGGQALRDAGYEFDICFTSVQ
KRAIRTLWTVLDAIDQMWLPVVRTWRLNERHYGGLTGLNKAETAAKHGEAQVKIWRRSYD
VPPPPMEPDHPFYSNISKDRRYADLTEDQLPSCESLKDTIARALPFWNEEIVPQIKEGKR
VLIAAHGNSLRGIVKHLEGLSEEAIMELNLPTGIPIVYELDKNLKPIKPMQFLGDEETVR
KAMEAVAAQGKAKK
>sp|P19022|CADH2_HUMAN Cadherin-2 OS=Homo sapiens OX=9606 GN=CDH2 PE=1 SV=4
MCRIAGALRTLLPLLAALLQASVEASGEIALCKTGFPEDVYSAVLSKDVHEGQPLLNVKF
SNCNGKRKVQYESSEPADFKVDEDGMVYAVRSFPLSSEHAKFLIYAQDKETQEKWQVAVK
LSLKPTLTEESVKESAEVEEIVFPRQFSKHSGHLQRQKRDWVIPPINLPENSRGPFPQEL
VRIRSDRDKNLSLRYSVTGPGADQPPTGIFIINPISGQLSVTKPLDREQIARFHLRAHAV
DINGNQVENPIDIVINVIDMNDNRPEFLHQVWNGTVPEGSKPGTYVMTVTAIDADDPNAL
NGMLRYRIVSQAPSTPSPNMFTINNETGDIITVAAGLDREKVQQYTLIIQATDMEGNPTY
GLSNTATAVITVTDVNDNPPEFTAMTFYGEVPENRVDIIVANLTVTDKDQPHTPAWNAVY
RISGGDPTGRFAIQTDPNSNDGLVTVVKPIDFETNRMFVLTVAAENQVPLAKGIQHPPQS
TATVSVTVIDVNENPYFAPNPKIIRQEEGLHAGTMLTTFTAQDPDRYMQQNIRYTKLSDP
ANWLKIDPVNGQITTIAVLDRESPNVKNNIYNATFLASDNGIPPMSGTGTLQIYLLDIND
NAPQVLPQEAETCETPDPNSINITALDYDIDPNAGPFAFDLPLSPVTIKRNWTITRLNGD
FAQLNLKIKFLEAGIYEVPIIITDSGNPPKSNISILRVKVCQCDSNGDCTDVDRIVGAGL
GTGAIIAILLCIIILLILVLMFVVWMKRRDKERQAKQLLIDPEDDVRDNILKYDEEGGGE

EDQDYDLSQLQQPDTVEPDAIKPVGIRRMDERPIHAEPQYPVRSAAPHPGDIGDFINEGL
KAADNDPTAPPYDSLLVFDYEGSGSTAGSLSSLNSSSSGGEQDYDYLNDWGPRFKKLADM
YGGGDD
>sp|P19801|AOC1_HUMAN Amiloride-sensitive amine oxidase [copper-containing] OS=Homo sapiens OX=9606 GN=AOC1 PE=1 SV=4
MPALGWAVAAILMLQTAMAEPSPGTLPRKAGVFSDLSNQELKAVHSFLWSKKELRLQPSS
TTTMAKNTVFLIEMLLPKKYHVLRFLDKGERHPVREARAVIFFGDQEHPNVTEFAVGPLP
GPCYMRALSPRPGYQSSWASRPISTAEYALLYHTLQEATKPLHQFFLNTTGFSFQDCHDR
CLAFTDVAPRGVASGQRRSWLIIQRYVEGYFLHPTGLELLVDHGSTDAGHWAVEQVWYNG
KFYGSPEELARKYADGEVDVVVLEDPLPGGKGHDSTEEPPLFSSHKPRGDFPSPIHVSGP
RLVQPHGPRFRLEGNAVLYGGWSFAFRLRSSSGLQVLNVHFGGERIAYEVSVQEAVALYG
GHTPAGMQTKYLDVGWGLGSVTHELAPGIDCPETATFLDTFHYYDADDPVHYPRALCLFE
MPTGVPLRRHFNSNFKGGFNFYAGLKGQVLVLRTTSTVYNYDYIWDFIFYPNGVMEAKMH
ATGYVHATFYTPEGLRHGTRLHTHLIGNIHTHLVHYRVDLDVAGTKNSFQTLQMKLENIT
NPWSPRHRVVQPTLEQTQYSWERQAAFRFKRKLPKYLLFTSPQENPWGHKRTYRLQIHSM
ADQVLPPGWQEEQAITWARYPLAVTKYRESELCSSSIYHQNDPWHPPVVFEQFLHNNENI
ENEDLVAWVTVGFLHIPHSEDIPNTATPGNSVGFLLRPFNFFPEDPSLASRDTVIVWPRD
NGPNYVQRWIPEDRDCSMPPPFSYNGTYRPV
>sp|P20339|RAB5A_HUMAN Ras-related protein Rab-5A OS=Homo sapiens OX=9606 GN=RAB5A PE=1 SV=2
MASRGATRPNGPNTGNKICQFKLVLLGESAVGKSSLVLRFVKGQFHEFQESTIGAAFLTQ
TVCLDDTTVKFEIWDTAGQERYHSLAPMYYRGAQAAIVVYDITNEESFARAKNWVKELQR
QASPNIVIALSGNKADLANKRAVDFQEAQSYADDNSLLFMETSAKTSMNVNEIFMAIAKK
LPKNEPQNPGANSARGRGVDLTEPTQPTRNQCCSN
>sp|P20340|RAB6A_HUMAN Ras-related protein Rab-6A OS=Homo sapiens OX=9606 GN=RAB6A PE=1 SV=3
MSTGGDFGNPLRKFKLVFLGEQSVGKTSLITRFMYDSFDNTYQATIGIDFLSKTMYLEDR
TVRLQLWDTAGQERFRSLIPSYIRDSTVAVVVYDITNVNSFQQTTKWIDDVRTERGSDVI
IMLVGNKTDLADKRQVSIEEGERKAKELNVMFIETSAKAGYNVKQLFRRVAAALPGMEST
QDRSREDMIDIKLEKPQEQPVSEGGCSC
>sp|P20839|IMDH1_HUMAN Inosine-5'-monophosphate dehydrogenase 1 OS=Homo sapiens OX=9606 GN=IMPDH1 PE=1 SV=2
MADYLISGGTGYVPEDGLTAQQLFASADGLTYNDFLILPGFIDFIADEVDLTSALTRKIT
LKTPLISSPMDTVTEADMAIAMALMGGIGFIHHNCTPEFQANEVRKVKKFEQGFITDPVV
LSPSHTVGDVLEAKMRHGFSGIPITETGTMGSKLVGIVTSRDIDFLAEKDHTTLLSEVMT
PRIELVVAPAGVTLKEANEILQRSKKGKLPIVNDCDELVAIIARTDLKKNRDYPLASKDS
QKQLLCGAAVGTREDDKYRLDLLTQAGVDVIVLDSSQGNSVYQIAMVHYIKQKYPHLQVI
GGNVVTAAQAKNLIDAGVDGLRVGMGCGSICITQEVMACGRPQGTAVYKVAEYARRFGVP
IIADGGIQTVGHVVKALALGASTVMMGSLLAATTEAPGEYFFSDGVRLKKYRGMGSLDAM
EKSSSSQKRYFSEGDKVKIAQGVSGSIQDKGSIQKFVPYLIAGIQHGCQDIGARSLSVLR
SMMYSGELKFEKRTMSAQIEGGVHGLHSYEKRLY
>sp|P21246|PTN_HUMAN Pleiotrophin OS=Homo sapiens OX=9606 GN=PTN PE=1 SV=1
MQAQQYQQQRRKFAAAFLAFIFILAAVDTAEAGKKEKPEKKVKKSDCGEWQWSVCVPTSG
DCGLGTREGTRTGAECKQTMKTQRCKIPCNWKKQFGAECKYQFQAWGECDLNTALKTRTG

SLKRALHNAECQKTVTISKPCGKLTKPKPQAESKKKKKEGKKQEKMLD
>sp|P21266|GSTM3_HUMAN Glutathione S-transferase Mu 3 OS=Homo sapiens OX=9606 GN=GSTM3 PE=1 SV=3
MSCESSMVLGYWDIRGLAHAIRLLLEFTDTSYEEKRYTCGEAPDYDRSQWLDVKFKLDLD
FPNLPYLLDGKNKITQSNAILRYIARKHNMCGETEEEKIRVDIIENQVMDFRTQLIRLCY
SSDHEKLKPQYLEELPGQLKQFSMFLGKFSWFAGEKLTFVDFLTYDILDQNRIFDPKCLD
EFPNLKAFMCRFEALEKIAAYLQSDQFCKMPINNKMAQWGNKPVC
>sp|P21281|VATB2_HUMAN V-type proton ATPase subunit B, brain isoform OS=Homo sapiens OX=9606 GN=ATP6V1B2 PE=1 SV=3
MALRAMRGIVNGAAPELPVPTGGPAVGAREQALAVSRNYLSQPRLTYKTVSGVNGPLVIL
DHVKFPRYAEIVHLTLPDGTKRSGQVLEVSGSKAVVQVFEGTSGIDAKKTSCEFTGDILR
TPVSEDMLGRVFNGSGKPIDRGPVVLAEDFLDIMGQPINPQCRIYPEEMIQTGISAIDGM
NSIARGQKIPIFSAAGLPHNEIAAQICRQAGLVKKSKDVVDYSEENFAIVFAAMGVNMET
ARFFKSDFEENGSMDNVCLFLNLANDPTIERIITPRLALTTAEFLAYQCEKHVLVILTDM
SSYAEALREVSAAREEVPGRRGFPGYMYTDLATIYERAGRVEGRNGSITQIPILTMPNDD
ITHPIPDLTGYITEGQIYVDRQLHNRQIYPPINVLPSLSRLMKSAIGEGMTRKDHADVSN
QLYACYAIGKDVQAMKAVVGEEALTSDDLLYLEFLQKFERNFIAQGPYENRTVFETLDIG
WQLLRIFPKEMLKRIPQSTLSEFYPRDSAKH
>sp|P21333|FLNA_HUMAN Filamin-A OS=Homo sapiens OX=9606 GN=FLNA PE=1 SV=4
MSSSHSRAGQSAAGAAPGGGVDTRDAEMPATEKDLAEDAPWKKIQQNTFTRWCNEHLKCV
SKRIANLQTDLSDGLRLIALLEVLSQKKMHRKHNQRPTFRQMQLENVSVALEFLDRESIK
LVSIDSKAIVDGNLKLILGLIWTLILHYSISMPMWDEEEDEEAKKQTPKQRLLGWIQNKL
PQLPITNFSRDWQSGRALGALVDSCAPGLCPDWDSWDASKPVTNAREAMQQADDWLGIPQ
VITPEEIVDPNVDEHSVMTYLSQFPKAKLKPGAPLRPKLNPKKARAYGPGIEPTGNMVKK
RAEFTVETRSAGQGEVLVYVEDPAGHQEEAKVTANNDKNRTFSVWYVPEVTGTHKVTVLF
AGQHIAKSPFEVYVDKSQGDASKVTAQGPGLEPSGNIANKTTYFEIFTAGAGTGEVEVVI
QDPMGQKGTVEPQLEARGDSTYRCSYQPTMEGVHTVHVTFAGVPIPRSPYTVTVGQACNP
SACRAVGRGLQPKGVRVKETADFKVYTKGAGSGELKVTVKGPKGEERVKQKDLGDGVYGF
EYYPMVPGTYIVTITWGGQNIGRSPFEVKVGTECGNQKVRAWGPGLEGGVVGKSADFVVE
AIGDDVGTLGFSVEGPSQAKIECDDKGDGSCDVRYWPQEAGEYAVHVLCNSEDIRLSPFM
ADIRDAPQDFHPDRVKARGPGLEKTGVAVNKPAEFTVDAKHGGKAPLRVQVQDNEGCPVE
ALVKDNGNGTYSCSYVPRKPVKHTAMVSWGGVSIPNSPFRVNVGAGSHPNKVKVYGPGVA
KTGLKAHEPTYFTVDCAEAGQGDVSIGIKCAPGVVGPAEADIDFDIIRNDNDTFTVKYTP
RGAGSYTIMVLFADQATPTSPIRVKVEPSHDASKVKAEGPGLSRTGVELGKPTHFTVNAK
AAGKGKLDVQFSGLTKGDAVRDVDIIDHHDNTYTVKYTPVQQGPVGVNVTYGGDPIPKSP
FSVAVSPSLDLSKIKVSGLGEKVDVGKDQEFTVKSKGAGGQGKVASKIVGPSGAAVPCKV
EPGLGADNSVVRFLPREEGPYEVEVTYDGVPVPGSPFPLEAVAPTKPSKVKAFGPGLQGG
SAGSPARFTIDTKGAGTGGLGLTVEGPCEAQLECLDNGDGTCSVSYVPTEPGDYNINILF
ADTHIPGSPFKAHVVPCFDASKVKCSGPGLERATAGEVGQFQVDCSSAGSAELTIEICSE
AGLPAEVYIQDHGDGTHTITYIPLCPGAYTVTIKYGGQPVPNFPSKLQVEPAVDTSGVQC
YGPGIEGQGVFREATTEFSVDARALTQTGGPHVKARVANPSGNLTETYVQDRGDGMYKVE
YTPYEEGLHSVDVTYDGSPVPSSPFQVPVTEGCDPSRVRVHGPGIQSGTTNKPNKFTVET
RGAGTGGLGLAVEGPSEAKMSCMDNKDGSCSVEYIPYEAGTYSLNVTYGGHQVPGSPFKV
PVHDVTDASKVKCSGPGLSPGMVRANLPQSFQVDTSKAGVAPLQVKVQGPKGLVEPVDVV

DNADGTQTVNYVPSREGPYSISVLYGDEEVPRSPFKVKVLPTHDASKVKASGPGLNTTGV
PASLPVEFTIDAKDAGEGLLAVQITDPEGKPKKTHIQDNHDGTYTVAYVPDVTGRYTILI
KYGGDEIPFSPYRVRAVPTGDASKCTVTVSIGGHGLGAGIGPTIQIGEETVITVDTKAAG
KGKVTCTVCTPDGSEVDVDVVENEDGTFDIFYTAPQPGKYVICVRFGGEHVPNSPFQVTA
LAGDQPSVQPPLRSQQLAPQYTYAQGGQQTWAPERPLVGVNGLDVTSLRPFDLVIPFTIK
KGEITGEVRMPSGKVAQPTITDNKDGTVTVRYAPSEAGLHEMDIRYDNMHIPGSPLQFYV
DYVNCGHVTAYGPGLTHGVVNKPATFTVNTKDAGEGGLSLAIEGPSKAEISCTDNQDGTC
SVSYLPVLPGDYSILVKYNEQHVPGSPFTARVTGDDSMRMSHLKVGSAADIPINISETDL
SLLTATVVPPSGREEPCLLKRLRNGHVGISFVPKETGEHLVHVKKNGQHVASSPIPVVIS
QSEIGDASRVRVSGQGLHEGHTFEPAEFIIDTRDAGYGGLSLSIEGPSKVDINTEDLEDG
TCRVTYCPTEPGNYIINIKFADQHVPGSPFSVKVTGEGRVKESITRRRRAPSVANVGSHC
DLSLKIPEISIQDMTAQVTSPSGKTHEAEIVEGENHTYCIRFVPAEMGTHTVSVKYKGQH
VPGSPFQFTVGPLGEGGAHKVRAGGPGLERAEAGVPAEFSIWTREAGAGGLAIAVEGPSK
AEISFEDRKDGSCGVAYVVQEPGDYEVSVKFNEEHIPDSPFVVPVASPSGDARRLTVSSL
QESGLKVNQPASFAVSLNGAKGAIDAKVHSPSGALEECYVTEIDQDKYAVRFIPRENGVY
LIDVKFNGTHIPGSPFKIRVGEPGHGGDPGLVSAYGAGLEGGVTGNPAEFVVNTSNAGAG
ALSVTIDGPSKVKMDCQECPEGYRVTYTPMAPGSYLISIKYGGPYHIGGSPFKAKVTGPR
LVSNHSLHETSSVFVDSLTKATCAPQHGAPGPGPADASKVVAKGLGLSKAYVGQKSSFTV
DCSKAGNNMLLVGVHGPRTPCEEILVKHVGSRLYSVSYLLKDKGEYTLVVKWGDEHIPGS
PYRVVVP
>sp|P21399|ACOHC_HUMAN Cytoplasmic aconitate hydratase OS=Homo sapiens OX=9606
GN=ACO1 PE=1 SV=3
MSNPFAHLAEPLDPVQPGKKFFNLNKLEDSRYGRLPFSIRVLLEAAIRNCDEFLVKKQDI
ENILHWNVTQHKNIEVPFKPARVILQDFTGVPAVVDFAAMRDAVKKLGGDPEKINPVCPA
DLVIDHSIQVDFNRRADSLQKNQDLEFERNRERFEFLKWGSQAFHNMRIIPPGSGIIHQV
NLEYLARVVFDQDGYYYPDSLVGTDSHTTMIDGLGILGWGVGGIEAEAVMLGQPISMVLP
QVIGYRLMGKPHPLVTSTDIVLTITKHLRQVGVVGKFVEFFGPGVAQLSIADRATIANMC
PEYGATAAFFPVDEVSITYLVQTGRDEEKLKYIKKYLQAVGMFRDFNDPSQDPDFTQVVE
LDLKTVVPCCSGPKRPQDKVAVSDMKKDFESCLGAKQGFKGFQVAPEHHNDHKTFIYDNT
EFTLAHGSVVIAAITSCTNTSNPSVMLGAGLLAKKAVDAGLNVMPYIKTSLSPGSGVVTY
YLQESGVMPYLSQLGFDVVGYGCMTCIGNSGPLPEPVVEAITQGDLVAVGVLSGNRNFEG
RVHPNTRANYLASPPLVIAYAIAGTIRIDFEKEPLGVNAKGQQVFLKDIWPTRDEIQAVE
RQYVIPGMFKEVYQKIETVNESWNALATPSDKLFFWNSKSTYIKSPPFFENLTLDLQPPK
SIVDAYVLLNLGDSVTTDHISPAGNIARNSPAARYLTNRGLTPREFNSYGSRRGNDAVMA
RGTFANIRLLNRFLNKQAPQTIHLPSGEILDVFDAAERYQQAGLPLIVLAGKEYGAGSSR
DWAAKGPFLLGIKAVLAESYERIHRSNLVGMGVIPLEYLPGENADALGLTGQERYTIIIP
ENLKPQMKVQVKLDTGKTFQAVMRFDTDVELTYFLNGGILNYMIRKMAK
>sp|P21695|GPDA_HUMAN Glycerol-3-phosphate dehydrogenase [NAD(+)], cytoplasmic
OS=Homo sapiens OX=9606 GN=GPD1 PE=1 SV=4
MASKKVCIVGSGNWGSAIAKIVGGNAAQLAQFDPRVTMWVFEEDIGGKKLTEIINTQHEN
VKYLPGHKLPPNVVAVPDVVQAAEDADILIFVVPHQFIGKICDQLKGHLKANATGISLIK
GVDEGPNGLKLISEVIGERLGIPMSVLMGANIASEVADEKFCETTIGCKDPAQGQLLKEL
MQTPNFRITVVQEVDTVEICGALKNVVAVGAGFCDGLGFGDNTKAAVIRLGLMEMIAFAK
LFCSGPVSSATFLESCGVADLITTCYGGRNRKVAEAFARTGKSIEQLEKELLNGQKLQGP

ETARELYSILQHKGLVDKFPLFMAVYKVCYEGQPVGEFIHCLQNHPEHM
>sp|P21796|VDAC1_HUMAN Voltage-dependent anion-selective channel protein 1 OS=Homo sapiens OX=9606 GN=VDAC1 PE=1 SV=2
MAVPPTYADLGKSARDVFTKGYGFGLIKLDLKTKSENGLEFTSSGSANTETTKVTGSLET
KYRWTEYGLTFTEKWNTDNTLGTEITVEDQLARGLKLTFDSSFSPNTGKKNAKIKTGYKR
EHINLGCDMDFDIAGPSIRGALVLGYEGWLAGYQMNFETAKSRVTQSNFAVGYKTDEFQL
HTNVNDGTEFGGSIYQKVNKKLETAVNLAWTAGNSNTRFGIAAKYQIDPDACFSAKVNNS
SLIGLGYTQTLKPGIKLTLSALLDGKNVNAGGHKLGLGLEFQA
>sp|P21926|CD9_HUMAN CD9 antigen OS=Homo sapiens OX=9606 GN=CD9 PE=1 SV=4
MPVKGGTKCIKYLLFGFNFIFWLAGIAVLAIGLWLRFDSQTKSIFEQETNNNNSSFYTGV
YILIGAGALMMLVGFLGCCGAVQESQCMLGLFFGFLLVIFAIEIAAAIWGYSHKDEVIKE
VQEFYKDTYNKLKTKDEPQRETLKAIHYALNCCGLAGGVEQFISDICPKKDVLETFTVKS
CPDAIKEVFDNKFHIIGAVGIGIAVVMIFGMIFSMILCCAIRRNREMV
>sp|P21964|COMT_HUMAN Catechol O-methyltransferase OS=Homo sapiens OX=9606 GN=COMT PE=1 SV=2
MPEAPPLLLAAVLLGLVLLVVLLLLLRHWGWGLCLIGWNEFILQPIHNLLMGDTKEQRIL
NHVLQHAEPGNAQSVLEAIDTYCEQKEWAMNVGDKKGKIVDAVIQEHQPSVLLELGAYCG
YSAVRMARLLSPGARLITIEINPDCAAITQRMVDFAGVKDKVTLVVGASQDIIPQLKKKY
DVDTLDMVFLDHWKDRYLPDTLLLEECGLLRKGTVLLADNVICPGAPDFLAHVRGSSCFE
CTHYQSFLEYREVVDGLEKAIYKGPGSEAGP
>sp|P21980|TGM2_HUMAN Protein-glutamine gamma-glutamyltransferase 2 OS=Homo sapiens OX=9606 GN=TGM2 PE=1 SV=2
MAEELVLERCDLELETNGRDHHTADLCREKLVVRRGQPFWLTLHFEGRNYEASVDSLTFS
VVTGPAPSQEAGTKARFPLRDAVEEGDWTATVVDQQDCTLSLQLTTPANAPIGLYRLSLE
ASTGYQGSSFVLGHFILLFNAWCPADAVYLDSEEERQEYVLTQQGFIYQGSAKFIKNIPW
NFGQFEDGILDICLILLDVNPKFLKNAGRDCSRRSSPVYVGRVVSGMVNCNDDQGVLLGR
WDNNYGDGVSPMSWIGSVDILRRWKNHGCQRVKYGQCWVFAAVACTVLRCLGIPTRVVTN
YNSAHDQNSNLLIEYFRNEFGEIQGDKSEMIWNFHCWVESWMTRPDLQPGYEGWQALDPT
PQEKSEGTYCCGPVPVRAIKEGDLSTKYDAPFVFAEVNADVVDWIQQDDGSVHKSINRSL
IVGLKISTKSVGRDEREDITHTYKYPEGSSEEREAFTRANHLNKLAEKEETGMAMRIRVG
QSMNMGSDFDVFAHITNNTAEEYVCRLLLCARTVSYNGILGPECGTKYLLNLNLEPFSEK
SVPLCILYEKYRDCLTESNLIKVRALLVEPVINSYLLAERDLYLENPEIKIRILGEPKQK
RKLVAEVSLQNPLPVALEGCTFTVEGAGLTEEQKTVEIPDPVEAGEEVKVRMDLLPLHMG
LHKLVVNFESDKLKAVKGFRNVIIGPA
>sp|P22059|OSBP1_HUMAN Oxysterol-binding protein 1 OS=Homo sapiens OX=9606 GN=OSBP PE=1 SV=1
MAATELRGVVGPGPAAIAALGGGGAGPPVVGGGGGRGDAGPGSGAASGTVVAAAAGGPGP
GAGGVAAAGPAPAPPTGGSGGSGAGGSGSAREGWLFKWTNYIKGYQRRWFVLSNGLLSYY
RSKAEMRHTCRGTINLATANITVEDSCNFIISNGGAQTYHLKASSEVERQRWVTALELAK
AKAVKMLAESDESGDEESVSQTDKTELQNTLRTLSSKVEDLSTCNDLIAKHGTALQRSLS
ELESLKLPAESNEKIKQVNERATLFRITSNAMINACRDFLMLAQTHSKKWQKSLQYERDQ
RIRLEETLEQLAKQHNHLERAFRGATVLPANTPGNVGSGKDQCCSGKGDMSDEDDENEFF
DAPEIITMPENLGHKRTGSNISGASSDISLDEQYKHQLEETKKEKRTRIPYKPNYSLNLW
SIMKNCIGKELSKIPMPVNFNEPLSMLQRLTEDLEYHELLDRAAKCENSLEQLCYVAAFT

VSSYSTTVFRTSKPFNPLLGETFELDRLEENGYRSLCEQVSHHPPAAAHHAESKNGWTLR
QEIKITSKFRGKYLSIMPLGTIHCIFHATGHHYTWKKVTTTVHNIIVGKLWIDQSGEIDI
VNHKTGDKCNLKFVPYSYFSRDVARKVTGEVTDPSGKVHFALLGTWDEKMECFKVQPVIG
ENGGDARQRGHEAEESRVMLWKRNPLPKNAENMYYFSELALTLNAWESGTAPTDSRLRPD
QRLMENGRWDEANAEKQRLEEKQRLSRKKREAEAMKATEDGTPYDPYKALWFERKKDPVT
KELTHIYRGEYWECKEKQDWSSCPDIF
>sp|P22061|PIMT_HUMAN Protein-L-isoaspartate(D-aspartate) O-methyltransferase OS=Homo sapiens OX=9606 GN=PCMT1 PE=1 SV=4
MAWKSGGASHSELIHNLRKNGIIKTDKVFEVMLATDRSHYAKCNPYMDSPQSIGFQATIS
APHMHAYALELLFDQLHEGAKALDVGSGSGILTACFARMVGCTGKVIGIDHIKELVDDSV
NNVRKDDPTLLSSGRVQLVVGDGRMGYAEEAPYDAIHVGAAAPVVPQALIDQLKPGGRLI
LPVGPAGGNQMLEQYDKLQDGSIKMKPLMGVIYVPLTDKEKQWSRWK
>sp|P22234|PUR6_HUMAN Bifunctional phosphoribosylaminoimidazole carboxylase/phosphoribosylaminoimidazole succinocarboxamide synthetase OS=Homo sapiens OX=9606 GN=PAICS PE=1 SV=3
MATAEVLNIGKKLYEGKTKEVYELLDSPGKVLLQSKDQITAGNAARKNHLEGKAAISNKI
TSCIFQLLQEAGIKTAFTRKCGETAFIAPQCEMIPIEWVCRRIATGSFLKRNPGVKEGYK
FYPPKVELFFKDDANNDPQWSEEQLIAAKFCFAGLLIGQTEVDIMSHATQAIFEILEKSW
LPQNCTLVDMKIEFGVDVTTKEIVLADVIDNDSWRLWPSGDRSQQKDKQSYRDLKEVTPE
GLQMVKKNFEWVAERVELLLKSESQCRVVVLMGSTSDLGHCEKIKKACGNFGIPCELRVT
SAHKGPDETLRIKAEYEGDGIPTVFVAVAGRSNGLGPVMSGNTAYPVISCPPLTPDWGVQ
DVWSSLRLPSGLGCSTVLSPEGSAQFAAQIFGLSNHLVWSKLRASILNTWISLKQADKKI
RECNL
>sp|P22314|UBA1_HUMAN Ubiquitin-like modifier-activating enzyme 1 OS=Homo sapiens OX=9606 GN=UBA1 PE=1 SV=3
MSSSPLSKKRRVSGPDPKPGSNCSPAQSVLSEVPSVPTNGMAKNGSEADIDEGLYSRQLY
VLGHEAMKRLQTSSVLVSGLRGLGVEIAKNIILGGVKAVTLHDQGTAQWADLSSQFYLRE
EDIGKNRAEVSQPRLAELNSYVPVTAYTGPLVEDFLSGFQVVVLTNTPLEDQLRVGEFCH
NRGIKLVVADTRGLFGQLFCDFGEEMILTDSNGEQPLSAMVSMVTKDNPGVVTCLDEARH
GFESGDFVSFSEVQGMVELNGNQPMEIKVLGPYTFSICDTSNFSDYIRGGIVSQVKVPKK
ISFKSLVASLAEPDFVVTDFAKFSRPAQLHIGFQALHQFCAQHGRPPRPRNEEDAAELVA
LAQAVNARALPAVQQNNLDEDLIRKLAYVAAGDLAPINAFIGGLAAQEVMKACSGKFMPI
MQWLYFDALECLPEDKEVLTEDKCLQRQNRYDGQVAVFGSDLQEKLGKQKYFLVGAGAIG
CELLKNFAMIGLGCGEGGEIIVTDMDTIEKSNLNRQFLFRPWDVTKLKSDTAAAAVRQMN
PHIRVTSHQNRVGPDTERIYDDDFFQNLDGVANALDNVDARMYMDRRCVYYRKPLLESGT
LGTKGNVQVVIPFLTESYSSSQDPPEKSIPICTLKNFPNAIEHTLQWARDEFEGLFKQPA
ENVNQYLTDPKFVERTLRLAGTQPLEVLEAVQRSLVLQRPQTWADCVTWACHHWHTQYSN
NIRQLLHNFPPDQLTSSGAPFWSGPKRCPHPLTFDVNNPLHLDYVMAAANLFAQTYGLTG
SQDRAAVATFLQSVQVPEFTPKSGVKIHVSDQELQSANASVDDSRLEELKATLPSPDKLP
GFKMYPIDFEKDDDSNFHMDFIVAASNLRAENYDIPSADRHKSKLIAGKIIPAIATTTAA
VVGLVCLELYKVVQGHRQLDSYKNGFLNLALPFFGFSEPLAAPRHQYYNQEWTLWDRFEV
QGLQPNGEEMTLKQFLDYFKTEHKLEITMLSQGVSMLYSFFMPAAKLKERLDQPMTEIVS
RVSKRKLGRHVRALVLELCCNDESGEDVEVPYVRYTIR

>sp|P22694|KAPCB_HUMAN cAMP-dependent protein kinase catalytic subunit beta OS=Homo sapiens OX=9606 GN=PRKACB PE=1 SV=2
MGNAATAKKGSEVESVKEFLAKAKEDFLKKWENPTQNNAGLEDFERKKTLGTGSFGRVML
VKHKATEQYYAMKILDKQKVVKLKQIEHTLNEKRILQAVNFPFLVRLEYAFKDNSNLYMV
MEYVPGGEMFSHLRRIGRFSEPHARFYAAQIVLTFEYLHSLDLIYRDLKPENLLIDHQGY
IQVTDFGFAKRVKGRTWTLCGTPEYLAPEIILSKGYNKAVDWWALGVLIYEMAAGYPPFF
ADQPIQIYEKIVSGKVRFPSHFSSDLKDLLRNLLQVDLTKRFGNLKNGVSDIKTHKWFAT
TDWIAIYQRKVEAPFIPKFRGSGDTSNFDDYEEEDIRVSITEKCAKEFGEF
>sp|P22748|CAH4_HUMAN Carbonic anhydrase 4 OS=Homo sapiens OX=9606 GN=CA4 PE=1 SV=2
MRMLLALLALSAARPSASAESHWCYEVQAESSNYPCLVPVKWGGNCQKDRQSPINIVTTK
AKVDKKLGRFFFSGYDKKQTWTVQNNGHSVMMLLENKASISGGGLPAPYQAKQLHLHWSD
LPYKGSEHSLDGEHFAMEMHIVHEKEKGTSRNVKEAQDPEDEIAVLAFLVEAGTQVNEGF
QPLVEALSNIPKPEMSTTMAESSLLDLLPKEEKLRHYFRYLGSLTTPTCDEKVVWTVFRE
PIQLHREQILAFSQKLYYDKEQTVSMKDNVRPLQQLGQRTVIKSGAPGRPLPWALPALLG
PMLACLLAGFLR
>sp|P22914|CRYGS_HUMAN Gamma-crystallin S OS=Homo sapiens OX=9606 GN=CRYGS PE=1 SV=4
MSKTGTKITFYEDKNFQGRRYDCDCDCADFHTYLSRCNSIKVEGGTWAVYERPNFAGYMY
ILPQGEYPEYQRWMGLNDRLSSCRAVHLPSGGQYKIQIFEKGDFSGQMYETTEDCPSIME
QFHMREIHSCKVLEGVWIFYELPNYRGRQYLLDKKEYRKPIDWGAASPAVQSFRRIVE
>sp|P23141|EST1_HUMAN Liver carboxylesterase 1 OS=Homo sapiens OX=9606 GN=CES1 PE=1 SV=2
MWLRAFILATLSASAAWGHPSSPPVVDTVHGKVLGKFVSLEGFAQPVAIFLGIPFAKPPL
GPLRFTPPQPAEPWSFVKNATSYPPMCTQDPKAGQLLSELFTNRKENIPLKLSEDCLYLN
IYTPADLTKKNRLPVMVWIHGGGLMVGAASTYDGLALAAHENVVVVTIQYRLGIWGFFST
GDEHSRGNWGHLDQVAALRWVQDNIASFGGNPGSVTIFGESAGGESVSVLVLSPLAKNLF
HRAISESGVALTSVLVKKGDVKPLAEQIAITAGCKTTTSAVMVHCLRQKTEEELLETTLK
MKFLSLDLQGDPRESQPLLGTVIDGMLLLKTPEELQAERNFHTVPYMVGINKQEFGWLIP
MQLMSYPLSEGQLDQKTAMSLLWKSYPLVCIAKELIPEATEKYLGGTDDTVKKKDLFLDL
IADVMFGVPSVIVARNHRDAGAPTYMYEFQYRPSFSSDMKPKTVIGDHGDELFSVFGAPF
LKEGASEEEIRLSKMVMKFWANFARNGNPNGEGLPHWPEYNQKEGYLQIGANTQAAQKLK
DKEVAFWTNLFAKKAVEKPPQTEHIEL
>sp|P23284|PPIB_HUMAN Peptidyl-prolyl cis-trans isomerase B OS=Homo sapiens OX=9606 GN=PPIB PE=1 SV=2
MLRLSERNMKVLLAAALIAGSVFFLLLPGPSAADEKKKGPKVTVKVYFDLRIGDEDVGRV
IFGLFGKTVPKTVDNFVALATGEKGFGYKNSKFHRVIKDFMIQGGDFTRGDGTGGKSIYG
ERFPDENFKLKHYGPGWVSMANAGKDTNGSQFFITTVKTAWLDGKHVVFGKVLEGMEVVR
KVESTKTDSRDKPLKDVIIADCGKIEVEKPFAIAKE
>sp|P23381|SYWC_HUMAN Tryptophan--tRNA ligase, cytoplasmic OS=Homo sapiens OX=9606 GN=WARS1 PE=1 SV=2
MPNSEPASLLELFNSIATQGELVRSLKAGNASKDEIDSAVKMLVSLKMSYKAAAGEDYKA
DCPPGNPAPTSNHGPDATEAEEDFVDPWTVQTSSAKGIDYDKLIVRFGSSKIDKELINRI
ERATGQRPHHFLRRGIFFSHRDMNQVLDAYENKKPFYLYTGRGPSSEAMHVGHLIPFIFT

KWLQDVFNVPLVIQMTDDEKYLWKDLTLDQAYSYAVENAKDIIACGFDINKTFIFSDLDY
MGMSSGFYKNVVKIQKHVTFNQVKGIFGFTDSDCIGKISFPAIQAAPSFSNSFPQIFRDR
TDIQCLIPCAIDQDPYFRMTRDVAPRIGYPKPALLHSTFFPALQGAQTKMSASDPNSSIF
LTDTAKQIKTKVNKHAFSGGRDTIEEHRQFGGNCDVDVSFMYLTFFLEDDDKLEQIRKDY
TSGAMLTGELKKALIEVLQPLIAEHQARRKEVTDEIVKEFMTPRKLSFDFQ
>sp|P23526|SAHH_HUMAN Adenosylhomocysteinase OS=Homo sapiens OX=9606 GN=AHCY PE=1 SV=4
MSDKLPYKVADIGLAAWGRKALDIAENEMPGLMRMRERYSASKPLKGARIAGCLHMTVET
AVLIETLVTLGAEVQWSSCNIFSTQDHAAAAIAKAGIPVYAWKGETDEEYLWCIEQTLYF
KDGPLNMILDDGGDLTNLIHTKYPQLLPGIRGISEETTTGVHNLYKMMANGILKVPAINV
NDSVTKSKFDNLYGCRESLIDGIKRATDVMIAGKVAVVAGYGDVGKGCAQALRGFGARVI
ITEIDPINALQAAMEGYEVTTMDEACQEGNIFVTTTGCIDIILGRHFEQMKDDAIVCNIG
HFDVEIDVKWLNENAVEKVNIKPQVDRYRLKNGRRIILLAEGRLVNLGCAMGHPSFVMSN
SFTNQVMAQIELWTHPDKYPVGVHFLPKKLDEAVAEAHLGKLNVKLTKLTEKQAQYLGMS
CDGPFKPDHYRY
>sp|P23528|COF1_HUMAN Cofilin-1 OS=Homo sapiens OX=9606 GN=CFL1 PE=1 SV=3
MASGVAVSDGVIKVFNDMKVRKSSTPEEVKKRKKAVLFCLSEDKKNIILEEGKEILVGDV
GQTVDDPYATFVKMLPDKDCRYALYDATYETKESKKEDLVFIFWAPESAPLKSKMIYASS
KDAIKKKLTGIKHELQANCYEEVKDRCTLAEKLGGSAVISLEGKPL
>sp|P24539|AT5F1_HUMAN ATP synthase F(0) complex subunit B1, mitochondrial OS=Homo sapiens OX=9606 GN=ATP5PB PE=1 SV=2
MLSRVVLSAAATAAPSLKNAAFLGPGVLQATRTFHTGQPHLVPVPPLPEYGGKVRYGLIP
EEFFQFLYPKTGVTGPYVLGTGLILYALSKEIYVISAETFTALSVLGVMVYGIKKYGPFV
ADFADKLNEQKLAQLEEAKQASIQHIQNAIDTEKSQQALVQKRHYLFDVQRNNIAMALEV
TYRERLYRVYKEVKNRLDYHISVQNMMRRKEQEHMINWVEKHVVQSISTQQEKETIAKCI
ADLKLLAKKAQAQPVM
>sp|P25391|LAMA1_HUMAN Laminin subunit alpha-1 OS=Homo sapiens OX=9606 GN=LAMA1 PE=1 SV=2
MRGGVLLVLLLCVAAQCRQRGLFPAILNLASNAHISTNATCGEKGPEMFCKLVEHVPGRP
VRNPQCRICDGNSANPRERHPISHAIDGTNNWWQSPSIQNGREYHWVTITLDLRQVFQVA
YVIIKAANAPRPGNWILERSLDGTTFSPWQYYAVSDSECLSRYNITPRRGPPTYRADDEV
ICTSYYSRLVPLEHGEIHTSLINGRPSADDLSPKLLEFTSARYIRLRLQRIRTLNADLMT
LSHREPKELDPIVTRRYYYSIKDISVGGMCICYGHASSCPWDETTKKLQCQCEHNTCGES
CNRCCPGYHQQPWRPGTVSSGNTCEACNCHNKAKDCYYDESVAKQKKSLNTAGQFRGGGV
CINCLQNTMGINCETCIDGYYRPHKVSPYEDEPCRPCNCDPVGSLSSVCIKDDLHSDLHN
GKQPGQCPCKEGYTGEKCDRCQLGYKDYPTCVSCGCNPVGSASDEPCTGPCVCKENVEGK
ACDRCKPGFYNLKEKNPRGCSECFCFGVSDVCSSLSWPVGQVNSMSGWLVTDLISPRKIP
SQQDALGGRHQVSINNTAVMQRLAPKYYWAAPEAYLGNKLTAFGGFLKYTVSYDIPVETV
DSNLMSHADVIIKGNGLTLSTQAEGLSLQPYEEYLNVVRLVPENFQDFHSKRQIDRDQLM
TVLANVTHLLIRANYNSAKMALYRLESVSLDIASSNAIDLVVAADVEHCECPQGYTGTSC
ESCLSGYYRVDGILFGGICQPCECHGHAAECNVHGVCIACAHNTTGVHCEQCLPGFYGEP
SRGTPGDCQPCACPLTIASNNFSPTCHLNDGDEVVCDWCAPGYSGAWCERCADGYYGNPT
VPGESCVPCDCSGNVDPSEAGHCDSVTGECLKCLGNTDGAHCERCADGFYGDAVTAKNCR
ACECHVKGSHSAVCHLETGLCDCKPNVTGQQCDQCLHGYYGLDSGHGCRPCNCSVAGSVS

DGCTDEGQCHCVPGVAGKRCDRCAHGFYAYQDGSCTPCDCPHTQNTCDPETGECVCPPHT
QGVKCEECEDGHWGYDAEVGCQACNCSLVGSTHHRCDVVTGHCQCKSKFGGRACDQCSLG
YRDFPDCVPCDCDLRGTSGDACNLEQGLCGCVEETGACPCKENVFGPQCNECREGTFALR
ADNPLGCSPCFCSGLSHLCSELEDYVRTPVTLGSDQPLLRVVSQSNLRGTTEGVYYQAPD
FLLDAATVRQHIRAEPFYWRLPQQFQGDQLMAYGGKLKYSVAFYSLDGVGTSNFEPQVLI
KGGRIRKQVIYMDAPAPENGVRQEQEVAMRENFWKYFNSVSEKPVTREDFMSVLSDIEYI
LIKASYGQGLQQSRISDISMEVGRKAEKLHPEEEVASLLENCVCPPGTVGFSCQDCAPGY
HRGKLPAGSDRGPRPLVAPCVPCSCNNHSDTCDPNTGKCLNCGDNTAGDHCDVCTSGYYG
KVTGSASDCALCACPHSPPASFSPTCVLEGDHDFRCDACLLGYEGKHCERCSSSYYGNPQ
TPGGSCQKCDCNPHGSVHGDCDRTSGQCVCRLGASGLRCDECEPRHILMETDCVSCDDEC
VGVLLNDLDEIGDAVLSLNLTGIIPVPYGILSNLENTTKYLQESLLKENMQKDLGKIKLE
GVAEETDNLQKKLTRMLASTQKVNRATERIFKESQDLAIAIERLQMSITEIMEKTTLNQT
LDEDFLLPNSTLQNMQQNGTSLLEIMQIRDFTQLHQNATLELKAAEDLLSQIQENYQKPL
EELEVLKEAASHVLSKHNNELKAAEALVREAEAKMQESNHLLLMVNANLREFSDKKLHVQ
EEQNLTSELIVQGRGLIDAAAAQTDAVQDALEHLEDHQDKLLLWSAKIRHHIDDLVMHMS
QRNAVDLVYRAEDHAAEFQRLADVLYSGLENIRNVSLNATSAAYVHYNIQSLIEESEELA
RDAHRTVTETSLLSESLVSNGKAAVQRSSRFLKEGNNLSRKLPGIALELSELRNKTNRFQ
ENAVEITRQTNESLLILRAIPKGIRDKGAKTKELATSASQSAVSTLRDVAGLSQELLNTS
ASLSRVNTTLRETHQLLQDSTMATLLAGRKVKDVEIQANLLFDRLKPLKMLEENLSRNLS
EIKLLISQARKQAASIKVAVSADRDCIRAYQPQISSTNYNTLTLNVKTQEPDNLLFYLGS
STASDFLAVEMRRGRVAFLWDLGSGSTRLEFPDFPIDDNRWHSIHVARFGNIGSLSVKEM
SSNQKSPTKTSKSPGTANVLDVNNSTLMFVGGLGGQIKKSPAVKVTHFKGCLGEAFLNGK
SIGLWNYIEREGKCRGCFGSSQNEDPSFHFDGSGYSVVEKSLPATVTQIIMLFNTFSPNG
LLLYLGSYGTKDFLSIELFRGRVKVMTDLGSGPITLLTDRRYNNGTWYKIAFQRNRKQGV
LAVIDAYNTSNKETKQGETPGASSDLNRLDKDPIYVGGLPRSRVVRRGVTTKSFVGCIKN
LEISRSTFDLLRNSYGVRKGCLLEPIRSVSFLKGGYIELPPKSLSPESEWLVTFATTNSS
GIILAALGGDVEKRGDREEAHVPFFSVMLIGGNIEVHVNPGDGTGLRKALLHAPTGTCSD
GQAHSISLVRNRRIITVQLDENNPVEMKLGTLVESRTINVSNLYVGGIPEGEGTSLLTMR
RSFHGCIKNLIFNLELLDFNSAVGHEQVDLDTCWLSERPKLAPDAEDSKLLPEPRAFPEQ
CVVDAALEYVPGAHQFGLTQNSHFILPFNQSAVRKKLSVELSIRTFASSGLIYYMAHQNQ
ADYAVLQLHGGRLHFMFDLGKGRTKVSHPALLSDGKWHTVKTDYVKRKGFITVDGRESPM
VTVVGDGTMLDVEGLFYLGGLPSQYQARKIGNITHSIPACIGDVTVNSKQLDKDSPVSAF
TVNRCYAVAQEGTYFDGSGYAALVKEGYKVQSDVNITLEFRTSSQNGVLLGISTAKVDAI
GLELVDGKVLFHVNNGAGRITAAYEPKTATVLCDGKWHTLQANKSKHRITLIVDGNAVGA
ESPHTQSTSVDTNNPIYVGGYPAGVKQKCLRSQTSFRGCLRKLALIKSPQVQSFDFSRAF
ELHGVFLHSCPGTES
>sp|P25787|PSA2_HUMAN Proteasome subunit alpha type-2 OS=Homo sapiens OX=9606
GN=PSMA2 PE=1 SV=2
MAERGYSFSLTTFSPSGKLVQIEYALAAVAGGAPSVGIKAANGVVLATEKKQKSILYDER
SVHKVEPITKHIGLVYSGMGPDYRVLVHRARKLAQQYYLVYQEPIPTAQLVQRVASVMQE
YTQSGGVRPFGVSLLICGWNEGRPYLFQSDPSGAYFAWKATAMGKNYVNGKTFLEKRYNE
DLELEDAIHTAILTLKESFEGQMTEDNIEVGICNEAGFRRLTPTEVKDYLAAIA
>sp|P25788|PSA3_HUMAN Proteasome subunit alpha type-3 OS=Homo sapiens OX=9606
GN=PSMA3 PE=1 SV=2

MSSIGTGYDLSASTFSPDGRVFQVEYAMKAVENSSTAIGIRCKDGVVFGVEKLVLSKLYE
EGSNKRLFNVDRHVGMAVAGLLADARSLADIAREEASNFRSNFGYNIPLKHLADRVAMYV
HAYTLYSAVRPFGCSFMLGSYSVNDGAQLYMIDPSGVSYGYWGCAIGKARQAAKTEIEKL
QMKEMTCRDIVKEVAKIIYIVHDEVKDKAFELELSWVGELTNGRHEIVPKDIREEAEKYA
KESLKEEDESDDDNM
>sp|P25789|PSA4_HUMAN Proteasome subunit alpha type-4 OS=Homo sapiens OX=9606 GN=PSMA4 PE=1 SV=1
MSRRYDSRTTIFSPEGRLYQVEYAMEAIGHAGTCLGILANDGVLLAAERRNIHKLLDEVF
FSEKIYKLNEDMACSVAGITSDANVLTNELRLIAQRYLLQYQEPIPCEQLVTALCDIKQA
YTQFGGKRPFGVSLLYIGWDKHYGFQLYQSDPSGNYGGWKATCIGNNSAAAVSMLKQDYK
EGEMTLKSALALAIKVLNKTMDVSKLSAEKVEIATLTRENGKTVIRVLKQKEVEQLIKKH
EEEEAKAEREKKEKEQKEKDK
>sp|P26038|MOES_HUMAN Moesin OS=Homo sapiens OX=9606 GN=MSN PE=1 SV=3
MPKTISVRVTTMDAELEFAIQPNTTGKQLFDQVVKTIGLREVWFFGLQYQDTKGFSTWLK
LNKKVTAQDVRKESPLLFKFRAKFYPEDVSEELIQDITQRLFFLQVKEGILNDDIYCPPE
TAVLLASYAVQSKYGDFNKEVHKSGYLAGDKLLPQRVLEQHKLNKDQWEERIQVWHEEHR
GMLREDAVLEYLKIAQDLEMYGVNYFSIKNKKGSELWLGVDALGLNIYEQNDRLTPKIGF
PWSEIRNISFNDKKFVIKPIDKKAPDFVFYAPRLRINKRILALCMGNHELYMRRRKPDTI
EVQQMKAQAREEKHQKQMERAMLENEKKKREMAEKEKEKIEREKEELMERLKQIEEQTKK
AQQELEEQTRRALELEQERKRAQSEAEKLAKERQEAEEAKEALLQASRDQKKTQEQLALE
MAELTARISQLEMARQKKESEAVEWQQKAQMVQEDLEKTRAELKTAMSTPHVAEPAENEQ
DEQDENGAEASADLRADAMAKDRSEEERTTEAEKNERVQKHLKALTSELANARDESKKTA
NDMIHAENMRLGRDKYKTLRQIRQGNTKQRIDEFESM
>sp|P26232|CTNA2_HUMAN Catenin alpha-2 OS=Homo sapiens OX=9606 GN=CTNNA2 PE=1 SV=5
MTSATSPIILKWDPKSLEIRTLTVERLLEPLVTQVTTLVNTSNKGPSGKKKGRSKKAHVL
AASVEQATQNFLEKGEQIAKESQDLKEELVAAVEDVRKQGETMRIASSEFADDPCSSVKR
GTMVRAARALLSAVTRLLILADMADVMRLLSHLKIVEEALEAVKNATNEQDLANRFKEFG
KEMVKLNYVAARRQQELKDPHCRDEMAAARGALKKNATMLYTASQAFLRHPDVAATRANR
DYVFKQVQEAIAGISNAAQATSPTDEAKGHTGIGELAAALNEFDNKIILDPMTFSEARFR
PSLEERLESIISGAALMADSSCTRDDRRERIVAECNAVRQALQDLLSEYMNNTGRKEKGD
PLNIAIDKMTKKTRDLRRQLRKAVMDHISDSFLETNVPLLVLIEAAKSGNEKEVKEYAQV
FREHANKLVEVANLACSISNNEEGVKLVRMAATQIDSLCPQVINAALTLAARPQSKVAQD
NMDVFKDQWEKQVRVLTEAVDDITSVDDFLSVSENHILEDVNKCVIALQEGDVDTLDRTA
GAIRGRAARVIHIINAEMENYEAGVYTEKVLEATKLLSETVMPRFAEQVEVAIEALSANV
PQPFEENEFIDASRLVYDGVRDIRKAVLMIRTPEELEDDSDFEQEDYDVRSRTSVQTEDD
QLIAGQSARAIMAQLPQEEKAKIAEQVEIFHQEKSKLDAEVAKWDDSGNDIIVLAKQMCM
IMMEMTDFTRGKGPLKNTSDVINAAKKIAEAGSRMDKLARAVADQCPDSACKQDLLAYLQ
RIALYCHQLNICSKVKAEVQNLGGELIVSGTGVQSTFTTFYEVDCDVIDGGRASQLSTHL
PTCAEGAPIGSGSSDSSMLDSATSLIQAAKNLMNAVVLTVKASYVASTKYQKVYGTAAVN
SPVVSWKMKAPEKKPLVKREKPEEFQTRVRRGSQKKHISPVQALSEFKAMDSF
>sp|P26447|S10A4_HUMAN Protein S100-A4 OS=Homo sapiens OX=9606 GN=S100A4 PE=1 SV=1
MACPLEKALDVMVSTFHKYSGKEGDKFKLNKSELKELLTRELPSFLGKRTDEAAFQKLMS

NLDSNRDNEVDFQEYCVFLSCIAMMCNEFFEGFPDKQPRKK
>sp|P26640|SYVC_HUMAN Valine--tRNA ligase OS=Homo sapiens OX=9606 GN=VARS1 PE=1 SV=4
MSTLYVSPHPDAFPSLRALIAARYGEAGEGPGWGGAHPRICLQPPPTSRTPFPPPRLPAL
EQGPGGLWVWGATAVAQLLWPAGLGGPGGSRAAVLVQQWVSYADTELIPAACGATLPALG
LRSSAQDPQAVLGALGRALSPLEEWLRLHTYLAGEAPTLADLAAVTALLLPFRYVLDPPA
RRIWNNVTRWFVTCVRQPEFRAVLGEVVLYSGARPLSHQPGPEAPALPKTAAQLKKEAKK
REKLEKFQQKQKIQQQQPPPGEKKPKPEKREKRDPGVITYDLPTPPGEKKDVSGPMPDSY
SPRYVEAAWYPWWEQQGFFKPEYGRPNVSAANPRGVFMMCIPPPNVTGSLHLGHALTNAI
QDSLTRWHRMRGETTLWNPGCDHAGIATQVVVEKKLWREQGLSRHQLGREAFLQEVWKWK
EEKGDRIYHQLKKLGSSLDWDRACFTMDPKLSAAVTEAFVRLHEEGIIYRSTRLVNWSCT
LNSAISDIEVDKKELTGRTLLSVPGYKEKVEFGVLVSFAYKVQGSDSDEEVVVATTRIET
MLGDVAVAVHPKDTRYQHLKGKNVIHPFLSRSLPIVFDEFVDMDFGTGAVKITPAHDQND
YEVGQRHGLEAISIMDSRGALINVPPPFLGLPRFEARKAVLVALKERGLFRGIEDNPMVV
PLCNRSKDVVEPLLRPQWYVRCGEMAQAASAAVTRGDLRILPEAHQRTWHAWMDNIREWC
ISRQLWWGHRIPAYFVTVSDPAVPPGEDPDGRYWVSGRNEAEAREKAAKEFGVSPDKISL
QQDEDVLDTWFSSGLFPLSILGWPNQSEDLSVFYPGTLLETGHDILFFWVARMVMLGLKL
TGRLPFREVYLHAIVRDAHGRKMSKSLGNVIDPLDVIYGISLQGLHNQLLNSNLDPSEVE
KAKEGQKADFPAGIPECGTDALRFGLCAYMSQGRDINLDVNRILGYRHFCNKLWNATKFA
LRGLGKGFVPSPTSQPGGHESLVDRWIRSRLTEAVRLSNQGFQAYDFPAVTTAQYSFWLY
ELCDVYLECLKPVLNGVDQVAAECARQTLYTCLDVGLRLLSPFMPFVTEELFQRLPRRMP
QAPPSLCVTPYPEPSECSWKDPEAEAALELALSITRAVRSLRADYNLTRIRPDCFLEVAD
EATGALASAVSGYVQALASAGVVAVLALGAPAPQGCAVALASDRCSIHLQLQGLVDPARE
LGKLQAKRVEAQRQAQRLRERRAASGYPVKVPLEVQEADEAKLQQTEAELRKVDEAIALF
QKML
>sp|P26998|CRBB3_HUMAN Beta-crystallin B3 OS=Homo sapiens OX=9606 GN=CRYBB3 PE=1 SV=4
MAEQHGAPEQAAAGKSHGDLGGSYKVILYELENFQGKRCELSAECPSLTDSLLEKVGSIQ
VESGPWLAFESRAFRGEQFVLEKGDYPRWDAWSNSRDSDSLLSLRPLNIDSPHHKLHLFE
NPAFSGRKMEIVDDDVPSLWAHGFQDRVASVRAINGTWVGYEFPGYRGRQYVFERGEYRH
WNEWDASQPQLQSVRRIRDQKWHKRGRFPSS
>sp|P27348|1433T_HUMAN 14-3-3 protein theta OS=Homo sapiens OX=9606 GN=YWHAQ PE=1 SV=1
MEKTELIQKAKLAEQAERYDDMATCMKAVTEQGAELSNEERNLLSVAYKNVVGGRRSAWR
VISSIEQKTDTSDKKLQLIKDYREKVESELRSICTTVLELLDKYLIANATNPESKVFYLK
MKGDYFRYLAEVACGDDRKQTIDNSQGAYQEAFDISKKEMQPTHPIRLGLALNFSVFYYE
ILNNPELACTLAKTAFDEAIAELDTLNEDSYKDSTLIMQLLRDNLTLWTSDSAGEECDAA
EGAEN
>sp|P27361|MK03_HUMAN Mitogen-activated protein kinase 3 OS=Homo sapiens OX=9606 GN=MAPK3 PE=1 SV=4
MAAAAAQGGGGGEPRRTEGVGPGVPGEVEMVKGQPFDVGPRYTQLQYIGEGAYGMVSSAY
DHVRKTRVAIKKISPFEHQTYCQRTLREIQILLRFRHENVIGIRDILRASTLEAMRDVYI
VQDLMETDLYKLLKSQQLSNDHICYFLYQILRGLKYIHSANVLHRDLKPSNLLINTTCDL
KICDFGLARIADPEHDHTGFLTEYVATRWYRAPEIMLNSKGYTKSIDIWSVGCILAEMLS

NRPIFPGKHYLDQLNHILGILGSPSQEDLNCIINMKARNYLQSLPSKTKVAWAKLFPKSD
SKALDLLDRMLTFNPNKRITVEEALAHPYLEQYYDPTDEPVAEEPFTFAMELDDLPKERL
KELIFQETARFQPGVLEAP
>sp|P27635|RL10_HUMAN Large ribosomal subunit protein uL16 OS=Homo sapiens OX=9606 GN=RPL10 PE=1 SV=5
MGRRPARCYRYCKNKPYPKSRFCRGVPDAKIRIFDLGRKKAKVDEFPLCGHMVSDEYEQL
SSEALEAARICANKYMVKSCGKDGFHIRVRLHPFHVIRINKMLSCAGADRLQTGMRGAFG
KPQGTVARVHIGQVIMSIRTKLQNKEHVIEALRRAKFKFPGRQKIHISKKWGFTKFNADE
FEDMVAEKRLIPDGCGVKYIPSRGPLDKWRALHS
>sp|P27816|MAP4_HUMAN Microtubule-associated protein 4 OS=Homo sapiens OX=9606 GN=MAP4 PE=1 SV=3
MADLSLADALTEPSPDIEGEIKRDFIATLEAEAFDDVVGETVGKTDYIPLLDVDEKTGNS
ESKKKPCSETSQIEDTPSSKPTLLANGGHGVEGSDTTGSPTEFLEEKMAYQEYPNSQNWP
EDTNFCFQPEQVVDPIQTDPFKMYHDDDLADLVFPSSATADTSIFAGQNDPLKDSYGMSP
CNTAVVPQGWSVEALNSPHSESFVSPEAVAEPPQPTAVPLELAKEIEMASEERPPAQALE
IMMGLKTTDMAPSKETEMALAKDMALATKTEVALAKDMESPTKLDVTLAKDMQPSMESDM
ALVKDMELPTEKEVALVKDVRWPTETDVSSAKNVVLPTETEVAPAKDVTLLKETERASPI
KMDLAPSKDMGPPKENKKETERASPIKMDLAPSKDMGPPKENKIVPAKDLVLLSEIEVAQ
ANDIISSTEISSAEKVALSSETEVALARDMTLPPETNVILTKDKALPLEAEVAPVKDMAQ
LPETEIAPAKDVAPSTVKEVGLLKDMSPLSETEMALGKDVTPPPETEVVLIKNVCLPPEM
EVALTEDQVPALKTEAPLAKDGVLTLANNVTPAKDVPPLSETEATPVPIKDMEIAQTQKG
ISEDSHLESLQDVGQSAAPTFMISPETVTGTGKKCSLPAEEDSVLEKLGERKPCNSQPSE
LSSETSGIARPEEGRPVVSGTGNDITTPPNKELPPSPEKKTKPLATTQPAKTSTSKAKTQ
PTSLPKQPAPTTIGGLNKKPMSLASGLVPAAPPKRPAVASARPSILPSKDVKPKPIADAK
APEKRASPSKPASAPASRSGSKSTQTVAKTTTAAAVASTGPSSRSPSTLLPKKPTAIKTE
GKPAEVKKMTAKSVPADLSRPKSTSTSSMKKTTTLSGTAPAAGVVPSRVKATPMPSRPST
TPFIDKKPTSAKPSSTTPRLSRLATNTSAPDLKNVRSKVGSTENIKHQPGGGRAKVEKKT
EAAATTRKPESNAVTKTAGPIASAQKQPAGKVQIVSKKVSYSHIQSKCGSKDNIKHVPGG
GNVQIQNKKVDISKVSSKCGSKANIKHKPGGGDVKIESQKLNFKEKAQAKVGSLDNVGHL
PAGGAVKTEGGGSEAPLCPGPPAGEEPAISEAAPEAGAPTSASGLNGHPTLSGGGDQREA
QTLDSQIQETSI
>sp|P27824|CALX_HUMAN Calnexin OS=Homo sapiens OX=9606 GN=CANX PE=1 SV=2
MEGKWLLCMLLVLGTAIVEAHDGHDDDVIDIEDDLDDVIEEVEDSKPDTTAPPSSPKVTY
KAPVPTGEVYFADSFDRGTLSGWILSKAKKDDTDDEIAKYDGKWEVEEMKESKLPGDKGL
VLMSRAKHHAISAKLNKPFLFDTKPLIVQYEVNFQNGIECGGAYVKLLSKTPELNLDQFH
DKTPYTIMFGPDKCGEDYKLHFIFRHKNPKTGIYEEKHAKRPDADLKTYFTDKKTHLYTL
ILNPDNSFEILVDQSVVNSGNLLNDMTPPVNPSREIEDPEDRKPEDWDERPKIPDPEAVK
PDDWDEDAPAKIPDEEATKPEGWLDDEPEYVPDPDAEKPEDWDEDMDGEWEAPQIANPRC
ESAPGCGVWQRPVIDNPNYKGKWKPPMIDNPSYQGIWKPRKIPNPDFFEDLEPFRMTPFS
AIGLELWSMTSDIFFDNFIICADRRIVDDWANDGWGLKKAADGAAEPGVVGQMIEAAEER
PWLWVVYILTVALPVFLVILFCCSGKKQTSGMEYKKTDAPQPDVKEEEEEKEEEKDKGDE
EEEGEEKLEEKQKSDAEEDGGTVSQEEEDRKPKAEEDEILNRSPRNRKPRRE
>sp|P28070|PSB4_HUMAN Proteasome subunit beta type-4 OS=Homo sapiens OX=9606 GN=PSMB4 PE=1 SV=4

MEAFLGSRSGLWAGGPAPGQFYRIPSTPDSFMDPASALYRGPITRTQNPMVTGTSVLGVK
FEGGVVIAADMLGSYGSLARFRNISRIMRVNNSTMLGASGDYADFQYLKQVLGQMVIDEE
LLGDGHSYSPRAIHSWLTRAMYSRRSKMNPLWNTMVIGGYADGESFLGYVDMLGVAYEAP
SLATGYGAYLAQPLLREVLEKQPVLSQTEARDLVERCMRVLYYRDARSYNRFQIATVTEK
GVEIEGPLSTETNWDIAHMISGFE
>sp|P28074|PSB5_HUMAN Proteasome subunit beta type-5 OS=Homo sapiens OX=9606 GN=PSMB5 PE=1 SV=3
MALASVLERPLPVNQRGFFGLGGRADLLDLGPGSLSDGLSLAAPGWGVPEEPGIEMLHGT
TTLAFKFRHGVIVAADSRATAGAYIASQTVKKVIEINPYLLGTMAGGAADCSFWERLLAR
QCRIYELRNKERISVAAASKLLANMVYQYKGMGLSMGTMICGWDKRGPGLYYVDSEGNRI
SGATFSVGSGSVYAYGVMDRGYSYDLEVEQAYDLARRAIYQATYRDAYSGGAVNLYHVRE
DGWIRVSSDNVADLHEKYSGSTP
>sp|P28161|GSTM2_HUMAN Glutathione S-transferase Mu 2 OS=Homo sapiens OX=9606 GN=GSTM2 PE=1 SV=2
MPMTLGYWNIRGLAHSIRLLLEYTDSSYEEKKYTMGDAPDYDRSQWLNEKFKLGLDFPNL
PYLIDGTHKITQSNAILRYIARKHNLCGESEKEQIREDILENQFMDSRMQLAKLCYDPDF
EKLKPEYLQALPEMLKLYSQFLGKQPWFLGDKITFVDFIAYDVLERNQVFEPSCLDAFPN
LKDFISRFEGLEKISAYMKSSRFLPRPVFTKMAVWGNK
>sp|P28482|MK01_HUMAN Mitogen-activated protein kinase 1 OS=Homo sapiens OX=9606 GN=MAPK1 PE=1 SV=3
MAAAAAAGAGPEMVRGQVFDVGPRYTNLSYIGEGAYGMVCSAYDNVNKVRVAIKKISPFE
HQTYCQRTLREIKILLRFRHENIIGINDIIRAPTIEQMKDVYIVQDLMETDLYKLLKTQH
LSNDHICYFLYQILRGLKYIHSANVLHRDLKPSNLLLNTTCDLKICDFGLARVADPDHDH
TGFLTEYVATRWYRAPEIMLNSKGYTKSIDIWSVGCILAEMLSNRPIFPGKHYLDQLNHI
LGILGSPSQEDLNCIINLKARNYLLSLPHKNKVPWNRLFPNADSKALDLLDKMLTFNPHK
RIEVEQALAHPYLEQYYDPSDEPIAEAPFKFDMELDDLPKEKLKELIFEETARFQPGYRS
>sp|P29218|IMPA1_HUMAN Inositol monophosphatase 1 OS=Homo sapiens OX=9606 GN=IMPA1 PE=1 SV=1
MADPWQECMDYAVTLARQAGEVVCEAIKNEMNVMLKSSPVDLVTATDQKVEKMLISSIKE
KYPSHSFIGEESVAAGEKSILTDNPTWIIDPIDGTTNFVHRFPFVAVSIGFAVNKKIEFG
VVYSCVEGKMYTARKGKGAFCNGQKLQVSQQEDITKSLLVTELGSSRTPETVRMVLSNME
KLFCIPVHGIRSVGTAAVNMCLVATGGADAYYEMGIHCWDVAGAGIIVTEAGGVLMDVTG
GPFDLMSRRVIAANNRILAERIAKEIQVIPLQRDDED
>sp|P29317|EPHA2_HUMAN Ephrin type-A receptor 2 OS=Homo sapiens OX=9606 GN=EPHA2 PE=1 SV=2
MELQAARACFALLWGCALAAAAAAQGKEVVLLDFAAAGGELGWLTHPYGKGWDLMQNIMN
DMPIYMYSVCNVMSGDQDNWLRTNWVYRGEAERIFIELKFTVRDCNSFPGGASSCKETFN
LYYAESDLDYGTNFQKRLFTKIDTIAPDEITVSSDFEARHVKLNVEERSVGPLTRKGFYL
AFQDIGACVALLSVRVYYKKCPELLQGLAHFPETIAGSDAPSLATVAGTCVDHAVVPPGG
EEPRMHCAVDGEWLVPIGQCLCQAGYEKVEDACQACSPGFFKFEASESPCLECPEHTLPS
PEGATSCECEEGFFRAPQDPASMPCTRPPSAPHYLTAVGMGAKVELRWTPPQDSGGREDI
VYSVTCEQCWPESGECGPCEASVRYSEPPHGLTRTSVTVSDLEPHMNYTFTVEARNGVSG
LVTSRSFRTASVSINQTEPPKVRLEGRSTTSLSVSWSIPPPQQSRVWKYEVTYRKKGDSN
SYNVRRTEGFSVTLDDLAPDTTYLVQVQALTQEGQGAGSKVHEFQTLSPEGSGNLAVIGG

VAVGVVLLLVLAGVGFFIHRRRKNQRARQSPEDVYFSKSEQLKPLKTYVDPHTYEDPNQA
VLKFTTEIHPSCVTRQKVIGAGEFGEVYKGMLKTSSGKKEVPVAIKTLKAGYTEKQRVDF
LGEAGIMGQFSHHNIIRLEGVISKYKPMMIITEYMENGALDKFLREKDGEFSVLQLVGML
RGIAAGMKYLANMNYVHRDLAARNILVNSNLVCKVSDFGLSRVLEDDPEATYTTSGGKIP
IRWTAPEAISYRKFTSASDVWSFGIVMWEVMTYGERPYWELSNHEVMKAINDGFRLPTPM
DCPSAIYQLMMQCWQQERARRPKFADIVSILDKLIRAPDSLKTLADFDPRVSIRLPSTSG
SEGVPFRTVSEWLESIKMQQYTEHFMAAGYTAIEKVVQMTNDDIKRIGVRLPGHQKRIAY
SLLGLKDQVNTVGIPI
>sp|P29400|CO4A5_HUMAN Collagen alpha-5(IV) chain OS=Homo sapiens OX=9606
GN=COL4A5 PE=1 SV=2
MKLRGVSLAAGLFLLALSLWGQPAEAAACYGCSPGSKCDCSGIKGEKGERGFPGLEGHPG
LPGFPGPEGPPGPRGQKGDDGIPGPPGPKGIRGPPGLPGFPGTPGLPGMPGHDGAPGPQG
IPGCNGTKGERGFPGSPGFPGLQGPPGPPGIPGMKGEPGSIIMSSLPGPKGNPGYPGPPG
IQGLPGPTGIPGPIGPPGPPGLMGPPGPPGLPGPKGNMGLNFQGPKGEKGEQGLQGPPGP
PGQISEQKRPIDVEFQKGDQGLPGDRGPPGPPGIRGPPGPPGGEKGEKGEQGEPGKRGKP
GKDGENGQPGIPGLPGDPGYPGEPGRDGEKGQKGDTGPPGPPGLVIPRPGTGITIGEKGN
IGLPGLPGEKGERGFPGIQGPPGLPGPPGAAVMGPPGPPGFPGERGQKGDEGPPGISIPG
PPGLDGQPGAPGLPGPPGPAGPHIPPSDEICEPGPPGPPGSPGDKGLQGEQGVKGDKGDT
CFNCIGTGISGPPGQPGLPGLPGPPGSLGFPGQKGEKGQAGATGPKGLPGIPGAPGAPGF
PGSKGEPGDILTFPGMKGDKGELGSPGAPGLPGLPGTPGQDGLPGLPGPKGEPGGITFKG
ERGPPGNPGLPGLPGNIGPMGPPGFGPPGPVGEKGIQGVAGNPGQPGIPGPKGDPGQTIT
QPGKPGLPGNPGRDGDVGLPGDPGLPGQPGLPGIPGSKGEPGIPGIGLPGPPGPKGFPGI
PGPPGAPGTPGRIGLEGPPGPPGFPGPKGEPGFALPGPPGPPGLPGFKGALGPKGDRGFP
GPPGPPGRTGLDGLPGPKGDVGPNGQPGPMGPPGLPGIGVQGPPGPPGIPGPIGQPGLHG
IPGEKGDPGPPGLDVPGPPGERGSPGIPGAPGPIGPPGSPGLPGKAGASGFPGTKGEMGM
MGPPGPPGPLGIPGRSGVPGLKGDDGLQGQPGLPGPTGEKGSKGEPGLPGPPGPMDPNLL
GSKGEKGEPGLPGIPGVSGPKGYQGLPGDPGQPGLSGQPGLPGPPGPKGNPGLPGQPGLI
GPPGLKGTIGDMGFPGPQGVEGPPGPSGVPGQPGSPGLPGQKGDKGDPGISSIGLPGLPG
PKGEPGLPGYPGNPGIKGSVGDPGLPGLPGTPGAKGQPGLPGFPGTPGPPGPKGISGPPG
NPGLPGEPGPVGGGGHPGQPGPPGEKGKPGQDGIPGPAGQKGEPGQPGFGNPGPPGLPGL
SGQKGDGGLPGIPGNPGLPGPKGEPGFHGFPGVQGPPGPPGSPGPALEGPKGNPGPQGPP
GRPGLPGPEGPPGLPGNGGIKGEKGNPGQPGLPGLPGLKGDQGPPGLQGNPGRPGLNGMK
GDPGLPGVPGFPGMKGPSGVPGSAGPEGEPGLIGPPGPPGLPGPSGQSIIIKGDAGPPGI
PGQPGLKGLPGPQGPQGLPGPTGPPGDPGRNGLPGFDGAGGRKGDPGLPGQPGTRGLDGP
PGPDGLQGPPGPPGTSSVAHGFLITRHSQTTDAPQCPQGTLQVYEGFSLLYVQGNKRAHG
QDLGTAGSCLRRFSTMPFMFCNINNVCNFASRNDYSYWLSTPEPMPMSMQPLKGQSIQPF
ISRCAVCEAPAVVIAVHSQTIQIPHCPQGWDSLWIGYSFMMHTSAGAEGSGQALASPGSC
LEEFRSAPFIECHGRGTCNYYANSYSFWLATVDVSDMFSKPQSETLKAGDLRTRISRCQV
CMKRT
>sp|P29401|TKT_HUMAN Transketolase OS=Homo sapiens OX=9606 GN=TKT PE=1 SV=3
MESYHKPDQQKLQALKDTANRLRISSIQATTAAGSGHPTSCCSAAEIMAVLFFHTMRYKS
QDPRNPHNDRFVLSKGHAAPILYAVWAEAGFLAEAELLNLRKISSDLDGHPVPKQAFTDV
ATGSLGQGLGAACGMAYTGKYFDKASYRVYCLLGDGELSEGSVWEAMAFASIYKLDNLVA
ILDINRLGQSDPAPLQHQMDIYQKRCEAFGWHAIIVDGHSVEELCKAFGQAKHQPTAIIA

KTFKGRGITGVEDKESWHGKPLPKNMAEQIIQEIYSQIQSKKKILATPPQEDAPSVDIAN
IRMPSLPSYKVGDKIATRKAYGQALAKLGHASDRIIALDGDTKNSTFSEIFKKEHPDRFI
ECYIAEQNMVSIAVGCATRNRTVPFCSTFAAFFTRAFDQIRMAAISESNINLCGSHCGVS
IGEDGPSQMALEDLAMFRSVPTSTVFYPSDGVATEKAVELAANTKGICFIRTSRPENAII
YNNNEDFQVGQAKVVLKSKDDQVTVIGAGVTLHEALAAAELLKKEKINIRVLDPFTIKPL
DRKLILDSARATKGRILTVEDHYYEGGIGEAVSSAVVGEPGITVTHLAVNRVPRSGKPAE
LLKMFGIDRDAIAQAVRGLITKA
>sp|P29992|GNA11_HUMAN Guanine nucleotide-binding protein subunit alpha-11 OS=Homo sapiens OX=9606 GN=GNA11 PE=1 SV=2
MTLESMMACCLSDEVKESKRINAEIEKQLRRDKRDARRELKLLLLGTGESGKSTFIKQMR
IIHGAGYSEEDKRGFTKLVYQNIFTAMQAMIRAMETLKILYKYEQNKANALLIREVDVEK
VTTFEHQYVSAIKTLWEDPGIQECYDRRREYQLSDSAKYYLTDVDRIATLGYLPTQQDVL
RVRVPTTGIIEYPFDLENIIFRMVDVGGQRSERRKWIHCFENVTSIMFLVALSEYDQVLV
ESDNENRMEESKALFRTIITYPWFQNSSVILFLNKKDLLEDKILYSHLVDYFPEFDGPQR
DAQAAREFILKMFVDLNPDSDKIIYSHFTCATDTENIRFVFAAVKDTILQLNLKEYNLV
>sp|P30041|PRDX6_HUMAN Peroxiredoxin-6 OS=Homo sapiens OX=9606 GN=PRDX6 PE=1 SV=3
MPGGLLLGDVAPNFEANTTVGRIRFHDFLGDSWGILFSHPRDFTPVCTTELGRAAKLAPE
FAKRNVKLIALSIDSVEDHLAWSKDINAYNCEEPTEKLPFPIIDDRNRELAILLGMLDPA
EKDEKGMPVTARVVFVFGPDKKLKLSILYPATTGRNFDEILRVVISLQLTAEKRVATPVD
WKDGDSVMVLPTIPEEEAKKLFPKGVFTKELPSGKKYLRYTPQP
>sp|P30086|PEBP1_HUMAN Phosphatidylethanolamine-binding protein 1 OS=Homo sapiens OX=9606 GN=PEBP1 PE=1 SV=3
MPVDLSKWSGPLSLQEVDEQPQHPLHVTYAGAAVDELGKVLTPTQVKNRPTSISWDGLDS
GKLYTLVLTDPDAPSRKDPKYREWHHFLVVNMKGNDISSGTVLSDYVGSGPPKGTGLHRY
VWLVYEQDRPLKCDEPILSNRSGDHRGKFKVASFRKKYELRAPVAGTCYQAEWDDYVPKL
YEQLSGK
>sp|P30101|PDIA3_HUMAN Protein disulfide-isomerase A3 OS=Homo sapiens OX=9606 GN=PDIA3 PE=1 SV=4
MRLRRLALFPGVALLLAAARLAAASDVLELTDDNFESRISDTGSAGLMLVEFFAPWCGHC
KRLAPEYEAAATRLKGIVPLAKVDCTANTNTCNKYGVSGYPTLKIFRDGEEAGAYDGPRT
ADGIVSHLKKQAGPASVPLRTEEEFKKFISDKDASIVGFFDDSFSEAHSEFLKAASNLRD
NYRFAHTNVESLVNEYDDNGEGIILFRPSHLTNKFEDKTVAYTEQKMTSGKIKKFIQENI
FGICPHMTEDNKDLIQGKDLLIAYYDVDYEKNAKGSNYWRNRVMMVAKKFLDAGHKLNFA
VASRKTFSHELSDFGLESTAGEIPVVAIRTAKGEKFVMQEEFSRDGKALERFLQDYFDGN
LKRYLKSEPIPESNDGPVKVVVAENFDEIVNNENKDVLIEFYAPWCGHCKNLEPKYKELG
EKLSKDPNIVIAKMDATANDVPSPYEVRGFPTIYFSPANKKLNPKKYEGGRELSDFISYL
QREATNPPVIQEEKPKKKKKAQEDL
>sp|P30153|2AAA_HUMAN Serine/threonine-protein phosphatase 2A 65 kDa regulatory subunit A alpha isoform OS=Homo sapiens OX=9606 GN=PPP2R1A PE=1 SV=4
MAAADGDDSLYPIAVLIDELRNEDVQLRLNSIKKLSTIALALGVERTRSELLPFLTDTIY
DEDEVLLALAEQLGTFTTLVGGPEYVHCLLPPLESLATVEETVVRDKAVESLRAISHEHS
PSDLEAHFVPLVKRLAGGDWFTSRTSACGLFSVCYPRVSSAVKAELRQYFRNLCSDDTPM
VRRAAASKLGEFAKVLELDNVKSEIIPMFSNLASDEQDSVRLLAVEACVNIAQLLPQEDL

EALVMPTLRQAAEDKSWRVRYMVADKFTELQKAVGPEITKTDLVPAFQNLMKDCEAEVRA
AASHKVKEFCENLSADCRENVIMSQILPCIKELVSDANQHVKSALASVIMGLSPILGKDN
TIEHLLPLFLAQLKDECPEVRLNIISNLDCVNEVIGIRQLSQSLLPAIVELAEDAKWRVR
LAIIEYMPLLAGQLGVEFFDEKLNSLCMAWLVDHVYAIREAATSNLKKLVEKFGKEWAHA
TIIPKVLAMSGDPNYLHRMTTLFCINVLSEVCGQDITTKHMLPTVLRMAGDPVANVRFNV
AKSLQKIGPILDNSTLQSEVKPILEKLTQDQDVDVKYFAQEALTVLSLA
>sp|P30301|MIP_HUMAN Lens fiber major intrinsic protein OS=Homo sapiens OX=9606 GN=MIP PE=1 SV=1
MWELRSASFWRAIFAEFFATLFYVFFGLGSSLRWAPGPLHVLQVAMAFGLALATLVQSVG
HISGAHVNPAVTFAFLVGSQMSLLRAFCYMAAQLLGAVAGAAVLYSVTPPAVRGNLALNT
LHPAVSVGQATTVEIFLTLQFVLCIFATYDERRNGQLGSVALAVGFSLALGHLFGMYYTG
AGMNPARSFAPAILTGNFTNHWVYWVGPIIGGGLGSLLYDFLLFPRLKSISERLSVLKGA
KPDVSNGQPEVTGEPVELNTQAL
>sp|P30566|PUR8_HUMAN Adenylosuccinate lyase OS=Homo sapiens OX=9606 GN=ADSL PE=1 SV=2
MAAGGDHGSPDSYRSPLASRYASPEMCFVFSDRYKFRTWRQLWLWLAEAEQTLGLPITDE
QIQEMKSNLENIDFKMAAEEEKRLRHDVMAHVHTFGHCCPKAAGIIHLGATSCYVGDNTD
LIILRNALDLLLPKLARVISRLADFAKERASLPTLGFTHFQPAQLTTVGKRCCLWIQDLC
MDLQNLKRVRDDLRFRGVKGTTGTQASFLQLFEGDDHKVEQLDKMVTEKAGFKRAFIITG
QTYTRKVDIEVLSVLASLGASVHKICTDIRLLANLKEMEEPFEKQQIGSSAMPYKRNPMR
SERCCSLARHLMTLVMDPLQTASVQWFERTLDDSANRRICLAEAFLTADTILNTLQNISE
GLVVYPKVIERRIRQELPFMATENIIMAMVKAGGSRQDCHEKIRVLSQQAASVVKQEGGD
NDLIERIQVDAYFSPIHSQLDHLLDPSSFTGRASQQVQRFLEEEVYPLLKPYESVMKVKA
ELCL
>sp|P30711|GSTT1_HUMAN Glutathione S-transferase theta-1 OS=Homo sapiens OX=9606 GN=GSTT1 PE=1 SV=4
MGLELYLDLLSQPCRAVYIFAKKNDIPFELRIVDLIKGQHLSDAFAQVNPLKKVPALKDG
DFTLTESVAILLYLTRKYKVPDYWYPQDLQARARVDEYLAWQHTTLRRSCLRALWHKVMF
PVFLGEPVSPQTLAATLAELDVTLQLLEDKFLQNKAFLTGPHISLADLVAITELMHPVGA
GCQVFEGRPKLATWRQRVEAAVGEDLFQEAHEVILKAKDFPPADPTIKQKLMPWVLAMIR
>sp|P30740|ILEU_HUMAN Leukocyte elastase inhibitor OS=Homo sapiens OX=9606 GN=SERPINB1 PE=1 SV=1
MEQLSSANTRFALDLFLALSENNPAGNIFISPFSISSAMAMVFLGTRGNTAAQLSKTFHF
NTVEEVHSRFQSLNADINKRGASYILKLANRLYGEKTYNFLPEFLVSTQKTYGADLASVD
FQHASEDARKTINQWVKGQTEGKIPELLASGMVDNMTKLVLVNAIYFKGNWKDKFMKEAT
TNAPFRLNKKDRKTVKMMYQKKKFAYGYIEDLKCRVLELPYQGEELSMVILLPDDIEDES
TGLKKIEEQLTLEKLHEWTKPENLDFIEVNVSLPRFKLEESYTLNSDLARLGVQDLFNSS
KADLSGMSGARDIFISKIVHKSFVEVNEEGTEAAAATAGIATFCMLMPEENFTADHPFLF
FIRHNSSGSILFLGRFSSP
>sp|P31150|GDIA_HUMAN Rab GDP dissociation inhibitor alpha OS=Homo sapiens OX=9606 GN=GDI1 PE=1 SV=2
MDEEYDVIVLGTGLTECILSGIMSVNGKKVLHMDRNPYYGGESSSITPLEELYKRFQLLE
GPPESMGRGRDWNVDLIPKFLMANGQLVKMLLYTEVTRYLDFKVVEGSFVYKGGKIYKVP
STETEALASNLMGMFEKRRFRKFLVFVANFDENDPKTFEGVDPQTTSMRDVYRKFDLGQD

VIDFTGHALALYRTDDYLDQPCLETVNRIKLYSESLARYGKSPYLYPLYGLGELPQGFAR
LSAIYGGTYMLNKPVDDIIMENGKVVGVKSEGEVARCKQLICDPSYIPDRVRKAGQVIRI
ICILSHPIKNTNDANSCQIIIPQNQVNRKSDIYVCMISYAHNVAAQGKYIAIASTTVETT
DPEKEVEPALELLEPIDQKFVAISDLYEPIDDGCESQVFCSCSYDATTHFETTCNDIKDI
YKRMAGTAFDFENMKRKQNDVFGEAEQ
>sp|P31153|METK2_HUMAN S-adenosylmethionine synthase isoform type-2 OS=Homo sapiens OX=9606 GN=MAT2A PE=1 SV=1
MNGQLNGFHEAFIEEGTFLFTSESVGEGHPDKICDQISDAVLDAHLQQDPDAKVACETVA
KTGMILLAGEITSRAAVDYQKVVREAVKHIGYDDSSKGFDYKTCNVLVALEQQSPDIAQG
VHLDRNEEDIGAGDQGLMFGYATDETEECMPLTIVLAHKLNAKLAELRRNGTLPWLRPDS
KTQVTVQYMQDRGAVLPIRVHTIVISVQHDEEVCLDEMRDALKEKVIKAVVPAKYLDEDT
IYHLQPSGRFVIGGPQGDAGLTGRKIIVDTYGGWGAHGGGAFSGKDYTKVDRSAAYAARW
VAKSLVKGGLCRRVLVQVSYAIGVSHPLSISIFHYGTSQKSERELLEIVKKNFDLRPGVI
VRDLDLKKPIYQRTAAYGHFGRDSFPWEVPKKLKY
>sp|P31213|S5A2_HUMAN 3-oxo-5-alpha-steroid 4-dehydrogenase 2 OS=Homo sapiens OX=9606 GN=SRD5A2 PE=1 SV=2
MQVQCQQSPVLAGSATLVALGALALYVAKPSGYGKHTESLKPAATRLPARAAWFLQELPS
FAVPAGILARQPLSLFGPPGTVLLGLFCLHYFHRTFVYSLLNRGRPYPAILILRGTAFCT
GNGVLQGYYLIYCAEYPDGWYTDIRFSLGVFLFILGMGINIHSDYILRQLRKPGEISYRI
PQGGLFTYVSGANFLGEIIEWIGYALATWSLPALAFAFFSLCFLGLRAFHHHRFYLKMFE
DYPKSRKALIPFIF
>sp|P31689|DNJA1_HUMAN DnaJ homolog subfamily A member 1 OS=Homo sapiens OX=9606 GN=DNAJA1 PE=1 SV=2
MVKETTYYDVLGVKPNATQEELKKAYRKLALKYHPDKNPNEGEKFKQISQAYEVLSDAKK
RELYDKGGEQAIKEGGAGGGFGSPMDIFDMFFGGGGRMQRERRGKNVVHQLSVTLEDLYN
GATRKLALQKNVICDKCEGRGGKKGAVECCPNCRGTGMQIRIHQIGPGMVQQIQSVCMEC
QGHGERISPKDRCKSCNGRKIVREKKILEVHIDKGMKDGQKITFHGEGDQEPGLEPGDII
IVLDQKDHAVFTRRGEDLFMCMDIQLVEALCGFQKPISTLDNRTIVITSHPGQIVKHGDI
KCVLNEGMPIYRRPYEKGRLIIEFKVNFPENGFLSPDKLSLLEKLLPERKEVEETDEMDQ
VELVDFDPNQERRRHYNGEAYEDDEHHPRGGVQCQTS
>sp|P31939|PUR9_HUMAN Bifunctional purine biosynthesis protein ATIC OS=Homo sapiens OX=9606 GN=ATIC PE=1 SV=3
MAPGQLALFSVSDKTGLVEFARNLTALGLNLVASGGTAKALRDAGLAVRDVSELTGFPEM
LGGRVKTLHPAVHAGILARNIPEDNADMARLDFNLIRVVACNLYPFVKTVASPGVTVEEA
VEQIDIGGVTLLRAAAKNHARVTVVCEPEDYVVVSTEMQSSESKDTSLETRRQLALKAFT
HTAQYDEAISDYFRKQYSKGVSQMPLRYGMNPHQTPAQLYTLQPKLPITVLNGAPGFINL
CDALNAWQLVKELKEALGIPAAASFKHVSPAGAAVGIPLSEDEAKVCMVYDLYKTLTPIS
AAYARARGADRMSSFGDFVALSDVCDVPTAKIISREVSDGIIAPGYEEEALTILSKKKNG
NYCVLQMDQSYKPDENEVRTLFGLHLSQKRNNGVVDKSLFSNVVTKNKDLPESALRDLIV
ATIAVKYTQSNSVCYAKNGQVIGIGAGQQSRIHCTRLAGDKANYWWLRHHPQVLSMKFKT
GVKRAEISNAIDQYVTGTIGEDEDLIKWKALFEEVPELLTEAEKKEWVEKLTEVSISSDA
FFPFRDNVDRAKRSGVAYIAAPSGSAADKVVIEACDELGIILAHTNLRLFHH
>sp|P31946|1433B_HUMAN 14-3-3 protein beta/alpha OS=Homo sapiens OX=9606 GN=YWHAB PE=1 SV=3

MTMDKSELVQKAKLAEQAERYDDMAAAMKAVTEQGHELSNEERNLLSVAYKNVVGARRSS
WRVISSIEQKTERNEKKQQMGKEYREKIEAELQDICNDVLELLDKYLIPNATQPESKVFY
LKMKGDYFRYLSEVASGDNKQTTVSNSQQAYQEAFEISKKEMQPTHPIRLGLALNFSVFY
YEILNSPEKACSLAKTAFDEAIAELDTLNEESYKDSTLIMQLLRDNLTLWTSENQGDEGD
AGEGEN
>sp|P31947|1433S_HUMAN 14-3-3 protein sigma OS=Homo sapiens OX=9606 GN=SFN PE=1 SV=1
MERASLIQKAKLAEQAERYEDMAAFMKGAVEKGEELSCEERNLLSVAYKNVVGGQRAAWR
VLSSIEQKSNEEGSEEKGPEVREYREKVETELQGVCDTVLGLLDSHLIKEAGDAESRVFY
LKMKGDYYRYLAEVATGDDKKRIIDSARSAYQEAMDISKKEMPPTNPIRLGLALNFSVFH
YEIANSPEEAISLAKTTFDEAMADLHTLSEDSYKDSTLIMQLLRDNLTLWTADNAGEEGG
EAPQEPQS
>sp|P31948|STIP1_HUMAN Stress-induced-phosphoprotein 1 OS=Homo sapiens OX=9606 GN=STIP1 PE=1 SV=1
MEQVNELKEKGNKALSVGNIDDALQCYSEAIKLDPHNHVLYSNRSAAYAKKGDYQKAYED
GCKTVDLKPDWGKGYSRKAAALEFLNRFEEAKRTYEEGLKHEANNPQLKEGLQNMEARLA
ERKFMNPFNMPNLYQKLESDPRTRTLLSDPTYRELIEQLRNKPSDLGTKLQDPRIMTTLS
VLLGVDLGSMDEEEEIATPPPPPPPKKETKPEPMEEDLPENKKQALKEKELGNDAYKKKD
FDTALKHYDKAKELDPTNMTYITNQAAVYFEKGDYNKCRELCEKAIEVGRENREDYRQIA
KAYARIGNSYFKEEKYKDAIHFYNKSLAEHRTPDVLKKCQQAEKILKEQERLAYINPDLA
LEEKNKGNECFQKGDYPQAMKHYTEAIKRNPKDAKLYSNRAACYTKLLEFQLALKDCEEC
IQLEPTFIKGYTRKAAALEAMKDYTKAMDVYQKALDLDSSCKEAADGYQRCMMAQYNRHD
SPEDVKRRAMADPEVQQIMSDPAMRLILEQMQKDPQALSEHLKNPVIAQKIQKLMDVGLI
AIR
>sp|P32119|PRDX2_HUMAN Peroxiredoxin-2 OS=Homo sapiens OX=9606 GN=PRDX2 PE=1 SV=5
MASGNARIGKPAPDFKATAVVDGAFKEVKLSDYKGKYVVLFFYPLDFTFVCPTEIIAFSN
RAEDFRKLGCEVLGVSVDSQFTHLAWINTPRKEGGLGPLNIPLLADVTRRLSEDYGVLKT
DEGIAYRGLFIIDGKGVLRQITVNDLPVGRSVDEALRLVQAFQYTDEHGEVCPAGWKPGS
DTIKPNVDDSKEYFSKHN
>sp|P32320|CDD_HUMAN Cytidine deaminase OS=Homo sapiens OX=9606 GN=CDA PE=1 SV=2
MAQKRPACTLKPECVQQLLVCSQEAKKSAYCPYSHFPVGAALLTQEGRIFKGCNIENACY
PLGICAERTAIQKAVSEGYKDFRAIAIASDMQDDFISPCGACRQVMREFGTNWPVYMTKP
DGTYIVMTVQELLPSSFGPEDLQKTQ
>sp|P32754|HPPD_HUMAN 4-hydroxyphenylpyruvate dioxygenase OS=Homo sapiens OX=9606 GN=HPD PE=1 SV=2
MTTYSDKGAKPERGRFLHFHSVTFWVGNAKQAASFYCSKMGFEPLAYRGLETGSREVVSH
VIKQGKIVFVLSSALNPWNKEMGDHLVKHGDGVKDIAFEVEDCDYIVQKARERGAKIMRE
PWVEQDKFGKVKFAVLQTYGDTTHTLVEKMNYIGQFLPGYEAPAFMDPLLPKLPKCSLEM
IDHIVGNQPDQEMVSASEWYLKNLQFHRFWSVDDTQVHTEYSSLRSIVVANYEESIKMPI
NEPAPGKKKSQIQEYVDYNGGAGVQHIALKTEDIITAIRHLRERGLEFLSVPSTYYKQLR
EKLKTAKIKVKENIDALEELKILVDYDEKGYLLQIFTKPVQDRPTLFLEVIQRHNHQGFG
AGNFNSLFKAFEEEQNLRGNLTNMETNGVVPGM
>sp|P32856|STX2_HUMAN Syntaxin-2 OS=Homo sapiens OX=9606 GN=STX2 PE=1 SV=3

MRDRLPDLTACRKNDDGDTVVVVEKDHFMDDFFHQVEEIRNSIDKITQYVEEVKKNHSII
LSAPNPEGKIKEELEDLNKEIKKTANKIRAKLKAIEQSFDQDESGNRTSVDLRIRRTQHS
VLSRKFVEAMAEYNEAQTLFRERSKGRIQRQLEITGRTTTDDELEEMLESGKPSIFTSDI
ISDSQITRQALNEIESRHKDIMKLETSIRELHEMFMDMAMFVETQGEMINNIERNVMNAT
DYVEHAKEETKKAIKYQSKARRKKWIIIAVSVVLVAIIALIIGLSVGK
>sp|P33527|MRP1_HUMAN Multidrug resistance-associated protein 1 OS=Homo sapiens OX=9606 GN=ABCC1 PE=1 SV=3
MALRGFCSADGSDPLWDWNVTWNTSNPDFTKCFQNTVLVWVPCFYLWACFPFYFLYLSRH
DRGYIQMTPLNKTKTALGFLLWIVCWADLFYSFWERSRGIFLAPVFLVSPTLLGITMLLA
TFLIQLERRKGVQSSGIMLTFWLVALVCALAILRSKIMTALKEDAQVDLFRDITFYVYFS
LLLIQLVLSCFSDRSPLFSETIHDPNPCPESSASFLSRITFWWITGLIVRGYRQPLEGSD
LWSLNKEDTSEQVVPVLVKNWKKECAKTRKQPVKVVYSSKDPAQPKESSKVDANEEVEAL
IVKSPQKEWNPSLFKVLYKTFGPYFLMSFFFKAIHDLMMFSGPQILKLLIKFVNDTKAPD
WQGYFYTVLLFVTACLQTLVLHQYFHICFVSGMRIKTAVIGAVYRKALVITNSARKSSTV
GEIVNLMSVDAQRFMDLATYINMIWSAPLQVILALYLLWLNLGPSVLAGVAVMVLMVPVN
AVMAMKTKTYQVAHMKSKDNRIKLMNEILNGIKVLKLYAWELAFKDKVLAIRQEELKVLK
KSAYLSAVGTFTWVCTPFLVALCTFAVYVTIDENNILDAQTAFVSLALFNILRFPLNILP
MVISSIVQASVSLKRLRIFLSHEELEPDSIERRPVKDGGGTNSITVRNATFTWARSDPPT
LNGITFSIPEGALVAVVGQVGCGKSSLLSALLAEMDKVEGHVAIKGSVAYVPQQAWIQND
SLRENILFGCQLEEPYYRSVIQACALLPDLEILPSGDRTEIGEKGVNLSGGQKQRVSLAR
AVYSNADIYLFDDPLSAVDAHVGKHIFENVIGPKGMLKNKTRILVTHSMSYLPQVDVIIV
MSGGKISEMGSYQELLARDGAFAEFLRTYASTEQEQDAEENGVTGVSGPGKEAKQMENGM
LVTDSAGKQLQRQLSSSSSYSGDISRHHNSTAELQKAEAKKEETWKLMEADKAQTGQVKL
SVYWDYMKAIGLFISFLSIFLFMCNHVSALASNYWLSLWTDDPIVNGTQEHTKVRLSVYG
ALGISQGIAVFGYSMAVSIGGILASRCLHVDLLHSILRSPMSFFERTPSGNLVNRFSKEL
DTVDSMIPEVIKMFMGSLFNVIGACIVILLATPIAAIIIPPLGLIYFFVQRFYVASSRQL
KRLESVSRSPVYSHFNETLLGVSVIRAFEEQERFIHQSDLKVDENQKAYYPSIVANRWLA
VRLECVGNCIVLFAALFAVISRHSLSAGLVGLSVSYSLQVTTYLNWLVRMSSEMETNIVA
VERLKEYSETEKEAPWQIQETAPPSSWPQVGRVEFRNYCLRYREDLDFVLRHINVTINGG
EKVGIVGRTGAGKSSLTLGLFRINESAEGEIIIDGINIAKIGLHDLRFKITIIPQDPVLF
SGSLRMNLDPFSQYSDEEVWTSLELAHLKDFVSALPDKLDHECAEGGENLSVGQRQLVCL
ARALLRKTKILVLDEATAAVDLETDDLIQSTIRTQFEDCTVLTIAHRLNTIMDYTRVIVL
DKGEIQEYGAPSDLLQQRGLFYSMAKDAGLV
>sp|P33778|H2B1B_HUMAN Histone H2B type 1-B OS=Homo sapiens OX=9606 GN=H2BC3 PE=1 SV=2
MPEPSKSAPAPKKGSKKAITKAQKKDGKKRKRSRKESYSIYVYKVLKQVHPDTGISSKAM
GIMNSFVNDIFERIAGEASRLAHYNKRSTITSREIQTAVRLLLPGELAKHAVSEGTKAVT
KYTSSK
>sp|P34096|RNAS4_HUMAN Ribonuclease 4 OS=Homo sapiens OX=9606 GN=RNASE4 PE=1 SV=3
MALQRTHSLLLLLLLTLLGLGLVQPSYGQDGMYQRFLRQHVHPEETGGSDRYCNLMMQRR
KMTLYHCKRFNTFIHEDIWNIRSICSTTNIQCKNGKMNCHEGVVKVTDCRDTGSSRAPNC
RYRAIASTRRVVIACEGNPQVPVHFDG

>sp|P34896|GLYC_HUMAN Serine hydroxymethyltransferase, cytosolic OS=Homo sapiens OX=9606 GN=SHMT1 PE=1 SV=1
MTMPVNGAHKDADLWSSHDKMLAQPLKDSDVEVYNIIKKESNRQRVGLELIASENFASRA
VLEALGSCLNNKYSEGYPGQRYYGGTEFIDELETLCQKRALQAYKLDPQCWGVNVQPYSG
SPANFAVYTALVEPHGRIMGLDLPDGGHLTHGFMTDKKKISATSIFFESMPYKVNPDTGY
INYDQLEENARLFHPKLIIAGTSCYSRNLEYARLRKIADENGAYLMADMAHISGLVAAGV
VPSPFEHCHVVTTTTHKTLRGCRAGMIFYRKGVKSVDPKTGKEILYNLESLINSAVFPGL
QGGPHNHAIAGVAVALKQAMTLEFKVYQHQVVANCRALSEALTELGYKIVTGGSDNHLIL
VDLRSKGTDGGRAEKVLEACSIACNKNTCPGDRSALRPSGLRLGTPALTSRGLLEKDFQK
VAHFIHRGIELTLQIQSDTGVRATLKEFKERLAGDKYQAAVQALREEVESFASLFPLPGL
PDF
>sp|P34913|HYES_HUMAN Bifunctional epoxide hydrolase 2 OS=Homo sapiens OX=9606 GN=EPHX2 PE=1 SV=2
MTLRAAVFDLDGVLALPAVFGVLGRTEEALALPRGLLNDAFQKGGPEGATTRLMKGEITL
SQWIPLMEENCRKCSETAKVCLPKNFSIKEIFDKAISARKINRPMLQAALMLRKKGFTTA
ILTNTWLDDRAERDGLAQLMCELKMHFDFLIESCQVGMVKPEPQIYKFLLDTLKASPSEV
VFLDDIGANLKPARDLGMVTILVQDTDTALKELEKVTGIQLLNTPAPLPTSCNPSDMSHG
YVTVKPRVRLHFVELGSGPAVCLCHGFPESWYSWRYQIPALAQAGYRVLAMDMKGYGESS
APPEIEEYCMEVLCKEMVTFLDKLGLSQAVFIGHDWGGMLVWYMALFYPERVRAVASLNT
PFIPANPNMSPLESIKANPVFDYQLYFQEPGVAEAELEQNLSRTFKSLFRASDESVLSMH
KVCEAGGLFVNSPEEPSLSRMVTEEEIQFYVQQFKKSGFRGPLNWYRNMERNWKWACKSL
GRKILIPALMVTAEKDFVLVPQMSQHMEDWIPHLKRGHIEDCGHWTQMDKPTEVNQILIK
WLDSDARNPPVVSKM
>sp|P34932|HSP74_HUMAN Heat shock 70 kDa protein 4 OS=Homo sapiens OX=9606 GN=HSPA4 PE=1 SV=4
MSVVGIDLGFQSCYVAVARAGGIETIANEYSDRCTPACISFGPKNRSIGAAAKSQVISNA
KNTVQGFKRFHGRAFSDPFVEAEKSNLAYDIVQLPTGLTGIKVTYMEEERNFTTEQVTAM
LLSKLKETAESVLKKPVVDCVVSVPCFYTDAERRSVMDATQIAGLNCLRLMNETTAVALA
YGIYKQDLPALEEKPRNVVFVDMGHSAYQVSVCAFNRGKLKVLATAFDTTLGGRKFDEVL
VNHFCEEFGKKYKLDIKSKIRALLRLSQECEKLKKLMSANASDLPLSIECFMNDVDVSGT
MNRGKFLEMCNDLLARVEPPLRSVLEQTKLKKEDIYAVEIVGGATRIPAVKEKISKFFGK
ELSTTLNADEAVTRGCALQCAILSPAFKVREFSITDVVPYPISLRWNSPAEEGSSDCEVF
SKNHAAPFSKVLTFYRKEPFTLEAYYSSPQDLPYPDPAIAQFSVQKVTPQSDGSSSKVKV
KVRVNVHGIFSVSSASLVEVHKSEENEEPMETDQNAKEEEKMQVDQEEPHVEEQQQQTPA
ENKAESEEMETSQAGSKDKKMDQPPQAKKAKVKTSTVDLPIENQLLWQIDREMLNLYIEN
EGKMIMQDKLEKERNDAKNAVEEYVYEMRDKLSGEYEKFVSEDDRNSFTLKLEDTENWLY
EDGEDQPKQVYVDKLAELKNLGQPIKIRFQESEERPKLFEELGKQIQQYMKIISSFKNKE
DQYDHLDAADMTKVEKSTNEAMEWMNNKLNLQNKQSLTMDPVVKSKEIEAKIKELTSTCS
PIISKPKPKVEPPKEEQKNAEQNGPVDGQGDNPGPQAAEQGTDTAVPSDSDKKLPEMDID
>sp|P35221|CTNA1_HUMAN Catenin alpha-1 OS=Homo sapiens OX=9606 GN=CTNNA1 PE=1 SV=1
MTAVHAGNINFKWDPKSLEIRTLAVERLLEPLVTQVTTLVNTNSKGPSNKKRGRSKKAHV
LAASVEQATENFLEKGDKIAKESQFLKEELVAAVEDVRKQGDLMKAAAGEFADDPCSSVK
RGNMVRAARALLSAVTRLLILADMADVYKLLVQLKVVEDGILKLRNAGNEQDLGIQYKAL

KPEVDKLNIMAAKRQQELKDVGHRDQMAAARGILQKNVPILYTASQACLQHPDVAAYKAN
RDLIYKQLQQAVTGISNAAQATASDDASQHQGGGGGELAYALNNFDKQIIVDPLSFSEER
FRPSLEERLESIISGAALMADSSCTRDDRRERIVAECNAVRQALQDLLSEYMGNAGRKER
SDALNSAIDKMTKKTRDLRRQLRKAVMDHVSDSFLETNVPLLVLIEAAKNGNEKEVKEYA
QVFREHANKLIEVANLACSISNNEEGVKLVRMSASQLEALCPQVINAALALAAKPQSKLA
QENMDLFKEQWEKQVRVLTDAVDDITSIDDFLAVSENHILEDVNKCVIALQEKDVDGLDR
TAGAIRGRAARVIHVVTSEMDNYEPGVYTEKVLEATKLLSNTVMPRFTEQVEAAVEALSS
DPAQPMDENEFIDASRLVYDGIRDIRKAVLMIRTPEELDDSDFETEDFDVRSRTSVQTED
DQLIAGQSARAIMAQLPQEQKAKIAEQVASFQEEKSKLDAEVSKWDDSGNDIIVLAKQMC
MIMMEMTDFTRGKGPLKNTSDVISAAKKIAEAGSRMDKLGRTIADHCPDSACKQDLLAYL
QRIALYCHQLNICSKVKAEVQNLGGELVVSGVDSAMSLIQAAKNLMNAVVQTVKASYVAS
TKYQKSQGMASLNLPAVSWKMKAPEKKPLVKREKQDETQTKIKRASQKKHVNPVQALSEF
KAMDSI
>sp|P35222|CTNB1_HUMAN Catenin beta-1 OS=Homo sapiens OX=9606 GN=CTNNB1 PE=1
SV=1
MATQADLMELDMAMEPDRKAAVSHWQQQSYLDSGIHSGATTTAPSLSGKGNPEEEDVDTS
QVLYEWEQGFSQSFTQEQVADIDGQYAMTRAQRVRAAMFPETLDEGMQIPSTQFDAAHPT
NVQRLAEPSQMLKHAVVNLINYQDDAELATRAIPELTKLLNDEDQVVVNKAAVMVHQLSK
KEASRHAIMRSPQMVSAIVRTMQNTNDVETARCTAGTLHNLSHHREGLLAIFKSGGIPAL
VKMLGSPVDSVLFYAITTLHNLLLHQEGAKMAVRLAGGLQKMVALLNKTNVKFLAITTDC
LQILAYGNQESKLIILASGGPQALVNIMRTYTYEKLLWTTSRVLKVLSVCSSNKPAIVEA
GGMQALGLHLTDPSQRLVQNCLWTLRNLSDAATKQEGMEGLLGTLVQLLGSDDINVVTCA
AGILSNLTCNNYKNKMMVCQVGGIEALVRTVLRAGDREDITEPAICALRHLTSRHQEAEM
AQNAVRLHYGLPVVVKLLHPPSHWPLIKATVGLIRNLALCPANHAPLREQGAIPRLVQLL
VRAHQDTQRRTSMGGTQQQFVEGVRMEEIVEGCTGALHILARDVHNRIVIRGLNTIPLFV
QLLYSPIENIQRVAAGVLCELAQDKEAAEAIEAEGATAPLTELLHSRNEGVATYAAAVLF
RMSEDKPQDYKKRLSVELTSSLFRTEPMAWNETADLGLDIGAQGEPLGYRQDDPSYRSFH
SGGYGQDALGMDPMMEHEMGGHHPGADYPVDGLPDLGHAQDLMDGLPPGDSNQLAWFDTD
L
>sp|P35237|SPB6_HUMAN Serpin B6 OS=Homo sapiens OX=9606 GN=SERPINB6 PE=1 SV=3
MDVLAEANGTFALNLLKTLGKDNSKNVFFSPMSMSCALAMVYMGAKGNTAAQMAQILSFN
KSGGGGDIHQGFQSLLTEVNKTGTQYLLRMANRLFGEKSCDFLSSFRDSCQKFYQAEMEE
LDFISAVEKSRKHINTWVAEKTEGKIAELLSPGSVDPLTRLVLVNAVYFRGNWDEQFDKE
NTEERLFKVSKNEEKPVQMMFKQSTFKKTYIGEIFTQILVLPYVGKELNMIIMLPDETTD
LRTVEKELTYEKFVEWTRLDMMDEEEVEVSLPRFKLEESYDMESVLRNLGMTDAFELGKA
DFSGMSQTDLSLSKVVHKSFVEVNEEGTEAAAATAAIMMMRCARFVPRFCADHPFLFFIQ
HSKTNGILFCGRFSSP
>sp|P35241|RADI_HUMAN Radixin OS=Homo sapiens OX=9606 GN=RDX PE=1 SV=1
MPKPINVRVTTMDAELEFAIQPNTTGKQLFDQVVKTVGLREVWFFGLQYVDSKGYSTWLK
LNKKVTQQDVKKENPLQFKFRAKFFPEDVSEELIQEITQRLFFLQVKEAILNDEIYCPPE
TAVLLASYAVQAKYGDYNKEIHKPGYLANDRLLPQRVLEQHKLTKEQWEERIQNWHEEHR
GMLREDSMMEYLKIAQDLEMYGVNYFEIKNKKGTELWLGVDALGLNIYEHDDKLTPKIGF
PWSEIRNISFNDKKFVIKPIDKKAPDFVFYAPRLRINKRILALCMGNHELYMRRRKPDTI
EVQQMKAQAREEKHQKQLERAQLENEKKKREIAEKEKERIEREKEELMERLKQIEEQTIK

AQKELEEQTRKALELDQERKRAKEEAERLEKERRAAEEAKSAIAKQAADQMKNQEQLAAE
LAEFTAKIALLEEAKKKKEEEATEWQHKAFAAQEDLEKTKEELKTVMSAPPPPPPPPVIP
PTENEHDEHDENNAEASAELSNEGVMNHRSEEERVTETQKNERVKKQLQALSSELAQARD
ETKKTQNDVLHAENVKAGRDKYKTLRQIRQGNTKQRIDEFEAM
>sp|P35527|K1C9_HUMAN Keratin, type I cytoskeletal 9 OS=Homo sapiens OX=9606 GN=KRT9 PE=1 SV=3
MSCRQFSSSYLSRSGGGGGGGLGSGGSIRSSYSRFSSSGGGGGGGRFSSSSGYGGGSSRV
CGRGGGGSFGYSYGGGSGGGFSASSLGGGFGGGSRGFGGASGGGYSSSGGFGGGFGGGSG
GGFGGGYGSGFGGFGGFGGGAGGGDGGILTANEKSTMQELNSRLASYLDKVQALEEANND
LENKIQDWYDKKGPAAIQKNYSPYYNTIDDLKDQIVDLTVGNNKTLLDIDNTRMTLDDFR
IKFEMEQNLRQGVDADINGLRQVLDNLTMEKSDLEMQYETLQEELMALKKNHKEEMSQLT
GQNSGDVNVEINVAPGKDLTKTLNDMRQEYEQLIAKNRKDIENQYETQITQIEHEVSSSG
QEVQSSAKEVTQLRHGVQELEIELQSQLSKKAALEKSLEDTKNRYCGQLQMIQEQISNLE
AQITDVRQEIECQNQEYSLLLSIKMRLEKEIETYHNLLEGGQEDFESSGAGKIGLGGRGG
SGGSYGRGSRGGSGGSYGGGGSGGGYGGGSGSRGGSGGSYGGGSGSGGGSGGGYGGGSGG
GHSGGSGGGHSGGSGGNYGGGSGSGGGSGGGYGGGSGSRGGSGGSHGGGSGFGGESGGSY
GGGEEASGSGGGYGGGSGKSSHS
>sp|P35555|FBN1_HUMAN Fibrillin-1 OS=Homo sapiens OX=9606 GN=FBN1 PE=1 SV=4
MRRGRLLEIALGFTVLLASYTSHGADANLEAGNVKETRASRAKRRGGGGHDALKGPNVCG
SRYNAYCCPGWKTLPGGNQCIVPICRHSCGDGFCSRPNMCTCPSGQIAPSCGSRSIQHCN
IRCMNGGSCSDDHCLCQKGYIGTHCGQPVCESGCLNGGRCVAPNRCACTYGFTGPQCERD
YRTGPCFTVISNQMCQGQLSGIVCTKTLCCATVGRAWGHPCEMCPAQPHPCRRGFIPNIR
TGACQDVDECQAIPGLCQGGNCINTVGSFECKCPAGHKLNEVSQKCEDIDECSTIPGICE
GGECTNTVSSYFCKCPPGFYTSPDGTRCIDVRPGYCYTALTNGRCSNQLPQSITKMQCCC
DAGRCWSPGVTVAPEMCPIRATEDFNKLCSVPMVIPGRPEYPPPPLGPIPPVLPVPPGFP
PGPQIPVPRPPVEYLYPSREPPRVLPVNVTDYCQLVRYLCQNGRCIPTPGSYRCECNKGF
QLDLRGECIDVDECEKNPCAGGECINNQGSYTCQCRAGYQSTLTRTECRDIDECLQNGRI
CNNGRCINTDGSFHCVCNAGFHVTRDGKNCEDMDECSIRNMCLNGMCINEDGSFKCICKP
GFQLASDGRYCKDINECETPGICMNGRCVNTDGSYRCECFPGLAVGLDGRVCVDTHMRST
CYGGYKRGQCIKPLFGAVTKSECCCASTEYAFGEPCQPCPAQNSAEYQALCSSGPGMTSA
GSDINECALDPDICPNGICENLRGTYKCICNSGYEVDSTGKNCVDINECVLNSLLCDNGQ
CRNTPGSFVCTCPKGFIYKPDLKTCEDIDECESSPCINGVCKNSPGSFICECSSESTLDP
TKTICIETIKGTCWQTVIDGRCEININGATLKSQCCSSLGAAWGSPCTLCQVDPICGKGY
SRIKGTQCEDIDECEVFPGVCKNGLCVNTRGSFKCQCPSGMTLDATGRICLDIRLETCFL
RYEDEECTLPIAGRHRMDACCCSVGAAWGTEECEECPMRNTPEYEELCPRGPGFATKEIT
NGKPFFKDINECKMIPSLCTHGKCRNTIGSFKCRCDSGFALDSEERNCTDIDECRISPDL
CGRGQCVNTPGDFECKCDEGYESGFMMMKNCMDIDECQRDPLLCRGGVCHNTEGSYRCEC
PPGHQLSPNISACIDINECELSAHLCPNGRCVNLIGKYQCACNPGYHSTPDRLFCVDIDE
CSIMNGGCETFCTNSEGSYECSCQPGFALMPDQRSCTDIDECEDNPNICDGGQCTNIPGE
YRCLCYDGFMASEDMKTCVDVNECDLNPNICLSGTCENTKGSFICHCDMGYSGKKGKTGC
TDINECEIGAHNCGKHAVCTNTAGSFKCSCSPGWIGDGIKCTDLDECSNGTHMCSQHADC
KNTMGSYRCLCKEGYTGDGFTCTDLDECSENLNLCGNGQCLNAPGGYRCECDMGFVPSAD
GKACEDIDECSLPNICVFGTCHNLPGLFRCECEIGYELDRSGGNCTDVNECLDPTTCISG
NCVNTPGSYICDCPPDFELNPTRVGCVDTRSGNCYLDIRPRGDNGDTACSNEIGVGVSKA

SCCCSLGKAWGTPCEMCPAVNTSEYKILCPGGEGFRPNPITVILEDIDECQELPGLCQGG
KCINTFGSFQCRCPTGYYLNEDTRVCDDVNECETPGICGPGTCYNTVGNYTCICPPDYMQ
VNGGNNCMDMRRSLCYRNYYADNQTCDGELLFNMTKKMCCCSYNIGRAWNKPCEQCPIPS
TDEFATLCGSQRPGFVIDIYTGLPVDIDECREIPGVCENGVCINMVGSFRCECPVGFFYN
DKLLVCEDIDECQNGPVCQRNAECINTAGSYRCDCKPGYRFTSTGQCNDRNECQEIPNIC
SHGQCIDTVGSFYCLCHTGFKTNDDQTMCLDINECERDACGNGTCRNTIGSFNCRCNHGF
ILSHNNDCIDVDECASGNGNLCRNGQCINTVGSFQCQCNEGYEVAPDGRTCVDINECLLE
PRKCAPGTCQNLDGSYRCICPPGYSLQNEKCEDIDECVEEPEICALGTCSNTEGSFKCLC
PEGFSLSSSGRRCQDLRMSYCYAKFEGGKCSSPKSRNHSKQECCCALKGEGWGDPCELCP
TEPDEAFRQICPYGSGIIVGPDDSAVDMDECKEPDVCKHGQCINTDGSYRCECPFGYILA
GNECVDTDECSVGNPCGNGTCKNVIGGFECTCEEGFEPGPMMTCEDINECAQNPLLCAFR
CVNTYGSYECKCPVGYVLREDRRMCKDEDECEEGKHDCTEKQMECKNLIGTYMCICGPGY
QRRPDGEGCVDENECQTKPGICENGRCLNTRGSYTCECNDGFTASPNQDECLDNREGYCF
TEVLQNMCQIGSSNRNPVTKSECCCDGGRGWGPHCEICPFQGTVAFKKLCPHGRGFMTNG
ADIDECKVIHDVCRNGECVNDRGSYHCICKTGYTPDITGTSCVDLNECNQAPKPCNFICK
NTEGSYQCSCPKGYILQEDGRSCKDLDECATKQHNCQFLCVNTIGGFTCKCPPGFTQHHT
SCIDNNECTSDINLCGSKGICQNTPGSFTCECQRGFSLDQTGSSCEDVDECEGNHRCQHG
CQNIIGGYRCSCPQGYLQHYQWNQCVDENECLSAHICGGASCHNTLGSYKCMCPAGFQYE
QFSGGCQDINECGSAQAPCSYGCSNTEGGYLCGCPPGYFRIGQGHCVSGMGMGRGNPEPP
VSGEMDDNSLSPEACYECKINGYPKRGRKRRSTNETDASNIEDQSETEANVSLASWDVEK
TAIFAFNISHVSNKVRILELLPALTTLTNHNRYLIESGNEDGFFKINQKEGISYLHFTKK
KPVAGTYSLQISSTPLYKKKELNQLEDKYDKDYLSGELGDNLKMKIQVLLH
>sp|P35573|GDE_HUMAN Glycogen debranching enzyme OS=Homo sapiens OX=9606 GN=AGL PE=1 SV=3
MGHSKQIRILLLNEMEKLEKTLFRLEQGYELQFRLGPTLQGKAVTVYTNYPFPGETFNRE
KFRSLDWENPTEREDDSDKYCKLNLQQSGSFQYYFLQGNEKSGGGYIVVDPILRVGADNH
VLPLDCVTLQTFLAKCLGPFDEWESRLRVAKESGYNMIHFTPLQTLGLSRSCYSLANQLE
LNPDFSRPNRKYTWNDVGQLVEKLKKEWNVICITDVVYNHTAANSKWIQEHPECAYNLVN
SPHLKPAWVLDRALWRFSCDVAEGKYKEKGIPALIENDHHMNSIRKIIWEDIFPKLKLWE
FFQVDVNKAVEQFRRLLTQENRRVTKSDPNQHLTIIQDPEYRRFGCTVDMNIALTTFIPH
DKGPAAIEECCNWFHKRMEELNSEKHRLINYHQEQAVNCLLGNVFYERLAGHGPKLGPVT
RKHPLVTRYFTFPFEEIDFSMEESMIHLPNKACFLMAHNGWVMGDDPLRNFAEPGSEVYL
RRELICWGDSVKLRYGNKPEDCPYLWAHMKKYTEITATYFQGVRLDNCHSTPLHVAEYML
DAARNLQPNLYVVAELFTGSEDLDNVFVTRLGISSLIREAMSAYNSHEEGRLVYRYGGEP
VGSFVQPCLRPLMPAIAHALFMDITHDNECPIVHRSAYDALPSTTIVSMACCASGSTRGY
DELVPHQISVVSEERFYTKWNPEALPSNTGEVNFQSGIIAARCAISKLHQELGAKGFIQV
YVDQVDEDIVAVTRHSPSIHQSVVAVSRTAFRNPKTSFYSKEVPQMCIPGKIEEVVLEAR
TIERNTKPYRKDENSINGTPDITVEIREHIQLNESKIVKQAGVATKGPNEYIQEIEFENL
SPGSVIIFRVSLDPHAQVAVGILRNHLTQFSPHFKSGSLAVDNADPILKIPFASLASRLT
LAELNQILYRCESEEKEDGGGCYDIPNWSALKYAGLQGLMSVLAEIRPKNDLGHPFCNNL
RSGDWMIDYVSNRLISRSGTIAEVGKWLQAMFFYLKQIPRYLIPCYFDAILIGAYTTLLD
TAWKQMSSFVQNGSTFVKHLSLGSVQLCGVGKFPSLPILSPALMDVPYRLNEITKEKEQC
CVSLAAGLPHFSSGIFRCWGRDTFIALRGILLITGRYVEARNIILAFAGTLRHGLIPNLL
GEGIYARYNCRDAVWWWLQCIQDYCKMVPNGLDILKCPVSRMYPTDDSAPLPAGTLDQPL

FEVIQEAMQKHMQGIQFRERNAGPQIDRNMKDEGFNITAGVDEETGFVYGGNRFNCGTWM
DKMGESDRARNRGIPATPRDGSAVEIVGLSKSAVRWLLELSKKNIFPYHEVTVKRHGKAI
KVSYDEWNRKIQDNFEKLFHVSEDPSDLNEKHPNLVHKRGIYKDSYGASSPWCDYQLRPN
FTIAMVVAPELFTTEKAWKALEIAEKKLLGPLGMKTLDPDDMVYCGIYDNALDNDNYNLA
KGFNYHQGPEWLWPIGYFLRAKLYFSRLMGPETTAKTIVLVKNVLSRHYVHLERSPWKGL
PELTNENAQYCPFSCETQAWSIATILETLYDL
>sp|P35579|MYH9_HUMAN Myosin-9 OS=Homo sapiens OX=9606 GN=MYH9 PE=1 SV=4
MAQQAADKYLYVDKNFINNPLAQADWAAKKLVWVPSDKSGFEPASLKEEVGEEAIVELVE
NGKKVKVNKDDIQKMNPPKFSKVEDMAELTCLNEASVLHNLKERYYSGLIYTYSGLFCVV
INPYKNLPIYSEEIVEMYKGKKRHEMPPHIYAITDTAYRSMMQDREDQSILCTGESGAGK
TENTKKVIQYLAYVASSHKSKKDQGELERQLLQANPILEAFGNAKTVKNDNSSRFGKFIR
INFDVNGYIVGANIETYLLEKSRAIRQAKEERTFHIFYYLLSGAGEHLKTDLLLEPYNKY
RFLSNGHVTIPGQQDKDMFQETMEAMRIMGIPEEEQMGLLRVISGVLQLGNIVFKKERNT
DQASMPDNTAAQKVSHLLGINVTDFTRGILTPRIKVGRDYVQKAQTKEQADFAIEALAKA
TYERMFRWLVLRINKALDKTKRQGASFIGILDIAGFEIFDLNSFEQLCINYTNEKLQQLF
NHTMFILEQEEYQREGIEWNFIDFGLDLQPCIDLIEKPAGPPGILALLDEECWFPKATDK
SFVEKVMQEQGTHPKFQKPKQLKDKADFCIIHYAGKVDYKADEWLMKNMDPLNDNIATLL
HQSSDKFVSELWKDVDRIIGLDQVAGMSETALPGAFKTRKGMFRTVGQLYKEQLAKLMAT
LRNTNPNFVRCIIPNHEKKAGKLDPHLVLDQLRCNGVLEGIRICRQGFPNRVVFQEFRQR
YEILTPNSIPKGFMDGKQACVLMIKALELDSNLYRIGQSKVFFRAGVLAHLEEERDLKIT
DVIIGFQACCRGYLARKAFAKRQQQLTAMKVLQRNCAAYLKLRNWQWWRLFTKVKPLLQV
SRQEEEMMAKEEELVKVREKQLAAENRLTEMETLQSQLMAEKLQLQEQLQAETELCAEAE
ELRARLTAKKQELEEICHDLEARVEEEEERCQHLQAEKKKMQQNIQELEEQLEEEESARQ
KLQLEKVTTEAKLKKLEEEQIILEDQNCKLAKEKKLLEDRIAEFTTNLTEEEEKSKSLAK
LKNKHEAMITDLEERLRREEKQRQELEKTRRKLEGDSTDLSDQIAELQAQIAELKMQLAK
KEEELQAALARVEEEAAQKNMALKKIRELESQISELQEDLESERASRNKAEKQKRDLGEE
LEALKTELEDTLDSTAAQQELRSKREQEVNILKKTLEEEAKTHEAQIQEMRQKHSQAVEE
LAEQLEQTKRVKANLEKAKQTLENERGELANEVKVLLQGKGDSEHKRKKVEAQLQELQVK
FNEGERVRTELADKVTKLQVELDNVTGLLSQSDSKSSKLTKDFSALESQLQDTQELLQEE
NRQKLSLSTKLKQVEDEKNSFREQLEEEEEAKHNLEKQIATLHAQVADMKKKMEDSVGCL
ETAEEVKRKLQKDLEGLSQRHEEKVAAYDKLEKTKTRLQQELDDLLVDLDHQRQSACNLE
KKQKKFDQLLAEEKTISAKYAEERDRAEAEAREKETKALSLARALEEAMEQKAELERLNK
QFRTEMEDLMSSKDDVGKSVHELEKSKRALEQQVEEMKTQLEELEDELQATEDAKLRLEV
NLQAMKAQFERDLQGRDEQSEEKKKQLVRQVREMEAELEDERKQRSMAVAARKKLEMDLK
DLEAHIDSANKNRDEAIKQLRKLQAQMKDCMRELDDTRASREEILAQAKENEKKLKSMEA
EMIQLQEELAAAERAKRQAQQERDELADEIANSSGKGALALEEKRRLEARIAQLEEELEE
EQGNTELINDRLKKANLQIDQINTDLNLERSHAQKNENARQQLERQNKELKVKLQEMEGT
VKSKYKASITALEAKIAQLEEQLDNETKERQAACKQVRRTEKKLKDVLLQVDDERRNAEQ
YKDQADKASTRLKQLKRQLEEAEEEAQRANASRRKLQRELEDATETADAMNREVSSLKNK
LRRGDLPFVVPRRMARKGAGDGSDEEVDGKADGAEAKPAE
>sp|P35580|MYH10_HUMAN Myosin-10 OS=Homo sapiens OX=9606 GN=MYH10 PE=1 SV=3
MAQRTGLEDPERYLFVDRAVIYNPATQADWTAKKLVWIPSERHGFEAASIKEERGDEVMV
ELAENGKKAMVNKDDIQKMNPPKFSKVEDMAELTCLNEASVLHNLKDRYYSGLIYTYSGL
FCVVINPYKNLPIYSENIIEMYRGKKRHEMPPHIYAISESAYRCMLQDREDQSILCTGES

GAGKTENTKKVIQYLAHVASSHKGRKDHNIPGELERQLLQANPILESFGNAKTVKNDNSS
RFGKFIRINFDVTGYIVGANIETYLLEKSRAVRQAKDERTFHIFYQLLSGAGEHLKSDLL
LEGFNNYRFLSNGYIPIPGQQDKDNFQETMEAMHIMGFSHEEILSMLKVVSSVLQFGNIS
FKKERNTDQASMPENTVAQKLCHLLGMNVMEFTRAILTPRIKVGRDYVQKAQTKEQADFA
VEALAKATYERLFRWLVHRINKALDRTKRQGASFIGILDIAGFEIFELNSFEQLCINYTN
EKLQQLFNHTMFILEQEEYQREGIEWNFIDFGLDLQPCIDLIERPANPPGVLALLDEECW
FPKATDKTFVEKLVQEQGSHSKFQKPRQLKDKADFCIIHYAGKVDYKADEWLMKNMDPLN
DNVATLLHQSSDRFVAELWKDVDRIVGLDQVTGMTETAFGSAYKTKKGMFRTVGQLYKES
LTKLMATLRNTNPNFVRCIIPNHEKRAGKLDPHLVLDQLRCNGVLEGIRICRQGFPNRIV
FQEFRQRYEILTPNAIPKGFMDGKQACERMIRALELDPNLYRIGQSKIFFRAGVLAHLEE
ERDLKITDIIIFFQAVCRGYLARKAFAKKQQQLSALKVLQRNCAAYLKLRHWQWWRVFTK
VKPLLQVTRQEEELQAKDEELLKVKEKQTKVEGELEEMERKHQQLLEEKNILAEQLQAET
ELFAEAEEMRARLAAKKQELEEILHDLESRVEEEEERNQILQNEKKKMQAHIQDLEEQLD
EEEGARQKLQLEKVTAEAKIKKMEEEILLLEDQNSKFIKEKKLMEDRIAECSSQLAEEEE
KAKNLAKIRNKQEVMISDLEERLKKEEKTRQELEKAKRKLDGETTDLQDQIAELQAQIDE
LKLQLAKKEEELQGALARGDDETLHKNNALKVVRELQAQIAELQEDFESEKASRNKAEKQ
KRDLSEELEALKTELEDTLDTTAAQQELRTKREQEVAELKKALEEETKNHEAQIQDMRQR
HATALEELSEQLEQAKRFKANLEKNKQGLETDNKELACEVKVLQQVKAESEHKRKKLDAQ
VQELHAKVSEGDRLRVELAEKASKLQNELDNVSTLLEEAEKKGIKFAKDAASLESQLQDT
QELLQEETRQKLNLSSRIRQLEEEKNSLQEQQEEEEEARKNLEKQVLALQSQLADTKKKV
DDDLGTIESLEEAKKKLLKDAEALSQRLEEKALAYDKLEKTKNRLQQELDDLTVDLDHQR
QVASNLEKKQKKFDQLLAEEKSISARYAEERDRAEAEAREKETKALSLARALEEALEAKE
EFERQNKQLRADMEDLMSSKDDVGKNVHELEKSKRALEQQVEEMRTQLEELEDELQATED
AKLRLEVNMQAMKAQFERDLQTRDEQNEEKKRLLIKQVRELEAELEDERKQRALAVASKK
KMEIDLKDLEAQIEAANKARDEVIKQLRKLQAQMKDYQRELEEARASRDEIFAQSKESEK
KLKSLEAEILQLQEELASSERARRHAEQERDELADEITNSASGKSALLDEKRRLEARIAQ
LEEELEEEQSNMELLNDRFRKTTLQVDTLNAELAAERSAAQKSDNARQQLERQNKELKAK
LQELEGAVKSKFKATISALEAKIGQLEEQLEQEAKERAAANKLVRRTEKKLKEIFMQVED
ERRHADQYKEQMEKANARMKQLKRQLEEAEEEATRANASRRKLQRELDDATEANEGLSRE
VSTLKNRLRRGGPISFSSSRSGRRQLHLEGASLELSDDDTESKTSDVNETQPPQSE
>sp|P35606|COPB2_HUMAN Coatomer subunit beta' OS=Homo sapiens OX=9606 GN=COPB2
PE=1 SV=2
MPLRLDIKRKLTARSDRVKSVDLHPTEPWMLASLYNGSVCVWNHETQTLVKTFEVCDLPV
RAAKFVARKNWVVTGADDMQIRVFNYNTLERVHMFEAHSDYIRCIAVHPTQPFILTSSDD
MLIKLWDWDKKWSCSQVFEGHTHYVMQIVINPKDNNQFASASLDRTIKVWQLGSSSPNFT
LEGHEKGVNCIDYYSGGDKPYLISGADDRLVKIWDYQNKTCVQTLEGHAQNVSCASFHPE
LPIIITGSEDGTVRIWHSSTYRLESTLNYGMERVWCVASLRGSNNVALGYDEGSIIVKLG
REEPAMSMDANGKIIWAKHSEVQQANLKAMGDAEIKDGERLPLAVKDMGSCEIYPQTIQH
NPNGRFVVVCGDGEYIIYTAMALRNKSFGSAQEFAWAHDSSEYAIRESNSIVKIFKNFKE
KKSFKPDFGAESIYGGFLLGVRSVNGLAFYDWDNTELIRRIEIQPKHIFWSDSGELVCIA
TEESFFILKYLSEKVLAAQETHEGVTEDGIEDAFEVLGEIQEIVKTGLWVGDCFIYTSSV
NRLNYYVGGEIVTIAHLDRTMYLLGYIPKDNRLYLGDKELNIISYSLLVSVLEYQTAVMR
RDFSMADKVLPTIPKEQRTRVAHFLEKQGFKQQALTVSTDPEHRFELALQLGELKIAYQL
AVEAESEQKWKQLAELAISKCQFGLAQECLHHAQDYGGLLLLATASGNANMVNKLAEGAE

RDGKNNVAFMSYFLQGKVDACLELLIRTGRLPEAAFLARTYLPSQVSRVVKLWRENLSKV
NQKAAESLADPTEYENLFPGLKEAFVVEEWVKETHADLWPAKQYPLVTPNEERNVMEEGK
DFQPSRSTAQQELDGKPASPTPVIVASHTANKEEKSLLELEVDLDNLELEDIDTTDINLD
EDILDD
>sp|P35609|ACTN2_HUMAN Alpha-actinin-2 OS=Homo sapiens OX=9606 GN=ACTN2 PE=1 SV=1
MNQIEPGVQYNYVYDEDEYMIQEEEWDRDLLLDPAWEKQQRKTFTAWCNSHLRKAGTQIE
NIEEDFRNGLKLMLLLEVISGERLPKPDRGKMRFHKIANVNKALDYIASKGVKLVSIGAE
EIVDGNVKMTLGMIWTIILRFAIQDISVEETSAKEGLLLWCQRKTAPYRNVNIQNFHTSW
KDGLGLCALIHRHRPDLIDYSKLNKDDPIGNINLAMEIAEKHLDIPKMLDAEDIVNTPKP
DERAIMTYVSCFYHAFAGAEQAETAANRICKVLAVNQENERLMEEYERLASELLEWIRRT
IPWLENRTPEKTMQAMQKKLEDFRDYRRKHKPPKVQEKCQLEINFNTLQTKLRISNRPAF
MPSEGKMVSDIAGAWQRLEQAEKGYEEWLLNEIRRLERLEHLAEKFRQKASTHETWAYGK
EQILLQKDYESASLTEVRALLRKHEAFESDLAAHQDRVEQIAAIAQELNELDYHDAVNVN
DRCQKICDQWDRLGTLTQKRREALERMEKLLETIDQLHLEFAKRAAPFNNWMEGAMEDLQ
DMFIVHSIEEIQSLITAHEQFKATLPEADGERQSIMAIQNEVEKVIQSYNIRISSSNPYS
TVTMDELRTKWDKVKQLVPIRDQSLQEELARQHANERLRRQFAAQANAIGPWIQNKMEEI
ARSSIQITGALEDQMNQLKQYEHNIINYKNNIDKLEGDHQLIQEALVFDNKHTNYTMEHI
RVGWELLLTTIARTINEVETQILTRDAKGITQEQMNEFRASFNHFDRRKNGLMDHEDFRA
CLISMGYDLGEAEFARIMTLVDPNGQGTVTFQSFIDFMTRETADTDTAEQVIASFRILAS
DKPYILAEELRRELPPDQAQYCIKRMPAYSGPGSVPGALDYAAFSSALYGESDL
>sp|P35611|ADDA_HUMAN Alpha-adducin OS=Homo sapiens OX=9606 GN=ADD1 PE=1 SV=2
MNGDSRAAVVTSPPPTTAPHKERYFDRVDENNPEYLRERNMAPDLRQDFNMMEQKKRVSM
ILQSPAFCEELESMIQEQFKKGKNPTGLLALQQIADFMTTNVPNVYPAAPQGGMAALNMS
LGMVTPVNDLRGSDSIAYDKGEKLLRCKLAAFYRLADLFGWSQLIYNHITTRVNSEQEHF
LIVPFGLLYSEVTASSLVKINLQGDIVDRGSTNLGVNQAGFTLHSAIYAARPDVKCVVHI
HTPAGAAVSAMKCGLLPISPEALSLGEVAYHDYHGILVDEEEKVLIQKNLGPKSKVLILR
NHGLVSVGESVEEAFYYIHNLVVACEIQVRTLASAGGPDNLVLLNPEKYKAKSRSPGSPV
GEGTGSPPKWQIGEQEFEALMRMLDNLGYRTGYPYRYPALREKSKKYSDVEVPASVTGYS
FASDGDSGTCSPLRHSFQKQQREKTRWLNSGRGDEASEEGQNGSSPKSKTKWTKEDGHRT
STSAVPNLFVPLNTNPKEVQEMRNKIREQNLQDIKTAGPQSQVLCGVVMDRSLVQGELVT
ASKAIIEKEYQPHVIVSTTGPNPFTTLTDRELEEYRREVERKQKGSEENLDEAREQKEKS
PPDQPAVPHPPPSTPIKLEEDLVPEPTTGDDSDAATFKPTLPDLSPDEPSEALGFPMLEK
EEEAHRPPSPTEAPTEASPEPAPDPAPVAEEAAPSAVEEGAAADPGSDGSPGKSPSKKKK
KFRTPSFLKKSKKKSDS
>sp|P35612|ADDB_HUMAN Beta-adducin OS=Homo sapiens OX=9606 GN=ADD2 PE=1 SV=3
MSEETVPEAASPPPPQGQPYFDRFSEDDPEYMRLRNRAADLRQDFNLMEQKKRVTMILQS
PSFREELEGLIQEQMKKGNNSSNIWALRQIADFMASTSHAVFPTSSMNVSMMTPINDLHT
ADSLNLAKGERLMRCKISSVYRLLDLYGWAQLSDTYVTLRVSKEQDHFLISPKGVSCSEV
TASSLIKVNILGEVVEKGSSCFPVDTTGFCLHSAIYAARPDVRCIIHLHTPATAAVSAMK
WGLLPVSHNALLVGDMAYYDFNGEMEQEADRINLQKCLGPTCKILVLRNHGVVALGDTVE
EAFYKIFHLQAACEIQVSALSSAGGVENLILLEQEKHRPHEVGSVQWAGSTFGPMQKSRL
GEHEFEALMRMLDNLGYRTGYTYRHPFVQEKTKHKSEVEIPATVTAFVFEEDGAPVPALR
QHAQKQQKEKTRWLNTPNTYLRVNVADEVQRSMGSPRPKTTWMKADEVEKSSSGMPIRIE

NPNQFVPLYTDPQEVLEMRNKIREQNRQDVKSAGPQSQLLASVIAEKSRSPSTESQLMSK
GDEDTKDDSEETVPNPFSQLTDQELEEYKKEVERKKLELDGEKETAPEEPGSPAKSAPAS
PVQSPAKEAETKSPLVSPSKSLEEGTKKTETSKAATTEPETTQPEGVVVNGREEEQTAEE
ILSKGLSQMTTSADTDVDTSKDKTESVTSGPMSPEGSPSKSPSKKKKKFRTPSFLKKSKK
KEKVES
>sp|P35625|TIMP3_HUMAN Metalloproteinase inhibitor 3 OS=Homo sapiens OX=9606 GN=TIMP3 PE=1 SV=2
MTPWLGLIVLLGSWSLGDWGAEACTCSPSHPQDAFCNSDIVIRAKVVGKKLVKEGPFGTL
VYTIKQMKMYRGFTKMPHVQYIHTEASESLCGLKLEVNKYQYLLTGRVYDGKMYTGLCNF
VERWDQLTLSQRKGLNYRYHLGCNCKIKSCYYLPCFVTSKNECLWTDMLSNFGYPGYQSK
HYACIRQKGGYCSWYRGWAPPDKSIINATDP
>sp|P35749|MYH11_HUMAN Myosin-11 OS=Homo sapiens OX=9606 GN=MYH11 PE=1 SV=3
MAQKGQLSDDEKFLFVDKNFINSPVAQADWAAKRLVWVPSEKQGFEAASIKEEKGDEVVV
ELVENGKKVTVGKDDIQKMNPPKFSKVEDMAELTCLNEASVLHNLRERYFSGLIYTYSGL
FCVVVNPYKHLPIYSEKIVDMYKGKKRHEMPPHIYAIADTAYRSMLQDREDQSILCTGES
GAGKTENTKKVIQYLAVVASSHKGKKDTSITGELEKQLLQANPILEAFGNAKTVKNDNSS
RFGKFIRINFDVTGYIVGANIETYLLEKSRAIRQARDERTFHIFYYMIAGAKEKMRSDLL
LEGFNNYTFLSNGFVPIPAAQDDEMFQETVEAMAIMGFSEEEQLSILKVVSSVLQLGNIV
FKKERNTDQASMPDNTAAQKVCHLMGINVTDFTRSILTPRIKVGRDVVQKAQTKEQADFA
VEALAKATYERLFRWILTRVNKALDKTHRQGASFLGILDIAGFEIFEVNSFEQLCINYTN
EKLQQLFNHTMFILEQEEYQREGIEWNFIDFGLDLQPCIELIERPNNPPGVLALLDEECW
FPKATDKSFVEKLCTEQGSHPKFQKPKQLKDKTEFSIIHYAGKVDYNASAWLTKNMDPLN
DNVTSLLNASSDKFVADLWKDVDRIVGLDQMAKMTESSLPSASKTKKGMFRTVGQLYKEQ
LGKLMTTLRNTTPNFVRCIIPNHEKRSGKLDAFLVLEQLRCNGVLEGIRICRQGFPNRIV
FQEFRQRYEILAANAIPKGFMDGKQACILMIKALELDPNLYRIGQSKIFFRTGVLAHLEE
ERDLKITDVIMAFQAMCRGYLARKAFAKRQQQLTAMKVIQRNCAAYLKLRNWQWWRLFTK
VKPLLQVTRQEEEMQAKEDELQKTKERQQKAENELKELEQKHSQLTEEKNLLQEQLQAET
ELYAEAEEMRVRLAAKKQELEEILHEMEARLEEEEDRGQQLQAERKKMAQQMLDLEEQLE
EEEAARQKLQLEKVTAEAKIKKLEDEILVMDDQNNKLSKERKLLEERISDLTTNLAEEEE
KAKNLTKLKNKHESMISELEVRLKKEEKSRQELEKLKRKLEGDASDFHEQIADLQAQIAE
LKMQLAKKEEELQAALARLDDEIAQKNNALKKIRELEGHISDLQEDLDSERAARNKAEKQ
KRDLGEELEALKTELEDTLDSTATQQELRAKREQEVTVLKKALDEETRSHEAQVQEMRQK
HAQAVEELTEQLEQFKRAKANLDKNKQTLEKENADLAGELRVLGQAKQEVEHKKKKLEAQ
VQELQSKCSDGERARAELNDKVHKLQNEVESVTGMLNEAEGKAIKLAKDVASLSSQLQDT
QELLQEETRQKLNVSTKLRQLEEERNSLQDQLDEEMEAKQNLERHISTLNIQLSDSKKKL
QDFASTVEALEEGKKRFQKEIENLTQQYEEKAAAYDKLEKTKNRLQQELDDLVVDLDNQR
QLVSNLEKKQRKFDQLLAEEKNISSKYADERDRAEAEAREKETKALSLARALEEALEAKE
ELERTNKMLKAEMEDLVSSKDDVGKNVHELEKSKRALETQMEEMKTQLEELEDELQATED
AKLRLEVNMQALKGQFERDLQARDEQNEEKRRQLQRQLHEYETELEDERKQRALAAAAKK
KLEGDLKDLELQADSAIKGREEAIKQLRKLQAQMKDFQRELEDARASRDEIFATAKENEK
KAKSLEADLMQLQEDLAAAERARKQADLEKEELAEELASSLSGRNALQDEKRRLEARIAQ
LEEELEEEQGNMEAMSDRVRKATQQAEQLSNELATERSTAQKNESARQQLERQNKELRSK
LHEMEGAVKSKFKSTIAALEAKIAQLEEQVEQEAREKQAATKSLKQKDKKLKEILLQVED
ERKMAEQYKEQAEKGNARVKQLKRQLEEAEEESQRINANRRKLQRELDEATESNEAMGRE

VNALKSKLRRGNETSFVPSRRSGGRRVIENADGSEEETDTRDADFNGTKASE
>sp|P35908|K22E_HUMAN Keratin, type II cytoskeletal 2 epidermal OS=Homo sapiens OX=9606 GN=KRT2 PE=1 SV=2
MSCQISCKSRGRGGGGGGFRGFSSGSAVVSGGSRRSTSSFSCLSRHGGGGGGFGGGGFGS
RSLVGLGGTKSISISVAGGGGGFGAAGGFGGRGGGFGGGSSFGGGSGFSGGGFGGGGFGG
GRFGGFGGPGGVGGLGGPGGFGPGGYPGGIHEVSVNQSLLQPLNVKVDPEIQNVKAQERE
QIKTLNNKFASFIDKVRFLEQQNQVLQTKWELLQQMNVGTRPINLEPIFQGYIDSLKRYL
DGLTAERTSQNSELNNMQDLVEDYKKKYEDEINKRTAAENDFVTLKKDVDNAYMIKVELQ
SKVDLLNQEIEFLKVLYDAEISQIHQSVTDTNVILSMDNSRNLDLDSIIAEVKAQYEEIA
QRSKEEAEALYHSKYEELQVTVGRHGDSLKEIKIEISELNRVIQRLQGEIAHVKKQCKNV
QDAIADAEQRGEHALKDARNKLNDLEEALQQAKEDLARLLRDYQELMNVKLALDVEIATY
RKLLEGEECRMSGDLSSNVTVSVTSSTISSNVASKAAFGGSGGRGSSSGGGYSSGSSSYG
SGGRQSGSRGGSGGGGSISGGGYGSGGGSGGRYGSGGGSKGGSISGGGYGSGGGKHSSGG
GSRGGSSSGGGYGSGGGGSSSVKGSSGEAFGSSVTFSFR
>sp|P36405|ARL3_HUMAN ADP-ribosylation factor-like protein 3 OS=Homo sapiens OX=9606 GN=ARL3 PE=1 SV=2
MGLLSILRKLKSAPDQEVRILLLGLDNAGKTTLLKQLASEDISHITPTQGFNIKSVQSQG
FKLNVWDIGGQRKIRPYWKNYFENTDILIYVIDSADRKRFEETGQELAELLEEEKLSCVP
VLIFANKQDLLTAAPASEIAEGLNLHTIRDRVWQIQSCSALTGEGVQDGMNWVCKNVNAK
KK
>sp|P36578|RL4_HUMAN Large ribosomal subunit protein uL4 OS=Homo sapiens OX=9606 GN=RPL4 PE=1 SV=5
MACARPLISVYSEKGESSGKNVTLPAVFKAPIRPDIVNFVHTNLRKNNRQPYAVSELAGH
QTSAESWGTGRAVARIPRVRGGGTHRSGQGAFGNMCRGGRMFAPTKTWRRWHRRVNTTQK
RYAICSALAASALPALVMSKGHRIEEVPELPLVVEDKVEGYKKTKEAVLLLKKLKAWNDI
KKVYASQRMRAGKGKMRNRRRIQRRGPCIIYNEDNGIIKAFRNIPGITLLNVSKLNILKL
APGGHVGRFCIWTESAFRKLDELYGTWRKAASLKSNYNLPMHKMINTDLSRILKSPEIQR
ALRAPRKKIHRRVLKKNPLKNLRIMLKLNPYAKTMRRNTILRQARNHKLRVDKAAAAAAA
LQAKSDEKAAVAGKKPVVGKKGKKAAVGVKKQKKPLVGKKAAATKKPAPEKKPAEKKPTT
EEKKPAA
>sp|P36871|PGM1_HUMAN Phosphoglucomutase-1 OS=Homo sapiens OX=9606 GN=PGM1 PE=1 SV=3
MVKIVTVKTQAYQDQKPGTSGLRKRVKVFQSSANYAENFIQSIISTVEPAQRQEATLVVG
GDGRFYMKEAIQLIARIAAANGIGRLVIGQNGILSTPAVSCIIRKIKAIGGIILTASHNP
GGPNGDFGIKFNISNGGPAPEAITDKIFQISKTIEEYAVCPDLKVDLGVLGKQQFDLENK
FKPFTVEIVDSVEAYATMLRSIFDFSALKELLSGPNRLKIRIDAMHGVVGPYVKKILCEE
LGAPANSAVNCVPLEDFGGHHPDPNLTYAADLVETMKSGEHDFGAAFDGDGDRNMILGKH
GFFVNPSDSVAVIAANIFSIPYFQQTGVRGFARSMPTSGALDRVASATKIALYETPTGWK
FFGNLMDASKLSLCGEESFGTGSDHIREKDGLWAVLAWLSILATRKQSVEDILKDHWQKY
GRNFFTRYDYEEVEAEGANKMMKDLEALMFDRSFVGKQFSANDKVYTVEKADNFEYSDPV
DGSISRNQGLRLIFTDGSRIVFRLSGTGSAGATIRLYIDSYEKDVAKINQDPQVMLAPLI
SIALKVSQLQERTGRTAPTVIT
>sp|P36955|PEDF_HUMAN Pigment epithelium-derived factor OS=Homo sapiens OX=9606 GN=SERPINF1 PE=1 SV=4

MQALVLLLCIGALLGHSSCQNPASPPEEGSPDPDSTGALVEEEDPFFKVPVNKLAAAVSN
FGYDLYRVRSSTSPTTNVLLSPLSVATALSALSLGAEQRTESIIHRALYYDLISSPDIHG
TYKELLDTVTAPQKNLKSASRIVFEKKLRIKSSFVAPLEKSYGTRPRVLTGNPRLDLQEI
NNWVQAQMKGKLARSTKEIPDEISILLLGVAHFKGQWVTKFDSRKTSLEDFYLDEERTVR
VPMMSDPKAVLRYGLDSDLSCKIAQLPLTGSMSIIFFLPLKVTQNLTLIEESLTSEFIHD
IDRELKTVQAVLTVPKLKLSYEGEVTKSLQEMKLQSLFDSPDFSKITGKPIKLTQVEHRA
GFEWNEDGAGTTPSPGLQPAHLTFPLDYHLNQPFIFVLRDTDTGALLFIGKILDPRGP
>sp|P36969|GPX4_HUMAN Phospholipid hydroperoxide glutathione peroxidase GPX4
OS=Homo sapiens OX=9606 GN=GPX4 PE=1 SV=3
MSLGRLCRLLKPALLCGALAAPGLAGTMCASRDDWRCARSMHEFSAKDIDGHMVNLDKYR
GFVCIVTNVASQUGKTEVNYTQLVDLHARYAECGLRILAFPCNQFGKQEPGSNEEIKEFA
AGYNVKFDMFSKICVNGDDAHPLWKWMKIQPKGKGILGNAIKWNFTKFLIDKNGCVVKRY
GPMEEPLVIEKDLPHYF
>sp|P37059|DHB2_HUMAN 17-beta-hydroxysteroid dehydrogenase type 2 OS=Homo sapiens
OX=9606 GN=HSD17B2 PE=1 SV=1
MSTFFSDTAWICLAVPTVLCGTVFCKYKKSSGQLWSWMVCLAGLCAVCLLILSPFWGLIL
FSVSCFLMYTYLSGQELLPVDQKAVLVTGGDCGLGHALCKYLDELGFTVFAGVLNENGPG
AEELRRTCSPRLSVLQMDITKPVQIKDAYSKVAAMLQDRGLWAVINNAGVLGFPTDGELL
LMTDYKQCMAVNFFGTVEVTKTFLPLLRKSKGRLVNVSSMGGGAPMERLASYGSSKAAVT
MFSSVMRLELSKWGIKVASIQPGGFLTNIAGTSDKWEKLEKDILDHLPAEVQEDYGQDYI
LAQRNFLLLINSLASKDFSPVLRDIQHAILAKSPFAYYTPGKGAYLWICLAHYLPIGIYD
YFAKRHFGQDKPMPRALRMPNYKKKAT
>sp|P37235|HPCL1_HUMAN Hippocalcin-like protein 1 OS=Homo sapiens OX=9606
GN=HPCAL1 PE=1 SV=3
MGKQNSKLRPEVLQDLRENTEFTDHELQEWYKGFLKDCPTGHLTVDEFKKIYANFFPYGD
ASKFAEHVFRTFDTNGDGTIDFREFIIALSVTSRGKLEQKLKWAFSMYDLDGNGYISRSE
MLEIVQAIYKMVSSVMKMPEDESTPEKRTDKIFRQMDTNNDGKLSLEEFIRGAKSDPSIV
RLLQCDPSSASQF
>sp|P37802|TAGL2_HUMAN Transgelin-2 OS=Homo sapiens OX=9606 GN=TAGLN2 PE=1 SV=3
MANRGPAYGLSREVQQKIEKQYDADLEQILIQWITTQCRKDVGRPQPGRENFQNWLKDGT
VLCELINALYPEGQAPVKKIQASTMAFKQMEQISQFLQAAERYGINTTDIFQTVDLWEGK
NMACVQRTLMNLGGLAVARDDGLFSGDPNWFPKKSKENPRNFSDNQLQEGKNVIGLQMGT
NRGASQAGMTGYGMPRQIL
>sp|P37837|TALDO_HUMAN Transaldolase OS=Homo sapiens OX=9606 GN=TALDO1 PE=1
SV=2
MSSSPVKRQRMESALDQLKQFTTVVADTGDFHAIDEYKPQDATTNPSLILAAAQMPAYQE
LVEEAIAYGRKLGGSQEDQIKNAIDKLFVLFGAEILKKIPGRVSTEVDARLSFDKDAMVA
RARRLIELYKEAGISKDRILIKLSSTWEGIQAGKELEEQHGIHCNMTLLFSFAQAVACAE
AGVTLISPFVGRILDWHVANTDKKSYEPLEDPGVKSVTKIYNYYKKFSYKTIVMGASFRN
TGEIKALAGCDFLTISPKLLGELLQDNAKLVPVLSAKAAQASDLEKIHLDEKSFRWLHNE
DQMAVEKLSDGIRKFAADAVKLERMLTERMFNAENGK
>sp|P38606|VATA_HUMAN V-type proton ATPase catalytic subunit A OS=Homo sapiens
OX=9606 GN=ATP6V1A PE=1 SV=2
MDFSKLPKILDEDKESTFGYVHGVSGPVVTACDMAGAAMYELVRVGHSELVGEIIRLEGD

MATIQVYEETSGVSVGDPVLRTGKPLSVELGPGIMGAIFDGIQRPLSDISSQTQSIYIPR
GVNVSALSRDIKWDFTPCKNLRVGSHITGGDIYGIVSENSLIKHKIMLPPRNRGTVTYIA
PPGNYDTSDVVLELEFEGVKEKFTMVQVWPVRQVRPVTEKLPANHPLLTGQRVLDALFPC
VQGGTTAIPGAFGCGKTVISQSLSKYSNSDVIIYVGCGERGNEMSEVLRDFPELTMEVDG
KVESIMKRTALVANTSNMPVAAREASIYTGITLSEYFRDMGYHVSMMADSTSRWAEALRE
ISGRLAEMPADSGYPAYLGARLASFYERAGRVKCLGNPEREGSVSIVGAVSPPGGDFSDP
VTSATLGIVQVFWGLDKKLAQRKHFPSVNWLISYSKYMRALDEYYDKHFTEFVPLRTKAK
EILQEEEDLAEIVQLVGKASLAETDKITLEVAKLIKDDFLQQNGYTPYDRFCPFYKTVGM
LSNMIAFYDMARRAVETTAQSDNKITWSIIREHMGDILYKLSSMKFKDPLKDGEAKIKSD
YAQLLEDMQNAFRSLED
>sp|P39023|RL3_HUMAN Large ribosomal subunit protein uL3 OS=Homo sapiens OX=9606
GN=RPL3 PE=1 SV=2
MSHRKFSAPRHGSLGFLPRKRSSRHRGKVKSFPKDDPSKPVHLTAFLGYKAGMTHIVREV
DRPGSKVNKKEVVEAVTIVETPPMVVVGIVGYVETPRGLRTFKTVFAEHISDECKRRFYK
NWHKSKKKAFTKYCKKWQDEDGKKQLEKDFSSMKKYCQVIRVIAHTQMRLLPLRQKKAHL
MEIQVNGGTVAEKLDWARERLEQQVPVNQVFGQDEMIDVIGVTKGKGYKGVTSRWHTKKL
PRKTHRGLRKVACIGAWHPARVAFSVARAGQKGYHHRTEINKKIYKIGQGYLIKDGKLIK
NNASTDYDLSDKSINPLGGFVHYGEVTNDFVMLKGCVVGTKKRVLTLRKSLLVQTKRRAL
EKIDLKFIDTTSKFGHGRFQTMEEKKAFMGPLKKDRIAKEEGA
>sp|P39060|COIA1_HUMAN Collagen alpha-1(XVIII) chain OS=Homo sapiens OX=9606
GN=COL18A1 PE=1 SV=5
MAPYPCGCHILLLLLFCCLAAARANLLNLNWLWFNNEDTSHAATTIPEPQGPLPVQPTADT
TTHVTPRNGSTEPATAPGSPEPPSELLEDGQDTPTSAESPDAPEENIAGVGAEILNVAKG
IRSFVQLWNDTVPTESLARAETLVLETPVGPLALAGPSSTPQENGTTLWPSRGIPSSPGA
HTTEAGTLPAPTPSPPSLGRPWAPLTGPSVPPPSSGRASLSSLLGGAPPWGSLQDPDSQG
LSPAAAAPSQQLQRPDVRLRTPLLHPLVMGSLGKHAAPSAFSSGLPGALSQVAVTTLTRD
SGAWVSHVANSVGPGLANNSALLGADPEAPAGRCLPLPPSLPVCGHLGISRFWLPNHLHH
ESGEQVRAGARAWGGLLQTHCHPFLAWFFCLLLVPPCGSVPPPAPPPCCQFCEALQDACW
SRLGGGRLPVACASLPTQEDGYCVLIGPAAERISEEVGLLQLLGDPPPQQVTQTDDPDVG
LAYVFGPDANSGQVARYHFPSLFFRDFSLLFHIRPATEGPGVLFAITDSAQAMVLLGVKL
SGVQDGHQDISLLYTEPGAGQTHTAASFRLPAFVGQWTHLALSVAGGFVALYVDCEEFQR
MPLARSSRGLELEPGAGLFVAQAGGADPDKFQGVIAELKVRRDPQVSPMHCLDEEGDDSD
GASGDSGSGLGDARELLREETGAALKPRLPAPPPVTTPPLAGGSSTEDSRSEEVEEQTTV
ASLGAQTLPGSDSVSTWDGSVRTPGGRVKEGGLKGQKGEPGVPGPPGRAGPPGSPCLPGP
PGLPCPVSPLGPAGPALQTVPGPQGPPGPPGRDGTPGRDGEPGDPGEDGKPGDTGPQGFP
GTPGDVGPKGDKGDPGVGERGPPGPQGPPGPPGPSFRHDKLTFIDMEGSGFGGDLEALRG
PRGFPGPPGPPGVPGLPGEPGRFGVNSSDVPGPAGLPGVPGREGPPGFPGLPGPPGPPGR
EGPPGRTGQKGSLGEAGAPGHKGSKGAPGPAGARGESGLAGAPGPAGPPGPPGPPGPPGP
GLPAGFDDMEGSGGPFWSTARSADGPQGPPGLPGLKGDPGVPGLPGAKGEVGADGVPGFP
GLPGREGIAGPQGPKGDRGSRGEKGDPGKDGVGQPGLPGPPGPPGPVVYVSEQDGSVLSV
PGPEGRPGFAGFPGPAGPKGNLGSKGERGSPGPKGEKGEPGSIFSPDGGALGPAQKGAKG
EPGFRGPPGPYGRPGYKGEIGFPGRPGRPGMNGLKGEKGEPGDASLGFGMRGMPGPPGPP
GPPGPPGTPVYDSNVFAESSRPGPPGLPGNQGPPGPKGAKGEVGPPGPPGQFPFDFLQLE
AEMKGEKGDRGDAGQKGERGEPGGGGFFGSSLPGPPGPPGPPGPRGYPGIPGPKGESIRG

QPGPPGPQGPPGIGYEGRQGPPGPPGPPGPPSFPGPHRQTISVPGPPGPPGPPGPPGTMG
ASSGVRLWATRQAMLGQVHEVPEGWLIFVAEQEELYVRVQNGFRKVQLEARTPLPRGTDN
EVAALQPPVVQLHDSNPYPRREHPHPTARPWRADDILASPPRLPEPQPYPGAPHHSSYVH
LRPARPTSPPAHSHRDFQPVLHLVALNSPLSGGMRGIRGADFQCFQQARAVGLAGTFRAF
LSSRLQDLYSIVRRADRAAVPIVNLKDELLFPSWEALFSGSEGPLKPGARIFSFDGKDVL
RHPTWPQKSVWHGSDPNGRRLTESYCETWRTEAPSATGQASSLLGGRLLGQSAASCHHAY
IVLCIENSFMTASK
>sp|P39210|MPV17_HUMAN Protein Mpv17 OS=Homo sapiens OX=9606 GN=MPV17 PE=1 SV=1
MALWRAYQRALAAHPWKVQVLTAGSLMGLGDIISQQLVERRGLQEHQRGRTLTMVSLGCG
FVGPVVGGWYKVLDRFIPGTTKVDALKKMLLDQGGFAPCFLGCFLPLVGALNGLSAQDNW
AKLQRDYPDALITNYYLWPAVQLANFYLVPLHYRLAVVQCVAVIWNSYLSWKAHRL
>sp|P40123|CAP2_HUMAN Adenylyl cyclase-associated protein 2 OS=Homo sapiens OX=9606 GN=CAP2 PE=1 SV=1
MANMQGLVERLERAVSRLESLSAESHRPPGNCGEVNGVIAGVAPSVEAFDKLMDSMVAEF
LKNSRILAGDVETHAEMVHSAFQAQRAFLLMASQYQQPHENDVAALLKPISEKIQEIQTF
RERNRGSNMFNHLSAVSESIPALGWIAVSPKPGPYVKEMNDAATFYTNRVLKDYKHSDLR
HVDWVKSYLNIWSELQAYIKEHHTTGLTWSKTGPVASTVSAFSVLSSGPGLPPPPPPLPP
PGPPPLFENEGKKEESSPSRSALFAQLNQGEAITKGLRHVTDDQKTYKNPSLRAQGGQTQ
SPTKSHTPSPTSPKSYPSQKHAPVLELEGKKWRVEYQEDRNDLVISETELKQVAYIFKCE
KSTIQIKGKVNSIIIDNCKKLGLVFDNVVGIVEVINSQDIQIQVMGRVPTISINKTEGCH
IYLSEDALDCEIVSAKSSEMNILIPQDGDYREFPIPEQFKTAWDGSKLITEPAEIMA
>sp|P40227|TCPZ_HUMAN T-complex protein 1 subunit zeta OS=Homo sapiens OX=9606 GN=CCT6A PE=1 SV=3
MAAVKTLNPKAEVARAQAALAVNISAARGLQDVLRTNLGPKGTMKMLVSGAGDIKLTKDG
NVLLHEMQIQHPTASLIAKVATAQDDITGDGTTSNVLIIGELLKQADLYISEGLHPRIIT
EGFEAAKEKALQFLEEVKVSREMDRETLIDVARTSLRTKVHAELADVLTEAVVDSILAIK
KQDEPIDLFMIEIMEMKHKSETDTSLIRGLVLDHGARHPDMKKRVEDAYILTCNVSLEYE
KTEVNSGFFYKSAEEREKLVKAERKFIEDRVKKIIELKRKVCGDSDKGFVVINQKGIDPF
SLDALSKEGIVALRRAKRRNMERLTLACGGVALNSFDDLSPDCLGHAGLVYEYTLGEEKF
TFIEKCNNPRSVTLLIKGPNKHTLTQIKDAVRDGLRAVKNAIDDGCVVPGAGAVEVAMAE
ALIKHKPSVKGRAQLGVQAFADALLIIPKVLAQNSGFDLQETLVKIQAEHSESGQLVGVD
LNTGEPMVAAEVGVWDNYCVKKQLLHSCTVIATNILLVDEIMRAGMSSLKG
>sp|P40429|RL13A_HUMAN Large ribosomal subunit protein uL13 OS=Homo sapiens OX=9606 GN=RPL13A PE=1 SV=2
MAEVQVLVLDGRGHLLGRLAAIVAKQVLLGRKVVVVRCEGINISGNFYRNKLKYLAFLRK
RMNTNPSRGPYHFRAPSRIFWRTVRGMLPHKTKRGQAALDRLKVFDGIPPPYDKKKRMVV
PAALKVVRLKPTRKFAYLGRLAHEVGWKYQAVTATLEEKRKEKAKIHYRKKKQLMRLRKQ
AEKNVEKKIDKYTEVLKTHGLLV
>sp|P40925|MDHC_HUMAN Malate dehydrogenase, cytoplasmic OS=Homo sapiens OX=9606 GN=MDH1 PE=1 SV=4
MSEPIRVLVTGAAGQIAYSLLYSIGNGSVFGKDQPIILVLLDITPMMGVLDGVLMELQDC
ALPLLKDVIATDKEDVAFKDLDVAILVGSMPRREGMERKDLLKANVKIFKSQGAALDKYA
KKSVKVIVVGNPANTNCLTASKSAPSIPKENFSCLTRLDHNRAKAQIALKLGVTANDVKN

VIIWGNHSSTQYPDVNHAKVKLQGKEVGVYEALKDDSWLKGEFVTTVQQRGAAVIKARKL
SSAMSAAKAICDHVRDIWFGTPEGEFVSMGVISDGNSYGVPDDLLYSFPVVIKNKTWKFV
EGLPINDFSREKMDLTAKELTEEKESAFEFLSSA
>sp|P40939|ECHA_HUMAN Trifunctional enzyme subunit alpha, mitochondrial OS=Homo sapiens OX=9606 GN=HADHA PE=1 SV=2
MVACRAIGILSRFSAFRILRSRGYICRNFTGSSALLTRTHINYGVKGDVAVVRINSPNSK
VNTLSKELHSEFSEVMNEIWASDQIRSAVLISSKPGCFIAGADINMLAACKTLQEVTQLS
QEAQRIVEKLEKSTKPIVAAINGSCLGGGLEVAISCQYRIATKDRKTVLGTPEVLLGALP
GAGGTQRLPKMVGVPAALDMMLTGRSIRADRAKKMGLVDQLVEPLGPGLKPPEERTIEYL
EEVAITFAKGLADKKISPKRDKGLVEKLTAYAMTIPFVRQQVYKKVEEKVRKQTKGLYPA
PLKIIDVVKTGIEQGSDAGYLCESQKFGELVMTKESKALMGLYHGQVLCKKNKFGAPQKD
VKHLAILGAGLMGAGIAQVSVDKGLKTILKDATLTALDRGQQQVFKGLNDKVKKKALTSF
ERDSIFSNLTGQLDYQGFEKADMVIEAVFEDLSLKHRVLKEVEAVIPDHCIFASNTSALP
ISEIAAVSKRPEKVIGMHYFSPVDKMQLLEIITTEKTSKDTSASAVAVGLKQGKVIIVVK
DGPGFYTTRCLAPMMSEVIRILQEGVDPKKLDSLTTSFGFPVGAATLVDEVGVDVAKHVA
EDLGKVFGERFGGGNPELLTQMVSKGFLGRKSGKGFYIYQEGVKRKDLNSDMDSILASLK
LPPKSEVSSDEDIQFRLVTRFVNEAVMCLQEGILATPAEGDIGAVFGLGFPPCLGGPFRF
VDLYGAQKIVDRLKKYEAAYGKQFTPCQLLADHANSPNKKFYQ
>sp|P41091|IF2G_HUMAN Eukaryotic translation initiation factor 2 subunit 3 OS=Homo sapiens OX=9606 GN=EIF2S3 PE=1 SV=3
MAGGEAGVTLGQPHLSRQDLTTLDVTKLTPLSHEVISRQATINIGTIGHVAHGKSTVVKA
ISGVHTVRFKNELERNITIKLGYANAKIYKLDDPSCPRPECYRSCGSSTPDEFPTDIPGT
KGNFKLVRHVSFVDCPGHDILMATMLNGAAVMDAALLLIAGNESCPQPQTSEHLAAIEIM
KLKHILILQNKIDLVKESQAKEQYEQILAFVQGTVAEGAPIIPISAQLKYNIEVVCEYIV
KKIPVPPRDFTSEPRLIVIRSFDVNKPGCEVDDLKGGVAGGSILKGVLKVGQEIEVRPGI
VSKDSEGKLMCKPIFSKIVSLFAEHNDLQYAAPGGLIGVGTKIDPTLCRADRMVGQVLGA
VGALPEIFTELEISYFLLRRLLGVRTEGDKKAAKVQKLSKNEVLMVNIGSLSTGGRVSAV
KADLGKIVLTNPVCTEVGEKIALSRRVEKHWRLIGWGQIRRGVTIKPTVDDD
>sp|P41222|PTGDS_HUMAN Prostaglandin-H2 D-isomerase OS=Homo sapiens OX=9606 GN=PTGDS PE=1 SV=1
MATHHTLWMGLALLGVLGDLQAAPEAQVSVQPNFQQDKFLGRWFSAGLASNSSWLREKKA
ALSMCKSVVAPATDGGLNLTSTFLRKNQCETRTMLLQPAGSLGSYSYRSPHWGSTYSVSV
VETDYDQYALLYSQGSKGPGEDFRMATLYSRTQTPRAELKEKFTAFCKAQGFTEDTIVFL
PQTDKCMTEQ
>sp|P41250|GARS_HUMAN Glycine--tRNA ligase OS=Homo sapiens OX=9606 GN=GARS1 PE=1 SV=3
MPSPRPVLLRGARAALLLLLPPRLLARPSLLLRRSLSAASCPPISLPAAASRSSMDGAGA
EEVLAPLRLAVRQQGDLVRKLKEDKAPQVDVDKAVAELKARKRVLEAKELALQPKDDIVD
RAKMEDTLKRRFFYDQAFAIYGGVSGLYDFGPVGCALKNNIIQTWRQHFIQEEQILEIDC
TMLTPEPVLKTSGHVDKFADFMVKDVKNGECFRADHLLKAHLQKLMSDKKCSVEKKSEME
SVLAQLDNYGQQELADLFVNYNVKSPITGNDLSPPVSFNLMFKTFIGPGGNMPGYLRPET
AQGIFLNFKRLLEFNQGKLPFAAAQIGNSFRNEISPRSGLIRVREFTMAEIEHFVDPSEK
DHPKFQNVADLHLYLYSAKAQVSGQSARKMRLGDAVEQGVINNTVLGYFIGRIYLYLTKV
GISPDKLRFRQHMENEMAHYACDCWDAESKTSYGWIEIVGCADRSCYDLSCHARATKVPL

VAEKPLKEPKTVNVVQFEPSKGAIGKAYKKDAKLVMEYLAICDECYITEMEMLLNEKGEF
TIETEGKTFQLTKDMINVKRFQKTLYVEEVVPNVIEPSFGLGRIMYTVFEHTFHVREGDE
QRTFFSFPAVVAPFKCSVLPLSQNQEFMPFVKELSEALTRHGVSHKVDDSSGSIGRRYAR
TDEIGVAFGVTIDFDTVNKTPHTATLRDRDSMRQIRAEISELPSIVQDLANGNITWADVE
ARYPLFEGQETGKKETIEE
>sp|P42025|ACTY_HUMAN Beta-centractin OS=Homo sapiens OX=9606 GN=ACTR1B PE=1 SV=1
MESYDIIANQPVVIDNGSGVIKAGFAGDQIPKYCFPNYVGRPKHMRVMAGALEGDLFIGP
KAEEHRGLLTIRYPMEHGVVRDWNDMERIWQYVYSKDQLQTFSEEHPVLLTEAPLNPSKN
REKAAEVFFETFNVPALFISMQAVLSLYATGRTTGVVLDSGDGVTHAVPIYEGFAMPHSI
MRVDIAGRDVSRYLRLLLRKEGVDFHTSAEFEVVRTIKERACYLSINPQKDEALETEKVQ
YTLPDGSTLDVGPARFRAPELLFQPDLVGDESEGLHEVVAFAIHKSDMDLRRTLFANIVL
SGGSTLFKGFGDRLLSEVKKLAPKDIKIKISAPQERLYSTWIGGSILASLDTFKKMWVSK
KEYEEDGSRAIHRKTF
>sp|P42766|RL35_HUMAN Large ribosomal subunit protein uL29 OS=Homo sapiens OX=9606 GN=RPL35 PE=1 SV=2
MAKIKARDLRGKKKEELLKQLDDLKVELSQLRVAKVTGGAASKLSKIRVVRKSIARVLTV
INQTQKENLRKFYKGKKYKPLDLRPKKTRAMRRRLNKHEENLKTKKQQRKERLYPLRKYA
VKA
>sp|P43007|SATT_HUMAN Neutral amino acid transporter A OS=Homo sapiens OX=9606 GN=SLC1A4 PE=1 SV=1
MEKSNETNGYLDSAQAGPAAGPGAPGTAAGRARRCAGFLRRQALVLLTVSGVLAGAGLGA
ALRGLSLSRTQVTYLAFPGEMLLRMLRMIILPLVVCSLVSGAASLDASCLGRLGGIAVAY
FGLTTLSASALAVALAFIIKPGSGAQTLQSSDLGLEDSGPPPVPKETVDSFLDLARNLFP
SNLVVAAFRTYATDYKVVTQNSSSGNVTHEKIPIGTEIEGMNILGLVLFALVLGVALKKL
GSEGEDLIRFFNSLNEATMVLVSWIMWYVPVGIMFLVGSKIVEMKDIIVLVTSLGKYIFA
SILGHVIHGGIVLPLIYFVFTRKNPFRFLLGLLAPFATAFATCSSSATLPSMMKCIEENN
GVDKRISRFILPIGATVNMDGAAIFQCVAAVFIAQLNNVELNAGQIFTILVTATASSVGA
AGVPAGGVLTIAIILEAIGLPTHDLPLILAVDWIVDRTTTVVNVEGDALGAGILHHLNQK
ATKKGEQELAEVKVEAIPNCKSEEETSPLVTHQNPAGPVASAPELESKESVL
>sp|P43034|LIS1_HUMAN Platelet-activating factor acetylhydrolase IB subunit beta OS=Homo sapiens OX=9606 GN=PAFAH1B1 PE=1 SV=2
MVLSQRQRDELNRAIADYLRSNGYEEAYSVFKKEAELDVNEELDKKYAGLLEKKWTSVIR
LQKKVMELESKLNEAKEEFTSGGPLGQKRDPKEWIPRPPEKYALSGHRSPVTRVIFHPVF
SVMVSASEDATIKVWDYETGDFERTLKGHTDSVQDISFDHSGKLLASCSADMTIKLWDFQ
GFECIRTMHGHDHNVSSVAIMPNGDHIVSASRDKTIKMWEVQTGYCVKTFTGHREWVRMV
RPNQDGTLIASCSNDQTVRVWVVATKECKAELREHEHVVECISWAPESSYSSISEATGSE
TKKSGKPGPFLLSGSRDKTIKMWDVSTGMCLMTLVGHDNWVRGVLFHSGGKFILSCADDK
TLRVWDYKNKRCMKTLNAHEHFVTSLDFHKTAPYVVTGSVDQTVKVWECR
>sp|P43243|MATR3_HUMAN Matrin-3 OS=Homo sapiens OX=9606 GN=MATR3 PE=1 SV=2
MSKSFQQSSLSRDSQGHGRDLSAAGIGLLAAATQSLSMPASLGRMNQGTARLASLMNLGM
SSSLNQQGAHSALSSASTSSHNLQSIFNIGSRGPLPLSSQHRGDADQASNILASFGLSAR
DLDELSRYPEDKITPENLPQILLQLKRRRTEEGPTLSYGRDGRSATREPPYRVPRDDWEE
KRHFRRDSFDDRGPSLNPVLDYDHGSRSQESGYYDRMDYEDDRLRDGERCRDDSFFGETS

HNYHKFDSEYERMGRGPGPLQERSLFEKKRGAPPSSNIEDFHGLLPKGYPHLCSICDLPV
HSNKEWSQHINGASHSRRCQLLLEIYPEWNPDNDTGHTMGDPFMLQQSTNPAPGILGPPP
PSFHLGGPAVGPRGNLGAGNGNLQGPRHMQKGRVETSRVVHIMDFQRGKNLRYQLLQLVE
PFGVISNHLILNKINEAFIEMATTEDAQAAVDYYTTTPALVFGKPVRVHLSQKYKRIKKP
EGKPDQKFDQKQELGRVIHLSNLPHSGYSDSAVLKLAEPYGKIKNYILMRMKSQAFIEME
TREDAMAMVDHCLKKALWFQGRCVKVDLSEKYKKLVLRIPNRGIDLLKKDKSRKRSYSPD
GKESPSDKKSKTDGSQKTESSTEGKEQEEKSGEDGEKDTKDDQTEQEPNMLLESEDELLV
DEEEAAALLESGSSVGDETDLANLGDVASDGKKEPSDKAVKKDGSASAAAKKKLKKVDKI
EELDQENEAALENGIKNEENTEPGAESSENADDPNKDTSENADGQSDENKDDYTIPDEYR
IGPYQPNVPVGIDYVIPKTGFYCKLCSLFYTNEEVAKNTHCSSLPHYQKLKKFLNKLAEE
RRQKKET
>sp|P43320|CRBB2_HUMAN Beta-crystallin B2 OS=Homo sapiens OX=9606 GN=CRYBB2 PE=1 SV=2
MASDHQTQAGKPQSLNPKIIIFEQENFQGHSHELNGPCPNLKETGVEKAGSVLVQAGPWV
GYEQANCKGEQFVFEKGEYPRWDSWTSSRRTDSLSSLRPIKVDSQEHKIILYENPNFTGK
KMEIIDDDVPSFHAHGYQEKVSSVRVQSGTWVGYQYPGYRGLQYLLEKGDYKDSSDFGAP
HPQVQSVRRIRDMQWHQRGAFHPSN
>sp|P43353|AL3B1_HUMAN Aldehyde dehydrogenase family 3 member B1 OS=Homo sapiens OX=9606 GN=ALDH3B1 PE=1 SV=1
MDPLGDTLRRLREAFHAGRTRPAEFRAAQLQGLGRFLQENKQLLHDALAQDLHKSAFESE
VSEVAISQGEVTLALRNLRAWMKDERVPKNLATQLDSAFIRKEPFGLVLIIAPWNYPLNL
TLVPLVGALAAGNCVVLKPSEISKNVEKILAEVLPQYVDQSCFAVVLGGPQETGQLLEHR
FDYIFFTGSPRVGKIVMTAAAKHLTPVTLELGGKNPCYVDDNCDPQTVANRVAWFRYFNA
GQTCVAPDYVLCSPEMQERLLPALQSTITRFYGDDPQSSPNLGRIINQKQFQRLRALLGC
GRVAIGGQSDESDRYIAPTVLVDVQEMEPVMQEEIFGPILPIVNVQSLDEAIEFINRREK
PLALYAFSNSSQVVKRVLTQTSSGGFCGNDGFMHMTLASLPFGGVGASGMGRYHGKFSFD
TFSHHRACLLRSPGMEKLNALRYPPQSPRRLRMLLVAMEAQGCSCTLL
>sp|P45880|VDAC2_HUMAN Voltage-dependent anion-selective channel protein 2 OS=Homo sapiens OX=9606 GN=VDAC2 PE=1 SV=2
MATHGQTCARPMCIPPSYADLGKAARDIFNKGFGFGLVKLDVKTKSCSGVEFSTSGSSNT
DTGKVTGTLETKYKWCEYGLTFTEKWNTDNTLGTEIAIEDQICQGLKLTFDTTFSPNTGK
KSGKIKSSYKRECINLGCDVDFDFAGPAIHGSAVFGYEGWLAGYQMTFDSAKSKLTRNNF
AVGYRTGDFQLHTNVNDGTEFGGSIYQKVCEDLDTSVNLAWTSGTNCTRFGIAAKYQLDP
TASISAKVNNSSLIGVGYTQTLRPGVKLTLSALVDGKSINAGGHKVGLALELEA
>sp|P46439|GSTM5_HUMAN Glutathione S-transferase Mu 5 OS=Homo sapiens OX=9606 GN=GSTM5 PE=1 SV=3
MPMTLGYWDIRGLAHAIRLLLEYTDSSYVEKKYTLGDAPDYDRSQWLNEKFKLGLDFPNL
PYLIDGAHKITQSNAILRYIARKHNLCGETEEEKIRVDILENQVMDNHMELVRLCYDPDF
EKLKPKYLEELPEKLKLYSEFLGKRPWFAGDKITFVDFLAYDVLDMKRIFEPKCLDAFLN
LKDFISRFEGLKKISAYMKSSQFLRGLLFGKSATWNSK
>sp|P46776|RL27A_HUMAN Large ribosomal subunit protein uL15 OS=Homo sapiens OX=9606 GN=RPL27A PE=1 SV=2
MPSRLRKTRKLRGHVSHGHGRIGKHRKHPGGRGNAGGLHHHRINFDKYHPGYFGKVGMKH
YHLKRNQSFCPTVNLDKLWTLVSEQTRVNAAKNKTGAAPIIDVVRSGYYKVLGKGKLPKQ

PVIVKAKFFSRRAEEKIKSVGGACVLVA
>sp|P46777|RL5_HUMAN Large ribosomal subunit protein uL18 OS=Homo sapiens OX=9606 GN=RPL5 PE=1 SV=3
MGFVKVVKNKAYFKRYQVKFRRRREGKTDYYARKRLVIQDKNKYNTPKYRMIVRVTNRDI
ICQIAYARIEGDMIVCAAYAHELPKYGVKVGLTNYAAAYCTGLLLARRLLNRFGMDKIYE
GQVEVTGDEYNVESIDGQPGAFTCYLDAGLARTTTGNKVFGALKGAVDGGLSIPHSTKRF
PGYDSESKEFNAEVHRKHIMGQNVADYMRYLMEEDEDAYKKQFSQYIKNSVTPDMMEEMY
KKAHAAIRENPVYEKKPKKEVKKKRWNRPKMSLAQKKDRVAQKKASFLRAQERAAES
>sp|P46778|RL21_HUMAN Large ribosomal subunit protein eL21 OS=Homo sapiens OX=9606 GN=RPL21 PE=1 SV=2
MTNTKGKRRGTRYMFSRPFRKHGVVPLATYMRIYKKGDIVDIKGMGTVQKGMPHKCYHGK
TGRVYNVTQHAVGIVVNKQVKGKILAKRINVRIEHIKHSKSRDSFLKRVKENDQKKKEAK
EKGTWVQLKRQPAPPREAHFVRTNGKEPELLEPIPYEFMA
>sp|P46781|RS9_HUMAN Small ribosomal subunit protein uS4 OS=Homo sapiens OX=9606 GN=RPS9 PE=1 SV=3
MPVARSWVCRKTYVTPRRPFEKSRLDQELKLIGEYGLRNKREVWRVKFTLAKIRKAAREL
LTLDEKDPRRLFEGNALLRRLVRIGVLDEGKMKLDYILGLKIEDFLERRLQTQVFKLGLA
KSIHHARVLIRQRHIRVRKQVVNIPSFIVRLDSQKHIDFSLRSPYGGGRPGRVKRKNAKK
GQGGAGAGDDEEED
>sp|P46926|GNPI1_HUMAN Glucosamine-6-phosphate isomerase 1 OS=Homo sapiens OX=9606 GN=GNPDA1 PE=1 SV=1
MKLIILEHYSQASEWAAKYIRNRIIQFNPGPEKYFTLGLPTGSTPLGCYKKLIEYYKNGD
LSFKYVKTFNMDEYVGLPRDHPESYHSFMWNNFFKHIDIHPENTHILDGNAVDLQAECDA
FEEKIKAAGGIELFVGGIGPDGHIAFNEPGSSLVSRTRVKTLAMDTILANARFFDGELTK
VPTMALTVGVGTVMDAREVMILITGAHKAFALYKAIEEGVNHMWTVSAFQQHPRTVFVCD
EDATLELKVKTVKYFKGLMLVHNKLVDPLYSIKEKETEKSQSSKKPYSD
>sp|P46976|GLYG_HUMAN Glycogenin-1 OS=Homo sapiens OX=9606 GN=GYG1 PE=1 SV=4
MTDQAFVTLTTNDAYAKGALVLGSSLKQHRTTRRLVVLATPQVSDSMRKVLETVFDEVIM
VDVLDSGDSAHLTLMKRPELGVTLTKLHCWSLTQYSKCVFMDADTLVLANIDDLFDREEL
SAAPDPGWPDCFNSGVFVYQPSVETYNQLLHLASEQGSFDGGDQGILNTFFSSWATTDIR
KHLPFIYNLSSISIYSYLPAFKVFGASAKVVHFLGRVKPWNYTYDPKTKSVKSEAHDPNM
THPEFLILWWNIFTTNVLPLLQQFGLVKDTCSYVNVLSDLVYTLAFSCGFCRKEDVSGAI
SHLSLGEIPAMAQPFVSSEERKERWEQGQADYMGADSFDNIKRKLDTYLQ
>sp|P47897|SYQ_HUMAN Glutamine--tRNA ligase OS=Homo sapiens OX=9606 GN=QARS1 PE=1 SV=1
MAALDSLSLFTSLGLSEQKARETLKNSALSAQLREAATQAQQTLGSTIDKATGILLYGLA
SRLRDTRRLSFLVSYIASKKIHTEPQLSAALEYVRSHPLDPIDTVDFERECGVGVIVTPE
QIEEAVEAAINRHRPQLLVERYHFNMGLLMGEARAVLKWADGKMIKNEVDMQVLHLLGPK
LEADLEKKFKVAKARLEETDRRTAKDVVENGETADQTLSLMEQLRGEALKFHKPGENYKT
PGYVVTPHTMNLLKQHLEITGGQVRTRFPPEPNGILHIGHAKAINFNFGYAKANNGICFL
RFDDTNPEKEEAKFFTAICDMVAWLGYTPYKVTYASDYFDQLYAWAVELIRRGLAYVCHQ
RGEELKGHNTLPSPWRDRPMEESLLLFEAMRKGKFSEGEATLRMKLVMEDGKMDPVAYRV
KYTPHHRTGDKWCIYPTYDYTHCLCDSIEHITHSLCTKEFQARRSSYFWLCNALDVYCPV
QWEYGRLNLHYAVVSKRKILQLVATGAVRDWDDPRLFTLTALRRRGFPPEAINNFCARVG

VTVAQTTMEPHLLEACVRDVLNDTAPRAMAVLESLRVIITNFPAAKSLDIQVPNFPADET
KGFHQVPFAPIVFIERTDFKEEPEPGFKRLAWGQPVGLRHTGYVIELQHVVKGPSGCVES
LEVTCRRADAGEKPKAFIHWVSQPLMCEVRLYERLFQHKNPEDPTEVPGGFLSDLNLASL
HVVDAALVDCSVALAKPFDKFQFERLGYFSVDPDSHQGKLVFNRTVTLKEDPGKV
>sp|P47914|RL29_HUMAN Large ribosomal subunit protein eL29 OS=Homo sapiens OX=9606 GN=RPL29 PE=1 SV=2
MAKSKNHTTHNQSRKWHRNGIKKPRSQRYESLKGVDPKFLRNMRFAKKHNKKGLKKMQAN
NAKAMSARAEAIKALVKPKEVKPKIPKGVSRKLDRLAYIAHPKLGKRARARIAKGLRLCR
PKAKAKAKAKDQTKAQAAAPASVPAQAPKRTQAPTKASE
>sp|P48029|SC6A8_HUMAN Sodium- and chloride-dependent creatine transporter 1 OS=Homo sapiens OX=9606 GN=SLC6A8 PE=1 SV=1
MAKKSAENGIYSVSGDEKKGPLIAPGPDGAPAKGDGPVGLGTPGGRLAVPPRETWTRQMD
FIMSCVGFAVGLGNVWRFPYLCYKNGGGVFLIPYVLIALVGGIPIFFLEISLGQFMKAGS
INVWNICPLFKGLGYASMVIVFYCNTYYIMVLAWGFYYLVKSFTTTLPWATCGHTWNTPD
CVEIFRHEDCANASLANLTCDQLADRRSPVIEFWENKVLRLSGGLEVPGALNWEVTLCLL
ACWVLVYFCVWKGVKSTGKIVYFTATFPYVVLVVLLVRGVLLPGALDGIIYYLKPDWSKL
GSPQVWIDAGTQIFFSYAIGLGALTALGSYNRFNNNCYKDAIILALINSGTSFFAGFVVF
SILGFMAAEQGVHISKVAESGPGLAFIAYPRAVTLMPVAPLWAALFFFMLLLLGLDSQFV
GVEGFITGLLDLLPASYYFRFQREISVALCCALCFVIDLSMVTDGGMYVFQLFDYYSASG
TTLLWQAFWECVVVAWVYGADRFMDDIACMIGYRPCPWMKWCWSFFTPLVCMGIFIFNVV
YYEPLVYNNTYVYPWWGEAMGWAFALSSMLCVPLHLLGCLLRAKGTMAERWQHLTQPIWG
LHHLEYRAQDADVRGLTTLTPVSESSKVVVVESVM
>sp|P48147|PPCE_HUMAN Prolyl endopeptidase OS=Homo sapiens OX=9606 GN=PREP PE=1 SV=2
MLSLQYPDVYRDETAVQDYHGHKICDPYAWLEDPDSEQTKAFVEAQNKITVPFLEQCPIR
GLYKERMTELYDYPKYSCHFKKGKRYFYFYNTGLQNQRVLYVQDSLEGEARVFLDPNILS
DDGTVALRGYAFSEDGEYFAYGLSASGSDWVTIKFMKVDGAKELPDVLERVKFSCMAWTH
DGKGMFYNSYPQQDGKSDGTETSTNLHQKLYYHVLGTDQSEDILCAEFPDEPKWMGGAEL
SDDGRYVLLSIREGCDPVNRLWYCDLQQESSGIAGILKWVKLIDNFEGEYDYVTNEGTVF
TFKTNRQSPNYRVINIDFRDPEESKWKVLVPEHEKDVLEWIACVRSNFLVLCYLHDVKNI
LQLHDLTTGALLKTFPLDVGSIVGYSGQKKDTEIFYQFTSFLSPGIIYHCDLTKEELEPR
VFREVTVKGIDASDYQTVQIFYPSKDGTKIPMFIVHKKGIKLDGSHPAFLYGYGGFNISI
TPNYSVSRLIFVRHMGGILAVANIRGGGEYGETWHKGGILANKQNCFDDFQCAAEYLIKE
GYTSPKRLTINGGSNGGLLVAACANQRPDLFGCVIAQVGVMDMLKFHKYTIGHAWTTDYG
CSDSKQHFEWLVKYSPLHNVKLPEADDIQYPSMLLLTADHDDRVVPLHSLKFIATLQYIV
GRSRKQSNPLLIHVDTKAGHGAGKPTAKVIEEVSDMFAFIARCLNVDWIP
>sp|P48163|MAOX_HUMAN NADP-dependent malic enzyme OS=Homo sapiens OX=9606 GN=ME1 PE=1 SV=1
MEPEAPRRRHTHQRGYLLTRNPHLNKDLAFTLEERQQLNIHGLLPPSFNSQEIQVLRVVK
NFEHLNSDFDRYLLLMDLQDRNEKLFYRVLTSDIEKFMPIVYTPTVGLACQQYSLVFRKP
RGLFITIHDRGHIASVLNAWPEDVIKAIVVTDGERILGLGDLGCNGMGIPVGKLALYTAC
GGMNPQECLPVILDVGTENEELLKDPLYIGLRQRRVRGSEYDDFLDEFMEAVSSKYGMNC
LIQFEDFANVNAFRLLNKYRNQYCTFNDDIQGTASVAVAGLLAALRITKNKLSDQTILFQ
GAGEAALGIAHLIVMALEKEGLPKEKAIKKIWLVDSKGLIVKGRASLTQEKEKFAHEHEE

MKNLEAIVQEIKPTALIGVAAIGGAFSEQILKDMAAFNERPIIFALSNPTSKAECSAEQC
YKITKGRAIFASGSPFDPVTLPNGQTLYPGQGNNSYVFPGVALGVVACGLRQITDNIFLT
TAEVIAQQVSDKHLEEGRLYPPLNTIRDVSLKIAEKIVKDAYQEKTATVYPEPQNKEAFV
RSQMYSTDYDQILPDCYSWPEEVQKIQTKVDQ
>sp|P48165|CXA8_HUMAN Gap junction alpha-8 protein OS=Homo sapiens OX=9606 GN=GJA8 PE=1 SV=3
MGDWSFLGNILEEVNEHSTVIGRVWLTVLFIFRILILGTAAEFVWGDEQSDFVCNTQQPG
CENVCYDEAFPISHIRLWVLQIIFVSTPSLMYVGHAVHYVRMEEKRKSREAEELGQQAGT
NGGPDQGSVKKSSGSKGTKKFRLEGTLLRTYICHIIFKTLFEVGFIVGHYFLYGFRILPL
YRCSRWPCPNVVDCFVSRPTEKTIFILFMLSVASVSLFLNVMELGHLGLKGIRSALKRPV
EQPLGEIPEKSLHSIAVSSIQKAKGYQLLEEEKIVSHYFPLTEVGMVETSPLPAKPFNQF
EEKISTGPLGDLSRGYQETLPSYAQVGAQEVEGEGPPAEEGAEPEVGEKKEEAERLTTEE
QEKVAVPEGEKVETPGVDKEGEKEEPQSEKVSKQGLPAEKTPSLCPELTTDDARPLSRLS
KASSRARSDDLTV
>sp|P48426|PI42A_HUMAN Phosphatidylinositol 5-phosphate 4-kinase type-2 alpha OS=Homo sapiens OX=9606 GN=PIP4K2A PE=1 SV=2
MATPGNLGSSVLASKTKTKKKHFVAQKVKLFRASDPLLSVLMWGVNHSINELSHVQIPVM
LMPDDFKAYSKIKVDNHLFNKENMPSHFKFKEYCPMVFRNLRERFGIDDQDFQNSLTRSA
PLPNDSQARSGARFHTSYDKRYIIKTITSEDVAEMHNILKKYHQYIVECHGITLLPQFLG
MYRLNVDGVEIYVIVTRNVFSHRLSVYRKYDLKGSTVAREASDKEKAKELPTLKDNDFIN
EGQKIYIDDNNKKVFLEKLKKDVEFLAQLKLMDYSLLVGIHDVERAEQEEVECEENDGEE
EGESDGTHPVGTPPDSPGNTLNSSPPLAPGEFDPNIDVYGIKCHENSPRKEVYFMAIIDI
LTHYDAKKKAAHAAKTVKHGAGAEISTVNPEQYSKRFLDFIGHILT
>sp|P48449|LSS_HUMAN Lanosterol synthase OS=Homo sapiens OX=9606 GN=LSS PE=1 SV=1
MTEGTCLRRRGGPYKTEPATDLGRWRLNCERGRQTWTYLQDERAGREQTGLEAYALGLDT
KNYFKDLPKAHTAFEGALNGMTFYVGLQAEDGHWTGDYGGPLFLLPGLLITCHVARIPLP
AGYREEIVRYLRSVQLPDGGWGLHIEDKSTVFGTALNYVSLRILGVGPDDPDLVRARNIL
HKKGGAVAIPSWGKFWLAVLNVYSWEGLNTLFPEMWLFPDWAPAHPSTLWCHCRQVYLPM
SYCYAVRLSAAEDPLVQSLRQELYVEDFASIDWLAQRNNVAPDELYTPHSWLLRVVYALL
NLYEHHHSAHLRQRAVQKLYEHIVADDRFTKSISIGPISKTINMLVRWYVDGPASTAFQE
HVSRIPDYLWMGLDGMKMQGTNGSQIWDTAFAIQALLEAGGHHRPEFSSCLQKAHEFLRL
SQVPDNPPDYQKYYRQMRKGGFSFSTLDCGWIVSDCTAEALKAVLLLQEKCPHVTEHIPR
ERLCDAVAVLLNMRNPDGGFATYETKRGGHLLELLNPSEVFGDIMIDYTYVECTSAVMQA
LKYFHKRFPEHRAAEIRETLTQGLEFCRRQQRADGSWEGSWGVCFTYGTWFGLEAFACMG
QTYRDGTACAEVSRACDFLLSRQMADGGWGEDFESCEERRYLQSAQSQIHNTCWAMMGLM
AVRHPDIEAQERGVRCLLEKQLPNGDWPQENIAGVFNKSCAISYTSYRNIFPIWALGRFS
QLYPERALAGHP
>sp|P48509|CD151_HUMAN CD151 antigen OS=Homo sapiens OX=9606 GN=CD151 PE=1 SV=3
MGEFNEKKTTCGTVCLKYLLFTYNCCFWLAGLAVMAVGIWTLALKSDYISLLASGTYLAT
AYILVVAGTVVMVTGVLGCCATFKERRNLLRLYFILLLIIFLLEIIAGILAYAYYQQLNT
ELKENLKDTMTKRYHQPGHEAVTSAVDQLQQEFHCCGSNNSQDWRDSEWIRSQEAGGRVV
PDSCCKTVVALCGQRDHASNIYKVEGGCITKLETFIQEHLRVIGAVGIGIACVQVFGMIF
TCCLYRSLKLEHY

>sp|P48637|GSHB_HUMAN Glutathione synthetase OS=Homo sapiens OX=9606 GN=GSS PE=1 SV=1
MATNWGSLLQDKQQLEELARQAVDRALAEGVLLRTSQEPTSSEVVSYAPFTLFPSLVPSA
LLEQAYAVQMDFNLLVDAVSQNAAFLEQTLSSTIKQDDFTARLFDIHKQVLKEGIAQTVF
LGLNRSDYMFQRSADGSPALKQIEINTISASFGGLASRTPAVHRHVLSVLSKTKEAGKIL
SNNPSKGLALGIAKAWELYGSPNALVLLIAQEKERNIFDQRAIENELLARNIHVIRRTFE
DISEKGSLDQDRRLFVDGQEIAVVYFRDGYMPRQYSLQNWEARLLLERSHAAKCPDIATQ
LAGTKKVQQELSRPGMLEMLLPGQPEAVARLRATFAGLYSLDVGEEGDQAIAEALAAPSR
FVLKPQREGGGNNLYGEEMVQALKQLKDSEERASYILMEKIEPEPFENCLLRPGSPARVV
QCISELGIFGVYVRQEKTLVMNKHVGHLLRTKAIEHADGGVAAGVAVLDNPYPV
>sp|P48643|TCPE_HUMAN T-complex protein 1 subunit epsilon OS=Homo sapiens OX=9606 GN=CCT5 PE=1 SV=1
MASMGTLAFDEYGRPFLIIKDQDRKSRLMGLEALKSHIMAAKAVANTMRTSLGPNGLDKM
MVDKDGDVTVTNDGATILSMMDVDHQIAKLMVELSKSQDDEIGDGTTGVVVLAGALLEEA
EQLLDRGIHPIRIADGYEQAARVAIEHLDKISDSVLVDIKDTEPLIQTAKTTLGSKVVNS
CHRQMAEIAVNAVLTVADMERRDVDFELIKVEGKVGGRLEDTKLIKGVIVDKDFSHPQMP
KKVEDAKIAILTCPFEPPKPKTKHKLDVTSVEDYKALQKYEKEKFEEMIQQIKETGANLA
ICQWGFDDEANHLLLQNNLPAVRWVGGPEIELIAIATGGRIVPRFSELTAEKLGFAGLVQ
EISFGTTKDKMLVIEQCKNSRAVTIFIRGGNKMIIEEAKRSLHDALCVIRNLIRDNRVVY
GGGAAEISCALAVSQEADKCPTLEQYAMRAFADALEVIPMALSENSGMNPIQTMTEVRAR
QVKEMNPALGIDCLHKGTNDMKQQHVIETLIGKKQQISLATQMVRMILKIDDIRKPGESE
E
>sp|P49207|RL34_HUMAN Large ribosomal subunit protein eL34 OS=Homo sapiens OX=9606 GN=RPL34 PE=1 SV=3
MVQRLTYRRRLSYNTASNKTRLSRTPGNRIVYLYTKKVGKAPKSACGVCPGRLRGVRAVR
PKVLMRLSKTKKHVSRAYGGSMCAKCVRDRIKRAFLIEEQKIVVKVLKAQAQSQKAK
>sp|P49327|FAS_HUMAN Fatty acid synthase OS=Homo sapiens OX=9606 GN=FASN PE=1 SV=3
MEEVVIAGMSGKLPESENLQEFWDNLIGGVDMVTDDDRRWKAGLYGLPRRSGKLKDLSRF
DASFFGVHPKQAHTMDPQLRLLLEVTYEAIVDGGINPDSLRGTHTGVWVGVSGSETSEAL
SRDPETLVGYSMVGCQRAMMANRLSFFFDFRGPSIALDTACSSSLMALQNAYQAIHSGQC
PAAIVGGINVLLKPNTSVQFLRLGMLSPEGTCKAFDTAGNGYCRSEGVVAVLLTKKSLAR
RVYATILNAGTNTDGFKEQGVTFPSGDIQEQLIRSLYQSAGVAPESFEYIEAHGTGTKVG
DPQELNGITRALCATRQEPLLIGSTKSNMGHPEPASGLAALAKVLLSLEHGLWAPNLHFH
SPNPEIPALLDGRLQVVDQPLPVRGGNVGINSFGFGGSNVHIILRPNTQPPPAPAPHATL
PRLLRASGRTPEAVQKLLEQGLRHSQDLAFLSMLNDIAAVPATAMPFRGYAVLGGERGGP
EVQQVPAGERPLWFICSGMGTQWRGMGLSLMRLDRFRDSILRSDEAVKPFGLKVSQLLLS
TDESTFDDIVHSFVSLTAIQIGLIDLLSCMGLRPDGIVGHSLGEVACGYADGCLSQEEAV
LAAYWRGQCIKEAHLPPGAMAAVGLSWEECKQRCPPGVVPACHNSKDTVTISGPQAPVFE
FVEQLRKEGVFAKEVRTGGMAFHSYFMEAIAPPLLQELKKVIREPKPRSARWLSTSIPEA
QWHSSLARTSSAEYNVNNLVSPVLFQEALWHVPEHAVVLEIAPHALLQAVLKRGLKPSCT
IIPLMKKDHRDNLEFFLAGIGRLHLSGIDANPNALFPPVEFPAPRGTPLISPLIKWDHSL
AWDVPAAEDFPNGSGSPSAAIYNIDTSSESPDHYLVDHTLDGRVLFPATGYLSIVWKTLA
RALGLGVEQLPVVFEDVVLHQATILPKTGTVSLEVRLLEASRAFEVSENGNLVVSGKVYQ
WDDPDPRLFDHPESPTPNPTEPLFLAQAEVYKELRLRGYDYGPHFQGILEASLEGDSGRL

LWKDNWVSFMDTMLQMSILGSAKHGLYLPTRVTAIHIDPATHRQKLYTLQDKAQVADVVV
SRWLRVTVAGGVHISGLHTESAPRRQQEQQVPILEKFCFTPHTEEGCLSERAALQEELQL
CKGLVQALQTKVTQQGLKMVVPGLDGAQIPRDPSQQELPRLLSAACRLQLNGNLQLELAQ
VLAQERPKLPEDPLLSGLLDSPALKACLDTAVENMPSLKMKVVEVLAGHGHLYSRIPGLL
SPHPLLQLSYTATDRHPQALEAAQAELQQHDVAQGQWDPADPAPSALGSADLLVCNCAVA
ALGDPASALSNMVAALREGGFLLLHTLLRGHPLGDIVAFLTSTEPQYGQGILSQDAWESL
FSRVSLRLVGLKKSFYGSTLFLCRRPTPQDSPIFLPVDDTSFRWVESLKGILADEDSSRP
VWLKAINCATSGVVGLVNCLRREPGGNRLRCVLLSNLSSTSHVPEVDPGSAELQKVLQGD
LVMNVYRDGAWGAFRHFLLEEDKPEEPTAHAFVSTLTRGDLSSIRWVCSSLRHAQPTCPG
AQLCTVYYASLNFRDIMLATGKLSPDAIPGKWTSQDSLLGMEFSGRDASGKRVMGLVPAK
GLATSVLLSPDFLWDVPSNWTLEEAASVPVVYSTAYYALVVRGRVRPGETLLIHSGSGGV
GQAAIAIALSLGCRVFTTVGSAEKRAYLQARFPQLDSTSFANSRDTSFEQHVLWHTGGKG
VDLVLNSLAEEKLQASVRCLATHGRFLEIGKFDLSQNHPLGMAIFLKNVTFHGVLLDAFF
NESSADWREVWALVQAGIRDGVVRPLKCTVFHGAQVEDAFRYMAQGKHIGKVVVQVLAEE
PEAVLKGAKPKLMSAISKTFCPAHKSYIIAGGLGGFGLELAQWLIQRGVQKLVLTSRSGI
RTGYQAKQVRRWRRQGVQVQVSTSNISSLEGARGLIAEAAQLGPVGGVFNLAVVLRDGLL
ENQTPEFFQDVCKPKYSGTLNLDRVTREACPELDYFVVFSSVSCGRGNAGQSNYGFANSA
MERICEKRRHEGLPGLAVQWGAIGDVGILVETMSTNDTIVSGTLPQRMASCLEVLDLFLN
QPHMVLSSFVLAEKAAAYRDRDSQRDLVEAVAHILGIRDLAAVNLDSSLADLGLDSLMSV
EVRQTLERELNLVLSVREVRQLTLRKLQELSSKADEASELACPTPKEDGLAQQQTQLNLR
SLLVNPEGPTLMRLNSVQSSERPLFLVHPIEGSTTVFHSLASRLSIPTYGLQCTRAAPLD
SIHSLAAYYIDCIRQVQPEGPYRVAGYSYGACVAFEMCSQLQAQQSPAPTHNSLFLFDGS
PTYVLAYTQSYRAKLTPGCEAEAETEAICFFVQQFTDMEHNRVLEALLPLKGLEERVAAA
VDLIIKSHQGLDRQELSFAARSFYYKLRAAEQYTPKAKYHGNVMLLRAKTGGAYGEDLGA
DYNLSQVCDGKVSVHVIEGDHRTLLEGSGLESIISIIHSSLAEPRVSVREG
>sp|P49368|TCPG_HUMAN T-complex protein 1 subunit gamma OS=Homo sapiens OX=9606
GN=CCT3 PE=1 SV=4
MMGHRPVLVLSQNTKRESGRKVQSGNINAAKTIADIIRTCLGPKSMMKMLLDPMGGIVMT
NDGNAILREIQVQHPAAKSMIEISRTQDEEVGDGTTSVIILAGEMLSVAEHFLEQQMHPT
VVISAYRKALDDMISTLKKISIPVDISDSDMMLNIINSSITTKAISRWSSLACNIALDAV
KMVQFEENGRKEIDIKKYARVEKIPGGIIEDSCVLRGVMINKDVTHPRMRRYIKNPRIVL
LDSSLEYKKGESQTDIEITREEDFTRILQMEEEYIQQLCEDIIQLKPDVVITEKGISDLA
QHYLMRANITAIRRVRKTDNNRIARACGARIVSRPEELREDDVGTGAGLLEIKKIGDEYF
TFITDCKDPKACTILLRGASKEILSEVERNLQDAMQVCRNVLLDPQLVPGGGASEMAVAH
ALTEKSKAMTGVEQWPYRAVAQALEVIPRTLIQNCGASTIRLLTSLRAKHTQENCETWGV
NGETGTLVDMKELGIWEPLAVKLQTYKTAVETAVLLLRIDDIVSGHKKKGDDQSRQGGAP
DAGQE
>sp|P49419|AL7A1_HUMAN Alpha-aminoadipic semialdehyde dehydrogenase OS=Homo
sapiens OX=9606 GN=ALDH7A1 PE=1 SV=5
MWRLPRALCVHAAKTSKLSGPWSRPAAFMSTLLINQPQYAWLKELGLREENEGVYNGSWG
GRGEVITTYCPANNEPIARVRQASVADYEETVKKAREAWKIWADIPAPKRGEIVRQIGDA
LREKIQVLGSLVSLEMGKILVEGVGEVQEYVDICDYAVGLSRMIGGPILPSERSGHALIE
QWNPVGLVGIITAFNFPVAVYGWNNAIAMICGNVCLWKGAPTTSLISVAVTKIIAKVLED
NKLPGAICSLTCGGADIGTAMAKDERVNLLSFTGSTQVGKQVGLMVQERFGRSLLELGGN

NAIIAFEDADLSLVVPSALFAAVGTAGQRCTTARRLFIHESIHDEVVNRLKKAYAQIRVG
NPWDPNVLYGPLHTKQAVSMFLGAVEEAKKEGGTVVYGGKVMDRPGNYVEPTIVTGLGHD
ASIAHTETFAPILYVFKFKNEEEVFAWNNEVKQGLSSSIFTKDLGRIFRWLGPKGSDCGI
VNVNIPTSGAEIGGAFGGEKHTGGGRESGSDAWKQYMRRSTCTINYSKDLPLAQGIKFQ
>sp|P49448|DHE4_HUMAN Glutamate dehydrogenase 2, mitochondrial OS=Homo sapiens OX=9606 GN=GLUD2 PE=1 SV=2
MYRYLAKALLPSRAGPAALGSAANHSAALLGRGRGQPAAASQPGLALAARRHYSELVADR
EDDPNFFKMVEGFFDRGASIVEDKLVKDLRTQESEEQKRNRVRGILRIIKPCNHVLSLSF
PIRRDDGSWEVIEGYRAQHSQHRTPCKGGIRYSTDVSVDEVKALASLMTYKCAVVDVPFG
GAKAGVKINPKNYTENELEKITRRFTMELAKKGFIGPGVDVPAPDMNTGEREMSWIADTY
ASTIGHYDINAHACVTGKPISQGGIHGRISATGRGVFHGIENFINEASYMSILGMTPGFR
DKTFVVQGFGNVGLHSMRYLHRFGAKCIAVGESDGSIWNPDGIDPKELEDFKLQHGSILG
FPKAKPYEGSILEVDCDILIPAATEKQLTKSNAPRVKAKIIAEGANGPTTPEADKIFLER
NILVIPDLYLNAGGVTVSYFEWLKNLNHVSYGRLTFKYERDSNYHLLLSVQESLERKFGK
HGGTIPIVPTAEFQDSISGASEKDIVHSALAYTMERSARQIMHTAMKYNLGLDLRTAAYV
NAIEKVFKVYSEAGVTFT
>sp|P49591|SYSC_HUMAN Serine--tRNA ligase, cytoplasmic OS=Homo sapiens OX=9606 GN=SARS1 PE=1 SV=3
MVLDLDLFRVDKGGDPALIRETQEKRFKDPGLVDQLVKADSEWRRCRFRADNLNKLKNLC
SKTIGEKMKKKEPVGDDESVPENVLSFDDLTADALANLKVSQIKKVRLLIDEAILKCDAE
RIKLEAERFENLREIGNLLHPSVPISNDEDVDNKVERIWGDCTVRKKYSHVDLVVMVDGF
EGEKGAVVAGSRGYFLKGVLVFLEQALIQYALRTLGSRGYIPIYTPFFMRKEVMQEVAQL
SQFDEELYKVIGKGSEKSDDNSYDEKYLIATSEQPIAALHRDEWLRPEDLPIKYAGLSTC
FRQEVGSHGRDTRGIFRVHQFEKIEQFVYSSPHDNKSWEMFEEMITTAEEFYQSLGIPYH
IVNIVSGSLNHAASKKLDLEAWFPGSGAFRELVSCSNCTDYQARRLRIRYGQTKKMMDKV
EFVHMLNATMCATTRTICAILENYQTEKGITVPEKLKEFMPPGLQELIPFVKPAPIEQEP
SKKQKKQHEGSKKKAAARDVTLENRLQNMEVTDA
>sp|P49720|PSB3_HUMAN Proteasome subunit beta type-3 OS=Homo sapiens OX=9606 GN=PSMB3 PE=1 SV=2
MSIMSYNGGAVMAMKGKNCVAIAADRRFGIQAQMVTTDFQKIFPMGDRLYIGLAGLATDV
QTVAQRLKFRLNLYELKEGRQIKPYTLMSMVANLLYEKRFGPYYTEPVIAGLDPKTFKPF
ICSLDLIGCPMVTDDFVVSGTCAEQMYGMCESLWEPNMDPDHLFETISQAMLNAVDRDAV
SGMGVIVHIIEKDKITTRTLKARMD
>sp|P49721|PSB2_HUMAN Proteasome subunit beta type-2 OS=Homo sapiens OX=9606 GN=PSMB2 PE=1 SV=1
MEYLIGIQGPDYVLVASDRVAASNIVQMKDDHDKMFKMSEKILLLCVGEAGDTVQFAEYI
QKNVQLYKMRNGYELSPTAAANFTRRNLADCLRSRTPYHVNLLLAGYDEHEGPALYYMDY
LAALAKAPFAAHGYGAFLTLSILDRYYTPTISRERAVELLRKCLEELQKRFILNLPTFSV
RIIDKNGIHDLDNISFPKQGS
>sp|P49842|STK19_HUMAN Inactive serine/threonine-protein kinase 19 OS=Homo sapiens OX=9606 GN=STK19 PE=2 SV=3
MSWKRHHLIPETFGVKRRRKRGPVESDPLRGEPGSARAAVSELMQLFPRGLFEDALPPIV
LRSQVYSLVPDRTVADRQLKELQEQGEIRIVQLGFDLDAHGIIFTEDYRTRVLKACDGRP
YAGAVQKFLASVLPACGDLSFQQDQMTQTFGFRDSEITHLVNAGVLTVRDAGSWWLAVPG

AGRFIKYFVKGRQAVLSMVRKAKYRELLLSELLGRRAPVVVRLGLTYHVHDLIGAQLVDC
ISTTSGTLLRLPET
>sp|P50148|GNAQ_HUMAN Guanine nucleotide-binding protein G(q) subunit alpha OS=Homo sapiens OX=9606 GN=GNAQ PE=1 SV=4
MTLESIMACCLSEEAKEARRINDEIERQLRRDKRDARRELKLLLLGTGESGKSTFIKQMR
IIHGSGYSDEDKRGFTKLVYQNIFTAMQAMIRAMDTLKIPYKYEHNKAHAQLVREVDVEK
VSAFENPYVDAIKSLWNDPGIQECYDRRREYQLSDSTKYYLNDLDRVADPAYLPTQQDVL
RVRVPTTGIIEYPFDLQSVIFRMVDVGGQRSERRKWIHCFENVTSIMFLVALSEYDQVLV
ESDNENRMEESKALFRTIITYPWFQNSSVILFLNKKDLLEEKIMYSHLVDYFPEYDGPQR
DAQAAREFILKMFVDLNPDSDKIIYSHFTCATDTENIRFVFAAVKDTILQLNLKEYNLV
>sp|P50395|GDIB_HUMAN Rab GDP dissociation inhibitor beta OS=Homo sapiens OX=9606 GN=GDI2 PE=1 SV=2
MNEEYDVIVLGTGLTECILSGIMSVNGKKVLHMDRNPYYGGESASITPLEDLYKRFKIPG
SPPESMGRGRDWNVDLIPKFLMANGQLVKMLLYTEVTRYLDFKVTEGSFVYKGGKIYKVP
STEAEALASSLMGLFEKRRFRKFLVYVANFDEKDPRTFEGIDPKKTTMRDVYKKFDLGQD
VIDFTGHALALYRTDDYLDQPCYETINRIKLYSESLARYGKSPYLYPLYGLGELPQGFAR
LSAIYGGTYMLNKPIEEIIVQNGKVIGVKSEGEIARCKQLICDPSYVKDRVEKVGQVIRV
ICILSHPIKNTNDANSCQIIIPQNQVNRKSDIYVCMISFAHNVAAQGKYIAIVSTTVETK
EPEKEIRPALELLEPIEQKFVSISDLLVPKDLGTESQIFISRTYDATTHFETTCDDIKNI
YKRMTGSEFDFEEMKRKKNDIYGED
>sp|P50453|SPB9_HUMAN Serpin B9 OS=Homo sapiens OX=9606 GN=SERPINB9 PE=1 SV=1
METLSNASGTFAIRLLKILCQDNPSHNVFCSPVSISSALAMVLLGAKGNTATQMAQALSL
NTEEDIHRAFQSLLTEVNKAGTQYLLRTANRLFGEKTCQFLSTFKESCLQFYHAELKELS
FIRAAEESRKHINTWVSKKTEGKIEELLPGSSIDAETRLVLVNAIYFKGKWNEPFDETYT
REMPFKINQEEQRPVQMMYQEATFKLAHVGEVRAQLLELPYARKELSLLVLLPDDGVELS
TVEKSLTFEKLTAWTKPDCMKSTEVEVLLPKFKLQEDYDMESVLRHLGIVDAFQQGKADL
SAMSAERDLCLSKFVHKSFVEVNEEGTEAAAASSCFVVAECCMESGPRFCADHPFLFFIR
HNRANSILFCGRFSSP
>sp|P50454|SERPH_HUMAN Serpin H1 OS=Homo sapiens OX=9606 GN=SERPINH1 PE=1 SV=2
MRSLLLLSAFCLLEAALAAEVKKPAAAAAPGTAEKLSPKAATLAERSAGLAFSLYQAMAK
DQAVENILVSPVVVASSLGLVSLGGKATTASQAKAVLSAEQLRDEEVHAGLGELLRSLSN
STARNVTWKLGSRLYGPSSVSFADDFVRSSKQHYNCEHSKINFRDKRSALQSINEWAAQT
TDGKLPEVTKDVERTDGALLVNAMFFKPHWDEKFHHKMVDNRGFMVTRSYTVGVMMMHRT
GLYNYYDDEKEKLQIVEMPLAHKLSSLIILMPHHVEPLERLEKLLTKEQLKIWMGKMQKK
AVAISLPKGVVEVTHDLQKHLAGLGLTEAIDKNKADLSRMSGKKDLYLASVFHATAFELD
TDGNPFDQDIYGREELRSPKLFYADHPFIFLVRDTQSGSLLFIGRLVRPKGDKMRDEL
>sp|P50914|RL14_HUMAN Large ribosomal subunit protein eL14 OS=Homo sapiens OX=9606 GN=RPL14 PE=1 SV=4
MVFRRFVEVGRVAYVSFGPHAGKLVAIVDVIDQNRALVDGPCTQVRRQAMPFKCMQLTDF
ILKFPHSAHQKYVRQAWQKADINTKWAATRWAKKIEARERKAKMTDFDRFKVMKAKKMRN
RIIKNEVKKLQKAALLKASPKKAPGTKGTAAAAAAAAAAKVPAKKITAASKKAPAQKVPA
QKATGQKAAPAPKAQKGQKAPAQKAPAPKASGKKA
>sp|P50990|TCPQ_HUMAN T-complex protein 1 subunit theta OS=Homo sapiens OX=9606 GN=CCT8 PE=1 SV=4

MALHVPKAPGFAQMLKEGAKHFSGLEEAVYRNIQACKELAQTTRTAYGPNGMNKMVINHL
EKLFVTNDAATILRELEVQHPAAKMIVMASHMQEQEVGDGTNFVLVFAGALLELAEELLR
IGLSVSEVIEGYEIACRKAHEILPNLVCCSAKNLRDIDEVSSLLRTSIMSKQYGNEVFLA
KLIAQACVSIFPDSGHFNVDNIRVCKILGSGISSSSVLHGMVFKKETEGDVTSVKDAKIA
VYSCPFDGMITETKGTVLIKTAEELMNFSKGEENLMDAQVKAIADTGANVVVTGGKVADM
ALHYANKYNIMLVRLNSKWDLRRLCKTVGATALPRLTPPVLEEMGHCDSVYLSEVGDTQV
VVFKHEKEDGAISTIVLRGSTDNLMDDIERAVDDGVNTFKVLTRDKRLVPGGGATEIELA
KQITSYGETCPGLEQYAIKKFAEAFEAIPRALAENSGVKANEVISKLYAVHQEGNKNVGL
DIEAEVPAVKDMLEAGILDTYLGKYWAIKLATNAAVTVLRVDQIIMAKPAGGPKPPSGKK
DWDDDQND
>sp|P50991|TCPD_HUMAN T-complex protein 1 subunit delta OS=Homo sapiens OX=9606
GN=CCT4 PE=1 SV=4
MPENVAPRSGATAGAAGGRGKGAYQDRDKPAQIRFSNISAAKAVADAIRTSLGPKGMDKM
IQDGKGDVTITNDGATILKQMQVLHPAARMLVELSKAQDIEAGDGTTSVVIIAGSLLDSC
TKLLQKGIHPTIISESFQKALEKGIEILTDMSRPVELSDRETLLNSATTSLNSKVVSQYS
SLLSPMSVNAVMKVIDPATATSVDLRDIKIVKKLGGTIDDCELVEGLVLTQKVSNSGITR
VEKAKIGLIQFCLSAPKTDMDNQIVVSDYAQMDRVLREERAYILNLVKQIKKTGCNVLLI
QKSILRDALSDLALHFLNKMKIMVIKDIEREDIEFICKTIGTKPVAHIDQFTADMLGSAE
LAEEVNLNGSGKLLKITGCASPGKTVTIVVRGSNKLVIEEAERSIHDALCVIRCLVKKRA
LIAGGGAPEIELALRLTEYSRTLSGMESYCVRAFADAMEVIPSTLAENAGLNPISTVTEL
RNRHAQGEKTAGINVRKGGISNILEELVVQPLLVSVSALTLATETVRSILKIDDVVNTR
>sp|P50993|AT1A2_HUMAN Sodium/potassium-transporting ATPase subunit alpha-2
OS=Homo sapiens OX=9606 GN=ATP1A2 PE=1 SV=1
MGRGAGREYSPAATTAENGGGKKKQKEKELDELKKEVAMDDHKLSLDELGRKYQVDLSKG
LTNQRAQDVLARDGPNALTPPPTTPEWVKFCRQLFGGFSILLWIGAILCFLAYGIQAAME
DEPSNDNLYLGVVLAAVVIVTGCFSYYQEAKSSKIMDSFKNMVPQQALVIREGEKMQINA
EEVVVGDLVEVKGGDRVPADLRIISSHGCKVDNSSLTGESEPQTRSPEFTHENPLETRNI
CFFSTNCVEGTARGIVIATGDRTVMGRIATLASGLEVGRTPIAMEIEHFIQLITGVAVFL
GVSFFVLSLILGYSWLEAVIFLIGIIVANVPEGLLATVTVCLTLTAKRMARKNCLVKNLE
AVETLGSTSTICSDKTGTLTQNRMTVAHMWFDNQIHEADTTEDQSGATFDKRSPTWTALS
RIAGLCNRAVFKAGQENISVSKRDTAGDASESALLKCIELSCGSVRKMRDRNPKVAEIPF
NSTNKYQLSIHEREDSPQSHVLVMKGAPERILDRCSTILVQGKEIPLDKEMQDAFQNAYM
ELGGLGERVLGFCQLNLPSGKFPRGFKFDTDELNFPTEKLCFVGLMSMIDPPRAAVPDAV
GKCRSAGIKVIMVTGDHPITAKAIAKGVGIISEGNETVEDIAARLNIPMSQVNPREAKAC
VVHGSDLKDMTSEQLDEILKNHTEIVFARTSPQQKLIIVEGCQRQGAIVAVTGDGVNDSP
ALKKADIGIAMGISGSDVSKQAADMILLDDNFASIVTGVEEGRLIFDNLKKSIAYTLTSN
IPEITPFLLFIIANIPLPLGTVTILCIDLGTDMVPAISLAYEAAESDIMKRQPRNSQTDK
LVNERLISMAYGQIGMIQALGGFFTYFVILAENGFLPSRLLGIRLDWDDRTMNDLEDSYG
QEWTYEQRKVVEFTCHTAFFASIVVVQWADLIICKTRRNSVFQQGMKNKILIFGLLEETA
LAAFLSYCPGMGVALRMYPLKVTWWFCAFPYSLLIFIYDEVRKLILRRYPGGWVEKETYY
>sp|P51148|RAB5C_HUMAN Ras-related protein Rab-5C OS=Homo sapiens OX=9606
GN=RAB5C PE=1 SV=2
MAGRGGAARPNGPAAGNKICQFKLVLLGESAVGKSSLVLRFVKGQFHEYQESTIGAAFLT
QTVCLDDTTVKFEIWDTAGQERYHSLAPMYYRGAQAAIVVYDITNTDTFARAKNWVKELQ

RQASPNIVIALAGNKADLASKRAVEFQEAQAYADDNSLLFMETSAKTAMNVNEIFMAIAK
KLPKNEPQNATGAPGRNRGVDLQENNPASRSQCCSN
>sp|P51149|RAB7A_HUMAN Ras-related protein Rab-7a OS=Homo sapiens OX=9606
GN=RAB7A PE=1 SV=1
MTSRKKVLLKVIILGDSGVGKTSLMNQYVNKKFSNQYKATIGADFLTKEVMVDDRLVTMQ
IWDTAGQERFQSLGVAFYRGADCCVLVFDVTAPNTFKTLDSWRDEFLIQASPRDPENFPF
VVLGNKIDLENRQVATKRAQAWCYSKNNIPYFETSAKEAINVEQAFQTIARNALKQETEV
ELYNEFPEPIKLDKNDRAKASAESCSC
>sp|P51636|CAV2_HUMAN Caveolin-2 OS=Homo sapiens OX=9606 GN=CAV2 PE=1 SV=2
MGLETEKADVQLFMDDDSYSHHSGLEYADPEKFADSDQDRDPHRLNSHLKLGFEDVIAEP
VTTHSFDKVWICSHALFEISKYVMYKFLTVFLAIPLAFIAGILFATLSCLHIWILMPFVK
TCLMVLPSVQTIWKSVTDVIIAPLCTSVGRCFSSVSLQLSQD
>sp|P51648|AL3A2_HUMAN Aldehyde dehydrogenase family 3 member A2 OS=Homo sapiens
OX=9606 GN=ALDH3A2 PE=1 SV=1
MELEVRRVRQAFLSGRSRPLRFRLQQLEALRRMVQEREKDILTAIAADLCKSEFNVYSQE
VITVLGEIDFMLENLPEWVTAKPVKKNVLTMLDEAYIQPQPLGVVLIIGAWNYPFVLTIQ
PLIGAIAAGNAVIIKPSELSENTAKILAKLLPQYLDQDLYIVINGGVEETTELLKQRFDH
IFYTGNTAVGKIVMEAAAKHLTPVTLELGGKSPCYIDKDCDLDIVCRRITWGKYMNCGQT
CIAPDYILCEASLQNQIVWKIKETVKEFYGENIKESPDYERIINLRHFKRILSLLEGQKI
AFGGETDEATRYIAPTVLTDVDPKTKVMQEEIFGPILPIVPVKNVDEAINFINEREKPLA
LYVFSHNHKLIKRMIDETSSGGVTGNDVIMHFTLNSFPFGGVGSSGMGAYHGKHSFDTFS
HQRPCLLKSLKREGANKLRYPPNSQSKVDWGKFFLLKRFNKEKLGLLLLTFLGIVAAVLV
KAEYY
>sp|P51805|PLXA3_HUMAN Plexin-A3 OS=Homo sapiens OX=9606 GN=PLXNA3 PE=1 SV=3
MPSVCLLLLLFLAVGGALGNRPFRAFVVTDTTLTHLAVHRVTGEVFVGAVNRVFKLAPNL
TELRAHVTGPVEDNARCYPPPSMRVCAHRLAPVDNINKLLLIDYAARRLVACGSIWQGIC
QFLRLDDLFKLGEPHHRKEHYLSGAQEPDSMAGVIVEQGQGPSKLFVGTAVDGKSEYFPT
LSSRKLISDEDSADMFSLVYQDEFVSSQIKIPSDTLSLYPAFDIYYIYGFVSASFVYFLT
LQLDTQQTLLDTAGEKFFTSKIVRMCAGDSEFYSYVEFPIGCSWRGVEYRLVQSAHLAKP
GLLLAQALGVPADEDVLFTIFSQGQKNRASPPRQTILCLFTLSNINAHIRRRIQSCYRGE
GTLALPWLLNKELPCINTPMQINGNFCGLVLNQPLGGLHVIEGLPLLADSTDGMASVAAY
TYRQHSVVFIGTRSGSLKKVRVDGFQDAHLYETVPVVDGSPILRDLLFSPDHRHIYLLSE
KQVSQLPVETCEQYQSCAACLGSGDPHCGWCVLRHRCCREGACLGASAPHGFAEELSKCV
QVRVRPNNVSVTSPGVQLTVTLHNVPDLSAGVSCAFEAAAENEAVLLPSGELLCPSPSLQ
ELRALTRGHGATRTVRLQLLSKETGVRFAGADFVFYNCSVLQSCMSCVGSPYPCHWCKYR
HTCTSRPHECSFQEGRVHSPEGCPEILPSGDLLIPVGVMQPLTLRAKNLPQPQSGQKNYE
CVVRVQGRQQRVPAVRFNSSSVQCQNASYSYEGDEHGDTELDFSVVWDGDFPIDKPPSFR
ALLYKCWAQRPSCGLCLKADPRFNCGWCISEHRCQLRTHCPAPKTNWMHLSQKGTRCSHP
RITQIHPLVGPKEGGTRVTIVGENLGLLSREVGLRVAGVRCNSIPAEYISAERIVCEMEE
SLVPSPPPGPVELCVGDCSADFRTQSEQVYSFVTPTFDQVSPSRGPASGGTRLTISGSSL
DAGSRVTVTVRDSECQFVRRDAKAIVCISPLSTLGPSQAPITLAIDRANISSPGLIYTYT
QDPTVTRLEPTWSIINGSTAITVSGTHLLTVQEPRVRAKYRGIETTNTCQVINDTAMLCK
APGIFLGRPQPRAQGEHPDEFGFLLDHVQTARSLNRSSFTYYPDPSFEPLGPSGVLDVKP
GSHVVLKGKNLIPAAAGSSRLNYTVLIGGQPCSLTVSDTQLLCDSPSQTGRQPVMVLVGG

LEFWLGTLHISAERALTLPAMMGLAAGGGLLLLAITAVLVAYKRKTQDADRTLKRLQLQM
DNLESRVALECKEAFAELQTDINELTNHMDEVQIPFLDYRTYAVRVLFPGIEAHPVLKEL
DTPPNVEKALRLFGQLLHSRAFVLTFIHTLEAQSSFSMRDRGTVASLTMVALQSRLDYAT
GLLKQLLADLIEKNLESKNHPKLLLRRTESVAEKMLTNWFTFLLHKFLKECAGEPLFLLY
CAIKQQMEKGPIDAITGEARYSLSEDKLIRQQIDYKTLTLHCVCPENEGSAQVPVKVLNC
DSITQAKDKLLDTVYKGIPYSQRPKAEDMDLEWRQGRMTRIILQDEDVTTKIECDWKRLN
SLAHYQVTDGSLVALVPKQVSAYNMANSFTFTRSLSRYESLLRTASSPDSLRSRAPMITP
DQETGTKLWHLVKNHDHADHREGDRGSKMVSEIYLTRLLATKGTLQKFVDDLFETVFSTA
HRGSALPLAIKYMFDFLDEQADQRQISDPDVRHTWKSNCLPLRFWVNVIKNPQFVFDIHK
NSITDACLSVVAQTFMDSCSTSEHRLGKDSPSNKLLYAKDIPNYKSWVERYYRDIAKMAS
ISDQDMDAYLVEQSRLHASDFSVLSALNELYFYVTKYRQEILTALDRDASCRKHKLRQKL
EQIISLVSSDS
>sp|P52209|6PGD_HUMAN 6-phosphogluconate dehydrogenase, decarboxylating OS=Homo sapiens OX=9606 GN=PGD PE=1 SV=3
MAQADIALIGLAVMGQNLILNMNDHGFVVCAFNRTVSKVDDFLANEAKGTKVVGAQSLKE
MVSKLKKPRRIILLVKAGQAVDDFIEKLVPLLDTGDIIIDGGNSEYRDTTRRCRDLKAKG
ILFVGSGVSGGEEGARYGPSLMPGGNKEAWPHIKTIFQGIAAKVGTGEPCCDWVGDEGAG
HFVKMVHNGIEYGDMQLICEAYHLMKDVLGMAQDEMAQAFEDWNKTELDSFLIEITANIL
KFQDTDGKHLLPKIRDSAGQKGTGKWTAISALEYGVPVTLIGEAVFARCLSSLKDERIQA
SKKLKGPQKFQFDGDKKSFLEDIRKALYASKIISYAQGFMLLRQAATEFGWTLNYGGIAL
MWRGGCIIRSVFLGKIKDAFDRNPELQNLLLDDFFKSAVENCQDSWRRAVSTGVQAGIPM
PCFTTALSFYDGYRHEMLPASLIQAQRDYFGAHTYELLAKPGQFIHTNWTGHGGTVSSSS
YNA
>sp|P52294|IMA5_HUMAN Importin subunit alpha-5 OS=Homo sapiens OX=9606 GN=KPNA1 PE=1 SV=3
MTTPGKENFRLKSYKNKSLNPDEMRRRREEEGLQLRKQKREEQLFKRRNVATAEEETEEE
VMSDGGFHEAQISNMEMAPGGVITSDMIEMIFSKSPEQQLSATQKFRKLLSKEPNPPIDE
VISTPGVVARFVEFLKRKENCTLQFESAWVLTNIASGNSLQTRIVIQAGAVPIFIELLSS
EFEDVQEQAVWALGNIAGDSTMCRDYVLDCNILPPLLQLFSKQNRLTMTRNAVWALSNLC
RGKSPPPEFAKVSPCLNVLSWLLFVSDTDVLADACWALSYLSDGPNDKIQAVIDAGVCRR
LVELLMHNDYKVVSPALRAVGNIVTGDDIQTQVILNCSALQSLLHLLSSPKESIKKEACW
TISNITAGNRAQIQTVIDANIFPALISILQTAEFRTRKEAAWAITNATSGGSAEQIKYLV
ELGCIKPLCDLLTVMDSKIVQVALNGLENILRLGEQEAKRNGTGINPYCALIEEAYGLDK
IEFLQSHENQEIYQKAFDLIEHYFGTEDEDSSIAPQVDLNQQQYIFQQCEAPMEGFQL
>sp|P52306|GDS1_HUMAN Rap1 GTPase-GDP dissociation stimulator 1 OS=Homo sapiens OX=9606 GN=RAP1GDS1 PE=1 SV=3
MDNLSDTLKKLKITAVDKTEDSLEGCLDCLLQALAQNNTETSEKIQASGILQLFASLLTP
QSSCKAKVANIIAEVAKNEFMRIPCVDAGLISPLVQLLNSKDQEVLLQTGRALGNICYDS
HEGRSAVDQAGGAQIVIDHLRSLCSITDPANEKLLTVFCGMLMNYSNENDSLQAQLINMG
VIPTLVKLLGIHCQNAALTEMCLVAFGNLAELESSKEQFASTNIAEELVKLFKKQIEHDK
REMIFEVLAPLAENDAIKLQLVEAGLVECLLEIVQQKVDSDKEDDITELKTGSDLMVLLL
LGDESMQKLFEGGKGSVFQRVLSWIPSNNHQLQLAGALAIANFARNDANCIHMVDNGIVE
KLMDLLDRHVEDGNVTVQHAALSALRNLAIPVINKAKMLSAGVTEAVLKFLKSEMPPVQF
KLLGTLRMLIDAQAEAAEQLGKNVKLVERLVEWCEAKDHAGVMGESNRLLSALIRHSKSK

DVIKTIVQSGGIKHLVTMATSEHVIMQNEALVALALIAALELGTAEKDLESAKLVQILHR
LLADERSAPEIKYNSMVLICALMGSECLHKEVQDLAFLDVVSKLRSHENKSVAQQASLTE
QRLTVES
>sp|P52565|GDIR1_HUMAN Rho GDP-dissociation inhibitor 1 OS=Homo sapiens OX=9606 GN=ARHGDIA PE=1 SV=3
MAEQEPTAEQLAQIAAENEEDEHSVNYKPPAQKSIQEIQELDKDDESLRKYKEALLGRVA
VSADPNVPNVVVTGLTLVCSSAPGPLELDLTGDLESFKKQSFVLKEGVEYRIKISFRVNR
EIVSGMKYIQHTYRKGVKIDKTDYMVGSYGPRAEEYEFLTPVEEAPKGMLARGSYSIKSR
FTDDDKTDHLSWEWNLTIKKDWKD
>sp|P52758|RIDA_HUMAN 2-iminobutanoate/2-iminopropanoate deaminase OS=Homo sapiens OX=9606 GN=RIDA PE=1 SV=1
MSSLIRRVISTAKAPGAIGPYSQAVLVDRTIYISGQIGMDPSSGQLVSGGVAEEAKQALK
NMGEILKAAGCDFTNVVKTTVLLADINDFNTVNEIYKQYFKSNFPARAAYQVAALPKGSR
IEIEAVAIQGPLTTASL
>sp|P52907|CAZA1_HUMAN F-actin-capping protein subunit alpha-1 OS=Homo sapiens OX=9606 GN=CAPZA1 PE=1 SV=3
MADFDDRVSDEEKVRIAAKFITHAPPGEFNEVFNDVRLLLNNDNLLREGAAHAFAQYNMD
QFTPVKIEGYEDQVLITEHGDLGNSRFLDPRNKISFKFDHLRKEASDPQPEEADGGLKSW
RESCDSALRAYVKDHYSNGFCTVYAKTIDGQQTIIACIESHQFQPKNFWNGRWRSEWKFT
ITPPTAQVVGVLKIQVHYYEDGNVQLVSHKDVQDSLTVSNEAQTAKEFIKIIENAENEYQ
TAISENYQTMSDTTFKALRRQLPVTRTKIDWNKILSYKIGKEMQNA
>sp|P53004|BIEA_HUMAN Biliverdin reductase A OS=Homo sapiens OX=9606 GN=BLVRA PE=1 SV=2
MNAEPERKFGVVVVGVGRAGSVRMRDLRNPHPSSAFLNLIGFVSRRELGSIDGVQQISLE
DALSSQEVEVAYICSESSSHEDYIRQFLNAGKHVLVEYPMTLSLAAAQELWELAEQKGKV
LHEEHVELLMEEFAFLKKEVVGKDLLKGSLLFTAGPLEEERFGFPAFSGISRLTWLVSLF
GELSLVSATLEERKEDQYMKMTVCLETEKKSPLSWIEEKGPGLKRNRYLSFHFKSGSLEN
VPNVGVNKNIFLKDQNIFVQKLLGQFSEKELAAEKKRILHCLGLAEEIQKYCCSRK
>sp|P53007|TXTP_HUMAN Tricarboxylate transport protein, mitochondrial OS=Homo sapiens OX=9606 GN=SLC25A1 PE=1 SV=2
MPAPRAPRALAAAAPASGKAKLTHPGKAILAGGLAGGIEICITFPTEYVKTQLQLDERSH
PPRYRGIGDCVRQTVRSHGVLGLYRGLSSLLYGSIPKAAVRFGMFEFLSNHMRDAQGRLD
STRGLLCGLGAGVAEAVVVVCPMETIKVKFIHDQTSPNPKYRGFFHGVREIVREQGLKGT
YQGLTATVLKQGSNQAIRFFVMTSLRNWYRGDNPNKPMNPLITGVFGAIAGAASVFGNTP
LDVIKTRMQGLEAHKYRNTWDCGLQILKKEGLKAFYKGTVPRLGRVCLDVAIVFVIYDEV
VKLLNKVWKTD
>sp|P53420|CO4A4_HUMAN Collagen alpha-4(IV) chain OS=Homo sapiens OX=9606 GN=COL4A4 PE=1 SV=3
MWSLHIVLMRCSFRLTKSLATGPWSLILILFSVQYVYGSGKKYIGPCGGRDCSVCHCVPE
KGSRGPPGPPGPQGPIGPLGAPGPIGLSGEKGMRGDRGPPGAAGDKGDKGPTGVPGFPGL
DGIPGHPGPPGPRGKPGMSGHNGSRGDPGFPGGRGALGPGGPLGHPGEKGEKGNSVFILG
AVKGIQGDRGDPGLPGLPGSWGAGGPAGPTGYPGEPGLVGPPGQPGRPGLKGNPGVGVKG
QMGDPGEVGQQGSPGPTLLVEPPDFCLYKGEKGIKGIPGMVGLPGPPGRKGESGIGAKGE
KGIPGFPGPRGDPGSYGSPGFPGLKGELGLVGDPGLFGLIGPKGDPGNRGHPGPPGVLVT

PPLPLKGPPGDPGFPGRYGETGDVGPPGPPGLLGRPGEACAGMIGPPGPQGFPGLPGLPG
EAGIPGRPDSAPGKPGKPGSPGLPGAPGLQGLPGSSVIYCSVGNPGPQGIKGKVGPPGGR
GPKGEKGNEGLCACEPGPMGPPGPPGLPGRQGSKGDLGLPGWLGTKGDPGPPGAEGPPGL
PGKHGASGPPGNKGAKGDMVVSRVKGHKGERGPDGPPGFPGQPGSHGRDGHAGEKGDPGP
PGDHEDATPGGKGFPGPLGPPGKAGPVGPPGLGFPGPPGERGHPGVPGHPGVRGPDGLKG
QKGDTISCNVTYPGRHGPPGFDGPPGPKGFPGPQGAPGLSGSDGHKGRPGTPGTAEIPGP
PGFRGDMGDPGFGGEKGSSPVGPPGPPGSPGVNGQKGIPGDPAFGHLGPPGKRGLSGVPG
IKGPRGDPGCPGAEGPAGIPGFLGLKGPKGREGHAGFPGVPGPPGHSCERGAPGIPGQPG
LPGYPGSPGAPGGKGQPGDVGPPGPAGMKGLPGLPGRPGAHGPPGLPGIPGPFGDDGLPG
PPGPKGPRGLPGFPGFPGERGKPGAEGCPGAKGEPGEKGMSGLPGDRGLRGAKGAIGPPG
DEGEMAIISQKGTPGEPGPPGDDGFPGERGDKGTPGMQGRRGEPGRYGPPGFHRGEPGEK
GQPGPPGPPGPPGSTGLRGFIGFPGLPGDQGEPGSPGPPGFSGIDGARGPKGNKGDPASH
FGPPGPKGEPGSPGCPGHFGASGEQGLPGIQGPRGSPGRPGPPGSSGPPGCPGDHGMPGL
RGQPGEMGDPGPRGLQGDPGIPGPPGIKGPSGSPGLNGLHGLKGQKGTKGASGLHDVGPP
GPVGIPGLKGERGDPGSPGISPPGPRGKKGPPGPPGSSGPPGPAGATGRAPKDIPDPGPP
GDQGPPGPDGPRGAPGPPGLPGSVDLLRGEPGDCGLPGPPGPPGPPGPPGYKGFPGCDGK
DGQKGPVGFPGPQGPHGFPGPPGEKGLPGPPGRKGPTGLPGPRGEPGPPADVDDCPRIPG
LPGAPGMRGPEGAMGLPGMRGPSGPGCKGEPGLDGRRGVDGVPGSPGPPGRKGDTGEDGY
PGGPGPPGPIGDPGPKGFGPGYLGGFLLVLHSQTDQEPTCPLGMPRLWTGYSLLYLEGQE
KAHNQDLGLAGSCLPVFSTLPFAYCNIHQVCHYAQRNDRSYWLASAAPLPMMPLSEEAIR
PYVSRCAVCEAPAQAVAVHSQDQSIPPCPQTWRSLWIGYSFLMHTGAGDQGGGQALMSPG
SCLEDFRAAPFLECQGRQGTCHFFANKYSFWLTTVKADLQFSSAPAPDTLKESQAQRQKI
SRCQVCVKYS
>sp|P53621|COPA_HUMAN Coatomer subunit alpha OS=Homo sapiens OX=9606 GN=COPA
PE=1 SV=2
MLTKFETKSARVKGLSFHPKRPWILTSLHNGVIQLWDYRMCTLIDKFDEHDGPVRGIDFH
KQQPLFVSGGDDYKIKVWNYKLRRCLFTLLGHLDYIRTTFFHHEYPWILSASDDQTIRVW
NWQSRTCVCVLTGHNHYVMCAQFHPTEDLVVSASLDQTVRVWDISGLRKKNLSPGAVESD
VRGITGVDLFGTTDAVVKHVLEGHDRGVNWAAFHPTMPLIVSGADDRQVKIWRMNESKAW
EVDTCRGHYNNVSCAVFHPRQELILSNSEDKSIRVWDMSKRTGVQTFRRDHDRFWVLAAH
PNLNLFAAGHDGGMIVFKLERERPAYAVHGNMLHYVKDRFLRQLDFNSSKDVAVMQLRSG
SKFPVFNMSYNPAENAVLLCTRASNLENSTYDLYTIPKDADSQNPDAPEGKRSSGLTAVW
VARNRFAVLDRMHSLLIKNLKNEITKKVQVPNCDEIFYAGTGNLLLRDADSITLFDVQQK
RTLASVKISKVKYVIWSADMSHVALLAKHAIVICNRKLDALCNIHENIRVKSGAWDESGV
FIYTTSNHIKYAVTTGDHGIIRTLDLPIYVTRVKGNNVYCLDRECRPRVLTIDPTEFKFK
LALINRKYDEVLHMVRNAKLVGQSIIAYLQKKGYPEVALHFVKDEKTRFSLALECGNIEI
ALEAAKALDDKNCWEKLGEVALLQGNHQIVEMCYQRTKNFDKLSFLYLITGNLEKLRKMM
KIAEIRKDMSGHYQNALYLGDVSERVRILKNCGQKSLAYLTAATHGLDEEAESLKETFDP
EKETIPDIDPNAKLLQPPAPIMPLDTNWPLLTVSKGFFEGTIASKGKGGALAADIDIDTV
GTEGWGEDAELQLDEDGFVEATEGLGDDALGKGQEEGGGWDVEEDLELPPELDISPGAAG
GAEDGFFVPPTKGTSPTQIWCNNSQLPVDHILAGSFETAMRLLHDQVGVIQFGPYKQLFL
QTYARGRTTYQALPCLPSMYGYPNRNWKDAGLKNGVPAVGLKLNDLIQRLQLCYQLTTVG
KFEEAVEKFRSILLSVPLLVVDNKQEIAEAQQLITICREYIVGLSVETERKKLPKETLEQ
QKRICEMAAYFTHSNLQPVHMILVLRTALNLFFKLKNFKTAATFARRLLELGPKPEVAQQ

TRKILSACEKNPTDAYQLNYDMHNPFDICAASYRPIYRGKPVEKCPLSGACYSPEFKGQI
CRVTTVTEIGKDVIGLRISPLQFR
>sp|P53672|CRBA2_HUMAN Beta-crystallin A2 OS=Homo sapiens OX=9606 GN=CRYBA2 PE=1
SV=3
MSSAPAPGPAPASLTLWDEEDFQGRRCRLLSDCANVCERGGLPRVRSVKVENGVWVAFEY
PDFQGQQFILEKGDYPRWSAWSGSSSHNSNQLLSFRPVLCANHNDSRVTLFEGDNFQGCK
FDLVDDYPSLPSMGWASKDVGSLKVSSGAWVAYQYPGYRGYQYVLERDRHSGEFCTYGEL
GTQAHTGQLQSIRRVQH
>sp|P53673|CRBA4_HUMAN Beta-crystallin A4 OS=Homo sapiens OX=9606 GN=CRYBA4 PE=1
SV=3
MTLQCTKSAGPWKMVVWDEDGFQGRRHEFTAECPSVLELGFETVRSLKVLSGAWVGFEHA
GFQGQQYILERGEYPSWDAWGGNTAYPAERLTSFRPAACANHRDSRLTIFEQENFLGKKG
ELSDDYPSLQAMGWEGNEVGSFHVHSGAWVCSQFPGYRGFQYVLECDHHSGDYKHFREWG
SHAPTFQVQSIRRIQQ
>sp|P53674|CRBB1_HUMAN Beta-crystallin B1 OS=Homo sapiens OX=9606 GN=CRYBB1 PE=1
SV=2
MSQAAKASASATVAVNPGPDTKGKGAPPAGTSPSPGTTLAPTTVPITSAKAAELPPGNYR
LVVFELENFQGRRAEFSGECSNLADRGFDRVRSIIVSAGPWVAFEQSNFRGEMFILEKGE
YPRWNTWSSSYRSDRLMSFRPIKMDAQEHKISLFEGANFKGNTIEIQGDDAPSLWVYGFS
DRVGSVKVSSGTWVGYQYPGYRGYQYLLEPGDFRHWNEWGAFQPQMQSLRRLRDKQWHLE
GSFPVLATEPPK
>sp|P54136|SYRC_HUMAN Arginine--tRNA ligase, cytoplasmic OS=Homo sapiens OX=9606
GN=RARS1 PE=1 SV=2
MDVLVSECSARLLQQEEEIKSLTAEIDRLKNCGCLGASPNLEQLQEENLKLKYRLNILRK
SLQAERNKPTKNMINIISRLQEVFGHAIKAAYPDLENPPLLVTPSQQAKFGDYQCNSAMG
ISQMLKTKEQKVNPREIAENITKHLPDNECIEKVEIAGPGFINVHLRKDFVSEQLTSLLV
NGVQLPALGENKKVIVDFSSPNIAKEMHVGHLRSTIIGESISRLFEFAGYDVLRLNHVGD
WGTQFGMLIAHLQDKFPDYLTVSPPIGDLQVFYKESKKRFDTEEEFKKRAYQCVVLLQGK
NPDITKAWKLICDVSRQELNKIYDALDVSLIERGESFYQDRMNDIVKEFEDRGFVQVDDG
RKIVFVPGCSIPLTIVKSDGGYTYDTSDLAAIKQRLFEEKADMIIYVVDNGQSVHFQTIF
AAAQMIGWYDPKVTRVFHAGFGVVLGEDKKKFKTRSGETVRLMDLLGEGLKRSMDKLKEK
ERDKVLTAEELNAAQTSVAYGCIKYADLSHNRLNDYIFSFDKMLDDRGNTAAYLLYAFTR
IRSIARLANIDEEMLQKAARETKILLDHEKEWKLGRCILRFPEILQKILDDLFLHTLCDY
IYELATAFTEFYDSCYCVEKDRQTGKILKVNMWRMLLCEAVAAVMAKGFDILGIKPVQRM
>sp|P54289|CA2D1_HUMAN Voltage-dependent calcium channel subunit alpha-2/delta-1
OS=Homo sapiens OX=9606 GN=CACNA2D1 PE=1 SV=3
MAAGCLLALTLTLFQSLLIGPSSEEPFPSAVTIKSWVDKMQEDLVTLAKTASGVNQLVDI
YEKYQDLYTVEPNNARQLVEIAARDIEKLLSNRSKALVRLALEAEKVQAAHQWREDFASN
EVVYYNAKDDLDPEKNDSEPGSQRIKPVFIEDANFGRQISYQHAAVHIPTDIYEGSTIVL
NELNWTSALDEVFKKNREEDPSLLWQVFGSATGLARYYPASPWVDNSRTPNKIDLYDVRR
RPWYIQGAASPKDMLILVDVSGSVSGLTLKLIRTSVSEMLETLSDDDFVNVASFNSNAQD
VSCFQHLVQANVRNKKVLKDAVNNITAKGITDYKKGFSFAFEQLLNYNVSRANCNKIIML
FTDGGEERAQEIFNKYNKDKKVRVFTFSVGQHNYDRGPIQWMACENKGYYYEIPSIGAIR
INTQEYLDVLGRPMVLAGDKAKQVQWTNVYLDALELGLVITGTLPVFNITGQFENKTNLK

NQLILGVMGVDVSLEDIKRLTPRFTLCPNGYYFAIDPNGYVLLHPNLQPKPIGVGIPTIN
LRKRRPNIQNPKSQEPVTLDFLDAELENDIKVEIRNKMIDGESGEKTFRTLVKSQDERYI
DKGNRTYTWTPVNGTDYSLALVLPTYSFYYIKAKLEETITQARYSETLKPDNFEESGYTF
IAPRDYCNDLKISDNNTEFLLNFNEFIDRKTPNNPSCNADLINRVLLDAGFTNELVQNYW
SKQKNIKGVKARFVVTDGGITRVYPKEAGENWQENPETYEDSFYKRSLDNDNYVFTAPYF
NKSGPGAYESGIMVSKAVEIYIQGKLLKPAVVGIKIDVNSWIENFTKTSIRDPCAGPVCD
CKRNSDVMDCVILDDGGFLLMANHDDYTNQIGRFFGEIDPSLMRHLVNISVYAFNKSYDY
QSVCEPGAAPKQGAGHRSAYVPSVADILQIGWWATAAAWSILQQFLLSLTFPRLLEAVEM
EDDDFTASLSKQSCITEQTQYFFDNDSKSFSGVLDCGNCSRIFHGEKLMNTNLIFIMVES
KGTCPCDTRLLIQAEQTSDGPNPCDMVKQPRYRKGPDVCFDNNVLEDYTDCGGVSGLNPS
LWYIIGIQFLLLWLVSGSTHRLL
>sp|P54687|BCAT1_HUMAN Branched-chain-amino-acid aminotransferase, cytosolic OS=Homo sapiens OX=9606 GN=BCAT1 PE=1 SV=3
MKDCSNGCSAECTGEGGSKEVVGTFKAKDLIVTPATILKEKPDPNNLVFGTVFTDHMLTV
EWSSEFGWEKPHIKPLQNLSLHPGSSALHYAVELFEGLKAFRGVDNKIRLFQPNLNMDRM
YRSAVRATLPVFDKEELLECIQQLVKLDQEWVPYSTSASLYIRPTFIGTEPSLGVKKPTK
ALLFVLLSPVGPYFSSGTFNPVSLWANPKYVRAWKGGTGDCKMGGNYGSSLFAQCEAVDN
GCQQVLWLYGEDHQITEVGTMNLFLYWINEDGEEELATPPLDGIILPGVTRRCILDLAHQ
WGEFKVSERYLTMDDLTTALEGNRVREMFGSGTACVVCPVSDILYKGETIHIPTMENGPK
LASRILSKLTDIQYGREESDWTIVLS
>sp|P54709|AT1B3_HUMAN Sodium/potassium-transporting ATPase subunit beta-3 OS=Homo sapiens OX=9606 GN=ATP1B3 PE=1 SV=1
MTKNEKKSLNQSLAEWKLFIYNPTTGEFLGRTAKSWGLILLFYLVFYGFLAALFSFTMWV
MLQTLNDEVPKYRDQIPSPGLMVFPKPVTALEYTFSRSDPTSYAGYIEDLKKFLKPYTLE
EQKNLTVCPDGALFEQKGPVYVACQFPISLLQACSGMNDPDFGYSQGNPCILVKMNRIIG
LKPEGVPRIDCVSKNEDIPNVAVYPHNGMIDLKYFPYYGKKLHVGYLQPLVAVQVSFAPN
NTGKEVTVECKIDGSANLKSQDDRDKFLGRVMFKITARA
>sp|P54753|EPHB3_HUMAN Ephrin type-B receptor 3 OS=Homo sapiens OX=9606 GN=EPHB3 PE=1 SV=2
MARARPPPPPSPPPGLLPLLPPLLLLPLLLLPAGCRALEETLMDTKWVTSELAWTSHPES
GWEEVSGYDEAMNPIRTYQVCNVRESSQNNWLRTGFIWRRDVQRVYVELKFTVRDCNSIP
NIPGSCKETFNLFYYEADSDVASASSPFWMENPYVKVDTIAPDESFSRLDAGRVNTKVRS
FGPLSKAGFYLAFQDQGACMSLISVRAFYKKCASTTAGFALFPETLTGAEPTSLVIAPGT
CIPNAVEVSVPLKLYCNGDGEWMVPVGACTCATGHEPAAKESQCRPCPPGSYKAKQGEGP
CLPCPPNSRTTSPAASICTCHNNFYRADSDSADSACTTVPSPPRGVISNVNETSLILEWS
EPRDLGGRDDLLYNVICKKCHGAGGASACSRCDDNVEFVPRQLGLTERRVHISHLLAHTR
YTFEVQAVNGVSGKSPLPPRYAAVNITTNQAAPSEVPTLRLHSSSGSSLTLSWAPPERPN
GVILDYEMKYFEKSEGIASTVTSQMNSVQLDGLRPDARYVVQVRARTVAGYGQYSRPAEF
ETTSERGSGAQQLQEQLPLIVGSATAGLVFVVAVVVIAIVCLRKQRHGSDSEYTEKLQQY
IAPGMKVYIDPFTYEDPNEAVREFAKEIDVSCVKIEEVIGAGEFGEVCRGRLKQPGRREV
FVAIKTLKVGYTERQRRDFLSEASIMGQFDHPNIIRLEGVVTKSRPVMILTEFMENCALD
SFLRLNDGQFTVIQLVGMLRGIAAGMKYLSEMNYVHRDLAARNILVNSNLVCKVSDFGLS
RFLEDDPSDPTYTSSLGGKIPIRWTAPEAIAYRKFTSASDVWSYGIVMWEVMSYGERPYW
DMSNQDVINAVEQDYRLPPPMDCPTALHQLMLDCWVRDRNLRPKFSQIVNTLDKLIRNAA

SLKVIASAQSGMSQPLLDRTVPDYTTFTTVGDWLDAIKMGRYKESFVSAGFASFDLVAQM
TAEDLLRIGVTLAGHQKKILSSIQDMRLQMNQTLPVQV
>sp|P54851|EMP2_HUMAN Epithelial membrane protein 2 OS=Homo sapiens OX=9606 GN=EMP2 PE=1 SV=1
MLVLLAFIIAFHITSAALLFIATVDNAWWVGDEFFADVWRICTNNTNCTVINDSFQEYST
LQAVQATMILSTILCCIAFFIFVLQLFRLKQGERFVLTSIIQLMSCLCVMIAASIYTDRR
EDIHDKNAKFYPVTREGSYGYSYILAWVAFACTFISGMMYLILRKRK
>sp|P54920|SNAA_HUMAN Alpha-soluble NSF attachment protein OS=Homo sapiens OX=9606 GN=NAPA PE=1 SV=3
MDNSGKEAEAMALLAEAERKVKNSQSFFSGLFGGSSKIEEACEIYARAANMFKMAKNWSA
AGNAFCQAAQLHLQLQSKHDAATCFVDAGNAFKKADPQEAINCLMRAIEIYTDMGRFTIA
AKHHISIAEIYETELVDIEKAIAHYEQSADYYKGEESNSSANKCLLKVAGYAALLEQYQK
AIDIYEQVGTNAMDSPLLKYSAKDYFFKAALCHFCIDMLNAKLAVQKYEELFPAFSDSRE
CKLMKKLLEAHEEQNVDSYTESVKEYDSISRLDQWLTTMLLRIKKTIQGDEEDLR
>sp|P55001|MFAP2_HUMAN Microfibrillar-associated protein 2 OS=Homo sapiens OX=9606 GN=MFAP2 PE=1 SV=1
MRAAYLFLLFLPAGLLAQGQYDLDPLPPFPDHVQYTHYSDQIDNPDYYDYQEVTPRPSEE
QFQFQSQQQVQQEVIPAPTPEPGNAELEPTEPGPLDCREEQYPCTRLYSIHRPCKQCLNE
VCFYSLRRVYVINKEICVRTVCAHEELLRADLCRDKFSKCGVMASSGLCQSVAASCARSC
GSC
>sp|P55011|S12A2_HUMAN Solute carrier family 12 member 2 OS=Homo sapiens OX=9606 GN=SLC12A2 PE=1 SV=1
MEPRPTAPSSGAPGLAGVGETPSAAALAAARVELPGTAVPSVPEDAAPASRDGGGVRDEG
PAAAGDGLGRPLGPTPSQSRFQVDLVSENAGRAAAAAAAAAAAAAAAGAGAGAKQTPADG
EASGESEPAKGSEEAKGRFRVNFVDPAASSSAEDSLSDAAGVGVDGPNVSFQNGGDTVLS
EGSSLHSGGGGGSGHHQHYYYDTHTNTYYLRTFGHNTMDAVPRIDHYRHTAAQLGEKLLR
PSLAELHDELEKEPFEDGFANGEESTPTRDAVVTYTAESKGVVKFGWIKGVLVRCMLNIW
GVMLFIRLSWIVGQAGIGLSVLVIMMATVVTTITGLSTSAIATNGFVRGGGAYYLISRSL
GPEFGGAIGLIFAFANAVAVAMYVVGFAETVVELLKEHSILMIDEINDIRIIGAITVVIL
LGISVAGMEWEAKAQIVLLVILLLAIGDFVIGTFIPLESKKPKGFFGYKSEIFNENFGPD
FREEETFFSVFAIFFPAATGILAGANISGDLADPQSAIPKGTLLAILITTLVYVGIAVSV
GSCVVRDATGNVNDTIVTELTNCTSAACKLNFDFSSCESSPCSYGLMNNFQVMSMVSGFT
PLISAGIFSATLSSALASLVSAPKIFQALCKDNIYPAFQMFAKGYGKNNEPLRGYILTFL
IALGFILIAELNVIAPIISNFFLASYALINFSVFHASLAKSPGWRPAFKYYNMWISLLGA
ILCCIVMFVINWWAALLTYVIVLGLYIYVTYKKPDVNWGSSTQALTYLNALQHSIRLSGV
EDHVKNFRPQCLVMTGAPNSRPALLHLVHDFTKNVGLMICGHVHMGPRRQAMKEMSIDQA
KYQRWLIKNKMKAFYAPVHADDLREGAQYLMQAAGLGRMKPNTLVLGFKKDWLQADMRDV
DMYINLFHDAFDIQYGVVVIRLKEGLDISHLQGQEELLSSQEKSPGTKDVVVSVEYSKKS
DLDTSKPLSEKPITHKVEEEDGKTATQPLLKKESKGPIVPLNVADQKLLEASTQFQKKQG
KNTIDVWWLFDDGGLTLLIPYLLTTKKKWKDCKIRVFIGGKINRIDHDRRAMATLLSKFR
IDFSDIMVLGDINTKPKKENIIAFEEIIEPYRLHEDDKEQDIADKMKEDEPWRITDNELE
LYKTKTYRQIRLNELLKEHSSTANIIVMSLPVARKGAVSSALYMAWLEALSKDLPPILLV
RGNHQSVLTFYS
>sp|P55060|XPO2_HUMAN Exportin-2 OS=Homo sapiens OX=9606 GN=CSE1L PE=1 SV=3

MELSDANLQTLTEYLKKTLDPDPAIRRPAEKFLESVEGNQNYPLLLLTLLEKSQDNVIKV
CASVTFKNYIKRNWRIVEDEPNKICEADRVAIKANIVHLMLSSPEQIQKQLSDAISIIGR
EDFPQKWPDLLTEMVNRFQSGDFHVINGVLRTAHSLFKRYRHEFKSNELWTEIKLVLDAF
ALPLTNLFKATIELCSTHANDASALRILFSSLILISKLFYSLNFQDLPEFFEDNMETWMN
NFHTLLTLDNKLLQTDDEEEAGLLELLKSQICDNAALYAQKYDEEFQRYLPRFVTAIWNL
LVTTGQEVKYDLLVSNAIQFLASVCERPHYKNLFEDQNTLTSICEKVIVPNMEFRAADEE
AFEDNSEEYIRRDLEGSDIDTRRRAACDLVRGLCKFFEGPVTGIFSGYVNSMLQEYAKNP
SVNWKHKDAAIYLVTSLASKAQTQKHGITQANELVNLTEFFVNHILPDLKSANVNEFPVL
KADGIKYIMIFRNQVPKEHLLVSIPLLINHLQAESIVVHTYAAHALERLFTMRGPNNATL
FTAAEIAPFVEILLTNLFKALTLPGSSENEYIMKAIMRSFSLLQEAIIPYIPTLITQLTQ
KLLAVSKNPSKPHFNHYMFEAICLSIRITCKANPAAVVNFEEALFLVFTEILQNDVQEFI
PYVFQVMSLLLETHKNDIPSSYMALFPHLLQPVLWERTGNIPALVRLLQAFLERGSNTIA
SAAADKIPGLLGVFQKLIASKANDHQGFYLLNSIIEHMPPESVDQYRKQIFILLFQRLQN
SKTTKFIKSFLVFINLYCIKYGALALQEIFDGIQPKMFGMVLEKIIIPEIQKVSGNVEKK
ICAVGITKLLTECPPMMDTEYTKLWTPLLQSLIGLFELPEDDTIPDEEHFIDIEDTPGYQ
TAFSQLAFAGKKEHDPVGQMVNNPKIHLAQSLHKLSTACPGRVPSMVSTSLNAEALQYLQ
GYLQAASVTLL
>sp|P55064|AQP5_HUMAN Aquaporin-5 OS=Homo sapiens OX=9606 GN=AQP5 PE=1 SV=1
MKKEVCSVAFLKAVFAEFLATLIFVFFGLGSALKWPSALPTILQIALAFGLAIGTLAQAL
GPVSGGHINPAITLALLVGNQISLLRAFFYVAAQLVGAIAGAGILYGVAPLNARGNLAVN
ALNNNTTQGQAMVVELILTFQLALCIFASTDSRRTSPVGSPALSIGLSVTLGHLVGIYFT
GCSMNPARSFGPAVVMNRFSPAHWVFWVGPIVGAVLAAILYFYLLFPNSLSLSERVAIIK
GTYEPDEDWEEQREERKKTMELTTR
>sp|P55072|TERA_HUMAN Transitional endoplasmic reticulum ATPase OS=Homo sapiens OX=9606 GN=VCP PE=1 SV=4
MASGADSKGDDLSTAILKQKNRPNRLIVDEAINEDNSVVSLSQPKMDELQLFRGDTVLLK
GKKRREAVCIVLSDDTCSDEKIRMNRVVRNNLRVRLGDVISIQPCPDVKYGKRIHVLPID
DTVEGITGNLFEVYLKPYFLEAYRPIRKGDIFLVRGGMRAVEFKVVETDPSPYCIVAPDT
VIHCEGEPIKREDEEESLNEVGYDDIGGCRKQLAQIKEMVELPLRHPALFKAIGVKPPRG
ILLYGPPGTGKTLIARAVANETGAFFFLINGPEIMSKLAGESESNLRKAFEEAEKNAPAI
IFIDELDAIAPKREKTHGEVERRIVSQLLTLMDGLKQRAHVIVMAATNRPNSIDPALRRF
GRFDREVDIGIPDATGRLEILQIHTKNMKLADDVDLEQVANETHGHVGADLAALCSEAAL
QAIRKKMDLIDLEDETIDAEVMNSLAVTMDDFRWALSQSNPSALRETVVEVPQVTWEDIG
GLEDVKRELQELVQYPVEHPDKFLKFGMTPSKGVLFYGPPGCGKTLLAKAIANECQANFI
SIKGPELLTMWFGESEANVREIFDKARQAAPCVLFFDELDSIAKARGGNIGDGGGAADRV
INQILTEMDGMSTKKNVFIIGATNRPDIIDPAILRPGRLDQLIYIPLPDEKSRVAILKAN
LRKSPVAKDVDLEFLAKMTNGFSGADLTEICQRACKLAIRESIESEIRRERERQTNPSAM
EVEEDDPVPEIRRDHFEEAMRFARRSVSDNDIRKYEMFAQTLQQSRGFGSFRFPSGNQGG
AGPSQGSGGGTGGSVYTEDNDDDLYG
>sp|P55084|ECHB_HUMAN Trifunctional enzyme subunit beta, mitochondrial OS=Homo sapiens OX=9606 GN=HADHB PE=1 SV=3
MTILTYPFKNLPTASKWALRFSIRPLSCSSQLRAAPAVQTKTKKTLAKPNIRNVVVVDGV
RTPFLLSGTSYKDLMPHDLARAALTGLLHRTSVPKEVVDYIIFGTVIQEVKTSNVAREAA
LGAGFSDKTPAHTVTMACISANQAMTTGVGLIASGQCDVIVAGGVELMSDVPIRHSRKMR

KLMLDLNKAKSMGQRLSLISKFRFNFLAPELPAVSEFSTSETMGHSADRLAAAFAVSRLE
QDEYALRSHSLAKKAQDEGLLSDVVPFKVPGKDTVTKDNGIRPSSLEQMAKLKPAFIKPY
GTVTAANSSFLTDGASAMLIMAEEKALAMGYKPKAYLRDFMYVSQDPKDQLLLGPTYATP
KVLEKAGLTMNDIDAFEFHEAFSGQILANFKAMDSDWFAENYMGRKTKVGLPPLEKFNNW
GGSLSLGHPFGATGCRLVMAAANRLRKEGGQYGLVAACAAGGQGHAMIVEAYPK
>sp|P55209|NP1L1_HUMAN Nucleosome assembly protein 1-like 1 OS=Homo sapiens OX=9606 GN=NAP1L1 PE=1 SV=1
MADIDNKEQSELDQDLDDVEEVEEEETGEETKLKARQLTVQMMQNPQILAALQERLDGLV
ETPTGYIESLPRVVKRRVNALKNLQVKCAQIEAKFYEEVHDLERKYAVLYQPLFDKRFEI
INAIYEPTEEECEWKPDEEDEISEELKEKAKIEDEKKDEEKEDPKGIPEFWLTVFKNVDL
LSDMVQEHDEPILKHLKDIKVKFSDAGQPMSFVLEFHFEPNEYFTNEVLTKTYRMRSEPD
DSDPFSFDGPEIMGCTGCQIDWKKGKNVTLKTIKKKQKHKGRGTVRTVTKTVSNDSFFNF
FAPPEVPESGDLDDDAEAILAADFEIGHFLRERIIPRSVLYFTGEAIEDDDDDYDEEGEE
ADEEGEEEGDEENDPDYDPKKDQNPAECKQQ
>sp|P55263|ADK_HUMAN Adenosine kinase OS=Homo sapiens OX=9606 GN=ADK PE=1 SV=2
MAAAEEEPKPKKLKVEAPQALRENILFGMGNPLLDISAVVDKDFLDKYSLKPNDQILAED
KHKELFDELVKKFKVEYHAGGSTQNSIKVAQWMIQQPHKAATFFGCIGIDKFGEILKRKA
AEAHVDAHYYEQNEQPTGTCAACITGDNRSLIANLAAANCYKKEKHLDLEKNWMLVEKAR
VCYIAGFFLTVSPESVLKVAHHASENNRIFTLNLSAPFISQFYKESLMKVMPYVDILFGN
ETEAATFAREQGFETKDIKEIAKKTQALPKMNSKRQRIVIFTQGRDDTIMATESEVTAFA
VLDQDQKEIIDTNGAGDAFVGGFLSQLVSDKPLTECIRAGHYAASIIIRRTGCTFPEKPD
FH
>sp|P55268|LAMB2_HUMAN Laminin subunit beta-2 OS=Homo sapiens OX=9606 GN=LAMB2 PE=1 SV=2
MELTSRERGRGQPLPWELRLGLLLSVLAATLAQAPAPDVPGCSRGSCYPATGDLLVGRAD
RLTASSTCGLNGPQPYCIVSHLQDEKKCFLCDSRRPFSARDNPHSHRIQNVVTSFAPQRR
AAWWQSENGIPAVTIQLDLEAEFHFTHLIMTFKTFRPAAMLVERSADFGRTWHVYRYFSY
DCGADFPGVPLAPPRHWDDVVCESRYSEIEPSTEGEVIYRVLDPAIPIPDPYSSRIQNLL
KITNLRVNLTRLHTLGDNLLDPRREIREKYYYALYELVVRGNCFCYGHASECAPAPGAPA
HAEGMVHGACICKHNTRGLNCEQCQDFYRDLPWRPAEDGHSHACRKCECHGHTHSCHFDM
AVYLASGNVSGGVCDGCQHNTAGRHCELCRPFFYRDPTKDLRDPAVCRSCDCDPMGSQDG
GRCDSHDDPALGLVSGQCRCKEHVVGTRCQQCRDGFFGLSISDRLGCRRCQCNARGTVPG
STPCDPNSGSCYCKRLVTGRGCDRCLPGHWGLSHDLLGCRPCDCDVGGALDPQCDEGTGQ
CHCRQHMVGRRCEQVQPGYFRPFLDHLIWEAEDTRGQVLDVVERLVTPGETPSWTGSGFV
RLQEGQTLEFLVASVPKAMDYDLLLRLEPQVPEQWAELELIVQRPGPVPAHSLCGHLVPK
DDRIQGTLQPHARYLIFPNPVCLEPGISYKLHLKLVRTGGSAQPETPYSGPGLLIDSLVL
LPRVLVLEMFSGGDAAALERQATFERYQCHEEGLVPSKTSPSEACAPLLISLSTLIYNGA
LPCQCNPQGSLSSECNPHGGQCLCKPGVVGRRCDLCAPGYYGFGPTGCQACQCSHEGALS
SLCEKTSGQCLCRTGAFGLRCDRCQRGQWGFPSCRPCVCNGHADECNTHTGACLGCRDHT
GGEHCERCIAGFHGDPRLPYGGQCRPCPCPEGPGSQRHFATSCHQDEYSQQIVCHCRAGY
TGLRCEACAPGHFGDPSRPGGRCQLCECSGNIDPMDPDACDPHTGQCLRCLHHTEGPHCA
HCKPGFHGQAARQSCHRCTCNLLGTNPQQCPSPDQCHCDPSSGQCPCLPNVQGPSCDRCA
PNFWNLTSGHGCQPCACHPSRARGPTCNEFTGQCHCRAGFGGRTCSECQELHWGDPGLQC
HACDCDSRGIDTPQCHRFTGHCSCRPGVSGVRCDQCARGFSGIFPACHPCHACFGDWDRV

VQDLAARTQRLEQRAQELQQTGVLGAFESSFWHMQEKLGIVQGIVGARNTSAASTAQLVE
ATEELRREIGEATEHLTQLEADLTDVQDENFNANHALSGLERDRLALNLTLRQLDQHLDL
LKHSNFLGAYDSIRHAHSQSAEAERRANTSALAVPSPVSNSASARHRTEALMDAQKEDFN
SKHMANQRALGKLSAHTHTLSLTDINELVCGAPGDAPCATSPCGGAGCRDEDGQPRCGGL
SCNGAAATADLALGRARHTQAELQRALAEGGSILSRVAETRRQASEAQQRAQAALDKANA
SRGQVEQANQELQELIQSVKDFLNQEGADPDSIEMVATRVLELSIPASAEQIQHLAGAIA
ERVRSLADVDAILARTVGDVRRAEQLLQDARRARSWAEDEKQKAETVQAALEEAQRAQGI
AQGAIRGAVADTRDTEQTLYQVQERMAGAERALSSAGERARQLDALLEALKLKRAGNSLA
ASTAEETAGSAQGRAQEAEQLLRGPLGDQYQTVKALAERKAQGVLAAQARAEQLRDEARD
LLQAAQDKLQRLQELEGTYEENERALESKAAQLDGLEARMRSVLQAINLQVQIYNTCQ
>sp|P55290|CAD13_HUMAN Cadherin-13 OS=Homo sapiens OX=9606 GN=CDH13 PE=1 SV=1
MQPRTPLVLCVLLSQVLLLTSAEDLDCTPGFQQKVFHINQPAEFIEDQSILNLTFSDCKG
NDKLRYEVSSPYFKVNSDGGLVALRNITAVGKTLFVHARTPHAEDMAELVIVGGKDIQGS
LQDIFKFARTSPVPRQKRSIVVSPILIPENQRQPFPRDVGKVVDSDRPERSKFRLTGKGV
DQEPKGIFRINENTGSVSVTRTLDREVIAVYQLFVETTDVNGKTLEGPVPLEVIVIDQND
NRPIFREGPYIGHVMEGSPTGTTVMRMTAFDADDPATDNALLRYNIRQQTPDKPSPNMFY
IDPEKGDIVTVVSPALLDRETLENPKYELIIEAQDMAGLDVGLTGTATATIMIDDKNDHS
PKFTKKEFQATVEEGAVGVIVNLTVEDKDDPTTGAWRAAYTIINGNPGQSFEIHTNPQTN
EGMLSVVKPLDYEISAFHTLLIKVENEDPLVPDVSYGPSSTATVHITVLDVNEGPVFYPD
PMMVTRQEDLSVGSVLLTVNATDPDSLQHQTIRYSVYKDPAGWLNINPINGTVDTTAVLD
RESPFVDNSVYTALFLAIDSGNPPATGTGTLLITLEDVNDNAPFIYPTVAEVCDDAKNLS
VVILGASDKDLHPNTDPFKFEIHKQAVPDKVWKISKINNTHALVSLLQNLNKANYNLPIM
VTDSGKPPMTNITDLRVQVCSCRNSKVDCNAAGALRFSLPSVLLLSLFSLACL
>sp|P55344|LMIP_HUMAN Lens fiber membrane intrinsic protein OS=Homo sapiens OX=9606 GN=LIM2 PE=1 SV=2
MYSFMGGGLFCAWVGTILLVVAMATDHWMQYRLSGSFAHQGLWRYCLGNKCYLQTDSIAY
WNATRAFMILSALCAISGIIMGIMAFAHQPTFSRISRPFSAGIMFFSSTLFVVLALAIYT
GVTVSFLGRRFGDWRFSWSYILGWVAVLMTFFAGIFYMCAYRVHECRRLSTPR
>sp|P55786|PSA_HUMAN Puromycin-sensitive aminopeptidase OS=Homo sapiens OX=9606 GN=NPEPPS PE=1 SV=2
MWLAAAAPSLARRLLFLGPPPPPLLLLVFSRSSRRRLHSLGLAAMPEKRPFERLPADVSP
INYSLCLKPDLLDFTFEGKLEAAAQVRQATNQIVMNCADIDIITASYAPEGDEEIHATGF
NYQNEDEKVTLSFPSTLQTGTGTLKIDFVGELNDKMKGFYRSKYTTPSGEVRYAAVTQFE
ATDARRAFPCWDEPAIKATFDISLVVPKDRVALSNMNVIDRKPYPDDENLVEVKFARTPV
MSTYLVAFVVGEYDFVETRSKDGVCVRVYTPVGKAEQGKFALEVAAKTLPFYKDYFNVPY
PLPKIDLIAIADFAAGAMENWGLVTYRETALLIDPKNSCSSSRQWVALVVGHELAHQWFG
NLVTMEWWTHLWLNEGFASWIEYLCVDHCFPEYDIWTQFVSADYTRAQELDALDNSHPIE
VSVGHPSEVDEIFDAISYSKGASVIRMLHDYIGDKDFKKGMNMYLTKFQQKNAATEDLWE
SLENASGKPIAAVMNTWTKQMGFPLIYVEAEQVEDDRLLRLSQKKFCAGGSYVGEDCPQW
MVPITISTSEDPNQAKLKILMDKPEMNVVLKNVKPDQWVKLNLGTVGFYRTQYSSAMLES
LLPGIRDLSLPPVDRLGLQNDLFSLARAGIISTVEVLKVMEAFVNEPNYTVWSDLSCNLG
ILSTLLSHTDFYEEIQEFVKDVFSPIGERLGWDPKPGEGHLDALLRGLVLGKLGKAGHKA
TLEEARRRFKDHVEGKQILSADLRSPVYLTVLKHGDGTTLDIMLKLHKQADMQEEKNRIE
RVLGATLLPDLIQKVLTFALSEEVRPQDTVSVIGGVAGGSKHGRKAAWKFIKDNWEELYN

RYQGGFLISRLIKLSVEGFAVDKMAGEVKAFFESHPAPSAERTIQQCCENILLNAAWLKR
DAESIHQYLLQRKASPPTV
>sp|P55884|EIF3B_HUMAN Eukaryotic translation initiation factor 3 subunit B OS=Homo sapiens OX=9606 GN=EIF3B PE=1 SV=3
MQDAENVAVPEAAEERAEPGQQQPAAEPPPAEGLLRPAGPGAPEAAGTEASSEEVGIAEA
GPESEVRTEPAAEAEAASGPSESPSPPAAEELPGSHAEPPVPAQGEAPGEQARDERSDSR
AQAVSEDAGGNEGRAAEAEPRALENGDADEPSFSDPEDFVDDVSEEELLGDVLKDRPQEA
DGIDSVIVVDNVPQVGPDRLEKLKNVIHKIFSKFGKITNDFYPEEDGKTKGYIFLEYASP
AHAVDAVKNADGYKLDKQHTFRVNLFTDFDKYMTISDEWDIPEKQPFKDLGNLRYWLEEA
ECRDQYSVIFESGDRTSIFWNDVKDPVSIEERARWTETYVRWSPKGTYLATFHQRGIALW
GGEKFKQIQRFSHQGVQLIDFSPCERYLVTFSPLMDTQDDPQAIIIWDILTGHKKRGFHC
ESSAHWPIFKWSHDGKFFARMTLDTLSIYETPSMGLLDKKSLKISGIKDFSWSPGGNIIA
FWVPEDKDIPARVTLMQLPTRQEIRVRNLFNVVDCKLHWQKNGDYLCVKVDRTPKGTQGV
VTNFEIFRMREKQVPVDVVEMKETIIAFAWEPNGSKFAVLHGEAPRISVSFYHVKNNGKI
ELIKMFDKQQANTIFWSPQGQFVVLAGLRSMNGALAFVDTSDCTVMNIAEHYMASDVEWD
PTGRYVVTSVSWWSHKVDNAYWLWTFQGRLLQKNNKDRFCQLLWRPRPPTLLSQEQIKQI
KKDLKKYSKIFEQKDRLSQSKASKELVERRRTMMEDFRKYRKMAQELYMEQKNERLELRG
GVDTDELDSNVDDWEEETIEFFVTEEIIPLGNQE
>sp|P56134|ATPK_HUMAN ATP synthase subunit f, mitochondrial OS=Homo sapiens OX=9606 GN=ATP5MF PE=1 SV=3
MASVGECPAPVPVKDKKLLEVKLGELPSWILMRDFSPSGIFGAFQRGYYRYYNKYINVKK
GSISGITMVLACYVLFSYSFSYKHLKHERLRKYH
>sp|P58546|MTPN_HUMAN Myotrophin OS=Homo sapiens OX=9606 GN=MTPN PE=1 SV=2
MCDKEFMWALKNGDLDEVKDYVAKGEDVNRTLEGGRKPLHYAADCGQLEILEFLLLKGAD
INAPDKHHITPLLSAVYEGHVSCVKLLLSKGADKTVKGPDGLTAFEATDNQAIKALLQ
>sp|P59998|ARPC4_HUMAN Actin-related protein 2/3 complex subunit 4 OS=Homo sapiens OX=9606 GN=ARPC4 PE=1 SV=3
MTATLRPYLSAVRATLQAALCLENFSSQVVERHNKPEVEVRSSKELLLQPVTISRNEKEK
VLIEGSINSVRVSIAVKQADEIEKILCHKFMRFMMMRAENFFILRRKPVEGYDISFLITN
FHTEQMYKHKLVDFVIHFMEEIDKEISEMKLSVNARARIVAEEFLKNF
>sp|P60033|CD81_HUMAN CD81 antigen OS=Homo sapiens OX=9606 GN=CD81 PE=1 SV=1
MGVEGCTKCIKYLLFVFNFVFWLAGGVILGVALWLRHDPQTTNLLYLELGDKPAPNTFYV
GIYILIAVGAVMMFVGFLGCYGAIQESQCLLGTFFTCLVILFACEVAAGIWGFVNKDQIA
KDVKQFYDQALQQAVVDDDANNAKAVVKTFHETLDCCGSSTLTALTTSVLKNNLCPSGSN
IISNLFKEDCHQKIDDLFSGKLYLIGIAAIVVAVIMIFEMILSMVLCCGIRNSSVY
>sp|P60174|TPIS_HUMAN Triosephosphate isomerase OS=Homo sapiens OX=9606 GN=TPI1 PE=1 SV=4
MAPSRKFFVGGNWKMNGRKQSLGELIGTLNAAKVPADTEVVCAPPTAYIDFARQKLDPKI
AVAAQNCYKVTNGAFTGEISPGMIKDCGATWVVLGHSERRHVFGESDELIGQKVAHALAE
GLGVIACIGEKLDEREAGITEKVVFEQTKVIADNVKDWSKVVLAYEPVWAIGTGKTATPQ
QAQEVHEKLRGWLKSNVSDAVAQSTRIIYGGSVTGATCKELASQPDVDGFLVGGASLKPE
FVDIINAKQ
>sp|P60228|EIF3E_HUMAN Eukaryotic translation initiation factor 3 subunit E OS=Homo sapiens OX=9606 GN=EIF3E PE=1 SV=1

MAEYDLTTRIAHFLDRHLVFPLLEFLSVKEIYNEKELLQGKLDLLSDTNMVDFAMDVYKN
LYSDDIPHALREKRTTVVAQLKQLQAETEPIVKMFEDPETTRQMQSTRDGRMLFDYLADK
HGFRQEYLDTLYRYAKFQYECGNYSGAAEYLYFFRVLVPATDRNALSSLWGKLASEILMQ
NWDAAMEDLTRLKETIDNNSVSSPLQSLQQRTWLIHWSLFVFFNHPKGRDNIIDLFLYQP
QYLNAIQTMCPHILRYLTTAVITNKDVRKRRQVLKDLVKVIQQESYTYKDPITEFVECLY
VNFDFDGAQKKLRECESVLVNDFFLVACLEDFIENARLFIFETFCRIHQCISINMLADKL
NMTPEEAERWIVNLIRNARLDAKIDSKLGHVVMGNNAVSPYQQVIEKTKSLSFRSQMLAM
NIEKKLNQNSRSEAPNWATQDSGFY
>sp|P60709|ACTB_HUMAN Actin, cytoplasmic 1 OS=Homo sapiens OX=9606 GN=ACTB PE=1 SV=1
MDDDIAALVVDNGSGMCKAGFAGDDAPRAVFPSIVGRPRHQGVMVGMGQKDSYVGDEAQS
KRGILTLKYPIEHGIVTNWDDMEKIWHHTFYNELRVAPEEHPVLLTEAPLNPKANREKMT
QIMFETFNTPAMYVAIQAVLSLYASGRTTGIVMDSGDGVTHTVPIYEGYALPHAILRLDL
AGRDLTDYLMKILTERGYSFTTTAEREIVRDIKEKLCYVALDFEQEMATAASSSSLEKSY
ELPDGQVITIGNERFRCPEALFQPSFLGMESCGIHETTFNSIMKCDVDIRKDLYANTVLS
GGTTMYPGIADRMQKEITALAPSTMKIKIIAPPERKYSVWIGGSILASLSTFQQMWISKQ
EYDESGPSIVHRKCF
>sp|P60763|RAC3_HUMAN Ras-related C3 botulinum toxin substrate 3 OS=Homo sapiens OX=9606 GN=RAC3 PE=1 SV=1
MQAIKCVVVGDGAVGKTCLLISYTTNAFPGEYIPTVFDNYSANVMVDGKPVNLGLWDTAG
QEDYDRLRPLSYPQTDVFLICFSLVSPASFENVRAKWYPEVRHHCPHTPILLVGTKLDLR
DDKDTIERLRDKKLAPITYPQGLAMAREIGSVKYLECSALTQRGLKTVFDEAIRAVLCPP
PVKKPGKKCTVF
>sp|P60842|IF4A1_HUMAN Eukaryotic initiation factor 4A-I OS=Homo sapiens OX=9606 GN=EIF4A1 PE=1 SV=1
MSASQDSRSRDNGPDGMEPEGVIESNWNEIVDSFDDMNLSESLLRGIYAYGFEKPSAIQQ
RAILPCIKGYDVIAQAQSGTGKTATFAISILQQIELDLKATQALVLAPTRELAQQIQKVV
MALGDYMGASCHACIGGTNVRAEVQKLQMEAPHIIVGTPGRVFDMLNRRYLSPKYIKMFV
LDEADEMLSRGFKDQIYDIFQKLNSNTQVVLLSATMPSDVLEVTKKFMRDPIRILVKKEE
LTLEGIRQFYINVEREEWKLDTLCDLYETLTITQAVIFINTRRKVDWLTEKMHARDFTVS
AMHGDMDQKERDVIMREFRSGSSRVLITTDLLARGIDVQQVSLVINYDLPTNRENYIHRI
GRGGRFGRKGVAINMVTEEDKRTLRDIETFYNTSIEEMPLNVADLI
>sp|P60880|SNP25_HUMAN Synaptosomal-associated protein 25 OS=Homo sapiens OX=9606 GN=SNAP25 PE=1 SV=1
MAEDADMRNELEEMQRRADQLADESLESTRRMLQLVEESKDAGIRTLVMLDEQGEQLERI
EEGMDQINKDMKEAEKNLTDLGKFCGLCVCPCNKLKSSDAYKKAWGNNQDGVVASQPARV
VDEREQMAISGGFIRRVTNDARENEMDENLEQVSGIIGNLRHMALDMGNEIDTQNRQIDR
IMEKADSNKTRIDEANQRATKMLGSG
>sp|P60891|PRPS1_HUMAN Ribose-phosphate pyrophosphokinase 1 OS=Homo sapiens OX=9606 GN=PRPS1 PE=1 SV=2
MPNIKIFSGSSHQDLSQKIADRLGLELGKVVTKKFSNQETCVEIGESVRGEDVYIVQSGC
GEINDNLMELLIMINACKIASASRVTAVIPCFPYARQDKKDKSRAPISAKLVANMLSVAG
ADHIITMDLHASQIQGFFDIPVDNLYAEPAVLKWIRENISEWRNCTIVSPDAGGAKRVTS
IADRLNVDFALIHKERKKANEVDRMVLVGDVKDRVAILVDDMADTCGTICHAADKLLSAG

ATRVYAILTHGIFSGPAISRINNACFEAVVVTNTIPQEDKMKHCSKIQVIDISMILAEAI
RRTHNGESVSYLFSHVPL
>sp|P60900|PSA6_HUMAN Proteasome subunit alpha type-6 OS=Homo sapiens OX=9606
GN=PSMA6 PE=1 SV=1
MSRGSSAGFDRHITIFSPEGRLYQVEYAFKAINQGGLTSVAVRGKDCAVIVTQKKVPDKL
LDSSTVTHLFKITENIGCVMTGMTADSRSQVQRARYEAANWKYKYGYEIPVDMLCKRIAD
ISQVYTQNAEMRPLGCCMILIGIDEEQGPQVYKCDPAGYYCGFKATAAGVKQTESTSFLE
KKVKKKFDWTFEQTVETAITCLSTVLSIDFKPSEIEVGVVTVENPKFRILTEAEIDAHLV
ALAERD
>sp|P60953|CDC42_HUMAN Cell division control protein 42 homolog OS=Homo sapiens
OX=9606 GN=CDC42 PE=1 SV=2
MQTIKCVVVGDGAVGKTCLLISYTTNKFPSEYVPTVFDNYAVTVMIGGEPYTLGLFDTAG
QEDYDRLRPLSYPQTDVFLVCFSVVSPSSFENVKEKWVPEITHHCPKTPFLLVGTQIDLR
DDPSTIEKLAKNKQKPITPETAEKLARDLKAVKYVECSALTQKGLKNVFDEAILAALEPP
EPKKSRRCVLL
>sp|P60981|DEST_HUMAN Destrin OS=Homo sapiens OX=9606 GN=DSTN PE=1 SV=3
MASGVQVADEVCRIFYDMKVRKCSTPEEIKKRKKAVIFCLSADKKCIIVEEGKEILVGDV
GVTITDPFKHFVGMLPEKDCRYALYDASFETKESRKEELMFFLWAPELAPLKSKMIYASS
KDAIKKKFQGIKHECQANGPEDLNRACIAEKLGGSLIVAFEGCPV
>sp|P61006|RAB8A_HUMAN Ras-related protein Rab-8A OS=Homo sapiens OX=9606
GN=RAB8A PE=1 SV=1
MAKTYDYLFKLLLIGDSGVGKTCVLFRFSEDAFNSTFISTIGIDFKIRTIELDGKRIKLQ
IWDTAGQERFRTITTAYYRGAMGIMLVYDITNEKSFDNIRNWIRNIEEHASADVEKMILG
NKCDVNDKRQVSKERGEKLALDYGIKFMETSAKANINVENAFFTLARDIKAKMDKKLEGN
SPQGSNQGVKITPDQQKRSSFFRCVLL
>sp|P61018|RAB4B_HUMAN Ras-related protein Rab-4B OS=Homo sapiens OX=9606
GN=RAB4B PE=1 SV=1
MAETYDFLFKFLVIGSAGTGKSCLLHQFIENKFKQDSNHTIGVEFGSRVVNVGGKTVKLQ
IWDTAGQERFRSVTRSYYRGAAGALLVYDITSRETYNSLAAWLTDARTLASPNIVVILCG
NKKDLDPEREVTFLEASRFAQENELMFLETSALTGENVEEAFLKCARTILNKIDSGELDP
ERMGSGIQYGDASLRQLRQPRSAQAVAPQPCGC
>sp|P61019|RAB2A_HUMAN Ras-related protein Rab-2A OS=Homo sapiens OX=9606
GN=RAB2A PE=1 SV=1
MAYAYLFKYIIIGDTGVGKSCLLLQFTDKRFQPVHDLTIGVEFGARMITIDGKQIKLQIW
DTAGQESFRSITRSYYRGAAGALLVYDITRRDTFNHLTTWLEDARQHSNSNMVIMLIGNK
SDLESRREVKKEEGEAFAREHGLIFMETSAKTASNVEEAFINTAKEIYEKIQEGVFDINN
EANGIKIGPQHAATNATHAGNQGGQQAGGGCC
>sp|P61020|RAB5B_HUMAN Ras-related protein Rab-5B OS=Homo sapiens OX=9606
GN=RAB5B PE=1 SV=1
MTSRSTARPNGQPQASKICQFKLVLLGESAVGKSSLVLRFVKGQFHEYQESTIGAAFLTQ
SVCLDDTTVKFEIWDTAGQERYHSLAPMYYRGAQAAIVVYDITNQETFARAKTWVKELQR
QASPSIVIALAGNKADLANKRMVEYEEAQAYADDNSLLFMETSAKTAMNVNDLFLAIAKK
LPKSEPQNLGGAAGRSRGVDLHEQSQQNKSQCCSN

>sp|P61026|RAB10_HUMAN Ras-related protein Rab-10 OS=Homo sapiens OX=9606 GN=RAB10 PE=1 SV=1
MAKKTYDLLFKLLLIGDSGVGKTCVLFRFSDDAFNTTFISTIGIDFKIKTVELQGKKIKL
QIWDTAGQERFHTITTSYYRGAMGIMLVYDITNGKSFENISKWLRNIDEHANEDVERMLL
GNKCDMDDKRVVPKGKGEQIAREHGIRFFETSAKANINIEKAFLTLAEDILRKTPVKEPN
SENVDISSGGGVTGWKSKCC
>sp|P61081|UBC12_HUMAN NEDD8-conjugating enzyme Ubc12 OS=Homo sapiens OX=9606 GN=UBE2M PE=1 SV=1
MIKLFSLKQQKKEEESAGGTKGSSKKASAAQLRIQKDINELNLPKTCDISFSDPDDLLNF
KLVICPDEGFYKSGKFVFSFKVGQGYPHDPPKVKCETMVYHPNIDLEGNVCLNILREDWK
PVLTINSIIYGLQYLFLEPNPEDPLNKEAAEVLQNNRRLFEQNVQRSMRGGYIGSTYFER
CLK
>sp|P61088|UBE2N_HUMAN Ubiquitin-conjugating enzyme E2 N OS=Homo sapiens OX=9606 GN=UBE2N PE=1 SV=1
MAGLPRRIIKETQRLLAEPVPGIKAEPDESNARYFHVVIAGPQDSPFEGGTFKLELFLPE
EYPMAAPKVRFMTKIYHPNVDKLGRICLDILKDKWSPALQIRTVLLSIQALLSAPNPDDP
LANDVAEQWKTNEAQAIETARAWTRLYAMNNI
>sp|P61106|RAB14_HUMAN Ras-related protein Rab-14 OS=Homo sapiens OX=9606 GN=RAB14 PE=1 SV=4
MATAPYNYSYIFKYIIIGDMGVGKSCLLHQFTEKKFMADCPHTIGVEFGTRIIEVSGQKI
KLQIWDTAGQERFRAVTRSYYRGAAGALMVYDITRRSTYNHLSSWLTDARNLTNPNTVII
LIGNKADLEAQRDVTYEEAKQFAEENGLLFLEASAKTGENVEDAFLEAAKKIYQNIQDGS
LDLNAAESGVQHKPSAPQGGRLTSEPQPQREGCGC
>sp|P61158|ARP3_HUMAN Actin-related protein 3 OS=Homo sapiens OX=9606 GN=ACTR3 PE=1 SV=3
MAGRLPACVVDCGTGYTKLGYAGNTEPQFIIPSCIAIKESAKVGDQAQRRVMKGVDDLDF
FIGDEAIEKPTYATKWPIRHGIVEDWDLMERFMEQVIFKYLRAEPEDHYFLLTEPPLNTP
ENREYTAEIMFESFNVPGLYIAVQAVLALAASWTSRQVGERTLTGTVIDSGDGVTHVIPV
AEGYVIGSCIKHIPIAGRDITYFIQQLLRDREVGIPPEQSLETAKAVKERYSYVCPDLVK
EFNKYDTDGSKWIKQYTGINAISKKEFSIDVGYERFLGPEIFFHPEFANPDFTQPISEVV
DEVIQNCPIDVRRPLYKNIVLSGGSTMFRDFGRRLQRDLKRTVDARLKLSEELSGGRLKP
KPIDVQVITHHMQRYAVWFGGSMLASTPEFYQVCHTKKDYEEIGPSICRHNPVFGVMS
>sp|P61160|ARP2_HUMAN Actin-related protein 2 OS=Homo sapiens OX=9606 GN=ACTR2 PE=1 SV=1
MDSQGRKVVVCDNGTGFVKCGYAGSNFPEHIFPALVGRPIIRSTTKVGNIEIKDLMVGDE
ASELRSMLEVNYPMENGIVRNWDDMKHLWDYTFGPEKLNIDTRNCKILLTEPPMNPTKNR
EKIVEVMFETYQFSGVYVAIQAVLTLYAQGLLTGVVVDSGDGVTHICPVYEGFSLPHLTR
RLDIAGRDITRYLIKLLLLRGYAFNHSADFETVRMIKEKLCYVGYNIEQEQKLALETTVL
VESYTLPDGRIIKVGGERFEAPEALFQPHLINVEGVGVAELLFNTIQAADIDTRSEFYKH
IVLSGGSTMYPGLPSRLERELKQLYLERVLKGDVEKLSKFKIRIEDPPRRKHMVFLGGAV
LADIMKDKDNFWMTRQEYQEKGVRVLEKLGVTVR
>sp|P61163|ACTZ_HUMAN Alpha-centractin OS=Homo sapiens OX=9606 GN=ACTR1A PE=1 SV=1
MESYDVIANQPVVIDNGSGVIKAGFAGDQIPKYCFPNYVGRPKHVRVMAGALEGDIFIGP

KAEEHRGLLSIRYPMEHGIVKDWNDMERIWQYVYSKDQLQTFSEEHPVLLTEAPLNPRKN
RERAAEVFFETFNVPALFISMQAVLSLYATGRTTGVVLDSGDGVTHAVPIYEGFAMPHSI
MRIDIAGRDVSRFLRLYLRKEGYDFHSSSEFEIVKAIKERACYLSINPQKDETLETEKAQ
YYLPDGSTIEIGPSRFRAPELLFRPDLIGEESEGIHEVLVFAIQKSDMDLRRTLFSNIVL
SGGSTLFKGFGDRLLSEVKKLAPKDVKIRISAPQERLYSTWIGGSILASLDTFKKMWVSK
KEYEEDGARSIHRKTF
>sp|P61225|RAP2B_HUMAN Ras-related protein Rap-2b OS=Homo sapiens OX=9606 GN=RAP2B PE=1 SV=1
MREYKVVVLGSGGVGKSALTVQFVTGSFIEKYDPTIEDFYRKEIEVDSSPSVLEILDTAG
TEQFASMRDLYIKNGQGFILVYSLVNQQSFQDIKPMRDQIIRVKRYERVPMILVGNKVDL
EGEREVSYGEGKALAEEWSCPFMETSAKNKASVDELFAEIVRQMNYAAQPNGDEGCCSAC
VIL
>sp|P61247|RS3A_HUMAN Small ribosomal subunit protein eS1 OS=Homo sapiens OX=9606 GN=RPS3A PE=1 SV=2
MAVGKNKRLTKGGKKGAKKKVVDPFSKKDWYDVKAPAMFNIRNIGKTLVTRTQGTKIASD
GLKGRVFEVSLADLQNDEVAFRKFKLITEDVQGKNCLTNFHGMDLTRDKMCSMVKKWQTM
IEAHVDVKTTDGYLLRLFCVGFTKKRNNQIRKTSYAQHQQVRQIRKKMMEIMTREVQTND
LKEVVNKLIPDSIGKDIEKACQSIYPLHDVFVRKVKMLKKPKFELGKLMELHGEGSSSGK
ATGDETGAKVERADGYEPPVQESV
>sp|P61313|RL15_HUMAN Large ribosomal subunit protein eL15 OS=Homo sapiens OX=9606 GN=RPL15 PE=1 SV=2
MGAYKYIQELWRKKQSDVMRFLLRVRCWQYRQLSALHRAPRPTRPDKARRLGYKAKQGYV
IYRIRVRRGGRKRPVPKGATYGKPVHHGVNQLKFARSLQSVAEERAGRHCGALRVLNSYW
VGEDSTYKFFEVILIDPFHKAIRRNPDTQWITKPVHKHREMRGLTSAGRKSRGLGKGHKF
HHTIGGSRRAAWRRRNTLQLHRYR
>sp|P61457|PHS_HUMAN Pterin-4-alpha-carbinolamine dehydratase OS=Homo sapiens OX=9606 GN=PCBD1 PE=1 SV=2
MAGKAHRLSAEERDQLLPNLRAVGWNELEGRDAIFKQFHFKDFNRAFGFMTRVALQAEKL
DHHPEWFNVYNKVHITLSTHECAGLSERDINLASFIEQVAVSMT
>sp|P61513|RL37A_HUMAN Large ribosomal subunit protein eL43 OS=Homo sapiens OX=9606 GN=RPL37A PE=1 SV=2
MAKRTKKVGIVGKYGTRYGASLRKMVKKIEISQHAKYTCSFCGKTKMKRRAVGIWHCGSC
MKTVAGGAWTYNTTSAVTVKSAIRRLKELKDQ
>sp|P61586|RHOA_HUMAN Transforming protein RhoA OS=Homo sapiens OX=9606 GN=RHOA PE=1 SV=1
MAAIRKKLVIVGDGACGKTCLLIVFSKDQFPEVYVPTVFENYVADIEVDGKQVELALWDT
AGQEDYDRLRPLSYPDTDVILMCFSIDSPDSLENIPEKWTPEVKHFCPNVPIILVGNKKD
LRNDEHTRRELAKMKQEPVKPEEGRDMANRIGAFGYMECSAKTKDGVREVFEMATRAALQ
ARRGKKKSGCLVL
>sp|P61626|LYSC_HUMAN Lysozyme C OS=Homo sapiens OX=9606 GN=LYZ PE=1 SV=1
MKALIVLGLVLLSVTVQGKVFERCELARTLKRLGMDGYRGISLANWMCLAKWESGYNTRA
TNYNAGDRSTDYGIFQINSRYWCNDGKTPGAVNACHLSCSALLQDNIADAVACAKRVVRD
PQGIRAWVAWRNRCQNRDVRQYVQGCGV

>sp|P61970|NTF2_HUMAN Nuclear transport factor 2 OS=Homo sapiens OX=9606 GN=NUTF2 PE=1 SV=1
MGDKPIWEQIGSSFIQHYYQLFDNDRTQLGAIYIDASCLTWEGQQFQGKAAIVEKLSSLP
FQKIQHSITAQDHQPTPDSCIISMVVGQLKADEDPIMGFHQMFLLKNINDAWVCTNDMFR
LALHNFG
>sp|P61981|1433G_HUMAN 14-3-3 protein gamma OS=Homo sapiens OX=9606 GN=YWHAG PE=1 SV=2
MVDREQLVQKARLAEQAERYDDMAAAMKNVTELNEPLSNEERNLLSVAYKNVVGARRSSW
RVISSIEQKTSADGNEKKIEMVRAYREKIEKELEAVCQDVLSLLDNYLIKNCSETQYESK
VFYLKMKGDYYRYLAEVATGEKRATVVESSEKAYSEAHEISKEHMQPTHPIRLGLALNYS
VFYYEIQNAPEQACHLAKTAFDDAIAELDTLNEDSYKDSTLIMQLLRDNLTLWTSDQQDD
DGGEGNN
>sp|P62136|PP1A_HUMAN Serine/threonine-protein phosphatase PP1-alpha catalytic subunit OS=Homo sapiens OX=9606 GN=PPP1CA PE=1 SV=1
MSDSEKLNLDSIIGRLLEVQGSRPGKNVQLTENEIRGLCLKSREIFLSQPILLELEAPLK
ICGDIHGQYYDLLRLFEYGGFPPESNYLFLGDYVDRGKQSLETICLLLAYKIKYPENFFL
LRGNHECASINRIYGFYDECKRRYNIKLWKTFTDCFNCLPIAAIVDEKIFCCHGGLSPDL
QSMEQIRRIMRPTDVPDQGLLCDLLWSDPDKDVQGWGENDRGVSFTFGAEVVAKFLHKHD
LDLICRAHQVVEDGYEFFAKRQLVTLFSAPNYCGEFDNAGAMMSVDETLMCSFQILKPAD
KNKGKYGQFSGLNPGGRPITPPRNSAKAKK
>sp|P62191|PRS4_HUMAN 26S proteasome regulatory subunit 4 OS=Homo sapiens OX=9606 GN=PSMC1 PE=1 SV=1
MGQSQSGGHGPGGGKKDDKDKKKKYEPPVPTRVGKKKKKTKGPDAASKLPLVTPHTQCRL
KLLKLERIKDYLLMEEEFIRNQEQMKPLEEKQEEERSKVDDLRGTPMSVGTLEEIIDDNH
AIVSTSVGSEHYVSILSFVDKDLLEPGCSVLLNHKVHAVIGVLMDDTDPLVTVMKVEKAP
QETYADIGGLDNQIQEIKESVELPLTHPEYYEEMGIKPPKGVILYGPPGTGKTLLAKAVA
NQTSATFLRVVGSELIQKYLGDGPKLVRELFRVAEEHAPSIVFIDEIDAIGTKRYDSNSG
GEREIQRTMLELLNQLDGFDSRGDVKVIMATNRIETLDPALIRPGRIDRKIEFPLPDEKT
KKRIFQIHTSRMTLADDVTLDDLIMAKDDLSGADIKAICTEAGLMALRERRMKVTNEDFK
KSKENVLYKKQEGTPEGLYL
>sp|P62195|PRS8_HUMAN 26S proteasome regulatory subunit 8 OS=Homo sapiens OX=9606 GN=PSMC5 PE=1 SV=1
MALDGPEQMELEEGKAGSGLRQYYLSKIEELQLIVNDKSQNLRRLQAQRNELNAKVRLLR
EELQLLQEQGSYVGEVVRAMDKKKVLVKVHPEGKFVVDVDKNIDINDVTPNCRVALRNDS
YTLHKILPNKVDPLVSLMMVEKVPDSTYEMIGGLDKQIKEIKEVIELPVKHPELFEALGI
AQPKGVLLYGPPGTGKTLLARAVAHHTDCTFIRVSGSELVQKFIGEGARMVRELFVMARE
HAPSIIFMDEIDSIGSSRLEGGSGGDSEVQRTMLELLNQLDGFEATKNIKVIMATNRIDI
LDSALLRPGRIDRKIEFPPPNEEARLDILKIHSRKMNLTRGINLRKIAELMPGASGAEVK
GVCTEAGMYALRERRVHVTQEDFEMAVAKVMQKDSEKNMSIKKLWK
>sp|P62241|RS8_HUMAN Small ribosomal subunit protein eS8 OS=Homo sapiens OX=9606 GN=RPS8 PE=1 SV=2
MGISRDNWHKRRKTGGKRKPYHKKRKYELGRPAANTKIGPRRIHTVRVRGGNKKYRALRL
DVGNFSWGSECCTRKTRIIDVVYNASNNELVRTKTLVKNCIVLIDSTPYRQWYESHYALP
LGRKKGAKLTPEEEEILNKKRSKKIQKKYDERKKNAKISSLLEEQFQQGKLLACIASRPG

QCGRADGYVLEGKELEFYLRKIKARKGK
>sp|P62258|1433E_HUMAN 14-3-3 protein epsilon OS=Homo sapiens OX=9606 GN=YWHAE PE=1 SV=1
MDDREDLVYQAKLAEQAERYDEMVESMKKVAGMDVELTVEERNLLSVAYKNVIGARRASW
RIISSIEQKEENKGGEDKLKMIREYRQMVETELKLICCDILDVLDKHLIPAANTGESKVF
YYKMKGDYHRYLAEFATGNDRKEAAENSLVAYKAASDIAMTELPPTHPIRLGLALNFSVF
YYEILNSPDRACRLAKAAFDDAIAELDTLSEESYKDSTLIMQLLRDNLTLWTSDMQGDGE
EQNKEALQDVEDENQ
>sp|P62266|RS23_HUMAN Small ribosomal subunit protein uS12 OS=Homo sapiens OX=9606 GN=RPS23 PE=1 SV=3
MGKCRGLRTARKLRSHRRDQKWHDKQYKKAHLGTALKANPFGGASHAKGIVLEKVGVEAK
QPNSAIRKCVRVQLIKNGKKITAFVPNDGCLNFIEENDEVLVAGFGRKGHAVGDIPGVRF
KVVKVANVSLLALYKGKKERPRS
>sp|P62269|RS18_HUMAN Small ribosomal subunit protein uS13 OS=Homo sapiens OX=9606 GN=RPS18 PE=1 SV=3
MSLVIPEKFQHILRVLNTNIDGRRKIAFAITAIKGVGRRYAHVVLRKADIDLTKRAGELT
EDEVERVITIMQNPRQYKIPDWFLNRQKDVKDGKYSQVLANGLDNKLREDLERLKKIRAH
RGLRHFWGLRVRGQHTKTTGRRGRTVGVSKKK
>sp|P62280|RS11_HUMAN Small ribosomal subunit protein uS17 OS=Homo sapiens OX=9606 GN=RPS11 PE=1 SV=3
MADIQTERAYQKQPTIFQNKKRVLLGETGKEKLPRYYKNIGLGFKTPKEAIEGTYIDKKC
PFTGNVSIRGRILSGVVTKMKMQRTIVIRRDYLHYIRKYNRFEKRHKNMSVHLSPCFRDV
QIGDIVTVGECRPLSKTVRFNVLKVTKAAGTKKQFQKF
>sp|P62424|RL7A_HUMAN Large ribosomal subunit protein eL8 OS=Homo sapiens OX=9606 GN=RPL7A PE=1 SV=2
MPKGKKAKGKKVAPAPAVVKKQEAKKVVNPLFEKRPKNFGIGQDIQPKRDLTRFVKWPRY
IRLQRQRAILYKRLKVPPAINQFTQALDRQTATQLLKLAHKYRPETKQEKKQRLLARAEK
KAAGKGDVPTKRPPVLRAGVNTVTTLVENKKAQLVVIAHDVDPIELVVFLPALCRKMGVP
YCIIKGKARLGRLVHRKTCTTVAFTQVNSEDKGALAKLVEAIRTNYNDRYDEIRRHWGGN
VLGPKSVARIAKLEKAKAKELATKLG
>sp|P62491|RB11A_HUMAN Ras-related protein Rab-11A OS=Homo sapiens OX=9606 GN=RAB11A PE=1 SV=3
MGTRDDEYDYLFKVVLIGDSGVGKSNLLSRFTRNEFNLESKSTIGVEFATRSIQVDGKTI
KAQIWDTAGQERYRAITSAYYRGAVGALLVYDIAKHLTYENVERWLKELRDHADSNIVIM
LVGNKSDLRHLRAVPTDEARAFAEKNGLSFIETSALDSTNVEAAFQTILTEIYRIVSQKQ
MSDRRENDMSPSNNVVPIHVPPTTENKPKVQCCQNI
>sp|P62701|RS4X_HUMAN Small ribosomal subunit protein eS4, X isoform OS=Homo sapiens OX=9606 GN=RPS4X PE=1 SV=2
MARGPKKHLKRVAAPKHWMLDKLTGVFAPRPSTGPHKLRECLPLIIFLRNRLKYALTGDE
VKKICMQRFIKIDGKVRTDITYPAGFMDVISIDKTGENFRLIYDTKGRFAVHRITPEEAK
YKLCKVRKIFVGTKGIPHLVTHDARTIRYPDPLIKVNDTIQIDLETGKITDFIKFDTGNL
CMVTGGANLGRIGVITNRERHPGSFDVVHVKDANGNSFATRLSNIFVIGKGNKPWISLPR
GKGIRLTIAEERDKRLAAKQSSG

>sp|P62745|RHOB_HUMAN Rho-related GTP-binding protein RhoB OS=Homo sapiens OX=9606 GN=RHOB PE=1 SV=1
MAAIRKKLVVVGDGACGKTCLLIVFSKDEFPEVYVPTVFENYVADIEVDGKQVELALWDT
AGQEDYDRLRPLSYPDTDVILMCFSVDSPDSLENIPEKWVPEVKHFCPNVPIILVANKKD
LRSDEHVRTELARMKQEPVRTDDGRAMAVRIQAYDYLECSAKTKEGVREVFETATRAALQ
KRYGSQNGCINCCKVL
>sp|P62750|RL23A_HUMAN Large ribosomal subunit protein uL23 OS=Homo sapiens OX=9606 GN=RPL23A PE=1 SV=1
MAPKAKKEAPAPPKAEAKAKALKAKKAVLKGVHSHKKKKIRTSPTFRRPKTLRLRRQPKY
PRKSAPRRNKLDHYAIIKFPLTTESAMKKIEDNNTLVFIVDVKANKHQIKQAVKKLYDID
VAKVNTLIRPDGEKKAYVRLAPDYDALDVANKIGII
>sp|P62753|RS6_HUMAN Small ribosomal subunit protein eS6 OS=Homo sapiens OX=9606 GN=RPS6 PE=1 SV=1
MKLNISFPATGCQKLIEVDDERKLRTFYEKRMATEVAADALGEEWKGYVVRISGGNDKQG
FPMKQGVLTHGRVRLLLSKGHSCYRPRRTGERKRKSVRGCIVDANLSVLNLVIVKKGEKD
IPGLTDTTVPRRLGPKRASRIRKLFNLSKEDDVRQYVVRKPLNKEGKKPRTKAPKIQRLV
TPRVLQHKRRRIALKKQRTKKNKEEAAEYAKLLAKRMKEAKEKRQEQIAKRRRLSSLRAS
TSKSESSQK
>sp|P62805|H4_HUMAN Histone H4 OS=Homo sapiens OX=9606 GN=H4C1 PE=1 SV=2
MSGRGKGGKGLGKGGAKRHRKVLRDNIQGITKPAIRRLARRGGVKRISGLIYEETRGVLK
VFLENVIRDAVTYTEHAKRKTVTAMDVVYALKRQGRTLYGFGG
>sp|P62820|RAB1A_HUMAN Ras-related protein Rab-1A OS=Homo sapiens OX=9606 GN=RAB1A PE=1 SV=3
MSSMNPEYDYLFKLLLIGDSGVGKSCLLLRFADDTYTESYISTIGVDFKIRTIELDGKTI
KLQIWDTAGQERFRTITSSYYRGAHGIIVVYDVTDQESFNNVKQWLQEIDRYASENVNKL
LVGNKCDLTTKKVVDYTTAKEFADSLGIPFLETSAKNATNVEQSFMTMAAEIKKRMGPGA
TAGGAEKSNVKIQSTPVKQSGGGCC
>sp|P62826|RAN_HUMAN GTP-binding nuclear protein Ran OS=Homo sapiens OX=9606 GN=RAN PE=1 SV=3
MAAQGEPQVQFKLVLVGDGGTGKTTFVKRHLTGEFEKKYVATLGVEVHPLVFHTNRGPIK
FNVWDTAGQEKFGGLRDGYYIQAQCAIIMFDVTSRVTYKNVPNWHRDLVRVCENIPIVLC
GNKVDIKDRKVKAKSIVFHRKKNLQYYDISAKSNYNFEKPFLWLARKLIGDPNLEFVAMP
ALAPPEVVMDPALAAQYEHDLEVAQTTALPDEDDDL
>sp|P62834|RAP1A_HUMAN Ras-related protein Rap-1A OS=Homo sapiens OX=9606 GN=RAP1A PE=1 SV=1
MREYKLVVLGSGGVGKSALTVQFVQGIFVEKYDPTIEDSYRKQVEVDCQQCMLEILDTAG
TEQFTAMRDLYMKNGQGFALVYSITAQSTFNDLQDLREQILRVKDTEDVPMILVGNKCDL
EDERVVGKEQGQNLARQWCNCAFLESSAKSKINVNEIFYDLVRQINRKTPVEKKKPKKKS
CLLL
>sp|P62847|RS24_HUMAN Small ribosomal subunit protein eS24 OS=Homo sapiens OX=9606 GN=RPS24 PE=1 SV=1
MNDTVTIRTRKFMTNRLLQRKQMVIDVLHPGKATVPKTEIREKLAKMYKTTPDVIFVFGF
RTHFGGGKTTGFGMIYDSLDYAKKNEPKHRLARHGLYEKKKTSRKQRKERKNRMKKVRGT
AKANVGAGKKPKE

>sp|P62851|RS25_HUMAN Small ribosomal subunit protein eS25 OS=Homo sapiens OX=9606 GN=RPS25 PE=1 SV=1
MPPKDDKKKKDAGKSAKKDKDPVNKSGGKAKKKKWSKGKVRDKLNNLVLFDKATYDKLCK
EVPNYKLITPAVVSERLKIRGSLARAALQELLSKGLIKLVSKHRAQVIYTRNTKGGDAPA
AGEDA
>sp|P62861|RS30_HUMAN Ubiquitin-like FUBI-ribosomal protein eS30 fusion protein OS=Homo sapiens OX=9606 GN=FAU PE=1 SV=2
MQLFVRAQELHTFEVTGQETVAQIKAHVASLEGIAPEDQVVLLAGAPLEDEATLGQCGVE
ALTTLEVAGRMLGGKVHGSLARAGKVRGQTPKVAKQEKKKKKTGRAKRRMQYNRRFVNVV
PTFGKKKGPNANS
>sp|P62873|GBB1_HUMAN Guanine nucleotide-binding protein G(I)/G(S)/G(T) subunit beta-1 OS=Homo sapiens OX=9606 GN=GNB1 PE=1 SV=3
MSELDQLRQEAEQLKNQIRDARKACADATLSQITNNIDPVGRIQMRTRRTLRGHLAKIYA
MHWGTDSRLLVSASQDGKLIIWDSYTTNKVHAIPLRSSWVMTCAYAPSGNYVACGGLDNI
CSIYNLKTREGNVRVSRELAGHTGYLSCCRFLDDNQIVTSSGDTTCALWDIETGQQTTTF
TGHTGDVMSLSLAPDTRLFVSGACDASAKLWDVREGMCRQTFTGHESDINAICFFPNGNA
FATGSDDATCRLFDLRADQELMTYSHDNIICGITSVSFSKSGRLLLAGYDDFNCNVWDAL
KADRAGVLAGHDNRVSCLGVTDDGMAVATGSWDSFLKIWN
>sp|P62879|GBB2_HUMAN Guanine nucleotide-binding protein G(I)/G(S)/G(T) subunit beta-2 OS=Homo sapiens OX=9606 GN=GNB2 PE=1 SV=3
MSELEQLRQEAEQLRNQIRDARKACGDSTLTQITAGLDPVGRIQMRTRRTLRGHLAKIYA
MHWGTDSRLLVSASQDGKLIIWDSYTTNKVHAIPLRSSWVMTCAYAPSGNFVACGGLDNI
CSIYSLKTREGNVRVSRELPGHTGYLSCCRFLDDNQIITSSGDTTCALWDIETGQQTVGF
AGHSGDVMSLSLAPDGRTFVSGACDASIKLWDVRDSMCRQTFIGHESDINAVAFFPNGYA
FTTGSDDATCRLFDLRADQELLMYSHDNIICGITSVAFSRSGRLLLAGYDDFNCNIWDAM
KGDRAGVLAGHDNRVSCLGVTDDGMAVATGSWDSFLKIWN
>sp|P62891|RL39_HUMAN Large ribosomal subunit protein eL39 OS=Homo sapiens OX=9606 GN=RPL39 PE=1 SV=2
MSSHKTFRIKRFLAKKQKQNRPIPQWIRMKTGNKIRYNSKRRHWRRTKLGL
>sp|P62910|RL32_HUMAN Large ribosomal subunit protein eL32 OS=Homo sapiens OX=9606 GN=RPL32 PE=1 SV=2
MAALRPLVKPKIVKKRTKKFIRHQSDRYVKIKRNWRKPRGIDNRVRRRFKGQILMPNIGY
GSNKKTKHMLPSGFRKFLVHNVKELEVLLMCNKSYCAEIAHNVSSKNRKAIVERAAQLAI
RVTNPNARLRSEENE
>sp|P62917|RL8_HUMAN Large ribosomal subunit protein uL2 OS=Homo sapiens OX=9606 GN=RPL8 PE=1 SV=2
MGRVIRGQRKGAGSVFRAHVKHRKGAARLRAVDFAERHGYIKGIVKDIIHDPGRGAPLAK
VVFRDPYRFKKRTELFIAAEGIHTGQFVYCGKKAQLNIGNVLPVGTMPEGTIVCCLEEKP
GDRGKLARASGNYATVISHNPETKKTRVKLPSGSKKVISSANRAVVGVVAGGGRIDKPIL
KAGRAYHKYKAKRNCWPRVRGVAMNPVEHPFGGGNHQHIGKPSTIRRDAPAGRKVGLIAA
RRTGRLRGTKTVQEKEN
>sp|P62937|PPIA_HUMAN Peptidyl-prolyl cis-trans isomerase A OS=Homo sapiens OX=9606 GN=PPIA PE=1 SV=2
MVNPTVFFDIAVDGEPLGRVSFELFADKVPKTAENFRALSTGEKGFGYKGSCFHRIIPGF

MCQGGDFTRHNGTGGKSIYGEKFEDENFILKHTGPGILSMANAGPNTNGSQFFICTAKTE
WLDGKHVVFGKVKEGMNIVEAMERFGSRNGKTSKKITIADCGQLE
>sp|P63000|RAC1_HUMAN Ras-related C3 botulinum toxin substrate 1 OS=Homo sapiens OX=9606 GN=RAC1 PE=1 SV=1
MQAIKCVVVGDGAVGKTCLLISYTTNAFPGEYIPTVFDNYSANVMVDGKPVNLGLWDTAG
QEDYDRLRPLSYPQTDVFLICFSLVSPASFENVRAKWYPEVRHHCPNTPIILVGTKLDLR
DDKDTIEKLKEKKLTPITYPQGLAMAKEIGAVKYLECSALTQRGLKTVFDEAIRAVLCPP
PVKKRKRKCLLL
>sp|P63010|AP2B1_HUMAN AP-2 complex subunit beta OS=Homo sapiens OX=9606 GN=AP2B1 PE=1 SV=1
MTDSKYFTTNKKGEIFELKAELNNEKKEKRKEAVKKVIAAMTVGKDVSSLFPDVVNCMQT
DNLELKKLVYLYLMNYAKSQPDMAIMAVNSFVKDCEDPNPLIRALAVRTMGCIRVDKITE
YLCEPLRKCLKDEDPYVRKTAAVCVAKLHDINAQMVEDQGFLDSLRDLIADSNPMVVANA
VAALSEISESHPNSNLLDLNPQNINKLLTALNECTEWGQIFILDCLSNYNPKDDREAQSI
CERVTPRLSHANSAVVLSAVKVLMKFLELLPKDSDYYNMLLKKLAPPLVTLLSGEPEVQY
VALRNINLIVQKRPEILKQEIKVFFVKYNDPIYVKLEKLDIMIRLASQANIAQVLAELKE
YATEVDVDFVRKAVRAIGRCAIKVEQSAERCVSTLLDLIQTKVNYVVQEAIVVIRDIFRK
YPNKYESIIATLCENLDSLDEPDARAAMIWIVGEYAERIDNADELLESFLEGFHDESTQV
QLTLLTAIVKLFLKKPSETQELVQQVLSLATQDSDNPDLRDRGYIYWRLLSTDPVTAKEV
VLSEKPLISEETDLIEPTLLDELICHIGSLASVYHKPPNAFVEGSHGIHRKHLPIHHGST
DAGDSPVGTTTATNLEQPQVIPSQGDLLGDLLNLDLGPPVNVPQVSSMQMGAVDLLGGGL
DSLVGQSFIPSSVPATFAPSPTPAVVSSGLNDLFELSTGIGMAPGGYVAPKAVWLPAVKA
KGLEISGTFTHRQGHIYMEMNFTNKALQHMTDFAIQFNKNSFGVIPSTPLAIHTPLMPNQ
SIDVSLPLNTLGPVMKMEPLNNLQVAVKNNIDVFYFSCLIPLNVLFVEDGKMERQVFLAT
WKDIPNENELQFQIKECHLNADTVSSKLQNNNVYTIAKRNVEGQDMLYQSLKLTNGIWIL
AELRIQPGNPNYTLSLKCRAPEVSQYIYQVYDSILKN
>sp|P63027|VAMP2_HUMAN Vesicle-associated membrane protein 2 OS=Homo sapiens OX=9606 GN=VAMP2 PE=1 SV=3
MSATAATAPPAAPAGEGGPPAPPPNLTSNRRLQQTQAQVDEVVDIMRVNVDKVLERDQKL
SELDDRADALQAGASQFETSAAKLKRKYWWKNLKMMIILGVICAIILIIIIVYFST
>sp|P63092|GNAS2_HUMAN Guanine nucleotide-binding protein G(s) subunit alpha isoforms short OS=Homo sapiens OX=9606 GN=GNAS PE=1 SV=1
MGCLGNSKTEDQRNEEKAQREANKKIEKQLQKDKQVYRATHRLLLLGAGESGKSTIVKQM
RILHVNGFNGEGGEEDPQAARSNSDGEKATKVQDIKNNLKEAIETIVAAMSNLVPPVELA
NPENQFRVDYILSVMNVPDFDFPPEFYEHAKALWEDEGVRACYERSNEYQLIDCAQYFLD
KIDVIKQADYVPSDQDLLRCRVLTSGIFETKFQVDKVNFHMFDVGGQRDERRKWIQCFND
VTAIIFVVASSSYNMVIREDNQTNRLQEALNLFKSIWNNRWLRTISVILFLNKQDLLAEK
VLAGKSKIEDYFPEFARYTTPEDATPEPGEDPRVTRAKYFIRDEFLRISTASGDGRHYCY
PHFTCAVDTENIRRVFNDCRDIIQRMHLRQYELL
>sp|P63096|GNAI1_HUMAN Guanine nucleotide-binding protein G(i) subunit alpha-1 OS=Homo sapiens OX=9606 GN=GNAI1 PE=1 SV=2
MGCTLSAEDKAAVERSKMIDRNLREDGEKAAREVKLLLLGAGESGKSTIVKQMKIIHEAG
YSEEECKQYKAVVYSNTIQSIIAIIRAMGRLKIDFGDSARADDARQLFVLAGAAEEGFMT
AELAGVIKRLWKDSGVQACFNRSREYQLNDSAAYYLNDLDRIAQPNYIPTQQDVLRTRVK

TTGIVETHFTFKDLHFKMFDVGGQRSERKKWIHCFEGVTAIIFCVALSDYDLVLAEDEEM
NRMHESMKLFDSICNNKWFTDTSIILFLNKKDLFEEKIKKSPLTICYPEYAGSNTYEEAA
AYIQCQFEDLNKRKDTKEIYTHFTCATDTKNVQFVFDAVTDVIIKNNLKDCGLF
>sp|P63104|1433Z_HUMAN 14-3-3 protein zeta/delta OS=Homo sapiens OX=9606 GN=YWHAZ PE=1 SV=1
MDKNELVQKAKLAEQAERYDDMAACMKSVTEQGAELSNEERNLLSVAYKNVVGARRSSWR
VVSSIEQKTEGAEKKQQMAREYREKIETELRDICNDVLSLLEKFLIPNASQAESKVFYLK
MKGDYYRYLAEVAAGDDKKGIVDQSQQAYQEAFEISKKEMQPTHPIRLGLALNFSVFYYE
ILNSPEKACSLAKTAFDEAIAELDTLSEESYKDSTLIMQLLRDNLTLWTSDTQGDEAEAG
EGGEN
>sp|P63218|GBG5_HUMAN Guanine nucleotide-binding protein G(I)/G(S)/G(O) subunit gamma-5 OS=Homo sapiens OX=9606 GN=GNG5 PE=1 SV=3
MSGSSSVAAMKKVVQQLRLEAGLNRVKVSQAAADLKQFCLQNAQHDPLLTGVSSSTNPFR
PQKVCSFL
>sp|P63244|RACK1_HUMAN Small ribosomal subunit protein RACK1 OS=Homo sapiens OX=9606 GN=RACK1 PE=1 SV=3
MTEQMTLRGTLKGHNGWVTQIATTPQFPDMILSASRDKTIIMWKLTRDETNYGIPQRALR
GHSHFVSDVVISSDGQFALSGSWDGTLRLWDLTTGTTTRRFVGHTKDVLSVAFSSDNRQI
VSGSRDKTIKLWNTLGVCKYTVQDESHSEWVSCVRFSPNSSNPIIVSCGWDKLVKVWNLA
NCKLKTNHIGHTGYLNTVTVSPDGSLCASGGKDGQAMLWDLNEGKHLYTLDGGDIINALC
FSPNRYWLCAATGPSIKIWDLEGKIIVDELKQEVISTSSKAEPPQCTSLAWSADGQTLFA
GYTDNLVRVWQVTIGTR
>sp|P68032|ACTC_HUMAN Actin, alpha cardiac muscle 1 OS=Homo sapiens OX=9606 GN=ACTC1 PE=1 SV=1
MCDDEETTALVCDNGSGLVKAGFAGDDAPRAVFPSIVGRPRHQGVMVGMGQKDSYVGDEA
QSKRGILTLKYPIEHGIITNWDDMEKIWHHTFYNELRVAPEEHPTLLTEAPLNPKANREK
MTQIMFETFNVPAMYVAIQAVLSLYASGRTTGIVLDSGDGVTHNVPIYEGYALPHAIMRL
DLAGRDLTDYLMKILTERGYSFVTTAEREIVRDIKEKLCYVALDFENEMATAASSSSLEK
SYELPDGQVITIGNERFRCPETLFQPSFIGMESAGIHETTYNSIMKCDIDIRKDLYANNV
LSGGTTMYPGIADRMQKEITALAPSTMKIKIIAPPERKYSVWIGGSILASLSTFQQMWIS
KQEYDEAGPSIVHRKCF
>sp|P68104|EF1A1_HUMAN Elongation factor 1-alpha 1 OS=Homo sapiens OX=9606 GN=EEF1A1 PE=1 SV=1
MGKEKTHINIVVIGHVDSGKSTTTGHLIYKCGGIDKRTIEKFEKEAAEMGKGSFKYAWVL
DKLKAERERGITIDISLWKFETSKYYVTIIDAPGHRDFIKNMITGTSQADCAVLIVAAGV
GEFEAGISKNGQTREHALLAYTLGVKQLIVGVNKMDSTEPPYSQKRYEEIVKEVSTYIKK
IGYNPDTVAFVPISGWNGDNMLEPSANMPWFKGWKVTRKDGNASGTTLLEALDCILPPTR
PTDKPLRLPLQDVYKIGGIGTVPVGRVETGVLKPGMVVTFAPVNVTTEVKSVEMHHEALS
EALPGDNVGFNVKNVSVKDVRRGNVAGDSKNDPPMEAAGFTAQVIILNHPGQISAGYAPV
LDCHTAHIACKFAELKEKIDRRSGKKLEDGPKFLKSGDAAIVDMVPGKPMCVESFSDYPP
LGRFAVRDMRQTVAVGVIKAVDKKAAGAGKVTKSAQKAQKAK
>sp|P68363|TBA1B_HUMAN Tubulin alpha-1B chain OS=Homo sapiens OX=9606 GN=TUBA1B PE=1 SV=1
MRECISIHVGQAGVQIGNACWELYCLEHGIQPDGQMPSDKTIGGGDDSFNTFFSETGAGK

HVPRAVFVDLEPTVIDEVRTGTYRQLFHPEQLITGKEDAANNYARGHYTIGKEIIDLVLD
RIRKLADQCTGLQGFLVFHSFGGGTGSGFTSLLMERLSVDYGKKSKLEFSIYPAPQVSTA
VVEPYNSILTTHTTLEHSDCAFMVDNEAIYDICRRNLDIERPTYTNLNRLISQIVSSITA
SLRFDGALNVDLTEFQTNLVPYPRIHFPLATYAPVISAEKAYHEQLSVAEITNACFEPAN
QMVKCDPRHGKYMACCLLYRGDVVPKDVNAAIATIKTKRSIQFVDWCPTGFKVGINYQPP
TVVPGGDLAKVQRAVCMLSNTTAIAEAWARLDHKFDLMYAKRAFVHWYVGEGMEEGEFSE
AREDMAALEKDYEEVGVDSVEGEGEEEGEEY
>sp|P68431|H31_HUMAN Histone H3.1 OS=Homo sapiens OX=9606 GN=H3C1 PE=1 SV=2
MARTKQTARKSTGGKAPRKQLATKAARKSAPATGGVKKPHRYRPGTVALREIRRYQKSTE
LLIRKLPFQRLVREIAQDFKTDLRFQSSAVMALQEACEAYLVGLFEDTNLCAIHAKRVTI
MPKDIQLARRIRGERA
>sp|P69905|HBA_HUMAN Hemoglobin subunit alpha OS=Homo sapiens OX=9606 GN=HBA1
PE=1 SV=2
MVLSPADKTNVKAAWGKVGAHAGEYGAEALERMFLSFPTTKTYFPHFDLSHGSAQVKGHG
KKVADALTNAVAHVDDMPNALSALSDLHAHKLRVDPVNFKLLSHCLLVTLAAHLPAEFTP
AVHASLDKFLASVSTVLTSKYR
>sp|P78310|CXAR_HUMAN Coxsackievirus and adenovirus receptor OS=Homo sapiens
OX=9606 GN=CXADR PE=1 SV=1
MALLLCFVLLCGVVDFARSLSITTPEEMIEKAKGETAYLPCKFTLSPEDQGPLDIEWLIS
PADNQKVDQVIILYSGDKIYDDYYPDLKGRVHFTSNDLKSGDASINVTNLQLSDIGTYQC
KVKKAPGVANKKIHLVVLVKPSGARCYVDGSEEIGSDFKIKCEPKEGSLPLQYEWQKLSD
SQKMPTSWLAEMTSSVISVKNASSEYSGTYSCTVRNRVGSDQCLLRLNVVPPSNKAGLIA
GAIIGTLLALALIGLIIFCCRKKRREEKYEKEVHHDIREDVPPPKSRTSTARSYIGSNHS
SLGSMSPSNMEGYSKTQYNQVPSEDFERTPQSPTLPPAKVAAPNLSRMGAIPVMIPAQSK
DGSIV
>sp|P78324|SHPS1_HUMAN Tyrosine-protein phosphatase non-receptor type substrate 1
OS=Homo sapiens OX=9606 GN=SIRPA PE=1 SV=2
MEPAGPAPGRLGPLLCLLLAASCAWSGVAGEEELQVIQPDKSVLVAAGETATLRCTATSL
IPVGPIQWFRGAGPGRELIYNQKEGHFPRVTTVSDLTKRNNMDFSIRIGNITPADAGTYY
CVKFRKGSPDDVEFKSGAGTELSVRAKPSAPVVSGPAARATPQHTVSFTCESHGFSPRDI
TLKWFKNGNELSDFQTNVDPVGESVSYSIHSTAKVVLTREDVHSQVICEVAHVTLQGDPL
RGTANLSETIRVPPTLEVTQQPVRAENQVNVTCQVRKFYPQRLQLTWLENGNVSRTETAS
TVTENKDGTYNWMSWLLVNVSAHRDDVKLTCQVEHDGQPAVSKSHDLKVSAHPKEQGSNT
AAENTGSNERNIYIVVGVVCTLLVALLMAALYLVRIRQKKAQGSTSSTRLHEPEKNAREI
TQDTNDITYADLNLPKGKKPAPQAAEPNNHTEYASIQTSPQPASEDTLTYADLDMVHLNR
TPKQPAPKPEPSFSEYASVQVPRK
>sp|P78371|TCPB_HUMAN T-complex protein 1 subunit beta OS=Homo sapiens OX=9606
GN=CCT2 PE=1 SV=4
MASLSLAPVNIFKAGADEERAETARLTSFIGAIAIGDLVKSTLGPKGMDKILLSSGRDAS
LMVTNDGATILKNIGVDNPAAKVLVDMSRVQDDEVGDGTTSVTVLAAELLREAESLIAKK
IHPQTIIAGWREATKAAREALLSSAVDHGSDEVKFRQDLMNIAGTTLSSKLLTHHKDHFT
KLAVEAVLRLKGSGNLEAIHIIKKLGGSLADSYLDEGFLLDKKIGVNQPKRIENAKILIA
NTGMDTDKIKIFGSRVRVDSTAKVAEIEHAEKEKMKEKVERILKHGINCFINRQLIYNYP
EQLFGAAGVMAIEHADFAGVERLALVTGGEIASTFDHPELVKLGSCKLIEEVMIGEDKLI

HFSGVALGEACTIVLRGATQQILDEAERSLHDALCVLAQTVKDSRTVYGGGCSEMLMAHA
VTQLANRTPGKEAVAMESYAKALRMLPTIIADNAGYDSADLVAQLRAAHSEGNTTAGLDM
REGTIGDMAILGITESFQVKRQVLLSAAEAAEVILRVDNIIKAAPRKRVPDHHPC
>sp|P78385|KRT83_HUMAN Keratin, type II cuticular Hb3 OS=Homo sapiens OX=9606 GN=KRT83 PE=1 SV=2
MTCGFNSIGCGFRPGNFSCVSACGPRPSRCCITAAPYRGISCYRGLTGGFGSHSVCGGFR
AGSCGRSFGYRSGGVCGPSPPCITTVSVNESLLTPLNLEIDPNAQCVKQEEKEQIKSLNS
RFAAFIDKVRFLEQQNKLLETKLQFYQNRECCQSNLEPLFAGYIETLRREAECVEADSGR
LASELNHVQEVLEGYKKKYEEEVALRATAENEFVALKKDVDCAYLRKSDLEANVEALIQE
IDFLRRLYEEEIRILQSHISDTSVVVKLDNSRDLNMDCIVAEIKAQYDDIATRSRAEAES
WYRSKCEEMKATVIRHGETLRRTKEEINELNRMIQRLTAEVENAKCQNSKLEAAVAQSEQ
QGEAALSDARCKLAELEGALQKAKQDMACLIREYQEVMNSKLGLDIEIATYRRLLEGEEQ
RLCEGVEAVNVCVSSSRGGVVCGDLCVSGSRPVTGSVCSAPCNGNLVVSTGLCKPCGQLN
TTCGGGSCGQGRH
>sp|P80723|BASP1_HUMAN Brain acid soluble protein 1 OS=Homo sapiens OX=9606 GN=BASP1 PE=1 SV=2
MGGKLSKKKKGYNVNDEKAKEKDKKAEGAATEEEGTPKESEPQAAAEPAEAKEGKEKPDQ
DAEGKAEEKEGEKDAAAAKEEAPKAEPEKTEGAAEAKAEPPKAPEQEQAAPGPAAGGEAP
KAAEAAAAPAESAAPAAGEEPSKEEGEPKKTEAPAAPAAQETKSDGAPASDSKPGSSEAA
PSSKETPAATEAPSSTPKAQGPAASAEEPKPVEAPAANSDQTVTVKE
>sp|P83731|RL24_HUMAN Large ribosomal subunit protein eL24 OS=Homo sapiens OX=9606 GN=RPL24 PE=1 SV=1
MKVELCSFSGYKIYPGHGRRYARTDGKVFQFLNAKCESAFLSKRNPRQINWTVLYRRKHK
KGQSEEIQKKRTRRAVKFQRAITGASLADIMAKRNQKPEVRKAQREQAIRAAKEAKKAKQ
ASKKTAMAAAKAPTKAAPKQKIVKPVKVSAPRVGGKR
>sp|P84077|ARF1_HUMAN ADP-ribosylation factor 1 OS=Homo sapiens OX=9606 GN=ARF1 PE=1 SV=2
MGNIFANLFKGLFGKKEMRILMVGLDAAGKTTILYKLKLGEIVTTIPTIGFNVETVEYKN
ISFTVWDVGGQDKIRPLWRHYFQNTQGLIFVVDSNDRERVNEAREELMRMLAEDELRDAV
LLVFANKQDLPNAMNAAEITDKLGLHSLRHRNWYIQATCATSGDGLYEGLDWLSNQLRNQ
K
>sp|P84085|ARF5_HUMAN ADP-ribosylation factor 5 OS=Homo sapiens OX=9606 GN=ARF5 PE=1 SV=2
MGLTVSALFSRIFGKKQMRILMVGLDAAGKTTILYKLKLGEIVTTIPTIGFNVETVEYKN
ICFTVWDVGGQDKIRPLWRHYFQNTQGLIFVVDSNDRERVQESADELQKMLQEDELRDAV
LLVFANKQDMPNAMPVSELTDKLGLQHLRSRTWYVQATCATQGTGLYDGLDWLSHELSKR
>sp|P84095|RHOG_HUMAN Rho-related GTP-binding protein RhoG OS=Homo sapiens OX=9606 GN=RHOG PE=1 SV=1
MQSIKCVVVGDGAVGKTCLLICYTTNAFPKEYIPTVFDNYSAQSAVDGRTVNLNLWDTAG
QEEYDRLRTLSYPQTNVFVICFSIASPPSYENVRHKWHPEVCHHCPDVPILLVGTKKDLR
AQPDTLRRLKEQGQAPITPQQGQALAKQIHAVRYLECSALQQDGVKEVFAEAVRAVLNPT
PIKRGRSCILL
>sp|P84098|RL19_HUMAN Large ribosomal subunit protein eL19 OS=Homo sapiens OX=9606 GN=RPL19 PE=1 SV=1

MSMLRLQKRLASSVLRCGKKKVWLDPNETNEIANANSRQQIRKLIKDGLIIRKPVTVHSR
ARCRKNTLARRKGRHMGIGKRKGTANARMPEKVTWMRRMRILRRLLRRYRESKKIDRHMY
HSLYLKVKGNVFKNKRILMEHIHKLKADKARKKLLADQAEARRSKTKEARKRREERLQAK
KEEIIKTLSKEEETKK
>sp|P84157|MXRA7_HUMAN Matrix-remodeling-associated protein 7 OS=Homo sapiens OX=9606 GN=MXRA7 PE=1 SV=1
MEAPAELLAALPALATALALLLAWLLVRRGAAASPEPARAPPEPAPPAEATGAPAPSRPC
APEPAASPAGPEEPGEPAGLGELGEPAGPGEPEGPGDPAAAPAEAEEQAVEARQEEEQDL
DGEKGPSSEGPEEEDGEGFSFKYSPGKLRGNQYKKMMTKEELEEEQRVQKEQLAAIFKLM
KDNKETFGEMSDGDVQEQLRLYDM
>sp|P84243|H33_HUMAN Histone H3.3 OS=Homo sapiens OX=9606 GN=H3-3A PE=1 SV=2
MARTKQTARKSTGGKAPRKQLATKAARKSAPSTGGVKKPHRYRPGTVALREIRRYQKSTE
LLIRKLPFQRLVREIAQDFKTDLRFQSAAIGALQEASEAYLVGLFEDTNLCAIHAKRVTI
MPKDIQLARRIRGERA
>sp|P98160|PGBM_HUMAN Basement membrane-specific heparan sulfate proteoglycan core protein OS=Homo sapiens OX=9606 GN=HSPG2 PE=1 SV=4
MGWRAAGALLLALLLHGRLLAVTHGLRAYDGLSLPEDIETVTASQMRWTHSYLSDDEDML
ADSISGDDLGSGDLGSGDFQMVYFRALVNFTRSIEYSPQLEDAGSREFREVSEAVVDTLE
SEYLKIPGDQVVSVVFIKELDGWVFVELDVGSEGNADGAQIQEMLLRVISSGSVASYVTS
PQGFQFRRLGTVPQFPRACTEAEFACHSYNECVALEYRCDRRPDCRDMSDELNCEEPVLG
ISPTFSLLVETTSLPPRPETTIMRQPPVTHAPQPLLPGSVRPLPCGPQEAACRNGHCIPR
DYLCDGQEDCEDGSDELDCGPPPPCEPNEFPCGNGHCALKLWRCDGDFDCEDRTDEANCP
TKRPEEVCGPTQFRCVSTNMCIPASFHCDEESDCPDRSDEFGCMPPQVVTPPRESIQASR
GQTVTFTCVAIGVPTPIINWRLNWGHIPSHPRVTVTSEGGRGTLIIRDVKESDQGAYTCE
AMNARGMVFGIPDGVLELVPQRGPCPDGHFYLEHSAACLPCFCFGITSVCQSTRRFRDQI
RLRFDQPDDFKGVNVTMPAQPGTPPLSSTQLQIDPSLHEFQLVDLSRRFLVHDSFWALPE
QFLGNKVDSYGGSLRYNVRYELARGMLEPVQRPDVVLMGAGYRLLSRGHTPTQPGALNQR
QVQFSEEHWVHESGRPVQRAELLQVLQSLEAVLIQTVYNTKMASVGLSDIAMDTTVTHAT
SHGRAHSVEECRCPIGYSGLSCESCDAHFTRVPGGPYLGTCSGCNCNGHASSCDPVYGHC
LNCQHNTEGPQCNKCKAGFFGDAMKATATSCRPCPCPYIDASRRFSDTCFLDTDGQATCD
ACAPGYTGRRCESCAPGYEGNPIQPGGKCRPVNQEIVRCDERGSMGTSGEACRCKNNVVG
RLCNECADGSFHLSTRNPDGCLKCFCMGVSRHCTSSSWSRAQLHGASEEPGHFSLTNAAS
THTTNEGIFSPTPGELGFSSFHRLLSGPYFWSLPSRFLGDKVTSYGGELRFTVTQRSQPG
STPLHGQPLVVLQGNNIILEHHVAQEPSPGQPSTFIVPFREQAWQRPDGQPATREHLLMA
LAGIDTLLIRASYAQQPAESRVSGISMDVAVPEETGQDPALEVEQCSCPPGYRGPSCQDC
DTGYTRTPSGLYLGTCERCSCHGHSEACEPETGACQGCQHHTEGPRCEQCQPGYYGDAQR
GTPQDCQLCPCYGDPAAGQAAHTCFLDTDGHPTCDACSPGHSGRHCERCAPGYYGNPSQG
QPCQRDSQVPGPIGCNCDPQGSVSSQCDAAGQCQCKAQVEGLTCSHCRPHHFHLSASNPD
GCLPCFCMGITQQCASSAYTRHLISTHFAPGDFQGFALVNPQRNSRLTGEFTVEPVPEGA
QLSFGNFAQLGHESFYWQLPETYQGDKVAAYGGKLRYTLSYTAGPQGSPLSDPDVQITGN
NIMLVASQPALQGPERRSYEIMFREEFWRRPDGQPATREHLLMALADLDELLIRATFSSV
PLAASISAVSLEVAQPGPSNRPRALEVEECRCPPGYIGLSCQDCAPGYTRTGSGLYLGHC
ELCECNGHSDLCHPETGACSQCQHNAAGEFCELCAPGYYGDATAGTPEDCQPCACPLTNP
ENMFSRTCESLGAGGYRCTACEPGYTGQYCEQCGPGYVGNPSVQGGQCLPETNQAPLVVE

VHPARSIVPQGGSHSLRCQVSGSPPHYFYWSREDGRPVPSGTQQRHQGSELHFPSVQPSD
AGVYICTCRNLHQSNTSRAELLVTEAPSKPITVTVEEQRSQSVRPGADVTFICTAKSKSP
AYTLVWTRLHNGKLPTRAMDFNGILTIRNVQLSDAGTYVCTGSNMFAMDQGTATLHVQAS
GTLSAPVVSIHPPQLTVQPGQLAEFRCSATGSPTPTLEWTGGPGGQLPAKAQIHGGILRL
PAVEPTDQAQYLCRAHSSAGQQVARAVLHVHGGGGPRVQVSPERTQVHAGRTVRLYCRAA
GVPSATITWRKEGGSLPPQARSERTDIATLLIPAITTADAGFYLCVATSPAGTAQARIQV
VVLSASDASPPPVKIESSSPSVTEGQTLDLNCVVAGSAHAQVTWYRRGGSLPPHTQVHGS
RLRLPQVSPADSGEYVCRVENGSGPKEASITVSVLHGTHSGPSYTPVPGSTRPIRIEPSS
SHVAEGQTLDLNCVVPGQAHAQVTWHKRGGSLPARHQTHGSLLRLHQVTPADSGEYVCHV
VGTSGPLEASVLVTIEASVIPGPIPPVRIESSSSTVAEGQTLDLSCVVAGQAHAQVTWYK
RGGSLPARHQVRGSRLYIFQASPADAGQYVCRASNGMEASITVTVTGTQGANLAYPAGST
QPIRIEPSSSQVAEGQTLDLNCVVPGQSHAQVTWHKRGGSLPVRHQTHGSLLRLYQASPA
DSGEYVCRVLGSSVPLEASVLVTIEPAGSVPALGVTPTVRIESSSSQVAEGQTLDLNCLV
AGQAHAQVTWHKRGGSLPARHQVHGSRLRLLQVTPADSGEYVCRVVGSSGTQEASVLVTI
QQRLSGSHSQGVAYPVRIESSSASLANGHTLDLNCLVASQAPHTITWYKRGGSLPSRHQI
VGSRLRIPQVTPADSGEYVCHVSNGAGSRETSLIVTIQGSGSSHVPSVSPPIRIESSSPT
VVEGQTLDLNCVVARQPQAIITWYKRGGSLPSRHQTHGSHLRLHQMSVADSGEYVCRANN
NIDALEASIVISVSPSAGSPSAPGSSMPIRIESSSSHVAEGETLDLNCVVPGQAHAQVTW
HKRGGSLPSHHQTRGSRLRLHHVSPADSGEYVCRVMGSSGPLEASVLVTIEASGSSAVHV
PAPGGAPPIRIEPSSSRVAEGQTLDLKCVVPGQAHAQVTWHKRGGNLPARHQVHGPLLRL
NQVSPADSGEYSCQVTGSSGTLEASVLVTIEPSSPGPIPAPGLAQPIYIEASSSHVTEGQ
TLDLNCVVPGQAHAQVTWYKRGGSLPARHQTHGSQLRLHLVSPADSGEYVCRAASGPGPE
QEASFTVTVPPSEGSSYRLRSPVISIDPPSSTVQQGQDASFKCLIHDGAAPISLEWKTRN
QELEDNVHISPNGSIITIVGTRPSNHGTYRCVASNAYGVAQSVVNLSVHGPPTVSVLPEG
PVWVKVGKAVTLECVSAGEPRSSARWTRISSTPAKLEQRTYGLMDSHAVLQISSAKPSDA
GTYVCLAQNALGTAQKQVEVIVDTGAMAPGAPQVQAEEAELTVEAGHTATLRCSATGSPA
PTIHWSKLRSPLPWQHRLEGDTLIIPRVAQQDSGQYICNATSPAGHAEATIILHVESPPY
ATTVPEHASVQAGETVQLQCLAHGTPPLTFQWSRVGSSLPGRATARNELLHFERAAPEDS
GRYRCRVTNKVGSAEAFAQLLVQGPPGSLPATSIPAGSTPTVQVTPQLETKSIGASVEFH
CAVPSDRGTQLRWFKEGGQLPPGHSVQDGVLRIQNLDQSCQGTYICQAHGPWGKAQASAQ
LVIQALPSVLINIRTSVQTVVVGHAVEFECLALGDPKPQVTWSKVGGHLRPGIVQSGGVV
RIAHVELADAGQYRCTATNAAGTTQSHVLLLVQALPQISMPQEVRVPAGSAAVFPCIASG
YPTPDISWSKLDGSLPPDSRLENNMLMLPSVRPQDAGTYVCTATNRQGKVKAFAHLQVPE
RVVPYFTQTPYSFLPLPTIKDAYRKFEIKITFRPDSADGMLLYNGQKRVPGSPTNLANRQ
PDFISFGLVGGRPEFRFDAGSGMATIRHPTPLALGHFHTVTLLRSLTQGSLIVGDLAPVN
GTSQGKFQGLDLNEELYLGGYPDYGAIPKAGLSSGFIGCVRELRIQGEEIVFHDLNLTAH
GISHCPTCRDRPCQNGGQCHDSESSSYVCVCPAGFTGSRCEHSQALHCHPEACGPDATCV
NRPDGRGYTCRCHLGRSGLRCEEGVTVTTPSLSGAGSYLALPALTNTHHELRLDVEFKPL
APDGVLLFSGGKSGPVEDFVSLAMVGGHLEFRYELGSGLAVLRSAEPLALGRWHRVSAER
LNKDGSLRVNGGRPVLRSSPGKSQGLNLHTLLYLGGVEPSVPLSPATNMSAHFRGCVGEV
SVNGKRLDLTYSFLGSQGIGQCYDSSPCERQPCQHGATCMPAGEYEFQCLCRDGFKGDLC
EHEENPCQLREPCLHGGTCQGTRCLCLPGFSGPRCQQGSGHGIAESDWHLEGSGGNDAPG
QYGAYFHDDGFLAFPGHVFSRSLPEVPETIELEVRTSTASGLLLWQGVEVGEAGQGKDFI
SLGLQDGHLVFRYQLGSGEARLVSEDPINDGEWHRVTALREGRRGSIQVDGEELVSGRSP

GPNVAVNAKGSVYIGGAPDVATLTGGRFSSGITGCVKNLVLHSARPGAPPPQPLDLQHRA
QAGANTRPCPS
>sp|Q00169|PIPNA_HUMAN Phosphatidylinositol transfer protein alpha isoform OS=Homo sapiens OX=9606 GN=PITPNA PE=1 SV=2
MVLLKEYRVILPVSVDEYQVGQLYSVAEASKNETGGGEGVEVLVNEPYEKDGEKGQYTHK
IYHLQSKVPTFVRMLAPEGALNIHEKAWNAYPYCRTVITNEYMKEDFLIKIETWHKPDLG
TQENVHKLEPEAWKHVEAVYIDIADRSQVLSKDYKAEEDPAKFKSIKTGRGPLGPNWKQE
LVNQKDCPYMCAYKLVTVKFKWWGLQNKVENFIHKQERRLFTNFHRQLFCWLDKWVDLTM
DDIRRMEEETKRQLDEMRQKDPVKGMTADD
>sp|Q00534|CDK6_HUMAN Cyclin-dependent kinase 6 OS=Homo sapiens OX=9606 GN=CDK6 PE=1 SV=1
MEKDGLCRADQQYECVAEIGEGAYGKVFKARDLKNGGRFVALKRVRVQTGEEGMPLSTIR
EVAVLRHLETFEHPNVVRLFDVCTVSRTDRETKLTLVFEHVDQDLTTYLDKVPEPGVPTE
TIKDMMFQLLRGLDFLHSHRVVHRDLKPQNILVTSSGQIKLADFGLARIYSFQMALTSVV
VTLWYRAPEVLLQSSYATPVDLWSVGCIFAEMFRRKPLFRGSSDVDQLGKILDVIGLPGE
EDWPRDVALPRQAFHSKSAQPIEKFVTDIDELGKDLLLKCLTFNPAKRISAYSALSHPYF
QDLERCKENLDSHLPPSQNTSELNTA
>sp|Q00610|CLH1_HUMAN Clathrin heavy chain 1 OS=Homo sapiens OX=9606 GN=CLTC PE=1 SV=5
MAQILPIRFQEHLQLQNLGINPANIGFSTLTMESDKFICIREKVGEQAQVVIIDMNDPSN
PIRRPISADSAIMNPASKVIALKAGKTLQIFNIEMKSKMKAHTMTDDVTFWKWISLNTVA
LVTDNAVYHWSMEGESQPVKMFDRHSSLAGCQIINYRTDAKQKWLLLTGISAQQNRVVGA
MQLYSVDRKVSQPIEGHAASFAQFKMEGNAEESTLFCFAVRGQAGGKLHIIEVGTPPTGN
QPFPKKAVDVFFPPEAQNDFPVAMQISEKHDVVFLITKYGYIHLYDLETGTCIYMNRISG
ETIFVTAPHEATAGIIGVNRKGQVLSVCVEEENIIPYITNVLQNPDLALRMAVRNNLAGA
EELFARKFNALFAQGNYSEAAKVAANAPKGILRTPDTIRRFQSVPAQPGQTSPLLQYFGI
LLDQGQLNKYESLELCRPVLQQGRKQLLEKWLKEDKLECSEELGDLVKSVDPTLALSVYL
RANVPNKVIQCFAETGQVQKIVLYAKKVGYTPDWIFLLRNVMRISPDQGQQFAQMLVQDE
EPLADITQIVDVFMEYNLIQQCTAFLLDALKNNRPSEGPLQTRLLEMNLMHAPQVADAIL
GNQMFTHYDRAHIAQLCEKAGLLQRALEHFTDLYDIKRAVVHTHLLNPEWLVNYFGSLSV
EDSLECLRAMLSANIRQNLQICVQVASKYHEQLSTQSLIELFESFKSFEGLFYFLGSIVN
FSQDPDVHFKYIQAACKTGQIKEVERICRESNCYDPERVKNFLKEAKLTDQLPLIIVCDR
FDFVHDLVLYLYRNNLQKYIEIYVQKVNPSRLPVVIGGLLDVDCSEDVIKNLILVVRGQF
STDELVAEVEKRNRLKLLLPWLEARIHEGCEEPATHNALAKIYIDSNNNPERFLRENPYY
DSRVVGKYCEKRDPHLACVAYERGQCDLELINVCNENSLFKSLSRYLVRRKDPELWGSVL
LESNPYRRPLIDQVVQTALSETQDPEEVSVTVKAFMTADLPNELIELLEKIVLDNSVFSE
HRNLQNLLILTAIKADRTRVMEYINRLDNYDAPDIANIAISNELFEEAFAIFRKFDVNTS
AVQVLIEHIGNLDRAYEFAERCNEPAVWSQLAKAQLQKGMVKEAIDSYIKADDPSSYMEV
VQAANTSGNWEELVKYLQMARKKARESYVETELIFALAKTNRLAELEEFINGPNNAHIQQ
VGDRCYDEKMYDAAKLLYNNVSNFGRLASTLVHLGEYQAAVDGARKANSTRTWKEVCFAC
VDGKEFRLAQMCGLHIVVHADELEELINYYQDRGYFEELITMLEAALGLERAHMGMFTEL
AILYSKFKPQKMREHLELFWSRVNIPKVLRAAEQAHLWAELVFLYDKYEEYDNAIITMMN
HPTDAWKEGQFKDIITKVANVELYYRAIQFYLEFKPLLLNDLLMVLSPRLDHTRAVNYFS
KVKQLPLVKPYLRSVQNHNNKSVNESLNNLFITEEDYQALRTSIDAYDNFDNISLAQRLE

KHELIEFRRIAAYLFKGNNRWKQSVELCKKDSLYKDAMQYASESKDTELAEELLQWFLQE
EKRECFGACLFTCYDLLRPDVVLETAWRHNIMDFAMPYFIQVMKEYLTKVDKLDASESLR
KEEEQATETQPIVYGQPQLMLTAGPSVAVPPQAPFGYGYTAPPYGQPQPGFGYSM
>sp|Q00796|DHSO_HUMAN Sorbitol dehydrogenase OS=Homo sapiens OX=9606 GN=SORD PE=1 SV=4
MAAAAKPNNLSLVVHGPGDLRLENYPIPEPGPNEVLLRMHSVGICGSDVHYWEYGRIGNF
IVKKPMVLGHEASGTVEKVGSSVKHLKPGDRVAIEPGAPRENDEFCKMGRYNLSPSIFFC
ATPPDDGNLCRFYKHNAAFCYKLPDNVTFEEGALIEPLSVGIHACRRGGVTLGHKVLVCG
AGPIGMVTLLVAKAMGAAQVVVTDLSATRLSKAKEIGADLVLQISKESPQEIARKVEGQL
GCKPEVTIECTGAEASIQAGIYATRSGGNLVLVGLGSEMTTVPLLHAAIREVDIKGVFRY
CNTWPVAISMLASKSVNVKPLVTHRFPLEKALEAFETFKKGLGLKIMLKCDPSDQNP
>sp|Q00839|HNRPU_HUMAN Heterogeneous nuclear ribonucleoprotein U OS=Homo sapiens OX=9606 GN=HNRNPU PE=1 SV=6
MSSSPVNVKKLKVSELKEELKKRRLSDKGLKAELMERLQAALDDEEAGGRPAMEPGNGSL
DLGGDSAGRSGAGLEQEAAAGGDEEEEEEEEEEEGISALDGDQMELGEENGAAGAADSGP
MEEEEAASEDENGDDQGFQEGEDELGDEEEGAGDENGHGEQQPQPPATQQQQPQQQRGAA
KEAAGKSSGPTSLFAVTVAPPGARQGQQQAGGKKKAEGGGGGGRPGAPAAGDGKTEQKGG
DKKRGVKRPREDHGRGYFEYIEENKYSRAKSPQPPVEEEDEHFDDTVVCLDTYNCDLHFK
ISRDRLSASSLTMESFAFLWAGGRASYGVSKGKVCFEMKVTEKIPVRHLYTKDIDIHEVR
IGWSLTTSGMLLGEEEFSYGYSLKGIKTCNCETEDYGEKFDENDVITCFANFESDEVELS
YAKNGQDLGVAFKISKEVLAGRPLFPHVLCHNCAVEFNFGQKEKPYFPIPEEYTFIQNVP
LEDRVRGPKGPEEKKDCEVVMMIGLPGAGKTTWVTKHAAENPGKYNILGTNTIMDKMMVA
GFKKQMADTGKLNTLLQRAPQCLGKFIEIAARKKRNFILDQTNVSAAAQRRKMCLFAGFQ
RKAVVVCPKDEDYKQRTQKKAEVEGKDLPEHAVLKMKGNFTLPEVAECFDEITYVELQKE
EAQKLLEQYKEESKKALPPEKKQNTGSKKSNKNKSGKNQFNRGGGHRGRGGFNMRGGNFR
GGAPGNRGGYNRRGNMPQRGGGGGGSGGIGYPYPRAPVFPGRGSYSNRGNYNRGGMPNRG
NYNQNFRGRGNNRGYKNQSQGYNQWQQGQFWGQKPWSQHYHQGYY
>sp|Q01082|SPTB2_HUMAN Spectrin beta chain, non-erythrocytic 1 OS=Homo sapiens OX=9606 GN=SPTBN1 PE=1 SV=2
MTTTVATDYDNIEIQQQYSDVNNRWDVDDWDNENSSARLFERSRIKALADEREAVQKKTF
TKWVNSHLARVSCRITDLYTDLRDGRMLIKLLEVLSGERLPKPTKGRMRIHCLENVDKAL
QFLKEQRVHLENMGSHDIVDGNHRLTLGLIWTIILRFQIQDISVETEDNKEKKSAKDALL
LWCQMKTAGYPNVNIHNFTTSWRDGMAFNALIHKHRPDLIDFDKLKKSNAHYNLQNAFNL
AEQHLGLTKLLDPEDISVDHPDEKSIITYVVTYYHYFSKMKALAVEGKRIGKVLDNAIET
EKMIEKYESLASDLLEWIEQTIIILNNRKFANSLVGVQQQLQAFNTYRTVEKPPKFTEKG
NLEVLLFTIQSKMRANNQKVYMPREGKLISDINKAWERLEKAEHERELALRNELIRQEKL
EQLARRFDRKAAMRETWLSENQRLVSQDNFGFDLPAVEAATKKHEAIETDIAAYEERVQA
VVAVARELEAENYHDIKRITARKDNVIRLWEYLLELLRARRQRLEMNLGLQKIFQEMLYI
MDWMDEMKVLVLSQDYGKHLLGVEDLLQKHTLVEADIGIQAERVRGVNASAQKFATDGEG
YKPCDPQVIRDRVAHMEFCYQELCQLAAERRARLEESRRLWKFFWEMAEEEGWIREKEKI
LSSDDYGKDLTSVMRLLSKHRAFEDEMSGRSGHFEQAIKEGEDMIAEEHFGSEKIRERII
YIREQWANLEQLSAIRKKRLEEASLLHQFQADADDIDAWMLDILKIVSSSDVGHDEYSTQ
SLVKKHKDVAEEIANYRPTLDTLHEQASALPQEHAESPDVRGRLSGIEERYKEVAELTRL
RKQALQDTLALYKMFSEADACELWIDEKEQWLNNMQIPEKLEDLEVIQHRFESLEPEMNN

QASRVAVVNQIARQLMHSGHPSEKEIKAQQDKLNTRWSQFRELVDRKKDALLSALSIQNY
HLECNETKSWIREKTKVIESTQDLGNDLAGVMALQRKLTGMERDLVAIEAKLSDLQKEAE
KLESEHPDQAQAILSRLAEISDVWEEMKTTLKNREASLGEASKLQQFLRDLDDFQSWLSR
TQTAIASEDMPNTLTEAEKLLTQHENIKNEIDNYEEDYQKMRDMGEMVTQGQTDAQYMFL
RQRLQALDTGWNELHKMWENRQNLLSQSHAYQQFLRDTKQAEAFLNNQEYVLAHTEMPTT
LEGAEAAIKKQEDFMTTMDANEEKINAVVETGRRLVSDGNINSDRIQEKVDSIDDRHRKN
RETASELLMRLKDNRDLQKFLQDCQELSLWINEKMLTAQDMSYDEARNLHSKWLKHQAFM
AELASNKEWLDKIEKEGMQLISEKPETEAVVKEKLTGLHKMWEVLESTTQTKAQRLFDAN
KAELFTQSCADLDKWLHGLESQIQSDDYGKDLTSVNILLKKQQMLENQMEVRKKEIEELQ
SQAQALSQEGKSTDEVDSKRLTVQTKFMELLEPLNERKHNLLASKEIHQFNRDVEDEILW
VGERMPLATSTDHGHNLQTVQLLIKKNQTLQKEIQGHQPRIDDIFERSQNIVTDSSSLSA
EAIRQRLADLKQLWGLLIEETEKRHRRLEEAHRAQQYYFDAAEAEAWMSEQELYMMSEEK
AKDEQSAVSMLKKHQILEQAVEDYAETVHQLSKTSRALVADSHPESERISMRQSKVDKLY
AGLKDLAEERRGKLDERHRLFQLNREVDDLEQWIAEREVVAGSHELGQDYEHVTMLQERF
REFARDTGNIGQERVDTVNHLADELINSGHSDAATIAEWKDGLNEAWADLLELIDTRTQI
LAASYELHKFYHDAKEIFGRIQDKHKKLPEELGRDQNTVETLQRMHTTFEHDIQALGTQV
RQLQEDAARLQAAYAGDKADDIQKRENEVLEAWKSLLDACESRRVRLVDTGDKFRFFSMV
RDLMLWMEDVIRQIEAQEKPRDVSSVELLMNNHQGIKAEIDARNDSFTTCIELGKSLLAR
KHYASEEIKEKLLQLTEKRKEMIDKWEDRWEWLRLILEVHQFSRDASVAEAWLLGQEPYL
SSREIGQSVDEVEKLIKRHEAFEKSAATWDERFSALERLTTLELLEVRRQQEEEERKRRP
PSPEPSTKVSEEAESQQQWDTSKGEQVSQNGLPAEQGSPRMAETVDTSEMVNGATEQRTS
SKESSPIPSPTSDRKAKTALPAQSAATLPARTQETPSAQMEGFLNRKHEWEAHNKKASSR
SWHNVYCVINNQEMGFYKDAKTAASGIPYHSEVPVSLKEAVCEVALDYKKKKHVFKLRLN
DGNEYLFQAKDDEEMNTWIQAISSAISSDKHEVSASTQSTPASSRAQTLPTSVVTITSES
SPGKREKDKEKDKEKRFSLFGKKK
>sp|Q01469|FABP5_HUMAN Fatty acid-binding protein 5 OS=Homo sapiens OX=9606
GN=FABP5 PE=1 SV=3
MATVQQLEGRWRLVDSKGFDEYMKELGVGIALRKMGAMAKPDCIITCDGKNLTIKTESTL
KTTQFSCTLGEKFEETTADGRKTQTVCNFTDGALVQHQEWDGKESTITRKLKDGKLVVEC
VMNNVTCTRIYEKVE
>sp|Q01484|ANK2_HUMAN Ankyrin-2 OS=Homo sapiens OX=9606 GN=ANK2 PE=1 SV=4
MMNEDAAQKSDSGEKFNGSSQRRKRPKKSDSNASFLRAARAGNLDKVVEYLKGGIDINTC
NQNGLNALHLAAKEGHVGLVQELLGRGSSVDSATKKGNTALHIASLAGQAEVVKVLVKEG
ANINAQSQNGFTPLYMAAQENHIDVVKYLLENGANQSTATEDGFTPLAVALQQGHNQAVA
ILLENDTKGKVRLPALHIAARKDDTKSAALLLQNDHNADVQSKMMVNRTTESGFTPLHIA
AHYGNVNVATLLLNRGAAVDFTARNGITPLHVASKRGNTNMVKLLLDRGGQIDAKTRDGL
TPLHCAARSGHDQVVELLLERGAPLLARTKNGLSPLHMAAQGDHVECVKHLLQHKAPVDD
VTLDYLTALHVAAHCGHYRVTKLLLDKRANPNARALNGFTPLHIACKKNRIKVMELLVKY
GASIQAITESGLTPIHVAAFMGHLNIVLLLLQNGASPDVTNIRGETALHMAARAGQVEVV
RCLLRNGALVDARAREEQTPLHIASRLGKTEIVQLLLQHMAHPDAATTNGYTPLHISARE
GQVDVASVLLEAGAAHSLATKKGFTPLHVAAKYGSLDVAKLLLQRRAAADSAGKNGLTPL
HVAAHYDNQKVALLLLEKGASPHATAKNGYTPLHIAAKKNQMQIASTLLNYGAETNIVTK
QGVTPLHLASQEGHTDMVTLLLDKGANIHMSTKSGLTSLHLAAQEDKVNVADILTKHGAD
QDAHTKLGYTPLIVACHYGNVKMVNFLLKQGANVNAKTKNGYTPLHQAAQQGHTHIINVL

LQHGAKPNATTANGNTALAIAKRLGYISVVDTLKVVTEEVTTTTTTITEKHKLNVPETMT
EVLDVSDEEGDDTMTGDGGEYLRPEDLKELGDDSLPSSQFLDGMNYLRYSLEGGRSDSLR
SFSSDRSHTLSHASYLRDSAVMDDSVVIPSHQVSTLAKEAERNSYRLSWGTENLDNVALS
SSPIHSGFLVSFMVDARGGAMRGCRHNGLRIIIPPRKCTAPTRVTCRLVKRHRLATMPPM
VEGEGLASRLIEVGPSGAQFLGKLHLPTAPPPLNEGESLVSRILQLGPPGTKFLGPVIVE
IPHFAALRGKERELVVLRSENGDSWKEHFCDYTEDELNEILNGMDEVLDSPEDLEKKRIC
RIITRDFPQYFAVVSRIKQDSNLIGPEGGVLSSTVVPQVQAVFPEGALTKRIRVGLQAQP
MHSELVKKILGNKATFSPIVTLEPRRRKFHKPITMTIPVPKASSDVMLNGFGGDAPTLRL
LCSITGGTTPAQWEDITGTTPLTFVNECVSFTTNVSARFWLIDCRQIQESVTFASQVYRE
IICVPYMAKFVVFAKSHDPIEARLRCFCMTDDKVDKTLEQQENFAEVARSRDVEVLEGKP
IYVDCFGNLVPLTKSGQHHIFSFFAFKENRLPLFVKVRDTTQEPCGRLSFMKEPKSTRGL
VHQAICNLNITLPIYTKESESDQEQEEEIDMTSEKNDETESTETSVLKSHLVNEVPVLAS
PDLLSEVSEMKQDLIKMTAILTTDVSDKAGSIKVKELVKAAEEEPGEPFEIVERVKEDLE
KVNEILRSGTCTRDESSVQSSRSERGLVEEEWVIVSDEEIEEARQKAPLEITEYPCVEVR
IDKEIKGKVEKDSTGLVNYLTDDLNTCVPLPKEQLQTVQDKAGKKCEALAVGRSSEKEGK
DIPPDETQSTQKQHKPSLGIKKPVRRKLKEKQKQKEEGLQASAEKAELKKGSSEESLGED
PGLAPEPLPTVKATSPLIEETPIGSIKDKVKALQKRVEDEQKGRSKLPIRVKGKEDVPKK
TTHRPHPAASPSLKSERHAPGSPSPKTERHSTLSSSAKTERHPPVSPSSKTEKHSPVSPS
AKTERHSPASSSSKTEKHSPVSPSTKTERHSPVSSTKTERHPPVSPSGKTDKRPPVSPSG
RTEKHPPVSPGRTEKRLPVSPSGRTDKHQPVSTAGKTEKHLPVSPSGKTEKQPPVSPTSK
TERIEETMSVRELMKAFQSGQDPSKHKTGLFEHKSAKQKQPQEKGKVRVEKEKGPILTQR
EAQKTENQTIKRGQRLPVTGTAESKRGVRVSSIGVKKEDAAGGKEKVLSHKIPEPVQSVP
EEESHRESEVPKEKMADEQGDMDLQISPDRKTSTDFSEVIKQELEDNDKYQQFRLSEETE
KAQLHLDQVLTSPFNTTFPLDYMKDEFLPALSLQSGALDGSSESLKNEGVAGSPCGSLME
GTPQISSEESYKHEGLAETPETSPESLSFSPKKSEEQTGETKESTKTETTTEIRSEKEHP
TTKDITGGSEERGATVTEDSETSTESFQKEATLGSPKDTSPKRQDDCTGSCSVALAKETP
TGLTEEAACDEGQRTFGSSAHKTQTDSEVQESTATSDETKALPLPEASVKTDTGTESKPQ
GVIRSPQGLELALPSRDSEVLSAVADDSLAVSHKDSLEASPVLEDNSSHKTPDSLEPSPL
KESPCRDSLESSPVEPKMKAGIFPSHFPLPAAVAKTELLTEVASVRSRLLRDPDGSAEDD
SLEQTSLMESSGKSPLSPDTPSSEEVSYEVTPKTTDVSTPKPAVIHECAEEDDSENGEKK
RFTPEEEMFKMVTKIKMFDELEQEAKQKRDYKKEPKQEESSSSSDPDADCSVDVDEPKHT
GSGEDESGVPVLVTSESRKVSSSSESEPELAQLKKGADSGLLPEPVIRVQPPSPLPSSMD
SNSSPEEVQFQPVVSKQYTFKMNEDTQEEPGKSEEEKDSESHLAEDRHAVSTEAEDRSYD
KLNRDTDQPKICDGHGCEAMSPSSSAAPVSSGLQSPTGDDVDEQPVIYKESLALQGTHEK
DTEGEELDVSRAESPQADCPSESFSSSSSLPHCLVSEGKELDEDISATSSIQKTEVTKTD
ETFENLPKDCPSQDSSITTQTDRFSMDVPVSDLAENDEIYDPQITSPYENVPSQSFFSSE
ESKTQTDANHTTSFHSSEVYSVTITSPVEDVVVASSSSGTVLSKESNFEGQDIKMESQQE
STLWEMQSDSVSSSFEPTMSATTTVVGEQISKVIITKTDVDSDSWSEIREDDEAFEARVK
EEEQKIFGLMVDRQSQGTTPDTTPARTPTEEGTPTSEQNPFLFQEGKLFEMTRSGAIDMT
KRSYADESFHFFQIGQESREETLSEDVKEGATGADPLPLETSAESLALSESKETVDDEAD
LLPDDVSEEVEEIPASDAQLNSQMGISASTETPTKEAVSVGTKDLPTVQTGDIPPLSGVK
QISCPDSSEPAVQVQLDFSTLTRSVYSDRGDDSPDSSPEEQKSVIEIPTAPMENVPFTES
KSKIPVRTMPTSTPAPPSAEYESSVSEDFLSSVDEENKADEAKPKSKLPVKVPLQRVEQQ
LSDLDTSVQKTVAPQGQDMASIAPDNRSKSESDASSLDSKTKCPVKTRSYTETETESRER

AEELELESEEGATRPKILTSRLPVKSRSTTSSCRGGTSPTKESKEHFFDLYRNSIEFFEE
ISDEASKLVDRLTQSEREQEIVSDDESSSALEVSVIENLPPVETEHSVPEDIFDTRPIWD
ESIETLIERIPDENGHDHAEDPQDEQERIEERLAYIADHLGFSWTELARELDFTEEQIHQ
IRIENPNSLQDQSHALLKYWLERDGKHATDTNLVECLTKINRMDIVHLMETNTEPLQERI
SHSYAEIEQTITLDHSEGFSVLQEELCTAQHKQKEEQAVSKESETCDHPPIVSEEDISVG
YSTFQDGVPKTEGDSSATALFPQTHKEQVQQDFSGKMQDLPEESSLEYQQEYFVTTPGTE
TSETQKAMIVPSSPSKTPEEVSTPAEEEKLYLQTPTSSERGGSPIIQEPEEPSEHREESS
PRKTSLVIVESADNQPETCERLDEDAAFEKGDDMPEIPPETVTEEEYIDEHGHTVVKKVT
RKIIRRYVSSEGTEKEEIMVQGMPQEPVNIEEGDGYSKVIKRVVLKSDTEQSEDNNE
>sp|Q01518|CAP1_HUMAN Adenylyl cyclase-associated protein 1 OS=Homo sapiens OX=9606
GN=CAP1 PE=1 SV=5
MADMQNLVERLERAVGRLEAVSHTSDMHRGYADSPSKAGAAPYVQAFDSLLAGPVAEYLK
ISKEIGGDVQKHAEMVHTGLKLERALLVTASQCQQPAENKLSDLLAPISEQIKEVITFRE
KNRGSKLFNHLSAVSESIQALGWVAMAPKPGPYVKEMNDAAMFYTNRVLKEYKDVDKKHV
DWVKAYLSIWTELQAYIKEFHTTGLAWSKTGPVAKELSGLPSGPSAGSCPPPPPPCPPPP
PVSTISCSYESASRSSLFAQINQGESITHALKHVSDDMKTHKNPALKAQSGPVRSGPKPF
SAPKPQTSPSPKRATKKEPAVLELEGKKWRVENQENVSNLVIEDTELKQVAYIYKCVNTT
LQIKGKINSITVDNCKKLGLVFDDVVGIVEIINSKDVKVQVMGKVPTISINKTDGCHAYL
SKNSLDCEIVSAKSSEMNVLIPTEGGDFNEFPVPEQFKTLWNGQKLVTTVTEIAG
>sp|Q01813|PFKAP_HUMAN ATP-dependent 6-phosphofructokinase, platelet type OS=Homo
sapiens OX=9606 GN=PFKP PE=1 SV=2
MDADDSRAPKGSLRKFLEHLSGAGKAIGVLTSGGDAQGMNAAVRAVVRMGIYVGAKVYFI
YEGYQGMVDGGSNIAEADWESVSSILQVGGTIIGSARCQAFRTREGRLKAACNLLQRGIT
NLCVIGGDGSLTGANLFRKEWSGLLEELARNGQIDKEAVQKYAYLNVVGMVGSIDNDFCG
TDMTIGTDSALHRIIEVVDAIMTTAQSHQRTFVLEVMGRHCGYLALVSALACGADWVFLP
ESPPEEGWEEQMCVKLSENRARKKRLNIIIVAEGAIDTQNKPITSEKIKELVVTQLGYDT
RVTILGHVQRGGTPSAFDRILASRMGVEAVIALLEATPDTPACVVSLNGNHAVRLPLMEC
VQMTQDVQKAMDERRFQDAVRLRGRSFAGNLNTYKRLAIKLPDDQIPKTNCNVAVINVGA
PAAGMNAAVRSAVRVGIADGHRMLAIYDGFDGFAKGQIKEIGWTDVGGWTGQGGSILGTK
RVLPGKYLEEIATQMRTHSINALLIIGGFEAYLGLLELSAAREKHEEFCVPMVMVPATVS
NNVPGSDFSIGADTALNTITDTCDRIKQSASGTKRRVFIIETMGGYCGYLANMGGLAAGA
DAAYIFEEPFDIRDLQSNVEHLTEKMKTTIQRGLVLRNESCSENYTTDFIYQLYSEEGKG
VFDCRKNVLGHMQQGGAPSPFDRNFGTKISARAMEWITAKLKEARGRGKKFTTDDSICVL
GISKRNVIFQPVAELKKQTDFEHRIPKEQWWLKLRPLMKILAKYKASYDVSDSGQLEHVQ
PWSV
>sp|Q01955|CO4A3_HUMAN Collagen alpha-3(IV) chain OS=Homo sapiens OX=9606
GN=COL4A3 PE=1 SV=3
MSARTAPRPQVLLLPLLLVLLAAAPAASKGCVCKDKGQCFCDGAKGEKGEKGFPGPPGSP
GQKGFTGPEGLPGPQGPKGFPGLPGLTGSKGVRGISGLPGFSGSPGLPGTPGNTGPYGLV
GVPGCSGSKGEQGFPGLPGTLGYPGIPGAAGLKGQKGAPAKEEDIELDAKGDPGLPGAPG
PQGLPGPPGFPGPVGPPGPPGFFGFPGAMGPRGPKGHMGERVIGHKGERGVKGLTGPPGP
PGTVIVTLTGPDNRTDLKGEKGDKGAMGEPGPPGPSGLPGESYGSEKGAPGDPGLQGKPG
KDGVPGFPGSEGVKGNRGFPGLMGEDGIKGQKGDIGPPGFRGPTEYYDTYQEKGDEGTPG
PPGPRGARGPQGPSGPPGVPGSPGSSRPGLRGAPGWPGLKGSKGERGRPGKDAMGTPGSP

GCAGSPGLPGSPGPPGPPGDIVFRKGPPGDHGLPGYLGSPGIPGVDGPKGEPGLLCTQCP
YIPGPPGLPGLPGLHGVKGIPGRQGAAGLKGSPGSPGNTGLPGFPGFPGAQGDPGLKGEK
GETLQPEGQVGVPGDPGLRGQPGRKGLDGIPGTPGVKGLPGPKGELALSGEKGDQGPPGD
PGSPGSPGPAGPAGPPGYGPQGEPGLQGTQGVPGAPGPPGEAGPRGELSVSTPVPGPPGP
PGPPGHPGPQGPPGIPGSLGKCGDPGLPGPDGEPGIPGIGFPGPPGPKGDQGFPGTKGSL
GCPGKMGEPGLPGKPGLPGAKGEPAVAMPGGPGTPGFPGERGNSGEHGEIGLPGLPGLPG
TPGNEGLDGPRGDPGQPGPPGEQGPPGRCIEGPRGAQGLPGLNGLKGQQGRRGKTGPKGD
PGIPGLDRSGFPGETGSPGIPGHQGEMGPLGQRGYPGNPGILGPPGEDGVIGMMGFPGAI
GPPGPPGNPGTPGQRGSPGIPGVKGQRGTPGAKGEQGDKGNPGPSEISHVIGDKGEPGLK
GFAGNPGEKGNRGVPGMPGLKGLKGLPGPAGPPGPRGDLGSTGNPGEPGLRGIPGSMGNM
GMPGSKGKRGTLGFPGRAGRPGLPGIHGLQGDKGEPGYSEGTRPGPPGPTGDPGLPGDMG
KKGEMGQPGPPGHLGPAGPEGAPGSPGSPGLPGKPGPHGDLGFKGIKGLLGPPGIRGPPG
LPGFPGSPGPMGIRGDQGRDGIPGPAGEKGETGLLRAPPGPRGNPGAQGAKGDRGAPGFP
GLPGRKGAMGDAGPRGPTGIEGFPGPPGLPGAIIPGQTGNRGPPGSRGSPGAPGPPGPPG
SHVIGIKGDKGSMGHPGPKGPPGTAGDMGPPGRLGAPGTPGLPGPRGDPGFQGFPGVKGE
KGNPGFLGSIGPPGPIGPKGPPGVRGDPGTLKIISLPGSPGPPGTPGEPGMQGEPGPPGP
PGNLGPCGPRGKPGKDGKPGTPGPAGEKGNKGSKGEPGPAGSDGLPGLKGKRGDSGSPAT
WTTRGFVFTRHSQTTAIPSCPEGTVPLYSGFSFLFVQGNQRAHGQDLGTLGSCLQRFTTM
PFLFCNVNDVCNFASRNDYSYWLSTPALMPMNMAPITGRALEPYISRCTVCEGPAIAIAV
HSQTTDIPPCPHGWISLWKGFSFIMFTSAGSEGTGQALASPGSCLEEFRASPFLECHGRG
TCNYYSNSYSFWLASLNPERMFRKPIPSTVKAGELEKIISRCQVCMKKRH
>sp|Q02413|DSG1_HUMAN Desmoglein-1 OS=Homo sapiens OX=9606 GN=DSG1 PE=1 SV=2
MDWSFFRVVAMLFIFLVVVEVNSEFRIQVRDYNTKNGTIKWHSIRRQKREWIKFAAACRE
GEDNSKRNPIAKIHSDCAANQQVTYRISGVGIDQPPYGIFVINQKTGEINITSIVDREVT
PFFIIYCRALNSMGQDLERPLELRVRVLDINDNPPVFSMATFAGQIEENSNANTLVMILN
ATDADEPNNLNSKIAFKIIRQEPSDSPMFIINRNTGEIRTMNNFLDREQYGQYALAVRGS
DRDGGADGMSAECECNIKILDVNDNIPYMEQSSYTIEIQENTLNSNLLEIRVIDLDEEFS
ANWMAVIFFISGNEGNWFEIEMNERTNVGILKVVKPLDYEAMQSLQLSIGVRNKAEFHHS
IMSQYKLKASAISVTVLNVIEGPVFRPGSKTYVVTGNMGSNDKVGDFVATDLDTGRPSTT
VRYVMGNNPADLLAVDSRTGKLTLKNKVTKEQYNMLGGKYQGTILSIDDNLQRTCTGTIN
INIQSFGNDDRTNTEPNTKITTNTGRQESTSSTNYDTSTTSTDSSQVYSSEPGNGAKDLL
SDNVHFGPAGIGLLIMGFLVLGLVPFLMICCDCGGAPRSAAGFEPVPECSDGAIHSWAVE
GPQPEPRDITTVIPQIPPDNANIIECIDNSGVYTNEYGGREMQDLGGGERMTGFELTEGV
KTSGMPEICQEYSGTLRRNSMRECREGGLNMNFMESYFCQKAYAYADEDEGRPSNDCLLI
YDIEGVGSPAGSVGCCSFIGEDLDDSFLDTLGPKFKKLADISLGKESYPDLDPSWPPQST
EPVCLPQETEPVVSGHPPISPHFGTTTVISESTYPSGPGVLHPKPILDPLGYGNVTVTES
YTTSDTLKPSVHVHDNRPASNVVVTERVVGPISGADLHGMLEMPDLRDGSNVIVTERVIA
PSSSLPTSLTIHHPRESSNVVVTERVIQPTSGMIGSLSMHPELANAHNVIVTERVVSGAG
VTGISGTTGISGGIGSSGLVGTSMGAGSGALSGAGISGGGIGLSSLGGTASIGHMRSSSD
HHFNQTIGSASPSTARSRITKYSTVQYSK
>sp|Q02790|FKBP4_HUMAN Peptidyl-prolyl cis-trans isomerase FKBP4 OS=Homo sapiens
OX=9606 GN=FKBP4 PE=1 SV=3
MTAEEMKATESGAQSAPLPMEGVDISPKQDEGVLKVIKREGTGTEMPMIGDRVFVHYTGW
LLDGTKFDSSLDRKDKFSFDLGKGEVIKAWDIAIATMKVGEVCHITCKPEYAYGSAGSPP

KIPPNATLVFEVELFEFKGEDLTEEEDGGIIRRIQTRGEGYAKPNEGAIVEVALEGYYKD
KLFDQRELRFEIGEGENLDLPYGLERAIQRMEKGEHSIVYLKPSYAFGSVGKEKFQIPPN
AELKYELHLKSFEKAKESWEMNSEEKLEQSTIVKERGTVYFKEGKYKQALLQYKKIVSWL
EYESSFSNEEAQKAQALRLASHLNLAMCHLKLQAFSAAIESCNKALELDSNNEKGLFRRG
EAHLAVNDFELARADFQKVLQLYPNNKAAKTQLAVCQQRIRRQLAREKKLYANMFERLAE
EENKAKAEASSGDHPTDTEMKEEQKSNTAGSQSQVETEA
>sp|Q02878|RL6_HUMAN Large ribosomal subunit protein eL6 OS=Homo sapiens OX=9606 GN=RPL6 PE=1 SV=3
MAGEKVEKPDTKEKKPEAKKVDAGGKVKKGNLKAKKPKKGKPHCSRNPVLVRGIGRYSRS
AMYSRKAMYKRKYSAAKSKVEKKKKEKVLATVTKPVGGDKNGGTRVVKLRKMPRYYPTED
VPRKLLSHGKKPFSQHVRKLRASITPGTILIILTGRHRGKRVVFLKQLASGLLLVTGPLV
LNRVPLRRTHQKFVIATSTKIDISNVKIPKHLTDAYFKKKKLRKPRHQEGEIFDTEKEKY
EITEQRKIDQKAVDSQILPKIKAIPQLQGYLRSVFALTNGIYPHKLVF
>sp|Q02978|M2OM_HUMAN Mitochondrial 2-oxoglutarate/malate carrier protein OS=Homo sapiens OX=9606 GN=SLC25A11 PE=1 SV=3
MAATASAGAGGIDGKPRTSPKSVKFLFGGLAGMGATVFVQPLDLVKNRMQLSGEGAKTRE
YKTSFHALTSILKAEGLRGIYTGLSAGLLRQATYTTTRLGIYTVLFERLTGADGTPPGFL
LKAVIGMTAGATGAFVGTPAEVALIRMTADGRLPADQRRGYKNVFNALIRITREEGVLTL
WRGCIPTMARAVVVNAAQLASYSQSKQFLLDSGYFSDNILCHFCASMISGLVTTAASMPV
DIAKTRIQNMRMIDGKPEYKNGLDVLFKVVRYEGFFSLWKGFTPYYARLGPHTVLTFIFL
EQMNKAYKRLFLSG
>sp|Q03013|GSTM4_HUMAN Glutathione S-transferase Mu 4 OS=Homo sapiens OX=9606 GN=GSTM4 PE=1 SV=3
MSMTLGYWDIRGLAHAIRLLLEYTDSSYEEKKYTMGDAPDYDRSQWLNEKFKLGLDFPNL
PYLIDGAHKITQSNAILCYIARKHNLCGETEEEKIRVDILENQAMDVSNQLARVCYSPDF
EKLKPEYLEELPTMMQHFSQFLGKRPWFVGDKITFVDFLAYDVLDLHRIFEPNCLDAFPN
LKDFISRFEGLEKISAYMKSSRFLPKPLYTRVAVWGNK
>sp|Q03135|CAV1_HUMAN Caveolin-1 OS=Homo sapiens OX=9606 GN=CAV1 PE=1 SV=4
MSGGKYVDSEGHLYTVPIREQGNIYKPNNKAMADELSEKQVYDAHTKEIDLVNRDPKHLN
DDVVKIDFEDVIAEPEGTHSFDGIWKASFTTFTVTKYWFYRLLSALFGIPMALIWGIYFA
ILSFLHIWAVVPCIKSFLIEIQCISRVYSIYVHTVCDPLFEAVGKIFSNVRINLQKEI
>sp|Q04446|GLGB_HUMAN 1,4-alpha-glucan-branching enzyme OS=Homo sapiens OX=9606 GN=GBE1 PE=1 SV=3
MAAPMTPAARPEDYEAALNAALADVPELARLLEIDPYLKPYAVDFQRRYKQFSQILKNIG
ENEGGIDKFSRGYESFGVHRCADGGLYCKEWAPGAEGVFLTGDFNGWNPFSYPYKKLDYG
KWELYIPPKQNKSVLVPHGSKLKVVITSKSGEILYRISPWAKYVVREGDNVNYDWIHWDP
EHSYEFKHSRPKKPRSLRIYESHVGISSHEGKVASYKHFTCNVLPRIKGLGYNCIQLMAI
MEHAYYASFGYQITSFFAASSRYGTPEELQELVDTAHSMGIIVLLDVVHSHASKNSADGL
NMFDGTDSCYFHSGPRGTHDLWDSRLFAYSSWEILRFLLSNIRWWLEEYRFDGFRFDGVT
SMLYHHHGVGQGFSGDYSEYFGLQVDEDALTYLMLANHLVHTLCPDSITIAEDVSGMPAL
CSPISQGGGGFDYRLAMAIPDKWIQLLKEFKDEDWNMGDIVYTLTNRRYLEKCIAYAESH
DQALVGDKSLAFWLMDAEMYTNMSVLTPFTPVIDRGIQLHKMIRLITHGLGGEGYLNFMG
NEFGHPEWLDFPRKGNNESYHYARRQFHLTDDDLLRYKFLNNFDRDMNRLEERYGWLAAP
QAYVSEKHEGNKIIAFERAGLLFIFNFHPSKSYTDYRVGTALPGKFKIVLDSDAAEYGGH

QRLDHSTDFFSEAFEHNGRPYSLLVYIPSRVALILQNVDLPN
>sp|Q04695|K1C17_HUMAN Keratin, type I cytoskeletal 17 OS=Homo sapiens OX=9606 GN=KRT17 PE=1 SV=2
MTTSIRQFTSSSSIKGSSGLGGGSSRTSCRLSGGLGAGSCRLGSAGGLGSTLGGSSYSSC
YSFGSGGGYGSSFGGVDGLLAGGEKATMQNLNDRLASYLDKVRALEEANTELEVKIRDWY
QRQAPGPARDYSQYYRTIEELQNKILTATVDNANILLQIDNARLAADDFRTKFETEQALR
LSVEADINGLRRVLDELTLARADLEMQIENLKEELAYLKKNHEEEMNALRGQVGGEINVE
MDAAPGVDLSRILNEMRDQYEKMAEKNRKDAEDWFFSKTEELNREVATNSELVQSGKSEI
SELRRTMQALEIELQSQLSMKASLEGNLAETENRYCVQLSQIQGLIGSVEEQLAQLRCEM
EQQNQEYKILLDVKTRLEQEIATYRRLLEGEDAHLTQYKKEPVTTRQVRTIVEEVQDGKV
ISSREQVHQTTR
>sp|Q04760|LGUL_HUMAN Lactoylglutathione lyase OS=Homo sapiens OX=9606 GN=GLO1 PE=1 SV=4
MAEPQPPSGGLTDEAALSCCSDADPSTKDFLLQQTMLRVKDPKKSLDFYTRVLGMTLIQK
CDFPIMKFSLYFLAYEDKNDIPKEKDEKIAWALSRKATLELTHNWGTEDDETQSYHNGNS
DPRGFGHIGIAVPDVYSACKRFEELGVKFVKKPDDGKMKGLAFIQDPDGYWIEILNPNKM
ATLM
>sp|Q04917|1433F_HUMAN 14-3-3 protein eta OS=Homo sapiens OX=9606 GN=YWHAH PE=1 SV=4
MGDREQLLQRARLAEQAERYDDMASAMKAVTELNEPLSNEDRNLLSVAYKNVVGARRSSW
RVISSIEQKTMADGNEKKLEKVKAYREKIEKELETVCNDVLSLLDKFLIKNCNDFQYESK
VFYLKMKGDYYRYLAEVASGEKKNSVVEASEAAYKEAFEISKEQMQPTHPIRLGLALNFS
VFYYEIQNAPEQACLLAKQAFDDAIAELDTLNEDSYKDSTLIMQLLRDNLTLWTSDQQDE
EAGEGN
>sp|Q06830|PRDX1_HUMAN Peroxiredoxin-1 OS=Homo sapiens OX=9606 GN=PRDX1 PE=1 SV=1
MSSGNAKIGHPAPNFKATAVMPDGQFKDISLSDYKGKYVVFFFYPLDFTFVCPTEIIAFS
DRAEEFKKLNCQVIGASVDSHFCHLAWVNTPKKQGGLGPMNIPLVSDPKRTIAQDYGVLK
ADEGISFRGLFIIDDKGILRQITVNDLPVGRSVDETLRLVQAFQFTDKHGEVCPAGWKPG
SDTIKPDVQKSKEYFSKQK
>sp|Q07020|RL18_HUMAN Large ribosomal subunit protein eL18 OS=Homo sapiens OX=9606 GN=RPL18 PE=1 SV=2
MGVDIRHNKDRKVRRKEPKSQDIYLRLLVKLYRFLARRTNSTFNQVVLKRLFMSRTNRPP
LSLSRMIRKMKLPGRENKTAVVVGTITDDVRVQEVPKLKVCALRVTSRARSRILRAGGKI
LTFDQLALDSPKGCGTVLLSGPRKGREVYRHFGKAPGTPHSHTKPYVRSKGRKFERARGR
RASRGYKN
>sp|Q07283|TRHY_HUMAN Trichohyalin OS=Homo sapiens OX=9606 GN=TCHH PE=1 SV=2
MSPLLRSICDITEIFNQYVSHDCDGAALTKKDLKNLLEREFGAVLRRPHDPKTVDLILEL
LDLDSNGRVDFNEFLLFIFKVAQACYYALGQATGLDEEKRARCDGKESLLQDRRQEEDQR
RFEPRDRQLEEEPGQRRRQKRQEQERELAEGEEQSEKQERLEQRDRQRRDEELWRQRQEW
QEREERRAEEEQLQSCKGHETEEFPDEEQLRRRELLELRRKGREEKQQQRRERQDRVFQE
EEEKEWRKRETVLRKEEEKLQEEEPQRQRELQEEEEQLRKLERQELRRERQEEEQQQQRL
RREQQLRRKQEEERREQQEERREQQERREQQEERREQQLRREQEERREQQLRREQEEERR
EQQLRREQEEERREQQLRREQQLRREQQLRREQQLRREQQLRREQQLRREQQLRREQQLR

REQQLRREQEEERHEQKHEQERREQRLKREQEERRDWLKREEETERHEQERRKQQLKRDQ
EEERRERWLKLEEEERREQQERREQQLRREQEERREQRLKRQEEEERLQQRLRSEQQLRR
EQEERREQLLKREEEKRLEQERREQRLKREQEERRDQLLKREEERRQQRLKREQEERLEQ
RLKREEVERLEQEERREQRLKREEPEEERRQQLLKSEEQEERRQQQLRREQQERREQRLK
REEEEERLEQRLKREHEEERREQELAEEEQEQARERIKSRIPKWQWQLESEADARQSKVY
SRPRKQEGQRRRQEQEEKRRRRESELQWQEEERAHRQQQEEEQRRDFTWQWQAEEKSERG
RQRLSARPPLREQRERQLRAEERQQREQRFLPEEEEKEQRRRQRREREKELQFLEEEEQL
QRRERAQQLQEEEDGLQEDQERRRSQEQRRDQKWRWQLEEERKRRRHTLYAKPALQEQLR
KEQQLLQEEEEELQREEREKRRRQEQERQYREEEQLQQEEEQLLREEREKRRRQERERQY
RKDKKLQQKEEQLLGEEPEKRRRQEREKKYREEEELQQEEEQLLREEREKRRRQEWERQY
RKKDELQQEEEQLLREEREKRRLQERERQYREEEELQQEEEQLLGEERETRRRQELERQY
RKEEELQQEEEQLLREEPEKRRRQERERQCREEEELQQEEEQLLREEREKRRRQELERQY
REEEEVQQEEEQLLREEPEKRRRQELERQYREEEELQQEEEQLLREEQEKRRQERERQYR
EEEELQRQKRKQRYRDEDQRSDLKWQWEPEKENAVRDNKVYCKGRENEQFRQLEDSQLRD
RQSQQDLQHLLGEQQERDREQERRRWQQRDRHFPEEEQLEREEQKEAKRRDRKSQEEKQL
LREEREEKRRRQETDRKFREEEQLLQEREEQPLRRQERDRKFREEELRHQEQGRKFLEEE
QRLRRQERERKFLKEEQQLRCQEREQQLRQDRDRKFREEEQQLSRQERDRKFREEEQQVR
RQERERKFLEEEQQLRQERHRKFREEEQLLQEREEQQLHRQERDRKFLEEEQQLRRQERD
RKFREQELRSQEPERKFLEEEQQLHRQQRQRKFLQEEQQLRRQERGQQRRQDRDRKFREE
EQLRQEREEQQLSRQERDRKFRLEEQKVRRQEQERKFMEDEQQLRRQEGQQQLRQERDRK
FREDEQLLQEREEQQLHRQERDRKFLEEEPQLRRQEREQQLRHDRDRKFREEEQLLQEGE
EQQLRRQERDRKFREEEQQLRRQERERKFLQEEQQLRRQELERKFREEEQLRQETEQEQL
RRQERYRKILEEEQLRPEREEQQLRRQERDRKFREEEQLRQEREEQQLRSQESDRKFREE
EQLRQEREEQQLRPQQRDGKYRWEEEQLQLEEQEQRLRQERDRQYRAEEQFATQEKSRRE
EQELWQEEEQKRRQERERKLREEHIRRQQKEEQRHRQVGEIKSQEGKGHGRLLEPGTHQF
ASVPVRSSPLYEYIQEQRSQYRP
>sp|Q07960|RHG01_HUMAN Rho GTPase-activating protein 1 OS=Homo sapiens OX=9606
GN=ARHGAP1 PE=1 SV=1
MDPLSELQDDLTLDDTSEALNQLKLASIDEKNWPSDEMPDFPKSDDSKSSSPELVTHLKW
DDPYYDIARHQIVEVAGDDKYGRKIIVFSACRMPPSHQLDHSKLLGYLKHTLDQYVESDY
TLLYLHHGLTSDNKPSLSWLRDAYREFDRKYKKNIKALYIVHPTMFIKTLLILFKPLISF
KFGQKIFYVNYLSELSEHVKLEQLGIPRQVLKYDDFLKSTQKSPATAPKPMPPRPPLPNQ
QFGVSLQHLQEKNPEQEPIPIVLRETVAYLQAHALTTEGIFRRSANTQVVREVQQKYNMG
LPVDFDQYNELHLPAVILKTFLRELPEPLLTFDLYPHVVGFLNIDESQRVPATLQVLQTL
PEENYQVLRFLTAFLVQISAHSDQNKMTNTNLAVVFGPNLLWAKDAAITLKAINPINTFT
KFLLDHQGELFPSPDPSGL
>sp|Q08257|QOR_HUMAN Quinone oxidoreductase OS=Homo sapiens OX=9606 GN=CRYZ
PE=1 SV=1
MATGQKLMRAVRVFEFGGPEVLKLRSDIAVPIPKDHQVLIKVHACGVNPVETYIRSGTYS
RKPLLPYTPGSDVAGVIEAVGDNASAFKKGDRVFTSSTISGGYAEYALAADHTVYKLPEK
LDFKQGAAIGIPYFTAYRALIHSACVKAGESVLVHGASGGVGLAACQIARAYGLKILGTA
GTEEGQKIVLQNGAHEVFNHREVNYIDKIKKYVGEKGIDIIIEMLANVNLSKDLSLLSHG
GRVIVVGSRGTIEINPRDTMAKESSIIGVTLFSSTKEEFQQYAAALQAGMEIGWLKPVIG
SQYPLEKVAEAHENIIHGSGATGKMILLL

>sp|Q08357|S20A2_HUMAN Sodium-dependent phosphate transporter 2 OS=Homo sapiens OX=9606 GN=SLC20A2 PE=1 SV=1
MAMDEYLWMVILGFIIAFILAFSVGANDVANSFGTAVGSGVVTLRQACILASIFETTGSV
LLGAKVGETIRKGIIDVNLYNETVETLMAGEVSAMVGSAVWQLIASFLRLPISGTHCIVG
STIGFSLVAIGTKGVQWMELVKIVASWFISPLLSGFMSGLLFVLIRIFILKKEDPVPNGL
RALPVFYAATIAINVFSIMYTGAPVLGLVLPMWAIALISFGVALLFAFFVWLFVCPWMRR
KITGKLQKEGALSRVSDESLSKVQEAESPVFKELPGAKANDDSTIPLTGAAGETLGTSEG
TSAGSHPRAAYGRALSMTHGSVKSPISNGTFGFDGHTRSDGHVYHTVHKDSGLYKDLLHK
IHIDRGPEEKPAQESNYRLLRRNNSYTCYTAAICGLPVHATFRAADSSAPEDSEKLVGDT
VSYSKKRLRYDSYSSYCNAVAEAEIEAEEGGVEMKLASELADPDQPREDPAEEEKEEKDA
PEVHLLFHFLQVLTACFGSFAHGGNDVSNAIGPLVALWLIYKQGGVTQEAATPVWLLFYG
GVGICTGLWVWGRRVIQTMGKDLTPITPSSGFTIELASAFTVVIASNIGLPVSTTHCKVG
SVVAVGWIRSRKAVDWRLFRNIFVAWFVTVPVAGLFSAAVMALLMYGILPYV
>sp|Q09666|AHNK_HUMAN Neuroblast differentiation-associated protein AHNAK OS=Homo sapiens OX=9606 GN=AHNAK PE=1 SV=2
MEKEETTRELLLPNWQGSGSHGLTIAQRDDGVFVQEVTQNSPAARTGVVKEGDQIVGATI
YFDNLQSGEVTQLLNTMGHHTVGLKLHRKGDRSPEPGQTWTREVFSSCSSEVVLSGDDEE
YQRIYTTKIKPRLKSEDGVEGDLGETQSRTITVTRRVTAYTVDVTGREGAKDIDISSPEF
KIKIPRHELTEISNVDVETQSGKTVIRLPSGSGAASPTGSAVDIRAGAISASGPELQGAG
HSKLQVTMPGIKVGGSGVNVNAKGLDLGGRGGVQVPAVDISSSLGGRAVEVQGPSLESGD
HGKIKFPTMKVPKFGVSTGREGQTPKAGLRVSAPEVSVGHKGGKPGLTIQAPQLEVSVPS
ANIEGLEGKLKGPQITGPSLEGDLGLKGAKPQGHIGVDASAPQIGGSITGPSVEVQAPDI
DVQGPGSKLNVPKMKVPKFSVSGAKGEETGIDVTLPTGEVTVPGVSGDVSLPEIATGGLE
GKMKGTKVKTPEMIIQKPKISMQDVDLSLGSPKLKGDIKVSAPGVQGDVKGPQVALKGSR
VDIETPNLEGTLTGPRLGSPSGKTGTCRISMSEVDLNVAAPKVKGGVDVTLPRVEGKVKV
PEVDVRGPKVDVSAPDVEAHGPEWNLKMPKMKMPTFSTPGAKGEGPDVHMTLPKGDISIS
GPKVNVEAPDVNLEGLGGKLKGPDVKLPDMSVKTPKISMPDVDLHVKGTKVKGEYDVTVP
KLEGELKGPKVDIDAPDVDVHGPDWHLKMPKMKMPKFSVPGFKAEGPEVDVNLPKADVDI
SGPKIDVTAPDVSIEEPEGKLKGPKFKMPEMNIKVPKISMPDVDLHLKGPNVKGEYDVTM
PKVESEIKVPDVELKSAKMDIDVPDVEVQGPDWHLKMPKMKMPKFSMPGFKAEGPEVDVN
LPKADVDISGPKVGVEVPDVNIEGPEGKLKGPKFKMPEMNIKAPKISMPDVDLHMKGPKV
KGEYDMTVPKLEGDLKGPKVDVSAPDVEMQGPDWNLKMPKIKMPKFSMPSLKGEGPEFDV
NLSKANVDISAPKVDTNAPDLSLEGPEGKLKGPKFKMPEMHFRAPKMSLPDVDLDLKGPK
MKGNVDISAPKIEGEMQVPDVDIRGPKVDIKAPDVEGQGLDWSLKIPKMKMPKFSMPSLK
GEGPEVDVNLPKADVVVSGPKVDIEAPDVSLEGPEGKLKGPKFKMPEMHFKTPKISMPDV
DLHLKGPKVKGDVDVSVPKVEGEMKVPDVEIKGPKMDIDAPDVEVQGPDWHLKMPKMKMP
KFSMPGFKGEGREVDVNLPKADIDVSGPKVDVEVPDVSLEGPEGKLKGPKFKMPEMHFKA
PKISMPDVDLNLKGPKLKGDVDVSLPEVEGEMKVPDVDIKGPKVDISAPDVDVHGPDWHL
KMPKVKMPKFSMPGFKGEGPEVDVKLPKADVDVSGPKMDAEVPDVNIEGPDAKLKGPKFK
MPEMSIKPQKISIPDVGLHLKGPKMKGDYDVTVPKVEGEIKAPDVDIKGPKVDINAPDVE
VHGPDWHLKMPKVKMPKFSMPGFKGEGPEVDMNLPKADLGVSGPKVDIDVPDVNLEAPEG
KLKGPKFKMPSMNIQTHKISMPDVGLNLKAPKLKTDVDVSLPKVEGDLKGPEIDVKAPKM
DVNVGDIDIEGPEGKLKGPKFKMPEMHFKAPKISMPDVDLHLKGPKVKGDMDVSVPKVEG
EMKVPDVDIKGPKVDIDAPDVEVHDPDWHLKMPKMKMPKFSMPGFKAEGPEVDVNLPKAD

IDVSGPSVDTDAPDLDIEGPEGKLKGSKFKMPKLNIKAPKVSMPDVDLNLKGPKLKGEID
ASVPELEGDLRGPQVDVKGPFVEAEVPDVDLECPDAKLKGPKFKMPEMHFKAPKISMPDV
DLHLKGPKVKGDADVSVPKLEGDLTGPSVGVEVPDVELECPDAKLKGPKFKMPDMHFKAP
KISMPDVDLHLKGPKVKGDVDVSVPKLEGDLTGPSVGVEVPDVELECPDAKLKGPKFKMP
EMHFKTPKISMPDVDLHLKGPKVKGDMDVSVPKVEGEMKVPDVDIKGPKMDIDAPDVDVH
GPDWHLKMPKMKMPKFSMPGFKAEGPEVDVNLPKADVVVSGPKVDVEVPDVSLEGPEGKL
KGPKLKMPEMHFKAPKISMPDVDLHLKGPKVKGDVDVSLPKLEGDLTGPSVDVEVPDVEL
ECPDAKLKGPKFKMPEMHFKTPKISMPDVNLNLKGPKVKGDMDVSVPKVEGEMKVPDVDI
RGPKVDIDAPDVDVHGPDWHLKMPKMKMPKFSMPGFKGEGPEVDVNLPKADVDVSGPKVD
VEVPDVSLEGPEGKLKGPKFKMPEMHFKTPKISMPDVDFNLKGPKIKGDVDVSAPKLEGE
LKGPELDVKGPKLDADMPEVAVEGPNGKWKTPKFKMPDMHFKAPKISMPDLDLHLKSPKA
KGEVDVDVPKLEGDLKGPHVDVSGPDIDIEGPEGKLKGPKFKMPDMHFKAPNISMPDVDL
NLKGPKIKGDVDVSVPEVEGKLEVPDMNIRGPKVDVNAPDVQAPDWHLKMPKMKMPKFSM
PGFKAEGPEVDVNLPKADVDISGPKVDIEGPDVNIEGPEGKLKGPKLKMPEMNIKAPKIS
MPDFDLHLKGPKVKGDVDVSLPKVEGDLKGPEVDIKGPKVDINAPDVGVQGPDWHLKMPK
VKMPKFSMPGFKGEGPDGDVKLPKADIDVSGPKVDIEGPDVNIEGPEGKLKGPKFKMPEM
NIKAPKISMPDIDLNLKGPKVKGDVDVSLPKVEGDLKGPEVDIKGPKVDIDAPDVDVHGP
DWHLKMPKIKMPKISMPGFKGEGPDVDVNLPKADIDVSGPKVDVECPDVNIEGPEGKWKS
PKFKMPEMHFKTPKISMPDIDLNLTGPKIKGDVDVTGPKVEGDLKGPEVDLKGPKVDIDV
PDVNVQGPDWHLKMPKMKMPKFSMPGFKAEGPEVDVNLPKADVDVSGPKVDVEGPDVNIE
GPEGKLKGPKFKMPEMNIKAPKIPMPDFDLHLKGPKVKGDVDISLPKVEGDLKGPEVDIR
GPQVDIDVPDVGVQGPDWHLKMPKVKMPKFSMPGFKGEGPDVDVNLPKADLDVSGPKVDI
DVPDVNIEGPEGKLKGPKFKMPEMNIKAPKISMPDIDLNLKGPKVKGDMDVSLPKVEGDM
KVPDVDIKGPKVDINAPDVDVQGPDWHLKMPKIKMPKISMPGFKGEGPEVDVNLPKADLD
VSGPKVDVDVPDVNIEGPDAKLKGPKFKMPEMNIKAPKISMPDLDLNLKGPKMKGEVDVS
LANVEGDLKGPALDIKGPKIDVDAPDIDIHGPDAKLKGPKLKMPDMHVNMPKISMPEIDL
NLKGSKLKGDVDVSGPKLEGDIKAPSLDIKGPEVDVSGPKLNIEGKSKKSRFKLPKFNFS
GSKVQTPEVDVKGKKPDIDITGPKVDINAPDVEVQGKVKGSKFKMPFLSISSPKVSMPDV
ELNLKSPKVKGDLDIAGPNLEGDFKGPKVDIKAPEVNLNAPDVDVHGPDWNLKMPKMKMP
KFSVSGLKAEGPDVAVDLPKGDINIEGPSMNIEGPDLNVEGPEGGLKGPKFKMPDMNIKA
PKISMPDIDLNLKGPKVKGDVDISLPKLEGDLKGPEVDIKGPKVDINAPDVDVHGPDWHL
KMPKVKMPKFSMPGFKGEGPEVDVTLPKADIDISGPNVDVDVPDVNIEGPDAKLKGPKFK
MPEMNIKAPKISMPDFDLNLKGPKMKGDVVVSLPKVEGDLKGPEVDIKGPKVDIDTPDIN
IEGSEGKFKGPKFKIPEMHLKAPKISMPDIDLNLKGPKVKGDVDVSLPKMEGDLKGPEVD
IKGPKVDINAPDVDVQGPDWHLKMPKVKMPKFSMPGFKGEGPDVDVNLPKADLDVSGPKV
DIDVPDVNIEGPEGKLKGPKFKMPEMNIKAPKISMPDIDLNLKGPKVKGDMDVSLPKVEG
DMQVPDLDIKGPKVDINAPDVDVRGPDWHLKMPKIKMPKISMPGFKGEGPEVDVNLPKAD
LDVSGPKVDVDVPDVNIEGPDAKLKGPKFKMPEMNIKAPKISMPDFDLHLKGPKVKGDVD
VSLPKMEGDLKAPEVDIKGPKVDIDAPDVDVHGPDWHLKMPKVKMPKFSMPGFKGEGPEV
DVNLPKADIDVSGPKVDIDTPDIDIHGPEGKLKGPKFKMPDLHLKAPKISMPEVDLNLKG
PKMKGDVDVSLPKVEGDLKGPEVDIKGPKVDIDVPDVDVQGPDWHLKMPKVKMPKFSMPG
FKGEGPDVDVNLPKADLDVSGPKVDIDVPDVNIEGPDAKLKGPKFKMPEMNIKAPKISMP
DFDLHLKGPKVKGDVDVSLPKVEGDLKGPEVDIKGPKVDIDAPDVDVHGPDWHLKMPKVK
MPKFSMPGFKGEGPDVDVTLPKADIEISGPKVDIDAPDVSIEGPDAKLKGPKFKMPEMNI

KAPKISMPDIDFNLKGPKVKGDVDVSLPKVEGDLKGPEIDIKGPSLDIDTPDVNIEGPEG
KLKGPKFKMPEMNIKAPKISMPDFDLHLKGPKVKGDVDVSLPKVESDLKGPEVDIEGPEG
KLKGPKFKMPDVHFKSPQISMSDIDLNLKGPKIKGDMDISVPKLEGDLKGPKVDVKGPKV
GIDTPDIDIHGPEGKLKGPKFKMPDLHLKAPKISMPEVDLNLKGPKVKGDMDISLPKVEG
DLKGPEVDIRDPKVDIDVPDVDVQGPDWHLKMPKVKMPKFSMPGFKGEGPDVDVNLPKAD
IDVSGPKVDVDVPDVNIEGPDAKLKGPKFKMPEMSIKAPKISMPDIDLNLKGPKVKGDVD
VTLPKVEGDLKGPEADIKGPKVDINTPDVDVHGPDWHLKMPKVKMPKFSMPGFKGEGPDV
DVSLPKADIDVSGPKVDVDIPDVNIEGPDAKLKGPKFKMPEINIKAPKISIPDVDLDLKG
PKVKGDFDVSVPKVEGTLKGPEVDLKGPRLDFEGPDAKLSGPSLKMPSLEISAPKVTAPD
VDLHLKAPKIGFSGPKLEGGEVDLKGPKVEAPSLDVHMDSPDINIEGPDVKIPKFKKPKF
GFGAKSPKADIKSPSLDVTVPEAELNLETPEISVGGKGKKSKFKMPKIHMSGPKIKAKKQ
GFDLNVPGGEIDASLKAPDVDVNIAGPDAALKVDVKSPKTKKTMFGKMYFPDVEFDIKSP
KFKAEAPLPSPKLEGELQAPDLELSLPAIHVEGLDIKAKAPKVKMPDVDISVPKIEGDLK
GPKVQANLGAPDINIEGLDAKVKTPSFGISAPQVSIPDVNVNLKGPKIKGDVPSVGLEGP
DVDLQGPEAKIKFPKFSMPKIGIPGVKMEGGGAEVHAQLPSLEGDLRGPDVKLEGPDVSL
KGPGVDLPSVNLSMPKVSGPDLDLNLKGPSLKGDLDASVPSMKVHAPGLNLSGVGGKMQV
GGDGVKVPGIDATTKLNVGAPDVTLRGPSLQGDLAVSGDIKCPKVSVGAPDLSLEASEGS
IKLPKMKLPQFGISTPGSDLHVNAKGPQVSGELKGPGVDVNLKGPRISAPNVDFNLEGPK
VKGSLGATGEIKGPTVGGGLPGIGVQGLEGNLQMPGIKSSGCDVNLPGVNVKLPTGQISG
PEIKGGLKGSEVGFHGAAPDISVKGPAFNMASPESDFGINLKGPKIKGGADVSGGVSAPD
ISLGEGHLSVKGSGGEWKGPQVSSALNLDTSKFAGGLHFSGPKVEGGVKGGQIGLQAPGL
SVSGPQGHLESGSGKVTFPKMKIPKFTFSGRELVGREMGVDVHFPKAEASIQAGAGDGEW
EESEVKLKKSKIKMPKFNFSKPKGKGGVTGSPEASISGSKGDLKSSKASLGSLEGEAEAE
ASSPKGKFSLFKSKKPRHRSNSFSDEREFSGPSTPTGTLEFEGGEVSLEGGKVKGKHGKL
KFGTFGGLGSKSKGHYEVTGSDDETGKLQGSGVSLASKKSRLSSSSSNDSGNKVGIQLPE
VELSVSTKKE
>tr|Q0QF37|Q0QF37_HUMAN Malate dehydrogenase (Fragment) OS=Homo sapiens OX=9606
GN=MDH2 PE=2 SV=1
QNNAKVAVLGASGGIGQPLSLLLKNSPLVSRLTLYDIAHTPGVAADLSHIETKAAVKGYL
GPEQLPDCLKGCDVVVIPAGVPRKPGMTRDDLFNTNATIVATLTAACAQHCPEAMICVIA
NPVNSTIPITAEVFKKHGVYNPNKIFGVTTLDIVRANTFVAELKGLDPARVNVPVIGGHA
GKTIIPLISQCTPKVDFPQDQLTALTGRIQEAGTEVVKAKAGAGSATLSMAYAGARFVFS
LVDAMNGKEGVVECSFVKSQETECTYFSTPLLLGKKGIEKNLGIGKVSSFEEKMISDAIP
ELKAS
>sp|Q10567|AP1B1_HUMAN AP-1 complex subunit beta-1 OS=Homo sapiens OX=9606
GN=AP1B1 PE=1 SV=3
MTDSKYFTTTKKGEIFELKAELNSDKKEKKKEAVKKVIASMTVGKDVSALFPDVVNCMQT
DNLELKKLVYLYLMNYAKSQPDMAIMAVNTFVKDCEDPNPLIRALAVRTMGCIRVDKITE
YLCEPLRKCLKDEDPYVRKTAAVCVAKLHDINAQLVEDQGFLDTLKDLISDSNPMVVANA
VAALSEIAESHPSSNLLDLNPQSINKLLTALNECTEWGQIFILDCLANYMPKDDREAQSI
CERVTPRLSHANSAVVLSAVKVLMKFMEMLSKDLDYYGTLLKKLAPPLVTLLSAEPELQY
VALRNINLIVQKRPEILKHEMKVFFVKYNDPIYVKLEKLDIMIRLASQANIAQVLAELKE
YATEVDVDFVRKAVRAIGRCAIKVEQSAERCVSTLLDLIQTKVNYVVQEAIVVIKDIFRK
YPNKYESVIATLCENLDSLDEPEARAAMIWIVGEYAERIDNADELLESFLEGFHDESTQV

QLQLLTAIVKLFLKKPTETQELVQQVLSLATQDSDNPDLRDRGYIYWRLLSTDPVAAKEV
VLAEKPLISEETDLIEPTLLDELICYIGTLASVYHKPPSAFVEGGRGVVHKSLPPRTASS
ESAESPETAPTGAPPGEQPDVIPAQGDLLGDLLNLDLGPPVSGPPLATSSVQMGAVDLLG
GGLDSLMGDEPEGIGGTNFVAPPTAAVPANLGAPIGSGLSDLFDLTSGVGTLSGSYVAPK
AVWLPAMKAKGLEISGTFTRQVGSISMDLQLTNKALQVMTDFAIQFNRNSFGLAPAAPLQ
VHAPLSPNQTVEISLPLSTVGSVMKMEPLNNLQVAVKNNIDVFYFSTLYPLHILFVEDGK
MDRQMFLATWKDIPNENEAQFQIRDCPLNAEAASSKLQSSNIFTVAKRNVEGQDMLYQSL
KLTNGIWVLAELRIQPGNPSCTDLELSLKCRAPEVSQHVYQAYETILKN
>sp|Q12756|KIF1A_HUMAN Kinesin-like protein KIF1A OS=Homo sapiens OX=9606 GN=KIF1A PE=1 SV=2
MAGASVKVAVRVRPFNSREMSRDSKCIIQMSGSTTTIVNPKQPKETPKSFSFDYSYWSHT
SPEDINYASQKQVYRDIGEEMLQHAFEGYNVCIFAYGQTGAGKSYTMMGKQEKDQQGIIP
QLCEDLFSRINDTTNDNMSYSVEVSYMEIYCERVRDLLNPKNKGNLRVREHPLLGPYVED
LSKLAVTSYNDIQDLMDSGNKARTVAATNMNETSSRSHAVFNIIFTQKRHDAETNITTEK
VSKISLVDLAGSERADSTGAKGTRLKEGANINKSLTTLGKVISALAEMDSGPNKNKKKKK
TDFIPYRDSVLTWLLRENLGGNSRTAMVAALSPADINYDETLSTLRYADRAKQIRCNAVI
NEDPNNKLIRELKDEVTRLRDLLYAQGLGDITDMTNALVGMSPSSSLSALSSRAASVSSL
HERILFAPGSEEAIERLKETEKIIAELNETWEEKLRRTEAIRMEREALLAEMGVAMREDG
GTLGVFSPKKTPHLVNLNEDPLMSECLLYYIKDGITRVGREDGERRQDIVLSGHFIKEEH
CVFRSDSRGGSEAVVTLEPCEGADTYVNGKKVTEPSILRSGNRIIMGKSHVFRFNHPEQA
RQERERTPCAETPAEPVDWAFAQRELLEKQGIDMKQEMEQRLQELEDQYRREREEATYLL
EQQRLDYESKLEALQKQMDSRYYPEVNEEEEEPEDEVQWTERECELALWAFRKWKWYQFT
SLRDLLWGNAIFLKEANAISVELKKKVQFQFVLLTDTLYSPLPPDLLPPEAAKDRETRPF
PRTIVAVEVQDQKNGATHYWTLEKLRQRLDLMREMYDRAAEVPSSVIEDCDNVVTGGDPF
YDRFPWFRLVGRAFVYLSNLLYPVPLVHRVAIVSEKGEVKGFLRVAVQAISADEEAPDYG
SGVRQSGTAKISFDDQHFEKFQSESCPVVGMSRSGTSQEELRIVEGQGQGADVGPSADEV
NNNTCSAVPPEGLLLDSSEKAALDGPLDAALDHLRLGNTFTFRVTVLQASSISAEYADIF
CQFNFIHRHDEAFSTEPLKNTGRGPPLGFYHVQNIAVEVTKSFIEYIKSQPIVFEVFGHY
QQHPFPPLCKDVLSPLRPSRRHFPRVMPLSKPVPATKLSTLTRPCPGPCHCKYDLLVYFE
ICELEANGDYIPAVVDHRGGMPCMGTFLLHQGIQRRITVTLLHETGSHIRWKEVRELVVG
RIRNTPETDESLIDPNILSLNILSSGYIHPAQDDRTFYQFEAAWDSSMHNSLLLNRVTPY
REKIYMTLSAYIEMENCTQPAVVTKDFCMVFYSRDAKLPASRSIRNLFGSGSLRASESNR
VTGVYELSLCHVADAGSPGMQRRRRRVLDTSVAYVRGEENLAGWRPRSDSLILDHQWELE
KLSLLQEVEKTRHYLLLREKLETAQRPVPEALSPAFSEDSESHGSSSASSPLSAEGRPSP
LEAPNERQRELAVKCLRLLTHTFNREYTHSHVCVSASESKLSEMSVTLLRDPSMSPLGVA
TLTPSSTCPSLVEGRYGATDLRTPQPCSRPASPEPELLPEADSKKLPSPARATETDKEPQ
RLLVPDIQEIRVSPIVSKKGYLHFLEPHTSGWARRFVVVRRPYAYMYNSDKDTVERFVLN
LATAQVEYSEDQQAMLKTPNTFAVCTEHRGILLQAASDKDMHDWLYAFNPLLAGTIRSKL
SRRRSAQMRV
>sp|Q12765|SCRN1_HUMAN Secernin-1 OS=Homo sapiens OX=9606 GN=SCRN1 PE=1 SV=2
MAAAPPSYCFVAFPPRAKDGLVVFGKNSARPRDEVQEVVYFSAADHEPESKVECTYISID
QVPRTYAIMISRPAWLWGAEMGANEHGVCIANEAINTREPAAEIEALLGMDLVRLGLERG
ETAKEALDVIVSLLEEHGQGGNYFEDANSCHSFQSAYLIVDRDEAWVLETIGKYWAAEKV
TEGVRCICSQLSLTTKMDAEHPELRSYAQSQGWWTGEGEFNFSEVFSPVEDHLDCGAGKD

SLEKQEESITVQTMMNTLRDKASGVCIDSEFFLTTASGVSVLPQNRSSPCIHYFTGTPDP
SRSIFKPFIFVDDVKLVPKTQSPCFGDDDPAKKEPRFQEKPDRRHELYKAHEWARAIIES
DQEQGRKLRSTMLELEKQGLEAMEEILTSSEPLDPAEVGDLFYDCVDTEIKFFK
>sp|Q12797|ASPH_HUMAN Aspartyl/asparaginyl beta-hydroxylase OS=Homo sapiens OX=9606 GN=ASPH PE=1 SV=3
MAQRKNAKSSGNSSSSGSGSGSTSAGSSSPGARRETKHGGHKNGRKGGLSGTSFFTWFMV
IALLGVWTSVAVVWFDLVDYEEVLGKLGIYDADGDGDFDVDDAKVLLGLKERSTSEPAVP
PEEAEPHTEPEEQVPVEAEPQNIEDEAKEQIQSLLHEMVHAEHVEGEDLQQEDGPTGEPQ
QEDDEFLMATDVDDRFETLEPEVSHEETEHSYHVEETVSQDCNQDMEEMMSEQENPDSSE
PVVEDERLHHDTDDVTYQVYEEQAVYEPLENEGIEITEVTAPPEDNPVEDSQVIVEEVSI
FPVEEQQEVPPETNRKTDDPEQKAKVKKKKPKLLNKFDKTIKAELDAAEKLRKRGKIEEA
VNAFKELVRKYPQSPRARYGKAQCEDDLAEKRRSNEVLRGAIETYQEVASLPDVPADLLK
LSLKRRSDRQQFLGHMRGSLLTLQRLVQLFPNDTSLKNDLGVGYLLIGDNDNAKKVYEEV
LSVTPNDGFAKVHYGFILKAQNKIAESIPYLKEGIESGDPGTDDGRFYFHLGDAMQRVGN
KEAYKWYELGHKRGHFASVWQRSLYNVNGLKAQPWWTPKETGYTELVKSLERNWKLIRDE
GLAVMDKAKGLFLPEDENLREKGDWSQFTLWQQGRRNENACKGAPKTCTLLEKFPETTGC
RRGQIKYSIMHPGTHVWPHTGPTNCRLRMHLGLVIPKEGCKIRCANETKTWEEGKVLIFD
DSFEHEVWQDASSFRLIFIVDVWHPELTPQQRRSLPAI
>sp|Q12816|TROP_HUMAN Trophinin OS=Homo sapiens OX=9606 GN=TRO PE=1 SV=3
MDRRNDYGYRVPLFQGPLPPPGSLGLPFPPDIQTETTEEDSVLLMHTLLAATKDSLAMDP
PVVNRPKKSKTKKAPIKTITKAAPAAPPVPAANEIATNKPKITWQALNLPVITQISQALP
TTEVTNTQASSVTAQPKKANKMKRVTAKAAQGSQSPTGHEGGTIQLKSPLQVLKLPVISQ
NIHAPIANESASSQALITSIKPKKASKAKKAANKAIASATEVSLAATATHTATTQGQITN
ETASIHTTAASIRTKKASKARKTIAKVINTDTEHIEALNVTDAATRQIEASVVAIRPKKS
KGKKAASRGPNSVSEISEAPLATQIVTNQALAATLRVKRGSRARKAATKARATESQTPNA
DQGAQAKIASAQTNVSALETQVAAAVQALADDYLAQLSLEPTTRTRGKRNRKSKHLNGDE
RSGSNYRRIPWGRRPAPPRDVAILQERANKLVKYLLVKDQTKIPIKRSDMLRDVIQEYDE
YFPEIIERASYTLEKMFRVNLKEIDKQSSLYILISTQESSAGILGTTKDTPKLGLLMVIL
SVIFMNGNKASEAVIWEVLRKLGLRPGVRHSLFGEVRKLITDEFVKQKYLEYKRVPNSRP
PEYEFFWGLRSYHETSKMKVLKFACRVQKKDPKDWAVQYREAVEMEVQAAAVAVAEAEAR
AEARAQMGIGEEAVAGPWNWDDMDIDCLTREELGDDAQAWSRFSFEIEARAQENADASTN
VNFSRGASTRAGFSDGASISFNGAPSSSGGFSGGPGITFGVAPSTSASFSNTASISFGGT
LSTSSSFSSAASISFGCAHSTSTSFSSEASISFGGMPCTSASFSGGVSSSFSGPLSTSAT
FSGGASSGFGGTLSTTAGFSGVLSTSTSFGSAPTTSTVFSSALSTSTGFGGILSTSVCFG
GSPSSSGSFGGTLSTSICFGGSPCTSTGFGGTLSTSVSFGGSSSTSANFGGTLSTSICFD
GSPSTGAGFGGALNTSASFGSVLNTSTGFGGAMSTSADFGGTLSTSVCFGGSPGTSVSFG
SALNTNAGYGGAVSTNTDFGGTLSTSVCFGGSPSTSAGFGGALNTNASFGCAVSTSASFS
GAVSTSACFSGAPITNPGFGGAFSTSAGFGGALSTAADFGGTPSNSIGFGAAPSTSVSFG
GAHGTSLCFGGAPSTSLCFGSASNTNLCFGGPPSTSACFSGATSPSFCDGPSTSTGFSFG
NGLSTNAGFGGGLNTSAGFGGGLGTSAGFSGGLSTSSGFDGGLGTSAGFGGGPGTSTGFG
GGLGTSAGFSGGLGTSAGFGGGLVTSDGFGGGLGTNASFGSTLGTSAGFSGGLSTSDGFG
SRPNASFDRGLSTIIGFGSGSNTSTGFTGEPSTSTGFSSGPSSIVGFSGGPSTGVGFCSG
PSTSGFSGGPSTGAGFGGGPNTGAGFGGGPSTSAGFGSGAASLGACGFSYG
>sp|Q12934|BFSP1_HUMAN Filensin OS=Homo sapiens OX=9606 GN=BFSP1 PE=1 SV=3

MYRRSYVFQTRKEQYEHADEASRAAEPERPADEGWAGATSLAALQGLGERVAAHVQRARA
LEQRHAGLRRQLDAFQRLGELAGPEDALARQVESNRQRVRDLEAERARLERQGTEAQRAL
DEFRSKYENECECQLLLKEMLERLNKEADEALLHNLRLQLEAQFLQDDISAAKDRHKKNL
LEVQTYISILQQIIHTTPPASIVTSGMREEKLLTEREVAALRSQLEEGREVLSHLQAQRV
ELQAQTTTLEQAIKSAHECYDDEIQLYNEQIETLRKEIEETERVLEKSSYDCRQLAVAQQ
TLKNELDRYHRIIEIEGNRLTSAFIETPIPLFTQSHGVSLSTGSGGKDLTRALQDITAAK
PRQKALPKNVPRRKEIITKDKTNGALEDAPLKGLEDTKLVQVVLKEESESKFESESKEVS
PLTQEGAPEDVPDGGQISKGFGKLYRKVKEKVRSPKEPETPTELYTKERHVLVTGDANYV
DPRFYVSSITAKGGVAVSVAEDSVLYDGQVEPSPESPKPPLENGQVGLQEKEDGQPIDQQ
PIDKEIEPDGAELEGPEEKREGEERDEESRRPCAMVTPGAEEPSIPEPPKPAADQDGAEV
LGTRSRSLPEKGPPKALAYKTVEVVESIEKISTESIQTYEETAVIVETMIGKTKSDKKKS
GEKSS
>sp|Q12955|ANK3_HUMAN Ankyrin-3 OS=Homo sapiens OX=9606 GN=ANK3 PE=1 SV=3
MAHAASQLKKNRDLEINAEEEPEKKRKHRKRSRDRKKKSDANASYLRAARAGHLEKALDY
IKNGVDINICNQNGLNALHLASKEGHVEVVSELLQREANVDAATKKGNTALHIASLAGQA
EVVKVLVTNGANVNAQSQNGFTPLYMAAQENHLEVVKFLLDNGASQSLATEDGFTPLAVA
LQQGHDQVVSLLLENDTKGKVRLPALHIAARKDDTKAAALLLQNDNNADVESKSGFTPLH
IAAHYGNINVATLLLNRAAAVDFTARNDITPLHVASKRGNANMVKLLLDRGAKIDAKTRD
GLTPLHCGARSGHEQVVEMLLDRAAPILSKTKNGLSPLHMATQGDHLNCVQLLLQHNVPV
DDVTNDYLTALHVAAHCGHYKVAKVLLDKKANPNAKALNGFTPLHIACKKNRIKVMELLL
KHGASIQAVTESGLTPIHVAAFMGHVNIVSQLMHHGASPNTTNVRGETALHMAARSGQAE
VVRYLVQDGAQVEAKAKDDQTPLHISARLGKADIVQQLLQQGASPNAATTSGYTPLHLSA
REGHEDVAAFLLDHGASLSITTKKGFTPLHVAAKYGKLEVANLLLQKSASPDAAGKSGLT
PLHVAAHYDNQKVALLLLDQGASPHAAAKNGYTPLHIAAKKNQMDIATTLLEYGADANAV
TRQGIASVHLAAQEGHVDMVSLLLGRNANVNLSNKSGLTPLHLAAQEDRVNVAEVLVNQG
AHVDAQTKMGYTPLHVGCHYGNIKIVNFLLQHSAKVNAKTKNGYTPLHQAAQQGHTHIIN
VLLQNNASPNELTVNGNTALGIARRLGYISVVDTLKIVTEETMTTTTVTEKHKMNVPETM
NEVLDMSDDEVRKANAPEMLSDGEYISDVEEGEDAMTGDTDKYLGPQDLKELGDDSLPAE
GYMGFSLGARSASLRSFSSDRSYTLNRSSYARDSMMIEELLVPSKEQHLTFTREFDSDSL
RHYSWAADTLDNVNLVSSPIHSGFLVSFMVDARGGSMRGSRHHGMRIIIPPRKCTAPTRI
TCRLVKRHKLANPPPMVEGEGLASRLVEMGPAGAQFLGPVIVEIPHFGSMRGKERELIVL
RSENGETWKEHQFDSKNEDLTELLNGMDEELDSPEELGKKRICRIITKDFPQYFAVVSRI
KQESNQIGPEGGILSSTTVPLVQASFPEGALTKRIRVGLQAQPVPDEIVKKILGNKATFS
PIVTVEPRRRKFHKPITMTIPVPPPSGEGVSNGYKGDTTPNLRLLCSITGGTSPAQWEDI
TGTTPLTFIKDCVSFTTNVSARFWLADCHQVLETVGLATQLYRELICVPYMAKFVVFAKM
NDPVESSLRCFCMTDDKVDKTLEQQENFEEVARSKDIEVLEGKPIYVDCYGNLAPLTKGG
QQLVFNFYSFKENRLPFSIKIRDTSQEPCGRLSFLKEPKTTKGLPQTAVCNLNITLPAHK
KETESDQDDEIEKTDRRQSFASLALRKRYSYLTEPGMIERSTGATRSLPTTYSYKPFFST
RPYQSWTTAPITVPGPAKSGFTSLSSSSNTPSASPLKSIWSVSTPSPIKSTLGASTTSS
VKSISDVASPIRSFRTMSSPIKTVVSQSPYNIQVSSGTLARAPAVTEATPLKGLASNSTF
SSRTSPVTTAGSLLERSSITMTPPASPKSNINMYSSSLPFKSIITSAAPLISSPLKSVVS
PVKSAVDVISSAKITMASSLSSPVKQMPGHAEVALVNGSISPLKYPSSSTLINGCKATAT
LQEKISSATNSVSSVVSAATDTVEKVFSTTTAMPFSPLRSYVSAAPSAFQSLRTPSASAL
YTSLGSSISATTSSVTSSIITVPVYSVVNVLPEPALKKLPDSNSFTKSAAALLSPIKTLT

TETHPQPHFSRTSSPVKSSLFLAPSALKLSTPSSLSSSQEILKDVAEMKEDLMRMTAILQ
TDVPEEKPFQPELPKEGRIDDEEPFKIVEKVKEDLVKVSEILKKDVCVDNKGSPKSPKSD
KGHSPEDDWIEFSSEEIREARQQAAASQSPSLPERVQVKAKAASEKDYNLTKVIDYLTND
IGSSSLTNLKYKFEDAKKDGEERQKRVLKPAIALQEHKLKMPPASMRTSTSEKELCKMAD
SFFGTDTILESPDDFSQHDQDKSPLSDSGFETRSEKTPSAPQSAESTGPKPLFHEVPIPP
VITETRTEVVHVIRSYDPSAGDVPQTQPEEPVSPKPSPTFMELEPKPTTSSIKEKVKAFQ
MKASSEEDDHNRVLSKGMRVKEETHITTTTRMVYHSPPGGEGASERIEETMSVHDIMKAF
QSGRDPSKELAGLFEHKSAVSPDVHKSAAETSAQHAEKDNQMKPKLERIIEVHIEKGNQA
EPTEVIIRETKKHPEKEMYVYQKDLSRGDINLKDFLPEKHDAFPCSEEQGQQEEEELTAE
ESLPSYLESSRVNTPVSQEEDSRPSSAQLISDDSYKTLKLLSQHSIEYHDDELSELRGES
YRFAEKMLLSEKLDVSHSDTEESVTDHAGPPSSELQGSDKRSREKIATAPKKEILSKIYK
DVSENGVGKVSKDEHFDKVTVLHYSGNVSSPKHAMWMRFTEDRLDRGREKLIYEDRVDRT
VKEAEEKLTEVSQFFRDKTEKLNDELQSPEKKARPKNGKEYSSQSPTSSSPEKVLLTELL
ASNDEWVKARQHGPDGQGFPKAEEKAPSLPSSPEKMVLSQQTEDSKSTVEAKGSISQSKA
PDGPQSGFQLKQSKLSSIRLKFEQGTHAKSKDMSQEDRKSDGQSRIPVKKIQESKLPVYQ
VFAREKQQKAIDLPDESVSVQKDFMVLKTKDEHAQSNEIVVNDSGSDNVKKQRTEMSSKA
MPDSFSEQQAKDLACHITSDLATRGPWDKKVFRTWESSGATNNKSQKEKLSHVLVHDVRE
NHIGHPESKSVDQKNEFMSVTERERKLLTNGSLSEIKEMTVKSPSKKVLYREYVVKEGDH
PGGLLDQPSRRSESSAVSHIPVRVADERRMLSSNIPDGFCEQSAFPKHELSQKLSQSSMS
KETVETQHFNSIEDEKVTYSEISKVSKHQSYVGLCPPLEETETSPTKSPDSLEFSPGKES
PSSDVFDHSPIDGLEKLAPLAQTEGGKEIKTLPVYVSFVQVGKQYEKEIQQGGVKKIISQ
ECKTVQETRGTFYTTRQQKQPPSPQGSPEDDTLEQVSFLDSSGKSPLTPETPSSEEVSYE
FTSKTPDSLIAYIPGKPSPIPEVSEESEEEEQAKSTSLKQTTVEETAVEREMPNDVSKDS
NQRPKNNRVAYIEFPPPPPLDADQIESDKKHHYLPEKEVDMIEVNLQDEHDKYQLAEPVI
RVQPPSPVPPGADVSDSSDDESIYQPVPVKKYTFKLKEVDDEQKEKPKASAEKASNQKEL
ESNGSGKDNEFGLGLDSPQNEIAQNGNNDQSITECSIATTAEFSHDTDATEIDSLDGYDL
QDEDDGLTESDSKLPIQAMEIKKDIWNTEGILKPADRSFSQSKLEVIEEEGKVGPDEDKP
PSKSSSSEKTPDKTDQKSGAQFFTLEGRHPDRSVFPDTYFSYKVDEEFATPFKTVATKGL
DFDPWSNNRGDDEVFDSKSREDETKPFGLAVEDRSPATTPDTTPARTPTDESTPTSEPNP
FPFHEGKMFEMTRSGAIDMSKRDFVEERLQFFQIGEHTSEGKSGDQGEGDKSMVTATPQP
QSGDTTVETNLERNVETPTVEPNPSIPTSGECQEGTSSSGSLEKSAAATNTSKVDPKLRT
PIKMGISASTMTMKKEGPGEITDKIEAVMTSCQGLENETITMISNTANSQMGVRPHEKHD
FQKDNFNNNNNLDSSTIQTDNIMSNIVLTEHSAPTCTTEKDNPVKVSSGKKTGVLQGHCV
RDKQKVLGEQQKTKELIGIRQKSKLPIKATSPKDTFPPNHMSNTKASKMKQVSQSEKTKA
LTTSSCVDVKSRIPVKNTHRDNIIAVRKACATQKQGQPEKGKAKQLPSKLPVKVRSTCVT
TTTTTATTTTTTTTTTTTSCTVKVRKSQLKEVCKHSIEYFKGISGETLKLVDRLSEEEKK
MQSELSDEEESTSRNTSLSETSRGGQPSVTTKSARDKKTEAAPLKSKSEKAGSEKRSSRR
TGPQSPCERTDIRMAIVADHLGLSWTELARELNFSVDEINQIRVENPNSLISQSFMLLKK
WVTRDGKNATTDALTSVLTKINRIDIVTLLEGPIFDYGNISGTRSFADENNVFHDPVDGW
QNETSSGNLESCAQARRVTGGLLDRLDDSPDQCRDSITSYLKGEAGKFEANGSHTEITPE
AKTKSYFPESQNDVGKQSTKETLKPKIHGSGHVEEPASPLAAYQKSLEETSKLIIEETKP
CVPVSMKKMSRTSPADGKPRLSLHEEEGSSGSEQKQGEGFKVKTKKEIRHVEKKSHS
>sp|Q13057|COASY_HUMAN Bifunctional coenzyme A synthase OS=Homo sapiens OX=9606
GN=COASY PE=1 SV=4

MAVFRSGLLVLTTPLASLAPRLASILTSAARLVNHTLYVHLQPGMSLEGPAQPQSSPVQA
TFEVLDFITHLYAGADVHRHLDVRILLTNIRTKSTFLPPLPTSVQNLAHPPEVVLTDFQT
LDGSQYNPVKQQLVRYATSCYSCCPRLASVLLYSDYGIGEVPVEPLDVPLPSTIRPASPV
AGSPKQPVRGYYRGAVGGTFDRLHNAHKVLLSVACILAQEQLVVGVADKDLLKSKLLPEL
LQPYTERVEHLSEFLVDIKPSLTFDVIPLLDPYGPAGSDPSLEFLVVSEETYRGGMAINR
FRLENDLEELALYQIQLLKDLRHTENEEDKVSSSSFRQRMLGNLLRPPYERPELPTCLYV
IGLTGISGSGKSSIAQRLKGLGAFVIDSDHLGHRAYAPGGPAYQPVVEAFGTDILHKDGI
INRKVLGSRVFGNKKQLKILTDIMWPIIAKLAREEMDRAVAEGKRVCVIDAAVLLEAGWQ
NLVHEVWTAVIPETEAVRRIVERDGLSEAAAQSRLQSQMSGQQLVEQSHVVLSTLWEPHI
TQRQVEKAWALLQKRIPKTHQALD
>sp|Q13098|CSN1_HUMAN COP9 signalosome complex subunit 1 OS=Homo sapiens OX=9606 GN=GPS1 PE=1 SV=4
MPLPVQVFNLQGAVEPMQIDVDPQEDPQNAPDVNYVVENPSLDLEQYAASYSGLMRIERL
QFIADHCPTLRVEALKMALSFVQRTFNVDMYEEIHRKLSEATRSSLRELQNAPDAIPESG
VEPPALDTAWVEATRKKALLKLEKLDTDLKNYKGNSIKESIRRGHDDLGDHYLDCGDLSN
ALKCYSRARDYCTSAKHVINMCLNVIKVSVYLQNWSHVLSYVSKAESTPEIAEQRGERDS
QTQAILTKLKCAAGLAELAARKYKQAAKCLLLASFDHCDFPELLSPSNVAIYGGLCALAT
FDRQELQRNVISSSSFKLFLELEPQVRDIIFKFYESKYASCLKMLDEMKDNLLLDMYLAP
HVRTLYTQIRNRALIQYFSPYVSADMHRMAAAFNTTVAALEDELTQLILEGLISARVDSH
SKILYARDVDQRSTTFEKSLLMGKEFQRRAKAMMLRAAVLRNQIHVKSPPREGSQGELTP
ANSQSRMSTNM
>sp|Q13126|MTAP_HUMAN S-methyl-5'-thioadenosine phosphorylase OS=Homo sapiens OX=9606 GN=MTAP PE=1 SV=2
MASGTTTTAVKIGIIGGTGLDDPEILEGRTEKYVDTPFGKPSDALILGKIKNVDCVLLAR
HGRQHTIMPSKVNYQANIWALKEEGCTHVIVTTACGSLREEIQPGDIVIIDQFIDRTTMR
PQSFYDGSHSCARGVCHIPMAEPFCPKTREVLIETAKKLGLRCHSKGTMVTIEGPRFSSR
AESFMFRTWGADVINMTTVPEVVLAKEAGICYASIAMATDYDCWKEHEEAVSVDRVLKTL
KENANKAKSLLLTTIPQIGSTEWSETLHNLKNMAQFSVLLPRH
>sp|Q13162|PRDX4_HUMAN Peroxiredoxin-4 OS=Homo sapiens OX=9606 GN=PRDX4 PE=1 SV=1
MEALPLLAATTPDHGRHRRLLLLPLLLFLLPAGAVQGWETEERPRTREEECHFYAGGQVY
PGEASRVSVADHSLHLSKAKISKPAPYWEGTAVIDGEFKELKLTDYRGKYLVFFFYPLDF
TFVCPTEIIAFGDRLEEFRSINTEVVACSVDSQFTHLAWINTPRRQGGLGPIRIPLLSDL
THQISKDYGVYLEDSGHTLRGLFIIDDKGILRQITLNDLPVGRSVDETLRLVQAFQYTDK
HGEVCPAGWKPGSETIIPDPAGKLKYFDKLN
>sp|Q13200|PSMD2_HUMAN 26S proteasome non-ATPase regulatory subunit 2 OS=Homo sapiens OX=9606 GN=PSMD2 PE=1 SV=3
MEEGGRDKAPVQPQQSPAAAPGGTDEKPSGKERRDAGDKDKEQELSEEDKQLQDELEMLV
ERLGEKDTSLYRPALEELRRQIRSSTTSMTSVPKPLKFLRPHYGKLKEIYENMAPGENKR
FAADIISVLAMTMSGERECLKYRLVGSQEELASWGHEYVRHLAGEVAKEWQELDDAEKVQ
REPLLTLVKEIVPYNMAHNAEHEACDLLMEIEQVDMLEKDIDENAYAKVCLYLTSCVNYV
PEPENSALLRCALGVFRKFSRFPEALRLALMLNDMELVEDIFTSCKDVVVQKQMAFMLGR
HGVFLELSEDVEEYEDLTEIMSNVQLNSNFLALARELDIMEPKVPDDIYKTHLENNRFGG
SGSQVDSARMNLASSFVNGFVNAAFGQDKLLTDDGNKWLYKNKDHGMLSAAASLGMILLW

DVDGGLTQIDKYLYSSEDYIKSGALLACGIVNSGVRNECDPALALLSDYVLHNSNTMRLG
SIFGLGLAYAGSNREDVLTLLLPVMGDSKSSMEVAGVTALACGMIAVGSCNGDVTSTILQ
TIMEKSETELKDTYARWLPLGLGLNHLGKGEAIEAILAALEVVSEPFRSFANTLVDVCAY
AGSGNVLKVQQLLHICSEHFDSKEKEEDKDKKEKKDKDKKEAPADMGAHQGVAVLGIALI
AMGEEIGAEMALRTFGHLLRYGEPTLRRAVPLALALISVSNPRLNILDTLSKFSHDADPE
VSYNSIFAMGMVGSGTNNARLAAMLRQLAQYHAKDPNNLFMVRLAQGLTHLGKGTLTLCP
YHSDRQLMSQVAVAGLLTVLVSFLDVRNIILGKSHYVLYGLVAAMQPRMLVTFDEELRPL
PVSVRVGQAVDVVGQAGKPKTITGFQTHTTPVLLAHGERAELATEEFLPVTPILEGFVIL
RKNPNYDL
>sp|Q13404|UB2V1_HUMAN Ubiquitin-conjugating enzyme E2 variant 1 OS=Homo sapiens OX=9606 GN=UBE2V1 PE=1 SV=2
MAATTGSGVKVPRNFRLLEELEEGQKGVGDGTVSWGLEDDEDMTLTRWTGMIIGPPRTIY
ENRIYSLKIECGPKYPEAPPFVRFVTKINMNGVNSSNGVVDPRAISVLAKWQNSYSIKVV
LQELRRLMMSKENMKLPQPPEGQCYSN
>sp|Q13443|ADAM9_HUMAN Disintegrin and metalloproteinase domain-containing protein 9 OS=Homo sapiens OX=9606 GN=ADAM9 PE=1 SV=1
MGSGARFPSGTLRVRWLLLLGLVGPVLGAARPGFQQTSHLSSYEIITPWRLTRERREAPR
PYSKQVSYVIQAEGKEHIIHLERNKDLLPEDFVVYTYNKEGTLITDHPNIQNHCHYRGYV
EGVHNSSIALSDCFGLRGLLHLENASYGIEPLQNSSHFEHIIYRMDDVYKEPLKCGVSNK
DIEKETAKDEEEEPPSMTQLLRRRRAVLPQTRYVELFIVVDKERYDMMGRNQTAVREEMI
LLANYLDSMYIMLNIRIVLVGLEIWTNGNLINIVGGAGDVLGNFVQWREKFLITRRRHDS
AQLVLKKGFGGTAGMAFVGTVCSRSHAGGINVFGQITVETFASIVAHELGHNLGMNHDDG
RDCSCGAKSCIMNSGASGSRNFSSCSAEDFEKLTLNKGGNCLLNIPKPDEAYSAPSCGNK
LVDAGEECDCGTPKECELDPCCEGSTCKLKSFAECAYGDCCKDCRFLPGGTLCRGKTSEC
DVPEYCNGSSQFCQPDVFIQNGYPCQNNKAYCYNGMCQYYDAQCQVIFGSKAKAAPKDCF
IEVNSKGDRFGNCGFSGNEYKKCATGNALCGKLQCENVQEIPVFGIVPAIIQTPSRGTKC
WGVDFQLGSDVPDPGMVNEGTKCGAGKICRNFQCVDASVLNYDCDVQKKCHGHGVCNSNK
NCHCENGWAPPNCETKGYGGSVDSGPTYNEMNTALRDGLLVFFFLIVPLIVCAIFIFIKR
DQLWRSYFRKKRSQTYESDGKNQANPSRQPGSVPRHVSPVTPPREVPIYANRFAVPTYAA
KQPQQFPSRPPPPQPKVSSQGNLIPARPAPAPPLYSSLT
>sp|Q13444|ADA15_HUMAN Disintegrin and metalloproteinase domain-containing protein 15 OS=Homo sapiens OX=9606 GN=ADAM15 PE=1 SV=4
MRLALLWALGLLGAGSPLPSWPLPNIGGTEEQQAESEKAPREPLEPQVLQDDLPISLKKV
LQTSLPEPLRIKLELDGDSHILELLQNRELVPGRPTLVWYQPDGTRVVSEGHTLENCCYQ
GRVRGYAGSWVSICTCSGLRGLVVLTPERSYTLEQGPGDLQGPPIISRIQDLHLPGHTCA
LSWRESVHTQKPPEHPLGQRHIRRRRDVVTETKTVELVIVADHSEAQKYRDFQHLLNRTL
EVALLLDTFFRPLNVRVALVGLEAWTQRDLVEISPNPAVTLENFLHWRRAHLLPRLPHDS
AQLVTGTSFSGPTVGMAIQNSICSPDFSGGVNMDHSTSILGVASSIAHELGHSLGLDHDL
PGNSCPCPGPAPAKTCIMEASTDFLPGLNFSNCSRRALEKALLDGMGSCLFERLPSLPPM
AAFCGNMFVEPGEQCDCGFLDDCVDPCCDSLTCQLRPGAQCASDGPCCQNCQLRPSGWQC
RPTRGDCDLPEFCPGDSSQCPPDVSLGDGEPCAGGQAVCMHGRCASYAQQCQSLWGPGAQ
PAAPLCLQTANTRGNAFGSCGRNPSGSYVSCTPRDAICGQLQCQTGRTQPLLGSIRDLLW
ETIDVNGTELNCSWVHLDLGSDVAQPLLTLPGTACGPGLVCIDHRCQRVDLLGAQECRSK
CHGHGVCDSNRHCYCEEGWAPPDCTTQLKATSSLTTGLLLSLLVLLVLVMLGASYWYRAR

LHQRLCQLKGPTCQYRAAQSGPSERPGPPQRALLARGTKQASALSFPAPPSRPLPPDPVS
KRLQAELADRPNPPTRPLPADPVVRSPKSQGPAKPPPPRKPLPADPQGRCPSGDLPGPGA
GIPPLVVPSRPAPPPPTVSSLYL
>sp|Q13449|LSAMP_HUMAN Limbic system-associated membrane protein OS=Homo sapiens OX=9606 GN=LSAMP PE=1 SV=2
MVRRVQPDRKQLPLVLLRLLCLLPTGLPVRSVDFNRGTDNITVRQGDTAILRCVVEDKNS
KVAWLNRSGIIFAGHDKWSLDPRVELEKRHSLEYSLRIQKVDVYDEGSYTCSVQTQHEPK
TSQVYLIVQVPPKISNISSDVTVNEGSNVTLVCMANGRPEPVITWRHLTPTGREFEGEEE
YLEILGITREQSGKYECKAANEVSSADVKQVKVTVNYPPTITESKSNEATTGRQASLKCE
ASAVPAPDFEWYRDDTRINSANGLEIKSTEGQSSLTVTNVTEEHYGNYTCVAANKLGVTN
ASLVLFRPGSVRGINGSISLAVPLWLLAASLLCLLSKC
>sp|Q13505|MTX1_HUMAN Metaxin-1 OS=Homo sapiens OX=9606 GN=MTX1 PE=1 SV=3
MLLGGPPRSPRSGTSPKGPWSSTGHVQFGKSPQTWPRRTRPRSPEPAAPSGVRGSTWTRR
RDSPRRAGPTALSRYVGHLWMGRRPPSPEARGPVPRSSAASRARRSLASPGISPGPLTAT
IGGAVAGGGPRQGRAEAHKEVFPGQRVGKMAAPMELFCWSGGWGLPSVDLDSLAVLTYAR
FTGAPLKVHKISNPWQSPSGTLPALRTSHGEVISVPHKIITHLRKEKYNADYDLSARQGA
DTLAFMSLLEEKLLPVLVHTFWIDTKNYVEVTRKWYAEAMPFPLNFFLPGRMQRQYMERL
QLLTGEHRPEDEEELEKELYREARECLTLLSQRLGSQKFFFGDAPASLDAFVFSYLALLL
QAKLPSGKLQVHLRGLHNLCAYCTHILSLYFPWDGAEVPPQRQTPAGPETEEEPYRRRNQ
ILSVLAGLAAMVGYALLSGIVSIQRATPARAPGTRTLGMAEEDEEE
>sp|Q13515|BFSP2_HUMAN Phakinin OS=Homo sapiens OX=9606 GN=BFSP2 PE=1 SV=1
MSERRVVVDLPTSASSSMPLQRRRASFRGPRSSSSLESPPASRTNAMSGLVRAPGVYVGT
APSGCIGGLGARVTRRALGISSVFLQGLRSSGLATVPAPGLERDHGAVEDLGGCLVEYMA
KVHALEQVSQELETQLRMHLESKATRSGNWGALRASWASSCQQVGEAVLENARLMLQTET
IQAGADDFKERYENEQPFRKAAEEEINSLYKVIDEANLTKMDLESQIESLKEELGSLSRN
YEEDVKLLHKQLAGCELEQMDAPIGTGLDDILETIRIQWERDVEKNRVEAGALLQAKQQA
EVAHMSQTQEEKLAAALRVELHNTSCQVQSLQAETESLRALKRGLENTLHDAKHWHDMEL
QNLGAVVGRLEAELREIRAEAEQQQQERAHLLARKCQLQKDVASYHALLDREESG
>sp|Q13617|CUL2_HUMAN Cullin-2 OS=Homo sapiens OX=9606 GN=CUL2 PE=1 SV=2
MSLKPRVVDFDETWNKLLTTIKAVVMLEYVERATWNDRFSDIYALCVAYPEPLGERLYTE
TKIFLENHVRHLHKRVLESEEQVLVMYHRYWEEYSKGADYMDCLYRYLNTQFIKKNKLTE
ADLQYGYGGVDMNEPLMEIGELALDMWRKLMVEPLQAILIRMLLREIKNDRGGEDPNQKV
IHGVINSFVHVEQYKKKFPLKFYQEIFESPFLTETGEYYKQEASNLLQESNCSQYMEKVL
GRLKDEEIRCRKYLHPSSYTKVIHECQQRMVADHLQFLHAECHNIIRQEKKNDMANMYVL
LRAVSTGLPHMIQELQNHIHDEGLRATSNLTQENMPTLFVESVLEVHGKFVQLINTVLNG
DQHFMSALDKALTSVVNYREPKSVCKAPELLAKYCDNLLKKSAKGMTENEVEDRLTSFIT
VFKYIDDKDVFQKFYARMLAKRLIHGLSMSMDSEEAMINKLKQACGYEFTSKLHRMYTDM
SVSADLNNKFNNFIKNQDTVIDLGISFQIYVLQAGAWPLTQAPSSTFAIPQELEKSVQMF
ELFYSQHFSGRKLTWLHYLCTGEVKMNYLGKPYVAMVTTYQMAVLLAFNNSETVSYKELQ
DSTQMNEKELTKTIKSLLDVKMINHDSEKEDIDAESSFSLNMNFSSKRTKFKITTSMQKD
TPQEMEQTRSAVDEDRKMYLQAAIVRIMKARKVLRHNALIQEVISQSRARFNPSISMIKK
CIEVLIDKQYIERSQASADEYSYVA
>sp|Q13618|CUL3_HUMAN Cullin-3 OS=Homo sapiens OX=9606 GN=CUL3 PE=1 SV=2
MSNLSKGTGSRKDTKMRIRAFPMTMDEKYVNSIWDLLKNAIQEIQRKNNSGLSFEELYRN

AYTMVLHKHGEKLYTGLREVVTEHLINKVREDVLNSLNNNFLQTLNQAWNDHQTAMVMIR
DILMYMDRVYVQQNNVENVYNLGLIIFRDQVVRYGCIRDHLRQTLLDMIARERKGEVVDR
GAIRNACQMLMILGLEGRSVYEEDFEAPFLEMSAEFFQMESQKFLAENSASVYIKKVEAR
INEEIERVMHCLDKSTEEPIVKVVERELISKHMKTIVEMENSGLVHMLKNGKTEDLGCMY
KLFSRVPNGLKTMCECMSSYLREQGKALVSEEGEGKNPVDYIQGLLDLKSRFDRFLLESF
NNDRLFKQTIAGDFEYFLNLNSRSPEYLSLFIDDKLKKGVKGLTEQEVETILDKAMVLFR
FMQEKDVFERYYKQHLARRLLTNKSVSDDSEKNMISKLKTECGCQFTSKLEGMFRDMSIS
NTTMDEFRQHLQATGVSLGGVDLTVRVLTTGYWPTQSATPKCNIPPAPRHAFEIFRRFYL
AKHSGRQLTLQHHMGSADLNATFYGPVKKEDGSEVGVGGAQVTGSNTRKHILQVSTFQMT
ILMLFNNREKYTFEEIQQETDIPERELVRALQSLACGKPTQRVLTKEPKSKEIENGHIFT
VNDQFTSKLHRVKIQTVAAKQGESDPERKETRQKVDDDRKHEIEAAIVRIMKSRKKMQHN
VLVAEVTQQLKARFLPSPVVIKKRIEGLIEREYLARTPEDRKVYTYVA
>sp|Q13797|ITA9_HUMAN Integrin alpha-9 OS=Homo sapiens OX=9606 GN=ITGA9 PE=1 SV=2
MGGPAAPRGAGRLRALLLALVVAGIPAGAYNLDPQRPVHFQGPADSFFGYAVLEHFHDNT
RWVLVGAPKADSKYSPSVKSPGAVFKCRVHTNPDRRCTELDMARGKNRGTSCGKTCREDR
DDEWMGVSLARQPKADGRVLACAHRWKNIYYEADHILPHGFCYIIPSNLQAKGRTLIPCY
EEYKKKYGEEHGSCQAGIAGFFTEELVVMGAPGSFYWAGTIKVLNLTDNTYLKLNDEVIM
NRRYTYLGYAVTAGHFSHPSTIDVVGGAPQDKGIGKVYIFRADRRSGTLIKIFQASGKKM
GSYFGSSLCAVDLNGDGLSDLLVGAPMFSEIRDEGQVTVYINRGNGALEEQLALTGDGAY
NAHFGESIASLDDLDNDGFPDVAIGAPKEDDFAGAVYIYHGDAGGIVPQYSMKLSGQKIN
PVLRMFGQSISGGIDMDGNGYPDVTVGAFMSDSVVLLRARPVITVDVSIFLPGSINITAP
QCHDGQQPVNCLNVTTCFSFHGKHVPGEIGLNYVLMADVAKKEKGQMPRVYFVLLGETMG
QVTEKLQLTYMEETCRHYVAHVKRRVQDVISPIVFEAAYSLSEHVTGEEERELPPLTPVL
RWKKGQKIAQKNQTVFERNCRSEDCAADLQLQGKLLLSSMDEKTLYLALGAVKNISLNIS
ISNLGDDAYDANVSFNVSRELFFINMWQKEEMGISCELLESDFLKCSVGFPFMRSKSKYE
FSVIFDTSHLSGEEEVLSFIVTAQSGNTERSESLHDNTLVLMVPLMHEVDTSITGIMSPT
SFVYGESVDAANFIQLDDLECHFQPINITLQVYNTGPSTLPGSSVSISFPNRLSSGGAEM
FHVQEMVVGQEKGNCSFQKNPTPCIIPQEQENIFHTIFAFFTKSGRKVLDCEKPGISCLT
AHCNFSALAKEESRTIDIYMLLNTEILKKDSSSVIQFMSRAKVKVDPALRVVEIAHGNPE
EVTVVFEALHNLEPRGYVVGWIIAISLLVGILIFLLLAVLLWKMGFFRRRYKEIIEAEKN
RKENEDSWDWVQKNQ
>sp|Q13813|SPTN1_HUMAN Spectrin alpha chain, non-erythrocytic 1 OS=Homo sapiens
OX=9606 GN=SPTAN1 PE=1 SV=3
MDPSGVKVLETAEDIQERRQQVLDRYHRFKELSTLRRQKLEDSYRFQFFQRDAEELEKWI
QEKLQIASDENYKDPTNLQGKLQKHQAFEAEVQANSGAIVKLDETGNLMISEGHFASETI
RTRLMELHRQWELLLEKMREKGIKLLQAQKLVQYLRECEDVMDWINDKEAIVTSEELGQD
LEHVEVLQKKFEEFQTDMAAHEERVNEVNQFAAKLIQEQHPEEELIKTKQDEVNAAWQRL
KGLALQRQGKLFGAAEVQRFNRDVDETISWIKEKEQLMASDDFGRDLASVQALLRKHEGL
ERDLAALEDKVKALCAEADRLQQSHPLSATQIQVKREELITNWEQIRTLAAERHARLNDS
YRLQRFLADFRDLTSWVTEMKALINADELASDVAGAEALLDRHQEHKGEIDAHEDSFKSA
DESGQALLAAGHYASDEVREKLTVLSEERAALLELWELRRQQYEQCMDLQLFYRDTEQVD
NWMSKQEAFLLNEDLGDSLDSVEALLKKHEDFEKSLSAQEEKITALDEFATKLIQNNHYA
MEDVATRRDALLSRRNALHERAMRRRAQLADSFHLQQFFRDSDELKSWVNEKMKTATDEA
YKDPSNLQGKVQKHQAFEAELSANQSRIDALEKAGQKLIDVNHYAKDEVAARMNEVISLW

KKLLEATELKGIKLREANQQQQFNRNVEDIELWLYEVEGHLASDDYGKDLTNVQNLQKKH
ALLEADVAAHQDRIDGITIQARQFQDAGHFDAENIKKKQEALVARYEALKEPMVARKQKL
ADSLRLQQLFRDVEDEETWIREKEPIAASTNRGKDLIGVQNLLKKHQALQAEIAGHEPRI
KAVTQKGNAMVEEGHFAAEDVKAKLHELNQKWEALKAKASQRRQDLEDSLQAQQYFADAN
EAESWMREKEPIVGSTDYGKDEDSAEALLKKHEALMSDLSAYGSSIQALREQAQSCRQQV
APTDDETGKELVLALYDYQEKSPREVTMKKGDILTLLNSTNKDWWKVEVNDRQGFVPAAY
VKKLDPAQSASRENLLEEQGSIALRQEQIDNQTRITKEAGSVSLRMKQVEELYHSLLELG
EKRKGMLEKSCKKFMLFREANELQQWINEKEAALTSEEVGADLEQVEVLQKKFDDFQKDL
KANESRLKDINKVAEDLESEGLMAEEVQAVQQQEVYGMMPRDETDSKTASPWKSARLMVH
TVATFNSIKELNERWRSLQQLAEERSQLLGSAHEVQRFHRDADETKEWIEEKNQALNTDN
YGHDLASVQALQRKHEGFERDLAALGDKVNSLGETAERLIQSHPESAEDLQEKCTELNQA
WSSLGKRADQRKAKLGDSHDLQRFLSDFRDLMSWINGIRGLVSSDELAKDVTGAEALLER
HQEHRTEIDARAGTFQAFEQFGQQLLAHGHYASPEIKQKLDILDQERADLEKAWVQRRMM
LDQCLELQLFHRDCEQAENWMAAREAFLNTEDKGDSLDSVEALIKKHEDFDKAINVQEEK
IAALQAFADQLIAAGHYAKGDISSRRNEVLDRWRRLKAQMIEKRSKLGESQTLQQFSRDV
DEIEAWISEKLQTASDESYKDPTNIQSKHQKHQAFEAELHANADRIRGVIDMGNSLIERG
ACAGSEDAVKARLAALADQWQFLVQKSAEKSQKLKEANKQQNFNTGIKDFDFWLSEVEAL
LASEDYGKDLASVNNLLKKHQLLEADISAHEDRLKDLNSQADSLMTSSAFDTSQVKDKRD
TINGRFQKIKSMAASRRAKLNESHRLHQFFRDMDDEESWIKEKKLLVGSEDYGRDLTGVQ
NLRKKHKRLEAELAAHEPAIQGVLDTGKKLSDDNTIGKEEIQQRLAQFVEHWKELKQLAA
ARGQRLEESLEYQQFVANVEEEEAWINEKMTLVASEDYGDTLAAIQGLLKKHEAFETDFT
VHKDRVNDVCTNGQDLIKKNNHHEENISSKMKGLNGKVSDLEKAAAQRKAKLDENSAFLQ
FNWKADVVESWIGEKENSLKTDDYGRDLSSVQTLLTKQETFDAGLQAFQQEGIANITALK
DQLLAAKHVQSKAIEARHASLMKRWSQLLANSAARKKKLLEAQSHFRKVEDLFLTFAKKA
SAFNSWFENAEEDLTDPVRCNSLEEIKALREAHDAFRSSLSSAQADFNQLAELDRQIKSF
RVASNPYTWFTMEALEETWRNLQKIIKERELELQKEQRRQEENDKLRQEFAQHANAFHQW
IQETRTYLLDGSCMVEESGTLESQLEATKRKHQEIRAMRSQLKKIEDLGAAMEEALILDN
KYTEHSTVGLAQQWDQLDQLGMRMQHNLEQQIQARNTTGVTEEALKEFSMMFKHFDKDKS
GRLNHQEFKSCLRSLGYDLPMVEEGEPDPEFEAILDTVDPNRDGHVSLQEYMAFMISRET
ENVKSSEEIESAFRALSSEGKPYVTKEELYQNLTREQADYCVSHMKPYVDGKGRELPTAF
DYVEFTRSLFVN
>sp|Q13867|BLMH_HUMAN Bleomycin hydrolase OS=Homo sapiens OX=9606 GN=BLMH PE=1 SV=1
MSSSGLNSEKVAALIQKLNSDPQFVLAQNVGTTHDLLDICLKRATVQRAQHVFQHAVPQE
GKPITNQKSSGRCWIFSCLNVMRLPFMKKLNIEEFEFSQSYLFFWDKVERCYFFLSAFVD
TAQRKEPEDGRLVQFLLMNPANDGGQWDMLVNIVEKYGVIPKKCFPESYTTEATRRMNDI
LNHKMREFCIRLRNLVHSGATKGEISATQDVMMEEIFRVVCICLGNPPETFTWEYRDKDK
NYQKIGPITPLEFYREHVKPLFNMEDKICLVNDPRPQHKYNKLYTVEYLSNMVGGRKTLY
NNQPIDFLKKMVAASIKDGEAVWFGCDVGKHFNSKLGLSDMNLYDHELVFGVSLKNMNKA
ERLTFGESLMTHAMTFTAVSEKDDQDGAFTKWRVENSWGEDHGHKGYLCMTDEWFSEYVY
EVVVDRKHVPEEVLAVLEQEPIILPAWDPMGALAE
>sp|Q13885|TBB2A_HUMAN Tubulin beta-2A chain OS=Homo sapiens OX=9606 GN=TUBB2A PE=1 SV=1
MREIVHIQAGQCGNQIGAKFWEVISDEHGIDPTGSYHGDSDLQLERINVYYNEAAGNKYV

PRAILVDLEPGTMDSVRSGPFGQIFRPDNFVFGQSGAGNNWAKGHYTEGAELVDSVLDVV
RKESESCDCLQGFQLTHSLGGGTGSGMGTLLISKIREEYPDRIMNTFSVMPSPKVSDTVV
EPYNATLSVHQLVENTDETYSIDNEALYDICFRTLKLTTPTYGDLNHLVSATMSGVTTCL
RFPGQLNADLRKLAVNMVPFPRLHFFMPGFAPLTSRGSQQYRALTVPELTQQMFDSKNMM
AACDPRHGRYLTVAAIFRGRMSMKEVDEQMLNVQNKNSSYFVEWIPNNVKTAVCDIPPRG
LKMSATFIGNSTAIQELFKRISEQFTAMFRRKAFLHWYTGEGMDEMEFTEAESNMNDLVS
EYQQYQDATADEQGEFEEEEGEDEA
>sp|Q14019|COTL1_HUMAN Coactosin-like protein OS=Homo sapiens OX=9606 GN=COTL1 PE=1 SV=3
MATKIDKEACRAAYNLVRDDGSAVIWVTFKYDGSTIVPGEQGAEYQHFIQQCTDDVRLFA
FVRFTTGDAMSKRSKFALITWIGENVSGLQRAKTGTDKTLVKEVVQNFAKEFVISDRKEL
EEDFIKSELKKAGGANYDAQTE
>sp|Q14031|CO4A6_HUMAN Collagen alpha-6(IV) chain OS=Homo sapiens OX=9606 GN=COL4A6 PE=1 SV=3
MLINKLWLLLVTLCLTEELAAAGEKSYGKPCGGQDCSGSCQCFPEKGARGRPGPIGIQGP
TGPQGFTGSTGLSGLKGERGFPGLLGPYGPKGDKGPMGVPGFLGINGIPGHPGQPGPRGP
PGLDGCNGTQGAVGFPGPDGYPGLLGPPGLPGQKGSKGDPVLAPGSFKGMKGDPGLPGLD
GITGPQGAPGFPGAVGPAGPPGLQGPPGPPGPLGPDGNMGLGFQGEKGVKGDVGLPGPAG
PPPSTGELEFMGFPKGKKGSKGEPGPKGFPGISGPPGFPGLGTTGEKGEKGEKGIPGLPG
PRGPMGSEGVQGPPGQQGKKGTLGFPGLNGFQGIEGQKGDIGLPGPDVFIDIDGAVISGN
PGDPGVPGLPGLKGDEGIQGLRGPSGVPGLPALSGVPGALGPQGFPGLKGDQGNPGRTTI
GAAGLPGRDGLPGPPGPPGPPSPEFETETLHNKESGFPGLRGEQGPKGNLGLKGIKGDSG
FCACDGGVPNTGPPGEPGPPGPWGLIGLPGLKGARGDRGSGGAQGPAGAPGLVGPLGPSG
PKGKKGEPILSTIQGMPGDRGDSGSQGFRGVIGEPGKDGVPGLPGLPGLPGDGGQGFPGE
KGLPGLPGEKGHPGPPGLPGNGLPGLPGPRGLPGDKGKDGLPGQQGLPGSKGITLPCIIP
GSYGPSGFPGTPGFPGPKGSRGLPGTPGQPGSSGSKGEPGSPGLVHLPELPGFPGPRGEK
GLPGFPGLPGKDGLPGMIGSPGLPGSKGATGDIFGAENGAPGEQGLQGLTGHKGFLGDSG
LPGLKGVHGKPGLLGPKGERGSPGTPGQVGQPGTPGSSGPYGIKGKSGLPGAPGFPGISG
HPGKKGTRGKKGPPGSIVKKGLPGLKGLPGNPGLVGLKGSPGSPGVAGLPALSGPKGEKG
SVGFVGFPGIPGLPGIPGTRGLKGIPGSTGKMGPSGRAGTPGEKGDRGNPGPVGIPSPRR
PMSNLWLKGDKGSQGSAGSNGFPGPRGDKGEAGRPGPPGLPGAPGLPGIIKGVSGKPGPP
GFMGIRGLPGLKGSSGITGFPGMPGESGSQGIRGSPGLPGASGLPGLKGDNGQTVEISGS
PGPKGQPGESGFKGTKGRDGLIGNIGFPGNKGEDGKVGVSGDVGLPGAPGFPGVAGMRGE
PGLPGSSGHQGAIGPLGSPGLIGPKGFPGFPGLHGLNGLPGTKGTHGTPGPSITGVPGPA
GLPGPKGEKGYPGIGIGAPGKPGLRGQKGDRGFPGLQGPAGLPGAPGISLPSLIAGQPGD
PGRPGLDGERGRPGPAGPPGPPGPSSNQGDTGDPGFPGIPGPKGPKGDQGIPGFSGLPGE
LGLKGMRGEPGFMGTPGKVGPPGDPGFPGMKGKAGPRGSSGLQGDPGQTPTAEAVQVPPG
PLGLPGIDGIPGLTGDPGAQGPVGLQGSKGLPGIPGKDGPSGLPGPPGALGDPGLPGLQG
PPGFEGAPGQQGPFGMPGMPGQSMRVGYTLVKHSQSEQVPPCPIGMSQLWVGYSLLFVEG
QEKAHNQDLGFAGSCLPRFSTMPFIYCNINEVCHYARRNDKSYWLSTTAPIPMMPVSQTQ
IPQYISRCSVCEAPSQAIAVHSQDITIPQCPLGWRSLWIGYSFLMHTAAGAEGGGQSLVS
PGSCLEDFRATPFIECSGARGTCHYFANKYSFWLTTVEERQQFGELPVSETLKAGQLHTR
VSRCQVCMKSL
>sp|Q14112|NID2_HUMAN Nidogen-2 OS=Homo sapiens OX=9606 GN=NID2 PE=1 SV=3

MEGDRVAGRPVLSSLPVLLLLPLLMLRAAALHPDELFPHGESWGDQLLQEGDDESSAVVK
LANPLHFYEARFSNLYVGTNGIISTQDFPRETQYVDYDFPTDFPAIAPFLADIDTSHGRG
RVLYREDTSPAVLGLAARYVRAGFPRSARFTPTHAFLATWEQVGAYEEVKRGALPSGELN
TFQAVLASDGSDSYALFLYPANGLQFLGTRPKESYNVQLQLPARVGFCRGEADDLKSEGP
YFSLTSTEQSVKNLYQLSNLGIPGVWAFHIGSTSPLDNVRPAAVGDLSAAHSSVPLGRSF
SHATALESDYNEDNLDYYDVNEEEAEYLPGEPEEALNGHSSIDVSFQSKVDTKPLEESST
LDPHTKEGTSLGEVGGPDLKGQVEPWDERETRSPAPPEVDRDSLAPSWETPPPYPENGSI
QPYPDGGPVPSEMDVPPAHPEEEIVLRSYPASGHTTPLSRGTYEVGLEDNIGSNTEVFTY
NAANKETCEHNHRQCSRHAFCTDYATGFCCHCQSKFYGNGKHCLPEGAPHRVNGKVSGHL
HVGHTPVHFTDVDLHAYIVGNDGRAYTAISHIPQPAAQALLPLTPIGGLFGWLFALEKPG
SENGFSLAGAAFTHDMEVTFYPGEETVRITQTAEGLDPENYLSIKTNIQGQVPYVSANFT
AHISPYKELYHYSDSTVTSTSSRDYSLTFGAINQTWSYRIHQNITYQVCRHAPRHPSFPT
TQQLNVDRVFALYNDEERVLRFAVTNQIGPVKEDSDPTPGNPCYDGSHMCDTTARCHPGT
GVDYTCECASGYQGDGRNCVDENECATGFHRCGPNSVCINLPGSYRCECRSGYEFADDRH
TCILITPPANPCEDGSHTCAPAGQARCVHHGGSTFSCACLPGYAGDGHQCTDVDECSENR
CHPAATCYNTPGSFSCRCQPGYYGDGFQCIPDSTSSLTPCEQQQRHAQAQYAYPGARFHI
PQCDEQGNFLPLQCHGSTGFCWCVDPDGHEVPGTQTPPGSTPPHCGPSPEPTQRPPTICE
RWRENLLEHYGGTPRDDQYVPQCDDLGHFIPLQCHGKSDFCWCVDKDGREVQGTRSQPGT
TPACIPTVAPPMVRPTPRPDVTPPSVGTFLLYTQGQQIGYLPLNGTRLQKDAAKTLLSLH
GSIIVGIDYDCRERMVYWTDVAGRTISRAGLELGAEPETIVNSGLISPEGLAIDHIRRTM
YWTDSVLDKIESALLDGSERKVLFYTDLVNPRAIAVDPIRGNLYWTDWNREAPKIETSSL
DGENRRILINTDIGLPNGLTFDPFSKLLCWADAGTKKLECTLPDGTGRRVIQNNLKYPFS
IVSYADHFYHTDWRRDGVVSVNKHSGQFTDEYLPEQRSHLYGITAVYPYCPTGRK
>sp|Q14152|EIF3A_HUMAN Eukaryotic translation initiation factor 3 subunit A OS=Homo sapiens OX=9606 GN=EIF3A PE=1 SV=1
MPAYFQRPENALKRANEFLEVGKKQPALDVLYDVMKSKKHRTWQKIHEPIMLKYLELCVD
LRKSHLAKEGLYQYKNICQQVNIKSLEDVVRAYLKMAEEKTEAAKEESQQMVLDIEDLDN
IQTPESVLLSAVSGEDTQDRTDRLLLTPWVKFLWESYRQCLDLLRNNSRVERLYHDIAQQ
AFKFCLQYTRKAEFRKLCDNLRMHLSQIQRHHNQSTAINLNNPESQSMHLETRLVQLDSA
ISMELWQEAFKAVEDIHGLFSLSKKPPKPQLMANYYNKVSTVFWKSGNALFHASTLHRLY
HLSREMRKNLTQDEMQRMSTRVLLATLSIPITPERTDIARLLDMDGIIVEKQRRLATLLG
LQAPPTRIGLINDMVRFNVLQYVVPEVKDLYNWLEVEFNPLKLCERVTKVLNWVREQPEK
EPELQQYVPQLQNNTILRLLQQVSQIYQSIEFSRLTSLVPFVDAFQLERAIVDAARHCDL
QVRIDHTSRTLSFGSDLNYATREDAPIGPHLQSMPSEQIRNQLTAMSSVLAKALEVIKPA
HILQEKEEQHQLAVTAYLKNSRKEHQRILARRQTIEERKERLESLNIQREKEELEQREAE
LQKVRKAEEERLRQEAKEREKERILQEHEQIKKKTVRERLEQIKKTELGAKAFKDIDIED
LEELDPDFIMAKQVEQLEKEKKELQERLKNQEKKIDYFERAKRLEEIPLIKSAYEEQRIK
DMDLWEQQEEERITTMQLEREKALEHKNRMSRMLEDRDLFVMRLKAARQSVYEEKLKQFE
ERLAEERHNRLEERKRQRKEERRITYYREKEEEEQRRAEEQMLKEREERERAERAKREEE
LREYQERVKKLEEVERKKRQRELEIEERERRREEERRLGDSSLSRKDSRWGDRDSEGTWR
KGPEADSEWRRGPPEKEWRRGEGRDEDRSHRRDEERPRRLGDDEDREPSLRPDDDRVPRR
GMDDDRGPRRGPEEDRFSRRGADDDRPSWRNTDDDRPPRRIADEDRGNWRHADDDRPPRR
GLDEDRGSWRTADEDRGPRRGMDDDRGPRRGGADDERSSWRNADDDRGPRRGLDDDRGPR
RGMDDDRGPRRGMDDDRGPRRGMDDDRGPRRGLDDDRGPWRNADDDRIPRRGAEDDRGPW

RNMDDDRLSRRADDDRFPRRGDDSRPGPWRPLVKPGGWREKEKAREESWGPPRESRPSEE
REWDREKERDRDNQDREENDKDPERERDRERDVDREDRFRRPRDEGGWRRGPAEESSSWR
DSSRRDDRDRDDRRRERDDRRDLRERRDLRDDRDRRGPPLRSEREEVSSWRRADDRKDDR
VEERDPPRRVPPPALSRDRERDRDREREGEKEKASWRAEKDRESLRRTKNETDEDGWTTV
RR
>sp|Q14203|DCTN1_HUMAN Dynactin subunit 1 OS=Homo sapiens OX=9606 GN=DCTN1 PE=1 SV=3
MAQSKRHVYSRTPSGSRMSAEASARPLRVGSRVEVIGKGHRGTVAYVGATLFATGKWVGV
ILDEAKGKNDGTVQGRKYFTCDEGHGIFVRQSQIQVFEDGADTTSPETPDSSASKVLKRE
GTDTTAKTSKLRGLKPKKAPTARKTTTRRPKPTRPASTGVAGASSSLGPSGSASAGELSS
SEPSTPAQTPLAAPIIPTPVLTSPGAVPPLPSPSKEEEGLRAQVRDLEEKLETLRLKRAE
DKAKLKELEKHKIQLEQVQEWKSKMQEQQADLQRRLKEARKEAKEALEAKERYMEEMADT
ADAIEMATLDKEMAEERAESLQQEVEALKERVDELTTDLEILKAEIEEKGSDGAASSYQL
KQLEEQNARLKDALVRMRDLSSSEKQEHVKLQKLMEKKNQELEVVRQQRERLQEELSQAE
STIDELKEQVDAALGAEEMVEMLTDRNLNLEEKVRELRETVGDLEAMNEMNDELQENARE
TELELREQLDMAGARVREAQKRVEAAQETVADYQQTIKKYRQLTAHLQDVNRELTNQQEA
SVERQQQPPPETFDFKIKFAETKAHAKAIEMELRQMEVAQANRHMSLLTAFMPDSFLRPG
GDHDCVLVLLLMPRLICKAELIRKQAQEKFELSENCSERPGLRGAAGEQLSFAAGLVYSL
SLLQATLHRYEHALSQCSVDVYKKVGSLYPEMSAHERSLDFLIELLHKDQLDETVNVEPL
TKAIKYYQHLYSIHLAEQPEDCTMQLADHIKFTQSALDCMSVEVGRLRAFLQGGQEATDI
ALLLRDLETSCSDIRQFCKKIRRRMPGTDAPGIPAALAFGPQVSDTLLDCRKHLTWVVAV
LQEVAAAAAQLIAPLAENEGLLVAALEELAFKASEQIYGTPSSSPYECLRQSCNILISTM
NKLATAMQEGEYDAERPPSKPPPVELRAAALRAEITDAEGLGLKLEDRETVIKELKKSLK
IKGEELSEANVRLSLLEKKLDSAAKDADERIEKVQTRLEETQALLRKKEKEFEETMDALQ
ADIDQLEAEKAELKQRLNSQSKRTIEGLRGPPPSGIATLVSGIAGEEQQRGAIPGQAPGS
VPGPGLVKDSPLLLQQISAMRLHISQLQHENSILKGAQMKASLASLPPLHVAKLSHEGPG
SELPAGALYRKTSQLLETLNQLSTHTHVVDITRTSPAAKSPSAQLMEQVAQLKSLSDTVE
KLKDEVLKETVSQRPGATVPTDFATFPSSAFLRAKEEQQDDTVYMGKVTFSCAAGFGQRH
RLVLTQEQLHQLHSRLIS
>sp|Q14204|DYHC1_HUMAN Cytoplasmic dynein 1 heavy chain 1 OS=Homo sapiens OX=9606 GN=DYNC1H1 PE=1 SV=5
MSEPGGGGGEDGSAGLEVSAVQNVADVSVLQKHLRKLVPLLLEDGGEAPAALEAALEEKS
ALEQMRKFLSDPQVHTVLVERSTLKEDVGDEGEEEKEFISYNINIDIHYGVKSNSLAFIK
RTPVIDADKPVSSQLRVLTLSEDSPYETLHSFISNAVAPFFKSYIRESGKADRDGDKMAP
SVEKKIAELEMGLLHLQQNIEIPEISLPIHPMITNVAKQCYERGEKPKVTDFGDKVEDPT
FLNQLQSGVNRWIREIQKVTKLDRDPASGTALQEISFWLNLERALYRIQEKRESPEVLLT
LDILKHGKRFHATVSFDTDTGLKQALETVNDYNPLMKDFPLNDLLSATELDKIRQALVAI
FTHLRKIRNTKYPIQRALRLVEAISRDLSSQLLKVLGTRKLMHVAYEEFEKVMVACFEVF
QTWDDEYEKLQVLLRDIVKRKREENLKMVWRINPAHRKLQARLDQMRKFRRQHEQLRAVI
VRVLRPQVTAVAQQNQGEVPEPQDMKVAEVLFDAADANAIEEVNLAYENVKEVDGLDVSK
EGTEAWEAAMKRYDERIDRVETRITARLRDQLGTAKNANEMFRIFSRFNALFVRPHIRGA
IREYQTQLIQRVKDDIESLHDKFKVQYPQSQACKMSHVRDLPPVSGSIIWAKQIDRQLTA
YMKRVEDVLGKGWENHVEGQKLKQDGDSFRMKLNTQEIFDDWARKVQQRNLGVSGRIFTI
ESTRVRGRTGNVLKLKVNFLPEIITLSKEVRNLKWLGFRVPLAIVNKAHQANQLYPFAIS

LIESVRTYERTCEKVEERNTISLLVAGLKKEVQALIAEGIALVWESYKLDPYVQRLAETV
FNFQEKVDDLLIIEEKIDLEVRSLETCMYDHKTFSEILNRVQKAVDDLNLHSYSNLPIWV
NKLDMEIERILGVRLQAGLRAWTQVLLGQAEDKAEVDMDTDAPQVSHKPGGEPKIKNVVH
ELRITNQVIYLNPPIEECRYKLYQEMFAWKMVVLSLPRIQSQRYQVGVHYELTEEEKFYR
NALTRMPDGPVALEESYSAVMGIVSEVEQYVKVWLQYQCLWDMQAENIYNRLGEDLNKWQ
ALLVQIRKARGTFDNAETKKEFGPVVIDYGKVQSKVNLKYDSWHKEVLSKFGQMLGSNMT
EFHSQISKSRQELEQHSVDTASTSDAVTFITYVQSLKRKIKQFEKQVELYRNGQRLLEKQ
RFQFPPSWLYIDNIEGEWGAFNDIMRRKDSAIQQQVANLQMKIVQEDRAVESRTTDLLTD
WEKTKPVTGNLRPEEALQALTIYEGKFGRLKDDREKCAKAKEALELTDTGLLSGSEERVQ
VALEELQDLKGVWSELSKVWEQIDQMKEQPWVSVQPRKLRQNLDALLNQLKSFPARLRQY
ASYEFVQRLLKGYMKINMLVIELKSEALKDRHWKQLMKRLHVNWVVSELTLGQIWDVDLQ
KNEAIVKDVLLVAQGEMALEEFLKQIREVWNTYELDLVNYQNKCRLIRGWDDLFNKVKEH
INSVSAMKLSPYYKVFEEDALSWEDKLNRIMALFDVWIDVQRRWVYLEGIFTGSADIKHL
LPVETQRFQSISTEFLALMKKVSKSPLVMDVLNIQGVQRSLERLADLLGKIQKALGEYLE
RERSSFPRFYFVGDEDLLEIIGNSKNVAKLQKHFKKMFAGVSSIILNEDNSVVLGISSRE
GEEVMFKTPVSITEHPKINEWLTLVEKEMRVTLAKLLAESVTEVEIFGKATSIDPNTYIT
WIDKYQAQLVVLSAQIAWSENVETALSSMGGGGDAAPLHSVLSNVEVTLNVLADSVLMEQ
PPLRRRKLEHLITELVHQRDVTRSLIKSKIDNAKSFEWLSQMRFYFDPKQTDVLQQLSIQ
MANAKFNYGFEYLGVQDKLVQTPLTDRCYLTMTQALEARLGGSPFGPAGTGKTESVKALG
HQLGRFVLVFNCDETFDFQAMGRIFVGLCQVGAWGCFDEFNRLEERMLSAVSQQVQCIQE
ALREHSNPNYDKTSAPITCELLNKQVKVSPDMAIFITMNPGYAGRSNLPDNLKKLFRSLA
MTKPDRQLIAQVMLYSQGFRTAEVLANKIVPFFKLCDEQLSSQSHYDFGLRALKSVLVSA
GNVKRERIQKIKREKEERGEAVDEGEIAENLPEQEILIQSVCETMVPKLVAEDIPLLFSL
LSDVFPGVQYHRGEMTALREELKKVCQEMYLTYGDGEEVGGMWVEKVLQLYQITQINHGL
MMVGPSGSGKSMAWRVLLKALERLEGVEGVAHIIDPKAISKDHLYGTLDPNTREWTDGLF
THVLRKIIDSVRGELQKRQWIVFDGDVDPEWVENLNSVLDDNKLLTLPNGERLSLPPNVR
IMFEVQDLKYATLATVSRCGMVWFSEDVLSTDMIFNNFLARLRSIPLDEGEDEAQRRRKG
KEDEGEEAASPMLQIQRDAATIMQPYFTSNGLVTKALEHAFQLEHIMDLTRLRCLGSLFS
MLHQACRNVAQYNANHPDFPMQIEQLERYIQRYLVYAILWSLSGDSRLKMRAELGEYIRR
ITTVPLPTAPNIPIIDYEVSISGEWSPWQAKVPQIEVETHKVAAPDVVVPTLDTVRHEAL
LYTWLAEHKPLVLCGPPGSGKTMTLFSALRALPDMEVVGLNFSSATTPELLLKTFDHYCE
YRRTPNGVVLAPVQLGKWLVLFCDEINLPDMDKYGTQRVISFIRQMVEHGGFYRTSDQTW
VKLERIQFVGACNPPTDPGRKPLSHRFLRHVPVVYVDYPGPASLTQIYGTFNRAMLRLIP
SLRTYAEPLTAAMVEFYTMSQERFTQDTQPHYIYSPREMTRWVRGIFEALRPLETLPVEG
LIRIWAHEALRLFQDRLVEDEERRWTDENIDTVALKHFPNIDREKAMSRPILYSNWLSKD
YIPVDQEELRDYVKARLKVFYEEELDVPLVLFNEVLDHVLRIDRIFRQPQGHLLLIGVSG
AGKTTLSRFVAWMNGLSVYQIKVHRKYTGEDFDEDLRTVLRRSGCKNEKIAFIMDESNVL
DSGFLERMNTLLANGEVPGLFEGDEYATLMTQCKEGAQKEGLMLDSHEELYKWFTSQVIR
NLHVVFTMNPSSEGLKDRAATSPALFNRCVLNWFGDWSTEALYQVGKEFTSKMDLEKPNY
IVPDYMPVVYDKLPQPPSHREAIVNSCVFVHQTLHQANARLAKRGGRTMAITPRHYLDFI
NHYANLFHEKRSELEEQQMHLNVGLRKIKETVDQVEELRRDLRIKSQELEVKNAAANDKL
KKMVKDQQEAEKKKVMSQEIQEQLHKQQEVIADKQMSVKEDLDKVEPAVIEAQNAVKSIK
KQHLVEVRSMANPPAAVKLALESICLLLGESTTDWKQIRSIIMRENFIPTIVNFSAEEIS
DAIREKMKKNYMSNPSYNYEIVNRASLACGPMVKWAIAQLNYADMLKRVEPLRNELQKLE

DDAKDNQQKANEVEQMIRDLEASIARYKEEYAVLISEAQAIKADLAAVEAKVNRSTALLK
SLSAERERWEKTSETFKNQMSTIAGDCLLSAAFIAYAGYFDQQMRQNLFTTWSHHLQQAN
IQFRTDIARTEYLSNADERLRWQASSLPADDLCTENAIMLKRFNRYPLIIDPSGQATEFI
MNEYKDRKITRTSFLDDAFRKNLESALRFGNPLLVQDVESYDPVLNPVLNREVRRTGGRV
LITLGDQDIDLSPSFVIFLSTRDPTVEFPPDLCSRVTFVNFTVTRSSLQSQCLNEVLKAE
RPDVDEKRSDLLKLQGEFQLRLRQLEKSLLQALNEVKGRILDDDTIITTLENLKREAAEV
TRKVEETDIVMQEVETVSQQYLPLSTACSSIYFTMESLKQIHFLYQYSLQFFLDIYHNVL
YENPNLKGVTDHTQRLSIITKDLFQVAFNRVARGMLHQDHITFAMLLARIKLKGTVGEPT
YDAEFQHFLRGNEIVLSAGSTPRIQGLTVEQAEAVVRLSCLPAFKDLIAKVQADEQFGIW
LDSSSPEQTVPYLWSEETPATPIGQAIHRLLLIQAFRPDRLLAMAHMFVSTNLGESFMSI
MEQPLDLTHIVGTEVKPNTPVLMCSVPGYDASGHVEDLAAEQNTQITSIAIGSAEGFNQA
DKAINTAVKSGRWVMLKNVHLAPGWLMQLEKKLHSLQPHACFRLFLTMEINPKVPVNLLR
AGRIFVFEPPPGVKANMLRTFSSIPVSRICKSPNERARLYFLLAWFHAIIQERLRYAPLG
WSKKYEFGESDLRSACDTVDTWLDDTAKGRQNISPDKIPWSALKTLMAQSIYGGRVDNEF
DQRLLNTFLERLFTTRSFDSEFKLACKVDGHKDIQMPDGIRREEFVQWVELLPDTQTPSW
LGLPNNAERVLLTTQGVDMISKMLKMQMLEDEDDLAYAETEKKTRTDSTSDGRPAWMRTL
HTTASNWLHLIPQTLSHLKRTVENIKDPLFRFFEREVKMGAKLLQDVRQDLADVVQVCEG
KKKQTNYLRTLINELVKGILPRSWSHYTVPAGMTVIQWVSDFSERIKQLQNISLAAASGG
AKELKNIHVCLGGLFVPEAYITATRQYVAQANSWSLEELCLEVNVTTSQGATLDACSFGV
TGLKLQGATCNNNKLSLSNAISTALPLTQLRWVKQTNTEKKASVVTLPVYLNFTRADLIF
TVDFEIATKEDPRSFYERGVAVLCTE
>sp|Q14240|IF4A2_HUMAN Eukaryotic initiation factor 4A-II OS=Homo sapiens OX=9606
GN=EIF4A2 PE=1 SV=2
MSGGSADYNREHGGPEGMDPDGVIESNWNEIVDNFDDMNLKESLLRGIYAYGFEKPSAIQ
QRAIIPCIKGYDVIAQAQSGTGKTATFAISILQQLEIEFKETQALVLAPTRELAQQIQKV
ILALGDYMGATCHACIGGTNVRNEMQKLQAEAPHIVVGTPGRVFDMLNRRYLSPKWIKMF
VLDEADEMLSRGFKDQIYEIFQKLNTSIQVVLLSATMPTDVLEVTKKFMRDPIRILVKKE
ELTLEGIKQFYINVEREEWKLDTLCDLYETLTITQAVIFLNTRRKVDWLTEKMHARDFTV
SALHGDMDQKERDVIMREFRSGSSRVLITTDLLARGIDVQQVSLVINYDLPTNRENYIHR
IGRGGRFGRKGVAINFVTEEDKRILRDIETFYNTTVEEMPMNVADLI
>sp|Q14254|FLOT2_HUMAN Flotillin-2 OS=Homo sapiens OX=9606 GN=FLOT2 PE=1 SV=2
MGNCHTVGPNEALVVSGGCCGSDYKQYVFGGWAWAWWCISDTQRISLEIMTLQPRCEDVE
TAEGVALTVTGVAQVKIMTEKELLAVACEQFLGKNVQDIKNVVLQTLEGHLRSILGTLTV
EQIYQDRDQFAKLVREVAAPDVGRMGIEILSFTIKDVYDKVDYLSSLGKTQTAVVQRDAD
IGVAEAERDAGIREAECKKEMLDVKFMADTKIADSKRAFELQKSAFSEEVNIKTAEAQLA
YELQGAREQQKIRQEEIEIEVVQRKKQIAVEAQEILRTDKELIATVRRPAEAEAHRIQQI
AEGEKVKQVLLAQAEAEKIRKIGEAEAAVIEAMGKAEAERMKLKAEAYQKYGDAAKMALV
LEALPQIAAKIAAPLTKVDEIVVLSGDNSKVTSEVNRLLAELPASVHALTGVDLSKIPLI
KKATGVQV
>sp|Q14315|FLNC_HUMAN Filamin-C OS=Homo sapiens OX=9606 GN=FLNC PE=1 SV=3
MMNNSGYSDAGLGLGDETDEMPSTEKDLAEDAPWKKIQQNTFTRWCNEHLKCVGKRLTDL
QRDLSDGLRLIALLEVLSQKRMYRKFHPRPNFRQMKLENVSVALEFLEREHIKLVSIDSK
AIVDGNLKLILGLIWTLILHYSISMPMWEDEDDEDARKQTPKQRLLGWIQNKVPQLPITN
FNRDWQDGKALGALVDNCAPGLCPDWEAWDPNQPVENAREAMQQADDWLGVPQVIAPEEI

VDPNVDEHSVMTYLSQFPKAKLKPGAPVRSKQLNPKKAIAYGPGIEPQGNTVLQPAHFTV
QTVDAGVGEVLVYIEDPEGHTEEAKVVPNNDKDRTYAVSYVPKVAGLHKVTVLFAGQNIE
RSPFEVNVGMALGDANKVSARGPGLEPVGNVANKPTYFDIYTAGAGTGDVAVVIVDPQGR
RDTVEVALEDKGDSTFRCTYRPAMEGPHTVHVAFAGAPITRSPFPVHVSEACNPNACRAS
GRGLQPKGVRVKEVADFKVFTKGAGSGELKVTVKGPKGTEEPVKVREAGDGVFECEYYPV
VPGKYVVTITWGGYAIPRSPFEVQVSPEAGVQKVRAWGPGLETGQVGKSADFVVEAIGTE
VGTLGFSIEGPSQAKIECDDKGDGSCDVRYWPTEPGEYAVHVICDDEDIRDSPFIAHILP
APPDCFPDKVKAFGPGLEPTGCIVDKPAEFTIDARAAGKGDLKLYAQDADGCPIDIKVIP
NGDGTFRCSYVPTKPIKHTIIISWGGVNVPKSPFRVNVGEGSHPERVKVYGPGVEKTGLK
ANEPTYFTVDCSEAGQGDVSIGIKCAPGVVGPAEADIDFDIIKNDNDTFTVKYTPPGAGR
YTIMVLFANQEIPASPFHIKVDPSHDASKVKAEGPGLNRTGVEVGKPTHFTVLTKGAGKA
KLDVQFAGTAKGEVVRDFEIIDNHDYSYTVKYTAVQQGNMAVTVTYGGDPVPKSPFVVNV
APPLDLSKIKVQGLNSKVAVGQEQAFSVNTRGAGGQGQLDVRMTSPSRRPIPCKLEPGGG
AEAQAVRYMPPEEGPYKVDITYDGHPVPGSPFAVEGVLPPDPSKVCAYGPGLKGGLVGTP
APFSIDTKGAGTGGLGLTVEGPCEAKIECQDNGDGSCAVSYLPTEPGEYTINILFAEAHI
PGSPFKATIRPVFDPSKVRASGPGLERGKVGEAATFTVDCSEAGEAELTIEILSDAGVKA
EVLIHNNADGTYHITYSPAFPGTYTITIKYGGHPVPKFPTRVHVQPAVDTSGVKVSGPGV
EPHGVLREVTTEFTVDARSLTATGGNHVTARVLNPSGAKTDTYVTDNGDGTYRVQYTAYE
EGVHLVEVLYDEVAVPKSPFRVGVTEGCDPTRVRAFGPGLEGGLVNKANRFTVETRGAGT
GGLGLAIEGPSEAKMSCKDNKDGSCTVEYIPFTPGDYDVNITFGGRPIPGSPFRVPVKDV
VDPGKVKCSGPGLGAGVRARVPQTFTVDCSQAGRAPLQVAVLGPTGVAEPVEVRDNGDGT
HTVHYTPATDGPYTVAVKYADQEVPRSPFKIKVLPAHDASKVRASGPGLNASGIPASLPV
EFTIDARDAGEGLLTVQILDPEGKPKKANIRDNGDGTYTVSYLPDMSGRYTITIKYGGDE
IPYSPFRIHALPTGDASKCLVTVSIGGHGLGACLGPRIQIGQETVITVDAKAAGEGKVTC
TVSTPDGAELDVDVVENHDGTFDIYYTAPEPGKYVITIRFGGEHIPNSPFHVLACDPLPH
EEEPSEVPQLRQPYAPPRPGARPTHWATEEPVVPVEPMESMLRPFNLVIPFAVQKGELTG
EVRMPSGKTARPNITDNKDGTITVRYAPTEKGLHQMGIKYDGNHIPGSPLQFYVDAINSR
HVSAYGPGLSHGMVNKPATFTIVTKDAGEGGLSLAVEGPSKAEITCKDNKDGTCTVSYLP
TAPGDYSIIVRFDDKHIPGSPFTAKITGDDSMRTSQLNVGTSTDVSLKITESDLSQLTAS
IRAPSGNEEPCLLKRLPNRHIGISFTPKEVGEHVVSVRKSGKHVTNSPFKILVGPSEIGD
ASKVRVWGKGLSEGHTFQVAEFIVDTRNAGYGGLGLSIEGPSKVDINCEDMEDGTCKVTY
CPTEPGTYIINIKFADKHVPGSPFTVKVTGEGRMKESITRRRQAPSIATIGSTCDLNLKI
PGNWFQMVSAQERLTRTFTRSSHTYTRTERTEISKTRGGETKREVRVEESTQVGGDPFPA
VFGDFLGRERLGSFGSITRQQEGEASSQDMTAQVTSPSGKVEAAEIVEGEDSAYSVRFVP
QEMGPHTVAVKYRGQHVPGSPFQFTVGPLGEGGAHKVRAGGTGLERGVAGVPAEFSIWTR
EAGAGGLSIAVEGPSKAEIAFEDRKDGSCGVSYVVQEPGDYEVSIKFNDEHIPDSPFVVP
VASLSDDARRLTVTSLQETGLKVNQPASFAVQLNGARGVIDARVHTPSGAVEECYVSELD
SDKHTIRFIPHENGVHSIDVKFNGAHIPGSPFKIRVGEQSQAGDPGLVSAYGPGLEGGTT
GVSSEFIVNTLNAGSGALSVTIDGPSKVQLDCRECPEGHVVTYTPMAPGNYLIAIKYGGP
QHIVGSPFKAKVTGPRLSGGHSLHETSTVLVETVTKSSSSRGSSYSSIPKFSSDASKVVT
RGPGLSQAFVGQKNSFTVDCSKAGTNMMMVGVHGPKTPCEEVYVKHMGNRVYNVTYTVKE
KGDYILIVKWGDESVPGSPFKVKVP
>sp|Q14344|GNA13_HUMAN Guanine nucleotide-binding protein subunit alpha-13 OS=Homo sapiens OX=9606 GN=GNA13 PE=1 SV=2

MADFLPSRSVLSVCFPGCLLTSGEAEQQRKSKEIDKCLSREKTYVKRLVKILLLGAGESG
KSTFLKQMRIIHGQDFDQRAREEFRPTIYSNVIKGMRVLVDAREKLHIPWGDNSNQQHGD
KMMSFDTRAPMAAQGMVETRVFLQYLPAIRALWADSGIQNAYDRRREFQLGESVKYFLDN
LDKLGEPDYIPSQQDILLARRPTKGIHEYDFEIKNVPFKMVDVGGQRSERKRWFECFDSV
TSILFLVSSSEFDQVLMEDRLTNRLTESLNIFETIVNNRVFSNVSIILFLNKTDLLEEKV
QIVSIKDYFLEFEGDPHCLRDVQKFLVECFRNKRRDQQQKPLYHHFTTAINTENIRLVFR
DVKDTILHDNLKQLMLQ
>sp|Q14376|GALE_HUMAN UDP-glucose 4-epimerase OS=Homo sapiens OX=9606 GN=GALE PE=1 SV=2
MAEKVLVTGGAGYIGSHTVLELLEAGYLPVVIDNFHNAFRGGGSLPESLRRVQELTGRSV
EFEEMDILDQGALQRLFKKYSFMAVIHFAGLKAVGESVQKPLDYYRVNLTGTIQLLEIMK
AHGVKNLVFSSSATVYGNPQYLPLDEAHPTGGCTNPYGKSKFFIEEMIRDLCQADKTWNA
VLLRYFNPTGAHASGCIGEDPQGIPNNLMPYVSQVAIGRREALNVFGNDYDTEDGTGVRD
YIHVVDLAKGHIAALRKLKEQCGCRIYNLGTGTGYSVLQMVQAMEKASGKKIPYKVVARR
EGDVAACYANPSLAQEELGWTAALGLDRMCEDLWRWQKQNPSGFGTQA
>sp|Q14558|KPRA_HUMAN Phosphoribosyl pyrophosphate synthase-associated protein 1 OS=Homo sapiens OX=9606 GN=PRPSAP1 PE=1 SV=2
MNAARTGYRVFSANSTAACTELAKRITERLGAELGKSVVYQETNGETRVEIKESVRGQDI
FIIQTIPRDVNTAVMELLIMAYALKTACARNIIGVIPYFPYSKQSKMRKRGSIVCKLLAS
MLAKAGLTHIITMDLHQKEIQGFFSFPVDNLRASPFLLQYIQEEIPNYRNAVIVAKSPDA
AKRAQSYAERLRLGLAVIHGEAQCTELDMDDGRHSPPMVKNATVHPGLELPLMMAKEKPP
ITVVGDVGGRIAIIVDDIIDDVESFVAAAEILKERGAYKIYVMATHGILSAEAPRLIEES
SVDEVVVTNTVPHEVQKLQCPKIKTVDISLILSEAIRRIHNGESMAYLFRNITVDD
>sp|Q14692|BMS1_HUMAN Ribosome biogenesis protein BMS1 homolog OS=Homo sapiens OX=9606 GN=BMS1 PE=1 SV=1
MEAKDQKKHRKKNSGPKAAKKKKRLLQDLQLGDEEDARKRNPKAFAVQSAVRMARSFHRT
QDLKTKKHHIPVVDRTPLEPPPIVVVVMGPPKVGKSTLIQCLIRNFTRQKLTEIRGPVTI
VSGKKRRLTIIECGCDINMMIDLAKVADLVLMLIDASFGFEMETFEFLNICQVHGFPKIM
GVLTHLDSFKHNKQLKKTKKRLKHRFWTEVYPGAKLFYLSGMVHGEYQNQEIHNLGRFIT
VMKFRPLTWQTSHPYILADRMEDLTNPEDIRTNIKCDRKVSLYGYLRGAHLKNKSQIHMP
GVGDFAVSDISFLPDPCALPEQQKKRCLNEKEKLVYAPLSGVGGVLYDKDAVYVDLGGSH
VFQDEVGPTHELVQSLISTHSTIDAKMASSRVTLFSDSKPLGSEDIDNQGLMMPKEEKQM
DLNTGRMRRKAIFGDEDESGDSDDEEDDEMSEDDGLENGSSDEEAEEEENAEMTDQYMAV
KGIKRRKLELEEDSEMDLPAFADSDDDLERSSAEEGEAEEADESSEEEDCTAGEKGISGS
KAAGEGSKAGLSPANCQSDRVNLEKSLLMKKAALPTFDSGHCTAEEVFASEDESEESSSL
SAEEEDSENEEAIRKKLSKPSQVSSGQKLGPQNFIDETSDIENLLKEEEDYKEENNDSKE
TSGALKWKEDLSRKAAEAFLRQQQAAPNLRKLIYGTVTEDNEEEDDDTLEELGGLFRVNQ
PDRECKHKADSLDCSRFLVEAPHDWDLEEVMNSIRDCFVTGKWEDDKDAAKVLAEDEELY
GDFEDLETGDVHKGKSGPNTQNEDIEKEVKEEIDPDEEESAKKKHLDKKRKLKEMFDAEY
DEGESTYFDDLKGEMQKQAQLNRAEFEDQDDEARVQYEGFRPGMYVRIEIENVPCEFVQN
FDPHYPIILGGLGNSEGNVGYVQMRLKKHRWYKKILKSRDPIIFSVGWRRFQTIPLYYIE
DHNGRQRLLKYTPQHMHCGAAFWGPITPQGTGFLAIQSVSGIMPDFRIAATGVVLDLDKS
IKIVKKLKLTGFPYKIFKNTSFIKGMFNSALEVAKFEGAVIRTVSGIRGQIKKALRAPEG
AFRASFEDKLLMSDIVFMRTWYPVSIPAFYNPVTSLLKPVGEKDTWSGMRTTGQLRLAHG

VRLKANKDSLYKPILRQKKHFNSLHIPKALQKALPFKNKPKTQAKAGKVPKDRRRPAVIR
EPHERKILALLDALSTVHSQKMKKAKEQRHLHNKEHFRAKQKEEEEKLKRQKDLRKKLFR
IQGQKERRNQKSSLKGAEGQLQ
>sp|Q14697|GANAB_HUMAN Neutral alpha-glucosidase AB OS=Homo sapiens OX=9606 GN=GANAB PE=1 SV=3
MAAVAAVAARRRRSWASLVLAFLGVCLGITLAVDRSNFKTCEESSFCKRQRSIRPGLSPY
RALLDSLQLGPDSLTVHLIHEVTKVLLVLELQGLQKNMTRFRIDELEPRRPRYRVPDVLV
ADPPIARLSVSGRDENSVELTMAEGPYKIILTARPFRLDLLEDRSLLLSVNARGLLEFEH
QRAPRVSQGSKDPAEGDGAQPEETPRDGDKPEETQGKAEKDEPGAWEETFKTHSDSKPYG
PMSVGLDFSLPGMEHVYGIPEHADNLRLKVTEGGEPYRLYNLDVFQYELYNPMALYGSVP
VLLAHNPHRDLGIFWLNAAETWVDISSNTAGKTLFGKMMDYLQGSGETPQTDVRWMSETG
IIDVFLLLGPSISDVFRQYASLTGTQALPPLFSLGYHQSRWNYRDEADVLEVDQGFDDHN
LPCDVIWLDIEHADGKRYFTWDPSRFPQPRTMLERLASKRRKLVAIVDPHIKVDSGYRVH
EELRNLGLYVKTRDGSDYEGWCWPGSAGYPDFTNPTMRAWWANMFSYDNYEGSAPNLFVW
NDMNEPSVFNGPEVTMLKDAQHYGGWEHRDVHNIYGLYVHMATADGLRQRSGGMERPFVL
ARAFFAGSQRFGAVWTGDNTAEWDHLKISIPMCLSLGLVGLSFCGADVGGFFKNPEPELL
VRWYQMGAYQPFFRAHAHLDTGRREPWLLPSQHNDIIRDALGQRYSLLPFWYTLLYQAHR
EGIPVMRPLWVQYPQDVTTFNIDDQYLLGDALLVHPVSDSGAHGVQVYLPGQGEVWYDIQ
SYQKHHGPQTLYLPVTLSSIPVFQRGGTIVPRWMRVRRSSECMKDDPITLFVALSPQGTA
QGELFLDDGHTFNYQTRQEFLLRRFSFSGNTLVSSSADPEGHFETPIWIERVVIIGAGKP
AAVVLQTKGSPESRLSFQHDPETSVLVLRKPGINVASDWSIHLR
>sp|Q14764|MVP_HUMAN Major vault protein OS=Homo sapiens OX=9606 GN=MVP PE=1 SV=4
MATEEFIIRIPPYHYIHVLDQNSNVSRVEVGPKTYIRQDNERVLFAPMRMVTVPPRHYCT
VANPVSRDAQGLVLFDVTGQVRLRHADLEIRLAQDPFPLYPGEVLEKDITPLQVVLPNTA
LHLKALLDFEDKDGDKVVAGDEWLFEGPGTYIPRKEVEVVEIIQATIIRQNQALRLRARK
ECWDRDGKERVTGEEWLVTTVGAYLPAVFEEVLDLVDAVILTEKTALHLRARRNFRDFRG
VSRRTGEEWLVTVQDTEAHVPDVHEEVLGVVPITTLGPHNYCVILDPVGPDGKNQLGQKR
VVKGEKSFFLQPGEQLEQGIQDVYVLSEQQGLLLRALQPLEEGEDEEKVSHQAGDHWLIR
GPLEYVPSAKVEVVEERQAIPLDENEGIYVQDVKTGKVRAVIGSTYMLTQDEVLWEKELP
PGVEELLNKGQDPLADRGEKDTAKSLQPLAPRNKTRVVSYRVPHNAAVQVYDYREKRARV
VFGPELVSLGPEEQFTVLSLSAGRPKRPHARRALCLLLGPDFFTDVITIETADHARLQLQ
LAYNWHFEVNDRKDPQETAKLFSVPDFVGDACKAIASRVRGAVASVTFDDFHKNSARIIR
TAVFGFETSEAKGPDGMALPRPRDQAVFPQNGLVVSSVDVQSVEPVDQRTRDALQRSVQL
AIEITTNSQEAAAKHEAQRLEQEARGRLERQKILDQSEAEKARKELLELEALSMAVESTG
TAKAEAESRAEAARIEGEGSVLQAKLKAQALAIETEAELQRVQKVRELELVYARAQLELE
VSKAQQLAEVEVKKFKQMTEAIGPSTIRDLAVAGPEMQVKLLQSLGLKSTLITDGSTPIN
LFNTAFGLLGMGPEGQPLGRRVASGPSPGEGISPQSAQAPQAPGDNHVVPVLR
>sp|Q14767|LTBP2_HUMAN Latent-transforming growth factor beta-binding protein 2 OS=Homo sapiens OX=9606 GN=LTBP2 PE=1 SV=3
MRPRTKARSPGRALRNPWRGFLPLTLALFVGAGHAQRDPVGRYEPAGGDANRLRRPGGSY
PAAAAAKVYSLFREQDAPVAGLQPVERAQPGWGSPRRPTEAEARRPSRAQQSRRVQPPAQ
TRRSTPLGQQQPAPRTRAAPALPRLGTPQRSGAAPPTPPRGRLTGRNVCGGQCCPGWTTA
NSTNHCIKPVCEPPCQNRGSCSRPQLCVCRSGFRGARCEEVIPDEEFDPQNSRLAPRRWA

ERSPNLRRSSAAGEGTLARAQPPAPQSPPAPQSPPAGTLSGLSQTHPSQQHVGLSRTVRL
HPTATASSQLSSNALPPGPGLEQRDGTQQAVPLEHPSSPWGLNLTEKIKKIKIVFTPTIC
KQTCARGHCANSCERGDTTTLYSQGGHGHDPKSGFRIYFCQIPCLNGGRCIGRDECWCPA
NSTGKFCHLPIPQPDREPPGRGSRPRALLEAPLKQSTFTLPLSNQLASVNPSLVKVHIHH
PPEASVQIHQVAQVRGGVEEALVENSVETRPPPWLPASPGHSLWDSNNIPARSGEPPRPL
PPAAPRPRGLLGRCYLNTVNGQCANPLLELTTQEDCCGSVGAFWGVTLCAPCPPRPASPV
IENGQLECPQGYKRLNLTHCQDINECLTLGLCKDAECVNTRGSYLCTCRPGLMLDPSRSR
CVSDKAISMLQGLCYRSLGPGTCTLPLAQRITKQICCCSRVGKAWGSECEKCPLPGTEAF
REICPAGHGYTYASSDIRLSMRKAEEEELARPPREQGQRSSGALPGPAERQPLRVVTDTW
LEAGTIPDKGDSQAGQVTTSVTHAPAWVTGNATTPPMPEQGIAEIQEEQVTPSTDVLVTL
STPGIDRCAAGATNVCGPGTCVNLPDGYRCVCSPGYQLHPSQAYCTDDNECLRDPCKGKG
RCINRVGSYSCFCYPGYTLATSGATQECQDINECEQPGVCSGGQCTNTEGSYHCECDQGY
IMVRKGHCQDINECRHPGTCPDGRCVNSPGSYTCLACEEGYRGQSGSCVDVNECLTPGVC
AHGKCTNLEGSFRCSCEQGYEVTSDEKGCQDVDECASRASCPTGLCLNTEGSFACSACEN
GYWVNEDGTACEDLDECAFPGVCPSGVCTNTAGSFSCKDCDGGYRPSPLGDSCEDVDECE
DPQSSCLGGECKNTVGSYQCLCPQGFQLANGTVCEDVNECMGEEHCAPHGECLNSHGSFF
CLCAPGFVSAEGGTSCQDVDECATTDPCVGGHCVNTEGSFNCLCETGFQPSPESGECVDI
DECEDYGDPVCGTWKCENSPGSYRCVLGCQPGFHMAPNGDCIDIDECANDTMCGSHGFCD
NTDGSFRCLCDQGFEISPSGWDCVDVNECELMLAVCGAALCENVEGSFLCLCASDLEEYD
AQEGHCRPRGAGGQSMSEAPTGDHAPAPTRMDCYSGQKGHAPCSSVLGRNTTQAECCCTQ
GASWGDACDLCPSEDSAEFSEICPSGKGYIPVEGAWTFGQTMYTDADECVIFGPGLCPNG
RCLNTVPGYVCLCNPGFHYDASHKKCEDHDECQDLACENGECVNTEGSFHCFCSPPLTLD
LSQQRCMNSTSSTEDLPDHDIHMDICWKKVTNDVCSEPLRGHRTTYTECCCQDGEAWSQQ
CALCPPRSSEVYAQLCNVARIEAEREAGVHFRPGYEYGPGPDDLHYSIYGPDGAPFYNYL
GPEDTVPEPAFPNTAGHSADRTPILESPLQPSELQPHYVASHPEPPAGFEGLQAEECGIL
NGCENGRCVRVREGYTCDCFEGFQLDAAHMACVDVNECDDLNGPAVLCVHGYCENTEGSY
RCHCSPGYVAEAGPPHCTAKE
>tr|Q14936|Q14936_HUMAN Interferon-gamma receptor alpha chain OS=Homo sapiens
OX=9606 PE=3 SV=1
MALLFLLPLVMQGVSRAEMGTADLGPSSVPTPTNVTIESYNMNPIVYWEYQIMPQVPVFT
VEVKNYGVKNSEWIDACINISHHYCNISDHVGDPSNSLWVRVKARVGQKESAYAKSEEFA
VCRDGKIGPPKLDIRKEEKQIMIDIFHPSVFVNGDEQEVDYDPETTCYIRVYNVYVRMNG
SEIQYKILTQKEDDCDEIQCQLAIPVSSLNSQYCSAEGVLHVWGVTTEKSKEVCITIFNS
SIKGSLWIPVVAALVLSLVFICFYIKKINPLKEKSIILPKSLISVVRSATLETKPESKYV
SLITSYQPFSLEKEVVCEEPLSPATVPGMHTEDNPGKVEHTEELSSITEVVTTEENIPDV
VPGSHLTPIERESSSPLSSNQSEPGSIALNSYHSRNCSESDHSRNGFDTDSSCLESHSSL
SDSEFPPNNKGEIKTEGQELITVIKAPTSFGYDKPHVLVDLLVDDSGKESLIGYRPTEDS
KEFS
>sp|Q14974|IMB1_HUMAN Importin subunit beta-1 OS=Homo sapiens OX=9606 GN=KPNB1
PE=1 SV=2
MELITILEKTVSPDRLELEAAQKFLERAAVENLPTFLVELSRVLANPGNSQVARVAAGLQ
IKNSLTSKDPDIKAQYQQRWLAIDANARREVKNYVLQTLGTETYRPSSASQCVAGIACAE
IPVNQWPELIPQLVANVTNPNSTEHMKESTLEAIGYICQDIDPEQLQDKSNEILTAIIQG
MRKEEPSNNVKLAATNALLNSLEFTKANFDKESERHFIMQVVCEATQCPDTRVRVAALQN

LVKIMSLYYQYMETYMGPALFAITIEAMKSDIDEVALQGIEFWSNVCDEEMDLAIEASEA
AEQGRPPEHTSKFYAKGALQYLVPILTQTLTKQDENDDDDDWNPCKAAGVCLMLLATCCE
DDIVPHVLPFIKEHIKNPDWRYRDAAVMAFGCILEGPEPSQLKPLVIQAMPTLIELMKDP
SVVVRDTAAWTVGRICELLPEAAINDVYLAPLLQCLIEGLSAEPRVASNVCWAFSSLAEA
AYEAADVADDQEEPATYCLSSSFELIVQKLLETTDRPDGHQNNLRSSAYESLMEIVKNSA
KDCYPAVQKTTLVIMERLQQVLQMESHIQSTSDRIQFNDLQSLLCATLQNVLRKVQHQDA
LQISDVVMASLLRMFQSTAGSGGVQEDALMAVSTLVEVLGGEFLKYMEAFKPFLGIGLKN
YAEYQVCLAAVGLVGDLCRALQSNIIPFCDEVMQLLLENLGNENVHRSVKPQILSVFGDI
ALAIGGEFKKYLEVVLNTLQQASQAQVDKSDYDMVDYLNELRESCLEAYTGIVQGLKGDQ
ENVHPDVMLVQPRVEFILSFIDHIAGDEDHTDGVVACAAGLIGDLCTAFGKDVLKLVEAR
PMIHELLTEGRRSKTNKAKTLATWATKELRKLKNQA
>sp|Q14997|PSME4_HUMAN Proteasome activator complex subunit 4 OS=Homo sapiens
OX=9606 GN=PSME4 PE=1 SV=2
MEPAERAGVGEPPEPGGRPEPGPRGFVPQKEIVYNKLLPYAERLDAESDLQLAQIKCNLG
RAVQLQELWPGGLFWTRKLSTYIRLYGRKFSKEDHVLFIKLLYELVSIPKLEISMMQGFA
RLLINLLKKKELLSRADLELPWRPLYDMVERILYSKTEHLGLNWFPNSVENILKTLVKSC
RPYFPADATAEMLEEWRPLMCPFDVTMQKAITYFEIFLPTSLPPELHHKGFKLWFDELIG
LWVSVQNLPQWEGQLVNLFARLATDNIGYIDWDPYVPKIFTRILRSLNLPVGSSQVLVPR
FLTNAYDIGHAVIWITAMMGGPSKLVQKHLAGLFNSITSFYHPSNNGRWLNKLMKLLQRL
PNSVVRRLHRERYKKPSWLTPVPDSHKLTDQDVTDFVQCIIQPVLLAMFSKTGSLEAAQA
LQNLALMRPELVIPPVLERTYPALETLTEPHQLTATLSCVIGVARSLVSGGRWFPEGPTH
MLPLLMRALPGVDPNDFSKCMITFQFIATFSTLVPLVDCSSVLQERNDLTEVERELCSAT
AEFEDFVLQFMDRCFGLIESSTLEQTREETETEKMTHLESLVELGLSSTFSTILTQCSKE
IFMVALQKVFNFSTSHIFETRVAGRMVADMCRAAVKCCPEESLKLFVPHCCSVITQLTMN
DDVLNDEELDKELLWNLQLLSEITRVDGRKLLLYREQLVKILQRTLHLTCKQGYTLSCNL
LHHLLRSTTLIYPTEYCSVPGGFDKPPSEYFPIKDWGKPGDLWNLGIQWHVPSSEEVSFA
FYLLDSFLQPELVKLQHCGDGKLEMSRDDILQSLTIVHNCLIGSGNLLPPLKGEPVTNLV
PSMVSLEETKLYTGLEYDLSRENHREVIATVIRKLLNHILDNSEDDTKSLFLIIKIIGDL
LQFQGSHKHEFDSRWKSFNLVKKSMENRLHGKKQHIRALLIDRVMLQHELRTLTVEGCEY
KKIHQDMIRDLLRLSTSSYSQVRNKAQQTFFAALGAYNFCCRDIIPLVLEFLRPDRQGVT
QQQFKGALYCLLGNHSGVCLANLHDWDCIVQTWPAIVSSGLSQAMSLEKPSIVRLFDDLA
EKIHRQYETIGLDFTIPKSCVEIAELLQQSKNPSINQILLSPEKIKEGIKRQQEKNADAL
RNYENLVDTLLDGVEQRNLPWKFEHIGIGLLSLLLRDDRVLPLRAIRFFVENLNHDAIVV
RKMAISAVAGILKQLKRTHKKLTINPCEISGCPKPTQIIAGDRPDNHWLHYDSKTIPRTK
KEWESSCFVEKTHWGYYTWPKNMVVYAGVEEQPKLGRSREDMTEAEQIIFDHFSDPKFVE
QLITFLSLEDRKGKDKFNPRRFCLFKGIFRNFDDAFLPVLKPHLEHLVADSHESTQRCVA
EIIAGLIRGSKHWTFEKVEKLWELLCPLLRTALSNITVETYNDWGACIATSCESRDPRKL
HWLFELLLESPLSGEGGSFVDACRLYVLQGGLAQQEWRVPELLHRLLKYLEPKLTQVYKN
VRERIGSVLTYIFMIDVSLPNTTPTISPHVPEFTARILEKLKPLMDVDEEIQNHVMEENG
IGEEDERTQGIKLLKTILKWLMASAGRSFSTAVTEQLQLLPLFFKIAPVENDNSYDELKR
DAKLCLSLMSQGLLYPHQVPLVLQVLKQTARSSSWHARYTVLTYLQTMVFYNLFIFLNNE
DAVKDIRWLVISLLEDEQLEVREMAATTLSGLLQCNFLTMDSPMQIHFEQLCKTKLPKKR
KRDPGSVGDTIPSAELVKRHAGVLGLGACVLSSPYDVPTWMPQLLMNLSAHLNDPQPIEM
TVKKTLSNFRRTHHDNWQEHKQQFTDDQLLVLTDLLVSPCYYA

>tr|Q14CA1|Q14CA1_HUMAN NRCAM protein OS=Homo sapiens OX=9606 GN=NRCAM PE=2 SV=1
MQLKIMPKKKRLSAGRVPLILFLCQMISALEVPLDPKLLEDLVQPPTITQQSPKDYIIDP
RENIVIQCEAKGKPPPSFSWTRNGTHFDIDKDPLVTMKPGTGTLIINIMSEGKAETYEGV
YQCTARNERGAAVSNNIVVRPSRSPLWTKEKLEPITLQSGQSLVLPCRPPIGLPPPIIFW
MDNSFQRLPQSERVSQGLNGDLYFSNVLPEDTREDYICYARFNHTQTIQQKQPISVKVIS
AKSSRERPPTFLTPEGNASNKEELRGNVLSLECIAEGLPTPIIYWAKEDGMLPKNRTVYK
NFEKTLQIIHVSEADSGNYQCIAKNALGAIHHTISVRVKAAPYWITAPQNLVLSPGEDGT
LICRANGNPKPRISWLTNGVPIEIAPDDPSRKIDGDTIIFSNVQERSSAVYQCNASNEYG
YLLANAFVNVLAEPPRILTPANTLYQVIANRPALLDCAFFGSPLPTIEWFKGAKGSALHE
DIYVLHENGTLEIPVAQKDSTGTYTCVARNKLGMAKNEVHLEIKDATWIVKQPEYAVVQR
GSMVSFECKVKHDHTLSLTVLWLKDNRELPSDERFTVDKDHLVVADVSDDDSGTYTCVAN
TTLDSVSASAVLSVVAPTPTPAPVYDVPNPPFDLELTDQLDKSVQLSWTPGDDNNSPITK
FIIEYEDAMHKPGLWHHQTEVSGTQTTAQLKLSPYVNYSFRVMAVNSIGKSLPSEASEQY
LTKASEPDKNPTAVEGLGSEPDNLVITWKPLNGFESNGPGLQYKVSWRQKDGDDEWTSVV
VANVSKYIVSGTPTFVPYLIKVQALNDMGFAPEPAVVMGHSGEDLPMVAPGNVRVNVVNS
TLAEVHWDPVPLKSIRGHLQGYRIYYWKTQSSSKRNRRHIEKKILTFQGSKTHGMLPGLE
PFSHYTLNVRVVNGKGEGPASPDRVFNTPEGVPSAPSSLKIVNPTLDSLTLEWDPPSHPN
GILTEYTLKYQPINSTHELGPLVDLKIPANKTRWTLKNLNFSTRYKFYFYAQTSAGSGSQ
ITEEAVTTVDEAMASRQVDIATQGWFIGLMCAVALLILILLIVCFIRRNKGGKYPVKEKE
DAHADPEIQPMKEDDGTFGEYSDAEDHKPLKKGSRTPSDRTVKKEDSDDSLVDYGEGVNG
QFNEDGSFIGQYSGKKEKEPAEGNESSEAPSPVNAMNSFV
>sp|Q15008|PSMD6_HUMAN 26S proteasome non-ATPase regulatory subunit 6 OS=Homo sapiens OX=9606 GN=PSMD6 PE=1 SV=1
MPLENLEEEGLPKNPDLRIAQLRFLLSLPEHRGDAAVRDELMAAVRDNNMAPYYEALCKS
LDWQIDVDLLNKMKKANEDELKRLDEELEDAEKNLGESEIRDAMMAKAEYLCRIGDKEGA
LTAFRKTYDKTVALGHRLDIVFYLLRIGLFYMDNDLITRNTEKAKSLIEEGGDWDRRNRL
KVYQGLYCVAIRDFKQAAELFLDTVSTFTSYELMDYKTFVTYTVYVSMIALERPDLREKV
IKGAEILEVLHSLPAVRQYLFSLYECRYSVFFQSLAVVEQEMKKDWLFAPHYRYYVREMR
IHAYSQLLESYRSLTLGYMAEAFGVGVEFIDQELSRFIAAGRLHCKIDKVNEIVETNRPD
SKNWQYQETIKKGDLLLNRVQKLSRVINM
>sp|Q15036|SNX17_HUMAN Sorting nexin-17 OS=Homo sapiens OX=9606 GN=SNX17 PE=1 SV=1
MHFSIPETESRSGDSGGSAYVAYNIHVNGVLHCRVRYSQLLGLHEQLRKEYGANVLPAFP
PKKLFSLTPAEVEQRREQLEKYMQAVRQDPLLGSSETFNSFLRRAQQETQQVPTEEVSLE
VLLSNGQKVLVNVLTSDQTEDVLEAVAAKLDLPDDLIGYFSLFLVREKEDGAFSFVRKLQ
EFELPYVSVTSLRSQEYKIVLRKSYWDSAYDDDVMENRVGLNLLYAQTVSDIERGWILVT
KEQHRQLKSLQEKVSKKEFLRLAQTLRHYGYLRFDACVADFPEKDCPVVVSAGNSELSLQ
LRLPGQQLREGSFRVTRMRCWRVTSSVPLPSGSTSSPGRGRGEVRLELAFEYLMSKDRLQ
WVTITSPQAIMMSICLQSMVDELMVKKSGGSIRKMLRRRVGGTLRRSDSQQAVKSPPLLE
SPDATRESMVKLSSKLSAVSLRGIGSPSTDASASDVHGNFAFEGIGDEDL
>sp|Q15102|PA1B3_HUMAN Platelet-activating factor acetylhydrolase IB subunit alpha1 OS=Homo sapiens OX=9606 GN=PAFAH1B3 PE=1 SV=1
MSGEENPASKPTPVQDVQGDGRWMSLHHRFVADSKDKEPEVVFIGDSLVQLMHQCEIWRE

LFSPLHALNFGIGGDGTQHVLWRLENGELEHIRPKIVVVWVGTNNHGHTAEQVTGGIKAI
VQLVNERQPQARVVVLGLLPRGQHPNPLREKNRQVNELVRAALAGHPRAHFLDADPGFVH
SDGTISHHDMYDYLHLSRLGYTPVCRALHSLLLRLLAQDQGQGAPLLEPAP
>sp|Q15120|PDK3_HUMAN [Pyruvate dehydrogenase (acetyl-transferring)] kinase isozyme 3, mitochondrial OS=Homo sapiens OX=9606 GN=PDK3 PE=1 SV=1
MRLFRWLLKQPVPKQIERYSRFSPSPLSIKQFLDFGRDNACEKTSYMFLRKELPVRLANT
MREVNLLPDNLLNRPSVGLVQSWYMQSFLELLEYENKSPEDPQVLDNFLQVLIKVRNRHN
DVVPTMAQGVIEYKEKFGFDPFISTNIQYFLDRFYTNRISFRMLINQHTLLFGGDTNPVH
PKHIGSIDPTCNVADVVKDAYETAKMLCEQYYLVAPELEVEEFNAKAPDKPIQVVYVPSH
LFHMLFELFKNSMRATVELYEDRKEGYPAVKTLVTLGKEDLSIKISDLGGGVPLRKIDRL
FNYMYSTAPRPSLEPTRAAPLAGFGYGLPISRLYARYFQGDLKLYSMEGVGTDAVIYLKA
LSSESFERLPVFNKSAWRHYKTTPEADDWSNPSSEPRDASKYKAKQ
>sp|Q15124|PGM5_HUMAN Phosphoglucomutase-like protein 5 OS=Homo sapiens OX=9606 GN=PGM5 PE=1 SV=2
MEGSPIPVLTVPTAPYEDQRPAGGGGLRRPTGLFEGQRNYLPNFIQSVLSSIDLRDRQGC
TMVVGSDGRYFSRTAIEIVVQMAAANGIGRLIIGQNGILSTPAVSCIIRKIKAAGGIILT
ASHCPGGPGGEFGVKFNVANGGPAPDVVSDKIYQISKTIEEYAICPDLRIDLSRLGRQEF
DLENKFKPFRVEIVDPVDIYLNLLRTIFDFHAIKGLLTGPSQLKIRIDAMHGVMGPYVRK
VLCDELGAPANSAINCVPLEDFGGQHPDPNLTYATTLLEAMKGGEYGFGAAFDADGDRYM
ILGQNGFFVSPSDSLAIIAANLSCIPYFRQMGVRGFGRSMPTSMALDRVAKSMKVPVYET
PAGWRFFSNLMDSGRCNLCGEESFGTGSDHLREKDGLWAVLVWLSIIAARKQSVEEIVRD
HWAKFGRHYYCRFDYEGLDPKTTYYIMRDLEALVTDKSFIGQQFAVGSHVYSVAKTDSFE
YVDPVDGTVTKKQGLRIIFSDASRLIFRLSSSSGVRATLRLYAESYERDPSGHDQEPQAV
LSPLIAIALKISQIHERTGRRGPTVIT
>sp|Q15149|PLEC_HUMAN Plectin OS=Homo sapiens OX=9606 GN=PLEC PE=1 SV=3
MVAGMLMPRDQLRAIYEVLFREGVMVAKKDRRPRSLHPHVPGVTNLQVMRAMASLRARGL
VRETFAWCHFYWYLTNEGIAHLRQYLHLPPEIVPASLQRVRRPVAMVMPARRTPHVQAVQ
GPLGSPPKRGPLPTEEQRVYRRKELEEVSPETPVVPATTQRTLARPGPEPAPATDERDRV
QKKTFTKWVNKHLIKAQRHISDLYEDLRDGHNLISLLEVLSGDSLPREKGRMRFHKLQNV
QIALDYLRHRQVKLVNIRNDDIADGNPKLTLGLIWTIILHFQISDIQVSGQSEDMTAKEK
LLLWSQRMVEGYQGLRCDNFTSSWRDGRLFNAIIHRHKPLLIDMNKVYRQTNLENLDQAF
SVAERDLGVTRLLDPEDVDVPQPDEKSIITYVSSLYDAMPRVPDVQDGVRANELQLRWQE
YRELVLLLLQWMRHHTAAFEERRFPSSFEEIEILWSQFLKFKEMELPAKEADKNRSKGIY
QSLEGAVQAGQLKVPPGYHPLDVEKEWGKLHVAILEREKQLRSEFERLECLQRIVTKLQM
EAGLCEEQLNQADALLQSDVRLLAAGKVPQRAGEVERDLDKADSMIRLLFNDVQTLKDGR
HPQGEQMYRRVYRLHERLVAIRTEYNLRLKAGVAAPATQVAQVTLQSVQRRPELEDSTLR
YLQDLLAWVEENQHRVDGAEWGVDLPSVEAQLGSHRGLHQSIEEFRAKIERARSDEGQLS
PATRGAYRDCLGRLDLQYAKLLNSSKARLRSLESLHSFVAAATKELMWLNEKEEEEVGFD
WSDRNTNMTAKKESYSALMRELELKEKKIKELQNAGDRLLREDHPARPTVESFQAALQTQ
WSWMLQLCCCIEAHLKENAAYFQFFSDVREAEGQLQKLQEALRRKYSCDRSATVTRLEDL
LQDAQDEKEQLNEYKGHLSGLAKRAKAVVQLKPRHPAHPMRGRLPLLAVCDYKQVEVTVH
KGDECQLVGPAQPSHWKVLSSSGSEAAVPSVCFLVPPPNQEAQEAVTRLEAQHQALVTLW
HQLHVDMKSLLAWQSLRRDVQLIRSWSLATFRTLKPEEQRQALHSLELHYQAFLRDSQDA
GGFGPEDRLMAEREYGSCSHHYQQLLQSLEQGAQEESRCQRCISELKDIRLQLEACETRT

VHRLRLPLDKEPARECAQRIAEQQKAQAEVEGLGKGVARLSAEAEKVLALPEPSPAAPTL
RSELELTLGKLEQVRSLSAIYLEKLKTISLVIRGTQGAEEVLRAHEEQLKEAQAVPATLP
ELEATKASLKKLRAQAEAQQPTFDALRDELRGAQEVGERLQQRHGERDVEVERWRERVAQ
LLERWQAVLAQTDVRQRELEQLGRQLRYYRESADPLGAWLQDARRRQEQIQAMPLADSQA
VREQLRQEQALLEEIERHGEKVEECQRFAKQYINAIKDYELQLVTYKAQLEPVASPAKKP
KVQSGSESVIQEYVDLRTHYSELTTLTSQYIKFISETLRRMEEEERLAEQQRAEERERLA
EVEAALEKQRQLAEAHAQAKAQAEREAKELQQRMQEEVVRREEAAVDAQQQKRSIQEELQ
QLRQSSEAEIQAKARQAEAAERSRLRIEEEIRVVRLQLEATERQRGGAEGELQALRARAE
EAEAQKRQAQEEAERLRRQVQDESQRKRQAEVELASRVKAEAEAAREKQRALQALEELRL
QAEEAERRLRQAEVERARQVQVALETAQRSAEAELQSKRASFAEKTAQLERSLQEEHVAV
AQLREEAERRAQQQAEAERAREEAERELERWQLKANEALRLRLQAEEVAQQKSLAQAEAE
KQKEEAEREARRRGKAEEQAVRQRELAEQELEKQRQLAEGTAQQRLAAEQELIRLRAETE
QGEQQRQLLEEELARLQREAAAATQKRQELEAELAKVRAEMEVLLASKARAEEESRSTSE
KSKQRLEAEAGRFRELAEEAARLRALAEEAKRQRQLAEEDAARQRAEAERVLAEKLAAIG
EATRLKTEAEIALKEKEAENERLRRLAEDEAFQRRRLEEQAAQHKADIEERLAQLRKASD
SELERQKGLVEDTLRQRRQVEEEILALKASFEKAAAGKAELELELGRIRSNAEDTLRSKE
QAELEAARQRQLAAEEERRRREAEERVQKSLAAEEEAARQRKAALEEVERLKAKVEEARR
LRERAEQESARQLQLAQEAAQKRLQAEEKAHAFAVQQKEQELQQTLQQEQSVLDQLRGEA
EAARRAAEEAEEARVQAEREAAQSRRQVEEAERLKQSAEEQAQARAQAQAAAEKLRKEAE
QEAARRAQAEQAALRQKQAADAEMEKHKKFAEQTLRQKAQVEQELTTLRLQLEETDHQKN
LLDEELQRLKAEATEAARQRSQVEEELFSVRVQMEELSKLKARIEAENRALILRDKDNTQ
RFLQEEAEKMKQVAEEAARLSVAAQEAARLRQLAEEDLAQQRALAEKMLKEKMQAVQEAT
RLKAEAELLQQQKELAQEQARRLQEDKEQMAQQLAEETQGFQRTLEAERQRQLEMSAEAE
RLKLRVAEMSRAQARAEEDAQRFRKQAEEIGEKLHRTELATQEKVTLVQTLEIQRQQSDH
DAERLREAIAELEREKEKLQQEAKLLQLKSEEMQTVQQEQLLQETQALQQSFLSEKDSLL
QRERFIEQEKAKLEQLFQDEVAKAQQLREEQQRQQQQMEQERQRLVASMEEARRRQHEAE
EGVRRKQEELQQLEQQRRQQEELLAEENQRLREQLQLLEEQHRAALAHSEEVTASQVAAT
KTLPNGRDALDGPAAEAEPEHSFDGLRRKVSAQRLQEAGILSAEELQRLAQGHTTVDELA
RREDVRHYLQGRSSIAGLLLKATNEKLSVYAALQRQLLSPGTALILLEAQAASGFLLDPV
RNRRLTVNEAVKEGVVGPELHHKLLSAERAVTGYKDPYTGQQISLFQAMQKGLIVREHGI
RLLEAQIATGGVIDPVHSHRVPVDVAYRRGYFDEEMNRVLADPSDDTKGFFDPNTHENLT
YLQLLERCVEDPETGLCLLPLTDKAAKGGELVYTDSEARDVFEKATVSAPFGKFQGKTVT
IWEIINSEYFTAEQRRDLLRQFRTGRITVEKIIKIIITVVEEQEQKGRLCFEGLRSLVPA
AELLESRVIDRELYQQLQRGERSVRDVAEVDTVRRALRGANVIAGVWLEEAGQKLSIYNA
LKKDLLPSDMAVALLEAQAGTGHIIDPATSARLTVDEAVRAGLVGPEFHEKLLSAEKAVT
GYRDPYTGQSVSLFQALKKGLIPREQGLRLLDAQLSTGGIVDPSKSHRVPLDVACARGCL
DEETSRALSAPRADAKAYSDPSTGEPATYGELQQRCRPDQLTGLSLLPLSEKAARARQEE
LYSELQARETFEKTPVEVPVGGFKGRTVTVWELISSEYFTAEQRQELLRQFRTGKVTVEK
VIKILITIVEEVETLRQERLSFSGLRAPVPASELLASGVLSRAQFEQLKDGKTTVKDLSE
LGSVRTLLQGSGCLAGIYLEDTKEKVSIYEAMRRGLLRATTAALLLEAQAATGFLVDPVR
NQRLYVHEAVKAGVVGPELHEQLLSAEKAVTGYRDPYSGSTISLFQAMQKGLVLRQHGIR
LLEAQIATGGIIDPVHSHRVPVDVAYQRGYFSEEMNRVLADPSDDTKGFFDPNTHENLTY
RQLLERCVEDPETGLRLLPLKGAEKAEVVETTQVYTEEETRRAFEETQIDIPGGGSHGGS
TMSLWEVMQSDLIPEEQRAQLMADFQAGRVTKERMIIIIIEIIEKTEIIRQQGLASYDYV

RRRLTAEDLFEARIISLETYNLLREGTRSLREALEAESAWCYLYGTGSVAGVYLPGSRQT
LSIYQALKKGLLSAEVARLLLEAQAATGFLLDPVKGERLTVDEAVRKGLVGPELHDRLLS
AERAVTGYRDPYTEQTISLFQAMKKELIPTEEALRLLDAQLATGGIVDPRLGFHLPLEVA
YQRGYLNKDTHDQLSEPSEVRSYVDPSTDERLSYTQLLRRCRRDDGTGQLLLPLSDARKL
TFRGLRKQITMEELVRSQVMDEATALQLREGLTSIEEVTKNLQKFLEGTSCIAGVFVDAT
KERLSVYQAMKKGIIRPGTAFELLEAQAATGYVIDPIKGLKLTVEEAVRMGIVGPEFKDK
LLSAERAVTGYKDPYSGKLISLFQAMKKGLILKDHGIRLLEAQIATGGIIDPEESHRLPV
EVAYKRGLFDEEMNEILTDPSDDTKGFFDPNTEENLTYLQLMERCITDPQTGLCLLPLKE
KKRERKTSSKSSVRKRRVVIVDPETGKEMSVYEAYRKGLIDHQTYLELSEQECEWEEITI
SSSDGVVKSMIIDRRSGRQYDIDDAIAKNLIDRSALDQYRAGTLSITEFADMLSGNAGGF
RSRSSSVGSSSSYPISPAVSRTQLASWSDPTEETGPVAGILDTETLEKVSITEAMHRNLV
DNITGQRLLEAQACTGGIIDPSTGERFPVTDAVNKGLVDKIMVDRINLAQKAFCGFEDPR
TKTKMSAAQALKKGWLYYEAGQRFLEVQYLTGGLIEPDTPGRVPLDEALQRGTVDARTAQ
KLRDVGAYSKYLTCPKTKLKISYKDALDRSMVEEGTGLRLLEAAAQSTKGYYSPYSVSGS
GSTAGSRTGSRTGSRAGSRRGSFDATGSGFSMTFSSSSYSSSGYGRRYASGSSASLGGPE
SAVA
>sp|Q15223|NECT1_HUMAN Nectin-1 OS=Homo sapiens OX=9606 GN=NECTIN1 PE=1 SV=3
MARMGLAGAAGRWWGLALGLTAFFLPGVHSQVVQVNDSMYGFIGTDVVLHCSFANPLPSV
KITQVTWQKSTNGSKQNVAIYNPSMGVSVLAPYRERVEFLRPSFTDGTIRLSRLELEDEG
VYICEFATFPTGNRESQLNLTVMAKPTNWIEGTQAVLRAKKGQDDKVLVATCTSANGKPP
SVVSWETRLKGEAEYQEIRNPNGTVTVISRYRLVPSREAHQQSLACIVNYHMDRFKESLT
LNVQYEPEVTIEGFDGNWYLQRMDVKLTCKADANPPATEYHWTTLNGSLPKGVEAQNRTL
FFKGPINYSLAGTYICEATNPIGTRSGQVEVNITEFPYTPSPPEHGRRAGPVPTAIIGGV
AGSILLVLIVVGGIVVALRRRRHTFKGDYSTKKHVYGNGYSKAGIPQHHPPMAQNLQYPD
DSDDEKKAGPLGGSSYEEEEEEEEGGGGGERKVGGPHPKYDEDAKRPYFTVDEAEARQDG
YGDRTLGYQYDPEQLDLAENMVSQNDGSFISKKEWYV
>sp|Q15257|PTPA_HUMAN Serine/threonine-protein phosphatase 2A activator OS=Homo sapiens OX=9606 GN=PTPA PE=1 SV=3
MAEGERQPPPDSSEEAPPATQNFIIPKKEIHTVPDMGKWKRSQAYADYIGFILTLNEGVK
GKKLTFEYRVSEMWNEVHEEKEQAAKQSVSCDECIPLPRAGHCAPSEAIEKLVALLNTLD
RWIDETPPVDQPSRFGNKAYRTWYAKLDEEAENLVATVVPTHLAAAVPEVAVYLKESVGN
STRIDYGTGHEAAFAAFLCCLCKIGVLRVDDQIAIVFKVFNRYLEVMRKLQKTYRMEPAG
SQGVWGLDDFQFLPFIWGSSQLIDHPYLEPRHFVDEKAVNENHKDYMFLECILFITEMKT
GPFAEHSNQLWNISAVPSWSKVNQGLIRMYKAECLEKFPVIQHFKFGSLLPIHPVTSG
>sp|Q15286|RAB35_HUMAN Ras-related protein Rab-35 OS=Homo sapiens OX=9606 GN=RAB35 PE=1 SV=1
MARDYDHLFKLLIIGDSGVGKSSLLLRFADNTFSGSYITTIGVDFKIRTVEINGEKVKLQ
IWDTAGQERFRTITSTYYRGTHGVIVVYDVTSAESFVNVKRWLHEINQNCDDVCRILVGN
KNDDPERKVVETEDAYKFAGQMGIQLFETSAKENVNVEEMFNCITELVLRAKKDNLAKQQ
QQQQNDVVKLTKNSKRKKRCC
>sp|Q15435|PP1R7_HUMAN Protein phosphatase 1 regulatory subunit 7 OS=Homo sapiens OX=9606 GN=PPP1R7 PE=1 SV=1
MAAERGAGQQQSQEMMEVDRRVESEESGDEEGKKHSSGIVADLSEQSLKDGEERGEEDPE
EEHELPVDMETINLDRDAEDVDLNHYRIGKIEGFEVLKKVKTLCLRQNLIKCIENLEELQ

SLRELDLYDNQIKKIENLEALTELEILDISFNLLRNIEGVDKLTRLKKLFLVNNKISKIE
NLSNLHQLQMLELGSNRIRAIENIDTLTNLESLFLGKNKITKLQNLDALTNLTVLSMQSN
RLTKIEGLQNLVNLRELYLSHNGIEVIEGLENNNKLTMLDIASNRIKKIENISHLTELQE
FWMNDNLLESWSDLDELKGARSLETVYLERNPLQKDPQYRRKVMLALPSVRQIDATFVRF
>sp|Q15582|BGH3_HUMAN Transforming growth factor-beta-induced protein ig-h3 OS=Homo sapiens OX=9606 GN=TGFBI PE=1 SV=1
MALFVRLLALALALALGPAATLAGPAKSPYQLVLQHSRLRGRQHGPNVCAVQKVIGTNRK
YFTNCKQWYQRKICGKSTVISYECCPGYEKVPGEKGCPAALPLSNLYETLGVVGSTTTQL
YTDRTEKLRPEMEGPGSFTIFAPSNEAWASLPAEVLDSLVSNVNIELLNALRYHMVGRRV
LTDELKHGMTLTSMYQNSNIQIHHYPNGIVTVNCARLLKADHHATNGVVHLIDKVISTIT
NNIQQIIEIEDTFETLRAAVAASGLNTMLEGNGQYTLLAPTNEAFEKIPSETLNRILGDP
EALRDLLNNHILKSAMCAEAIVAGLSVETLEGTTLEVGCSGDMLTINGKAIISNKDILAT
NGVIHYIDELLIPDSAKTLFELAAESDVSTAIDLFRQAGLGNHLSGSERLTLLAPLNSVF
KDGTPPIDAHTRNLLRNHIIKDQLASKYLYHGQTLETLGGKKLRVFVYRNSLCIENSCIA
AHDKRGRYGTLFTMDRVLTPPMGTVMDVLKGDNRFSMLVAAIQSAGLTETLNREGVYTVF
APTNEAFRALPPRERSRLLGDAKELANILKYHIGDEILVSGGIGALVRLKSLQGDKLEVS
LKNNVVSVNKEPVAEPDIMATNGVVHVITNVLQPPANRPQERGDELADSALEIFKQASAF
SRASQRSVRLAPVYQKLLERMKH
>sp|Q15628|TRADD_HUMAN Tumor necrosis factor receptor type 1-associated DEATH domain protein OS=Homo sapiens OX=9606 GN=TRADD PE=1 SV=2
MAAGQNGHEEWVGSAYLFVESSLDKVVLSDAYAHPQQKVAVYRALQAALAESGGSPDVLQ
MLKIHRSDPQLIVQLRFCGRQPCGRFLRAYREGALRAALQRSLAAALAQHSVPLQLELRA
GAERLDALLADEERCLSCILAQQPDRLRDEELAELEDALRNLKCGSGARGGDGEVASAPL
QPPVPSLSEVKPPPPPPPAQTFLFQGQPVVNRPLSLKDQQTFARSVGLKWRKVGRSLQRG
CRALRDPALDSLAYEYEREGLYEQAFQLLRRFVQAEGRRATLQRLVEALEENELTSLAED
LLGLTDPNGGLA
>sp|Q15631|TSN_HUMAN Translin OS=Homo sapiens OX=9606 GN=TSN PE=1 SV=1
MSVSEIFVELQGFLAAEQDIREEIRKVVQSLEQTAREILTLLQGVHQGAGFQDIPKRCLK
AREHFGTVKTHLTSLKTKFPAEQYYRFHEHWRFVLQRLVFLAAFVVYLETETLVTREAVT
EILGIEPDREKGFHLDVEDYLSGVLILASELSRLSVNSVTAGDYSRPLHISTFINELDSG
FRLLNLKNDSLRKRYDGLKYDVKKVEEVVYDLSIRGFNKETAAACVEK
>sp|Q15907|RB11B_HUMAN Ras-related protein Rab-11B OS=Homo sapiens OX=9606 GN=RAB11B PE=1 SV=4
MGTRDDEYDYLFKVVLIGDSGVGKSNLLSRFTRNEFNLESKSTIGVEFATRSIQVDGKTI
KAQIWDTAGQERYRAITSAYYRGAVGALLVYDIAKHLTYENVERWLKELRDHADSNIVIM
LVGNKSDLRHLRAVPTDEARAFAEKNNLSFIETSALDSTNVEEAFKNILTEIYRIVSQKQ
IADRAAHDESPGNNVVDISVPPTTDGQKPNKLQCCQNL
>sp|Q16270|IBP7_HUMAN Insulin-like growth factor-binding protein 7 OS=Homo sapiens OX=9606 GN=IGFBP7 PE=1 SV=1
MERPSLRALLLGAAGLLLLLLPLSSSSSSDTCGPCEPASCPPLPPLGCLLGETRDACGCC
PMCARGEGEPCGGGGAGRGYCAPGMECVKSRKRRKGKAGAAAGGPGVSGVCVCKSRYPVC
GSDGTTYPSGCQLRAASQRAESRGEKAITQVSKGTCEQGPSIVTPPKDIWNVTGAQVYLS
CEVIGIPTPVLIWNKVKRGHYGVQRTELLPGDRDNLAIQTRGGPEKHEVTGWVLVSPLSK
EDAGEYECHASNSQGQASASAKITVVDALHEIPVKKGEGAEL

>sp|Q16401|PSMD5_HUMAN 26S proteasome non-ATPase regulatory subunit 5 OS=Homo sapiens OX=9606 GN=PSMD5 PE=1 SV=3
MAAQALALLREVARLEAPLEELRALHSVLQAVPLNELRQQAAELRLGPLFSLLNENHREK
TTLCVSILERLLQAMEPVHVARNLRVDLQRGLIHPDDSVKILTLSQIGRIVENSDAVTEI
LNNAELLKQIVYCIGGENLSVAKAAIKSLSRISLTQAGLEALFESNLLDDLKSVMKTNDI
VRYRVYELIIEISSVSPESLNYCTTSGLVTQLLRELTGEDVLVRATCIEMVTSLAYTHHG
RQYLAQEGVIDQISNIIVGADSDPFSSFYLPGFVKFFGNLAVMDSPQQICERYPIFVEKV
FEMIESQDPTMIGVAVDTVGILGSNVEGKQVLQKTGTRFERLLMRIGHQSKNAPVELKIR
CLDAISSLLYLPPEQQTDDLLRMTESWFSSLSRDPLELFRGISSQPFPELHCAALKVFTA
IANQPWAQKLMFNSPGFVEYVVDRSVEHDKASKDAKYELVKALANSKTIAEIFGNPNYLR
LRTYLSEGPYYVKPVSTTAVEGAE
>sp|Q16531|DDB1_HUMAN DNA damage-binding protein 1 OS=Homo sapiens OX=9606 GN=DDB1 PE=1 SV=1
MSYNYVVTAQKPTAVNGCVTGHFTSAEDLNLLIAKNTRLEIYVVTAEGLRPVKEVGMYGK
IAVMELFRPKGESKDLLFILTAKYNACILEYKQSGESIDIITRAHGNVQDRIGRPSETGI
IGIIDPECRMIGLRLYDGLFKVIPLDRDNKELKAFNIRLEELHVIDVKFLYGCQAPTICF
VYQDPQGRHVKTYEVSLREKEFNKGPWKQENVEAEASMVIAVPEPFGGAIIIGQESITYH
NGDKYLAIAPPIIKQSTIVCHNRVDPNGSRYLLGDMEGRLFMLLLEKEEQMDGTVTLKDL
RVELLGETSIAECLTYLDNGVVFVGSRLGDSQLVKLNVDSNEQGSYVVAMETFTNLGPIV
DMCVVDLERQGQGQLVTCSGAFKEGSLRIIRNGIGIHEHASIDLPGIKGLWPLRSDPNRE
TDDTLVLSFVGQTRVLMLNGEEVEETELMGFVDDQQTFFCGNVAHQQLIQITSASVRLVS
QEPKALVSEWKEPQAKNISVASCNSSQVVVAVGRALYYLQIHPQELRQISHTEMEHEVAC
LDITPLGDSNGLSPLCAIGLWTDISARILKLPSFELLHKEMLGGEIIPRSILMTTFESSH
YLLCALGDGALFYFGLNIETGLLSDRKKVTLGTQPTVLRTFRSLSTTNVFACSDRPTVIY
SSNHKLVFSNVNLKEVNYMCPLNSDGYPDSLALANNSTLTIGTIDEIQKLHIRTVPLYES
PRKICYQEVSQCFGVLSSRIEVQDTSGGTTALRPSASTQALSSSVSSSKLFSSSTAPHET
SFGEEVEVHNLLIIDQHTFEVLHAHQFLQNEYALSLVSCKLGKDPNTYFIVGTAMVYPEE
AEPKQGRIVVFQYSDGKLQTVAEKEVKGAVYSMVEFNGKLLASINSTVRLYEWTTEKELR
TECNHYNNIMALYLKTKGDFILVGDLMRSVLLLAYKPMEGNFEEIARDFNPNWMSAVEIL
DDDNFLGAENAFNLFVCQKDSAATTDEERQHLQEVGLFHLGEFVNVFCHGSLVMQNLGET
STPTQGSVLFGTVNGMIGLVTSLSESWYNLLLDMQNRLNKVIKSVGKIEHSFWRSFHTER
KTEPATGFIDGDLIESFLDISRPKMQEVVANLQYDDGSGMKREATADDLIKVVEELTRIH
>sp|Q16555|DPYL2_HUMAN Dihydropyrimidinase-related protein 2 OS=Homo sapiens OX=9606 GN=DPYSL2 PE=1 SV=1
MSYQGKKNIPRITSDRLLIKGGKIVNDDQSFYADIYMEDGLIKQIGENLIVPGGVKTIEA
HSRMVIPGGIDVHTRFQMPDQGMTSADDFFQGTKAALAGGTTMIIDHVVPEPGTSLLAAF
DQWREWADSKSCCDYSLHVDISEWHKGIQEEMEALVKDHGVNSFLVYMAFKDRFQLTDCQ
IYEVLSVIRDIGAIAQVHAENGDIIAEEQQRILDLGITGPEGHVLSRPEEVEAEAVNRAI
TIANQTNCPLYITKVMSKSSAEVIAQARKKGTVVYGEPITASLGTDGSHYWSKNWAKAAA
FVTSPPLSPDPTTPDFLNSLLSCGDLQVTGSAHCTFNTAQKAVGKDNFTLIPEGTNGTEE
RMSVIWDKAVVTGKMDENQFVAVTSTNAAKVFNLYPRKGRIAVGSDADLVIWDPDSVKTI
SAKTHNSSLEYNIFEGMECRGSPLVVISQGKIVLEDGTLHVTEGSGRYIPRKPFPDFVYK
RIKARSRLAELRGVPRGLYDGPVCEVSVTPKTVTPASSAKTSPAKQQAPPVRNLHQSGFS
LSGAQIDDNIPRRTTQRIVAPPGGRANITSLG

>sp|Q16563|SYPL1_HUMAN Synaptophysin-like protein 1 OS=Homo sapiens OX=9606 GN=SYPL1 PE=1 SV=1
MAPNIYLVRQRISRLGQRMSGFQINLNPLKEPLGFIKVLEWIASIFAFATCGGFKGQTEI
QVNCPPAVTENKTVTATFGYPFRLNEASFQPPPGVNICDVNWKDYVLIGDYSSSAQFYVT
FAVFVFLYCIAALLLYVGYTSLYLDSRKLPMIDFVVTLVATFLWLVSTSAWAKALTDIKI
ATGHNIIDELPPCKKKAVLCYFGSVTSMGSLNVSVIFGFLNMILWGGNAWFVYKETSLHS
PSNTSAPHSQGGIPPPTGI
>sp|Q16643|DREB_HUMAN Drebrin OS=Homo sapiens OX=9606 GN=DBN1 PE=1 SV=4
MAGVSFSGHRLELLAAYEEVIREESAADWALYTYEDGSDDLKLAASGEGGLQELSGHFEN
QKVMYGFCSVKDSQAALPKYVLINWVGEDVPDARKCACASHVAKVAEFFQGVDVIVNASS
VEDIDAGAIGQRLSNGLARLSSPVLHRLRLREDENAEPVGTTYQKTDAAVEMKRINREQF
WEQAKKEEELRKEEERKKALDERLRFEQERMEQERQEQEERERRYREREQQIEEHRRKQQ
TLEAEEAKRRLKEQSIFGDHRDEEEETHMKKSESEVEEAAAIIAQRPDNPREFFKQQERV
ASASAGSCDVPSPFNHRPGSHLDSHRRMAPTPIPTRSPSDSSTASTPVAEQIERALDEVT
SSQPPPLPPPPPPAQETQEPSPILDSEETRAAAPQAWAGPMEEPPQAQAPPRGPGSPAED
LMFMESAEQAVLAAPVEPATADATEIHDAADTIETDTATADTTVANNVPPAATSLIDLWP
GNGEGASTLQGEPRAPTPPSGTEVTLAEVPLLDEVAPEPLLPAGEGCATLLNFDELPEPP
ATFCDPEEVEGESLAAPQTPTLPSALEELEQEQEPEPHLLTNGETTQKEGTQASEGYFSQ
SQEEEFAQSEELCAKAPPPVFYNKPPEIDITCWDADPVPEEEEGFEGGD
>sp|Q16773|KAT1_HUMAN Kynurenine--oxoglutarate transaminase 1 OS=Homo sapiens OX=9606 GN=KYAT1 PE=1 SV=1
MAKQLQARRLDGIDYNPWVEFVKLASEHDVVNLGQGFPDFPPPDFAVEAFQHAVSGDFML
NQYTKTFGYPPLTKILASFFGELLGQEIDPLRNVLVTVGGYGALFTAFQALVDEGDEVII
IEPFFDCYEPMTMMAGGRPVFVSLKPGPIQNGELGSSSNWQLDPMELAGKFTSRTKALVL
NTPNNPLGKVFSREELELVASLCQQHDVVCITDEVYQWMVYDGHQHISIASLPGMWERTL
TIGSAGKTFSATGWKVGWVLGPDHIMKHLRTVHQNSVFHCPTQSQAAVAESFEREQLLFR
QPSSYFVQFPQAMQRCRDHMIRSLQSVGLKPIIPQGSYFLITDISDFKRKMPDLPGAVDE
PYDRRFVKWMIKNKGLVAIPVSIFYSVPHQKHFDHYIRFCFVKDEATLQAMDEKLRKWKV
EL
>sp|Q16775|GLO2_HUMAN Hydroxyacylglutathione hydrolase, mitochondrial OS=Homo sapiens OX=9606 GN=HAGH PE=1 SV=2
MVVGRGLLGRRSLAALGAACARRGLGPALLGVFCHTDLRKNLTVDEGTMKVEVLPALTDN
YMYLVIDDETKEAAIVDPVQPQKVVDAARKHGVKLTTVLTTHHHWDHAGGNEKLVKLESG
LKVYGGDDRIGALTHKITHLSTLQVGSLNVKCLATPCHTSGHICYFVSKPGGSEPPAVFT
GDTLFVAGCGKFYEGTADEMCKALLEVLGRLPPDTRVYCGHEYTINNLKFARHVEPGNAA
IREKLAWAKEKYSIGEPTVPSTLAEEFTYNPFMRVREKTVQQHAGETDPVTTMRAVRREK
DQFKMPRD
>sp|Q16787|LAMA3_HUMAN Laminin subunit alpha-3 OS=Homo sapiens OX=9606 GN=LAMA3 PE=1 SV=3
MAAAARPRGRALGPVLPPTPLLLLVLRVLPACGATARDPGAAAGLSLHPTYFNLAEAARI
WATATCGERGPGEGRPQPELYCKLVGGPTAPGSGHTIQGQFCDYCNSEDPRKAHPVTNAI
DGSERWWQSPPLSSGTQYNRVNLTLDLGQLFHVAYILIKFANSPRPDLWVLERSVDFGST
YSPWQYFAHSKVDCLKEFGREANMAVTRDDDVLCVTEYSRIVPLENGEVVVSLINGRPGA
KNFTFSHTLREFTKATNIRLRFLRTNTLLGHLISKAQRDPTVTRRYYYSIKDISIGGQCV

CNGHAEVCNINNPEKLFRCECQHHTCGETCDRCCTGYNQRRWRPAAWEQSHECEACNCHG
HASNCYYDPDVERQQASLNTQGIYAGGGVCINCQHNTAGVNCEQCAKGYYRPYGVPVDAP
DGCIPCSCDPEHADGCEQGSGRCHCKPNFHGDNCEKCAIGYYNFPFCLRIPIFPVSTPSS
EDPVAGDIKGCDCNLEGVLPEICDAHGRCLCRPGVEGPRCDTCRSGFYSFPICQACWCSA
LGSYQMPCSSVTGQCECRPGVTGQRCDRCLSGAYDFPHCQGSSSACDPAGTINSNLGYCQ
CKLHVEGPTCSRCKLLYWNLDKENPSGCSECKCHKAGTVSGTGECRQGDGDCHCKSHVGG
DSCDTCEDGYFALEKSNYFGCQGCQCDIGGALSSMCSGPSGVCQCREHVVGKVCQRPENN
YYFPDLHHMKYEIEDGSTPNGRDLRFGFDPLAFPEFSWRGYAQMTSVQNDVRITLNVGKS
SGSLFRVILRYVNPGTEAVSGHITIYPSWGAAQSKEIIFLPSKEPAFVTVPGNGFADPFS
ITPGIWVACIKAEGVLLDYLVLLPRDYYEASVLQLPVTEPCAYAGPPQENCLLYQHLPVT
RFPCTLACEARHFLLDGEPRPVAVRQPTPAHPVMVDLSGREVELHLRLRIPQVGHYVVVV
EYSTEAAQLFVVDVNVKSSGSVLAGQVNIYSCNYSVLCRSAVIDHMSRIAMYELLADADI
QLKGHMARFLLHQVCIIPIEEFSAEYVRPQVHCIASYGRFVNQSATCVSLAHETPPTALI
LDVLSGRPFPHLPQQSSPSVDVLPGVTLKAPQNQVTLRGRVPHLGRYVFVIHFYQAAHPT
FPAQVSVDGGWPRAGSFHASFCPHVLGCRDQVIAEGQIEFDISEPEVAATVKVPEGKSLV
LVRVLVVPAENYDYQILHKKSMDKSLEFITNCGKNSFYLDPQTASRFCKNSARSLVAFYH
KGALPCECHPTGATGPHCSPEGGQCPCQPNVIGRQCTRCATGHYGFPRCKPCSCGRRLCE
EMTGQCRCPPRTVRPQCEVCETHSFSFHPMAGCEGCNCSRRGTIEAAMPECDRDSGQCRC
KPRITGRQCDRCASGFYRFPECVPCNCNRDGTEPGVCDPGTGACLCKENVEGTECNVCRE
GSFHLDPANLKGCTSCFCFGVNNQCHSSHKRRTKFVDMLGWHLETADRVDIPVSFNPGSN
SMVADLQELPATIHSASWVAPTSYLGDKVSSYGGYLTYQAKSFGLPGDMVLLEKKPDVQL
TGQHMSIIYEETNTPRPDRLHHGRVHVVEGNFRHASSRAPVSREELMTVLSRLADVRIQG
LYFTETQRLTLSEVGLEEASDTGSGRIALAVEICACPPAYAGDSCQGCSPGYYRDHKGLY
TGRCVPCNCNGHSNQCQDGSGICVNCQHNTAGEHCERCQEGYYGNAVHGSCRACPCPHTN
SFATGCVVNGGDVRCSCKAGYTGTQCERCAPGYFGNPQKFGGSCQPCSCNSNGQLGSCHP
LTGDCINQEPKDSSPAEECDDCDSCVMTLLNDLATMGEQLRLVKSQLQGLSASAGLLEQM
RHMETQAKDLRNQLLNYRSAISNHGSKIEGLERELTDLNQEFETLQEKAQVNSRKAQTLN
NNVNRATQSAKELDVKIKNVIRNVHILLKQISGTDGEGNNVPSGDFSREWAEAQRMMREL
RNRNFGKHLREAEADKRESQLLLNRIRTWQKTHQGENNGLANSIRDSLNEYEAKLSDLRA
RLQEAAAQAKQANGLNQENERALGAIQRQVKEINSLQSDFTKYLTTADSSLLQTNIALQL
MEKSQKEYEKLAASLNEARQELSDKVRELSRSAGKTSLVEEAEKHARSLQELAKQLEEIK
RNASGDELVRCAVDAATAYENILNAIKAAEDAANRAASASESALQTVIKEDLPRKAKTLS
SNSDKLLNEAKMTQKKLKQEVSPALNNLQQTLNIVTVQKEVIDTNLTTLRDGLHGIQRGD
IDAMISSAKSMVRKANDITDEVLDGLNPIQTDVERIKDTYGRTQNEDFKKALTDADNSVN
KLTNKLPDLWRKIESINQQLLPLGNISDNMDRIRELIQQARDAASKVAVPMRFNGKSGVE
VRLPNDLEDLKGYTSLSLFLQRPNSRENGGTENMFVMYLGNKDASRDYIGMAVVDGQLTC
VYNLGDREAELQVDQILTKSETKEAVMDRVKFQRIYQFARLNYTKGATSSKPETPGVYDM
DGRNSNTLLNLDPENVVFYVGGYPPDFKLPSRLSFPPYKGCIELDDLNENVLSLYNFKKT
FNLNTTEVEPCRRRKEESDKNYFEGTGYARVPTQPHAPIPTFGQTIQTTVDRGLLFFAEN
GDRFISLNIEDGKLMVRYKLNSELPKERGVGDAINNGRDHSIQIKIGKLQKRMWINVDVQ
NTIIDGEVFDFSTYYLGGIPIAIRERFNISTPAFRGCMKNLKKTSGVVRLNDTVGVTKKC
SEDWKLVRSASFSRGGQLSFTDLGLPPTDHLQASFGFQTFQPSGILLDHQTWTRNLQVTL
EDGYIELSTSDSGGPIFKSPQTYMDGLLHYVSVISDNSGLRLLIDDQLLRNSKRLKHISS
SRQSLRLGGSNFEGCISNVFVQRLSLSPEVLDLTSNSLKRDVSLGGCSLNKPPFLMLLKG

STRFNKTKTFRINQLLQDTPVASPRSVKVWQDACSPLPKTQANHGALQFGDIPTSHLLFK
LPQELLKPRSQFAVDMQTTSSRGLVFHTGTKNSFMALYLSKGRLVFALGTDGKKLRIKSK
EKCNDGKWHTVVFGHDGEKGRLVVDGLRAREGSLPGNSTISIRAPVYLGSPPSGKPKSLP
TNSFVGCLKNFQLDSKPLYTPSSSFGVSSCLGGPLEKGIYFSEEGGHVVLAHSVLLGPEF
KLVFSIRPRSLTGILIHIGSQPGKHLCVYLEAGKVTASMDSGAGGTSTSVTPKQSLCDGQ
WHSVAVTIKQHILHLELDTDSSYTAGQIPFPPASTQEPLHLGGAPANLTTLRIPVWKSFF
GCLRNIHVNHIPVPVTEALEVQGPVSLNGCPDQ
>sp|Q16851|UGPA_HUMAN UTP--glucose-1-phosphate uridylyltransferase OS=Homo sapiens OX=9606 GN=UGP2 PE=1 SV=5
MSRFVQDLSKAMSQDGASQFQEVIRQELELSVKKELEKILTTASSHEFEHTKKDLDGFRK
LFHRFLQEKGPSVDWGKIQRPPEDSIQPYEKIKARGLPDNISSVLNKLVVVKLNGGLGTS
MGCKGPKSLIGVRNENTFLDLTVQQIEHLNKTYNTDVPLVLMNSFNTDEDTKKILQKYNH
CRVKIYTFNQSRYPRINKESLLPVAKDVSYSGENTEAWYPPGHGDIYASFYNSGLLDTFI
GEGKEYIFVSNIDNLGATVDLYILNHLMNPPNGKRCEFVMEVTNKTRADVKGGTLTQYEG
KLRLVEIAQVPKAHVDEFKSVSKFKIFNTNNLWISLAAVKRLQEQNAIDMEIIVNAKTLD
GGLNVIQLETAVGAAIKSFENSLGINVPRSRFLPVKTTSDLLLVMSNLYSLNAGSLTMSE
KREFPTVPLVKLGSSFTKVQDYLRRFESIPDMLELDHLTVSGDVTFGKNVSLKGTVIIIA
NHGDRIDIPPGAVLENKIVSGNLRILDH
>sp|Q16891|MIC60_HUMAN MICOS complex subunit MIC60 OS=Homo sapiens OX=9606 GN=IMMT PE=1 SV=1
MLRACQLSGVTAAAQSCLCGKFVLRPLRPCRRYSTSGSSGLTTGKIAGAGLLFVGGGIGG
TILYAKWDSHFRESVEKTIPYSDKLFEMVLGPAAYNVPLPKKSIQSGPLKISSVSEVMKE
SKQPASQLQKQKGDTPASATAPTEAAQIISAAGDTLSVPAPAVQPEESLKTDHPEIGEGK
PTPALSEEASSSSIRERPPEEVAARLAQQEKQEQVKIESLAKSLEDALRQTASVTLQAIA
AQNAAVQAVNAHSNILKAAMDNSEIAGEKKSAQWRTVEGALKERRKAVDEAADALLKAKE
ELEKMKSVIENAKKKEVAGAKPHITAAEGKLHNMIVDLDNVVKKVQAAQSEAKVVSQYHE
LVVQARDDFKRELDSITPEVLPGWKGMSVSDLADKLSTDDLNSLIAHAHRRIDQLNRELA
EQKATEKQHITLALEKQKLEEKRAFDSAVAKALEHHRSEIQAEQDRKIEEVRDAMENEMR
TQLRRQAAAHTDHLRDVLRVQEQELKSEFEQNLSEKLSEQELQFRRLSQEQVDNFTLDIN
TAYARLRGIEQAVQSHAVAEEEARKAHQLWLSVEALKYSMKTSSAETPTIPLGSAVEAIK
ANCSDNEFTQALTAAIPPESLTRGVYSEETLRARFYAVQKLARRVAMIDETRNSLYQYFL
SYLQSLLLFPPQQLKPPPELCPEDINTFKLLSYASYCIEHGDLELAAKFVNQLKGESRRV
AQDWLKEARMTLETKQIVEILTAYASAVGIGTTQVQPE
>sp|Q17RB0|RTL8B_HUMAN Retrotransposon Gag-like protein 8B OS=Homo sapiens OX=9606 GN=RTL8B PE=1 SV=1
MEGRVQLMKALLARPLRPAARRWRNPIPFPETFDGDTDRLPEFIVQTSSYMFVDENTFSN
DALKVTFLITRLTGPALQWVIPYIKKESPLLSDYRGFLAEMKRVFGWEEDEDF
>sp|Q27J81|INF2_HUMAN Inverted formin-2 OS=Homo sapiens OX=9606 GN=INF2 PE=1 SV=2
MSVKEGAQRKWAALKEKLGPQDSDPTEANLESADPELCIRLLQMPSVVNYSGLRKRLEGS
DGGWMVQFLEQSGLDLLLEALARLSGRGVARISDALLQLTCVSCVRAVMNSRQGIEYILS
NQGYVRQLSQALDTSNVMVKKQVFELLAALCIYSPEGHVLTLDALDHYKTVCSQQYRFSI
VMNELSGSDNVPYVVTLLSVINAVILGPEDLRARTQLRNEFIGLQLLDVLARLRDLEDAD
LLIQLEAFEEAKAEDEEELLRVSGGVDMSSHQEVFASLFHKVSCSPVSAQLLSVLQGLLH
LEPTLRSSQLLWEALESLVNRAVLLASDAQECTLEEVVERLLSVKGRPRPSPLVKAHKSV

QANLDQSQRGSSPQNTTTPKPSVEGQQPAAAAACEPVDHAQSESILKVSQPRALEQQAST
PPPPPPPPLLPGSSAEPPPPPPPPPLPSVGAKALPTAPPPPPLPGLGAMAPPAPPLPPPL
PGSCEFLPPPPPPLPGLGCPPPPPPLLPGMGWGPPPPPPPLLPCTCSPPVAGGMEEVIVA
QVDHGLGSAWVPSHRRVNPPTLRMKKLNWQKLPSNVAREHNSMWASLSSPDAEAVEPDFS
SIERLFSFPAAKPKEPTMVAPRARKEPKEITFLDAKKSLNLNIFLKQFKCSNEEVAAMIR
AGDTTKFDVEVLKQLLKLLPEKHEIENLRAFTEERAKLASADHFYLLLLAIPCYQLRIEC
MLLCEGAAAVLDMVRPKAQLVLAACESLLTSRQLPIFCQLILRIGNFLNYGSHTGDADGF
KISTLLKLTETKSQQNRVTLLHHVLEEAEKSHPDLLQLPRDLEQPSQAAGINLEIIRSEA
SSNLKKLLETERKVSASVAEVQEQYTERLQASISAFRALDELFEAIEQKQRELADYLCED
AQQLSLEDTFSTMKAFRDLFLRALKENKDRKEQAAKAERRKQQLAEEEARRPRGEDGKPV
RKGPGKQEEVCVIDALLADIRKGFQLRKTARGRGDTDGGSKAASMDPPRATEPVATSNPA
GDPVGSTRCPASEPGLDATTASESRGWDLVDAVTPGPQPTLEQLEEGGPRPLERRSSWYV
DASDVLTTEDPQCPQPLEGAWPVTLGDAQALKPLKFSSNQPPAAGSSRQDAKDPTSLLGV
LQAEADSTSEGLEDAVHSRGARPPAAGPGGDEDEDEEDTAPESALDTSLDKSFSEDAVTD
SSGSGTLPRARGRASKGTGKRRKKRPSRSQEEVPPDSDDNKTKKLCVIQ
>sp|Q2TAA2|IAH1_HUMAN Isoamyl acetate-hydrolyzing esterase 1 homolog OS=Homo sapiens OX=9606 GN=IAH1 PE=1 SV=1
MALCEAAGCGSALLWPRLLLFGDSITQFSFQQGGWGASLADRLVRKCDVLNRGFSGYNTR
WAKIILPRLIRKGNSLDIPVAVTIFFGANDSALKDENPKQHIPLEEYAANLKSMVQYLKS
VDIPENRVILITPTPLCETAWEEQCIIQGCKLNRLNSVVGEYANACLQVAQDCGTDVLDL
WTLMQDSQDFSSYLSDGLHLSPKGNEFLFSHLWPLIEKKVSSLPLLLPYWRDVAEAKPEL
SLLGDGDH
>tr|Q32Q12|Q32Q12_HUMAN Nucleoside diphosphate kinase OS=Homo sapiens OX=9606 GN=NME1-NME2 PE=1 SV=1
MVLLSTLGIVFQGEGPPISSCDTGTMANCERTFIAIKPDGVQRGLVGEIIKRFEQKGFRL
VGLKFMQASEDLLKEHYVDLKDRPFFAGLVKYMHSGPVVAMVWEGLNVVKTGRVMLGETN
PADSKPGTIRGDFCIQVGRTMANLERTFIAIKPDGVQRGLVGEIIKRFEQKGFRLVAMKF
LRASEEHLKQHYIDLKDRPFFPGLVKYMNSGPVVAMVWEGLNVVKTGRVMLGETNPADSK
PGTIRGDFCIQVGRNIIHGSDSVKSAEKEISLWFKPEELVDYKSCAHDWVYE
>sp|Q3LXA3|TKFC_HUMAN Triokinase/FMN cyclase OS=Homo sapiens OX=9606 GN=TKFC PE=1 SV=2
MTSKKLVNSVAGCADDALAGLVACNPNLQLLQGHRVALRSDLDSLKGRVALLSGGGSGHE
PAHAGFIGKGMLTGVIAGAVFTSPAVGSILAAIRAVAQAGTVGTLLIVKNYTGDRLNFGL
AREQARAEGIPVEMVVIGDDSAFTVLKKAGRRGLCGTVLIHKVAGALAEAGVGLEEIAKQ
VNVVAKAMGTLGVSLSSCSVPGSKPTFELSADEVELGLGIHGEAGVRRIKMATADEIVKL
MLDHMTNTTNASHVPVQPGSSVVMMVNNLGGLSFLELGIIADATVRSLEGRGVKIARALV
GTFMSALEMPGISLTLLLVDEPLLKLIDAETTAAAWPNVAAVSITGRKRSRVAPAEPQEA
PDSTAAGGSASKRMALVLERVCSTLLGLEEHLNALDRAAGDGDCGTTHSRAARAIQEWLK
EGPPPASPAQLLSKLSVLLLEKMGGSSGALYGLFLTAAAQPLKAKTSLPAWSAAMDAGLE
AMQKYGKAAPGDRTMLDSLWAAGQELQAWKSPGADLLQVLTKAVKSAEAAAEATKNMEAG
AGRASYISSARLEQPDPGAVAAAAILRAILEVLQS
>sp|Q3SXM5|HSDL1_HUMAN Inactive hydroxysteroid dehydrogenase-like protein 1 OS=Homo sapiens OX=9606 GN=HSDL1 PE=1 SV=3
MAAVDSFYLLYREIARSCNCYMEALALVGAWYTARKSITVICDFYSLIRLHFIPRLGSRA

DLIKQYGRWAVVSGATDGIGKAYAEELASRGLNIILISRNEEKLQVVAKDIADTYKVETD
IIVADFSSGREIYLPIREALKDKDVGILVNNVGVFYPYPQYFTQLSEDKLWDIINVNIAA
ASLMVHVVLPGMVERKKGAIVTISSGSCCKPTPQLAAFSASKAYLDHFSRALQYEYASKG
IFVQSLIPFYVATSMTAPSNFLHRCSWLVPSPKVYAHHAVSTLGISKRTTGYWSHSIQFL
FAQYMPEWLWVWGANILNRSLRKEALSCTA
>sp|Q3SY77|UD3A2_HUMAN UDP-glucuronosyltransferase 3A2 OS=Homo sapiens OX=9606 GN=UGT3A2 PE=2 SV=1
MAGQRVLLLVGFLLPGVLLSEAAKILTISTVGGSHYLLMDRVSQILQDHGHNVTMLNHKR
GPFMPDFKKEEKSYQVISWLAPEDHQREFKKSFDFFLEETLGGRGKFENLLNVLEYLALQ
CSHFLNRKDIMDSLKNENFDMVIVETFDYCPFLIAEKLGKPFVAILSTSFGSLEFGLPIP
LSYVPVFRSLLTDHMDFWGRVKNFLMFFSFCRRQQHMQSTFDNTIKEHFTEGSRPVLSHL
LLKAELWFINSDFAFDFARPLLPNTVYVGGLMEKPIKPVPQDLENFIAKFGDSGFVLVTL
GSMVNTCQNPEIFKEMNNAFAHLPQGVIWKCQCSHWPKDVHLAANVKIVDWLPQSDLLAH
PSIRLFVTHGGQNSIMEAIQHGVPMVGIPLFGDQPENMVRVEAKKFGVSIQLKKLKAETL
ALKMKQIMEDKRYKSAAVAASVILRSHPLSPTQRLVGWIDHVLQTGGATHLKPYVFQQPW
HEQYLLDVFVFLLGLTLGTLWLCGKLLGMAVWWLRGARKVKET
>sp|Q3ZCM7|TBB8_HUMAN Tubulin beta-8 chain OS=Homo sapiens OX=9606 GN=TUBB8 PE=1 SV=2
MREIVLTQIGQCGNQIGAKFWEVISDEHAIDSAGTYHGDSHLQLERINVYYNEASGGRYV
PRAVLVDLEPGTMDSVRSGPFGQVFRPDNFIFGQCGAGNNWAKGHYTEGAELMESVMDVV
RKEAESCDCLQGFQLTHSLGGGTGSGMGTLLLSKIREEYPDRIINTFSILPSPKVSDTVV
EPYNATLSVHQLIENADETFCIDNEALYDICSKTLKLPTPTYGDLNHLVSATMSGVTTCL
RFPGQLNADLRKLAVNMVPFPRLHFFMPGFAPLTSRGSQQYRALTVAELTQQMFDAKNMM
AACDPRHGRYLTAAAIFRGRMPMREVDEQMFNIQDKNSSYFADWLPNNVKTAVCDIPPRG
LKMSATFIGNNTAIQELFKRVSEQFTAMFRRKAFLHWYTGEGMDEMEFTEAESNMNDLVS
EYQQYQDATAEEEEDEEYAEEEVA
>sp|Q53FA7|QORX_HUMAN Quinone oxidoreductase PIG3 OS=Homo sapiens OX=9606 GN=TP53I3 PE=1 SV=2
MLAVHFDKPGGPENLYVKEVAKPSPGEGEVLLKVAASALNRADLMQRQGQYDPPPGASNI
LGLEASGHVAELGPGCQGHWKIGDTAMALLPGGGQAQYVTVPEGLLMPIPEGLTLTQAAA
IPEAWLTAFQLLHLVGNVQAGDYVLIHAGLSGVGTAAIQLTRMAGAIPLVTAGSQKKLQM
AEKLGAAAGFNYKKEDFSEATLKFTKGAGVNLILDCIGGSYWEKNVNCLALDGRWVLYGL
MGGGDINGPLFSKLLFKRGSLITSLLRSRDNKYKQMLVNAFTEQILPHFSTEGPQRLLPV
LDRIYPVTEIQEAHKYMEANKNIGKIVLELPQ
>tr|Q53FI7|Q53FI7_HUMAN Four and a half LIM domains 1 variant (Fragment) OS=Homo sapiens OX=9606 PE=2 SV=1
MAEKFDCHYCRDPLQGKKYVQKDGHHCCLKCFDKFCANTCVECRKPIGADSKEVHYKNRF
WHDTCFRCAKCLHPLANETFVAKDNKILCNKCTTREDSPKCKGCFKAIVAGDQNVEYKGT
VWHKDCFTCSNCKQVIGTGSFFPKGEDFYCVTCHETKFAEHCVKCNKAITSGGITYQDQP
WHADCFVCVTCSKKLAGQRFTAVEDQYYCVDCYKNFVAKKCAGCKNPITGFGKGSSVVAY
EGQSWHDYCFHCKKCSVNLANKRFVFHQEQVYCPDCAKKL
>tr|Q53G25|Q53G25_HUMAN Ribosomal protein S5 variant (Fragment) OS=Homo sapiens OX=9606 PE=2 SV=1
MTEWETAAPAVAETPDIKLFGKWSTDDVQINDISLQDYIAVKEKYAKYLPHSAGRYAAKR

FRKAQCPIVERLTNSMMMHGRNNGKKLMTVRIVKHAFEIIHLLTGENPLQVLVNAIINSG
PREDSTRIGRAGTVRRQAVDVSPLRRVNQAIWLLCTGAREAAFRNIKTIAECLADELINA
AKGSSNFYAIKKRDELERVAKSNR
>sp|Q53GQ0|DHB12_HUMAN Very-long-chain 3-oxoacyl-CoA reductase OS=Homo sapiens OX=9606 GN=HSD17B12 PE=1 SV=2
MESALPAAGFLYWVGAGTVAYLALRISYSLFTALRVWGVGNEAGVGPGLGEWAVVTGSTD
GIGKSYAEELAKHGMKVVLISRSKDKLDQVSSEIKEKFKVETRTIAVDFASEDIYDKIKT
GLAGLEIGILVNNVGMSYEYPEYFLDVPDLDNVIKKMININILSVCKMTQLVLPGMVERS
KGAILNISSGSGMLPVPLLTIYSATKTFVDFFSQCLHEEYRSKGVFVQSVLPYFVATKLA
KIRKPTLDKPSPETFVKSAIKTVGLQSRTNGYLIHALMGSIISNLPSWIYLKIVMNMNKS
TRAHYLKKTKKN
>sp|Q562R1|ACTBL_HUMAN Beta-actin-like protein 2 OS=Homo sapiens OX=9606 GN=ACTBL2 PE=1 SV=2
MTDNELSALVVDNGSGMCKAGFGGDDAPRAVFPSMIGRPRHQGVMVGMGQKDCYVGDEAQ
SKRGVLTLKYPIEHGVVTNWDDMEKIWYHTFYNELRVAPDEHPILLTEAPLNPKINREKM
TQIMFEAFNTPAMYVAIQAVLSLYASGRTTGIVMDSGDGVTHIVPIYEGYALPHAILRLD
LAGRDLTDYLMKILTERGYNFTTTAEREIVRDVKEKLCYVALDFEQEMVRAAASSSPERS
YELPDGQVITIGNERFRCPEAIFQPSFLGIESSGIHETTFNSIMKCDVDIRKDLYANTVL
SGGSTMYPGIADRMQKEIITLAPSTMKIKIIAPPERKYSVWIGGSILASLSTFQQMWISK
QEYDEAGPPIVHRKCF
>tr|Q562Z4|Q562Z4_HUMAN Actin-like protein (Fragment) OS=Homo sapiens OX=9606 GN=ACT PE=4 SV=1
KIWRHTFYNELRVAPEEHPVLLTEAPLNPKANREKMTQIMFETFNTPAMYVAIQAVLSLY
ASGRTTGIVMDSGDGVTHTVPIYKGYALPHAILRLDLAGRDLT
>tr|Q567P1|Q567P1_HUMAN IGL@ protein OS=Homo sapiens OX=9606 GN=IGL@ PE=1 SV=1
MASFPLLLTLLTHCAGSWAQSVLTQPPSASGTPGQRVPISCSGSSSNIGSNTVNWYQQFP
GTAPKLLIYSNNQRPSGVPDRFSGSKSGTSASLAISGLQSEDDAVYHCATWDDNLNSWVF
GGGTKLTVLSQPKAAPSVTLFPPSSEELQANKATLVCLISDFYPGAVTVAWKADSSPVKA
GVETTTPSKQSNNKYAASSYLSLTPEQWKSHKSYSCQVTHEGSTVEKTVAPTECS
>sp|Q58EX2|SDK2_HUMAN Protein sidekick-2 OS=Homo sapiens OX=9606 GN=SDK2 PE=1 SV=3
MWGLLIWTLLALHQIRAARAQDDVSPYFKTEPVRTQVHLEGNRLVLTCMAEGSWPLEFKW
LHNNRELTKFSLEYRYMITSLDRTHAGFYRCIVRNRMGALLQRQTEVQVAYMGSFEEGEK
HQSVSHGEAAVIRAPRIASFPQPQVTWFRDGRKIPPSSRIAITLENTLVILSTVAPDAGR
YYVQAVNDKNGDNKTSQPITLTVENVGGPADPIAPTIIIPPKNTSVVAGTSEVTLECVAN
ARPLIKLHIIWKKDGVLLSGGISDHNRRLTIPNPTGSDAGYYECEAVLRSSSVPSVVRGA
YLSVLEPPQFVKEPERHITAEMEKVVDIPCQAKGVPPPSITWYKDAAVVEVEKLTRFRQR
NDGGLQISGLVPDDTGMFQCFARNAAGEVQTSTYLAVTSIAPNITRGPLDSTVIDGMSVV
LACETSGAPRPAITWQKGERILASGSVQLPRFTPLESGSLLISPTHISDAGTYTCLATNS
RGVDEASADLVVWARTRITKPPQDQSVIKGTQASMVCGVTHDPRVTIRYIWEKDGATLGT
ESHPRIRLDRNGSLHISQTWSGDIGTYTCRVISAGGNDSRSAHLRVRQLPHAPEHPVATL
STVERRAINLTWTKPFDGNSPLIRYILEMSENNAPWTVLLASVDPKATSVTVKGLVPARS
YQFRLCAVNDVGKGQFSKDTERVSLPEEPPTAPPQNVIASGRTNQSIMIQWQPPPESHQN
GILKGYIIRYCLAGLPVGYQFKNITDADVNNLLLEDLIIWTNYEIEVAAYNSAGLGVYSS

KVTEWTLQGVPTVPPGNVHAEATNSTTIRFTWNAPSPQFINGINQGYKLIAWEPEQEEEV
TMVTARPNFQDSIHVGFVSGLKKFTEYFTSVLCFTTPGDGPRSTPQLVRTHEDVPGPVGH
LSFSEILDTSLKVSWQEPGEKNGILTGYRISWEEYNRTNTRVTHYLPNVTLEYRVTGLTA
LTTYTIEVAAMTSKGQGQVSASTISSGVPPELPGPPTNLGISNIGPRSVTLQFRPGYDGK
TSISRWLVEAQVGVVGEGEEWLLIHQLSNEPDARSMEVPDLNPFTCYSFRMRQVNIVGTS
PPSQPSRKIQTLQAPPDMAPANVSLRTASETSLWLRWMPLPEMEYNGNPESVGYKIKYSR
SDGHGKTLSHVVQDRVERDYTIEDLEEWTEYRVQVQAFNAIGSGPWSQTVVGRTRESVPS
SGPTNVSALATTSSSMLVRWSEVPEADRNGLVLGYKVMYKEKDSDTQPRFWLVEGNSSRS
AQLTGLGKYVLYEVQVLAFTRIGDGSPSHPPILERTLDDVPGPPMGILFPEVRTTSVRLI
WQPPAAPNGIILAYQITHRLNTTTANTATVEVLAPSARQYTATGLKPESVYLFRITAQTR
KGWGEAAEALVVTTEKRDRPQPPSRPMVQQEDVRARSVLLSWEPGSDGLSPVRYYTIQTR
ELPSGRWALHSASVSHNASSFIVDRLKPFTSYKFRVKATNDIGDSEFSEESESLTTLQAA
PDEAPTILSVTPHTTTSVLIRWQPPAEDKINGILLGFRIRYRELLYEGLRGFTLRGINNP
GATWAELTSMYSMRNLSRPSLTQYELDNLNKHRRYEIRMSVYNAVGEGPSSPPQEVFVGE
AVPTAAPRNVVVHGATATQLDVTWEPPPLDSQNGDIQGYKIYFWEAQRGNLTERVKTLFL
AENSVKLKNLTGYTAYMVSVAAFNAAGDGPRSTPTQGQTQQAAPSAPSSVKFSELTTTSV
NVSWEAPQFPNGILEGYRLVYEPCSPVDGVSKIVTVDVKGNSPLWLKVKDLAEGVTYRFR
IRAKTFTYGPEIEANVTTGPGEGAPGPPGVPIIVRYSSAIAIHWSSGDPGKGPITRYVIE
ARPSDEGLWDILIKDIPKEVSSYTFSMDILKPGVSYDFRVIAVNDYGFGTPSSPSQSVPA
QKANPFYEEWWFLVVIALVGLIFILLLVFVLIIRGQSKKYAKKTDSGNSAKSGALGHSEM
MSLDESSFPALELNNRRLSVKNSFCRKNGLYTRSPPRPSPGSLHYSDEDVTKYNDLIPAE
SSSLTEKPSEISDSQGSDSEYEVDSNHQKAHSFVNHYISDPTYYNSWRRQQKGISRAQAY
SYTESDSGEPDHTTVTNSTSTQQGSLFRPKASRTPTPQNPPNPPSQQSTLYRPPSSLAPG
SRAPIAGFSSFV
>tr|Q59FD5|Q59FD5_HUMAN Glycoprotein M6B isoform 1 variant (Fragment) OS=Homo sapiens OX=9606 PE=2 SV=1
RSPHPSRQSPAMGCFECCIKCLGGVPYASLVATILCFSGVALFCGCGHVALAGTVAILEQ
HFSTNASDHALLSEVIQLMQYVIYGIASFFFLYGIILLAEGFYTTSAVKELHGEFKTTAC
GRCISGMFVFLTYVLGVAWLGVFGFSAVPVFMFYNIWSTCEVIKSPQTNGTTGVEQICVD
IRQYGIIPWNAFPGKICGSALENICNTNEFYMSYHLFIVACAGAGATVIALIHFLMILSS
NWAYLKDASKMQAYQDIKAKEEQELQDIQSRSKEQLNSYT
>tr|Q59FV6|Q59FV6_HUMAN Actin-related protein 3 (Fragment) OS=Homo sapiens OX=9606 PE=2 SV=1
RVMKGVDDLDFFIGDEAIEKPTYATKWPIRHGIVEDWDLMERFMEQVIFKYLRAEPEDHY
FLLTEPPLNTPENREYTAEIMFESFNVPGLYIAVQAVLALAASWTSRQVGERTLTGTVIN
SGDGVTHVIPVAEGYVIGSCIKHIPIAGRDITYFIQQLLRDREVGIPPEQSLETAKAVKE
RYSYVCPDLVKEFNKYDTDGSKWIKQYTGINAISKKEFSIDVGYERFLGPEIFFHPEFAN
PDFTQPISEVVDEVIQNCPIDVRRPLYKNIVLSGGSTMFRDFGRRLQRDLKRTVDARLKL
SEELSGGRLKPKPIDVQVITHHMQRYAVWFGGSMLASTPEFYQVCHTKKDYEEIGPSICR
HNPVFGVMS
>tr|Q59GP4|Q59GP4_HUMAN IlvB (Bacterial acetolactate synthase)-like isoform 1 variant (Fragment) OS=Homo sapiens OX=9606 PE=2 SV=1
LYCSTCPWAQVDKASVRHGGENVAAVLRAHGVRFIFTLVGGHISPLLVACEKLGIRVVDT
RHEVTAVFAADAMARLSGTVGVAAVTAGPGLTNTVTAVKNAQMAQSPILLLGGAASTLLQ

NRGALQAVDQLSLFRPLCKFCVSVRRVRDIVPTLRAAMAAAQSGTPGRWRMVMLSSAGAE
GLGTCYGLSVLPVPAGPVFVELPVDVLYPYFMVQKEMVPAKPPKGLVGRVVSWYLENYLA
NLFAGAWEPQPEGPLPLDIPQASPQQVQRCVEILSRAKRPLMVLGSQALLTPTSADKLRA
AVETLGVPCFLGGMARGLLGRNHPLHIRENRSAALKKADVIVLAGTVCDFRLSYGRVLSH
SSKIIIVNRNREEMLLNSDIFWKPQEAVQGEPLNPPVHICAPPSPSPPGFCCSWLEPGTV
IEAQTLP
>tr|Q5CZ91|Q5CZ91_HUMAN Methionine aminopeptidase OS=Homo sapiens OX=9606
GN=DKFZp781C0419 PE=3 SV=1
MCTMHKDEKAKREVSSWTVEGDINTDPWAGYRYTGKLRPHYPLMPTRPVPSYIQRPDYAD
HPLGMPESEQALKGTSQIKLLSSEDIEGMRLVCRLAREVLDVAAGMIKPGVTTEEIDHAV
HLACIARNCYPSPLNYYNFPKSCCTSVNEVICHGIPDRRPLQEGDIVNVDITLYRNGYHG
DLNETFFVGEVDDGARKLVQTTYECLMQAIDAVKPGVRYRELGNIIQKHAQANGFSVVRS
YCGHGIHKLFHTAPNVPHYAKNKAVGVMKSGHVFTIEPMICEGGWQDETWPDGWTAVTRD
GKRSAQFEHTLLVTDTGCEILTRRLDSARPHFMSQF
>tr|Q5JWE9|Q5JWE9_HUMAN GNAS complex locus OS=Homo sapiens OX=9606 GN=GNAS
PE=1 SV=3
MGVRNCLYGNNMSGQRDIPPEIGEQPEQPPLEAPGAAAPGAGPSPAEEMETEPPHNEPIP
VENDGEACGPPEVSRPNFQVLNPAFREAGAHGSYSPPPEEAMPFEAEQPSLGGFWPTLEQ
PGFPSGVHAGLEAFGPALMEPGAFSGARPGLGGYSPPPEEAMPFEFDQPAQRGCSQLLLQ
VPDLAPGGPGAAGVPGAPPEEPQALRPAKAGSRGGYSPPPEETMPFELDGEGFGDDSPPP
GLSRVIAQVDGSSQFAAVAASSAVRLTPAANAPPLWVPGAIGSPSQEAVRPPSNFTGSSP
WMEISGPPFEIGSAPAGVDDTPVNMDSPPIALDGPPIKVSGAPDKRERAERPPVEEEAAE
MEGAADAAEGGKVPSPGYGSPAAGAASADTAARAAPAAPADPDSGATPEDPDSGTAPADP
DSGAFAADPDSGAAPAAPADPDSGAAPDAPADPDSGAAPDAPADPDAGAAPEAPAAPAAA
ETRAAHVAPAAPDAGAPTAPAASATRAAQVRRAASAAPASGARRKIHLRPPSPEIQAADP
PTPRPTRASAWRGKSESSRGRRVYYDEGVASSDDDSSGDESDDGTSGCLRWFQHRRNRRR
RKPQRNLLRNFLVQAFGGCFGRSESPQPKASRSLKVKKVPLAEKRRQMRKEALEKRAQKR
AEKKRSKLIDKQLQDEKMGYMCTHRLLLLGAGESGKSTIVKQMRILHVNGFNGDEKATKV
QDIKNNLKEAIETIVAAMSNLVPPVELANPENQFRVDYILSVMNVPDFDFPPEFYEHAKA
LWEDEGVRACYERSNEYQLIDCAQYFLDKIDVIKQADYVPSDQDLLRCRVLTSGIFETKF
QVDKVNFHMFDVGGQRDERRKWIQCFNDVTAIIFVVASSSYNMVIREDNQTNRLQEALNL
FKSIWNNRWLRTISVILFLNKQDLLAEKVLAGKSKIEDYFPEFARYTTPEDATPEPGEDP
RVTRAKYFIRDEFLRISTASGDGRHYCYPHFTCAVDTENIRRVFNDCRDIIQRMHLRQYE
LL
>sp|Q5SSJ5|HP1B3_HUMAN Heterochromatin protein 1-binding protein 3 OS=Homo sapiens
OX=9606 GN=HP1BP3 PE=1 SV=1
MATDTSQGELVHPKALPLIVGAQLIHADKLGEKVEDSTMPIRRTVNSTRETPPKSKLAEG
EEEKPEPDISSEESVSTVEEQENETPPATSSEAEQPKGEPENEEKEENKSSEETKKDEKD
QSKEKEKKVKKTIPSWATLSASQLARAQKQTPMASSPRPKMDAILTEAIKACFQKSGASV
VAIRKYIIHKYPSLELERRGYLLKQALKRELNRGVIKQVKGKGASGSFVVVQKSRKTPQK
SRNRKNRSSAVDPEPQVKLEDVLPLAFTRLCEPKEASYSLIRKYVSQYYPKLRVDIRPQL
LKNALQRAVERGQLEQITGKGASGTFQLKKSGEKPLLGGSLMEYAILSAIAAMNEPKTCS
TTALKKYVLENHPGTNSNYQMHLLKKTLQKCEKNGWMEQISGKGFSGTFQLCFPYYPSPG
VLFPKKEPDDSRDEDEDEDESSEEDSEDEEPPPKRRLQKKTPAKSPGKAASVKQRGSKPA

PKVSAAQRGKARPLPKKAPPKAKTPAKKTRPSSTVIKKPSGGSSKKPATSARKEVKLPGK
GKSTMKKSFRVKK
>sp|Q5T4S7|UBR4_HUMAN E3 ubiquitin-protein ligase UBR4 OS=Homo sapiens OX=9606
GN=UBR4 PE=1 SV=1
MATSGGEEAAAAAPAPGTPATGADTTPGWEVAVRPLLSASYSAFEMKELPQLVASVIESE
SEILHHEKQYEPFYSSFVALSTHYITTVCSLIPRNQLQSVAAACKVLIEFSLLRLENPDE
ACAVSQKHLILLIKGLCTGCSRLDRTEIITFTAMMKSAKLPQTVKTLSDVEDQKELASPV
SPELRQKEVQMNFLNQLTSVFNPRTVASQPISTQTLVEGENDEQSSTDQASAIKTKNVFI
AQNVASLQELGGSEKLLRVCLNLPYFLRYINRFQDAVLANSFFIMPATVADATAVRNGFH
SLVIDVTMALDTLSLPVLEPLNPSRLQDVTVLSLSCLYAGVSVATCMAILHVGSAQQVRT
GSTSSKEDDYESDAATIVQKCLEIYDMIGQAISSSRRAGGEHYQNFQLLGAWCLLNSLFL
ILNLSPTALADKGKEKDPLAALRVRDILSRTKEGVGSPKLGPGKGHQGFGVLSVILANHA
IKLLTSLFQDLQVEALHKGWETDGPPAALSIMAQSTSIQRIQRLIDSVPLMNLLLTLLST
SYRKACVLQRQRKGSMSSDASASTDSNTYYEDDFSSTEEDSSQDDDSEPILGQWFEETIS
PSKEKAAPPPPPPPPPLESSPRVKSPSKQAPGEKGNILASRKDPELFLGLASNILNFITS
SMLNSRNNFIRNYLSVSLSEHHMATLASIIKEVDKDGLKGSSDEEFAAALYHFNHSLVTS
DLQSPNLQNTLLQQLGVAPFSEGPWPLYIHPQSLSVLSRLLLIWQHKASAQGDPDVPECL
KVWDRFLSTMKQNALQGVVPSETEDLNVEHLQMLLLIFHNFTETGRRAILSLFVQIIQEL
SVNMDAQMRFVPLILARLLLIFDYLLHQYSKAPVYLFEQVQHNLLSPPFGWASGSQDSNS
RRATTPLYHGFKEVEENWSKHFSSDAVPHPRFYCVLSPEASEDDLNRLDSVACDVLFSKL
VKYDELYAALTALLAAGSQLDTVRRKENKNVTALEACALQYYFLILWRILGILPPSKTYI
NQLSMNSPEMSECDILHTLRWSSRLRISSYVNWIKDHLIKQGMKAEHASSLLELASTTKC
SSVKYDVEIVEEYFARQISSFCSIDCTTILQLHEIPSLQSIYTLDAAISKVQVSLDEHFS
KMAAETDPHKSSEITKNLLPATLQLIDTYASFTRAYLLQNFNEEGTTEKPSKEKLQGFAA
VLAIGSSRCKANTLGPTLVQNLPSSVQTVCESWNNINTNEFPNIGSWRNAFANDTIPSES
YISAVQAAHLGTLCSQSLPLAASLKHTLLSLVRLTGDLIVWSDEMNPPQVIRTLLPLLLE
SSTESVAEISSNSLERILGPAESDEFLARVYEKLITGCYNILANHADPNSGLDESILEEC
LQYLEKQLESSQARKAMEEFFSDSGELVQIMMATANENLSAKFCNRVLKFFTKLFQLTEK
SPNPSLLHLCGSLAQLACVEPVRLQAWLTRMTTSPPKDSDQLDVIQENRQLLQLLTTYIV
RENSQVGEGVCAVLLGTLTPMATEMLANGDGTGFPELMVVMATLASAGQGAGHLQLHNAA
VDWLSRCKKYLSQKNVVEKLNANVMHGKHVMILECTCHIMSYLADVTNALSQSNGQGPSH
LSVDGEERAIEVDSDWVEELAVEEEDSQAEDSDEDSLCNKLCTFTITQKEFMNQHWYHCH
TCKMVDGVGVCTVCAKVCHKDHEISYAKYGSFFCDCGAKEDGSCLALVKRTPSSGMSSTM
KESAFQSEPRISESLVRHASTSSPADKAKVTISDGKVADEEKPKKSSLCRTVEGCREELQ
NQANFSFAPLVLDMLNFLMDAIQTNFQQASAVGSSSRAQQALSELHTVEKAVEMTDQLMV
PTLGSQEGAFENVRMNYSGDQGQTIRQLISAHVLRRVAMCVLSSPHGRRQHLAVSHEKGK
ITVLQLSALLKQADSSKRKLTLTRLASAPVPFTVLSLTGNPCKEDYLAVCGLKDCHVLTF
SSSGSVSDHLVLHPQLATGNFIIKAVWLPGSQTELAIVTADFVKIYDLCVDALSPTFYFL
LPSSKIRDVTFLFNEEGKNIIVIMSSAGYIYTQLMEEASSAQQGPFYVTNVLEINHEDLK
DSNSQVAGGGVSVYYSHVLQMLFFSYCQGKSFAATISRTTLEVLQLFPINIKSSNGGSKT
SPALCQWSEVMNHPGLVCCVQQTTGVPLVVMVKPDTFLIQEIKTLPAKAKIQDMVAIRHT
ACNEQQRTTMILLCEDGSLRIYMANVENTSYWLQPSLQPSSVISIMKPVRKRKTATITTR
TSSQVTFPIDFFEHNQQLTDVEFGGNDLLQVYNAQQIKHRLNSTGMYVANTKPGGFTIEI
SNNNSTMVMTGMRIQIGTQAIERAPSYIEIFGRTMQLNLSRSRWFDFPFTREEALQADKK

LNLFIGASVDPAGVTMIDAVKIYGKTKEQFGWPDEPPEEFPSASVSNICPSNLNQSNGTG
DSDSAAPTTTSGTVLERLVVSSLEALESCFAVGPIIEKERNKNAAQELATLLLSLPAPAS
VQQQSKSLLASLHTSRSAYHSHKDQALLSKAVQCLNTSSKEGKDLDPEVFQRLVITARSI
AIMRPNNLVHFTESKLPQMETEGMDEGKEPQKQLEGDCCSFITQLVNHFWKLHASKPKNA
FLAPACLPGLTHIEATVNALVDIIHGYCTCELDCINTASKIYMQMLLCPDPAVSFSCKQA
LIRVLRPRNKRRHVTLPSSPRSNTPMGDKDDDDDDDADEKMQSSGIPNGGHIRQESQEQS
EVDHGDFEMVSESMVLETAENVNNGNPSPLEALLAGAEGFPPMLDIPPDADDETMVELAI
ALSLQQDQQGSSSSALGLQSLGLSGQAPSSSSLDAGTLSDTTASAPASDDEGSTAATDGS
TLRTSPADHGGSVGSESGGSAVDSVAGEHSVSGRSSAYGDATAEGHPAGPGSVSSSTGAI
STTTGHQEGDGSEGEGEGETEGDVHTSNRLHMVRLMLLERLLQTLPQLRNVGGVRAIPYM
QVILMLTTDLDGEDEKDKGALDNLLSQLIAELGMDKKDVSKKNERSALNEVHLVVMRLLS
VFMSRTKSGSKSSICESSSLISSATAAALLSSGAVDYCLHVLKSLLEYWKSQQNDEEPVA
TSQLLKPHTTSSPPDMSPFFLRQYVKGHAADVFEAYTQLLTEMVLRLPYQIKKITDTNSR
IPPPVFDHSWFYFLSEYLMIQQTPFVRRQVRKLLLFICGSKEKYRQLRDLHTLDSHVRGI
KKLLEEQGIFLRASVVTASSGSALQYDTLISLMEHLKACAEIAAQRTINWQKFCIKDDSV
LYFLLQVSFLVDEGVSPVLLQLLSCALCGSKVLAALAASSGSSSASSSSAPVAASSGQAT
TQSKSSTKKSKKEEKEKEKDGETSGSQEDQLCTALVNQLNKFADKETLIQFLRCFLLESN
SSSVRWQAHCLTLHIYRNSSKSQQELLLDLMWSIWPELPAYGRKAAQFVDLLGYFSLKTP
QTEKKLKEYSQKAVEILRTQNHILTNHPNSNIYNTLSGLVEFDGYYLESDPCLVCNNPEV
PFCYIKLSSIKVDTRYTTTQQVVKLIGSHTISKVTVKIGDLKRTKMVRTINLYYNNRTVQ
AIVELKNKPARWHKAKKVQLTPGQTEVKIDLPLPIVASNLMIEFADFYENYQASTETLQC
PRCSASVPANPGVCGNCGENVYQCHKCRSINYDEKDPFLCNACGFCKYARFDFMLYAKPC
CAVDPIENEEDRKKAVSNINTLLDKADRVYHQLMGHRPQLENLLCKVNEAAPEKPQDDSG
TAGGISSTSASVNRYILQLAQEYCGDCKNSFDELSKIIQKVFASRKELLEYDLQQREAAT
KSSRTSVQPTFTASQYRALSVLGCGHTSSTKCYGCASAVTEHCITLLRALATNPALRHIL
VSQGLIRELFDYNLRRGAAAMREEVRQLMCLLTRDNPEATQQMNDLIIGKVSTALKGHWA
NPDLASSLQYEMLLLTDSISKEDSCWELRLRCALSLFLMAVNIKTPVVVENITLMCLRIL
QKLIKPPAPTSKKNKDVPVEALTTVKPYCNEIHAQAQLWLKRDPKASYDAWKKCLPIRGI
DGNGKAPSKSELRHLYLTEKYVWRWKQFLSRRGKRTSPLDLKLGHNNWLRQVLFTPATQA
ARQAACTIVEALATIPSRKQQVLDLLTSYLDELSIAGECAAEYLALYQKLITSAHWKVYL
AARGVLPYVGNLITKEIARLLALEEATLSTDLQQGYALKSLTGLLSSFVEVESIKRHFKS
RLVGTVLNGYLCLRKLVVQRTKLIDETQDMLLEMLEDMTTGTESETKAFMAVCIETAKRY
NLDDYRTPVFIFERLCSIIYPEENEVTEFFVTLEKDPQQEDFLQGRMPGNPYSSNEPGIG
PLMRDIKNKICQDCDLVALLEDDSGMELLVNNKIISLDLPVAEVYKKVWCTTNEGEPMRI
VYRMRGLLGDATEEFIESLDSTTDEEEDEEEVYKMAGVMAQCGGLECMLNRLAGIRDFKQ
GRHLLTVLLKLFSYCVKVKVNRQQLVKLEMNTLNVMLGTLNLALVAEQESKDSGGAAVAE
QVLSIMEIILDESNAEPLSEDKGNLLLTGDKDQLVMLLDQINSTFVRSNPSVLQGLLRII
PYLSFGEVEKMQILVERFKPYCNFDKYDEDHSGDDKVFLDCFCKIAAGIKNNSNGHQLKD
LILQKGITQNALDYMKKHIPSAKNLDADIWKKFLSRPALPFILRLLRGLAIQHPGTQVLI
GTDSIPNLHKLEQVSSDEGIGTLAENLLEALREHPDVNKKIDAARRETRAEKKRMAMAMR
QKALGTLGMTTNEKGQVVTKTALLKQMEELIEEPGLTCCICREGYKFQPTKVLGIYTFTK
RVALEEMENKPRKQQGYSTVSHFNIVHYDCHLAAVRLARGREEWESAALQNANTKCNGLL
PVWGPHVPESAFATCLARHNTYLQECTGQREPTYQLNIHDIKLLFLRFAMEQSFSADTGG
GGRESNIHLIPYIIHTVLYVLNTTRATSREEKNLQGFLEQPKEKWVESAFEVDGPYYFTV

LALHILPPEQWRATRVEILRRLLVTSQARAVAPGGATRLTDKAVKDYSAYRSSLLFWALV
DLIYNMFKKVPTSNTEGGWSCSLAEYIRHNDMPIYEAADKALKTFQEEFMPVETFSEFLD
VAGLLSEITDPESFLKDLLNSVP
>sp|Q5T5P2|SKT_HUMAN Sickle tail protein homolog OS=Homo sapiens OX=9606
GN=KIAA1217 PE=1 SV=2
MEENESQKCEPCLPYSADRRQMQEQGKGNLHVTSPEDAECRRTKERLSNGNSRGSVSKSS
RNIPRRHTLGGPRSSKEILGMQTSEMDRKREAFLEHLKQKYPHHASAIMGHQERLRDQTR
SPKLSHSPQPPSLGDPVEHLSETSADSLEAMSEGDAPTPFSRGSRTRASLPVVRSTNQTK
ERSLGVLYLQYGDETKQLRMPNEITSADTIRALFVSAFPQQLTMKMLESPSVAIYIKDES
RNVYYELNDVRNIQDRSLLKVYNKDPAHAFNHTPKTMNGDMRMQRELVYARGDGPGAPRP
GSTAHPPHAIPNSPPSTPVPHSMPPSPSRIPYGGTRSMVVPGNATIPRDRISSLPVSRPI
SPSPSAILERRDVKPDEDMSGKNIAMYRNEGFYADPYLYHEGRMSIASSHGGHPLDVPDH
IIAYHRTAIRSASAYCNPSMQAEMHMEQSLYRQKSRKYPDSHLPTLGSKTPPASPHRVSD
LRMIDMHAHYNAHGPPHTMQPDRASPSRQAFKKEPGTLVYIEKPRSAAGLSSLVDLGPPL
MEKQVFAYSTATIPKDRETRERMQAMEKQIASLTGLVQSALFKGPITSYSKDASSEKMMK
TTANRNHTDSAGTPHVSGGKMLSALESTVPPSQPPPVGTSAIHMSLLEMRRSVAELRLQL
QQMRQLQLQNQELLRAMMKKAELEISGKVMETMKRLEDPVQRQRVLVEQERQKYLHEEEK
IVKKLCELEDFVEDLKKDSTAASRLVTLKDVEDGAFLLRQVGEAVATLKGEFPTLQNKMR
AILRIEVEAVRFLKEEPHKLDSLLKRVRSMTDVLTMLRRHVTDGLLKGTDAAQAAQYMAM
EKATAAEVLKSQEEAAHTSGQPFHSTGAPGDAKSEVVPLSGMMVRHAQSSPVVIQPSQHS
VALLNPAQNLPHVASSPAVPQEATSTLQMSQAPQSPQIPMNGSAMQSLFIEEIHSVSAKN
RAVSIEKAEKKWEEKRQNLDHYNGKEFEKLLEEAQANIMKSIPNLEMPPATGPLPRGDAP
VDKVELSEDSPNSEQDLEKLGGKSPPPPPPPPRRSYLPGSGLTTTRSGDVVYTGRKENIT
AKASSEDAGPSPQTRATKYPAEEPASAWTPSPPPVTTSSSKDEEEEEEEGDKIMAELQAF
QKCSFMDVNSNSHAEPSRADSHVKDTRSGATVPPKEKKNLEFFHEDVRKSDVEYENGPQM
EFQKVTTGAVRPSDPPKWERGMENSISDASRTSEYKTEIIMKENSISNMSLLRDSRNYSQ
ETVPKASFGFSGISPLEDEINKGSKISGLQYSIPDTENQTLNYGKTKEMEKQNTDKCHVS
SHTRLTESSVHDFKTEDQEVITTDFGQVVLRPKEARHANVNPNEDGESSSSSPTEENAAT
DNIAFMITETTVQVLSSGEVHDIVSQKGEDIQTVNIDARKEMTPRQEGTDNEDPVVCLDK
KPVIIIFDEPMDIRSAYKRLSTIFEECDEELERMMMEEKIEEEEEEENGDSVVQNNNTSQ
MSHKKVAPGNLRTGQQVETKSQPHSLATETRNPGGQEMNRTELNKFSHVDSPNSECKGED
ATDDQFESPKKKFKFKFPKKQLAALTQAIRTGTKTGKKTLQVVVYEEEEEDGTLKQHKEA
KRFEIARSQPEDTPENTVRRQEQPSIESTSPISRTDEIRKNTYRTLDSLEQTIKQLENTI
SEMSPKALVDTSCSSNRDSVASSSHIAQEASPRPLLVPDEGPTALEPPTSIPSASRKGSS
GAPQTSRMPVPMSAKNRPGTLDKPGKQSKLQDPRQYRQANGSAKKSGGDFKPTSPSLPAS
KIPALSPSSGKSSSLPSSSGDSSNLPNPPATKPSIASNPLSPQTGPPAHSASLIPSVSNG
SLKFQSLTHTGKGHHLSFSPQSQNGRAPPPLSFSSSPPSPASSVSLNQGAKGTRTIHTPS
LTSYKAQNGSSSKATPSTAKETS
>sp|Q5T749|KPRP_HUMAN Keratinocyte proline-rich protein OS=Homo sapiens OX=9606
GN=KPRP PE=1 SV=1
MCDQQQIQCRLPLQQCCVKGPSFCSSQSPFAQSQVVVQAPCEMQIVDCPASCPVQVCQVS
DQAPCQSQTTQVKCQSKTKQVKGQAQCQSKTTQVKGQAASQSQTSSVQSQAPCQSEVSYV
QCEASQPVQTCFVECAPVCYTETCYVECPVQNYVPCPAPQPVQMYRGRPAVCQPQGRFST
QCQYQGSYSSCGPQFQSRATCNNYTPQFQLRPSYSSCFPQYRSRTSFSPCVPQCQTQGSY

GSFTEQHRSRSTSRCLPPPRRLQLFPRSCSPPRRFEPCSSSYLPLRPSEGFPNYCTPPRR
SEPIYNSRCPRRPISSCSQRRGPKCRIEISSPCCPRQVPPQRCPVEIPPIRRRSQSCGPQ
PSWGASCPELRPHVEPRPLPSFCPPRRLDQCPESPLQRCPPPAPRPRLRPEPCISLEPRP
RPLPRQLSEPCLYPEPLPALRPTPRPVPLPRPGQCEIPEPRPCLQPCEHPEPCPRPEPIP
LPAPCPSPEPCRETWRSPSPCWGPNPVPYPGDLGCHESSPHRLDTEAPYCGPSSYNQGQE
SGAGCGPGDVFPERRGQDGHGDQGNAFAGVKGEAKSAYF
>sp|Q5T9C9|PI5L1_HUMAN Phosphatidylinositol 4-phosphate 5-kinase-like protein 1 OS=Homo sapiens OX=9606 GN=PIP5KL1 PE=2 SV=2
MAAPSPGPREVLAPSPEAGCRAVTSSRRGLLWRLRDKQSRLGLFEISPGHELHGMTCMMQ
AGLWAATQVSMDHPPTGPPSRDDFSEVLTQVHEGFELGTLAGPAFAWLRRSLGLAEEDYQ
AALGPGGPYLQFLSTSKSKASFFLSHDQRFFLKTQGRREVQALLAHLPRYVQHLQRHPHS
LLARLLGVHSLRVDRGKKTYFIVMQSVFYPAGRISERYDIKGCEVSRWVDPAPEGSPLVL
VLKDLNFQGKTINLGPQRSWFLRQMELDTTFLRELNVLDYSLLIAFQRLHEDERGPGSSL
IFRTARSVQGAQSPEESRAQNRRLLPDAPNALHILDGPEQRYFLGVVDLATVYGLRKRLE
HLWKTLRYPGRTFSTVSPARYARRLCQWVEAHTE
>sp|Q5TA50|CPTP_HUMAN Ceramide-1-phosphate transfer protein OS=Homo sapiens OX=9606 GN=CPTP PE=1 SV=1
MDDSETGFNLKVVLVSFKQCLDEKEEVLLDPYIASWKGLVRFLNSLGTIFSFISKDVVSK
LRIMERLRGGPQSEHYRSLQAMVAHELSNRLVDLERRSHHPESGCRTVLRLHRALHWLQL
FLEGLRTSPEDARTSALCADSYNASLAAYHPWVVRRAVTVAFCTLPTREVFLEAMNVGPP
EQAVQMLGEALPFIQRVYNVSQKLYAEHSLLDLP
>sp|Q5TAX3|TUT4_HUMAN Terminal uridylyltransferase 4 OS=Homo sapiens OX=9606 GN=TUT4 PE=1 SV=3
MEESKTLKSENHEPKKNVICEESKAVQVIGNQTLKARNDKSVKEIENSSPNRNSSKKNKQ
NDICIEKTEVKSCKVNAANLPGPKDLGLVLRDQSHCKAKKFPNSPVKAEKATISQAKSEK
ATSLQAKAEKSPKSPNSVKAEKASSYQMKSEKVPSSPAEAEKGPSLLLKDMRQKTELQQI
GKKIPSSFTSVDKVNIEAVGGEKCALQNSPRSQKQQTCTDNTGDSDDSASGIEDVSDDLS
KMKNDESNKENSSEMDYLENATVIDESALTPEQRLGLKQAEERLERDHIFRLEKRSPEYT
NCRYLCKLCLIHIENIQGAHKHIKEKRHKKNILEKQEESELRSLPPPSPAHLAALSVAVI
ELAKEHGITDDDLRVRQEIVEEMSKVITTFLPECSLRLYGSSLTRFALKSSDVNIDIKFP
PKMNHPDLLIKVLGILKKNVLYVDVESDFHAKVPVVVCRDRKSGLLCRVSAGNDMACLTT
DLLTALGKIEPVFIPLVLAFRYWAKLCYIDSQTDGGIPSYCFALMVMFFLQQRKPPLLPC
LLGSWIEGFDPKRMDDFQLKGIVEEKFVKWECNSSSATEKNSIAEENKAKADQPKDDTKK
TETDNQSNAMKEKHGKSPLALETPNRVSLGQLWLELLKFYTLDFALEEYVICVRIQDILT
RENKNWPKRRIAIEDPFSVKRNVARSLNSQLVYEYVVERFRAAYRYFACPQTKGGNKSTV
DFKKREKGKISNKKPVKSNNMATNGCILLGETTEKINAEREQPVQCDEMDCTSQRCIIDN
NNLLVNELDFADHGQDSSSLSTSKSSEIEPKLDKKQDDLAPSETCLKKELSQCNCIDLSK
SPDPDKSTGTDCRSNLETESSHQSVCTDTSATSCNCKATEDASDLNDDDNLPTQELYYVF
DKFILTSGKPPTIVCSICKKDGHSKNDCPEDFRKIDLKPLPPMTNRFREILDLVCKRCFD
ELSPPCSEQHNREQILIGLEKFIQKEYDEKARLCLFGSSKNGFGFRDSDLDICMTLEGHE
NAEKLNCKEIIENLAKILKRHPGLRNILPITTAKVPIVKFEHRRSGLEGDISLYNTLAQH
NTRMLATYAAIDPRVQYLGYTMKVFAKRCDIGDASRGSLSSYAYILMVLYFLQQRKPPVI
PVLQEIFDGKQIPQRMVDGWNAFFFDKTEELKKRLPSLGKNTESLGELWLGLLRFYTEEF
DFKEYVISIRQKKLLTTFEKQWTSKCIAIEDPFDLNHNLGAGVSRKMTNFIMKAFINGRK

LFGTPFYPLIGREAEYFFDSRVLTDGELAPNDRCCRVCGKIGHYMKDCPKRKSLLFRLKK
KDSEEEKEGNEEEKDSRDVLDPRDLHDTRDFRDPRDLRCFICGDAGHVRRECPEVKLARQ
RNSSVAAAQLVRNLVNAQQVAGSAQQQGDQSIRTRQSSECSESPSYSPQPQPFPQNSSQS
AAITQPSSQPGSQPKLGPPQQGAQPPHQVQMPLYNFPQSPPAQYSPMHNMGLLPMHPLQI
PAPSWPIHGPVIHSAPGSAPSNIGLNDPSIIFAQPAARPVAIPNTSHDGHWPRTVAPNSL
VNSGAVGNSEPGFRGLTPPIPWEHAPRPHFPLVPASWPYGLHQNFMHQGNARFQPNKPFY
TQDRCATRRCRERCPHPPRGNVSE
>sp|Q5TDP6|LGSN_HUMAN Lengsin OS=Homo sapiens OX=9606 GN=LGSN PE=1 SV=1
MNNEEDLLQEDSTRDEGNETEANSMNTLRRTRKKVTKPYVCSTEVGETDMSNSNDCMRDS
SQILTPPQLSSRMKHIRQAMAKNRLQFVRFEATDLHGVSRSKTIPAHFFQEKVSHGVCMP
RGYLEVIPNPKDNEMNNIRATCFNSDIVLMPELSTFRVLPWADRTARVICDTFTVTGEPL
LTSPRYIAKRQLSHLQASGFSLLSAFIYDFCIFGVPEILNSKIISFPALTFLNNHDQPFM
QELVDGLYHTGANVESFSSSTRPGQMEISFLPEFGISSADNAFTLRTGVKEVARKYNYIA
SFFIETGFCDSGILSHSLWDVDRKKNMFCSTSGTEQLTITGKKWLAGLLKHSAALSCLMA
PSVSCRKRYSKDRKDLKKSVPTTWGYNDNSCIFNIKCHGEKGTRIENKLGSATANPYLVL
AATVAAGLDGLHSSNEVLAGPDESTDFYQVEPSEIPLKLEDALVALEEDQCLRQALGETF
IRYFVAMKKYELENEEIAAERNKFLEYFI
>sp|Q5VU97|CAHD1_HUMAN VWFA and cache domain-containing protein 1 OS=Homo sapiens
OX=9606 GN=CACHD1 PE=2 SV=2
MARQPEEEETAVARARRPPLWLLCLVACWLLGAGAEADFSILDEAQVLASQMRRLAAEEL
GVVTMQRIFNSFVYTEKISNGESEVQQLAKKIREKFNRYLDVVNRNKQVVEASYTAHLTS
PLTAIQDCCTIPPSMMEFDGNFNTNVSRTISCDRLSTTVNSRAFNPGRDLNSVLADNLKS
NPGIKWQYFSSEEGIFTVFPAHKFRCKGSYEHRSRPIYVSTVRPQSKHIVVILDHGASVT
DTQLQIAKDAAQVILSAIDEHDKISVLTVADTVRTCSLDQCYKTFLSPATSETKRKMSTF
VSSVKSSDSPTQHAVGFQKAFQLIRSTNNNTKFQANTDMVIIYLSAGITSKDSSEEDKKA
TLQVINEENSFLNNSVMILTYALMNDGVTGLKELAFLRDLAEQNSGKYGVPDRMALPVIK
GSMMVLNQLSNLETTVGRFYTNLPNRMIDEAVFSLPFSDEMGDGLIMTVSKPCYFGNLLL
GIVGVDVNLAYILEDVTYYQDSLASYTFLIDDKGYTLMHPSLTRPYLLSEPPLHTDIIHY
ENIPKFELVRQNILSLPLGSQIIAVPVNSSLSWHINKLRETGKEAYNVSYAWKMVQDTSF
ILCIVVIQPEIPVKQLKNLNTVPSSKLLYHRLDLLGQPSACLHFKQLATLESPTIMLSAG
SFSSPYEHLSQPETKRMVEHYTAYLSDNTRLIANPGLKFSVRNEVMATSHVTDEWMTQME
MSSLNTYIVRRYIATPNGVLRIYPGSLMDKAFDPTRRQWYLHAVANPGLISLTGPYLDVG
GAGYVVTISHTIHSSSTQLSSGHTVAVMGIDFTLRYFYKVLMDLLPVCNQDGGNKIRCFI
MEDRGYLVAHPTLIDPKGHAPVEQQHITHKEPLVANDILNHPNFVKKNLCNSFSDRTVQR
FYKFNTSLAGDLTNLVHGSHCSKYRLARIPGTNAFVGIVNETCDSLAFCACSMVDRLCLN
CHRMEQNECECPCECPLEVNECTGNLTNAENRNPSCEVHQEPVTYTAIDPGLQDALHQCV
NSRCSQRLESGDCFGVLDCEWCMVDSDGKTHLDKPYCAPQKECFGGIVGAKSPYVDDMGA
IGDEVITLNMIKSAPVGPVAGGIMGCIMVLVLAVYAYRHQIHRRSHQHMSPLAAQEMSVR
MSNLENDRDERDDDSHEDRGIISNTRFIAAVIERHAHSPERRRRYWGRSGTESDHGYSTM
SPQEDSENPPCNNDPLSAGVDVGNHDEDLDLDTPPQTAALLSHKFHHYRSHHPTLHHSHH
LQAAVTVHTVDAEC
>sp|Q5VWZ2|LYPL1_HUMAN Lysophospholipase-like protein 1 OS=Homo sapiens OX=9606
GN=LYPLAL1 PE=1 SV=3
MAAASGSVLQRCIVSPAGRHSASLIFLHGSGDSGQGLRMWIKQVLNQDLTFQHIKIIYPT

APPRSYTPMKGGISNVWFDRFKITNDCPEHLESIDVMCQVLTDLIDEEVKSGIKKNRILI
GGFSMGGCMAIHLAYRNHQDVAGVFALSSFLNKASAVYQALQKSNGVLPELFQCHGTADE
LVLHSWAEETNSMLKSLGVTTKFHSFPNVYHELSKTELDILKLWILTKLPGEMEKQK
>sp|Q658P3|STEA3_HUMAN Metalloreductase STEAP3 OS=Homo sapiens OX=9606 GN=STEAP3 PE=1 SV=2
MPEEMDKPLISLHLVDSDSSLAKVPDEAPKVGILGSGDFARSLATRLVGSGFKVVVGSRN
PKRTARLFPSAAQVTFQEEAVSSPEVIFVAVFREHYSSLCSLSDQLAGKILVDVSNPTEQ
EHLQHRESNAEYLASLFPTCTVVKAFNVISAWTLQAGPRDGNRQVPICGDQPEAKRAVSE
MALAMGFMPVDMGSLASAWEVEAMPLRLLPAWKVPTLLALGLFVCFYAYNFVRDVLQPYV
QESQNKFFKLPVSVVNTTLPCVAYVLLSLVYLPGVLAAALQLRRGTKYQRFPDWLDHWLQ
HRKQIGLLSFFCAALHALYSFCLPLRRAHRYDLVNLAVKQVLANKSHLWVEEEVWRMEIY
LSLGVLALGTLSLLAVTSLPSIANSLNWREFSFVQSSLGFVALVLSTLHTLTYGWTRAFE
ESRYKFYLPPTFTLTLLVPCVVILAKALFLLPCISRRLARIRRGWERESTIKFTLPTDHA
LAEKTSHV
>sp|Q68D91|MBLC2_HUMAN Acyl-coenzyme A thioesterase MBLAC2 OS=Homo sapiens OX=9606 GN=MBLAC2 PE=1 SV=3
MSALEWYAHKSLGDGIFWIQERFYESGNRANIWLVRGSEQDVVIDTGLGLRSLPEYLYSS
GLLQDREAKEDAARRPLLAVATHVHFDHSGGLYQFDRVAVHHAEAEALARGDNFETVTWL
SDSEVVRTPSPGWRARQFRVQAVQPTLILQDGDVINLGDRQLTVMHMPGHSRGSICLHDK
DRKILFSGDVVYDGSLIDWLPYSRISDYVGTCERLIELVDRGLVEKVLPGHFNTFGAERL
FRLASNYISKAGICHKVSTFAMRSLASLALRVTNSRTSP
>sp|Q6BCY4|NB5R2_HUMAN NADH-cytochrome b5 reductase 2 OS=Homo sapiens OX=9606 GN=CYB5R2 PE=1 SV=1
MNSRRREPITLQDPEAKYPLPLIEKEKISHNTRRFRFGLPSPDHVLGLPVGNYVQLLAKI
DNELVVRAYTPVSSDDDRGFVDLIIKIYFKNVHPQYPEGGKMTQYLENMKIGETIFFRGP
RGRLFYHGPGNLGIRPDQTSEPKKTLADHLGMIAGGTGITPMLQLIRHITKDPSDRTRMS
LIFANQTEEDILVRKELEEIARTHPDQFNLWYTLDRPPIGWKYSSGFVTADMIKEHLPPP
AKSTLILVCGPPPLIQTAAHPNLEKLGYTQDMIFTY
>sp|Q6DKJ4|NXN_HUMAN Nucleoredoxin OS=Homo sapiens OX=9606 GN=NXN PE=1 SV=2
MSGFLEELLGEKLVTGGGEEVDVHSLGARGISLLGLYFGCSLSAPCAQLSASLAAFYGRL
RGDAAAGPGPGAGAGAAAEPEPRRRLEIVFVSSDQDQRQWQDFVRDMPWLALPYKEKHRK
LKLWNKYRISNIPSLIFLDATTGKVVCRNGLLVIRDDPEGLEFPWGPKPFREVIAGPLLR
NNGQSLESSSLEGSHVGVYFSAHWCPPCRSLTRVLVESYRKIKEAGQNFEIIFVSADRSE
ESFKQYFSEMPWLAVPYTDEARRSRLNRLYGIQGIPTLIMLDPQGEVITRQGRVEVLNDE
DCREFPWHPKPVLELSDSNAAQLNEGPCLVLFVDSEDDGESEAAKQLIQPIAEKIIAKYK
AKEEEAPLLFFVAGEDDMTDSLRDYTNLPEAAPLLTILDMSARAKYVMDVEEITPAIVEA
FVNDFLAEKLKPEPI
>tr|Q6FGX3|Q6FGX3_HUMAN RAB6A protein OS=Homo sapiens OX=9606 GN=RAB6A PE=2 SV=1
MSTGGDFGNPLRKFKLVFLGEQSVGKTSLITRFMYDSFDNTYQATIGIDFLSKTMYLEDR
TIRLQLWDTAGQERFRSLIPSYIRDSAAAVVVYDITNVNSFQQTTKWIDDVRTERGSDVI
IMLVGNKTDLADKRQVSIEEGERKAKELNVMFIETSAKAGYDVKQLFRRVAAALPGMEST
QDRSREDMIDIKLEKPQEQPVSEGGCSC

>sp|Q6GMV2|SMYD5_HUMAN Histone-lysine N-trimethyltransferase SMYD5 OS=Homo sapiens OX=9606 GN=SMYD5 PE=1 SV=2
MAASMCDVFSFCVGVAGRARVSVEVRFVSSAKGKGLFATQLIRKGETIFVERPLVAAQFL
WNALYRYRACDHCLRALEKAEENAQRLTGKPGQVLPHPELCTVRKDLHQNCPHCQVMYCS
AECRLAATEQYHQVLCPGPSQDDPLHPLNKLQEAWRSIHYPPETASIMLMARMVATVKQA
KDKDRWIRLFSQFCNKTANEEEEIVHKLLGDKFKGQLELLRRLFTEALYEEAVSQWFTPD
GFRSLFALVGTNGQGIGTSSLSQWVHACDTLELKPQDREQLDAFIDQLYKDIEAATGEFL
NCEGSGLFVLQSCCNHSCVPNAETSFPENNFLLHVTALEDIKPGEEICISYLDCCQRERS
RHSRHKILRENYLFVCSCPKCLAEADEPNVTSEEEEEEEEEEEGEPEDAELGDEMTDV
>sp|Q6IAA8|LTOR1_HUMAN Ragulator complex protein LAMTOR1 OS=Homo sapiens OX=9606 GN=LAMTOR1 PE=1 SV=2
MGCCYSSENEDSDQDREERKLLLDPSSPPTKALNGAEPNYHSLPSARTDEQALLSSILAK
TASNIIDVSAADSQGMEQHEYMDRARQYSTRLAVLSSSLTHWKKLPPLPSLTSQPHQVLA
SEPIPFSDLQQVSRIAAYAYSALSQIRVDAKEELVVQFGIP
>sp|Q6IQ22|RAB12_HUMAN Ras-related protein Rab-12 OS=Homo sapiens OX=9606 GN=RAB12 PE=1 SV=3
MDPGAALQRRAGGGGGLGAGSPALSGGQGRRRKQPPRPADFKLQVIIIGSRGVGKTSLME
RFTDDTFCEACKSTVGVDFKIKTVELRGKKIRLQIWDTAGQERFNSITSAYYRSAKGIIL
VYDITKKETFDDLPKWMKMIDKYASEDAELLLVGNKLDCETDREITRQQGEKFAQQITGM
RFCEASAKDNFNVDEIFLKLVDDILKKMPLDILRNELSNSILSLQPEPEIPPELPPPRPH
VRCC
>sp|Q6KB66|K2C80_HUMAN Keratin, type II cytoskeletal 80 OS=Homo sapiens OX=9606 GN=KRT80 PE=1 SV=2
MACRSCVVGFSSLSSCEVTPVGSPRPGTSGWDSCRAPGPGFSSRSLTGCWSAGTISKVTV
NPGLLVPLDVKLDPAVQQLKNQEKEEMKALNDKFASLIGKVQALEQRNQLLETRWSFLQG
QDSAIFDLGHLYEEYQGRLQEELRKVSQERGQLEANLLQVLEKVEEFRIRYEDEISKRTD
MEFTFVQLKKDLDAECLHRTELETKLKSLESFVELMKTIYEQELKDLAAQVKDVSVTVGM
DSRCHIDLSGIVEEVKAQYDAVAARSLEEAEAYSRSQLEEQAARSAEYGSSLQSSRSEIA
DLNVRIQKLRSQILSVKSHCLKLEENIKTAEEQGELAFQDAKTKLAQLEAALQQAKQDMA
RQLRKYQELMNVKLALDIEIATYRKLVEGEEGRMDSPSATVVSAVQSRCKTAASRSGLSK
APSRKKKGSKGPVIKITEMSEKYFSQESEVSE
>sp|Q6N022|TEN4_HUMAN Teneurin-4 OS=Homo sapiens OX=9606 GN=TENM4 PE=1 SV=2
MDVKERKPYRSLTRRRDAERRYTSSSADSEEGKAPQKSYSSSETLKAYDQDARLAYGSRV
KDIVPQEAEEFCRTGANFTLRELGLEEVTPPHGTLYRTDIGLPHCGYSMGAGSDADMEAD
TVLSPEHPVRLWGRSTRSGRSSCLSSRANSNLTLTDTEHENTETDHPGGLQNHARLRTPP
PPLSHAHTPNQHHAASINSLNRGNFTPRSNPSPAPTDHSLSGEPPAGGAQEPAHAQENWL
LNSNIPLETRNLGKQPFLGTLQDNLIEMDILGASRHDGAYSDGHFLFKPGGTSPLFCTTS
PGYPLTSSTVYSPPPRPLPRSTFARPAFNLKKPSKYCNWKCAALSAIVISATLVILLAYF
VAMHLFGLNWHLQPMEGQMYEITEDTASSWPVPTDVSLYPSGGTGLETPDRKGKGTTEGK
PSSFFPEDSFIDSGEIDVGRRASQKIPPGTFWRSQVFIDHPVHLKFNVSLGKAALVGIYG
RKGLPPSHTQFDFVELLDGRRLLTQEARSLEGTPRQSRGTVPPSSHETGFIQYLDSGIWH
LAFYNDGKESEVVSFLTTAIESVDNCPSNCYGNGDCISGTCHCFLGFLGPDCGRASCPVL
CSGNGQYMKGRCLCHSGWKGAECDVPTNQCIDVACSNHGTCITGTCICNPGYKGESCEEV
DCMDPTCSGRGVCVRGECHCSVGWGGTNCETPRATCLDQCSGHGTFLPDTGLCSCDPSWT

GHDCSIEICAADCGGHGVCVGGTCRCEDGWMGAACDQRACHPRCAEHGTCRDGKCECSPG
WNGEHCTIAHYLDRVVKEGCPGLCNGNGRCTLDLNGWHCVCQLGWRGAGCDTSMETACGD
SKDNDGDGLVDCMDPDCCLQPLCHINPLCLGSPNPLDIIQETQVPVSQQNLHSFYDRIKF
LVGRDSTHIIPGENPFDGGHACVIRGQVMTSDGTPLVGVNISFVNNPLFGYTISRQDGSF
DLVTNGGISIILRFERAPFITQEHTLWLPWDRFFVMETIIMRHEENEIPSCDLSNFARPN
PVVSPSPLTSFASSCAEKGPIVPEIQALQEEISISGCKMRLSYLSSRTPGYKSVLRISLT
HPTIPFNLMKVHLMVAVEGRLFRKWFAAAPDLSYYFIWDKTDVYNQKVFGLSEAFVSVGY
EYESCPDLILWEKRTTVLQGYEIDASKLGGWSLDKHHALNIQSGILHKGNGENQFVSQQP
PVIGSIMGNGRRRSISCPSCNGLADGNKLLAPVALTCGSDGSLYVGDFNYIRRIFPSGNV
TNILELRNKDFRHSHSPAHKYYLATDPMSGAVFLSDSNSRRVFKIKSTVVVKDLVKNSEV
VAGTGDQCLPFDDTRCGDGGKATEATLTNPRGITVDKFGLIYFVDGTMIRRIDQNGIIST
LLGSNDLTSARPLSCDSVMDISQVHLEWPTDLAINPMDNSLYVLDNNVVLQISENHQVRI
VAGRPMHCQVPGIDHFLLSKVAIHATLESATALAVSHNGVLYIAETDEKKINRIRQVTTS
GEISLVAGAPSGCDCKNDANCDCFSGDDGYAKDAKLNTPSSLAVCADGELYVADLGNIRI
RFIRKNKPFLNTQNMYELSSPIDQELYLFDTTGKHLYTQSLPTGDYLYNFTYTGDGDITL
ITDNNGNMVNVRRDSTGMPLWLVVPDGQVYWVTMGTNSALKSVTTQGHELAMMTYHGNSG
LLATKSNENGWTTFYEYDSFGRLTNVTFPTGQVSSFRSDTDSSVHVQVETSSKDDVTITT
NLSASGAFYTLLQDQVRNSYYIGADGSLRLLLANGMEVALQTEPHLLAGTVNPTVGKRNV
TLPIDNGLNLVEWRQRKEQARGQVTVFGRRLRVHNRNLLSLDFDRVTRTEKIYDDHRKFT
LRILYDQAGRPSLWSPSSRLNGVNVTYSPGGYIAGIQRGIMSERMEYDQAGRITSRIFAD
GKTWSYTYLEKSMVLLLHSQRQYIFEFDKNDRLSSVTMPNVARQTLETIRSVGYYRNIYQ
PPEGNASVIQDFTEDGHLLHTFYLGTGRRVIYKYGKLSKLAETLYDTTKVSFTYDETAGM
LKTINLQNEGFTCTIRYRQIGPLIDRQIFRFTEEGMVNARFDYNYDNSFRVTSMQAVINE
TPLPIDLYRYDDVSGKTEQFGKFGVIYYDINQIITTAVMTHTKHFDAYGRMKEVQYEIFR
SLMYWMTVQYDNMGRVVKKELKVGPYANTTRYSYEYDADGQLQTVSINDKPLWRYSYDLN
GNLHLLSPGNSARLTPLRYDIRDRITRLGDVQYKMDEDGFLRQRGGDIFEYNSAGLLIKA
YNRAGSWSVRYRYDGLGRRVSSKSSHSHHLQFFYADLTNPTKVTHLYNHSSSEITSLYYD
LQGHLFAMELSSGDEFYIACDNIGTPLAVFSGTGLMIKQILYTAYGEIYMDTNPNFQIII
GYHGGLYDPLTKLVHMGRRDYDVLAGRWTSPDHELWKHLSSSNVMPFNLYMFKNNNPISN
SQDIKCFMTDVNSWLLTFGFQLHNVIPGYPKPDMDAMEPSYELIHTQMKTQEWDNSKSIL
GVQCEVQKQLKAFVTLERFDQLYGSTITSCQQAPKTKKFASSGSVFGKGVKFALKDGRVT
TDIISVANEDGRRVAAILNHAHYLENLHFTIDGVDTHYFVKPGPSEGDLAILGLSGGRRT
LENGVNVTVSQINTVLNGRTRRYTDIQLQYGALCLNTRYGTTLDEEKARVLELARQRAVR
QAWAREQQRLREGEEGLRAWTEGEKQQVLSTGRVQGYDGFFVISVEQYPELSDSANNIHF
MRQSEMGRR
>sp|Q6NUS8|UD3A1_HUMAN UDP-glucuronosyltransferase 3A1 OS=Homo sapiens OX=9606
GN=UGT3A1 PE=2 SV=1
MVGQRVLLLVAFLLSGVLLSEAAKILTISTLGGSHYLLLDRVSQILQEHGHNVTMLHQSG
KFLIPDIKEEEKSYQVIRWFSPEDHQKRIKKHFDSYIETALDGRKESEALVKLMEIFGTQ
CSYLLSRKDIMDSLKNENYDLVFVEAFDFCSFLIAEKLVKPFVAILPTTFGSLDFGLPSP
LSYVPVFPSLLTDHMDFWGRVKNFLMFFSFSRSQWDMQSTFDNTIKEHFPEGSRPVLSHL
LLKAELWFVNSDFAFDFARPLLPNTVYIGGLMEKPIKPVPQDLDNFIANFGDAGFVLVAF
GSMLNTHQSQEVLKKMHNAFAHLPQGVIWTCQSSHWPRDVHLATNVKIVDWLPQSDLLAH
PSIRLFVTHGGQNSVMEAIRHGVPMVGLPVNGDQHGNMVRVVAKNYGVSIRLNQVTADTL

TLTMKQVIEDKRYKSAVVAASVILHSQPLSPAQRLVGWIDHILQTGGATHLKPYAFQQPW
HEQYLIDVFVFLLGLTLGTMWLCGKLLGVVARWLRGARKVKKT
>sp|Q6NZI2|CAVN1_HUMAN Caveolae-associated protein 1 OS=Homo sapiens OX=9606 GN=CAVIN1 PE=1 SV=1
MEDPTLYIVERPLPGYPDAEAPEPSSAGAQAAEEPSGAGSEELIKSDQVNGVLVLSLLDK
IIGAVDQIQLTQAQLEERQAEMEGAVQSIQGELSKLGKAHATTSNTVSKLLEKVRKVSVN
VKTVRGSLERQAGQIKKLEVNEAELLRRRNFKVMIYQDEVKLPAKLSISKSLKESEALPE
KEGEELGEGERPEEDAAALELSSDEAVEVEEVIEESRAERIKRSGLRRVDDFKKAFSKEK
MEKTKVRTRENLEKTRLKTKENLEKTRHTLEKRMNKLGTRLVPAERREKLKTSRDKLRKS
FTPDHVVYARSKTAVYKVPPFTFHVKKIREGQVEVLKATEMVEVGADDDEGGAERGEAGD
LRRGSSPDVHALLEITEESDAVLVDKSDSD
>sp|Q6PCB0|VWA1_HUMAN von Willebrand factor A domain-containing protein 1 OS=Homo sapiens OX=9606 GN=VWA1 PE=1 SV=1
MLPWTALGLALSLRLALARSGAERGPPASAPRGDLMFLLDSSASVSHYEFSRVREFVGQL
VAPLPLGTGALRASLVHVGSRPYTEFPFGQHSSGEAAQDAVRASAQRMGDTHTGLALVYA
KEQLFAEASGARPGVPKVLVWVTDGGSSDPVGPPMQELKDLGVTVFIVSTGRGNFLELSA
AASAPAEKHLHFVDVDDLHIIVQELRGSILDAMRPQQLHATEITSSGFRLAWPPLLTADS
GYYVLELVPSAQPGAARRQQLPGNATDWIWAGLDPDTDYDVALVPESNVRLLRPQILRVR
TRPGEAGPGASGPESGAGPAPTQLAALPAPEEAGPERIVISHARPRSLRVSWAPALGSAA
ALGYHVQFGPLRGGEAQRVEVPAGRNCTTLQGLAPGTAYLVTVTAAFRSGRESALSAKAC
TPDGPRPRPRPVPRAPTPGTASREP
>sp|Q6PIU2|NCEH1_HUMAN Neutral cholesterol ester hydrolase 1 OS=Homo sapiens OX=9606 GN=NCEH1 PE=1 SV=3
MRSSCVLLTALVALAAYYVYIPLPGSVSDPWKLMLLDATFRGAQQVSNLIHYLGLSHHLL
ALNFIIVSFGKKSAWSSAQVKVTDTDFDGVEVRVFEGPPKPEEPLKRSVVYIHGGGWALA
SAKIRYYDELCTAMAEELNAVIVSIEYRLVPKVYFPEQIHDVVRATKYFLKPEVLQKYMV
DPGRICISGDSAGGNLAAALGQQFTQDASLKNKLKLQALIYPVLQALDFNTPSYQQNVNT
PILPRYVMVKYWVDYFKGNYDFVQAMIVNNHTSLDVEEAAAVRARLNWTSLLPASFTKNY
KPVVQTTGNARIVQELPQLLDARSAPLIADQAVLQLLPKTYILTCEHDVLRDDGIMYAKR
LESAGVEVTLDHFEDGFHGCMIFTSWPTNFSVGIRTRNSYIKWLDQNL
>sp|Q6PJG6|BRAT1_HUMAN BRCA1-associated ATM activator 1 OS=Homo sapiens OX=9606 GN=BRAT1 PE=1 SV=2
MDPECAQLLPALCAVLVDPRQPVADDTCLEKLLDWFKTVTEGESSVVLLQEHPCLVELLS
HVLKVQDLSSGVLSFSLRLAGTFAAQENCFQYLQQGELLPGLFGEPGPLGRATWAVPTVR
SGWIQGLRSLAQHPSALRFLADHGAVDTIFSLQGDSSLFVASAASQLLVHVLALSMRGGA
EGQPCLPGGDWPACAQKIMDHVEESLCSAATPKVTQALNVLTTTFGRCQSPWTEALWVRL
SPRVACLLERDPIPAAHSFVDLLLCVARSPVFSSSDGSLWETVARALSCLGPTHMGPLAL
GILKLEHCPQALRTQAFQVLLQPLACVLKATVQAPGPPGLLDGTADDATTVDTLLASKSS
CAGLLCRTLAHLEELQPLPQRPSPWPQASLLGATVTVLRLCDGSAAPASSVGGHLCGTLA
GCVRVQRAALDFLGTLSQGTGPQELVTQALAVLLECLESPGSSPTVLKKAFQATLRWLLS
SPKTPGCSDLGPLIPQFLRELFPVLQKRLCHPCWEVRDSALEFLTQLSRHWGGQADFRCA
LLASEVPQLALQLLQDPESYVRASAVTAMGQLSSQGLHAPTSPEHAEARQSLFLELLHIL
SVDSEGFPRRAVMQVFTEWLRDGHADAAQDTEQFVATVLQAASRDLDWEVRAQGLELALV
FLGQTLGPPRTHCPYAVALPEVAPAQPLTEALRALCHVGLFDFAFCALFDCDRPVAQKSC

DLLLFLRDKIASYSSLREARGSPNTASAEATLPRWRAGEQAQPPGDQEPEAVLAMLRSLD
LEGLRSTLAESSDHVEKSPQSLLQDMLATGGFLQGDEADCY
>sp|Q6S8J3|POTEE_HUMAN POTE ankyrin domain family member E OS=Homo sapiens
OX=9606 GN=POTEE PE=2 SV=3
MVVEVDSMPAASSVKKPFGLRSKMGKWCCRCFPCYRESGKSNVGTSGDHDDSAMKTLRSK
MGKWCHHCFPCCRGSGKSNVGASGDHDDSAMKTLRNKMGKWCCHCFPCCRGSGKSKVGAW
GDYDDSAFMEPRYHVRGEDLDKLHRAAWWGKVPRKDLIVMLRDTDVNKKDKQKRTALHLA
SANGNSEVVKLLLDRRCQLNVLDNKKRTALIKAVQCQEDECALMLLEHGTDPNIPDEYGN
TTLHYAIYNEDKLMAKALLLYGADIESKNKHGLTPLLLGVHEQKQQVVKFLIKKKANLNA
LDRYGRTALILAVCCGSASIVSLLLEQNIDVSSQDLSGQTAREYAVSSHHHVICQLLSDY
KEKQMLKISSENSNPEQELKLTSEEESQRFKGSENSQPEKMSQELEINKDGDREVEEEMK
KHESNNVGLLENLTNGVTAGNGDNGLIPQRKSRTPENQQFPDNESEEYHRICELLSDYKE
KQMPKYSSENSNPEQDLKLTSEEESQRLKGSENGQPEKRSQEPEINKDGDRELENFMAIE
EMKKHGSTHVGFPENLTNGATAGNGDDGLIPPRKSRTPESQQFPDTENEEYHSDEQNDTQ
KQFCEEQNTGILHDEILIHEEKQIEVVEKMNSELSLSCKKEKDVLHENSTLREEIAMLRL
ELDTMKHQSQLREKKYLEDIESVKKKNDNLLKALQLNELTMDDDTAVLVIDNGSGMCKAG
FAGDDAPRAVFPSIVGRPRQQGMMGGMHQKESYVGKEAQSKRGILTLKYPMEHGIITNWD
DMEKIWHHTFYNELRVAPEEHPILLTEAPLNPKANREKMTQIMFETFNTPAMYVAIQAVP
SLYTSGRTTGIVMDSGDGVTHTVPIYEGNALPHATLRLDLAGRELPDYLMKILTERGYRF
TTMAEREIVRDIKEKLCYVALDFEQEMATAASSSSLEKSYELPDGQVITIGNERFRCPEA
LFQPCFLGMESCGIHETTFNSIMKSDVDIRKDLYTNTVLSGGTTMYPGMAHRMQKEIAAL
APSMMKIRIIAPPKRKYSVWVGGSILASLSTFQQMWISKQEYDESGPSIVHRKCF
>tr|Q6TGM5|Q6TGM5_HUMAN cytochrome-b5 reductase (Fragment) OS=Homo sapiens
OX=9606 PE=2 SV=1
QGKGKFAIRPDKKSNPIIRTVKSVGTIAGGTGITPMLQVIRAIMKDPDDHTVCHLLFANQ
>tr|Q6TXQ4|Q6TXQ4_HUMAN H3L-like histone (Fragment) OS=Homo sapiens OX=9606 PE=1
SV=1
APRKQLATKAARKSAPSTGGVKKPHRYRPGTVALREIRRYQKSTELLVRKLPFQRLVREI
AQDFKTDLRFQSAAIGALQEASEAYLVNLFEDTNLCAIHAKRVTIMP
>sp|Q6UWM7|LCTL_HUMAN Lactase-like protein OS=Homo sapiens OX=9606 GN=LCTL PE=1
SV=2
MKPVWVATLLWMLLLVPRLGAARKGSPEEASFYYGTFPLGFSWGVGSSAYQTEGAWDQDG
KGPSIWDVFTHSGKGKVLGNETADVACDGYYKVQEDIILLRELHVNHYRFSLSWPRLLPT
GIRAEQVNKKGIEFYSDLIDALLSSNITPIVTLHHWDLPQLLQVKYGGWQNVSMANYFRD
YANLCFEAFGDRVKHWITFSDPRAMAEKGYETGHHAPGLKLRGTGLYKAAHHIIKAHAKA
WHSYNTTWRSKQQGLVGISLNCDWGEPVDISNPKDLEAAERYLQFCLGWFANPIYAGDYP
QVMKDYIGRKSAEQGLEMSRLPVFSLQEKSYIKGTSDFLGLGHFTTRYITERNYPSRQGP
SYQNDRDLIELVDPNWPDLGSKWLYSVPWGFRRLLNFAQTQYGDPPIYVMENGASQKFHC
TQLCDEWRIQYLKGYINEMLKAIKDGANIKGYTSWSLLDKFEWEKGYSDRYGFYYVEFND
RNKPRYPKASVQYYKKIIIANGFPNPREVESWYLKALETCSINNQMLAAEPLLSHMQMVT
EIVVPTVCSLCVLITAVLLMLLLRRQS
>sp|Q6UY14|ATL4_HUMAN ADAMTS-like protein 4 OS=Homo sapiens OX=9606 GN=ADAMTSL4
PE=1 SV=2
MENWTGRPWLYLLLLLSLPQLCLDQEVLSGHSLQTPTEEGQGPEGVWGPWVQWASCSQPC

GVGVQRRSRTCQLPTVQLHPSLPLPPRPPRHPEALLPRGQGPRPQTSPETLPLYRTQSRG
RGGPLRGPASHLGREETQEIRAARRSRLRDPIKPGMFGYGRVPFALPLHRNRRHPRSPPR
SELSLISSRGEEAIPSPTPRAEPFSANGSPQTELPPTELSVHTPSPQAEPLSPETAQTEV
APRTRPAPLRHHPRAQASGTEPPSPTHSLGEGGFFRASPQPRRPSSQGWASPQVAGRRPD
PFPSVPRGRGQQGQGPWGTGGTPHGPRLEPDPQHPGAWLPLLSNGPHASSLWSLFAPSSP
IPRCSGESEQLRACSQAPCPPEQPDPRALQCAAFNSQEFMGQLYQWEPFTEVQGSQRCEL
NCRPRGFRFYVRHTEKVQDGTLCQPGAPDICVAGRCLSPGCDGILGSGRRPDGCGVCGGD
DSTCRLVSGNLTDRGGPLGYQKILWIPAGALRLQIAQLRPSSNYLALRGPGGRSIINGNW
AVDPPGSYRAGGTVFRYNRPPREEGKGESLSAEGPTTQPVDVYMIFQEENPGVFYQYVIS
SPPPILENPTPEPPVPQLQPEILRVEPPLAPAPRPARTPGTLQRQVRIPQMPAPPHPRTP
LGSPAAYWKRVGHSACSASCGKGVWRPIFLCISRESGEELDERSCAAGARPPASPEPCHG
TPCPPYWEAGEWTSCSRSCGPGTQHRQLQCRQEFGGGGSSVPPERCGHLPRPNITQSCQL
RLCGHWEVGSPWSQCSVRCGRGQRSRQVRCVGNNGDEVSEQECASGPPQPPSREACDMGP
CTTAWFHSDWSSKCSAECGTGIQRRSVVCLGSGAALGPGQGEAGAGTGQSCPTGSRPPDM
RACSLGPCERTWRWYTGPWGECSSECGSGTQRRDIICVSKLGTEFNVTSPSNCSHLPRPP
ALQPCQGQACQDRWFSTPWSPCSRSCQGGTQTREVQCLSTNQTLSTRCPPQLRPSRKRPC
NSQPCSQRPDDQCKDSSPHCPLVVQARLCVYPYYTATCCRSCAHVLERSPQDPS
>sp|Q6XQN6|PNCB_HUMAN Nicotinate phosphoribosyltransferase OS=Homo sapiens OX=9606
GN=NAPRT PE=1 SV=2
MAAEQDPEARAAARPLLTDLYQATMALGYWRAGRARDAAEFELFFRRCPFGGAFALAAGL
RDCVRFLRAFRLRDADVQFLASVLPPDTDPAFFEHLRALDCSEVTVRALPEGSLAFPGVP
LLQVSGPLLVVQLLETPLLCLVSYASLVATNAARLRLIAGPEKRLLEMGLRRAQGPDGGL
TASTYSYLGGFDSSSNVLAGQLRGVPVAGTLAHSFVTSFSGSEVPPDPMLAPAAGEGPGV
DLAAKAQVWLEQVCAHLGLGVQEPHPGERAAFVAYALAFPRAFQGLLDTYSVWRSGLPNF
LAVALALGELGYRAVGVRLDSGDLLQQAQEIRKVFRAAAAQFQVPWLESVLIVVSNNIDE
EALARLAQEGSEVNVIGIGTSVVTCPQQPSLGGVYKLVAVGGQPRMKLTEDPEKQTLPGS
KAAFRLLGSDGSPLMDMLQLAEEPVPQAGQELRVWPPGAQEPCTVRPAQVEPLLRLCLQQ
GQLCEPLPSLAESRALAQLSLSRLSPEHRRLRSPAQYQVVLSERLQALVNSLCAGQSP
>sp|Q6ZMM2|ATL5_HUMAN ADAMTS-like protein 5 OS=Homo sapiens OX=9606
GN=ADAMTSL5 PE=1 SV=3
MGKLRPGRVEWLASGHTERPHLFQNLLLFLWALLNCGLGVSAQGPGEWTPWVSWTRCSSS
CGRGVSVRSRRCLRLPGEEPCWGDSHEYRLCQLPDCPPGAVPFRDLQCALYNGRPVLGTQ
KTYQWVPFHGAPNQCDLNCLAEGHAFYHSFGRVLDGTACSPGAQGVCVAGRCLSAGCDGL
LGSGALEDRCGRCGGANDSCLFVQRVFRDAGAFAGYWNVTLIPEGARHIRVEHRSRNHLA
LMGGDGRYVLNGHWVVSPPGTYEAAGTHVVYTRDTGPQETLQAAGPTSHDLLLQVLLQEP
NPGIEFEFWLPRERYSPFQARVQALGWPLRQPQPRGVEPQPPAAPAVTPAQTPTLAPDPC
PPCPDTRGRAHRLLHYCGSDFVFQARVLGHHHQAQETRYEVRIQLVYKNRSPLRAREYVW
APGHCPCPMLAPHRDYLMAVQRLVSPDGTQDQLLLPHAGYARPWSPAEDSRIRLTARRCP
G
>tr|Q6ZNS6|Q6ZNS6_HUMAN Adenosylhomocysteinase OS=Homo sapiens OX=9606 PE=2
SV=1
MYFLCFFCLFVSGRKWSRQFLLSFWCTVCSAHQGTIFLSLKIQFADQKQEFNKRPTKIGR
RSLSRSISQSSTDSYSSAASYTDSSDDETSPRDKQQKNSKGSSDFCVKNIKQAEFGRREI
EIAEQEMPALMALRKRAQGEKPLAGAKIVGCTHITAQTAVLMETLGALGAQCRWAACNIY

STLNEVAAALAESGFPVFAWKGESEDDFWWCIDRCVNVEGWQPNMILDDGGDLTHWIYKK
YPNMFKKIKGIVEESVTGVHRLYQLSKAGKLCVPAMNVNDSVTKQKFDNLYCCRESILDG
LKRTTDMMFGGKQVVVCGYGEVGKGCCAALKAMGSIVYVTEIDPICALQACMDGFRLVKL
NEVIRQVDIVITCTGNKNVVTREHLDRMKNSCIVCNMGHSNTEIDVASLRTPELTWERVR
SQVDHVIWPDGKRIVLLAEGRLLNLSCSTMPTFVLSITATTQALALIELYNAPEGRYKQD
VYLLPKKMDEYVASLHLPTFDAHLTELTDEQAKYLGLNKNGPFKPNYYRY
>sp|Q6ZUX7|LHPL2_HUMAN LHFPL tetraspan subfamily member 2 protein OS=Homo sapiens OX=9606 GN=LHFPL2 PE=1 SV=2
MCHVIVTCRSMLWTLLSIVVAFAELIAFMSADWLIGKARSRGGVEPAGPGGGSPEPYHPT
LGIYARCIRNPGVQHFQRDTLCGPYAESFGEIASGFWQATAIFLAVGIFILCMVALVSVF
TMCVQSIMKKSIFNVCGLLQGIAGLFLILGLILYPAGWGCQKAIDYCGHYASAYKPGDCS
LGWAFYTAIGGTVLTFICAVFSAQAEIATSSDKVQEEIEEGKNLICLL
>sp|Q6ZVH7|ESPNL_HUMAN Espin-like protein OS=Homo sapiens OX=9606 GN=ESPNL PE=1 SV=3
MEKQRALVAAKDGDVATLERLLEAGALGPGITDALGAGLVHHATRAGHLDCVKFLVQRAQ
LPGNQRAHNGATPAHDAAATGSLAELCWLVREGGCGLQDQDASGVSPLHLAARFGHPVLV
EWLLHEGHSATLETREGARPLHHAAVSGDLTCLKLLTAAHGSSVNRRTRSGASPLYLACQ
EGHLHLAQFLVKDCGADVHLRALDGMSALHAAAARGHYSLVVWLVTFTDIGLTARDNEGA
TALHFAARGGHTPILDRLLLMGTPILRDSWGGTPLHDAAENGQMECCQTLVSHHVDPSLR
DEDGYTAADLAEYHGHRDCAQYLREVAQPVPLLMTPPPPPFPPPPLLATRRSLEDGRRGG
PGPGNPSPMSLSPAWPGHPDQPLPREQMTSPAPPRIITSATADPEGTETALAGDTSDGLA
ALQLDGLPSGDIDGLVPTRDERGQPIPEWKRQVMVRKLQARLGAESSAEAQDNGGSSGPT
EQAAWRYSQTHQAILGPFGELLTEDDLVYLEKQIADLQLRRRCQEYESELGRLAAELQAL
LPEPLVSITVNSHFLPRAPGLEVEEASIPAAEPAGSAEASEVAPGVQPLPFWCSHISRLV
RSLSLLLKGVHGLVQGDEKPSTRPLQDTCREASASPPRSEAQRQIQEWGVSVRTLRGNFE
SASGPLCGFNPGPCEPGAQHRQCLSGCWPALPKPRSGLASGEPRPGDTEEASDSGISCEE
VPSEAGAAAGPDLASLRKERIIMLFLSHWRRSAYTPALKTVACRTLGARHAGLRGQEAAR
SPGPPSPPSEGPRLGHLWQQRSTITHLLGNWKAIMAHVPARQLRRLSRQPRGALSPEQFL
PHVDGAPVPYSSLSLDLFMLGYFQLLECDLPAEERKLRHLLCFEVFEHLGTHGWEAVRAF
HKAVTDEVAAGRRAWTDGFEDIKARFFGSSQRPAWDTEPGRKSGLTLLGPLPHAAVPCSG
PEPTAQRLGSRSQQGSFNGEDICGYINRSFAFWKEKEAEMFNFGE
>sp|Q71DI3|H32_HUMAN Histone H3.2 OS=Homo sapiens OX=9606 GN=H3C15 PE=1 SV=3
MARTKQTARKSTGGKAPRKQLATKAARKSAPATGGVKKPHRYRPGTVALREIRRYQKSTE
LLIRKLPFQRLVREIAQDFKTDLRFQSSAVMALQEASEAYLVGLFEDTNLCAIHAKRVTI
MPKDIQLARRIRGERA
>sp|Q71U36|TBA1A_HUMAN Tubulin alpha-1A chain OS=Homo sapiens OX=9606 GN=TUBA1A PE=1 SV=1
MRECISIHVGQAGVQIGNACWELYCLEHGIQPDGQMPSDKTIGGGDDSFNTFFSETGAGK
HVPRAVFVDLEPTVIDEVRTGTYRQLFHPEQLITGKEDAANNYARGHYTIGKEIIDLVLD
RIRKLADQCTGLQGFLVFHSFGGGTGSGFTSLLMERLSVDYGKKSKLEFSIYPAPQVSTA
VVEPYNSILTTHTTLEHSDCAFMVDNEAIYDICRRNLDIERPTYTNLNRLIGQIVSSITA
SLRFDGALNVDLTEFQTNLVPYPRIHFPLATYAPVISAEKAYHEQLSVAEITNACFEPAN
QMVKCDPRHGKYMACCLLYRGDVVPKDVNAAIATIKTKRTIQFVDWCPTGFKVGINYQPP
TVVPGGDLAKVQRAVCMLSNTTAIAEAWARLDHKFDLMYAKRAFVHWYVGEGMEEGEFSE

AREDMAALEKDYEEVGVDSVEGEGEEEGEEY
>sp|Q7L2H7|EIF3M_HUMAN Eukaryotic translation initiation factor 3 subunit M OS=Homo sapiens OX=9606 GN=EIF3M PE=1 SV=1
MSVPAFIDISEEDQAAELRAYLKSKGAEISEENSEGGLHVDLAQIIEACDVCLKEDDKDV
ESVMNSVVSLLLILEPDKQEALIESLCEKLVKFREGERPSLRLQLLSNLFHGMDKNTPVR
YTVYCSLIKVAASCGAIQYIPTELDQVRKWISDWNLTTEKKHTLLRLLYEALVDCKKSDA
ASKVMVELLGSYTEDNASQARVDAHRCIVRALKDPNAFLFDHLLTLKPVKFLEGELIHDL
LTIFVSAKLASYVKFYQNNKDFIDSLGLLHEQNMAKMRLLTFMGMAVENKEISFDTMQQE
LQIGADDVEAFVIDAVRTKMVYCKIDQTQRKVVVSHSTHRTFGKQQWQQLYDTLNAWKQN
LNKVKNSLLSLSDT
>sp|Q7L523|RRAGA_HUMAN Ras-related GTP-binding protein A OS=Homo sapiens OX=9606 GN=RRAGA PE=1 SV=1
MPNTAMKKKVLLMGKSGSGKTSMRSIIFANYIARDTRRLGATIDVEHSHVRFLGNLVLNL
WDCGGQDTFMENYFTSQRDNIFRNVEVLIYVFDVESRELEKDMHYYQSCLEAILQNSPDA
KIFCLVHKMDLVQEDQRDLIFKEREEDLRRLSRPLECACFRTSIWDETLYKAWSSIVYQL
IPNVQQLEMNLRNFAQIIEADEVLLFERATFLVISHYQCKEQRDVHRFEKISNIIKQFKL
SCSKLAASFQSMEVRNSNFAAFIDIFTSNTYVMVVMSDPSIPSAATLINIRNARKHFEKL
ERVDGPKHSLLMR
>sp|Q7L576|CYFP1_HUMAN Cytoplasmic FMR1-interacting protein 1 OS=Homo sapiens OX=9606 GN=CYFIP1 PE=1 SV=1
MAAQVTLEDALSNVDLLEELPLPDQQPCIEPPPSSLLYQPNFNTNFEDRNAFVTGIARYI
EQATVHSSMNEMLEEGQEYAVMLYTWRSCSRAIPQVKCNEQPNRVEIYEKTVEVLEPEVT
KLMNFMYFQRNAIERFCGEVRRLCHAERRKDFVSEAYLITLGKFINMFAVLDELKNMKCS
VKNDHSAYKRAAQFLRKMADPQSIQESQNLSMFLANHNKITQSLQQQLEVISGYEELLAD
IVNLCVDYYENRMYLTPSEKHMLLKVMGFGLYLMDGSVSNIYKLDAKKRINLSKIDKYFK
QLQVVPLFGDMQIELARYIKTSAHYEENKSRWTCTSSGSSPQYNICEQMIQIREDHMRFI
SELARYSNSEVVTGSGRQEAQKTDAEYRKLFDLALQGLQLLSQWSAHVMEVYSWKLVHPT
DKYSNKDCPDSAEEYERATRYNYTSEEKFALVEVIAMIKGLQVLMGRMESVFNHAIRHTV
YAALQDFSQVTLREPLRQAIKKKKNVIQSVLQAIRKTVCDWETGHEPFNDPALRGEKDPK
SGFDIKVPRRAVGPSSTQLYMVRTMLESLIADKSGSKKTLRSSLEGPTILDIEKFHRESF
FYTHLINFSETLQQCCDLSQLWFREFFLELTMGRRIQFPIEMSMPWILTDHILETKEASM
MEYVLYSLDLYNDSAHYALTRFNKQFLYDEIEAEVNLCFDQFVYKLADQIFAYYKVMAGS
LLLDKRLRSECKNQGATIHLPPSNRYETLLKQRHVQLLGRSIDLNRLITQRVSAAMYKSL
ELAIGRFESEDLTSIVELDGLLEINRMTHKLLSRYLTLDGFDAMFREANHNVSAPYGRIT
LHVFWELNYDFLPNYCYNGSTNRFVRTVLPFSQEFQRDKQPNAQPQYLHGSKALNLAYSS
IYGSYRNFVGPPHFQVICRLLGYQGIAVVMEELLKVVKSLLQGTILQYVKTLMEVMPKIC
RLPRHEYGSPGILEFFHHQLKDIVEYAELKTVCFQNLREVGNAILFCLLIEQSLSLEEVC
DLLHAAPFQNILPRVHVKEGERLDAKMKRLESKYAPLHLVPLIERLGTPQQIAIAREGDL
LTKERLCCGLSMFEVILTRIRSFLDDPIWRGPLPSNGVMHVDECVEFHRLWSAMQFVYCI
PVGTHEFTVEQCFGDGLHWAGCMIIVLLGQQRRFAVLDFCYHLLKVQKHDGKDEIIKNVP
LKKMVERIRKFQILNDEIITILDKYLKSGDGEGTPVEHVRCFQPPIHQSLASS
>sp|Q7L5N1|CSN6_HUMAN COP9 signalosome complex subunit 6 OS=Homo sapiens OX=9606 GN=COPS6 PE=1 SV=1
MAAAAAAAAATNGTGGSSGMEVDAAVVPSVMACGVTGSVSVALHPLVILNISDHWIRMRS

QEGRPVQVIGALIGKQEGRNIEVMNSFELLSHTVEEKIIIDKEYYYTKEEQFKQVFKELE
FLGWYTTGGPPDPSDIHVHKQVCEIIESPLFLKLNPMTKHTDLPVSVFESVIDIINGEAT
MLFAELTYTLATEEAERIGVDHVARMTATGSGENSTVAEHLIAQHSAIKMLHSRVKLILE
YVKASEAGEVPFNHEILREAYALCHCLPVLSTDKFKTDFYDQCNDVGLMAYLGTITKTCN
TMNQFVNKFNVLYDRQGIGRRMRGLFF
>sp|Q7RTR2|NLRC3_HUMAN NLR family CARD domain-containing protein 3 OS=Homo sapiens OX=9606 GN=NLRC3 PE=1 SV=2
MRKQEVRTGREAGQGHGTGSPAEQVKALMDLLAGKGSQGSQAPQALDRTPDAPLGPCSND
SRIQRHRKALLSKVGGGPELGGPWHRLASLLLVEGLTDLQLREHDFTQVEATRGGGHPAR
TVALDRLFLPLSRVSVPPRVSITIGVAGMGKTTLVRHFVRLWAHGQVGKDFSLVLPLTFR
DLNTHEKLCADRLICSVFPHVGEPSLAVAVPARALLILDGLDECRTPLDFSNTVACTDPK
KEIPVDHLITNIIRGNLFPEVSIWITSRPSASGQIPGGLVDRMTEIRGFNEEEIKVCLEQ
MFPEDQALLGWMLSQVQADRALYLMCTVPAFCRLTGMALGHLWRSRTGPQDAELWPPRTL
CELYSWYFRMALSGEGQEKGKASPRIEQVAHGGRKMVGTLGRLAFHGLLKKKYVFYEQDM
KAFGVDLALLQGAPCSCFLQREETLASSVAYCFTHLSLQEFVAAAYYYGASRRAIFDLFT
ESGVSWPRLGFLTHFRSAAQRAMQAEDGRLDVFLRFLSGLLSPRVNALLAGSLLAQGEHQ
AYRTQVAELLQGCLRPDAAVCARAINVLHCLHELQHTELARSVEEAMESGALARLTGPAH
RAALAYLLQVSDACAQEANLSLSLSQGVLQSLLPQLLYCRKLRLDTNQFQDPVMELLGSV
LSGKDCRIQKISLAENQISNKGAKALARSLLVNRSLTSLDLRGNSIGPQGAKALADALKI
NRTLTSLSLQGNTVRDDGARSMAEALASNRTLSMLHLQKNSIGPMGAQRMADALKQNRSL
KELMFSSNSIGDGGAKALAEALKVNQGLESLDLQSNSISDAGVAALMGALCTNQTLLSLS
LRENSISPEGAQAIAHALCANSTLKNLDLTANLLHDQGARAIAVAVRENRTLTSLHLQWN
FIQAGAAQALGQALQLNRSLTSLDLQENAIGDDGACAVARALKVNTALTALYLQVASIGA
SGAQVLGEALAVNRTLEILDLRGNAIGVAGAKALANALKVNSSLRRLNLQENSLGMDGAI
CIATALSGNHRLQHINLQGNHIGDSGARMISEAIKTNAPTCTVEM
>sp|Q7Z406|MYH14_HUMAN Myosin-14 OS=Homo sapiens OX=9606 GN=MYH14 PE=1 SV=2
MAAVTMSVPGRKAPPRPGPVPEAAQPFLFTPRGPSAGGGPGSGTSPQVEWTARRLVWVPS
ELHGFEAAALRDEGEEEAEVELAESGRRLRLPRDQIQRMNPPKFSKAEDMAELTCLNEAS
VLHNLRERYYSGLIYTYSGLFCVVINPYKQLPIYTEAIVEMYRGKKRHEVPPHVYAVTEG
AYRSMLQDREDQSILCTGESGAGKTENTKKVIQYLAHVASSPKGRKEPGVPGELERQLLQ
ANPILEAFGNAKTVKNDNSSRFGKFIRINFDVAGYIVGANIETYLLEKSRAIRQAKDECS
FHIFYQLLGGAGEQLKADLLLEPCSHYRFLTNGPSSSPGQERELFQETLESLRVLGFSHE
EIISMLRMVSAVLQFGNIALKRERNTDQATMPDNTAAQKLCRLLGLGVTDFSRALLTPRI
KVGRDYVQKAQTKEQADFALEALAKATYERLFRWLVLRLNRALDRSPRQGASFLGILDIA
GFEIFQLNSFEQLCINYTNEKLQQLFNHTMFVLEQEEYQREGIPWTFLDFGLDLQPCIDL
IERPANPPGLLALLDEECWFPKATDKSFVEKVAQEQGGHPKFQRPRHLRDQADFSVLHYA
GKVDYKANEWLMKNMDPLNDNVAALLHQSTDRLTAEIWKDVEGIVGLEQVSSLGDGPPGG
RPRRGMFRTVGQLYKESLSRLMATLSNTNPSFVRCIVPNHEKRAGKLEPRLVLDQLRCNG
VLEGIRICRQGFPNRILFQEFRQRYEILTPNAIPKGFMDGKQACEKMIQALELDPNLYRV
GQSKIFFRAGVLAQLEEERDLKVTDIIVSFQAAARGYLARRAFQKRQQQQSALRVMQRNC
AAYLKLRHWQWWRLFTKVKPLLQVTRQDEVLQARAQELQKVQELQQQSAREVGELQGRVA
QLEEERARLAEQLRAEAELCAEAEETRGRLAARKQELELVVSELEARVGEEEECSRQMQT
EKKRLQQHIQELEAHLEAEEGARQKLQLEKVTTEAKMKKFEEDLLLLEDQNSKLSKERKL
LEDRLAEFSSQAAEEEEKVKSLNKLRLKYEATIADMEDRLRKEEKGRQELEKLKRRLDGE

SSELQEQMVEQQQRAEELRAQLGRKEEELQAALARAEDEGGARAQLLKSLREAQAALAEA
QEDLESERVARTKAEKQRRDLGEELEALRGELEDTLDSTNAQQELRSKREQEVTELKKTL
EEETRIHEAAVQELRQRHGQALGELAEQLEQARRGKGAWEKTRLALEAEVSELRAELSSL
QTARQEGEQRRRRLELQLQEVQGRAGDGERARAEAAEKLQRAQAELENVSGALNEAESKT
IRLSKELSSTEAQLHDAQELLQEETRAKLALGSRVRAMEAEAAGLREQLEEEAAARERAG
RELQTAQAQLSEWRRRQEEEAGALEAGEEARRRAAREAEALTQRLAEKTETVDRLERGRR
RLQQELDDATMDLEQQRQLVSTLEKKQRKFDQLLAEEKAAVLRAVEERERAEAEGREREA
RALSLTRALEEEQEAREELERQNRALRAELEALLSSKDDVGKSVHELERACRVAEQAAND
LRAQVTELEDELTAAEDAKLRLEVTVQALKTQHERDLQGRDEAGEERRRQLAKQLRDAEV
ERDEERKQRTLAVAARKKLEGELEELKAQMASAGQGKEEAVKQLRKMQAQMKELWREVEE
TRTSREEIFSQNRESEKRLKGLEAEVLRLQEELAASDRARRQAQQDRDEMADEVANGNLS
KAAILEEKRQLEGRLGQLEEELEEEQSNSELLNDRYRKLLLQVESLTTELSAERSFSAKA
ESGRQQLERQIQELRGRLGEEDAGARARHKMTIAALESKLAQAEEQLEQETRERILSGKL
VRRAEKRLKEVVLQVEEERRVADQLRDQLEKGNLRVKQLKRQLEEAEEEASRAQAGRRRL
QRELEDVTESAESMNREVTTLRNRLRRGPLTFTTRTVRQVFRLEEGVASDEEAEEAQPGS
GPSPEPEGSPPAHPQ
>sp|Q7Z4W1|DCXR_HUMAN L-xylulose reductase OS=Homo sapiens OX=9606 GN=DCXR PE=1
SV=2
MELFLAGRRVLVTGAGKGIGRGTVQALHATGARVVAVSRTQADLDSLVRECPGIEPVCVD
LGDWEATERALGSVGPVDLLVNNAAVALLQPFLEVTKEAFDRSFEVNLRAVIQVSQIVAR
GLIARGVPGAIVNVSSQCSQRAVTNHSVYCSTKGALDMLTKVMALELGPHKIRVNAVNPT
VVMTSMGQATWSDPHKAKTMLNRIPLGKFAEVEHVVNAILFLLSDRSGMTTGSTLPVEGG
FWAC
>sp|Q7Z553|MDGA2_HUMAN MAM domain-containing glycosylphosphatidylinositol anchor
protein 2 OS=Homo sapiens OX=9606 GN=MDGA2 PE=1 SV=2
MDLLYGLVWLLTVLLEGISGQGVYAPPTVRIVHSGLACNIEEERYSERVYTIREGETLEL
TCLVTGHPRPQIRWTKTAGSASDRFQDSSVFNETLRITNIQRHQGGRYYCKAENGLGSPA
IKSIRVDVYYLDDPVVTVHQSIGEAKEQFYYERTVFLRCVANSNPPVRYSWRRGQEVLLQ
GSDKGVEIYEPFFTQGETKILKLKNLRPQDYANYSCIASVRNVCNIPDKMVSFRLSNKTA
SPSIKLLVDDPIVVNPGEAITLVCVTTGGEPAPSLTWVRSFGTLPEKTVLNGGTLTIPAI
TSDDAGTYSCIANNNVGNPAKKSTNIIVRALKKGRFWITPDPYHKDDNIQIGREVKISCQ
VEAVPSEELTFSWFKNGRPLRSSERMVITQTDPDVSPGTTNLDIIDLKFTDFGTYTCVAS
LKGGGISDISIDVNISSSTVPPNLTVPQEKSPLVTREGDTIELQCQVTGKPKPIILWSRA
DKEVAMPDGSMQMESYDGTLRIVNVSREMSGMYRCQTSQYNGFNVKPREALVQLIVQYPP
AVEPAFLEIRQGQDRSVTMSCRVLRAYPIRVLTYEWRLGNKLLRTGQFDSQEYTEYAVKS
LSNENYGVYNCSIINEAGAGRCSFLVTGKAYAPEFYYDTYNPVWQNRHRVYSYSLQWTQM
NPDAVDRIVAYRLGIRQAGQQRWWEQEIKINGNIQKGELITYNLTELIKPEAYEVRLTPL
TKFGEGDSTIRVIKYSAPVNPHLREFHCGFEDGNICLFTQDDTDNFDWTKQSTATRNTKY
TPNTGPNADRSGSKEGFYMYIETSRPRLEGEKARLLSPVFSIAPKNPYGPTNTAYCFSFF
YHMYGQHIGVLNVYLRLKGQTTIENPLWSSSGNKGQRWNEAHVNIYPITSFQLIFEGIRG
PGIEGDIAIDDVSIAEGECAKQDLATKNSVDGAVGILVHIWLFPIIVLISILSPRR
>sp|Q7Z6K1|THAP5_HUMAN THAP domain-containing protein 5 OS=Homo sapiens OX=9606
GN=THAP5 PE=1 SV=2
MPRYCAAICCKNRRGRNNKDRKLSFYPFPLHDKERLEKWLKNMKRDSWVPSKYQFLCSDH

FTPDSLDIRWGIRYLKQTAVPTIFSLPEDNQGKDPSKKKSQKKNLEDEKEVCPKAKSEES
FVLNETKKNIVNTDVPHQHPELLHSSSLVKPPAPKTGSIQNNMLTLNLVKQHTGKPESTL
ETSVNQDTGRGGFHTCFENLNSTTITLTTSNSESIHQSLETQEVLEVTTSHLANPNFTSN
SMEIKSAQENPFLFSTINQTVEELNTNKESVIAIFVPAENSKPSVNSFISAQKETTEMED
TDIEDSLYKDVDYGTEVLQIEHSYCRQDINKEHLWQKVSKLHSKITLLELKEQQTLGRLK
SLEALIRQLKQENWLSEENVKIIENHFTTYEVTMI
>sp|Q7Z7A1|CNTRL_HUMAN Centriolin OS=Homo sapiens OX=9606 GN=CNTRL PE=1 SV=2
MKKGSQQKIFSKAKIPSSSHSPIPSSMSNMRSRSLSPLIGSETLPFHSGGQWCEQVEIAD
ENNMLLDYQDHKGADSHAGVRYITEALIKKLTKQDNLALIKSLNLSLSKDGGKKFKYIEN
LEKCVKLEVLNLSYNLIGKIEKLDKLLKLRELNLSYNKISKIEGIENMCNLQKLNLAGNE
IEHIPVWLGKKLKSLRVLNLKGNKISSLQDISKLKPLQDLISLILVENPVVTLPHYLQFT
IFHLRSLESLEGQPVTTQDRQEAFERFSLEEVERLERDLEKKMIETEELKSKQTRFLEEI
KNQDKLNKSLKEEAMLQKQSCEELKSDLNTKNELLKQKTIELTRACQKQYELEQELAFYK
IDAKFEPLNYYPSEYAEIDKAPDESPYIGKSRYKRNMFATESYIIDSAQAVQIKKMEPDE
QLRNDHMNLRGHTPLDTQLEDKEKKISAAQTRLSELHDEIEKAEQQILRATEEFKQLEEA
IQLKKISEAGKDLLYKQLSGRLQLVNKLRQEALDLELQMEKQKQEIAGKQKEIKDLQIAI
DSLDSKDPKHSHMKAQKSGKEQQLDIMNKQYQQLESRLDEILSRIAKETEEIKDLEEQLT
EGQIAANEALKKDLEGVISGLQEYLGTIKGQATQAQNECRKLRDEKETLLQRLTEVEQER
DQLEIVAMDAENMRKELAELESALQEQHEVNASLQQTQGDLSAYEAELEARLNLRDAEAN
QLKEELEKVTRLTQLEQSALQAELEKERQALKNALGKAQFSEEKEQENSELHAKLKHLQD
DNNLLKQQLKDFQNHLNHVVDGLVRPEEVAARVDELRRKLKLGTGEMNIHSPSDVLGKSL
ADLQKQFSEILARSKWERDEAQVRERKLQEEMALQQEKLATGQEEFRQACERALEARMNF
DKRQHEARIQQMENEIHYLQENLKSMEEIQGLTDLQLQEADEEKERILAQLRELEKKKKL
EDAKSQEQVFGLDKELKKLKKAVATSDKLATAELTIAKDQLKSLHGTVMKINQERAEELQ
EAERFSRKAAQAARDLTRAEAEIELLQNLLRQKGEQFRLEMEKTGVGTGANSQVLEIEKL
NETMERQRTEIARLQNVLDLTGSDNKGGFENVLEEIAELRREVSYQNDYISSMADPFKRR
GYWYFMPPPPSSKVSSHSSQATKDSGVGLKYSASTPVRKPRPGQQDGKEGSQPPPASGYW
VYSPIRSGLHKLFPSRDADSGGDSQEESELDDQEEPPFVPPPGYMMYTVLPDGSPVPQGM
ALYAPPPPLPNNSRPLTPGTVVYGPPPAGAPMVYGPPPPNFSIPFIPMGVLHCNVPEHHN
LENEVSRLEDIMQHLKSKKREERWMRASKRQSEKEMEELHHNIDDLLQEKKSLECEVEEL
HRTVQKRQQQKDFIDGNVESLMTELEIEKSLKHHEDIVDEIECIEKTLLKRRSELREADR
LLAEAESELSCTKEKTKNAVEKFTDAKRSLLQTESDAEELERRAQETAVNLVKADQQLRS
LQADAKDLEQHKIKQEEILKEINKIVAAKDSDFQCLSKKKEKLTEELQKLQKDIEMAERN
EDHHLQVLKESEVLLQAKRAELEKLKSQVTSQQQEMAVLDRQLGHKKEELHLLQGSMVQA
KADLQEALRLGETEVTEKCNHIREVKSLLEELSFQKGELNVQISERKTQLTLIKQEIEKE
EENLQVVLRQMSKHKTELKNILDMLQLENHELQGLKLQHDQRVSELEKTQVAVLEEKLEL
ENLQQISQQQKGEIEWQKQLLERDKREIERMTAESRALQSCVECLSKEKEDLQEKCDIWE
KKLAQTKRVLAAAEENSKMEQSNLEKLELNVRKLQQELDQLNRDKLSLHNDISAMQQQLQ
EKREAVNSLQEELANVQDHLNLAKQDLLHTTKHQDVLLSEQTRLQKDISEWANRFEDCQK
EEETKQQQLQVLQNEIEENKLKLVQQEMMFQRLQKERESEESKLETSKVTLKEQQHQLEK
ELTDQKSKLDQVLSKVLAAEERVRTLQEEERWCESLEKTLSQTKRQLSEREQQLVEKSGE
LLALQKEADSMRADFSLLRNQFLTERKKAEKQVASLKEALKIQRSQLEKNLLEQKQENSC
IQKEMATIELVAQDNHERARRLMKELNQMQYEYTELKKQMANQKDLERRQMEISDAMRTL
KSEVKDEIRTSLKNLNQFLPELPADLEAILERNENLEGELESLKENLPFTMNEGPFEEKL

NFSQVHIMDEHWRGEALREKLRHREDRLKAQLRHCMSKQAEVLIKGKRQTEGTLHSLRRQ
VDALGELVTSTSADSASSPSLSQLESSLTEDSQLGQNQEKNASAR
>sp|Q86T03|PP4P1_HUMAN Type 1 phosphatidylinositol 4,5-bisphosphate 4-phosphatase OS=Homo sapiens OX=9606 GN=PIP4P1 PE=1 SV=1
MAADGERSPLLSEPIDGGAGGNGLVGPGGSGAGPGGGLTPSAPPYGAAFPPFPEGHPAVL
PGEDPPPYSPLTSPDSGSAPMITCRVCQSLINVEGKMHQHVVKCGVCNEATPIKNAPPGK
KYVRCPCNCLLICKVTSQRIACPRPYCKRIINLGPVHPGPLSPEPQPMGVRVICGHCKNT
FLWTEFTDRTLARCPHCRKVSSIGRRYPRKRCICCFLLGLLLAVTATGLAFGTWKHARRY
GGIYAAWAFVILLAVLCLGRALYWACMKVSHPVQNFS
>sp|Q86UE4|LYRIC_HUMAN Protein LYRIC OS=Homo sapiens OX=9606 GN=MTDH PE=1 SV=2
MAARSWQDELAQQAEEGSARLREMLSVGLGFLRTELGLDLGLEPKRYPGWVILVGTGALG
LLLLFLLGYGWAAACAGARKKRRSPPRKREEAAAVPAAAPDDLALLKNLRSEEQKKKNRK
KLSEKPKPNGRTVEVAEGEAVRTPQSVTAKQPPEIDKKNEKSKKNKKKSKSDAKAVQNSS
RHDGKEVDEGAWETKISHREKRQQRKRDKVLTDSGSLDSTIPGIENTITVTTEQLTTASF
PVGSKKNKGDSHLNVQVSNFKSGKGDSTLQVSSGLNENLTVNGGGWNEKSVKLSSQISAG
EEKWNSVSPASAGKRKTEPSAWSQDTGDANTNGKDWGRSWSDRSIFSGIGSTAEPVSQST
TSDYQWDVSRNQPYIDDEWSGLNGLSSADPNSDWNAPAEEWGNWVDEERASLLKSQEPIP
DDQKVSDDDKEKGEGALPTGKSKKKKKKKKKQGEDNSTAQDTEELEKEIREDLPVNTSKT
RPKQEKAFSLKTISTSDPAEVLVKNSQPIKTLPPATSTEPSVILSKSDSDKSSSQVPPIL
QETDKSKSNTKQNSVPPSQTKSETSWESPKQIKKKKKARRET
>sp|Q86UP2|KTN1_HUMAN Kinectin OS=Homo sapiens OX=9606 GN=KTN1 PE=1 SV=1
MEFYESAYFIVLIPSIVITVIFLFFWLFMKETLYDEVLAKQKREQKLIPTKTDKKKAEKK
KNKKKEIQNGNLHESDSESVPRDFKLSDALAVEDDQVAPVPLNVVETSSSVRERKKKEKK
QKPVLEEQVIKESDASKIPGKKVEPVPVTKQPTPPSEAAASKKKPGQKKSKNGSDDQDKK
VETLMVPSKRQEALPLHQETKQESGSGKKKASSKKQKTENVFVDEPLIHATTYIPLMDNA
DSSPVVDKREVIDLLKPDQVEGIQKSGTKKLKTETDKENAEVKFKDFLLSLKTMMFSEDE
ALCVVDLLKEKSGVIQDALKKSSKGELTTLIHQLQEKDKLLAAVKEDAAATKDRCKQLTQ
EMMTEKERSNVVITRMKDRIGTLEKEHNVFQNKIHVSYQETQQMQMKFQQVREQMEAEIA
HLKQENGILRDAVSNTTNQLESKQSAELNKLRQDYARLVNELTEKTGKLQQEEVQKKNAE
QAATQLKVQLQEAERRWEEVQSYIRKRTAEHEAAQQDLQSKFVAKENEVQSLHSKLTDTL
VSKQQLEQRLMQLMESEQKRVNKEESLQMQVQDILEQNEALKAQIQQFHSQIAAQTSASV
LAEELHKVIAEKDKQIKQTEDSLASERDRLTSKEEELKDIQNMNFLLKAEVQKLQALANE
QAAAAHELEKMQQSVYVKDDKIRLLEEQLQHEISNKMEEFKILNDQNKALKSEVQKLQTL
VSEQPNKDVVEQMEKCIQEKDEKLKTVEELLETGLIQVATKEEELNAIRTENSSLTKEVQ
DLKAKQNDQVSFASLVEELKKVIHEKDGKIKSVEELLEAELLKVANKEKTVQDLKQEIKA
LKEEIGNVQLEKAQQLSITSKVQELQNLLKGKEEQMNTMKAVLEEKEKDLANTGKWLQDL
QEENESLKAHVQEVAQHNLKEASSASQFEELEIVLKEKENELKRLEAMLKERESDLSSKT
QLLQDVQDENKLFKSQIEQLKQQNYQQASSFPPHEELLKVISEREKEISGLWNELDSLKD
AVEHQRKKNNDLREKNWEAMEALASTEKMLQDKVNKTSKERQQQVEAVELEAKEVLKKLF
PKVSVPSNLSYGEWLHGFEKKAKECMAGTSGSEEVKVLEHKLKEADEMHTLLQLECEKYK
SVLAETEGILQKLQRSVEQEENKWKVKVDESHKTIKQMQSSFTSSEQELERLRSENKDIE
NLRREREHLEMELEKAEMERSTYVTEVRELKDLLTELQKKLDDSYSEAVRQNEELNLLKA
QLNETLTKLRTEQNERQKVAGDLHKAQQSLELIQSKIVKAAGDTTVIENSDVSPETESSE
KETMSVSLNQTVTQLQQLLQAVNQQLTKEKEHYQVLE

>sp|Q86UX2|ITIH5_HUMAN Inter-alpha-trypsin inhibitor heavy chain H5 OS=Homo sapiens OX=9606 GN=ITIH5 PE=2 SV=2
MLLLLGLCLGLSLCVGSQEEAQSWGHSSEQDGLRVPRQVRLLQRLKTKPLMTEFSVKSTI
ISRYAFTTVSCRMLNRASEDQDIEFQMQIPAAAFITNFTMLIGDKVYQGEITEREKKSGD
RVKEKRNKTTEENGEKGTEIFRASAVIPSKDKAAFFLSYEELLQRRLGKYEHSISVRPQQ
LSGRLSVDVNILESAGIASLEVLPLHNSRQRGSGRGEDDSGPPPSTVINQNETFANIIFK
PTVVQQARIAQNGILGDFIIRYDVNREQSIGDIQVLNGYFVHYFAPKDLPPLPKNVVFVL
DSSASMVGTKLRQTKDALFTILHDLRPQDRFSIIGFSNRIKVWKDHLISVTPDSIRDGKV
YIHHMSPTGGTDINGALQRAIRLLNKYVAHSGIGDRSVSLIVFLTDGKPTVGETHTLKIL
NNTREAARGQVCIFTIGIGNDVDFRLLEKLSLENCGLTRRVHEEEDAGSQLIGFYDEIRT
PLLSDIRIDYPPSSVVQATKTLFPNYFNGSEIIIAGKLVDRKLDHLHVEVTASNSKKFII
LKTDVPVRPQKAGKDVTGSPRPGGDGEGDTNHIERLWSYLTTKELLSSWLQSDDEPEKER
LRQRAQALAVSYRFLTPFTSMKLRGPVPRMDGLEEAHGMSAAMGPEPVVQSVRGAGTQPG
PLLKKPYQPRIKISKTSVDGDPHFVVDFPLSRLTVCFNIDGQPGDILRLVSDHRDSGVTV
NGELIGAPAPPNGHKKQRTYLRTITILINKPERSYLEITPSRVILDGGDRLVLPCNQSVV
VGSWGLEVSVSANANVTVTIQGSIAFVILIHLYKKPAPFQRHHLGFYIANSEGLSSNCHG
LLGQFLNQDARLTEDPAGPSQNLTHPLLLQVGEGPEAVLTVKGHQVPVVWKQRKIYNGEE
QIDCWFARNNAAKLIDGEYKDYLAFHPFDTGMTLGQGMSREL
>sp|Q86VP6|CAND1_HUMAN Cullin-associated NEDD8-dissociated protein 1 OS=Homo sapiens OX=9606 GN=CAND1 PE=1 SV=2
MASASYHISNLLEKMTSSDKDFRFMATNDLMTELQKDSIKLDDDSERKVVKMILKLLEDK
NGEVQNLAVKCLGPLVSKVKEYQVETIVDTLCTNMLSDKEQLRDISSIGLKTVIGELPPA
SSGSALAANVCKKITGRLTSAIAKQEDVSVQLEALDIMADMLSRQGGLLVNFHPSILTCL
LPQLTSPRLAVRKRTIIALGHLVMSCGNIVFVDLIEHLLSELSKNDSMSTTRTYIQCIAA
ISRQAGHRIGEYLEKIIPLVVKFCNVDDDELREYCIQAFESFVRRCPKEVYPHVSTIINI
CLKYLTYDPNYNYDDEDEDENAMDADGGDDDDQGSDDEYSDDDDMSWKVRRAAAKCLDAV
VSTRHEMLPEFYKTVSPALISRFKEREENVKADVFHAYLSLLKQTRPVQSWLCDPDAMEQ
GETPLTMLQSQVPNIVKALHKQMKEKSVKTRQCCFNMLTELVNVLPGALTQHIPVLVPGI
IFSLNDKSSSSNLKIDALSCLYVILCNHSPQVFHPHVQALVPPVVACVGDPFYKITSEAL
LVTQQLVKVIRPLDQPSSFDATPYIKDLFTCTIKRLKAADIDQEVKERAISCMGQIICNL
GDNLGSDLPNTLQIFLERLKNEITRLTTVKALTLIAGSPLKIDLRPVLGEGVPILASFLR
KNQRALKLGTLSALDILIKNYSDSLTAAMIDAVLDELPPLISESDMHVSQMAISFLTTLA
KVYPSSLSKISGSILNELIGLVRSPLLQGGALSAMLDFFQALVVTGTNNLGYMDLLRMLT
GPVYSQSTALTHKQSYYSIAKCVAALTRACPKEGPAVVGQFIQDVKNSRSTDSIRLLALL
SLGEVGHHIDLSGQLELKSVILEAFSSPSEEVKSAASYALGSISVGNLPEYLPFVLQEIT
SQPKRQYLLLHSLKEIISSASVVGLKPYVENIWALLLKHCECAEEGTRNVVAECLGKLTL
IDPETLLPRLKGYLISGSSYARSSVVTAVKFTISDHPQPIDPLLKNCIGDFLKTLEDPDL
NVRRVALVTFNSAAHNKPSLIRDLLDTVLPHLYNETKVRKELIREVEMGPFKHTVDDGLD
IRKAAFECMYTLLDSCLDRLDIFEFLNHVEDGLKDHYDIKMLTFLMLVRLSTLCPSAVLQ
RLDRLVEPLRATCTTKVKANSVKQEFEKQDELKRSAMRAVAALLTIPEAEKSPLMSEFQS
QISSNPELAAIFESIQKDSSSTNLESMDTS
>sp|Q86X76|NIT1_HUMAN Deaminated glutathione amidase OS=Homo sapiens OX=9606 GN=NIT1 PE=1 SV=2
MLGFITRPPHRFLSLLCPGLRIPQLSVLCAQPRPRAMAISSSSCELPLVAVCQVTSTPDK

QQNFKTCAELVREAARLGACLAFLPEAFDFIARDPAETLHLSEPLGGKLLEEYTQLAREC
GLWLSLGGFHERGQDWEQTQKIYNCHVLLNSKGAVVATYRKTHLCDVEIPGQGPMCESNS
TMPGPSLESPVSTPAGKIGLAVCYDMRFPELSLALAQAGAEILTYPSAFGSITGPAHWEV
LLRARAIETQCYVVAAAQCGRHHEKRASYGHSMVVDPWGTVVARCSEGPGLCLARIDLNY
LRQLRRHLPVFQHRRPDLYGNLGHPLS
>sp|Q86Y39|NDUAB_HUMAN NADH dehydrogenase [ubiquinone] 1 alpha subcomplex subunit 11 OS=Homo sapiens OX=9606 GN=NDUFA11 PE=1 SV=3
MAPKVFRQYWDIPDGTDCHRKAYSTTSIASVAGLTAAAYRVTLNPPGTFLEGVAKVGQYT
FTAAAVGAVFGLTTCISAHVREKPDDPLNYFLGGCAGGLTLGARTHNYGIGAAACVYFGI
AASLVKMGRLEGWEVFAKPKV
>sp|Q86YS6|RAB43_HUMAN Ras-related protein Rab-43 OS=Homo sapiens OX=9606 GN=RAB43 PE=1 SV=1
MAGPGPGPGDPDEQYDFLFKLVLVGDASVGKTCVVQRFKTGAFSERQGSTIGVDFTMKTL
EIQGKRVKLQIWDTAGQERFRTITQSYYRSANGAILAYDITKRSSFLSVPHWIEDVRKYA
GSNIVQLLIGNKSDLSELREVSLAEAQSLAEHYDILCAIETSAKDSSNVEEAFLRVATEL
IMRHGGPLFSEKSPDHIQLNSKDIGEGWGCGC
>sp|Q86YZ3|HORN_HUMAN Hornerin OS=Homo sapiens OX=9606 GN=HRNR PE=1 SV=2
MPKLLQGVITVIDVFYQYATQHGEYDTLNKAELKELLENEFHQILKNPNDPDTVDIILQS
LDRDHNKKVDFTEYLLMIFKLVQARNKIIGKDYCQVSGSKLRDDTHQHQEEQEETEKEEN
KRQESSFSHSSWSAGENDSYSRNVRGSLKPGTESISRRLSFQRDFSGQHNSYSGQSSSYG
EQNSDSHQSSGRGQCGSGSGQSPNYGQHGSGSGQSSSNDTHGSGSGQSSGFSQHKSSSGQ
SSGYSQHGSGSGHSSGYGQHGSRSGQSSRGERHRSSSGSSSSYGQHGSGSRQSLGHGRQG
SGSRQSPSHVRHGSGSGHSSSHGQHGSGSSYSYSRGHYESGSGQTSGFGQHESGSGQSSG
YSKHGSGSGHSSSQGQHGSTSGQASSSGQHGSSSRQSSSYGQHESASRHSSGRGQHSSGS
GQSPGHGQRGSGSGQSPSSGQHGTGFGRSSSSGPYVSGSGYSSGFGHHESSSEHSSGYTQ
HGSGSGHSSGHGQHGSRSGQSSRGERQGSSAGSSSSYGQHGSGSRQSLGHSRHGSGSGQS
PSPSRGRHESGSRQSSSYGPHGYGSGRSSSRGPYESGSGHSSGLGHQESRSGQSSGYGQH
GSSSGHSSTHGQHGSTSGQSSSCGQHGATSGQSSSHGQHGSGSSQSSRYGQQGSGSGQSP
SRGRHGSDFGHSSSYGQHGSGSGWSSSNGPHGSVSGQSSGFGHKSGSGQSSGYSQHGSGS
SHSSGYRKHGSRSGQSSRSEQHGSSSGLSSSYGQHGSGSHQSSGHGRQGSGSGHSPSRVR
HGSSSGHSSSHGQHGSGTSCSSSCGHYESGSGQASGFGQHESGSGQGYSQHGSASGHFSS
QGRHGSTSGQSSSSGQHDSSSGQSSSYGQHESASHHASGRGRHGSGSGQSPGHGQRGSGS
GQSPSYGRHGSGSGRSSSSGRHGSGSGQSSGFGHKSSSGQSSGYTQHGSGSGHSSSYEQH
GSRSGQSSRSEQHGSSSGSSSSYGQHGSGSRQSLGHGQHGSGSGQSPSPSRGRHGSGSGQ
SSSYGPYRSGSGWSSSRGPYESGSGHSSGLGHRESRSGQSSGYGQHGSSSGHSSTHGQHG
STSGQSSSCGQHGASSGQSSSHGQHGSGSSQSSGYGRQGSGSGQSPGHGQRGSGSRQSPS
YGRHGSGSGRSSSSGQHGSGLGESSGFGHHESSSGQSSSYSQHGSGSGHSSGYGQHGSRS
GQSSRGERHGSSSGSSSHYGQHGSGSRQSSGHGRQGSGSGHSPSRGRHGSGLGHSSSHGQ
HGSGSGRSSSRGPYESRSGHSSVFGQHESGSGHSSAYSQHGSGSGHFCSQGQHGSTSGQS
STFDQEGSSTGQSSSYGHRGSGSSQSSGYGRHGAGSGQSPSRGRHGSGSGHSSSYGQHGS
GSGWSSSSGRHGSGSGQSSGFGHHESSSWQSSGCTQHGSGSGHSSSYEQHGSRSGQSSRG
ERHGSSSGSSSSYGQHGSGSRQSLGHGQHGSGSGQSPSPSRGRHGSGSGQSSSYSPYGSG
SGWSSSRGPYESGSSHSSGLGHRESRSGQSSGYGQHGSSSGHSSTHGQHGSTSGQSSSCG
QHGASSGQSSSHGQHGSGSSQSSGYGRQGSGSGQSPGHGQRGSGSRQSPSYGRHGSGSGR

SSSSGQHGSGLGESSGFGHHESSSGQSSSYSQHGSGSGHSSGYGQHGSRSGQSSRGERHG
SSSRSSSRYGQHGSGSRQSSGHGRQGSGSGQSPSRGRHGSGLGHSSSHGQHGSGSGRSSS
RGPYESRSGHSSVFGQHESGSGHSSAYSQHGSGSGHFCSQGQHGSTSGQSSTFDQEGSST
GQSSSHGQHGSGSSQSSSYGQQGSGSGQSPSRGRHGSGSGHSSSYGQHGSGSGWSSSSGR
HGSGSGQSSGFGHHESSSWQSSGYTQHGSGSGHSSSYEQHGSRSGQSSRGEQHGSSSGSS
SSYGQHGSGSRQSLGHGQHGSGSGQSPSPSRGRHGSGSGQSSSYGPYGSGSGWSSSRGPY
ESGSGHSSGLGHRESRSGQSSGYGQHGSSSGHSSTHGQHGSASGQSSSCGQHGASSGQSS
SHGQHGSGSSQSSGYGRQGSGSGQSPGHGQRGSGSRQSPSYGRHGSGSGRSSSSGQHGPG
LGESSGFGHHESSSGQSSSYSQHGSGSGHSSGYGQHGSRSGQSSRGERHGSSSGSSSRYG
QHGSGSRQSSGHGRQGSGSGHSPSRGRHGSGSGHSSSHGQHGSGSGRSSSRGPYESRSGH
SSVFGQHESGSGHSSAYSQHGSGSGHFCSQGQHGSTSGQSSTFDQEGSSTGQSSSHGQHG
SGSSQSSSYGQQGSGSGQSPSRGRHGSGSGHSSSYGQHGSGSGWSSSSGRHGSGSGQSSG
FGHHESSSWQSSGYTQHGSGSGHSSSYEQHGSRSGQSSRGERHGSSSGSSSSYGQHGSGS
RQSLGHGQHGSGSGQSPSPSRGRHGSGSGQSSSYSPYGSGSGWSSSRGPYESGSGHSSGL
GHRESRSGQSSGYGQHGSSSGHSSTHGQHGSTSGQSSSCGQHGASSGQSSSHGQHGSGSS
QSSGYGRQGSGSGQSPGHGQRGSGSRQSPSYGRHGSGSGRSSSSGQHGSGLGESSGFGHH
ESSSGQSSSYSQHGSGSGHSSGYGQHGSRSGQSSRGERHGSSSGSSSHYGQHGSGSRQSS
GHGRQGSGSGQSPSRGRHGSGLGHSSSHGQHGSGSGRSSSRGPYESRLGHSSVFGQHESG
SGHSSAYSQHGSGSGHFCSQGQHGSTSGQSSTFDQEGSSTGQSSSYGHRGSGSSQSSGYG
RHGAGSGQSLSHGRHGSGSGQSSSYGQHGSGSGQSSGYSQHGSGSGQDGYSYCKGGSNHD
GGSSGSYFLSFPSSTSPYEYVQEQRCYFYQ
>sp|Q8EUY3|PYRF_MALP2 Orotidine 5'-phosphate decarboxylase OS=Malacoplasma penetrans (strain HF-2) OX=272633 GN=pyrF PE=3 SV=1
MSNREVIIALDFDSLDKTCKFLNLFENQNLFVKIGMELFYQNGLQVLHEIKNRGHKIFLD
LKLHDIPNTVYHAVKGLMKFDVDIITVHCAGGLTMLNQAKQAVFDQNKNTKIIGITQLTS
TSETEMQTQQKISTSLQDSVLNYAKLAKEANIDGVVSSVWETKKIKEQNGNNFLVINPGI
RLEKDDSGDQKRVASPLDAKNQLADFIVVGRPITKDNNPLEKYLEIKRMFV
>sp|Q8IVW8|SPNS2_HUMAN Sphingosine-1-phosphate transporter SPNS2 OS=Homo sapiens OX=9606 GN=SPNS2 PE=1 SV=2
MMCLECASAAAGGAEEEEADAERRRRRRGAQRGAGGSGCCGARGAGGAGVSAAGDEVQTL
SGSVRRAPTGPPGTPGTPGCAATAKGPGAQQPKPASLGRGRGAAAAILSLGNVLNYLDRY
TVAGVLLDIQQHFGVKDRGAGLLQSVFICSFMVAAPIFGYLGDRFNRKVILSCGIFFWSA
VTFSSSFIPQQYFWLLVLSRGLVGIGEASYSTIAPTIIGDLFTKNTRTLMLSVFYFAIPL
GSGLGYITGSSVKQAAGDWHWALRVSPVLGMITGTLILILVPATKRGHADQLGDQLKART
SWLRDMKALIRNRSYVFSSLATSAVSFATGALGMWIPLYLHRAQVVQKTAETCNSPPCGA
KDSLIFGAITCFTGFLGVVTGAGATRWCRLKTQRADPLVCAVGMLGSAIFICLIFVAAKS
SIVGAYICIFVGETLLFSNWAITADILMYVVIPTRRATAVALQSFTSHLLGDAGSPYLIG
FISDLIRQSTKDSPLWEFLSLGYALMLCPFVVVLGGMFFLATALFFVSDRARAEQQVNQL
AMPPASVKV
>sp|Q8IW45|NNRD_HUMAN ATP-dependent (S)-NAD(P)H-hydrate dehydratase OS=Homo sapiens OX=9606 GN=NAXD PE=1 SV=1
MVTRAGAGTAVAGAVVVALLSAALALYGPPLDAVLERAFSLRKAHSIKDMENTLQLVRNI
IPPLSSTKHKGQDGRIGVVGGCQEYTGAPYFAAISALKVGADLSHVFCASAAAPVIKAYS
PELIVHPVLDSPNAVHEVEKWLPRLHALVVGPGLGRDDALLRNVQGILEVSKARDIPVVI

DADGLWLVAQQPALIHGYRKAVLTPNHVEFSRLYDAVLRGPMDSDDSHGSVLRLSQALGN
VTVVQKGERDILSNGQQVLVCSQEGSSRRCGGQGDLLSGSLGVLVHWALLAGPQKTNGSS
PLLVAAFGACSLTRQCNHQAFQKHGRSTTTSDMIAEVGAAFSKLFET
>sp|Q8IWA5|CTL2_HUMAN Choline transporter-like protein 2 OS=Homo sapiens OX=9606
GN=SLC44A2 PE=1 SV=3
MGDERPHYYGKHGTPQKYDPTFKGPIYNRGCTDIICCVFLLLAIVGYVAVGIIAWTHGDP
RKVIYPTDSRGEFCGQKGTKNENKPYLFYFNIVKCASPLVLLEFQCPTPQICVEKCPDRY
LTYLNARSSRDFEYYKQFCVPGFKNNKGVAEVLQDGDCPAVLIPSKPLARRCFPAIHAYK
GVLMVGNETTYEDGHGSRKNITDLVEGAKKANGVLEARQLAMRIFEDYTVSWYWIIIGLV
IAMAMSLLFIILLRFLAGIMVWVMIIMVILVLGYGIFHCYMEYSRLRGEAGSDVSLVDLG
FQTDFRVYLHLRQTWLAFMIILSILEVIIILLLIFLRKRILIAIALIKEASRAVGYVMCS
LLYPLVTFFLLCLCIAYWASTAVFLSTSNEAVYKIFDDSPCPFTAKTCNPETFPSSNESR
QCPNARCQFAFYGGESGYHRALLGLQIFNAFMFFWLANFVLALGQVTLAGAFASYYWALR
KPDDLPAFPLFSAFGRALRYHTGSLAFGALILAIVQIIRVILEYLDQRLKAAENKFAKCL
MTCLKCCFWCLEKFIKFLNRNAYIMIAIYGTNFCTSARNAFFLLMRNIIRVAVLDKVTDF
LFLLGKLLIVGSVGILAFFFFTHRIRIVQDTAPPLNYYWVPILTVIVGSYLIAHGFFSVY
GMCVDTLFLCFLEDLERNDGSAERPYFMSSTLKKLLNKTNKKAAES
>sp|Q8IY17|PLPL6_HUMAN Patatin-like phospholipase domain-containing protein 6 OS=Homo
sapiens OX=9606 GN=PNPLA6 PE=1 SV=3
MEAPLQTGMMGTSSHGLATNSSGAKVAERDGFQDVLAPGEGSAGRICGAQPVPFVPQVLG
VMIGAGVAVVVTAVLILLVVRRLRVPKTPAPDGPRYRFRKRDKVLFYGRKIMRKVSQSTS
SLVDTSVSATSRPRMRKKLKMLNIAKKILRIQKETPTLQRKEPPPAVLEADLTEGDLANS
HLPSEVLYMLKNVRVLGHFEKPLFLELCRHMVFQRLGQGDYVFRPGQPDASIYVVQDGLL
ELCLPGPDGKECVVKEVVPGDSVNSLLSILDVITGHQHPQRTVSARAARDSTVLRLPVEA
FSAVFTKYPESLVRVVQIIMVRLQRVTFLALHNYLGLTNELFSHEIQPLRLFPSPGLPTR
TSPVRGSKRMVSTSATDEPRETPGRPPDPTGAPLPGPTGDPVKPTSLETPSAPLLSRCVS
MPGDISGLQGGPRSDFDMAYERGRISVSLQEEASGGSLAAPARTPTQEPREQPAGACEYS
YCEDESATGGCPFGPYQGRQTSSIFEAAKQELAKLMRIEDPSLLNSRVLLHHAKAGTIIA
RQGDQDVSLHFVLWGCLHVYQRMIDKAEDVCLFVAQPGELVGQLAVLTGEPLIFTLRAQR
DCTFLRISKSDFYEIMRAQPSVVLSAAHTVAARMSPFVRQMDFAIDWTAVEAGRALYRQG
DRSDCTYIVLNGRLRSVIQRGSGKKELVGEYGRGDLIGVVEALTRQPRATTVHAVRDTEL
AKLPEGTLGHIKRRYPQVVTRLIHLLSQKILGNLQQLQGPFPAGSGLGVPPHSELTNPAS
NLATVAILPVCAEVPMVAFTLELQHALQAIGPTLLLNSDIIRARLGASALDSIQEFRLSG
WLAQQEDAHRIVLYQTDASLTPWTVRCLRQADCILIVGLGDQEPTLGQLEQMLENTAVRA
LKQLVLLHREEGAGPTRTVEWLNMRSWCSGHLHLRCPRRLFSRRSPAKLHELYEKVFSRR
ADRHSDFSRLARVLTGNTIALVLGGGGARGCSHIGVLKALEEAGVPVDLVGGTSIGSFIG
ALYAEERSASRTKQRAREWAKSMTSVLEPVLDLTYPVTSMFTGSAFNRSIHRVFQDKQIE
DLWLPYFNVTTDITASAMRVHKDGSLWRYVRASMTLSGYLPPLCDPKDGHLLMDGGYINN
LPADIARSMGAKTVIAIDVGSQDETDLSTYGDSLSGWWLLWKRLNPWADKVKVPDMAEIQ
SRLAYVSCVRQLEVVKSSSYCEYLRPPIDCFKTMDFGKFDQIYDVGYQYGKAVFGGWSRG
NVIEKMLTDRRSTDLNESRRADVLAFPSSGFTDLAEIVSRIEPPTSYVSDGCADGEESDC
LTEYEEDAGPDCSRDEGGSPEGASPSTASEMEEEKSILRQRRCLPQEPPGSATDA
>sp|Q8IYS4|DAAF8_HUMAN Dynein axonemal assembly factor 8 OS=Homo sapiens OX=9606
GN=DNAAF8 PE=1 SV=3

MASNDKGMAPSLGSPWASQMGPWDAILKAVKDQLPSLDSDSPLSDYGEEELFIFQRNQTS
LIPDLSEELAEDPADGDKSRAWVAAAEESLPEPVLVPAELATEPGCRQNTRTKDASSQEG
RDPGRPFESSGEVSALLGMAEEPPRWLEGDLGSLSFNTKGSQGPPWDPQAEATLSCHEGD
PKAEPLSTASQESVNRRALRQERRKMIETDILQKVTRDACGPTSSDKGGVKEAPCHAAES
APRSKMPLVEPPEGPPVLSLQQLEAWDLDDILQSLAGQEDNQGNRAPGTVWWAADHRQVQ
DRMVPSAHNRLMEQLALLCTTQSKASACARKVPADTPQDTKEADSGSRCASRKQGSQAGP
GPQLAQGMRLNAESPTIFIDLRQMELPDHLSPESSSHSSSDSEEEEEEEMAALGDAEGAS
PSSLGLRTCTGKSQLLQQLRAFQKGTAQPELPASKGPAGGRAQAPEDTAGSRTGRKQHMK
LCAKGQSAQARLPRGRPRALGDVPEPGAAREALMPPLEQL
>sp|Q8IZ83|A16A1_HUMAN Aldehyde dehydrogenase family 16 member A1 OS=Homo sapiens OX=9606 GN=ALDH16A1 PE=1 SV=2
MAATRAGPRAREIFTSLEYGPVPESHACALAWLDTQDRCLGHYVNGKWLKPEHRNSVPCQ
DPITGENLASCLQAQAEDVAAAVEAARMAFKGWSAHPGVVRAQHLTRLAEVIQKHQRLLW
TLESLVTGRAVREVRDGDVQLAQQLLHYHAIQASTQEEALAGWEPMGVIGLILPPTFSFL
EMMWRICPALAVGCTVVALVPPASPAPLLLAQLAGELGPFPGILNVLSGPASLVPILASQ
PGIRKVAFCGAPEEGRALRRSLAGECAELGLALGTESLLLLTDTADVDSAVEGVVDAAWS
DRGPGGLRLLIQESVWDEAMRRLQERMGRLRSGRGLDGAVDMGARGAAACDLVQRFVREA
QSQGAQVFQAGDVPSERPFYPPTLVSNLPPASPCAQVEVPWPVVVASPFRTAKEALLVAN
GTPRGGSASVWSERLGQALELGYGLQVGTVWINAHGLRDPSVPTGGCKESGCSWHGGPDG
LYEYLRPSGTPARLSCLSKNLNYDTFGLAVPSTLPAGPEIGPSPAPPYGLFVGGRFQAPG
ARSSRPIRDSSGNLHGYVAEGGAKDIRGAVEAAHQAFPGWAGQSPGARAALLWALAAALE
RRKSTLASRLERQGAELKAAEAEVELSARRLRAWGARVQAQGHTLQVAGLRGPVLRLREP
LGVLAVVCPDEWPLLAFVSLLAPALAYGNTVVMVPSAACPLLALEVCQDMATVFPAGLAN
VVTGDRDHLTRCLALHQDVQAMWYFGSAQGSQFVEWASAGNLKPVWASRGCPRAWDQEAE
GAGPELGLRVARTKALWLPMGD
>sp|Q8IZT6|ASPM_HUMAN Abnormal spindle-like microcephaly-associated protein OS=Homo sapiens OX=9606 GN=ASPM PE=1 SV=2
MANRRVGRGCWEVSPTERRPPAGLRGPAAEEEASSPPVLSLSHFCRSPFLCFGDVLLGAS
RTLSLALDNPNEEVAEVKISHFPAADLGFSVSQRCFVLQPKEKIVISVNWTPLKEGRVRE
IMTFLVNDVLKHQAILLGNAEEQKKKKRSLWDTIKKKKISASTSHNRRVSNIQNVNKTFS
VSQKVDRVRSPLQACENLAMNEGGPPTENNSLILEENKIPISPISPAFNECHGATCLPLS
VRRSTTYSSLHASENRELLNVHSANVSKVSFNEKAVTETSFNSVNVNGQRGENSKLSLTP
NCSSTLNITQSQIHFLSPDSFVNNSHGANNELELVTCLSSDMFMKDNSQPVHLESTIAHE
IYQKILSPDSFIKDNYGLNQDLESESVNPILSPNQFLKDNMAYMCTSQQTCKVPLSNENS
QVPQSPEDWRKSEVSPRIPECQGSKSPKAIFEELVEMKSNYYSFIKQNNPKFSAVQDISS
HSHNKQPKRRPILSATVTKRKATCTRENQTEINKPKAKRCLNSAVGEHEKVINNQKEKED
FHSYLPIIDPILSKSKSYKNEVTPSSTTASVARKRKSDGSMEDANVRVAITEHTEVREIK
RIHFSPSEPKTSAVKKTKNVTTPISKRISNREKLNLKKKTDLSIFRTPISKTNKRTKPII
AVAQSSLTFIKPLKTDIPRHPMPFAAKNMFYDERWKEKQEQGFTWWLNFILTPDDFTVKT
NISEVNAATLLLGIENQHKISVPRAPTKEEMSLRAYTARCRLNRLRRAACRLFTSEKMVK
AIKKLEIEIEARRLIVRKDRHLWKDVGERQKVLNWLLSYNPLWLRIGLETTYGELISLED
NSDVTGLAMFILNRLLWNPDIAAEYRHPTVPHLYRDGHEEALSKFTLKKLLLLVCFLDYA
KISRLIDHDPCLFCKDAEFKASKEILLAFSRDFLSGEGDLSRHLGLLGLPVNHVQTPFDE
FDFAVTNLAVDLQCGVRLVRTMELLTQNWDLSKKLRIPAISRLQKMHNVDIVLQVLKSRG

IELSDEHGNTILSKDIVDRHREKTLRLLWKIAFAFQVDISLNLDQLKEEIAFLKHTKSIK
KTISLLSCHSDDLINKKKGKRDSGSFEQYSENIKLLMDWVNAVCAFYNKKVENFTVSFSD
GRVLCYLIHHYHPCYVPFDAICQRTTQTVECTQTGSVVLNSSSESDDSSLDMSLKAFDHE
NTSELYKELLENEKKNFHLVRSAVRDLGGIPAMINHSDMSNTIPDEKVVITYLSFLCARL
LDLRKEIRAARLIQTTWRKYKLKTDLKRHQEREKAARIIQLAVINFLAKQRLRKRVNAAL
VIQKYWRRVLAQRKLLMLKKEKLEKVQNKAASLIQGYWRRYSTRQRFLKLKYYSIILQSR
IRMIIAVTSYKRYLWATVTIQRHWRAYLRRKQDQQRYEMLKSSTLIIQSMFRKWKQRKMQ
SQVKATVILQRAFREWHLRKQAKEENSAIIIQSWYRMHKELRKYIYIRSCVVIIQKRFRC
FQAQKLYKRRKESILTIQKYYKAYLKGKIERTNYLQKRAAAIQLQAAFRRLKAHNLCRQI
RAACVIQSYWRMRQDRVRFLNLKKTIIKFQAHVRKHQQRQKYKKMKKAAVIIQTHFRAYI
FAMKVLASYQKTRSAVIVLQSAYRGMQARKMYIHILTSVIKIQSYYRAYVSKKEFLSLKN
ATIKLQSTVKMKQTRKQYLHLRAAALFIQQCYRSKKIAAQKREEYMQMRESCIKLQAFVR
GYLVRKQMRLQRKAVISLQSYFRMRKARQYYLKMYKAIIVIQNYYHAYKAQVNQRKNFLQ
VKKAATCLQAAYRGYKVRQLIKQQSIAALKIQSAFRGYNKRVKYQSVLQSIIKIQRWYRA
YKTLHDTRTHFLKTKAAVISLQSAYRGWKVRKQIRREHQAALKIQSAFRMAKAQKQFRLF
KTAALVIQQNFRAWTAGRKQCMEYIELRHAVLVLQSMWKGKTLRRQLQRQHKCAIIIQSY
YRMHVQQKKWKIMKKAALLIQKYYRAYSIGREQNHLYLKTKAAVVTLQSAYRGMKVRKRI
KDCNKAAVTIQSKYRAYKTKKKYATYRASAIIIQRWYRGIKITNHQHKEYLNLKKTAIKI
QSVYRGIRVRRHIQHMHRAATFIKAMFKMHQSRISYHTMRKAAIVIQVRCRAYYQGKMQR
EKYLTILKAVKVLQASFRGVRVRRTLRKMQTAATLIQSNYRRYRQQTYFNKLKKITKTVQ
QRYWAMKERNIQFQRYNKLRHSVIYIQAIFRGKKARRHLKMMHIAATLIQRRFRTLMMRR
RFLSLKKTAILIQRKYRAHLCTKHHLQFLQVQNAVIKIQSSYRRWMIRKRMREMHRAATF
IQSTFRMHRLHMRYQALKQASVVIQQQYQANRAAKLQRQHYLRQRHSAVILQAAFRGMKT
RRHLKSMHSSATLIQSRFRSLLVRRRFISLKKATIFVQRKYRATICAKHKLYQFLHLRKA
AITIQSSYRRLMVKKKLQEMQRAAVLIQATFRMYRTYITFQTWKHASILIQQHYRTYRAA
KLQRENYIRQWHSAVVIQAAYKGMKARQLLREKHKASIVIQSTYRMYRQYCFYQKLQWAT
KIIQEKYRANKKKQKVFQHNELKKETCVQAGFQDMNIKKQIQEQHQAAIIIQKHCKAFKI
RKHYLHLRATVVSIQRRYRKLTAVRTQAVICIQSYYRGFKVRKDIQNMHRAATLIQSFYR
MHRAKVDYETKKTAIVVIQNYYRLYVRVKTERKNFLAVQKSVRTIQAAFRGMKVRQKLKN
VSEEKMAAIVNQSALCCYRSKTQYEAVQSEGVMIQEWYKASGLACSQEAEYHSQSRAAVT
IQKAFCRMVTRKLETQKCAALRIQFFLQMAVYRRRFVQQKRAAITLQHYFRTWQTRKQFL
LYRKAAVVLQNHYRAFLSAKHQRQVYLQIRSSVIIIQARSKGFIQKRKFQEIKNSTIKIQ
AMWRRYRAKKYLCKVKAACKIQAWYRCWRAHKEYLAILKAVKIIQGCFYTKLERTRFLNV
RASAIIIQRKWRAILPAKIAHEHFLMIKRHRAACLIQAHYRGYKGRQVFLRQKSAALIIQ
KYIRAREAGKHERIKYIEFKKSTVILQALVRGWLVRKRFLEQRAKIRLLHFTAAAYYHLN
AVRIQRAYKLYLAVKNANKQVNSVICIQRWFRARLQEKRFIQKYHSIKKIEHEGQECLSQ
RNRAASVIQKAVRHFLLRKKQEKFTSGIIKIQALWRGYSWRKKNDCTKIKAIRLSLQVVN
REIREENKLYKRTALALHYLLTYKHLSAILEALKHLEVVTRLSPLCCENMAQSGAISKIF
VLIRSCNRSIPCMEVIRYAVQVLLNVSKYEKTTSAVYDVENCIDILLELLQIYREKPGNK
VADKGGSIFTKTCCLLAILLKTTNRASDVRSRSKVVDRIYSLYKLTAHKHKMNTERILYK
QKKNSSISIPFIPETPVRTRIVSRLKPDWVLRRDNMEEITNPLQAIQMVMDTLGIPY
>sp|Q8N0W3|FCSK_HUMAN L-fucose kinase OS=Homo sapiens OX=9606 GN=FCSK PE=1 SV=2
MEQPKGVDWTVIILTCQYKDSVQVFQRELEVRQKREQIPAGTLLLAVEDPEKRVGSGGAT
LNALLVAAEHLSARAGFTVVTSDVLHSAWILILHMGRDFPFDDCGRAFTCLPVENPEAPV

EALVCNLDCLLDIMTYRLGPGSPPGVWVCSTDMLLSVPANPGISWDSFRGARVIALPGSP
AYAQNHGVYLTDPQGLVLDIYYQGTEAEIQRCVRPDGRVPLVSGVVFFSVETAERLLATH
VSPPLDACTYLGLDSGARPVQLSLFFDILHCMAENVTREDFLVGRPPELGQGDADVAGYL
QSARAQLWRELRDQPLTMAYVSSGSYSYMTSSASEFLLSLTLPGAPGAQIVHSQVEEQQL
LAAGSSVVSCLLEGPVQLGPGSVLQHCHLQGPIHIGAGCLVTGLDTAHSKALHGRELRDL
VLQGHHTRLHGSPGHAFTLVGRLDSWERQGAGTYLNVPWSEFFKRTGVRAWDLWDPETLP
AEYCLPSARLFPVLHPSRELGPQDLLWMLDHQEDGGEALRAWRASWRLSWEQLQPCLDRA
ATLASRRDLFFRQALHKARHVLEARQDLSLRPLIWAAVREGCPGPLLATLDQVAAGAGDP
GVAARALACVADVLGCMAEGRGGLRSGPAANPEWMRPFSYLECGDLAAGVEALAQERDKW
LSRPALLVRAARHYEGAGQILIRQAVMSAQHFVSTEQVELPGPGQWVVAECPARVDFSGG
WSDTPPLAYELGGAVLGLAVRVDGRRPIGARARRIPEPELWLAVGPRQDEMTVKIVCRCL
ADLRDYCQPHAPGALLKAAFICAGIVHVHSELQLSEQLLRTFGGGFELHTWSELPHGSGL
GTSSILAGTALAALQRAAGRVVGTEALIHAVLHLEQVLTTGGGWQDQVGGLMPGIKVGRS
RAQLPLKVEVEEVTVPEGFVQKLNDHLLLVYTGKTRLARNLLQDVLRSWYARLPAVVQNA
HSLVRQTEECAEGFRQGSLPLLGQCLTSYWEQKKLMAPGCEPLTVRRMMDVLAPHVHGQS
LAGAGGGGFLYLLTKEPQQKEALEAVLAKTEGLGNYSIHLVEVDTQGLSLKLLGTEASTC
CPFP
>sp|Q8N126|CADM3_HUMAN Cell adhesion molecule 3 OS=Homo sapiens OX=9606
GN=CADM3 PE=1 SV=1
MGAPAASLLLLLLLFACCWAPGGANLSQDDSQPWTSDETVVAGGTVVLKCQVKDHEDSSL
QWSNPAQQTLYFGEKRALRDNRIQLVTSTPHELSISISNVALADEGEYTCSIFTMPVRTA
KSLVTVLGIPQKPIITGYKSSLREKDTATLNCQSSGSKPAARLTWRKGDQELHGEPTRIQ
EDPNGKTFTVSSSVTFQVTREDDGASIVCSVNHESLKGADRSTSQRIEVLYTPTAMIRPD
PPHPREGQKLLLHCEGRGNPVPQQYLWEKEGSVPPLKMTQESALIFPFLNKSDSGTYGCT
ATSNMGSYKAYYTLNVNDPSPVPSSSSTYHAIIGGIVAFIVFLLLIMLIFLGHYLIRHKG
TYLTHEAKGSDDAPDADTAIINAEGGQSGGDDKKEYFI
>sp|Q8N196|SIX5_HUMAN Homeobox protein SIX5 OS=Homo sapiens OX=9606 GN=SIX5 PE=1
SV=3
MATLPAEPSAGPAAGGEAVAAAAATEEEEEEARQLLQTLQAAEGEAAAAAGAGAGAAAAG
AEGPGSPGVPGSPPEAASEPPTGLRFSPEQVACVCEALLQAGHAGRLSRFLGALPPAERL
RGSDPVLRARALVAFQRGEYAELYRLLESRPFPAAHHAFLQDLYLRARYHEAERARGRAL
GAVDKYRLRKKFPLPKTIWDGEETVYCFKERSRAALKACYRGNRYPTPDEKRRLATLTGL
SLTQVSNWFKNRRQRDRTGAGGGAPCKSESDGNPTTEDESSRSPEDLERGAAPVSAEAAA
QGSIFLAGTGPPAPCPASSSILVNGSFLAASGSPAVLLNGGPVIINGLALGEASSLGPLL
LTGGGGAPPPQPSPQGASETKTSLVLDPQTGEVRLEEAQSEAPETKGAQVAAPGPALGEE
VLGPLAQVVPGPPTAATFPLPPGPVPAVAAPQVVPLSPPPGYPTGLSPTSPLLNLPQVVP
TSQVVTLPQAVGPLQLLAAGPGSPVKVAAAAGPANVHLINSGVGVTALQLPSATAPGNFL
LANPVSGSPIVTGVALQQGKIILTATFPTSMLVSQVLPPAPGLALPLKPETAISVPEGGL
PVAPSPALPEAHALGTLSAQQPPPAAATTSSTSLPFSPDSPGLLPNFPAPPPEGLMLSPA
AVPVWSAGLELSAGTEGLLEAEKGLGTQAPHTVLRLPDPDPEGLLLGATAGGEVDEGLEA
EAKVLTQLQSVPVEEPLEL
>sp|Q8N2K0|ABD12_HUMAN Lysophosphatidylserine lipase ABHD12 OS=Homo sapiens
OX=9606 GN=ABHD12 PE=1 SV=2
MRKRTEPVALEHERCAAAGSSSSGSAAAALDADCRLKQNLRLTGPAAAEPRCAADAGMKR

ALGRRKGVWLRLRKILFCVLGLYIAIPFLIKLCPGIQAKLIFLNFVRVPYFIDLKKPQDQ
GLNHTCNYYLQPEEDVTIGVWHTVPAVWWKNAQGKDQMWYEDALASSHPIILYLHGNAGT
RGGDHRVELYKVLSSLGYHVVTFDYRGWGDSVGTPSERGMTYDALHVFDWIKARSGDNPV
YIWGHSLGTGVATNLVRRLCERETPPDALILESPFTNIREEAKSHPFSVIYRYFPGFDWF
FLDPITSSGIKFANDENVKHISCPLLILHAEDDPVVPFQLGRKLYSIAAPARSFRDFKVQ
FVPFHSDLGYRHKYIYKSPELPRILREFLGKSEPEHQH
>sp|Q8N335|GPD1L_HUMAN Glycerol-3-phosphate dehydrogenase 1-like protein OS=Homo sapiens OX=9606 GN=GPD1L PE=1 SV=1
MAAAPLKVCIVGSGNWGSAVAKIIGNNVKKLQKFASTVKMWVFEETVNGRKLTDIINNDH
ENVKYLPGHKLPENVVAMSNLSEAVQDADLLVFVIPHQFIHRICDEITGRVPKKALGITL
IKGIDEGPEGLKLISDIIREKMGIDISVLMGANIANEVAAEKFCETTIGSKVMENGLLFK
ELLQTPNFRITVVDDADTVELCGALKNIVAVGAGFCDGLRCGDNTKAAVIRLGLMEMIAF
ARIFCKGQVSTATFLESCGVADLITTCYGGRNRRVAEAFARTGKTIEELEKEMLNGQKLQ
GPQTSAEVYRILKQKGLLDKFPLFTAVYQICYESRPVQEMLSCLQSHPEHT
>sp|Q8N3E9|PLCD3_HUMAN 1-phosphatidylinositol 4,5-bisphosphate phosphodiesterase delta-3 OS=Homo sapiens OX=9606 GN=PLCD3 PE=1 SV=3
MLCGRWRRCRRPPEEPPVAAQVAAQVAAPVALPSPPTPSDGGTKRPGLRALKKMGLTEDE
DVRAMLRGSRLRKIRSRTWHKERLYRLQEDGLSVWFQRRIPRAPSQHIFFVQHIEAVREG
HQSEGLRRFGGAFAPARCLTIAFKGRRKNLDLAAPTAEEAQRWVRGLTKLRARLDAMSQR
ERLDHWIHSYLHRADSNQDSKMSFKEIKSLLRMVNVDMNDMYAYLLFKECDHSNNDRLEG
AEIEEFLRRLLKRPELEEIFHQYSGEDRVLSAPELLEFLEDQGEEGATLARAQQLIQTYE
LNETAKQHELMTLDGFMMYLLSPEGAALDNTHTCVFQDMNQPLAHYFISSSHNTYLTDSQ
IGGPSSTEAYVRAFAQGCRCVELDCWEGPGGEPVIYHGHTLTSKILFRDVVQAVRDHAFT
LSPYPVILSLENHCGLEQQAAMARHLCTILGDMLVTQALDSPNPEELPSPEQLKGRVLVK
GKKLPAARSEDGRALSDREEEEEDDEEEEEEVEAAAQRRLAKQISPELSALAVYCHATRL
RTLHPAPNAPQPCQVSSLSERKAKKLIREAGNSFVRHNARQLTRVYPLGLRMNSANYSPQ
EMWNSGCQLVALNFQTPGYEMDLNAGRFLVNGQCGYVLKPACLRQPDSTFDPEYPGPPRT
TLSIQVLTAQQLPKLNAEKPHSIVDPLVRIEIHGVPADCARQETDYVLNNGFNPRWGQTL
QFQLRAPELALVRFVVEDYDATSPNDFVGQFTLPLSSLKQGYRHIHLLSKDGASLSPATL
FIQIRIQRS
>sp|Q8N3J6|CADM2_HUMAN Cell adhesion molecule 2 OS=Homo sapiens OX=9606 GN=CADM2 PE=2 SV=1
MIWKRSAVLRFYSVCGLLLQGSQGQFPLTQNVTVVEGGTAILTCRVDQNDNTSLQWSNPA
QQTLYFDDKKALRDNRIELVRASWHELSISVSDVSLSDEGQYTCSLFTMPVKTSKAYLTV
LGVPEKPQISGFSSPVMEGDLMQLTCKTSGSKPAADIRWFKNDKEIKDVKYLKEEDANRK
TFTVSSTLDFRVDRSDDGVAVICRVDHESLNATPQVAMQVLEIHYTPSVKIIPSTPFPQE
GQPLILTCESKGKPLPEPVLWTKDGGELPDPDRMVVSGRELNILFLNKTDNGTYRCEATN
TIGQSSAEYVLIVHDVPNTLLPTTIIPSLTTATVTTTVAITTSPTTSATTSSIRDPNALA
GQNGPDHALIGGIVAVVVFVTLCSIFLLGRYLARHKGTYLTNEAKGAEDAPDADTAIINA
EGSQVNAEEKKEYFI
>sp|Q8N5V2|NGEF_HUMAN Ephexin-1 OS=Homo sapiens OX=9606 GN=NGEF PE=1 SV=2
METRESEDLEKTRRKSASDQWNTDNEPAKVKPELLPEKEETSQADQDIQDKEPHCHIPIK
RNSIFNRSIRRKSKAKARDNPERNASCLADSQDNGKSVNEPLTLNIPWSRMPPCRTAMQT
DPGAQEMSESSSTPGNGATPEEWPALADSPTTLTEALRMIHPIPADSWRNLIEQIGLLYQ

EYRDKSTLQEIETRRQQDAEIEDNTNGSPASEDTPEEEEEEEEEEEPASPPERKTLPQIC
LLSNPHSRFNLWQDLPEIRSSGVLEILQPEEIKLQEAMFELVTSEASYYKSLNLLVSHFM
ENERIRKILHPSEAHILFSNVLDVLAVSERFLLELEHRMEENIVISDVCDIVYRYAADHF
SVYITYVSNQTYQERTYKQLLQEKAAFRELIAQLELDPKCRGLPFSSFLILPFQRITRLK
LLVQNILKRVEERSERECTALDAHKELEMVVKACNEGVRKMSRTEQMISIQKKMEFKIKS
VPIISHSRWLLKQGELQQMSGPKTSRTLRTKKLFHEIYLFLFNDLLVICRQIPGDKYQVF
DSAPRGLLRVEELEDQGQTLANVFILRLLENADDREATYMLKASSQSEMKRWMTSLAPNR
RTKFVSFTSRLLDCPQVQCVHPYVAQQPDELTLELADILNILDKTDDGWIFGERLHDQER
GWFPSSMTEEILNPKIRSQNLKECFRVHKMDDPQRSQNKDRRKLGSRNRQ
>sp|Q8N7G0|PO5F2_HUMAN POU domain, class 5, transcription factor 2 OS=Homo sapiens OX=9606 GN=POU5F2 PE=1 SV=1
MAGHRPSNHFCPLPGSGGGGPRGPMPLRVDTLTWLSTQAAPGRVMVWPAVRPGICPGPDV
WRIPLGPLPHEFRGWIAPCRPRLGASEAGDWLRRPSEGALPGPYIALRSIPKLPPPEDIS
GILKELQQLAKELRQKRLSLGYSQADVGIAVGALFGKVLSQTTICRFEAQQLSVANMWKL
RPLLKKWLKEVEAENLLGLCKMEMILQQSGKWRRASRERRIGNSLEKFFQRCPKPTPQQI
SHIAGCLQLQKDVVRVWFYNRSKMGSRPTNDASPREIVGTAGPPCPGAPVCFHLGLGLPV
DIPHYTRLYSAGVAHSSAPATTLGLLRF
>sp|Q8N8N7|PTGR2_HUMAN Prostaglandin reductase 2 OS=Homo sapiens OX=9606 GN=PTGR2 PE=1 SV=1
MIVQRVVLNSRPGKNGNPVAENFRMEEVYLPDNINEGQVQVRTLYLSVDPYMRCRMNEDT
GTDYITPWQLSQVVDGGGIGIIEESKHTNLTKGDFVTSFYWPWQTKVILDGNSLEKVDPQ
LVDGHLSYFLGAIGMPGLTSLIGIQEKGHITAGSNKTMVVSGAAGACGSVAGQIGHFLGC
SRVVGICGTHEKCILLTSELGFDAAINYKKDNVAEQLRESCPAGVDVYFDNVGGNISDTV
ISQMNENSHIILCGQISQYNKDVPYPPPLSPAIEAIQKERNITRERFLVLNYKDKFEPGI
LQLSQWFKEGKLKIKETVINGLENMGAAFQSMMTGGNIGKQIVCISEEISL
>sp|Q8NC56|LEMD2_HUMAN LEM domain-containing protein 2 OS=Homo sapiens OX=9606 GN=LEMD2 PE=1 SV=1
MAGLSDLELRRELQALGFQPGPITDTTRDVYRNKLRRLRGEARLRDEERLREEARPRGEE
RLREEARLREDAPLRARPAAASPRAEPWLSQPASGSAYATPGAYGDIRPSAASWVGSRGL
AYPARPAQLRRRASVRGSSEEDEDARTPDRATQGPGLAARRWWAASPAPARLPSSLLGPD
PRPGLRATRAGPAGAARARPEVGRRLERWLSRLLLWASLGLLLVFLGILWVKMGKPSAPQ
EAEDNMKLLPVDCERKTDEFCQAKQKAALLELLHELYNFLAIQAGNFECGNPENLKSKCI
PVMEAQEYIANVTSSSSAKFEAALTWILSSNKDVGIWLKGEDQSELVTTVDKVVCLESAH
PRMGVGCRLSRALLTAVTNVLIFFWCLAFLWGLLILLKYRWRKLEEEEQAMYEMVKKIID
VVQDHYVDWEQDMERYPYVGILHVRDSLIPPQSRRRMKRVWDRAVEFLASNESRIQTESH
RVAGEDMLVWRWTKPSSFSDSER
>sp|Q8NCW5|NNRE_HUMAN NAD(P)H-hydrate epimerase OS=Homo sapiens OX=9606 GN=NAXE PE=1 SV=2
MSRLRALLGLGLLVAGSRVPRIKSQTIACRSGPTWWGPQRLNSGGRWDSEVMASTVVKYL
SQEEAQAVDQELFNEYQFSVDQLMELAGLSCATAIAKAYPPTSMSRSPPTVLVICGPGNN
GGDGLVCARHLKLFGYEPTIYYPKRPNKPLFTALVTQCQKMDIPFLGEMPAEPMTIDELY
ELVVDAIFGFSFKGDVREPFHSILSVLKGLTVPIASIDIPSGWDVEKGNAGGIQPDLLIS
LTAPKKSATQFTGRYHYLGGRFVPPALEKKYQLNLPPYPDTECVYRLQ
>sp|Q8NF91|SYNE1_HUMAN Nesprin-1 OS=Homo sapiens OX=9606 GN=SYNE1 PE=1 SV=4

MATSRGASRCPRDIANVMQRLQDEQEIVQKRTFTKWINSHLAKRKPPMVVDDLFEDMKDG
VKLLALLEVLSGQKLPCEQGRRMKRIHAVANIGTALKFLEGRKIKLVNINSTDIADGRPS
IVLGLMWTIILYFQIEELTSNLPQLQSLSSSASSVDSIVSSETPSPPSKRKVTTKIQGNA
KKALLKWVQYTAGKQTGIEVKDFGKSWRSGVAFHSVIHAIRPELVDLETVKGRSNRENLE
DAFTIAETELGIPRLLDPEDVDVDKPDEKSIMTYVAQFLKHYPDIHNASTDGQEDDEILP
GFPSFANSVQNFKREDRVIFKEMKVWIEQFERDLTRAQMVESNLQDKYQSFKHFRVQYEM
KRKQIEHLIQPLHRDGKLSLDQALVKQSWDRVTSRLFDWHIQLDKSLPAPLGTIGAWLYR
AEVALREEITVQQVHEETANTIQRKLEQHKDLLQNTDAHKRAFHEIYRTRSVNGIPVPPD
QLEDMAERFHFVSSTSELHLMKMEFLELKYRLLSLLVLAESKLKSWIIKYGRRESVEQLL
QNYVSFIENSKFFEQYEVTYQILKQTAEMYVKADGSVEEAENVMKFMNETTAQWRNLSVE
VRSVRSMLEEVISNWDRYGNTVASLQAWLEDAEKMLNQSENAKKDFFRNLPHWIQQHTAM
NDAGNFLIETCDEMVSRDLKQQLLLLNGRWRELFMEVKQYAQADEMDRMKKEYTDCVVTL
SAFATEAHKKLSEPLEVSFMNVKLLIQDLEDIEQRVPVMDAQYKIITKTAHLITKESPQE
EGKEMFATMSKLKEQLTKVKECYSPLLYESQQLLIPLEELEKQMTSFYDSLGKINEIITV
LEREAQSSALFKQKHQELLACQENCKKTLTLIEKGSQSVQKFVTLSNVLKHFDQTRLQRQ
IADIHVAFQSMVKKTGDWKKHVETNSRLMKKFEESRAELEKVLRIAQEGLEEKGDPEELL
RRHTEFFSQLDQRVLNAFLKACDELTDILPEQEQQGLQEAVRKLHKQWKDLQGEAPYHLL
HLKIDVEKNRFLASVEECRTELDRETKLMPQEGSEKIIKEHRVFFSDKGPHHLCEKRLQL
IEELCVKLPVRDPVRDTPGTCHVTLKELRAAIDSTYRKLMEDPDKWKDYTSRFSEFSSWI
STNETQLKGIKGEAIDTANHGEVKRAVEEIRNGVTKRGETLSWLKSRLKVLTEVSSENEA
QKQGDELAKLSSSFKALVTLLSEVEKMLSNFGDCVQYKEIVKNSLEELISGSKEVQEQAE
KILDTENLFEAQQLLLHHQQKTKRISAKKRDVQQQIAQAQQGEGGLPDRGHEELRKLEST
LDGLERSRERQERRIQVTLRKWERFETNKETVVRYLFQTGSSHERFLSFSSLESLSSELE
QTKEFSKRTESIAVQAENLVKEASEIPLGPQNKQLLQQQAKSIKEQVKKLEDTLEEDIKT
MEMVKTKWDHFGSNFETLSVWITEKEKELNALETSSSAMDMQISQIKVTIQEIESKLSSI
VGLEEEAQSFAQFVTTGESARIKAKLTQIRRYGEELREHAQCLEGTILGHLSQQQKFEEN
LRKIQQSVSEFEDKLAVPIKICSSATETYKVLQEHMDLCQALESLSSAITAFSASARKVV
NRDSCVQEAAALQQQYEDILRRAKERQTALENLLAHWQRLEKELSSFLTWLERGEAKASS
PEMDISADRVKVEGELQLIQALQNEVVSQASFYSKLLQLKESLFSVASKDDVKMMKLHLE
QLDERWRDLPQIINKRINFLQSVVAEHQQFDELLLSFSVWIKLFLSELQTTSEISIMDHQ
VALTRHKDHAAEVESKKGELQSLQGHLAKLGSLGRAEDLHLLQGKAEDCFQLFEEASQVV
ERRQLALSHLAEFLQSHASLSGILRQLRQTVEATNSMNKNESDLIEKDLNDALQNAKALE
SAAVSLDGILSKAQYHLKIGSSEQRTSCRATADQLCGEVERIQNLLGTKQSEADALAVLK
KAFQDQKEELLKSIEDIEERTDKERLKEPTRQALQQRLRVFNQLEDELNSHEHELCWLKD
KAKQIAQKDVAFAPEVDREINRLEVTWDDTKRLIHENQGQCCGLIDLMREYQNLKSAVSK
VLENASSVIVTRTTIKDQEDLKWAFSKHETAKNKMNYKQKDLDNFTSKGKHLLSELKKIH
SSDFSLVKTDMESTVDKWLDVSEKLEENMDRLRVSLSIWDDVLSTRDEIEGWSNNCVPQM
AENISNLDNHLRAEELLKEFESEVKNKALRLEELHSKVNDLKELTKNLETPPDLQFIEAD
LMQKLEHAKEITEVAKGTLKDFTAQSTQVEKFINDITTWFTKVEESLMNCAQNETCEALK
KVKDIQKELQSQQSNISSTQENLNSLCRKYHSAELESLGRAMTGLIKKHEAVSQLCSKTQ
ASLQESLEKHFSESMQEFQEWFLGAKAAAKESSDRTGDSKVLEAKLHDLQNILDSVSDGQ
SKLDAVTQEGQTLYAHLSKQIVSSIQEQITKANEEFQAFLKQCLKDKQALQDCASELGSF
EDQHRKLNLWIHEMEERFNTENLGESKQHIPEKKNEVHKVEMFLGELLAARESLDKLSQR
GQLLSEEGHGAGQEGRLCSQLLTSHQNLLRMTKEKLRSCQVALQEHEALEEALQSMWFWV

KAIQDRLACAESTLGSKDTLEKRLSQIQDILLMKGEGEVKLNMAIGKGEQALRSSNKEGQ
RVIQTQLETLKEVWADIMSSSVHAQSTLESVISQWNDYVERKNQLEQWMESVDQKIEHPL
QPQPGLKEKFVLLDHLQSILSEAEDHTRALHRLIAKSRELYEKTEDESFKDTAQEELKTQ
FNDIMTVAKEKMRKVEEIVKDHLMYLDAVHEFTDWLHSAKEELHRWSDMSGDSSATQKKL
SKIKELIDSREIGASRLSRVESLAPEVKQNTTASGCELMHTEMQALRADWKQWEDSVFQT
QSCLENLVSQMALSEQEFSGQVAQLEQALEQFSALLKTWAQQLTLLEGKNTDEEIVECWH
KGQEILDALQKAEPRTEDLKSQLNELCRFSRDLSTYSGKVSGLIKEYNCLCLQASKGCQN
KEQILQQRFRKAFRDFQQWLVNAKITTAKCFDIPQNISEVSTSLQKIQEFLSESENGQHK
LNMMLSKGELLSTLLTKEKAKGIQAKVTAAKEDWKNFHSNLHQKESALENLKIQMKDFEV
SAEPIQDWLSKTEKMVHESSNRLYDLPAKRREQQKLQSVLEEIHCYEPQLNRLKEKAQQL
WEGQAASKSFRHRVSQLSSQYLALSNLTKEKVSRLDRIVAEHNQFSLGIKELQDWMTDAI
HMLDSYCHPTSDKSVLDSRTLKLEALLSVKQEKEIQMKMIVTRGESVLQNTSPEGIPTIQ
QQLQSVKDMWASLLSAGIRCKSQLEGALSKWTSYQDGVRQFSGWMDSMEANLNESERQHA
ELRDKTTMLGKAKLLNEEVLSYSSLLETIEVKGAGMTEHYVTQLELQDLQERYRAIQERA
KEAVTKSEKLVRLHQEYQRDLKAFEVWLGQEQEKLDQYSVLEGDAHTHETTLRDLQELQV
HCAEGQALLNSVLHTREDVIPSGIPQAEDRALESLRQDWQAYQHRLSETRTQFNNVVNKL
RLMEQKFQQVDEWLKTAEEKVSPRTRRQSNRATKEIQLHQMKKWHEEVTAYRDEVEEVGA
RAQEILDESHVNSRMGCQATQLTSRYQALLLQVLEQIKFLEEEIQSLEESESSLSSYSDW
YGSTHKNFKNVATKIDKVDTVMMGKKLKTLEVLLKDMEKGHSLLKSAREKGERAVKYLEE
GEAERLRKEIHDHMEQLKELTSTVRKEHMTLEKGLHLAKEFSDKCKALTQWIAEYQEILH
VPEEPKMELYEKKAQLSKYKSLQQTVLSHEPSVKSVREKGEALLELVQDVTLKDKIDQLQ
SDYQDLCSIGKEHVFSLEAKVKDHEDYNSELQEVEKWLLQMSGRLVAPDLLETSSLETIT
QQLAHHKAMMEEIAGFEDRLNNLQMKGDTLIGQCADHLQAKLKQNVHAHLQGTKDSYSAI
CSTAQRMYQSLEHELQKHVSRQDTLQQCQAWLSAVQPDLEPSPQPPLSRAEAIKQVKHFR
ALQEQARTYLDLLCSMCDLSNASVKTTAKDIQQTEQTIEQKLVQAQNLTQGWEEIKHLKS
ELWIYLQDADQQLQNMKRRHSELELNIAQNMVSQVKDFVKKLQSKQASVNTIIEKVNKLT
KKEESPEHKEINHLNDQWLDLCRQSNNLCLQREEDLQRTRDYHDCMNVVEVFLEKFTTEW
DNLARSDAESTAVHLEALKKLALALQERKYAIEDLKDQKQKMIEHLNLDDKELVKEQTSH
LEQRWFQLEDLIKRKIQVSVTNLEELNVVQSRFQELMEWAEEQQPNIAEALKQSPPPDMA
QNLLMDHLAICSELEAKQMLLKSLIKDADRVMADLGLNERQVIQKALSDAQSHVNCLSDL
VGQRRKYLNKALSEKTQFLMAVFQATSQIQQHERKIMFREHICLLPDDVSKQVKTCKSAQ
ASLKTYQNEVTGLWAQGRELMKEVTEQEKSEVLGKLQELQSVYDSVLQKCSHRLQELEKN
LVSRKHFKEDFDKACHWLKQADIVTFPEINLMNESSELHTQLAKYQNILEQSPEYENLLL
TLQRTGQTILPSLNEVDHSYLSEKLNALPRQFNVIVALAKDKFYKVQEAILARKEYASLI
ELTTQSLSELEAQFLRMSKVPTDLAVEEALSLQDGCRAILDEVAGLGEAVDELNQKKEGF
RSTGQPWQPDKMLHLVTLYHRLKRQTEQRVSLLEDTTSAYQEHEKMCQQLERQLKSVKEE
QSKVNEETLPAEEKLKMYHSLAGSLQDSGIVLKRVTIHLEDLAPHLDPLAYEKARHQIQS
WQGELKLLTSAIGETVTECESRMVQSIDFQTEMSRSLDWLRRVKAELSGPVYLDLNLQDI
QEEIRKIQIHQEEVQSSLRIMNALSHKEKEKFTKAKELISADLEHSLAELSELDGDIQEA
LRTRQATLTEIYSQCQRYYQVFQAANDWLEDAQELLQLAGNGLDVESAEENLKSHMEFFS
TEDQFHSNLEELHSLVATLDPLIKPTGKEDLEQKVASLELRSQRMSRDSGAQVDLLQRCT
AQWHDYQKAREEVIELMNDTEKKLSEFSLLKTSSSHEAEEKLSEHKALVSVVNSFHEKIV
ALEEKASQLEKTGNDASKATLSRSMTTVWQRWTRLRAVAQDQEKILEDAVDEWTGFNNKV
KKATEMIDQLQDKLPGSSAEKASKAELLTLLEYHDTFVLELEQQQSALGMLRQQTLSMLQ

DGAAPTPGEEPPLMQEITAMQDRCLNMQEKVKTNGKLVKQELKDREMVETQINSVKCWVQ
ETKEYLGNPTIEIDAQLEELQILLTEATNHRQNIEKMAEEQKEKYLGLYTILPSELSLQL
AEVALDLKIRDQIQDKIKEVEQSKATSQELSRQIQKLAKDLTTILTKLKAKTDNVVQAKT
DQKVLGEELDGCNSKLMELDAAVQKFLEQNGQLGKPLAKKIGKLTELHQQTIRQAENRLS
KLNQAASHLEEYNEMLELILKWIEKAKVLAHGTIAWNSASQLREQYILHQTLLEESKEID
SELEAMTEKLQYLTSVYCTEKMSQQVAELGRETEELRQMIKIRLQNLQDAAKDMKKFEAE
LKKLQAALEQAQATLTSPEVGRLSLKEQLSHRQHLLSEMESLKPKVQAVQLCQSALRIPE
DVVASLPLCHAALRLQEEASRLQHTAIQQCNIMQEAVVQYEQYEQEMKHLQQLIEGAHRE
IEDKPVATSNIQELQAQISRHEELAQKIKGYQEQIASLNSKCKMLTMKAKHATMLLTVTE
VEGLAEGTEDLDGELLPTPSAHPSVVMMTAGRCHTLLSPVTEESGEEGTNSEISSPPACR
SPSPVANTDASVNQDIAYYQALSAERLQTDAAKIHPSTSASQEFYEPGLEPSATAKLGDL
QRSWETLKNVISEKQRTLYEALERQQKYQDSLQSISTKMEAIELKLSESPEPGRSPESQM
AEHQALMDEILMLQDEINELQSSLAEELVSESCEADPAEQLALQSTLTVLAERMSTIRMK
ASGKRQLLEEKLNDQLEEQRQEQALQRYRCEADELDSWLLSTKATLDTALSPPKEPMDME
AQLMDCQNMLVEIEQKVVALSELSVHNENLLLEGKAHTKDEAEQLAGKLRRLKGSLLELQ
RALHDKQLNMQGTAQEKEESDVDLTATQSPGVQEWLAQARTTWTQQRQSSLQQQKELEQE
LAEQKSLLRSVASRGEEILIQHSAAETSGDAGEKPDVLSQELGMEGEKSSAEDQMRMKWE
SLHQEFSTKQKLLQNVLEQEQEQVLYSRPNRLLSGVPLYKGDVPTQDKSAVTSLLDGLNQ
AFEEVSSQSGGAKRQSIHLEQKLYDGVSATSTWLDDVEERLFVATALLPEETETCLFNQE
ILAKDIKEMSEEMDKNKNLFSQAFPENGDNRDVIEDTLGCLLGRLSLLDSVVNQRCHQMK
ERLQQILNFQNDLKVLFTSLADNKYIILQKLANVFEQPVAEQIEAIQQAEDGLKEFDAGI
IELKRRGDKLQVEQPSMQELSKLQDMYDELMMIIGSRRSGLNQNLTLKSQYERALQDLAD
LLETGQEKMAGDQKIIVSSKEEIQQLLDKHKEYFQGLESHMILTETLFRKIISFAVQKET
QFHTELMAQASAVLKRAHKRGVELEYILETWSHLDEDQQELSRQLEVVESSIPSVGLVEE
NEDRLIDRITLYQHLKSSLNEYQPKLYQVLDDGKRLLISISCSDLESQLNQLGECWLSNT
NKMSKELHRLETILKHWTRYQSESADLIHWLQSAKDRLEFWTQQSVTVPQELEMVRDHLN
AFLEFSKEVDAQSSLKSSVLSTGNQLLRLKKVDTATLRSELSRIDSQWTDLLTNIPAVQE
KLHQLQMDKLPSRHAISEVMSWISLMENVIQKDEDNIKNSIGYKAIHEYLQKYKGFKIDI
NCKQLTVDFVNQSVLQISSQDVESKRSDKTDFAEQLGAMNKSWQILQGLVTEKIQLLEGL
LESWSEYENNVQCLKTWFETQEKRLKQQHRIGDQASVQNALKDCQDLEDLIKAKEKEVEK
IEQNGLALIQNKKEDVSSIVMSTLRELGQTWANLDHMVGQLKILLKSVLDQWSSHKVAFD
KINSYLMEARYSLSRFRLLTGSLEAVQVQVDNLQNLQDDLEKQERSLQKFGSITNQLLKE
CHPPVTETLTNTLKEVNMRWNNLLEEIAEQLQSSKALLQLWQRYKDYSKQCASTVQQQED
RTNELLKAATNKDIADDEVATWIQDCNDLLKGLGTVKDSLFFLHELGEQLKQQVDASAAS
AIQSDQLSLSQHLCALEQALCKQQTSLQAGVLDYETFAKSLEALEAWIVEAEEILQGQDP
SHSSDLSTIQERMEELKGQMLKFSSMAPDLDRLNELGYRLPLNDKEIKRMQNLNRHWSLI
SSQTTERFSKLQSFLLQHQTFLEKCETWMEFLVQTEQKLAVEISGNYQHLLEQQRAHELF
QAEMFSRQQILHSIIIDGQRLLEQGQVDDRDEFNLKLTLLSNQWQGVIRRAQQRRGIIDS
QIRQWQRYREMAEKLRKWLVEVSYLPMSGLGSVPIPLQQARTLFDEVQFKEKVFLRQQGS
YILTVEAGKQLLLSADSGAEAALQAELAEIQEKWKSASMRLEEQKKKLAFLLKDWEKCEK
GIADSLEKLRTFKKKLSQSLPDHHEELHAEQMRCKELENAVGSWTDDLTQLSLLKDTLSA
YISADDISILNERVELLQRQWEELCHQLSLRRQQIGERLNEWAVFSEKNKELCEWLTQME
SKVSQNGDILIEEMIEKLKKDYQEEIAIAQENKIQLQQMGERLAKASHESKASEIEYKLG
KVNDRWQHLLDLIAARVKKLKETLVAVQQLDKNMSSLRTWLAHIESELAKPIVYDSCNSE

EIQRKLNEQQELQRDIEKHSTGVASVLNLCEVLLHDCDACATDAECDSIQQATRNLDRRW
RNICAMSMERRLKIEETWRLWQKFLDDYSRFEDWLKSSERTAAFPSSSGVIYTVAKEELK
KFEAFQRQVHECLTQLELINKQYRRLARENRTDSACSLKQMVHEGNQRWDNLQKRVTSIL
RRLKHFIGQREEFETARDSILVWLTEMDLQLTNIEHFSECDVQAKIKQLKAFQQEISLNH
NKIEQIIAQGEQLIEKSEPLDAAIIEEELDELRRYCQEVFGRVERYHKKLIRLPLPDDEH
DLSDRELELEDSAALSDLHWHDRSADSLLSPQPSSNLSLSLAQPLRSERSGRDTPASVDS
IPLEWDHDYDLSRDLESAMSRALPSEDEEGQDDKDFYLRGAVGLSGDHSALESQIRQLGK
ALDDSRFQIQQTENIIRSKTPTGPELDTSYKGYMKLLGECSSSIDSVKRLEHKLKEEEES
LPGFVNLHSTETQTAGVIDRWELLQAQALSKELRMKQNLQKWQQFNSDLNSIWAWLGDTE
EELEQLQRLELSTDIQTIELQIKKLKELQKAVDHRKAIILSINLCSPEFTQADSKESRDL
QDRLSQMNGRWDRVCSLLEEWRGLLQDALMQCQGFHEMSHGLLLMLENIDRRKNEIVPID
SNLDAEILQDHHKQLMQIKHELLESQLRVASLQDMSCQLLVNAEGTDCLEAKEKVHVIGN
RLKLLLKEVSRHIKELEKLLDVSSSQQDLSSWSSADELDTSGSVSPTSGRSTPNRQKTPR
GKCSLSQPGPSVSSPHSRSTKGGSDSSLSEPGPGRSGRGFLFRVLRAALPLQLLLLLLIG
LACLVPMSEEDYSCALSNNFARSFHPMLRYTNGPPPL
>sp|Q8NFZ8|CADM4_HUMAN Cell adhesion molecule 4 OS=Homo sapiens OX=9606
GN=CADM4 PE=1 SV=1
MGRARRFQWPLLLLWAAAAGPGAGQEVQTENVTVAEGGVAEITCRLHQYDGSIVVIQNPA
RQTLFFNGTRALKDERFQLEEFSPRRVRIRLSDARLEDEGGYFCQLYTEDTHHQIATLTV
LVAPENPVVEVREQAVEGGEVELSCLVPRSRPAATLRWYRDRKELKGVSSSQENGKVWSV
ASTVRFRVDRKDDGGIIICEAQNQALPSGHSKQTQYVLDVQYSPTARIHASQAVVREGDT
LVLTCAVTGNPRPNQIRWNRGNESLPERAEAVGETLTLPGLVSADNGTYTCEASNKHGHA
RALYVLVVYDPGAVVEAQTSVPYAIVGGILALLVFLIICVLVGMVWCSVRQKGSYLTHEA
SGLDEQGEAREAFLNGSDGHKRKEEFFI
>sp|Q8NHU6|TDRD7_HUMAN Tudor domain-containing protein 7 OS=Homo sapiens OX=9606
GN=TDRD7 PE=1 SV=2
MLEGDLVSKMLRAVLQSHKNGVALPRLQGEYRSLTGDWIPFKQLGFPTLEAYLRSVPAVV
RIETSRSGEITCYAMACTETARIAQLVARQRSSKRKTGRQVNCQMRVKKTMPFFLEGKPK
ATLRQPGFASNFSVGKKPNPAPLRDKGNSVGVKPDAEMSPYMLHTTLGNEAFKDIPVQRH
VTMSTNNRFSPKASLQPPLQMHLSRTSTKEMSDNLNQTVEKPNVKPPASYTYKMDEVQNR
IKEILNKHNNGIWISKLPHFYKELYKEDLNQGILQQFEHWPHICTVEKPCSGGQDLLLYP
AKRKQLLRSELDTEKVPLSPLPGPKQTPPLKGCPTVMAGDFKEKVADLLVKYTSGLWASA
LPKAFEEMYKVKFPEDALKNLASLSDVCSIDYISGNPQKAILYAKLPLPTDKIQKDAGQA
HGDNDIKAMVEQEYLQVEESIAESANTFMEDITVPPLMIPTEASPSVLVVELSNTNEVVI
RYVGKDYSAAQELMEDEMKEYYSKNPKITPVQAVNVGQLLAVNAEEDAWLRAQVISTEEN
KIKVCYVDYGFSENVEKSKAYKLNPKFCSLSFQATKCKLAGLEVLSDDPDLVKVVESLTC
GKIFAVEILDKADIPLVVLYDTSGEDDININATCLKAICDKSLEVHLQVDAMYTNVKVTN
ICSDGTLYCQVPCKGLNKLSDLLRKIEDYFHCKHMTSECFVSLPFCGKICLFHCKGKWLR
VEITNVHSSRALDVQFLDSGTVTSVKVSELREIPPRFLQEMIAIPPQAIKCCLADLPQSI
GMWTPDAVLWLRDSVLNCSDCSIKVTKVDETRGIAHVYLFTPKNFPDPHRSINRQITNAD
LWKHQKDVFLSAISSGADSPNSKNGNMPMSGNTGENFRKNLTDVIKKSMVDHTSAFSTEE
LPPPVHLSKPGEHMDVYVPVACHPGYFVIQPWQEIHKLEVLMEEMILYYSVSEERHIAVE
KDQVYAAKVENKWHRVLLKGILTNGLVSVYELDYGKHELVNIRKVQPLVDMFRKLPFQAV
TAQLAGVKCNQWSEEASMVFRNHVEKKPLVALVQTVIENANPWDRKVVVYLVDTSLPDTD

TWIHDFMSEYLIELSKVN
>sp|Q8TAT6|NPL4_HUMAN Nuclear protein localization protein 4 homolog OS=Homo sapiens OX=9606 GN=NPLOC4 PE=1 SV=3
MAESIIIRVQSPDGVKRITATKRETAATFLKKVAKEFGFQNNGFSVYINRNKTGEITASS
NKSLNLLKIKHGDLLFLFPSSLAGPSSEMETSVPPGFKVFGAPNVVEDEIDQYLSKQDGK
IYRSRDPQLCRHGPLGKCVHCVPLEPFDEDYLNHLEPPVKHMSFHAYIRKLTGGADKGKF
VALENISCKIKSGCEGHLPWPNGICTKCQPSAITLNRQKYRHVDNIMFENHTVADRFLDF
WRKTGNQHFGYLYGRYTEHKDIPLGIRAEVAAIYEPPQIGTQNSLELLEDPKAEVVDEIA
AKLGLRKVGWIFTDLVSEDTRKGTVRYSRNKDTYFLSSEECITAGDFQNKHPNMCRLSPD
GHFGSKFVTAVATGGPDNQVHFEGYQVSNQCMALVRDECLLPCKDAPELGYAKESSSEQY
VPDVFYKDVDKFGNEITQLARPLPVEYLIIDITTTFPKDPVYTFSISQNPFPIENRDVLG
ETQDFHSLATYLSQNTSSVFLDTISDFHLLLFLVTNEVMPLQDSISLLLEAVRTRNEELA
QTWKRSEQWATIEQLCSTVGGQLPGLHEYGAVGGSTHTATAAMWACQHCTFMNQPGTGHC
EMCSLPRT
>sp|Q8TBB5|KLDC4_HUMAN Kelch domain-containing protein 4 OS=Homo sapiens OX=9606 GN=KLHDC4 PE=1 SV=1
MGKKGKKEKKGRGAEKTAAKMEKKVSKRSRKEEEDLEALIAHFQTLDAKRTQTVELPCPP
PSPRLNASLSVHPEKDELILFGGEYFNGQKTFLYNELYVYNTRKDTWTKVDIPSPPPRRC
AHQAVVVPQGGGQLWVFGGEFASPNGEQFYHYKDLWVLHLATKTWEQVKSTGGPSGRSGH
RMVAWKRQLILFGGFHESTRDYIYYNDVYAFNLDTFTWSKLSPSGTGPTPRSGCQMSVTP
QGGIVVYGGYSKQRVKKDVDKGTRHSDMFLLKPEDGREDKWVWTRMNPSGVKPTPRSGFS
VAMAPNHQTLFFGGVCDEEEEESLSGEFFNDLYFYDATRNRWFEGQLKGPKSEKKKRRRG
RKEEPEGGSRPACGGAGTQGPVQLVKEVVAEDGTVVTIKQVLTAPGSAGQPRSEDEDSLE
EAGSPAPGPCPRSNAMLAVKHGVLYVYGGMFEAGDRQVTLSDLHCLDLHRMEAWKALVEM
DPETQEWLEETDSEEDSEEVEGAEGGVDDEDSGEESGAED
>sp|Q8TBF2|PXL2B_HUMAN Prostamide/prostaglandin F synthase OS=Homo sapiens OX=9606 GN=PRXL2B PE=1 SV=1
MSTVDLARVGACILKHAVTGEAVELRSLWREHACVVAGLRRFGCVVCRWIAQDLSSLAGL
LDQHGVRLVGVGPEALGLQEFLDGDYFAGELYLDESKQLYKELGFKRYNSLSILPAALGK
PVRDVAAKAKAVGIQGNLSGDLLQSGGLLVVSKGGDKVLLHFVQKSPGDYVPKEHILQVL
GISAEVCASDPPQCDREV
>sp|Q8TCT0|CERK1_HUMAN Ceramide kinase OS=Homo sapiens OX=9606 GN=CERK PE=1 SV=1
MGATGAAEPLQSVLWVKQQRCAVSLEPARALLRWWRSPGPGAGAPGADACSVPVSEIIAV
EETDVHGKHQGSGKWQKMEKPYAFTVHCVKRARRHRWKWAQVTFWCPEEQLCHLWLQTLR
EMLEKLTSRPKHLLVFINPFGGKGQGKRIYERKVAPLFTLASITTDIIVTEHANQAKETL
YEINIDKYDGIVCVGGDGMFSEVLHGLIGRTQRSAGVDQNHPRAVLVPSSLRIGIIPAGS
TDCVCYSTVGTSDAETSALHIVVGDSLAMDVSSVHHNSTLLRYSVSLLGYGFYGDIIKDS
EKKRWLGLARYDFSGLKTFLSHHCYEGTVSFLPAQHTVGSPRDRKPCRAGCFVCRQSKQQ
LEEEQKKALYGLEAAEDVEEWQVVCGKFLAINATNMSCACRRSPRGLSPAAHLGDGSSDL
ILIRKCSRFNFLRFLIRHTNQQDQFDFTFVEVYRVKKFQFTSKHMEDEDSDLKEGGKKRF
GHICSSHPSCCCTVSNSSWNCDGEVLHSPAIEVRVHCQLVRLFARGIEENPKPDSHS
>sp|Q8TD20|GTR12_HUMAN Solute carrier family 2, facilitated glucose transporter member 12 OS=Homo sapiens OX=9606 GN=SLC2A12 PE=2 SV=1
MVPVENTEGPSLLNQKGTAVETEGSGSRHPPWARGCGMFTFLSSVTAAVSGLLVGYELGI

ISGALLQIKTLLALSCHEQEMVVSSLVIGALLASLTGGVLIDRYGRRTAIILSSCLLGLG
SLVLILSLSYTVLIVGRIAIGVSISLSSIATCVYIAEIAPQHRRGLLVSLNELMIVIGIL
SAYISNYAFANVFHGWKYMFGLVIPLGVLQAIAMYFLPPSPRFLVMKGQEGAASKVLGRL
RALSDTTEELTVIKSSLKDEYQYSFWDLFRSKDNMRTRIMIGLTLVFFVQITGQPNILFY
ASTVLKSVGFQSNEAASLASTGVGVVKVISTIPATLLVDHVGSKTFLCIGSSVMAASLVT
MGIVNLNIHMNFTHICRSHNSINQSLDESVIYGPGNLSTNNNTLRDHFKGISSHSRSSLM
PLRNDVDKRGETTSASLLNAGLSHTEYQIVTDPGDVPAFLKWLSLASLLVYVAAFSIGLG
PMPWLVLSEIFPGGIRGRAMALTSSMNWGINLLISLTFLTVTDLIGLPWVCFIYTIMSLA
SLLFVVMFIPETKGCSLEQISMELAKVNYVKNNICFMSHHQEELVPKQPQKRKPQEQLLE
CNKLCGRGQSRQLSPET
>sp|Q8TDB4|HUMMR_HUMAN Protein MGARP OS=Homo sapiens OX=9606 GN=MGARP PE=1 SV=1
MYLRRAVSKTLALPLRAPPNPAPLGKDASLRRMSSNRFPGSSGSNMIYYLVVGVTVSAGG
YYAYKTVTSDQAKHTEHKTNLKEKTKAEIHPFQGEKENVAETEKASSEAPEELIVEAEVV
DAEESPSATVVVIKEASACPGHVEAAPETTAVSAETGPEVTDAAARETTEVNPETTPEVT
NAALDEAVTIDNDKDTTKNETSDEYAELEEENSPAESESSAGDDLQEEASVGSEAASAQG
>sp|Q8TDQ7|GNPI2_HUMAN Glucosamine-6-phosphate isomerase 2 OS=Homo sapiens OX=9606 GN=GNPDA2 PE=1 SV=1
MRLVILDNYDLASEWAAKYICNRIIQFKPGQDRYFTLGLPTGSTPLGCYKKLIEYHKNGH
LSFKYVKTFNMDEYVGLPRNHPESYHSYMWNNFFKHIDIDPNNAHILDGNAADLQAECDA
FENKIKEAGGIDLFVGGIGPDGHIAFNEPGSSLVSRTRLKTLAMDTILANAKYFDGDLSK
VPTMALTVGVGTVMDAREVMILITGAHKAFALYKAIEEGVNHMWTVSAFQQHPRTIFVCD
EDATLELRVKTVKYFKGLMHVHNKLVDPLFSMKDGN
>sp|Q8WUK0|PTPM1_HUMAN Phosphatidylglycerophosphatase and protein-tyrosine phosphatase 1 OS=Homo sapiens OX=9606 GN=PTPMT1 PE=1 SV=1
MAATALLEAGLARVLFYPTLLYTLFRGKVPGRAHRDWYHRIDPTVLLGALPLRSLTRQLV
QDENVRGVITMNEEYETRFLCNSSQEWKRLGVEQLRLSTVDMTGIPTLDNLQKGVQFALK
YQSLGQCVYVHCKAGRSRSATMVAAYLIQVHKWSPEEAVRAIAKIRSYIHIRPGQLDVLK
EFHKQITARATKDGTFVISKT
>sp|Q8WUM4|PDC6I_HUMAN Programmed cell death 6-interacting protein OS=Homo sapiens OX=9606 GN=PDCD6IP PE=1 SV=1
MATFISVQLKKTSEVDLAKPLVKFIQQTYPSGGEEQAQYCRAAEELSKLRRAAVGRPLDK
HEGALETLLRYYDQICSIEPKFPFSENQICLTFTWKDAFDKGSLFGGSVKLALASLGYEK
SCVLFNCAALASQIAAEQNLDNDEGLKIAAKHYQFASGAFLHIKETVLSALSREPTVDIS
PDTVGTLSLIMLAQAQEVFFLKATRDKMKDAIIAKLANQAADYFGDAFKQCQYKDTLPKE
VFPVLAAKHCIMQANAEYHQSILAKQQKKFGEEIARLQHAAELIKTVASRYDEYVNVKDF
SDKINRALAAAKKDNDFIYHDRVPDLKDLDPIGKATLVKSTPVNVPISQKFTDLFEKMVP
VSVQQSLAAYNQRKADLVNRSIAQMREATTLANGVLASLNLPAAIEDVSGDTVPQSILTK
SRSVIEQGGIQTVDQLIKELPELLQRNREILDESLRLLDEEEATDNDLRAKFKERWQRTP
SNELYKPLRAEGTNFRTVLDKAVQADGQVKECYQSHRDTIVLLCKPEPELNAAIPSANPA
KTMQGSEVVNVLKSLLSNLDEVKKEREGLENDLKSVNFDMTSKFLTALAQDGVINEEALS
VTELDRVYGGLTTKVQESLKKQEGLLKNIQVSHQEFSKMKQSNNEANLREEVLKNLATAY
DNFVELVANLKEGTKFYNELTEILVRFQNKCSDIVFARKTERDELLKDLQQSIAREPSAP
SIPTPAYQSSPAGGHAPTPPTPAPRTMPPTKPQPPARPPPPVLPANRAPSATAPSPVGAG

TAAPAPSQTPGSAPPPQAQGPPYPTYPGYPGYCQMPMPMGYNPYAYGQYNMPYPPVYHQS
PGQAPYPGPQQPSYPFPQPPQQSYYPQQ
>sp|Q8WWZ3|EDAD_HUMAN Ectodysplasin-A receptor-associated adapter protein OS=Homo sapiens OX=9606 GN=EDARADD PE=1 SV=3
MGLRTTKQMGRGTKAPGHQEDHMVKEPVEDTDPSTLSFNMSDKYPIQDTELPKAEECDTI
TLNCPRNSDMKNQGEENGFPDSTGDPLPEISKDNSCKENCTCSSCLLRAPTISDLLNDQD
LLDVIRIKLDPCHPTVKNWRNFASKWGMSYDELCFLEQRPQSPTLEFLLRNSQRTVGQLM
ELCRLYHRADVEKVLRRWVDEEWPKRERGDPSRHF
>tr|Q92468|Q92468_HUMAN GTPase HRas (Fragment) OS=Homo sapiens OX=9606 GN=c-bas/has PE=3 SV=1
MTEYKLVVVGAGGVGKSALTIQLIQNHFVDEYDPTIEDSYRKQVVIDGETCLLDILDTAG
LEEYSAMRDQYMRTGEGFLCVFAINNTKSFEDIHQYREQIKRVKDSDDVPMV
>sp|Q92520|FAM3C_HUMAN Protein FAM3C OS=Homo sapiens OX=9606 GN=FAM3C PE=1 SV=1
MRVAGAAKLVVAVAVFLLTFYVISQVFEIKMDASLGNLFARSALDTAARSTKPPRYKCGI
SKACPEKHFAFKMASGAANVVGPKICLEDNVLMSGVKNNVGRGINVALANGKTGEVLDTK
YFDMWGGDVAPFIEFLKAIQDGTIVLMGTYDDGATKLNDEARRLIADLGSTSITNLGFRD
NWVFCGGKGIKTKSPFEQHIKNNKDTNKYEGWPEVVEMEGCIPQKQD
>sp|Q92522|H1X_HUMAN Histone H1.10 OS=Homo sapiens OX=9606 GN=H1-10 PE=1 SV=1
MSVELEEALPVTTAEGMAKKVTKAGGSAALSPSKKRKNSKKKNQPGKYSQLVVETIRRLG
ERNGSSLAKIYTEAKKVPWFDQQNGRTYLKYSIKALVQNDTLLQVKGTGANGSFKLNRKK
LEGGGERRGAPAAATAPAPTAHKAKKAAPGAAGSRRADKKPARGQKPEQRSHKKGAGAKK
DKGGKAKKTAAAGGKKVKKAAKPSVPKVPKGRK
>sp|Q92530|PSMF1_HUMAN Proteasome inhibitor PI31 subunit OS=Homo sapiens OX=9606 GN=PSMF1 PE=1 SV=2
MAGLEVLFASAAPAITCRQDALVCFLHWEVVTHGYFGLGVGDQPGPNDKKSELLPAGWNN
NKDLYVLRYEYKDGSRKLLVKAITVESSMILNVLEYGSQQVADLTLNLDDYIDAEHLGDF
HRTYKNSEELRSRIVSGIITPIHEQWEKANVSSPHREFPPATAREVDPLRIPPHHPHTSR
QPPWCDPLGPFVVGGEDLDPFGPRRGGMIVDPLRSGFPRALIDPSSGLPNRLPPGAVPPG
ARFDPFGPIGTSPPGPNPDHLPPPGYDDMYL
>sp|Q92542|NICA_HUMAN Nicastrin OS=Homo sapiens OX=9606 GN=NCSTN PE=1 SV=2
MATAGGGSGADPGSRGLLRLLSFCVLLAGLCRGNSVERKIYIPLNKTAPCVRLLNATHQI
GCQSSISGDTGVIHVVEKEEDLQWVLTDGPNPPYMVLLESKHFTRDLMEKLKGRTSRIAG
LAVSLTKPSPASGFSPSVQCPNDGFGVYSNSYGPEFAHCREIQWNSLGNGLAYEDFSFPI
FLLEDENETKVIKQCYQDHNLSQNGSAPTFPLCAMQLFSHMHAVISTATCMRRSSIQSTF
SINPEIVCDPLSDYNVWSMLKPINTTGTLKPDDRVVVAATRLDSRSFFWNVAPGAESAVA
SFVTQLAAAEALQKAPDVTTLPRNVMFVFFQGETFDYIGSSRMVYDMEKGKFPVQLENVD
SFVELGQVALRTSLELWMHTDPVSQKNESVRNQVEDLLATLEKSGAGVPAVILRRPNQSQ
PLPPSSLQRFLRARNISGVVLADHSGAFHNKYYQSIYDTAENINVSYPEWLSPEEDLNFV
TDTAKALADVATVLGRALYELAGGTNFSDTVQADPQTVTRLLYGFLIKANNSWFQSILRQ
DLRSYLGDGPLQHYIAVSSPTNTTYVVQYALANLTGTVVNLTREQCQDPSKVPSENKDLY
EYSWVQGPLHSNETDRLPRCVRSTARLARALSPAFELSQWSSTEYSTWTESRWKDIRARI
FLIASKELELITLTVGFGILIFSLIVTYCINAKADVLFIAPREPGAVSY

>sp|Q92597|NDRG1_HUMAN Protein NDRG1 OS=Homo sapiens OX=9606 GN=NDRG1 PE=1 SV=1
MSREMQDVDLAEVKPLVEKGETITGLLQEFDVQEQDIETLHGSVHVTLCGTPKGNRPVIL
TYHDIGMNHKTCYNPLFNYEDMQEITQHFAVCHVDAPGQQDGAASFPAGYMYPSMDQLAE
MLPGVLQQFGLKSIIGMGTGAGAYILTRFALNNPEMVEGLVLINVNPCAEGWMDWAASKI
SGWTQALPDMVVSHLFGKEEMQSNVEVVHTYRQHIVNDMNPGNLHLFINAYNSRRDLEIE
RPMPGTHTVTLQCPALLVVGDSSPAVDAVVECNSKLDPTKTTLLKMADCGGLPQISQPAK
LAEAFKYFVQGMGYMPSASMTRLMRSRTASGSSVTSLDGTRSRSHTSEGTRSRSHTSEGT
RSRSHTSEGAHLDITPNSGAAGNSAGPKSMEVSC
>sp|Q92614|MY18A_HUMAN Unconventional myosin-XVIIIa OS=Homo sapiens OX=9606 GN=MYO18A PE=1 SV=3
MFNLMKKDKDKDGGRKEKKEKKEKKERMSAAELRSLEEMSLRRGFFNLNRSSKRESKTRL
EISNPIPIKVASGSDLHLTDIDSDSNRGSVILDSGHLSTASSSDDLKGEEGSFRGSVLQR
AAKFGSLAKQNSQMIVKRFSFSQRSRDESASETSTPSEHSAAPSPQVEVRTLEGQLVQHP
GPGIPRPGHRSRAPELVTKKFPVDLRLPPVVPLPPPTLRELELQRRPTGDFGFSLRRTTM
LDRGPEGQACRRVVHFAEPGAGTKDLALGLVPGDRLVEINGHNVESKSRDEIVEMIRQSG
DSVRLKVQPIPELSELSRSWLRSGEGPRREPSDAKTEEQIAAEEAWNETEKVWLVHRDGF
SLASQLKSEELNLPEGKVRVKLDHDGAILDVDEDDVEKANAPSCDRLEDLASLVYLNESS
VLHTLRQRYGASLLHTYAGPSLLVLGPRGAPAVYSEKVMHMFKGCRREDMAPHIYAVAQT
AYRAMLMSRQDQSIILLGSSGSGKTTSCQHLVQYLATIAGISGNKVFSVEKWQALYTLLE
AFGNSPTIINGNATRFSQILSLDFDQAGQVASASIQTMLLEKLRVARRPASEATFNVFYY
LLACGDGTLRTELHLNHLAENNVFGIVPLAKPEEKQKAAQQFSKLQAAMKVLGISPDEQK
ACWFILAAIYHLGAAGATKEAAEAGRKQFARHEWAQKAAYLLGCSLEELSSAIFKHQHKG
GTLQRSTSFRQGPEESGLGDGTGPKLSALECLEGMAAGLYSELFTLLVSLVNRALKSSQH
SLCSMMIVDTPGFQNPEQGGSARGASFEELCHNYTQDRLQRLFHERTFVQELERYKEENI
ELAFDDLEPPTDDSVAAVDQASHQSLVRSLARTDEARGLLWLLEEEALVPGASEDTLLER
LFSYYGPQEGDKKGQSPLLHSSKPHHFLLGHSHGTNWVEYNVTGWLNYTKQNPATQNAPR
LLQDSQKKIISNLFLGRAGSATVLSGSIAGLEGGSQLALRRATSMRKTFTTGMAAVKKKS
LCIQMKLQVDALIDTIKKSKLHFVHCFLPVAEGWAGEPRSASSRRVSSSSELDLPSGDHC
EAGLLQLDVPLLRTQLRGSRLLDAMRMYRQGYPDHMVFSEFRRRFDVLAPHLTKKHGRNY
IVVDERRAVEELLECLDLEKSSCCMGLSRVFFRAGTLARLEEQRDEQTSRNLTLFQAACR
GYLARQHFKKRKIQDLAIRCVQKNIKKNKGVKDWPWWKLFTTVRPLIEVQLSEEQIRNKD
EEIQQLRSKLEKAEKERNELRLNSDRLESRISELTSELTDERNTGESASQLLDAETAERL
RAEKEMKELQTQYDALKKQMEVMEMEVMEARLIRAAEINGEVDDDDAGGEWRLKYERAVR
EVDFTKKRLQQEFEDKLEVEQQNKRQLERRLGDLQADSEESQRALQQLKKKCQRLTAELQ
DTKLHLEGQQVRNHELEKKQRRFDSELSQAHEEAQREKLQREKLQREKDMLLAEAFSLKQ
QLEEKDMDIAGFTQKVVSLEAELQDISSQESKDEASLAKVKKQLRDLEAKVKDQEEELDE
QAGTIQMLEQAKLRLEMEMERMRQTHSKEMESRDEEVEEARQSCQKKLKQMEVQLEEEYE
DKQKVLREKRELEGKLATLSDQVNRRDFESEKRLRKDLKRTKALLADAQLMLDHLKNSAP
SKREIAQLKNQLEESEFTCAAAVKARKAMEVEIEDLHLQIDDIAKAKTALEEQLSRLQRE
KNEIQNRLEEDQEDMNELMKKHKAAVAQASRDLAQINDLQAQLEEANKEKQELQEKLQAL
QSQVEFLEQSMVDKSLVSRQEAKIRELETRLEFERTQVKRLESLASRLKENMEKLTEERD
QRIAAENREKEQNKRLQRQLRDTKEEMGELARKEAEASRKKHELEMDLESLEAANQSLQA
DLKLAFKRIGDLQAAIEDEMESDENEDLINSLQDMVTKYQKRKNKLEGDSDVDSELEDRV

DGVKSWLSKNKGPSKAASDDGSLKSSSPTSYWKSLAPDRSDDEHDPLDNTSRPRYSHSYL
SDSDTEAKLTETNA
>sp|Q92696|PGTA_HUMAN Geranylgeranyl transferase type-2 subunit alpha OS=Homo sapiens OX=9606 GN=RABGGTA PE=1 SV=2
MHGRLKVKTSEEQAEAKRLEREQKLKLYQSATQAVFQKRQAGELDESVLELTSQILGANP
DFATLWNCRREVLQQLETQKSPEELAALVKAELGFLESCLRVNPKSYGTWHHRCWLLGRL
PEPNWTRELELCARFLEVDERNFHCWDYRRFVATQAAVPPAEELAFTDSLITRNFSNYSS
WHYRSCLLPQLHPQPDSGPQGRLPEDVLLKELELVQNAFFTDPNDQSAWFYHRWLLGRAD
PQDALRCLHVSRDEACLTVSFSRPLLVGSRMEILLLMVDDSPLIVEWRTPDGRNRPSHVW
LCDLPAASLNDQLPQHTFRVIWTAGDVQKECVLLKGRQEGWCRDSTTDEQLFRCELSVEK
STVLQSELESCKELQELEPENKWCLLTIILLMRALDPLLYEKETLQYFQTLKAVDPMRAT
YLDDLRSKFLLENSVLKMEYAEVRVLHLAHKDLTVLCHLEQLLLVTHLDLSHNRLRTLPP
ALAALRCLEVLQASDNAIESLDGVTNLPRLQELLLCNNRLQQPAVLQPLASCPRLVLLNL
QGNPLCQAVGILEQLAELLPSVSSVLT
>sp|Q92729|PTPRU_HUMAN Receptor-type tyrosine-protein phosphatase U OS=Homo sapiens OX=9606 GN=PTPRU PE=1 SV=2
MARAQALVLALTFQLCAPETETPAAGCTFEEASDPAVPCEYSQAQYDDFQWEQVRIHPGT
RAPADLPHGSYLMVNTSQHAPGQRAHVIFQSLSENDTHCVQFSYFLYSRDGHSPGTLGVY
VRVNGGPLGSAVWNMTGSHGRQWHQAELAVSTFWPNEYQVLFEALISPDRRGYMGLDDIL
LLSYPCAKAPHFSRLGDVEVNAGQNASFQCMAAGRAAEAERFLLQRQSGALVPAAGVRHI
SHRRFLATFPLAAVSRAEQDLYRCVSQAPRGAGVSNFAELIVKEPPTPIAPPQLLRAGPT
YLIIQLNTNSIIGDGPIVRKEIEYRMARGPWAEVHAVSLQTYKLWHLDPDTEYEISVLLT
RPGDGGTGRPGPPLISRTKCAEPMRAPKGLAFAEIQARQLTLQWEPLGYNVTRCHTYTVS
LCYHYTLGSSHNQTIRECVKTEQGVSRYTIKNLLPYRNVHVRLVLTNPEGRKEGKEVTFQ
TDEDVPSGIAAESLTFTPLEDMIFLKWEEPQEPNGLITQYEISYQSIESSDPAVNVPGPR
RTISKLRNETYHVFSNLHPGTTYLFSVRARTGKGFGQAALTEITTNISAPSFDYADMPSP
LGESENTITVLLRPAQGRGAPISVYQVIVEEERARRLRREPGGQDCFPVPLTFEAALARG
LVHYFGAELAASSLPEAMPFTVGDNQTYRGFWNPPLEPRKAYLIYFQAASHLKGETRLNC
IRIARKAACKESKRPLEVSQRSEEMGLILGICAGGLAVLILLLGAIIVIIRKGRDHYAYS
YYPKPVNMTKATVNYRQEKTHMMSAVDRSFTDQSTLQEDERLGLSFMDTHGYSTRGDQRS
GGVTEASSLLGGSPRRPCGRKGSPYHTGQLHPAVRVADLLQHINQMKTAEGYGFKQEYES
FFEGWDATKKKDKVKGSRQEPMPAYDRHRVKLHPMLGDPNADYINANYIDGYHRSNHFIA
TQGPKPEMVYDFWRMVWQEHCSSIVMITKLVEVGRVKCSRYWPEDSDTYGDIKIMLVKTE
TLAEYVVRTFALERRGYSARHEVRQFHFTAWPEHGVPYHATGLLAFIRRVKASTPPDAGP
IVIHCSAGTGRTGCYIVLDVMLDMAECEGVVDIYNCVKTLCSRRVNMIQTEEQYIFIHDA
ILEACLCGETTIPVSEFKATYKEMIRIDPQSNSSQLREEFQTLNSVTPPLDVEECSIALL
PRNRDKNRSMDVLPPDRCLPFLISTDGDSNNYINAALTDSYTRSAAFIVTLHPLQSTTPD
FWRLVYDYGCTSIVMLNQLNQSNSAWPCLQYWPEPGRQQYGLMEVEFMSGTADEDLVARV
FRVQNISRLQEGHLLVRHFQFLRWSAYRDTPDSKKAFLHLLAEVDKWQAESGDGRTIVHC
LNGGGRSGTFCACATVLEMIRCHNLVDVFFAAKTLRNYKPNMVETMDQYHFCYDVALEYL
EGLESR
>sp|Q92743|HTRA1_HUMAN Serine protease HTRA1 OS=Homo sapiens OX=9606 GN=HTRA1 PE=1 SV=1
MQIPRAALLPLLLLLLAAPASAQLSRAGRSAPLAAGCPDRCEPARCPPQPEHCEGGRARD

ACGCCEVCGAPEGAACGLQEGPCGEGLQCVVPFGVPASATVRRRAQAGLCVCASSEPVCG
SDANTYANLCQLRAASRRSERLHRPPVIVLQRGACGQGQEDPNSLRHKYNFIADVVEKIA
PAVVHIELFRKLPFSKREVPVASGSGFIVSEDGLIVTNAHVVTNKHRVKVELKNGATYEA
KIKDVDEKADIALIKIDHQGKLPVLLLGRSSELRPGEFVVAIGSPFSLQNTVTTGIVSTT
QRGGKELGLRNSDMDYIQTDAIINYGNSGGPLVNLDGEVIGINTLKVTAGISFAIPSDKI
KKFLTESHDRQAKGKAITKKKYIGIRMMSLTSSKAKELKDRHRDFPDVISGAYIIEVIPD
TPAEAGGLKENDVIISINGQSVVSANDVSDVIKRESTLNMVVRRGNEDIMITVIPEEIDP
>sp|Q92747|ARC1A_HUMAN Actin-related protein 2/3 complex subunit 1A OS=Homo sapiens OX=9606 GN=ARPC1A PE=1 SV=2
MSLHQFLLEPITCHAWNRDRTQIALSPNNHEVHIYKKNGSQWVKAHELKEHNGHITGIDW
APKSDRIVTCGADRNAYVWSQKDGVWKPTLVILRINRAATFVKWSPLENKFAVGSGARLI
SVCYFESENDWWVSKHIKKPIRSTVLSLDWHPNNVLLAAGSCDFKCRVFSAYIKEVDEKP
ASTPWGSKMPFGQLMSEFGGSGTGGWVHGVSFSASGSRLAWVSHDSTVSVADASKSVQVS
TLKTEFLPLLSVSFVSENSVVAAGHDCCPMLFNYDDRGCLTFVSKLDIPKQSIQRNMSAM
ERFRNMDKRATTEDRNTALETLHQNSITQVSIYEVDKQDCRKFCTTGIDGAMTIWDFKTL
ESSIQGLRIM
>sp|Q92823|NRCAM_HUMAN Neuronal cell adhesion molecule OS=Homo sapiens OX=9606 GN=NRCAM PE=1 SV=3
MQLKIMPKKKRLSAGRVPLILFLCQMISALEVPLDPKLLEDLVQPPTITQQSPKDYIIDP
RENIVIQCEAKGKPPPSFSWTRNGTHFDIDKDPLVTMKPGTGTLIINIMSEGKAETYEGV
YQCTARNERGAAVSNNIVVRPSRSPLWTKEKLEPITLQSGQSLVLPCRPPIGLPPPIIFW
MDNSFQRLPQSERVSQGLNGDLYFSNVLPEDTREDYICYARFNHTQTIQQKQPISVKVIS
VDELNDTIAANLSDTEFYGAKSSRERPPTFLTPEGNASNKEELRGNVLSLECIAEGLPTP
IIYWAKEDGMLPKNRTVYKNFEKTLQIIHVSEADSGNYQCIAKNALGAIHHTISVRVKAA
PYWITAPQNLVLSPGEDGTLICRANGNPKPRISWLTNGVPIEIAPDDPSRKIDGDTIIFS
NVQERSSAVYQCNASNEYGYLLANAFVNVLAEPPRILTPANTLYQVIANRPALLDCAFFG
SPLPTIEWFKGAKGSALHEDIYVLHENGTLEIPVAQKDSTGTYTCVARNKLGMAKNEVHL
EIKDPTWIVKQPEYAVVQRGSMVSFECKVKHDHTLSLTVLWLKDNRELPSDERFTVDKDH
LVVADVSDDDSGTYTCVANTTLDSVSASAVLSVVAPTPTPAPVYDVPNPPFDLELTDQLD
KSVQLSWTPGDDNNSPITKFIIEYEDAMHKPGLWHHQTEVSGTQTTAQLKLSPYVNYSFR
VMAVNSIGKSLPSEASEQYLTKASEPDKNPTAVEGLGSEPDNLVITWKPLNGFESNGPGL
QYKVSWRQKDGDDEWTSVVVANVSKYIVSGTPTFVPYLIKVQALNDMGFAPEPAVVMGHS
GEDLPMVAPGNVRVNVVNSTLAEVHWDPVPLKSIRGHLQGYRIYYWKTQSSSKRNRRHIE
KKILTFQGSKTHGMLPGLEPFSHYTLNVRVVNGKGEGPASPDRVFNTPEGVPSAPSSLKI
VNPTLDSLTLEWDPPSHPNGILTEYTLKYQPINSTHELGPLVDLKIPANKTRWTLKNLNF
STRYKFYFYAQTSAGSGSQITEEAVTTVDEAGILPPDVGAGKVQAVNPRISNLTAAAAET
YANISWEYEGPEHVNFYVEYGVAGSKEEWRKEIVNGSRSFFGLKGLMPGTAYKVRVGAVG
DSGFVSSEDVFETGPAMASRQVDIATQGWFIGLMCAVALLILILLIVCFIRRNKGGKYPV
KEKEDAHADPEIQPMKEDDGTFGEYSDAEDHKPLKKGSRTPSDRTVKKEDSDDSLVDYGE
GVNGQFNEDGSFIGQYSGKKEKEPAEGNESSEAPSPVNAMNSFV
>sp|Q92905|CSN5_HUMAN COP9 signalosome complex subunit 5 OS=Homo sapiens OX=9606 GN=COPS5 PE=1 SV=4
MAASGSGMAQKTWELANNMQEAQSIDEIYKYDKKQQQEILAAKPWTKDHHYFKYCKISAL
ALLKMVMHARSGGNLEVMGLMLGKVDGETMIIMDSFALPVEGTETRVNAQAAAYEYMAAY

IENAKQVGRLENAIGWYHSHPGYGCWLSGIDVSTQMLNQQFQEPFVAVVIDPTRTISAGK
VNLGAFRTYPKGYKPPDEGPSEYQTIPLNKIEDFGVHCKQYYALEVSYFKSSLDRKLLEL
LWNKYWVNTLSSSSLLTNADYTTGQVFDLSEKLEQSEAQLGRGSFMLGLETHDRKSEDKL
AKATRDSCKTTIEAIHGLMSQVIKDKLFNQINIS
>sp|Q92973|TNPO1_HUMAN Transportin-1 OS=Homo sapiens OX=9606 GN=TNPO1 PE=1 SV=2
MVWDRQTKMEYEWKPDEQGLQQILQLLKESQSPDTTIQRTVQQKLEQLNQYPDFNNYLIF
VLTKLKSEDEPTRSLSGLILKNNVKAHFQNFPNGVTDFIKSECLNNIGDSSPLIRATVGI
LITTIASKGELQNWPDLLPKLCSLLDSEDYNTCEGAFGALQKICEDSAEILDSDVLDRPL
NIMIPKFLQFFKHSSPKIRSHAVACVNQFIISRTQALMLHIDSFIENLFALAGDEEPEVR
KNVCRALVMLLEVRMDRLLPHMHNIVEYMLQRTQDQDENVALEACEFWLTLAEQPICKDV
LVRHLPKLIPVLVNGMKYSDIDIILLKGDVEEDETIPDSEQDIRPRFHRSRTVAQQHDED
GIEEEDDDDDEIDDDDTISDWNLRKCSAAALDVLANVYRDELLPHILPLLKELLFHHEWV
VKESGILVLGAIAEGCMQGMIPYLPELIPHLIQCLSDKKALVRSITCWTLSRYAHWVVSQ
PPDTYLKPLMTELLKRILDSNKRVQEAACSAFATLEEEACTELVPYLAYILDTLVFAFSK
YQHKNLLILYDAIGTLADSVGHHLNKPEYIQMLMPPLIQKWNMLKDEDKDLFPLLECLSS
VATALQSGFLPYCEPVYQRCVNLVQKTLAQAMLNNAQPDQYEAPDKDFMIVALDLLSGLA
EGLGGNIEQLVARSNILTLMYQCMQDKMPEVRQSSFALLGDLTKACFQHVKPCIADFMPI
LGTNLNPEFISVCNNATWAIGEISIQMGIEMQPYIPMVLHQLVEIINRPNTPKTLLENTA
ITIGRLGYVCPQEVAPMLQQFIRPWCTSLRNIRDNEEKDSAFRGICTMISVNPSGVIQDF
IFFCDAVASWINPKDDLRDMFCKILHGFKNQVGDENWRRFSDQFPLPLKERLAAFYGV
>sp|Q93034|CUL5_HUMAN Cullin-5 OS=Homo sapiens OX=9606 GN=CUL5 PE=1 SV=4
MATSNLLKNKGSLQFEDKWDFMRPIVLKLLRQESVTKQQWFDLFSDVHAVCLWDDKGPAK
IHQALKEDILEFIKQAQARVLSHQDDTALLKAYIVEWRKFFTQCDILPKPFCQLEITLMG
KQGSNKKSNVEDSIVRKLMLDTWNESIFSNIKNRLQDSAMKLVHAERLGEAFDSQLVIGV
RESYVNLCSNPEDKLQIYRDNFEKAYLDSTERFYRTQAPSYLQQNGVQNYMKYADAKLKE
EEKRALRYLETRRECNSVEALMECCVNALVTSFKETILAECQGMIKRNETEKLHLMFSLM
DKVPNGIEPMLKDLEEHIISAGLADMVAAAETITTDSEKYVEQLLTLFNRFSKLVKEAFQ
DDPRFLTARDKAYKAVVNDATIFKLELPLKQKGVGLKTQPESKCPELLANYCDMLLRKTP
LSKKLTSEEIEAKLKEVLLVLKYVQNKDVFMRYHKAHLTRRLILDISADSEIEENMVEWL
REVGMPADYVNKLARMFQDIKVSEDLNQAFKEMHKNNKLALPADSVNIKILNAGAWSRSS
EKVFVSLPTELEDLIPEVEEFYKKNHSGRKLHWHHLMSNGIITFKNEVGQYDLEVTTFQL
AVLFAWNQRPREKISFENLKLATELPDAELRRTLWSLVAFPKLKRQVLLYEPQVNSPKDF
TEGTLFSVNQEFSLIKNAKVQKRGKINLIGRLQLTTERMREEENEGIVQLRILRTQEAII
QIMKMRKKISNAQLQTELVEILKNMFLPQKKMIKEQIEWLIEHKYIRRDESDINTFIYMA
>sp|Q93050|VPP1_HUMAN V-type proton ATPase 116 kDa subunit a 1 OS=Homo sapiens
OX=9606 GN=ATP6V0A1 PE=1 SV=3
MGELFRSEEMTLAQLFLQSEAAYCCVSELGELGKVQFRDLNPDVNVFQRKFVNEVRRCEE
MDRKLRFVEKEIRKANIPIMDTGENPEVPFPRDMIDLEANFEKIENELKEINTNQEALKR
NFLELTELKFILRKTQQFFDEMADPDLLEESSSLLEPSEMGRGTPLRLGFVAGVINRERI
PTFERMLWRVCRGNVFLRQAEIENPLEDPVTGDYVHKSVFIIFFQGDQLKNRVKKICEGF
RASLYPCPETPQERKEMASGVNTRIDDLQMVLNQTEDHRQRVLQAAAKNIRVWFIKVRKM
KAIYHTLNLCNIDVTQKCLIAEVWCPVTDLDSIQFALRRGTEHSGSTVPSILNRMQTNQT
PPTYNKTNKFTYGFQNIVDAYGIGTYREINPAPYTIITFPFLFAVMFGDFGHGILMTLFA
VWMVLRESRILSQKNENEMFSTVFSGRYIILLMGVFSMYTGLIYNDCFSKSLNIFGSSWS

VRPMFTYNWTEETLRGNPVLQLNPALPGVFGGPYPFGIDPIWNIATNKLTFLNSFKMKMS
VILGIIHMLFGVSLSLFNHIYFKKPLNIYFGFIPEIIFMTSLFGYLVILIFYKWTAYDAH
TSENAPSLLIHFINMFLFSYPESGYSMLYSGQKGIQCFLVVVALLCVPWMLLFKPLVLRR
QYLRRKHLGTLNFGGIRVGNGPTEEDAEIIQHDQLSTHSEDADEPSEDEVFDFGDTMVHQ
AIHTIEYCLGCISNTASYLRLWALSLAHAQLSEVLWTMVIHIGLSVKSLAGGLVLFFFFT
AFATLTVAILLIMEGLSAFLHALRLHWVEFQNKFYSGTGFKFLPFSFEHIREGKFEE
>sp|Q93088|BHMT1_HUMAN Betaine--homocysteine S-methyltransferase 1 OS=Homo sapiens OX=9606 GN=BHMT PE=1 SV=2
MPPVGGKKAKKGILERLNAGEIVIGDGGFVFALEKRGYVKAGPWTPEAAVEHPEAVRQLH
REFLRAGSNVMQTFTFYASEDKLENRGNYVLEKISGQEVNEAACDIARQVADEGDALVAG
GVSQTPSYLSCKSETEVKKVFLQQLEVFMKKNVDFLIAEYFEHVEEAVWAVETLIASGKP
VAATMCIGPEGDLHGVPPGECAVRLVKAGASIIGVNCHFDPTISLKTVKLMKEGLEAARL
KAHLMSQPLAYHTPDCNKQGFIDLPEFPFGLEPRVATRWDIQKYAREAYNLGVRYIGGCC
GFEPYHIRAIAEELAPERGFLPPASEKHGSWGSGLDMHTKPWVRARARKEYWENLRIASG
RPYNPSMSKPDGWGVTKGTAELMQQKEATTEQQLKELFEKQKFKSQ
>sp|Q969H8|MYDGF_HUMAN Myeloid-derived growth factor OS=Homo sapiens OX=9606 GN=MYDGF PE=1 SV=1
MAAPSGGWNGVGASLWAALLLGAVALRPAEAVSEPTTVAFDVRPGGVVHSFSHNVGPGDK
YTCMFTYASQGGTNEQWQMSLGTSEDHQHFTCTIWRPQGKSYLYFTQFKAEVRGAEIEYA
MAYSKAAFERESDVPLKTEEFEVTKTAVAHRPGAFKAELSKLVIVAKASRTEL
>sp|Q969Q0|RL36L_HUMAN Ribosomal protein eL42-like OS=Homo sapiens OX=9606 GN=RPL36AL PE=1 SV=3
MVNVPKTRRTFCKKCGKHQPHKVTQYKKGKDSLYAQGRRRYDRKQSGYGGQTKPIFRKKA
KTTKKIVLRLECVEPNCRSKRMLAIKRCKHFELGGDKKRKGQVIQF
>sp|Q969Q5|RAB24_HUMAN Ras-related protein Rab-24 OS=Homo sapiens OX=9606 GN=RAB24 PE=1 SV=1
MSGQRVDVKVVMLGKEYVGKTSLVERYVHDRFLVGPYQNTIGAAFVAKVMSVGDRTVTLG
IWDTAGSERYEAMSRIYYRGAKAAIVCYDLTDSSSFERAKFWVKELRSLEEGCQIYLCGT
KSDLLEEDRRRRRVDFHDVQDYADNIKAQLFETSSKTGQSVDELFQKVAEDYVSVAAFQV
MTEDKGVDLGQKPNPYFYSCCHH
>sp|Q969T9|WBP2_HUMAN WW domain-binding protein 2 OS=Homo sapiens OX=9606 GN=WBP2 PE=1 SV=1
MALNKNHSEGGGVIVNNTESILMSYDHVELTFNDMKNVPEAFKGTKKGTVYLTPYRVIFL
SKGKDAMQSFMMPFYLMKDCEIKQPVFGANYIKGTVKAEAGGGWEGSASYKLTFTAGGAI
EFGQRMLQVASQASRGEVPSGAYGYSYMPSGAYVYPPPVANGMYPCPPGYPYPPPPPEFY
PGPPMMDGAMGYVQPPPPPYPGPMEPPVSGPDVPSTPAAEAKAAEAAASAYYNPGNPHNV
YMPTSQPPPPPYYPPEDKKTQ
>sp|Q96C23|GALM_HUMAN Galactose mutarotase OS=Homo sapiens OX=9606 GN=GALM PE=1 SV=1
MASVTRAVFGELPSGGGTVEKFQLQSDLLRVDIISWGCTITALEVKDRQGRASDVVLGFA
ELEGYLQKQPYFGAVIGRVANRIAKGTFKVDGKEYHLAINKEPNSLHGGVRGFDKVLWTP
RVLSNGVQFSRISPDGEEGYPGELKVWVTYTLDGGELIVNYRAQASQATPVNLTNHSYFN
LAGQASPNINDHEVTIEADTYLPVDETLIPTGEVAPVQGTAFDLRKPVELGKHLQDFHLN
GFDHNFCLKGSKEKHFCARVHHAASGRVLEVYTTQPGVQFYTGNFLDGTLKGKNGAVYPK

HSGFCLETQNWPDAVNQPRFPPVLLRPGEEYDHTTWFKFSVA
>sp|Q96CC6|RHDF1_HUMAN Inactive rhomboid protein 1 OS=Homo sapiens OX=9606 GN=RHBDF1 PE=1 SV=2
MSEARRDSTSSLQRKKPPWLKLDIPSAVPLTAEEPSFLQPLRRQAFLRSVSMPAETAHIS
SPHHELRRPVLQRQTSITQTIRRGTADWFGVSKDSDSTQKWQRKSIRHCSQRYGKLKPQV
LRELDLPSQDNVSLTSTETPPPLYVGPCQLGMQKIIDPLARGRAFRVADDTAEGLSAPHT
PVTPGAASLCSFSSSRSGFHRLPRRRKRESVAKMSFRAAAALMKGRSVRDGTFRRAQRRS
FTPASFLEEDTTDFPDELDTSFFAREGILHEELSTYPDEVFESPSEAALKDWEKAPEQAD
LTGGALDRSELERSHLMLPLERGWRKQKEGAAAPQPKVRLRQEVVSTAGPRRGQRIAVPV
RKLFAREKRPYGLGMVGRLTNRTYRKRIDSFVKRQIEDMDDHRPFFTYWLTFVHSLVTIL
AVCIYGIAPVGFSQHETVDSVLRNRGVYENVKYVQQENFWIGPSSEALIHLGAKFSPCMR
QDPQVHSFIRSAREREKHSACCVRNDRSGCVQTSEEECSSTLAVWVKWPIHPSAPELAGH
KRQFGSVCHQDPRVCDEPSSEDPHEWPEDITKWPICTKNSAGNHTNHPHMDCVITGRPCC
IGTKGRCEITSREYCDFMRGYFHEEATLCSQVHCMDDVCGLLPFLNPEVPDQFYRLWLSL
FLHAGILHCLVSICFQMTVLRDLEKLAGWHRIAIIYLLSGVTGNLASAIFLPYRAEVGPA
GSQFGILACLFVELFQSWQILARPWRAFFKLLAVVLFLFTFGLLPWIDNFAHISGFISGL
FLSFAFLPYISFGKFDLYRKRCQIIIFQVVFLGLLAGLVVLFYVYPVRCEWCEFLTCIPF
TDKFCEKYELDAQLH
>sp|Q96CD0|FBXL8_HUMAN F-box/LRR-repeat protein 8 OS=Homo sapiens OX=9606 GN=FBXL8 PE=1 SV=1
MAEPGEGLPEEVLALIFRHLSLRDRAAAARVCRAWAAAATCSAVWHDTKISCECELEGML
PPYLSACLDHIHNLRLEFEPSRKPSRRAAIELLMVLAGRAPGLRGLRLECRGEKPLFDAG
RDVLEAVHAVCGAASQLRHLDLRRLSFTLDDALVLQAARSCPELHSLFLDNSTLVGSVGP
GSVLELLEACPRLRALGLHLASLSHAILEALAAPDRAPFALLALRCACPEDARASPLPNE
AWVALRRRHPGLAVELELEPALPAESVTRVLQPAVPVAALRLNLSGDTVGPVRFAAHHYA
ATLCALEVRAAASAELNAALEELAARCAALREVHCFCVVSHSVLDAFRAHCPRLRTYTLK
LTREPHPWRPTLVA
>sp|Q96CN7|ISOC1_HUMAN Isochorismatase domain-containing protein 1 OS=Homo sapiens OX=9606 GN=ISOC1 PE=1 SV=3
MAAAEPAVLALPNSGAGGAGAPSGTVPVLFCFSVFARPSSVPHGAGYELLIQKFLSLYGD
QIDMHRKFVVQLFAEEWGQYVDLPKGFAVSERCKVRLVPLQIQLTTLGNLTPSSTVFFCC
DMQERFRPAIKYFGDIISVGQRLLQGARILGIPVIVTEQYPKGLGSTVQEIDLTGVKLVL
PKTKFSMVLPEVEAALAEIPGVRSVVLFGVETHVCIQQTALELVGRGVEVHIVADATSSR
SMMDRMFALERLARTGIIVTTSEAVLLQLVADKDHPKFKEIQNLIKASAPESGLLSKV
>sp|Q96DA2|RB39B_HUMAN Ras-related protein Rab-39B OS=Homo sapiens OX=9606 GN=RAB39B PE=1 SV=1
MEAIWLYQFRLIVIGDSTVGKSCLIRRFTEGRFAQVSDPTVGVDFFSRLVEIEPGKRIKL
QIWDTAGQERFRSITRAYYRNSVGGLLLFDITNRRSFQNVHEWLEETKVHVQPYQIVFVL
VGHKCDLDTQRQVTRHEAEKLAAAYGMKYIETSARDAINVEKAFTDLTRDIYELVKRGEI
TIQEGWEGVKSGFVPNVVHSSEEVVKSERRCLC
>sp|Q96EF6|FBX17_HUMAN F-box only protein 17 OS=Homo sapiens OX=9606 GN=FBXO17 PE=1 SV=1
MGARLSRRRLPADPSLALDALPPELLVQVLSHVPPRSLVTRCRPVCRAWRDIVDGPTVWL
LQLARDRSAEGRALYAVAQRCLPSNEDKEEFPLCALARYCLRAPFGRNLIFNSCGEQGFR

GWEVEHGGNGWAIEKNLTPVPGAPSQTCFVTSFEWCSKRQLVDLVMEGVWQELLDSAQIE
ICVADWWGARENCGCVYQLRVRLLDVYEKEVVKFSASPDPVLQWTERGCRQVSHVFTNFG
KGIRYVSFEQYGRDVSSWVGHYGALVTHSSVRVRIRLS
>sp|Q96F07|CYFP2_HUMAN Cytoplasmic FMR1-interacting protein 2 OS=Homo sapiens
OX=9606 GN=CYFIP2 PE=1 SV=2
MTTHVTLEDALSNVDLLEELPLPDQQPCIEPPPSSIMYQANFDTNFEDRNAFVTGIARYI
EQATVHSSMNEMLEEGHEYAVMLYTWRSCSRAIPQVKCNEQPNRVEIYEKTVEVLEPEVT
KLMKFMYFQRKAIERFCSEVKRLCHAERRKDFVSEAYLLTLGKFINMFAVLDELKNMKCS
VKNDHSAYKRAAQFLRKMADPQSIQESQNLSMFLANHNRITQCLHQQLEVIPGYEELLAD
IVNICVDYYENKMYLTPSEKHMLLKVMGFGLYLMDGNVSNIYKLDAKKRINLSKIDKFFK
QLQVVPLFGDMQIELARYIKTSAHYEENKSKWTCTQSSISPQYNICEQMVQIRDDHIRFI
SELARYSNSEVVTGSGLDSQKSDEEYRELFDLALRGLQLLSKWSAHVMEVYSWKLVHPTD
KFCNKDCPGTAEEYERATRYNYTSEEKFAFVEVIAMIKGLQVLMGRMESVFNQAIRNTIY
AALQDFAQVTLREPLRQAVRKKKNVLISVLQAIRKTICDWEGGREPPNDPCLRGEKDPKG
GFDIKVPRRAVGPSSTQACQWSPRALFHPTGGTQGRRGCRSLLYMVRTMLESLIADKSGS
KKTLRSSLDGPIVLAIEDFHKQSFFFTHLLNISEALQQCCDLSQLWFREFFLELTMGRRI
QFPIEMSMPWILTDHILETKEPSMMEYVLYPLDLYNDSAYYALTKFKKQFLYDEIEAEVN
LCFDQFVYKLADQIFAYYKAMAGSVLLDKRFRAECKNYGVIIPYPPSNRYETLLKQRHVQ
LLGRSIDLNRLITQRISAAMYKSLDQAISRFESEDLTSIVELEWLLEINRLTHRLLCKHM
TLDSFDAMFREANHNVSAPYGRITLHVFWELNFDFLPNYCYNGSTNRFVRTAIPFTQEPQ
RDKPANVQPYYLYGSKPLNIAYSHIYSSYRNFVGPPHFKTICRLLGYQGIAVVMEELLKI
VKSLLQGTILQYVKTLIEVMPKICRLPRHEYGSPGILEFFHHQLKDIIEYAELKTDVFQS
LREVGNAILFCLLIEQALSQEEVCDLLHAAPFQNILPRVYIKEGERLEVRMKRLEAKYAP
LHLVPLIERLGTPQQIAIAREGDLLTKERLCCGLSMFEVILTRIRSYLQDPIWRGPPPTN
GVMHVDECVEFHRLWSAMQFVYCIPVGTNEFTAEQCFGDGLNWAGCSIIVLLGQQRRFDL
FDFCYHLLKVQRQDGKDEIIKNVPLKKMADRIRKYQILNNEVFAILNKYMKSVETDSSTV
EHVRCFQPPIHQSLATTC
>sp|Q96FV2|SCRN2_HUMAN Secernin-2 OS=Homo sapiens OX=9606 GN=SCRN2 PE=1 SV=3
MASSSPDSPCSCDCFVSVPPASAIPAVIFAKNSDRPRDEVQEVVFVPAGTHTPGSRLQCT
YIEVEQVSKTHAVILSRPSWLWGAEMGANEHGVCIGNEAVWTKEPVGEGEALLGMDLLRL
ALERSSSAQEALHVITGLLEHYGQGGNCLEDAAPFSYHSTFLLADRTEAWVLETAGRLWA
AQRIQEGARNISNQLSIGTDISAQHPELRTHAQAKGWWDGQGAFDFAQIFSLTQQPVRME
AAKARFQAGRELLRQRQGGITAEVMMGILRDKESGICMDSGGFRTTASMVSVLPQDPTQP
CVHFLTATPDPSRSVFKPFIFGMGVAQAPQVLSPTFGAQDPVRTLPRFQTQVDRRHTLYR
GHQAALGLMERDQDRGQQLQQKQQDLEQEGLEATQGLLAGEWAPPLWELGSLFQAFVKRE
SQAYA
>sp|Q96FW1|OTUB1_HUMAN Ubiquitin thioesterase OTUB1 OS=Homo sapiens OX=9606
GN=OTUB1 PE=1 SV=2
MAAEEPQQQKQEPLGSDSEGVNCLAYDEAIMAQQDRIQQEIAVQNPLVSERLELSVLYKE
YAEDDNIYQQKIKDLHKKYSYIRKTRPDGNCFYRAFGFSHLEALLDDSKELQRFKAVSAK
SKEDLVSQGFTEFTIEDFHNTFMDLIEQVEKQTSVADLLASFNDQSTSDYLVVYLRLLTS
GYLQRESKFFEHFIEGGRTVKEFCQQEVEPMCKESDHIHIIALAQALSVSIQVEYMDRGE
GGTTNPHIFPEGSEPKVYLLYRPGHYDILYK

>sp|Q96FZ7|CHMP6_HUMAN Charged multivesicular body protein 6 OS=Homo sapiens OX=9606 GN=CHMP6 PE=1 SV=3
MGNLFGRKKQSRVTEQDKAILQLKQQRDKLRQYQKRIAQQLERERALARQLLRDGRKERA
KLLLKKKRYQEQLLDRTENQISSLEAMVQSIEFTQIEMKVMEGLQFGNECLNKMHQVMSI
EEVERILDETQEAVEYQRQIDELLAGSFTQEDEDAILEELSAITQEQIELPEVPSEPLPE
KIPENVPVKARPRQAELVAAS
>sp|Q96HP0|DOCK6_HUMAN Dedicator of cytokinesis protein 6 OS=Homo sapiens OX=9606 GN=DOCK6 PE=1 SV=3
MAASERRAFAHKINRTVAAEVRKQVSRERSGSPHSSRRCSSSLGVPLTEVVEPLDFEDVL
LSRPPDAEPGPLRDLVEFPADDLELLLQPRECRTTEPGIPKDEKLDAQVRAAVEMYIEDW
VIVHRRYQYLSAAYSPVTTDTQRERQKGLPRQVFEQDASGDERSGPEDSNDSRRGSGSPE
DTPRSSGASSIFDLRNLAADSLLPSLLERAAPEDVDRRNETLRRQHRPPALLTLYPAPDE
DEAVERCSRPEPPREHFGQRILVKCLSLKFEIEIEPIFGILALYDVREKKKISENFYFDL
NSDSMKGLLRAHGTHPAISTLARSAIFSVTYPSPDIFLVIKLEKVLQQGDISECCEPYMV
LKEVDTAKNKEKLEKLRLAAEQFCTRLGRYRMPFAWTAVHLANIVSSAGQLDRDSDSEGE
RRPAWTDRRRRGPQDRASSGDDACSFSGFRPATLTVTNFFKQEAERLSDEDLFKFLADMR
RPSSLLRRLRPVTAQLKIDISPAPENPHFCLSPELLHIKPYPDPRGRPTKEILEFPAREV
YAPHTSYRNLLYVYPHSLNFSSRQGSVRNLAVRVQYMTGEDPSQALPVIFGKSSCSEFTR
EAFTPVVYHNKSPEFYEEFKLHLPACVTENHHLLFTFYHVSCQPRPGTALETPVGFTWIP
LLQHGRLRTGPFCLPVSVDQPPPSYSVLTPDVALPGMRWVDGHKGVFSVELTAVSSVHPQ
DPYLDKFFTLVHVLEEGAFPFRLKDTVLSEGNVEQELRASLAALRLASPEPLVAFSHHVL
DKLVRLVIRPPIISGQIVNLGRGAFEAMAHVVSLVHRSLEAAQDARGHCPQLAAYVHYAF
RLPGTEPSLPDGAPPVTVQAATLARGSGRPASLYLARSKSISSSNPDLAVAPGSVDDEVS
RILASKLLHEELALQWVVSSSAVREAILQHAWFFFQLMVKSMALHLLLGQRLDTPRKLRF
PGRFLDDITALVGSVGLEVITRVHKDVELAEHLNASLAFFLSDLLSLVDRGFVFSLVRAH
YKQVATRLQSSPNPAALLTLRMEFTRILCSHEHYVTLNLPCCPLSPPASPSPSVSSTTSQ
SSTFSSQAPDPKVTSMFELSGPFRQQHFLAGLLLTELALALEPEAEGAFLLHKKAISAVH
SLLCGHDTDPRYAEATVKARVAELYLPLLSIARDTLPRLHDFAEGPGQRSRLASMLDSDT
EGEGDIAGTINPSVAMAIAGGPLAPGSRASISQGPPTASRAGCALSAESSRTLLACVLWV
LKNTEPALLQRWATDLTLPQLGRLLDLLYLCLAAFEYKGKKAFERINSLTFKKSLDMKAR
LEEAILGTIGARQEMVRRSRERSPFGNPENVRWRKSVTHWKQTSDRVDKTKDEMEHEALV
EGNLATEASLVVLDTLEIIVQTVMLSEARESVLGAVLKVVLYSLGSAQSALFLQHGLATQ
RALVSKFPELLFEEDTELCADLCLRLLRHCGSRISTIRTHASASLYLLMRQNFEIGHNFA
RVKMQVTMSLSSLVGTTQNFSEEHLRRSLKTILTYAEEDMGLRDSTFAEQVQDLMFNLHM
ILTDTVKMKEHQEDPEMLIDLMYRIARGYQGSPDLRLTWLQNMAGKHAELGNHAEAAQCM
VHAAALVAEYLALLEDHRHLPVGCVSFQNISSNVLEESAISDDILSPDEEGFCSGKHFTE
LGLVGLLEQAAGYFTMGGLYEAVNEVYKNLIPILEAHRDYKKLAAVHGKLQEAFTKIMHQ
SSGWERVFGTYFRVGFYGAHFGDLDEQEFVYKEPSITKLAEISHRLEEFYTERFGDDVVE
IIKDSNPVDKSKLDSQKAYIQITYVEPYFDTYELKDRVTYFDRNYGLRTFLFCTPFTPDG
RAHGELPEQHKRKTLLSTDHAFPYIKTRIRVCHREETVLTPVEVAIEDMQKKTRELAFAT
EQDPPDAKMLQMVLQGSVGPTVNQGPLEVAQVFLAEIPEDPKLFRHHNKLRLCFKDFCKK
CEDALRKNKALIGPDQKEYHRELERNYCRLREALQPLLTQRLPQLMAPTPPGLRNSLNRA
SFRKADL

>sp|Q96IU4|ABHEB_HUMAN Putative protein-lysine deacylase ABHD14B OS=Homo sapiens OX=9606 GN=ABHD14B PE=1 SV=1
MAASVEQREGTIQVQGQALFFREALPGSGQARFSVLLLHGIRFSSETWQNLGTLHRLAQA
GYRAVAIDLPGLGHSKEAAAPAPIGELAPGSFLAAVVDALELGPPVVISPSLSGMYSLPF
LTAPGSQLPGFVPVAPICTDKINAANYASVKTPALIVYGDQDPMGQTSFEHLKQLPNHRV
LIMKGAGHPCYLDKPEEWHTGLLDFLQGLQ
>sp|Q96KP4|CNDP2_HUMAN Cytosolic non-specific dipeptidase OS=Homo sapiens OX=9606 GN=CNDP2 PE=1 SV=2
MAALTTLFKYIDENQDRYIKKLAKWVAIQSVSAWPEKRGEIRRMMEVAAADVKQLGGSVE
LVDIGKQKLPDGSEIPLPPILLGRLGSDPQKKTVCIYGHLDVQPAALEDGWDSEPFTLVE
RDGKLYGRGSTDDKGPVAGWINALEAYQKTGQEIPVNVRFCLEGMEESGSEGLDELIFAR
KDTFFKDVDYVCISDNYWLGKKKPCITYGLRGICYFFIEVECSNKDLHSGVYGGSVHEAM
TDLILLMGSLVDKRGNILIPGINEAVAAVTEEEHKLYDDIDFDIEEFAKDVGAQILLHSH
KKDILMHRWRYPSLSLHGIEGAFSGSGAKTVIPRKVVGKFSIRLVPNMTPEVVGEQVTSY
LTKKFAELRSPNEFKVYMGHGGKPWVSDFSHPHYLAGRRAMKTVFGVEPDLTREGGSIPV
TLTFQEATGKNVMLLPVGSADDGAHSQNEKLNRYNYIEGTKMLAAYLYEVSQLKD
>sp|Q96NU7|HUTI_HUMAN Probable imidazolonepropionase OS=Homo sapiens OX=9606 GN=AMDHD1 PE=1 SV=2
MASGHSLLLENAQQVVLVCARGERFLARDALRSLAVLEGASLVVGKDGFIKAIGPADVIQ
RQFSGETFEEIIDCSGKCILPGLVDAHTHPVWAGERVHEFAMKLAGATYMEIHQAGGGIH
FTVERTRQATEEELFRSLQQRLQCMMRAGTTLVECKSGYGLDLETELKMLRVIERARREL
DIGISATYCGAHSVPKGKTATEAADDIINNHLPKLKELGRNGEIHVDNIDVFCEKGVFDL
DSTRRILQRGKDIGLQINFHGDELHPMKAAELGAELGAQAISHLEEVSDEGIVAMATARC
SAILLPTTAYMLRLKQPRARKMLDEGVIVALGSDFNPNAYCFSMPMVMHLACVNMRMSMP
EALAAATINAAYALGKSHTHGSLEVGKQGDLIIINSSRWEHLIYQFGGHHELIEYVIAKG
KLIYKT
>sp|Q96QK1|VPS35_HUMAN Vacuolar protein sorting-associated protein 35 OS=Homo sapiens OX=9606 GN=VPS35 PE=1 SV=2
MPTTQQSPQDEQEKLLDEAIQAVKVQSFQMKRCLDKNKLMDALKHASNMLGELRTSMLSP
KSYYELYMAISDELHYLEVYLTDEFAKGRKVADLYELVQYAGNIIPRLYLLITVGVVYVK
SFPQSRKDILKDLVEMCRGVQHPLRGLFLRNYLLQCTRNILPDEGEPTDEETTGDISDSM
DFVLLNFAEMNKLWVRMQHQGHSRDREKRERERQELRILVGTNLVRLSQLEGVNVERYKQ
IVLTGILEQVVNCRDALAQEYLMECIIQVFPDEFHLQTLNPFLRACAELHQNVNVKNIII
ALIDRLALFAHREDGPGIPADIKLFDIFSQQVATVIQSRQDMPSEDVVSLQVSLINLAMK
CYPDRVDYVDKVLETTVEIFNKLNLEHIATSSAVSKELTRLLKIPVDTYNNILTVLKLKH
FHPLFEYFDYESRKSMSCYVLSNVLDYNTEIVSQDQVDSIMNLVSTLIQDQPDQPVEDPD
PEDFADEQSLVGRFIHLLRSEDPDQQYLILNTARKHFGAGGNQRIRFTLPPLVFAAYQLA
FRYKENSKVDDKWEKKCQKIFSFAHQTISALIKAELAELPLRLFLQGALAAGEIGFENHE
TVAYEFMSQAFSLYEDEISDSKAQLAAITLIIGTFERMKCFSEENHEPLRTQCALAASKL
LKKPDQGRAVSTCAHLFWSGRNTDKNGEELHGGKRVMECLKKALKIANQCMDPSLQVQLF
IEILNRYIYFYEKENDAVTIQVLNQLIQKIREDLPNLESSEETEQINKHFHNTLEHLRLR
RESPESEGPIYEGLIL
>sp|Q96S19|MTL26_HUMAN Methyltransferase-like 26 OS=Homo sapiens OX=9606 GN=METTL26 PE=1 SV=2

MLVAAAAERNKDPILHVLRQYLDPAQRGVRVLEVASGSGQHAAHFARAFPLAEWQPSDVD
QRCLDSIAATTQAQGLTNVKAPLHLDVTWGWEHWGGILPQSLDLLLCINMAHVSPLRCTE
GLFRAAGHLLKPRALLITYGPYAINGKISPQSNVDFDLMLRCRNPEWGLRDTALLEDLGK
ASGLLLERMVDMPANNKCLIFRKN
>sp|Q96S97|MYADM_HUMAN Myeloid-associated differentiation marker OS=Homo sapiens OX=9606 GN=MYADM PE=1 SV=2
MPVTVTRTTITTTTTSSSGLGSPMIVGSPRALTQPLGLLRLLQLVSTCVAFSLVASVGAW
TGSMGNWSMFTWCFCFSVTLIILIVELCGLQARFPLSWRNFPITFACYAALFCLSASIIY
PTTYVQFLSHGRSRDHAIAATFFSCIACVAYATEVAWTRARPGEITGYMATVPGLLKVLE
TFVACIIFAFISDPNLYQHQPALEWCVAVYAICFILAAIAILLNLGECTNVLPIPFPSFL
SGLALLSVLLYATALVLWPLYQFDEKYGGQPRRSRDVSCSRSHAYYVCAWDRRLAVAILT
AINLLAYVADLVHSAHLVFVKV
>sp|Q99426|TBCB_HUMAN Tubulin-folding cofactor B OS=Homo sapiens OX=9606 GN=TBCB PE=1 SV=2
MEVTGVSAPTVTVFISSSLNTFRSEKRYSRSLTIAEFKCKLELLVGSPASCMELELYGVD
DKFYSKLDQEDALLGSYPVDDGCRIHVIDHSGARLGEYEDVSRVEKYTISQEAYDQRQDT
VRSFLKRSKLGRYNEEERAQQEAEAAQRLAEEKAQASSIPVGSRCEVRAAGQSPRRGTVM
YVGLTDFKPGYWIGVRYDEPLGKNDGSVNGKRYFECQAKYGAFVKPAVVTVGDFPEEDYG
LDEI
>sp|Q99447|PCY2_HUMAN Ethanolamine-phosphate cytidylyltransferase OS=Homo sapiens OX=9606 GN=PCYT2 PE=1 SV=1
MIRNGRGAAGGAEQPGPGGRRAVRVWCDGCYDMVHYGHSNQLRQARAMGDYLIVGVHTDE
EIAKHKGPPVFTQEERYKMVQAIKWVDEVVPAAPYVTTLETLDKYNCDFCVHGNDITLTV
DGRDTYEEVKQAGRYRECKRTQGVSTTDLVGRMLLVTKAHHSSQEMSSEYREYADSFGKC
PGGRNPWTGVSQFLQTSQKIIQFASGKEPQPGETVIYVAGAFDLFHIGHVDFLEKVHRLA
ERPYIIAGLHFDQEVNHYKGKNYPIMNLHERTLSVLACRYVSEVVIGAPYAVTAELLSHF
KVDLVCHGKTEIIPDRDGSDPYQEPKRRGIFRQIDSGSNLTTDLIVQRIITNRLEYEARN
QKKEAKELAFLEAARQQAAQPLGERDGDF
>sp|Q99497|PARK7_HUMAN Parkinson disease protein 7 OS=Homo sapiens OX=9606 GN=PARK7 PE=1 SV=2
MASKRALVILAKGAEEMETVIPVDVMRRAGIKVTVAGLAGKDPVQCSRDVVICPDASLED
AKKEGPYDVVVLPGGNLGAQNLSESAAVKEILKEQENRKGLIAAICAGPTALLAHEIGFG
SKVTTHPLAKDKMMNGGHYTYSENRVEKDGLILTSRGPGTSFEFALAIVEALNGKEVAAQ
VKAPLVLKD
>sp|Q99536|VAT1_HUMAN Synaptic vesicle membrane protein VAT-1 homolog OS=Homo sapiens OX=9606 GN=VAT1 PE=1 SV=2
MSDEREVAEAATGEDASSPPPKTEAASDPQHPAASEGAAAAAASPPLLRCLVLTGFGGYD
KVKLQSRPAAPPAPGPGQLTLRLRACGLNFADLMARQGLYDRLPPLPVTPGMEGAGVVIA
VGEGVSDRKAGDRVMVLNRSGMWQEEVTVPSVQTFLIPEAMTFEEAAALLVNYITAYMVL
FDFGNLQPGHSVLVHMAAGGVGMAAVQLCRTVENVTVFGTASASKHEALKENGVTHPIDY
HTTDYVDEIKKISPKGVDIVMDPLGGSDTAKGYNLLKPMGKVVTYGMANLLTGPKRNLMA
LARTWWNQFSVTALQLLQANRAVCGFHLGYLDGEVELVSGVVARLLALYNQGHIKPHIDS
VWPFEKVADAMKQMQEKKNVGKVLLVPGPEKEN
>sp|Q99569|PKP4_HUMAN Plakophilin-4 OS=Homo sapiens OX=9606 GN=PKP4 PE=1 SV=2

MPAPEQASLVEEGQPQTRQEAASTGPGMEPETTATTILASVKEQELQFQRLTRELEVERQ
IVASQLERCRLGAESPSIASTSSTEKSFPWRSTDVPNTGVSKPRVSDAVQPNNYLIRTEP
EQGTLYSPEQTSLHESEGSLGNSRSSTQMNSYSDSGYQEAGSFHNSQNVSKADNRQQHSF
IGSTNNHVVRNSRAEGQTLVQPSVANRAMRRVSSVPSRAQSPSYVISTGVSPSRGSLRTS
LGSGFGSPSVTDPRPLNPSAYSSTTLPAARAASPYSQRPASPTAIRRIGSVTSRQTSNPN
GPTPQYQTTARVGSPLTLTDAQTRVASPSQGQVGSSSPKRSGMTAVPQHLGPSLQRTVHD
MEQFGQQQYDIYERMVPPRPDSLTGLRSSYASQHSQLGQDLRSAVSPDLHITPIYEGRTY
YSPVYRSPNHGTVELQGSQTALYRTGSVGIGNLQRTSSQRSTLTYQRNNYALNTTATYAE
PYRPIQYRVQECNYNRLQHAVPADDGTTRSPSIDSIQKDPREFAWRDPELPEVIHMLQHQ
FPSVQANAAAYLQHLCFGDNKVKMEVCRLGGIKHLVDLLDHRVLEVQKNACGALRNLVFG
KSTDENKIAMKNVGGIPALLRLLRKSIDAEVRELVTGVLWNLSSCDAVKMTIIRDALSTL
TNTVIVPHSGWNNSSFDDDHKIKFQTSLVLRNTTGCLRNLSSAGEEARKQMRSCEGLVDS
LLYVIHTCVNTSDYDSKTVENCVCTLRNLSYRLELEVPQARLLGLNELDDLLGKESPSKD
SEPSCWGKKKKKKKRTPQEDQWDGVGPIPGLSKSPKGVEMLWHPSVVKPYLTLLAESSNP
ATLEGSAGSLQNLSAGNWKFAAYIRAAVRKEKGLPILVELLRMDNDRVVSSVATALRNMA
LDVRNKELIGKYAMRDLVNRLPGGNGPSVLSDETMAAICCALHEVTSKNMENAKALADSG
GIEKLVNITKGRGDRSSLKVVKAAAQVLNTLWQYRDLRSIYKKDGWNQNHFITPVSTLER
DRFKSHPSLSTTNQQMSPIIQSVGSTSSSPALLGIRDPRSEYDRTQPPMQYYNSQGDATH
KGLYPGSSKPSPIYISSYSSPAREQNRRLQHQQLYYSQDDSNRKNFDAYRLYLQSPHSYE
DPYFDDRVHFPASTDYSTQYGLKSTTNYVDFYSTKRPSYRAEQYPGSPDSWV
>sp|Q99598|TSNAX_HUMAN Translin-associated protein X OS=Homo sapiens OX=9606
GN=TSNAX PE=1 SV=1
MSNKEGSGGFRKRKHDNFPHNQRREGKDVNSSSPVMLAFKSFQQELDARHDKYERLVKLS
RDITVESKRTIFLLHRITSAPDMEDILTESEIKLDGVRQKIFQVAQELSGEDMHQFHRAI
TTGLQEYVEAVSFQHFIKTRSLISMDEINKQLIFTTEDNGKENKTPSSDAQDKQFGTWRL
RVTPVDYLLGVADLTGELMRMCINSVGNGDIDTPFEVSQFLRQVYDGFSFIGNTGPYEVS
KKLYTLKQSLAKVENACYALKVRGSEIPKHMLADVFSVKTEMIDQEEGIS
>sp|Q99613|EIF3C_HUMAN Eukaryotic translation initiation factor 3 subunit C OS=Homo
sapiens OX=9606 GN=EIF3C PE=1 SV=1
MSRFFTTGSDSESESSLSGEELVTKPVGGNYGKQPLLLSEDEEDTKRVVRSAKDKRFEEL
TNLIRTIRNAMKIRDVTKCLEEFELLGKAYGKAKSIVDKEGVPRFYIRILADLEDYLNEL
WEDKEGKKKMNKNNAKALSTLRQKIRKYNRDFESHITSYKQNPEQSADEDAEKNEEDSEG
SSDEDEDEDGVSAATFLKKKSEAPSGESRKFLKKMDDEDEDSEDSEDDEDWDTGSTSSDS
DSEEEEGKQTALASRFLKKAPTTDEDKKAAEKKREDKAKKKHDRKSKRLDEEEEDNEGGE
WERVRGGVPLVKEKPKMFAKGTEITHAVVIKKLNEILQARGKKGTDRAAQIELLQLLVQI
AAENNLGEGVIVKIKFNIIASLYDYNPNLATYMKPEMWGKCLDCINELMDILFANPNIFV
GENILEESENLHNADQPLRVRGCILTLVERMDEEFTKIMQNTDPHSQEYVEHLKDEAQVC
AIIERVQRYLEEKGTTEEVCRIYLLRILHTYYKFDYKAHQRQLTPPEGSSKSEQDQAENE
GEDSAVLMERLCKYIYAKDRTDRIRTCAILCHIYHHALHSRWYQARDLMLMSHLQDNIQH
ADPPVQILYNRTMVQLGICAFRQGLTKDAHNALLDIQSSGRAKELLGQGLLLRSLQERNQ
EQEKVERRRQVPFHLHINLELLECVYLVSAMLLEIPYMAAHESDARRRMISKQFHHQLRV
GERQPLLGPPESMREHVVAASKAMKMGDWKTCHSFIINEKMNGKVWDLFPEADKVRTMLV
RKIQEESLRTYLFTYSSVYDSISMETLSDMFELDLPTVHSIISKMIINEELMASLDQPTQ
TVVMHRTEPTAQQNLALQLAEKLGSLVENNERVFDHKQGTYGGYFRDQKDGYRKNEGYMR

RGGYRQQQSQTAY
>sp|Q99685|MGLL_HUMAN Monoglyceride lipase OS=Homo sapiens OX=9606 GN=MGLL PE=1 SV=2
MPEESSPRRTPQSIPYQDLPHLVNADGQYLFCRYWKPTGTPKALIFVSHGAGEHSGRYEE
LARMLMGLDLLVFAHDHVGHGQSEGERMVVSDFHVFVRDVLQHVDSMQKDYPGLPVFLLG
HSMGGAIAILTAAERPGHFAGMVLISPLVLANPESATTFKVLAAKVLNLVLPNLSLGPID
SSVLSRNKTEVDIYNSDPLICRAGLKVCFGIQLLNAVSRVERALPKLTVPFLLLQGSADR
LCDSKGAYLLMELAKSQDKTLKIYEGAYHVLHKELPEVTNSVFHEINMWVSQRTATAGTA
SPP
>sp|Q99733|NP1L4_HUMAN Nucleosome assembly protein 1-like 4 OS=Homo sapiens OX=9606 GN=NAP1L4 PE=1 SV=1
MADHSFSDGVPSDSVEAAKNASNTEKLTDQVMQNPRVLAALQERLDNVPHTPSSYIETLP
KAVKRRINALKQLQVRCAHIEAKFYEEVHDLERKYAALYQPLFDKRREFITGDVEPTDAE
SEWHSENEEEEKLAGDMKSKVVVTEKAAATAEEPDPKGIPEFWFTIFRNVDMLSELVQEY
DEPILKHLQDIKVKFSDPGQPMSFVLEFHFEPNDYFTNSVLTKTYKMKSEPDKADPFSFE
GPEIVDCDGCTIDWKKGKNVTVKTIKKKQKHKGRGTVRTITKQVPNESFFNFFNPLKASG
DGESLDEDSEFTLASDFEIGHFFRERIVPRAVLYFTGEAIEDDDNFEEGEEGEEEELEGD
EEGEDEDDAEINPKV
>sp|Q99832|TCPH_HUMAN T-complex protein 1 subunit eta OS=Homo sapiens OX=9606 GN=CCT7 PE=1 SV=2
MMPTPVILLKEGTDSSQGIPQLVSNISACQVIAEAVRTTLGPRGMDKLIVDGRGKATISN
DGATILKLLDVVHPAAKTLVDIAKSQDAEVGDGTTSVTLLAAEFLKQVKPYVEEGLHPQI
IIRAFRTATQLAVNKIKEIAVTVKKADKVEQRKLLEKCAMTALSSKLISQQKAFFAKMVV
DAVMMLDDLLQLKMIGIKKVQGGALEDSQLVAGVAFKKTFSYAGFEMQPKKYHNPKIALL
NVELELKAEKDNAEIRVHTVEDYQAIVDAEWNILYDKLEKIHHSGAKVVLSKLPIGDVAT
QYFADRDMFCAGRVPEEDLKRTMMACGGSIQTSVNALSADVLGRCQVFEETQIGGERYNF
FTGCPKAKTCTFILRGGAEQFMEETERSLHDAIMIVRRAIKNDSVVAGGGAIEMELSKYL
RDYSRTIPGKQQLLIGAYAKALEIIPRQLCDNAGFDATNILNKLRARHAQGGTWYGVDIN
NEDIADNFEAFVWEPAMVRINALTAASEAACLIVSVDETIKNPRSTVDAPTAAGRGRGRG
RPH
>sp|Q99880|H2B1L_HUMAN Histone H2B type 1-L OS=Homo sapiens OX=9606 GN=H2BC13 PE=1 SV=3
MPELAKSAPAPKKGSKKAVTKAQKKDGKKRKRSRKESYSVYVYKVLKQVHPDTGISSKAM
GIMNSFVNDIFERIASEASRLAHYNKRSTITSREIQTAVRLLLPGELAKHAVSEGTKAVT
KYTSSK
>sp|Q99944|EGFL8_HUMAN Epidermal growth factor-like protein 8 OS=Homo sapiens OX=9606 GN=EGFL8 PE=1 SV=1
MGSRAELCTLLGGFSFLLLLIPGEGAKGGSLRESQGVCSKQTLVVPLHYNESYSQPVYKP
YLTLCAGRRICSTYRTMYRVMWREVRREVQQTHAVCCQGWKKRHPGALTCEAICAKPCLN
GGVCVRPDQCECAPGWGGKHCHVDVDECRTSITLCSHHCFNTAGSFTCGCPHDLVLGVDG
RTCMEGSPEPPTSASILSVAVREAEKDERALKQEIHELRGRLERLEQWAGQAGAWVRAVL
PVPPEELQPEQVAELWGRGDRIESLSDQVLLLEERLGACSCEDNSLGLGVNHR
>sp|Q99969|RARR2_HUMAN Retinoic acid receptor responder protein 2 OS=Homo sapiens OX=9606 GN=RARRES2 PE=1 SV=1

MRRLLIPLALWLGAVGVGVAELTEAQRRGLQVALEEFHKHPPVQWAFQETSVESAVDTPF
PAGIFVRLEFKLQQTSCRKRDWKKPECKVRPNGRKRKCLACIKLGSEDKVLGRLVHCPIE
TQVLREAEEHQETQCLRVQRAGEDPHSFYFPGQFAFSKALPRS
>sp|Q9BQA1|MEP50_HUMAN Methylosome protein WDR77 OS=Homo sapiens OX=9606 GN=WDR77 PE=1 SV=1
MRKETPPPLVPPAAREWNLPPNAPACMERQLEAARYRSDGALLLGASSLSGRCWAGSLWL
FKDPCAAPNEGFCSAGVQTEAGVADLTWVGERGILVASDSGAVELWELDENETLIVSKFC
KYEHDDIVSTVSVLSSGTQAVSGSKDICIKVWDLAQQVVLSSYRAHAAQVTCVAASPHKD
SVFLSCSEDNRILLWDTRCPKPASQIGCSAPGYLPTSLAWHPQQSEVFVFGDENGTVSLV
DTKSTSCVLSSAVHSQCVTGLVFSPHSVPFLASLSEDCSLAVLDSSLSELFRSQAHRDFV
RDATWSPLNHSLLTTVGWDHQVVHHVVPTEPLPAPGPASVTE
>sp|Q9BQS8|FYCO1_HUMAN FYVE and coiled-coil domain-containing protein 1 OS=Homo sapiens OX=9606 GN=FYCO1 PE=1 SV=3
MASTNAESQLQRIIRDLQDAVTELSKEFQEAGEPITDDSTSLHKFSYKLEYLLQFDQKEK
ATLLGNKKDYWDYFCACLAKVKGANDGIRFVKSISELRTSLGKGRAFIRYSLVHQRLADT
LQQCFMNTKVTSDWYYARSPFLQPKLSSDIVGQLYELTEVQFDLASRGFDLDAAWPTFAR
RTLTTGSSAYLWKPPSRSSSMSSLVSSYLQTQEMVSNFDLNSPLNNEALEGFDEMRLELD
QLEVREKQLRERMQQLDRENQELRAAVSQQGEQLQTERERGRTAAEDNVRLTCLVAELQK
QWEVTQATQNTVKELQTCLQGLELGAAEKEEDYHTALRRLESMLQPLAQELEATRDSLDK
KNQHLASFPGWLAMAQQKADTASDTKGRQEPIPSDAAQEMQELGEKLQALERERTKVEEV
NRQQSAQLEQLVKELQLKEDARASLERLVKEMAPLQEELSGKGQEADQLWRRLQELLAHT
SSWEEELAELRREKKQQQEEKELLEQEVRSLTRQLQFLETQLAQVSQHVSDLEEQKKQLI
QDKDHLSQQVGMLERLAGPPGPELPVAGEKNEALVPVNSSLQEAWGKPEEEQRGLQEAQL
DDTKVQEGSQEEELRQANRELEKELQNVVGRNQLLEGKLQALQADYQALQQRESAIQGSL
ASLEAEQASIRHLGDQMEASLLAVRKAKEAMKAQMAEKEAILQSKEGECQQLREEVEQCQ
QLAEARHRELRALESQCQQQTQLIEVLTAEKGQQGVGPPTDNEARELAAQLALSQAQLEV
HQGEVQRLQAQVVDLQAKMRAALDDQDKVQSQLSMAEAVLREHKTLVQQLKEQNEALNRA
HVQELLQCSEREGALQEERADEAQQREEELRALQEELSQAKCSSEEAQLEHAELQEQLHR
ANTDTAELGIQVCALTVEKERVEEALACAVQELQDAKEAASREREGLERQVAGLQQEKES
LQEKLKAAKAAAGSLPGLQAQLAQAEQRAQSLQEAAHQELNTLKFQLSAEIMDYQSRLKN
AGEECKSLRGQLEEQGRQLQAAEEAVEKLKATQADMGEKLSCTSNHLAECQAAMLRKDKE
GAALREDLERTQKELEKATTKIQEYYNKLCQEVTNRERNDQKMLADLDDLNRTKKYLEER
LIELLRDKDALWQKSDALEFQQKLSAEERWLGDTEANHCLDCKREFSWMVRRHHCRICGR
IFCYYCCNNYVLSKHGGKKERCCRACFQKLSEGPGSPDSSGSGTSQGEPSPALSPASPGP
QATGGQGANTDYRPPDDAVFDIITDEELCQIQESGSSLPETPTETDSLDPNAAEQDTTST
SLTPEDTEDMPVGQDSEICLLKSGELMIKVPLTVDEIASFGEGSRELFVRSSTYSLIPIT
VAEAGLTISWVFSSDPKSISFSVVFQEAEDTPLDQCKVLIPTTRCNSHKENIQGQLKVRT
PGIYMLIFDNTFSRFVSKKVFYHLTVDRPVIYDGSDFL
>sp|Q9BRJ7|TIRR_HUMAN Tudor-interacting repair regulator protein OS=Homo sapiens OX=9606 GN=NUDT16L1 PE=1 SV=1
MSTAAVPELKQISRVEAMRLGPGWSHSCHAMLYAANPGQLFGRIPMRFSVLMQMRFDGLL
GFPGGFVDRRFWSLEDGLNRVLGLGLGCLRLTEADYLSSHLTEGPHRVVAHLYARQLTLE
QLHAVEISAVHSRDHGLEVLGLVRVPLYTQKDRVGGFPNFLSNAFVSTAKCQLLFALKVL
NMMPEEKLVEALAAATEKQKKALEKLLPASS

>sp|Q9BRP4|PAAF1_HUMAN Proteasomal ATPase-associated factor 1 OS=Homo sapiens OX=9606 GN=PAAF1 PE=1 SV=2
MAAPLRIQSDWAQALRKDEGEAWLSCHPPGKPSLYGSLTCQGIGLDGIPEVTASEGFTVN
EINKKSIHISCPKENASSKFLAPYTTFSRIHTKSITCLDISSRGGLGVSSSTDGTMKIWQ
ASNGELRRVLEGHVFDVNCCRFFPSGLVVLSGGMDAQLKIWSAEDASCVVTFKGHKGGIL
DTAIVDRGRNVVSASRDGTARLWDCGRSACLGVLADCGSSINGVAVGAADNSINLGSPEQ
MPSEREVGTEAKMLLLAREDKKLQCLGLQSRQLVFLFIGSDAFNCCTFLSGFLLLAGTQD
GNIYQLDVRSPRAPVQVIHRSGAPVLSLLSVRDGFIASQGDGSCFIVQQDLDYVTELTGA
DCDPVYKVATWEKQIYTCCRDGLVRRYQLSDL
>sp|Q9BRX2|PELO_HUMAN Protein pelota homolog OS=Homo sapiens OX=9606 GN=PELO PE=1 SV=2
MKLVRKNIEKDNAGQVTLVPEEPEDMWHTYNLVQVGDSLRASTIRKVQTESSTGSVGSNR
VRTTLTLCVEAIDFDSQACQLRVKGTNIQENEYVKMGAYHTIELEPNRQFTLAKKQWDSV
VLERIEQACDPAWSADVAAVVMQEGLAHICLVTPSMTLTRAKVEVNIPRKRKGNCSQHDR
ALERFYEQVVQAIQRHIHFDVVKCILVASPGFVREQFCDYLFQQAVKTDNKLLLENRSKF
LQVHASSGHKYSLKEALCDPTVASRLSDTKAAGEVKALDDFYKMLQHEPDRAFYGLKQVE
KANEAMAIDTLLISDELFRHQDVATRSRYVRLVDSVKENAGTVRIFSSLHVSGEQLSQLT
GVAAILRFPVPELSDQEGDSSSEED
>sp|Q9BTE6|AASD1_HUMAN Alanyl-tRNA editing protein Aarsd1 OS=Homo sapiens OX=9606 GN=AARSD1 PE=1 SV=2
MAFWCQRDSYAREFTTTVVSCCPAELQTEGSNGKKEVLSGFQVVLEDTVLFPEGGGQPDD
RGTINDISVLRVTRRGEQADHFTQTPLDPGSQVLVRVDWERRFDHMQQHSGQHLITAVAD
HLFKLKTTSWELGRFRSAIELDTPSMTAEQVAAIEQSVNEKIRDRLPVNVRELSLDDPEV
EQVSGRGLPDDHAGPIRVVNIEGVDSNMCCGTHVSNLSDLQVIKILGTEKGKKNRTNLIF
LSGNRVLKWMERSHGTEKALTALLKCGAEDHVEAVKKLQNSTKILQKNNLNLLRDLAVHI
AHSLRNSPDWGGVVILHRKEGDSEFMNIIANEIGSEETLLFLTVGDEKGGGLFLLAGPPA
SVETLGPRVAEVLEGKGAGKKGRFQGKATKMSRRMEAQALLQDYISTQSAKE
>tr|Q9BTI6|Q9BTI6_HUMAN Flotillin OS=Homo sapiens OX=9606 GN=FLOT2 PE=1 SV=1
MGNCHTVGPNEALVVSGGCCGSDYKQYVFGGWAWAWWCISDTQRISLEIMTLQPRCEDVE
TAEGVALTVTGVAQVKIMTEKELLAVACEQFLGKNVQDIKNVVLQTLEGHLRSILGTLTV
EQIYQDRDQFAKLVREVAAPDVGRMGIEILSFTIKDVYDKVDYLSSLGKTQTAVVQRDAD
IGVAEAERDAGIREAECKKEMLDVKFMADTKIADSKRAFELQKSAFSEEVNIKTAEAQLA
YELQGAREQQKIRQEEIEIEVVQRKKQIAVEAQEILRTDKELIATVRRPAEAEAHRIQQI
AEGEKVKQVLLAQAEAEKIRKIGEAEATVIEAMGKAEAERMKLKAEAYQKYGDAAKMALV
LEALPQIAAKIAAPLPVPSGKIKNS
>sp|Q9BTU6|P4K2A_HUMAN Phosphatidylinositol 4-kinase type 2-alpha OS=Homo sapiens OX=9606 GN=PI4K2A PE=1 SV=1
MDETSPLVSPERAQPPDYTFPSGSGAHFPQVPGGAVRVAAAAGSGPSPPGSPGHDRERQP
LLDRARGAAAQGQTQTVAAQAQALAAQAAAAAHAAQAHRERNEFPEDPEFEAVVRQAELA
IERCIFPERIYQGSSGSYFVKDPQGRIIAVFKPKNEEPYGHLNPKWTKWLQKLCCPCCFG
RDCLVLNQGYLSEAGASLVDQKLELNIVPRTKVVYLASETFNYSAIDRVKSRGKRLALEK
VPKVGQRFNRIGLPPKVGSFQLFVEGYKDADYWLRRFEAEPLPENTNRQLLLQFERLVVL
DYIIRNTDRGNDNWLIKYDCPMDSSSSRDTDWVVVKEPVIKVAAIDNGLAFPLKHPDSWR
AYPFYWAWLPQAKVPFSQEIKDLILPKISDPNFVKDLEEDLYELFKKDPGFDRGQFHKQI

AVMRGQILNLTQALKDNKSPLHLVQMPPVIVETARSHQRSSSESYTQSFQSRKPFFSWW
>sp|Q9BTV4|TMM43_HUMAN Transmembrane protein 43 OS=Homo sapiens OX=9606 GN=TMEM43 PE=1 SV=1
MAANYSSTSTRREHVKVKTSSQPGFLERLSETSGGMFVGLMAFLLSFYLIFTNEGRALKT
ATSLAEGLSLVVSPDSIHSVAPENEGRLVHIIGALRTSKLLSDPNYGVHLPAVKLRRHVE
MYQWVETEESREYTEDGQVKKETRYSYNTEWRSEIINSKNFDREIGHKNPSAMAVESFMA
TAPFVQIGRFFLSSGLIDKVDNFKSLSLSKLEDPHVDIIRRGDFFYHSENPKYPEVGDLR
VSFSYAGLSGDDPDLGPAHVVTVIARQRGDQLVPFSTKSGDTLLLLHHGDFSAEEVFHRE
LRSNSMKTWGLRAAGWMAMFMGLNLMTRILYTLVDWFPVFRDLVNIGLKAFAFCVATSLT
LLTVAAGWLFYRPLWALLIAGLALVPILVARTRVPAKKLE
>sp|Q9BTW9|TBCD_HUMAN Tubulin-specific chaperone D OS=Homo sapiens OX=9606 GN=TBCD PE=1 SV=2
MALSDEPAAGGPEEEAEDETLAFGAALEAFGESAETRALLGRLREVHGGGAEREVALERF
RVIMDKYQEQPHLLDPHLEWMMNLLLDIVQDQTSPASLVHLAFKFLYIITKVRGYKTFLR
LFPHEVADVEPVLDLVTIQNPKDHEAWETRYMLLLWLSVTCLIPFDFSRLDGNLLTQPGQ
ARMSIMDRILQIAESYLIVSDKARDAAAVLVSRFITRPDVKQSKMAEFLDWSLCNLARSS
FQTMQGVITMDGTLQALAQIFKHGKREDCLPYAATVLRCLDGCRLPESNQTLLRKLGVKL
VQRLGLTFLKPKVAAWRYQRGCRSLAANLQLLTQGQSEQKPLILTEDDDEDDDVPEGVER
VIEQLLVGLKDKDTVVRWSAAKGIGRMAGRLPRALADDVVGSVLDCFSFQETDKAWHGGC
LALAELGRRGLLLPSRLVDVVAVILKALTYDEKRGACSVGTNVRDAACYVCWAFARAYEP
QELKPFVTAISSALVIAAVFDRDINCRRAASAAFQENVGRQGTFPHGIDILTTADYFAVG
NRSNCFLVISVFIAGFPEYTQPMIDHLVTMKISHWDGVIRELAARALHNLAQQAPEFSAT
QVFPRLLSMTLSPDLHMRHGSILACAEVAYALYKLAAQENRPVTDHLDEQAVQGLKQIHQ
QLYDRQLYRGLGGQLMRQAVCVLIEKLSLSKMPFRGDTVIDGWQWLINDTLRHLHLISSH
SRQQMKDAAVSALAALCSEYYMKEPGEADPAIQEELITQYLAELRNPEEMTRCGFSLALG
ALPGFLLKGRLQQVLTGLRAVTHTSPEDVSFAESRRDGLKAIARICQTVGVKAGAPDEAV
CGENVSQIYCALLGCMDDYTTDSRGDVGTWVRKAAMTSLMDLTLLLARSQPELIEAHTCE
RIMCCVAQQASEKIDRFRAHAASVFLTLLHFDSPPIPHVPHRGELEKLFPRSDVASVNWS
APSQAFPRITQLLGLPTYRYHVLLGLVVSLGGLTESTIRHSTQSLFEYMKGIQSDPQALG
SFSGTLLQIFEDNLLNERVSVPLLKTLDHVLTHGCFDIFTTEEDHPFAVKLLALCKKEIK
NSKDIQKLLSGIAVFCEMVQFPGDVRRQALLQLCLLLCHRFPLIRKTTASQVYETLLTYS
DVVGADVLDEVVTVLSDTAWDAELAVVREQRNRLCDLLGVPRPQLVPQPGAC
>sp|Q9BU40|CRDL1_HUMAN Chordin-like protein 1 OS=Homo sapiens OX=9606 GN=CHRDL1 PE=1 SV=2
MRKKWKMGGMKYIFSLLFFLLLEGGKTEQVKHSETYCMFQDKKYRVGERWHPYLEPYGLV
YCVNCICSENGNVLCSRVRCPNVHCLSPVHIPHLCCPRCPDSLPPVNNKVTSKSCEYNGT
TYQHGELFVAEGLFQNRQPNQCTQCSCSEGNVYCGLKTCPKLTCAFPVSVPDSCCRVCRG
DGELSWEHSDGDIFRQPANREARHSYHRSHYDPPPSRQAGGLSRFPGARSHRGALMDSQQ
ASGTIVQIVINNKHKHGQVCVSNGKTYSHGESWHPNLRAFGIVECVLCTCNVTKQECKKI
HCPNRYPCKYPQKIDGKCCKVCPGKKAKELPGQSFDNKGYFCGEETMPVYESVFMEDGET
TRKIALETERPPQVEVHVWTIRKGILQHFHIEKISKRMFEELPHFKLVTRTTLSQWKIFT
EGEAQISQMCSSRVCRTELEDLVKVLYLERSEKGHC
>sp|Q9BWD1|THIC_HUMAN Acetyl-CoA acetyltransferase, cytosolic OS=Homo sapiens OX=9606 GN=ACAT2 PE=1 SV=2

MNAGSDPVVIVSAARTIIGSFNGALAAVPVQDLGSTVIKEVLKRATVAPEDVSEVIFGHV
LAAGCGQNPVRQASVGAGIPYSVPAWSCQMICGSGLKAVCLAVQSIGIGDSSIVVAGGME
NMSKAPHLAYLRTGVKIGEMPLTDSILCDGLTDAFHNCHMGITAENVAKKWQVSREDQDK
VAVLSQNRTENAQKAGHFDKEIVPVLVSTRKGLIEVKTDEFPRHGSNIEAMSKLKPYFLT
DGTGTVTPANASGINDGAAAVVLMKKSEADKRGLTPLARIVSWSQVGVEPSIMGIGPIPA
IKQAVTKAGWSLEDVDIFEINEAFAAVSAAIVKELGLNPEKVNIEGGAIALGHPLGASGC
RILVTLLHTLERMGRSRGVAALCIGGGMGIAMCVQRE
>sp|Q9BWQ8|LFG2_HUMAN Protein lifeguard 2 OS=Homo sapiens OX=9606 GN=FAIM2 PE=1 SV=1
MTQGKLSVANKAPGTEGQQQVHGEKKEAPAVPSAPPSYEEATSGEGMKAGAFPPAPTAVP
LHPSWAYVDPSSSSSYDNGFPTGDHELFTTFSWDDQKVRRVFVRKVYTILLIQLLVTLAV
VALFTFCDPVKDYVQANPGWYWASYAVFFATYLTLACCSGPRRHFPWNLILLTVFTLSMA
YLTGMLSSYYNTTSVLLCLGITALVCLSVTVFSFQTKFDFTSCQGVLFVLLMTLFFSGLI
LAILLPFQYVPWLHAVYAALGAGVFTLFLALDTQLLMGNRRHSLSPEEYIFGALNIYLDI
IYIFTFFLQLFGTNRE
>sp|Q9BX67|JAM3_HUMAN Junctional adhesion molecule C OS=Homo sapiens OX=9606 GN=JAM3 PE=1 SV=1
MALRRPPRLRLCARLPDFFLLLLFRGCLIGAVNLKSSNRTPVVQEFESVELSCIITDSQT
SDPRIEWKKIQDEQTTYVFFDNKIQGDLAGRAEILGKTSLKIWNVTRRDSALYRCEVVAR
NDRKEIDEIVIELTVQVKPVTPVCRVPKAVPVGKMATLHCQESEGHPRPHYSWYRNDVPL
PTDSRANPRFRNSSFHLNSETGTLVFTAVHKDDSGQYYCIASNDAGSARCEEQEMEVYDL
NIGGIIGGVLVVLAVLALITLGICCAYRRGYFINNKQDGESYKNPGKPDGVNYIRTDEEG
DFRHKSSFVI
>sp|Q9BXK5|B2L13_HUMAN Bcl-2-like protein 13 OS=Homo sapiens OX=9606 GN=BCL2L13 PE=1 SV=1
MASSSTVPLGFHYETKYVVLSYLGLLSQEKLQEQHLSSPQGVQLDIASQSLDQEILLKVK
TEIEEELKSLDKEISEAFTSTGFDRHTSPVFSPANPESSMEDCLAHLGEKVSQELKEPLH
KALQMLLSQPVTYQAFRECTLETTVHASGWNKILVPLVLLRQMLLELTRRGQEPLSALLQ
FGVTYLEDYSAEYIIQQGGWGTVFSLESEEEEYPGITAEDSNDIYILPSDNSGQVSPPES
PTVTTSWQSESLPVSLSASQSWHTESLPVSLGPESWQQIAMDPEEVKSLDSNGAGEKSEN
NSSNSDIVHVEKEEVPEGMEEAAVASVVLPARELQEALPEAPAPLLPHITATSLLGTREP
DTEVITVEKSSPATSLFVELDEEEVKAATTEPTEVEEVVPALEPTETLLSEKEINAREES
LVEELSPASEKKPVPPSEGKSRLSPAGEMKPMPLSEGKSILLFGGAAAVAILAVAIGVAL
ALRKK
>sp|Q9BXM0|PRAX_HUMAN Periaxin OS=Homo sapiens OX=9606 GN=PRX PE=1 SV=2
MEARSRSAEELRRAELVEIIVETEAQTGVSGINVAGGGKEGIFVRELREDSPAARSLSLQ
EGDQLLSARVFFENFKYEDALRLLQCAEPYKVSFCLKRTVPTGDLALRPGTVSGYEIKGP
RAKVAKLNIQSLSPVKKKKMVPGALGVPADLAPVDVEFSFPKFSRLRRGLKAEAVKGPVP
AAPARRRLQLPRLRVREVAEEAQAARLAAAAPPPRKAKVEAEVAAGARFTAPQVELVGPR
LPGAEVGVPQVSAPKAAPSAEAAGGFALHLPTLGLGAPAPPAVEAPAVGIQVPQVELPAL
PSLPTLPTLPCLETREGAVSVVVPTLDVAAPTVGVDLALPGAEVEARGEAPEVALKMPRL
SFPRFGARAKEVAEAKVAKVSPEARVKGPRLRMPTFGLSLLEPRPAAPEVVESKLKLPTI
KMPSLGIGVSGPEVKVPKGPEVKLPKAPEVKLPKVPEAALPEVRLPEVELPKVSEMKLPK
VPEMAVPEVRLPEVELPKVSEMKLPKVPEMAVPEVRLPEVQLLKVSEMKLPKVPEMAVPE

VRLPEVQLPKVSEMKLPEVSEVAVPEVRLPEVQLPKVPEMKVPEMKLPKVPEMKLPEMKL
PEVQLPKVPEMAVPDVHLPEVQLPKVPEMKLPEMKLPEVKLPKVPEMAVPDVHLPEVQLP
KVPEMKLPKMPEMAVPEVRLPEVQLPKVSEMKLPKVPEMAVPDVHLPEVQLPKVCEMKVP
DMKLPEIKLPKVPEMAVPDVHLPEVQLPKVSEIRLPEMQVPKVPDVHLPKAPEVKLPRAP
EVQLKATKAEQAEGMEFGFKMPKMTMPKLGRAESPSRGKPGEAGAEVSGKLVTLPCLQPE
VDGEAHVGVPSLTLPSVELDLPGALGLQGQVPAAKMGKGERVEGPEVAAGVREVGFRVPS
VEIVTPQLPAVEIEEGRLEMIETKVKPSSKFSLPKFGLSGPKVAKAEAEGAGRATKLKVS
KFAISLPKARVGAEAEAKGAGEAGLLPALDLSIPQLSLDAHLPSGKVEVAGADLKFKGPR
FALPKFGVRGRDTEAAELVPGVAELEGKGWGWDGRVKMPKLKMPSFGLARGKEAEVQGDR
ASPGEKAESTAVQLKIPEVELVTLGAQEEGRAEGAVAVSGMQLSGLKVSTAGQVVTEGHD
AGLRMPPLGISLPQVELTGFGEAGTPGQQAQSTVPSAEGTAGYRVQVPQVTLSLPGAQVA
GGELLVGEGVFKMPTVTVPQLELDVGLSREAQAGEAATGEGGLRLKLPTLGARARVGGEG
AEEQPPGAERTFCLSLPDVELSPSGGNHAEYQVAEGEGEAGHKLKVRLPRFGLVRAKEGA
EEGEKAKSPKLRLPRVGFSQSEMVTGEGSPSPEEEEEEEEEGSGEGASGRRGRVRVRLPR
VGLAAPSKASRGQEGDAAPKSPVREKSPKFRFPRVSLSPKARSGSGDQEEGGLRVRLPSV
GFSETGAPGPARMEGAQAAAV
>sp|Q9BXM7|PINK1_HUMAN Serine/threonine-protein kinase PINK1, mitochondrial OS=Homo sapiens OX=9606 GN=PINK1 PE=1 SV=1
MAVRQALGRGLQLGRALLLRFTGKPGRAYGLGRPGPAAGCVRGERPGWAAGPGAEPRRVG
LGLPNRLRFFRQSVAGLAARLQRQFVVRAWGCAGPCGRAVFLAFGLGLGLIEEKQAESRR
AVSACQEIQAIFTQKSKPGPDPLDTRRLQGFRLEEYLIGQSIGKGCSAAVYEATMPTLPQ
NLEVTKSTGLLPGRGPGTSAPGEGQERAPGAPAFPLAIKMMWNISAGSSSEAILNTMSQE
LVPASRVALAGEYGAVTYRKSKRGPKQLAPHPNIIRVLRAFTSSVPLLPGALVDYPDVLP
SRLHPEGLGHGRTLFLVMKNYPCTLRQYLCVNTPSPRLAAMMLLQLLEGVDHLVQQGIAH
RDLKSDNILVELDPDGCPWLVIADFGCCLADESIGLQLPFSSWYVDRGGNGCLMAPEVST
ARPGPRAVIDYSKADAWAVGAIAYEIFGLVNPFYGQGKAHLESRSYQEAQLPALPESVPP
DVRQLVRALLQREASKRPSARVAANVLHLSLWGEHILALKNLKLDKMVGWLLQQSAATLL
ANRLTEKCCVETKMKMLFLANLECETLCQAALLLCSWRAAL
>sp|Q9BXW6|OSBL1_HUMAN Oxysterol-binding protein-related protein 1 OS=Homo sapiens OX=9606 GN=OSBPL1A PE=1 SV=2
MNTEAEQQLLHHARNGNAEEVRQLLETMARNEVIADINCKGRSKSNLGWTPLHLACYFGH
RQVVQDLLKAGAEVNVLNDMGDTPLHRAAFTGRKELVMLLLEYNADTTIVNGSGQTAKEV
THAEEIRSMLEAVERTQQRKLEELLLAAAREGKTTELTALLNRPNPPDVNCSDQLGNTPL
HCAAYRAHKQCALKLLRSGADPNLKNKNDQKPLDLAQGAEMKHILVGNKVIYKALKRYEG
PLWKSSRFFGWRLFWVVLEHGVLSWYRKQPDAVHNIYRQGCKHLTQAVCTVKSTDSCLFF
IKCFDDTIHGFRVPKNSLQQSREDWLEAIEEHSAYSTHYCSQDQLTDEEEEDTVSAADLK
KSLEKAQSCQQRLDREISNFLKMIKECDMAKEMLPSFLQKVEVVSEASRETCVALTDCLN
LFTKQEGVRNFKLEQEQEKNKILSEALETLATEHHELEQSLVKGSPPASILSEDEFYDAL
SDSESERSLSRLEAVTARSFEEEGEHLGSRKHRMSEEKDCGGGDALSNGIKKHRTSLPSP
MFSRNDFSIWSILRKCIGMELSKITMPVIFNEPLSFLQRLTEYMEHTYLIHKASSLSDPV
ERMQCVAAFAVSAVASQWERTGKPFNPLLGETYELVRDDLGFRLISEQVSHHPPISAFHA
EGLNNDFIFHGSIYPKLKFWGKSVEAEPKGTITLELLEHNEAYTWTNPTCCVHNIIVGKL
WIEQYGNVEIINHKTGDKCVLNFKPCGLFGKELHKVEGYIQDKSKKKLCALYGKWTECLY
SVDPATFDAYKKNDKKNTEEKKNSKQMSTSEELDEMPVPDSESVFIIPGSVLLWRIAPRP

PNSAQMYNFTSFAMVLNEVDKDMESVIPKTDCRLRPDIRAMENGEIDQASEEKKRLEEKQ
RAARKNRSKSEEDWKTRWFHQGPNPYNGAQDWIYSGSYWDRNYFNLPDIY
>sp|Q9BY67|CADM1_HUMAN Cell adhesion molecule 1 OS=Homo sapiens OX=9606 GN=CADM1 PE=1 SV=2
MASVVLPSGSQCAAAAAAAAPPGLRLRLLLLLFSAAALIPTGDGQNLFTKDVTVIEGEVA
TISCQVNKSDDSVIQLLNPNRQTIYFRDFRPLKDSRFQLLNFSSSELKVSLTNVSISDEG
RYFCQLYTDPPQESYTTITVLVPPRNLMIDIQKDTAVEGEEIEVNCTAMASKPATTIRWF
KGNTELKGKSEVEEWSDMYTVTSQLMLKVHKEDDGVPVICQVEHPAVTGNLQTQRYLEVQ
YKPQVHIQMTYPLQGLTREGDALELTCEAIGKPQPVMVTWVRVDDEMPQHAVLSGPNLFI
NNLNKTDNGTYRCEASNIVGKAHSDYMLYVYDPPTTIPPPTTTTTTTTTTTTILTIITD
SRAGEEGSIRAVDHAVIGGVVAVVVFAMLCLLIILGRYFARHKGTYFTHEAKGADDAADA
DTAIINAEGGQNNSEEKKEYFI
>sp|Q9BYZ2|LDH6B_HUMAN L-lactate dehydrogenase A-like 6B OS=Homo sapiens OX=9606 GN=LDHAL6B PE=1 SV=3
MSWTVPVVRASQRVSSVGANFLCLGMALCPRQATRIPLNGTWLFTPVSKMATVKSELIER
FTSEKPVHHSKVSIIGTGSVGMACAISILLKGLSDELALVDLDEDKLKGETMDLQHGSPF
TKMPNIVCSKDYFVTANSNLVIITAGARQEKGETRLNLVQRNVAIFKLMISSIVQYSPHC
KLIIVSNPVDILTYVAWKLSAFPKNRIIGSGCNLDTARFRFLIGQKLGIHSESCHGWILG
EHGDSSVPVWSGVNIAGVPLKDLNSDIGTDKDPEQWKNVHKEVTATAYEIIKMKGYTSWA
IGLSVADLTESILKNLRRIHPVSTIIKGLYGIDEEVFLSIPCILGENGITNLIKIKLTPE
EEAHLKKSAKTLWEIQNKLKL
>sp|Q9BZJ4|S2539_HUMAN Probable mitochondrial glutathione transporter SLC25A39 OS=Homo sapiens OX=9606 GN=SLC25A39 PE=1 SV=2
MADQDPAGISPLQQMVASGTGAVVTSLFMTPLDVVKVRLQSQRPSMASELMPSSRLWSLS
YTKLPSSLQSTGKCLLYCNGVLEPLYLCPNGARCATWFQDPTRFTGTMDAFVKIVRHEGT
RTLWSGLPATLVMTVPATAIYFTAYDQLKAFLCGRALTSDLYAPMVAGALARLGTVTVIS
PLELMRTKLQAQHVSYRELGACVRTAVAQGGWRSLWLGWGPTALRDVPFSALYWFNYELV
KSWLNGFRPKDQTSVGMSFVAGGISGTVAAVLTLPFDVVKTQRQVALGAMEAVRVNPLHV
DSTWLLLRRIRAESGTKGLFAGFLPRIIKAAPSCAIMISTYEFGKSFFQRLNQDRLLGG
>sp|Q9C0C9|UBE2O_HUMAN (E3-independent) E2 ubiquitin-conjugating enzyme OS=Homo sapiens OX=9606 GN=UBE2O PE=1 SV=3
MADPAAPTPAAPAPAQAPAPAPEAVPAPAAAPVPAPAPASDSASGPSSDSGPEAGSQRLL
FSHDLVSGRYRGSVHFGLVRLIHGEDSDSEGEEEGRGSSGCSEAGGAGHEEGRASPLRRG
YVRVQWYPEGVKQHVKETKLKLEDRSVVPRDVVRHMRSTDSQCGTVIDVNIDCAVKLIGT
NCIIYPVNSKDLQHIWPFMYGDYIAYDCWLGKVYDLKNQIILKLSNGARCSMNTEDGAKL
YDVCPHVSDSGLFFDDSYGFYPGQVLIGPAKIFSSVQWLSGVKPVLSTKSKFRVVVEEVQ
VVELKVTWITKSFCPGGTDSVSPPPSVITQENLGRVKRLGCFDHAQRQLGERCLYVFPAK
VEPAKIAWECPEKNCAQGEGSMAKKVKRLLKKQVVRIMSCSPDTQCSRDHSMEDPDKKGE
SKTKSEAESASPEETPDGSASPVEMQDEGAEEPHEAGEQLPPFLLKEGRDDRLHSAEQDA
DDEAADDTDDTSSVTSSASSTTSSQSGSGTSRKKSIPLSIKNLKRKHKRKKNKITRDFKP
GDRVAVEVVTTMTSADVMWQDGSVECNIRSNDLFPVHHLDNNEFCPGDFVVDKRVQSCPD
PAVYGVVQSGDHIGRTCMVKWFKLRPSGDDVELIGEEEDVSVYDIADHPDFRFRTTDIVI
RIGNTEDGAPHKEDEPSVGQVARVDVSSKVEVVWADNSKTIILPQHLYNIESEIEESDYD
SVEGSTSGASSDEWEDDSDSWETDNGLVEDEHPKIEEPPIPPLEQPVAPEDKGVVISEEA

ATAAVQGAVAMAAPMAGLMEKAGKDGPPKSFRELKEAIKILESLKNMTVEQLLTGSPTSP
TVEPEKPTREKKFLDDIKKLQENLKKTLDNVAIVEEEKMEAVPDVERKEDKPEGQSPVKA
EWPSETPVLCQQCGGKPGVTFTSAKGEVFSVLEFAPSNHSFKKIEFQPPEAKKFFSTVRK
EMALLATSLPEGIMVKTFEDRMDLFSALIKGPTRTPYEDGLYLFDIQLPNIYPAVPPHFC
YLSQCSGRLNPNLYDNGKVCVSLLGTWIGKGTERWTSKSSLLQVLISIQGLILVNEPYYN
EAGFDSDRGLQEGYENSRCYNEMALIRVVQSMTQLVRRPPEVFEQEIRQHFSTGGWRLVN
RIESWLETHALLEKAQALPNGVPKASSSPEPPAVAELSDSGQQEPEDGGPAPGEASQGSD
SEGGAQGLASASRDHTDQTSETAPDASVPPSVKPKKRRKSYRSFLPEKSGYPDIGFPLFP
LSKGFIKSIRGVLTQFRAALLEAGMPECTEDK
>sp|Q9GZM7|TINAL_HUMAN Tubulointerstitial nephritis antigen-like OS=Homo sapiens OX=9606 GN=TINAGL1 PE=1 SV=1
MWRCPLGLLLLLPLAGHLALGAQQGRGRRELAPGLHLRGIRDAGGRYCQEQDLCCRGRAD
DCALPYLGAICYCDLFCNRTVSDCCPDFWDFCLGVPPPFPPIQGCMHGGRIYPVLGTYWD
NCNRCTCQENRQWQCDQEPCLVDPDMIKAINQGNYGWQAGNHSAFWGMTLDEGIRYRLGT
IRPSSSVMNMHEIYTVLNPGEVLPTAFEASEKWPNLIHEPLDQGNCAGSWAFSTAAVASD
RVSIHSLGHMTPVLSPQNLLSCDTHQQQGCRGGRLDGAWWFLRRRGVVSDHCYPFSGRER
DEAGPAPPCMMHSRAMGRGKRQATAHCPNSYVNNNDIYQVTPVYRLGSNDKEIMKELMEN
GPVQALMEVHEDFFLYKGGIYSHTPVSLGRPERYRRHGTHSVKITGWGEETLPDGRTLKY
WTAANSWGPAWGERGHFRIVRGVNECDIESFVLGVWGRVGMEDMGHH
>sp|Q9GZP4|PITH1_HUMAN PITH domain-containing protein 1 OS=Homo sapiens OX=9606 GN=PITHD1 PE=1 SV=1
MSHGHSHGGGGCRCAAEREEPPEQRGLAYGLYLRIDLERLQCLNESREGSGRGVFKPWEE
RTDRSKFVESDADEELLFNIPFTGNVKLKGIIIMGEDDDSHPSEMRLYKNIPQMSFDDTE
REPDQTFSLNRDLTGELEYATKISRFSNVYHLSIHISKNFGADTTKVFYIGLRGEWTELR
RHEVTICNYEASANPADHRVHQVTPQTHFIS
>sp|Q9GZR7|DDX24_HUMAN ATP-dependent RNA helicase DDX24 OS=Homo sapiens OX=9606 GN=DDX24 PE=1 SV=1
MKLKDTKSRPKQSSCGKFQTKGIKVVGKWKEVKIDPNMFADGQMDDLVCFEELTDYQLVS
PAKNPSSLFSKEAPKRKAQAVSEEEEEEEGKSSSPKKKIKLKKSKNVATEGTSTQKEFEV
KDPELEAQGDDMVCDDPEAGEMTSENLVQTAPKKKKNKGKKGLEPSQSTAAKVPKKAKTW
IPEVHDQKADVSAWKDLFVPRPVLRALSFLGFSAPTPIQALTLAPAIRDKLDILGAAETG
SGKTLAFAIPMIHAVLQWQKRNAAPPPSNTEAPPGETRTEAGAETRSPGKAEAESDALPD
DTVIESEALPSDIAAEARAKTGGTVSDQALLFGDDDAGEGPSSLIREKPVPKQNENEEEN
LDKEQTGNLKQELDDKSATCKAYPKRPLLGLVLTPTRELAVQVKQHIDAVARFTGIKTAI
LVGGMSTQKQQRMLNRRPEIVVATPGRLWELIKEKHYHLRNLRQLRCLVVDEADRMVEKG
HFAELSQLLEMLNDSQYNPKRQTLVFSATLTLVHQAPARILHKKHTKKMDKTAKLDLLMQ
KIGMRGKPKVIDLTRNEATVETLTETKIHCETDEKDFYLYYFLMQYPGRSLVFANSISCI
KRLSGLLKVLDIMPLTLHACMHQKQRLRNLEQFARLEDCVLLATDVAARGLDIPKVQHVI
HYQVPRTSEIYVHRSGRTARATNEGLSLMLIGPEDVINFKKIYKTLKKDEDIPLFPVQTK
YMDVVKERIRLARQIEKSEYRNFQACLHNSWIEQAAAALEIELEEDMYKGGKADQQEERR
RQKQMKVLKKELRHLLSQPLFTESQKTKYPTQSGKPPLLVSAPSKSESALSCLSKQKKKK
TKKPKEPQPEQPQPSTSAN
>sp|Q9GZT8|NIF3L_HUMAN NIF3-like protein 1 OS=Homo sapiens OX=9606 GN=NIF3L1 PE=1 SV=2

MLSSCVRPVPTTVRFVDSLICNSSRSFMDLKALLSSLNDFASLSFAESWDNVGLLVEPSP
PHTVNTLFLTNDLTEEVMEEVLQKKADLILSYHPPIFRPMKRITWNTWKERLVIRALENR
VGIYSPHTAYDAAPQGVNNWLAKGLGACTSRPIHPSKAPNYPTEGNHRVEFNVNYTQDLD
KVMSAVKGIDGVSVTSFSARTGNEEQTRINLNCTQKALMQVVDFLSRNKQLYQKTEILSL
EKPLLLHTGMGRLCTLDESVSLATMIDRIKRHLKLSHIRLALGVGRTLESQVKVVALCAG
SGSSVLQGVEADLYLTGEMSHHDTLDAASQGINVILCEHSNTERGFLSDLRDMLDSHLEN
KINIILSETDRDPLQVV
>sp|Q9H0C3|TM117_HUMAN Transmembrane protein 117 OS=Homo sapiens OX=9606
GN=TMEM117 PE=1 SV=1
MGKDFRYYFQHPWSRMIVAYLVIFFNFLIFAEDPVSHSQTEANVIVVGNCFSFVTNKYPR
GVGWRILKVLLWLLAILTGLIAGKFLFHQRLFGQLLRLKMFREDHGSWMTMFFSTILFLF
IFSHIYNTILLMDGNMGAYIITDYMGIRNESFMKLAAVGTWMGDFVTAWMVTDMMLQDKP
YPDWGKSARAFWKKGNVRITLFWTVLFTLTSVVVLVITTDWISWDKLNRGFLPSDEVSRA
FLASFILVFDLLIVMQDWEFPHFMGDVDVNLPGLHTPHMQFKIPFFQKIFKEEYRIHITG
KWFNYGIIFLVLILDLNMWKNQIFYKPHEYGQYIGPGQKIYTVKDSESLKDLNRTKLSWE
WRSNHTNPRTNKTYVEGDMFLHSRFIGASLDVKCLAFVPSLIAFVWFGFFIWFFGRFLKN
EPRMENQDKTYTRMKRKSPSEHSKDMGITRENTQASVEDPLNDPSLVCIRSDFNEIVYKS
SHLTSENLSSQLNESTSATEADQDPTTSKSTPTN
>sp|Q9H0N5|PHS2_HUMAN Pterin-4-alpha-carbinolamine dehydratase 2 OS=Homo sapiens
OX=9606 GN=PCBD2 PE=1 SV=4
MAAVLGALGATRRLLAALRGQSLGLAAMSSGTHRLTAEERNQAILDLKAAGWSELSERDA
IYKEFSFHNFNQAFGFMSRVALQAEKMNHHPEWFNVYNKVQITLTSHDCGELTKKDVKLA
KFIEKAAASV
>sp|Q9H0U4|RAB1B_HUMAN Ras-related protein Rab-1B OS=Homo sapiens OX=9606
GN=RAB1B PE=1 SV=1
MNPEYDYLFKLLLIGDSGVGKSCLLLRFADDTYTESYISTIGVDFKIRTIELDGKTIKLQ
IWDTAGQERFRTITSSYYRGAHGIIVVYDVTDQESYANVKQWLQEIDRYASENVNKLLVG
NKSDLTTKKVVDNTTAKEFADSLGIPFLETSAKNATNVEQAFMTMAAEIKKRMGPGAASG
GERPNLKIDSTPVKPAGGGCC
>sp|Q9H0X4|F234A_HUMAN Protein FAM234A OS=Homo sapiens OX=9606 GN=FAM234A
PE=1 SV=1
MLDHKDLEAEIHPLKNEERKSQENLGNPSKNEDNVKSAPPQSRLSRCRAAAFFLSLFLCL
FVVFVVSFVIPCPDRPASQRMWRIDYSAAVIYDFLAVDDINGDRIQDVLFLYKNTNSSNN
FSRSCVDEGFSSPCTFAAAVSGANGSTLWERPVAQDVALVECAVPQPRGSEAPSACILVG
RPSSFIAVNLFTGETLWNHSSSFSGNASILSPLLQVPDVDGDGAPDLLVLTQEREEVSGH
LYSGSTGHQIGLRGSLGVDGESGFLLHVTRTGAHYILFPCASSLCGCSVKGLYEKVTGSG
GPFKSDPHWESMLNATTRRMLSHSSGAVRYLMHVPGNAGADVLLVGSEAFVLLDGQELTP
RWTPKAAHVLRKPIFGRYKPDTLAVAVENGTGTDRQILFLDLGTGAVLCSLALPSLPGGP
LSASLPTADHRSAFFFWGLHELGSTSETETGEARHSLYMFHPTLPRVLLELANVSTHIVA
FDAVLFEPSRHAAYILLTGPADSEAPGLVSVIKHKVRDLVPSSRVVRLGEGGPDSDQAIR
DRFSRLRYQSEA
>sp|Q9H3S3|TMPS5_HUMAN Transmembrane protease serine 5 OS=Homo sapiens OX=9606
GN=TMPRSS5 PE=1 SV=2
MSLMLDDQPPMEAQYAEEGPGPGIFRAEPGDQQHPISQAVCWRSMRRGCAVLGALGLLAG

AGVGSWLLVLYLCPAASQPISGTLQDEEITLSCSEASAEEALLPALPKTVSFRINSEDFL
LEAQVRDQPRWLLVCHEGWSPALGLQICWSLGHLRLTHHKGVNLTDIKLNSSQEFAQLSP
RLGGFLEEAWQPRNNCTSGQVVSLRCSECGARPLASRIVGGQSVAPGRWPWQASVALGFR
HTCGGSVLAPRWVVTAAHCMHSFRLARLSSWRVHAGLVSHSAVRPHQGALVERIIPHPLY
SAQNHDYDVALLRLQTALNFSDTVGAVCLPAKEQHFPKGSRCWVSGWGHTHPSHTYSSDM
LQDTVVPLFSTQLCNSSCVYSGALTPRMLCAGYLDGRADACQGDSGGPLVCPDGDTWRLV
GVVSWGRGCAEPNHPGVYAKVAEFLDWIHDTAQDSLL
>sp|Q9H3Z4|DNJC5_HUMAN DnaJ homolog subfamily C member 5 OS=Homo sapiens OX=9606 GN=DNAJC5 PE=1 SV=1
MADQRQRSLSTSGESLYHVLGLDKNATSDDIKKSYRKLALKYHPDKNPDNPEAADKFKEI
NNAHAILTDATKRNIYDKYGSLGLYVAEQFGEENVNTYFVLSSWWAKALFVFCGLLTCCY
CCCCLCCCFNCCCGKCKPKAPEGEETEFYVSPEDLEAQLQSDEREATDTPIVIQPASATE
TTQLTADSHPSYHTDGFN
>sp|Q9H4A4|AMPB_HUMAN Aminopeptidase B OS=Homo sapiens OX=9606 GN=RNPEP PE=1 SV=2
MASGEHSPGSGAARRPLHSAQAVDVASASNFRAFELLHLHLDLRAEFGPPGPGAGSRGLS
GTAVLDLRCLEPEGAAELRLDSHPCLEVTAAALRRERPGSEEPPAEPVSFYTQPFSHYGQ
ALCVSFPQPCRAAERLQVLLTYRVGEGPGVCWLAPEQTAGKKKPFVYTQGQAVLNRAFFP
CFDTPAVKYKYSALIEVPDGFTAVMSASTWEKRGPNKFFFQMCQPIPSYLIALAIGDLVS
AEVGPRSRVWAEPCLIDAAKEEYNGVIEEFLATGEKLFGPYVWGRYDLLFMPPSFPFGGM
ENPCLTFVTPCLLAGDRSLADVIIHEISHSWFGNLVTNANWGEFWLNEGFTMYAQRRIST
ILFGAAYTCLEAATGRALLRQHMDITGEENPLNKLRVKIEPGVDPDDTYNETPYEKGFCF
VSYLAHLVGDQDQFDSFLKAYVHEFKFRSILADDFLDFYLEYFPELKKKRVDIIPGFEFD
RWLNTPGWPPYLPDLSPGDSLMKPAEELAQLWAAEELDMKAIEAVAISPWKTYQLVYFLD
KILQKSPLPPGNVKKLGDTYPSISNARNAELRLRWGQIVLKNDHQEDFWKVKEFLHNQGK
QKYTLPLYHAMMGGSEVAQTLAKETFASTASQLHSNVVNYVQQIVAPKGS
>sp|Q9H4B7|TBB1_HUMAN Tubulin beta-1 chain OS=Homo sapiens OX=9606 GN=TUBB1 PE=1 SV=1
MREIVHIQIGQCGNQIGAKFWEMIGEEHGIDLAGSDRGASALQLERISVYYNEAYGRKYV
PRAVLVDLEPGTMDSIRSSKLGALFQPDSFVHGNSGAGNNWAKGHYTEGAELIENVLEVV
RHESESCDCLQGFQIVHSLGGGTGSGMGTLLMNKIREEYPDRIMNSFSVMPSPKVSDTVV
EPYNAVLSIHQLIENADACFCIDNEALYDICFRTLKLTTPTYGDLNHLVSLTMSGITTSL
RFPGQLNADLRKLAVNMVPFPRLHFFMPGFAPLTAQGSQQYRALSVAELTQQMFDARNTM
AACDLRRGRYLTVACIFRGKMSTKEVDQQLLSVQTRNSSCFVEWIPNNVKVAVCDIPPRG
LSMAATFIGNNTAIQEIFNRVSEHFSAMFKRKAFVHWYTSEGMDINEFGEAENNIHDLVS
EYQQFQDAKAVLEEDEEVTEEAEMEPEDKGH
>sp|Q9H4G0|E41L1_HUMAN Band 4.1-like protein 1 OS=Homo sapiens OX=9606 GN=EPB41L1 PE=1 SV=2
MTTETGPDSEVKKAQEEAPQQPEAAAAVTTPVTPAGHGHPEANSNEKHPSQQDTRPAEQS
LDMEEKDYSEADGLSERTTPSKAQKSPQKIAKKYKSAICRVTLLDASEYECEVEKHGRGQ
VLFDLVCEHLNLLEKDYFGLTFCDADSQKNWLDPSKEIKKQIRSSPWNFAFTVKFYPPDP
AQLTEDITRYYLCLQLRADIITGRLPCSFVTHALLGSYAVQAELGDYDAEEHVGNYVSEL
RFAPNQTRELEERIMELHKTYRGMTPGEAEIHFLENAKKLSMYGVDLHHAKDSEGIDIML
GVCANGLLIYRDRLRINRFAWPKILKISYKRSNFYIKIRPGEYEQFESTIGFKLPNHRSA

KRLWKVCIEHHTFFRLVSPEPPPKGFLVMGSKFRYSGRTQAQTRQASALIDRPAPFFERS
SSKRYTMSRSLDGAEFSRPASVSENHDAGPDGDKRDEDGESGGQRSEAEEGEVRTPTKIK
ELKPEQETTPRHKQEFLDKPEDVLLKHQASINELKRTLKEPNSKLIHRDRDWERERRLPS
SPASPSPKGTPEKANERAGLREGSEEKVKPPRPRAPESDTGDEDQDQERDTVFLKDNHLA
IERKCSSITVSSTSSLEAEVDFTVIGDYHGSAFEDFSRSLPELDRDKSDSDTEGLLFSRD
LNKGAPSQDDESGGIEDSPDRGACSTPDMPQFEPVKTETMTVSSLAIRKKIEPEAVLQTR
VSAMDNTQQVDGSASVGREFIATTPSITTETISTTMENSLKSGKGAAAMIPGPQTVATEI
RSLSPIIGKDVLTSTYGATAETLSTSTTTHVTKTVKGGFSETRIEKRIIITGDEDVDQDQ
ALALAIKEAKLQHPDMLVTKAVVYRETDPSPEERDKKPQES
>sp|Q9H857|NT5D2_HUMAN 5'-nucleotidase domain-containing protein 2 OS=Homo sapiens OX=9606 GN=NT5DC2 PE=1 SV=1
MRVESGSAQERGILLESLSTLLEKTTASHEGRAPGNRELTDLLPPEVCSLLNPAAIYANN
EISLRDVEVYGFDYDYTLAQYADALHPEIFSTARDILIEHYKYPEGIRKYDYNPSFAIRG
LHYDIQKSLLMKIDAFHYVQLGTAYRGLQPVPDEEVIELYGGTQHIPLYQMSGFYGKGPS
IKQFMDIFSLPEMALLSCVVDYFLGHSLEFDQAHLYKDVTDAIRDVHVKGLMYQWIEQDM
EKYILRGDETFAVLSRLVAHGKQLFLITNSPFSFVDKGMRHMVGPDWRQLFDVVIVQADK
PSFFTDRRKPFRKLDEKGSLQWDRITRLEKGKIYRQGNLFDFLRLTEWRGPRVLYFGDHL
YSDLADLMLRHGWRTGAIIPELEREIRIINTEQYMHSLTWQQALTGLLERMQTYQDAESR
QVLAAWMKERQELRCITKALFNAQFGSIFRTFHNPTYFSRRLVRFSDLYMASLSCLLNYR
VDFTFYPRRTPLQHEAPLWMDQLCTGCMKTPFLGDMAHIR
>sp|Q9H902|REEP1_HUMAN Receptor expression-enhancing protein 1 OS=Homo sapiens OX=9606 GN=REEP1 PE=1 SV=1
MVSWIISRLVVLIFGTLYPAYYSYKAVKSKDIKEYVKWMMYWIIFALFTTAETFTDIFLC
WFPFYYELKIAFVAWLLSPYTKGSSLLYRKFVHPTLSSKEKEIDDCLVQAKDRSYDALVH
FGKRGLNVAATAAVMAASKGQGALSERLRSFSMQDLTTIRGDGAPAPSGPPPPGSGRASG
KHGQPKMSRSASESASSSGTA
>sp|Q9H9T3|ELP3_HUMAN Elongator complex protein 3 OS=Homo sapiens OX=9606 GN=ELP3 PE=1 SV=2
MRQKRKGDLSPAELMMLTIGDVIKQLIEAHEQGKDIDLNKVKTKTAAKYGLSAQPRLVDI
IAAVPPQYRKVLMPKLKAKPIRTASGIAVVAVMCKPHRCPHISFTGNICVYCPGGPDSDF
EYSTQSYTGYEPTSMRAIRARYDPFLQTRHRIEQLKQLGHSVDKVEFIVMGGTFMALPEE
YRDYFIRNLHDALSGHTSNNIYEAVKYSERSLTKCIGITIETRPDYCMKRHLSDMLTYGC
TRLEIGVQSVYEDVARDTNRGHTVKAVCESFHLAKDSGFKVVAHMMPDLPNVGLERDIEQ
FTEFFENPAFRPDGLKLYPTLVIRGTGLYELWKSGRYKSYSPSDLVELVARILALVPPWT
RVYRVQRDIPMPLVSSGVEHGNLRELALARMKDLGIQCRDVRTREVGIQEIHHKVRPYQV
ELVRRDYVANGGWETFLSYEDPDQDILIGLLRLRKCSEETFRFELGGGVSIVRELHVYGS
VVPVSSRDPTKFQHQGFGMLLMEEAERIAREEHGSGKIAVISGVGTRNYYRKIGYRLQGP
YMVKMLK
>sp|Q9HA64|KT3K_HUMAN Ketosamine-3-kinase OS=Homo sapiens OX=9606 GN=FN3KRP PE=1 SV=2
MEELLRRELGCSSVRATGHSGGGCISQGRSYDTDQGRVFVKVNPKAEARRMFEGEMASLT
AILKTNTVKVPKPIKVLDAPGGGSVLVMEHMDMRHLSSHAAKLGAQLADLHLDNKKLGEM
RLKEAGTVGRGGGQEERPFVARFGFDVVTCCGYLPQVNDWQEDWVVFYARQRIQPQMDMV
EKESGDREALQLWSALQLKIPDLFRDLEIIPALLHGDLWGGNVAEDSSGPVIFDPASFYG

HSEYELAIAGMFGGFSSSFYSAYHGKIPKAPGFEKRLQLYQLFHYLNHWNHFGSGYRGSS
LNIMRNLVK
>sp|Q9HAB8|PPCS_HUMAN Phosphopantothenate--cysteine ligase OS=Homo sapiens OX=9606
GN=PPCS PE=1 SV=2
MAEMDPVAEFPQPPGAARWAEVMARFAARLGAQGRRVVLVTSGGTKVPLEARPVRFLDNF
SSGRRGATSAEAFLAAGYGVLFLYRARSAFPYAHRFPPQTWLSALRPSGPALSGLLSLEA
EENALPGFAEALRSYQEAAAAGTFLAVEFTTLADYLHLLQAAAQALNPLGPSAMFYLAAA
VSDFYVPVSEMPEHKIQSSGGPLQITMKMVPKLLSPLVKDWAPKAFIISFKLETDPAIVI
NRARKALEIYQHQVVVANILESRQSFVFIVTKDSETKLLLSEEEIEKGVEIEEKIVDNLQ
SRHTAFIGDRN
>sp|Q9HB19|PKHA2_HUMAN Pleckstrin homology domain-containing family A member 2
OS=Homo sapiens OX=9606 GN=PLEKHA2 PE=1 SV=3
MPYVDRQNRICGFLDIEEHENSGKFLRRYFILDTQANCLLWYMDNPQNLAMGAGAVGALQ
LTYISKVSIATPKQKPKTPFCFVINALSQRYFLQANDQKDMKDWVEALNQASKITVPKGG
GLPMTTEVLKSLAAPPALEKKPQVAYKTEIIGGVVVHTPISQNGGDGQEGSEPGSHTILR
RSQSYIPTSGCRASTGPPLIKSGYCVKQGNVRKSWKRRFFALDDFTICYFKCEQDREPLR
TIFLKDVLKTHECLVKSGDLLMRDNLFEIITSSRTFYVQADSPEDMHSWIKEIGAAVQAL
KCHPRETSFSRSISLTRPGSSSLSSGPNSILCRGRPPLEEKKALCKAPSVASSWQPWTPV
PQAGEKLLPPGDTSEDSLFTPRPGEGSAPGVLPSSRIRHRSEPQHPKEKPFMFNLDDENI
RTSDV
>sp|Q9HB90|RRAGC_HUMAN Ras-related GTP-binding protein C OS=Homo sapiens OX=9606
GN=RRAGC PE=1 SV=1
MSLQYGAEETPLAGSYGAADSFPKDFGYGVEEEEEEAAAAGGGVGAGAGGGCGPGGADSS
KPRILLMGLRRSGKSSIQKVVFHKMSPNETLFLESTNKIYKDDISNSSFVNFQIWDFPGQ
MDFFDPTFDYEMIFRGTGALIYVIDAQDDYMEALTRLHITVSKAYKVNPDMNFEVFIHKV
DGLSDDHKIETQRDIHQRANDDLADAGLEKLHLSFYLTSIYDHSIFEAFSKVVQKLIPQL
PTLENLLNIFISNSGIEKAFLFDVVSKIYIATDSSPVDMQSYELCCDMIDVVIDVSCIYG
LKEDGSGSAYDKESMAIIKLNNTTVLYLKEVTKFLALVCILREESFERKGLIDYNFHCFR
KAIHEVFEVGVTSHRSCGHQTSASSLKALTHNGTPRNAI
>sp|Q9HBH1|DEFM_HUMAN Peptide deformylase, mitochondrial OS=Homo sapiens OX=9606
GN=PDF PE=1 SV=1
MARLWGALSLWPLWAAVPWGGAAAVGVRACSSTAAPDGVEGPALRRSYWRHLRRLVLGPP
EPPFSHVCQVGDPVLRGVAAPVERAQLGGPELQRLTQRLVQVMRRRRCVGLSAPQLGVPR
QVLALELPEALCRECPPRQRALRQMEPFPLRVFVNPSLRVLDSRLVTFPEGCESVAGFLA
CVPRFQAVQISGLDPNGEQVVWQASGWAARIIQHEMDHLQGCLFIDKMDSRTFTNVYWMK
VND
>sp|Q9HC10|OTOF_HUMAN Otoferlin OS=Homo sapiens OX=9606 GN=OTOF PE=1 SV=3
MALLIHLKTVSELRGRGDRIAKVTFRGQSFYSRVLENCEDVADFDETFRWPVASSIDRNE
MLEIQVFNYSKVFSNKLIGTFRMVLQKVVEESHVEVTDTLIDDNNAIIKTSLCVEVRYQA
TDGTVGSWDDGDFLGDESLQEEEKDSQETDGLLPGSRPSSRPPGEKSFRRAGRSVFSAMK
LGKNRSHKEEPQRPDEPAVLEMEDLDHLAIRLGDGLDPDSVSLASVTALTTNVSNKRSKP
DIKMEPSAGRPMDYQVSITVIEARQLVGLNMDPVVCVEVGDDKKYTSMKESTNCPYYNEY
FVFDFHVSPDVMFDKIIKISVIHSKNLLRSGTLVGSFKMDVGTVYSQPEHQFHHKWAILS
DPDDISSGLKGYVKCDVAVVGKGDNIKTPHKANETDEDDIEGNLLLPEGVPPERQWARFY

VKIYRAEGLPRMNTSLMANVKKAFIGENKDLVDPYVQVFFAGQKGKTSVQKSSYEPLWNE
QVVFTDLFPPLCKRMKVQIRDSDKVNDVAIGTHFIDLRKISNDGDKGFLPTLGPAWVNMY
GSTRNYTLLDEHQDLNEGLGEGVSFRARLLLGLAVEIVDTSNPELTSSTEVQVEQATPIS
ESCAGKMEEFFLFGAFLEASMIDRRNGDKPITFEVTIGNYGNEVDGLSRPQRPRPRKEPG
DEEEVDLIQNASDDEAGDAGDLASVSSTPPMRPQVTDRNYFHLPYLERKPCIYIKSWWPD
QRRRLYNANIMDHIADKLEEGLNDIQEMIKTEKSYPERRLRGVLEELSCGCCRFLSLADK
DQGHSSRTRLDRERLKSCMRELENMGQQARMLRAQVKRHTVRDKLRLCQNFLQKLRFLAD
EPQHSIPDIFIWMMSNNKRVAYARVPSKDLLFSIVEEETGKDCAKVKTLFLKLPGKRGFG
SAGWTVQAKVELYLWLGLSKQRKEFLCGLPCGFQEVKAAQGLGLHAFPPVSLVYTKKQAF
QLRAHMYQARSLFAADSSGLSDPFARVFFINQSQCTEVLNETLCPTWDQMLVFDNLELYG
EAHELRDDPPIIVIEIYDQDSMGKADFMGRTFAKPLVKMADEAYCPPRFPPQLEYYQIYR
GNATAGDLLAAFELLQIGPAGKADLPPINGPVDVDRGPIMPVPMGIRPVLSKYRVEVLFW
GLRDLKRVNLAQVDRPRVDIECAGKGVQSSLIHNYKKNPNFNTLVKWFEVDLPENELLHP
PLNIRVVDCRAFGRYTLVGSHAVSSLRRFIYRPPDRSAPSWNTTVRLLRRCRVLCNGGSS
SHSTGEVVVTMEPEVPIKKLETMVKLDATSEAVVKVDVAEEEKEKKKKKKGTAEEPEEEE
PDESMLDWWSKYFASIDTMKEQLRQQEPSGIDLEEKEEVDNTEGLKGSMKGKEKARAAKE
EKKKKTQSSGSGQGSEAPEKKKPKIDELKVYPKELESEFDNFEDWLHTFNLLRGKTGDDE
DGSTEEERIVGRFKGSLCVYKVPLPEDVSREAGYDSTYGMFQGIPSNDPINVLVRVYVVR
ATDLHPADINGKADPYIAIRLGKTDIRDKENYISKQLNPVFGKSFDIEASFPMESMLTVA
VYDWDLVGTDDLIGETKIDLENRFYSKHRATCGIAQTYSTHGYNIWRDPMKPSQILTRLC
KDGKVDGPHFGPPGRVKVANRVFTGPSEIEDENGQRKPTDEHVALLALRHWEDIPRAGCR
LVPEHVETRPLLNPDKPGIEQGRLELWVDMFPMDMPAPGTPLDISPRKPKKYELRVIIWN
TDEVVLEDDDFFTGEKSSDIFVRGWLKGQQEDKQDTDVHYHSLTGEGNFNWRYLFPFDYL
AAEEKIVISKKESMFSWDETEYKIPARLTLQIWDADHFSADDFLGAIELDLNRFPRGAKT
AKQCTMEMATGEVDVPLVSIFKQKRVKGWWPLLARNENDEFELTGKVEAELHLLTAEEAE
KNPVGLARNEPDPLEKPNRPDTSFIWFLNPLKSARYFLWHTYRWLLLKLLLLLLLLLLA
LFLYSVPGYLVKKILGA
>sp|Q9HC38|GLOD4_HUMAN Glyoxalase domain-containing protein 4 OS=Homo sapiens
OX=9606 GN=GLOD4 PE=1 SV=1
MAARRALHFVFKVGNRFQTARFYRDVLGMKVESCSVARLECSGAISAHCSDYTRITEDSF
SKPYDGKWSKTMVGFGPEDDHFVAELTYNYGVGDYKLGNDFMGITLASSQAVSNARKLEW
PLTEVAEGVFETEAPGGYKFYLQNRSLPQSDPVLKVTLAVSDLQKSLNYWCNLLGMKIYE
KDEEKQRALLGYADNQCKLELQGVKGGVDHAAAFGRIAFSCPQKELPDLEDLMKRENQKI
LTPLVSLDTPGKATVQVVILADPDGHEICFVGDEAFRELSKMDPEGSKLLDDAMAADKSD
EWFAKHNKPKASG
>sp|Q9HCB6|SPON1_HUMAN Spondin-1 OS=Homo sapiens OX=9606 GN=SPON1 PE=1 SV=2
MRLSPAPLKLSRTPALLALALPLAAALAFSDETLDKVPKSEGYCSRILRAQGTRREGYTE
FSLRVEGDPDFYKPGTSYRVTLSAAPPSYFRGFTLIALRENREGDKEEDHAGTFQIIDEE
ETQFMSNCPVAVTESTPRRRTRIQVFWIAPPAGTGCVILKASIVQKRIIYFQDEGSLTKK
LCEQDSTFDGVTDKPILDCCACGTAKYRLTFYGNWSEKTHPKDYPRRANHWSAIIGGSHS
KNYVLWEYGGYASEGVKQVAELGSPVKMEEEIRQQSDEVLTVIKAKAQWPAWQPLNVRAA
PSAEFSVDRTRHLMSFLTMMGPSPDWNVGLSAEDLCTKECGWVQKVVQDLIPWDAGTDSG
VTYESPNKPTIPQEKIRPLTSLDHPQSPFYDPEGGSITQVARVVIERIARKGEQCNIVPD
NVDDIVADLAPEEKDEDDTPETCIYSNWSPWSACSSSTCDKGKRMRQRMLKAQLDLSVPC

PDTQDFQPCMGPGCSDEDGSTCTMSEWITWSPCSISCGMGMRSRERYVKQFPEDGSVCTL
PTEETEKCTVNEECSPSSCLMTEWGEWDECSATCGMGMKKRHRMIKMNPADGSMCKAETS
QAEKCMMPECHTIPCLLSPWSEWSDCSVTCGKGMRTRQRMLKSLAELGDCNEDLEQVEKC
MLPECPIDCELTEWSQWSECNKSCGKGHVIRTRMIQMEPQFGGAPCPETVQRKKCRIRKC
LRNPSIQKLRWREARESRRSEQLKEESEGEQFPGCRMRPWTAWSECTKLCGGGIQERYMT
VKKRFKSSQFTSCKDKKEIRACNVHPC
>sp|Q9HCJ1|ANKH_HUMAN Mineralization regulator ANKH OS=Homo sapiens OX=9606 GN=ANKH PE=1 SV=2
MVKFPALTHYWPLIRFLVPLGITNIAIDFGEQALNRGIAAVKEDAVEMLASYGLAYSLMK
FFTGPMSDFKNVGLVFVNSKRDRTKAVLCMVVAGAIAAVFHTLIAYSDLGYYIINKLHHV
DESVGSKTRRAFLYLAAFPFMDAMAWTHAGILLKHKYSFLVGCASISDVIAQVVFVAILL
HSHLECREPLLIPILSLYMGALVRCTTLCLGYYKNIHDIIPDRSGPELGGDATIRKMLSF
WWPLALILATQRISRPIVNLFVSRDLGGSSAATEAVAILTATYPVGHMPYGWLTEIRAVY
PAFDKNNPSNKLVSTSNTVTAAHIKKFTFVCMALSLTLCFVMFWTPNVSEKILIDIIGVD
FAFAELCVVPLRIFSFFPVPVTVRAHLTGWLMTLKKTFVLAPSSVLRIIVLIASLVVLPY
LGVHGATLGVGSLLAGFVGESTMVAIAACYVYRKQKKKMENESATEGEDSAMTDMPPTEE
VTDIVEMREENE
>sp|Q9HCK4|ROBO2_HUMAN Roundabout homolog 2 OS=Homo sapiens OX=9606 GN=ROBO2 PE=1 SV=2
MSLLMFTQLLLCGFLYVRVDGSRLRQEDFPPRIVEHPSDVIVSKGEPTTLNCKAEGRPTP
TIEWYKDGERVETDKDDPRSHRMLLPSGSLFFLRIVHGRRSKPDEGSYVCVARNYLGEAV
SRNASLEVALLRDDFRQNPTDVVVAAGEPAILECQPPRGHPEPTIYWKKDKVRIDDKEER
ISIRGGKLMISNTRKSDAGMYTCVGTNMVGERDSDPAELTVFERPTFLRRPINQVVLEEE
AVEFRCQVQGDPQPTVRWKKDDADLPRGRYDIKDDYTLRIKKTMSTDEGTYMCIAENRVG
KMEASATLTVRAPPQFVVRPRDQIVAQGRTVTFPCETKGNPQPAVFWQKEGSQNLLFPNQ
PQQPNSRCSVSPTGDLTITNIQRSDAGYYICQALTVAGSILAKAQLEVTDVLTDRPPPII
LQGPANQTLAVDGTALLKCKATGDPLPVISWLKEGFTFPGRDPRATIQEQGTLQIKNLRI
SDTGTYTCVATSSSGETSWSAVLDVTESGATISKNYDLSDLPGPPSKPQVTDVTKNSVTL
SWQPGTPGTLPASAYIIEAFSQSVSNSWQTVANHVKTTLYTVRGLRPNTIYLFMVRAINP
QGLSDPSPMSDPVRTQDISPPAQGVDHRQVQKELGDVLVRLHNPVVLTPTTVQVTWTVDR
QPQFIQGYRVMYRQTSGLQATSSWQNLDAKVPTERSAVLVNLKKGVTYEIKVRPYFNEFQ
GMDSESKTVRTTEEAPSAPPQSVTVLTVGSYNSTSISVSWDPPPPDHQNGIIQEYKIWCL
GNETRFHINKTVDAAIRSVIIGGLFPGIQYRVEVAASTSAGVGVKSEPQPIIIGRRNEVV
ITENNNSITEQITDVVKQPAFIAGIGGACWVILMGFSIWLYWRRKKRKGLSNYAVTFQRG
DGGLMSNGSRPGLLNAGDPSYPWLADSWPATSLPVNNSNSGPNEIGNFGRGDVLPPVPGQ
GDKTATMLSDGAIYSSIDFTTKTSYNSSSQITQATPYATTQILHSNSIHELAVDLPDPQW
KSSIQQKTDLMGFGYSLPDQNKGNNGGKGGKKKKNKNSSKPQKNNGSTWANVPLPPPPVQ
PLPGTELEHYAVEQQENGYDSDSWCPPLPVQTYLHQGLEDELEEDDDRVPTPPVRGVASS
PAISFGQQSTATLTPSPREEMQPMLQAHLDELTRAYQFDIAKQTWHIQSNNQPPQPPVPP
LGYVSGALISDLETDVADDDADDEEEALEIPRPLRALDQTPGSSMDNLDSSVTGKAFTSS
QRPRPTSPFSTDSNTSAALSQSQRPRPTKKHKGGRMDQQPALPHRREGMTDEEALVPYSK
PSFPSPGGHSSSGTASSKGSTGPRKTEVLRAGHQRNASDLLDIGYMGSNSQGQFTGEL
>tr|Q9NP01|Q9NP01_HUMAN Fibrillin 15 (Fragment) OS=Homo sapiens OX=9606 PE=2 SV=1
VLVTVVFIFLSYNKMLSSPCINGVCKNSPGSFICECSSESTLDPTKTICIETIKGTCWQT

VIDGRCEININGATLKSQCCSSLGAAWGSPCTLCQVDPICGKGYSRIKGTQCEDIDECEV
FPGVCKNGLCVNTRGSFKCQCPSGMTLDATGRICLDIRLETCFLRYEDEECTLPIAGRHR
MDACCCSVGAAWGTEECEECPMRNTPEYEELCPRGPGFATKEITNGKPFFKDINECKMIP
SLCTHGKCRNTIGSFKCRCDSGFALDSEERNCTDIDECRISPDLCGRGQCVNTPGDFECK
CDEGYESGFMMMKNCMDIDECQRDPLLCRGGVCHNTEGSYRCECPPGHQLSPNISACIDI
NECELSAHLCPNGRCVNLIGKYQCACNPGYHSTPDRLFCVDIDECSIMNGGCETFCTNSE
GSYECSCQPGFALMPDQRSCTDIDECEDNPNICDGGQCTNIPGEYRCLCYDGFMASEDMK
TCVDVNECDLNPNICLSGTCENTKGSFICHCDMGYSG
>sp|Q9NP72|RAB18_HUMAN Ras-related protein Rab-18 OS=Homo sapiens OX=9606
GN=RAB18 PE=1 SV=1
MDEDVLTTLKILIIGESGVGKSSLLLRFTDDTFDPELAATIGVDFKVKTISVDGNKAKLA
IWDTAGQERFRTLTPSYYRGAQGVILVYDVTRRDTFVKLDNWLNELETYCTRNDIVNMLV
GNKIDKENREVDRNEGLKFARKHSMLFIEASAKTCDGVQCAFEELVEKIIQTPGLWESEN
QNKGVKLSHREEGQGGGACGGYCSVL
>sp|Q9NPQ8|RIC8A_HUMAN Synembryn-A OS=Homo sapiens OX=9606 GN=RIC8A PE=1 SV=3
MEPRAVAEAVETGEEDVIMEALRSYNQEHSQSFTFDDAQQEDRKRLAELLVSVLEQGLPP
SHRVIWLQSVRILSRDRNCLDPFTSRQSLQALACYADISVSEGSVPESADMDVVLESLKC
LCNLVLSSPVAQMLAAEARLVVKLTERVGLYRERSFPHDVQFFDLRLLFLLTALRTDVRQ
QLFQELKGVRLLTDTLELTLGVTPEGNPPPTLLPSQETERAMEILKVLFNITLDSIKGEV
DEEDAALYRHLGTLLRHCVMIATAGDRTEEFHGHAVNLLGNLPLKCLDVLLTLEPHGDST
EFMGVNMDVIRALLIFLEKRLHKTHRLKESVAPVLSVLTECARMHRPARKFLKAQVLPPL
RDVRTRPEVGEMLRNKLVRLMTHLDTDVKRVAAEFLFVLCSESVPRFIKYTGYGNAAGLL
AARGLMAGGRPEGQYSEDEDTDTDEYKEAKASINPVTGRVEEKPPNPMEGMTEEQKEHEA
MKLVTMFDKLSRNRVIQPMGMSPRGHLTSLQDAMCETMEQQLSSDPDSDPD
>sp|Q9NQ79|CRAC1_HUMAN Cartilage acidic protein 1 OS=Homo sapiens OX=9606
GN=CRTAC1 PE=1 SV=2
MAPSADPGMSRMLPFLLLLWFLPITEGSQRAEPMFTAVTNSVLPPDYDSNPTQLNYGVAV
TDVDHDGDFEIVVAGYNGPNLVLKYDRAQKRLVNIAVDERSSPYYALRDRQGNAIGVTAC
DIDGDGREEIYFLNTNNAFSGVATYTDKLFKFRNNRWEDILSDEVNVARGVASLFAGRSV
ACVDRKGSGRYSIYIANYAYGNVGPDALIEMDPEASDLSRGILALRDVAAEAGVSKYTGG
RGVSVGPILSSSASDIFCDNENGPNFLFHNRGDGTFVDAAASAGVDDPHQHGRGVALADF
NRDGKVDIVYGNWNGPHRLYLQMSTHGKVRFRDIASPKFSMPSPVRTVITADFDNDQELE
IFFNNIAYRSSSANRLFRVIRREHGDPLIEELNPGDALEPEGRGTGGVVTDFDGDGMLDL
ILSHGESMAQPLSVFRGNQGFNNNWLRVVPRTRFGAFARGAKVVLYTKKSGAHLRIIDGG
SGYLCEMEPVAHFGLGKDEASSVEVTWPDGKMVSRNVASGEMNSVLEILYPRDEDTLQDP
APLECGQGFSQQENGHCMDTNECIQFPFVCPRDKPVCVNTYGSYRCRTNKKCSRGYEPNE
DGTACVGTLGQSPGPRPTTPTAAAATAAAAAAGAATAAPVLVDGDLNLGSVVKESCEPS
C
>sp|Q9NQC3|RTN4_HUMAN Reticulon-4 OS=Homo sapiens OX=9606 GN=RTN4 PE=1 SV=2
MEDLDQSPLVSSSDSPPRPQPAFKYQFVREPEDEEEEEEEEEDEDEDLEELEVLERKPA
AGLSAAPVPTAPAAGAPLMDFGNDFVPPAPRGPLPAAPPVAPERQPSWDPSPVSSTVPAP
SPLSAAAVSPSKLPEDDEPPARPPPPPPASVSPQAEPVWTPPAPAPAAPPSTPAAPKRRG
SSGSVDETLFALPAASEPVIRSSAENMDLKEQPGNTISAGQEDFPSVLLETAASLPSLSP
LSAASFKEHEYLGNLSTVLPTEGTLQENVSEASKEVSEKAKTLLIDRDLTEFSELEYSEM

GSSFSVSPKAESAVIVANPREEIIVKNKDEEEKLVSNNILHNQQELPTALTKLVKEDEVV
SSEKAKDSFNEKRVAVEAPMREEYADFKPFERVWEVKDSKEDSDMLAAGGKIESNLESKV
DKKCFADSLEQTNHEKDSESSNDDTSFPSTPEGIKDRSGAYITCAPFNPAATESIATNIF
PLLGDPTSENKTDEKKIEEKKAQIVTEKNTSTKTSNPFLVAAQDSETDYVTTDNLTKVTE
EVVANMPEGLTPDLVQEACESELNEVTGTKIAYETKMDLVQTSEVMQESLYPAAQLCPSF
EESEATPSPVLPDIVMEAPLNSAVPSAGASVIQPSSSPLEASSVNYESIKHEPENPPPYE
EAMSVSLKKVSGIKEEIKEPENINAALQETEAPYISIACDLIKETKLSAEPAPDFSDYSE
MAKVEQPVPDHSELVEDSSPDSEPVDLFSDDSIPDVPQKQDETVMLVKESLTETSFESMI
EYENKEKLSALPPEGGKPYLESFKLSLDNTKDTLLPDEVSTLSKKEKIPLQMEELSTAVY
SNDDLFISKEAQIRETETFSDSSPIEIIDEFPTLISSKTDSFSKLAREYTDLEVSHKSEI
ANAPDGAGSLPCTELPHDLSLKNIQPKVEEKISFSDDFSKNGSATSKVLLLPPDVSALAT
QAEIESIVKPKVLVKEAEKKLPSDTEKEDRSPSAIFSAELSKTSVVDLLYWRDIKKTGVV
FGASLFLLLSLTVFSIVSVTAYIALALLSVTISFRIYKGVIQAIQKSDEGHPFRAYLESE
VAISEELVQKYSNSALGHVNCTIKELRRLFLVDDLVDSLKFAVLMWVFTYVGALFNGLTL
LILALISLFSVPVIYERHQAQIDHYLGLANKNVKDAMAKIQAKIPGLKRKAE
>sp|Q9NQE9|HINT3_HUMAN Adenosine 5'-monophosphoramidase HINT3 OS=Homo sapiens OX=9606 GN=HINT3 PE=1 SV=1
MAEEQVNRSAGLAPDCEASATAETTVSSVGTCEAAGKSPEPKDYDSTCVFCRIAGRQDPG
TELLHCENEDLICFKDIKPAATHHYLVVPKKHIGNCRTLRKDQVELVENMVTVGKTILER
NNFTDFTNVRMGFHMPPFCSISHLHLHVLAPVDQLGFLSKLVYRVNSYWFITADHLIEKL
RT
>sp|Q9NQR4|NIT2_HUMAN Omega-amidase NIT2 OS=Homo sapiens OX=9606 GN=NIT2 PE=1 SV=1
MTSFRLALIQLQISSIKSDNVTRACSFIREAATQGAKIVSLPECFNSPYGAKYFPEYAEK
IPGESTQKLSEVAKECSIYLIGGSIPEEDAGKLYNTCAVFGPDGTLLAKYRKIHLFDIDV
PGKITFQESKTLSPGDSFSTFDTPYCRVGLGICYDMRFAELAQIYAQRGCQLLVYPGAFN
LTTGPAHWELLQRSRAVDNQVYVATASPARDDKASYVAWGHSTVVNPWGEVLAKAGTEEA
IVYSDIDLKKLAEIRQQIPVFRQKRSDLYAVEMKKP
>sp|Q9NQW7|XPP1_HUMAN Xaa-Pro aminopeptidase 1 OS=Homo sapiens OX=9606 GN=XPNPEP1 PE=1 SV=3
MPPKVTSELLRQLRQAMRNSEYVTEPIQAYIIPSGDAHQSEYIAPCDCRRAFVSGFDGSA
GTAIITEEHAAMWTDGRYFLQAAKQMDSNWTLMKMGLKDTPTQEDWLVSVLPEGSRVGVD
PLIIPTDYWKKMAKVLRSAGHHLIPVKENLVDKIWTDRPERPCKPLLTLGLDYTGISWKD
KVADLRLKMAERNVMWFVVTALDEIAWLFNLRGSDVEHNPVFFSYAIIGLETIMLFIDGD
RIDAPSVKEHLLLDLGLEAEYRIQVHPYKSILSELKALCADLSPREKVWVSDKASYAVSE
TIPKDHRCCMPYTPICIAKAVKNSAESEGMRRAHIKDAVALCELFNWLEKEVPKGGVTEI
SAADKAEEFRRQQADFVDLSFPTISSTGPNGAIIHYAPVPETNRTLSLDEVYLIDSGAQY
KDGTTDVTRTMHFGTPTAYEKECFTYVLKGHIAVSAAVFPTGTKGHLLDSFARSALWDSG
LDYLHGTGHGVGSFLNVHEGPCGISYKTFSDEPLEAGMIVTDEPGYYEDGAFGIRIENVV
LVVPVKTKYNFNNRGSLTFEPLTLVPIQTKMIDVDSLTDKECDWLNNYHLTCRDVIGKEL
QKQGRQEALEWLIRETQPISKQH
>sp|Q9NT22|EMIL3_HUMAN EMILIN-3 OS=Homo sapiens OX=9606 GN=EMILIN3 PE=1 SV=2
MGRRRLLVWLCAVAALLSGAQARGTPLLARPAPPGASRYSLYTTGWRPRLRPGPHKALCA
YVVHRNVTCILQEGAESYVKAEYRQCRWGPKCPGTVTYRTVLRPKYKVGYKTVTDLAWRC

CPGFTGKRCPEHLTDHGAASPQLEPEPQIPSGQLDPGPRPPSYSRAAPSPHGRKGPGLFG
ERLERLEGDVQRLAQTYGTLSGLVASHEDPNRMTGGPRAPAVPVGFGVIPEGLVGPGDRA
RGPLTPPLDEILSKVTEVSNTLQTKVQLLDKVHGLALGHEAHLQRLREAPPSPLTSLALL
EEYVDRRLHRLWGSLLDGFEQKLQGVQSECDLRVQEVRRQCEEGQAASRRLHQSLDGREL
ALRQELSQLGSQLQGLSVSGRGSCCGQLALINARMDGLERALQAVTETQRGPGAPAGDEL
TRLSAAMLEGGVDGLLEGLETLNGTEGGARGCCLRLDMGGWGVGGFGTMLEERVQSLEER
LATLAGELSHDSASPGRSARPLVQTELAVLEQRLVSLETSCTPSTTSAILDSLVAEVKAW
QSRSEALLRQVASHAALLQQLNGTVAEVQGQLAEGTGSSLQGEITLLKVNLNSVSKSLTG
LSDSVSQYSDAFLAANTSLDERERKVEAEVQAIQEQVSSQGSRLQAGHRQVLNLRGELEQ
LKAGVAKVASGLSRCQDTAQKLQHTVGHFDQRVAQVEGACRRLGLLAAGLDSLPTEPLRP
REGLWSHVDQLNRTLAQHTQDIARLRDDLLDCQAQLAEQVRPGQAN
>sp|Q9NTI2|AT8A2_HUMAN Phospholipid-transporting ATPase IB OS=Homo sapiens OX=9606
GN=ATP8A2 PE=1 SV=3
MLNGAGLDKALKMSLPRRSRIRSSVGPVRSSLGYKKAEDEMSRATSVGDQLEAPARTIYL
NQPHLNKFRDNQISTAKYSVLTFLPRFLYEQIRRAANAFFLFIALLQQIPDVSPTGRYTT
LVPLIIILTIAGIKEIVEDFKRHKADNAVNKKKTIVLRNGMWHTIMWKEVAVGDIVKVVN
GQYLPADVVLLSSSEPQAMCYVETANLDGETNLKIRQGLSHTADMQTREVLMKLSGTIEC
EGPNRHLYDFTGNLNLDGKSLVALGPDQILLRGTQLRNTQWVFGIVVYTGHDTKLMQNST
KAPLKRSNVEKVTNVQILVLFGILLVMALVSSAGALYWNRSHGEKNWYIKKMDTTSDNFG
YNLLTFIILYNNLIPISLLVTLEVVKYTQALFINWDTDMYYIGNDTPAMARTSNLNEELG
QVKYLFSDKTGTLTCNIMNFKKCSIAGVTYGHFPELAREPSSDDFCRMPPPCSDSCDFDD
PRLLKNIEDRHPTAPCIQEFLTLLAVCHTVVPEKDGDNIIYQASSPDEAALVKGAKKLGF
VFTARTPFSVIIEAMGQEQTFGILNVLEFSSDRKRMSVIVRTPSGRLRLYCKGADNVIFE
RLSKDSKYMEETLCHLEYFATEGLRTLCVAYADLSENEYEEWLKVYQEASTILKDRAQRL
EECYEIIEKNLLLLGATAIEDRLQAGVPETIATLLKAEIKIWVLTGDKQETAINIGYSCR
LVSQNMALILLKEDSLDATRAAITQHCTDLGNLLGKENDVALIIDGHTLKYALSFEVRRS
FLDLALSCKAVICCRVSPLQKSEIVDVVKKRVKAITLAIGDGANDVGMIQTAHVGVGISG
NEGMQATNNSDYAIAQFSYLEKLLLVHGAWSYNRVTKCILYCFYKNVVLYIIELWFAFVN
GFSGQILFERWCIGLYNVIFTALPPFTLGIFERSCTQESMLRFPQLYKITQNGEGFNTKV
FWGHCINALVHSLILFWFPMKALEHDTVLTSGHATDYLFVGNIVYTYVVVTVCLKAGLET
TAWTKFSHLAVWGSMLTWLVFFGIYSTIWPTIPIAPDMRGQATMVLSSAHFWLGLFLVPT
ACLIEDVAWRAAKHTCKKTLLEEVQELETKSRVLGKAVLRDSNGKRLNERDRLIKRLGRK
TPPTLFRGSSLQQGVPHGYAFSQEEHGAVSQEEVIRAYDTTKKKSRKK
>sp|Q9NTJ4|MA2C1_HUMAN Alpha-mannosidase 2C1 OS=Homo sapiens OX=9606
GN=MAN2C1 PE=1 SV=1
MAAAPALKHWRTTLERVEKFVSPLYFTDCNLRGRLFGASCPVAVLSSFLTPERLPYQEAV
QRDFRPAQVGDSFGPTWWTCWFRVELTIPEAWVGQEVHLCWESDGEGLVWRDGEPVQGLT
KEGEKTSYVLTDRLGERDPRSLTLYVEVACNGLLGAGKGSMIAAPDPEKMFQLSRAELAV
FHRDVHMLLVDLELLLGIAKGLGKDNQRSFQALYTANQMVNVCDPAQPETFPVAQALASR
FFGQHGGESQHTIHATGHCHIDTAWLWPFKETVRKCARSWVTALQLMERNPEFIFACSQA
QQLEWVKSRYPGLYSRIQEFACRGQFVPVGGTWVEMDGNLPSGEAMVRQFLQGQNFFLQE
FGKMCSEFWLPDTFGYSAQLPQIMHGCGIRRFLTQKLSWNLVNSFPHHTFFWEGLDGSRV
LVHFPPGDSYGMQGSVEEVLKTVANNRDKGRANHSAFLFGFGDGGGGPTQTMLDRLKRLS
NTDGLPRVQLSSPRQLFSALESDSEQLCTWVGELFLELHNGTYTTHAQIKKGNRECERIL

HDVELLSSLALARSAQFLYPAAQLQHLWRLLLLNQFHDVVTGSCIQMVAEEAMCHYEDIR
SHGNTLLSAAAAALCAGEPGPEGLLIVNTLPWKRIEVMALPKPGGAHSLALVTVPSMGYA
PVPPPTSLQPLLPQQPVFVVQETDGSVTLDNGIIRVKLDPTGRLTSLVLVASGREAIAEG
AVGNQFVLFDDVPLYWDAWDVMDYHLETRKPVLGQAGTLAVGTEGGLRGSAWFLLQISPN
SRLSQEVVLDVGCPYVRFHTEVHWHEAHKFLKVEFPARVRSSQATYEIQFGHLQRPTHYN
TSWDWARFEVWAHRWMDLSEHGFGLALLNDCKYGASVRGSILSLSLLRAPKAPDATADTG
RHEFTYALMPHKGSFQDAGVIQAAYSLNFPLLALPAPSPAPATSWSAFSVSSPAVVLETV
KQAESSPQRRSLVLRLYEAHGSHVDCWLHLSLPVQEAILCDLLERPDPAGHLTLRDNRLK
LTFSPFQVLSLLLVLQPPPH
>sp|Q9NTK5|OLA1_HUMAN Obg-like ATPase 1 OS=Homo sapiens OX=9606 GN=OLA1 PE=1 SV=2
MPPKKGGDGIKPPPIIGRFGTSLKIGIVGLPNVGKSTFFNVLTNSQASAENFPFCTIDPN
ESRVPVPDERFDFLCQYHKPASKIPAFLNVVDIAGLVKGAHNGQGLGNAFLSHISACDGI
FHLTRAFEDDDITHVEGSVDPIRDIEIIHEELQLKDEEMIGPIIDKLEKVAVRGGDKKLK
PEYDIMCKVKSWVIDQKKPVRFYHDWNDKEIEVLNKHLFLTSKPMVYLVNLSEKDYIRKK
NKWLIKIKEWVDKYDPGALVIPFSGALELKLQELSAEERQKYLEANMTQSALPKIIKAGF
AALQLEYFFTAGPDEVRAWTIRKGTKAPQAAGKIHTDFEKGFIMAEVMKYEDFKEEGSEN
AVKAAGKYRQQGRNYIVEDGDIIFFKFNTPQQPKKK
>sp|Q9NUH8|TM14B_HUMAN Transmembrane protein 14B OS=Homo sapiens OX=9606 GN=TMEM14B PE=1 SV=1
MEKPLFPLVPLHWFGFGYTALVVSGGIVGYVKTGSVPSLAAGLLFGSLAGLGAYQLYQDP
RNVWGFLAATSVTFVGVMGMRSYYYGKFMPVGLIAGASLLMAAKVGVRMLMTSD
>sp|Q9NUQ9|CYRIB_HUMAN CYFIP-related Rac1 interactor B OS=Homo sapiens OX=9606 GN=CYRIB PE=1 SV=1
MGNLLKVLTCTDLEQGPNFFLDFENAQPTESEKEIYNQVNVVLKDAEGILEDLQSYRGAG
HEIREAIQHPADEKLQEKAWGAVVPLVGKLKKFYEFSQRLEAALRGLLGALTSTPYSPTQ
HLEREQALAKQFAEILHFTLRFDELKMTNPAIQNDFSYYRRTLSRMRINNVPAEGENEVN
NELANRMSLFYAEATPMLKTLSDATTKFVSENKNLPIENTTDCLSTMASVCRVMLETPEY
RSRFTNEETVSFCLRVMVGVIILYDHVHPVGAFAKTSKIDMKGCIKVLKDQPPNSVEGLL
NALRYTTKHLNDETTSKQIKSMLQ
>sp|Q9NUU7|DD19A_HUMAN ATP-dependent RNA helicase DDX19A OS=Homo sapiens OX=9606 GN=DDX19A PE=1 SV=1
MATDSWALAVDEQEAAVKSMTNLQIKEEKVKADTNGIIKTSTTAEKTDEEEKEDRAAQSL
LNKLIRSNLVDNTNQVEVLQRDPNSPLYSVKSFEELRLKPQLLQGVYAMGFNRPSKIQEN
ALPMMLAEPPQNLIAQSQSGTGKTAAFVLAMLSRVEPSDRYPQCLCLSPTYELALQTGKV
IEQMGKFYPELKLAYAVRGNKLERGQKISEQIVIGTPGTVLDWCSKLKFIDPKKIKVFVL
DEADVMIATQGHQDQSIRIQRMLPRNCQMLLFSATFEDSVWKFAQKVVPDPNVIKLKREE
ETLDTIKQYYVLCSSRDEKFQALCNLYGAITIAQAMIFCHTRKTASWLAAELSKEGHQVA
LLSGEMMVEQRAAVIERFREGKEKVLVTTNVCARGIDVEQVSVVINFDLPVDKDGNPDNE
TYLHRIGRTGRFGKRGLAVNMVDSKHSMNILNRIQEHFNKKIERLDTDDLDEIEKIAN
>sp|Q9NV96|CC50A_HUMAN Cell cycle control protein 50A OS=Homo sapiens OX=9606 GN=TMEM30A PE=1 SV=1
MAMNYNAKDEVDGGPPCAPGGTAKTRRPDNTAFKQQRLPAWQPILTAGTVLPIFFIIGLI
FIPIGIGIFVTSNNIREIEIDYTGTEPSSPCNKCLSPDVTPCFCTINFTLEKSFEGNVFM

YYGLSNFYQNHRRYVKSRDDSQLNGDSSALLNPSKECEPYRRNEDKPIAPCGAIANSMFN
DTLELFLIGNDSYPIPIALKKKGIAWWTDKNVKFRNPPGGDNLEERFKGTTKPVNWLKPV
YMLDSDPDNNGFINEDFIVWMRTAALPTFRKLYRLIERKSDLHPTLPAGRYSLNVTYNYP
VHYFDGRKRMILSTISWMGGKNPFLGIAYIAVGSISFLLGVVLLVINHKYRNSSNTADIT
I
>sp|Q9NY33|DPP3_HUMAN Dipeptidyl peptidase 3 OS=Homo sapiens OX=9606 GN=DPP3 PE=1 SV=2
MADTQYILPNDIGVSSLDCREAFRLLSPTERLYAYHLSRAAWYGGLAVLLQTSPEAPYIY
ALLSRLFRAQDPDQLRQHALAEGLTEEEYQAFLVYAAGVYSNMGNYKSFGDTKFVPNLPK
EKLERVILGSEAAQQHPEEVRGLWQTCGELMFSLEPRLRHLGLGKEGITTYFSGNCTMED
AKLAQDFLDSQNLSAYNTRLFKEVDGEGKPYYEVRLASVLGSEPSLDSEVTSKLKSYEFR
GSPFQVTRGDYAPILQKVVEQLEKAKAYAANSHQGQMLAQYIESFTQGSIEAHKRGSRFW
IQDKGPIVESYIGFIESYRDPFGSRGEFEGFVAVVNKAMSAKFERLVASAEQLLKELPWP
PTFEKDKFLTPDFTSLDVLTFAGSGIPAGINIPNYDDLRQTEGFKNVSLGNVLAVAYATQ
REKLTFLEEDDKDLYILWKGPSFDVQVGLHELLGHGSGKLFVQDEKGAFNFDQETVINPE
TGEQIQSWYRSGETWDSKFSTIASSYEECRAESVGLYLCLHPQVLEIFGFEGADAEDVIY
VNWLNMVRAGLLALEFYTPEAFNWRQAHMQARFVILRVLLEAGEGLVTITPTTGSDGRPD
ARVRLDRSKIRSVGKPALERFLRRLQVLKSTGDVAGGRALYEGYATVTDAPPECFLTLRD
TVLLRKESRKLIVQPNTRLEGSDVQLLEYEASAAGLIRSFSERFPEDGPELEEILTQLAT
ADARFWKGPSEAPSGQA
>sp|Q9NZ01|TECR_HUMAN Very-long-chain enoyl-CoA reductase OS=Homo sapiens OX=9606 GN=TECR PE=1 SV=1
MKHYEVEILDAKTREKLCFLDKVEPHATIAEIKNLFTKTHPQWYPARQSLRLDPKGKSLK
DEDVLQKLPVGTTATLYFRDLGAQISWVTVFLTEYAGPLFIYLLFYFRVPFIYGHKYDFT
SSRHTVVHLACICHSFHYIKRLLETLFVHRFSHGTMPLRNIFKNCTYYWGFAAWMAYYIN
HPLYTPPTYGAQQVKLALAIFVICQLGNFSIHMALRDLRPAGSKTRKIPYPTKNPFTWLF
LLVSCPNYTYEVGSWIGFAIMTQCLPVALFSLVGFTQMTIWAKGKHRSYLKEFRDYPPLR
MPIIPFLL
>sp|Q9NZ32|ARP10_HUMAN Actin-related protein 10 OS=Homo sapiens OX=9606 GN=ACTR10 PE=1 SV=1
MPLYEGLGSGGEKTAVVIDLGEAFTKCGFAGETGPRCIIPSVIKRAGMPKPVRVVQYNIN
TEELYSYLKEFIHILYFRHLLVNPRDRRVVIIESVLCPSHFRETLTRVLFKYFEVPSVLL
APSHLMALLTLGINSAMVLDCGYRESLVLPIYEGIPVLNCWGALPLGGKALHKELETQLL
EQCTVDTSVAKEQSLPSVMGSVPEGVLEDIKARTCFVSDLKRGLKIQAAKFNIDGNNERP
SPPPNVDYPLDGEKILHILGSIRDSVVEILFEQDNEEQSVATLILDSLIQCPIDTRKQLA
ENLVVIGGTSMLPGFLHRLLAEIRYLVEKPKYKKALGTKTFRIHTPPAKANCVAWLGGAI
FGALQDILGSRSVSKEYYNQTGRIPDWCSLNNPPLEMMFDVGKTQPPLMKRAFSTEK
>sp|Q9NZA1|CLIC5_HUMAN Chloride intracellular channel protein 5 OS=Homo sapiens OX=9606 GN=CLIC5 PE=1 SV=3
MNDEDYSTIYDTIQNERTYEVPDQPEENESPHYDDVHEYLRPENDLYATQLNTHEYDFVS
VYTIKGEETSLASVQSEDRGYLLPDEIYSELQEAHPGEPQEDRGISMEGLYSSTQDQQLC
AAELQENGSVMKEDLPSPSSFTIQHSKAFSTTKYSCYSDAEGLEEKEGAHMNPEIYLFVK
AGIDGESIGNCPFSQRLFMILWLKGVVFNVTTVDLKRKPADLHNLAPGTHPPFLTFNGDV
KTDVNKIEEFLEETLTPEKYPKLAAKHRESNTAGIDIFSKFSAYIKNTKQQNNAALERGL

TKALKKLDDYLNTPLPEEIDANTCGEDKGSRRKFLDGDELTLADCNLLPKLHVVKIVAKK
YRNYDIPAEMTGLWRYLKNAYARDEFTNTCAADSEIELAYADVAKRLSRS
>sp|Q9NZH0|GPC5B_HUMAN G-protein coupled receptor family C group 5 member B OS=Homo sapiens OX=9606 GN=GPRC5B PE=2 SV=2
MFVASERKMRAHQVLTFLLLFVITSVASENASTSRGCGLDLLPQYVSLCDLDAIWGIVVE
AVAGAGALITLLLMLILLVRLPFIKEKEKKSPVGLHFLFLLGTLGLFGLTFAFIIQEDET
ICSVRRFLWGVLFALCFSCLLSQAWRVRRLVRHGTGPAGWQLVGLALCLMLVQVIIAVEW
LVLTVLRDTRPACAYEPMDFVMALIYDMVLLVVTLGLALFTLCGKFKRWKLNGAFLLITA
FLSVLIWVAWMTMYLFGNVKLQQGDAWNDPTLAITLAASGWVFVIFHAIPEIHCTLLPAL
QENTPNYFDTSQPRMRETAFEEDVQLPRAYMENKAFSMDEHNAALRTAGFPNGSLGKRPS
GSLGKRPSAPFRSNVYQPTEMAVVLNGGTIPTAPPSHTGRHLW
>sp|Q9NZJ7|MTCH1_HUMAN Mitochondrial carrier homolog 1 OS=Homo sapiens OX=9606 GN=MTCH1 PE=1 SV=1
MGASDPEVAPWARGGAAGMAGAGAGAGARGGAAAGVEARARDPPPAHRAHPRHPRPAAQP
SARRMDGGSGGLGSGDNAPTTEALFVALGAGVTALSHPLLYVKLLIQVGHEPMPPTLGTN
VLGRKVLYLPSFFTYAKYIVQVDGKIGLFRGLSPRLMSNALSTVTRGSMKKVFPPDEIEQ
VSNKDDMKTSLKKVVKETSYEMMMQCVSRMLAHPLHVISMRCMVQFVGREAKYSGVLSSI
GKIFKEEGLLGFFVGLIPHLLGDVVFLWGCNLLAHFINAYLVDDSVSDTPGGLGNDQNPG
SQFSQALAIRSYTKFVMGIAVSMLTYPFLLVGDLMAVNNCGLQAGLPPYSPVFKSWIHCW
KYLSVQGQLFRGSSLLFRRVSSGSCFALE
>sp|Q9NZL4|HPBP1_HUMAN Hsp70-binding protein 1 OS=Homo sapiens OX=9606 GN=HSPBP1 PE=1 SV=2
MSDEGSRGSRLPLALPPASQGCSSGGGGGGSSAGGSGNSRPPRNLQGLLQMAITAGSEEP
DPPPEPMSEERRQWLQEAMSAAFRGQREEVEQMKSCLRVLSQPMPPTAGEAEQAADQQER
EGALELLADLCENMDNAADFCQLSGMHLLVGRYLEAGAAGLRWRAAQLIGTCSQNVAAIQ
EQVLGLGALRKLLRLLDRDACDTVRVKALFAISCLVREQEAGLLQFLRLDGFSVLMRAMQ
QQVQKLKVKSAFLLQNLLVGHPEHKGTLCSMGMVQQLVALVRTEHSPFHEHVLGALCSLV
TDFPQGVRECREPELGLEELLRHRCQLLQQHEEYQEELEFCEKLLQTCFSSPADDSMDR
>sp|Q9NZL9|MAT2B_HUMAN Methionine adenosyltransferase 2 subunit beta OS=Homo sapiens OX=9606 GN=MAT2B PE=1 SV=1
MVGREKELSIHFVPGSCRLVEEEVNIPNRRVLVTGATGLLGRAVHKEFQQNNWHAVGCGF
RRARPKFEQVNLLDSNAVHHIIHDFQPHVIVHCAAERRPDVVENQPDAASQLNVDASGNL
AKEAAAVGAFLIYISSDYVFDGTNPPYREEDIPAPLNLYGKTKLDGEKAVLENNLGAAVL
RIPILYGEVEKLEESAVTVMFDKVQFSNKSANMDHWQQRFPTHVKDVATVCRQLAEKRML
DPSIKGTFHWSGNEQMTKYEMACAIADAFNLPSSHLRPITDSPVLGAQRPRNAQLDCSKL
ETLGIGQRTPFRIGIKESLWPFLIDKRWRQTVFH
>sp|Q9P035|HACD3_HUMAN Very-long-chain (3R)-3-hydroxyacyl-CoA dehydratase 3 OS=Homo sapiens OX=9606 GN=HACD3 PE=1 SV=2
MENQVLTPHVYWAQRHRELYLRVELSDVQNPAISITENVLHFKAQGHGAKGDNVYEFHLE
FLDLVKPEPVYKLTQRQVNITVQKKVSQWWERLTKQEKRPLFLAPDFDRWLDESDAEMEL
RAKEEERLNKLRLESEGSPETLTNLRKGYLFMYNLVQFLGFSWIFVNLTVRFCILGKESF
YDTFHTVADMMYFCQMLAVVETINAAIGVTTSPVLPSLIQLLGRNFILFIIFGTMEEMQN
KAVVFFVFYLWSAIEIFRYSFYMLTCIDMDWKVLTWLRYTLWIPLYPLGCLAEAVSVIQS
IPIFNETGRFSFTLPYPVKIKVRFSFFLQIYLIMIFLGLYINFRHLYKQRRRRYGQKKKK

IH
>sp|Q9P0J0|NDUAD_HUMAN NADH dehydrogenase [ubiquinone] 1 alpha subcomplex subunit 13 OS=Homo sapiens OX=9606 GN=NDUFA13 PE=1 SV=3
MAASKVKQDMPPPGGYGPIDYKRNLPRRGLSGYSMLAIGIGTLIYGHWSIMKWNRERRRL
QIEDFEARIALLPLLQAETDRRTLQMLRENLEEEAIIMKDVPDWKVGESVFHTTRWVPPL
IGELYGLRTTEEALHASHGFMWYT
>sp|Q9P0L0|VAPA_HUMAN Vesicle-associated membrane protein-associated protein A OS=Homo sapiens OX=9606 GN=VAPA PE=1 SV=3
MASASGAMAKHEQILVLDPPTDLKFKGPFTDVVTTNLKLRNPSDRKVCFKVKTTAPRRYC
VRPNSGIIDPGSTVTVSVMLQPFDYDPNEKSKHKFMVQTIFAPPNTSDMEAVWKEAKPDE
LMDSKLRCVFEMPNENDKLNDMEPSKAVPLNASKQDGPMPKPHSVSLNDTETRKLMEECK
RLQGEMMKLSEENRHLRDEGLRLRKVAHSDKPGSTSTASFRDNVTSPLPSLLVVIAAIFI
GFFLGKFIL
>sp|Q9P0S9|TM14C_HUMAN Transmembrane protein 14C OS=Homo sapiens OX=9606 GN=TMEM14C PE=1 SV=1
MQDTGSVVPLHWFGFGYAALVASGGIIGYVKAGSVPSLAAGLLFGSLAGLGAYQLSQDPR
NVWVFLATSGTLAGIMGMRFYHSGKFMPAGLIAGASLLMVAKVGVSMFNRPH
>sp|Q9P265|DIP2B_HUMAN Disco-interacting protein 2 homolog B OS=Homo sapiens OX=9606 GN=DIP2B PE=1 SV=3
MAERGLEPSPAAVAALPPEVRAQLAELELELSEGDITQKGYEKKRSKLLSPYSPQTQETD
SAVQKELRNQTPAPSAAQTSAPSKYHRTRSGGARDERYRSDIHTEAVQAALAKHKEQKMA
LPMPTKRRSTFVQSPADACTPPDTSSASEDEGSLRRQAALSAALQQSLQNAESWINRSIQ
GSSTSSSASSTLSHGEVKGTSGSLADVFANTRIENFSAPPDVTTTTSSSSSSSSIRPANI
DLPPSGIVKGMHKGSNRSSLMDTADGVPVSSRVSTKIQQLLNTLKRPKRPPLKEFFVDDS
EEIVEVPQPDPNQPKPEGRQMTPVKGEPLGVICNWPPALESALQRWGTTQAKCSCLTALD
MTGKPVYTLTYGKLWSRSLKLAYTLLNKLGTKNEPVLKPGDRVALVYPNNDPVMFMVAFY
GCLLAEVIPVPIEVPLTRKDAGGQQIGFLLGSCGIALALTSEVCLKGLPKTQNGEIVQFK
GWPRLKWVVTDSKYLSKPPKDWQPHISPAGTEPAYIEYKTSKEGSVMGVTVSRLAMLSHC
QALSQACNYSEGETIVNVLDFKKDAGLWHGMFANVMNKMHTISVPYSVMKTCPLSWVQRV
HAHKAKVALVKCRDLHWAMMAHRDQRDVSLSSLRMLIVTDGANPWSVSSCDAFLSLFQSH
GLKPEAICPCATSAEAMTVAIRRPGVPGAPLPGRAILSMNGLSYGVIRVNTEDKNSALTV
QDVGHVMPGGMMCIVKPDGPPQLCKTDEIGEICVSSRTGGMMYFGLAGVTKNTFEVIPVN
SAGSPVGDVPFIRSGLLGFVGPGSLVFVVGKMDGLLMVSGRRHNADDIVATGLAVESIKT
VYRGRIAVFSVSVFYDERIVVVAEQRPDASEEDSFQWMSRVLQAIDSIHQVGVYCLALVP
ANTLPKTPLGGIHISQTKQLFLEGSLHPCNILMCPHTCVTNLPKPRQKQPGVGPASVMVG
NLVAGKRIAQAAGRDLGQIEENDLVRKHQFLAEILQWRAQATPDHVLFMLLNAKGTTVCT
ASCLQLHKRAERIASVLGDKGHLNAGDNVVLLYPPGIELIAAFYGCLYAGCIPVTVRPPH
AQNLTATLPTVRMIVDVSKAACILTSQTLMRLLRSREAAAAVDVKTWPTIIDTDDLPRKR
LPQLYKPPTPEMLAYLDFSVSTTGMLTGVKMSHSAVNALCRAIKLQCELYSSRQIAICLD
PYCGLGFALWCLCSVYSGHQSVLIPPMELENNLFLWLSTVNQYKIRDTFCSYSVMELCTK
GLGNQVEVLKTRGINLSCVRTCVVVAEERPRVALQQSFSKLFKDIGLSPRAVSTTFGSRV
NVAICLQGTSGPDPTTVYVDLKSLRHDRVRLVERGAPQSLLLSESGKILPGVKVVIVNPE
TKGPVGDSHLGEIWVNSPHTASGYYTIYDSETLQADHFNTRLSFGDAAQTLWARTGYLGF
VRRTELTAATGERHDALYVVGALDETLELRGLRYHPIDIETSVSRIHRSIAECAVFTWTN

LLVVVVELCGSEQEALDLVPLVTNVVLEEHYLIVGVVVVVDPGVIPINSRGEKQRMHLRD
SFLADQLDPIYVAYNM
>sp|Q9P273|TEN3_HUMAN Teneurin-3 OS=Homo sapiens OX=9606 GN=TENM3 PE=1 SV=3
MDVKERRPYCSLTKSRREKERRYTNSSADNEECRVPTQKSYSSSETLKAFDHDSSRLLYG
NRVKDLVHREADEFTRQGQNFTLRQLGVCEPATRRGLAFCAEMGLPHRGYSISAGSDADT
ENEAVMSPEHAMRLWGRGVKSGRSSCLSSRSNSALTLTDTEHENKSDSENEQPASNQGQS
TLQPLPPSHKQHSAQHHPSITSLNRNSLTNRRNQSPAPPAALPAELQTTPESVQLQDSWV
LGSNVPLESRHFLFKTGTGTTPLFSTATPGYTMASGSVYSPPTRPLPRNTLSRSAFKFKK
SSKYCSWKCTALCAVGVSVLLAILLSYFIAMHLFGLNWQLQQTENDTFENGKVNSDTMPT
NTVSLPSGDNGKLGGFTQENNTIDSGELDIGRRAIQEIPPGIFWRSQLFIDQPQFLKFNI
SLQKDALIGVYGRKGLPPSHTQYDFVELLDGSRLIAREQRSLLETERAGRQARSVSLHEA
GFIQYLDSGIWHLAFYNDGKNAEQVSFNTIVIESVVECPRNCHGNGECVSGTCHCFPGFL
GPDCSRAACPVLCSGNGQYSKGRCLCFSGWKGTECDVPTTQCIDPQCGGRGICIMGSCAC
NSGYKGESCEEADCIDPGCSNHGVCIHGECHCSPGWGGSNCEILKTMCPDQCSGHGTYLQ
ESGSCTCDPNWTGPDCSNEICSVDCGSHGVCMGGTCRCEEGWTGPACNQRACHPRCAEHG
TCKDGKCECSQGWNGEHCTIEGCPGLCNSNGRCTLDQNGWHCVCQPGWRGAGCDVAMETL
CTDSKDNEGDGLIDCMDPDCCLQSSCQNQPYCRGLPDPQDIISQSLQSPSQQAAKSFYDR
ISFLIGSDSTHVIPGESPFNKSLASVIRGQVLTADGTPLIGVNVSFFHYPEYGYTITRQD
GMFDLVANGGASLTLVFERSPFLTQYHTVWIPWNVFYVMDTLVMKKEENDIPSCDLSGFV
RPNPIIVSSPLSTFFRSSPEDSPIIPETQVLHEETTIPGTDLKLSYLSSRAAGYKSVLKI
TMTQSIIPFNLMKVHLMVAVVGRLFQKWFPASPNLAYTFIWDKTDAYNQKVYGLSEAVVS
VGYEYESCLDLTLWEKRTAILQGYELDASNMGGWTLDKHHVLDVQNGILYKGNGENQFIS
QQPPVVSSIMGNGRRRSISCPSCNGQADGNKLLAPVALACGIDGSLYVGDFNYVRRIFPS
GNVTSVLELSSNPAHRYYLATDPVTGDLYVSDTNTRRIYRPKSLTGAKDLTKNAEVVAGT
GEQCLPFDEARCGDGGKAVEATLMSPKGMAVDKNGLIYFVDGTMIRKVDQNGIISTLLGS
NDLTSARPLTCDTSMHISQVRLEWPTDLAINPMDNSIYVLDNNVVLQITENRQVRIAAGR
PMHCQVPGVEYPVGKHAVQTTLESATAIAVSYSGVLYITETDEKKINRIRQVTTDGEISL
VAGIPSECDCKNDANCDCYQSGDGYAKDAKLSAPSSLAASPDGTLYIADLGNIRIRAVSK
NKPLLNSMNFYEVASPTDQELYIFDINGTHQYTVSLVTGDYLYNFSYSNDNDITAVTDSN
GNTLRIRRDPNRMPVRVVSPDNQVIWLTIGTNGCLKSMTAQGLELVLFTYHGNSGLLATK
SDETGWTTFFDYDSEGRLTNVTFPTGVVTNLHGDMDKAITVDIESSSREEDVSITSNLSS
IDSFYTMVQDQLRNSYQIGYDGSLRIIYASGLDSHYQTEPHVLAGTANPTVAKRNMTLPG
ENGQNLVEWRFRKEQAQGKVNVFGRKLRVNGRNLLSVDFDRTTKTEKIYDDHRKFLLRIA
YDTSGHPTLWLPSSKLMAVNVTYSSTGQIASIQRGTTSEKVDYDGQGRIVSRVFADGKTW
SYTYLEKSMVLLLHSQRQYIFEYDMWDRLSAITMPSVARHTMQTIRSIGYYRNIYNPPES
NASIITDYNEEGLLLQTAFLGTSRRVLFKYRRQTRLSEILYDSTRVSFTYDETAGVLKTV
NLQSDGFICTIRYRQIGPLIDRQIFRFSEDGMVNARFDYSYDNSFRVTSMQGVINETPLP
IDLYQFDDISGKVEQFGKFGVIYYDINQIISTAVMTYTKHFDAHGRIKEIQYEIFRSLMY
WITIQYDNMGRVTKREIKIGPFANTTKYAYEYDVDGQLQTVYLNEKIMWRYNYDLNGNLH
LLNPSNSARLTPLRYDLRDRITRLGDVQYRLDEDGFLRQRGTEIFEYSSKGLLTRVYSKG
SGWTVIYRYDGLGRRVSSKTSLGQHLQFFYADLTYPTRITHVYNHSSSEITSLYYDLQGH
LFAMEISSGDEFYIASDNTGTPLAVFSSNGLMLKQIQYTAYGEIYFDSNIDFQLVIGFHG
GLYDPLTKLIHFGERDYDILAGRWTTPDIEIWKRIGKDPAPFNLYMFRNNNPASKIHDVK
DYITDVNSWLVTFGFHLHNAIPGFPVPKFDLTEPSYELVKSQQWDDIPPIFGVQQQVARQ

AKAFLSLGKMAEVQVSRRRAGGAQSWLWFATVKSLIGKGVMLAVSQGRVQTNVLNIANED
CIKVAAVLNNAFYLENLHFTIEGKDTHYFIKTTTPESDLGTLRLTSGRKALENGINVTVS
QSTTVVNGRTRRFADVEMQFGALALHVRYGMTLDEEKARILEQARQRALARAWAREQQRV
RDGEEGARLWTEGEKRQLLSAGKVQGYDGYYVLSVEQYPELADSANNIQFLRQSEIGRR
>sp|Q9P2T1|GMPR2_HUMAN GMP reductase 2 OS=Homo sapiens OX=9606 GN=GMPR2 PE=1 SV=1
MPHIDNDVKLDFKDVLLRPKRSTLKSRSEVDLTRSFSFRNSKQTYSGVPIIAANMDTVGT
FEMAKVLCKFSLFTAVHKHYSLVQWQEFAGQNPDCLEHLAASSGTGSSDFEQLEQILEAI
PQVKYICLDVANGYSEHFVEFVKDVRKRFPQHTIMAGNVVTGEMVEELILSGADIIKVGI
GPGSVCTTRKKTGVGYPQLSAVMECADAAHGLKGHIISDGGCSCPGDVAKAFGAGADFVM
LGGMLAGHSESGGELIERDGKKYKLFYGMSSEMAMKKYAGGVAEYRASEGKTVEVPFKGD
VEHTIRDILGGIRSTCTYVGAAKLKELSRRTTFIRVTQQVNPIFSEAC
>sp|Q9UBB4|ATX10_HUMAN Ataxin-10 OS=Homo sapiens OX=9606 GN=ATXN10 PE=1 SV=1
MAAPRPPPARLSGVMVPAPIQDLEALRALTALFKEQRNRETAPRTIFQRVLDILKKSSHA
VELACRDPSQVENLASSLQLITECFRCLRNACIECSVNQNSIRNLDTIGVAVDLILLFRE
LRVEQESLLTAFRCGLQFLGNIASRNEDSQSIVWVHAFPELFLSCLNHPDKKIVAYSSMI
LFTSLNHERMKELEENLNIAIDVIDAYQKHPESEWPFLIITDLFLKSPELVQAMFPKLNN
QERVTLLDLMIAKITSDEPLTKDDIPVFLRHAELIASTFVDQCKTVLKLASEEPPDDEEA
LATIRLLDVLCEMTVNTELLGYLQVFPGLLERVIDLLRVIHVAGKETTNIFSNCGCVRAE
GDISNVANGFKSHLIRLIGNLCYKNKDNQDKVNELDGIPLILDNCNISDSNPFLTQWVIY
AIRNLTEDNSQNQDLIAKMEEQGLADASLLKKVGFEVEKKGEKLILKSTRDTPKP
>sp|Q9UBN7|HDAC6_HUMAN Histone deacetylase 6 OS=Homo sapiens OX=9606 GN=HDAC6 PE=1 SV=2
MTSTGQDSTTTRQRRSRQNPQSPPQDSSVTSKRNIKKGAVPRSIPNLAEVKKKGKMKKLG
QAMEEDLIVGLQGMDLNLEAEALAGTGLVLDEQLNEFHCLWDDSFPEGPERLHAIKEQLI
QEGLLDRCVSFQARFAEKEELMLVHSLEYIDLMETTQYMNEGELRVLADTYDSVYLHPNS
YSCACLASGSVLRLVDAVLGAEIRNGMAIIRPPGHHAQHSLMDGYCMFNHVAVAARYAQQ
KHRIRRVLIVDWDVHHGQGTQFTFDQDPSVLYFSIHRYEQGRFWPHLKASNWSTTGFGQG
QGYTINVPWNQVGMRDADYIAAFLHVLLPVALEFQPQLVLVAAGFDALQGDPKGEMAATP
AGFAQLTHLLMGLAGGKLILSLEGGYNLRALAEGVSASLHTLLGDPCPMLESPGAPCRSA
QASVSCALEALEPFWEVLVRSTETVERDNMEEDNVEESEEEGPWEPPVLPILTWPVLQSR
TGLVYDQNMMNHCNLWDSHHPEVPQRILRIMCRLEELGLAGRCLTLTPRPATEAELLTCH
SAEYVGHLRATEKMKTRELHRESSNFDSIYICPSTFACAQLATGAACRLVEAVLSGEVLN
GAAVVRPPGHHAEQDAACGFCFFNSVAVAARHAQTISGHALRILIVDWDVHHGNGTQHMF
EDDPSVLYVSLHRYDHGTFFPMGDEGASSQIGRAAGTGFTVNVAWNGPRMGDADYLAAWH
RLVLPIAYEFNPELVLVSAGFDAARGDPLGGCQVSPEGYAHLTHLLMGLASGRIILILEG
GYNLTSISESMAACTRSLLGDPPPLLTLPRPPLSGALASITETIQVHRRYWRSLRVMKVE
DREGPSSSKLVTKKAPQPAKPRLAERMTTREKKVLEAGMGKVTSASFGEESTPGQTNSET
AVVALTQDQPSEAATGGATLAQTISEAAIGGAMLGQTTSEEAVGGATPDQTTSEETVGGA
ILDQTTSEDAVGGATLGQTTSEEAVGGATLAQTTSEAAMEGATLDQTTSEEAPGGTELIQ
TPLASSTDHQTPPTSPVQGTTPQISPSTLIGSLRTLELGSESQGASESQAPGEENLLGEA
AGGQDMADSMLMQGSRGLTDQAIFYAVTPLPWCPHLVAVCPIPAAGLDVTQPCGDCGTIQ
ENWVCLSCYQVYCGRYINGHMLQHHGNSGHPLVLSYIDLSAWCYYCQAYVHHQALLDVKN
IAHQNKFGEDMPHPH

>sp|Q9UBW8|CSN7A_HUMAN COP9 signalosome complex subunit 7a OS=Homo sapiens OX=9606 GN=COPS7A PE=1 SV=1
MSAEVKVTGQNQEQFLLLAKSAKGAALATLIHQVLEAPGVYVFGELLDMPNVRELAESDF
ASTFRLLTVFAYGTYADYLAEARNLPPLTEAQKNKLRHLSVVTLAAKVKCIPYAVLLEAL
ALRNVRQLEDLVIEAVYADVLRGSLDQRNQRLEVDYSIGRDIQRQDLSAIARTLQEWCVG
CEVVLSGIEEQVSRANQHKEQQLGLKQQIESEVANLKKTIKVTTAAAAAATSQDPEQHLT
ELREPAPGTNQRQPSKKASKGKGLRGSAKIWSKSN
>tr|Q9UFA7|Q9UFA7_HUMAN Gamma-crystallin S OS=Homo sapiens OX=9606 GN=DKFZp434A0627 PE=2 SV=1
MYILPQGEYPEYQRWMGLNDRLSSCRAVHLPSGGQYKIQIFEKGDFSGQMYETTEDCPSI
MEQFHMREIHSCKVLEGVWIFYELPNYRGRQYLLDKKEYRKPIDWGAASPAVQSFRRIVE
>sp|Q9UGL1|KDM5B_HUMAN Lysine-specific demethylase 5B OS=Homo sapiens OX=9606 GN=KDM5B PE=1 SV=3
MEAATTLHPGPRPALPLGGPGPLGEFLPPPECPVFEPSWEEFADPFAFIHKIRPIAEQTG
ICKVRPPPDWQPPFACDVDKLHFTPRIQRLNELEAQTRVKLNFLDQIAKYWELQGSTLKI
PHVERKILDLFQLNKLVAEEGGFAVVCKDRKWTKIATKMGFAPGKAVGSHIRGHYERILN
PYNLFLSGDSLRCLQKPNLTTDTKDKEYKPHDIPQRQSVQPSETCPPARRAKRMRAEAMN
IKIEPEETTEARTHNLRRRMGCPTPKCENEKEMKSSIKQEPIERKDYIVENEKEKPKSRS
KKATNAVDLYVCLLCGSGNDEDRLLLCDGCDDSYHTFCLIPPLHDVPKGDWRCPKCLAQE
CSKPQEAFGFEQAARDYTLRTFGEMADAFKSDYFNMPVHMVPTELVEKEFWRLVSTIEED
VTVEYGADIASKEFGSGFPVRDGKIKLSPEEEEYLDSGWNLNNMPVMEQSVLAHITADIC
GMKLPWLYVGMCFSSFCWHIEDHWSYSINYLHWGEPKTWYGVPGYAAEQLENVMKKLAPE
LFVSQPDLLHQLVTIMNPNTLMTHEVPVYRTNQCAGEFVITFPRAYHSGFNQGFNFAEAV
NFCTVDWLPLGRQCVEHYRLLHRYCVFSHDEMICKMASKADVLDVVVASTVQKDMAIMIE
DEKALRETVRKLGVIDSERMDFELLPDDERQCVKCKTTCFMSAISCSCKPGLLVCLHHVK
ELCSCPPYKYKLRYRYTLDDLYPMMNALKLRAESYNEWALNVNEALEAKINKKKSLVSFK
ALIEESEMKKFPDNDLLRHLRLVTQDAEKCASVAQQLLNGKRQTRYRSGGGKSQNQLTVN
ELRQFVTQLYALPCVLSQTPLLKDLLNRVEDFQQHSQKLLSEETPSAAELQDLLDVSFEF
DVELPQLAEMRIRLEQARWLEEVQQACLDPSSLTLDDMRRLIDLGVGLAPYSAVEKAMAR
LQELLTVSEHWDDKAKSLLKARPRHSLNSLATAVKEIEEIPAYLPNGAALKDSVQRARDW
LQDVEGLQAGGRVPVLDTLIELVTRGRSIPVHLNSLPRLETLVAEVQAWKECAVNTFLTE
NSPYSLLEVLCPRCDIGLLGLKRKQRKLKEPLPNGKKKSTKLESLSDLERALTESKETAS
AMATLGEARLREMEALQSLRLANEGKLLSPLQDVDIKICLCQKAPAAPMIQCELCRDAFH
TSCVAVPSISQGLRIWLCPHCRRSEKPPLEKILPLLASLQRIRVRLPEGDALRYMIERTV
NWQHRAQQLLSSGNLKFVQDRVGSGLLYSRWQASAGQVSDTNKVSQPPGTTSFSLPDDWD
NRTSYLHSPFSTGRSCIPLHGVSPEVNELLMEAQLLQVSLPEIQELYQTLLAKPSPAQQT
DRSSPVRPSSEKNDCCRGKRDGINSLERKLKRRLEREGLSSERWERVKKMRTPKKKKIKL
SHPKDMNNFKLERERSYELVRSAETHSLPSDTSYSEQEDSEDEDAICPAVSCLQPEGDEV
DWVQCDGSCNQWFHQVCVGVSPEMAEKEDYICVRCTVKDAPSRK
>sp|Q9UGT4|SUSD2_HUMAN Sushi domain-containing protein 2 OS=Homo sapiens OX=9606 GN=SUSD2 PE=1 SV=1
MKPALLPWALLLLATALGPGPGPTADAQESCSMRCGALDGPCSCHPTCSGLGTCCLDFRD
FCLEILPYSGSMMGGKDFVVRHFKMSSPTDASVICRFKDSIQTLGHVDSSGQVHCVSPLL
YESGRIPFTVSLDNGHSFPRAGTWLAVHPNKVSMMEKSELVNETRWQYYGTANTSGNLSL

TWHVKSLPTQTITIELWGYEETGMPYSQEWTAKWSYLYPLATHIPNSGSFTFTPKPAPPS
YQRWRVGALRIIDSKNYAGQKDVQALWTNDHALAWHLSDDFREDPVAWARTQCQAWEELE
DQLPNFLEELPDCPCTLTQARADSGRFFTDYGCDMEQGSVCTYHPGAVHCVRSVQASLRY
GSGQQCCYTADGTQLLTADSSGGSTPDRGHDWGAPPFRTPPRVPSMSHWLYDVLSFYYCC
LWAPDCPRYMQRRPSNDCRNYRPPRLASAFGDPHFVTFDGTNFTFNGRGEYVLLEAALTD
LRVQARAQPGTMSNGTETRGTGLTAVAVQEGNSDVVEVRLANRTGGLEVLLNQEVLSFTE
QSWMDLKGMFLSVAAGDRVSIMLASGAGLEVSVQGPFLSVSVLLPEKFLTHTHGLLGTLN
NDPTDDFTLHSGRVLPPGTSPQELFLFGANWTVHNASSLLTYDSWFLVHNFLYQPKHDPT
FEPLFPSETTLNPSLAQEAAKLCGDDHFCNFDVAATGSLSTGTATRVAHQLHQRRMQSLQ
PVVSCGWLAPPPNGQKEGNRYLAGSTIYFHCDNGYSLAGAETSTCQADGTWSSPTPKCQP
GRSYAVLLGIIFGGLAVVAAVALVYVLLRRRKGNTHVWGAQP
>sp|Q9UHB9|SRP68_HUMAN Signal recognition particle subunit SRP68 OS=Homo sapiens OX=9606 GN=SRP68 PE=1 SV=2
MAAEKQVPGGGGGGGSGGGGGSGGGGSGGGRGAGGEENKENERPSAGSKANKEFGDSLSL
EILQIIKESQQQHGLRHGDFQRYRGYCSRRQRRLRKTLNFKMGNRHKFTGKKVTEELLTD
NRYLLLVLMDAERAWSYAMQLKQEANTEPRKRFHLLSRLRKAVKHAEELERLCESNRVDA
KTKLEAQAYTAYLSGMLRFEHQEWKAAIEAFNKCKTIYEKLASAFTEEQAVLYNQRVEEI
SPNIRYCAYNIGDQSAINELMQMRLRSGGTEGLLAEKLEALITQTRAKQAATMSEVEWRG
RTVPVKIDKVRIFLLGLADNEAAIVQAESEETKERLFESMLSECRDAIQVVREELKPDQK
QRDYILEGEPGKVSNLQYLHSYLTYIKLSTAIKRNENMAKGLQRALLQQQPEDDSKRSPR
PQDLIRLYDIILQNLVELLQLPGLEEDKAFQKEIGLKTLVFKAYRCFFIAQSYVLVKKWS
EALVLYDRVLKYANEVNSDAGAFKNSLKDLPDVQELITQVRSEKCSLQAAAILDANDAHQ
TETSSSQVKDNKPLVERFETFCLDPSLVTKQANLVHFPPGFQPIPCKPLFFDLALNHVAF
PPLEDKLEQKTKSGLTGYIKGIFGFRS
>sp|Q9UHG3|PCYOX_HUMAN Prenylcysteine oxidase 1 OS=Homo sapiens OX=9606 GN=PCYOX1 PE=1 SV=3
MGRVVAELVSSLLGLWLLLCSCGCPEGAELRAPPDKIAIIGAGIGGTSAAYYLRQKFGKD
VKIDLFEREEVGGRLATMMVQGQEYEAGGSVIHPLNLHMKRFVKDLGLSAVQASGGLLGI
YNGETLVFEESNWFIINVIKLVWRYGFQSLRMHMWVEDVLDKFMRIYRYQSHDYAFSSVE
KLLHALGGDDFLGMLNRTLLETLQKAGFSEKFLNEMIAPVMRVNYGQSTDINAFVGAVSL
SCSDSGLWAVEGGNKLVCSGLLQASKSNLISGSVMYIEEKTKTKYTGNPTKMYEVVYQIG
TETRSDFYDIVLVATPLNRKMSNITFLNFDPPIEEFHQYYQHIVTTLVKGELNTSIFSSR
PIDKFGLNTVLTTDNSDLFINSIGIVPSVREKEDPEPSTDGTYVWKIFSQETLTKAQILK
LFLSYDYAVKKPWLAYPHYKPPEKCPSIILHDRLYYLNGIECAASAMEMSAIAAHNAALL
AYHRWNGHTDMIDQDGLYEKLKTEL
>sp|Q9UHN6|CEIP2_HUMAN Inactive cell surface hyaluronidase CEMIP2 OS=Homo sapiens OX=9606 GN=CEMIP2 PE=1 SV=1
MYATDSRGHSPAFLQPQNGNSRHPSGYVPGKVVPLRPPPPPKSQASAKFTSIRREDRATF
AFSPEEQQAQRESQKQKRHKNTFICFAITSFSFFIALAIILGISSKYAPDENCPDQNPRL
RNWDPGQDSAKQVVIKEGDMLRLTSDATVHSIVIQDGGLLVFGDNKDGSRNITLRTHYIL
IQDGGALHIGAEKCRYKSKATITLYGKSDEGESMPTFGKKFIGVEAGGTLELHGARKASW
TLLARTLNSSGLPFGSYTFEKDFSRGLNVRVIDQDTAKILESERFDTHEYRNESRRLQEF
LRFQDPGRIVAIAVGDSAAKSLLQGTIQMIQERLGSELIQGLGYRQAWALVGVIDGGSTS
CNESVRNYENHSSGGKALAQREFYTVDGQKFSVTAYSEWIEGVSLSGFRVEVVDGVKLNL

LDDVSSWKPGDQIVVASTDYSMYQAEEFTLLPCSECSHFQVKVKETPQFLHMGEIIDGVD
MRAEVGILTRNIVIQGEVEDSCYAENQCQFFDYDTFGGHIMIMKNFTSVHLSYVELKHMG
QQQMGRYPVHFHLCGDVDYKGGYRHATFVDGLSIHHSFSRCITVHGTNGLLIKDTIGFDT
LGHCFFLEDGIEQRNTLFHNLGLLTKPGTLLPTDRNNSMCTTMRDKVFGNYIPVPATDCM
AVSTFWIAHPNNNLINNAAAGSQDAGIWYLFHKEPTGESSGLQLLAKPELTPLGIFYNNR
VHSNFKAGLFIDKGVKTTNSSAADPREYLCLDNSARFRPHQDANPEKPRVAALIDRLIAF
KNNDNGAWVRGGDIIVQNSAFADNGIGLTFASDGSFPSDEGSSQEVSESLFVGESRNYGF
QGGQNKYVGTGGIDQKPRTLPRNRTFPIRGFQIYDGPIHLTRSTFKKYVPTPDRYSSAIG
FLMKNSWQITPRNNISLVKFGPHVSLNVFFGKPGPWFEDCEMDGDKNSIFHDIDGSVTGY
KDAYVGRMDNYLIRHPSCVNVSKWNAVICSGTYAQVYVQTWSTQNLSMTITRDEYPSNPM
VLRGINQKAAFPQYQPVVMLEKGYTIHWNGPAPRTTFLYLVNFNKNDWIRVGLCYPSNTS
FQVTFGYLQRQNGSLSKIEEYEPVHSLEELQRKQSERKFYFDSSTGLLFLYLKAKSHRHG
HSYCSSQGCERVKIQAATDSKDISNCMAKAYPQYYRKPSVVKRMPAMLTGLCQGCGTRQV
VFTSDPHKSYLPVQFQSPDKAETQRGDPSVISVNGTDFTFRSAGVLLLVVDPCSVPFRLT
EKTVFPLADVSRIEEYLKTGIPPRSIVLLSTRGEIKQLNISHLLVPLGLAKPAHLYDKGS
TIFLGFSGNFKPSWTKLFTSPAGQGLGVLEQFIPLQLDEYGCPRATTVRRRDLELLKQAS
KAH
>sp|Q9UHY7|ENOPH_HUMAN Enolase-phosphatase E1 OS=Homo sapiens OX=9606
GN=ENOPH1 PE=1 SV=1
MVVLSVPAEVTVILLDIEGTTTPIAFVKDILFPYIEENVKEYLQTHWEEEECQQDVSLLR
KQAEEDAHLDGAVPIPAASGNGVDDLQQMIQAVVDNVCWQMSLDRKTTALKQLQGHMWRA
AFTAGRMKAEFFADVVPAVRKWREAGMKVYIYSSGSVEAQKLLFGHSTEGDILELVDGHF
DTKIGHKVESESYRKIADSIGCSTNNILFLTDVTREASAAEEADVHVAVVVRPGNAGLTD
DEKTYYSLITSFSELYLPSST
>sp|Q9UI40|NCKX2_HUMAN Sodium/potassium/calcium exchanger 2 OS=Homo sapiens
OX=9606 GN=SLC24A2 PE=1 SV=1
MDLQQSTTITSLEKWCLDESLSGCRRHYSVKKKLKLIRVLGLFMGLVAISTVSFSISAFS
ETDTQSTGEASVVSGPRVAQGYHQRTLLDLNDKILDYTPQPPLSKEGESENSTDHAQGDY
PKDIFSLEERRKGAIILHVIGMIYMFIALAIVCDEFFVPSLTVITEKLGISDDVAGATFM
AAGGSAPELFTSLIGVFIAHSNVGIGTIVGSAVFNILFVIGMCALFSREILNLTWWPLFR
DVSFYIVDLIMLIIFFLDNVIMWWESLLLLTAYFCYVVFMKFNVQVEKWVKQMINRNKVV
KVTAPEAQAKPSAARDKDEPTLPAKPRLQRGGSSASLHNSLMRNSIFQLMIHTLDPLAEE
LGSYGKLKYYDTMTEEGRFREKASILHKIAKKKCHVDENERQNGAANHVEKIELPNSTST
DVEMTPSSDASEPVQNGNLSHNIEGAEAQTADEEEDQPLSLAWPSETRKQVTFLIVFPIV
FPLWITLPDVRKPSSRKFFPITFFGSITWIAVFSYLMVWWAHQVGETIGISEEIMGLTIL
AAGTSIPDLITSVIVARKGLGDMAVSSSVGSNIFDITVGLPLPWLLYTVIHRFQPVAVSS
NGLFCAIVLLFIMLLFVILSIALCKWRMNKILGFIMFGLYFVFLVVSVLLEDRILTCPVS
I
>sp|Q9UIA9|XPO7_HUMAN Exportin-7 OS=Homo sapiens OX=9606 GN=XPO7 PE=1 SV=3
MADHVQSLAQLENLCKQLYETTDTTTRLQAEKALVEFTNSPDCLSKCQLLLERGSSSYSQ
LLAATCLTKLVSRTNNPLPLEQRIDIRNYVLNYLATRPKLATFVTQALIQLYARITKLGW
FDCQKDDYVFRNAITDVTRFLQDSVEYCIIGVTILSQLTNEINQADTTHPLTKHRKIASS
FRDSSLFDIFTLSCNLLKQASGKNLNLNDESQHGLLMQLLKLTHNCLNFDFIGTSTDESS
DDLCTVQIPTSWRSAFLDSSTLQLFFDLYHSIPPSFSPLVLSCLVQIASVRRSLFNNAER

AKFLSHLVDGVKRILENPQSLSDPNNYHEFCRLLARLKSNYQLGELVKVENYPEVIRLIA
NFTVTSLQHWEFAPNSVHYLLSLWQRLAASVPYVKATEPHMLETYTPEVTKAYITSRLES
VHIILRDGLEDPLEDTGLVQQQLDQLSTIGRCEYEKTCALLVQLFDQSAQSYQELLQSAS
ASPMDIAVQEGRLTWLVYIIGAVIGGRVSFASTDEQDAMDGELVCRVLQLMNLTDSRLAQ
AGNEKLELAMLSFFEQFRKIYIGDQVQKSSKLYRRLSEVLGLNDETMVLSVFIGKIITNL
KYWGRCEPITSKTLQLLNDLSIGYSSVRKLVKLSAVQFMLNNHTSEHFSFLGINNQSNLT
DMRCRTTFYTALGRLLMVDLGEDEDQYEQFMLPLTAAFEAVAQMFSTNSFNEQEAKRTLV
GLVRDLRGIAFAFNAKTSFMMLFEWIYPSYMPILQRAIELWYHDPACTTPVLKLMAELVH
NRSQRLQFDVSSPNGILLFRETSKMITMYGNRILTLGEVPKDQVYALKLKGISICFSMLK
AALSGSYVNFGVFRLYGDDALDNALQTFIKLLLSIPHSDLLDYPKLSQSYYSLLEVLTQD
HMNFIASLEPHVIMYILSSISEGLTALDTMVCTGCCSCLDHIVTYLFKQLSRSTKKRTTP
LNQESDRFLHIMQQHPEMIQQMLSTVLNIIIFEDCRNQWSMSRPLLGLILLNEKYFSDLR
NSIVNSQPPEKQQAMHLCFENLMEGIERNLLTKNRDRFTQNLSAFRREVNDSMKNSTYGV
NSNDMMS
>sp|Q9UIG0|BAZ1B_HUMAN Tyrosine-protein kinase BAZ1B OS=Homo sapiens OX=9606
GN=BAZ1B PE=1 SV=2
MAPLLGRKPFPLVKPLPGEEPLFTIPHTQEAFRTREEYEARLERYSERIWTCKSTGSSQL
THKEAWEEEQEVAELLKEEFPAWYEKLVLEMVHHNTASLEKLVDTAWLEIMTKYAVGEEC
DFEVGKEKMLKVKIVKIHPLEKVDEEATEKKSDGACDSPSSDKENSSQIAQDHQKKETVV
KEDEGRRESINDRARRSPRKLPTSLKKGERKWAPPKFLPHKYDVKLQNEDKIISNVPADS
LIRTERPPNKEIVRYFIRHNALRAGTGENAPWVVEDELVKKYSLPSKFSDFLLDPYKYMT
LNPSTKRKNTGSPDRKPSKKSKTDNSSLSSPLNPKLWCHVHLKKSLSGSPLKVKNSKNSK
SPEEHLEEMMKMMSPNKLHTNFHIPKKGPPAKKPGKHSDKPLKAKGRSKGILNGQKSTGN
SKSPKKGLKTPKTKMKQMTLLDMAKGTQKMTRAPRNSGGTPRTSSKPHKHLPPAALHLIA
YYKENKDREDKRSALSCVISKTARLLSSEDRARLPEELRSLVQKRYELLEHKKRWASMSE
EQRKEYLKKKREELKKKLKEKAKERREKEMLERLEKQKRYEDQELTGKNLPAFRLVDTPE
GLPNTLFGDVAMVVEFLSCYSGLLLPDAQYPITAVSLMEALSADKGGFLYLNRVLVILLQ
TLLQDEIAEDYGELGMKLSEIPLTLHSVSELVRLCLRRSDVQEESEGSDTDDNKDSAAFE
DNEVQDEFLEKLETSEFFELTSEEKLQILTALCHRILMTYSVQDHMETRQQMSAELWKER
LAVLKEENDKKRAEKQKRKEMEAKNKENGKVENGLGKTDRKKEIVKFEPQVDTEAEDMIS
AVKSRRLLAIQAKKEREIQEREMKVKLERQAEEERIRKHKAAAEKAFQEGIAKAKLVMRR
TPIGTDRNHNRYWLFSDEVPGLFIEKGWVHDSIDYRFNHHCKDHTVSGDEDYCPRSKKAN
LGKNASMNTQHGTATEVAVETTTPKQGQNLWFLCDSQKELDELLNCLHPQGIRESQLKER
LEKRYQDIIHSIHLARKPNLGLKSCDGNQELLNFLRSDLIEVATRLQKGGLGYVEETSEF
EARVISLEKLKDFGECVIALQASVIKKFLQGFMAPKQKRRKLQSEDSAKTEEVDEEKKMV
EEAKVASALEKWKTAIREAQTFSRMHVLLGMLDACIKWDMSAENARCKVCRKKGEDDKLI
LCDECNKAFHLFCLRPALYEVPDGEWQCPACQPATARRNSRGRNYTEESASEDSEDDESD
EEEEEEEEEEEEDYEVAGLRLRPRKTIRGKHSVIPPAARSGRRPGKKPHSTRRSQPKAP
PVDDAEVDELVLQTKRSSRRQSLELQKCEEILHKIVKYRFSWPFREPVTRDEAEDYYDVI
THPMDFQTVQNKCSCGSYRSVQEFLTDMKQVFTNAEVYNCRGSHVLSCMVKTEQCLVALL
HKHLPGHPYVRRKRKKFPDRLAEDEGDSEPEAVGQSRGRRQKK
>sp|Q9UIW2|PLXA1_HUMAN Plexin-A1 OS=Homo sapiens OX=9606 GN=PLXNA1 PE=1 SV=3
MPLPPRSLQVLLLLLLLLLLLPGMWAEAGLPRAGGGSQPPFRTFSASDWGLTHLVVHEQT
GEVYVGAVNRIYKLSGNLTLLRAHVTGPVEDNEKCYPPPSVQSCPHGLGSTDNVNKLLLL

DYAANRLLACGSASQGICQFLRLDDLFKLGEPHHRKEHYLSSVQEAGSMAGVLIAGPPGQ
GQAKLFVGTPIDGKSEYFPTLSSRRLMANEEDADMFGFVYQDEFVSSQLKIPSDTLSKFP
AFDIYYVYSFRSEQFVYYLTLQLDTQLTSPDAAGEHFFTSKIVRLCVDDPKFYSYVEFPI
GCEQAGVEYRLVQDAYLSRPGRALAHQLGLAEDEDVLFTVFAQGQKNRVKPPKESALCLF
TLRAIKEKIKERIQSCYRGEGKLSLPWLLNKELGCINSPLQIDDDFCGQDFNQPLGGTVT
IEGTPLFVDKDDGLTAVAAYDYRGRTVVFAGTRSGRIRKILVDLSNPGGRPALAYESVVA
QEGSPILRDLVLSPNHQYLYAMTEKQVTRVPVESCVQYTSCELCLGSRDPHCGWCVLHSI
CSRRDACERADEPQRFAADLLQCVQLTVQPRNVSVTMSQVPLVLQAWNVPDLSAGVNCSF
EDFTESESVLEDGRIHCRSPSAREVAPITRGQGDQRVVKLYLKSKETGKKFASVDFVFYN
CSVHQSCLSCVNGSFPCHWCKYRHVCTHNVADCAFLEGRVNVSEDCPQILPSTQIYVPVG
VVKPITLAARNLPQPQSGQRGYECLFHIPGSPARVTALRFNSSSLQCQNSSYSYEGNDVS
DLPVNLSVVWNGNFVIDNPQNIQAHLYKCPALRESCGLCLKADPRFECGWCVAERRCSLR
HHCAADTPASWMHARHGSSRCTDPKILKLSPETGPRQGGTRLTITGENLGLRFEDVRLGV
RVGKVLCSPVESEYISAEQIVCEIGDASSVRAHDALVEVCVRDCSPHYRALSPKRFTFVT
PTFYRVSPSRGPLSGGTWIGIEGSHLNAGSDVAVSVGGRPCSFSWRNSREIRCLTPPGQS
PGSAPIIININRAQLTNPEVKYNYTEDPTILRIDPEWSINSGGTLLTVTGTNLATVREPR
IRAKYGGIERENGCLVYNDTTMVCRAPSVANPVRSPPELGERPDELGFVMDNVRSLLVLN
STSFLYYPDPVLEPLSPTGLLELKPSSPLILKGRNLLPPAPGNSRLNYTVLIGSTPCTLT
VSETQLLCEAPNLTGQHKVTVRAGGFEFSPGTLQVYSDSLLTLPAIVGIGGGGGLLLLVI
VAVLIAYKRKSRDADRTLKRLQLQMDNLESRVALECKEAFAELQTDIHELTNDLDGAGIP
FLDYRTYAMRVLFPGIEDHPVLKEMEVQANVEKSLTLFGQLLTKKHFLLTFIRTLEAQRS
FSMRDRGNVASLIMTALQGEMEYATGVLKQLLSDLIEKNLESKNHPKLLLRRTESVAEKM
LTNWFTFLLYKFLKECAGEPLFMLYCAIKQQMEKGPIDAITGEARYSLSEDKLIRQQIDY
KTLTLNCVNPENENAPEVPVKGLDCDTVTQAKEKLLDAAYKGVPYSQRPKAADMDLEWRQ
GRMARIILQDEDVTTKIDNDWKRLNTLAHYQVTDGSSVALVPKQTSAYNISNSSTFTKSL
SRYESMLRTASSPDSLRSRTPMITPDLESGTKLWHLVKNHDHLDQREGDRGSKMVSEIYL
TRLLATKGTLQKFVDDLFETIFSTAHRGSALPLAIKYMFDFLDEQADKHQIHDADVRHTW
KSNCLPLRFWVNVIKNPQFVFDIHKNSITDACLSVVAQTFMDSCSTSEHKLGKDSPSNKL
LYAKDIPNYKSWVERYYADIAKMPAISDQDMSAYLAEQSRLHLSQFNSMSALHEIYSYIT
KYKDEILAALEKDEQARRQRLRSKLEQVVDTMALSS
>sp|Q9UJ70|NAGK_HUMAN N-acetyl-D-glucosamine kinase OS=Homo sapiens OX=9606
GN=NAGK PE=1 SV=4
MAAIYGGVEGGGTRSEVLLVSEDGKILAEADGLSTNHWLIGTDKCVERINEMVNRAKRKA
GVDPLVPLRSLGLSLSGGDQEDAGRILIEELRDRFPYLSESYLITTDAAGSIATATPDGG
VVLISGTGSNCRLINPDGSESGCGGWGHMMGDEGSAYWIAHQAVKIVFDSIDNLEAAPHD
IGYVKQAMFHYFQVPDRLGILTHLYRDFDKCRFAGFCRKIAEGAQQGDPLSRYIFRKAGE
MLGRHIVAVLPEIDPVLFQGKIGLPILCVGSVWKSWELLKEGFLLALTQGREIQAQNFFS
SFTLMKLRHSSALGGASLGARHIGHLLPMDYSANAIAFYSYTFS
>sp|Q9UKE5|TNIK_HUMAN TRAF2 and NCK-interacting protein kinase OS=Homo sapiens
OX=9606 GN=TNIK PE=1 SV=1
MASDSPARSLDEIDLSALRDPAGIFELVELVGNGTYGQVYKGRHVKTGQLAAIKVMDVTG
DEEEEIKQEINMLKKYSHHRNIATYYGAFIKKNPPGMDDQLWLVMEFCGAGSVTDLIKNT
KGNTLKEEWIAYICREILRGLSHLHQHKVIHRDIKGQNVLLTENAEVKLVDFGVSAQLDR
TVGRRNTFIGTPYWMAPEVIACDENPDATYDFKSDLWSLGITAIEMAEGAPPLCDMHPMR

ALFLIPRNPAPRLKSKKWSKKFQSFIESCLVKNHSQRPATEQLMKHPFIRDQPNERQVRI
QLKDHIDRTKKKRGEKDETEYEYSGSEEEEEENDSGEPSSILNLPGESTLRRDFLRLQLA
NKERSEALRRQQLEQQQRENEEHKRQLLAERQKRIEEQKEQRRRLEEQQRREKELRKQQE
REQRRHYEEQMRREEERRRAEHEQEYIRRQLEEEQRQLEILQQQLLHEQALLLEYKRKQL
EEQRQAERLQRQLKQERDYLVSLQHQRQEQRPVEKKPLYHYKEGMSPSEKPAWAKEVEER
SRLNRQSSPAMPHKVANRISDPNLPPRSESFSISGVQPARTPPMLRPVDPQIPHLVAVKS
QGPALTASQSVHEQPTKGLSGFQEALNVTSHRVEMPRQNSDPTSENPPLPTRIEKFDRSS
WLRQEEDIPPKVPQRTTSISPALARKNSPGNGSALGPRLGSQPIRASNPDLRRTEPILES
PLQRTSSGSSSSSSTPSSQPSSQGGSQPGSQAGSSERTRVRANSKSEGSPVLPHEPAKVK
PEESRDITRPSRPASYKKAIDEDLTALAKELRELRIEETNRPMKKVTDYSSSSEESESSE
EEEEDGESETHDGTVAVSDIPRLIPTGAPGSNEQYNVGMVGTHGLETSHADSFSGSISRE
GTLMIRETSGEKKRSGHSDSNGFAGHINLPDLVQQSHSPAGTPTEGLGRVSTHSQEMDSG
TEYGMGSSTKASFTPFVDPRVYQTSPTDEDEEDEESSAAALFTSELLRQEQAKLNEARKI
SVVNVNPTNIRPHSDTPEIRKYKKRFNSEILCAALWGVNLLVGTENGLMLLDRSGQGKVY
NLINRRRFQQMDVLEGLNVLVTISGKKNKLRVYYLSWLRNRILHNDPEVEKKQGWITVGD
LEGCIHYKVVKYERIKFLVIALKNAVEIYAWAPKPYHKFMAFKSFADLQHKPLLVDLTVE
EGQRLKVIFGSHTGFHVIDVDSGNSYDIYIPSHIQGNITPHAIVILPKTDGMEMLVCYED
EGVYVNTYGRITKDVVLQWGEMPTSVAYIHSNQIMGWGEKAIEIRSVETGHLDGVFMHKR
AQRLKFLCERNDKVFFASVRSGGSSQVFFMTLNRNSMMNW
>sp|Q9UKJ3|GPTC8_HUMAN G patch domain-containing protein 8 OS=Homo sapiens OX=9606
GN=GPATCH8 PE=1 SV=2
MADRFSRFNEDRDFQGNHFDQYEEGHLEIEQASLDKPIESDNIGHRLLQKHGWKLGQGLG
KSLQGRTDPIPIVVKYDVMGMGRMEMELDYAEDATERRRVLEVEKEDTEELRQKYKDYVD
KEKAIAKALEDLRANFYCELCDKQYQKHQEFDNHINSYDHAHKQRLKDLKQREFARNVSS
RSRKDEKKQEKALRRLHELAEQRKQAECAPGSGPMFKPTTVAVDEEGGEDDKDESATNSG
TGATASCGLGSEFSTDKGGPFTAVQITNTTGLAQAPGLASQGISFGIKNNLGTPLQKLGV
SFSFAKKAPVKLESIASVFKDHAEEGTSEDGTKPDEKSSDQGLQKVGDSDGSSNLDGKKE
DEDPQDGGSLASTLSKLKRMKREEGAGATEPEYYHYIPPAHCKVKPNFPFLLFMRASEQM
DGDNTTHPKNAPESKKGSSPKPKSCIKAAASQGAEKTVSEVSEQPKETSMTEPSEPGSKA
EAKKALGGDVSDQSLESHSQKVSETQMCESNSSKETSLATPAGKESQEGPKHPTGPFFPV
LSKDESTALQWPSELLIFTKAEPSISYSCNPLYFDFKLSRNKDARTKGTEKPKDIGSSSK
DHLQGLDPGEPNKSKEVGGEKIVRSSGGRMDAPASGSACSGLNKQEPGGSHGSETEDTGR
SLPSKKERSGKSHRHKKKKKHKKSSKHKRKHKADTEEKSSKAESGEKSKKRKKRKRKKNK
SSAPADSERGPKPEPPGSGSPAPPRRRRRAQDDSQRRSLPAEEGSSGKKDEGGGGSSSQD
HGGRKHKGELPPSSCQRRAGTKRSSRSSHRSQPSSGDEDSDDASSHRLHQKSPSQYSEEE
EEEDSGSEHSRSRSRSGRRHSSHRSSRRSYSSSSDASSDQSCYSRQRSYSDDSYSDYSDR
SRRHSKRSHDSDDSDYASSKHRSKRHKYSSSDDDYSLSCSQSRSRSRSHTRERSRSRGRS
RSSSCSRSRSKRRSRSTTAHSWQRSRSYSRDRSRSTRSPSQRSGSRKRSWGHESPEERHS
GRRDFIRSKIYRSQSPHYFRSGRGEGPGKKDDGRGDDSKATGPPSQNSNIGTGRGSEGDC
SPEDKNSVTAKLLLEKIQSRKVERKPSVSEEVQATPNKAGPKLKDPPQGYFGPKLPPSLG
NKPVLPLIGKLPATRKPNKKCEESGLERGEEQEQSETEEGPPGSSDALFGHQFPSEETTG
PLLDPPPEESKSGEATADHPVAPLGTPAHSDCYPGDPTISHNYLPDPSDGDTLESLDSSS
QPGPVESSLLPIAPDLEHFPSYAPPSGDPSIESTDGAEDASLAPLESQPITFTPEEMEKY
SKLQQAAQQHIQQQLLAKQVKAFPASAALAPATPALQPIHIQQPATASATSITTVQHAIL

QHHAAAAAAAIGIHPHPHPQPLAQVHHIPQPHLTPISLSHLTHSIIPGHPATFLASHPIH
IIPASAIHPGPFTFHPVPHAALYPTLLAPRPAAAAATALHLHPLLHPIFSGQDLQHPPSH
GT
>sp|Q9UKS6|PACN3_HUMAN Protein kinase C and casein kinase substrate in neurons protein 3 OS=Homo sapiens OX=9606 GN=PACSIN3 PE=1 SV=2
MAPEEDAGGEALGGSFWEAGNYRRTVQRVEDGHRLCGDLVSCFQERARIEKAYAQQLADW
ARKWRGTVEKGPQYGTLEKAWHAFFTAAERLSALHLEVREKLQGQDSERVRAWQRGAFHR
PVLGGFRESRAAEDGFRKAQKPWLKRLKEVEASKKSYHAARKDEKTAQTRESHAKADSAV
SQEQLRKLQERVERCAKEAEKTKAQYEQTLAELHRYTPRYMEDMEQAFETCQAAERQRLL
FFKDMLLTLHQHLDLSSSEKFHELHRDLHQGIEAASDEEDLRWWRSTHGPGMAMNWPQFE
EWSLDTQRTISRKEKGGRSPDEVTLTSIVPTRDGTAPPPQSPGSPGTGQDEEWSDEESPR
KAATGVRVRALYDYAGQEADELSFRAGEELLKMSEEDEQGWCQGQLQSGRIGLYPANYVE
CVGA
>sp|Q9UKX2|MYH2_HUMAN Myosin-2 OS=Homo sapiens OX=9606 GN=MYH2 PE=1 SV=1
MSSDSELAVFGEAAPFLRKSERERIEAQNRPFDAKTSVFVAEPKESFVKGTIQSREGGKV
TVKTEGGATLTVKDDQVFPMNPPKYDKIEDMAMMTHLHEPAVLYNLKERYAAWMIYTYSG
LFCVTVNPYKWLPVYKPEVVTAYRGKKRQEAPPHIFSISDNAYQFMLTDRENQSILITGE
SGAGKTVNTKRVIQYFATIAVTGEKKKEEITSGKIQGTLEDQIISANPLLEAFGNAKTVR
NDNSSRFGKFIRIHFGTTGKLASADIETYLLEKSRVVFQLKAERSYHIFYQITSNKKPEL
IEMLLITTNPYDYPFVSQGEISVASIDDQEELMATDSAIDILGFTNEEKVSIYKLTGAVM
HYGNLKFKQKQREEQAEPDGTEVADKAAYLQSLNSADLLKALCYPRVKVGNEYVTKGQTV
EQVSNAVGALAKAVYEKMFLWMVARINQQLDTKQPRQYFIGVLDIAGFEIFDFNSLEQLC
INFTNEKLQQFFNHHMFVLEQEEYKKEGIEWTFIDFGMDLAACIELIEKPMGIFSILEEE
CMFPKATDTSFKNKLYDQHLGKSANFQKPKVVKGKAEAHFALIHYAGVVDYNITGWLEKN
KDPLNETVVGLYQKSAMKTLAQLFSGAQTAEGEGAGGGAKKGGKKKGSSFQTVSALFREN
LNKLMTNLRSTHPHFVRCIIPNETKTPGAMEHELVLHQLRCNGVLEGIRICRKGFPSRIL
YADFKQRYKVLNASAIPEGQFIDSKKASEKLLASIDIDHTQYKFGHTKVFFKAGLLGLLE
EMRDDKLAQLITRTQARCRGFLARVEYQRMVERREAIFCIQYNIRSFMNVKHWPWMKLFF
KIKPLLKSAETEKEMATMKEEFQKIKDELAKSEAKRKELEEKMVTLLKEKNDLQLQVQAE
AEGLADAEERCDQLIKTKIQLEAKIKEVTERAEDEEEINAELTAKKRKLEDECSELKKDI
DDLELTLAKVEKEKHATENKVKNLTEEMAGLDETIAKLTKEKKALQEAHQQTLDDLQAEE
DKVNTLTKAKIKLEQQVDDLEGSLEQEKKLRMDLERAKRKLEGDLKLAQESIMDIENEKQ
QLDEKLKKKEFEISNLQSKIEDEQALGIQLQKKIKELQARIEELEEEIEAERASRAKAEK
QRSDLSRELEEISERLEEAGGATSAQIEMNKKREAEFQKMRRDLEEATLQHEATAATLRK
KHADSVAELGEQIDNLQRVKQKLEKEKSEMKMEIDDLASNVETVSKAKGNLEKMCRTLED
QLSELKSKEEEQQRLINDLTAQRGRLQTESGEFSRQLDEKEALVSQLSRGKQAFTQQIEE
LKRQLEEEIKAKNALAHALQSSRHDCDLLREQYEEEQESKAELQRALSKANTEVAQWRTK
YETDAIQRTEELEEAKKKLAQRLQAAEEHVEAVNAKCASLEKTKQRLQNEVEDLMLDVER
TNAACAALDKKQRNFDKILAEWKQKCEETHAELEASQKEARSLGTELFKIKNAYEESLDQ
LETLKRENKNLQQEISDLTEQIAEGGKRIHELEKIKKQVEQEKCELQAALEEAEASLEHE
EGKILRIQLELNQVKSEVDRKIAEKDEEIDQLKRNHIRIVESMQSTLDAEIRSRNDAIRL
KKKMEGDLNEMEIQLNHANRMAAEALRNYRNTQGILKDTQIHLDDALRSQEDLKEQLAMV
ERRANLLQAEIEELRATLEQTERSRKIAEQELLDASERVQLLHTQNTSLINTKKKLETDI
SQMQGEMEDILQEARNAEEKAKKAITDAAMMAEELKKEQDTSAHLERMKKNMEQTVKDLQ

LRLDEAEQLALKGGKKQIQKLEARVRELEGEVESEQKRNAEAVKGLRKHERRVKELTYQT
EEDRKNILRLQDLVDKLQAKVKSYKRQAEEAEEQSNTNLAKFRKLQHELEEAEERADIAE
SQVNKLRVKSREVHTKVISEE
>sp|Q9UL25|RAB21_HUMAN Ras-related protein Rab-21 OS=Homo sapiens OX=9606 GN=RAB21 PE=1 SV=3
MAAAGGGGGGAAAAGRAYSFKVVLLGEGCVGKTSLVLRYCENKFNDKHITTLQASFLTKK
LNIGGKRVNLAIWDTAGQERFHALGPIYYRDSNGAILVYDITDEDSFQKVKNWVKELRKM
LGNEICLCIVGNKIDLEKERHVSIQEAESYAESVGAKHYHTSAKQNKGIEELFLDLCKRM
IETAQVDERAKGNGSSQPGTARRGVQIIDDEPQAQTSGGGCCSSG
>sp|Q9ULX7|CAH14_HUMAN Carbonic anhydrase 14 OS=Homo sapiens OX=9606 GN=CA14 PE=1 SV=1
MLFSALLLEVIWILAADGGQHWTYEGPHGQDHWPASYPECGNNAQSPIDIQTDSVTFDPD
LPALQPHGYDQPGTEPLDLHNNGHTVQLSLPSTLYLGGLPRKYVAAQLHLHWGQKGSPGG
SEHQINSEATFAELHIVHYDSDSYDSLSEAAERPQGLAVLGILIEVGETKNIAYEHILSH
LHEVRHKDQKTSVPPFNLRELLPKQLGQYFRYNGSLTTPPCYQSVLWTVFYRRSQISMEQ
LEKLQGTLFSTEEEPSKLLVQNYRALQPLNQRMVFASFIQAGSSYTTGEMLSLGVGILVG
CLCLLLAVYFIARKIRKKRLENRKSVVFTSAQATTEA
>sp|Q9UNF1|MAGD2_HUMAN Melanoma-associated antigen D2 OS=Homo sapiens OX=9606 GN=MAGED2 PE=1 SV=2
MSDTSESGAGLTRFQAEASEKDSSSMMQTLLTVTQNVEVPETPKASKALEVSEDVKVSKA
SGVSKATEVSKTPEAREAPATQASSTTQLTDTQVLAAENKSLAADTKKQNADPQAVTMPA
TETKKVSHVADTKVNTKAQETEAAPSQAPADEPEPESAAAQSQENQDTRPKVKAKKARKV
KHLDGEEDGSSDQSQASGTTGGRRVSKALMASMARRASRGPIAFWARRASRTRLAAWARR
ALLSLRSPKARRGKARRRAAKLQSSQEPEAPPPRDVALLQGRANDLVKYLLAKDQTKIPI
KRSDMLKDIIKEYTDVYPEIIERAGYSLEKVFGIQLKEIDKNDHLYILLSTLEPTDAGIL
GTTKDSPKLGLLMVLLSIIFMNGNRSSEAVIWEVLRKLGLRPGIHHSLFGDVKKLITDEF
VKQKYLDYARVPNSNPPEYEFFWGLRSYYETSKMKVLKFACKVQKKDPKEWAAQYREAME
ADLKAAAEAAAEAKARAEIRARMGIGLGSENAAGPCNWDEADIGPWAKARIQAGAEAKAK
AQESGSASTGASTSTNNSASASASTSGGFSAGASLTATLTFGLFAGLGGAGASTSGSSGA
CGFSYK
>sp|Q9UNH7|SNX6_HUMAN Sorting nexin-6 OS=Homo sapiens OX=9606 GN=SNX6 PE=1 SV=1
MMEGLDDGPDFLSEEDRGLKAINVDLQSDAALQVDISDALSERDKVKFTVHTKSSLPNFK
QNEFSVVRQHEEFIWLHDSFVENEDYAGYIIPPAPPRPDFDASREKLQKLGEGEGSMTKE
EFTKMKQELEAEYLAIFKKTVAMHEVFLCRVAAHPILRRDLNFHVFLEYNQDLSVRGKNK
KEKLEDFFKNMVKSADGVIVSGVKDVDDFFEHERTFLLEYHNRVKDASAKSDRMTRSHKS
AADDYNRIGSSLYALGTQDSTDICKFFLKVSELFDKTRKIEARVSADEDLKLSDLLKYYL
RESQAAKDLLYRRSRSLVDYENANKALDKARAKNKDVLQAETSQQLCCQKFEKISESAKQ
ELIDFKTRRVAAFRKNLVELAELELKHAKGNLQLLQNCLAVLNGDT
>sp|Q9UNM6|PSD13_HUMAN 26S proteasome non-ATPase regulatory subunit 13 OS=Homo sapiens OX=9606 GN=PSMD13 PE=1 SV=2
MKDVPGFLQQSQNSGPGQPAVWHRLEELYTKKLWHQLTLQVLDFVQDPCFAQGDGLIKLY
ENFISEFEHRVNPLSLVEIILHVVRQMTDPNVALTFLEKTREKVKSSDEAVILCKTAIGA
LKLNIGDLQVTKETIEDVEEMLNNLPGVTSVHSRFYDLSSKYYQTIGNHASYYKDALRFL
GCVDIKDLPVSEQQERAFTLGLAGLLGEGVFNFGELLMHPVLESLRNTDRQWLIDTLYAF

NSGNVERFQTLKTAWGQQPDLAANEAQLLRKIQLLCLMEMTFTRPANHRQLTFEEIAKSA
KITVNEVELLVMKALSVGLVKGSIDEVDKRVHMTWVQPRVLDLQQIKGMKDRLEFWCTDV
KSMEMLVEHQAHDILT
>sp|Q9UNS2|CSN3_HUMAN COP9 signalosome complex subunit 3 OS=Homo sapiens OX=9606 GN=COPS3 PE=1 SV=3
MASALEQFVNSVRQLSAQGQMTQLCELINKSGELLAKNLSHLDTVLGALDVQEHSLGVLA
VLFVKFSMPSVPDFETLFSQVQLFISTCNGEHIRYATDTFAGLCHQLTNALVERKQPLRG
IGILKQAIDKMQMNTNQLTSIHADLCQLCLLAKCFKPALPYLDVDMMDICKENGAYDAKH
FLCYYYYGGMIYTGLKNFERALYFYEQAITTPAMAVSHIMLESYKKYILVSLILLGKVQQ
LPKYTSQIVGRFIKPLSNAYHELAQVYSTNNPSELRNLVNKHSETFTRDNNMGLVKQCLS
SLYKKNIQRLTKTFLTLSLQDMASRVQLSGPQEAEKYVLHMIEDGEIFASINQKDGMVSF
HDNPEKYNNPAMLHNIDQEMLKCIELDERLKAMDQEITVNPQFVQKSMGSQEDDSGNKPS
SYS
>sp|Q9UNW1|MINP1_HUMAN Multiple inositol polyphosphate phosphatase 1 OS=Homo sapiens OX=9606 GN=MINPP1 PE=1 SV=1
MLRAPGCLLRTSVAPAAALAAALLSSLARCSLLEPRDPVASSLSPYFGTKTRYEDVNPVL
LSGPEAPWRDPELLEGTCTPVQLVALIRHGTRYPTVKQIRKLRQLHGLLQARGSRDGGAS
STGSRDLGAALADWPLWYADWMDGQLVEKGRQDMRQLALRLASLFPALFSRENYGRLRLI
TSSKHRCMDSSAAFLQGLWQHYHPGLPPPDVADMEFGPPTVNDKLMRFFDHCEKFLTEVE
KNATALYHVEAFKTGPEMQNILKKVAATLQVPVNDLNADLIQVAFFTCSFDLAIKGVKSP
WCDVFDIDDAKVLEYLNDLKQYWKRGYGYTINSRSSCTLFQDIFQHLDKAVEQKQRSQPI
SSPVILQFGHAETLLPLLSLMGYFKDKEPLTAYNYKKQMHRKFRSGLIVPYASNLIFVLY
HCENAKTPKEQFRVQMLLNEKVLPLAYSQETVSFYEDLKNHYKDILQSCQTSEECELARA
NSTSDEL
>sp|Q9UQD0|SCN8A_HUMAN Sodium channel protein type 8 subunit alpha OS=Homo sapiens OX=9606 GN=SCN8A PE=1 SV=1
MAARLLAPPGPDSFKPFTPESLANIERRIAESKLKKPPKADGSHREDDEDSKPKPNSDLE
AGKSLPFIYGDIPQGLVAVPLEDFDPYYLTQKTFVVLNRGKTLFRFSATPALYILSPFNL
IRRIAIKILIHSVFSMIIMCTILTNCVFMTFSNPPDWSKNVEYTFTGIYTFESLVKIIAR
GFCIDGFTFLRDPWNWLDFSVIMMAYITEFVNLGNVSALRTFRVLRALKTISVIPGLKTI
VGALIQSVKKLSDVMILTVFCLSVFALIGLQLFMGNLRNKCVVWPINFNESYLENGTKGF
DWEEYINNKTNFYTVPGMLEPLLCGNSSDAGQCPEGYQCMKAGRNPNYGYTSFDTFSWAF
LALFRLMTQDYWENLYQLTLRAAGKTYMIFFVLVIFVGSFYLVNLILAVVAMAYEEQNQA
TLEEAEQKEAEFKAMLEQLKKQQEEAQAAAMATSAGTVSEDAIEEEGEEGGGSPRSSSEI
SKLSSKSAKERRNRRKKRKQKELSEGEEKGDPEKVFKSESEDGMRRKAFRLPDNRIGRKF
SIMNQSLLSIPGSPFLSRHNSKSSIFSFRGPGRFRDPGSENEFADDEHSTVEESEGRRDS
LFIPIRARERRSSYSGYSGYSQGSRSSRIFPSLRRSVKRNSTVDCNGVVSLIGGPGSHIG
GRLLPEATTEVEIKKKGPGSLLVSMDQLASYGRKDRINSIMSVVTNTLVEELEESQRKCP
PCWYKFANTFLIWECHPYWIKLKEIVNLIVMDPFVDLAITICIVLNTLFMAMEHHPMTPQ
FEHVLAVGNLVFTGIFTAEMFLKLIAMDPYYYFQEGWNIFDGFIVSLSLMELSLADVEGL
SVLRSFRLLRVFKLAKSWPTLNMLIKIIGNSVGALGNLTLVLAIIVFIFAVVGMQLFGKS
YKECVCKINQDCELPRWHMHDFFHSFLIVFRVLCGEWIETMWDCMEVAGQAMCLIVFMMV
MVIGNLVVLNLFLALLLSSFSADNLAATDDDGEMNNLQISVIRIKKGVAWTKLKVHAFMQ
AHFKQREADEVKPLDELYEKKANCIANHTGADIHRNGDFQKNGNGTTSGIGSSVEKYIID

EDHMSFINNPNLTVRVPIAVGESDFENLNTEDVSSESDPEGSKDKLDDTSSSEGSTIDIK
PEVEEVPVEQPEEYLDPDACFTEGCVQRFKCCQVNIEEGLGKSWWILRKTCFLIVEHNWF
ETFIIFMILLSSGALAFEDIYIEQRKTIRTILEYADKVFTYIFILEMLLKWTAYGFVKFF
TNAWCWLDFLIVAVSLVSLIANALGYSELGAIKSLRTLRALRPLRALSRFEGMRVVVNAL
VGAIPSIMNVLLVCLIFWLIFSIMGVNLFAGKYHYCFNETSEIRFEIEDVNNKTECEKLM
EGNNTEIRWKNVKINFDNVGAGYLALLQVATFKGWMDIMYAAVDSRKPDEQPKYEDNIYM
YIYFVIFIIFGSFFTLNLFIGVIIDNFNQQKKKFGGQDIFMTEEQKKYYNAMKKLGSKKP
QKPIPRPLNKIQGIVFDFVTQQAFDIVIMMLICLNMVTMMVETDTQSKQMENILYWINLV
FVIFFTCECVLKMFALRHYYFTIGWNIFDFVVVILSIVGMFLADIIEKYFVSPTLFRVIR
LARIGRILRLIKGAKGIRTLLFALMMSLPALFNIGLLLFLVMFIFSIFGMSNFAYVKHEA
GIDDMFNFETFGNSMICLFQITTSAGWDGLLLPILNRPPDCSLDKEHPGSGFKGDCGNPS
VGIFFFVSYIIISFLIVVNMYIAIILENFSVATEESADPLSEDDFETFYEIWEKFDPDAT
QFIEYCKLADFADALEHPLRVPKPNTIELIAMDLPMVSGDRIHCLDILFAFTKRVLGDSG
ELDILRQQMEERFVASNPSKVSYEPITTTLRRKQEEVSAVVLQRAYRGHLARRGFICKKT
TSNKLENGGTHREKKESTPSTASLPSYDSVTKPEKEKQQRAEEGRRERAKRQKEVRESKC
>sp|Q9Y230|RUVB2_HUMAN RuvB-like 2 OS=Homo sapiens OX=9606 GN=RUVBL2 PE=1 SV=3
MATVTATTKVPEIRDVTRIERIGAHSHIRGLGLDDALEPRQASQGMVGQLAARRAAGVVL
EMIREGKIAGRAVLIAGQPGTGKTAIAMGMAQALGPDTPFTAIAGSEIFSLEMSKTEALT
QAFRRSIGVRIKEETEIIEGEVVEIQIDRPATGTGSKVGKLTLKTTEMETIYDLGTKMIE
SLTKDKVQAGDVITIDKATGKISKLGRSFTRARDYDAMGSQTKFVQCPDGELQKRKEVVH
TVSLHEIDVINSRTQGFLALFSGDTGEIKSEVREQINAKVAEWREEGKAEIIPGVLFIDE
VHMLDIESFSFLNRALESDMAPVLIMATNRGITRIRGTSYQSPHGIPIDLLDRLLIVSTT
PYSEKDTKQILRIRCEEEDVEMSEDAYTVLTRIGLETSLRYAIQLITAASLVCRKRKGTE
VQVDDIKRVYSLFLDESRSTQYMKEYQDAFLFNELKGETMDTS
>sp|Q9Y241|HIG1A_HUMAN HIG1 domain family member 1A, mitochondrial OS=Homo sapiens OX=9606 GN=HIGD1A PE=1 SV=1
MSTDTGVSLPSYEEDQGSKLIRKAKEAPFVPVGIAGFAAIVAYGLYKLKSRGNTKMSIHL
IHMRVAAQGFVVGAMTVGMGYSMYREFWAKPKP
>sp|Q9Y262|EIF3L_HUMAN Eukaryotic translation initiation factor 3 subunit L OS=Homo sapiens OX=9606 GN=EIF3L PE=1 SV=1
MSYPADDYESEAAYDPYAYPSDYDMHTGDPKQDLAYERQYEQQTYQVIPEVIKNFIQYFH
KTVSDLIDQKVYELQASRVSSDVIDQKVYEIQDIYENSWTKLTERFFKNTPWPEAEAIAP
QVGNDAVFLILYKELYYRHIYAKVSGGPSLEQRFESYYNYCNLFNYILNADGPAPLELPN
QWLWDIIDEFIYQFQSFSQYRCKTAKKSEEEIDFLRSNPKIWNVHSVLNVLHSLVDKSNI
NRQLEVYTSGGDPESVAGEYGRHSLYKMLGYFSLVGLLRLHSLLGDYYQAIKVLENIELN
KKSMYSRVPECQVTTYYYVGFAYLMMRRYQDAIRVFANILLYIQRTKSMFQRTTYKYEMI
NKQNEQMHALLAIALTMYPMRIDESIHLQLREKYGDKMLRMQKGDPQVYEELFSYSCPKF
LSPVVPNYDNVHPNYHKEPFLQQLKVFSDEVQQQAQLSTIRSFLKLYTTMPVAKLAGFLD
LTEQEFRIQLLVFKHKMKNLVWTSGISALDGEFQSASEVDFYIDKDMIHIADTKVARRYG
DFFIRQIHKFEELNRTLKKMGQRP
>sp|Q9Y277|VDAC3_HUMAN Voltage-dependent anion-selective channel protein 3 OS=Homo sapiens OX=9606 GN=VDAC3 PE=1 SV=1
MCNTPTYCDLGKAAKDVFNKGYGFGMVKIDLKTKSCSGVEFSTSGHAYTDTGKASGNLET
KYKVCNYGLTFTQKWNTDNTLGTEISWENKLAEGLKLTLDTIFVPNTGKKSGKLKASYKR

DCFSVGSNVDIDFSGPTIYGWAVLAFEGWLAGYQMSFDTAKSKLSQNNFALGYKAADFQL
HTHVNDGTEFGGSIYQKVNEKIETSINLAWTAGSNNTRFGIAAKYMLDCRTSLSAKVNNA
SLIGLGYTQTLRPGVKLTLSALIDGKNFSAGGHKVGLGFELEA
>sp|Q9Y281|COF2_HUMAN Cofilin-2 OS=Homo sapiens OX=9606 GN=CFL2 PE=1 SV=1
MASGVTVNDEVIKVFNDMKVRKSSTQEEIKKRKKAVLFCLSDDKRQIIVEEAKQILVGDI
GDTVEDPYTSFVKLLPLNDCRYALYDATYETKESKKEDLVFIFWAPESAPLKSKMIYASS
KDAIKKKFTGIKHEWQVNGLDDIKDRSTLGEKLGGNVVVSLEGKPL
>sp|Q9Y287|ITM2B_HUMAN Integral membrane protein 2B OS=Homo sapiens OX=9606 GN=ITM2B PE=1 SV=1
MVKVTFNSALAQKEAKKDEPKSGEEALIIPPDAVAVDCKDPDDVVPVGQRRAWCWCMCFG
LAFMLAGVILGGAYLYKYFALQPDDVYYCGIKYIKDDVILNEPSADAPAALYQTIEENIK
IFEEEEVEFISVPVPEFADSDPANIVHDFNKKLTAYLDLNLDKCYVIPLNTSIVMPPRNL
LELLINIKAGTYLPQSYLIHEHMVITDRIENIDHLGFFIYRLCHDKETYKLQRRETIKGI
QKREASNCFAIRHFENKFAVETLICS
>sp|Q9Y2A7|NCKP1_HUMAN Nck-associated protein 1 OS=Homo sapiens OX=9606 GN=NCKAP1 PE=1 SV=1
MSRSVLQPSQQKLAEKLTILNDRGVGMLTRLYNIKKACGDPKAKPSYLIDKNLESAVKFI
VRKFPAVETRNNNQQLAQLQKEKSEILKNLALYYFTFVDVMEFKDHVCELLNTIDVCQVF
FDITVNFDLTKNYLDLIITYTTLMILLSRIEERKAIIGLYNYAHEMTHGASDREYPRLGQ
MIVDYENPLKKMMEEFVPHSKSLSDALISLQMVYPRRNLSADQWRNAQLLSLISAPSTML
NPAQSDTMPCEYLSLDAMEKWIIFGFILCHGILNTDATALNLWKLALQSSSCLSLFRDEV
FHIHKAAEDLFVNIRGYNKRINDIRECKEAAVSHAGSMHRERRKFLRSALKELATVLSDQ
PGLLGPKALFVFMALSFARDEIIWLLRHADNMPKKSADDFIDKHIAELIFYMEELRAHVR
KYGPVMQRYYVQYLSGFDAVVLNELVQNLSVCPEDESIIMSSFVNTMTSLSVKQVEDGEV
FDFRGMRLDWFRLQAYTSVSKASLGLADHRELGKMMNTIIFHTKMVDSLVEMLVETSDLS
IFCFYSRAFEKMFQQCLELPSQSRYSIAFPLLCTHFMSCTHELCPEERHHIGDRSLSLCN
MFLDEMAKQARNLITDICTEQCTLSDQLLPKHCAKTISQAVNKKSKKQTGKKGEPEREKP
GVESMRKNRLVVTNLDKLHTALSELCFSINYVPNMVVWEHTFTPREYLTSHLEIRFTKSI
VGMTMYNQATQEIAKPSELLTSVRAYMTVLQSIENYVQIDITRVFNNVLLQQTQHLDSHG
EPTITSLYTNWYLETLLRQVSNGHIAYFPAMKAFVNLPTENELTFNAEEYSDISEMRSLS
ELLGPYGMKFLSESLMWHISSQVAELKKLVVENVDVLTQMRTSFDKPDQMAALFKRLSSV
DSVLKRMTIIGVILSFRSLAQEALRDVLSYHIPFLVSSIEDFKDHIPRETDMKVAMNVYE
LSSAAGLPCEIDPALVVALSSQKSENISPEEEYKIACLLMVFVAVSLPTLASNVMSQYSP
AIEGHCNNIHCLAKAINQIAAALFTIHKGSIEDRLKEFLALASSSLLKIGQETDKTTTRN
RESVYLLLDMIVQESPFLTMDLLESCFPYVLLRNAYHAVYKQSVTSSA
>sp|Q9Y2D5|PLAK2_HUMAN PALM2-AKAP2 fusion protein OS=Homo sapiens OX=9606 GN=PALM2AKAP2 PE=1 SV=4
MAEAELHKERLQAIAEKRKRQTEIEGKRQQLDEQILLLQHSKSKVLREKWLLQGIPAGTA
EEEEARRRQSEEDEFRVKQLEDNIQRLEQEIQTLESEESQISAKEQIILEKLKETEKSFK
DFQKGFSSTDGDAVNYISSQLPDLPILCSRTAEPSPGQDGTSRAAGVGWENVLLKEGESA
SNATETSGPDMTIKKPPQLSEDDIWLKSEGDNYSATLLEPAASSLSPDHKNMEIEVSVAE
CKSVPGITSTPHPMDHPSAFYSPPHNGLLTDHHESLDNDVAREIRYLDEVLEANCCDSAV
DGTYNGTSSPEPGAVVLVGGLSPPVHEATQPEPTERTASRQAPPHIELSNSSPDPMAEAE
RTNGHSPSQPRDALGDSLQVPVSPSSTTSSRCSSRDGEFTLTTLKKEAKFELRAFHEDKK

PSKLFEDDEHEKEQYCIRKVRPSEEMLELEKERRELIRSQAVKKNPGIAAKWWNPPQEKT
IEEQLDEEHLESHKKYKERKERRAQQEQLLLQKQLQQQQQQPPSQLCTAPASSHERASMI
DKAKEDIVTEQIDFSAARKQFQLMENSRQAVAKGQSTPRLFSIKPFYRPLGSVNSDKPLT
NPRPPSVGGPPEDSGASAAKGQKSPGALETPSAAGSQGNTASQGKEGPYSEPSKRGPLSK
LWAEDGEFTSARAVLTVVKDDDHGILDQFSRSVNVSLTQEELDSGLDELSVRSQDTTVLE
TLSNDFSMDNISDSGASNETTNALQENSLADFSLPQTPQTDNPSEGRGEGVSKSFSDHGF
YSPSSTLGDSPLVDDPLEYQAGLLVQNAIQQAIAEQVDKAVSKTSRDGAEQQGPEATVEE
AEAAAFGSEKPQSMFEPPQVSSPVQEKRDVLPKILPAEDRALRERGPPQPLPAVQPSGPI
NMEETRPEGSYFSKYSEAAELRSTASLLATQESDVMVGPFKLRSRKQRTLSMIEEEIRAA
QEREEELKRQRQVLQSTQSPRTKNAPSLPSRTCYKTAPGKIEKVKPPPSPTTEGPSLQPD
LAPEEAAGTQRPKNLMQTLMEDYETHKSKRRERMDDSSYTSKLLSCKVTSEVLEATRVNR
RKSALALRWEAGIYANQEEEDNE
>sp|Q9Y2H2|SAC2_HUMAN Phosphatidylinositide phosphatase SAC2 OS=Homo sapiens OX=9606 GN=INPP5F PE=1 SV=3
MELFQAKDHYILQQGERALWCSRRDGGLQLRPATDLLLAWNPICLGLVEGVIGKIQLHSD
LPWWLILIRQKALVGKLPGDHEVCKVTKIAVLSLSEMEPQDLELELCKKHHFGINKPEKI
IPSPDDSKFLLKTFTHIKSNVSAPNKKKVKESKEKEKLERRLLEELLKMFMDSESFYYSL
TYDLTNSVQRQSTGERDGRPLWQKVDDRFFWNKYMIQDLTEIGTPDVDFWIIPMIQGFVQ
IEELVVNYTESSDDEKSSPETPPQESTCVDDIHPRFLVALISRRSRHRAGMRYKRRGVDK
NGNVANYVETEQLIHVHNHTLSFVQTRGSVPVFWSQVGYRYNPRPRLDRSEKETVAYFCA
HFEEQLNIYKKQVIINLVDQAGREKIIGDAYLKQVLLFNNSHLTYVSFDFHEHCRGMKFE
NVQTLTDAIYDIILDMKWCWVDEAGVICKQEGIFRVNCMDCLDRTNVVQAAIARVVMEQQ
LKKLGVMPPEQPLPVKCNRIYQIMWANNGDSISRQYAGTAALKGDFTRTGERKLAGVMKD
GVNSANRYYLNRFKDAYRQAVIDLMQGIPVTEDLYSIFTKEKEHEALHKENQRSHQELIS
QLLQSYMKLLLPDDEKFHGGWALIDCDPSLIDATHRDVDVLLLLSNSAYYVAYYDDEVDK
VNQYQRLSLENLEKIEIGPEPTLFGKPKFSCMRLHYRYKEASGYFHTLRAVMRNPEEDGK
DTLQCIAEMLQITKQAMGSDLPIIEKKLERKSSKPHEDIIGIRSQNQGSLAQGKNFLMSK
FSSLNQKVKQTKSNVNIGNLRKLGNFTKPEMKVNFLKPNLKVNLWKSDSSLETMENTGVM
DKVQAESDGDMSSDNDSYHSDEFLTNSKSDEDRQLANSLESVGPIDYVLPSCGIIASAPR
LGSRSQSLSSTDSSVHAPSEITVAHGSGLGKGQESPLKKSPSAGDVHILTGFAKPMDIYC
HRFVQDAQNKVTHLSETRSVSQQASQERNQMTNQVSNETQSESTEQTPSRPSQLDVSLSA
TGPQFLSVEPAHSVASQKTPTSASSMLELETGLHVTPSPSESSSSRAVSPFAKIRSSMVQ
VASITQAGLTHGINFAVSKVQKSPPEPEIINQVQQNELKKMFIQCQTRIIQI
>sp|Q9Y2I1|NISCH_HUMAN Nischarin OS=Homo sapiens OX=9606 GN=NISCH PE=1 SV=3
MATARTFGPEREAEPAKEARVVGSELVDTYTVYIIQVTDGSHEWTVKHRYSDFHDLHEKL
VAERKIDKNLLPPKKIIGKNSRSLVEKREKDLEVYLQKLLAAFPGVTPRVLAHFLHFHFY
EINGITAALAEELFEKGEQLLGAGEVFAIGPLQLYAVTEQLQQGKPTCASGDAKTDLGHI
LDFTCRLKYLKVSGTEGPFGTSNIQEQLLPFDLSIFKSLHQVEISHCDAKHIRGLVASKP
TLATLSVRFSATSMKEVLVPEASEFDEWEPEGTTLEGPVTAVIPTWQALTTLDLSHNSVS
EIDESVKLIPKIEFLDLSHNGLLVVDNLQHLYNLVHLDLSYNKLSSLEGLHTKLGNIKTL
NLAGNLLESLSGLHKLYSLVNLDLRDNRIEQMEEVRSIGSLPCLEHVSLLNNPLSIIPDY
RTKVLAQFGERASEVCLDDTVTTEKELDTVEVLKAIQKAKEVKSKLSNPEKKGGEDSRLS
AAPCIRPSSSPPTVAPASASLPQPILSNQGIMFVQEEALASSLSSTDSLTPEHQPIAQGC
SDSLESIPAGQAASDDLRDVPGAVGGASPEHAEPEVQVVPGSGQIIFLPFTCIGYTATNQ

DFIQRLSTLIRQAIERQLPAWIEAANQREEGQGEQGEEEDEEEEEEEDVAENRYFEMGPP
DVEEEEGGGQGEEEEEEEEDEEAEEERLALEWALGADEDFLLEHIRILKVLWCFLIHVQG
SIRQFAACLVLTDFGIAVFEIPHQESRGSSQHILSSLRFVFCFPHGDLTEFGFLMPELCL
VLKVRHSENTLFIISDAANLHEFHADLRSCFAPQHMAMLCSPILYGSHTSLQEFLRQLLT
FYKVAGGCQERSQGCFPVYLVYSDKRMVQTAAGDYSGNIEWASCTLCSAVRRSCCAPSEA
VKSAAIPYWLLLTPQHLNVIKADFNPMPNRGTHNCRNRNSFKLSRVPLSTVLLDPTRSCT
QPRGAFADGHVLELLVGYRFVTAIFVLPHEKFHFLRVYNQLRASLQDLKTVVIAKTPGTG
GSPQGSFADGQPAERRASNDQRPQEVPAEALAPAPAEVPAPAPAAASASGPAKTPAPAEA
STSALVPEETPVEAPAPPPAEAPAQYPSEHLIQATSEENQIPSHLPACPSLRHVASLRGS
AIIELFHSSIAEVENEELRHLMWSSVVFYQTPGLEVTACVLLSTKAVYFVLHDGLRRYFS
EPLQDFWHQKNTDYNNSPFHISQCFVLKLSDLQSVNVGLFDQHFRLTGSTPMQVVTCLTR
DSYLTHCFLQHLMVVLSSLERTPSPEPVDKDFYSEFGNKTTGKMENYELIHSSRVKFTYP
SEEEIGDLTFTVAQKMAEPEKAPALSILLYVQAFQVGMPPPGCCRGPLRPKTLLLTSSEI
FLLDEDCVHYPLPEFAKEPPQRDRYRLDDGRRVRDLDRVLMGYQTYPQALTLVFDDVQGH
DLMGSVTLDHFGEVPGGPARASQGREVQWQVFVPSAESREKLISLLARQWEALCGRELPV
ELTG
>sp|Q9Y2I7|FYV1_HUMAN 1-phosphatidylinositol 3-phosphate 5-kinase OS=Homo sapiens
OX=9606 GN=PIKFYVE PE=1 SV=3
MATDDKTSPTLDSANDLPRSPTSPSHLTHFKPLTPDQDEPPFKSAYSSFVNLFRFNKERA
EGGQGEQQPLSGSWTSPQLPSRTQSVRSPTPYKKQLNEELQRRSSALDTRRKAEPTFGGH
DPRTAVQLRSLSTVLKRLKEIMEGKSQDSDLKQYWMPDSQCKECYDCSEKFTTFRRRHHC
RLCGQIFCSRCCNQEIPGKFMGYTGDLRACTYCRKIALSYAHSTDSNSIGEDLNALSDSA
CSVSVLDPSEPRTPVGSRKASRNIFLEDDLAWQSLIHPDSSNTPLSTRLVSVQEDAGKSP
ARNRSASITNLSLDRSGSPMVPSYETSVSPQANRTYVRTETTEDERKILLDSVQLKDLWK
KICHHSSGMEFQDHRYWLRTHPNCIVGKELVNWLIRNGHIATRAQAIAIGQAMVDGRWLD
CVSHHDQLFRDEYALYRPLQSTEFSETPSPDSDSVNSVEGHSEPSWFKDIKFDDSDTEQI
AEEGDDNLANSASPSKRTSVSSFQSTVDSDSAASISLNVELDNVNFHIKKPSKYPHVPPH
PADQKEYLISDTGGQQLSISDAFIKESLFNRRVEEKSKELPFTPLGWHHNNLELLREENG
EKQAMERLLSANHNHMMALLQQLLHSDSLSSSWRDIIVSLVCQVVQTVRPDVKNQDDDMD
IRQFVHIKKIPGGKKFDSVVVNGFVCTKNIAHKKMSSCIKNPKILLLKCSIEYLYREETK
FTCIDPIVLQEREFLKNYVQRIVDVRPTLVLVEKTVSRIAQDMLLEHGITLVINVKSQVL
ERISRMTQGDLVMSMDQLLTKPHLGTCHKFYMQIFQLPNEQTKTLMFFEGCPQHLGCTIK
LRGGSDYELARVKEILIFMICVAYHSQLEISFLMDEFAMPPTLMQNPSFHSLIEGRGHEG
AVQEQYGGGSIPWDPDIPPESLPCDDSSLLELRIVFEKGEQENKNLPQAVASVKHQEHST
TACPAGLPCAFFAPVPESLLPLPVDDQQDALGSEQPETLQQTVVLQDPKSQIRAFRDPLQ
DDTGLYVTEEVTSSEDKRKTYSLAFKQELKDVILCISPVITFREPFLLTEKGMRCSTRDY
FAEQVYWSPLLNKEFKEMENRRKKQLLRDLSGLQGMNGSIQAKSIQVLPSHELVSTRIAE
HLGDSQSLGRMLADYRARGGRIQPKNSDPFAHSKDASSTSSGQSGSKNEGDEERGLILSD
AVWSTKVDCLNPINHQRLCVLFSSSSAQSSNAPSACVSPWIVTMEFYGKNDLTLGIFLER
YCFRPSYQCPSMFCDTPMVHHIRRFVHGQGCVQIILKELDSPVPGYQHTILTYSWCRICK
QVTPVVALSNESWSMSFAKYLELRFYGHQYTRRANAEPCGHSIHHDYHQYFSYNQMVASF
SYSPIRLLEVCVPLPKIFIKRQAPLKVSLLQDLKDFFQKVSQVYVAIDERLASLKTDTFS
KTREEKMEDIFAQKEMEEGEFKNWIEKMQARLMSSSVDTPQQLQSVFESLIAKKQSLCEV
LQAWNNRLQDLFQQEKGRKRPSVPPSPGRLRQGEESKISAMDASPRNISPGLQNGEKEDR

FLTTLSSQSSTSSTHLQLPTPPEVMSEQSVGGPPELDTASSSEDVFDGHLLGSTDSQVKE
KSTMKAIFANLLPGNSYNPIPFPFDPDKHYLMYEHERVPIAVCEKEPSSIIAFALSCKEY
RNALEELSKATQWNSAEEGLPTNSTSDSRPKSSSPIRLPEMSGGQTNRTTETEPQPTKKA
SGMLSFFRGTAGKSPDLSSQKRETLRGADSAYYQVGQTGKEGTENQGVEPQDEVDGGDTQ
KKQLINPHVELQFSDANAKFYCRLYYAGEFHKMREVILDSSEEDFIRSLSHSSPWQARGG
KSGAAFYATEDDRFILKQMPRLEVQSFLDFAPHYFNYITNAVQQKRPTALAKILGVYRIG
YKNSQNNTEKKLDLLVMENLFYGRKMAQVFDLKGSLRNRNVKTDTGKESCDVVLLDENLL
KMVRDNPLYIRSHSKAVLRTSIHSDSHFLSSHLIIDYSLLVGRDDTSNELVVGIIDYIRT
FTWDKKLEMVVKSTGILGGQGKMPTVVSPELYRTRFCEAMDKYFLMVPDHWTGLGLNC
>sp|Q9Y2J2|E41L3_HUMAN Band 4.1-like protein 3 OS=Homo sapiens OX=9606 GN=EPB41L3 PE=1 SV=2
MTTESGSDSESKPDQEAEPQEAAGAQGRAGAPVPEPPKEEQQQALEQFAAAAAHSTPVRR
EVTDKEQEFAARAAKQLEYQQLEDDKLSQKSSSSKLSRSPLKIVKKPKSMQCKVILLDGS
EYTCDVEKRSRGQVLFDKVCEHLNLLEKDYFGLTYRDAENQKNWLDPAKEIKKQVRSGAW
HFSFNVKFYPPDPAQLSEDITRYYLCLQLRDDIVSGRLPCSFVTLALLGSYTVQSELGDY
DPDECGSDYISEFRFAPNHTKELEDKVIELHKSHRGMTPAEAEMHFLENAKKLSMYGVDL
HHAKDSEGVEIMLGVCASGLLIYRDRLRINRFAWPKVLKISYKRNNFYIKIRPGEFEQFE
STIGFKLPNHRAAKRLWKVCVEHHTFFRLLLPEAPPKKFLTLGSKFRYSGRTQAQTRRAS
ALIDRPAPYFERSSSKRYTMSRSLDGEVGTGQYATTKGISQTNLITTVTPEKKAEEERDE
EEDKRRKGEEVTPISAIRHEGKSPGLGTDSCPLSPPSTHCAPTSPTELRRRCKENDCKLP
GYEPSRAEHLPGEPALDSDGPGRPYLGDQDVAFSYRQQTGKGTTLFSFSLQLPESFPSLL
DDDGYLSFPNLSETNLLPQSLQHYLPIRSPSLVPCFLFIFFFLLSASFSVPYALTLSFPL
ALCLCYLEPKAASLSASLDNDPSDSSEEETDSERTDTAADGETTATESDQEEDAELKAQE
LEKTQDDLMKHQTNISELKRTFLETSTDTAVTNEWEKRLSTSPVRLAARQEDAPMIEPLV
PEETKQSSGEKLMDGSEIFSLLESARKPTEFIGGVTSTSQSWVQKMETKTESSGIETEPT
VHHLPLSTEKVVQETVLVEERRVVHASGDASYSAGDSGDAAAQPAFTGIKGKEGSALTEG
AKEEGGEEVAKAVLEQEETAAASRERQEEQSAAIHISETLEQKPHFESSTVKTETISFGS
VSPGGVKLEISTKEVPVVHTETKTITYESSQVDPGTDLEPGVLMSAQTITSETTSTTTTT
HITKTVKGGISETRIEKRIVITGDADIDHDQALAQAIKEAKEQHPDMSVTKVVVHKETEI
TPEDGED
>sp|Q9Y2S2|CRYL1_HUMAN Lambda-crystallin homolog OS=Homo sapiens OX=9606 GN=CRYL1 PE=1 SV=3
MASSAAGCVVIVGSGVIGRSWAMLFASGGFQVKLYDIEQQQIRNALENIRKEMKLLEQAG
SLKGSLSVEEQLSLISGCPNIQEAVEGAMHIQECVPEDLELKKKIFAQLDSIIDDRVILS
SSTSCLMPSKLFAGLVHVKQCIVAHPVNPPYYIPLVELVPHPETAPTTVDRTHALMKKIG
QCPMRVQKEVAGFVLNRLQYAIISEAWRLVEEGIVSPSDLDLVMSEGLGMRYAFIGPLET
MHLNAEGMLSYCDRYSEGIKHVLQTFGPIPEFSRATAEKVNQDMCMKVPDDPEHLAARRQ
WRDECLMRLAKLKSQVQPQ
>sp|Q9Y3A5|SBDS_HUMAN Ribosome maturation protein SBDS OS=Homo sapiens OX=9606 GN=SBDS PE=1 SV=4
MSIFTPTNQIRLTNVAVVRMKRAGKRFEIACYKNKVVGWRSGVEKDLDEVLQTHSVFVNV
SKGQVAKKEDLISAFGTDDQTEICKQILTKGEVQVSDKERHTQLEQMFRDIATIVADKCV
NPETKRPYTVILIERAMKDIHYSVKTNKSTKQQALEVIKQLKEKMKIERAHMRLRFILPV
NEGKKLKEKLKPLIKVIESEDYGQQLEIVCLIDPGCFREIDELIKKETKGKGSLEVLNLK

DVEEGDEKFE
>sp|Q9Y3L5|RAP2C_HUMAN Ras-related protein Rap-2c OS=Homo sapiens OX=9606 GN=RAP2C PE=1 SV=1
MREYKVVVLGSGGVGKSALTVQFVTGTFIEKYDPTIEDFYRKEIEVDSSPSVLEILDTAG
TEQFASMRDLYIKNGQGFILVYSLVNQQSFQDIKPMRDQIVRVKRYEKVPLILVGNKVDL
EPEREVMSSEGRALAQEWGCPFMETSAKSKSMVDELFAEIVRQMNYSSLPEKQDQCCTTC
VVQ
>sp|Q9Y3U8|RL36_HUMAN Large ribosomal subunit protein eL36 OS=Homo sapiens OX=9606 GN=RPL36 PE=1 SV=3
MALRYPMAVGLNKGHKVTKNVSKPRHSRRRGRLTKHTKFVRDMIREVCGFAPYERRAMEL
LKVSKDKRALKFIKKRVGTHIRAKRKREELSNVLAAMRKAAAKKD
>sp|Q9Y450|HBS1L_HUMAN HBS1-like protein OS=Homo sapiens OX=9606 GN=HBS1L PE=1 SV=1
MARHRNVRGYNYDEDFEDDDLYGQSVEDDYCISPSTAAQFIYSRRDKPSVEPVEEYDYED
LKESSNSVSNHQLSGFDQARLYSCLDHMREVLGDAVPDEILIEAVLKNKFDVQKALSGVL
EQDRVQSLKDKNEATVSTGKIAKGKPVDSQTSRSESEIVPKVAKMTVSGKKQTMGFEVPG
VSSEENGHSFHTPQKGPPIEDAIASSDVLETASKSANPPHTIQASEEQSSTPAPVKKSGK
LRQQIDVKAELEKRQGGKQLLNLVVIGHVDAGKSTLMGHMLYLLGNINKRTMHKYEQESK
KAGKASFAYAWVLDETGEERERGVTMDVGMTKFETTTKVITLMDAPGHKDFIPNMITGAA
QADVAVLVVDASRGEFEAGFETGGQTREHGLLVRSLGVTQLAVAVNKMDQVNWQQERFQE
ITGKLGHFLKQAGFKESDVGFIPTSGLSGENLITRSQSSELTKWYKGLCLLEQIDSFKPP
QRSIDKPFRLCVSDVFKDQGSGFCITGKIEAGYIQTGDRLLAMPPNETCTVKGITLHDEP
VDWAAAGDHVSLTLVGMDIIKINVGCIFCGPKVPIKACTRFRARILIFNIEIPITKGFPV
LLHYQTVSEPAVIKRLISVLNKSTGEVTKKKPKFLTKGQNALVELQTQRPIALELYKDFK
ELGRFMLRYGGSTIAAGVVTEIKE
>sp|Q9Y490|TLN1_HUMAN Talin-1 OS=Homo sapiens OX=9606 GN=TLN1 PE=1 SV=3
MVALSLKISIGNVVKTMQFEPSTMVYDACRIIRERIPEAPAGPPSDFGLFLSDDDPKKGI
WLEAGKALDYYMLRNGDTMEYRKKQRPLKIRMLDGTVKTIMVDDSKTVTDMLMTICARIG
ITNHDEYSLVRELMEEKKEEITGTLRKDKTLLRDEKKMEKLKQKLHTDDELNWLDHGRTL
REQGVEEHETLLLRRKFFYSDQNVDSRDPVQLNLLYVQARDDILNGSHPVSFDKACEFAG
FQCQIQFGPHNEQKHKAGFLDLKDFLPKEYVKQKGERKIFQAHKNCGQMSEIEAKVRYVK
LARSLKTYGVSFFLVKEKMKGKNKLVPRLLGITKECVMRVDEKTKEVIQEWNLTNIKRWA
ASPKSFTLDFGDYQDGYYSVQTTEGEQIAQLIAGYIDIILKKKKSKDHFGLEGDEESTML
EDSVSPKKSTVLQQQYNRVGKVEHGSVALPAIMRSGASGPENFQVGSMPPAQQQITSGQM
HRGHMPPLTSAQQALTGTINSSMQAVQAAQATLDDFDTLPPLGQDAASKAWRKNKMDESK
HEIHSQVDAITAGTASVVNLTAGDPAETDYTAVGCAVTTISSNLTEMSRGVKLLAALLED
EGGSGRPLLQAAKGLAGAVSELLRSAQPASAEPRQNLLQAAGNVGQASGELLQQIGESDT
DPHFQDALMQLAKAVASAAAALVLKAKSVAQRTEDSGLQTQVIAAATQCALSTSQLVACT
KVVAPTISSPVCQEQLVEAGRLVAKAVEGCVSASQAATEDGQLLRGVGAAATAVTQALNE
LLQHVKAHATGAGPAGRYDQATDTILTVTENIFSSMGDAGEMVRQARILAQATSDLVNAI
KADAEGESDLENSRKLLSAAKILADATAKMVEAAKGAAAHPDSEEQQQRLREAAEGLRMA
TNAAAQNAIKKKLVQRLEHAAKQAAASATQTIAAAQHAASTPKASAGPQPLLVQSCKAVA
EQIPLLVQGVRGSQAQPDSPSAQLALIAASQSFLQPGGKMVAAAKASVPTIQDQASAMQL
SQCAKNLGTALAELRTAAQKAQEACGPLEMDSALSVVQNLEKDLQEVKAAARDGKLKPLP

GETMEKCTQDLGNSTKAVSSAIAQLLGEVAQGNENYAGIAARDVAGGLRSLAQAARGVAA
LTSDPAVQAIVLDTASDVLDKASSLIEEAKKAAGHPGDPESQQRLAQVAKAVTQALNRCV
SCLPGQRDVDNALRAVGDASKRLLSDSLPPSTGTFQEAQSRLNEAAAGLNQAATELVQAS
RGTPQDLARASGRFGQDFSTFLEAGVEMAGQAPSQEDRAQVVSNLKGISMSSSKLLLAAK
ALSTDPAAPNLKSQLAAAARAVTDSINQLITMCTQQAPGQKECDNALRELETVRELLENP
VQPINDMSYFGCLDSVMENSKVLGEAMTGISQNAKNGNLPEFGDAISTASKALCGFTEAA
AQAAYLVGVSDPNSQAGQQGLVEPTQFARANQAIQMACQSLGEPGCTQAQVLSAATIVAK
HTSALCNSCRLASARTTNPTAKRQFVQSAKEVANSTANLVKTIKALDGAFTEENRAQCRA
ATAPLLEAVDNLSAFASNPEFSSIPAQISPEGRAAMEPIVISAKTMLESAGGLIQTARAL
AVNPRDPPSWSVLAGHSRTVSDSIKKLITSMRDKAPGQLECETAIAALNSCLRDLDQASL
AAVSQQLAPREGISQEALHTQMLTAVQEISHLIEPLANAARAEASQLGHKVSQMAQYFEP
LTLAAVGAASKTLSHPQQMALLDQTKTLAESALQLLYTAKEAGGNPKQAAHTQEALEEAV
QMMTEAVEDLTTTLNEAASAAGVVGGMVDSITQAINQLDEGPMGEPEGSFVDYQTTMVRT
AKAIAVTVQEMVTKSNTSPEELGPLANQLTSDYGRLASEAKPAAVAAENEEIGSHIKHRV
QELGHGCAALVTKAGALQCSPSDAYTKKELIECARRVSEKVSHVLAALQAGNRGTQACIT
AASAVSGIIADLDTTIMFATAGTLNREGTETFADHREGILKTAKVLVEDTKVLVQNAAGS
QEKLAQAAQSSVATITRLADVVKLGAASLGAEDPETQVVLINAVKDVAKALGDLISATKA
AAGKVGDDPAVWQLKNSAKVMVTNVTSLLKTVKAVEDEATKGTRALEATTEHIRQELAVF
CSPEPPAKTSTPEDFIRMTKGITMATAKAVAAGNSCRQEDVIATANLSRRAIADMLRACK
EAAYHPEVAPDVRLRALHYGRECANGYLELLDHVLLTLQKPSPELKQQLTGHSKRVAGSV
TELIQAAEAMKGTEWVDPEDPTVIAENELLGAAAAIEAAAKKLEQLKPRAKPKEADESLN
FEEQILEAAKSIAAATSALVKAASAAQRELVAQGKVGAIPANALDDGQWSQGLISAARMV
AAATNNLCEAANAAVQGHASQEKLISSAKQVAASTAQLLVACKVKADQDSEAMKRLQAAG
NAVKRASDNLVKAAQKAAAFEEQENETVVVKEKMVGGIAQIIAAQEEMLRKERELEEARK
KLAQIRQQQYKFLPSELRDEH
>sp|Q9Y4W6|AFG32_HUMAN AFG3-like protein 2 OS=Homo sapiens OX=9606 GN=AFG3L2 PE=1 SV=2
MAHRCLRLWGRGGCWPRGLQQLLVPGGVGPGEQPCLRTLYRFVTTQARASRNSLLTDIIA
AYQRFCSRPPKGFEKYFPNGKNGKKASEPKEVMGEKKESKPAATTRSSGGGGGGGGKRGG
KKDDSHWWSRFQKGDIPWDDKDFRMFFLWTALFWGGVMFYLLLKRSGREITWKDFVNNYL
SKGVVDRLEVVNKRFVRVTFTPGKTPVDGQYVWFNIGSVDTFERNLETLQQELGIEGENR
VPVVYIAESDGSFLLSMLPTVLIIAFLLYTIRRGPAGIGRTGRGMGGLFSVGETTAKVLK
DEIDVKFKDVAGCEEAKLEIMEFVNFLKNPKQYQDLGAKIPKGAILTGPPGTGKTLLAKA
TAGEANVPFITVSGSEFLEMFVGVGPARVRDLFALARKNAPCILFIDEIDAVGRKRGRGN
FGGQSEQENTLNQLLVEMDGFNTTTNVVILAGTNRPDILDPALLRPGRFDRQIFIGPPDI
KGRASIFKVHLRPLKLDSTLEKDKLARKLASLTPGFSGADVANVCNEAALIAARHLSDSI
NQKHFEQAIERVIGGLEKKTQVLQPEEKKTVAYHEAGHAVAGWYLEHADPLLKVSIIPRG
KGLGYAQYLPKEQYLYTKEQLLDRMCMTLGGRVSEEIFFGRITTGAQDDLRKVTQSAYAQ
IVQFGMNEKVGQISFDLPRQGDMVLEKPYSEATARLIDDEVRILINDAYKRTVALLTEKK
ADVEKVALLLLEKEVLDKNDMVELLGPRPFAEKSTYEEFVEGTGSLDEDTSLPEGLKDWN
KEREKEKEEPPGEKVAN
>tr|Q9Y509|Q9Y509_HUMAN VH3 protein (Fragment) OS=Homo sapiens OX=9606 GN=VH3 PE=2 SV=1
QVHLVESGGGVVQPGKSLRLSCEASGFTFSTYGMSWVRQAPGKGLDWVALISYDGSTQYY

AGSVKGRFTISRDNSKNTLYLQMTSLRVEDTAVYYCAKDGNYFDSVGYYYAGIDYWGQGT
LVTVSSASTKGPSVFPLAPSSKSTSGG
>sp|Q9Y5L0|TNPO3_HUMAN Transportin-3 OS=Homo sapiens OX=9606 GN=TNPO3 PE=1 SV=3
MEGAKPTLQLVYQAVQALYHDPDPSGKERASFWLGELQRSVHAWEISDQLLQIRQDVESC
YFAAQTMKMKIQTSFYELPTDSHASLRDSLLTHIQNLKDLSPVIVTQLALAIADLALQMP
SWKGCVQTLVEKYSNDVTSLPFLLEILTVLPEEVHSRSLRIGANRRTEIIEDLAFYSSTV
VSLLMTCVEKAGTDEKMLMKVFRCLGSWFNLGVLDSNFMANNKLLALLFEVLQQDKTSSN
LHEAASDCVCSALYAIENVETNLPLAMQLFQGVLTLETAYHMAVAREDLDKVLNYCRIFT
ELCETFLEKIVCTPGQGLGDLRTLELLLICAGHPQYEVVEISFNFWYRLGEHLYKTNDEV
IHGIFKAYIQRLLHALARHCQLEPDHEGVPEETDDFGEFRMRVSDLVKDLIFLIGSMECF
AQLYSTLKEGNPPWEVTEAVLFIMAAIAKSVDPENNPTLVEVLEGVVRLPETVHTAVRYT
SIELVGEMSEVVDRNPQFLDPVLGYLMKGLCEKPLASAAAKAIHNICSVCRDHMAQHFNG
LLEIARSLDSFLLSPEAAVGLLKGTALVLARLPLDKITECLSELCSVQVMALKKLLSQEP
SNGISSDPTVFLDRLAVIFRHTNPIVENGQTHPCQKVIQEIWPVLSETLNKHRADNRIVE
RCCRCLRFAVRCVGKGSAALLQPLVTQMVNVYHVHQHSCFLYLGSILVDEYGMEEGCRQG
LLDMLQALCIPTFQLLEQQNGLQNHPDTVDDLFRLATRFIQRSPVTLLRSQVVIPILQWA
IASTTLDHRDANCSVMRFLRDLIHTGVANDHEEDFELRKELIGQVMNQLGQQLVSQLLHT
CCFCLPPYTLPDVAEVLWEIMQVDRPTFCRWLENSLKGLPKETTVGAVTVTHKQLTDFHK
QVTSAEECKQVCWALRDFTRLFR
>sp|Q9Y5L5|LENEP_HUMAN Lens epithelial cell protein LEP503 OS=Homo sapiens OX=9606
GN=LENEP PE=1 SV=1
MQPRTQPLAQTLPFFLGGAPRDTGLRVPVIKMGTGWEGFQRTLKEVAYILLCCWCIKELL
D
>sp|Q9Y5P6|GMPPB_HUMAN Mannose-1-phosphate guanyltransferase beta OS=Homo
sapiens OX=9606 GN=GMPPB PE=1 SV=2
MKALILVGGYGTRLRPLTLSTPKPLVDFCNKPILLHQVEALAAAGVDHVILAVSYMSQVL
EKEMKAQEQRLGIRISMSHEEEPLGTAGPLALARDLLSETADPFFVLNSDVICDFPFQAM
VQFHRHHGQEGSILVTKVEEPSKYGVVVCEADTGRIHRFVEKPQVFVSNKINAGMYILSP
AVLQRIQLQPTSIEKEVFPIMAKEGQLYAMELQGFWMDIGQPKDFLTGMCLFLQSLRQKQ
PERLCSGPGIVGNVLVDPSARIGQNCSIGPNVSLGPGVVVEDGVCIRRCTVLRDARIRSH
SWLESCIVGWRCRVGQWVRMENVTVLGEDVIVNDELYLNGASVLPHKSIGESVPEPRIIM
>sp|Q9Y5R8|TPPC1_HUMAN Trafficking protein particle complex subunit 1 OS=Homo sapiens
OX=9606 GN=TRAPPC1 PE=1 SV=1
MTVHNLYLFDRNGVCLHYSEWHRKKQAGIPKEEEYKLMYGMLFSIRSFVSKMSPLDMKDG
FLAFQTSRYKLHYYETPTGIKVVMNTDLGVGPIRDVLHHIYSALYVELVVKNPLCPLGQT
VQSELFRSRLDSYVRSLPFFSARAG
>sp|Q9Y5W5|WIF1_HUMAN Wnt inhibitory factor 1 OS=Homo sapiens OX=9606 GN=WIF1
PE=1 SV=3
MARRSAFPAAALWLWSILLCLLALRAEAGPPQEESLYLWIDAHQARVLIGFEEDILIVSE
GKMAPFTHDFRKAQQRMPAIPVNIHSMNFTWQAAGQAEYFYEFLSLRSLDKGIMADPTVN
VPLLGTVPHKASVVQVGFPCLGKQDGVAAFEVDVIVMNSEGNTILQTPQNAIFFKTCQQA
ECPGGCRNGGFCNERRICECPDGFHGPHCEKALCTPRCMNGGLCVTPGFCICPPGFYGVN
CDKANCSTTCFNGGTCFYPGKCICPPGLEGEQCEISKCPQPCRNGGKCIGKSKCKCSKGY
QGDLCSKPVCEPGCGAHGTCHEPNKCQCQEGWHGRHCNKRYEASLIHALRPAGAQLRQHT

PSLKKAEERRDPPESNYIW
>sp|Q9Y624|JAM1_HUMAN Junctional adhesion molecule A OS=Homo sapiens OX=9606 GN=F11R PE=1 SV=1
MGTKAQVERKLLCLFILAILLCSLALGSVTVHSSEPEVRIPENNPVKLSCAYSGFSSPRV
EWKFDQGDTTRLVCYNNKITASYEDRVTFLPTGITFKSVTREDTGTYTCMVSEEGGNSYG
EVKVKLIVLVPPSKPTVNIPSSATIGNRAVLTCSEQDGSPPSEYTWFKDGIVMPTNPKST
RAFSNSSYVLNPTTGELVFDPLSASDTGEYSCEARNGYGTPMTSNAVRMEAVERNVGVIV
AAVLVTLILLGILVFGIWFAYSRGHFDRTKKGTSSKKVIYSQPSARSEGEFKQTSSFLV
>sp|Q9Y639|NPTN_HUMAN Neuroplastin OS=Homo sapiens OX=9606 GN=NPTN PE=1 SV=2
MSGSSLPSALALSLLLVSGSLLPGPGAAQNAGFVKSPMSETKLTGDAFELYCDVVGSPTP
EIQWWYAEVNRAESFRQLWDGARKRRVTVNTAYGSNGVSVLRITRLTLEDSGTYECRASN
DPKRNDLRQNPSITWIRAQATISVLQKPRIVTSEEVIIRDSPVLPVTLQCNLTSSSHTLT
YSYWTKNGVELSATRKNASNMEYRINKPRAEDSGEYHCVYHFVSAPKANATIEVKAAPDI
TGHKRSENKNEGQDATMYCKSVGYPHPDWIWRKKENGMPMDIVNTSGRFFIINKENYTEL
NIVNLQITEDPGEYECNATNAIGSASVVTVLRVRSHLAPLWPFLGILAEIIILVVIIVVY
EKRKRPDEVPDDDEPAGPMKTNSTNNHKDKNLRQRNTN
>sp|Q9Y6H8|CXA3_HUMAN Gap junction alpha-3 protein OS=Homo sapiens OX=9606 GN=GJA3 PE=1 SV=4
MGDWSFLGRLLENAQEHSTVIGKVWLTVLFIFRILVLGAAAEDVWGDEQSDFTCNTQQPG
CENVCYDRAFPISHIRFWALQIIFVSTPTLIYLGHVLHIVRMEEKKKEREEEEQLKRESP
SPKEPPQDNPSSRDDRGRVRMAGALLRTYVFNIIFKTLFEVGFIAGQYFLYGFELKPLYR
CDRWPCPNTVDCFISRPTEKTIFIIFMLAVACASLLLNMLEIYHLGWKKLKQGVTSRLGP
DASEAPLGTADPPPLPPSSRPPAVAIGFPPYYAHTAAPLGQARAVGYPGAPPPAADFKLL
ALTEARGKGQSAKLYNGHHHLLMTEQNWANQAAERQPPALKAYPAASTPAAPSPVGSSSP
PLAHEAEAGAAPLLLDGSGSSLEGSALAGTPEEEEQAVTTAAQMHQPPLPLGDPGRASKA
SRASSGRARPEDLAI
>sp|Q9Y6M5|ZNT1_HUMAN Proton-coupled zinc antiporter SLC30A1 OS=Homo sapiens OX=9606 GN=SLC30A1 PE=1 SV=3
MGCWGRNRGRLLCMLALTFMFMVLEVVVSRVTSSLAMLSDSFHMLSDVLALVVALVAERF
ARRTHATQKNTFGWIRAEVMGALVNAIFLTGLCFAILLEAIERFIEPHEMQQPLVVLGVG
VAGLLVNVLGLCLFHHHSGFSQDSGHGHSHGGHGHGHGLPKGPRVKSTRPGSSDINVAPG
EQGPDQEETNTLVANTSNSNGLKLDPADPENPRSGDTVEVQVNGNLVREPDHMELEEDRA
GQLNMRGVFLHVLGDALGSVIVVVNALVFYFSWKGCSEGDFCVNPCFPDPCKAFVEIINS
THASVYEAGPCWVLYLDPTLCVVMVCILLYTTYPLLKESALILLQTVPKQIDIRNLIKEL
RNVEGVEEVHELHVWQLAGSRIIATAHIKCEDPTSYMEVAKTIKDVFHNHGIHATTIQPE
FASVGSKSSVVPCELACRTQCALKQCCGTLPQAPSGKDAEKTPAVSISCLELSNNLEKKP
RRTKAENIPAVVIEIKNMPNKQPESSL
>sp|Q9Y6N5|SQOR_HUMAN Sulfide:quinone oxidoreductase, mitochondrial OS=Homo sapiens OX=9606 GN=SQOR PE=1 SV=1
MVPLVAVVSGPRAQLFACLLRLGTQQVGPLQLHTGASHAARNHYEVLVLGGGSGGITMAA
RMKRKVGAENVAIVEPSERHFYQPIWTLVGAGAKQLSSSGRPTASVIPSGVEWIKARVTE
LNPDKNCIHTDDDEKISYRYLIIALGIQLDYEKIKGLPEGFAHPKIGSNYSVKTVEKTWK
ALQDFKEGNAIFTFPNTPVKCAGAPQKIMYLSEAYFRKTGKRSKANIIFNTSLGAIFGVK
KYADALQEIIQERNLTVNYKKNLIEVRADKQEAVFENLDKPGETQVISYEMLHVTPPMSP

PDVLKTSPVADAAGWVDVDKETLQHRRYPNVFGIGDCTNLPTSKTAAAVAAQSGILDRTI
SVIMKNQTPTKKYDGYTSCPLVTGYNRVILAEFDYKAEPLETFPFDQSKERLSMYLMKAD
LMPFLYWNMMLRGYWGGPAFLRKLFHLGMS
