# Supplementary material for: Effects of Aging on Intrinsic Protein Disorder in Human Lenses and Zonules
Source: Cell Biochem Biophys. 2024 Aug 8;82(4):3667–79. doi: 10.1007/s12013-024-01455-x (PMC11576620; doi:10.1007/s12013-024-01455-x)
Supplement: Supplementary file 3 — Supplementary S4 58 year old Lens fasta [file 12013_2024_1455_MOESM3_ESM.pdf]

>sp|P30301|MIP_HUMAN Lens fiber major intrinsic protein OS=Homo sapiens OX=9606 GN=MIP PE=1 SV=1
MWELRSASFWRAIFAEFFATLFYVFFGLGSSLRWAPGPLHVLQVAMAFGLALATLVQSVG
HISGAHVNPAVTFAFLVGSQMSLLRAFCYMAAQLLGAVAGAAVLYSVTPPAVRGNLALNT
LHPAVSVGQATTVEIFLTLQFVLCIFATYDERRNGQLGSVALAVGFSLALGHLFGMYYTG
AGMNPARSFAPAILTGNFTNHWVYWVGPIIGGGLGSLLYDFLLFPRLKSISERLSVLKGA
KPDVSNGQPEVTGEPVELNTQAL
>sp|P02489|CRYAA_HUMAN Alpha-crystallin A chain OS=Homo sapiens OX=9606 GN=CRYAA PE=1 SV=2
MDVTIQHPWFKRTLGPFYPSRLFDQFFGEGLFEYDLLPFLSSTISPYYRQSLFRTVLDSG
ISEVRSDRDKFVIFLDVKHFSPEDLTVKVQDDFVEIHGKHNERQDDHGYISREFHRRYRL
PSNVDQSALSCSLSADGMLTFCGPKIQTGLDATHAERAIPVSREEKPTSAPSS
>sp|P02511|CRYAB_HUMAN Alpha-crystallin B chain OS=Homo sapiens OX=9606 GN=CRYAB PE=1 SV=2
MDIAIHHPWIRRPFFPFHSPSRLFDQFFGEHLLESDLFPTSTSLSPFYLRPPSFLRAPSW
FDTGLSEMRLEKDRFSVNLDVKHFSPEELKVKVLGDVIEVHGKHEERQDEHGFISREFHR
KYRIPADVDPLTITSSLSSDGVLTVNGPRKQVSGPERTIPITREEKPAVTAAPKK
>sp|P53674|CRBB1_HUMAN Beta-crystallin B1 OS=Homo sapiens OX=9606 GN=CRYBB1 PE=1 SV=2
MSQAAKASASATVAVNPGPDTKGKGAPPAGTSPSPGTTLAPTTVPITSAKAAELPPGNYR
LVVFELENFQGRRAEFSGECSNLADRGFDRVRSIIVSAGPWVAFEQSNFRGEMFILEKGE
YPRWNTWSSSYRSDRLMSFRPIKMDAQEHKISLFEGANFKGNTIEIQGDDAPSLWVYGFS
DRVGSVKVSSGTWVGYQYPGYRGYQYLLEPGDFRHWNEWGAFQPQMQSLRRLRDKQWHLE
GSFPVLATEPPK
>sp|P53673|CRBA4_HUMAN Beta-crystallin A4 OS=Homo sapiens OX=9606 GN=CRYBA4 PE=1 SV=3
MTLQCTKSAGPWKMVVWDEDGFQGRRHEFTAECPSVLELGFETVRSLKVLSGAWVGFEHA
GFQGQQYILERGEYPSWDAWGGNTAYPAERLTSFRPAACANHRDSRLTIFEQENFLGKKG
ELSDDYPSLQAMGWEGNEVGSFHVHSGAWVCSQFPGYRGFQYVLECDHHSGDYKHFREWG
SHAPTFQVQSIRRIQQ
>sp|P05813|CRBA1_HUMAN Beta-crystallin A3 OS=Homo sapiens OX=9606 GN=CRYBA1 PE=1 SV=4
METQAEQQELETLPTTKMAQTNPTPGSLGPWKITIYDQENFQGKRMEFTSSCPNVSERSF
DNVRSLKVESGAWIGYEHTSFCGQQFILERGEYPRWDAWSGSNAYHIERLMSFRPICSAN
HKESKMTIFEKENFIGRQWEISDDYPSLQAMGWFNNEVGSMKIQSGAWVCYQYPGYRGYQ
YILECDHHGGDYKHWREWGSHAQTSQIQSIRRIQQ
>sp|P43320|CRBB2_HUMAN Beta-crystallin B2 OS=Homo sapiens OX=9606 GN=CRYBB2 PE=1 SV=2
MASDHQTQAGKPQSLNPKIIIFEQENFQGHSHELNGPCPNLKETGVEKAGSVLVQAGPWV
GYEQANCKGEQFVFEKGEYPRWDSWTSSRRTDSLSSLRPIKVDSQEHKIILYENPNFTGK
KMEIIDDDVPSFHAHGYQEKVSSVRVQSGTWVGYQYPGYRGLQYLLEKGDYKDSSDFGAP
HPQVQSVRRIRDMQWHQRGAFHPSN
>sp|P48165|CXA8_HUMAN Gap junction alpha-8 protein OS=Homo sapiens OX=9606 GN=GJA8 PE=1 SV=3

MGDWSFLGNILEEVNEHSTVIGRVWLTVLFIFRILILGTAAEFVWGDEQSDFVCNTQQPG
CENVCYDEAFPISHIRLWVLQIIFVSTPSLMYVGHAVHYVRMEEKRKSREAEELGQQAGT
NGGPDQGSVKKSSGSKGTKKFRLEGTLLRTYICHIIFKTLFEVGFIVGHYFLYGFRILPL
YRCSRWPCPNVVDCFVSRPTEKTIFILFMLSVASVSLFLNVMELGHLGLKGIRSALKRPV
EQPLGEIPEKSLHSIAVSSIQKAKGYQLLEEEKIVSHYFPLTEVGMVETSPLPAKPFNQF
EEKISTGPLGDLSRGYQETLPSYAQVGAQEVEGEGPPAEEGAEPEVGEKKEEAERLTTEE
QEKVAVPEGEKVETPGVDKEGEKEEPQSEKVSKQGLPAEKTPSLCPELTTDDARPLSRLS
KASSRARSDDLTV
>sp|P22914|CRYGS_HUMAN Gamma-crystallin S OS=Homo sapiens OX=9606 GN=CRYGS PE=1 SV=4
MSKTGTKITFYEDKNFQGRRYDCDCDCADFHTYLSRCNSIKVEGGTWAVYERPNFAGYMY
ILPQGEYPEYQRWMGLNDRLSSCRAVHLPSGGQYKIQIFEKGDFSGQMYETTEDCPSIME
QFHMREIHSCKVLEGVWIFYELPNYRGRQYLLDKKEYRKPIDWGAASPAVQSFRRIVE
>sp|P00558|PGK1_HUMAN Phosphoglycerate kinase 1 OS=Homo sapiens OX=9606 GN=PGK1 PE=1 SV=3
MSLSNKLTLDKLDVKGKRVVMRVDFNVPMKNNQITNNQRIKAAVPSIKFCLDNGAKSVVL
MSHLGRPDGVPMPDKYSLEPVAVELKSLLGKDVLFLKDCVGPEVEKACANPAAGSVILLE
NLRFHVEEEGKGKDASGNKVKAEPAKIEAFRASLSKLGDVYVNDAFGTAHRAHSSMVGVN
LPQKAGGFLMKKELNYFAKALESPERPFLAILGGAKVADKIQLINNMLDKVNEMIIGGGM
AFTFLKVLNNMEIGTSLFDEEGAKIVKDLMSKAEKNGVKITLPVDFVTADKFDENAKTGQ
ATVASGIPAGWMGLDCGPESSKKYAEAVTRAKQIVWNGPVGVFEWEAFARGTKALMDEVV
KATSRGCITIIGGGDTATCCAKWNTEDKVSHVSTGGGASLELLEGKVLPGVDALSNI
>sp|Q03135|CAV1_HUMAN Caveolin-1 OS=Homo sapiens OX=9606 GN=CAV1 PE=1 SV=4
MSGGKYVDSEGHLYTVPIREQGNIYKPNNKAMADELSEKQVYDAHTKEIDLVNRDPKHLN
DDVVKIDFEDVIAEPEGTHSFDGIWKASFTTFTVTKYWFYRLLSALFGIPMALIWGIYFA
ILSFLHIWAVVPCIKSFLIEIQCISRVYSIYVHTVCDPLFEAVGKIFSNVRINLQKEI
>sp|Q12934|BFSP1_HUMAN Filensin OS=Homo sapiens OX=9606 GN=BFSP1 PE=1 SV=3
MYRRSYVFQTRKEQYEHADEASRAAEPERPADEGWAGATSLAALQGLGERVAAHVQRARA
LEQRHAGLRRQLDAFQRLGELAGPEDALARQVESNRQRVRDLEAERARLERQGTEAQRAL
DEFRSKYENECECQLLLKEMLERLNKEADEALLHNLRLQLEAQFLQDDISAAKDRHKKNL
LEVQTYISILQQIIHTTPPASIVTSGMREEKLLTEREVAALRSQLEEGREVLSHLQAQRV
ELQAQTTTLEQAIKSAHECYDDEIQLYNEQIETLRKEIEETERVLEKSSYDCRQLAVAQQ
TLKNELDRYHRIIEIEGNRLTSAFIETPIPLFTQSHGVSLSTGSGGKDLTRALQDITAAK
PRQKALPKNVPRRKEIITKDKTNGALEDAPLKGLEDTKLVQVVLKEESESKFESESKEVS
PLTQEGAPEDVPDGGQISKGFGKLYRKVKEKVRSPKEPETPTELYTKERHVLVTGDANYV
DPRFYVSSITAKGGVAVSVAEDSVLYDGQVEPSPESPKPPLENGQVGLQEKEDGQPIDQQ
PIDKEIEPDGAELEGPEEKREGEERDEESRRPCAMVTPGAEEPSIPEPPKPAADQDGAEV
LGTRSRSLPEKGPPKALAYKTVEVVESIEKISTESIQTYEETAVIVETMIGKTKSDKKKS
GEKSS
>tr|Q14CA1|Q14CA1_HUMAN NRCAM protein OS=Homo sapiens OX=9606 GN=NRCAM PE=2 SV=1
MQLKIMPKKKRLSAGRVPLILFLCQMISALEVPLDPKLLEDLVQPPTITQQSPKDYIIDP
RENIVIQCEAKGKPPPSFSWTRNGTHFDIDKDPLVTMKPGTGTLIINIMSEGKAETYEGV
YQCTARNERGAAVSNNIVVRPSRSPLWTKEKLEPITLQSGQSLVLPCRPPIGLPPPIIFW

MDNSFQRLPQSERVSQGLNGDLYFSNVLPEDTREDYICYARFNHTQTIQQKQPISVKVIS
AKSSRERPPTFLTPEGNASNKEELRGNVLSLECIAEGLPTPIIYWAKEDGMLPKNRTVYK
NFEKTLQIIHVSEADSGNYQCIAKNALGAIHHTISVRVKAAPYWITAPQNLVLSPGEDGT
LICRANGNPKPRISWLTNGVPIEIAPDDPSRKIDGDTIIFSNVQERSSAVYQCNASNEYG
YLLANAFVNVLAEPPRILTPANTLYQVIANRPALLDCAFFGSPLPTIEWFKGAKGSALHE
DIYVLHENGTLEIPVAQKDSTGTYTCVARNKLGMAKNEVHLEIKDATWIVKQPEYAVVQR
GSMVSFECKVKHDHTLSLTVLWLKDNRELPSDERFTVDKDHLVVADVSDDDSGTYTCVAN
TTLDSVSASAVLSVVAPTPTPAPVYDVPNPPFDLELTDQLDKSVQLSWTPGDDNNSPITK
FIIEYEDAMHKPGLWHHQTEVSGTQTTAQLKLSPYVNYSFRVMAVNSIGKSLPSEASEQY
LTKASEPDKNPTAVEGLGSEPDNLVITWKPLNGFESNGPGLQYKVSWRQKDGDDEWTSVV
VANVSKYIVSGTPTFVPYLIKVQALNDMGFAPEPAVVMGHSGEDLPMVAPGNVRVNVVNS
TLAEVHWDPVPLKSIRGHLQGYRIYYWKTQSSSKRNRRHIEKKILTFQGSKTHGMLPGLE
PFSHYTLNVRVVNGKGEGPASPDRVFNTPEGVPSAPSSLKIVNPTLDSLTLEWDPPSHPN
GILTEYTLKYQPINSTHELGPLVDLKIPANKTRWTLKNLNFSTRYKFYFYAQTSAGSGSQ
ITEEAVTTVDEAMASRQVDIATQGWFIGLMCAVALLILILLIVCFIRRNKGGKYPVKEKE
DAHADPEIQPMKEDDGTFGEYSDAEDHKPLKKGSRTPSDRTVKKEDSDDSLVDYGEGVNG
QFNEDGSFIGQYSGKKEKEPAEGNESSEAPSPVNAMNSFV
>sp|Q00796|DHSO_HUMAN Sorbitol dehydrogenase OS=Homo sapiens OX=9606 GN=SORD PE=1 SV=4
MAAAAKPNNLSLVVHGPGDLRLENYPIPEPGPNEVLLRMHSVGICGSDVHYWEYGRIGNF
IVKKPMVLGHEASGTVEKVGSSVKHLKPGDRVAIEPGAPRENDEFCKMGRYNLSPSIFFC
ATPPDDGNLCRFYKHNAAFCYKLPDNVTFEEGALIEPLSVGIHACRRGGVTLGHKVLVCG
AGPIGMVTLLVAKAMGAAQVVVTDLSATRLSKAKEIGADLVLQISKESPQEIARKVEGQL
GCKPEVTIECTGAEASIQAGIYATRSGGNLVLVGLGSEMTTVPLLHAAIREVDIKGVFRY
CNTWPVAISMLASKSVNVKPLVTHRFPLEKALEAFETFKKGLGLKIMLKCDPSDQNP
>sp|Q9ULX7|CAH14_HUMAN Carbonic anhydrase 14 OS=Homo sapiens OX=9606 GN=CA14 PE=1 SV=1
MLFSALLLEVIWILAADGGQHWTYEGPHGQDHWPASYPECGNNAQSPIDIQTDSVTFDPD
LPALQPHGYDQPGTEPLDLHNNGHTVQLSLPSTLYLGGLPRKYVAAQLHLHWGQKGSPGG
SEHQINSEATFAELHIVHYDSDSYDSLSEAAERPQGLAVLGILIEVGETKNIAYEHILSH
LHEVRHKDQKTSVPPFNLRELLPKQLGQYFRYNGSLTTPPCYQSVLWTVFYRRSQISMEQ
LEKLQGTLFSTEEEPSKLLVQNYRALQPLNQRMVFASFIQAGSSYTTGEMLSLGVGILVG
CLCLLLAVYFIARKIRKKRLENRKSVVFTSAQATTEA
>sp|Q13515|BFSP2_HUMAN Phakinin OS=Homo sapiens OX=9606 GN=BFSP2 PE=1 SV=1
MSERRVVVDLPTSASSSMPLQRRRASFRGPRSSSSLESPPASRTNAMSGLVRAPGVYVGT
APSGCIGGLGARVTRRALGISSVFLQGLRSSGLATVPAPGLERDHGAVEDLGGCLVEYMA
KVHALEQVSQELETQLRMHLESKATRSGNWGALRASWASSCQQVGEAVLENARLMLQTET
IQAGADDFKERYENEQPFRKAAEEEINSLYKVIDEANLTKMDLESQIESLKEELGSLSRN
YEEDVKLLHKQLAGCELEQMDAPIGTGLDDILETIRIQWERDVEKNRVEAGALLQAKQQA
EVAHMSQTQEEKLAAALRVELHNTSCQVQSLQAETESLRALKRGLENTLHDAKHWHDMEL
QNLGAVVGRLEAELREIRAEAEQQQQERAHLLARKCQLQKDVASYHALLDREESG
>sp|Q14254|FLOT2_HUMAN Flotillin-2 OS=Homo sapiens OX=9606 GN=FLOT2 PE=1 SV=2
MGNCHTVGPNEALVVSGGCCGSDYKQYVFGGWAWAWWCISDTQRISLEIMTLQPRCEDVE
TAEGVALTVTGVAQVKIMTEKELLAVACEQFLGKNVQDIKNVVLQTLEGHLRSILGTLTV

EQIYQDRDQFAKLVREVAAPDVGRMGIEILSFTIKDVYDKVDYLSSLGKTQTAVVQRDAD
IGVAEAERDAGIREAECKKEMLDVKFMADTKIADSKRAFELQKSAFSEEVNIKTAEAQLA
YELQGAREQQKIRQEEIEIEVVQRKKQIAVEAQEILRTDKELIATVRRPAEAEAHRIQQI
AEGEKVKQVLLAQAEAEKIRKIGEAEAAVIEAMGKAEAERMKLKAEAYQKYGDAAKMALV
LEALPQIAAKIAAPLTKVDEIVVLSGDNSKVTSEVNRLLAELPASVHALTGVDLSKIPLI
KKATGVQV
>sp|P68032|ACTC_HUMAN Actin, alpha cardiac muscle 1 OS=Homo sapiens OX=9606
GN=ACTC1 PE=1 SV=1
MCDDEETTALVCDNGSGLVKAGFAGDDAPRAVFPSIVGRPRHQGVMVGMGQKDSYVGDEA
QSKRGILTLKYPIEHGIITNWDDMEKIWHHTFYNELRVAPEEHPTLLTEAPLNPKANREK
MTQIMFETFNVPAMYVAIQAVLSLYASGRTTGIVLDSGDGVTHNVPIYEGYALPHAIMRL
DLAGRDLTDYLMKILTERGYSFVTTAEREIVRDIKEKLCYVALDFENEMATAASSSSLEK
SYELPDGQVITIGNERFRCPETLFQPSFIGMESAGIHETTYNSIMKCDIDIRKDLYANNV
LSGGTTMYPGIADRMQKEITALAPSTMKIKIIAPPERKYSVWIGGSILASLSTFQQMWIS
KQEYDEAGPSIVHRKCF
>sp|P00352|AL1A1_HUMAN Aldehyde dehydrogenase 1A1 OS=Homo sapiens OX=9606
GN=ALDH1A1 PE=1 SV=2
MSSSGTPDLPVLLTDLKIQYTKIFINNEWHDSVSGKKFPVFNPATEEELCQVEEGDKEDV
DKAVKAARQAFQIGSPWRTMDASERGRLLYKLADLIERDRLLLATMESMNGGKLYSNAYL
NDLAGCIKTLRYCAGWADKIQGRTIPIDGNFFTYTRHEPIGVCGQIIPWNFPLVMLIWKI
GPALSCGNTVVVKPAEQTPLTALHVASLIKEAGFPPGVVNIVPGYGPTAGAAISSHMDID
KVAFTGSTEVGKLIKEAAGKSNLKRVTLELGGKSPCIVLADADLDNAVEFAHHGVFYHQG
QCCIAASRIFVEESIYDEFVRRSVERAKKYILGNPLTPGVTQGPQIDKEQYDKILDLIES
GKKEGAKLECGGGPWGNKGYFVQPTVFSNVTDEMRIAKEEIFGPVQQIMKFKSLDDVIKR
ANNTFYGLSAGVFTKDIDKAITISSALQAGTVWVNCYGVVSAQCPFGGFKMSGNGRELGE
YGFHEYTEVKTVTVKISQKNS
>sp|P05023|AT1A1_HUMAN Sodium/potassium-transporting ATPase subunit alpha-1
OS=Homo sapiens OX=9606 GN=ATP1A1 PE=1 SV=1
MGKGVGRDKYEPAAVSEQGDKKGKKGKKDRDMDELKKEVSMDDHKLSLDELHRKYGTDLS
RGLTSARAAEILARDGPNALTPPPTTPEWIKFCRQLFGGFSMLLWIGAILCFLAYSIQAA
TEEEPQNDNLYLGVVLSAVVIITGCFSYYQEAKSSKIMESFKNMVPQQALVIRNGEKMSI
NAEEVVVGDLVEVKGGDRIPADLRIISANGCKVDNSSLTGESEPQTRSPDFTNENPLETR
NIAFFSTNCVEGTARGIVVYTGDRTVMGRIATLASGLEGGQTPIAAEIEHFIHIITGVAV
FLGVSFFILSLILEYTWLEAVIFLIGIIVANVPEGLLATVTVCLTLTAKRMARKNCLVKN
LEAVETLGSTSTICSDKTGTLTQNRMTVAHMWFDNQIHEADTTENQSGVSFDKTSATWLA
LSRIAGLCNRAVFQANQENLPILKRAVAGDASESALLKCIELCCGSVKEMRERYAKIVEI
PFNSTNKYQLSIHKNPNTSEPQHLLVMKGAPERILDRCSSILLHGKEQPLDEELKDAFQN
AYLELGGLGERVLGFCHLFLPDEQFPEGFQFDTDDVNFPIDNLCFVGLISMIDPPRAAVP
DAVGKCRSAGIKVIMVTGDHPITAKAIAKGVGIISEGNETVEDIAARLNIPVSQVNPRDA
KACVVHGSDLKDMTSEQLDDILKYHTEIVFARTSPQQKLIIVEGCQRQGAIVAVTGDGVN
DSPALKKADIGVAMGIAGSDVSKQAADMILLDDNFASIVTGVEEGRLIFDNLKKSIAYTL
TSNIPEITPFLIFIIANIPLPLGTVTILCIDLGTDMVPAISLAYEQAESDIMKRQPRNPK
TDKLVNERLISMAYGQIGMIQALGGFFTYFVILAENGFLPIHLLGLRVDWDDRWINDVED
SYGQQWTYEQRKIVEFTCHTAFFVSIVVVQWADLVICKTRRNSVFQQGMKNKILIFGLFE

ETALAAFLSYCPGMGVALRMYPLKPTWWFCAFPYSLLIFVYDEVRKLIIRRRPGGWVEKE
TYY
>tr|B4DXX3|B4DXX3_HUMAN Aldehyde dehydrogenase 1A1 OS=Homo sapiens OX=9606 PE=2
SV=1
MSSSGTPDLPVLLTDLKIQYTKIFINNEWHDSVSGKKFPVFNPATEEELCQVEEGDKEDV
DKAVKAARQAFQIGSPWRTMDASERGRLLYKLADLIERDRLLLATMESMNGGNTVVVKPA
EQTPLTALHVASLIKEAGFPPGVVNIVPGYGPTAGAAISSHMDIDKVAFTGSTEVGKLIK
EAAGKSNLKRVTLELGGKSPCIVLADADLDNAVEFAHHGVFYHQGQCCIAASRIFVEESI
YDEFVRRSVERAKKYILGNPLTPGVTQGPQIDKEQYDKILDLIESGKKEGAKLECGGGPW
GNKGYFVQPTVFSNVTDEMRIAKEEIFGPVQQIMKFKSLDDVIERANNTFYGLSAGVFTK
DIDKAITISSALQAGTVWVNCYGVGRVRFP
>sp|P13637|AT1A3_HUMAN Sodium/potassium-transporting ATPase subunit alpha-3
OS=Homo sapiens OX=9606 GN=ATP1A3 PE=1 SV=3
MGDKKDDKDSPKKNKGKERRDLDDLKKEVAMTEHKMSVEEVCRKYNTDCVQGLTHSKAQE
ILARDGPNALTPPPTTPEWVKFCRQLFGGFSILLWIGAILCFLAYGIQAGTEDDPSGDNL
YLGIVLAAVVIITGCFSYYQEAKSSKIMESFKNMVPQQALVIREGEKMQVNAEEVVVGDL
VEIKGGDRVPADLRIISAHGCKVDNSSLTGESEPQTRSPDCTHDNPLETRNITFFSTNCV
EGTARGVVVATGDRTVMGRIATLASGLEVGKTPIAIEIEHFIQLITGVAVFLGVSFFILS
LILGYTWLEAVIFLIGIIVANVPEGLLATVTVCLTLTAKRMARKNCLVKNLEAVETLGST
STICSDKTGTLTQNRMTVAHMWFDNQIHEADTTEDQSGTSFDKSSHTWVALSHIAGLCNR
AVFKGGQDNIPVLKRDVAGDASESALLKCIELSSGSVKLMRERNKKVAEIPFNSTNKYQL
SIHETEDPNDNRYLLVMKGAPERILDRCSTILLQGKEQPLDEEMKEAFQNAYLELGGLGE
RVLGFCHYYLPEEQFPKGFAFDCDDVNFTTDNLCFVGLMSMIDPPRAAVPDAVGKCRSAG
IKVIMVTGDHPITAKAIAKGVGIISEGNETVEDIAARLNIPVSQVNPRDAKACVIHGTDL
KDFTSEQIDEILQNHTEIVFARTSPQQKLIIVEGCQRQGAIVAVTGDGVNDSPALKKADI
GVAMGIAGSDVSKQAADMILLDDNFASIVTGVEEGRLIFDNLKKSIAYTLTSNIPEITPF
LLFIMANIPLPLGTITILCIDLGTDMVPAISLAYEAAESDIMKRQPRNPRTDKLVNERLI
SMAYGQIGMIQALGGFFSYFVILAENGFLPGNLVGIRLNWDDRTVNDLEDSYGQQWTYEQ
RKVVEFTCHTAFFVSIVVVQWADLIICKTRRNSVFQQGMKNKILIFGLFEETALAAFLSY
CPGMDVALRMYPLKPSWWFCAFPYSFLIFVYDEIRKLILRRNPGGWVEKETYY
>sp|Q9Y6H8|CXA3_HUMAN Gap junction alpha-3 protein OS=Homo sapiens OX=9606
GN=GJA3 PE=1 SV=4
MGDWSFLGRLLENAQEHSTVIGKVWLTVLFIFRILVLGAAAEDVWGDEQSDFTCNTQQPG
CENVCYDRAFPISHIRFWALQIIFVSTPTLIYLGHVLHIVRMEEKKKEREEEEQLKRESP
SPKEPPQDNPSSRDDRGRVRMAGALLRTYVFNIIFKTLFEVGFIAGQYFLYGFELKPLYR
CDRWPCPNTVDCFISRPTEKTIFIIFMLAVACASLLLNMLEIYHLGWKKLKQGVTSRLGP
DASEAPLGTADPPPLPPSSRPPAVAIGFPPYYAHTAAPLGQARAVGYPGAPPPAADFKLL
ALTEARGKGQSAKLYNGHHHLLMTEQNWANQAAERQPPALKAYPAASTPAAPSPVGSSSP
PLAHEAEAGAAPLLLDGSGSSLEGSALAGTPEEEEQAVTTAAQMHQPPLPLGDPGRASKA
SRASSGRARPEDLAI
>sp|P07320|CRGD_HUMAN Gamma-crystallin D OS=Homo sapiens OX=9606 GN=CRYGD PE=1
SV=3
MGKITLYEDRGFQGRHYECSSDHPNLQPYLSRCNSARVDSGCWMLYEQPNYSGLQYFLRR
GDYADHQQWMGLSDSVRSCRLIPHSGSHRIRLYEREDYRGQMIEFTEDCSCLQDRFRFNE

IHSLNVLEGSWVLYELSNYRGRQYLLMPGDYRRYQDWGATNARVGSLRRVIDFS
>sp|Q14203|DCTN1_HUMAN Dynactin subunit 1 OS=Homo sapiens OX=9606 GN=DCTN1 PE=1 SV=3
MAQSKRHVYSRTPSGSRMSAEASARPLRVGSRVEVIGKGHRGTVAYVGATLFATGKWVGV
ILDEAKGKNDGTVQGRKYFTCDEGHGIFVRQSQIQVFEDGADTTSPETPDSSASKVLKRE
GTDTTAKTSKLRGLKPKKAPTARKTTTRRPKPTRPASTGVAGASSSLGPSGSASAGELSS
SEPSTPAQTPLAAPIIPTPVLTSPGAVPPLPSPSKEEEGLRAQVRDLEEKLETLRLKRAE
DKAKLKELEKHKIQLEQVQEWKSKMQEQQADLQRRLKEARKEAKEALEAKERYMEEMADT
ADAIEMATLDKEMAEERAESLQQEVEALKERVDELTTDLEILKAEIEEKGSDGAASSYQL
KQLEEQNARLKDALVRMRDLSSSEKQEHVKLQKLMEKKNQELEVVRQQRERLQEELSQAE
STIDELKEQVDAALGAEEMVEMLTDRNLNLEEKVRELRETVGDLEAMNEMNDELQENARE
TELELREQLDMAGARVREAQKRVEAAQETVADYQQTIKKYRQLTAHLQDVNRELTNQQEA
SVERQQQPPPETFDFKIKFAETKAHAKAIEMELRQMEVAQANRHMSLLTAFMPDSFLRPG
GDHDCVLVLLLMPRLICKAELIRKQAQEKFELSENCSERPGLRGAAGEQLSFAAGLVYSL
SLLQATLHRYEHALSQCSVDVYKKVGSLYPEMSAHERSLDFLIELLHKDQLDETVNVEPL
TKAIKYYQHLYSIHLAEQPEDCTMQLADHIKFTQSALDCMSVEVGRLRAFLQGGQEATDI
ALLLRDLETSCSDIRQFCKKIRRRMPGTDAPGIPAALAFGPQVSDTLLDCRKHLTWVVAV
LQEVAAAAAQLIAPLAENEGLLVAALEELAFKASEQIYGTPSSSPYECLRQSCNILISTM
NKLATAMQEGEYDAERPPSKPPPVELRAAALRAEITDAEGLGLKLEDRETVIKELKKSLK
IKGEELSEANVRLSLLEKKLDSAAKDADERIEKVQTRLEETQALLRKKEKEFEETMDALQ
ADIDQLEAEKAELKQRLNSQSKRTIEGLRGPPPSGIATLVSGIAGEEQQRGAIPGQAPGS
VPGPGLVKDSPLLLQQISAMRLHISQLQHENSILKGAQMKASLASLPPLHVAKLSHEGPG
SELPAGALYRKTSQLLETLNQLSTHTHVVDITRTSPAAKSPSAQLMEQVAQLKSLSDTVE
KLKDEVLKETVSQRPGATVPTDFATFPSSAFLRAKEEQQDDTVYMGKVTFSCAAGFGQRH
RLVLTQEQLHQLHSRLIS
>sp|P48637|GSHB_HUMAN Glutathione synthetase OS=Homo sapiens OX=9606 GN=GSS PE=1 SV=1
MATNWGSLLQDKQQLEELARQAVDRALAEGVLLRTSQEPTSSEVVSYAPFTLFPSLVPSA
LLEQAYAVQMDFNLLVDAVSQNAAFLEQTLSSTIKQDDFTARLFDIHKQVLKEGIAQTVF
LGLNRSDYMFQRSADGSPALKQIEINTISASFGGLASRTPAVHRHVLSVLSKTKEAGKIL
SNNPSKGLALGIAKAWELYGSPNALVLLIAQEKERNIFDQRAIENELLARNIHVIRRTFE
DISEKGSLDQDRRLFVDGQEIAVVYFRDGYMPRQYSLQNWEARLLLERSHAAKCPDIATQ
LAGTKKVQQELSRPGMLEMLLPGQPEAVARLRATFAGLYSLDVGEEGDQAIAEALAAPSR
FVLKPQREGGGNNLYGEEMVQALKQLKDSEERASYILMEKIEPEPFENCLLRPGSPARVV
QCISELGIFGVYVRQEKTLVMNKHVGHLLRTKAIEHADGGVAAGVAVLDNPYPV
>sp|Q6UWM7|LCTL_HUMAN Lactase-like protein OS=Homo sapiens OX=9606 GN=LCTL PE=1 SV=2
MKPVWVATLLWMLLLVPRLGAARKGSPEEASFYYGTFPLGFSWGVGSSAYQTEGAWDQDG
KGPSIWDVFTHSGKGKVLGNETADVACDGYYKVQEDIILLRELHVNHYRFSLSWPRLLPT
GIRAEQVNKKGIEFYSDLIDALLSSNITPIVTLHHWDLPQLLQVKYGGWQNVSMANYFRD
YANLCFEAFGDRVKHWITFSDPRAMAEKGYETGHHAPGLKLRGTGLYKAAHHIIKAHAKA
WHSYNTTWRSKQQGLVGISLNCDWGEPVDISNPKDLEAAERYLQFCLGWFANPIYAGDYP
QVMKDYIGRKSAEQGLEMSRLPVFSLQEKSYIKGTSDFLGLGHFTTRYITERNYPSRQGP
SYQNDRDLIELVDPNWPDLGSKWLYSVPWGFRRLLNFAQTQYGDPPIYVMENGASQKFHC

TQLCDEWRIQYLKGYINEMLKAIKDGANIKGYTSWSLLDKFEWEKGYSDRYGFYYVEFND
RNKPRYPKASVQYYKKIIIANGFPNPREVESWYLKALETCSINNQMLAAEPLLSHMQMVT
EIVVPTVCSLCVLITAVLLMLLLRRQS
>sp|Q92823|NRCAM_HUMAN Neuronal cell adhesion molecule OS=Homo sapiens OX=9606 GN=NRCAM PE=1 SV=3
MQLKIMPKKKRLSAGRVPLILFLCQMISALEVPLDPKLLEDLVQPPTITQQSPKDYIIDP
RENIVIQCEAKGKPPPSFSWTRNGTHFDIDKDPLVTMKPGTGTLIINIMSEGKAETYEGV
YQCTARNERGAAVSNNIVVRPSRSPLWTKEKLEPITLQSGQSLVLPCRPPIGLPPPIIFW
MDNSFQRLPQSERVSQGLNGDLYFSNVLPEDTREDYICYARFNHTQTIQQKQPISVKVIS
VDELNDTIAANLSDTEFYGAKSSRERPPTFLTPEGNASNKEELRGNVLSLECIAEGLPTP
IIYWAKEDGMLPKNRTVYKNFEKTLQIIHVSEADSGNYQCIAKNALGAIHHTISVRVKAA
PYWITAPQNLVLSPGEDGTLICRANGNPKPRISWLTNGVPIEIAPDDPSRKIDGDTIIFS
NVQERSSAVYQCNASNEYGYLLANAFVNVLAEPPRILTPANTLYQVIANRPALLDCAFFG
SPLPTIEWFKGAKGSALHEDIYVLHENGTLEIPVAQKDSTGTYTCVARNKLGMAKNEVHL
EIKDPTWIVKQPEYAVVQRGSMVSFECKVKHDHTLSLTVLWLKDNRELPSDERFTVDKDH
LVVADVSDDDSGTYTCVANTTLDSVSASAVLSVVAPTPTPAPVYDVPNPPFDLELTDQLD
KSVQLSWTPGDDNNSPITKFIIEYEDAMHKPGLWHHQTEVSGTQTTAQLKLSPYVNYSFR
VMAVNSIGKSLPSEASEQYLTKASEPDKNPTAVEGLGSEPDNLVITWKPLNGFESNGPGL
QYKVSWRQKDGDDEWTSVVVANVSKYIVSGTPTFVPYLIKVQALNDMGFAPEPAVVMGHS
GEDLPMVAPGNVRVNVVNSTLAEVHWDPVPLKSIRGHLQGYRIYYWKTQSSSKRNRRHIE
KKILTFQGSKTHGMLPGLEPFSHYTLNVRVVNGKGEGPASPDRVFNTPEGVPSAPSSLKI
VNPTLDSLTLEWDPPSHPNGILTEYTLKYQPINSTHELGPLVDLKIPANKTRWTLKNLNF
STRYKFYFYAQTSAGSGSQITEEAVTTVDEAGILPPDVGAGKVQAVNPRISNLTAAAAET
YANISWEYEGPEHVNFYVEYGVAGSKEEWRKEIVNGSRSFFGLKGLMPGTAYKVRVGAVG
DSGFVSSEDVFETGPAMASRQVDIATQGWFIGLMCAVALLILILLIVCFIRRNKGGKYPV
KEKEDAHADPEIQPMKEDDGTFGEYSDAEDHKPLKKGSRTPSDRTVKKEDSDDSLVDYGE
GVNGQFNEDGSFIGQYSGKKEKEPAEGNESSEAPSPVNAMNSFV
>sp|P55064|AQP5_HUMAN Aquaporin-5 OS=Homo sapiens OX=9606 GN=AQP5 PE=1 SV=1
MKKEVCSVAFLKAVFAEFLATLIFVFFGLGSALKWPSALPTILQIALAFGLAIGTLAQAL
GPVSGGHINPAITLALLVGNQISLLRAFFYVAAQLVGAIAGAGILYGVAPLNARGNLAVN
ALNNNTTQGQAMVVELILTFQLALCIFASTDSRRTSPVGSPALSIGLSVTLGHLVGIYFT
GCSMNPARSFGPAVVMNRFSPAHWVFWVGPIVGAVLAAILYFYLLFPNSLSLSERVAIIK
GTYEPDEDWEEQREERKKTMELTTR
>sp|P30041|PRDX6_HUMAN Peroxiredoxin-6 OS=Homo sapiens OX=9606 GN=PRDX6 PE=1 SV=3
MPGGLLLGDVAPNFEANTTVGRIRFHDFLGDSWGILFSHPRDFTPVCTTELGRAAKLAPE
FAKRNVKLIALSIDSVEDHLAWSKDINAYNCEEPTEKLPFPIIDDRNRELAILLGMLDPA
EKDEKGMPVTARVVFVFGPDKKLKLSILYPATTGRNFDEILRVVISLQLTAEKRVATPVD
WKDGDSVMVLPTIPEEEAKKLFPKGVFTKELPSGKKYLRYTPQP
>sp|P60709|ACTB_HUMAN Actin, cytoplasmic 1 OS=Homo sapiens OX=9606 GN=ACTB PE=1 SV=1
MDDDIAALVVDNGSGMCKAGFAGDDAPRAVFPSIVGRPRHQGVMVGMGQKDSYVGDEAQS
KRGILTLKYPIEHGIVTNWDDMEKIWHHTFYNELRVAPEEHPVLLTEAPLNPKANREKMT
QIMFETFNTPAMYVAIQAVLSLYASGRTTGIVMDSGDGVTHTVPIYEGYALPHAILRLDL

AGRDLTDYLMKILTERGYSFTTTAEREIVRDIKEKLCYVALDFEQEMATAASSSSLEKSY
ELPDGQVITIGNERFRCPEALFQPSFLGMESCGIHETTFNSIMKCDVDIRKDLYANTVLS
GGTTMYPGIADRMQKEITALAPSTMKIKIIAPPERKYSVWIGGSILASLSTFQQMWISKQ
EYDESGPSIVHRKCF
>sp|P11166|GTR1_HUMAN Solute carrier family 2, facilitated glucose transporter member 1 OS=Homo sapiens OX=9606 GN=SLC2A1 PE=1 SV=2
MEPSSKKLTGRLMLAVGGAVLGSLQFGYNTGVINAPQKVIEEFYNQTWVHRYGESILPTT
LTTLWSLSVAIFSVGGMIGSFSVGLFVNRFGRRNSMLMMNLLAFVSAVLMGFSKLGKSFE
MLILGRFIIGVYCGLTTGFVPMYVGEVSPTALRGALGTLHQLGIVVGILIAQVFGLDSIM
GNKDLWPLLLSIIFIPALLQCIVLPFCPESPRFLLINRNEENRAKSVLKKLRGTADVTHD
LQEMKEESRQMMREKKVTILELFRSPAYRQPILIAVVLQLSQQLSGINAVFYYSTSIFEK
AGVQQPVYATIGSGIVNTAFTVVSLFVVERAGRRTLHLIGLAGMAGCAILMTIALALLEQ
LPWMSYLSIVAIFGFVAFFEVGPGPIPWFIVAELFSQGPRPAAIAVAGFSNWTSNFIVGM
CFQYVEQLCGPYVFIIFTVLLVLFFIFTYFKVPETKGRTFDEIASGFRQGGASQSDKTPE
ELFHPLGADSQV
>sp|O75955|FLOT1_HUMAN Flotillin-1 OS=Homo sapiens OX=9606 GN=FLOT1 PE=1 SV=3
MFFTCGPNEAMVVSGFCRSPPVMVAGGRVFVLPCIQQIQRISLNTLTLNVKSEKVYTRHG
VPISVTGIAQVKIQGQNKEMLAAACQMFLGKTEAEIAHIALETLEGHQRAIMAHMTVEEI
YKDRQKFSEQVFKVASSDLVNMGISVVSYTLKDIHDDQDYLHSLGKARTAQVQKDARIGE
AEAKRDAGIREAKAKQEKVSAQYLSEIEMAKAQRDYELKKAAYDIEVNTRRAQADLAYQL
QVAKTKQQIEEQRVQVQVVERAQQVAVQEQEIARREKELEARVRKPAEAERYKLERLAEA
EKSQLIMQAEAEAASVRMRGEAEAFAIGARARAEAEQMAKKAEAFQLYQEAAQLDMLLEK
LPQVAEEISGPLTSANKITLVSSGSGTMGAAKVTGEVLDILTRLPESVERLTGVSISQVN
HKPLRTA
>sp|P53672|CRBA2_HUMAN Beta-crystallin A2 OS=Homo sapiens OX=9606 GN=CRYBA2 PE=1 SV=3
MSSAPAPGPAPASLTLWDEEDFQGRRCRLLSDCANVCERGGLPRVRSVKVENGVWVAFEY
PDFQGQQFILEKGDYPRWSAWSGSSSHNSNQLLSFRPVLCANHNDSRVTLFEGDNFQGCK
FDLVDDYPSLPSMGWASKDVGSLKVSSGAWVAYQYPGYRGYQYVLERDRHSGEFCTYGEL
GTQAHTGQLQSIRRVQH
>sp|P04406|G3P_HUMAN Glyceraldehyde-3-phosphate dehydrogenase OS=Homo sapiens OX=9606 GN=GAPDH PE=1 SV=3
MGKVKVGVNGFGRIGRLVTRAAFNSGKVDIVAINDPFIDLNYMVYMFQYDSTHGKFHGTV
KAENGKLVINGNPITIFQERDPSKIKWGDAGAEYVVESTGVFTTMEKAGAHLQGGAKRVI
ISAPSADAPMFVMGVNHEKYDNSLKIISNASCTTNCLAPLAKVIHDNFGIVEGLMTTVHA
ITATQKTVDGPSGKLWRDGRGALQNIIPASTGAAKAVGKVIPELNGKLTGMAFRVPTANV
SVVDLTCRLEKPAKYDDIKKVVKQASEGPLKGILGYTEHQVVSSDFNSDTHSSTFDAGAG
IALNDHFVKLISWYDNEFGYSNRVVDLMAHMASKE
>sp|P35237|SPB6_HUMAN Serpin B6 OS=Homo sapiens OX=9606 GN=SERPINB6 PE=1 SV=3
MDVLAEANGTFALNLLKTLGKDNSKNVFFSPMSMSCALAMVYMGAKGNTAAQMAQILSFN
KSGGGGDIHQGFQSLLTEVNKTGTQYLLRMANRLFGEKSCDFLSSFRDSCQKFYQAEMEE
LDFISAVEKSRKHINTWVAEKTEGKIAELLSPGSVDPLTRLVLVNAVYFRGNWDEQFDKE
NTEERLFKVSKNEEKPVQMMFKQSTFKKTYIGEIFTQILVLPYVGKELNMIIMLPDETTD
LRTVEKELTYEKFVEWTRLDMMDEEEVEVSLPRFKLEESYDMESVLRNLGMTDAFELGKA

DFSGMSQTDLSLSKVVHKSFVEVNEEGTEAAAATAAIMMMRCARFVPRFCADHPFLFFIQ
HSKTNGILFCGRFSSP
>sp|O00410|IPO5_HUMAN Importin-5 OS=Homo sapiens OX=9606 GN=IPO5 PE=1 SV=4
MAAAAAEQQQFYLLLGNLLSPDNVVRKQAEETYENIPGQSKITFLLQAIRNTTAAEEARQ
MAAVLLRRLLSSAFDEVYPALPSDVQTAIKSELLMIIQMETQSSMRKKVCDIAAELARNL
IDEDGNNQWPEGLKFLFDSVSSQNVGLREAALHIFWNFPGIFGNQQQHYLDVIKRMLVQC
MQDQEHPSIRTLSARATAAFILANEHNVALFKHFADLLPGFLQAVNDSCYQNDDSVLKSL
VEIADTVPKYLRPHLEATLQLSLKLCGDTSLNNMQRQLALEVIVTLSETAAAMLRKHTNI
VAQTIPQMLAMMVDLEEDEDWANADELEDDDFDSNAVAGESALDRMACGLGGKLVLPMIK
EHIMQMLQNPDWKYRHAGLMALSAIGEGCHQQMEGILNEIVNFVLLFLQDPHPRVRYAAC
NAVGQMATDFAPGFQKKFHEKVIAALLQTMEDQGNQRVQAHAAAALINFTEDCPKSLLIP
YLDNLVKHLHSIMVLKLQELIQKGTKLVLEQVVTSIASVADTAEEKFVPYYDLFMPSLKH
IVENAVQKELRLLRGKTIECISLIGLAVGKEKFMQDASDVMQLLLKTQTDFNDMEDDDPQ
ISYMISAWARMCKILGKEFQQYLPVVMGPLMKTASIKPEVALLDTQDMENMSDDDGWEFV
NLGDQQSFGIKTAGLEEKSTACQMLVCYAKELKEGFVEYTEQVVKLMVPLLKFYFHDGVR
VAAAESMPLLLECARVRGPEYLTQMWHFMCDALIKAIGTEPDSDVLSEIMHSFAKCIEVM
GDGCLNNEHFEELGGILKAKLEEHFKNQELRQVKRQDEDYDEQVEESLQDEDDNDVYILT
KVSDILHSIFSSYKEKVLPWFEQLLPLIVNLICPHRPWPDRQWGLCIFDDVIEHCSPASF
KYAEYFLRPMLQYVCDNSPEVRQAAAYGLGVMAQYGGDNYRPFCTEALPLLVRVIQSADS
KTKENVNATENCISAVGKIMKFKPDCVNVEEVLPHWLSWLPLHEDKEEAVQTFNYLCDLI
ESNHPIVLGPNNTNLPKIFSIIAEGEMHEAIKHEDPCAKRLANVVRQVQTSGGLWTECIA
QLSPEQQAAIQELLNSA
>sp|P63000|RAC1_HUMAN Ras-related C3 botulinum toxin substrate 1 OS=Homo sapiens OX=9606 GN=RAC1 PE=1 SV=1
MQAIKCVVVGDGAVGKTCLLISYTTNAFPGEYIPTVFDNYSANVMVDGKPVNLGLWDTAG
QEDYDRLRPLSYPQTDVFLICFSLVSPASFENVRAKWYPEVRHHCPNTPIILVGTKLDLR
DDKDTIEKLKEKKLTPITYPQGLAMAKEIGAVKYLECSALTQRGLKTVFDEAIRAVLCPP
PVKKRKRKCLLL
>sp|O43491|E41L2_HUMAN Band 4.1-like protein 2 OS=Homo sapiens OX=9606 GN=EPB41L2 PE=1 SV=1
MTTEVGSVSEVKKDSSQLGTDATKEKPKEVAENQQNQSSDPEEEKGSQPPPAAESQSSLR
RQKREKETSESRGISRFIPPWLKKQKSYTLVVAKDGGDKKEPTQAVVEEQVLDKEEPLPE
EQRQAKGDAEEMAQKKQEIKVEVKEEKPSVSKEEKPSVSKVEMQPTELVSKEREEKVKET
QEDKLEGGAAKRETKEVQTNELKAEKASQKVTKKTKTVQCKVTLLDGTEYSCDLEKHAKG
QVLFDKVCEHLNLLEKDYFGLLFQESPEQKNWLDPAKEIKRQLRNLPWLFTFNVKFYPPD
PSQLTEDITRYFLCLQLRQDIASGRLPCSFVTHALLGSYTLQAELGDYDPEEHGSIDLSE
FQFAPTQTKELEEKVAELHKTHRGLSPAQADSQFLENAKRLSMYGVDLHHAKDSEGVDIK
LGVCANGLLIYKDRLRINRFAWPKILKISYKRSNFYIKVRPAELEQFESTIGFKLPNHRA
AKRLWKVCVEHHTFYRLVSPEQPPKAKFLTLGSKFRYSGRTQAQTRQASTLIDRPAPHFE
RTSSKRVSRSLDGAPIGVMDQSLMKDFPGAAGEISAYGPGLVSIAVVQDGDGRREVRSPT
KAPHLQLIEGKKNSLRVEGDNIYVRHSNLMLEELDKAQEDILKHQASISELKRNFMESTP
EPRPNEWEKRRITPLSLQTQGSSHETLNIVEEKKRAEVGKDERVITEEMNGKEISPGSGP
GEIRKVEPVTQKDSTSLSSESSSSSSESEEEDVGEYRPHHRVTEGTIREEQEYEEEVEEE
PRPAAKVVEREEAVPEASPVTQAGASVITVETVIQENVGAQKIPGEKSVHEGALKQDMGE

EAEEEPQKVNGEVSHVDIDVLPQIICCSEPPVVKTEMVTISDASQRTEISTKEVPIVQTE
TKTITYESPQIDGGAGGDSGTLLTAQTITSESVSTTTTTHITKTVKGGISETRIEKRIVI
TGDGDIDHDQALAQAIREAREQHPDMSVTRVVVHKETELAEEGED
>sp|P07315|CRGC_HUMAN Gamma-crystallin C OS=Homo sapiens OX=9606 GN=CRYGC PE=1 SV=2
MGKITFYEDRAFQGRSYETTTDCPNLQPYFSRCNSIRVESGCWMLYERPNYQGQQYLLRR
GEYPDYQQWMGLSDSIRSCCLIPQTVSHRLRLYEREDHKGLMMELSEDCPSIQDRFHLSE
IRSLHVLEGCWVLYELPNYRGRQYLLRPQEYRRCQDWGAMDAKAGSLRRVVDLY
>sp|P16152|CBR1_HUMAN Carbonyl reductase [NADPH] 1 OS=Homo sapiens OX=9606 GN=CBR1 PE=1 SV=3
MSSGIHVALVTGGNKGIGLAIVRDLCRLFSGDVVLTARDVTRGQAAVQQLQAEGLSPRFH
QLDIDDLQSIRALRDFLRKEYGGLDVLVNNAGIAFKVADPTPFHIQAEVTMKTNFFGTRD
VCTELLPLIKPQGRVVNVSSIMSVRALKSCSPELQQKFRSETITEEELVGLMNKFVEDTK
KGVHQKEGWPSSAYGVTKIGVTVLSRIHARKLSEQRKGDKILLNACCPGWVRTDMAGPKA
TKSPEEGAETPVYLALLPPDAEGPHGQFVSEKRVEQW
>sp|P30086|PEBP1_HUMAN Phosphatidylethanolamine-binding protein 1 OS=Homo sapiens OX=9606 GN=PEBP1 PE=1 SV=3
MPVDLSKWSGPLSLQEVDEQPQHPLHVTYAGAAVDELGKVLTPTQVKNRPTSISWDGLDS
GKLYTLVLTDPDAPSRKDPKYREWHHFLVVNMKGNDISSGTVLSDYVGSGPPKGTGLHRY
VWLVYEQDRPLKCDEPILSNRSGDHRGKFKVASFRKKYELRAPVAGTCYQAEWDDYVPKL
YEQLSGK
>sp|P35222|CTNB1_HUMAN Catenin beta-1 OS=Homo sapiens OX=9606 GN=CTNNB1 PE=1 SV=1
MATQADLMELDMAMEPDRKAAVSHWQQQSYLDSGIHSGATTTAPSLSGKGNPEEEDVDTS
QVLYEWEQGFSQSFTQEQVADIDGQYAMTRAQRVRAAMFPETLDEGMQIPSTQFDAAHPT
NVQRLAEPSQMLKHAVVNLINYQDDAELATRAIPELTKLLNDEDQVVVNKAAVMVHQLSK
KEASRHAIMRSPQMVSAIVRTMQNTNDVETARCTAGTLHNLSHHREGLLAIFKSGGIPAL
VKMLGSPVDSVLFYAITTLHNLLLHQEGAKMAVRLAGGLQKMVALLNKTNVKFLAITTDC
LQILAYGNQESKLIILASGGPQALVNIMRTYTYEKLLWTTSRVLKVLSVCSSNKPAIVEA
GGMQALGLHLTDPSQRLVQNCLWTLRNLSDAATKQEGMEGLLGTLVQLLGSDDINVVTCA
AGILSNLTCNNYKNKMMVCQVGGIEALVRTVLRAGDREDITEPAICALRHLTSRHQEAEM
AQNAVRLHYGLPVVVKLLHPPSHWPLIKATVGLIRNLALCPANHAPLREQGAIPRLVQLL
VRAHQDTQRRTSMGGTQQQFVEGVRMEEIVEGCTGALHILARDVHNRIVIRGLNTIPLFV
QLLYSPIENIQRVAAGVLCELAQDKEAAEAIEAEGATAPLTELLHSRNEGVATYAAAVLF
RMSEDKPQDYKKRLSVELTSSLFRTEPMAWNETADLGLDIGAQGEPLGYRQDDPSYRSFH
SGGYGQDALGMDPMMEHEMGGHHPGADYPVDGLPDLGHAQDLMDGLPPGDSNQLAWFDTD
L
>sp|Q8N3J6|CADM2_HUMAN Cell adhesion molecule 2 OS=Homo sapiens OX=9606 GN=CADM2 PE=2 SV=1
MIWKRSAVLRFYSVCGLLLQGSQGQFPLTQNVTVVEGGTAILTCRVDQNDNTSLQWSNPA
QQTLYFDDKKALRDNRIELVRASWHELSISVSDVSLSDEGQYTCSLFTMPVKTSKAYLTV
LGVPEKPQISGFSSPVMEGDLMQLTCKTSGSKPAADIRWFKNDKEIKDVKYLKEEDANRK
TFTVSSTLDFRVDRSDDGVAVICRVDHESLNATPQVAMQVLEIHYTPSVKIIPSTPFPQE
GQPLILTCESKGKPLPEPVLWTKDGGELPDPDRMVVSGRELNILFLNKTDNGTYRCEATN

TIGQSSAEYVLIVHDVPNTLLPTTIIPSLTTATVTTTVAITTSPTTSATTSSIRDPNALA
GQNGPDHALIGGIVAVVVFVTLCSIFLLGRYLARHKGTYLTNEAKGAEDAPDADTAIINA
EGSQVNAEEKKEYFI
>sp|P51149|RAB7A_HUMAN Ras-related protein Rab-7a OS=Homo sapiens OX=9606
GN=RAB7A PE=1 SV=1
MTSRKKVLLKVIILGDSGVGKTSLMNQYVNKKFSNQYKATIGADFLTKEVMVDDRLVTMQ
IWDTAGQERFQSLGVAFYRGADCCVLVFDVTAPNTFKTLDSWRDEFLIQASPRDPENFPF
VVLGNKIDLENRQVATKRAQAWCYSKNNIPYFETSAKEAINVEQAFQTIARNALKQETEV
ELYNEFPEPIKLDKNDRAKASAESCSC
>sp|Q53FA7|QORX_HUMAN Quinone oxidoreductase PIG3 OS=Homo sapiens OX=9606
GN=TP53I3 PE=1 SV=2
MLAVHFDKPGGPENLYVKEVAKPSPGEGEVLLKVAASALNRADLMQRQGQYDPPPGASNI
LGLEASGHVAELGPGCQGHWKIGDTAMALLPGGGQAQYVTVPEGLLMPIPEGLTLTQAAA
IPEAWLTAFQLLHLVGNVQAGDYVLIHAGLSGVGTAAIQLTRMAGAIPLVTAGSQKKLQM
AEKLGAAAGFNYKKEDFSEATLKFTKGAGVNLILDCIGGSYWEKNVNCLALDGRWVLYGL
MGGGDINGPLFSKLLFKRGSLITSLLRSRDNKYKQMLVNAFTEQILPHFSTEGPQRLLPV
LDRIYPVTEIQEAHKYMEANKNIGKIVLELPQ
>sp|Q9Y2J2|E41L3_HUMAN Band 4.1-like protein 3 OS=Homo sapiens OX=9606 GN=EPB41L3
PE=1 SV=2
MTTESGSDSESKPDQEAEPQEAAGAQGRAGAPVPEPPKEEQQQALEQFAAAAAHSTPVRR
EVTDKEQEFAARAAKQLEYQQLEDDKLSQKSSSSKLSRSPLKIVKKPKSMQCKVILLDGS
EYTCDVEKRSRGQVLFDKVCEHLNLLEKDYFGLTYRDAENQKNWLDPAKEIKKQVRSGAW
HFSFNVKFYPPDPAQLSEDITRYYLCLQLRDDIVSGRLPCSFVTLALLGSYTVQSELGDY
DPDECGSDYISEFRFAPNHTKELEDKVIELHKSHRGMTPAEAEMHFLENAKKLSMYGVDL
HHAKDSEGVEIMLGVCASGLLIYRDRLRINRFAWPKVLKISYKRNNFYIKIRPGEFEQFE
STIGFKLPNHRAAKRLWKVCVEHHTFFRLLLPEAPPKKFLTLGSKFRYSGRTQAQTRRAS
ALIDRPAPYFERSSSKRYTMSRSLDGEVGTGQYATTKGISQTNLITTVTPEKKAEEERDE
EEDKRRKGEEVTPISAIRHEGKSPGLGTDSCPLSPPSTHCAPTSPTELRRRCKENDCKLP
GYEPSRAEHLPGEPALDSDGPGRPYLGDQDVAFSYRQQTGKGTTLFSFSLQLPESFPSLL
DDDGYLSFPNLSETNLLPQSLQHYLPIRSPSLVPCFLFIFFFLLSASFSVPYALTLSFPL
ALCLCYLEPKAASLSASLDNDPSDSSEEETDSERTDTAADGETTATESDQEEDAELKAQE
LEKTQDDLMKHQTNISELKRTFLETSTDTAVTNEWEKRLSTSPVRLAARQEDAPMIEPLV
PEETKQSSGEKLMDGSEIFSLLESARKPTEFIGGVTSTSQSWVQKMETKTESSGIETEPT
VHHLPLSTEKVVQETVLVEERRVVHASGDASYSAGDSGDAAAQPAFTGIKGKEGSALTEG
AKEEGGEEVAKAVLEQEETAAASRERQEEQSAAIHISETLEQKPHFESSTVKTETISFGS
VSPGGVKLEISTKEVPVVHTETKTITYESSQVDPGTDLEPGVLMSAQTITSETTSTTTTT
HITKTVKGGISETRIEKRIVITGDADIDHDQALAQAIKEAKEQHPDMSVTKVVVHKETEI
TPEDGED
>tr|A4D2P2|A4D2P2_HUMAN Ras-related C3 botulinum toxin substrate 1 OS=Homo sapiens
OX=9606 GN=RAC1 PE=3 SV=1
MVDGKPVNLGLWDTAGQEDYDRLRPLSYPQTDVFLICFSLVSPASFENVRAKWYPEVRHH
CPNTPIILVGTKLDLRDDKDTIEKLKEKKLTPITYPQGLAMAKEIGAVKYLECSALTQRG
LKTVFDEAIRAVLCPPPVKKRKRKCLLL

>sp|P07316|CRGB_HUMAN Gamma-crystallin B OS=Homo sapiens OX=9606 GN=CRYGB PE=1 SV=3
MGKITFYEDRAFQGRSYECTTDCPNLQPYFSRCNSIRVESGCWMIYERPNYQGHQYFLRR
GEYPDYQQWMGLSDSIRSCCLIPPHSGAYRMKIYDRDELRGQMSELTDDCISVQDRFHLT
EIHSLNVLEGSWILYEMPNYRGRQYLLRPGEYRRFLDWGAPNAKVGSLRRVMDLY
>sp|P32754|HPPD_HUMAN 4-hydroxyphenylpyruvate dioxygenase OS=Homo sapiens OX=9606 GN=HPD PE=1 SV=2
MTTYSDKGAKPERGRFLHFHSVTFWVGNAKQAASFYCSKMGFEPLAYRGLETGSREVVSH
VIKQGKIVFVLSSALNPWNKEMGDHLVKHGDGVKDIAFEVEDCDYIVQKARERGAKIMRE
PWVEQDKFGKVKFAVLQTYGDTTHTLVEKMNYIGQFLPGYEAPAFMDPLLPKLPKCSLEM
IDHIVGNQPDQEMVSASEWYLKNLQFHRFWSVDDTQVHTEYSSLRSIVVANYEESIKMPI
NEPAPGKKKSQIQEYVDYNGGAGVQHIALKTEDIITAIRHLRERGLEFLSVPSTYYKQLR
EKLKTAKIKVKENIDALEELKILVDYDEKGYLLQIFTKPVQDRPTLFLEVIQRHNHQGFG
AGNFNSLFKAFEEEQNLRGNLTNMETNGVVPGM
>sp|P48163|MAOX_HUMAN NADP-dependent malic enzyme OS=Homo sapiens OX=9606 GN=ME1 PE=1 SV=1
MEPEAPRRRHTHQRGYLLTRNPHLNKDLAFTLEERQQLNIHGLLPPSFNSQEIQVLRVVK
NFEHLNSDFDRYLLLMDLQDRNEKLFYRVLTSDIEKFMPIVYTPTVGLACQQYSLVFRKP
RGLFITIHDRGHIASVLNAWPEDVIKAIVVTDGERILGLGDLGCNGMGIPVGKLALYTAC
GGMNPQECLPVILDVGTENEELLKDPLYIGLRQRRVRGSEYDDFLDEFMEAVSSKYGMNC
LIQFEDFANVNAFRLLNKYRNQYCTFNDDIQGTASVAVAGLLAALRITKNKLSDQTILFQ
GAGEAALGIAHLIVMALEKEGLPKEKAIKKIWLVDSKGLIVKGRASLTQEKEKFAHEHEE
MKNLEAIVQEIKPTALIGVAAIGGAFSEQILKDMAAFNERPIIFALSNPTSKAECSAEQC
YKITKGRAIFASGSPFDPVTLPNGQTLYPGQGNNSYVFPGVALGVVACGLRQITDNIFLT
TAEVIAQQVSDKHLEEGRLYPPLNTIRDVSLKIAEKIVKDAYQEKTATVYPEPQNKEAFV
RSQMYSTDYDQILPDCYSWPEEVQKIQTKVDQ
>sp|P29401|TKT_HUMAN Transketolase OS=Homo sapiens OX=9606 GN=TKT PE=1 SV=3
MESYHKPDQQKLQALKDTANRLRISSIQATTAAGSGHPTSCCSAAEIMAVLFFHTMRYKS
QDPRNPHNDRFVLSKGHAAPILYAVWAEAGFLAEAELLNLRKISSDLDGHPVPKQAFTDV
ATGSLGQGLGAACGMAYTGKYFDKASYRVYCLLGDGELSEGSVWEAMAFASIYKLDNLVA
ILDINRLGQSDPAPLQHQMDIYQKRCEAFGWHAIIVDGHSVEELCKAFGQAKHQPTAIIA
KTFKGRGITGVEDKESWHGKPLPKNMAEQIIQEIYSQIQSKKKILATPPQEDAPSVDIAN
IRMPSLPSYKVGDKIATRKAYGQALAKLGHASDRIIALDGDTKNSTFSEIFKKEHPDRFI
ECYIAEQNMVSIAVGCATRNRTVPFCSTFAAFFTRAFDQIRMAAISESNINLCGSHCGVS
IGEDGPSQMALEDLAMFRSVPTSTVFYPSDGVATEKAVELAANTKGICFIRTSRPENAII
YNNNEDFQVGQAKVVLKSKDDQVTVIGAGVTLHEALAAAELLKKEKINIRVLDPFTIKPL
DRKLILDSARATKGRILTVEDHYYEGGIGEAVSSAVVGEPGITVTHLAVNRVPRSGKPAE
LLKMFGIDRDAIAQAVRGLITKA
>sp|P55344|LMIP_HUMAN Lens fiber membrane intrinsic protein OS=Homo sapiens OX=9606 GN=LIM2 PE=1 SV=2
MYSFMGGGLFCAWVGTILLVVAMATDHWMQYRLSGSFAHQGLWRYCLGNKCYLQTDSIAY
WNATRAFMILSALCAISGIIMGIMAFAHQPTFSRISRPFSAGIMFFSSTLFVVLALAIYT
GVTVSFLGRRFGDWRFSWSYILGWVAVLMTFFAGIFYMCAYRVHECRRLSTPR

>sp|P00387|NB5R3_HUMAN NADH-cytochrome b5 reductase 3 OS=Homo sapiens OX=9606 GN=CYB5R3 PE=1 SV=3
MGAQLSTLGHMVLFPVWFLYSLLMKLFQRSTPAITLESPDIKYPLRLIDREIISHDTRRF
RFALPSPQHILGLPVGQHIYLSARIDGNLVVRPYTPISSDDDKGFVDLVIKVYFKDTHPK
FPAGGKMSQYLESMQIGDTIEFRGPSGLLVYQGKGKFAIRPDKKSNPIIRTVKSVGMIAG
GTGITPMLQVIRAIMKDPDDHTVCHLLFANQTEKDILLRPELEELRNKHSARFKLWYTLD
RAPEAWDYGQGFVNEEMIRDHLPPPEEEPLVLMCGPPPMIQYACLPNLDHVGHPTERCFV
F
>sp|Q15413|RYR3_HUMAN Ryanodine receptor 3 OS=Homo sapiens OX=9606 GN=RYR3 PE=1 SV=3
MAEGGEGGEDEIQFLRTEDEVVLQCIATIHKEQRKFCLAAEGLGNRLCFLEPTSEAKYIP
PDLCVCNFVLEQSLSVRALQEMLANTGENGGEGAAQGGGHRTLLYGHAVLLRHSFSGMYL
TCLTTSRSQTDKLAFDVGLREHATGEACWWTIHPASKQRSEGEKVRIGDDLILVSVSSER
YLHLSVSNGNIQVDASFMQTLWNVHPTCSGSSIEEGYLLGGHVVRLFHGHDECLTIPSTD
QNDSQHRRIFYEAGGAGTRARSLWRVEPLRISWSGSNIRWGQAFRLRHLTTGHYLALTED
QGLILQDRAKSDTKSTAFSFRASKELKEKLDSSHKRDIEGMGVPEIKYGDSVCFVQHIAS
GLWVTYKAQDAKTSRLGPLKRKVILHQEGHMDDGLTLQRCQREESQAARIIRNTTALFSQ
FVSGNNRTAAPITLPIEEVLQTLQDLIAYFQPPEEEMRHEDKQNKLRSLKNRQNLFKEEG
MLALVLNCIDRLNVYNSVAHFAGIAREESGMAWKEILNLLYKLLAALIRGNRNNCAQFSN
NLDWLISKLDRLESSSGILEVLHCILTESPEALNLIAEGHIKSIISLLDKHGRNHKVLDI
LCSLCLCNGVAVRANQNLICDNLLPRRNLLLQTRLINDVTSIRPNIFLGVAEGSAQYKKW
YFELIIDQVDPFLTAEPTHLRVGWASSSGYAPYPGGGEGWGGNGVGDDLYSYGFDGLHLW
SGRIPRAVASINQHLLRSDDVVSCCLDLGVPSISFRINGQPVQGMFENFNTDGLFFPVMS
FSAGVKVRFLMGGRHGEFKFLPPSGYAPCYEALLPKEKMRLEPVKEYKRDADGIRDLLGT
TQFLSQASFIPCPVDTSQVILPPHLEKIRDRLAENIHELWGMNKIELGWTFGKIRDDNKR
QHPCLVEFSKLPETEKNYNLQMSTETLKTLLALGCHIAHVNPAAEEDLKKVKLPKNYMMS
NGYKPAPLDLSDVKLLPPQEILVDKLAENAHNVWAKDRIKQGWTYGIQQDLKNKRNPRLV
PYALLDERTKKSNRDSLREAVRTFVGYGYNIEPSDQELADSAVEKVSIDKIRFFRVERSY
AVRSGKWYFEFEVVTGGDMRVGWARPGCRPDVELGADDQAFVFEGNRGQRWHQGSGYFGR
TWQPGDVVGCMINLDDASMIFTLNGELLITNKGSELAFADYEIENGFVPICCLGLSQIGR
MNLGTDASTFKFYTMCGLQEGFEPFAVNMNRDVAMWFSKRLPTFVNVPKDHPHIEVMRID
GTMDSPPCLKVTHKTFGTQNSNADMIYCRLSMPVECHSSFSHSPCLDSEAFQKRKQMQEI
LSHTTTQCYYAIRIFAGQDPSCVWVGWVTPDYHLYSEKFDLNKNCTVTVTLGDERGRVHE
SVKRSNCYMVWGGDIVASSQRSNRSNVDLEIGCLVDLAMGMLSFSANGKELGTCYQVEPN
TKVFPAVFLQPTSTSLFQFELGKLKNAMPLSAAIFRSEEKNPVPQCPPRLDVQTIQPVLW
SRMPNSFLKVETERVSERHGWVVQCLEPLQMMALHIPEENRCVDILELCEQEDLMRFHYH
TLRLYSAVCALGNSRVAYALCSHVDLSQLFYAIDNKYLPGLLRSGFYDLLISIHLASAKE
RKLMMKNEYIIPITSTTRNIRLFPDESKRHGLPGVGLRTCLKPGFRFSTPCFVVTGEDHQ
KQSPEIPLESLRTKALSMLTEAVQCSGAHIRDPVGGSVEFQFVPVLKLIGTLLVMGVFDD
DDVRQILLLIDPSVFGEHSAGTEEGAEKEEVTQVEEKAVEAGEKAGKEAPVKGLLQTRLP
ESVKLQMCELLSYLCDCELQHRVEAIVAFGDIYVSKLQANQKFRYNELMQALNMSAALTA
RKTKEFRSPPQEQINMLLNFQLGENCPCPEEIREELYDFHEDLLLHCGVPLEEEEEEEED
TSWTGKLCALVYKIKGPPKPEKEQPTEEEERCPTTLKELISQTMICWAQEDQIQDSELVR
MMFNLLRRQYDSIGELLQALRKTYTISHTSVSDTINLLAALGQIRSLLSVRMGKEEELLM

INGLGDIMNNKVFYQHPNLMRVLGMHETVMEVMVNVLGTEKSQIAFPKMVASCCRFLCYF
CRISRQNQKAMFEHLSYLLENSSVGLASPSMRGSTPLDVAASSVMDNNELALSLEEPDLE
KVVTYLAGCGLQSCPMLLAKGYPDVGWNPIEGERYLSFLRFAVFVNSESVEENASVVVKL
LIRRPECFGPALRGEGGNGLLAAMQGAIKISENPALDLPSQGYKREVSTGDDEEEEEIVH
MGNAIMSFYSALIDLLGRCAPEMHLIQTGKGEAIRIRSILRSLVPTEDLVGIISIPLKLP
SLNKDGSVSEPDMAANFCPDHKAPMVLFLDRVYGIKDQTFLLHLLEVGFLPDLRASASLD
TVSLSTTEAALALNRYICSAVLPLLTRCAPLFAGTEHCTSLIDSTLQTIYRLSKGRSLTK
AQRDTIEECLLAICNHLRPSMLQQLLRRLVFDVPQLNEYCKMPLKLLTNHYEQCWKYYCL
PSGWGSYGLAVEEELHLTEKLFWGIFDSLSHKKYDPDLFRMALPCLSAIAGALPPDYLDT
RITATLEKQISVDADGNFDPKPINTMNFSLPEKLEYIVTKYAEHSHDKWACDKSQSGWKY
GISLDENVKTHPLIRPFKTLTEKEKEIYRWPARESLKTMLAVGWTVERTKEGEALVQQRE
NEKLRSVSQANQGNSYSPAPLDLSNVVLSRELQGMVEVVAENYHNIWAKKKKLELESKGG
GSHPLLVPYDTLTAKEKFKDREKAQDLFKFLQVNGIIVSRGMKDMELDASSMEKRFAYKF
LKKILKYVDSAQEFIAHLEAIVSSGKTEKSPRDQEIKFFAKVLLPLVDQYFTSHCLYFLS
SPLKPLSSSGYASHKEKEMVAGLFCKLAALVRHRISLFGSDSTTMVSCLHILAQTLDTRT
VMKSGSELVKAGLRAFFENAAEDLEKTSENLKLGKFTHSRTQIKGVSQNINYTTVALLPI
LTSIFEHVTQHQFGMDLLLGDVQISCYHILCSLYSLGTGKNIYVERQRPALGECLASLAA
AIPVAFLEPTLNRYNPLSVFNTKTPRERSILGMPDTVEDMCPDIPQLEGLMKEINDLAES
GARYTEMPHVIEVILPMLCNYLSYWWERGPENLPPSTGPCCTKVTSEHLSLILGNILKII
NNNLGIDEASWMKRIAVYAQPIISKARPDLLRSHFIPTLEKLKKKAVKTVQEEEQLKADG
KGDTQEAELLILDEFAVLCRDLYAFYPMLIRYVDNNRSNWLKSPDADSDQLFRMVAEVFI
LWCKSHNFKREEQNFVIQNEINNLAFLTGDSKSKMSKAMQVKSGGQDQERKKTKRRGDLY
SIQTSLIVAALKKMLPIGLNMCTPGDQELISLAKSRYSHRDTDEEVREHLRNNLHLQEKS
DDPAVKWQLNLYKDVLKSEEPFNPEKTVERVQRISAAVFHLEQVEQPLRSKKAVWHKLLS
KQRKRAVVACFRMAPLYNLPRHRSINLFLHGYQRFWIETEEYSFEEKLVQDLAKSPKVEE
EEEEETEKQPDPLHQIILYFSRNALTERSKLEDDPLYTSYSSMMAKSCQSGEDEEEDEDK
EKTFEEKEMEKQKTLYQQARLHERGAAEMVLQMISASKGEMSPMVVETLKLGIAILNGGN
AGVQQKMLDYLKEKKDAGFFQSLSGLMQSCSVLDLNAFERQNKAEGLGMVTEEGTLIVRE
RGEKVLQNDEFTRDLFRFLQLLCEGHNSDFQNFLRTQMGNTTTVNVIISTVDYLLRLQES
ISDFYWYYSGKDIIDESGQHNFSKALAVTKQIFNSLTEYIQGPCIGNQQSLAHSRLWDAV
VGFLHVFANMQMKLSQDSSQIELLKELLDLLQDMVVMLLSLLEGNVVNGTIGKQMVDTLV
ESSTNVEMILKFFDMFLKLKDLTSSDTFKEYDPDGKGIISKKEFQKAMEGQKQYTQSEID
FLLSCAEADENDMFNYVDFVDRFHEPAKDIGFNVAVLLTNLSEHMPNDSRLKCLLDPAES
VLNYFEPYLGRIEIMGGAKKIERVYFEISESSRTQWEKPQVKESKRQFIFDVVNEGGEQE
KMELFVNFCEDTIFEMQLASQISESDSADRPEEEEEDEDSSYVLEIAGEEEEDGSLEPAS
AFAMACASVKRNVTDFLKRATLKNLRKQYRNVKKMTAKELVKVLFSFFWMLFVGLFQLLF
TILGGIFQILWSTVFGGGLVEGAKNIRVTKILGDMPDPTQFGIHDDTMEAERAEVMEPGI
TTELVHFIKGEKGDTDIMSDLFGLHPKKEGSLKHGPEVGLGDLSEIIGKDEPPTLESTVQ
KKRKAQAAEMKAANEAEGKVESEKADMEDGEKEDKDKEEEQAEYLWTEVTKKKKRRCGQK
VEKPEAFTANFFKGLEIYQTKLLHYLARNFYNLRFLALFVAFAINFILLFYKVTEEPLEE
ETEDVANLWNSFNDEEEEEAMVFFVLQESTGYMAPTLRALAIIHTIISLVCVVGYYCLKV
PLVVFKREKEIARKLEFDGLYITEQPSEDDIKGQWDRLVINTPSFPNNYWDKFVKRKVIN
KYGDLYGAERIAELLGLDKNALDFSPVEETKAEAASLVSWLSSIDMKYHIWKLGVVFTDN
SFLYLAWYTTMSVLGHYNNFFFAAHLLDIAMGFKTLRTILSSVTHNGKQLVLTVGLLAVV

VYLYTVVAFNFFRKFYNKSEDDDEPDMKCDDMMTCYLFHMYVGVRAGGGIGDEIEDPAGD
PYEMYRIVFDITFFFFVIVILLAIIQGLIIDAFGELRDQQEQVREDMETKCFICGIGNDY
FDTTPHGFETHTLQEHNLANYLFFLMYLINKDETEHTGQESYVWKMYQERCWDFFPAGDC
FRKQYEDQLG
>sp|P20340|RAB6A_HUMAN Ras-related protein Rab-6A OS=Homo sapiens OX=9606
GN=RAB6A PE=1 SV=3
MSTGGDFGNPLRKFKLVFLGEQSVGKTSLITRFMYDSFDNTYQATIGIDFLSKTMYLEDR
TVRLQLWDTAGQERFRSLIPSYIRDSTVAVVVYDITNVNSFQQTTKWIDDVRTERGSDVI
IMLVGNKTDLADKRQVSIEEGERKAKELNVMFIETSAKAGYNVKQLFRRVAAALPGMEST
QDRSREDMIDIKLEKPQEQPVSEGGCSC
>sp|P61586|RHOA_HUMAN Transforming protein RhoA OS=Homo sapiens OX=9606 GN=RHOA
PE=1 SV=1
MAAIRKKLVIVGDGACGKTCLLIVFSKDQFPEVYVPTVFENYVADIEVDGKQVELALWDT
AGQEDYDRLRPLSYPDTDVILMCFSIDSPDSLENIPEKWTPEVKHFCPNVPIILVGNKKD
LRNDEHTRRELAKMKQEPVKPEEGRDMANRIGAFGYMECSAKTKDGVREVFEMATRAALQ
ARRGKKKSGCLVL
>sp|P62745|RHOB_HUMAN Rho-related GTP-binding protein RhoB OS=Homo sapiens OX=9606
GN=RHOB PE=1 SV=1
MAAIRKKLVVVGDGACGKTCLLIVFSKDEFPEVYVPTVFENYVADIEVDGKQVELALWDT
AGQEDYDRLRPLSYPDTDVILMCFSVDSPDSLENIPEKWVPEVKHFCPNVPIILVANKKD
LRSDEHVRTELARMKQEPVRTDDGRAMAVRIQAYDYLECSAKTKEGVREVFETATRAALQ
KRYGSQNGCINCCKVL
>sp|Q71U36|TBA1A_HUMAN Tubulin alpha-1A chain OS=Homo sapiens OX=9606 GN=TUBA1A
PE=1 SV=1
MRECISIHVGQAGVQIGNACWELYCLEHGIQPDGQMPSDKTIGGGDDSFNTFFSETGAGK
HVPRAVFVDLEPTVIDEVRTGTYRQLFHPEQLITGKEDAANNYARGHYTIGKEIIDLVLD
RIRKLADQCTGLQGFLVFHSFGGGTGSGFTSLLMERLSVDYGKKSKLEFSIYPAPQVSTA
VVEPYNSILTTHTTLEHSDCAFMVDNEAIYDICRRNLDIERPTYTNLNRLIGQIVSSITA
SLRFDGALNVDLTEFQTNLVPYPRIHFPLATYAPVISAEKAYHEQLSVAEITNACFEPAN
QMVKCDPRHGKYMACCLLYRGDVVPKDVNAAIATIKTKRTIQFVDWCPTGFKVGINYQPP
TVVPGGDLAKVQRAVCMLSNTTAIAEAWARLDHKFDLMYAKRAFVHWYVGEGMEEGEFSE
AREDMAALEKDYEEVGVDSVEGEGEEEGEEY
>tr|Q6FGX3|Q6FGX3_HUMAN RAB6A protein OS=Homo sapiens OX=9606 GN=RAB6A PE=2
SV=1
MSTGGDFGNPLRKFKLVFLGEQSVGKTSLITRFMYDSFDNTYQATIGIDFLSKTMYLEDR
TIRLQLWDTAGQERFRSLIPSYIRDSAAAVVVYDITNVNSFQQTTKWIDDVRTERGSDVI
IMLVGNKTDLADKRQVSIEEGERKAKELNVMFIETSAKAGYDVKQLFRRVAAALPGMEST
QDRSREDMIDIKLEKPQEQPVSEGGCSC
>sp|P13645|K1C10_HUMAN Keratin, type I cytoskeletal 10 OS=Homo sapiens OX=9606
GN=KRT10 PE=1 SV=6
MSVRYSSSKHYSSSRSGGGGGGGGCGGGGGVSSLRISSSKGSLGGGFSSGGFSGGSFSRG
SSGGGCFGGSSGGYGGLGGFGGGSFRGSYGSSSFGGSYGGIFGGGSFGGGSFGGGSFGGG
GFGGGGFGGGFGGGFGGDGGLLSGNEKVTMQNLNDRLASYLDKVRALEESNYELEGKIKE
WYEKHGNSHQGEPRDYSKYYKTIDDLKNQILNLTTDNANILLQIDNARLAADDFRLKYEN

EVALRQSVEADINGLRRVLDELTLTKADLEMQIESLTEELAYLKKNHEEEMKDLRNVSTG
DVNVEMNAAPGVDLTQLLNNMRSQYEQLAEQNRKDAEAWFNEKSKELTTEIDNNIEQISS
YKSEITELRRNVQALEIELQSQLALKQSLEASLAETEGRYCVQLSQIQAQISALEEQLQQ
IRAETECQNTEYQQLLDIKIRLENEIQTYRSLLEGEGSSGGGGRGGGSFGGGYGGGSSGG
GSSGGGHGGGHGGSSGGGYGGGSSGGGSSGGGYGGGSSSGGHGGSSSGGYGGGSSGGGGG
GYGGGSSGGGSSSGGGYGGGSSSGGHKSSSSGSVGESSSKGPRY
>sp|P11171|EPB41_HUMAN Protein 4.1 OS=Homo sapiens OX=9606 GN=EPB41 PE=1 SV=4
MTTEKSLVTEAENSQHQQKEEGEEAINSGQQEPQQEESCQTAAEGDNWCEQKLKASNGDT
PTHEDLTKNKERTSESRGLSRLFSSFLKRPKSQVSEEEGKEVESDKEKGEGGQKEIEFGT
SLDEEIILKAPIAAPEPELKTDPSLDLHSLSSAETQPAQEELREDPDFEIKEGEGLEECS
KIEVKEESPQSKAETELKASQKPIRKHRNMHCKVSLLDDTVYECVVEKHAKGQDLLKRVC
EHLNLLEEDYFGLAIWDNATSKTWLDSAKEIKKQVRGVPWNFTFNVKFYPPDPAQLTEDI
TRYYLCLQLRQDIVAGRLPCSFATLALLGSYTIQSELGDYDPELHGVDYVSDFKLAPNQT
KELEEKVMELHKSYRSMTPAQADLEFLENAKKLSMYGVDLHKAKDLEGVDIILGVCSSGL
LVYKDKLRINRFPWPKVLKISYKRSSFFIKIRPGEQEQYESTIGFKLPSYRAAKKLWKVC
VEHHTFFRLTSTDTIPKSKFLALGSKFRYSGRTQAQTRQASALIDRPAPHFERTASKRAS
RSLDGAAAVDSADRSPRPTSAPAITQGQVAEGGVLDASAKKTVVPKAQKETVKAEVKKED
EPPEQAEPEPTEAWKVEKTHIEVTVPTSNGDQTQKLAEKTEDLIRMRKKKRERLDGENIY
IRHSNLMLEDLDKSQEEIKKHHASISELKKNFMESVPEPRPSEWDKRLSTHSPFRTLNIN
GQIPTGEGPPLVKTQTVTISDNANAVKSEIPTKDVPIVHTETKTITYEAAQTDDNSGDLD
PGVLLTAQTITSETPSSTTTTQITKTVKGGISETRIEKRIVITGDADIDHDQVLVQAIKE
AKEQHPDMSVTKVVVHQETEIADE
>sp|P13591|NCAM1_HUMAN Neural cell adhesion molecule 1 OS=Homo sapiens OX=9606
GN=NCAM1 PE=1 SV=3
MLQTKDLIWTLFFLGTAVSLQVDIVPSQGEISVGESKFFLCQVAGDAKDKDISWFSPNGE
KLTPNQQRISVVWNDDSSSTLTIYNANIDDAGIYKCVVTGEDGSESEATVNVKIFQKLMF
KNAPTPQEFREGEDAVIVCDVVSSLPPTIIWKHKGRDVILKKDVRFIVLSNNYLQIRGIK
KTDEGTYRCEGRILARGEINFKDIQVIVNVPPTIQARQNIVNATANLGQSVTLVCDAEGF
PEPTMSWTKDGEQIEQEEDDEKYIFSDDSSQLTIKKVDKNDEAEYICIAENKAGEQDATI
HLKVFAKPKITYVENQTAMELEEQVTLTCEASGDPIPSITWRTSTRNISSEEKASWTRPE
KQETLDGHMVVRSHARVSSLTLKSIQYTDAGEYICTASNTIGQDSQSMYLEVQYAPKLQG
PVAVYTWEGNQVNITCEVFAYPSATISWFRDGQLLPSSNYSNIKIYNTPSASYLEVTPDS
ENDFGNYNCTAVNRIGQESLEFILVQADTPSSPSIDQVEPYSSTAQVQFDEPEATGGVPI
LKYKAEWRAVGEEVWHSKWYDAKEASMEGIVTIVGLKPETTYAVRLAALNGKGLGEISAA
SEFKTQPVQGEPSAPKLEGQMGEDGNSIKVNLIKQDDGGSPIRHYLVRYRALSSEWKPEI
RLPSGSDHVMLKSLDWNAEYEVYVVAENQQGKSKAAHFVFRTSAQPTAIPANGSPTSGLS
TGAIVGILIVIFVLLLVVVDITCYFLNKCGLFMCIAVNLCGKAGPGAKGKDMEEGKAAFS
KDESKEPIVEVRTEEERTPNHDGGKHTEPNETTPLTEPEKGPVEAKPECQETETKPAPAE
VKTVPNDATQTKENESKA
>sp|Q99536|VAT1_HUMAN Synaptic vesicle membrane protein VAT-1 homolog OS=Homo
sapiens OX=9606 GN=VAT1 PE=1 SV=2
MSDEREVAEAATGEDASSPPPKTEAASDPQHPAASEGAAAAAASPPLLRCLVLTGFGGYD
KVKLQSRPAAPPAPGPGQLTLRLRACGLNFADLMARQGLYDRLPPLPVTPGMEGAGVVIA
VGEGVSDRKAGDRVMVLNRSGMWQEEVTVPSVQTFLIPEAMTFEEAAALLVNYITAYMVL

FDFGNLQPGHSVLVHMAAGGVGMAAVQLCRTVENVTVFGTASASKHEALKENGVTHPIDY
HTTDYVDEIKKISPKGVDIVMDPLGGSDTAKGYNLLKPMGKVVTYGMANLLTGPKRNLMA
LARTWWNQFSVTALQLLQANRAVCGFHLGYLDGEVELVSGVVARLLALYNQGHIKPHIDS
VWPFEKVADAMKQMQEKKNVGKVLLVPGPEKEN
>sp|P09936|UCHL1_HUMAN Ubiquitin carboxyl-terminal hydrolase isozyme L1 OS=Homo sapiens OX=9606 GN=UCHL1 PE=1 SV=2
MQLKPMEINPEMLNKVLSRLGVAGQWRFVDVLGLEEESLGSVPAPACALLLLFPLTAQHE
NFRKKQIEELKGQEVSPKVYFMKQTIGNSCGTIGLIHAVANNQDKLGFEDGSVLKQFLSE
TEKMSPEDRAKCFEKNEAIQAAHDAVAQEGQCRVDDKVNFHFILFNNVDGHLYELDGRMP
FPVNHGASSEDTLLKDAAKVCREFTEREQGEVRFSAVALCKAA
>sp|Q99497|PARK7_HUMAN Parkinson disease protein 7 OS=Homo sapiens OX=9606 GN=PARK7 PE=1 SV=2
MASKRALVILAKGAEEMETVIPVDVMRRAGIKVTVAGLAGKDPVQCSRDVVICPDASLED
AKKEGPYDVVVLPGGNLGAQNLSESAAVKEILKEQENRKGLIAAICAGPTALLAHEIGFG
SKVTTHPLAKDKMMNGGHYTYSENRVEKDGLILTSRGPGTSFEFALAIVEALNGKEVAAQ
VKAPLVLKD
>sp|Q9HCJ1|ANKH_HUMAN Mineralization regulator ANKH OS=Homo sapiens OX=9606 GN=ANKH PE=1 SV=2
MVKFPALTHYWPLIRFLVPLGITNIAIDFGEQALNRGIAAVKEDAVEMLASYGLAYSLMK
FFTGPMSDFKNVGLVFVNSKRDRTKAVLCMVVAGAIAAVFHTLIAYSDLGYYIINKLHHV
DESVGSKTRRAFLYLAAFPFMDAMAWTHAGILLKHKYSFLVGCASISDVIAQVVFVAILL
HSHLECREPLLIPILSLYMGALVRCTTLCLGYYKNIHDIIPDRSGPELGGDATIRKMLSF
WWPLALILATQRISRPIVNLFVSRDLGGSSAATEAVAILTATYPVGHMPYGWLTEIRAVY
PAFDKNNPSNKLVSTSNTVTAAHIKKFTFVCMALSLTLCFVMFWTPNVSEKILIDIIGVD
FAFAELCVVPLRIFSFFPVPVTVRAHLTGWLMTLKKTFVLAPSSVLRIIVLIASLVVLPY
LGVHGATLGVGSLLAGFVGESTMVAIAACYVYRKQKKKMENESATEGEDSAMTDMPPTEE
VTDIVEMREENE
>sp|P31150|GDIA_HUMAN Rab GDP dissociation inhibitor alpha OS=Homo sapiens OX=9606 GN=GDI1 PE=1 SV=2
MDEEYDVIVLGTGLTECILSGIMSVNGKKVLHMDRNPYYGGESSSITPLEELYKRFQLLE
GPPESMGRGRDWNVDLIPKFLMANGQLVKMLLYTEVTRYLDFKVVEGSFVYKGGKIYKVP
STETEALASNLMGMFEKRRFRKFLVFVANFDENDPKTFEGVDPQTTSMRDVYRKFDLGQD
VIDFTGHALALYRTDDYLDQPCLETVNRIKLYSESLARYGKSPYLYPLYGLGELPQGFAR
LSAIYGGTYMLNKPVDDIIMENGKVVGVKSEGEVARCKQLICDPSYIPDRVRKAGQVIRI
ICILSHPIKNTNDANSCQIIIPQNQVNRKSDIYVCMISYAHNVAAQGKYIAIASTTVETT
DPEKEVEPALELLEPIDQKFVAISDLYEPIDDGCESQVFCSCSYDATTHFETTCNDIKDI
YKRMAGTAFDFENMKRKQNDVFGEAEQ
>sp|P35908|K22E_HUMAN Keratin, type II cytoskeletal 2 epidermal OS=Homo sapiens OX=9606 GN=KRT2 PE=1 SV=2
MSCQISCKSRGRGGGGGGFRGFSSGSAVVSGGSRRSTSSFSCLSRHGGGGGGFGGGGFGS
RSLVGLGGTKSISISVAGGGGGFGAAGGFGGRGGGFGGGSSFGGGSGFSGGGFGGGGFGG
GRFGGFGGPGGVGGLGGPGGFGPGGYPGGIHEVSVNQSLLQPLNVKVDPEIQNVKAQERE
QIKTLNNKFASFIDKVRFLEQQNQVLQTKWELLQQMNVGTRPINLEPIFQGYIDSLKRYL
DGLTAERTSQNSELNNMQDLVEDYKKKYEDEINKRTAAENDFVTLKKDVDNAYMIKVELQ

SKVDLLNQEIEFLKVLYDAEISQIHQSVTDTNVILSMDNSRNLDLDSIIAEVKAQYEEIA
QRSKEEAEALYHSKYEELQVTVGRHGDSLKEIKIEISELNRVIQRLQGEIAHVKKQCKNV
QDAIADAEQRGEHALKDARNKLNDLEEALQQAKEDLARLLRDYQELMNVKLALDVEIATY
RKLLEGEECRMSGDLSSNVTVSVTSSTISSNVASKAAFGGSGGRGSSSGGGYSSGSSSYG
SGGRQSGSRGGSGGGGSISGGGYGSGGGSGGRYGSGGGSKGGSISGGGYGSGGGKHSSGG
GSRGGSSSGGGYGSGGGGSSSVKGSSGEAFGSSVTFSFR
>sp|P50395|GDIB_HUMAN Rab GDP dissociation inhibitor beta OS=Homo sapiens OX=9606
GN=GDI2 PE=1 SV=2
MNEEYDVIVLGTGLTECILSGIMSVNGKKVLHMDRNPYYGGESASITPLEDLYKRFKIPG
SPPESMGRGRDWNVDLIPKFLMANGQLVKMLLYTEVTRYLDFKVTEGSFVYKGGKIYKVP
STEAEALASSLMGLFEKRRFRKFLVYVANFDEKDPRTFEGIDPKKTTMRDVYKKFDLGQD
VIDFTGHALALYRTDDYLDQPCYETINRIKLYSESLARYGKSPYLYPLYGLGELPQGFAR
LSAIYGGTYMLNKPIEEIIVQNGKVIGVKSEGEIARCKQLICDPSYVKDRVEKVGQVIRV
ICILSHPIKNTNDANSCQIIIPQNQVNRKSDIYVCMISFAHNVAAQGKYIAIVSTTVETK
EPEKEIRPALELLEPIEQKFVSISDLLVPKDLGTESQIFISRTYDATTHFETTCDDIKNI
YKRMTGSEFDFEEMKRKKNDIYGED
>sp|P61020|RAB5B_HUMAN Ras-related protein Rab-5B OS=Homo sapiens OX=9606
GN=RAB5B PE=1 SV=1
MTSRSTARPNGQPQASKICQFKLVLLGESAVGKSSLVLRFVKGQFHEYQESTIGAAFLTQ
SVCLDDTTVKFEIWDTAGQERYHSLAPMYYRGAQAAIVVYDITNQETFARAKTWVKELQR
QASPSIVIALAGNKADLANKRMVEYEEAQAYADDNSLLFMETSAKTAMNVNDLFLAIAKK
LPKSEPQNLGGAAGRSRGVDLHEQSQQNKSQCCSN
>tr|Q14936|Q14936_HUMAN Interferon-gamma receptor alpha chain OS=Homo sapiens
OX=9606 PE=3 SV=1
MALLFLLPLVMQGVSRAEMGTADLGPSSVPTPTNVTIESYNMNPIVYWEYQIMPQVPVFT
VEVKNYGVKNSEWIDACINISHHYCNISDHVGDPSNSLWVRVKARVGQKESAYAKSEEFA
VCRDGKIGPPKLDIRKEEKQIMIDIFHPSVFVNGDEQEVDYDPETTCYIRVYNVYVRMNG
SEIQYKILTQKEDDCDEIQCQLAIPVSSLNSQYCSAEGVLHVWGVTTEKSKEVCITIFNS
SIKGSLWIPVVAALVLSLVFICFYIKKINPLKEKSIILPKSLISVVRSATLETKPESKYV
SLITSYQPFSLEKEVVCEEPLSPATVPGMHTEDNPGKVEHTEELSSITEVVTTEENIPDV
VPGSHLTPIERESSSPLSSNQSEPGSIALNSYHSRNCSESDHSRNGFDTDSSCLESHSSL
SDSEFPPNNKGEIKTEGQELITVIKAPTSFGYDKPHVLVDLLVDDSGKESLIGYRPTEDS
KEFS
>sp|P0CG48|UBC_HUMAN Polyubiquitin-C OS=Homo sapiens OX=9606 GN=UBC PE=1 SV=3
MQIFVKTLTGKTITLEVEPSDTIENVKAKIQDKEGIPPDQQRLIFAGKQLEDGRTLSDYN
IQKESTLHLVLRLRGGMQIFVKTLTGKTITLEVEPSDTIENVKAKIQDKEGIPPDQQRLI
FAGKQLEDGRTLSDYNIQKESTLHLVLRLRGGMQIFVKTLTGKTITLEVEPSDTIENVKA
KIQDKEGIPPDQQRLIFAGKQLEDGRTLSDYNIQKESTLHLVLRLRGGMQIFVKTLTGKT
ITLEVEPSDTIENVKAKIQDKEGIPPDQQRLIFAGKQLEDGRTLSDYNIQKESTLHLVLR
LRGGMQIFVKTLTGKTITLEVEPSDTIENVKAKIQDKEGIPPDQQRLIFAGKQLEDGRTL
SDYNIQKESTLHLVLRLRGGMQIFVKTLTGKTITLEVEPSDTIENVKAKIQDKEGIPPDQ
QRLIFAGKQLEDGRTLSDYNIQKESTLHLVLRLRGGMQIFVKTLTGKTITLEVEPSDTIE
NVKAKIQDKEGIPPDQQRLIFAGKQLEDGRTLSDYNIQKESTLHLVLRLRGGMQIFVKTL
TGKTITLEVEPSDTIENVKAKIQDKEGIPPDQQRLIFAGKQLEDGRTLSDYNIQKESTLH

LVLRLRGGMQIFVKTLTGKTITLEVEPSDTIENVKAKIQDKEGIPPDQQRLIFAGKQLED
GRTLSDYNIQKESTLHLVLRLRGGV
>sp|A2RU48|SMCO3_HUMAN Single-pass membrane and coiled-coil domain-containing protein 3 OS=Homo sapiens OX=9606 GN=SMCO3 PE=1 SV=1
MAQSDFLYPENPKRREEVNRLHQQLLDCLSDSFDVTNKLTEVLNMHLGCRLASIEMKRDG
TIKENCDLIIQAIMKIQKELQKVDEALKDKLEPTLYRKLQDIKEKETDKIAIVQKVISVI
LGEATSAASAVAVKLVGSNVTTGIINKLVTVLAQIGASLLGSIGVAVLGLGIDMIVRAIL
GAVEKTQLQAAIKSYEKHLVEFKSASEKYNHAITEVINTVKHQMK
>sp|P68363|TBA1B_HUMAN Tubulin alpha-1B chain OS=Homo sapiens OX=9606 GN=TUBA1B PE=1 SV=1
MRECISIHVGQAGVQIGNACWELYCLEHGIQPDGQMPSDKTIGGGDDSFNTFFSETGAGK
HVPRAVFVDLEPTVIDEVRTGTYRQLFHPEQLITGKEDAANNYARGHYTIGKEIIDLVLD
RIRKLADQCTGLQGFLVFHSFGGGTGSGFTSLLMERLSVDYGKKSKLEFSIYPAPQVSTA
VVEPYNSILTTHTTLEHSDCAFMVDNEAIYDICRRNLDIERPTYTNLNRLISQIVSSITA
SLRFDGALNVDLTEFQTNLVPYPRIHFPLATYAPVISAEKAYHEQLSVAEITNACFEPAN
QMVKCDPRHGKYMACCLLYRGDVVPKDVNAAIATIKTKRSIQFVDWCPTGFKVGINYQPP
TVVPGGDLAKVQRAVCMLSNTTAIAEAWARLDHKFDLMYAKRAFVHWYVGEGMEEGEFSE
AREDMAALEKDYEEVGVDSVEGEGEEEGEEY
>sp|Q93050|VPP1_HUMAN V-type proton ATPase 116 kDa subunit a 1 OS=Homo sapiens OX=9606 GN=ATP6V0A1 PE=1 SV=3
MGELFRSEEMTLAQLFLQSEAAYCCVSELGELGKVQFRDLNPDVNVFQRKFVNEVRRCEE
MDRKLRFVEKEIRKANIPIMDTGENPEVPFPRDMIDLEANFEKIENELKEINTNQEALKR
NFLELTELKFILRKTQQFFDEMADPDLLEESSSLLEPSEMGRGTPLRLGFVAGVINRERI
PTFERMLWRVCRGNVFLRQAEIENPLEDPVTGDYVHKSVFIIFFQGDQLKNRVKKICEGF
RASLYPCPETPQERKEMASGVNTRIDDLQMVLNQTEDHRQRVLQAAAKNIRVWFIKVRKM
KAIYHTLNLCNIDVTQKCLIAEVWCPVTDLDSIQFALRRGTEHSGSTVPSILNRMQTNQT
PPTYNKTNKFTYGFQNIVDAYGIGTYREINPAPYTIITFPFLFAVMFGDFGHGILMTLFA
VWMVLRESRILSQKNENEMFSTVFSGRYIILLMGVFSMYTGLIYNDCFSKSLNIFGSSWS
VRPMFTYNWTEETLRGNPVLQLNPALPGVFGGPYPFGIDPIWNIATNKLTFLNSFKMKMS
VILGIIHMLFGVSLSLFNHIYFKKPLNIYFGFIPEIIFMTSLFGYLVILIFYKWTAYDAH
TSENAPSLLIHFINMFLFSYPESGYSMLYSGQKGIQCFLVVVALLCVPWMLLFKPLVLRR
QYLRRKHLGTLNFGGIRVGNGPTEEDAEIIQHDQLSTHSEDADEPSEDEVFDFGDTMVHQ
AIHTIEYCLGCISNTASYLRLWALSLAHAQLSEVLWTMVIHIGLSVKSLAGGLVLFFFFT
AFATLTVAILLIMEGLSAFLHALRLHWVEFQNKFYSGTGFKFLPFSFEHIREGKFEE
>sp|P14923|PLAK_HUMAN Junction plakoglobin OS=Homo sapiens OX=9606 GN=JUP PE=1 SV=3
MEVMNLMEQPIKVTEWQQTYTYDSGIHSGANTCVPSVSSKGIMEEDEACGRQYTLKKTTT
YTQGVPPSQGDLEYQMSTTARAKRVREAMCPGVSGEDSSLLLATQVEGQATNLQRLAEPS
QLLKSAIVHLINYQDDAELATRALPELTKLLNDEDPVVVTKAAMIVNQLSKKEASRRALM
GSPQLVAAVVRTMQNTSDLDTARCTTSILHNLSHHREGLLAIFKSGGIPALVRMLSSPVE
SVLFYAITTLHNLLLYQEGAKMAVRLADGLQKMVPLLNKNNPKFLAITTDCLQLLAYGNQ
ESKLIILANGGPQALVQIMRNYSYEKLLWTTSRVLKVLSVCPSNKPAIVEAGGMQALGKH
LTSNSPRLVQNCLWTLRNLSDVATKQEGLESVLKILVNQLSVDDVNVLTCATGTLSNLTC
NNSKNKTLVTQNSGVEALIHAILRAGDKDDITEPAVCALRHLTSRHPEAEMAQNSVRLNY

GIPAIVKLLNQPNQWPLVKATIGLIRNLALCPANHAPLQEAAVIPRLVQLLVKAHQDAQR
HVAAGTQQPYTDGVRMEEIVEGCTGALHILARDPMNRMEIFRLNTIPLFVQLLYSSVENI
QRVAAGVLCELAQDKEAADAIDAEGASAPLMELLHSRNEGTATYAAAVLFRISEDKNPDY
RKRVSVELTNSLFKHDPAAWEAAQSMIPINEPYGDDMDATYRPMYSSDVPLDPLEMHMDM
DGDYPIDTYSDGLRPPYPTADHMLA
>sp|Q15149|PLEC_HUMAN Plectin OS=Homo sapiens OX=9606 GN=PLEC PE=1 SV=3
MVAGMLMPRDQLRAIYEVLFREGVMVAKKDRRPRSLHPHVPGVTNLQVMRAMASLRARGL
VRETFAWCHFYWYLTNEGIAHLRQYLHLPPEIVPASLQRVRRPVAMVMPARRTPHVQAVQ
GPLGSPPKRGPLPTEEQRVYRRKELEEVSPETPVVPATTQRTLARPGPEPAPATDERDRV
QKKTFTKWVNKHLIKAQRHISDLYEDLRDGHNLISLLEVLSGDSLPREKGRMRFHKLQNV
QIALDYLRHRQVKLVNIRNDDIADGNPKLTLGLIWTIILHFQISDIQVSGQSEDMTAKEK
LLLWSQRMVEGYQGLRCDNFTSSWRDGRLFNAIIHRHKPLLIDMNKVYRQTNLENLDQAF
SVAERDLGVTRLLDPEDVDVPQPDEKSIITYVSSLYDAMPRVPDVQDGVRANELQLRWQE
YRELVLLLLQWMRHHTAAFEERRFPSSFEEIEILWSQFLKFKEMELPAKEADKNRSKGIY
QSLEGAVQAGQLKVPPGYHPLDVEKEWGKLHVAILEREKQLRSEFERLECLQRIVTKLQM
EAGLCEEQLNQADALLQSDVRLLAAGKVPQRAGEVERDLDKADSMIRLLFNDVQTLKDGR
HPQGEQMYRRVYRLHERLVAIRTEYNLRLKAGVAAPATQVAQVTLQSVQRRPELEDSTLR
YLQDLLAWVEENQHRVDGAEWGVDLPSVEAQLGSHRGLHQSIEEFRAKIERARSDEGQLS
PATRGAYRDCLGRLDLQYAKLLNSSKARLRSLESLHSFVAAATKELMWLNEKEEEEVGFD
WSDRNTNMTAKKESYSALMRELELKEKKIKELQNAGDRLLREDHPARPTVESFQAALQTQ
WSWMLQLCCCIEAHLKENAAYFQFFSDVREAEGQLQKLQEALRRKYSCDRSATVTRLEDL
LQDAQDEKEQLNEYKGHLSGLAKRAKAVVQLKPRHPAHPMRGRLPLLAVCDYKQVEVTVH
KGDECQLVGPAQPSHWKVLSSSGSEAAVPSVCFLVPPPNQEAQEAVTRLEAQHQALVTLW
HQLHVDMKSLLAWQSLRRDVQLIRSWSLATFRTLKPEEQRQALHSLELHYQAFLRDSQDA
GGFGPEDRLMAEREYGSCSHHYQQLLQSLEQGAQEESRCQRCISELKDIRLQLEACETRT
VHRLRLPLDKEPARECAQRIAEQQKAQAEVEGLGKGVARLSAEAEKVLALPEPSPAAPTL
RSELELTLGKLEQVRSLSAIYLEKLKTISLVIRGTQGAEEVLRAHEEQLKEAQAVPATLP
ELEATKASLKKLRAQAEAQQPTFDALRDELRGAQEVGERLQQRHGERDVEVERWRERVAQ
LLERWQAVLAQTDVRQRELEQLGRQLRYYRESADPLGAWLQDARRRQEQIQAMPLADSQA
VREQLRQEQALLEEIERHGEKVEECQRFAKQYINAIKDYELQLVTYKAQLEPVASPAKKP
KVQSGSESVIQEYVDLRTHYSELTTLTSQYIKFISETLRRMEEEERLAEQQRAEERERLA
EVEAALEKQRQLAEAHAQAKAQAEREAKELQQRMQEEVVRREEAAVDAQQQKRSIQEELQ
QLRQSSEAEIQAKARQAEAAERSRLRIEEEIRVVRLQLEATERQRGGAEGELQALRARAE
EAEAQKRQAQEEAERLRRQVQDESQRKRQAEVELASRVKAEAEAAREKQRALQALEELRL
QAEEAERRLRQAEVERARQVQVALETAQRSAEAELQSKRASFAEKTAQLERSLQEEHVAV
AQLREEAERRAQQQAEAERAREEAERELERWQLKANEALRLRLQAEEVAQQKSLAQAEAE
KQKEEAEREARRRGKAEEQAVRQRELAEQELEKQRQLAEGTAQQRLAAEQELIRLRAETE
QGEQQRQLLEEELARLQREAAAATQKRQELEAELAKVRAEMEVLLASKARAEEESRSTSE
KSKQRLEAEAGRFRELAEEAARLRALAEEAKRQRQLAEEDAARQRAEAERVLAEKLAAIG
EATRLKTEAEIALKEKEAENERLRRLAEDEAFQRRRLEEQAAQHKADIEERLAQLRKASD
SELERQKGLVEDTLRQRRQVEEEILALKASFEKAAAGKAELELELGRIRSNAEDTLRSKE
QAELEAARQRQLAAEEERRRREAEERVQKSLAAEEEAARQRKAALEEVERLKAKVEEARR
LRERAEQESARQLQLAQEAAQKRLQAEEKAHAFAVQQKEQELQQTLQQEQSVLDQLRGEA
EAARRAAEEAEEARVQAEREAAQSRRQVEEAERLKQSAEEQAQARAQAQAAAEKLRKEAE

QEAARRAQAEQAALRQKQAADAEMEKHKKFAEQTLRQKAQVEQELTTLRLQLEETDHQKN
LLDEELQRLKAEATEAARQRSQVEEELFSVRVQMEELSKLKARIEAENRALILRDKDNTQ
RFLQEEAEKMKQVAEEAARLSVAAQEAARLRQLAEEDLAQQRALAEKMLKEKMQAVQEAT
RLKAEAELLQQQKELAQEQARRLQEDKEQMAQQLAEETQGFQRTLEAERQRQLEMSAEAE
RLKLRVAEMSRAQARAEEDAQRFRKQAEEIGEKLHRTELATQEKVTLVQTLEIQRQQSDH
DAERLREAIAELEREKEKLQQEAKLLQLKSEEMQTVQQEQLLQETQALQQSFLSEKDSLL
QRERFIEQEKAKLEQLFQDEVAKAQQLREEQQRQQQQMEQERQRLVASMEEARRRQHEAE
EGVRRKQEELQQLEQQRRQQEELLAEENQRLREQLQLLEEQHRAALAHSEEVTASQVAAT
KTLPNGRDALDGPAAEAEPEHSFDGLRRKVSAQRLQEAGILSAEELQRLAQGHTTVDELA
RREDVRHYLQGRSSIAGLLLKATNEKLSVYAALQRQLLSPGTALILLEAQAASGFLLDPV
RNRRLTVNEAVKEGVVGPELHHKLLSAERAVTGYKDPYTGQQISLFQAMQKGLIVREHGI
RLLEAQIATGGVIDPVHSHRVPVDVAYRRGYFDEEMNRVLADPSDDTKGFFDPNTHENLT
YLQLLERCVEDPETGLCLLPLTDKAAKGGELVYTDSEARDVFEKATVSAPFGKFQGKTVT
IWEIINSEYFTAEQRRDLLRQFRTGRITVEKIIKIIITVVEEQEQKGRLCFEGLRSLVPA
AELLESRVIDRELYQQLQRGERSVRDVAEVDTVRRALRGANVIAGVWLEEAGQKLSIYNA
LKKDLLPSDMAVALLEAQAGTGHIIDPATSARLTVDEAVRAGLVGPEFHEKLLSAEKAVT
GYRDPYTGQSVSLFQALKKGLIPREQGLRLLDAQLSTGGIVDPSKSHRVPLDVACARGCL
DEETSRALSAPRADAKAYSDPSTGEPATYGELQQRCRPDQLTGLSLLPLSEKAARARQEE
LYSELQARETFEKTPVEVPVGGFKGRTVTVWELISSEYFTAEQRQELLRQFRTGKVTVEK
VIKILITIVEEVETLRQERLSFSGLRAPVPASELLASGVLSRAQFEQLKDGKTTVKDLSE
LGSVRTLLQGSGCLAGIYLEDTKEKVSIYEAMRRGLLRATTAALLLEAQAATGFLVDPVR
NQRLYVHEAVKAGVVGPELHEQLLSAEKAVTGYRDPYSGSTISLFQAMQKGLVLRQHGIR
LLEAQIATGGIIDPVHSHRVPVDVAYQRGYFSEEMNRVLADPSDDTKGFFDPNTHENLTY
RQLLERCVEDPETGLRLLPLKGAEKAEVVETTQVYTEEETRRAFEETQIDIPGGGSHGGS
TMSLWEVMQSDLIPEEQRAQLMADFQAGRVTKERMIIIIIEIIEKTEIIRQQGLASYDYV
RRRLTAEDLFEARIISLETYNLLREGTRSLREALEAESAWCYLYGTGSVAGVYLPGSRQT
LSIYQALKKGLLSAEVARLLLEAQAATGFLLDPVKGERLTVDEAVRKGLVGPELHDRLLS
AERAVTGYRDPYTEQTISLFQAMKKELIPTEEALRLLDAQLATGGIVDPRLGFHLPLEVA
YQRGYLNKDTHDQLSEPSEVRSYVDPSTDERLSYTQLLRRCRRDDGTGQLLLPLSDARKL
TFRGLRKQITMEELVRSQVMDEATALQLREGLTSIEEVTKNLQKFLEGTSCIAGVFVDAT
KERLSVYQAMKKGIIRPGTAFELLEAQAATGYVIDPIKGLKLTVEEAVRMGIVGPEFKDK
LLSAERAVTGYKDPYSGKLISLFQAMKKGLILKDHGIRLLEAQIATGGIIDPEESHRLPV
EVAYKRGLFDEEMNEILTDPSDDTKGFFDPNTEENLTYLQLMERCITDPQTGLCLLPLKE
KKRERKTSSKSSVRKRRVVIVDPETGKEMSVYEAYRKGLIDHQTYLELSEQECEWEEITI
SSSDGVVKSMIIDRRSGRQYDIDDAIAKNLIDRSALDQYRAGTLSITEFADMLSGNAGGF
RSRSSSVGSSSSYPISPAVSRTQLASWSDPTEETGPVAGILDTETLEKVSITEAMHRNLV
DNITGQRLLEAQACTGGIIDPSTGERFPVTDAVNKGLVDKIMVDRINLAQKAFCGFEDPR
TKTKMSAAQALKKGWLYYEAGQRFLEVQYLTGGLIEPDTPGRVPLDEALQRGTVDARTAQ
KLRDVGAYSKYLTCPKTKLKISYKDALDRSMVEEGTGLRLLEAAAQSTKGYYSPYSVSGS
GSTAGSRTGSRTGSRAGSRRGSFDATGSGFSMTFSSSSYSSSGYGRRYASGSSASLGGPE
SAVA
>sp|Q5TDP6|LGSN_HUMAN Lengsin OS=Homo sapiens OX=9606 GN=LGSN PE=1 SV=1
MNNEEDLLQEDSTRDEGNETEANSMNTLRRTRKKVTKPYVCSTEVGETDMSNSNDCMRDS
SQILTPPQLSSRMKHIRQAMAKNRLQFVRFEATDLHGVSRSKTIPAHFFQEKVSHGVCMP

RGYLEVIPNPKDNEMNNIRATCFNSDIVLMPELSTFRVLPWADRTARVICDTFTVTGEPL
LTSPRYIAKRQLSHLQASGFSLLSAFIYDFCIFGVPEILNSKIISFPALTFLNNHDQPFM
QELVDGLYHTGANVESFSSSTRPGQMEISFLPEFGISSADNAFTLRTGVKEVARKYNYIA
SFFIETGFCDSGILSHSLWDVDRKKNMFCSTSGTEQLTITGKKWLAGLLKHSAALSCLMA
PSVSCRKRYSKDRKDLKKSVPTTWGYNDNSCIFNIKCHGEKGTRIENKLGSATANPYLVL
AATVAAGLDGLHSSNEVLAGPDESTDFYQVEPSEIPLKLEDALVALEEDQCLRQALGETF
IRYFVAMKKYELENEEIAAERNKFLEYFI
>sp|Q86VP6|CAND1_HUMAN Cullin-associated NEDD8-dissociated protein 1 OS=Homo sapiens OX=9606 GN=CAND1 PE=1 SV=2
MASASYHISNLLEKMTSSDKDFRFMATNDLMTELQKDSIKLDDDSERKVVKMILKLLEDK
NGEVQNLAVKCLGPLVSKVKEYQVETIVDTLCTNMLSDKEQLRDISSIGLKTVIGELPPA
SSGSALAANVCKKITGRLTSAIAKQEDVSVQLEALDIMADMLSRQGGLLVNFHPSILTCL
LPQLTSPRLAVRKRTIIALGHLVMSCGNIVFVDLIEHLLSELSKNDSMSTTRTYIQCIAA
ISRQAGHRIGEYLEKIIPLVVKFCNVDDDELREYCIQAFESFVRRCPKEVYPHVSTIINI
CLKYLTYDPNYNYDDEDEDENAMDADGGDDDDQGSDDEYSDDDDMSWKVRRAAAKCLDAV
VSTRHEMLPEFYKTVSPALISRFKEREENVKADVFHAYLSLLKQTRPVQSWLCDPDAMEQ
GETPLTMLQSQVPNIVKALHKQMKEKSVKTRQCCFNMLTELVNVLPGALTQHIPVLVPGI
IFSLNDKSSSSNLKIDALSCLYVILCNHSPQVFHPHVQALVPPVVACVGDPFYKITSEAL
LVTQQLVKVIRPLDQPSSFDATPYIKDLFTCTIKRLKAADIDQEVKERAISCMGQIICNL
GDNLGSDLPNTLQIFLERLKNEITRLTTVKALTLIAGSPLKIDLRPVLGEGVPILASFLR
KNQRALKLGTLSALDILIKNYSDSLTAAMIDAVLDELPPLISESDMHVSQMAISFLTTLA
KVYPSSLSKISGSILNELIGLVRSPLLQGGALSAMLDFFQALVVTGTNNLGYMDLLRMLT
GPVYSQSTALTHKQSYYSIAKCVAALTRACPKEGPAVVGQFIQDVKNSRSTDSIRLLALL
SLGEVGHHIDLSGQLELKSVILEAFSSPSEEVKSAASYALGSISVGNLPEYLPFVLQEIT
SQPKRQYLLLHSLKEIISSASVVGLKPYVENIWALLLKHCECAEEGTRNVVAECLGKLTL
IDPETLLPRLKGYLISGSSYARSSVVTAVKFTISDHPQPIDPLLKNCIGDFLKTLEDPDL
NVRRVALVTFNSAAHNKPSLIRDLLDTVLPHLYNETKVRKELIREVEMGPFKHTVDDGLD
IRKAAFECMYTLLDSCLDRLDIFEFLNHVEDGLKDHYDIKMLTFLMLVRLSTLCPSAVLQ
RLDRLVEPLRATCTTKVKANSVKQEFEKQDELKRSAMRAVAALLTIPEAEKSPLMSEFQS
QISSNPELAAIFESIQKDSSSTNLESMDTS
>sp|P10114|RAP2A_HUMAN Ras-related protein Rap-2a OS=Homo sapiens OX=9606 GN=RAP2A PE=1 SV=1
MREYKVVVLGSGGVGKSALTVQFVTGTFIEKYDPTIEDFYRKEIEVDSSPSVLEILDTAG
TEQFASMRDLYIKNGQGFILVYSLVNQQSFQDIKPMRDQIIRVKRYEKVPVILVGNKVDL
ESEREVSSSEGRALAEEWGCPFMETSAKSKTMVDELFAEIVRQMNYAAQPDKDDPCCSAC
NIQ
>sp|P11216|PYGB_HUMAN Glycogen phosphorylase, brain form OS=Homo sapiens OX=9606 GN=PYGB PE=1 SV=5
MAKPLTDSEKRKQISVRGLAGLGDVAEVRKSFNRHLHFTLVKDRNVATPRDYFFALAHTV
RDHLVGRWIRTQQHYYERDPKRIYYLSLEFYMGRTLQNTMVNLGLQNACDEAIYQLGLDL
EELEEIEEDAGLGNGGLGRLAACFLDSMATLGLAAYGYGIRYEFGIFNQKIVNGWQVEEA
DDWLRYGNPWEKARPEYMLPVHFYGRVEHTPDGVKWLDTQVVLAMPYDTPVPGYKNNTVN
TMRLWSAKAPNDFKLQDFNVGDYIEAVLDRNLAENISRVLYPNDNFFEGKELRLKQEYFV
VAATLQDIIRRFKSSKFGCRDPVRTCFETFPDKVAIQLNDTHPALSIPELMRILVDVEKV

DWDKAWEITKKTCAYTNHTVLPEALERWPVSMFEKLLPRHLEIIYAINQRHLDHVAALFP
GDVDRLRRMSVIEEGDCKRINMAHLCVIGSHAVNGVARIHSEIVKQSVFKDFYELEPEKF
QNKTNGITPRRWLLLCNPGLADTIVEKIGEEFLTDLSQLKKLLPLVSDEVFIRDVAKVKQ
ENKLKFSAFLEKEYKVKINPSSMFDVHVKRIHEYKRQLLNCLHVVTLYNRIKRDPAKAFV
PRTVMIGGKAAPGYHMAKLIIKLVTSIGDVVNHDPVVGDRLKVIFLENYRVSLAEKVIPA
ADLSQQISTAGTEASGTGNMKFMLNGALTIGTMDGANVEMAEEAGAENLFIFGLRVEDVE
ALDRKGYNAREYYDHLPELKQAVDQISSGFFSPKEPDCFKDIVNMLMHHDRFKVFADYEA
YMQCQAQVDQLYRNPKEWTKKVIRNIACSGKFSSDRTITEYAREIWGVEPSDLQIPPPNI
PRD
>sp|P84077|ARF1_HUMAN ADP-ribosylation factor 1 OS=Homo sapiens OX=9606 GN=ARF1 PE=1 SV=2
MGNIFANLFKGLFGKKEMRILMVGLDAAGKTTILYKLKLGEIVTTIPTIGFNVETVEYKN
ISFTVWDVGGQDKIRPLWRHYFQNTQGLIFVVDSNDRERVNEAREELMRMLAEDELRDAV
LLVFANKQDLPNAMNAAEITDKLGLHSLRHRNWYIQATCATSGDGLYEGLDWLSNQLRNQ
K
>tr|Q562Z4|Q562Z4_HUMAN Actin-like protein (Fragment) OS=Homo sapiens OX=9606 GN=ACT PE=4 SV=1
KIWRHTFYNELRVAPEEHPVLLTEAPLNPKANREKMTQIMFETFNTPAMYVAIQAVLSLY
ASGRTTGIVMDSGDGVTHTVPIYKGYALPHAILRLDLAGRDLT
>sp|P07738|PMGE_HUMAN Bisphosphoglycerate mutase OS=Homo sapiens OX=9606 GN=BPGM PE=1 SV=2
MSKYKLIMLRHGEGAWNKENRFCSWVDQKLNSEGMEEARNCGKQLKALNFEFDLVFTSVL
NRSIHTAWLILEELGQEWVPVESSWRLNERHYGALIGLNREQMALNHGEEQVRLWRRSYN
VTPPPIEESHPYYQEIYNDRRYKVCDVPLDQLPRSESLKDVLERLLPYWNERIAPEVLRG
KTILISAHGNSSRALLKHLEGISDEDIINITLPTGVPILLELDENLRAVGPHQFLGDQEA
IQAAIKKVEDQGKVKQAKK
>sp|P35241|RADI_HUMAN Radixin OS=Homo sapiens OX=9606 GN=RDX PE=1 SV=1
MPKPINVRVTTMDAELEFAIQPNTTGKQLFDQVVKTVGLREVWFFGLQYVDSKGYSTWLK
LNKKVTQQDVKKENPLQFKFRAKFFPEDVSEELIQEITQRLFFLQVKEAILNDEIYCPPE
TAVLLASYAVQAKYGDYNKEIHKPGYLANDRLLPQRVLEQHKLTKEQWEERIQNWHEEHR
GMLREDSMMEYLKIAQDLEMYGVNYFEIKNKKGTELWLGVDALGLNIYEHDDKLTPKIGF
PWSEIRNISFNDKKFVIKPIDKKAPDFVFYAPRLRINKRILALCMGNHELYMRRRKPDTI
EVQQMKAQAREEKHQKQLERAQLENEKKKREIAEKEKERIEREKEELMERLKQIEEQTIK
AQKELEEQTRKALELDQERKRAKEEAERLEKERRAAEEAKSAIAKQAADQMKNQEQLAAE
LAEFTAKIALLEEAKKKKEEEATEWQHKAFAAQEDLEKTKEELKTVMSAPPPPPPPPVIP
PTENEHDEHDENNAEASAELSNEGVMNHRSEEERVTETQKNERVKKQLQALSSELAQARD
ETKKTQNDVLHAENVKAGRDKYKTLRQIRQGNTKQRIDEFEAM
>sp|P54709|AT1B3_HUMAN Sodium/potassium-transporting ATPase subunit beta-3 OS=Homo sapiens OX=9606 GN=ATP1B3 PE=1 SV=1
MTKNEKKSLNQSLAEWKLFIYNPTTGEFLGRTAKSWGLILLFYLVFYGFLAALFSFTMWV
MLQTLNDEVPKYRDQIPSPGLMVFPKPVTALEYTFSRSDPTSYAGYIEDLKKFLKPYTLE
EQKNLTVCPDGALFEQKGPVYVACQFPISLLQACSGMNDPDFGYSQGNPCILVKMNRIIG
LKPEGVPRIDCVSKNEDIPNVAVYPHNGMIDLKYFPYYGKKLHVGYLQPLVAVQVSFAPN
NTGKEVTVECKIDGSANLKSQDDRDKFLGRVMFKITARA

>sp|P62834|RAP1A_HUMAN Ras-related protein Rap-1A OS=Homo sapiens OX=9606 GN=RAP1A PE=1 SV=1
MREYKLVVLGSGGVGKSALTVQFVQGIFVEKYDPTIEDSYRKQVEVDCQQCMLEILDTAG
TEQFTAMRDLYMKNGQGFALVYSITAQSTFNDLQDLREQILRVKDTEDVPMILVGNKCDL
EDERVVGKEQGQNLARQWCNCAFLESSAKSKINVNEIFYDLVRQINRKTPVEKKKPKKKS
CLLL
>sp|O94760|DDAH1_HUMAN N(G),N(G)-dimethylarginine dimethylaminohydrolase 1 OS=Homo sapiens OX=9606 GN=DDAH1 PE=1 SV=3
MAGLGHPAAFGRATHAVVRALPESLGQHALRSAKGEEVDVARAERQHQLYVGVLGSKLGL
QVVELPADESLPDCVFVEDVAVVCEETALITRPGAPSRRKEVDMMKEALEKLQLNIVEMK
DENATLDGGDVLFTGREFFVGLSKRTNQRGAEILADTFKDYAVSTVPVADGLHLKSFCSM
AGPNLIAIGSSESAQKALKIMQQMSDHRYDKLTVPDDIAANCIYLNIPNKGHVLLHRTPE
EYPESAKVYEKLKDHMLIPVSMSELEKVDGLLTCCSVLINKKVDS
>sp|O94856|NFASC_HUMAN Neurofascin OS=Homo sapiens OX=9606 GN=NFASC PE=1 SV=4
MARQPPPPWVHAAFLLCLLSLGGAIEIPMDPSIQNELTQPPTITKQSAKDHIVDPRDNIL
IECEAKGNPAPSFHWTRNSRFFNIAKDPRVSMRRRSGTLVIDFRSGGRPEEYEGEYQCFA
RNKFGTALSNRIRLQVSKSPLWPKENLDPVVVQEGAPLTLQCNPPPGLPSPVIFWMSSSM
EPITQDKRVSQGHNGDLYFSNVMLQDMQTDYSCNARFHFTHTIQQKNPFTLKVLTTRGVA
ERTPSFMYPQGTASSQMVLRGMDLLLECIASGVPTPDIAWYKKGGDLPSDKAKFENFNKA
LRITNVSEEDSGEYFCLASNKMGSIRHTISVRVKAAPYWLDEPKNLILAPGEDGRLVCRA
NGNPKPTVQWMVNGEPLQSAPPNPNREVAGDTIIFRDTQISSRAVYQCNTSNEHGYLLAN
AFVSVLDVPPRMLSPRNQLIRVILYNRTRLDCPFFGSPIPTLRWFKNGQGSNLDGGNYHV
YENGSLEIKMIRKEDQGIYTCVATNILGKAENQVRLEVKDPTRIYRMPEDQVARRGTTVQ
LECRVKHDPSLKLTVSWLKDDEPLYIGNRMKKEDDSLTIFGVAERDQGSYTCVASTELDQ
DLAKAYLTVLADQATPTNRLAALPKGRPDRPRDLELTDLAERSVRLTWIPGDANNSPITD
YVVQFEEDQFQPGVWHDHSKYPGSVNSAVLRLSPYVNYQFRVIAINEVGSSHPSLPSERY
RTSGAPPESNPGDVKGEGTRKNNMEITWTPMNATSAFGPNLRYIVKWRRRETREAWNNVT
VWGSRYVVGQTPVYVPYEIRVQAENDFGKGPEPESVIGYSGEDYPRAAPTEVKVRVMNST
AISLQWNRVYSDTVQGQLREYRAYYWRESSLLKNLWVSQKRQQASFPGDRLRGVVSRLFP
YSNYKLEMVVVNGRGDGPRSETKEFTTPEGVPSAPRRFRVRQPNLETINLEWDHPEHPNG
IMIGYTLKYVAFNGTKVGKQIVENFSPNQTKFTVQRTDPVSRYRFTLSARTQVGSGEAVT
EESPAPPNEATPTAAPPTLPPTTVGATGAVSSTDATAIAATTEATTVPIIPTVAPTTIAT
TTTVATTTTTTAAATTTTESPPTTTSGTKIHESAPDEQSIWNVTVLPNSKWANITWKHNF
GPGTDFVVEYIDSNHTKKTVPVKAQAQPIQLTDLYPGMTYTLRVYSRDNEGISSTVITFM
TSTAYTNNQADIATQGWFIGLMCAIALLVLILLIVCFIKRSRGGKYPVREKKDVPLGPED
PKEEDGSFDYSDEDNKPLQGSQTSLDGTIKQQESDDSLVDYGEGGEGQFNEDGSFIGQYT
VKKDKEETEGNESSEATSPVNAIYSLA
>sp|O95490|AGRL2_HUMAN Adhesion G protein-coupled receptor L2 OS=Homo sapiens OX=9606 GN=ADGRL2 PE=1 SV=2
MVSSGCRMRSLWFIIVISFLPNTEGFSRAALPFGLVRRELSCEGYSIDLRCPGSDVIMIE
SANYGRTDDKICDADPFQMENTDCYLPDAFKIMTQRCNNRTQCIVVTGSDVFPDPCPGTY
KYLEVQYECVPYIFVCPGTLKAIVDSPCIYEAEQKAGAWCKDPLQAADKIYFMPWTPYRT
DTLIEYASLEDFQNSRQTTTYKLPNRVDGTGFVVYDGAVFFNKERTRNIVKFDLRTRIKS
GEAIINYANYHDTSPYRWGGKTDIDLAVDENGLWVIYATEQNNGMIVISQLNPYTLRFEA

TWETVYDKRAASNAFMICGVLYVVRSVYQDNESETGKNSIDYIYNTRLNRGEYVDVPFPN
QYQYIAAVDYNPRDNQLYVWNNNFILRYSLEFGPPDPAQVPTTAVTITSSAELFKTIIST
TSTTSQKGPMSTTVAGSQEGSKGTKPPPAVSTTKIPPITNIFPLPERFCEALDSKGIKWP
QTQRGMMVERPCPKGTRGTASYLCMISTGTWNPKGPDLSNCTSHWVNQLAQKIRSGENAA
SLANELAKHTKGPVFAGDVSSSVRLMEQLVDILDAQLQELKPSEKDSAGRSYNKLQKREK
TCRAYLKAIVDTVDNLLRPEALESWKHMNSSEQAHTATMLLDTLEEGAFVLADNLLEPTR
VSMPTENIVLEVAVLSTEGQIQDFKFPLGIKGAGSSIQLSANTVKQNSRNGLAKLVFIIY
RSLGQFLSTENATIKLGADFIGRNSTIAVNSHVISVSINKESSRVYLTDPVLFTLPHIDP
DNYFNANCSFWNYSERTMMGYWSTQGCKLVDTNKTRTTCACSHLTNFAILMAHREIAYKD
GVHELLLTVITWVGIVISLVCLAICIFTFCFFRGLQSDRNTIHKNLCINLFIAEFIFLIG
IDKTKYAIACPIFAGLLHFFFLAAFAWMCLEGVQLYLMLVEVFESEYSRKKYYYVAGYLF
PATVVGVSAAIDYKSYGTEKACWLHVDNYFIWSFIGPVTFIILLNIIFLVITLCKMVKHS
NTLKPDSSRLENIKSWVLGAFALLCLLGLTWSFGLLFINEETIVMAYLFTIFNAFQGVFI
FIFHCALQKKVRKEYGKCFRHSYCCGGLPTESPHSSVKASTTRTSARYSSGTQSRIRRMW
NDTVRKQSESSFISGDINSTSTLNQGMTGNYLLTNPLLRPHGTNNPYNTLLAETVVCNAP
SAPVFNSPGHSLNNARDTSAMDTLPLNGNFNNSYSLHKGDYNDSVQVVDCGLSLNDTAFE
KMIISELVHNNLRGSSKTHNLELTLPVKPVIGGSSSEDDAIVADASSLMHSDNPGLELHH
KELEAPLIPQRTHSLLYQPQKKVKSEGTDSYVSQLTAEAEDHLQSPNRDSLYTSMPNLRD
SPYPESSPDMEEDLSPSRRSENEDIYYKSMPNLGAGHQLQMCYQISRGNSDGYIIPINKE
GCIPEGDVREGQMQLVTSL
>sp|P26038|MOES_HUMAN Moesin OS=Homo sapiens OX=9606 GN=MSN PE=1 SV=3
MPKTISVRVTTMDAELEFAIQPNTTGKQLFDQVVKTIGLREVWFFGLQYQDTKGFSTWLK
LNKKVTAQDVRKESPLLFKFRAKFYPEDVSEELIQDITQRLFFLQVKEGILNDDIYCPPE
TAVLLASYAVQSKYGDFNKEVHKSGYLAGDKLLPQRVLEQHKLNKDQWEERIQVWHEEHR
GMLREDAVLEYLKIAQDLEMYGVNYFSIKNKKGSELWLGVDALGLNIYEQNDRLTPKIGF
PWSEIRNISFNDKKFVIKPIDKKAPDFVFYAPRLRINKRILALCMGNHELYMRRRKPDTI
EVQQMKAQAREEKHQKQMERAMLENEKKKREMAEKEKEKIEREKEELMERLKQIEEQTKK
AQQELEEQTRRALELEQERKRAQSEAEKLAKERQEAEEAKEALLQASRDQKKTQEQLALE
MAELTARISQLEMARQKKESEAVEWQQKAQMVQEDLEKTRAELKTAMSTPHVAEPAENEQ
DEQDENGAEASADLRADAMAKDRSEEERTTEAEKNERVQKHLKALTSELANARDESKKTA
NDMIHAENMRLGRDKYKTLRQIRQGNTKQRIDEFESM
>sp|P55786|PSA_HUMAN Puromycin-sensitive aminopeptidase OS=Homo sapiens OX=9606 GN=NPEPPS PE=1 SV=2
MWLAAAAPSLARRLLFLGPPPPPLLLLVFSRSSRRRLHSLGLAAMPEKRPFERLPADVSP
INYSLCLKPDLLDFTFEGKLEAAAQVRQATNQIVMNCADIDIITASYAPEGDEEIHATGF
NYQNEDEKVTLSFPSTLQTGTGTLKIDFVGELNDKMKGFYRSKYTTPSGEVRYAAVTQFE
ATDARRAFPCWDEPAIKATFDISLVVPKDRVALSNMNVIDRKPYPDDENLVEVKFARTPV
MSTYLVAFVVGEYDFVETRSKDGVCVRVYTPVGKAEQGKFALEVAAKTLPFYKDYFNVPY
PLPKIDLIAIADFAAGAMENWGLVTYRETALLIDPKNSCSSSRQWVALVVGHELAHQWFG
NLVTMEWWTHLWLNEGFASWIEYLCVDHCFPEYDIWTQFVSADYTRAQELDALDNSHPIE
VSVGHPSEVDEIFDAISYSKGASVIRMLHDYIGDKDFKKGMNMYLTKFQQKNAATEDLWE
SLENASGKPIAAVMNTWTKQMGFPLIYVEAEQVEDDRLLRLSQKKFCAGGSYVGEDCPQW
MVPITISTSEDPNQAKLKILMDKPEMNVVLKNVKPDQWVKLNLGTVGFYRTQYSSAMLES
LLPGIRDLSLPPVDRLGLQNDLFSLARAGIISTVEVLKVMEAFVNEPNYTVWSDLSCNLG

ILSTLLSHTDFYEEIQEFVKDVFSPIGERLGWDPKPGEGHLDALLRGLVLGKLGKAGHKA
TLEEARRRFKDHVEGKQILSADLRSPVYLTVLKHGDGTTLDIMLKLHKQADMQEEKNRIE
RVLGATLLPDLIQKVLTFALSEEVRPQDTVSVIGGVAGGSKHGRKAAWKFIKDNWEELYN
RYQGGFLISRLIKLSVEGFAVDKMAGEVKAFFESHPAPSAERTIQQCCENILLNAAWLKR
DAESIHQYLLQRKASPPTV
>sp|Q01484|ANK2_HUMAN Ankyrin-2 OS=Homo sapiens OX=9606 GN=ANK2 PE=1 SV=4
MMNEDAAQKSDSGEKFNGSSQRRKRPKKSDSNASFLRAARAGNLDKVVEYLKGGIDINTC
NQNGLNALHLAAKEGHVGLVQELLGRGSSVDSATKKGNTALHIASLAGQAEVVKVLVKEG
ANINAQSQNGFTPLYMAAQENHIDVVKYLLENGANQSTATEDGFTPLAVALQQGHNQAVA
ILLENDTKGKVRLPALHIAARKDDTKSAALLLQNDHNADVQSKMMVNRTTESGFTPLHIA
AHYGNVNVATLLLNRGAAVDFTARNGITPLHVASKRGNTNMVKLLLDRGGQIDAKTRDGL
TPLHCAARSGHDQVVELLLERGAPLLARTKNGLSPLHMAAQGDHVECVKHLLQHKAPVDD
VTLDYLTALHVAAHCGHYRVTKLLLDKRANPNARALNGFTPLHIACKKNRIKVMELLVKY
GASIQAITESGLTPIHVAAFMGHLNIVLLLLQNGASPDVTNIRGETALHMAARAGQVEVV
RCLLRNGALVDARAREEQTPLHIASRLGKTEIVQLLLQHMAHPDAATTNGYTPLHISARE
GQVDVASVLLEAGAAHSLATKKGFTPLHVAAKYGSLDVAKLLLQRRAAADSAGKNGLTPL
HVAAHYDNQKVALLLLEKGASPHATAKNGYTPLHIAAKKNQMQIASTLLNYGAETNIVTK
QGVTPLHLASQEGHTDMVTLLLDKGANIHMSTKSGLTSLHLAAQEDKVNVADILTKHGAD
QDAHTKLGYTPLIVACHYGNVKMVNFLLKQGANVNAKTKNGYTPLHQAAQQGHTHIINVL
LQHGAKPNATTANGNTALAIAKRLGYISVVDTLKVVTEEVTTTTTTITEKHKLNVPETMT
EVLDVSDEEGDDTMTGDGGEYLRPEDLKELGDDSLPSSQFLDGMNYLRYSLEGGRSDSLR
SFSSDRSHTLSHASYLRDSAVMDDSVVIPSHQVSTLAKEAERNSYRLSWGTENLDNVALS
SSPIHSGFLVSFMVDARGGAMRGCRHNGLRIIIPPRKCTAPTRVTCRLVKRHRLATMPPM
VEGEGLASRLIEVGPSGAQFLGKLHLPTAPPPLNEGESLVSRILQLGPPGTKFLGPVIVE
IPHFAALRGKERELVVLRSENGDSWKEHFCDYTEDELNEILNGMDEVLDSPEDLEKKRIC
RIITRDFPQYFAVVSRIKQDSNLIGPEGGVLSSTVVPQVQAVFPEGALTKRIRVGLQAQP
MHSELVKKILGNKATFSPIVTLEPRRRKFHKPITMTIPVPKASSDVMLNGFGGDAPTLRL
LCSITGGTTPAQWEDITGTTPLTFVNECVSFTTNVSARFWLIDCRQIQESVTFASQVYRE
IICVPYMAKFVVFAKSHDPIEARLRCFCMTDDKVDKTLEQQENFAEVARSRDVEVLEGKP
IYVDCFGNLVPLTKSGQHHIFSFFAFKENRLPLFVKVRDTTQEPCGRLSFMKEPKSTRGL
VHQAICNLNITLPIYTKESESDQEQEEEIDMTSEKNDETESTETSVLKSHLVNEVPVLAS
PDLLSEVSEMKQDLIKMTAILTTDVSDKAGSIKVKELVKAAEEEPGEPFEIVERVKEDLE
KVNEILRSGTCTRDESSVQSSRSERGLVEEEWVIVSDEEIEEARQKAPLEITEYPCVEVR
IDKEIKGKVEKDSTGLVNYLTDDLNTCVPLPKEQLQTVQDKAGKKCEALAVGRSSEKEGK
DIPPDETQSTQKQHKPSLGIKKPVRRKLKEKQKQKEEGLQASAEKAELKKGSSEESLGED
PGLAPEPLPTVKATSPLIEETPIGSIKDKVKALQKRVEDEQKGRSKLPIRVKGKEDVPKK
TTHRPHPAASPSLKSERHAPGSPSPKTERHSTLSSSAKTERHPPVSPSSKTEKHSPVSPS
AKTERHSPASSSSKTEKHSPVSPSTKTERHSPVSSTKTERHPPVSPSGKTDKRPPVSPSG
RTEKHPPVSPGRTEKRLPVSPSGRTDKHQPVSTAGKTEKHLPVSPSGKTEKQPPVSPTSK
TERIEETMSVRELMKAFQSGQDPSKHKTGLFEHKSAKQKQPQEKGKVRVEKEKGPILTQR
EAQKTENQTIKRGQRLPVTGTAESKRGVRVSSIGVKKEDAAGGKEKVLSHKIPEPVQSVP
EEESHRESEVPKEKMADEQGDMDLQISPDRKTSTDFSEVIKQELEDNDKYQQFRLSEETE
KAQLHLDQVLTSPFNTTFPLDYMKDEFLPALSLQSGALDGSSESLKNEGVAGSPCGSLME
GTPQISSEESYKHEGLAETPETSPESLSFSPKKSEEQTGETKESTKTETTTEIRSEKEHP

TTKDITGGSEERGATVTEDSETSTESFQKEATLGSPKDTSPKRQDDCTGSCSVALAKETP
TGLTEEAACDEGQRTFGSSAHKTQTDSEVQESTATSDETKALPLPEASVKTDTGTESKPQ
GVIRSPQGLELALPSRDSEVLSAVADDSLAVSHKDSLEASPVLEDNSSHKTPDSLEPSPL
KESPCRDSLESSPVEPKMKAGIFPSHFPLPAAVAKTELLTEVASVRSRLLRDPDGSAEDD
SLEQTSLMESSGKSPLSPDTPSSEEVSYEVTPKTTDVSTPKPAVIHECAEEDDSENGEKK
RFTPEEEMFKMVTKIKMFDELEQEAKQKRDYKKEPKQEESSSSSDPDADCSVDVDEPKHT
GSGEDESGVPVLVTSESRKVSSSSESEPELAQLKKGADSGLLPEPVIRVQPPSPLPSSMD
SNSSPEEVQFQPVVSKQYTFKMNEDTQEEPGKSEEEKDSESHLAEDRHAVSTEAEDRSYD
KLNRDTDQPKICDGHGCEAMSPSSSAAPVSSGLQSPTGDDVDEQPVIYKESLALQGTHEK
DTEGEELDVSRAESPQADCPSESFSSSSSLPHCLVSEGKELDEDISATSSIQKTEVTKTD
ETFENLPKDCPSQDSSITTQTDRFSMDVPVSDLAENDEIYDPQITSPYENVPSQSFFSSE
ESKTQTDANHTTSFHSSEVYSVTITSPVEDVVVASSSSGTVLSKESNFEGQDIKMESQQE
STLWEMQSDSVSSSFEPTMSATTTVVGEQISKVIITKTDVDSDSWSEIREDDEAFEARVK
EEEQKIFGLMVDRQSQGTTPDTTPARTPTEEGTPTSEQNPFLFQEGKLFEMTRSGAIDMT
KRSYADESFHFFQIGQESREETLSEDVKEGATGADPLPLETSAESLALSESKETVDDEAD
LLPDDVSEEVEEIPASDAQLNSQMGISASTETPTKEAVSVGTKDLPTVQTGDIPPLSGVK
QISCPDSSEPAVQVQLDFSTLTRSVYSDRGDDSPDSSPEEQKSVIEIPTAPMENVPFTES
KSKIPVRTMPTSTPAPPSAEYESSVSEDFLSSVDEENKADEAKPKSKLPVKVPLQRVEQQ
LSDLDTSVQKTVAPQGQDMASIAPDNRSKSESDASSLDSKTKCPVKTRSYTETETESRER
AEELELESEEGATRPKILTSRLPVKSRSTTSSCRGGTSPTKESKEHFFDLYRNSIEFFEE
ISDEASKLVDRLTQSEREQEIVSDDESSSALEVSVIENLPPVETEHSVPEDIFDTRPIWD
ESIETLIERIPDENGHDHAEDPQDEQERIEERLAYIADHLGFSWTELARELDFTEEQIHQ
IRIENPNSLQDQSHALLKYWLERDGKHATDTNLVECLTKINRMDIVHLMETNTEPLQERI
SHSYAEIEQTITLDHSEGFSVLQEELCTAQHKQKEEQAVSKESETCDHPPIVSEEDISVG
YSTFQDGVPKTEGDSSATALFPQTHKEQVQQDFSGKMQDLPEESSLEYQQEYFVTTPGTE
TSETQKAMIVPSSPSKTPEEVSTPAEEEKLYLQTPTSSERGGSPIIQEPEEPSEHREESS
PRKTSLVIVESADNQPETCERLDEDAAFEKGDDMPEIPPETVTEEEYIDEHGHTVVKKVT
RKIIRRYVSSEGTEKEEIMVQGMPQEPVNIEEGDGYSKVIKRVVLKSDTEQSEDNNE
>sp|Q9H4G0|E41L1_HUMAN Band 4.1-like protein 1 OS=Homo sapiens OX=9606 GN=EPB41L1
PE=1 SV=2
MTTETGPDSEVKKAQEEAPQQPEAAAAVTTPVTPAGHGHPEANSNEKHPSQQDTRPAEQS
LDMEEKDYSEADGLSERTTPSKAQKSPQKIAKKYKSAICRVTLLDASEYECEVEKHGRGQ
VLFDLVCEHLNLLEKDYFGLTFCDADSQKNWLDPSKEIKKQIRSSPWNFAFTVKFYPPDP
AQLTEDITRYYLCLQLRADIITGRLPCSFVTHALLGSYAVQAELGDYDAEEHVGNYVSEL
RFAPNQTRELEERIMELHKTYRGMTPGEAEIHFLENAKKLSMYGVDLHHAKDSEGIDIML
GVCANGLLIYRDRLRINRFAWPKILKISYKRSNFYIKIRPGEYEQFESTIGFKLPNHRSA
KRLWKVCIEHHTFFRLVSPEPPPKGFLVMGSKFRYSGRTQAQTRQASALIDRPAPFFERS
SSKRYTMSRSLDGAEFSRPASVSENHDAGPDGDKRDEDGESGGQRSEAEEGEVRTPTKIK
ELKPEQETTPRHKQEFLDKPEDVLLKHQASINELKRTLKEPNSKLIHRDRDWERERRLPS
SPASPSPKGTPEKANERAGLREGSEEKVKPPRPRAPESDTGDEDQDQERDTVFLKDNHLA
IERKCSSITVSSTSSLEAEVDFTVIGDYHGSAFEDFSRSLPELDRDKSDSDTEGLLFSRD
LNKGAPSQDDESGGIEDSPDRGACSTPDMPQFEPVKTETMTVSSLAIRKKIEPEAVLQTR
VSAMDNTQQVDGSASVGREFIATTPSITTETISTTMENSLKSGKGAAAMIPGPQTVATEI
RSLSPIIGKDVLTSTYGATAETLSTSTTTHVTKTVKGGFSETRIEKRIIITGDEDVDQDQ

ALALAIKEAKLQHPDMLVTKAVVYRETDPSPEERDKKPQES
>sp|P14174|MIF_HUMAN Macrophage migration inhibitory factor OS=Homo sapiens OX=9606 GN=MIF PE=1 SV=4
MPMFIVNTNVPRASVPDGFLSELTQQLAQATGKPPQYIAVHVVPDQLMAFGGSSEPCALC
SLHSIGKIGGAQNRSYSKLLCGLLAERLRISPDRVYINYYDMNAANVGWNNSTFA
>sp|P15121|ALDR_HUMAN Aldo-keto reductase family 1 member B1 OS=Homo sapiens OX=9606 GN=AKR1B1 PE=1 SV=3
MASRLLLNNGAKMPILGLGTWKSPPGQVTEAVKVAIDVGYRHIDCAHVYQNENEVGVAIQ
EKLREQVVKREELFIVSKLWCTYHEKGLVKGACQKTLSDLKLDYLDLYLIHWPTGFKPGK
EFFPLDESGNVVPSDTNILDTWAAMEELVDEGLVKAIGISNFNHLQVEMILNKPGLKYKP
AVNQIECHPYLTQEKLIQYCQSKGIVVTAYSPLGSPDRPWAKPEDPSLLEDPRIKAIAAK
HNKTTAQVLIRFPMQRNLVVIPKSVTPERIAENFKVFDFELSSQDMTTLLSYNRNWRVCA
LLSCTSHKDYPFHEEF
>sp|P61088|UBE2N_HUMAN Ubiquitin-conjugating enzyme E2 N OS=Homo sapiens OX=9606 GN=UBE2N PE=1 SV=1
MAGLPRRIIKETQRLLAEPVPGIKAEPDESNARYFHVVIAGPQDSPFEGGTFKLELFLPE
EYPMAAPKVRFMTKIYHPNVDKLGRICLDILKDKWSPALQIRTVLLSIQALLSAPNPDDP
LANDVAEQWKTNEAQAIETARAWTRLYAMNNI
>sp|Q15257|PTPA_HUMAN Serine/threonine-protein phosphatase 2A activator OS=Homo sapiens OX=9606 GN=PTPA PE=1 SV=3
MAEGERQPPPDSSEEAPPATQNFIIPKKEIHTVPDMGKWKRSQAYADYIGFILTLNEGVK
GKKLTFEYRVSEMWNEVHEEKEQAAKQSVSCDECIPLPRAGHCAPSEAIEKLVALLNTLD
RWIDETPPVDQPSRFGNKAYRTWYAKLDEEAENLVATVVPTHLAAAVPEVAVYLKESVGN
STRIDYGTGHEAAFAAFLCCLCKIGVLRVDDQIAIVFKVFNRYLEVMRKLQKTYRMEPAG
SQGVWGLDDFQFLPFIWGSSQLIDHPYLEPRHFVDEKAVNENHKDYMFLECILFITEMKT
GPFAEHSNQLWNISAVPSWSKVNQGLIRMYKAECLEKFPVIQHFKFGSLLPIHPVTSG
>sp|P60953|CDC42_HUMAN Cell division control protein 42 homolog OS=Homo sapiens OX=9606 GN=CDC42 PE=1 SV=2
MQTIKCVVVGDGAVGKTCLLISYTTNKFPSEYVPTVFDNYAVTVMIGGEPYTLGLFDTAG
QEDYDRLRPLSYPQTDVFLVCFSVVSPSSFENVKEKWVPEITHHCPKTPFLLVGTQIDLR
DDPSTIEKLAKNKQKPITPETAEKLARDLKAVKYVECSALTQKGLKNVFDEAILAALEPP
EPKKSRRCVLL
>sp|P00338|LDHA_HUMAN L-lactate dehydrogenase A chain OS=Homo sapiens OX=9606 GN=LDHA PE=1 SV=2
MATLKDQLIYNLLKEEQTPQNKITVVGVGAVGMACAISILMKDLADELALVDVIEDKLKG
EMMDLQHGSLFLRTPKIVSGKDYNVTANSKLVIITAGARQQEGESRLNLVQRNVNIFKFI
IPNVVKYSPNCKLLIVSNPVDILTYVAWKISGFPKNRVIGSGCNLDSARFRYLMGERLGV
HPLSCHGWVLGEHGDSSVPVWSGMNVAGVSLKTLHPDLGTDKDKEQWKEVHKQVVESAYE
VIKLKGYTSWAIGLSVADLAESIMKNLRRVHPVSTMIKGLYGIKDDVFLSVPCILGQNGI
SDLVKVTLTSEEEARLKKSADTLWGIQKELQF
>sp|O00192|ARVC_HUMAN Splicing regulator ARVCF OS=Homo sapiens OX=9606 GN=ARVCF PE=1 SV=1
MEDCNVHSAASILASVKEQEARFERLTRALEQERRHVALQLERAQQPGMVSGGMGSGQPL
PMAWQQLVLQEQSPGSQASLATMPEAPDVLEETVTVEEDPGTPTSHVSIVTSEDGTTRRT

ETKVTKTVKTVTTRTVRQVPVGPDGLPLLDGGPPLGPFADGALDRHFLLRGGGPVATLSR
AYLSSGGGFPEGPEPRDSPSYGSLSRGLGMRPPRAGPLGPGPGDGCFTLPGHREAFPVGP
EPGPPGGRSLPERFQAEPYGLEDDTRSLAADDEGGPELEPDYGTATRRRPECGRGLHTRA
YEDTADDGGELADERPAFPMVTAPLAQPERGSMGSLDRLVRRSPSVDSARKEPRWRDPEL
PEVLAMLRHPVDPVKANAAAYLQHLCFENEGVKRRVRQLRGLPLLVALLDHPRAEVRRRA
CGALRNLSYGRDTDNKAAIRDCGGVPALVRLLRAARDNEVRELVTGTLWNLSSYEPLKMV
IIDHGLQTLTHEVIVPHSGWEREPNEDSKPRDAEWTTVFKNTSGCLRNVSSDGAEARRRL
RECEGLVDALLHALQSAVGRKDTDNKSVENCVCIMRNLSYHVHKEVPGADRYQEAEPGPL
GSAVGSQRRRRDDASCFGGKKAKEEWFHQGKKDGEMDRNFDTLDLPKRTEAAKGFELLYQ
PEVVRLYLSLLTESRNFNTLEAAAGALQNLSAGNWMWATYIRATVRKERGLPVLVELLQS
ETDKVVRAVAIALRNLSLDRRNKDLIGSYAMAELVRNVRNAQAPPRPGACLEEDTVVAVL
NTIHEIVSDSLDNARSLLQARGVPALVALVASSQSVREAKAASHVLQTVWSYKELRGTLQ
KDGWTKARFQSAAATAKGPKGALSPGGFDDSTLPLVDKSLEGEKTGSRDVIPMDALGPDG
YSTVDRRERRPRGASSAGEASEKEPLKLDPSRKAPPPGPSRPAVRLVDAVGDAKPQPVDS
WV
>sp|P06733|ENOA_HUMAN Alpha-enolase OS=Homo sapiens OX=9606 GN=ENO1 PE=1 SV=2
MSILKIHAREIFDSRGNPTVEVDLFTSKGLFRAAVPSGASTGIYEALELRDNDKTRYMGK
GVSKAVEHINKTIAPALVSKKLNVTEQEKIDKLMIEMDGTENKSKFGANAILGVSLAVCK
AGAVEKGVPLYRHIADLAGNSEVILPVPAFNVINGGSHAGNKLAMQEFMILPVGAANFRE
AMRIGAEVYHNLKNVIKEKYGKDATNVGDEGGFAPNILENKEGLELLKTAIGKAGYTDKV
VIGMDVAASEFFRSGKYDLDFKSPDDPSRYISPDQLADLYKSFIKDYPVVSIEDPFDQDD
WGAWQKFTASAGIQVVGDDLTVTNPKRIAKAVNEKSCNCLLLKVNQIGSVTESLQACKLA
QANGWGVMVSHRSGETEDTFIADLVVGLCTGQIKTGAPCRSERLAKYNQLLRIEEELGSK
AKFAGRNFRNPLAK
>sp|P06744|G6PI_HUMAN Glucose-6-phosphate isomerase OS=Homo sapiens OX=9606
GN=GPI PE=1 SV=4
MAALTRDPQFQKLQQWYREHRSELNLRRLFDANKDRFNHFSLTLNTNHGHILVDYSKNLV
TEDVMRMLVDLAKSRGVEAARERMFNGEKINYTEGRAVLHVALRNRSNTPILVDGKDVMP
EVNKVLDKMKSFCQRVRSGDWKGYTGKTITDVINIGIGGSDLGPLMVTEALKPYSSGGPR
VWYVSNIDGTHIAKTLAQLNPESSLFIIASKTFTTQETITNAETAKEWFLQAAKDPSAVA
KHFVALSTNTTKVKEFGIDPQNMFEFWDWVGGRYSLWSAIGLSIALHVGFDNFEQLLSGA
HWMDQHFRTTPLEKNAPVLLALLGIWYINCFGCETHAMLPYDQYLHRFAAYFQQGDMESN
GKYITKSGTRVDHQTGPIVWGEPGTNGQHAFYQLIHQGTKMIPCDFLIPVQTQHPIRKGL
HHKILLANFLAQTEALMRGKSTEEARKELQAAGKSPEDLERLLPHKVFEGNRPTNSIVFT
KLTPFMLGALVAMYEHKIFVQGIIWDINSFDQWGVELGKQLAKKIEPELDGSAQVTSHDA
STNGLINFIKQQREARVQ
>sp|P13798|ACPH_HUMAN Acylamino-acid-releasing enzyme OS=Homo sapiens OX=9606
GN=APEH PE=1 SV=4
MERQVLLSEPEEAAALYRGLSRQPALSAACLGPEVTTQYGGQYRTVHTEWTQRDLERMEN
IRFCRQYLVFHDGDSVVFAGPAGNSVETRGELLSRESPSGTMKAVLRKAGGTGPGEEKQF
LEVWEKNRKLKSFNLSALEKHGPVYEDDCFGCLSWSHSETHLLYVAEKKRPKAESFFQTK
ALDVSASDDEIARLKKPDQAIKGDQFVFYEDWGENMVSKSIPVLCVLDVESGNISVLEGV
PENVSPGQAFWAPGDAGVVFVGWWHEPFRLGIRFCTNRRSALYYVDLIGGKCELLSDDSL
AVSSPRLSPDQCRIVYLQYPSLIPHHQCSQLCLYDWYTKVTSVVVDVVPRQLGENFSGIY

CSLLPLGCWSADSQRVVFDSAQRSRQDLFAVDTQVGTVTSLTAGGSGGSWKLLTIDQDLM
VAQFSTPSLPPTLKVGFLPSAGKEQSVLWVSLEEAEPIPDIHWGIRVLQPPPEQENVQYA
GLDFEAILLQPGSPPDKTQVPMVVMPHGGPHSSFVTAWMLFPAMLCKMGFAVLLVNYRGS
TGFGQDSILSLPGNVGHQDVKDVQFAVEQVLQEEHFDASHVALMGGSHGGFISCHLIGQY
PETYRACVARNPVINIASMLGSTDIPDWCVVEAGFPFSSDCLPDLSVWAEMLDKSPIRYI
PQVKTPLLLMLGQEDRRVPFKQGMEYYRALKTRNVPVRLLLYPKSTHALSEVEVESDSFM
NAVLWLRTHLGS
>sp|P36969|GPX4_HUMAN Phospholipid hydroperoxide glutathione peroxidase GPX4 OS=Homo sapiens OX=9606 GN=GPX4 PE=1 SV=3
MSLGRLCRLLKPALLCGALAAPGLAGTMCASRDDWRCARSMHEFSAKDIDGHMVNLDKYR
GFVCIVTNVASQUGKTEVNYTQLVDLHARYAECGLRILAFPCNQFGKQEPGSNEEIKEFA
AGYNVKFDMFSKICVNGDDAHPLWKWMKIQPKGKGILGNAIKWNFTKFLIDKNGCVVKRY
GPMEEPLVIEKDLPHYF
>sp|P52565|GDIR1_HUMAN Rho GDP-dissociation inhibitor 1 OS=Homo sapiens OX=9606 GN=ARHGDIA PE=1 SV=3
MAEQEPTAEQLAQIAAENEEDEHSVNYKPPAQKSIQEIQELDKDDESLRKYKEALLGRVA
VSADPNVPNVVVTGLTLVCSSAPGPLELDLTGDLESFKKQSFVLKEGVEYRIKISFRVNR
EIVSGMKYIQHTYRKGVKIDKTDYMVGSYGPRAEEYEFLTPVEEAPKGMLARGSYSIKSR
FTDDDKTDHLSWEWNLTIKKDWKD
>sp|P04075|ALDOA_HUMAN Fructose-bisphosphate aldolase A OS=Homo sapiens OX=9606 GN=ALDOA PE=1 SV=2
MPYQYPALTPEQKKELSDIAHRIVAPGKGILAADESTGSIAKRLQSIGTENTEENRRFYR
QLLLTADDRVNPCIGGVILFHETLYQKADDGRPFPQVIKSKGGVVGIKVDKGVVPLAGTN
GETTTQGLDGLSERCAQYKKDGADFAKWRCVLKIGEHTPSALAIMENANVLARYASICQQ
NGIVPIVEPEILPDGDHDLKRCQYVTEKVLAAVYKALSDHHIYLEGTLLKPNMVTPGHAC
TQKFSHEEIAMATVTALRRTVPPAVTGITFLSGGQSEEEASINLNAINKCPLLKPWALTF
SYGRALQASALKAWGGKKENLKAAQEEYVKRALANSLACQGKYTPSGQAGAAASESLFVS
NHAY
>sp|P05026|AT1B1_HUMAN Sodium/potassium-transporting ATPase subunit beta-1 OS=Homo sapiens OX=9606 GN=ATP1B1 PE=1 SV=1
MARGKAKEEGSWKKFIWNSEKKEFLGRTGGSWFKILLFYVIFYGCLAGIFIGTIQVMLLT
ISEFKPTYQDRVAPPGLTQIPQIQKTEISFRPNDPKSYEAYVLNIVRFLEKYKDSAQRDD
MIFEDCGDVPSEPKERGDFNHERGERKVCRFKLEWLGNCSGLNDETYGYKEGKPCIIIKL
NRVLGFKPKPPKNESLETYPVMKYNPNVLPVQCTGKRDEDKDKVGNVEYFGLGNSPGFPL
QYYPYYGKLLQPKYLQPLLAVQFTNLTMDTEIRIECKAYGENIGYSEKDRFQGRFDVKIE
VKS
>sp|P08134|RHOC_HUMAN Rho-related GTP-binding protein RhoC OS=Homo sapiens OX=9606 GN=RHOC PE=1 SV=1
MAAIRKKLVIVGDGACGKTCLLIVFSKDQFPEVYVPTVFENYIADIEVDGKQVELALWDT
AGQEDYDRLRPLSYPDTDVILMCFSIDSPDSLENIPEKWTPEVKHFCPNVPIILVGNKKD
LRQDEHTRRELAKMKQEPVRSEEGRDMANRISAFGYLECSAKTKEGVREVFEMATRAGLQ
VRKNKRRRGCPIL
>sp|P11844|CRGA_HUMAN Gamma-crystallin A OS=Homo sapiens OX=9606 GN=CRYGA PE=2 SV=3

MGKITFYEDRDFQGRCYNCISDCPNLRVYFSRCNSIRVDSGCWMLYERPNYQGHQYFLRR
GKYPDYQHWMGLSDSVQSCRIIPHTSSHKLRLYERDDYRGLMSELTDDCACVPELFRLPE
IYSLHVLEGCWVLYEMPNYRGRQYLLRPGDYRRYHDWGGADAKVGSLRRVTDLY
>sp|P62258|1433E_HUMAN 14-3-3 protein epsilon OS=Homo sapiens OX=9606 GN=YWHAE PE=1 SV=1
MDDREDLVYQAKLAEQAERYDEMVESMKKVAGMDVELTVEERNLLSVAYKNVIGARRASW
RIISSIEQKEENKGGEDKLKMIREYRQMVETELKLICCDILDVLDKHLIPAANTGESKVF
YYKMKGDYHRYLAEFATGNDRKEAAENSLVAYKAASDIAMTELPPTHPIRLGLALNFSVF
YYEILNSPDRACRLAKAAFDDAIAELDTLSEESYKDSTLIMQLLRDNLTLWTSDMQGDGE
EQNKEALQDVEDENQ
>sp|Q9BY67|CADM1_HUMAN Cell adhesion molecule 1 OS=Homo sapiens OX=9606 GN=CADM1 PE=1 SV=2
MASVVLPSGSQCAAAAAAAAPPGLRLRLLLLLFSAAALIPTGDGQNLFTKDVTVIEGEVA
TISCQVNKSDDSVIQLLNPNRQTIYFRDFRPLKDSRFQLLNFSSSELKVSLTNVSISDEG
RYFCQLYTDPPQESYTTITVLVPPRNLMIDIQKDTAVEGEEIEVNCTAMASKPATTIRWF
KGNTELKGKSEVEEWSDMYTVTSQLMLKVHKEDDGVPVICQVEHPAVTGNLQTQRYLEVQ
YKPQVHIQMTYPLQGLTREGDALELTCEAIGKPQPVMVTWVRVDDEMPQHAVLSGPNLFI
NNLNKTDNGTYRCEASNIVGKAHSDYMLYVYDPPTTIPPPTTTTTTTTTTTTILTIITD
SRAGEEGSIRAVDHAVIGGVVAVVVFAMLCLLIILGRYFARHKGTYFTHEAKGADDAADA
DTAIINAEGGQNNSEEKKEYFI
>sp|O95834|EMAL2_HUMAN Echinoderm microtubule-associated protein-like 2 OS=Homo sapiens OX=9606 GN=EML2 PE=1 SV=1
MSSFGAGKTKEVIFSVEDGSVKMFLRGRPVPMMIPDELAPTYSLDTRSELPSCRLKLEWV
YGYRGRDCRANLYLLPTGEIVYFVASVAVLYSVEEQRQRHYLGHNDDIKCLAIHPDMVTI
ATGQVAGTTKEGKPLPPHVRIWDSVSLSTLHVLGLGVFDRAVCCVGFSKSNGGNLLCAVD
ESNDHMLSVWDWAKETKVVDVKCSNEAVLVATFHPTDPTVLITCGKSHIYFWTLEGGSLS
KRQGLFEKHEKPKYVLCVTFLEGGDVVTGDSGGNLYVWGKGGNRITQAVLGAHDGGVFGL
CALRDGTLVSGGGRDRRVVLWGSDYSKLQEVEVPEDFGPVRTVAEGHGDTLYVGTTRNSI
LQGSVHTGFSLLVQGHVEELWGLATHPSRAQFVTCGQDKLVHLWSSDSHQPLWSRIIEDP
ARSAGFHPSGSVLAVGTVTGRWLLLDTETHDLVAIHTDGNEQISVVSFSPDGAYLAVGSH
DNLVYVYTVDQGGRKVSRLGKCSGHSSFITHLDWAQDSSCFVTNSGDYEILYWDPATCKQ
ITSADAVRNMEWATATCVLGFGVFGIWSEGADGTDINAVARSHDGKLLASADDFGKVHLF
SYPCCQPRALSHKYGGHSSHVTNVAFLWDDSMALTTGGKDTSVLQWRVV
>sp|P00918|CAH2_HUMAN Carbonic anhydrase 2 OS=Homo sapiens OX=9606 GN=CA2 PE=1 SV=2
MSHHWGYGKHNGPEHWHKDFPIAKGERQSPVDIDTHTAKYDPSLKPLSVSYDQATSLRIL
NNGHAFNVEFDDSQDKAVLKGGPLDGTYRLIQFHFHWGSLDGQGSEHTVDKKKYAAELHL
VHWNTKYGDFGKAVQQPDGLAVLGIFLKVGSAKPGLQKVVDVLDSIKTKGKSADFTNFDP
RGLLPESLDYWTYPGSLTTPPLLECVTWIVLKEPISVSSEQVLKFRKLNFNGEGEPEELM
VDNWRPAQPLKNRQIKASFK
>sp|P04899|GNAI2_HUMAN Guanine nucleotide-binding protein G(i) subunit alpha-2 OS=Homo sapiens OX=9606 GN=GNAI2 PE=1 SV=3
MGCTVSAEDKAAAERSKMIDKNLREDGEKAAREVKLLLLGAGESGKSTIVKQMKIIHEDG
YSEEECRQYRAVVYSNTIQSIMAIVKAMGNLQIDFADPSRADDARQLFALSCTAEEQGVL

PDDLSGVIRRLWADHGVQACFGRSREYQLNDSAAYYLNDLERIAQSDYIPTQQDVLRTRV
KTTGIVETHFTFKDLHFKMFDVGGQRSERKKWIHCFEGVTAIIFCVALSAYDLVLAEDEE
MNRMHESMKLFDSICNNKWFTDTSIILFLNKKDLFEEKITHSPLTICFPEYTGANKYDEA
ASYIQSKFEDLNKRKDTKEIYTHFTCATDTKNVQFVFDAVTDVIIKNNLKDCGLF
>sp|P19022|CADH2_HUMAN Cadherin-2 OS=Homo sapiens OX=9606 GN=CDH2 PE=1 SV=4
MCRIAGALRTLLPLLAALLQASVEASGEIALCKTGFPEDVYSAVLSKDVHEGQPLLNVKF
SNCNGKRKVQYESSEPADFKVDEDGMVYAVRSFPLSSEHAKFLIYAQDKETQEKWQVAVK
LSLKPTLTEESVKESAEVEEIVFPRQFSKHSGHLQRQKRDWVIPPINLPENSRGPFPQEL
VRIRSDRDKNLSLRYSVTGPGADQPPTGIFIINPISGQLSVTKPLDREQIARFHLRAHAV
DINGNQVENPIDIVINVIDMNDNRPEFLHQVWNGTVPEGSKPGTYVMTVTAIDADDPNAL
NGMLRYRIVSQAPSTPSPNMFTINNETGDIITVAAGLDREKVQQYTLIIQATDMEGNPTY
GLSNTATAVITVTDVNDNPPEFTAMTFYGEVPENRVDIIVANLTVTDKDQPHTPAWNAVY
RISGGDPTGRFAIQTDPNSNDGLVTVVKPIDFETNRMFVLTVAAENQVPLAKGIQHPPQS
TATVSVTVIDVNENPYFAPNPKIIRQEEGLHAGTMLTTFTAQDPDRYMQQNIRYTKLSDP
ANWLKIDPVNGQITTIAVLDRESPNVKNNIYNATFLASDNGIPPMSGTGTLQIYLLDIND
NAPQVLPQEAETCETPDPNSINITALDYDIDPNAGPFAFDLPLSPVTIKRNWTITRLNGD
FAQLNLKIKFLEAGIYEVPIIITDSGNPPKSNISILRVKVCQCDSNGDCTDVDRIVGAGL
GTGAIIAILLCIIILLILVLMFVVWMKRRDKERQAKQLLIDPEDDVRDNILKYDEEGGGE
EDQDYDLSQLQQPDTVEPDAIKPVGIRRMDERPIHAEPQYPVRSAAPHPGDIGDFINEGL
KAADNDPTAPPYDSLLVFDYEGSGSTAGSLSSLNSSSSGGEQDYDYLNDWGPRFKKLADM
YGGGDD
>sp|P52209|6PGD_HUMAN 6-phosphogluconate dehydrogenase, decarboxylating OS=Homo sapiens OX=9606 GN=PGD PE=1 SV=3
MAQADIALIGLAVMGQNLILNMNDHGFVVCAFNRTVSKVDDFLANEAKGTKVVGAQSLKE
MVSKLKKPRRIILLVKAGQAVDDFIEKLVPLLDTGDIIIDGGNSEYRDTTRRCRDLKAKG
ILFVGSGVSGGEEGARYGPSLMPGGNKEAWPHIKTIFQGIAAKVGTGEPCCDWVGDEGAG
HFVKMVHNGIEYGDMQLICEAYHLMKDVLGMAQDEMAQAFEDWNKTELDSFLIEITANIL
KFQDTDGKHLLPKIRDSAGQKGTGKWTAISALEYGVPVTLIGEAVFARCLSSLKDERIQA
SKKLKGPQKFQFDGDKKSFLEDIRKALYASKIISYAQGFMLLRQAATEFGWTLNYGGIAL
MWRGGCIIRSVFLGKIKDAFDRNPELQNLLLDDFFKSAVENCQDSWRRAVSTGVQAGIPM
PCFTTALSFYDGYRHEMLPASLIQAQRDYFGAHTYELLAKPGQFIHTNWTGHGGTVSSSS
YNA
>sp|P62937|PPIA_HUMAN Peptidyl-prolyl cis-trans isomerase A OS=Homo sapiens OX=9606 GN=PPIA PE=1 SV=2
MVNPTVFFDIAVDGEPLGRVSFELFADKVPKTAENFRALSTGEKGFGYKGSCFHRIIPGF
MCQGGDFTRHNGTGGKSIYGEKFEDENFILKHTGPGILSMANAGPNTNGSQFFICTAKTE
WLDGKHVVFGKVKEGMNIVEAMERFGSRNGKTSKKITIADCGQLE
>sp|Q08257|QOR_HUMAN Quinone oxidoreductase OS=Homo sapiens OX=9606 GN=CRYZ PE=1 SV=1
MATGQKLMRAVRVFEFGGPEVLKLRSDIAVPIPKDHQVLIKVHACGVNPVETYIRSGTYS
RKPLLPYTPGSDVAGVIEAVGDNASAFKKGDRVFTSSTISGGYAEYALAADHTVYKLPEK
LDFKQGAAIGIPYFTAYRALIHSACVKAGESVLVHGASGGVGLAACQIARAYGLKILGTA
GTEEGQKIVLQNGAHEVFNHREVNYIDKIKKYVGEKGIDIIIEMLANVNLSKDLSLLSHG
GRVIVVGSRGTIEINPRDTMAKESSIIGVTLFSSTKEEFQQYAAALQAGMEIGWLKPVIG

SQYPLEKVAEAHENIIHGSGATGKMILLL
>sp|Q8TD20|GTR12_HUMAN Solute carrier family 2, facilitated glucose transporter member 12 OS=Homo sapiens OX=9606 GN=SLC2A12 PE=2 SV=1
MVPVENTEGPSLLNQKGTAVETEGSGSRHPPWARGCGMFTFLSSVTAAVSGLLVGYELGI
ISGALLQIKTLLALSCHEQEMVVSSLVIGALLASLTGGVLIDRYGRRTAIILSSCLLGLG
SLVLILSLSYTVLIVGRIAIGVSISLSSIATCVYIAEIAPQHRRGLLVSLNELMIVIGIL
SAYISNYAFANVFHGWKYMFGLVIPLGVLQAIAMYFLPPSPRFLVMKGQEGAASKVLGRL
RALSDTTEELTVIKSSLKDEYQYSFWDLFRSKDNMRTRIMIGLTLVFFVQITGQPNILFY
ASTVLKSVGFQSNEAASLASTGVGVVKVISTIPATLLVDHVGSKTFLCIGSSVMAASLVT
MGIVNLNIHMNFTHICRSHNSINQSLDESVIYGPGNLSTNNNTLRDHFKGISSHSRSSLM
PLRNDVDKRGETTSASLLNAGLSHTEYQIVTDPGDVPAFLKWLSLASLLVYVAAFSIGLG
PMPWLVLSEIFPGGIRGRAMALTSSMNWGINLLISLTFLTVTDLIGLPWVCFIYTIMSLA
SLLFVVMFIPETKGCSLEQISMELAKVNYVKNNICFMSHHQEELVPKQPQKRKPQEQLLE
CNKLCGRGQSRQLSPET
>sp|Q9NTI2|AT8A2_HUMAN Phospholipid-transporting ATPase IB OS=Homo sapiens OX=9606 GN=ATP8A2 PE=1 SV=3
MLNGAGLDKALKMSLPRRSRIRSSVGPVRSSLGYKKAEDEMSRATSVGDQLEAPARTIYL
NQPHLNKFRDNQISTAKYSVLTFLPRFLYEQIRRAANAFFLFIALLQQIPDVSPTGRYTT
LVPLIIILTIAGIKEIVEDFKRHKADNAVNKKKTIVLRNGMWHTIMWKEVAVGDIVKVVN
GQYLPADVVLLSSSEPQAMCYVETANLDGETNLKIRQGLSHTADMQTREVLMKLSGTIEC
EGPNRHLYDFTGNLNLDGKSLVALGPDQILLRGTQLRNTQWVFGIVVYTGHDTKLMQNST
KAPLKRSNVEKVTNVQILVLFGILLVMALVSSAGALYWNRSHGEKNWYIKKMDTTSDNFG
YNLLTFIILYNNLIPISLLVTLEVVKYTQALFINWDTDMYYIGNDTPAMARTSNLNEELG
QVKYLFSDKTGTLTCNIMNFKKCSIAGVTYGHFPELAREPSSDDFCRMPPPCSDSCDFDD
PRLLKNIEDRHPTAPCIQEFLTLLAVCHTVVPEKDGDNIIYQASSPDEAALVKGAKKLGF
VFTARTPFSVIIEAMGQEQTFGILNVLEFSSDRKRMSVIVRTPSGRLRLYCKGADNVIFE
RLSKDSKYMEETLCHLEYFATEGLRTLCVAYADLSENEYEEWLKVYQEASTILKDRAQRL
EECYEIIEKNLLLLGATAIEDRLQAGVPETIATLLKAEIKIWVLTGDKQETAINIGYSCR
LVSQNMALILLKEDSLDATRAAITQHCTDLGNLLGKENDVALIIDGHTLKYALSFEVRRS
FLDLALSCKAVICCRVSPLQKSEIVDVVKKRVKAITLAIGDGANDVGMIQTAHVGVGISG
NEGMQATNNSDYAIAQFSYLEKLLLVHGAWSYNRVTKCILYCFYKNVVLYIIELWFAFVN
GFSGQILFERWCIGLYNVIFTALPPFTLGIFERSCTQESMLRFPQLYKITQNGEGFNTKV
FWGHCINALVHSLILFWFPMKALEHDTVLTSGHATDYLFVGNIVYTYVVVTVCLKAGLET
TAWTKFSHLAVWGSMLTWLVFFGIYSTIWPTIPIAPDMRGQATMVLSSAHFWLGLFLVPT
ACLIEDVAWRAAKHTCKKTLLEEVQELETKSRVLGKAVLRDSNGKRLNERDRLIKRLGRK
TPPTLFRGSSLQQGVPHGYAFSQEEHGAVSQEEVIRAYDTTKKKSRKK
>sp|O15498|YKT6_HUMAN Synaptobrevin homolog YKT6 OS=Homo sapiens OX=9606 GN=YKT6 PE=1 SV=1
MKLYSLSVLYKGEAKVVLLKAAYDVSSFSFFQRSSVQEFMTFTSQLIVERSSKGTRASVK
EQDYLCHVYVRNDSLAGVVIADNEYPSRVAFTLLEKVLDEFSKQVDRIDWPVGSPATIHY
PALDGHLSRYQNPREADPMTKVQAELDETKIILHNTMESLLERGEKLDDLVSKSEVLGTQ
SKAFYKTARKQNSCCAIM
>sp|O95336|6PGL_HUMAN 6-phosphogluconolactonase OS=Homo sapiens OX=9606 GN=PGLS PE=1 SV=2

MAAPAPGLISVFSSSQELGAALAQLVAQRAACCLAGARARFALGLSGGSLVSMLARELPA
AVAPAGPASLARWTLGFCDERLVPFDHAESTYGLYRTHLLSRLPIPESQVITINPELPVE
EAAEDYAKKLRQAFQGDSIPVFDLLILGVGPDGHTCSLFPDHPLLQEREKIVAPISDSPK
PPPQRVTLTLPVLNAARTVIFVATGEGKAAVLKRILEDQEENPLPAALVQPHTGKLCWFL
DEAAARLLTVPFEKHSTL
>sp|P04264|K2C1_HUMAN Keratin, type II cytoskeletal 1 OS=Homo sapiens OX=9606 GN=KRT1 PE=1 SV=6
MSRQFSSRSGYRSGGGFSSGSAGIINYQRRTTSSSTRRSGGGGGRFSSCGGGGGSFGAGG
GFGSRSLVNLGGSKSISISVARGGGRGSGFGGGYGGGGFGGGGFGGGGFGGGGIGGGGFG
GFGSGGGGFGGGGFGGGGYGGGYGPVCPPGGIQEVTINQSLLQPLNVEIDPEIQKVKSRE
REQIKSLNNQFASFIDKVRFLEQQNQVLQTKWELLQQVDTSTRTHNLEPYFESFINNLRR
RVDQLKSDQSRLDSELKNMQDMVEDYRNKYEDEINKRTNAENEFVTIKKDVDGAYMTKVD
LQAKLDNLQQEIDFLTALYQAELSQMQTQISETNVILSMDNNRSLDLDSIIAEVKAQYED
IAQKSKAEAESLYQSKYEELQITAGRHGDSVRNSKIEISELNRVIQRLRSEIDNVKKQIS
NLQQSISDAEQRGENALKDAKNKLNDLEDALQQAKEDLARLLRDYQELMNTKLALDLEIA
TYRTLLEGEESRMSGECAPNVSVSVSTSHTTISGGGSRGGGGGGYGSGGSSYGSGGGSYG
SGGGGGGGRGSYGSGGSSYGSGGGSYGSGGGGGGHGSYGSGSSSGGYRGGSGGGGGGSSG
GRGSGGGSSGGSIGGRGSSSGGVKSSGGSSSVKFVSTTYSGVTR
>sp|P05091|ALDH2_HUMAN Aldehyde dehydrogenase, mitochondrial OS=Homo sapiens OX=9606 GN=ALDH2 PE=1 SV=2
MLRAAARFGPRLGRRLLSAAATQAVPAPNQQPEVFCNQIFINNEWHDAVSRKTFPTVNPS
TGEVICQVAEGDKEDVDKAVKAARAAFQLGSPWRRMDASHRGRLLNRLADLIERDRTYLA
ALETLDNGKPYVISYLVDLDMVLKCLRYYAGWADKYHGKTIPIDGDFFSYTRHEPVGVCG
QIIPWNFPLLMQAWKLGPALATGNVVVMKVAEQTPLTALYVANLIKEAGFPPGVVNIVPG
FGPTAGAAIASHEDVDKVAFTGSTEIGRVIQVAAGSSNLKRVTLELGGKSPNIIMSDADM
DWAVEQAHFALFFNQGQCCCAGSRTFVQEDIYDEFVERSVARAKSRVVGNPFDSKTEQGP
QVDETQFKKILGYINTGKQEGAKLLCGGGIAADRGYFIQPTVFGDVQDGMTIAKEEIFGP
VMQILKFKTIEEVVGRANNSTYGLAAAVFTKDLDKANYLSQALQAGTVWVNCYDVFGAQS
PFGGYKMSGSGRELGEYGLQAYTEVKTVTVKVPQKNS
>sp|P08758|ANXA5_HUMAN Annexin A5 OS=Homo sapiens OX=9606 GN=ANXA5 PE=1 SV=2
MAQVLRGTVTDFPGFDERADAETLRKAMKGLGTDEESILTLLTSRSNAQRQEISAAFKTL
FGRDLLDDLKSELTGKFEKLIVALMKPSRLYDAYELKHALKGAGTNEKVLTEIIASRTPE
ELRAIKQVYEEEYGSSLEDDVVGDTSGYYQRMLVVLLQANRDPDAGIDEAQVEQDAQALF
QAGELKWGTDEEKFITIFGTRSVSHLRKVFDKYMTISGFQIEETIDRETSGNLEQLLLAV
VKSIRSIPAYLAETLYYAMKGAGTDDHTLIRVMVSRSEIDLFNIRKEFRKNFATSLYSMI
KGDTSGDYKKALLLLCGEDD
>sp|P14618|KPYM_HUMAN Pyruvate kinase PKM OS=Homo sapiens OX=9606 GN=PKM PE=1 SV=4
MSKPHSEAGTAFIQTQQLHAAMADTFLEHMCRLDIDSPPITARNTGIICTIGPASRSVET
LKEMIKSGMNVARLNFSHGTHEYHAETIKNVRTATESFASDPILYRPVAVALDTKGPEIR
TGLIKGSGTAEVELKKGATLKITLDNAYMEKCDENILWLDYKNICKVVEVGSKIYVDDGL
ISLQVKQKGADFLVTEVENGGSLGSKKGVNLPGAAVDLPAVSEKDIQDLKFGVEQDVDMV
FASFIRKASDVHEVRKVLGEKGKNIKIISKIENHEGVRRFDEILEASDGIMVARGDLGIE
IPAEKVFLAQKMMIGRCNRAGKPVICATQMLESMIKKPRPTRAEGSDVANAVLDGADCIM

LSGETAKGDYPLEAVRMQHLIAREAEAAIYHLQLFEELRRLAPITSDPTEATAVGAVEAS
FKCCSGAIIVLTKSGRSAHQVARYRPRAPIIAVTRNPQTARQAHLYRGIFPVLCKDPVQE
AWAEDVDLRVNFAMNVGKARGFFKKGDVVIVLTGWRPGSGFTNTMRVVPVP
>sp|P16930|FAAA_HUMAN Fumarylacetoacetase OS=Homo sapiens OX=9606 GN=FAH PE=1 SV=2
MSFIPVAEDSDFPIHNLPYGVFSTRGDPRPRIGVAIGDQILDLSIIKHLFTGPVLSKHQD
VFNQPTLNSFMGLGQAAWKEARVFLQNLLSVSQARLRDDTELRKCAFISQASATMHLPAT
IGDYTDFYSSRQHATNVGIMFRDKENALMPNWLHLPVGYHGRASSVVVSGTPIRRPMGQM
KPDDSKPPVYGACKLLDMELEMAFFVGPGNRLGEPIPISKAHEHIFGMVLMNDWSARDIQ
KWEYVPLGPFLGKSFGTTVSPWVVPMDALMPFAVPNPKQDPRPLPYLCHDEPYTFDINLS
VNLKGEGMSQAATICKSNFKYMYWTMLQQLTHHSVNGCNLRPGDLLASGTISGPEPENFG
SMLELSWKGTKPIDLGNGQTRKFLLDGDEVIITGYCQGDGYRIGFGQCAGKVLPALLPS
>sp|P17858|PFKAL_HUMAN ATP-dependent 6-phosphofructokinase, liver type OS=Homo sapiens OX=9606 GN=PFKL PE=1 SV=6
MAAVDLEKLRASGAGKAIGVLTSGGDAQGMNAAVRAVTRMGIYVGAKVFLIYEGYEGLVE
GGENIKQANWLSVSNIIQLGGTIIGSARCKAFTTREGRRAAAYNLVQHGITNLCVIGGDG
SLTGANIFRSEWGSLLEELVAEGKISETTARTYSHLNIAGLVGSIDNDFCGTDMTIGTDS
ALHRIMEVIDAITTTAQSHQRTFVLEVMGRHCGYLALVSALASGADWLFIPEAPPEDGWE
NFMCERLGETRSRGSRLNIIIIAEGAIDRNGKPISSSYVKDLVVQRLGFDTRVTVLGHVQ
RGGTPSAFDRILSSKMGMEAVMALLEATPDTPACVVTLSGNQSVRLPLMECVQMTKEVQK
AMDDKRFDEATQLRGGSFENNWNIYKLLAHQKPPKEKSNFSLAILNVGAPAAGMNAAVRS
AVRTGISHGHTVYVVHDGFEGLAKGQVQEVGWHDVAGWLGRGGSMLGTKRTLPKGQLESI
VENIRIYGIHALLVVGGFEAYEGVLQLVEARGRYEELCIVMCVIPATISNNVPGTDFSLG
SDTAVNAAMESCDRIKQSASGTKRRVFIVETMGGYCGYLATVTGIAVGADAAYVFEDPFN
IHDLKVNVEHMTEKMKTDIQRGLVLRNEKCHDYYTTEFLYNLYSSEGKGVFDCRTNVLGH
LQQGGAPTPFDRNYGTKLGVKAMLWLSEKLREVYRKGRVFANAPDSACVIGLKKKAVAFS
PVTELKKDTDFEHRMPREQWWLSLRLMLKMLAQYRISMAAYVSGELEHVTRRTLSMDKGF
>sp|P18669|PGAM1_HUMAN Phosphoglycerate mutase 1 OS=Homo sapiens OX=9606 GN=PGAM1 PE=1 SV=2
MAAYKLVLIRHGESAWNLENRFSGWYDADLSPAGHEEAKRGGQALRDAGYEFDICFTSVQ
KRAIRTLWTVLDAIDQMWLPVVRTWRLNERHYGGLTGLNKAETAAKHGEAQVKIWRRSYD
VPPPPMEPDHPFYSNISKDRRYADLTEDQLPSCESLKDTIARALPFWNEEIVPQIKEGKR
VLIAAHGNSLRGIVKHLEGLSEEAIMELNLPTGIPIVYELDKNLKPIKPMQFLGDEETVR
KAMEAVAAQGKAKK
>sp|P22748|CAH4_HUMAN Carbonic anhydrase 4 OS=Homo sapiens OX=9606 GN=CA4 PE=1 SV=2
MRMLLALLALSAARPSASAESHWCYEVQAESSNYPCLVPVKWGGNCQKDRQSPINIVTTK
AKVDKKLGRFFFSGYDKKQTWTVQNNGHSVMMLLENKASISGGGLPAPYQAKQLHLHWSD
LPYKGSEHSLDGEHFAMEMHIVHEKEKGTSRNVKEAQDPEDEIAVLAFLVEAGTQVNEGF
QPLVEALSNIPKPEMSTTMAESSLLDLLPKEEKLRHYFRYLGSLTTPTCDEKVVWTVFRE
PIQLHREQILAFSQKLYYDKEQTVSMKDNVRPLQQLGQRTVIKSGAPGRPLPWALPALLG
PMLACLLAGFLR
>sp|P37837|TALDO_HUMAN Transaldolase OS=Homo sapiens OX=9606 GN=TALDO1 PE=1 SV=2

MSSSPVKRQRMESALDQLKQFTTVVADTGDFHAIDEYKPQDATTNPSLILAAAQMPAYQE
LVEEAIAYGRKLGGSQEDQIKNAIDKLFVLFGAEILKKIPGRVSTEVDARLSFDKDAMVA
RARRLIELYKEAGISKDRILIKLSSTWEGIQAGKELEEQHGIHCNMTLLFSFAQAVACAE
AGVTLISPFVGRILDWHVANTDKKSYEPLEDPGVKSVTKIYNYYKKFSYKTIVMGASFRN
TGEIKALAGCDFLTISPKLLGELLQDNAKLVPVLSAKAAQASDLEKIHLDEKSFRWLHNE
DQMAVEKLSDGIRKFAADAVKLERMLTERMFNAENGK
>sp|P54920|SNAA_HUMAN Alpha-soluble NSF attachment protein OS=Homo sapiens OX=9606 GN=NAPA PE=1 SV=3
MDNSGKEAEAMALLAEAERKVKNSQSFFSGLFGGSSKIEEACEIYARAANMFKMAKNWSA
AGNAFCQAAQLHLQLQSKHDAATCFVDAGNAFKKADPQEAINCLMRAIEIYTDMGRFTIA
AKHHISIAEIYETELVDIEKAIAHYEQSADYYKGEESNSSANKCLLKVAGYAALLEQYQK
AIDIYEQVGTNAMDSPLLKYSAKDYFFKAALCHFCIDMLNAKLAVQKYEELFPAFSDSRE
CKLMKKLLEAHEEQNVDSYTESVKEYDSISRLDQWLTTMLLRIKKTIQGDEEDLR
>sp|P62491|RB11A_HUMAN Ras-related protein Rab-11A OS=Homo sapiens OX=9606 GN=RAB11A PE=1 SV=3
MGTRDDEYDYLFKVVLIGDSGVGKSNLLSRFTRNEFNLESKSTIGVEFATRSIQVDGKTI
KAQIWDTAGQERYRAITSAYYRGAVGALLVYDIAKHLTYENVERWLKELRDHADSNIVIM
LVGNKSDLRHLRAVPTDEARAFAEKNGLSFIETSALDSTNVEAAFQTILTEIYRIVSQKQ
MSDRRENDMSPSNNVVPIHVPPTTENKPKVQCCQNI
>sp|P63096|GNAI1_HUMAN Guanine nucleotide-binding protein G(i) subunit alpha-1 OS=Homo sapiens OX=9606 GN=GNAI1 PE=1 SV=2
MGCTLSAEDKAAVERSKMIDRNLREDGEKAAREVKLLLLGAGESGKSTIVKQMKIIHEAG
YSEEECKQYKAVVYSNTIQSIIAIIRAMGRLKIDFGDSARADDARQLFVLAGAAEEGFMT
AELAGVIKRLWKDSGVQACFNRSREYQLNDSAAYYLNDLDRIAQPNYIPTQQDVLRTRVK
TTGIVETHFTFKDLHFKMFDVGGQRSERKKWIHCFEGVTAIIFCVALSDYDLVLAEDEEM
NRMHESMKLFDSICNNKWFTDTSIILFLNKKDLFEEKIKKSPLTICYPEYAGSNTYEEAA
AYIQCQFEDLNKRKDTKEIYTHFTCATDTKNVQFVFDAVTDVIIKNNLKDCGLF
>sp|Q01469|FABP5_HUMAN Fatty acid-binding protein 5 OS=Homo sapiens OX=9606 GN=FABP5 PE=1 SV=3
MATVQQLEGRWRLVDSKGFDEYMKELGVGIALRKMGAMAKPDCIITCDGKNLTIKTESTL
KTTQFSCTLGEKFEETTADGRKTQTVCNFTDGALVQHQEWDGKESTITRKLKDGKLVVEC
VMNNVTCTRIYEKVE
>sp|Q8IW45|NNRD_HUMAN ATP-dependent (S)-NAD(P)H-hydrate dehydratase OS=Homo sapiens OX=9606 GN=NAXD PE=1 SV=1
MVTRAGAGTAVAGAVVVALLSAALALYGPPLDAVLERAFSLRKAHSIKDMENTLQLVRNI
IPPLSSTKHKGQDGRIGVVGGCQEYTGAPYFAAISALKVGADLSHVFCASAAAPVIKAYS
PELIVHPVLDSPNAVHEVEKWLPRLHALVVGPGLGRDDALLRNVQGILEVSKARDIPVVI
DADGLWLVAQQPALIHGYRKAVLTPNHVEFSRLYDAVLRGPMDSDDSHGSVLRLSQALGN
VTVVQKGERDILSNGQQVLVCSQEGSSRRCGGQGDLLSGSLGVLVHWALLAGPQKTNGSS
PLLVAAFGACSLTRQCNHQAFQKHGRSTTTSDMIAEVGAAFSKLFET
>tr|Q562R0|Q562R0_HUMAN Actin-like protein (Fragment) OS=Homo sapiens OX=9606 GN=ACT PE=4 SV=1
KIWHHTFYNELRVAPEEHSILLTEAPLNPKANREKMTQIMFETFNTPAMYVAIQAVLSLY
TSGRTTGIVMDSGDGVTHTVPIYEGNALPHATLRLDLAGRELT

>sp|P02792|FRIL_HUMAN Ferritin light chain OS=Homo sapiens OX=9606 GN=FTL PE=1 SV=2
MSSQIRQNYSTDVEAAVNSLVNLYLQASYTYLSLGFYFDRDDVALEGVSHFFRELAEEKR
EGYERLLKMQNQRGGRALFQDIKKPAEDEWGKTPDAMKAAMALEKKLNQALLDLHALGSA
RTDPHLCDFLETHFLDEEVKLIKKMGDHLTNLHRLGGPEAGLGEYLFERLTLKHD
>sp|P04216|THY1_HUMAN Thy-1 membrane glycoprotein OS=Homo sapiens OX=9606
GN=THY1 PE=1 SV=2
MNLAISIALLLTVLQVSRGQKVTSLTACLVDQSLRLDCRHENTSSSPIQYEFSLTRETKK
HVLFGTVGVPEHTYRSRTNFTSKYNMKVLYLSAFTSKDEGTYTCALHHSGHSPPISSQNV
TVLRDKLVKCEGISLLAQNTSWLLLLLLSLSLLQATDFMSL
>sp|P09382|LEG1_HUMAN Galectin-1 OS=Homo sapiens OX=9606 GN=LGALS1 PE=1 SV=2
MACGLVASNLNLKPGECLRVRGEVAPDAKSFVLNLGKDSNNLCLHFNPRFNAHGDANTIV
CNSKDGGAWGTEQREAVFPFQPGSVAEVCITFDQANLTVKLPDGYEFKFPNRLNLEAINY
MAADGDFKIKCVAFD
>sp|P20339|RAB5A_HUMAN Ras-related protein Rab-5A OS=Homo sapiens OX=9606
GN=RAB5A PE=1 SV=2
MASRGATRPNGPNTGNKICQFKLVLLGESAVGKSSLVLRFVKGQFHEFQESTIGAAFLTQ
TVCLDDTTVKFEIWDTAGQERYHSLAPMYYRGAQAAIVVYDITNEESFARAKNWVKELQR
QASPNIVIALSGNKADLANKRAVDFQEAQSYADDNSLLFMETSAKTSMNVNEIFMAIAKK
LPKNEPQNPGANSARGRGVDLTEPTQPTRNQCCSN
>sp|P22314|UBA1_HUMAN Ubiquitin-like modifier-activating enzyme 1 OS=Homo sapiens
OX=9606 GN=UBA1 PE=1 SV=3
MSSSPLSKKRRVSGPDPKPGSNCSPAQSVLSEVPSVPTNGMAKNGSEADIDEGLYSRQLY
VLGHEAMKRLQTSSVLVSGLRGLGVEIAKNIILGGVKAVTLHDQGTAQWADLSSQFYLRE
EDIGKNRAEVSQPRLAELNSYVPVTAYTGPLVEDFLSGFQVVVLTNTPLEDQLRVGEFCH
NRGIKLVVADTRGLFGQLFCDFGEEMILTDSNGEQPLSAMVSMVTKDNPGVVTCLDEARH
GFESGDFVSFSEVQGMVELNGNQPMEIKVLGPYTFSICDTSNFSDYIRGGIVSQVKVPKK
ISFKSLVASLAEPDFVVTDFAKFSRPAQLHIGFQALHQFCAQHGRPPRPRNEEDAAELVA
LAQAVNARALPAVQQNNLDEDLIRKLAYVAAGDLAPINAFIGGLAAQEVMKACSGKFMPI
MQWLYFDALECLPEDKEVLTEDKCLQRQNRYDGQVAVFGSDLQEKLGKQKYFLVGAGAIG
CELLKNFAMIGLGCGEGGEIIVTDMDTIEKSNLNRQFLFRPWDVTKLKSDTAAAAVRQMN
PHIRVTSHQNRVGPDTERIYDDDFFQNLDGVANALDNVDARMYMDRRCVYYRKPLLESGT
LGTKGNVQVVIPFLTESYSSSQDPPEKSIPICTLKNFPNAIEHTLQWARDEFEGLFKQPA
ENVNQYLTDPKFVERTLRLAGTQPLEVLEAVQRSLVLQRPQTWADCVTWACHHWHTQYSN
NIRQLLHNFPPDQLTSSGAPFWSGPKRCPHPLTFDVNNPLHLDYVMAAANLFAQTYGLTG
SQDRAAVATFLQSVQVPEFTPKSGVKIHVSDQELQSANASVDDSRLEELKATLPSPDKLP
GFKMYPIDFEKDDDSNFHMDFIVAASNLRAENYDIPSADRHKSKLIAGKIIPAIATTTAA
VVGLVCLELYKVVQGHRQLDSYKNGFLNLALPFFGFSEPLAAPRHQYYNQEWTLWDRFEV
QGLQPNGEEMTLKQFLDYFKTEHKLEITMLSQGVSMLYSFFMPAAKLKERLDQPMTEIVS
RVSKRKLGRHVRALVLELCCNDESGEDVEVPYVRYTIR
>sp|P26998|CRBB3_HUMAN Beta-crystallin B3 OS=Homo sapiens OX=9606 GN=CRYBB3 PE=1
SV=4
MAEQHGAPEQAAAGKSHGDLGGSYKVILYELENFQGKRCELSAECPSLTDSLLEKVGSIQ
VESGPWLAFESRAFRGEQFVLEKGDYPRWDAWSNSRDSDSLLSLRPLNIDSPHHKLHLFE
NPAFSGRKMEIVDDDVPSLWAHGFQDRVASVRAINGTWVGYEFPGYRGRQYVFERGEYRH

WNEWDASQPQLQSVRRIRDQKWHKRGRFPSS
>sp|P35527|K1C9_HUMAN Keratin, type I cytoskeletal 9 OS=Homo sapiens OX=9606 GN=KRT9 PE=1 SV=3
MSCRQFSSSYLSRSGGGGGGGLGSGGSIRSSYSRFSSSGGGGGGGRFSSSSGYGGGSSRV
CGRGGGGSFGYSYGGGSGGGFSASSLGGGFGGGSRGFGGASGGGYSSSGGFGGGFGGGSG
GGFGGGYGSGFGGFGGFGGGAGGGDGGILTANEKSTMQELNSRLASYLDKVQALEEANND
LENKIQDWYDKKGPAAIQKNYSPYYNTIDDLKDQIVDLTVGNNKTLLDIDNTRMTLDDFR
IKFEMEQNLRQGVDADINGLRQVLDNLTMEKSDLEMQYETLQEELMALKKNHKEEMSQLT
GQNSGDVNVEINVAPGKDLTKTLNDMRQEYEQLIAKNRKDIENQYETQITQIEHEVSSSG
QEVQSSAKEVTQLRHGVQELEIELQSQLSKKAALEKSLEDTKNRYCGQLQMIQEQISNLE
AQITDVRQEIECQNQEYSLLLSIKMRLEKEIETYHNLLEGGQEDFESSGAGKIGLGGRGG
SGGSYGRGSRGGSGGSYGGGGSGGGYGGGSGSRGGSGGSYGGGSGSGGGSGGGYGGGSGG
GHSGGSGGGHSGGSGGNYGGGSGSGGGSGGGYGGGSGSRGGSGGSHGGGSGFGGESGGSY
GGGEEASGSGGGYGGGSGKSSHS
>sp|P50453|SPB9_HUMAN Serpin B9 OS=Homo sapiens OX=9606 GN=SERPINB9 PE=1 SV=1
METLSNASGTFAIRLLKILCQDNPSHNVFCSPVSISSALAMVLLGAKGNTATQMAQALSL
NTEEDIHRAFQSLLTEVNKAGTQYLLRTANRLFGEKTCQFLSTFKESCLQFYHAELKELS
FIRAAEESRKHINTWVSKKTEGKIEELLPGSSIDAETRLVLVNAIYFKGKWNEPFDETYT
REMPFKINQEEQRPVQMMYQEATFKLAHVGEVRAQLLELPYARKELSLLVLLPDDGVELS
TVEKSLTFEKLTAWTKPDCMKSTEVEVLLPKFKLQEDYDMESVLRHLGIVDAFQQGKADL
SAMSAERDLCLSKFVHKSFVEVNEEGTEAAAASSCFVVAECCMESGPRFCADHPFLFFIR
HNRANSILFCGRFSSP
>sp|P62828|RAN_RAT GTP-binding nuclear protein Ran OS=Rattus norvegicus OX=10116 GN=Ran PE=1 SV=3
MAAQGEPQVQFKLVLVGDGGTGKTTFVKRHLTGEFEKKYVATLGVEVHPLVFHTNRGPIK
FNVWDTAGQEKFGGLRDGYYIQAQCAIIMFDVTSRVTYKNVPNWHRDLVRVCENIPIVLC
GNKVDIKDRKVKAKSIVFHRKKNLQYYDISAKSNYNFEKPFLWLARKLIGDPNLEFVAMP
ALAPPEVVMDPALAAQYEHDLEVAQTTALPDEDDDL
>sp|Q13057|COASY_HUMAN Bifunctional coenzyme A synthase OS=Homo sapiens OX=9606 GN=COASY PE=1 SV=4
MAVFRSGLLVLTTPLASLAPRLASILTSAARLVNHTLYVHLQPGMSLEGPAQPQSSPVQA
TFEVLDFITHLYAGADVHRHLDVRILLTNIRTKSTFLPPLPTSVQNLAHPPEVVLTDFQT
LDGSQYNPVKQQLVRYATSCYSCCPRLASVLLYSDYGIGEVPVEPLDVPLPSTIRPASPV
AGSPKQPVRGYYRGAVGGTFDRLHNAHKVLLSVACILAQEQLVVGVADKDLLKSKLLPEL
LQPYTERVEHLSEFLVDIKPSLTFDVIPLLDPYGPAGSDPSLEFLVVSEETYRGGMAINR
FRLENDLEELALYQIQLLKDLRHTENEEDKVSSSSFRQRMLGNLLRPPYERPELPTCLYV
IGLTGISGSGKSSIAQRLKGLGAFVIDSDHLGHRAYAPGGPAYQPVVEAFGTDILHKDGI
INRKVLGSRVFGNKKQLKILTDIMWPIIAKLAREEMDRAVAEGKRVCVIDAAVLLEAGWQ
NLVHEVWTAVIPETEAVRRIVERDGLSEAAAQSRLQSQMSGQQLVEQSHVVLSTLWEPHI
TQRQVEKAWALLQKRIPKTHQALD
>sp|Q14974|IMB1_HUMAN Importin subunit beta-1 OS=Homo sapiens OX=9606 GN=KPNB1 PE=1 SV=2
MELITILEKTVSPDRLELEAAQKFLERAAVENLPTFLVELSRVLANPGNSQVARVAAGLQ
IKNSLTSKDPDIKAQYQQRWLAIDANARREVKNYVLQTLGTETYRPSSASQCVAGIACAE

IPVNQWPELIPQLVANVTNPNSTEHMKESTLEAIGYICQDIDPEQLQDKSNEILTAIIQG
MRKEEPSNNVKLAATNALLNSLEFTKANFDKESERHFIMQVVCEATQCPDTRVRVAALQN
LVKIMSLYYQYMETYMGPALFAITIEAMKSDIDEVALQGIEFWSNVCDEEMDLAIEASEA
AEQGRPPEHTSKFYAKGALQYLVPILTQTLTKQDENDDDDDWNPCKAAGVCLMLLATCCE
DDIVPHVLPFIKEHIKNPDWRYRDAAVMAFGCILEGPEPSQLKPLVIQAMPTLIELMKDP
SVVVRDTAAWTVGRICELLPEAAINDVYLAPLLQCLIEGLSAEPRVASNVCWAFSSLAEA
AYEAADVADDQEEPATYCLSSSFELIVQKLLETTDRPDGHQNNLRSSAYESLMEIVKNSA
KDCYPAVQKTTLVIMERLQQVLQMESHIQSTSDRIQFNDLQSLLCATLQNVLRKVQHQDA
LQISDVVMASLLRMFQSTAGSGGVQEDALMAVSTLVEVLGGEFLKYMEAFKPFLGIGLKN
YAEYQVCLAAVGLVGDLCRALQSNIIPFCDEVMQLLLENLGNENVHRSVKPQILSVFGDI
ALAIGGEFKKYLEVVLNTLQQASQAQVDKSDYDMVDYLNELRESCLEAYTGIVQGLKGDQ
ENVHPDVMLVQPRVEFILSFIDHIAGDEDHTDGVVACAAGLIGDLCTAFGKDVLKLVEAR
PMIHELLTEGRRSKTNKAKTLATWATKELRKLKNQA
>sp|Q5T9C9|PI5L1_HUMAN Phosphatidylinositol 4-phosphate 5-kinase-like protein 1
OS=Homo sapiens OX=9606 GN=PIP5KL1 PE=2 SV=2
MAAPSPGPREVLAPSPEAGCRAVTSSRRGLLWRLRDKQSRLGLFEISPGHELHGMTCMMQ
AGLWAATQVSMDHPPTGPPSRDDFSEVLTQVHEGFELGTLAGPAFAWLRRSLGLAEEDYQ
AALGPGGPYLQFLSTSKSKASFFLSHDQRFFLKTQGRREVQALLAHLPRYVQHLQRHPHS
LLARLLGVHSLRVDRGKKTYFIVMQSVFYPAGRISERYDIKGCEVSRWVDPAPEGSPLVL
VLKDLNFQGKTINLGPQRSWFLRQMELDTTFLRELNVLDYSLLIAFQRLHEDERGPGSSL
IFRTARSVQGAQSPEESRAQNRRLLPDAPNALHILDGPEQRYFLGVVDLATVYGLRKRLE
HLWKTLRYPGRTFSTVSPARYARRLCQWVEAHTE
>sp|Q8NF91|SYNE1_HUMAN Nesprin-1 OS=Homo sapiens OX=9606 GN=SYNE1 PE=1 SV=4
MATSRGASRCPRDIANVMQRLQDEQEIVQKRTFTKWINSHLAKRKPPMVVDDLFEDMKDG
VKLLALLEVLSGQKLPCEQGRRMKRIHAVANIGTALKFLEGRKIKLVNINSTDIADGRPS
IVLGLMWTIILYFQIEELTSNLPQLQSLSSSASSVDSIVSSETPSPPSKRKVTTKIQGNA
KKALLKWVQYTAGKQTGIEVKDFGKSWRSGVAFHSVIHAIRPELVDLETVKGRSNRENLE
DAFTIAETELGIPRLLDPEDVDVDKPDEKSIMTYVAQFLKHYPDIHNASTDGQEDDEILP
GFPSFANSVQNFKREDRVIFKEMKVWIEQFERDLTRAQMVESNLQDKYQSFKHFRVQYEM
KRKQIEHLIQPLHRDGKLSLDQALVKQSWDRVTSRLFDWHIQLDKSLPAPLGTIGAWLYR
AEVALREEITVQQVHEETANTIQRKLEQHKDLLQNTDAHKRAFHEIYRTRSVNGIPVPPD
QLEDMAERFHFVSSTSELHLMKMEFLELKYRLLSLLVLAESKLKSWIIKYGRRESVEQLL
QNYVSFIENSKFFEQYEVTYQILKQTAEMYVKADGSVEEAENVMKFMNETTAQWRNLSVE
VRSVRSMLEEVISNWDRYGNTVASLQAWLEDAEKMLNQSENAKKDFFRNLPHWIQQHTAM
NDAGNFLIETCDEMVSRDLKQQLLLLNGRWRELFMEVKQYAQADEMDRMKKEYTDCVVTL
SAFATEAHKKLSEPLEVSFMNVKLLIQDLEDIEQRVPVMDAQYKIITKTAHLITKESPQE
EGKEMFATMSKLKEQLTKVKECYSPLLYESQQLLIPLEELEKQMTSFYDSLGKINEIITV
LEREAQSSALFKQKHQELLACQENCKKTLTLIEKGSQSVQKFVTLSNVLKHFDQTRLQRQ
IADIHVAFQSMVKKTGDWKKHVETNSRLMKKFEESRAELEKVLRIAQEGLEEKGDPEELL
RRHTEFFSQLDQRVLNAFLKACDELTDILPEQEQQGLQEAVRKLHKQWKDLQGEAPYHLL
HLKIDVEKNRFLASVEECRTELDRETKLMPQEGSEKIIKEHRVFFSDKGPHHLCEKRLQL
IEELCVKLPVRDPVRDTPGTCHVTLKELRAAIDSTYRKLMEDPDKWKDYTSRFSEFSSWI
STNETQLKGIKGEAIDTANHGEVKRAVEEIRNGVTKRGETLSWLKSRLKVLTEVSSENEA
QKQGDELAKLSSSFKALVTLLSEVEKMLSNFGDCVQYKEIVKNSLEELISGSKEVQEQAE

KILDTENLFEAQQLLLHHQQKTKRISAKKRDVQQQIAQAQQGEGGLPDRGHEELRKLEST
LDGLERSRERQERRIQVTLRKWERFETNKETVVRYLFQTGSSHERFLSFSSLESLSSELE
QTKEFSKRTESIAVQAENLVKEASEIPLGPQNKQLLQQQAKSIKEQVKKLEDTLEEDIKT
MEMVKTKWDHFGSNFETLSVWITEKEKELNALETSSSAMDMQISQIKVTIQEIESKLSSI
VGLEEEAQSFAQFVTTGESARIKAKLTQIRRYGEELREHAQCLEGTILGHLSQQQKFEEN
LRKIQQSVSEFEDKLAVPIKICSSATETYKVLQEHMDLCQALESLSSAITAFSASARKVV
NRDSCVQEAAALQQQYEDILRRAKERQTALENLLAHWQRLEKELSSFLTWLERGEAKASS
PEMDISADRVKVEGELQLIQALQNEVVSQASFYSKLLQLKESLFSVASKDDVKMMKLHLE
QLDERWRDLPQIINKRINFLQSVVAEHQQFDELLLSFSVWIKLFLSELQTTSEISIMDHQ
VALTRHKDHAAEVESKKGELQSLQGHLAKLGSLGRAEDLHLLQGKAEDCFQLFEEASQVV
ERRQLALSHLAEFLQSHASLSGILRQLRQTVEATNSMNKNESDLIEKDLNDALQNAKALE
SAAVSLDGILSKAQYHLKIGSSEQRTSCRATADQLCGEVERIQNLLGTKQSEADALAVLK
KAFQDQKEELLKSIEDIEERTDKERLKEPTRQALQQRLRVFNQLEDELNSHEHELCWLKD
KAKQIAQKDVAFAPEVDREINRLEVTWDDTKRLIHENQGQCCGLIDLMREYQNLKSAVSK
VLENASSVIVTRTTIKDQEDLKWAFSKHETAKNKMNYKQKDLDNFTSKGKHLLSELKKIH
SSDFSLVKTDMESTVDKWLDVSEKLEENMDRLRVSLSIWDDVLSTRDEIEGWSNNCVPQM
AENISNLDNHLRAEELLKEFESEVKNKALRLEELHSKVNDLKELTKNLETPPDLQFIEAD
LMQKLEHAKEITEVAKGTLKDFTAQSTQVEKFINDITTWFTKVEESLMNCAQNETCEALK
KVKDIQKELQSQQSNISSTQENLNSLCRKYHSAELESLGRAMTGLIKKHEAVSQLCSKTQ
ASLQESLEKHFSESMQEFQEWFLGAKAAAKESSDRTGDSKVLEAKLHDLQNILDSVSDGQ
SKLDAVTQEGQTLYAHLSKQIVSSIQEQITKANEEFQAFLKQCLKDKQALQDCASELGSF
EDQHRKLNLWIHEMEERFNTENLGESKQHIPEKKNEVHKVEMFLGELLAARESLDKLSQR
GQLLSEEGHGAGQEGRLCSQLLTSHQNLLRMTKEKLRSCQVALQEHEALEEALQSMWFWV
KAIQDRLACAESTLGSKDTLEKRLSQIQDILLMKGEGEVKLNMAIGKGEQALRSSNKEGQ
RVIQTQLETLKEVWADIMSSSVHAQSTLESVISQWNDYVERKNQLEQWMESVDQKIEHPL
QPQPGLKEKFVLLDHLQSILSEAEDHTRALHRLIAKSRELYEKTEDESFKDTAQEELKTQ
FNDIMTVAKEKMRKVEEIVKDHLMYLDAVHEFTDWLHSAKEELHRWSDMSGDSSATQKKL
SKIKELIDSREIGASRLSRVESLAPEVKQNTTASGCELMHTEMQALRADWKQWEDSVFQT
QSCLENLVSQMALSEQEFSGQVAQLEQALEQFSALLKTWAQQLTLLEGKNTDEEIVECWH
KGQEILDALQKAEPRTEDLKSQLNELCRFSRDLSTYSGKVSGLIKEYNCLCLQASKGCQN
KEQILQQRFRKAFRDFQQWLVNAKITTAKCFDIPQNISEVSTSLQKIQEFLSESENGQHK
LNMMLSKGELLSTLLTKEKAKGIQAKVTAAKEDWKNFHSNLHQKESALENLKIQMKDFEV
SAEPIQDWLSKTEKMVHESSNRLYDLPAKRREQQKLQSVLEEIHCYEPQLNRLKEKAQQL
WEGQAASKSFRHRVSQLSSQYLALSNLTKEKVSRLDRIVAEHNQFSLGIKELQDWMTDAI
HMLDSYCHPTSDKSVLDSRTLKLEALLSVKQEKEIQMKMIVTRGESVLQNTSPEGIPTIQ
QQLQSVKDMWASLLSAGIRCKSQLEGALSKWTSYQDGVRQFSGWMDSMEANLNESERQHA
ELRDKTTMLGKAKLLNEEVLSYSSLLETIEVKGAGMTEHYVTQLELQDLQERYRAIQERA
KEAVTKSEKLVRLHQEYQRDLKAFEVWLGQEQEKLDQYSVLEGDAHTHETTLRDLQELQV
HCAEGQALLNSVLHTREDVIPSGIPQAEDRALESLRQDWQAYQHRLSETRTQFNNVVNKL
RLMEQKFQQVDEWLKTAEEKVSPRTRRQSNRATKEIQLHQMKKWHEEVTAYRDEVEEVGA
RAQEILDESHVNSRMGCQATQLTSRYQALLLQVLEQIKFLEEEIQSLEESESSLSSYSDW
YGSTHKNFKNVATKIDKVDTVMMGKKLKTLEVLLKDMEKGHSLLKSAREKGERAVKYLEE
GEAERLRKEIHDHMEQLKELTSTVRKEHMTLEKGLHLAKEFSDKCKALTQWIAEYQEILH
VPEEPKMELYEKKAQLSKYKSLQQTVLSHEPSVKSVREKGEALLELVQDVTLKDKIDQLQ

SDYQDLCSIGKEHVFSLEAKVKDHEDYNSELQEVEKWLLQMSGRLVAPDLLETSSLETIT
QQLAHHKAMMEEIAGFEDRLNNLQMKGDTLIGQCADHLQAKLKQNVHAHLQGTKDSYSAI
CSTAQRMYQSLEHELQKHVSRQDTLQQCQAWLSAVQPDLEPSPQPPLSRAEAIKQVKHFR
ALQEQARTYLDLLCSMCDLSNASVKTTAKDIQQTEQTIEQKLVQAQNLTQGWEEIKHLKS
ELWIYLQDADQQLQNMKRRHSELELNIAQNMVSQVKDFVKKLQSKQASVNTIIEKVNKLT
KKEESPEHKEINHLNDQWLDLCRQSNNLCLQREEDLQRTRDYHDCMNVVEVFLEKFTTEW
DNLARSDAESTAVHLEALKKLALALQERKYAIEDLKDQKQKMIEHLNLDDKELVKEQTSH
LEQRWFQLEDLIKRKIQVSVTNLEELNVVQSRFQELMEWAEEQQPNIAEALKQSPPPDMA
QNLLMDHLAICSELEAKQMLLKSLIKDADRVMADLGLNERQVIQKALSDAQSHVNCLSDL
VGQRRKYLNKALSEKTQFLMAVFQATSQIQQHERKIMFREHICLLPDDVSKQVKTCKSAQ
ASLKTYQNEVTGLWAQGRELMKEVTEQEKSEVLGKLQELQSVYDSVLQKCSHRLQELEKN
LVSRKHFKEDFDKACHWLKQADIVTFPEINLMNESSELHTQLAKYQNILEQSPEYENLLL
TLQRTGQTILPSLNEVDHSYLSEKLNALPRQFNVIVALAKDKFYKVQEAILARKEYASLI
ELTTQSLSELEAQFLRMSKVPTDLAVEEALSLQDGCRAILDEVAGLGEAVDELNQKKEGF
RSTGQPWQPDKMLHLVTLYHRLKRQTEQRVSLLEDTTSAYQEHEKMCQQLERQLKSVKEE
QSKVNEETLPAEEKLKMYHSLAGSLQDSGIVLKRVTIHLEDLAPHLDPLAYEKARHQIQS
WQGELKLLTSAIGETVTECESRMVQSIDFQTEMSRSLDWLRRVKAELSGPVYLDLNLQDI
QEEIRKIQIHQEEVQSSLRIMNALSHKEKEKFTKAKELISADLEHSLAELSELDGDIQEA
LRTRQATLTEIYSQCQRYYQVFQAANDWLEDAQELLQLAGNGLDVESAEENLKSHMEFFS
TEDQFHSNLEELHSLVATLDPLIKPTGKEDLEQKVASLELRSQRMSRDSGAQVDLLQRCT
AQWHDYQKAREEVIELMNDTEKKLSEFSLLKTSSSHEAEEKLSEHKALVSVVNSFHEKIV
ALEEKASQLEKTGNDASKATLSRSMTTVWQRWTRLRAVAQDQEKILEDAVDEWTGFNNKV
KKATEMIDQLQDKLPGSSAEKASKAELLTLLEYHDTFVLELEQQQSALGMLRQQTLSMLQ
DGAAPTPGEEPPLMQEITAMQDRCLNMQEKVKTNGKLVKQELKDREMVETQINSVKCWVQ
ETKEYLGNPTIEIDAQLEELQILLTEATNHRQNIEKMAEEQKEKYLGLYTILPSELSLQL
AEVALDLKIRDQIQDKIKEVEQSKATSQELSRQIQKLAKDLTTILTKLKAKTDNVVQAKT
DQKVLGEELDGCNSKLMELDAAVQKFLEQNGQLGKPLAKKIGKLTELHQQTIRQAENRLS
KLNQAASHLEEYNEMLELILKWIEKAKVLAHGTIAWNSASQLREQYILHQTLLEESKEID
SELEAMTEKLQYLTSVYCTEKMSQQVAELGRETEELRQMIKIRLQNLQDAAKDMKKFEAE
LKKLQAALEQAQATLTSPEVGRLSLKEQLSHRQHLLSEMESLKPKVQAVQLCQSALRIPE
DVVASLPLCHAALRLQEEASRLQHTAIQQCNIMQEAVVQYEQYEQEMKHLQQLIEGAHRE
IEDKPVATSNIQELQAQISRHEELAQKIKGYQEQIASLNSKCKMLTMKAKHATMLLTVTE
VEGLAEGTEDLDGELLPTPSAHPSVVMMTAGRCHTLLSPVTEESGEEGTNSEISSPPACR
SPSPVANTDASVNQDIAYYQALSAERLQTDAAKIHPSTSASQEFYEPGLEPSATAKLGDL
QRSWETLKNVISEKQRTLYEALERQQKYQDSLQSISTKMEAIELKLSESPEPGRSPESQM
AEHQALMDEILMLQDEINELQSSLAEELVSESCEADPAEQLALQSTLTVLAERMSTIRMK
ASGKRQLLEEKLNDQLEEQRQEQALQRYRCEADELDSWLLSTKATLDTALSPPKEPMDME
AQLMDCQNMLVEIEQKVVALSELSVHNENLLLEGKAHTKDEAEQLAGKLRRLKGSLLELQ
RALHDKQLNMQGTAQEKEESDVDLTATQSPGVQEWLAQARTTWTQQRQSSLQQQKELEQE
LAEQKSLLRSVASRGEEILIQHSAAETSGDAGEKPDVLSQELGMEGEKSSAEDQMRMKWE
SLHQEFSTKQKLLQNVLEQEQEQVLYSRPNRLLSGVPLYKGDVPTQDKSAVTSLLDGLNQ
AFEEVSSQSGGAKRQSIHLEQKLYDGVSATSTWLDDVEERLFVATALLPEETETCLFNQE
ILAKDIKEMSEEMDKNKNLFSQAFPENGDNRDVIEDTLGCLLGRLSLLDSVVNQRCHQMK
ERLQQILNFQNDLKVLFTSLADNKYIILQKLANVFEQPVAEQIEAIQQAEDGLKEFDAGI

IELKRRGDKLQVEQPSMQELSKLQDMYDELMMIIGSRRSGLNQNLTLKSQYERALQDLAD
LLETGQEKMAGDQKIIVSSKEEIQQLLDKHKEYFQGLESHMILTETLFRKIISFAVQKET
QFHTELMAQASAVLKRAHKRGVELEYILETWSHLDEDQQELSRQLEVVESSIPSVGLVEE
NEDRLIDRITLYQHLKSSLNEYQPKLYQVLDDGKRLLISISCSDLESQLNQLGECWLSNT
NKMSKELHRLETILKHWTRYQSESADLIHWLQSAKDRLEFWTQQSVTVPQELEMVRDHLN
AFLEFSKEVDAQSSLKSSVLSTGNQLLRLKKVDTATLRSELSRIDSQWTDLLTNIPAVQE
KLHQLQMDKLPSRHAISEVMSWISLMENVIQKDEDNIKNSIGYKAIHEYLQKYKGFKIDI
NCKQLTVDFVNQSVLQISSQDVESKRSDKTDFAEQLGAMNKSWQILQGLVTEKIQLLEGL
LESWSEYENNVQCLKTWFETQEKRLKQQHRIGDQASVQNALKDCQDLEDLIKAKEKEVEK
IEQNGLALIQNKKEDVSSIVMSTLRELGQTWANLDHMVGQLKILLKSVLDQWSSHKVAFD
KINSYLMEARYSLSRFRLLTGSLEAVQVQVDNLQNLQDDLEKQERSLQKFGSITNQLLKE
CHPPVTETLTNTLKEVNMRWNNLLEEIAEQLQSSKALLQLWQRYKDYSKQCASTVQQQED
RTNELLKAATNKDIADDEVATWIQDCNDLLKGLGTVKDSLFFLHELGEQLKQQVDASAAS
AIQSDQLSLSQHLCALEQALCKQQTSLQAGVLDYETFAKSLEALEAWIVEAEEILQGQDP
SHSSDLSTIQERMEELKGQMLKFSSMAPDLDRLNELGYRLPLNDKEIKRMQNLNRHWSLI
SSQTTERFSKLQSFLLQHQTFLEKCETWMEFLVQTEQKLAVEISGNYQHLLEQQRAHELF
QAEMFSRQQILHSIIIDGQRLLEQGQVDDRDEFNLKLTLLSNQWQGVIRRAQQRRGIIDS
QIRQWQRYREMAEKLRKWLVEVSYLPMSGLGSVPIPLQQARTLFDEVQFKEKVFLRQQGS
YILTVEAGKQLLLSADSGAEAALQAELAEIQEKWKSASMRLEEQKKKLAFLLKDWEKCEK
GIADSLEKLRTFKKKLSQSLPDHHEELHAEQMRCKELENAVGSWTDDLTQLSLLKDTLSA
YISADDISILNERVELLQRQWEELCHQLSLRRQQIGERLNEWAVFSEKNKELCEWLTQME
SKVSQNGDILIEEMIEKLKKDYQEEIAIAQENKIQLQQMGERLAKASHESKASEIEYKLG
KVNDRWQHLLDLIAARVKKLKETLVAVQQLDKNMSSLRTWLAHIESELAKPIVYDSCNSE
EIQRKLNEQQELQRDIEKHSTGVASVLNLCEVLLHDCDACATDAECDSIQQATRNLDRRW
RNICAMSMERRLKIEETWRLWQKFLDDYSRFEDWLKSSERTAAFPSSSGVIYTVAKEELK
KFEAFQRQVHECLTQLELINKQYRRLARENRTDSACSLKQMVHEGNQRWDNLQKRVTSIL
RRLKHFIGQREEFETARDSILVWLTEMDLQLTNIEHFSECDVQAKIKQLKAFQQEISLNH
NKIEQIIAQGEQLIEKSEPLDAAIIEEELDELRRYCQEVFGRVERYHKKLIRLPLPDDEH
DLSDRELELEDSAALSDLHWHDRSADSLLSPQPSSNLSLSLAQPLRSERSGRDTPASVDS
IPLEWDHDYDLSRDLESAMSRALPSEDEEGQDDKDFYLRGAVGLSGDHSALESQIRQLGK
ALDDSRFQIQQTENIIRSKTPTGPELDTSYKGYMKLLGECSSSIDSVKRLEHKLKEEEES
LPGFVNLHSTETQTAGVIDRWELLQAQALSKELRMKQNLQKWQQFNSDLNSIWAWLGDTE
EELEQLQRLELSTDIQTIELQIKKLKELQKAVDHRKAIILSINLCSPEFTQADSKESRDL
QDRLSQMNGRWDRVCSLLEEWRGLLQDALMQCQGFHEMSHGLLLMLENIDRRKNEIVPID
SNLDAEILQDHHKQLMQIKHELLESQLRVASLQDMSCQLLVNAEGTDCLEAKEKVHVIGN
RLKLLLKEVSRHIKELEKLLDVSSSQQDLSSWSSADELDTSGSVSPTSGRSTPNRQKTPR
GKCSLSQPGPSVSSPHSRSTKGGSDSSLSEPGPGRSGRGFLFRVLRAALPLQLLLLLLIG
LACLVPMSEEDYSCALSNNFARSFHPMLRYTNGPPPL
>sp|Q99832|TCPH_HUMAN T-complex protein 1 subunit eta OS=Homo sapiens OX=9606
GN=CCT7 PE=1 SV=2
MMPTPVILLKEGTDSSQGIPQLVSNISACQVIAEAVRTTLGPRGMDKLIVDGRGKATISN
DGATILKLLDVVHPAAKTLVDIAKSQDAEVGDGTTSVTLLAAEFLKQVKPYVEEGLHPQI
IIRAFRTATQLAVNKIKEIAVTVKKADKVEQRKLLEKCAMTALSSKLISQQKAFFAKMVV
DAVMMLDDLLQLKMIGIKKVQGGALEDSQLVAGVAFKKTFSYAGFEMQPKKYHNPKIALL

NVELELKAEKDNAEIRVHTVEDYQAIVDAEWNILYDKLEKIHHSGAKVVLSKLPIGDVAT
QYFADRDMFCAGRVPEEDLKRTMMACGGSIQTSVNALSADVLGRCQVFEETQIGGERYNF
FTGCPKAKTCTFILRGGAEQFMEETERSLHDAIMIVRRAIKNDSVVAGGGAIEMELSKYL
RDYSRTIPGKQQLLIGAYAKALEIIPRQLCDNAGFDATNILNKLRARHAQGGTWYGVDIN
NEDIADNFEAFVWEPAMVRINALTAASEAACLIVSVDETIKNPRSTVDAPTAAGRGRGRG
RPH
>sp|Q9BXM0|PRAX_HUMAN Periaxin OS=Homo sapiens OX=9606 GN=PRX PE=1 SV=2
MEARSRSAEELRRAELVEIIVETEAQTGVSGINVAGGGKEGIFVRELREDSPAARSLSLQ
EGDQLLSARVFFENFKYEDALRLLQCAEPYKVSFCLKRTVPTGDLALRPGTVSGYEIKGP
RAKVAKLNIQSLSPVKKKKMVPGALGVPADLAPVDVEFSFPKFSRLRRGLKAEAVKGPVP
AAPARRRLQLPRLRVREVAEEAQAARLAAAAPPPRKAKVEAEVAAGARFTAPQVELVGPR
LPGAEVGVPQVSAPKAAPSAEAAGGFALHLPTLGLGAPAPPAVEAPAVGIQVPQVELPAL
PSLPTLPTLPCLETREGAVSVVVPTLDVAAPTVGVDLALPGAEVEARGEAPEVALKMPRL
SFPRFGARAKEVAEAKVAKVSPEARVKGPRLRMPTFGLSLLEPRPAAPEVVESKLKLPTI
KMPSLGIGVSGPEVKVPKGPEVKLPKAPEVKLPKVPEAALPEVRLPEVELPKVSEMKLPK
VPEMAVPEVRLPEVELPKVSEMKLPKVPEMAVPEVRLPEVQLLKVSEMKLPKVPEMAVPE
VRLPEVQLPKVSEMKLPEVSEVAVPEVRLPEVQLPKVPEMKVPEMKLPKVPEMKLPEMKL
PEVQLPKVPEMAVPDVHLPEVQLPKVPEMKLPEMKLPEVKLPKVPEMAVPDVHLPEVQLP
KVPEMKLPKMPEMAVPEVRLPEVQLPKVSEMKLPKVPEMAVPDVHLPEVQLPKVCEMKVP
DMKLPEIKLPKVPEMAVPDVHLPEVQLPKVSEIRLPEMQVPKVPDVHLPKAPEVKLPRAP
EVQLKATKAEQAEGMEFGFKMPKMTMPKLGRAESPSRGKPGEAGAEVSGKLVTLPCLQPE
VDGEAHVGVPSLTLPSVELDLPGALGLQGQVPAAKMGKGERVEGPEVAAGVREVGFRVPS
VEIVTPQLPAVEIEEGRLEMIETKVKPSSKFSLPKFGLSGPKVAKAEAEGAGRATKLKVS
KFAISLPKARVGAEAEAKGAGEAGLLPALDLSIPQLSLDAHLPSGKVEVAGADLKFKGPR
FALPKFGVRGRDTEAAELVPGVAELEGKGWGWDGRVKMPKLKMPSFGLARGKEAEVQGDR
ASPGEKAESTAVQLKIPEVELVTLGAQEEGRAEGAVAVSGMQLSGLKVSTAGQVVTEGHD
AGLRMPPLGISLPQVELTGFGEAGTPGQQAQSTVPSAEGTAGYRVQVPQVTLSLPGAQVA
GGELLVGEGVFKMPTVTVPQLELDVGLSREAQAGEAATGEGGLRLKLPTLGARARVGGEG
AEEQPPGAERTFCLSLPDVELSPSGGNHAEYQVAEGEGEAGHKLKVRLPRFGLVRAKEGA
EEGEKAKSPKLRLPRVGFSQSEMVTGEGSPSPEEEEEEEEEGSGEGASGRRGRVRVRLPR
VGLAAPSKASRGQEGDAAPKSPVREKSPKFRFPRVSLSPKARSGSGDQEEGGLRVRLPSV
GFSETGAPGPARMEGAQAAAV
>sp|Q9NUQ9|CYRIB_HUMAN CYFIP-related Rac1 interactor B OS=Homo sapiens OX=9606
GN=CYRIB PE=1 SV=1
MGNLLKVLTCTDLEQGPNFFLDFENAQPTESEKEIYNQVNVVLKDAEGILEDLQSYRGAG
HEIREAIQHPADEKLQEKAWGAVVPLVGKLKKFYEFSQRLEAALRGLLGALTSTPYSPTQ
HLEREQALAKQFAEILHFTLRFDELKMTNPAIQNDFSYYRRTLSRMRINNVPAEGENEVN
NELANRMSLFYAEATPMLKTLSDATTKFVSENKNLPIENTTDCLSTMASVCRVMLETPEY
RSRFTNEETVSFCLRVMVGVIILYDHVHPVGAFAKTSKIDMKGCIKVLKDQPPNSVEGLL
NALRYTTKHLNDETTSKQIKSMLQ
>sp|Q9P273|TEN3_HUMAN Teneurin-3 OS=Homo sapiens OX=9606 GN=TENM3 PE=1 SV=3
MDVKERRPYCSLTKSRREKERRYTNSSADNEECRVPTQKSYSSSETLKAFDHDSSRLLYG
NRVKDLVHREADEFTRQGQNFTLRQLGVCEPATRRGLAFCAEMGLPHRGYSISAGSDADT
ENEAVMSPEHAMRLWGRGVKSGRSSCLSSRSNSALTLTDTEHENKSDSENEQPASNQGQS

TLQPLPPSHKQHSAQHHPSITSLNRNSLTNRRNQSPAPPAALPAELQTTPESVQLQDSWV
LGSNVPLESRHFLFKTGTGTTPLFSTATPGYTMASGSVYSPPTRPLPRNTLSRSAFKFKK
SSKYCSWKCTALCAVGVSVLLAILLSYFIAMHLFGLNWQLQQTENDTFENGKVNSDTMPT
NTVSLPSGDNGKLGGFTQENNTIDSGELDIGRRAIQEIPPGIFWRSQLFIDQPQFLKFNI
SLQKDALIGVYGRKGLPPSHTQYDFVELLDGSRLIAREQRSLLETERAGRQARSVSLHEA
GFIQYLDSGIWHLAFYNDGKNAEQVSFNTIVIESVVECPRNCHGNGECVSGTCHCFPGFL
GPDCSRAACPVLCSGNGQYSKGRCLCFSGWKGTECDVPTTQCIDPQCGGRGICIMGSCAC
NSGYKGESCEEADCIDPGCSNHGVCIHGECHCSPGWGGSNCEILKTMCPDQCSGHGTYLQ
ESGSCTCDPNWTGPDCSNEICSVDCGSHGVCMGGTCRCEEGWTGPACNQRACHPRCAEHG
TCKDGKCECSQGWNGEHCTIEGCPGLCNSNGRCTLDQNGWHCVCQPGWRGAGCDVAMETL
CTDSKDNEGDGLIDCMDPDCCLQSSCQNQPYCRGLPDPQDIISQSLQSPSQQAAKSFYDR
ISFLIGSDSTHVIPGESPFNKSLASVIRGQVLTADGTPLIGVNVSFFHYPEYGYTITRQD
GMFDLVANGGASLTLVFERSPFLTQYHTVWIPWNVFYVMDTLVMKKEENDIPSCDLSGFV
RPNPIIVSSPLSTFFRSSPEDSPIIPETQVLHEETTIPGTDLKLSYLSSRAAGYKSVLKI
TMTQSIIPFNLMKVHLMVAVVGRLFQKWFPASPNLAYTFIWDKTDAYNQKVYGLSEAVVS
VGYEYESCLDLTLWEKRTAILQGYELDASNMGGWTLDKHHVLDVQNGILYKGNGENQFIS
QQPPVVSSIMGNGRRRSISCPSCNGQADGNKLLAPVALACGIDGSLYVGDFNYVRRIFPS
GNVTSVLELSSNPAHRYYLATDPVTGDLYVSDTNTRRIYRPKSLTGAKDLTKNAEVVAGT
GEQCLPFDEARCGDGGKAVEATLMSPKGMAVDKNGLIYFVDGTMIRKVDQNGIISTLLGS
NDLTSARPLTCDTSMHISQVRLEWPTDLAINPMDNSIYVLDNNVVLQITENRQVRIAAGR
PMHCQVPGVEYPVGKHAVQTTLESATAIAVSYSGVLYITETDEKKINRIRQVTTDGEISL
VAGIPSECDCKNDANCDCYQSGDGYAKDAKLSAPSSLAASPDGTLYIADLGNIRIRAVSK
NKPLLNSMNFYEVASPTDQELYIFDINGTHQYTVSLVTGDYLYNFSYSNDNDITAVTDSN
GNTLRIRRDPNRMPVRVVSPDNQVIWLTIGTNGCLKSMTAQGLELVLFTYHGNSGLLATK
SDETGWTTFFDYDSEGRLTNVTFPTGVVTNLHGDMDKAITVDIESSSREEDVSITSNLSS
IDSFYTMVQDQLRNSYQIGYDGSLRIIYASGLDSHYQTEPHVLAGTANPTVAKRNMTLPG
ENGQNLVEWRFRKEQAQGKVNVFGRKLRVNGRNLLSVDFDRTTKTEKIYDDHRKFLLRIA
YDTSGHPTLWLPSSKLMAVNVTYSSTGQIASIQRGTTSEKVDYDGQGRIVSRVFADGKTW
SYTYLEKSMVLLLHSQRQYIFEYDMWDRLSAITMPSVARHTMQTIRSIGYYRNIYNPPES
NASIITDYNEEGLLLQTAFLGTSRRVLFKYRRQTRLSEILYDSTRVSFTYDETAGVLKTV
NLQSDGFICTIRYRQIGPLIDRQIFRFSEDGMVNARFDYSYDNSFRVTSMQGVINETPLP
IDLYQFDDISGKVEQFGKFGVIYYDINQIISTAVMTYTKHFDAHGRIKEIQYEIFRSLMY
WITIQYDNMGRVTKREIKIGPFANTTKYAYEYDVDGQLQTVYLNEKIMWRYNYDLNGNLH
LLNPSNSARLTPLRYDLRDRITRLGDVQYRLDEDGFLRQRGTEIFEYSSKGLLTRVYSKG
SGWTVIYRYDGLGRRVSSKTSLGQHLQFFYADLTYPTRITHVYNHSSSEITSLYYDLQGH
LFAMEISSGDEFYIASDNTGTPLAVFSSNGLMLKQIQYTAYGEIYFDSNIDFQLVIGFHG
GLYDPLTKLIHFGERDYDILAGRWTTPDIEIWKRIGKDPAPFNLYMFRNNNPASKIHDVK
DYITDVNSWLVTFGFHLHNAIPGFPVPKFDLTEPSYELVKSQQWDDIPPIFGVQQQVARQ
AKAFLSLGKMAEVQVSRRRAGGAQSWLWFATVKSLIGKGVMLAVSQGRVQTNVLNIANED
CIKVAAVLNNAFYLENLHFTIEGKDTHYFIKTTTPESDLGTLRLTSGRKALENGINVTVS
QSTTVVNGRTRRFADVEMQFGALALHVRYGMTLDEEKARILEQARQRALARAWAREQQRV
RDGEEGARLWTEGEKRQLLSAGKVQGYDGYYVLSVEQYPELADSANNIQFLRQSEIGRR
>sp|Q9UI40|NCKX2_HUMAN Sodium/potassium/calcium exchanger 2 OS=Homo sapiens OX=9606 GN=SLC24A2 PE=1 SV=1

MDLQQSTTITSLEKWCLDESLSGCRRHYSVKKKLKLIRVLGLFMGLVAISTVSFSISAFS
ETDTQSTGEASVVSGPRVAQGYHQRTLLDLNDKILDYTPQPPLSKEGESENSTDHAQGDY
PKDIFSLEERRKGAIILHVIGMIYMFIALAIVCDEFFVPSLTVITEKLGISDDVAGATFM
AAGGSAPELFTSLIGVFIAHSNVGIGTIVGSAVFNILFVIGMCALFSREILNLTWWPLFR
DVSFYIVDLIMLIIFFLDNVIMWWESLLLLTAYFCYVVFMKFNVQVEKWVKQMINRNKVV
KVTAPEAQAKPSAARDKDEPTLPAKPRLQRGGSSASLHNSLMRNSIFQLMIHTLDPLAEE
LGSYGKLKYYDTMTEEGRFREKASILHKIAKKKCHVDENERQNGAANHVEKIELPNSTST
DVEMTPSSDASEPVQNGNLSHNIEGAEAQTADEEEDQPLSLAWPSETRKQVTFLIVFPIV
FPLWITLPDVRKPSSRKFFPITFFGSITWIAVFSYLMVWWAHQVGETIGISEEIMGLTIL
AAGTSIPDLITSVIVARKGLGDMAVSSSVGSNIFDITVGLPLPWLLYTVIHRFQPVAVSS
NGLFCAIVLLFIMLLFVILSIALCKWRMNKILGFIMFGLYFVFLVVSVLLEDRILTCPVS
I
>sp|P11168|GTR2_HUMAN Solute carrier family 2, facilitated glucose transporter member 2 OS=Homo sapiens OX=9606 GN=SLC2A2 PE=1 SV=1
MTEDKVTGTLVFTVITAVLGSFQFGYDIGVINAPQQVIISHYRHVLGVPLDDRKAINNYV
INSTDELPTISYSMNPKPTPWAEEETVAAAQLITMLWSLSVSSFAVGGMTASFFGGWLGD
TLGRIKAMLVANILSLVGALLMGFSKLGPSHILIIAGRSISGLYCGLISGLVPMYIGEIA
PTALRGALGTFHQLAIVTGILISQIIGLEFILGNYDLWHILLGLSGVRAILQSLLLFFCP
ESPRYLYIKLDEEVKAKQSLKRLRGYDDVTKDINEMRKEREEASSEQKVSIIQLFTNSSY
RQPILVALMLHVAQQFSGINGIFYYSTSIFQTAGISKPVYATIGVGAVNMVFTAVSVFLV
EKAGRRSLFLIGMSGMFVCAIFMSVGLVLLNKFSWMSYVSMIAIFLFVSFFEIGPGPIPW
FMVAEFFSQGPRPAALAIAAFSNWTCNFIVALCFQYIADFCGPYVFFLFAGVLLAFTLFT
FFKVPETKGKSFEEIAAEFQKKSGSAHRPKAAVEMKFLGATETV
>sp|P47895|AL1A3_HUMAN Retinaldehyde dehydrogenase 3 OS=Homo sapiens OX=9606 GN=ALDH1A3 PE=1 SV=2
MATANGAVENGQPDRKPPALPRPIRNLEVKFTKIFINNEWHESKSGKKFATCNPSTREQI
CEVEEGDKPDVDKAVEAAQVAFQRGSPWRRLDALSRGRLLHQLADLVERDRATLAALETM
DTGKPFLHAFFIDLEGCIRTLRYFAGWADKIQGKTIPTDDNVVCFTRHEPIGVCGAITPW
NFPLLMLVWKLAPALCCGNTMVLKPAEQTPLTALYLGSLIKEAGFPPGVVNIVPGFGPTV
GAAISSHPQINKIAFTGSTEVGKLVKEAASRSNLKRVTLELGGKNPCIVCADADLDLAVE
CAHQGVFFNQGQCCTAASRVFVEEQVYSEFVRRSVEYAKKRPVGDPFDVKTEQGPQIDQK
QFDKILELIESGKKEGAKLECGGSAMEDKGLFIKPTVFSEVTDNMRIAKEEIFGPVQPIL
KFKSIEEVIKRANSTDYGLTAAVFTKNLDKALKLASALESGTVWINCYNALYAQAPFGGF
KMSGNGRELGEYALAEYTEVKTVTIKLGDKNP
>sp|Q96QV1|HHIP_HUMAN Hedgehog-interacting protein OS=Homo sapiens OX=9606 GN=HHIP PE=1 SV=3
MLKMLSFKLLLLAVALGFFEGDAKFGERNEGSGARRRRCLNGNPPKRLKRRDRRMMSQLE
LLSGGEMLCGGFYPRLSCCLRSDSPGLGRLENKIFSVTNNTECGKLLEEIKCALCSPHSQ
SLFHSPEREVLERDLVLPLLCKDYCKEFFYTCRGHIPGFLQTTADEFCFYYARKDGGLCF
PDFPRKQVRGPASNYLDQMEEYDKVEEISRKHKHNCFCIQEVVSGLRQPVGALHSGDGSQ
RLFILEKEGYVKILTPEGEIFKEPYLDIHKLVQSGIKGGDERGLLSLAFHPNYKKNGKLY
VSYTTNQERWAIGPHDHILRVVEYTVSRKNPHQVDLRTARVFLEVAELHRKHLGGQLLFG
PDGFLYIILGDGMITLDDMEEMDGLSDFTGSVLRLDVDTDMCNVPYSIPRSNPHFNSTNQ
PPEVFAHGLHDPGRCAVDRHPTDININLTILCSDSNGKNRSSARILQIIKGKDYESEPSL

LEFKPFSNGPLVGGFVYRGCQSERLYGSYVFGDRNGNFLTLQQSPVTKQWQEKPLCLGTS
GSCRGYFSGHILGFGEDELGEVYILSSSKSMTQTHNGKLYKIVDPKRPLMPEECRATVQP
AQTLTSECSRLCRNGYCTPTGKCCCSPGWEGDFCRTAKCEPACRHGGVCVRPNKCLCKKG
YLGPQCEQVDRNIRRVTRAGILDQIIDMTSYLLDLTSYIV
>sp|P00390|GSHR_HUMAN Glutathione reductase, mitochondrial OS=Homo sapiens OX=9606 GN=GSR PE=1 SV=2
MALLPRALSAGAGPSWRRAARAFRGFLLLLPEPAALTRALSRAMACRQEPQPQGPPPAAG
AVASYDYLVIGGGSGGLASARRAAELGARAAVVESHKLGGTCVNVGCVPKKVMWNTAVHS
EFMHDHADYGFPSCEGKFNWRVIKEKRDAYVSRLNAIYQNNLTKSHIEIIRGHAAFTSDP
KPTIEVSGKKYTAPHILIATGGMPSTPHESQIPGASLGITSDGFFQLEELPGRSVIVGAG
YIAVEMAGILSALGSKTSLMIRHDKVLRSFDSMISTNCTEELENAGVEVLKFSQVKEVKK
TLSGLEVSMVTAVPGRLPVMTMIPDVDCLLWAIGRVPNTKDLSLNKLGIQTDDKGHIIVD
EFQNTNVKGIYAVGDVCGKALLTPVAIAAGRKLAHRLFEYKEDSKLDYNNIPTVVFSHPP
IGTVGLTEDEAIHKYGIENVKTYSTSFTPMYHAVTKRKTKCVMKMVCANKEEKVVGIHMQ
GLGCDEMLQGFAVAVKMGATKADFDNTVAIHPTSSEELVTLR
>sp|P01034|CYTC_HUMAN Cystatin-C OS=Homo sapiens OX=9606 GN=CST3 PE=1 SV=1
MAGPLRAPLLLLAILAVALAVSPAAGSSPGKPPRLVGGPMDASVEEEGVRRALDFAVGEY
NKASNDMYHSRALQVVRARKQIVAGVNYFLDVELGRTTCTKTQPNLDNCPFHDQPHLKRK
AFCSFQIYAVPWQGTMTLSKSTCQDA
>sp|P01112|RASH_HUMAN GTPase HRas OS=Homo sapiens OX=9606 GN=HRAS PE=1 SV=1
MTEYKLVVVGAGGVGKSALTIQLIQNHFVDEYDPTIEDSYRKQVVIDGETCLLDILDTAG
QEEYSAMRDQYMRTGEGFLCVFAINNTKSFEDIHQYREQIKRVKDSDDVPMVLVGNKCDL
AARTVESRQAQDLARSYGIPYIETSAKTRQGVEDAFYTLVREIRQHKLRKLNPPDESGPG
CMSCKCVLS
>sp|P01857|IGHG1_HUMAN Immunoglobulin heavy constant gamma 1 OS=Homo sapiens OX=9606 GN=IGHG1 PE=1 SV=2
ASTKGPSVFPLAPSSKSTSGGTAALGCLVKDYFPEPVTVSWNSGALTSGVHTFPAVLQSS
GLYSLSSVVTVPSSSLGTQTYICNVNHKPSNTKVDKKVEPKSCDKTHTCPPCPAPELLGG
PSVFLFPPKPKDTLMISRTPEVTCVVVDVSHEDPEVKFNWYVDGVEVHNAKTKPREEQYN
STYRVVSVLTVLHQDWLNGKEYKCKVSNKALPAPIEKTISKAKGQPREPQVYTLPPSRDE
LTKNQVSLTCLVKGFYPSDIAVEWESNGQPENNYKTTPPVLDSDGSFFLYSKLTVDKSRW
QQGNVFSCSVMHEALHNHYTQKSLSLSPELQLEESCAEAQDGELDGLWTTITIFITLFLL
SVCYSATVTFFKVKWIFSSVVDLKQTIIPDYRNMIGQGA
>sp|P07900|HS90A_HUMAN Heat shock protein HSP 90-alpha OS=Homo sapiens OX=9606 GN=HSP90AA1 PE=1 SV=5
MPEETQTQDQPMEEEEVETFAFQAEIAQLMSLIINTFYSNKEIFLRELISNSSDALDKIR
YESLTDPSKLDSGKELHINLIPNKQDRTLTIVDTGIGMTKADLINNLGTIAKSGTKAFME
ALQAGADISMIGQFGVGFYSAYLVAEKVTVITKHNDDEQYAWESSAGGSFTVRTDTGEPM
GRGTKVILHLKEDQTEYLEERRIKEIVKKHSQFIGYPITLFVEKERDKEVSDDEAEEKED
KEEEKEKEEKESEDKPEIEDVGSDEEEEKKDGDKKKKKKIKEKYIDQEELNKTKPIWTRN
PDDITNEEYGEFYKSLTNDWEDHLAVKHFSVEGQLEFRALLFVPRRAPFDLFENRKKKNN
IKLYVRRVFIMDNCEELIPEYLNFIRGVVDSEDLPLNISREMLQQSKILKVIRKNLVKKC
LELFTELAEDKENYKKFYEQFSKNIKLGIHEDSQNRKKLSELLRYYTSASGDEMVSLKDY
CTRMKENQKHIYYITGETKDQVANSAFVERLRKHGLEVIYMIEPIDEYCVQQLKEFEGKT

LVSVTKEGLELPEDEEEKKKQEEKKTKFENLCKIMKDILEKKVEKVVVSNRLVTSPCCIV
TSTYGWTANMERIMKAQALRDNSTMGYMAAKKHLEINPDHSIIETLRQKAEADKNDKSVK
DLVILLYETALLSSGFSLEDPQTHANRIYRMIKLGLGIDEDDPTADDTSAAVTEEMPPLE
GDDDTSRMEEVD
>sp|P10909|CLUS_HUMAN Clusterin OS=Homo sapiens OX=9606 GN=CLU PE=1 SV=1
MMKTLLLFVGLLLTWESGQVLGDQTVSDNELQEMSNQGSKYVNKEIQNAVNGVKQIKTLI
EKTNEERKTLLSNLEEAKKKKEDALNETRESETKLKELPGVCNETMMALWEECKPCLKQT
CMKFYARVCRSGSGLVGRQLEEFLNQSSPFYFWMNGDRIDSLLENDRQQTHMLDVMQDHF
SRASSIIDELFQDRFFTREPQDTYHYLPFSLPHRRPHFFFPKSRIVRSLMPFSPYEPLNF
HAMFQPFLEMIHEAQQAMDIHFHSPAFQHPPTEFIREGDDDRTVCREIRHNSTGCLRMKD
QCDKCREILSVDCSTNNPSQAKLRRELDESLQVAERLTRKYNELLKSYQWKMLNTSSLLE
QLNEQFNWVSRLANLTQGEDQYYLRVTTVASHTSDSDVPSGVTEVVVKLFDSDPITVTVP
VEVSRKNPKFMETVAEKALQEYRKKHREE
>sp|P12955|PEPD_HUMAN Xaa-Pro dipeptidase OS=Homo sapiens OX=9606 GN=PEPD PE=1
SV=3
MAAATGPSFWLGNETLKVPLALFALNRQRLCERLRKNPAVQAGSIVVLQGGEETQRYCTD
TGVLFRQESFFHWAFGVTEPGCYGVIDVDTGKSTLFVPRLPASHATWMGKIHSKEHFKEK
YAVDDVQYVDEIASVLTSQKPSVLLTLRGVNTDSGSVCREASFDGISKFEVNNTILHPEI
VECRVFKTDMELEVLRYTNKISSEAHREVMKAVKVGMKEYELESLFEHYCYSRGGMRHSS
YTCICGSGENSAVLHYGHAGAPNDRTIQNGDMCLFDMGGEYYCFASDITCSFPANGKFTA
DQKAVYEAVLRSSRAVMGAMKPGVWWPDMHRLADRIHLEELAHMGILSGSVDAMVQAHLG
AVFMPHGLGHFLGIDVHDVGGYPEGVERIDEPGLRSLRTARHLQPGMVLTVEPGIYFIDH
LLDEALADPARASFLNREVLQRFRGFGGVRIEEDVVVTDSGIELLTCVPRTVEEIEACMA
GCDKAFTPFSGPK
>sp|P23526|SAHH_HUMAN Adenosylhomocysteinase OS=Homo sapiens OX=9606 GN=AHCY
PE=1 SV=4
MSDKLPYKVADIGLAAWGRKALDIAENEMPGLMRMRERYSASKPLKGARIAGCLHMTVET
AVLIETLVTLGAEVQWSSCNIFSTQDHAAAAIAKAGIPVYAWKGETDEEYLWCIEQTLYF
KDGPLNMILDDGGDLTNLIHTKYPQLLPGIRGISEETTTGVHNLYKMMANGILKVPAINV
NDSVTKSKFDNLYGCRESLIDGIKRATDVMIAGKVAVVAGYGDVGKGCAQALRGFGARVI
ITEIDPINALQAAMEGYEVTTMDEACQEGNIFVTTTGCIDIILGRHFEQMKDDAIVCNIG
HFDVEIDVKWLNENAVEKVNIKPQVDRYRLKNGRRIILLAEGRLVNLGCAMGHPSFVMSN
SFTNQVMAQIELWTHPDKYPVGVHFLPKKLDEAVAEAHLGKLNVKLTKLTEKQAQYLGMS
CDGPFKPDHYRY
>sp|P32119|PRDX2_HUMAN Peroxiredoxin-2 OS=Homo sapiens OX=9606 GN=PRDX2 PE=1
SV=5
MASGNARIGKPAPDFKATAVVDGAFKEVKLSDYKGKYVVLFFYPLDFTFVCPTEIIAFSN
RAEDFRKLGCEVLGVSVDSQFTHLAWINTPRKEGGLGPLNIPLLADVTRRLSEDYGVLKT
DEGIAYRGLFIIDGKGVLRQITVNDLPVGRSVDEALRLVQAFQYTDEHGEVCPAGWKPGS
DTIKPNVDDSKEYFSKHN
>sp|P46926|GNPI1_HUMAN Glucosamine-6-phosphate isomerase 1 OS=Homo sapiens
OX=9606 GN=GNPDA1 PE=1 SV=1
MKLIILEHYSQASEWAAKYIRNRIIQFNPGPEKYFTLGLPTGSTPLGCYKKLIEYYKNGD
LSFKYVKTFNMDEYVGLPRDHPESYHSFMWNNFFKHIDIHPENTHILDGNAVDLQAECDA

FEEKIKAAGGIELFVGGIGPDGHIAFNEPGSSLVSRTRVKTLAMDTILANARFFDGELTK
VPTMALTVGVGTVMDAREVMILITGAHKAFALYKAIEEGVNHMWTVSAFQQHPRTVFVCD
EDATLELKVKTVKYFKGLMLVHNKLVDPLYSIKEKETEKSQSSKKPYSD
>sp|P49721|PSB2_HUMAN Proteasome subunit beta type-2 OS=Homo sapiens OX=9606 GN=PSMB2 PE=1 SV=1
MEYLIGIQGPDYVLVASDRVAASNIVQMKDDHDKMFKMSEKILLLCVGEAGDTVQFAEYI
QKNVQLYKMRNGYELSPTAAANFTRRNLADCLRSRTPYHVNLLLAGYDEHEGPALYYMDY
LAALAKAPFAAHGYGAFLTLSILDRYYTPTISRERAVELLRKCLEELQKRFILNLPTFSV
RIIDKNGIHDLDNISFPKQGS
>sp|P53420|CO4A4_HUMAN Collagen alpha-4(IV) chain OS=Homo sapiens OX=9606 GN=COL4A4 PE=1 SV=3
MWSLHIVLMRCSFRLTKSLATGPWSLILILFSVQYVYGSGKKYIGPCGGRDCSVCHCVPE
KGSRGPPGPPGPQGPIGPLGAPGPIGLSGEKGMRGDRGPPGAAGDKGDKGPTGVPGFPGL
DGIPGHPGPPGPRGKPGMSGHNGSRGDPGFPGGRGALGPGGPLGHPGEKGEKGNSVFILG
AVKGIQGDRGDPGLPGLPGSWGAGGPAGPTGYPGEPGLVGPPGQPGRPGLKGNPGVGVKG
QMGDPGEVGQQGSPGPTLLVEPPDFCLYKGEKGIKGIPGMVGLPGPPGRKGESGIGAKGE
KGIPGFPGPRGDPGSYGSPGFPGLKGELGLVGDPGLFGLIGPKGDPGNRGHPGPPGVLVT
PPLPLKGPPGDPGFPGRYGETGDVGPPGPPGLLGRPGEACAGMIGPPGPQGFPGLPGLPG
EAGIPGRPDSAPGKPGKPGSPGLPGAPGLQGLPGSSVIYCSVGNPGPQGIKGKVGPPGGR
GPKGEKGNEGLCACEPGPMGPPGPPGLPGRQGSKGDLGLPGWLGTKGDPGPPGAEGPPGL
PGKHGASGPPGNKGAKGDMVVSRVKGHKGERGPDGPPGFPGQPGSHGRDGHAGEKGDPGP
PGDHEDATPGGKGFPGPLGPPGKAGPVGPPGLGFPGPPGERGHPGVPGHPGVRGPDGLKG
QKGDTISCNVTYPGRHGPPGFDGPPGPKGFPGPQGAPGLSGSDGHKGRPGTPGTAEIPGP
PGFRGDMGDPGFGGEKGSSPVGPPGPPGSPGVNGQKGIPGDPAFGHLGPPGKRGLSGVPG
IKGPRGDPGCPGAEGPAGIPGFLGLKGPKGREGHAGFPGVPGPPGHSCERGAPGIPGQPG
LPGYPGSPGAPGGKGQPGDVGPPGPAGMKGLPGLPGRPGAHGPPGLPGIPGPFGDDGLPG
PPGPKGPRGLPGFPGFPGERGKPGAEGCPGAKGEPGEKGMSGLPGDRGLRGAKGAIGPPG
DEGEMAIISQKGTPGEPGPPGDDGFPGERGDKGTPGMQGRRGEPGRYGPPGFHRGEPGEK
GQPGPPGPPGPPGSTGLRGFIGFPGLPGDQGEPGSPGPPGFSGIDGARGPKGNKGDPASH
FGPPGPKGEPGSPGCPGHFGASGEQGLPGIQGPRGSPGRPGPPGSSGPPGCPGDHGMPGL
RGQPGEMGDPGPRGLQGDPGIPGPPGIKGPSGSPGLNGLHGLKGQKGTKGASGLHDVGPP
GPVGIPGLKGERGDPGSPGISPPGPRGKKGPPGPPGSSGPPGPAGATGRAPKDIPDPGPP
GDQGPPGPDGPRGAPGPPGLPGSVDLLRGEPGDCGLPGPPGPPGPPGPPGYKGFPGCDGK
DGQKGPVGFPGPQGPHGFPGPPGEKGLPGPPGRKGPTGLPGPRGEPGPPADVDDCPRIPG
LPGAPGMRGPEGAMGLPGMRGPSGPGCKGEPGLDGRRGVDGVPGSPGPPGRKGDTGEDGY
PGGPGPPGPIGDPGPKGFGPGYLGGFLLVLHSQTDQEPTCPLGMPRLWTGYSLLYLEGQE
KAHNQDLGLAGSCLPVFSTLPFAYCNIHQVCHYAQRNDRSYWLASAAPLPMMPLSEEAIR
PYVSRCAVCEAPAQAVAVHSQDQSIPPCPQTWRSLWIGYSFLMHTGAGDQGGGQALMSPG
SCLEDFRAAPFLECQGRQGTCHFFANKYSFWLTTVKADLQFSSAPAPDTLKESQAQRQKI
SRCQVCVKYS
>sp|P55011|S12A2_HUMAN Solute carrier family 12 member 2 OS=Homo sapiens OX=9606 GN=SLC12A2 PE=1 SV=1
MEPRPTAPSSGAPGLAGVGETPSAAALAAARVELPGTAVPSVPEDAAPASRDGGGVRDEG
PAAAGDGLGRPLGPTPSQSRFQVDLVSENAGRAAAAAAAAAAAAAAAGAGAGAKQTPADG

EASGESEPAKGSEEAKGRFRVNFVDPAASSSAEDSLSDAAGVGVDGPNVSFQNGGDTVLS
EGSSLHSGGGGGSGHHQHYYYDTHTNTYYLRTFGHNTMDAVPRIDHYRHTAAQLGEKLLR
PSLAELHDELEKEPFEDGFANGEESTPTRDAVVTYTAESKGVVKFGWIKGVLVRCMLNIW
GVMLFIRLSWIVGQAGIGLSVLVIMMATVVTTITGLSTSAIATNGFVRGGGAYYLISRSL
GPEFGGAIGLIFAFANAVAVAMYVVGFAETVVELLKEHSILMIDEINDIRIIGAITVVIL
LGISVAGMEWEAKAQIVLLVILLLAIGDFVIGTFIPLESKKPKGFFGYKSEIFNENFGPD
FREEETFFSVFAIFFPAATGILAGANISGDLADPQSAIPKGTLLAILITTLVYVGIAVSV
GSCVVRDATGNVNDTIVTELTNCTSAACKLNFDFSSCESSPCSYGLMNNFQVMSMVSGFT
PLISAGIFSATLSSALASLVSAPKIFQALCKDNIYPAFQMFAKGYGKNNEPLRGYILTFL
IALGFILIAELNVIAPIISNFFLASYALINFSVFHASLAKSPGWRPAFKYYNMWISLLGA
ILCCIVMFVINWWAALLTYVIVLGLYIYVTYKKPDVNWGSSTQALTYLNALQHSIRLSGV
EDHVKNFRPQCLVMTGAPNSRPALLHLVHDFTKNVGLMICGHVHMGPRRQAMKEMSIDQA
KYQRWLIKNKMKAFYAPVHADDLREGAQYLMQAAGLGRMKPNTLVLGFKKDWLQADMRDV
DMYINLFHDAFDIQYGVVVIRLKEGLDISHLQGQEELLSSQEKSPGTKDVVVSVEYSKKS
DLDTSKPLSEKPITHKVEEEDGKTATQPLLKKESKGPIVPLNVADQKLLEASTQFQKKQG
KNTIDVWWLFDDGGLTLLIPYLLTTKKKWKDCKIRVFIGGKINRIDHDRRAMATLLSKFR
IDFSDIMVLGDINTKPKKENIIAFEEIIEPYRLHEDDKEQDIADKMKEDEPWRITDNELE
LYKTKTYRQIRLNELLKEHSSTANIIVMSLPVARKGAVSSALYMAWLEALSKDLPPILLV
RGNHQSVLTFYS
>sp|P62820|RAB1A_HUMAN Ras-related protein Rab-1A OS=Homo sapiens OX=9606
GN=RAB1A PE=1 SV=3
MSSMNPEYDYLFKLLLIGDSGVGKSCLLLRFADDTYTESYISTIGVDFKIRTIELDGKTI
KLQIWDTAGQERFRTITSSYYRGAHGIIVVYDVTDQESFNNVKQWLQEIDRYASENVNKL
LVGNKCDLTTKKVVDYTTAKEFADSLGIPFLETSAKNATNVEQSFMTMAAEIKKRMGPGA
TAGGAEKSNVKIQSTPVKQSGGGCC
>sp|Q00610|CLH1_HUMAN Clathrin heavy chain 1 OS=Homo sapiens OX=9606 GN=CLTC PE=1
SV=5
MAQILPIRFQEHLQLQNLGINPANIGFSTLTMESDKFICIREKVGEQAQVVIIDMNDPSN
PIRRPISADSAIMNPASKVIALKAGKTLQIFNIEMKSKMKAHTMTDDVTFWKWISLNTVA
LVTDNAVYHWSMEGESQPVKMFDRHSSLAGCQIINYRTDAKQKWLLLTGISAQQNRVVGA
MQLYSVDRKVSQPIEGHAASFAQFKMEGNAEESTLFCFAVRGQAGGKLHIIEVGTPPTGN
QPFPKKAVDVFFPPEAQNDFPVAMQISEKHDVVFLITKYGYIHLYDLETGTCIYMNRISG
ETIFVTAPHEATAGIIGVNRKGQVLSVCVEEENIIPYITNVLQNPDLALRMAVRNNLAGA
EELFARKFNALFAQGNYSEAAKVAANAPKGILRTPDTIRRFQSVPAQPGQTSPLLQYFGI
LLDQGQLNKYESLELCRPVLQQGRKQLLEKWLKEDKLECSEELGDLVKSVDPTLALSVYL
RANVPNKVIQCFAETGQVQKIVLYAKKVGYTPDWIFLLRNVMRISPDQGQQFAQMLVQDE
EPLADITQIVDVFMEYNLIQQCTAFLLDALKNNRPSEGPLQTRLLEMNLMHAPQVADAIL
GNQMFTHYDRAHIAQLCEKAGLLQRALEHFTDLYDIKRAVVHTHLLNPEWLVNYFGSLSV
EDSLECLRAMLSANIRQNLQICVQVASKYHEQLSTQSLIELFESFKSFEGLFYFLGSIVN
FSQDPDVHFKYIQAACKTGQIKEVERICRESNCYDPERVKNFLKEAKLTDQLPLIIVCDR
FDFVHDLVLYLYRNNLQKYIEIYVQKVNPSRLPVVIGGLLDVDCSEDVIKNLILVVRGQF
STDELVAEVEKRNRLKLLLPWLEARIHEGCEEPATHNALAKIYIDSNNNPERFLRENPYY
DSRVVGKYCEKRDPHLACVAYERGQCDLELINVCNENSLFKSLSRYLVRRKDPELWGSVL
LESNPYRRPLIDQVVQTALSETQDPEEVSVTVKAFMTADLPNELIELLEKIVLDNSVFSE

HRNLQNLLILTAIKADRTRVMEYINRLDNYDAPDIANIAISNELFEEAFAIFRKFDVNTS
AVQVLIEHIGNLDRAYEFAERCNEPAVWSQLAKAQLQKGMVKEAIDSYIKADDPSSYMEV
VQAANTSGNWEELVKYLQMARKKARESYVETELIFALAKTNRLAELEEFINGPNNAHIQQ
VGDRCYDEKMYDAAKLLYNNVSNFGRLASTLVHLGEYQAAVDGARKANSTRTWKEVCFAC
VDGKEFRLAQMCGLHIVVHADELEELINYYQDRGYFEELITMLEAALGLERAHMGMFTEL
AILYSKFKPQKMREHLELFWSRVNIPKVLRAAEQAHLWAELVFLYDKYEEYDNAIITMMN
HPTDAWKEGQFKDIITKVANVELYYRAIQFYLEFKPLLLNDLLMVLSPRLDHTRAVNYFS
KVKQLPLVKPYLRSVQNHNNKSVNESLNNLFITEEDYQALRTSIDAYDNFDNISLAQRLE
KHELIEFRRIAAYLFKGNNRWKQSVELCKKDSLYKDAMQYASESKDTELAEELLQWFLQE
EKRECFGACLFTCYDLLRPDVVLETAWRHNIMDFAMPYFIQVMKEYLTKVDKLDASESLR
KEEEQATETQPIVYGQPQLMLTAGPSVAVPPQAPFGYGYTAPPYGQPQPGFGYSM
>sp|Q13200|PSMD2_HUMAN 26S proteasome non-ATPase regulatory subunit 2 OS=Homo sapiens OX=9606 GN=PSMD2 PE=1 SV=3
MEEGGRDKAPVQPQQSPAAAPGGTDEKPSGKERRDAGDKDKEQELSEEDKQLQDELEMLV
ERLGEKDTSLYRPALEELRRQIRSSTTSMTSVPKPLKFLRPHYGKLKEIYENMAPGENKR
FAADIISVLAMTMSGERECLKYRLVGSQEELASWGHEYVRHLAGEVAKEWQELDDAEKVQ
REPLLTLVKEIVPYNMAHNAEHEACDLLMEIEQVDMLEKDIDENAYAKVCLYLTSCVNYV
PEPENSALLRCALGVFRKFSRFPEALRLALMLNDMELVEDIFTSCKDVVVQKQMAFMLGR
HGVFLELSEDVEEYEDLTEIMSNVQLNSNFLALARELDIMEPKVPDDIYKTHLENNRFGG
SGSQVDSARMNLASSFVNGFVNAAFGQDKLLTDDGNKWLYKNKDHGMLSAAASLGMILLW
DVDGGLTQIDKYLYSSEDYIKSGALLACGIVNSGVRNECDPALALLSDYVLHNSNTMRLG
SIFGLGLAYAGSNREDVLTLLLPVMGDSKSSMEVAGVTALACGMIAVGSCNGDVTSTILQ
TIMEKSETELKDTYARWLPLGLGLNHLGKGEAIEAILAALEVVSEPFRSFANTLVDVCAY
AGSGNVLKVQQLLHICSEHFDSKEKEEDKDKKEKKDKDKKEAPADMGAHQGVAVLGIALI
AMGEEIGAEMALRTFGHLLRYGEPTLRRAVPLALALISVSNPRLNILDTLSKFSHDADPE
VSYNSIFAMGMVGSGTNNARLAAMLRQLAQYHAKDPNNLFMVRLAQGLTHLGKGTLTLCP
YHSDRQLMSQVAVAGLLTVLVSFLDVRNIILGKSHYVLYGLVAAMQPRMLVTFDEELRPL
PVSVRVGQAVDVVGQAGKPKTITGFQTHTTPVLLAHGERAELATEEFLPVTPILEGFVIL
RKNPNYDL
>sp|Q15124|PGM5_HUMAN Phosphoglucomutase-like protein 5 OS=Homo sapiens OX=9606 GN=PGM5 PE=1 SV=2
MEGSPIPVLTVPTAPYEDQRPAGGGGLRRPTGLFEGQRNYLPNFIQSVLSSIDLRDRQGC
TMVVGSDGRYFSRTAIEIVVQMAAANGIGRLIIGQNGILSTPAVSCIIRKIKAAGGIILT
ASHCPGGPGGEFGVKFNVANGGPAPDVVSDKIYQISKTIEEYAICPDLRIDLSRLGRQEF
DLENKFKPFRVEIVDPVDIYLNLLRTIFDFHAIKGLLTGPSQLKIRIDAMHGVMGPYVRK
VLCDELGAPANSAINCVPLEDFGGQHPDPNLTYATTLLEAMKGGEYGFGAAFDADGDRYM
ILGQNGFFVSPSDSLAIIAANLSCIPYFRQMGVRGFGRSMPTSMALDRVAKSMKVPVYET
PAGWRFFSNLMDSGRCNLCGEESFGTGSDHLREKDGLWAVLVWLSIIAARKQSVEEIVRD
HWAKFGRHYYCRFDYEGLDPKTTYYIMRDLEALVTDKSFIGQQFAVGSHVYSVAKTDSFE
YVDPVDGTVTKKQGLRIIFSDASRLIFRLSSSSGVRATLRLYAESYERDPSGHDQEPQAV
LSPLIAIALKISQIHERTGRRGPTVIT
>sp|Q15286|RAB35_HUMAN Ras-related protein Rab-35 OS=Homo sapiens OX=9606 GN=RAB35 PE=1 SV=1
MARDYDHLFKLLIIGDSGVGKSSLLLRFADNTFSGSYITTIGVDFKIRTVEINGEKVKLQ

IWDTAGQERFRTITSTYYRGTHGVIVVYDVTSAESFVNVKRWLHEINQNCDDVCRILVGN
KNDDPERKVVETEDAYKFAGQMGIQLFETSAKENVNVEEMFNCITELVLRAKKDNLAKQQ
QQQQNDVVKLTKNSKRKKRCC
>sp|Q8TCT0|CERK1_HUMAN Ceramide kinase OS=Homo sapiens OX=9606 GN=CERK PE=1 SV=1
MGATGAAEPLQSVLWVKQQRCAVSLEPARALLRWWRSPGPGAGAPGADACSVPVSEIIAV
EETDVHGKHQGSGKWQKMEKPYAFTVHCVKRARRHRWKWAQVTFWCPEEQLCHLWLQTLR
EMLEKLTSRPKHLLVFINPFGGKGQGKRIYERKVAPLFTLASITTDIIVTEHANQAKETL
YEINIDKYDGIVCVGGDGMFSEVLHGLIGRTQRSAGVDQNHPRAVLVPSSLRIGIIPAGS
TDCVCYSTVGTSDAETSALHIVVGDSLAMDVSSVHHNSTLLRYSVSLLGYGFYGDIIKDS
EKKRWLGLARYDFSGLKTFLSHHCYEGTVSFLPAQHTVGSPRDRKPCRAGCFVCRQSKQQ
LEEEQKKALYGLEAAEDVEEWQVVCGKFLAINATNMSCACRRSPRGLSPAAHLGDGSSDL
ILIRKCSRFNFLRFLIRHTNQQDQFDFTFVEVYRVKKFQFTSKHMEDEDSDLKEGGKKRF
GHICSSHPSCCCTVSNSSWNCDGEVLHSPAIEVRVHCQLVRLFARGIEENPKPDSHS
>sp|Q969Q5|RAB24_HUMAN Ras-related protein Rab-24 OS=Homo sapiens OX=9606
GN=RAB24 PE=1 SV=1
MSGQRVDVKVVMLGKEYVGKTSLVERYVHDRFLVGPYQNTIGAAFVAKVMSVGDRTVTLG
IWDTAGSERYEAMSRIYYRGAKAAIVCYDLTDSSSFERAKFWVKELRSLEEGCQIYLCGT
KSDLLEEDRRRRRVDFHDVQDYADNIKAQLFETSSKTGQSVDELFQKVAEDYVSVAAFQV
MTEDKGVDLGQKPNPYFYSCCHH
>sp|Q99685|MGLL_HUMAN Monoglyceride lipase OS=Homo sapiens OX=9606 GN=MGLL PE=1
SV=2
MPEESSPRRTPQSIPYQDLPHLVNADGQYLFCRYWKPTGTPKALIFVSHGAGEHSGRYEE
LARMLMGLDLLVFAHDHVGHGQSEGERMVVSDFHVFVRDVLQHVDSMQKDYPGLPVFLLG
HSMGGAIAILTAAERPGHFAGMVLISPLVLANPESATTFKVLAAKVLNLVLPNLSLGPID
SSVLSRNKTEVDIYNSDPLICRAGLKVCFGIQLLNAVSRVERALPKLTVPFLLLQGSADR
LCDSKGAYLLMELAKSQDKTLKIYEGAYHVLHKELPEVTNSVFHEINMWVSQRTATAGTA
SPP
>sp|Q9H0C3|TM117_HUMAN Transmembrane protein 117 OS=Homo sapiens OX=9606
GN=TMEM117 PE=1 SV=1
MGKDFRYYFQHPWSRMIVAYLVIFFNFLIFAEDPVSHSQTEANVIVVGNCFSFVTNKYPR
GVGWRILKVLLWLLAILTGLIAGKFLFHQRLFGQLLRLKMFREDHGSWMTMFFSTILFLF
IFSHIYNTILLMDGNMGAYIITDYMGIRNESFMKLAAVGTWMGDFVTAWMVTDMMLQDKP
YPDWGKSARAFWKKGNVRITLFWTVLFTLTSVVVLVITTDWISWDKLNRGFLPSDEVSRA
FLASFILVFDLLIVMQDWEFPHFMGDVDVNLPGLHTPHMQFKIPFFQKIFKEEYRIHITG
KWFNYGIIFLVLILDLNMWKNQIFYKPHEYGQYIGPGQKIYTVKDSESLKDLNRTKLSWE
WRSNHTNPRTNKTYVEGDMFLHSRFIGASLDVKCLAFVPSLIAFVWFGFFIWFFGRFLKN
EPRMENQDKTYTRMKRKSPSEHSKDMGITRENTQASVEDPLNDPSLVCIRSDFNEIVYKS
SHLTSENLSSQLNESTSATEADQDPTTSKSTPTN
>sp|Q9HAB8|PPCS_HUMAN Phosphopantothenate--cysteine ligase OS=Homo sapiens OX=9606
GN=PPCS PE=1 SV=2
MAEMDPVAEFPQPPGAARWAEVMARFAARLGAQGRRVVLVTSGGTKVPLEARPVRFLDNF
SSGRRGATSAEAFLAAGYGVLFLYRARSAFPYAHRFPPQTWLSALRPSGPALSGLLSLEA
EENALPGFAEALRSYQEAAAAGTFLAVEFTTLADYLHLLQAAAQALNPLGPSAMFYLAAA
VSDFYVPVSEMPEHKIQSSGGPLQITMKMVPKLLSPLVKDWAPKAFIISFKLETDPAIVI

NRARKALEIYQHQVVVANILESRQSFVFIVTKDSETKLLLSEEEIEKGVEIEEKIVDNLQ
SRHTAFIGDRN
>sp|Q9NZJ7|MTCH1_HUMAN Mitochondrial carrier homolog 1 OS=Homo sapiens OX=9606 GN=MTCH1 PE=1 SV=1
MGASDPEVAPWARGGAAGMAGAGAGAGARGGAAAGVEARARDPPPAHRAHPRHPRPAAQP
SARRMDGGSGGLGSGDNAPTTEALFVALGAGVTALSHPLLYVKLLIQVGHEPMPPTLGTN
VLGRKVLYLPSFFTYAKYIVQVDGKIGLFRGLSPRLMSNALSTVTRGSMKKVFPPDEIEQ
VSNKDDMKTSLKKVVKETSYEMMMQCVSRMLAHPLHVISMRCMVQFVGREAKYSGVLSSI
GKIFKEEGLLGFFVGLIPHLLGDVVFLWGCNLLAHFINAYLVDDSVSDTPGGLGNDQNPG
SQFSQALAIRSYTKFVMGIAVSMLTYPFLLVGDLMAVNNCGLQAGLPPYSPVFKSWIHCW
KYLSVQGQLFRGSSLLFRRVSSGSCFALE
>sp|Q9UIG0|BAZ1B_HUMAN Tyrosine-protein kinase BAZ1B OS=Homo sapiens OX=9606 GN=BAZ1B PE=1 SV=2
MAPLLGRKPFPLVKPLPGEEPLFTIPHTQEAFRTREEYEARLERYSERIWTCKSTGSSQL
THKEAWEEEQEVAELLKEEFPAWYEKLVLEMVHHNTASLEKLVDTAWLEIMTKYAVGEEC
DFEVGKEKMLKVKIVKIHPLEKVDEEATEKKSDGACDSPSSDKENSSQIAQDHQKKETVV
KEDEGRRESINDRARRSPRKLPTSLKKGERKWAPPKFLPHKYDVKLQNEDKIISNVPADS
LIRTERPPNKEIVRYFIRHNALRAGTGENAPWVVEDELVKKYSLPSKFSDFLLDPYKYMT
LNPSTKRKNTGSPDRKPSKKSKTDNSSLSSPLNPKLWCHVHLKKSLSGSPLKVKNSKNSK
SPEEHLEEMMKMMSPNKLHTNFHIPKKGPPAKKPGKHSDKPLKAKGRSKGILNGQKSTGN
SKSPKKGLKTPKTKMKQMTLLDMAKGTQKMTRAPRNSGGTPRTSSKPHKHLPPAALHLIA
YYKENKDREDKRSALSCVISKTARLLSSEDRARLPEELRSLVQKRYELLEHKKRWASMSE
EQRKEYLKKKREELKKKLKEKAKERREKEMLERLEKQKRYEDQELTGKNLPAFRLVDTPE
GLPNTLFGDVAMVVEFLSCYSGLLLPDAQYPITAVSLMEALSADKGGFLYLNRVLVILLQ
TLLQDEIAEDYGELGMKLSEIPLTLHSVSELVRLCLRRSDVQEESEGSDTDDNKDSAAFE
DNEVQDEFLEKLETSEFFELTSEEKLQILTALCHRILMTYSVQDHMETRQQMSAELWKER
LAVLKEENDKKRAEKQKRKEMEAKNKENGKVENGLGKTDRKKEIVKFEPQVDTEAEDMIS
AVKSRRLLAIQAKKEREIQEREMKVKLERQAEEERIRKHKAAAEKAFQEGIAKAKLVMRR
TPIGTDRNHNRYWLFSDEVPGLFIEKGWVHDSIDYRFNHHCKDHTVSGDEDYCPRSKKAN
LGKNASMNTQHGTATEVAVETTTPKQGQNLWFLCDSQKELDELLNCLHPQGIRESQLKER
LEKRYQDIIHSIHLARKPNLGLKSCDGNQELLNFLRSDLIEVATRLQKGGLGYVEETSEF
EARVISLEKLKDFGECVIALQASVIKKFLQGFMAPKQKRRKLQSEDSAKTEEVDEEKKMV
EEAKVASALEKWKTAIREAQTFSRMHVLLGMLDACIKWDMSAENARCKVCRKKGEDDKLI
LCDECNKAFHLFCLRPALYEVPDGEWQCPACQPATARRNSRGRNYTEESASEDSEDDESD
EEEEEEEEEEEEDYEVAGLRLRPRKTIRGKHSVIPPAARSGRRPGKKPHSTRRSQPKAP
PVDDAEVDELVLQTKRSSRRQSLELQKCEEILHKIVKYRFSWPFREPVTRDEAEDYYDVI
THPMDFQTVQNKCSCGSYRSVQEFLTDMKQVFTNAEVYNCRGSHVLSCMVKTEQCLVALL
HKHLPGHPYVRRKRKKFPDRLAEDEGDSEPEAVGQSRGRRQKK
>sp|P34949|MPI_HUMAN Mannose-6-phosphate isomerase OS=Homo sapiens OX=9606 GN=MPI PE=1 SV=2
MAAPRVFPLSCAVQQYAWGKMGSNSEVARLLASSDPLAQIAEDKPYAELWMGTHPRGDAK
ILDNRISQKTLSQWIAENQDSLGSKVKDTFNGNLPFLFKVLSVETPLSIQAHPNKELAEK
LHLQAPQHYPDANHKPEMAIALTPFQGLCGFRPVEEIVTFLKKVPEFQFLIGDEAATHLK
QTMSHDSQAVASSLQSCFSHLMKSEKKVVVEQLNLLVKRISQQAAAGNNMEDIFGELLLQ

LHQQYPGDIGCFAIYFLNLLTLKPGEAMFLEANVPHAYLKGDCVECMACSDNTVRAGLTP
KFIDVPTLCEMLSYTPSSSKDRLFLPTRSQEDPYLSIYDPPVPDFTIMKTEVPGSVTEYK
VLALDSASILLMVQGTVIASTPTTQTPIPLQRGGVLFIGANESVSLKLTEPKDLLIFRAC
CLL
>sp|P61204|ARF3_HUMAN ADP-ribosylation factor 3 OS=Homo sapiens OX=9606 GN=ARF3 PE=1 SV=2
MGNIFGNLLKSLIGKKEMRILMVGLDAAGKTTILYKLKLGEIVTTIPTIGFNVETVEYKN
ISFTVWDVGGQDKIRPLWRHYFQNTQGLIFVVDSNDRERVNEAREELMRMLAEDELRDAV
LLVFANKQDLPNAMNAAEITDKLGLHSLRHRNWYIQATCATSGDGLYEGLDWLANQLKNK
K
>sp|Q01453|PMP22_HUMAN Peripheral myelin protein 22 OS=Homo sapiens OX=9606 GN=PMP22 PE=1 SV=1
MLLLLLSIIVLHVAVLVLLFVSTIVSQWIVGNGHATDLWQNCSTSSSGNVHHCFSSSPNE
WLQSVQATMILSIIFSILSLFLFFCQLFTLTKGGRFYITGIFQILAGLCVMSAAAIYTVR
HPEWHLNSDYSYGFAYILAWVAFPLALLSGVIYVILRKRE
>tr|Q53GE9|Q53GE9_HUMAN Elongation factor 1-alpha (Fragment) OS=Homo sapiens OX=9606 PE=2 SV=1
MGKEKTHINIVVIGHVDSGKSTTTGHLIYKCGGIDKRTIEKFEKEAAEMGKGSFKYAWVL
DKLKAERERGITIDISLWKFETSKYYVTIIDAPGHRDFIKNMITGTSQADCAVLIVAAGV
GEFEAGISKNGQTREHALLAYTLGVKQLIVGVNKMDSTEPPYSQKRYEEIVKEVSAYIKK
IGYNPDTVAFVPISGWNGDNMLEPSANMPWFKGWKVTRKDGNASGTTLLEALDCILPPTR
PTDKPLRLPLQDVYKIGGIGTVPVGRVETGVLKPGMVVTFAPVNVTTEVKSVEMHHEALS
EALPGDNVGFNVKNVSVKDVRRGNVAGDSKNDPPMEAAGFTAQVIILNHPGQISAGYAPV
LDCHTAHIACKFAELKEKIDRRSGKKLEDGPKFLKSGDAAIVDMVPGKPMCVESFSDYPP
LGRFAVRDMRQTVAVGVIKAVDKKAAGAGKVTKSAQKAQKAK
>sp|Q8IYS2|K2013_HUMAN Uncharacterized protein KIAA2013 OS=Homo sapiens OX=9606 GN=KIAA2013 PE=1 SV=1
MWLQQRLKGLPGLLSSSWARRLLCLLGLLLLLLWFGGSGARRAAGGLHLLPWSRGEPGAA
EPSACLEAATRAWRGLRERGEVVPLGPGVPALVANGFLALDVAANRLWVTPGEREPAVAP
DFVPFVQLRPLSALAEAGEAVLLLREGLLRRVRCLQLGSPGPGPVAAGPGPASVSGLAAG
SGRDCVLLQEDFLAHRGRPHVYLQRIQLNNPTERVAALQTVGPTAGPAPKAFTSTLEKVG
DHQFLLYSGRSPPTPTGLVHLVVVAAKKLVNRLQVAPKTQLDETVLWVVHVSGPINPQVL
KSKAAKELKALQDLARKEMLELLDMPAAELLQDHQLLWAQLFSPGVEMKKITDTHTPSGL
TVNLTLYYMLSCSPAPLLSPSLSHRERDQMESTLNYEDHCFSGHATMHAENLWPGRLSSV
QQILQLSDLWRLTLQKRGCKGLVKVGAPGILQGMVLSFGGLQFTENHLQFQADPDVLHNS
YALHGIRYKNDHINLAVLADAEGKPYLHVSVESRGQPVKIYACKAGCLDEPVELTSAPTG
HTFSVMVTQPITPLLYISTDLTHLQDLRHTLHLKAILAHDEHMAQQDPGLPFLFWFSVAS
LITLFHLFLFKLIYNEYCGPGAKPLFRSKEDPSV
>tr|B7Z705|B7Z705_HUMAN Chitinase domain-containing protein 1 OS=Homo sapiens OX=9606 PE=2 SV=1
MSVRGILRLGTQEYPSPLDPEVGPYCDTPTMRTLFNLLWLALACSPVHTTLSKSDAKKAA
SKTLLEKSQFSDKPVQDRGLVVTDLKAESVVLEHRSYCSAKARDRHFAGDVLGYVTPWNS
HGYDVTKVFGSKFTQISPVWLQLKRRGREMFEVTGLHDVDQGWMRAVRKHAKGLHIVPRL
LFEDWTYDDFRNVLDSEDEIEELSKTVVQVAKNQHFDGFVVEVWNQLLSQKRVGLIHMLT

HLAEALHQARLLALLVIPPAITPGTDQLGMFTHKEFEQLAPVLDGFSLMTYDYSTAHQPG
PNAPLSWVRACVQVLDPKSKWRSKILLGLNFYGMDYVTSKDAREPVVGARYIQTLKDHRP
RMVWDSQASEHFFEYEKSRSGRHVVFYPTLKSLQVRLELARELGVGVSIWELGQGLDYFY
DLP
>sp|O14787|TNPO2_HUMAN Transportin-2 OS=Homo sapiens OX=9606 GN=TNPO2 PE=1 SV=3
MDWQPDEQGLQQVLQLLKDSQSPNTATQRIVQDKLKQLNQFPDFNNYLIFVLTRLKSEDE
PTRSLSGLILKNNVKAHYQSFPPPVADFIKQECLNNIGDASSLIRATIGILITTIASKGE
LQMWPELLPQLCNLLNSEDYNTCEGAFGALQKICEDSSELLDSDALNRPLNIMIPKFLQF
FKHCSPKIRSHAIACVNQFIMDRAQALMDNIDTFIEHLFALAVDDDPEVRKNVCRALVML
LEVRIDRLIPHMHSIIQYMLQRTQDHDENVALEACEFWLTLAEQPICKEVLASHLVQLIP
ILVNGMKYSEIDIILLKGDVEEDEAVPDSEQDIKPRFHKSRTVTLPHEAERPDGSEDAED
DDDDDALSDWNLRKCSAAALDVLANVFREELLPHLLPLLKGLLFHPEWVVKESGILVLGA
IAEGCMQGMVPYLPELIPHLIQCLSDKKALVRSIACWTLSRYAHWVVSQPPDMHLKPLMT
ELLKRILDGNKRVQEAACSAFATLEEEACTELVPYLSYILDTLVFAFGKYQHKNLLILYD
AIGTLADSVGHHLNQPEYIQKLMPPLIQKWNELKDEDKDLFPLLECLSSVATALQSGFLP
YCEPVYQRCVTLVQKTLAQAMMYTQHPEQYEAPDKDFMIVALDLLSGLAEGLGGHVEQLV
ARSNIMTLLFQCMQDSMPEVRQSSFALLGDLTKACFIHVKPCIAEFMPILGTNLNPEFIS
VCNNATWAIGEICMQMGAEMQPYVQMVLNNLVEIINRPNTPKTLLENTGRLTSPSAIPAI
TIGRLGYVCPQEVAPMLQQFIRPWCTSLRNIRDNEEKDSAFRGICMMIGVNPGGVVQDFI
FFCDAVASWVSPKDDLRDMFYKILHGFKDQVGEDNWQQFSEQFPPLLKERLAAFYGV
>sp|P09972|ALDOC_HUMAN Fructose-bisphosphate aldolase C OS=Homo sapiens OX=9606
GN=ALDOC PE=1 SV=2
MPHSYPALSAEQKKELSDIALRIVAPGKGILAADESVGSMAKRLSQIGVENTEENRRLYR
QVLFSADDRVKKCIGGVIFFHETLYQKDDNGVPFVRTIQDKGIVVGIKVDKGVVPLAGTD
GETTTQGLDGLSERCAQYKKDGADFAKWRCVLKISERTPSALAILENANVLARYASICQQ
NGIVPIVEPEILPDGDHDLKRCQYVTEKVLAAVYKALSDHHVYLEGTLLKPNMVTPGHAC
PIKYTPEEIAMATVTALRRTVPPAVPGVTFLSGGQSEEEASFNLNAINRCPLPRPWALTF
SYGRALQASALNAWRGQRDNAGAATEEFIKRAEVNGLAAQGKYEGSGEDGGAAAQSLYIA
NHAY
>sp|P10768|ESTD_HUMAN S-formylglutathione hydrolase OS=Homo sapiens OX=9606 GN=ESD
PE=1 SV=2
MALKQISSNKCFGGLQKVFEHDSVELNCKMKFAVYLPPKAETGKCPALYWLSGLTCTEQN
FISKSGYHQSASEHGLVVIAPDTSPRGCNIKGEDESWDFGTGAGFYVDATEDPWKTNYRM
YSYVTEELPQLINANFPVDPQRMSIFGHSMGGHGALICALKNPGKYKSVSAFAPICNPVL
CPWGKKAFSGYLGTDQSKWKAYDATHLVKSYPGSQLDILIDQGKDDQFLLDGQLLPDNFI
AACTEKKIPVVFRLQEGYDHSYYFIATFITDHIRHHAKYLNA
>sp|P13639|EF2_HUMAN Elongation factor 2 OS=Homo sapiens OX=9606 GN=EEF2 PE=1 SV=4
MVNFTVDQIRAIMDKKANIRNMSVIAHVDHGKSTLTDSLVCKAGIIASARAGETRFTDTR
KDEQERCITIKSTAISLFYELSENDLNFIKQSKDGAGFLINLIDSPGHVDFSSEVTAALR
VTDGALVVVDCVSGVCVQTETVLRQAIAERIKPVLMMNKMDRALLELQLEPEELYQTFQR
IVENVNVIISTYGEGESGPMGNIMIDPVLGTVGFGSGLHGWAFTLKQFAEMYVAKFAAKG
EGQLGPAERAKKVEDMMKKLWGDRYFDPANGKFSKSATSPEGKKLPRTFCQLILDPIFKV
FDAIMNFKKEETAKLIEKLDIKLDSEDKDKEGKPLLKAVMRRWLPAGDALLQMITIHLPS
PVTAQKYRCELLYEGPPDDEAAMGIKSCDPKGPLMMYISKMVPTSDKGRFYAFGRVFSGL

VSTGLKVRIMGPNYTPGKKEDLYLKPIQRTILMMGRYVEPIEDVPCGNIVGLVGVDQFLV
KTGTITTFEHAHNMRVMKFSVSPVVRVAVEAKNPADLPKLVEGLKRLAKSDPMVQCIIEE
SGEHIIAGAGELHLEICLKDLEEDHACIPIKKSDPVVSYRETVSEESNVLCLSKSPNKHN
RLYMKARPFPDGLAEDIDKGEVSARQELKQRARYLAEKYEWDVAEARKIWCFGPDGTGPN
ILTDITKGVQYLNEIKDSVVAGFQWATKEGALCEENMRGVRFDVHDVTLHADAIHRGGGQ
IIPTARRCLYASVLTAQPRLMEPIYLVEIQCPEQVVGGIYGVLNRKRGHVFEESQVAGTP
MFVVKAYLPVNESFGFTADLRSNTGGQAFPQCVFDHWQILPGDPFDNSSRPSQVVAETRK
RKGLKEGIPALDNFLDKL
>sp|P21333|FLNA_HUMAN Filamin-A OS=Homo sapiens OX=9606 GN=FLNA PE=1 SV=4
MSSSHSRAGQSAAGAAPGGGVDTRDAEMPATEKDLAEDAPWKKIQQNTFTRWCNEHLKCV
SKRIANLQTDLSDGLRLIALLEVLSQKKMHRKHNQRPTFRQMQLENVSVALEFLDRESIK
LVSIDSKAIVDGNLKLILGLIWTLILHYSISMPMWDEEEDEEAKKQTPKQRLLGWIQNKL
PQLPITNFSRDWQSGRALGALVDSCAPGLCPDWDSWDASKPVTNAREAMQQADDWLGIPQ
VITPEEIVDPNVDEHSVMTYLSQFPKAKLKPGAPLRPKLNPKKARAYGPGIEPTGNMVKK
RAEFTVETRSAGQGEVLVYVEDPAGHQEEAKVTANNDKNRTFSVWYVPEVTGTHKVTVLF
AGQHIAKSPFEVYVDKSQGDASKVTAQGPGLEPSGNIANKTTYFEIFTAGAGTGEVEVVI
QDPMGQKGTVEPQLEARGDSTYRCSYQPTMEGVHTVHVTFAGVPIPRSPYTVTVGQACNP
SACRAVGRGLQPKGVRVKETADFKVYTKGAGSGELKVTVKGPKGEERVKQKDLGDGVYGF
EYYPMVPGTYIVTITWGGQNIGRSPFEVKVGTECGNQKVRAWGPGLEGGVVGKSADFVVE
AIGDDVGTLGFSVEGPSQAKIECDDKGDGSCDVRYWPQEAGEYAVHVLCNSEDIRLSPFM
ADIRDAPQDFHPDRVKARGPGLEKTGVAVNKPAEFTVDAKHGGKAPLRVQVQDNEGCPVE
ALVKDNGNGTYSCSYVPRKPVKHTAMVSWGGVSIPNSPFRVNVGAGSHPNKVKVYGPGVA
KTGLKAHEPTYFTVDCAEAGQGDVSIGIKCAPGVVGPAEADIDFDIIRNDNDTFTVKYTP
RGAGSYTIMVLFADQATPTSPIRVKVEPSHDASKVKAEGPGLSRTGVELGKPTHFTVNAK
AAGKGKLDVQFSGLTKGDAVRDVDIIDHHDNTYTVKYTPVQQGPVGVNVTYGGDPIPKSP
FSVAVSPSLDLSKIKVSGLGEKVDVGKDQEFTVKSKGAGGQGKVASKIVGPSGAAVPCKV
EPGLGADNSVVRFLPREEGPYEVEVTYDGVPVPGSPFPLEAVAPTKPSKVKAFGPGLQGG
SAGSPARFTIDTKGAGTGGLGLTVEGPCEAQLECLDNGDGTCSVSYVPTEPGDYNINILF
ADTHIPGSPFKAHVVPCFDASKVKCSGPGLERATAGEVGQFQVDCSSAGSAELTIEICSE
AGLPAEVYIQDHGDGTHTITYIPLCPGAYTVTIKYGGQPVPNFPSKLQVEPAVDTSGVQC
YGPGIEGQGVFREATTEFSVDARALTQTGGPHVKARVANPSGNLTETYVQDRGDGMYKVE
YTPYEEGLHSVDVTYDGSPVPSSPFQVPVTEGCDPSRVRVHGPGIQSGTTNKPNKFTVET
RGAGTGGLGLAVEGPSEAKMSCMDNKDGSCSVEYIPYEAGTYSLNVTYGGHQVPGSPFKV
PVHDVTDASKVKCSGPGLSPGMVRANLPQSFQVDTSKAGVAPLQVKVQGPKGLVEPVDVV
DNADGTQTVNYVPSREGPYSISVLYGDEEVPRSPFKVKVLPTHDASKVKASGPGLNTTGV
PASLPVEFTIDAKDAGEGLLAVQITDPEGKPKKTHIQDNHDGTYTVAYVPDVTGRYTILI
KYGGDEIPFSPYRVRAVPTGDASKCTVTVSIGGHGLGAGIGPTIQIGEETVITVDTKAAG
KGKVTCTVCTPDGSEVDVDVVENEDGTFDIFYTAPQPGKYVICVRFGGEHVPNSPFQVTA
LAGDQPSVQPPLRSQQLAPQYTYAQGGQQTWAPERPLVGVNGLDVTSLRPFDLVIPFTIK
KGEITGEVRMPSGKVAQPTITDNKDGTVTVRYAPSEAGLHEMDIRYDNMHIPGSPLQFYV
DYVNCGHVTAYGPGLTHGVVNKPATFTVNTKDAGEGGLSLAIEGPSKAEISCTDNQDGTC
SVSYLPVLPGDYSILVKYNEQHVPGSPFTARVTGDDSMRMSHLKVGSAADIPINISETDL
SLLTATVVPPSGREEPCLLKRLRNGHVGISFVPKETGEHLVHVKKNGQHVASSPIPVVIS
QSEIGDASRVRVSGQGLHEGHTFEPAEFIIDTRDAGYGGLSLSIEGPSKVDINTEDLEDG

TCRVTYCPTEPGNYIINIKFADQHVPGSPFSVKVTGEGRVKESITRRRRAPSVANVGSHC
DLSLKIPEISIQDMTAQVTSPSGKTHEAEIVEGENHTYCIRFVPAEMGTHTVSVKYKGQH
VPGSPFQFTVGPLGEGGAHKVRAGGPGLERAEAGVPAEFSIWTREAGAGGLAIAVEGPSK
AEISFEDRKDGSCGVAYVVQEPGDYEVSVKFNEEHIPDSPFVVPVASPSGDARRLTVSSL
QESGLKVNQPASFAVSLNGAKGAIDAKVHSPSGALEECYVTEIDQDKYAVRFIPRENGVY
LIDVKFNGTHIPGSPFKIRVGEPGHGGDPGLVSAYGAGLEGGVTGNPAEFVVNTSNAGAG
ALSVTIDGPSKVKMDCQECPEGYRVTYTPMAPGSYLISIKYGGPYHIGGSPFKAKVTGPR
LVSNHSLHETSSVFVDSLTKATCAPQHGAPGPGPADASKVVAKGLGLSKAYVGQKSSFTV
DCSKAGNNMLLVGVHGPRTPCEEILVKHVGSRLYSVSYLLKDKGEYTLVVKWGDEHIPGS
PYRVVVP
>sp|P34913|HYES_HUMAN Bifunctional epoxide hydrolase 2 OS=Homo sapiens OX=9606
GN=EPHX2 PE=1 SV=2
MTLRAAVFDLDGVLALPAVFGVLGRTEEALALPRGLLNDAFQKGGPEGATTRLMKGEITL
SQWIPLMEENCRKCSETAKVCLPKNFSIKEIFDKAISARKINRPMLQAALMLRKKGFTTA
ILTNTWLDDRAERDGLAQLMCELKMHFDFLIESCQVGMVKPEPQIYKFLLDTLKASPSEV
VFLDDIGANLKPARDLGMVTILVQDTDTALKELEKVTGIQLLNTPAPLPTSCNPSDMSHG
YVTVKPRVRLHFVELGSGPAVCLCHGFPESWYSWRYQIPALAQAGYRVLAMDMKGYGESS
APPEIEEYCMEVLCKEMVTFLDKLGLSQAVFIGHDWGGMLVWYMALFYPERVRAVASLNT
PFIPANPNMSPLESIKANPVFDYQLYFQEPGVAEAELEQNLSRTFKSLFRASDESVLSMH
KVCEAGGLFVNSPEEPSLSRMVTEEEIQFYVQQFKKSGFRGPLNWYRNMERNWKWACKSL
GRKILIPALMVTAEKDFVLVPQMSQHMEDWIPHLKRGHIEDCGHWTQMDKPTEVNQILIK
WLDSDARNPPVVSKM
>sp|P55060|XPO2_HUMAN Exportin-2 OS=Homo sapiens OX=9606 GN=CSE1L PE=1 SV=3
MELSDANLQTLTEYLKKTLDPDPAIRRPAEKFLESVEGNQNYPLLLLTLLEKSQDNVIKV
CASVTFKNYIKRNWRIVEDEPNKICEADRVAIKANIVHLMLSSPEQIQKQLSDAISIIGR
EDFPQKWPDLLTEMVNRFQSGDFHVINGVLRTAHSLFKRYRHEFKSNELWTEIKLVLDAF
ALPLTNLFKATIELCSTHANDASALRILFSSLILISKLFYSLNFQDLPEFFEDNMETWMN
NFHTLLTLDNKLLQTDDEEEAGLLELLKSQICDNAALYAQKYDEEFQRYLPRFVTAIWNL
LVTTGQEVKYDLLVSNAIQFLASVCERPHYKNLFEDQNTLTSICEKVIVPNMEFRAADEE
AFEDNSEEYIRRDLEGSDIDTRRRAACDLVRGLCKFFEGPVTGIFSGYVNSMLQEYAKNP
SVNWKHKDAAIYLVTSLASKAQTQKHGITQANELVNLTEFFVNHILPDLKSANVNEFPVL
KADGIKYIMIFRNQVPKEHLLVSIPLLINHLQAESIVVHTYAAHALERLFTMRGPNNATL
FTAAEIAPFVEILLTNLFKALTLPGSSENEYIMKAIMRSFSLLQEAIIPYIPTLITQLTQ
KLLAVSKNPSKPHFNHYMFEAICLSIRITCKANPAAVVNFEEALFLVFTEILQNDVQEFI
PYVFQVMSLLLETHKNDIPSSYMALFPHLLQPVLWERTGNIPALVRLLQAFLERGSNTIA
SAAADKIPGLLGVFQKLIASKANDHQGFYLLNSIIEHMPPESVDQYRKQIFILLFQRLQN
SKTTKFIKSFLVFINLYCIKYGALALQEIFDGIQPKMFGMVLEKIIIPEIQKVSGNVEKK
ICAVGITKLLTECPPMMDTEYTKLWTPLLQSLIGLFELPEDDTIPDEEHFIDIEDTPGYQ
TAFSQLAFAGKKEHDPVGQMVNNPKIHLAQSLHKLSTACPGRVPSMVSTSLNAEALQYLQ
GYLQAASVTLL
>sp|P60174|TPIS_HUMAN Triosephosphate isomerase OS=Homo sapiens OX=9606 GN=TPI1
PE=1 SV=4
MAPSRKFFVGGNWKMNGRKQSLGELIGTLNAAKVPADTEVVCAPPTAYIDFARQKLDPKI
AVAAQNCYKVTNGAFTGEISPGMIKDCGATWVVLGHSERRHVFGESDELIGQKVAHALAE

GLGVIACIGEKLDEREAGITEKVVFEQTKVIADNVKDWSKVVLAYEPVWAIGTGKTATPQ
QAQEVHEKLRGWLKSNVSDAVAQSTRIIYGGSVTGATCKELASQPDVDGFLVGGASLKPE
FVDIINAKQ
>sp|P61019|RAB2A_HUMAN Ras-related protein Rab-2A OS=Homo sapiens OX=9606
GN=RAB2A PE=1 SV=1
MAYAYLFKYIIIGDTGVGKSCLLLQFTDKRFQPVHDLTIGVEFGARMITIDGKQIKLQIW
DTAGQESFRSITRSYYRGAAGALLVYDITRRDTFNHLTTWLEDARQHSNSNMVIMLIGNK
SDLESRREVKKEEGEAFAREHGLIFMETSAKTASNVEEAFINTAKEIYEKIQEGVFDINN
EANGIKIGPQHAATNATHAGNQGGQQAGGGCC
>sp|P63104|1433Z_HUMAN 14-3-3 protein zeta/delta OS=Homo sapiens OX=9606 GN=YWHAZ
PE=1 SV=1
MDKNELVQKAKLAEQAERYDDMAACMKSVTEQGAELSNEERNLLSVAYKNVVGARRSSWR
VVSSIEQKTEGAEKKQQMAREYREKIETELRDICNDVLSLLEKFLIPNASQAESKVFYLK
MKGDYYRYLAEVAAGDDKKGIVDQSQQAYQEAFEISKKEMQPTHPIRLGLALNFSVFYYE
ILNSPEKACSLAKTAFDEAIAELDTLSEESYKDSTLIMQLLRDNLTLWTSDTQGDEAEAG
EGGEN
>sp|Q01082|SPTB2_HUMAN Spectrin beta chain, non-erythrocytic 1 OS=Homo sapiens
OX=9606 GN=SPTBN1 PE=1 SV=2
MTTTVATDYDNIEIQQQYSDVNNRWDVDDWDNENSSARLFERSRIKALADEREAVQKKTF
TKWVNSHLARVSCRITDLYTDLRDGRMLIKLLEVLSGERLPKPTKGRMRIHCLENVDKAL
QFLKEQRVHLENMGSHDIVDGNHRLTLGLIWTIILRFQIQDISVETEDNKEKKSAKDALL
LWCQMKTAGYPNVNIHNFTTSWRDGMAFNALIHKHRPDLIDFDKLKKSNAHYNLQNAFNL
AEQHLGLTKLLDPEDISVDHPDEKSIITYVVTYYHYFSKMKALAVEGKRIGKVLDNAIET
EKMIEKYESLASDLLEWIEQTIIILNNRKFANSLVGVQQQLQAFNTYRTVEKPPKFTEKG
NLEVLLFTIQSKMRANNQKVYMPREGKLISDINKAWERLEKAEHERELALRNELIRQEKL
EQLARRFDRKAAMRETWLSENQRLVSQDNFGFDLPAVEAATKKHEAIETDIAAYEERVQA
VVAVARELEAENYHDIKRITARKDNVIRLWEYLLELLRARRQRLEMNLGLQKIFQEMLYI
MDWMDEMKVLVLSQDYGKHLLGVEDLLQKHTLVEADIGIQAERVRGVNASAQKFATDGEG
YKPCDPQVIRDRVAHMEFCYQELCQLAAERRARLEESRRLWKFFWEMAEEEGWIREKEKI
LSSDDYGKDLTSVMRLLSKHRAFEDEMSGRSGHFEQAIKEGEDMIAEEHFGSEKIRERII
YIREQWANLEQLSAIRKKRLEEASLLHQFQADADDIDAWMLDILKIVSSSDVGHDEYSTQ
SLVKKHKDVAEEIANYRPTLDTLHEQASALPQEHAESPDVRGRLSGIEERYKEVAELTRL
RKQALQDTLALYKMFSEADACELWIDEKEQWLNNMQIPEKLEDLEVIQHRFESLEPEMNN
QASRVAVVNQIARQLMHSGHPSEKEIKAQQDKLNTRWSQFRELVDRKKDALLSALSIQNY
HLECNETKSWIREKTKVIESTQDLGNDLAGVMALQRKLTGMERDLVAIEAKLSDLQKEAE
KLESEHPDQAQAILSRLAEISDVWEEMKTTLKNREASLGEASKLQQFLRDLDDFQSWLSR
TQTAIASEDMPNTLTEAEKLLTQHENIKNEIDNYEEDYQKMRDMGEMVTQGQTDAQYMFL
RQRLQALDTGWNELHKMWENRQNLLSQSHAYQQFLRDTKQAEAFLNNQEYVLAHTEMPTT
LEGAEAAIKKQEDFMTTMDANEEKINAVVETGRRLVSDGNINSDRIQEKVDSIDDRHRKN
RETASELLMRLKDNRDLQKFLQDCQELSLWINEKMLTAQDMSYDEARNLHSKWLKHQAFM
AELASNKEWLDKIEKEGMQLISEKPETEAVVKEKLTGLHKMWEVLESTTQTKAQRLFDAN
KAELFTQSCADLDKWLHGLESQIQSDDYGKDLTSVNILLKKQQMLENQMEVRKKEIEELQ
SQAQALSQEGKSTDEVDSKRLTVQTKFMELLEPLNERKHNLLASKEIHQFNRDVEDEILW
VGERMPLATSTDHGHNLQTVQLLIKKNQTLQKEIQGHQPRIDDIFERSQNIVTDSSSLSA

EAIRQRLADLKQLWGLLIEETEKRHRRLEEAHRAQQYYFDAAEAEAWMSEQELYMMSEEK
AKDEQSAVSMLKKHQILEQAVEDYAETVHQLSKTSRALVADSHPESERISMRQSKVDKLY
AGLKDLAEERRGKLDERHRLFQLNREVDDLEQWIAEREVVAGSHELGQDYEHVTMLQERF
REFARDTGNIGQERVDTVNHLADELINSGHSDAATIAEWKDGLNEAWADLLELIDTRTQI
LAASYELHKFYHDAKEIFGRIQDKHKKLPEELGRDQNTVETLQRMHTTFEHDIQALGTQV
RQLQEDAARLQAAYAGDKADDIQKRENEVLEAWKSLLDACESRRVRLVDTGDKFRFFSMV
RDLMLWMEDVIRQIEAQEKPRDVSSVELLMNNHQGIKAEIDARNDSFTTCIELGKSLLAR
KHYASEEIKEKLLQLTEKRKEMIDKWEDRWEWLRLILEVHQFSRDASVAEAWLLGQEPYL
SSREIGQSVDEVEKLIKRHEAFEKSAATWDERFSALERLTTLELLEVRRQQEEEERKRRP
PSPEPSTKVSEEAESQQQWDTSKGEQVSQNGLPAEQGSPRMAETVDTSEMVNGATEQRTS
SKESSPIPSPTSDRKAKTALPAQSAATLPARTQETPSAQMEGFLNRKHEWEAHNKKASSR
SWHNVYCVINNQEMGFYKDAKTAASGIPYHSEVPVSLKEAVCEVALDYKKKKHVFKLRLN
DGNEYLFQAKDDEEMNTWIQAISSAISSDKHEVSASTQSTPASSRAQTLPTSVVTITSES
SPGKREKDKEKDKEKRFSLFGKKK
>sp|Q04446|GLGB_HUMAN 1,4-alpha-glucan-branching enzyme OS=Homo sapiens OX=9606
GN=GBE1 PE=1 SV=3
MAAPMTPAARPEDYEAALNAALADVPELARLLEIDPYLKPYAVDFQRRYKQFSQILKNIG
ENEGGIDKFSRGYESFGVHRCADGGLYCKEWAPGAEGVFLTGDFNGWNPFSYPYKKLDYG
KWELYIPPKQNKSVLVPHGSKLKVVITSKSGEILYRISPWAKYVVREGDNVNYDWIHWDP
EHSYEFKHSRPKKPRSLRIYESHVGISSHEGKVASYKHFTCNVLPRIKGLGYNCIQLMAI
MEHAYYASFGYQITSFFAASSRYGTPEELQELVDTAHSMGIIVLLDVVHSHASKNSADGL
NMFDGTDSCYFHSGPRGTHDLWDSRLFAYSSWEILRFLLSNIRWWLEEYRFDGFRFDGVT
SMLYHHHGVGQGFSGDYSEYFGLQVDEDALTYLMLANHLVHTLCPDSITIAEDVSGMPAL
CSPISQGGGGFDYRLAMAIPDKWIQLLKEFKDEDWNMGDIVYTLTNRRYLEKCIAYAESH
DQALVGDKSLAFWLMDAEMYTNMSVLTPFTPVIDRGIQLHKMIRLITHGLGGEGYLNFMG
NEFGHPEWLDFPRKGNNESYHYARRQFHLTDDDLLRYKFLNNFDRDMNRLEERYGWLAAP
QAYVSEKHEGNKIIAFERAGLLFIFNFHPSKSYTDYRVGTALPGKFKIVLDSDAAEYGGH
QRLDHSTDFFSEAFEHNGRPYSLLVYIPSRVALILQNVDLPN
>sp|Q10567|AP1B1_HUMAN AP-1 complex subunit beta-1 OS=Homo sapiens OX=9606
GN=AP1B1 PE=1 SV=3
MTDSKYFTTTKKGEIFELKAELNSDKKEKKKEAVKKVIASMTVGKDVSALFPDVVNCMQT
DNLELKKLVYLYLMNYAKSQPDMAIMAVNTFVKDCEDPNPLIRALAVRTMGCIRVDKITE
YLCEPLRKCLKDEDPYVRKTAAVCVAKLHDINAQLVEDQGFLDTLKDLISDSNPMVVANA
VAALSEIAESHPSSNLLDLNPQSINKLLTALNECTEWGQIFILDCLANYMPKDDREAQSI
CERVTPRLSHANSAVVLSAVKVLMKFMEMLSKDLDYYGTLLKKLAPPLVTLLSAEPELQY
VALRNINLIVQKRPEILKHEMKVFFVKYNDPIYVKLEKLDIMIRLASQANIAQVLAELKE
YATEVDVDFVRKAVRAIGRCAIKVEQSAERCVSTLLDLIQTKVNYVVQEAIVVIKDIFRK
YPNKYESVIATLCENLDSLDEPEARAAMIWIVGEYAERIDNADELLESFLEGFHDESTQV
QLQLLTAIVKLFLKKPTETQELVQQVLSLATQDSDNPDLRDRGYIYWRLLSTDPVAAKEV
VLAEKPLISEETDLIEPTLLDELICYIGTLASVYHKPPSAFVEGGRGVVHKSLPPRTASS
ESAESPETAPTGAPPGEQPDVIPAQGDLLGDLLNLDLGPPVSGPPLATSSVQMGAVDLLG
GGLDSLMGDEPEGIGGTNFVAPPTAAVPANLGAPIGSGLSDLFDLTSGVGTLSGSYVAPK
AVWLPAMKAKGLEISGTFTRQVGSISMDLQLTNKALQVMTDFAIQFNRNSFGLAPAAPLQ
VHAPLSPNQTVEISLPLSTVGSVMKMEPLNNLQVAVKNNIDVFYFSTLYPLHILFVEDGK

MDRQMFLATWKDIPNENEAQFQIRDCPLNAEAASSKLQSSNIFTVAKRNVEGQDMLYQSL
KLTNGIWVLAELRIQPGNPSCTDLELSLKCRAPEVSQHVYQAYETILKN
>sp|Q13098|CSN1_HUMAN COP9 signalosome complex subunit 1 OS=Homo sapiens OX=9606
GN=GPS1 PE=1 SV=4
MPLPVQVFNLQGAVEPMQIDVDPQEDPQNAPDVNYVVENPSLDLEQYAASYSGLMRIERL
QFIADHCPTLRVEALKMALSFVQRTFNVDMYEEIHRKLSEATRSSLRELQNAPDAIPESG
VEPPALDTAWVEATRKKALLKLEKLDTDLKNYKGNSIKESIRRGHDDLGDHYLDCGDLSN
ALKCYSRARDYCTSAKHVINMCLNVIKVSVYLQNWSHVLSYVSKAESTPEIAEQRGERDS
QTQAILTKLKCAAGLAELAARKYKQAAKCLLLASFDHCDFPELLSPSNVAIYGGLCALAT
FDRQELQRNVISSSSFKLFLELEPQVRDIIFKFYESKYASCLKMLDEMKDNLLLDMYLAP
HVRTLYTQIRNRALIQYFSPYVSADMHRMAAAFNTTVAALEDELTQLILEGLISARVDSH
SKILYARDVDQRSTTFEKSLLMGKEFQRRAKAMMLRAAVLRNQIHVKSPPREGSQGELTP
ANSQSRMSTNM
>sp|Q13813|SPTN1_HUMAN Spectrin alpha chain, non-erythrocytic 1 OS=Homo sapiens
OX=9606 GN=SPTAN1 PE=1 SV=3
MDPSGVKVLETAEDIQERRQQVLDRYHRFKELSTLRRQKLEDSYRFQFFQRDAEELEKWI
QEKLQIASDENYKDPTNLQGKLQKHQAFEAEVQANSGAIVKLDETGNLMISEGHFASETI
RTRLMELHRQWELLLEKMREKGIKLLQAQKLVQYLRECEDVMDWINDKEAIVTSEELGQD
LEHVEVLQKKFEEFQTDMAAHEERVNEVNQFAAKLIQEQHPEEELIKTKQDEVNAAWQRL
KGLALQRQGKLFGAAEVQRFNRDVDETISWIKEKEQLMASDDFGRDLASVQALLRKHEGL
ERDLAALEDKVKALCAEADRLQQSHPLSATQIQVKREELITNWEQIRTLAAERHARLNDS
YRLQRFLADFRDLTSWVTEMKALINADELASDVAGAEALLDRHQEHKGEIDAHEDSFKSA
DESGQALLAAGHYASDEVREKLTVLSEERAALLELWELRRQQYEQCMDLQLFYRDTEQVD
NWMSKQEAFLLNEDLGDSLDSVEALLKKHEDFEKSLSAQEEKITALDEFATKLIQNNHYA
MEDVATRRDALLSRRNALHERAMRRRAQLADSFHLQQFFRDSDELKSWVNEKMKTATDEA
YKDPSNLQGKVQKHQAFEAELSANQSRIDALEKAGQKLIDVNHYAKDEVAARMNEVISLW
KKLLEATELKGIKLREANQQQQFNRNVEDIELWLYEVEGHLASDDYGKDLTNVQNLQKKH
ALLEADVAAHQDRIDGITIQARQFQDAGHFDAENIKKKQEALVARYEALKEPMVARKQKL
ADSLRLQQLFRDVEDEETWIREKEPIAASTNRGKDLIGVQNLLKKHQALQAEIAGHEPRI
KAVTQKGNAMVEEGHFAAEDVKAKLHELNQKWEALKAKASQRRQDLEDSLQAQQYFADAN
EAESWMREKEPIVGSTDYGKDEDSAEALLKKHEALMSDLSAYGSSIQALREQAQSCRQQV
APTDDETGKELVLALYDYQEKSPREVTMKKGDILTLLNSTNKDWWKVEVNDRQGFVPAAY
VKKLDPAQSASRENLLEEQGSIALRQEQIDNQTRITKEAGSVSLRMKQVEELYHSLLELG
EKRKGMLEKSCKKFMLFREANELQQWINEKEAALTSEEVGADLEQVEVLQKKFDDFQKDL
KANESRLKDINKVAEDLESEGLMAEEVQAVQQQEVYGMMPRDETDSKTASPWKSARLMVH
TVATFNSIKELNERWRSLQQLAEERSQLLGSAHEVQRFHRDADETKEWIEEKNQALNTDN
YGHDLASVQALQRKHEGFERDLAALGDKVNSLGETAERLIQSHPESAEDLQEKCTELNQA
WSSLGKRADQRKAKLGDSHDLQRFLSDFRDLMSWINGIRGLVSSDELAKDVTGAEALLER
HQEHRTEIDARAGTFQAFEQFGQQLLAHGHYASPEIKQKLDILDQERADLEKAWVQRRMM
LDQCLELQLFHRDCEQAENWMAAREAFLNTEDKGDSLDSVEALIKKHEDFDKAINVQEEK
IAALQAFADQLIAAGHYAKGDISSRRNEVLDRWRRLKAQMIEKRSKLGESQTLQQFSRDV
DEIEAWISEKLQTASDESYKDPTNIQSKHQKHQAFEAELHANADRIRGVIDMGNSLIERG
ACAGSEDAVKARLAALADQWQFLVQKSAEKSQKLKEANKQQNFNTGIKDFDFWLSEVEAL
LASEDYGKDLASVNNLLKKHQLLEADISAHEDRLKDLNSQADSLMTSSAFDTSQVKDKRD

TINGRFQKIKSMAASRRAKLNESHRLHQFFRDMDDEESWIKEKKLLVGSEDYGRDLTGVQ
NLRKKHKRLEAELAAHEPAIQGVLDTGKKLSDDNTIGKEEIQQRLAQFVEHWKELKQLAA
ARGQRLEESLEYQQFVANVEEEEAWINEKMTLVASEDYGDTLAAIQGLLKKHEAFETDFT
VHKDRVNDVCTNGQDLIKKNNHHEENISSKMKGLNGKVSDLEKAAAQRKAKLDENSAFLQ
FNWKADVVESWIGEKENSLKTDDYGRDLSSVQTLLTKQETFDAGLQAFQQEGIANITALK
DQLLAAKHVQSKAIEARHASLMKRWSQLLANSAARKKKLLEAQSHFRKVEDLFLTFAKKA
SAFNSWFENAEEDLTDPVRCNSLEEIKALREAHDAFRSSLSSAQADFNQLAELDRQIKSF
RVASNPYTWFTMEALEETWRNLQKIIKERELELQKEQRRQEENDKLRQEFAQHANAFHQW
IQETRTYLLDGSCMVEESGTLESQLEATKRKHQEIRAMRSQLKKIEDLGAAMEEALILDN
KYTEHSTVGLAQQWDQLDQLGMRMQHNLEQQIQARNTTGVTEEALKEFSMMFKHFDKDKS
GRLNHQEFKSCLRSLGYDLPMVEEGEPDPEFEAILDTVDPNRDGHVSLQEYMAFMISRET
ENVKSSEEIESAFRALSSEGKPYVTKEELYQNLTREQADYCVSHMKPYVDGKGRELPTAF
DYVEFTRSLFVN
>sp|Q14240|IF4A2_HUMAN Eukaryotic initiation factor 4A-II OS=Homo sapiens OX=9606
GN=EIF4A2 PE=1 SV=2
MSGGSADYNREHGGPEGMDPDGVIESNWNEIVDNFDDMNLKESLLRGIYAYGFEKPSAIQ
QRAIIPCIKGYDVIAQAQSGTGKTATFAISILQQLEIEFKETQALVLAPTRELAQQIQKV
ILALGDYMGATCHACIGGTNVRNEMQKLQAEAPHIVVGTPGRVFDMLNRRYLSPKWIKMF
VLDEADEMLSRGFKDQIYEIFQKLNTSIQVVLLSATMPTDVLEVTKKFMRDPIRILVKKE
ELTLEGIKQFYINVEREEWKLDTLCDLYETLTITQAVIFLNTRRKVDWLTEKMHARDFTV
SALHGDMDQKERDVIMREFRSGSSRVLITTDLLARGIDVQQVSLVINYDLPTNRENYIHR
IGRGGRFGRKGVAINFVTEEDKRILRDIETFYNTTVEEMPMNVADLI
>sp|Q6BCY4|NB5R2_HUMAN NADH-cytochrome b5 reductase 2 OS=Homo sapiens OX=9606
GN=CYB5R2 PE=1 SV=1
MNSRRREPITLQDPEAKYPLPLIEKEKISHNTRRFRFGLPSPDHVLGLPVGNYVQLLAKI
DNELVVRAYTPVSSDDDRGFVDLIIKIYFKNVHPQYPEGGKMTQYLENMKIGETIFFRGP
RGRLFYHGPGNLGIRPDQTSEPKKTLADHLGMIAGGTGITPMLQLIRHITKDPSDRTRMS
LIFANQTEEDILVRKELEEIARTHPDQFNLWYTLDRPPIGWKYSSGFVTADMIKEHLPPP
AKSTLILVCGPPPLIQTAAHPNLEKLGYTQDMIFTY
>sp|Q6GMV2|SMYD5_HUMAN Histone-lysine N-trimethyltransferase SMYD5 OS=Homo
sapiens OX=9606 GN=SMYD5 PE=1 SV=2
MAASMCDVFSFCVGVAGRARVSVEVRFVSSAKGKGLFATQLIRKGETIFVERPLVAAQFL
WNALYRYRACDHCLRALEKAEENAQRLTGKPGQVLPHPELCTVRKDLHQNCPHCQVMYCS
AECRLAATEQYHQVLCPGPSQDDPLHPLNKLQEAWRSIHYPPETASIMLMARMVATVKQA
KDKDRWIRLFSQFCNKTANEEEEIVHKLLGDKFKGQLELLRRLFTEALYEEAVSQWFTPD
GFRSLFALVGTNGQGIGTSSLSQWVHACDTLELKPQDREQLDAFIDQLYKDIEAATGEFL
NCEGSGLFVLQSCCNHSCVPNAETSFPENNFLLHVTALEDIKPGEEICISYLDCCQRERS
RHSRHKILRENYLFVCSCPKCLAEADEPNVTSEEEEEEEEEEEGEPEDAELGDEMTDV
>sp|Q8IVW8|SPNS2_HUMAN Sphingosine-1-phosphate transporter SPNS2 OS=Homo sapiens
OX=9606 GN=SPNS2 PE=1 SV=2
MMCLECASAAAGGAEEEEADAERRRRRRGAQRGAGGSGCCGARGAGGAGVSAAGDEVQTL
SGSVRRAPTGPPGTPGTPGCAATAKGPGAQQPKPASLGRGRGAAAAILSLGNVLNYLDRY
TVAGVLLDIQQHFGVKDRGAGLLQSVFICSFMVAAPIFGYLGDRFNRKVILSCGIFFWSA
VTFSSSFIPQQYFWLLVLSRGLVGIGEASYSTIAPTIIGDLFTKNTRTLMLSVFYFAIPL

GSGLGYITGSSVKQAAGDWHWALRVSPVLGMITGTLILILVPATKRGHADQLGDQLKART
SWLRDMKALIRNRSYVFSSLATSAVSFATGALGMWIPLYLHRAQVVQKTAETCNSPPCGA
KDSLIFGAITCFTGFLGVVTGAGATRWCRLKTQRADPLVCAVGMLGSAIFICLIFVAAKS
SIVGAYICIFVGETLLFSNWAITADILMYVVIPTRRATAVALQSFTSHLLGDAGSPYLIG
FISDLIRQSTKDSPLWEFLSLGYALMLCPFVVVLGGMFFLATALFFVSDRARAEQQVNQL
AMPPASVKV
>sp|Q8NFZ8|CADM4_HUMAN Cell adhesion molecule 4 OS=Homo sapiens OX=9606 GN=CADM4 PE=1 SV=1
MGRARRFQWPLLLLWAAAAGPGAGQEVQTENVTVAEGGVAEITCRLHQYDGSIVVIQNPA
RQTLFFNGTRALKDERFQLEEFSPRRVRIRLSDARLEDEGGYFCQLYTEDTHHQIATLTV
LVAPENPVVEVREQAVEGGEVELSCLVPRSRPAATLRWYRDRKELKGVSSSQENGKVWSV
ASTVRFRVDRKDDGGIIICEAQNQALPSGHSKQTQYVLDVQYSPTARIHASQAVVREGDT
LVLTCAVTGNPRPNQIRWNRGNESLPERAEAVGETLTLPGLVSADNGTYTCEASNKHGHA
RALYVLVVYDPGAVVEAQTSVPYAIVGGILALLVFLIICVLVGMVWCSVRQKGSYLTHEA
SGLDEQGEAREAFLNGSDGHKRKEEFFI
>sp|Q92597|NDRG1_HUMAN Protein NDRG1 OS=Homo sapiens OX=9606 GN=NDRG1 PE=1 SV=1
MSREMQDVDLAEVKPLVEKGETITGLLQEFDVQEQDIETLHGSVHVTLCGTPKGNRPVIL
TYHDIGMNHKTCYNPLFNYEDMQEITQHFAVCHVDAPGQQDGAASFPAGYMYPSMDQLAE
MLPGVLQQFGLKSIIGMGTGAGAYILTRFALNNPEMVEGLVLINVNPCAEGWMDWAASKI
SGWTQALPDMVVSHLFGKEEMQSNVEVVHTYRQHIVNDMNPGNLHLFINAYNSRRDLEIE
RPMPGTHTVTLQCPALLVVGDSSPAVDAVVECNSKLDPTKTTLLKMADCGGLPQISQPAK
LAEAFKYFVQGMGYMPSASMTRLMRSRTASGSSVTSLDGTRSRSHTSEGTRSRSHTSEGT
RSRSHTSEGAHLDITPNSGAAGNSAGPKSMEVSC
>sp|Q92729|PTPRU_HUMAN Receptor-type tyrosine-protein phosphatase U OS=Homo sapiens OX=9606 GN=PTPRU PE=1 SV=2
MARAQALVLALTFQLCAPETETPAAGCTFEEASDPAVPCEYSQAQYDDFQWEQVRIHPGT
RAPADLPHGSYLMVNTSQHAPGQRAHVIFQSLSENDTHCVQFSYFLYSRDGHSPGTLGVY
VRVNGGPLGSAVWNMTGSHGRQWHQAELAVSTFWPNEYQVLFEALISPDRRGYMGLDDIL
LLSYPCAKAPHFSRLGDVEVNAGQNASFQCMAAGRAAEAERFLLQRQSGALVPAAGVRHI
SHRRFLATFPLAAVSRAEQDLYRCVSQAPRGAGVSNFAELIVKEPPTPIAPPQLLRAGPT
YLIIQLNTNSIIGDGPIVRKEIEYRMARGPWAEVHAVSLQTYKLWHLDPDTEYEISVLLT
RPGDGGTGRPGPPLISRTKCAEPMRAPKGLAFAEIQARQLTLQWEPLGYNVTRCHTYTVS
LCYHYTLGSSHNQTIRECVKTEQGVSRYTIKNLLPYRNVHVRLVLTNPEGRKEGKEVTFQ
TDEDVPSGIAAESLTFTPLEDMIFLKWEEPQEPNGLITQYEISYQSIESSDPAVNVPGPR
RTISKLRNETYHVFSNLHPGTTYLFSVRARTGKGFGQAALTEITTNISAPSFDYADMPSP
LGESENTITVLLRPAQGRGAPISVYQVIVEEERARRLRREPGGQDCFPVPLTFEAALARG
LVHYFGAELAASSLPEAMPFTVGDNQTYRGFWNPPLEPRKAYLIYFQAASHLKGETRLNC
IRIARKAACKESKRPLEVSQRSEEMGLILGICAGGLAVLILLLGAIIVIIRKGRDHYAYS
YYPKPVNMTKATVNYRQEKTHMMSAVDRSFTDQSTLQEDERLGLSFMDTHGYSTRGDQRS
GGVTEASSLLGGSPRRPCGRKGSPYHTGQLHPAVRVADLLQHINQMKTAEGYGFKQEYES
FFEGWDATKKKDKVKGSRQEPMPAYDRHRVKLHPMLGDPNADYINANYIDGYHRSNHFIA
TQGPKPEMVYDFWRMVWQEHCSSIVMITKLVEVGRVKCSRYWPEDSDTYGDIKIMLVKTE
TLAEYVVRTFALERRGYSARHEVRQFHFTAWPEHGVPYHATGLLAFIRRVKASTPPDAGP

IVIHCSAGTGRTGCYIVLDVMLDMAECEGVVDIYNCVKTLCSRRVNMIQTEEQYIFIHDA
ILEACLCGETTIPVSEFKATYKEMIRIDPQSNSSQLREEFQTLNSVTPPLDVEECSIALL
PRNRDKNRSMDVLPPDRCLPFLISTDGDSNNYINAALTDSYTRSAAFIVTLHPLQSTTPD
FWRLVYDYGCTSIVMLNQLNQSNSAWPCLQYWPEPGRQQYGLMEVEFMSGTADEDLVARV
FRVQNISRLQEGHLLVRHFQFLRWSAYRDTPDSKKAFLHLLAEVDKWQAESGDGRTIVHC
LNGGGRSGTFCACATVLEMIRCHNLVDVFFAAKTLRNYKPNMVETMDQYHFCYDVALEYL
EGLESR
>sp|Q969T9|WBP2_HUMAN WW domain-binding protein 2 OS=Homo sapiens OX=9606
GN=WBP2 PE=1 SV=1
MALNKNHSEGGGVIVNNTESILMSYDHVELTFNDMKNVPEAFKGTKKGTVYLTPYRVIFL
SKGKDAMQSFMMPFYLMKDCEIKQPVFGANYIKGTVKAEAGGGWEGSASYKLTFTAGGAI
EFGQRMLQVASQASRGEVPSGAYGYSYMPSGAYVYPPPVANGMYPCPPGYPYPPPPPEFY
PGPPMMDGAMGYVQPPPPPYPGPMEPPVSGPDVPSTPAAEAKAAEAAASAYYNPGNPHNV
YMPTSQPPPPPYYPPEDKKTQ
>sp|Q9BX67|JAM3_HUMAN Junctional adhesion molecule C OS=Homo sapiens OX=9606
GN=JAM3 PE=1 SV=1
MALRRPPRLRLCARLPDFFLLLLFRGCLIGAVNLKSSNRTPVVQEFESVELSCIITDSQT
SDPRIEWKKIQDEQTTYVFFDNKIQGDLAGRAEILGKTSLKIWNVTRRDSALYRCEVVAR
NDRKEIDEIVIELTVQVKPVTPVCRVPKAVPVGKMATLHCQESEGHPRPHYSWYRNDVPL
PTDSRANPRFRNSSFHLNSETGTLVFTAVHKDDSGQYYCIASNDAGSARCEEQEMEVYDL
NIGGIIGGVLVVLAVLALITLGICCAYRRGYFINNKQDGESYKNPGKPDGVNYIRTDEEG
DFRHKSSFVI
>sp|P0DP57|SLUR2_HUMAN Secreted Ly-6/uPAR domain-containing protein 2 OS=Homo
sapiens OX=9606 GN=SLURP2 PE=1 SV=1
MQLGTGLLLAAVLSLQLAAAEAIWCHQCTGFGGCSHGSRCLRDSTHCVTTATRVLSNTED
LPLVTKMCHIGCPDIPSLGLGPYVSIACCQTSLCNHD
>sp|P0DP58|LYNX1_HUMAN Ly-6/neurotoxin-like protein 1 OS=Homo sapiens OX=9606
GN=LYNX1 PE=1 SV=1
MTPLLTLILVVLMGLPLAQALDCHVCAYNGDNCFNPMRCPAMVAYCMTTRTYYTPTRMKV
SKSCVPRCFETVYDGYSKHASTTSCCQYDLCNGTGLATPATLALAPILLATLWGLL
>sp|Q9GZP4|PITH1_HUMAN PITH domain-containing protein 1 OS=Homo sapiens OX=9606
GN=PITHD1 PE=1 SV=1
MSHGHSHGGGGCRCAAEREEPPEQRGLAYGLYLRIDLERLQCLNESREGSGRGVFKPWEE
RTDRSKFVESDADEELLFNIPFTGNVKLKGIIIMGEDDDSHPSEMRLYKNIPQMSFDDTE
REPDQTFSLNRDLTGELEYATKISRFSNVYHLSIHISKNFGADTTKVFYIGLRGEWTELR
RHEVTICNYEASANPADHRVHQVTPQTHFIS
>sp|B3SHH9|TM114_HUMAN Transmembrane protein 114 OS=Homo sapiens OX=9606
GN=TMEM114 PE=1 SV=2
MRVHLGGLAGAAALTGALSFVLLAAAIGTDFWYIIDTERLERTGPGAQDLLGSINRSQPE
PLSSHSGLWRTCRVQSPCTPLMNPFRLENVTVSESSRQLLTMHGTFVILLPLSLILMVFG
GMTGFLSFLLQAYLLLLLTGILFLFGAMVTLAGISVYIAYSAAAFREALCLLEEKALLDQ
VDISFGWSLALGWISFIAELLTGAAFLAAARELSLRRRQDQAI
>sp|O00303|EIF3F_HUMAN Eukaryotic translation initiation factor 3 subunit F OS=Homo
sapiens OX=9606 GN=EIF3F PE=1 SV=1

MATPAVPVSAPPATPTPVPAAAPASVPAPTPAPAAAPVPAAAPASSSDPAAAAAATAAPG
QTPASAQAPAQTPAPALPGPALPGPFPGGRVVRLHPVILASIVDSYERRNEGAARVIGTL
LGTVDKHSVEVTNCFSVPHNESEDEVAVDMEFAKNMYELHKKVSPNELILGWYATGHDIT
EHSVLIHEYYSREAPNPIHLTVDTSLQNGRMSIKAYVSTLMGVPGRTMGVMFTPLTVKYA
YYDTERIGVDLIMKTCFSPNRVIGLSSDLQQVGGASARIQDALSTVLQYAEDVLSGKVSA
DNTVGRFLMSLVNQVPKIVPDDFETMLNSNINDLLMVTYLANLTQSQIALNEKLVNL
>sp|O00442|RTCA_HUMAN RNA 3'-terminal phosphate cyclase OS=Homo sapiens OX=9606 GN=RTCA PE=1 SV=1
MAGPRVEVDGSIMEGGGQILRVSTALSCLLGLPLRVQKIRAGRSTPGLRPQHLSGLEMIR
DLCDGQLEGAEIGSTEITFTPEKIKGGIHTADTKTAGSVCLLMQVSMPCVLFAASPSELH
LKGGTNAEMAPQIDYTVMVFKPIVEKFGFIFNCDIKTRGYYPKGGGEVIVRMSPVKQLNP
INLTERGCVTKIYGRAFVAGVLPFKVAKDMAAAAVRCIRKEIRDLYVNIQPVQEPKDQAF
GNGNGIIIIAETSTGCLFAGSSLGKRGVNADKVGIEAAEMLLANLRHGGTVDEYLQDQLI
VFMALANGVSRIKTGPVTLHTQTAIHFAEQIAKAKFIVKKSEDEEDAAKDTYIIECQGIG
MTNPNL
>sp|O14672|ADA10_HUMAN Disintegrin and metalloproteinase domain-containing protein 10 OS=Homo sapiens OX=9606 GN=ADAM10 PE=1 SV=1
MVLLRVLILLLSWAAGMGGQYGNPLNKYIRHYEGLSYNVDSLHQKHQRAKRAVSHEDQFL
RLDFHAHGRHFNLRMKRDTSLFSDEFKVETSNKVLDYDTSHIYTGHIYGEEGSFSHGSVI
DGRFEGFIQTRGGTFYVEPAERYIKDRTLPFHSVIYHEDDINYPHKYGPQGGCADHSVFE
RMRKYQMTGVEEVTQIPQEEHAANGPELLRKKRTTSAEKNTCQLYIQTDHLFFKYYGTRE
AVIAQISSHVKAIDTIYQTTDFSGIRNISFMVKRIRINTTADEKDPTNPFRFPNIGVEKF
LELNSEQNHDDYCLAYVFTDRDFDDGVLGLAWVGAPSGSSGGICEKSKLYSDGKKKSLNT
GIITVQNYGSHVPPKVSHITFAHEVGHNFGSPHDSGTECTPGESKNLGQKENGNYIMYAR
ATSGDKLNNNKFSLCSIRNISQVLEKKRNNCFVESGQPICGNGMVEQGEECDCGYSDQCK
DECCFDANQPEGRKCKLKPGKQCSPSQGPCCTAQCAFKSKSEKCRDDSDCAREGICNGFT
ALCPASDPKPNFTDCNRHTQVCINGQCAGSICEKYGLEECTCASSDGKDDKELCHVCCMK
KMDPSTCASTGSVQWSRHFSGRTITLQPGSPCNDFRGYCDVFMRCRLVDADGPLARLKKA
IFSPELYENIAEWIVAHWWAVLLMGIALIMLMAGFIKICSVHTPSSNPKLPPPKPLPGTL
KRRRPPQPIQQPQRQRPRESYQMGHMRR
>sp|P00450|CERU_HUMAN Ceruloplasmin OS=Homo sapiens OX=9606 GN=CP PE=1 SV=2
MKILILGIFLFLCSTPAWAKEKHYYIGIIETTWDYASDHGEKKLISVDTEHSNIYLQNGP
DRIGRLYKKALYLQYTDETFRTTIEKPVWLGFLGPIIKAETGDKVYVHLKNLASRPYTFH
SHGITYYKEHEGAIYPDNTTDFQRADDKVYPGEQYTYMLLATEEQSPGEGDGNCVTRIYH
SHIDAPKDIASGLIGPLIICKKDSLDKEKEKHIDREFVVMFSVVDENFSWYLEDNIKTYC
SEPEKVDKDNEDFQESNRMYSVNGYTFGSLPGLSMCAEDRVKWYLFGMGNEVDVHAAFFH
GQALTNKNYRIDTINLFPATLFDAYMVAQNPGEWMLSCQNLNHLKAGLQAFFQVQECNKS
SSKDNIRGKHVRHYYIAAEEIIWNYAPSGIDIFTKENLTAPGSDSAVFFEQGTTRIGGSY
KKLVYREYTDASFTNRKERGPEEEHLGILGPVIWAEVGDTIRVTFHNKGAYPLSIEPIGV
RFNKNNEGTYYSPNYNPQSRSVPPSASHVAPTETFTYEWTVPKEVGPTNADPVCLAKMYY
SAVEPTKDIFTGLIGPMKICKKGSLHANGRQKDVDKEFYLFPTVFDENESLLLEDNIRMF
TTAPDQVDKEDEDFQESNKMHSMNGFMYGNQPGLTMCKGDSVVWYLFSAGNEADVHGIYF
SGNTYLWRGERRDTANLFPQTSLTLHMWPDTEGTFNVECLTTDHYTGGMKQKYTVNQCRR
QSEDSTFYLGERTYYIAAVEVEWDYSPQREWEKELHHLQEQNVSNAFLDKGEFYIGSKYK

KVVYRQYTDSTFRVPVERKAEEEHLGILGPQLHADVGDKVKIIFKNMATRPYSIHAHGVQ
TESSTVTPTLPGETLTYVWKIPERSGAGTEDSACIPWAYYSTVDQVKDLYSGLIGPLIVC
RRPYLKVFNPRRKLEFALLFLVFDENESWYLDDNIKTYSDHPEKVNKDDEEFIESNKMHA
INGRMFGNLQGLTMHVGDEVNWYLMGMGNEIDLHTVHFHGHSFQYKHRGVYSSDVFDIFP
GTYQTLEMFPRTPGIWLLHCHVTDHIHAGMETTYTVLQNEDTKSG
>sp|P00491|PNPH_HUMAN Purine nucleoside phosphorylase OS=Homo sapiens OX=9606 GN=PNP PE=1 SV=2
MENGYTYEDYKNTAEWLLSHTKHRPQVAIICGSGLGGLTDKLTQAQIFDYGEIPNFPRST
VPGHAGRLVFGFLNGRACVMMQGRFHMYEGYPLWKVTFPVRVFHLLGVDTLVVTNAAGGL
NPKFEVGDIMLIRDHINLPGFSGQNPLRGPNDERFGDRFPAMSDAYDRTMRQRALSTWKQ
MGEQRELQEGTYVMVAGPSFETVAECRVLQKLGADAVGMSTVPEVIVARHCGLRVFGFSL
ITNKVIMDYESLEKANHEEVLAAGKQAAQKLEQFVSILMASIPLPDKAS
>sp|P18124|RL7_HUMAN Large ribosomal subunit protein uL30 OS=Homo sapiens OX=9606 GN=RPL7 PE=1 SV=1
MEGVEEKKKEVPAVPETLKKKRRNFAELKIKRLRKKFAQKMLRKARRKLIYEKAKHYHKE
YRQMYRTEIRMARMARKAGNFYVPAEPKLAFVIRIRGINGVSPKVRKVLQLLRLRQIFNG
TFVKLNKASINMLRIVEPYIAWGYPNLKSVNELIYKRGYGKINKKRIALTDNALIARSLG
KYGIICMEDLIHEIYTVGKRFKEANNFLWPFKLSSPRGGMKKKTTHFVEGGDAGNREDQI
NRLIRRMN
>sp|P26232|CTNA2_HUMAN Catenin alpha-2 OS=Homo sapiens OX=9606 GN=CTNNA2 PE=1 SV=5
MTSATSPIILKWDPKSLEIRTLTVERLLEPLVTQVTTLVNTSNKGPSGKKKGRSKKAHVL
AASVEQATQNFLEKGEQIAKESQDLKEELVAAVEDVRKQGETMRIASSEFADDPCSSVKR
GTMVRAARALLSAVTRLLILADMADVMRLLSHLKIVEEALEAVKNATNEQDLANRFKEFG
KEMVKLNYVAARRQQELKDPHCRDEMAAARGALKKNATMLYTASQAFLRHPDVAATRANR
DYVFKQVQEAIAGISNAAQATSPTDEAKGHTGIGELAAALNEFDNKIILDPMTFSEARFR
PSLEERLESIISGAALMADSSCTRDDRRERIVAECNAVRQALQDLLSEYMNNTGRKEKGD
PLNIAIDKMTKKTRDLRRQLRKAVMDHISDSFLETNVPLLVLIEAAKSGNEKEVKEYAQV
FREHANKLVEVANLACSISNNEEGVKLVRMAATQIDSLCPQVINAALTLAARPQSKVAQD
NMDVFKDQWEKQVRVLTEAVDDITSVDDFLSVSENHILEDVNKCVIALQEGDVDTLDRTA
GAIRGRAARVIHIINAEMENYEAGVYTEKVLEATKLLSETVMPRFAEQVEVAIEALSANV
PQPFEENEFIDASRLVYDGVRDIRKAVLMIRTPEELEDDSDFEQEDYDVRSRTSVQTEDD
QLIAGQSARAIMAQLPQEEKAKIAEQVEIFHQEKSKLDAEVAKWDDSGNDIIVLAKQMCM
IMMEMTDFTRGKGPLKNTSDVINAAKKIAEAGSRMDKLARAVADQCPDSACKQDLLAYLQ
RIALYCHQLNICSKVKAEVQNLGGELIVSGTGVQSTFTTFYEVDCDVIDGGRASQLSTHL
PTCAEGAPIGSGSSDSSMLDSATSLIQAAKNLMNAVVLTVKASYVASTKYQKVYGTAAVN
SPVVSWKMKAPEKKPLVKREKPEEFQTRVRRGSQKKHISPVQALSEFKAMDSF
>sp|P31946|1433B_HUMAN 14-3-3 protein beta/alpha OS=Homo sapiens OX=9606 GN=YWHAB PE=1 SV=3
MTMDKSELVQKAKLAEQAERYDDMAAAMKAVTEQGHELSNEERNLLSVAYKNVVGARRSS
WRVISSIEQKTERNEKKQQMGKEYREKIEAELQDICNDVLELLDKYLIPNATQPESKVFY
LKMKGDYFRYLSEVASGDNKQTTVSNSQQAYQEAFEISKKEMQPTHPIRLGLALNFSVFY
YEILNSPEKACSLAKTAFDEAIAELDTLNEESYKDSTLIMQLLRDNLTLWTSENQGDEGD
AGEGEN

>sp|P36955|PEDF_HUMAN Pigment epithelium-derived factor OS=Homo sapiens OX=9606 GN=SERPINF1 PE=1 SV=4
MQALVLLLCIGALLGHSSCQNPASPPEEGSPDPDSTGALVEEEDPFFKVPVNKLAAAVSN
FGYDLYRVRSSTSPTTNVLLSPLSVATALSALSLGAEQRTESIIHRALYYDLISSPDIHG
TYKELLDTVTAPQKNLKSASRIVFEKKLRIKSSFVAPLEKSYGTRPRVLTGNPRLDLQEI
NNWVQAQMKGKLARSTKEIPDEISILLLGVAHFKGQWVTKFDSRKTSLEDFYLDEERTVR
VPMMSDPKAVLRYGLDSDLSCKIAQLPLTGSMSIIFFLPLKVTQNLTLIEESLTSEFIHD
IDRELKTVQAVLTVPKLKLSYEGEVTKSLQEMKLQSLFDSPDFSKITGKPIKLTQVEHRA
GFEWNEDGAGTTPSPGLQPAHLTFPLDYHLNQPFIFVLRDTDTGALLFIGKILDPRGP
>sp|P40925|MDHC_HUMAN Malate dehydrogenase, cytoplasmic OS=Homo sapiens OX=9606 GN=MDH1 PE=1 SV=4
MSEPIRVLVTGAAGQIAYSLLYSIGNGSVFGKDQPIILVLLDITPMMGVLDGVLMELQDC
ALPLLKDVIATDKEDVAFKDLDVAILVGSMPRREGMERKDLLKANVKIFKSQGAALDKYA
KKSVKVIVVGNPANTNCLTASKSAPSIPKENFSCLTRLDHNRAKAQIALKLGVTANDVKN
VIIWGNHSSTQYPDVNHAKVKLQGKEVGVYEALKDDSWLKGEFVTTVQQRGAAVIKARKL
SSAMSAAKAICDHVRDIWFGTPEGEFVSMGVISDGNSYGVPDDLLYSFPVVIKNKTWKFV
EGLPINDFSREKMDLTAKELTEEKESAFEFLSSA
>sp|P49327|FAS_HUMAN Fatty acid synthase OS=Homo sapiens OX=9606 GN=FASN PE=1 SV=3
MEEVVIAGMSGKLPESENLQEFWDNLIGGVDMVTDDDRRWKAGLYGLPRRSGKLKDLSRF
DASFFGVHPKQAHTMDPQLRLLLEVTYEAIVDGGINPDSLRGTHTGVWVGVSGSETSEAL
SRDPETLVGYSMVGCQRAMMANRLSFFFDFRGPSIALDTACSSSLMALQNAYQAIHSGQC
PAAIVGGINVLLKPNTSVQFLRLGMLSPEGTCKAFDTAGNGYCRSEGVVAVLLTKKSLAR
RVYATILNAGTNTDGFKEQGVTFPSGDIQEQLIRSLYQSAGVAPESFEYIEAHGTGTKVG
DPQELNGITRALCATRQEPLLIGSTKSNMGHPEPASGLAALAKVLLSLEHGLWAPNLHFH
SPNPEIPALLDGRLQVVDQPLPVRGGNVGINSFGFGGSNVHIILRPNTQPPPAPAPHATL
PRLLRASGRTPEAVQKLLEQGLRHSQDLAFLSMLNDIAAVPATAMPFRGYAVLGGERGGP
EVQQVPAGERPLWFICSGMGTQWRGMGLSLMRLDRFRDSILRSDEAVKPFGLKVSQLLLS
TDESTFDDIVHSFVSLTAIQIGLIDLLSCMGLRPDGIVGHSLGEVACGYADGCLSQEEAV
LAAYWRGQCIKEAHLPPGAMAAVGLSWEECKQRCPPGVVPACHNSKDTVTISGPQAPVFE
FVEQLRKEGVFAKEVRTGGMAFHSYFMEAIAPPLLQELKKVIREPKPRSARWLSTSIPEA
QWHSSLARTSSAEYNVNNLVSPVLFQEALWHVPEHAVVLEIAPHALLQAVLKRGLKPSCT
IIPLMKKDHRDNLEFFLAGIGRLHLSGIDANPNALFPPVEFPAPRGTPLISPLIKWDHSL
AWDVPAAEDFPNGSGSPSAAIYNIDTSSESPDHYLVDHTLDGRVLFPATGYLSIVWKTLA
RALGLGVEQLPVVFEDVVLHQATILPKTGTVSLEVRLLEASRAFEVSENGNLVVSGKVYQ
WDDPDPRLFDHPESPTPNPTEPLFLAQAEVYKELRLRGYDYGPHFQGILEASLEGDSGRL
LWKDNWVSFMDTMLQMSILGSAKHGLYLPTRVTAIHIDPATHRQKLYTLQDKAQVADVVV
SRWLRVTVAGGVHISGLHTESAPRRQQEQQVPILEKFCFTPHTEEGCLSERAALQEELQL
CKGLVQALQTKVTQQGLKMVVPGLDGAQIPRDPSQQELPRLLSAACRLQLNGNLQLELAQ
VLAQERPKLPEDPLLSGLLDSPALKACLDTAVENMPSLKMKVVEVLAGHGHLYSRIPGLL
SPHPLLQLSYTATDRHPQALEAAQAELQQHDVAQGQWDPADPAPSALGSADLLVCNCAVA
ALGDPASALSNMVAALREGGFLLLHTLLRGHPLGDIVAFLTSTEPQYGQGILSQDAWESL
FSRVSLRLVGLKKSFYGSTLFLCRRPTPQDSPIFLPVDDTSFRWVESLKGILADEDSSRP
VWLKAINCATSGVVGLVNCLRREPGGNRLRCVLLSNLSSTSHVPEVDPGSAELQKVLQGD
LVMNVYRDGAWGAFRHFLLEEDKPEEPTAHAFVSTLTRGDLSSIRWVCSSLRHAQPTCPG

AQLCTVYYASLNFRDIMLATGKLSPDAIPGKWTSQDSLLGMEFSGRDASGKRVMGLVPAK
GLATSVLLSPDFLWDVPSNWTLEEAASVPVVYSTAYYALVVRGRVRPGETLLIHSGSGGV
GQAAIAIALSLGCRVFTTVGSAEKRAYLQARFPQLDSTSFANSRDTSFEQHVLWHTGGKG
VDLVLNSLAEEKLQASVRCLATHGRFLEIGKFDLSQNHPLGMAIFLKNVTFHGVLLDAFF
NESSADWREVWALVQAGIRDGVVRPLKCTVFHGAQVEDAFRYMAQGKHIGKVVVQVLAEE
PEAVLKGAKPKLMSAISKTFCPAHKSYIIAGGLGGFGLELAQWLIQRGVQKLVLTSRSGI
RTGYQAKQVRRWRRQGVQVQVSTSNISSLEGARGLIAEAAQLGPVGGVFNLAVVLRDGLL
ENQTPEFFQDVCKPKYSGTLNLDRVTREACPELDYFVVFSSVSCGRGNAGQSNYGFANSA
MERICEKRRHEGLPGLAVQWGAIGDVGILVETMSTNDTIVSGTLPQRMASCLEVLDLFLN
QPHMVLSSFVLAEKAAAYRDRDSQRDLVEAVAHILGIRDLAAVNLDSSLADLGLDSLMSV
EVRQTLERELNLVLSVREVRQLTLRKLQELSSKADEASELACPTPKEDGLAQQQTQLNLR
SLLVNPEGPTLMRLNSVQSSERPLFLVHPIEGSTTVFHSLASRLSIPTYGLQCTRAAPLD
SIHSLAAYYIDCIRQVQPEGPYRVAGYSYGACVAFEMCSQLQAQQSPAPTHNSLFLFDGS
PTYVLAYTQSYRAKLTPGCEAEAETEAICFFVQQFTDMEHNRVLEALLPLKGLEERVAAA
VDLIIKSHQGLDRQELSFAARSFYYKLRAAEQYTPKAKYHGNVMLLRAKTGGAYGEDLGA
DYNLSQVCDGKVSVHVIEGDHRTLLEGSGLESIISIIHSSLAEPRVSVREG
>sp|P54289|CA2D1_HUMAN Voltage-dependent calcium channel subunit alpha-2/delta-1
OS=Homo sapiens OX=9606 GN=CACNA2D1 PE=1 SV=3
MAAGCLLALTLTLFQSLLIGPSSEEPFPSAVTIKSWVDKMQEDLVTLAKTASGVNQLVDI
YEKYQDLYTVEPNNARQLVEIAARDIEKLLSNRSKALVRLALEAEKVQAAHQWREDFASN
EVVYYNAKDDLDPEKNDSEPGSQRIKPVFIEDANFGRQISYQHAAVHIPTDIYEGSTIVL
NELNWTSALDEVFKKNREEDPSLLWQVFGSATGLARYYPASPWVDNSRTPNKIDLYDVRR
RPWYIQGAASPKDMLILVDVSGSVSGLTLKLIRTSVSEMLETLSDDDFVNVASFNSNAQD
VSCFQHLVQANVRNKKVLKDAVNNITAKGITDYKKGFSFAFEQLLNYNVSRANCNKIIML
FTDGGEERAQEIFNKYNKDKKVRVFTFSVGQHNYDRGPIQWMACENKGYYYEIPSIGAIR
INTQEYLDVLGRPMVLAGDKAKQVQWTNVYLDALELGLVITGTLPVFNITGQFENKTNLK
NQLILGVMGVDVSLEDIKRLTPRFTLCPNGYYFAIDPNGYVLLHPNLQPKPIGVGIPTIN
LRKRRPNIQNPKSQEPVTLDFLDAELENDIKVEIRNKMIDGESGEKTFRTLVKSQDERYI
DKGNRTYTWTPVNGTDYSLALVLPTYSFYYIKAKLEETITQARYSETLKPDNFEESGYTF
IAPRDYCNDLKISDNNTEFLLNFNEFIDRKTPNNPSCNADLINRVLLDAGFTNELVQNYW
SKQKNIKGVKARFVVTDGGITRVYPKEAGENWQENPETYEDSFYKRSLDNDNYVFTAPYF
NKSGPGAYESGIMVSKAVEIYIQGKLLKPAVVGIKIDVNSWIENFTKTSIRDPCAGPVCD
CKRNSDVMDCVILDDGGFLLMANHDDYTNQIGRFFGEIDPSLMRHLVNISVYAFNKSYDY
QSVCEPGAAPKQGAGHRSAYVPSVADILQIGWWATAAAWSILQQFLLSLTFPRLLEAVEM
EDDDFTASLSKQSCITEQTQYFFDNDSKSFSGVLDCGNCSRIFHGEKLMNTNLIFIMVES
KGTCPCDTRLLIQAEQTSDGPNPCDMVKQPRYRKGPDVCFDNNVLEDYTDCGGVSGLNPS
LWYIIGIQFLLLWLVSGSTHRLL
>sp|P60842|IF4A1_HUMAN Eukaryotic initiation factor 4A-I OS=Homo sapiens OX=9606
GN=EIF4A1 PE=1 SV=1
MSASQDSRSRDNGPDGMEPEGVIESNWNEIVDSFDDMNLSESLLRGIYAYGFEKPSAIQQ
RAILPCIKGYDVIAQAQSGTGKTATFAISILQQIELDLKATQALVLAPTRELAQQIQKVV
MALGDYMGASCHACIGGTNVRAEVQKLQMEAPHIIVGTPGRVFDMLNRRYLSPKYIKMFV
LDEADEMLSRGFKDQIYDIFQKLNSNTQVVLLSATMPSDVLEVTKKFMRDPIRILVKKEE
LTLEGIRQFYINVEREEWKLDTLCDLYETLTITQAVIFINTRRKVDWLTEKMHARDFTVS

AMHGDMDQKERDVIMREFRSGSSRVLITTDLLARGIDVQQVSLVINYDLPTNRENYIHRI
GRGGRFGRKGVAINMVTEEDKRTLRDIETFYNTSIEEMPLNVADLI
>sp|P68104|EF1A1_HUMAN Elongation factor 1-alpha 1 OS=Homo sapiens OX=9606 GN=EEF1A1 PE=1 SV=1
MGKEKTHINIVVIGHVDSGKSTTTGHLIYKCGGIDKRTIEKFEKEAAEMGKGSFKYAWVL
DKLKAERERGITIDISLWKFETSKYYVTIIDAPGHRDFIKNMITGTSQADCAVLIVAAGV
GEFEAGISKNGQTREHALLAYTLGVKQLIVGVNKMDSTEPPYSQKRYEEIVKEVSTYIKK
IGYNPDTVAFVPISGWNGDNMLEPSANMPWFKGWKVTRKDGNASGTTLLEALDCILPPTR
PTDKPLRLPLQDVYKIGGIGTVPVGRVETGVLKPGMVVTFAPVNVTTEVKSVEMHHEALS
EALPGDNVGFNVKNVSVKDVRRGNVAGDSKNDPPMEAAGFTAQVIILNHPGQISAGYAPV
LDCHTAHIACKFAELKEKIDRRSGKKLEDGPKFLKSGDAAIVDMVPGKPMCVESFSDYPP
LGRFAVRDMRQTVAVGVIKAVDKKAAGAGKVTKSAQKAQKAK
>sp|Q07283|TRHY_HUMAN Trichohyalin OS=Homo sapiens OX=9606 GN=TCHH PE=1 SV=2
MSPLLRSICDITEIFNQYVSHDCDGAALTKKDLKNLLEREFGAVLRRPHDPKTVDLILEL
LDLDSNGRVDFNEFLLFIFKVAQACYYALGQATGLDEEKRARCDGKESLLQDRRQEEDQR
RFEPRDRQLEEEPGQRRRQKRQEQERELAEGEEQSEKQERLEQRDRQRRDEELWRQRQEW
QEREERRAEEEQLQSCKGHETEEFPDEEQLRRRELLELRRKGREEKQQQRRERQDRVFQE
EEEKEWRKRETVLRKEEEKLQEEEPQRQRELQEEEEQLRKLERQELRRERQEEEQQQQRL
RREQQLRRKQEEERREQQEERREQQERREQQEERREQQLRREQEERREQQLRREQEEERR
EQQLRREQEEERREQQLRREQQLRREQQLRREQQLRREQQLRREQQLRREQQLRREQQLR
REQQLRREQEEERHEQKHEQERREQRLKREQEERRDWLKREEETERHEQERRKQQLKRDQ
EEERRERWLKLEEEERREQQERREQQLRREQEERREQRLKRQEEEERLQQRLRSEQQLRR
EQEERREQLLKREEEKRLEQERREQRLKREQEERRDQLLKREEERRQQRLKREQEERLEQ
RLKREEVERLEQEERREQRLKREEPEEERRQQLLKSEEQEERRQQQLRREQQERREQRLK
REEEEERLEQRLKREHEEERREQELAEEEQEQARERIKSRIPKWQWQLESEADARQSKVY
SRPRKQEGQRRRQEQEEKRRRRESELQWQEEERAHRQQQEEEQRRDFTWQWQAEEKSERG
RQRLSARPPLREQRERQLRAEERQQREQRFLPEEEEKEQRRRQRREREKELQFLEEEEQL
QRRERAQQLQEEEDGLQEDQERRRSQEQRRDQKWRWQLEEERKRRRHTLYAKPALQEQLR
KEQQLLQEEEEELQREEREKRRRQEQERQYREEEQLQQEEEQLLREEREKRRRQERERQY
RKDKKLQQKEEQLLGEEPEKRRRQEREKKYREEEELQQEEEQLLREEREKRRRQEWERQY
RKKDELQQEEEQLLREEREKRRLQERERQYREEEELQQEEEQLLGEERETRRRQELERQY
RKEEELQQEEEQLLREEPEKRRRQERERQCREEEELQQEEEQLLREEREKRRRQELERQY
REEEEVQQEEEQLLREEPEKRRRQELERQYREEEELQQEEEQLLREEQEKRRQERERQYR
EEEELQRQKRKQRYRDEDQRSDLKWQWEPEKENAVRDNKVYCKGRENEQFRQLEDSQLRD
RQSQQDLQHLLGEQQERDREQERRRWQQRDRHFPEEEQLEREEQKEAKRRDRKSQEEKQL
LREEREEKRRRQETDRKFREEEQLLQEREEQPLRRQERDRKFREEELRHQEQGRKFLEEE
QRLRRQERERKFLKEEQQLRCQEREQQLRQDRDRKFREEEQQLSRQERDRKFREEEQQVR
RQERERKFLEEEQQLRQERHRKFREEEQLLQEREEQQLHRQERDRKFLEEEQQLRRQERD
RKFREQELRSQEPERKFLEEEQQLHRQQRQRKFLQEEQQLRRQERGQQRRQDRDRKFREE
EQLRQEREEQQLSRQERDRKFRLEEQKVRRQEQERKFMEDEQQLRRQEGQQQLRQERDRK
FREDEQLLQEREEQQLHRQERDRKFLEEEPQLRRQEREQQLRHDRDRKFREEEQLLQEGE
EQQLRRQERDRKFREEEQQLRRQERERKFLQEEQQLRRQELERKFREEEQLRQETEQEQL
RRQERYRKILEEEQLRPEREEQQLRRQERDRKFREEEQLRQEREEQQLRSQESDRKFREE
EQLRQEREEQQLRPQQRDGKYRWEEEQLQLEEQEQRLRQERDRQYRAEEQFATQEKSRRE

EQELWQEEEQKRRQERERKLREEHIRRQQKEEQRHRQVGEIKSQEGKGHGRLLEPGTHQF
ASVPVRSSPLYEYIQEQRSQYRP
>sp|Q09666|AHNK_HUMAN Neuroblast differentiation-associated protein AHNAK OS=Homo sapiens OX=9606 GN=AHNAK PE=1 SV=2
MEKEETTRELLLPNWQGSGSHGLTIAQRDDGVFVQEVTQNSPAARTGVVKEGDQIVGATI
YFDNLQSGEVTQLLNTMGHHTVGLKLHRKGDRSPEPGQTWTREVFSSCSSEVVLSGDDEE
YQRIYTTKIKPRLKSEDGVEGDLGETQSRTITVTRRVTAYTVDVTGREGAKDIDISSPEF
KIKIPRHELTEISNVDVETQSGKTVIRLPSGSGAASPTGSAVDIRAGAISASGPELQGAG
HSKLQVTMPGIKVGGSGVNVNAKGLDLGGRGGVQVPAVDISSSLGGRAVEVQGPSLESGD
HGKIKFPTMKVPKFGVSTGREGQTPKAGLRVSAPEVSVGHKGGKPGLTIQAPQLEVSVPS
ANIEGLEGKLKGPQITGPSLEGDLGLKGAKPQGHIGVDASAPQIGGSITGPSVEVQAPDI
DVQGPGSKLNVPKMKVPKFSVSGAKGEETGIDVTLPTGEVTVPGVSGDVSLPEIATGGLE
GKMKGTKVKTPEMIIQKPKISMQDVDLSLGSPKLKGDIKVSAPGVQGDVKGPQVALKGSR
VDIETPNLEGTLTGPRLGSPSGKTGTCRISMSEVDLNVAAPKVKGGVDVTLPRVEGKVKV
PEVDVRGPKVDVSAPDVEAHGPEWNLKMPKMKMPTFSTPGAKGEGPDVHMTLPKGDISIS
GPKVNVEAPDVNLEGLGGKLKGPDVKLPDMSVKTPKISMPDVDLHVKGTKVKGEYDVTVP
KLEGELKGPKVDIDAPDVDVHGPDWHLKMPKMKMPKFSVPGFKAEGPEVDVNLPKADVDI
SGPKIDVTAPDVSIEEPEGKLKGPKFKMPEMNIKVPKISMPDVDLHLKGPNVKGEYDVTM
PKVESEIKVPDVELKSAKMDIDVPDVEVQGPDWHLKMPKMKMPKFSMPGFKAEGPEVDVN
LPKADVDISGPKVGVEVPDVNIEGPEGKLKGPKFKMPEMNIKAPKISMPDVDLHMKGPKV
KGEYDMTVPKLEGDLKGPKVDVSAPDVEMQGPDWNLKMPKIKMPKFSMPSLKGEGPEFDV
NLSKANVDISAPKVDTNAPDLSLEGPEGKLKGPKFKMPEMHFRAPKMSLPDVDLDLKGPK
MKGNVDISAPKIEGEMQVPDVDIRGPKVDIKAPDVEGQGLDWSLKIPKMKMPKFSMPSLK
GEGPEVDVNLPKADVVVSGPKVDIEAPDVSLEGPEGKLKGPKFKMPEMHFKTPKISMPDV
DLHLKGPKVKGDVDVSVPKVEGEMKVPDVEIKGPKMDIDAPDVEVQGPDWHLKMPKMKMP
KFSMPGFKGEGREVDVNLPKADIDVSGPKVDVEVPDVSLEGPEGKLKGPKFKMPEMHFKA
PKISMPDVDLNLKGPKLKGDVDVSLPEVEGEMKVPDVDIKGPKVDISAPDVDVHGPDWHL
KMPKVKMPKFSMPGFKGEGPEVDVKLPKADVDVSGPKMDAEVPDVNIEGPDAKLKGPKFK
MPEMSIKPQKISIPDVGLHLKGPKMKGDYDVTVPKVEGEIKAPDVDIKGPKVDINAPDVE
VHGPDWHLKMPKVKMPKFSMPGFKGEGPEVDMNLPKADLGVSGPKVDIDVPDVNLEAPEG
KLKGPKFKMPSMNIQTHKISMPDVGLNLKAPKLKTDVDVSLPKVEGDLKGPEIDVKAPKM
DVNVGDIDIEGPEGKLKGPKFKMPEMHFKAPKISMPDVDLHLKGPKVKGDMDVSVPKVEG
EMKVPDVDIKGPKVDIDAPDVEVHDPDWHLKMPKMKMPKFSMPGFKAEGPEVDVNLPKAD
IDVSGPSVDTDAPDLDIEGPEGKLKGSKFKMPKLNIKAPKVSMPDVDLNLKGPKLKGEID
ASVPELEGDLRGPQVDVKGPFVEAEVPDVDLECPDAKLKGPKFKMPEMHFKAPKISMPDV
DLHLKGPKVKGDADVSVPKLEGDLTGPSVGVEVPDVELECPDAKLKGPKFKMPDMHFKAP
KISMPDVDLHLKGPKVKGDVDVSVPKLEGDLTGPSVGVEVPDVELECPDAKLKGPKFKMP
EMHFKTPKISMPDVDLHLKGPKVKGDMDVSVPKVEGEMKVPDVDIKGPKMDIDAPDVDVH
GPDWHLKMPKMKMPKFSMPGFKAEGPEVDVNLPKADVVVSGPKVDVEVPDVSLEGPEGKL
KGPKLKMPEMHFKAPKISMPDVDLHLKGPKVKGDVDVSLPKLEGDLTGPSVDVEVPDVEL
ECPDAKLKGPKFKMPEMHFKTPKISMPDVNLNLKGPKVKGDMDVSVPKVEGEMKVPDVDI
RGPKVDIDAPDVDVHGPDWHLKMPKMKMPKFSMPGFKGEGPEVDVNLPKADVDVSGPKVD
VEVPDVSLEGPEGKLKGPKFKMPEMHFKTPKISMPDVDFNLKGPKIKGDVDVSAPKLEGE
LKGPELDVKGPKLDADMPEVAVEGPNGKWTPKFKMPDMHFKAPKISMPDLDLHLKSPKA

KGEVDVDVPKLEGDLKGPHVDVSGPDIDIEGPEGKLKGPKFKMPDMHFKAPNISMPDVDL
NLKGPKIKGDVDVSVPEVEGKLEVPDMNIRGPKVDVNAPDVQAPDWHLKMPKMKMPKFSM
PGFKAEGPEVDVNLPKADVDISGPKVDIEGPDVNIEGPEGKLKGPKLKMPEMNIKAPKIS
MPDFDLHLKGPKVKGDVDVSLPKVEGDLKGPEVDIKGPKVDINAPDVGVQGPDWHLKMPK
VKMPKFSMPGFKGEGPDGDVKLPKADIDVSGPKVDIEGPDVNIEGPEGKLKGPKFKMPEM
NIKAPKISMPDIDLNLKGPKVKGDVDVSLPKVEGDLKGPEVDIKGPKVDIDAPDVDVHGP
DWHLKMPKIKMPKISMPGFKGEGPDVDVNLPKADIDVSGPKVDVECPDVNIEGPEGKWKS
PKFKMPEMHFKTPKISMPDIDLNLTGPKIKGDVDVTGPKVEGDLKGPEVDLKGPKVDIDV
PDVNVQGPDWHLKMPKMKMPKFSMPGFKAEGPEVDVNLPKADVDVSGPKVDVEGPDVNIE
GPEGKLKGPKFKMPEMNIKAPKIPMPDFDLHLKGPKVKGDVDISLPKVEGDLKGPEVDIR
GPQVDIDVPDVGVQGPDWHLKMPKVKMPKFSMPGFKGEGPDVDVNLPKADLDVSGPKVDI
DVPDVNIEGPEGKLKGPKFKMPEMNIKAPKISMPDIDLNLKGPKVKGDMDVSLPKVEGDM
KVPDVDIKGPKVDINAPDVDVQGPDWHLKMPKIKMPKISMPGFKGEGPEVDVNLPKADLD
VSGPKVDVDVPDVNIEGPDAKLKGPKFKMPEMNIKAPKISMPDLDLNLKGPKMKGEVDVS
LANVEGDLKGPALDIKGPKIDVDAPDIDIHGPDAKLKGPKLKMPDMHVNMPKISMPEIDL
NLKGSKLKGDVDVSGPKLEGDIKAPSLDIKGPEVDVSGPKLNIEGKSKKSRFKLPKFNFS
GSKVQTPEVDVKGKKPDIDITGPKVDINAPDVEVQGKVKGSKFKMPFLSISSPKVSMPDV
ELNLKSPKVKGDLDIAGPNLEGDFKGPKVDIKAPEVNLNAPDVDVHGPDWNLKMPKMKMP
KFSVSGLKAEGPDVAVDLPKGDINIEGPSMNIEGPDLNVEGPEGGLKGPKFKMPDMNIKA
PKISMPDIDLNLKGPKVKGDVDISLPKLEGDLKGPEVDIKGPKVDINAPDVDVHGPDWHL
KMPKVKMPKFSMPGFKGEGPEVDVTLPKADIDISGPNVDVDVPDVNIEGPDAKLKGPKFK
MPEMNIKAPKISMPDFDLNLKGPKMKGDVVVSLPKVEGDLKGPEVDIKGPKVDIDTPDIN
IEGSEGKFKGPKFKIPEMHLKAPKISMPDIDLNLKGPKVKGDVDVSLPKMEGDLKGPEVD
IKGPKVDINAPDVDVQGPDWHLKMPKVKMPKFSMPGFKGEGPDVDVNLPKADLDVSGPKV
DIDVPDVNIEGPEGKLKGPKFKMPEMNIKAPKISMPDIDLNLKGPKVKGDMDVSLPKVEG
DMQVPDLDIKGPKVDINAPDVDVRGPDWHLKMPKIKMPKISMPGFKGEGPEVDVNLPKAD
LDVSGPKVDVDVPDVNIEGPDAKLKGPKFKMPEMNIKAPKISMPDFDLHLKGPKVKGDVD
VSLPKMEGDLKAPEVDIKGPKVDIDAPDVDVHGPDWHLKMPKVKMPKFSMPGFKGEGPEV
DVNLPKADIDVSGPKVDIDTPDIDIHGPEGKLKGPKFKMPDLHLKAPKISMPEVDLNLKG
PKMKGDVDVSLPKVEGDLKGPEVDIKGPKVDIDVPDVDVQGPDWHLKMPKVKMPKFSMPG
FKGEGPDVDVNLPKADLDVSGPKVDIDVPDVNIEGPDAKLKGPKFKMPEMNIKAPKISMP
DFDLHLKGPKVKGDVDVSLPKVEGDLKGPEVDIKGPKVDIDAPDVDVHGPDWHLKMPKVK
MPKFSMPGFKGEGPDVDVTLPKADIEISGPKVDIDAPDVSIEGPDAKLKGPKFKMPEMNI
KAPKISMPDIDFNLKGPKVKGDVDVSLPKVEGDLKGPEIDIKGPSLDIDTPDVNIEGPEG
KLKGPKFKMPEMNIKAPKISMPDFDLHLKGPKVKGDVDVSLPKVESDLKGPEVDIEGPEG
KLKGPKFKMPDVHFKSPQISMSDIDLNLKGPKIKGDMDISVPKLEGDLKGPKVDVKGPKV
GIDTPDIDIHGPEGKLKGPKFKMPDLHLKAPKISMPEVDLNLKGPKVKGDMDISLPKVEG
DLKGPEVDIRDPKVDIDVPDVDVQGPDWHLKMPKVKMPKFSMPGFKGEGPDVDVNLPKAD
IDVSGPKVDVDVPDVNIEGPDAKLKGPKFKMPEMSIKAPKISMPDIDLNLKGPKVKGDVD
VTLPKVEGDLKGPEADIKGPKVDINTPDVDVHGPDWHLKMPKVKMPKFSMPGFKGEGPDV
DVSLPKADIDVSGPKVDVDIPDVNIEGPDAKLKGPKFKMPEINIKAPKISIPDVDLDLKG
PKVKGDFDVSVPKVEGTLKGPEVDLKGPRLDFEGPDAKLSGPSLKMPSLEISAPKVTAPD
VDLHLKAPKIGFSGPKLEGGEVDLKGPKVEAPSLDVHMDSPDINIEGPDVKIPKFKKPKF
GFGAKSPKADIKSPSLDVTVPEAELNLETPEISVGGKGKKSKFKMPKIHMSGPKIKAKKQ

GFDLNVPGGEIDASLKAPDVDVNIAGPDAALKVDVKSPKTKKTMFGKMYFPDVEFDIKSP
KFKAEAPLPSPKLEGELQAPDLELSLPAIHVEGLDIKAKAPKVKMPDVDISVPKIEGDLK
GPKVQANLGAPDINIEGLDAKVKTPSFGISAPQVSIPDVNVNLKGPKIKGDVPSVGLEGP
DVDLQGPEAKIKFPKFSMPKIGIPGVKMEGGGAEVHAQLPSLEGDLRGPDVKLEGPDVSL
KGPGVDLPSVNLSMPKVSGPDLDLNLKGPSLKGDLDASVPSMKVHAPGLNLSGVGGKMQV
GGDGVKVPGIDATTKLNVGAPDVTLRGPSLQGDLAVSGDIKCPKVSVGAPDLSLEASEGS
IKLPKMKLPQFGISTPGSDLHVNAKGPQVSGELKGPGVDVNLKGPRISAPNVDFNLEGPK
VKGSLGATGEIKGPTVGGGLPGIGVQGLEGNLQMPGIKSSGCDVNLPGVNVKLPTGQISG
PEIKGGLKGSEVGFHGAAPDISVKGPAFNMASPESDFGINLKGPKIKGGADVSGGVSAPD
ISLGEGHLSVKGSGGEWKGPQVSSALNLDTSKFAGGLHFSGPKVEGGVKGGQIGLQAPGL
SVSGPQGHLESGSGKVTFPKMKIPKFTFSGRELVGREMGVDVHFPKAEASIQAGAGDGEW
EESEVKLKKSKIKMPKFNFSKPKGKGGVTGSPEASISGSKGDLKSSKASLGSLEGEAEAE
ASSPKGKFSLFKSKKPRHRSNSFSDEREFSGPSTPTGTLEFEGGEVSLEGGKVKGKHGKL
KFGTFGGLGSKSKGHYEVTGSDDETGKLQGSGVSLASKKSRLSSSSSNDSGNKVGIQLPE
VELSVSTKKE
>sp|Q14204|DYHC1_HUMAN Cytoplasmic dynein 1 heavy chain 1 OS=Homo sapiens OX=9606
GN=DYNC1H1 PE=1 SV=5
MSEPGGGGGEDGSAGLEVSAVQNVADVSVLQKHLRKLVPLLLEDGGEAPAALEAALEEKS
ALEQMRKFLSDPQVHTVLVERSTLKEDVGDEGEEEKEFISYNINIDIHYGVKSNSLAFIK
RTPVIDADKPVSSQLRVLTLSEDSPYETLHSFISNAVAPFFKSYIRESGKADRDGDKMAP
SVEKKIAELEMGLLHLQQNIEIPEISLPIHPMITNVAKQCYERGEKPKVTDFGDKVEDPT
FLNQLQSGVNRWIREIQKVTKLDRDPASGTALQEISFWLNLERALYRIQEKRESPEVLLT
LDILKHGKRFHATVSFDTDTGLKQALETVNDYNPLMKDFPLNDLLSATELDKIRQALVAI
FTHLRKIRNTKYPIQRALRLVEAISRDLSSQLLKVLGTRKLMHVAYEEFEKVMVACFEVF
QTWDDEYEKLQVLLRDIVKRKREENLKMVWRINPAHRKLQARLDQMRKFRRQHEQLRAVI
VRVLRPQVTAVAQQNQGEVPEPQDMKVAEVLFDAADANAIEEVNLAYENVKEVDGLDVSK
EGTEAWEAAMKRYDERIDRVETRITARLRDQLGTAKNANEMFRIFSRFNALFVRPHIRGA
IREYQTQLIQRVKDDIESLHDKFKVQYPQSQACKMSHVRDLPPVSGSIIWAKQIDRQLTA
YMKRVEDVLGKGWENHVEGQKLKQDGDSFRMKLNTQEIFDDWARKVQQRNLGVSGRIFTI
ESTRVRGRTGNVLKLKVNFLPEIITLSKEVRNLKWLGFRVPLAIVNKAHQANQLYPFAIS
LIESVRTYERTCEKVEERNTISLLVAGLKKEVQALIAEGIALVWESYKLDPYVQRLAETV
FNFQEKVDDLLIIEEKIDLEVRSLETCMYDHKTFSEILNRVQKAVDDLNLHSYSNLPIWV
NKLDMEIERILGVRLQAGLRAWTQVLLGQAEDKAEVDMDTDAPQVSHKPGGEPKIKNVVH
ELRITNQVIYLNPPIEECRYKLYQEMFAWKMVVLSLPRIQSQRYQVGVHYELTEEEKFYR
NALTRMPDGPVALEESYSAVMGIVSEVEQYVKVWLQYQCLWDMQAENIYNRLGEDLNKWQ
ALLVQIRKARGTFDNAETKKEFGPVVIDYGKVQSKVNLKYDSWHKEVLSKFGQMLGSNMT
EFHSQISKSRQELEQHSVDTASTSDAVTFITYVQSLKRKIKQFEKQVELYRNGQRLLEKQ
RFQFPPSWLYIDNIEGEWGAFNDIMRRKDSAIQQQVANLQMKIVQEDRAVESRTTDLLTD
WEKTKPVTGNLRPEEALQALTIYEGKFGRLKDDREKCAKAKEALELTDTGLLSGSEERVQ
VALEELQDLKGVWSELSKVWEQIDQMKEQPWVSVQPRKLRQNLDALLNQLKSFPARLRQY
ASYEFVQRLLKGYMKINMLVIELKSEALKDRHWKQLMKRLHVNWVVSELTLGQIWDVDLQ
KNEAIVKDVLLVAQGEMALEEFLKQIREVWNTYELDLVNYQNKCRLIRGWDDLFNKVKEH
INSVSAMKLSPYYKVFEEDALSWEDKLNRIMALFDVWIDVQRRWVYLEGIFTGSADIKHL
LPVETQRFQSISTEFLALMKKVSKSPLVMDVLNIQGVQRSLERLADLLGKIQKALGEYLE

RERSSFPRFYFVGDEDLLEIIGNSKNVAKLQKHFKKMFAGVSSIILNEDNSVVLGISSRE
GEEVMFKTPVSITEHPKINEWLTLVEKEMRVTLAKLLAESVTEVEIFGKATSIDPNTYIT
WIDKYQAQLVVLSAQIAWSENVETALSSMGGGGDAAPLHSVLSNVEVTLNVLADSVLMEQ
PPLRRRKLEHLITELVHQRDVTRSLIKSKIDNAKSFEWLSQMRFYFDPKQTDVLQQLSIQ
MANAKFNYGFEYLGVQDKLVQTPLTDRCYLTMTQALEARLGGSPFGPAGTGKTESVKALG
HQLGRFVLVFNCDETFDFQAMGRIFVGLCQVGAWGCFDEFNRLEERMLSAVSQQVQCIQE
ALREHSNPNYDKTSAPITCELLNKQVKVSPDMAIFITMNPGYAGRSNLPDNLKKLFRSLA
MTKPDRQLIAQVMLYSQGFRTAEVLANKIVPFFKLCDEQLSSQSHYDFGLRALKSVLVSA
GNVKRERIQKIKREKEERGEAVDEGEIAENLPEQEILIQSVCETMVPKLVAEDIPLLFSL
LSDVFPGVQYHRGEMTALREELKKVCQEMYLTYGDGEEVGGMWVEKVLQLYQITQINHGL
MMVGPSGSGKSMAWRVLLKALERLEGVEGVAHIIDPKAISKDHLYGTLDPNTREWTDGLF
THVLRKIIDSVRGELQKRQWIVFDGDVDPEWVENLNSVLDDNKLLTLPNGERLSLPPNVR
IMFEVQDLKYATLATVSRCGMVWFSEDVLSTDMIFNNFLARLRSIPLDEGEDEAQRRRKG
KEDEGEEAASPMLQIQRDAATIMQPYFTSNGLVTKALEHAFQLEHIMDLTRLRCLGSLFS
MLHQACRNVAQYNANHPDFPMQIEQLERYIQRYLVYAILWSLSGDSRLKMRAELGEYIRR
ITTVPLPTAPNIPIIDYEVSISGEWSPWQAKVPQIEVETHKVAAPDVVVPTLDTVRHEAL
LYTWLAEHKPLVLCGPPGSGKTMTLFSALRALPDMEVVGLNFSSATTPELLLKTFDHYCE
YRRTPNGVVLAPVQLGKWLVLFCDEINLPDMDKYGTQRVISFIRQMVEHGGFYRTSDQTW
VKLERIQFVGACNPPTDPGRKPLSHRFLRHVPVVYVDYPGPASLTQIYGTFNRAMLRLIP
SLRTYAEPLTAAMVEFYTMSQERFTQDTQPHYIYSPREMTRWVRGIFEALRPLETLPVEG
LIRIWAHEALRLFQDRLVEDEERRWTDENIDTVALKHFPNIDREKAMSRPILYSNWLSKD
YIPVDQEELRDYVKARLKVFYEEELDVPLVLFNEVLDHVLRIDRIFRQPQGHLLLIGVSG
AGKTTLSRFVAWMNGLSVYQIKVHRKYTGEDFDEDLRTVLRRSGCKNEKIAFIMDESNVL
DSGFLERMNTLLANGEVPGLFEGDEYATLMTQCKEGAQKEGLMLDSHEELYKWFTSQVIR
NLHVVFTMNPSSEGLKDRAATSPALFNRCVLNWFGDWSTEALYQVGKEFTSKMDLEKPNY
IVPDYMPVVYDKLPQPPSHREAIVNSCVFVHQTLHQANARLAKRGGRTMAITPRHYLDFI
NHYANLFHEKRSELEEQQMHLNVGLRKIKETVDQVEELRRDLRIKSQELEVKNAAANDKL
KKMVKDQQEAEKKKVMSQEIQEQLHKQQEVIADKQMSVKEDLDKVEPAVIEAQNAVKSIK
KQHLVEVRSMANPPAAVKLALESICLLLGESTTDWKQIRSIIMRENFIPTIVNFSAEEIS
DAIREKMKKNYMSNPSYNYEIVNRASLACGPMVKWAIAQLNYADMLKRVEPLRNELQKLE
DDAKDNQQKANEVEQMIRDLEASIARYKEEYAVLISEAQAIKADLAAVEAKVNRSTALLK
SLSAERERWEKTSETFKNQMSTIAGDCLLSAAFIAYAGYFDQQMRQNLFTTWSHHLQQAN
IQFRTDIARTEYLSNADERLRWQASSLPADDLCTENAIMLKRFNRYPLIIDPSGQATEFI
MNEYKDRKITRTSFLDDAFRKNLESALRFGNPLLVQDVESYDPVLNPVLNREVRRTGGRV
LITLGDQDIDLSPSFVIFLSTRDPTVEFPPDLCSRVTFVNFTVTRSSLQSQCLNEVLKAE
RPDVDEKRSDLLKLQGEFQLRLRQLEKSLLQALNEVKGRILDDDTIITTLENLKREAAEV
TRKVEETDIVMQEVETVSQQYLPLSTACSSIYFTMESLKQIHFLYQYSLQFFLDIYHNVL
YENPNLKGVTDHTQRLSIITKDLFQVAFNRVARGMLHQDHITFAMLLARIKLKGTVGEPT
YDAEFQHFLRGNEIVLSAGSTPRIQGLTVEQAEAVVRLSCLPAFKDLIAKVQADEQFGIW
LDSSSPEQTVPYLWSEETPATPIGQAIHRLLLIQAFRPDRLLAMAHMFVSTNLGESFMSI
MEQPLDLTHIVGTEVKPNTPVLMCSVPGYDASGHVEDLAAEQNTQITSIAIGSAEGFNQA
DKAINTAVKSGRWVMLKNVHLAPGWLMQLEKKLHSLQPHACFRLFLTMEINPKVPVNLLR
AGRIFVFEPPPGVKANMLRTFSSIPVSRICKSPNERARLYFLLAWFHAIIQERLRYAPLG
WSKKYEFGESDLRSACDTVDTWLDDTAKGRQNISPDKIPWSALKTLMAQSIYGGRVDNEF

DQRLLNTFLERLFTTRSFDSEFKLACKVDGHKDIQMPDGIRREEFVQWVELLPDTQTPSW
LGLPNNAERVLLTTQGVDMISKMLKMQMLEDEDDLAYAETEKKTRTDSTSDGRPAWMRTL
HTTASNWLHLIPQTLSHLKRTVENIKDPLFRFFEREVKMGAKLLQDVRQDLADVVQVCEG
KKKQTNYLRTLINELVKGILPRSWSHYTVPAGMTVIQWVSDFSERIKQLQNISLAAASGG
AKELKNIHVCLGGLFVPEAYITATRQYVAQANSWSLEELCLEVNVTTSQGATLDACSFGV
TGLKLQGATCNNNKLSLSNAISTALPLTQLRWVKQTNTEKKASVVTLPVYLNFTRADLIF
TVDFEIATKEDPRSFYERGVAVLCTE
>sp|Q14315|FLNC_HUMAN Filamin-C OS=Homo sapiens OX=9606 GN=FLNC PE=1 SV=3
MMNNSGYSDAGLGLGDETDEMPSTEKDLAEDAPWKKIQQNTFTRWCNEHLKCVGKRLTDL
QRDLSDGLRLIALLEVLSQKRMYRKFHPRPNFRQMKLENVSVALEFLEREHIKLVSIDSK
AIVDGNLKLILGLIWTLILHYSISMPMWEDEDDEDARKQTPKQRLLGWIQNKVPQLPITN
FNRDWQDGKALGALVDNCAPGLCPDWEAWDPNQPVENAREAMQQADDWLGVPQVIAPEEI
VDPNVDEHSVMTYLSQFPKAKLKPGAPVRSKQLNPKKAIAYGPGIEPQGNTVLQPAHFTV
QTVDAGVGEVLVYIEDPEGHTEEAKVVPNNDKDRTYAVSYVPKVAGLHKVTVLFAGQNIE
RSPFEVNVGMALGDANKVSARGPGLEPVGNVANKPTYFDIYTAGAGTGDVAVVIVDPQGR
RDTVEVALEDKGDSTFRCTYRPAMEGPHTVHVAFAGAPITRSPFPVHVSEACNPNACRAS
GRGLQPKGVRVKEVADFKVFTKGAGSGELKVTVKGPKGTEEPVKVREAGDGVFECEYYPV
VPGKYVVTITWGGYAIPRSPFEVQVSPEAGVQKVRAWGPGLETGQVGKSADFVVEAIGTE
VGTLGFSIEGPSQAKIECDDKGDGSCDVRYWPTEPGEYAVHVICDDEDIRDSPFIAHILP
APPDCFPDKVKAFGPGLEPTGCIVDKPAEFTIDARAAGKGDLKLYAQDADGCPIDIKVIP
NGDGTFRCSYVPTKPIKHTIIISWGGVNVPKSPFRVNVGEGSHPERVKVYGPGVEKTGLK
ANEPTYFTVDCSEAGQGDVSIGIKCAPGVVGPAEADIDFDIIKNDNDTFTVKYTPPGAGR
YTIMVLFANQEIPASPFHIKVDPSHDASKVKAEGPGLNRTGVEVGKPTHFTVLTKGAGKA
KLDVQFAGTAKGEVVRDFEIIDNHDYSYTVKYTAVQQGNMAVTVTYGGDPVPKSPFVVNV
APPLDLSKIKVQGLNSKVAVGQEQAFSVNTRGAGGQGQLDVRMTSPSRRPIPCKLEPGGG
AEAQAVRYMPPEEGPYKVDITYDGHPVPGSPFAVEGVLPPDPSKVCAYGPGLKGGLVGTP
APFSIDTKGAGTGGLGLTVEGPCEAKIECQDNGDGSCAVSYLPTEPGEYTINILFAEAHI
PGSPFKATIRPVFDPSKVRASGPGLERGKVGEAATFTVDCSEAGEAELTIEILSDAGVKA
EVLIHNNADGTYHITYSPAFPGTYTITIKYGGHPVPKFPTRVHVQPAVDTSGVKVSGPGV
EPHGVLREVTTEFTVDARSLTATGGNHVTARVLNPSGAKTDTYVTDNGDGTYRVQYTAYE
EGVHLVEVLYDEVAVPKSPFRVGVTEGCDPTRVRAFGPGLEGGLVNKANRFTVETRGAGT
GGLGLAIEGPSEAKMSCKDNKDGSCTVEYIPFTPGDYDVNITFGGRPIPGSPFRVPVKDV
VDPGKVKCSGPGLGAGVRARVPQTFTVDCSQAGRAPLQVAVLGPTGVAEPVEVRDNGDGT
HTVHYTPATDGPYTVAVKYADQEVPRSPFKIKVLPAHDASKVRASGPGLNASGIPASLPV
EFTIDARDAGEGLLTVQILDPEGKPKKANIRDNGDGTYTVSYLPDMSGRYTITIKYGGDE
IPYSPFRIHALPTGDASKCLVTVSIGGHGLGACLGPRIQIGQETVITVDAKAAGEGKVTC
TVSTPDGAELDVDVVENHDGTFDIYYTAPEPGKYVITIRFGGEHIPNSPFHVLACDPLPH
EEEPSEVPQLRQPYAPPRPGARPTHWATEEPVVPVEPMESMLRPFNLVIPFAVQKGELTG
EVRMPSGKTARPNITDNKDGTITVRYAPTEKGLHQMGIKYDGNHIPGSPLQFYVDAINSR
HVSAYGPGLSHGMVNKPATFTIVTKDAGEGGLSLAVEGPSKAEITCKDNKDGTCTVSYLP
TAPGDYSIIVRFDDKHIPGSPFTAKITGDDSMRTSQLNVGTSTDVSLKITESDLSQLTAS
IRAPSGNEEPCLLKRLPNRHIGISFTPKEVGEHVVSVRKSGKHVTNSPFKILVGPSEIGD
ASKVRVWGKGLSEGHTFQVAEFIVDTRNAGYGGLGLSIEGPSKVDINCEDMEDGTCKVTY
CPTEPGTYIINIKFADKHVPGSPFTVKVTGEGRMKESITRRRQAPSIATIGSTCDLNLKI

PGNWFQMVSAQERLTRTFTRSSHTYTRTERTEISKTRGGETKREVRVEESTQVGGDPFPA
VFGDFLGRERLGSFGSITRQQEGEASSQDMTAQVTSPSGKVEAAEIVEGEDSAYSVRFVP
QEMGPHTVAVKYRGQHVPGSPFQFTVGPLGEGGAHKVRAGGTGLERGVAGVPAEFSIWTR
EAGAGGLSIAVEGPSKAEIAFEDRKDGSCGVSYVVQEPGDYEVSIKFNDEHIPDSPFVVP
VASLSDDARRLTVTSLQETGLKVNQPASFAVQLNGARGVIDARVHTPSGAVEECYVSELD
SDKHTIRFIPHENGVHSIDVKFNGAHIPGSPFKIRVGEQSQAGDPGLVSAYGPGLEGGTT
GVSSEFIVNTLNAGSGALSVTIDGPSKVQLDCRECPEGHVVTYTPMAPGNYLIAIKYGGP
QHIVGSPFKAKVTGPRLSGGHSLHETSTVLVETVTKSSSSRGSSYSSIPKFSSDASKVVT
RGPGLSQAFVGQKNSFTVDCSKAGTNMMMVGVHGPKTPCEEVYVKHMGNRVYNVTYTVKE
KGDYILIVKWGDESVPGSPFKVKVP
>sp|Q16563|SYPL1_HUMAN Synaptophysin-like protein 1 OS=Homo sapiens OX=9606
GN=SYPL1 PE=1 SV=1
MAPNIYLVRQRISRLGQRMSGFQINLNPLKEPLGFIKVLEWIASIFAFATCGGFKGQTEI
QVNCPPAVTENKTVTATFGYPFRLNEASFQPPPGVNICDVNWKDYVLIGDYSSSAQFYVT
FAVFVFLYCIAALLLYVGYTSLYLDSRKLPMIDFVVTLVATFLWLVSTSAWAKALTDIKI
ATGHNIIDELPPCKKKAVLCYFGSVTSMGSLNVSVIFGFLNMILWGGNAWFVYKETSLHS
PSNTSAPHSQGGIPPPTGI
>tr|Q32Q12|Q32Q12_HUMAN Nucleoside diphosphate kinase OS=Homo sapiens OX=9606
GN=NME1-NME2 PE=1 SV=1
MVLLSTLGIVFQGEGPPISSCDTGTMANCERTFIAIKPDGVQRGLVGEIIKRFEQKGFRL
VGLKFMQASEDLLKEHYVDLKDRPFFAGLVKYMHSGPVVAMVWEGLNVVKTGRVMLGETN
PADSKPGTIRGDFCIQVGRTMANLERTFIAIKPDGVQRGLVGEIIKRFEQKGFRLVAMKF
LRASEEHLKQHYIDLKDRPFFPGLVKYMNSGPVVAMVWEGLNVVKTGRVMLGETNPADSK
PGTIRGDFCIQVGRNIIHGSDSVKSAEKEISLWFKPEELVDYKSCAHDWVYE
>sp|Q5T4S7|UBR4_HUMAN E3 ubiquitin-protein ligase UBR4 OS=Homo sapiens OX=9606
GN=UBR4 PE=1 SV=1
MATSGGEEAAAAAPAPGTPATGADTTPGWEVAVRPLLSASYSAFEMKELPQLVASVIESE
SEILHHEKQYEPFYSSFVALSTHYITTVCSLIPRNQLQSVAAACKVLIEFSLLRLENPDE
ACAVSQKHLILLIKGLCTGCSRLDRTEIITFTAMMKSAKLPQTVKTLSDVEDQKELASPV
SPELRQKEVQMNFLNQLTSVFNPRTVASQPISTQTLVEGENDEQSSTDQASAIKTKNVFI
AQNVASLQELGGSEKLLRVCLNLPYFLRYINRFQDAVLANSFFIMPATVADATAVRNGFH
SLVIDVTMALDTLSLPVLEPLNPSRLQDVTVLSLSCLYAGVSVATCMAILHVGSAQQVRT
GSTSSKEDDYESDAATIVQKCLEIYDMIGQAISSSRRAGGEHYQNFQLLGAWCLLNSLFL
ILNLSPTALADKGKEKDPLAALRVRDILSRTKEGVGSPKLGPGKGHQGFGVLSVILANHA
IKLLTSLFQDLQVEALHKGWETDGPPAALSIMAQSTSIQRIQRLIDSVPLMNLLLTLLST
SYRKACVLQRQRKGSMSSDASASTDSNTYYEDDFSSTEEDSSQDDDSEPILGQWFEETIS
PSKEKAAPPPPPPPPPLESSPRVKSPSKQAPGEKGNILASRKDPELFLGLASNILNFITS
SMLNSRNNFIRNYLSVSLSEHHMATLASIIKEVDKDGLKGSSDEEFAAALYHFNHSLVTS
DLQSPNLQNTLLQQLGVAPFSEGPWPLYIHPQSLSVLSRLLLIWQHKASAQGDPDVPECL
KVWDRFLSTMKQNALQGVVPSETEDLNVEHLQMLLLIFHNFTETGRRAILSLFVQIIQEL
SVNMDAQMRFVPLILARLLLIFDYLLHQYSKAPVYLFEQVQHNLLSPPFGWASGSQDSNS
RRATTPLYHGFKEVEENWSKHFSSDAVPHPRFYCVLSPEASEDDLNRLDSVACDVLFSKL
VKYDELYAALTALLAAGSQLDTVRRKENKNVTALEACALQYYFLILWRILGILPPSKTYI
NQLSMNSPEMSECDILHTLRWSSRLRISSYVNWIKDHLIKQGMKAEHASSLLELASTTKC

SSVKYDVEIVEEYFARQISSFCSIDCTTILQLHEIPSLQSIYTLDAAISKVQVSLDEHFS
KMAAETDPHKSSEITKNLLPATLQLIDTYASFTRAYLLQNFNEEGTTEKPSKEKLQGFAA
VLAIGSSRCKANTLGPTLVQNLPSSVQTVCESWNNINTNEFPNIGSWRNAFANDTIPSES
YISAVQAAHLGTLCSQSLPLAASLKHTLLSLVRLTGDLIVWSDEMNPPQVIRTLLPLLLE
SSTESVAEISSNSLERILGPAESDEFLARVYEKLITGCYNILANHADPNSGLDESILEEC
LQYLEKQLESSQARKAMEEFFSDSGELVQIMMATANENLSAKFCNRVLKFFTKLFQLTEK
SPNPSLLHLCGSLAQLACVEPVRLQAWLTRMTTSPPKDSDQLDVIQENRQLLQLLTTYIV
RENSQVGEGVCAVLLGTLTPMATEMLANGDGTGFPELMVVMATLASAGQGAGHLQLHNAA
VDWLSRCKKYLSQKNVVEKLNANVMHGKHVMILECTCHIMSYLADVTNALSQSNGQGPSH
LSVDGEERAIEVDSDWVEELAVEEEDSQAEDSDEDSLCNKLCTFTITQKEFMNQHWYHCH
TCKMVDGVGVCTVCAKVCHKDHEISYAKYGSFFCDCGAKEDGSCLALVKRTPSSGMSSTM
KESAFQSEPRISESLVRHASTSSPADKAKVTISDGKVADEEKPKKSSLCRTVEGCREELQ
NQANFSFAPLVLDMLNFLMDAIQTNFQQASAVGSSSRAQQALSELHTVEKAVEMTDQLMV
PTLGSQEGAFENVRMNYSGDQGQTIRQLISAHVLRRVAMCVLSSPHGRRQHLAVSHEKGK
ITVLQLSALLKQADSSKRKLTLTRLASAPVPFTVLSLTGNPCKEDYLAVCGLKDCHVLTF
SSSGSVSDHLVLHPQLATGNFIIKAVWLPGSQTELAIVTADFVKIYDLCVDALSPTFYFL
LPSSKIRDVTFLFNEEGKNIIVIMSSAGYIYTQLMEEASSAQQGPFYVTNVLEINHEDLK
DSNSQVAGGGVSVYYSHVLQMLFFSYCQGKSFAATISRTTLEVLQLFPINIKSSNGGSKT
SPALCQWSEVMNHPGLVCCVQQTTGVPLVVMVKPDTFLIQEIKTLPAKAKIQDMVAIRHT
ACNEQQRTTMILLCEDGSLRIYMANVENTSYWLQPSLQPSSVISIMKPVRKRKTATITTR
TSSQVTFPIDFFEHNQQLTDVEFGGNDLLQVYNAQQIKHRLNSTGMYVANTKPGGFTIEI
SNNNSTMVMTGMRIQIGTQAIERAPSYIEIFGRTMQLNLSRSRWFDFPFTREEALQADKK
LNLFIGASVDPAGVTMIDAVKIYGKTKEQFGWPDEPPEEFPSASVSNICPSNLNQSNGTG
DSDSAAPTTTSGTVLERLVVSSLEALESCFAVGPIIEKERNKNAAQELATLLLSLPAPAS
VQQQSKSLLASLHTSRSAYHSHKDQALLSKAVQCLNTSSKEGKDLDPEVFQRLVITARSI
AIMRPNNLVHFTESKLPQMETEGMDEGKEPQKQLEGDCCSFITQLVNHFWKLHASKPKNA
FLAPACLPGLTHIEATVNALVDIIHGYCTCELDCINTASKIYMQMLLCPDPAVSFSCKQA
LIRVLRPRNKRRHVTLPSSPRSNTPMGDKDDDDDDDADEKMQSSGIPNGGHIRQESQEQS
EVDHGDFEMVSESMVLETAENVNNGNPSPLEALLAGAEGFPPMLDIPPDADDETMVELAI
ALSLQQDQQGSSSSALGLQSLGLSGQAPSSSSLDAGTLSDTTASAPASDDEGSTAATDGS
TLRTSPADHGGSVGSESGGSAVDSVAGEHSVSGRSSAYGDATAEGHPAGPGSVSSSTGAI
STTTGHQEGDGSEGEGEGETEGDVHTSNRLHMVRLMLLERLLQTLPQLRNVGGVRAIPYM
QVILMLTTDLDGEDEKDKGALDNLLSQLIAELGMDKKDVSKKNERSALNEVHLVVMRLLS
VFMSRTKSGSKSSICESSSLISSATAAALLSSGAVDYCLHVLKSLLEYWKSQQNDEEPVA
TSQLLKPHTTSSPPDMSPFFLRQYVKGHAADVFEAYTQLLTEMVLRLPYQIKKITDTNSR
IPPPVFDHSWFYFLSEYLMIQQTPFVRRQVRKLLLFICGSKEKYRQLRDLHTLDSHVRGI
KKLLEEQGIFLRASVVTASSGSALQYDTLISLMEHLKACAEIAAQRTINWQKFCIKDDSV
LYFLLQVSFLVDEGVSPVLLQLLSCALCGSKVLAALAASSGSSSASSSSAPVAASSGQAT
TQSKSSTKKSKKEEKEKEKDGETSGSQEDQLCTALVNQLNKFADKETLIQFLRCFLLESN
SSSVRWQAHCLTLHIYRNSSKSQQELLLDLMWSIWPELPAYGRKAAQFVDLLGYFSLKTP
QTEKKLKEYSQKAVEILRTQNHILTNHPNSNIYNTLSGLVEFDGYYLESDPCLVCNNPEV
PFCYIKLSSIKVDTRYTTTQQVVKLIGSHTISKVTVKIGDLKRTKMVRTINLYYNNRTVQ
AIVELKNKPARWHKAKKVQLTPGQTEVKIDLPLPIVASNLMIEFADFYENYQASTETLQC
PRCSASVPANPGVCGNCGENVYQCHKCRSINYDEKDPFLCNACGFCKYARFDFMLYAKPC

CAVDPIENEEDRKKAVSNINTLLDKADRVYHQLMGHRPQLENLLCKVNEAAPEKPQDDSG
TAGGISSTSASVNRYILQLAQEYCGDCKNSFDELSKIIQKVFASRKELLEYDLQQREAAT
KSSRTSVQPTFTASQYRALSVLGCGHTSSTKCYGCASAVTEHCITLLRALATNPALRHIL
VSQGLIRELFDYNLRRGAAAMREEVRQLMCLLTRDNPEATQQMNDLIIGKVSTALKGHWA
NPDLASSLQYEMLLLTDSISKEDSCWELRLRCALSLFLMAVNIKTPVVVENITLMCLRIL
QKLIKPPAPTSKKNKDVPVEALTTVKPYCNEIHAQAQLWLKRDPKASYDAWKKCLPIRGI
DGNGKAPSKSELRHLYLTEKYVWRWKQFLSRRGKRTSPLDLKLGHNNWLRQVLFTPATQA
ARQAACTIVEALATIPSRKQQVLDLLTSYLDELSIAGECAAEYLALYQKLITSAHWKVYL
AARGVLPYVGNLITKEIARLLALEEATLSTDLQQGYALKSLTGLLSSFVEVESIKRHFKS
RLVGTVLNGYLCLRKLVVQRTKLIDETQDMLLEMLEDMTTGTESETKAFMAVCIETAKRY
NLDDYRTPVFIFERLCSIIYPEENEVTEFFVTLEKDPQQEDFLQGRMPGNPYSSNEPGIG
PLMRDIKNKICQDCDLVALLEDDSGMELLVNNKIISLDLPVAEVYKKVWCTTNEGEPMRI
VYRMRGLLGDATEEFIESLDSTTDEEEDEEEVYKMAGVMAQCGGLECMLNRLAGIRDFKQ
GRHLLTVLLKLFSYCVKVKVNRQQLVKLEMNTLNVMLGTLNLALVAEQESKDSGGAAVAE
QVLSIMEIILDESNAEPLSEDKGNLLLTGDKDQLVMLLDQINSTFVRSNPSVLQGLLRII
PYLSFGEVEKMQILVERFKPYCNFDKYDEDHSGDDKVFLDCFCKIAAGIKNNSNGHQLKD
LILQKGITQNALDYMKKHIPSAKNLDADIWKKFLSRPALPFILRLLRGLAIQHPGTQVLI
GTDSIPNLHKLEQVSSDEGIGTLAENLLEALREHPDVNKKIDAARRETRAEKKRMAMAMR
QKALGTLGMTTNEKGQVVTKTALLKQMEELIEEPGLTCCICREGYKFQPTKVLGIYTFTK
RVALEEMENKPRKQQGYSTVSHFNIVHYDCHLAAVRLARGREEWESAALQNANTKCNGLL
PVWGPHVPESAFATCLARHNTYLQECTGQREPTYQLNIHDIKLLFLRFAMEQSFSADTGG
GGRESNIHLIPYIIHTVLYVLNTTRATSREEKNLQGFLEQPKEKWVESAFEVDGPYYFTV
LALHILPPEQWRATRVEILRRLLVTSQARAVAPGGATRLTDKAVKDYSAYRSSLLFWALV
DLIYNMFKKVPTSNTEGGWSCSLAEYIRHNDMPIYEAADKALKTFQEEFMPVETFSEFLD
VAGLLSEITDPESFLKDLLNSVP
>sp|Q8N126|CADM3_HUMAN Cell adhesion molecule 3 OS=Homo sapiens OX=9606 GN=CADM3 PE=1 SV=1
MGAPAASLLLLLLLFACCWAPGGANLSQDDSQPWTSDETVVAGGTVVLKCQVKDHEDSSL
QWSNPAQQTLYFGEKRALRDNRIQLVTSTPHELSISISNVALADEGEYTCSIFTMPVRTA
KSLVTVLGIPQKPIITGYKSSLREKDTATLNCQSSGSKPAARLTWRKGDQELHGEPTRIQ
EDPNGKTFTVSSSVTFQVTREDDGASIVCSVNHESLKGADRSTSQRIEVLYTPTAMIRPD
PPHPREGQKLLLHCEGRGNPVPQQYLWEKEGSVPPLKMTQESALIFPFLNKSDSGTYGCT
ATSNMGSYKAYYTLNVNDPSPVPSSSSTYHAIIGGIVAFIVFLLLIMLIFLGHYLIRHKG
TYLTHEAKGSDDAPDADTAIINAEGGQSGGDDKKEYFI
>sp|Q93088|BHMT1_HUMAN Betaine--homocysteine S-methyltransferase 1 OS=Homo sapiens OX=9606 GN=BHMT PE=1 SV=2
MPPVGGKKAKKGILERLNAGEIVIGDGGFVFALEKRGYVKAGPWTPEAAVEHPEAVRQLH
REFLRAGSNVMQTFTFYASEDKLENRGNYVLEKISGQEVNEAACDIARQVADEGDALVAG
GVSQTPSYLSCKSETEVKKVFLQQLEVFMKKNVDFLIAEYFEHVEEAVWAVETLIASGKP
VAATMCIGPEGDLHGVPPGECAVRLVKAGASIIGVNCHFDPTISLKTVKLMKEGLEAARL
KAHLMSQPLAYHTPDCNKQGFIDLPEFPFGLEPRVATRWDIQKYAREAYNLGVRYIGGCC
GFEPYHIRAIAEELAPERGFLPPASEKHGSWGSGLDMHTKPWVRARARKEYWENLRIASG
RPYNPSMSKPDGWGVTKGTAELMQQKEATTEQQLKELFEKQKFKSQ

>sp|Q9BRJ7|TIRR_HUMAN Tudor-interacting repair regulator protein OS=Homo sapiens OX=9606 GN=NUDT16L1 PE=1 SV=1
MSTAAVPELKQISRVEAMRLGPGWSHSCHAMLYAANPGQLFGRIPMRFSVLMQMRFDGLL
GFPGGFVDRRFWSLEDGLNRVLGLGLGCLRLTEADYLSSHLTEGPHRVVAHLYARQLTLE
QLHAVEISAVHSRDHGLEVLGLVRVPLYTQKDRVGGFPNFLSNAFVSTAKCQLLFALKVL
NMMPEEKLVEALAAATEKQKKALEKLLPASS
>sp|Q9NQE9|HINT3_HUMAN Adenosine 5'-monophosphoramidase HINT3 OS=Homo sapiens OX=9606 GN=HINT3 PE=1 SV=1
MAEEQVNRSAGLAPDCEASATAETTVSSVGTCEAAGKSPEPKDYDSTCVFCRIAGRQDPG
TELLHCENEDLICFKDIKPAATHHYLVVPKKHIGNCRTLRKDQVELVENMVTVGKTILER
NNFTDFTNVRMGFHMPPFCSISHLHLHVLAPVDQLGFLSKLVYRVNSYWFITADHLIEKL
RT
>sp|Q9P0S9|TM14C_HUMAN Transmembrane protein 14C OS=Homo sapiens OX=9606 GN=TMEM14C PE=1 SV=1
MQDTGSVVPLHWFGFGYAALVASGGIIGYVKAGSVPSLAAGLLFGSLAGLGAYQLSQDPR
NVWVFLATSGTLAGIMGMRFYHSGKFMPAGLIAGASLLMVAKVGVSMFNRPH
>sp|Q9UJ70|NAGK_HUMAN N-acetyl-D-glucosamine kinase OS=Homo sapiens OX=9606 GN=NAGK PE=1 SV=4
MAAIYGGVEGGGTRSEVLLVSEDGKILAEADGLSTNHWLIGTDKCVERINEMVNRAKRKA
GVDPLVPLRSLGLSLSGGDQEDAGRILIEELRDRFPYLSESYLITTDAAGSIATATPDGG
VVLISGTGSNCRLINPDGSESGCGGWGHMMGDEGSAYWIAHQAVKIVFDSIDNLEAAPHD
IGYVKQAMFHYFQVPDRLGILTHLYRDFDKCRFAGFCRKIAEGAQQGDPLSRYIFRKAGE
MLGRHIVAVLPEIDPVLFQGKIGLPILCVGSVWKSWELLKEGFLLALTQGREIQAQNFFS
SFTLMKLRHSSALGGASLGARHIGHLLPMDYSANAIAFYSYTFS
>sp|Q9Y2H2|SAC2_HUMAN Phosphatidylinositide phosphatase SAC2 OS=Homo sapiens OX=9606 GN=INPP5F PE=1 SV=3
MELFQAKDHYILQQGERALWCSRRDGGLQLRPATDLLLAWNPICLGLVEGVIGKIQLHSD
LPWWLILIRQKALVGKLPGDHEVCKVTKIAVLSLSEMEPQDLELELCKKHHFGINKPEKI
IPSPDDSKFLLKTFTHIKSNVSAPNKKKVKESKEKEKLERRLLEELLKMFMDSESFYYSL
TYDLTNSVQRQSTGERDGRPLWQKVDDRFFWNKYMIQDLTEIGTPDVDFWIIPMIQGFVQ
IEELVVNYTESSDDEKSSPETPPQESTCVDDIHPRFLVALISRRSRHRAGMRYKRRGVDK
NGNVANYVETEQLIHVHNHTLSFVQTRGSVPVFWSQVGYRYNPRPRLDRSEKETVAYFCA
HFEEQLNIYKKQVIINLVDQAGREKIIGDAYLKQVLLFNNSHLTYVSFDFHEHCRGMKFE
NVQTLTDAIYDIILDMKWCWVDEAGVICKQEGIFRVNCMDCLDRTNVVQAAIARVVMEQQ
LKKLGVMPPEQPLPVKCNRIYQIMWANNGDSISRQYAGTAALKGDFTRTGERKLAGVMKD
GVNSANRYYLNRFKDAYRQAVIDLMQGIPVTEDLYSIFTKEKEHEALHKENQRSHQELIS
QLLQSYMKLLLPDDEKFHGGWALIDCDPSLIDATHRDVDVLLLLSNSAYYVAYYDDEVDK
VNQYQRLSLENLEKIEIGPEPTLFGKPKFSCMRLHYRYKEASGYFHTLRAVMRNPEEDGK
DTLQCIAEMLQITKQAMGSDLPIIEKKLERKSSKPHEDIIGIRSQNQGSLAQGKNFLMSK
FSSLNQKVKQTKSNVNIGNLRKLGNFTKPEMKVNFLKPNLKVNLWKSDSSLETMENTGVM
DKVQAESDGDMSSDNDSYHSDEFLTNSKSDEDRQLANSLESVGPIDYVLPSCGIIASAPR
LGSRSQSLSSTDSSVHAPSEITVAHGSGLGKGQESPLKKSPSAGDVHILTGFAKPMDIYC
HRFVQDAQNKVTHLSETRSVSQQASQERNQMTNQVSNETQSESTEQTPSRPSQLDVSLSA
TGPQFLSVEPAHSVASQKTPTSASSMLELETGLHVTPSPSESSSSRAVSPFAKIRSSMVQ

VASITQAGLTHGINFAVSKVQKSPPEPEIINQVQQNELKKMFIQCQTRIIQI
>tr|Q9Y509|Q9Y509_HUMAN VH3 protein (Fragment) OS=Homo sapiens OX=9606 GN=VH3 PE=2 SV=1
QVHLVESGGGVVQPGKSLRLSCEASGFTFSTYGMSWVRQAPGKGLDWVALISYDGSTQYY
AGSVKGRFTISRDNSKNTLYLQMTSLRVEDTAVYYCAKDGNYFDSVGYYYAGIDYWGQGT
LVTVSSASTKGPSVFPLAPSSKSTSGG
>sp|Q15772|SPEG_HUMAN Striated muscle preferentially expressed protein kinase OS=Homo sapiens OX=9606 GN=SPEG PE=1 SV=4
MQKARGTRGEDAGTRAPPSPGVPPKRAKVGAGGGAPVAVAGAPVFLRPLKNAAVCAGSDV
RLRVVVSGTPQPSLRWFRDGQLLPAPAPEPSCLWLRRCGAQDAGVYSCMAQNERGRASCE
AVLTVLEVGDSETAEDDISDVQGTQRLELRDDGAFSTPTGGSDTLVGTSLDTPPTSVTGT
SEEQVSWWGSGQTVLEQEAGSGGGTRRLPGSPRQAQATGAGPRHLGVEPLVRASRANLVG
ASWGSEDSLSVASDLYGSAFSLYRGRALSIHVSVPQSGLRREEPDLQPQLASEAPRRPAQ
PPPSKSALLPPPSPRVGKRSPPGPPAQPAATPTSPHRRTQEPVLPEDTTTEEKRGKKSKS
SGPSLAGTAESRPQTPLSEASGRLSALGRSPRLVRAGSRILDKLQFFEERRRSLERSDSP
PAPLRPWVPLRKARSLEQPKSERGAPWGTPGASQEELRAPGSVAERRRLFQQKAASLDER
TRQRSPASDLELRFAQELGRIRRSTSREELVRSHESLRATLQRAPSPREPGEPPLFSRPS
TPKTSRAVSPAAAQPPSPSSAEKPGDEPGRPRSRGPAGRTEPGEGPQQEVRRRDQFPLTR
SRAIQECRSPVPPPAADPPEARTKAPPGRKREPPAQAVRFLPWATPGLEGAAVPQTLEKN
RAGPEAEKRLRRGPEEDGPWGPWDRRGARSQGKGRRARPTSPELESSDDSYVSAGEEPLE
APVFEIPLQNVVVAPGADVLLKCIITANPPPQVSWHKDGSALRSEGRLLLRAEGERHTLL
LREARAADAGSYMATATNELGQATCAASLTVRPGGSTSPFSSPITSDEEYLSPPEEFPEP
GETWPRTPTMKPSPSQNRRSSDTGSKAPPTFKVSLMDQSVREGQDVIMSIRVQGEPKPVV
SWLRNRQPVRPDQRRFAEEAEGGLCRLRILAAERGDAGFYTCKAVNEYGARQCEARLEVR
AHPESRSLAVLAPLQDVDVGAGEMALFECLVAGPTDVEVDWLCRGRLLQPALLKCKMHFD
GRKCKLLLTSVHEDDSGVYTCKLSTAKDELTCSARLTVRPSLAPLFTRLLEDVEVLEGRA
ARFDCKISGTPPPVVTWTHFGCPMEESENLRLRQDGGLHSLHIAHVGSEDEGLYAVSAVN
THGQAHCSAQLYVEEPRTAASGPSSKLEKMPSIPEEPEQGELERLSIPDFLRPLQDLEVG
LAKEAMLECQVTGLPYPTISWFHNGHRIQSSDDRRMTQYRDVHRLVFPAVGPQHAGVYKS
VIANKLGKAACYAHLYVTDVVPGPPDGAPQVVAVTGRMVTLTWNPPRSLDMAIDPDSLTY
TVQHQVLGSDQWTALVTGLREPGWAATGLRKGVQHIFRVLSTTVKSSSKPSPPSEPVQLL
EHGPTLEEAPAMLDKPDIVYVVEGQPASVTVTFNHVEAQVVWRSCRGALLEARAGVYELS
QPDDDQYCLRICRVSRRDMGALTCTARNRHGTQTCSVTLELAEAPRFESIMEDVEVGAGE
TARFAVVVEGKPLPDIMWYKDEVLLTESSHVSFVYEENECSLVVLSTGAQDGGVYTCTAQ
NLAGEVSCKAELAVHSAQTAMEVEGVGEDEDHRGRRLSDFYDIHQEIGRGAFSYLRRIVE
RSSGLEFAAKFIPSQAKPKASARREARLLARLQHDCVLYFHEAFERRRGLVIVTELCTEE
LLERIARKPTVCESEIRAYMRQVLEGIHYLHQSHVLHLDVKPENLLVWDGAAGEQQVRIC
DFGNAQELTPGEPQYCQYGTPEFVAPEIVNQSPVSGVTDIWPVGVVAFLCLTGISPFVGE
NDRTTLMNIRNYNVAFEETTFLSLSREARGFLIKVLVQDRLRPTAEETLEHPWFKTQAKG
AEVSTDHLKLFLSRRRWQRSQISYKCHLVLRPIPELLRAPPERVWVTMPRRPPPSGGLSS
SSDSEEEELEELPSVPRPLQPEFSGSRVSLTDIPTEDEALGTPETGAATPMDWQEQGRAP
SQDQEAPSPEALPSPGQEPAAGASPRRGELRRGSSAESALPRAGPRELGRGLHKAASVEL
PQRRSPSPGATRLARGGLGEGEYAQRLQALRQRLLRGGPEDGKVSGLRGPLLESLGGRAR
DPRMARAASSEAAPHHQPPLENRGLQKSSSFSQGEAEPRGRHRRAGAPLEIPVARLGARR

LQESPSLSALSEAQPSSPARPSAPKPSTPKSAEPSATTPSDAPQPPAPQPAQDKAPEPRP
EPVRASKPAPPPQALQTLALPLTPYAQIIQSLQLSGHAQGPSQGPAAPPSEPKPHAAVFA
RVASPPPGAPEKRVPSAGGPPVLAEKARVPTVPPRPGSSLSSSIENLESEAVFEAKFKRS
RESPLSLGLRLLSRSRSEERGPFRGAEEEDGIYRPSPAGTPLELVRRPERSRSVQDLRAV
GEPGLVRRLSLSLSQRLRRTPPAQRHPAWEARGGDGESSEGGSSARGSPVLAMRRRLSFT
LERLSSRLQRSGSSEDSGGASGRSTPLFGRLRRATSEGESLRRLGLPHNQLAAQAGATTP
SAESLGSEASATSGSSAPGESRSRLRWGFSRPRKDKGLSPPNLSASVQEELGHQYVRSES
DFPPVFHIKLKDQVLLEGEAATLLCLPAACPAPHISWMKDKKSLRSEPSVIIVSCKDGRQ
LLSIPRAGKRHAGLYECSATNVLGSITSSCTVAVARVPGKLAPPEVPQTYQDTALVLWKP
GDSRAPCTYTLERRVDGESVWHPVSSGIPDCYYNVTHLPVGVTVRFRVACANRAGQGPFS
NSSEKVFVRGTQDSSAVPSAAHQEAPVTSRPARARPPDSPTSLAPPLAPAAPTPPSVTVS
PSSPPTPPSQALSSLKAVGPPPQTPPRRHRGLQAARPAEPTLPSTHVTPSEPKPFVLDTG
TPIPASTPQGVKPVSSSTPVYVVTSFVSAPPAPEPPAPEPPPEPTKVTVQSLSPAKEVVS
SPGSSPRSSPRPEGTTLRQGPPQKPYTFLEEKARGRFGVVRACRENATGRTFVAKIVPYA
AEGKRRVLQEYEVLRTLHHERIMSLHEAYITPRYLVLIAESCGNRELLCGLSDRFRYSED
DVATYMVQLLQGLDYLHGHHVLHLDIKPDNLLLAPDNALKIVDFGSAQPYNPQALRPLGH
RTGTLEFMAPEMVKGEPIGSATDIWGAGVLTYIMLSGRSPFYEPDPQETEARIVGGRFDA
FQLYPNTSQSATLFLRKVLSVHPWSRPSLQDCLAHPWLQDAYLMKLRRQTLTFTTNRLKE
FLGEQRRRRAEAATRHKVLLRSYPGGP
>tr|Q5EBM2|Q5EBM2_HUMAN Ig-like domain-containing protein OS=Homo sapiens OX=9606
PE=1 SV=1
MDCTWRILLLVAVATGTHAQVQLVQSGAEVKKPGASVKVSCEVSGHTLTELSRHWVRQAP
GKGLEWMGGFDPEDGETVYAQTFQGRVTMTEDTSTDTAYMDLSNLRSDDTAVYYCATGVY
DVLTGYSRFDYWGQGTQVTVSSASTKGPSVFPLAPCSRSTSGGTAALGCLVKDYFPEPVT
VSWNSGALTSGVHTFPAVLQSSGLYSLSSVVTVPSSSLGTQTYTCNVNHKPSNTKVDKRV
ELKTPLGDTTHTCPRCPEPKSCDTPPPCPRCPEPKSCDTPPPCPRCPEPKSCDTPPPCPR
CPAPELLGGPSVFLFPPKPKDTLMISRTPEVTCVVVDVSHEDPEVQFKWYVDGVEVHNAK
TKPREEQYNSTFRVVSVLTVLHQDWLNGKEYKCKVSNKALPAPIEKTISKTKGQPREPQV
YTLPPSREEMTKNQVSLTCLVKGFYPSDIAVEWESSGQPENNYNTTPPMLDSDGSFFLYS
KLTVDKSRWQQGNIFSCSVMHEALHNRFTQKSLSLSPGK
>sp|Q9Y2K7|KDM2A_HUMAN Lysine-specific demethylase 2A OS=Homo sapiens OX=9606
GN=KDM2A PE=1 SV=3
MEPEEERIRYSQRLRGTMRRRYEDDGISDDEIEGKRTFDLEEKLHTNKYNANFVTFMEGK
DFNVEYIQRGGLRDPLIFKNSDGLGIKMPDPDFTVNDVKMCVGSRRMVDVMDVNTQKGIE
MTMAQWTRYYETPEEEREKLYNVISLEFSHTRLENMVQRPSTVDFIDWVDNMWPRHLKES
QTESTNAILEMQYPKVQKYCLMSVRGCYTDFHVDFGGTSVWYHIHQGGKVFWLIPPTAHN
LELYENWLLSGKQGDIFLGDRVSDCQRIELKQGYTFVIPSGWIHAVYTPTDTLVFGGNFL
HSFNIPMQLKIYNIEDRTRVPNKFRYPFYYEMCWYVLERYVYCITNRSHLTKEFQKESLS
MDLELNGLESGNGDEEAVDREPRRLSSRRSVLTSPVANGVNLDYDGLGKTCRSLPSLKKT
LAGDSSSDCSRGSHNGQVWDPQCAPRKDRQVHLTHFELEGLRCLVDKLESLPLHKKCVPT
GIEDEDALIADVKILLEELANSDPKLALTGVPIVQWPKRDKLKFPTRPKVRVPTIPITKP
HTMKPAPRLTPVRPAAASPIVSGARRRRVRCRKCKACVQGECGVCHYCRDMKKFGGPGRM
KQSCVLRQCLAPRLPHSVTCSLCGEVDQNEETQDFEKKLMECCICNEIVHPGCLQMDGEG
LLNEELPNCWECPKCYQEDSSEKAQKRKMEESDEEAVQAKVLRPLRSCDEPLTPPPHSPT

SMLQLIHDPVSPRGMVTRSSPGAGPSDHHSASRDERFKRRQLLRLQATERTMVREKENNP
SGKKELSEVEKAKIRGSYLTVTLQRPTKELHGTSIVPKLQAITASSANLRHSPRVLVQHC
PARTPQRGDEEGLGGEEEEEEEEEEEEDDSAEEGGAARLNGRGSWAQDGDESWMQREVWMS
VFRYLSRRELCECMRVCKTWYKWCCDKRLWTKIDLSRCKAIVPQALSGIIKRQPVSLDLS
WTNISKKQLTWLVNRLPGLKDLLLAGCSWSAVSALSTSSCPLLRTLDLRWAVGIKDPQIR
DLLTPPADKPGQDNRSKLRNMTDFRLAGLDITDATLRLIIRHMPLLSRLDLSHCSHLTDQ
SSNLLTAVGSSTRYSLTELNMAGCNKLTDQTLIYLRRIANVTLIDLRGCKQITRKACEHF
ISDLSINSLYCLSDEKLIQKIS
>sp|P22061|PIMT_HUMAN Protein-L-isoaspartate(D-aspartate) O-methyltransferase OS=Homo sapiens OX=9606 GN=PCMT1 PE=1 SV=4
MAWKSGGASHSELIHNLRKNGIIKTDKVFEVMLATDRSHYAKCNPYMDSPQSIGFQATIS
APHMHAYALELLFDQLHEGAKALDVGSGSGILTACFARMVGCTGKVIGIDHIKELVDDSV
NNVRKDDPTLLSSGRVQLVVGDGRMGYAEEAPYDAIHVGAAAPVVPQALIDQLKPGGRLI
LPVGPAGGNQMLEQYDKLQDGSIKMKPLMGVIYVPLTDKEKQWSRWK
>sp|P48147|PPCE_HUMAN Prolyl endopeptidase OS=Homo sapiens OX=9606 GN=PREP PE=1 SV=2
MLSLQYPDVYRDETAVQDYHGHKICDPYAWLEDPDSEQTKAFVEAQNKITVPFLEQCPIR
GLYKERMTELYDYPKYSCHFKKGKRYFYFYNTGLQNQRVLYVQDSLEGEARVFLDPNILS
DDGTVALRGYAFSEDGEYFAYGLSASGSDWVTIKFMKVDGAKELPDVLERVKFSCMAWTH
DGKGMFYNSYPQQDGKSDGTETSTNLHQKLYYHVLGTDQSEDILCAEFPDEPKWMGGAEL
SDDGRYVLLSIREGCDPVNRLWYCDLQQESSGIAGILKWVKLIDNFEGEYDYVTNEGTVF
TFKTNRQSPNYRVINIDFRDPEESKWKVLVPEHEKDVLEWIACVRSNFLVLCYLHDVKNI
LQLHDLTTGALLKTFPLDVGSIVGYSGQKKDTEIFYQFTSFLSPGIIYHCDLTKEELEPR
VFREVTVKGIDASDYQTVQIFYPSKDGTKIPMFIVHKKGIKLDGSHPAFLYGYGGFNISI
TPNYSVSRLIFVRHMGGILAVANIRGGGEYGETWHKGGILANKQNCFDDFQCAAEYLIKE
GYTSPKRLTINGGSNGGLLVAACANQRPDLFGCVIAQVGVMDMLKFHKYTIGHAWTTDYG
CSDSKQHFEWLVKYSPLHNVKLPEADDIQYPSMLLLTADHDDRVVPLHSLKFIATLQYIV
GRSRKQSNPLLIHVDTKAGHGAGKPTAKVIEEVSDMFAFIARCLNVDWIP
>sp|P78371|TCPB_HUMAN T-complex protein 1 subunit beta OS=Homo sapiens OX=9606 GN=CCT2 PE=1 SV=4
MASLSLAPVNIFKAGADEERAETARLTSFIGAIAIGDLVKSTLGPKGMDKILLSSGRDAS
LMVTNDGATILKNIGVDNPAAKVLVDMSRVQDDEVGDGTTSVTVLAAELLREAESLIAKK
IHPQTIIAGWREATKAAREALLSSAVDHGSDEVKFRQDLMNIAGTTLSSKLLTHHKDHFT
KLAVEAVLRLKGSGNLEAIHIIKKLGGSLADSYLDEGFLLDKKIGVNQPKRIENAKILIA
NTGMDTDKIKIFGSRVRVDSTAKVAEIEHAEKEKMKEKVERILKHGINCFINRQLIYNYP
EQLFGAAGVMAIEHADFAGVERLALVTGGEIASTFDHPELVKLGSCKLIEEVMIGEDKLI
HFSGVALGEACTIVLRGATQQILDEAERSLHDALCVLAQTVKDSRTVYGGGCSEMLMAHA
VTQLANRTPGKEAVAMESYAKALRMLPTIIADNAGYDSADLVAQLRAAHSEGNTTAGLDM
REGTIGDMAILGITESFQVKRQVLLSAAEAAEVILRVDNIIKAAPRKRVPDHHPC
>sp|Q13443|ADAM9_HUMAN Disintegrin and metalloproteinase domain-containing protein 9 OS=Homo sapiens OX=9606 GN=ADAM9 PE=1 SV=1
MGSGARFPSGTLRVRWLLLLGLVGPVLGAARPGFQQTSHLSSYEIITPWRLTRERREAPR
PYSKQVSYVIQAEGKEHIIHLERNKDLLPEDFVVYTYNKEGTLITDHPNIQNHCHYRGYV
EGVHNSSIALSDCFGLRGLLHLENASYGIEPLQNSSHFEHIIYRMDDVYKEPLKCGVSNK

DIEKETAKDEEEEPPSMTQLLRRRRAVLPQTRYVELFIVVDKERYDMMGRNQTAVREEMI
LLANYLDSMYIMLNIRIVLVGLEIWTNGNLINIVGGAGDVLGNFVQWREKFLITRRRHDS
AQLVLKKGFGGTAGMAFVGTVCSRSHAGGINVFGQITVETFASIVAHELGHNLGMNHDDG
RDCSCGAKSCIMNSGASGSRNFSSCSAEDFEKLTLNKGGNCLLNIPKPDEAYSAPSCGNK
LVDAGEECDCGTPKECELDPCCEGSTCKLKSFAECAYGDCCKDCRFLPGGTLCRGKTSEC
DVPEYCNGSSQFCQPDVFIQNGYPCQNNKAYCYNGMCQYYDAQCQVIFGSKAKAAPKDCF
IEVNSKGDRFGNCGFSGNEYKKCATGNALCGKLQCENVQEIPVFGIVPAIIQTPSRGTKC
WGVDFQLGSDVPDPGMVNEGTKCGAGKICRNFQCVDASVLNYDCDVQKKCHGHGVCNSNK
NCHCENGWAPPNCETKGYGGSVDSGPTYNEMNTALRDGLLVFFFLIVPLIVCAIFIFIKR
DQLWRSYFRKKRSQTYESDGKNQANPSRQPGSVPRHVSPVTPPREVPIYANRFAVPTYAA
KQPQQFPSRPPPPQPKVSSQGNLIPARPAPAPPLYSSLT
>sp|Q96FW1|OTUB1_HUMAN Ubiquitin thioesterase OTUB1 OS=Homo sapiens OX=9606
GN=OTUB1 PE=1 SV=2
MAAEEPQQQKQEPLGSDSEGVNCLAYDEAIMAQQDRIQQEIAVQNPLVSERLELSVLYKE
YAEDDNIYQQKIKDLHKKYSYIRKTRPDGNCFYRAFGFSHLEALLDDSKELQRFKAVSAK
SKEDLVSQGFTEFTIEDFHNTFMDLIEQVEKQTSVADLLASFNDQSTSDYLVVYLRLLTS
GYLQRESKFFEHFIEGGRTVKEFCQQEVEPMCKESDHIHIIALAQALSVSIQVEYMDRGE
GGTTNPHIFPEGSEPKVYLLYRPGHYDILYK
>sp|Q9NQ79|CRAC1_HUMAN Cartilage acidic protein 1 OS=Homo sapiens OX=9606
GN=CRTAC1 PE=1 SV=2
MAPSADPGMSRMLPFLLLLWFLPITEGSQRAEPMFTAVTNSVLPPDYDSNPTQLNYGVAV
TDVDHDGDFEIVVAGYNGPNLVLKYDRAQKRLVNIAVDERSSPYYALRDRQGNAIGVTAC
DIDGDGREEIYFLNTNNAFSGVATYTDKLFKFRNNRWEDILSDEVNVARGVASLFAGRSV
ACVDRKGSGRYSIYIANYAYGNVGPDALIEMDPEASDLSRGILALRDVAAEAGVSKYTGG
RGVSVGPILSSSASDIFCDNENGPNFLFHNRGDGTFVDAAASAGVDDPHQHGRGVALADF
NRDGKVDIVYGNWNGPHRLYLQMSTHGKVRFRDIASPKFSMPSPVRTVITADFDNDQELE
IFFNNIAYRSSSANRLFRVIRREHGDPLIEELNPGDALEPEGRGTGGVVTDFDGDGMLDL
ILSHGESMAQPLSVFRGNQGFNNNWLRVVPRTRFGAFARGAKVVLYTKKSGAHLRIIDGG
SGYLCEMEPVAHFGLGKDEASSVEVTWPDGKMVSRNVASGEMNSVLEILYPRDEDTLQDP
APLECGQGFSQQENGHCMDTNECIQFPFVCPRDKPVCVNTYGSYRCRTNKKCSRGYEPNE
DGTACVGTLGQSPGPRPTTPTAAAATAAAAAAAGAATAAPVLVDGDLNLGSVVKESCEPS
C
>sp|Q9UHN6|CEIP2_HUMAN Inactive cell surface hyaluronidase CEMIP2 OS=Homo sapiens
OX=9606 GN=CEMIP2 PE=1 SV=1
MYATDSRGHSPAFLQPQNGNSRHPSGYVPGKVVPLRPPPPPKSQASAKFTSIRREDRATF
AFSPEEQQAQRESQKQKRHKNTFICFAITSFSFFIALAIILGISSKYAPDENCPDQNPRL
RNWDPGQDSAKQVVIKEGDMLRLTSDATVHSIVIQDGGLLVFGDNKDGSRNITLRTHYIL
IQDGGALHIGAEKCRYKSKATITLYGKSDEGESMPTFGKKFIGVEAGGTLELHGARKASW
TLLARTLNSSGLPFGSYTFEKDFSRGLNVRVIDQDTAKILESERFDTHEYRNESRRLQEF
LRFQDPGRIVAIAVGDSAAKSLLQGTIQMIQERLGSELIQGLGYRQAWALVGVIDGGSTS
CNESVRNYENHSSGGKALAQREFYTVDGQKFSVTAYSEWIEGVSLSGFRVEVVDGVKLNL
LDDVSSWKPGDQIVVASTDYSMYQAEEFTLLPCSECSHFQVKVKETPQFLHMGEIIDGVD
MRAEVGILTRNIVIQGEVEDSCYAENQCQFFDYDTFGGHIMIMKNFTSVHLSYVELKHMG
QQQMGRYPVHFHLCGDVDYKGGYRHATFVDGLSIHHSFSRCITVHGTNGLLIKDTIGFDT

LGHCFFLEDGIEQRNTLFHNLGLLTKPGTLLPTDRNNSMCTTMRDKVFGNYIPVPATDCM
AVSTFWIAHPNNNLINNAAAGSQDAGIWYLFHKEPTGESSGLQLLAKPELTPLGIFYNNR
VHSNFKAGLFIDKGVKTTNSSAADPREYLCLDNSARFRPHQDANPEKPRVAALIDRLIAF
KNNDNGAWVRGGDIIVQNSAFADNGIGLTFASDGSFPSDEGSSQEVSESLFVGESRNYGF
QGGQNKYVGTGGIDQKPRTLPRNRTFPIRGFQIYDGPIHLTRSTFKKYVPTPDRYSSAIG
FLMKNSWQITPRNNISLVKFGPHVSLNVFFGKPGPWFEDCEMDGDKNSIFHDIDGSVTGY
KDAYVGRMDNYLIRHPSCVNVSKWNAVICSGTYAQVYVQTWSTQNLSMTITRDEYPSNPM
VLRGINQKAAFPQYQPVVMLEKGYTIHWNGPAPRTTFLYLVNFNKNDWIRVGLCYPSNTS
FQVTFGYLQRQNGSLSKIEEYEPVHSLEELQRKQSERKFYFDSSTGLLFLYLKAKSHRHG
HSYCSSQGCERVKIQAATDSKDISNCMAKAYPQYYRKPSVVKRMPAMLTGLCQGCGTRQV
VFTSDPHKSYLPVQFQSPDKAETQRGDPSVISVNGTDFTFRSAGVLLLVVDPCSVPFRLT
EKTVFPLADVSRIEEYLKTGIPPRSIVLLSTRGEIKQLNISHLLVPLGLAKPAHLYDKGS
TIFLGFSGNFKPSWTKLFTSPAGQGLGVLEQFIPLQLDEYGCPRATTVRRRDLELLKQAS
KAH
>sp|A1L0T0|HACL2_HUMAN 2-hydroxyacyl-CoA lyase 2 OS=Homo sapiens OX=9606 GN=ILVBL
PE=1 SV=2
METPAAAAPAGSLFPSFLLLACGTLVAALLGAAHRLGLFYQLLHKVDKASVRHGGENVAA
VLRAHGVRFIFTLVGGHISPLLVACEKLGIRVVDTRHEVTAVFAADAMARLSGTVGVAAV
TAGPGLTNTVTAVKNAQMAQSPILLLGGAASTLLQNRGALQAVDQLSLFRPLCKFCVSVR
RVRDIVPTLRAAMAAAQSGTPGPVFVELPVDVLYPYFMVQKEMVPAKPPKGLVGRVVSWY
LENYLANLFAGAWEPQPEGPLPLDIPQASPQQVQRCVEILSRAKRPLMVLGSQALLTPTS
ADKLRAAVETLGVPCFLGGMARGLLGRNHPLHIRENRSAALKKADVIVLAGTVCDFRLSY
GRVLSHSSKIIIVNRNREEMLLNSDIFWKPQEAVQGDVGSFVLKLVEGLQGQTWAPDWVE
ELREADRQKEQTFREKAAMPVAQHLNPVQVLQLVEETLPDNSILVVDGGDFVGTAAHLVQ
PRGPLRWLDPGAFGTLGVGAGFALGAKLCRPDAEVWCLFGDGAFGYSLIEFDTFVRHKIP
VMALVGNDAGWTQISREQVPSLGSNVACGLAYTDYHKAAMGLGARGLLLSRENEDQVVKV
LHDAQQQCRDGHPVVVNILIGRTDFRDGSIAV
>tr|O75546|O75546_HUMAN small monomeric GTPase (Fragment) OS=Homo sapiens
OX=9606 PE=2 SV=1
NHLTTWLEDARQHSNSNMVIMLIGNKSDLESRREVKKEEGEAFAREHGLIFMETSAKTAF
NVEEAFINTAKEIYEKIQEGVFDINNEANGIKIGPQHAATNATHAGNQGGQQAGGGCC
>sp|O94956|SO2B1_HUMAN Solute carrier organic anion transporter family member 2B1
OS=Homo sapiens OX=9606 GN=SLCO2B1 PE=1 SV=3
MGPRIGPAGEVPQVPDKETKATMGTENTPGGKASPDPQDVRPSVFHNIKLFVLCHSLLQL
AQLMISGYLKSSISTVEKRFGLSSQTSGLLASFNEVGNTALIVFVSYFGSRVHRPRMIGY
GAILVALAGLLMTLPHFISEPYRYDNTSPEDMPQDFKASLCLPTTSAPASAPSNGNCSSY
TETQHLSVVGIMFVAQTLLGVGGVPIQPFGISYIDDFAHNSNSPLYLGILFAVTMMGPGL
AFGLGSLMLRLYVDINQMPEGGISLTIKDPRWVGAWWLGFLIAAGAVALAAIPYFFFPKE
MPKEKRELQFRRKVLAVTDSPARKGKDSPSKQSPGESTKKQDGLVQIAPNLTVIQFIKVF
PRVLLQTLRHPIFLLVVLSQVCLSSMAAGMATFLPKFLERQFSITASYANLLIGCLSFPS
VIVGIVVGGVLVKRLHLGPVGCGALCLLGMLLCLFFSLPLFFIGCSSHQIAGITHQTSAH
PGLELSPSCMEACSCPLDGFNPVCDPSTRVEYITPCHAGCSSWVVQDALDNSQVFYTNCS
CVVEGNPVLAGSCDSTCSHLVVPFLLLVSLGSALACLTHTPSFMLILRGVKKEDKTLAVG
IQFMFLRILAWMPSPVIHGSAIDTTCVHWALSCGRRAVCRYYNNDLLRNRFIGLQFFFKT

GSVICFALVLAVLRQQDKEARTKESRSSPAVEQQLLVSGPGKKPEDSRV
