# Supplementary material for: Effects of Aging on Intrinsic Protein Disorder in Human Lenses and Zonules
Source: Cell Biochem Biophys. 2024 Aug 8;82(4):3667–79. doi: 10.1007/s12013-024-01455-x (PMC11576620; doi:10.1007/s12013-024-01455-x)
Supplement: Supplementary file 4 — Tukey HSD S1 Supplementary [file 12013_2024_1455_MOESM4_ESM.pdf]

Supplemental Table S1. Pairwise comparisons of lens age and zonules of various disorder predictors. The table presents the results of a statistical analysis comparing the means of different lens ages and zonular parameters through Tukey's honestly significant difference test, with family-wise error rates set at 0.05.

Pairwise comparisons for PER(VLXT):

Multiple Comparison of Means - Tukey HSD, FWER=0.05

| group1 | group2 | meandiff | p-adj | lower | upper | reject |
|---|---|---|---|---|---|---|
| 25/37-year-old Lens | 58-year-old Lens | -3.1991 | 0.0178 | -5.9523 | -0.4459 | True |
| 25/37-year-old Lens | Zonules | 1.2932 | 0.4951 | -1.3895 | 3.9759 | False |
| 58-year-old Lens | Zonules | 4.4923 | 0.0053 | 1.1088 | 7.8758 | True |

Pairwise comparisons for RNK(VLXT):

Multiple Comparison of Means - Tukey HSD, FWER=0.05

| group1 | group2 | meandiff | p-adj | lower | upper | reject |
|---|---|---|---|---|---|---|
| 25/37-year-old Lens | 58-year-old Lens | -0.027 | 0.0118 | -0.0491 | -0.0049 | True |
| 25/37-year-old Lens | Zonules | 0.0164 | 0.1762 | -0.0052 | 0.0379 | False |
| 58-year-old Lens | Zonules | 0.0434 | 0.0006 | 0.0162 | 0.0706 | True |

Pairwise comparisons for PER(VSL2B):

Multiple Comparison of Means - Tukey HSD, FWER=0.05

| group1 | group2 | meandiff | p-adj | lower | upper | reject |
|---|---|---|---|---|---|---|
| 25/37-year-old Lens | 58-year-old Lens | -3.6375 | 0.0349 | -7.0721 | -0.2029 | True |
| 25/37-year-old Lens | Zonules | 8.2083 | 0.0 | 4.8616 | 11.555 | True |
| 58-year-old Lens | Zonules | 11.8458 | 0.0 | 7.6249 | 16.0667 | True |

Pairwise comparisons for RNK(VSL2B):

Multiple Comparison of Means - Tukey HSD, FWER=0.05

| group1 | group2 | meandiff | p-adj | lower | upper | reject |
|---|---|---|---|---|---|---|
| 25/37-year-old Lens | 58-year-old Lens | -0.0237 | 0.0377 | -0.0463 | -0.0011 | True |
| 25/37-year-old Lens | Zonules | 0.0563 | 0.0 | 0.0343 | 0.0783 | True |
| 58-year-old Lens | Zonules | 0.08 | 0.0 | 0.0522 | 0.1078 | True |

Pairwise comparisons for PER(VL3):

Multiple Comparison of Means - Tukey HSD, FWER=0.05

| group1 | group2 | meandiff | p-adj | lower | upper | reject |
|---|---|---|---|---|---|---|
| 25/37-year-old Lens | 58-year-old Lens | -4.942 | 0.0088 | -8.8614 | -1.0226 | True |
| 25/37-year-old Lens | Zonules | 8.0078 | 0.0 | 4.1887 | 11.8269 | True |
| 58-year-old Lens | Zonules | 12.9498 | 0.0 | 8.1331 | 17.7665 | True |

```
Pairwise comparisons for RNK(VL3):
          Multiple Comparison of Means - Tukey HSD, FWER=0.05
==========================================================================
      group1            group2      meandiff p-adj  lower   upper  reject
--------------------------------------------------------------------------
25/37-year-old Lens 58-year-old Lens  -0.0357 0.004 -0.0618 -0.0095   True
25/37-year-old Lens          Zonules   0.0566   0.0  0.0311  0.0821   True
   58-year-old Lens          Zonules   0.0922   0.0  0.0601  0.1244   True
--------------------------------------------------------------------------

Pairwise comparisons for PER(IUP_S):
          Multiple Comparison of Means - Tukey HSD, FWER=0.05
==========================================================================
      group1            group2      meandiff p-adj   lower  upper  reject
--------------------------------------------------------------------------
25/37-year-old Lens 58-year-old Lens   -2.398 0.0563 -4.8455 0.0495  False
25/37-year-old Lens          Zonules   2.4513 0.0423  0.0664 4.8362   True
   58-year-old Lens          Zonules   4.8493 0.0005  1.8415 7.8571   True
--------------------------------------------------------------------------

Pairwise comparisons for RNK(IUP_S):
          Multiple Comparison of Means - Tukey HSD, FWER=0.05
==========================================================================
      group1            group2      meandiff p-adj   lower  upper  reject
--------------------------------------------------------------------------
25/37-year-old Lens 58-year-old Lens  -0.0126 0.1955 -0.0297 0.0045  False
25/37-year-old Lens          Zonules   0.0307    0.0  0.0141 0.0474   True
   58-year-old Lens          Zonules   0.0433    0.0  0.0223 0.0643   True
--------------------------------------------------------------------------

Pairwise comparisons for PER(IUP_L):
          Multiple Comparison of Means - Tukey HSD, FWER=0.05
==========================================================================
      group1            group2      meandiff p-adj   lower  upper  reject
--------------------------------------------------------------------------
25/37-year-old Lens 58-year-old Lens   -2.523 0.1276 -5.5722 0.5261  False
25/37-year-old Lens          Zonules   3.6511 0.0111    0.68 6.6222   True
   58-year-old Lens          Zonules   6.1741 0.0003  2.4269 9.9213   True
--------------------------------------------------------------------------

Pairwise comparisons for RNK(IUP_L):
          Multiple Comparison of Means - Tukey HSD, FWER=0.05
=========================================================================
      group1            group2      meandiff p-adj  lower  upper  reject
-------------------------------------------------------------------------
25/37-year-old Lens 58-year-old Lens  -0.0151 0.252 -0.0375 0.0072  False
25/37-year-old Lens          Zonules   0.0428   0.0   0.021 0.0646   True
   58-year-old Lens          Zonules    0.058   0.0  0.0305 0.0855   True
-------------------------------------------------------------------------

Pairwise comparisons for PER(PFIT):
           Multiple Comparison of Means - Tukey HSD, FWER=0.05
===========================================================================
      group1            group2      meandiff p-adj   lower   upper  reject
---------------------------------------------------------------------------
25/37-year-old Lens 58-year-old Lens   -4.214 0.0036  -7.274  -1.154   True
25/37-year-old Lens          Zonules   2.9453 0.0537 -0.0364   5.927  False
```

```
   58-year-old Lens          Zonules   7.1594    0.0  3.3988 10.9199   True
---------------------------------------------------------------------------

Pairwise comparisons for RNK(PFIT):
            Multiple Comparison of Means - Tukey HSD, FWER=0.05
===========================================================================
       group1            group2      meandiff p-adj   lower   upper  reject
---------------------------------------------------------------------------
25/37-year-old Lens 58-year-old Lens  -0.0284  0.007 -0.0503 -0.0064   True
25/37-year-old Lens          Zonules   0.0308 0.0022  0.0094  0.0522   True
   58-year-old Lens          Zonules   0.0592    0.0  0.0322  0.0862   True
---------------------------------------------------------------------------

Pairwise comparisons for PER(MDP):
            Multiple Comparison of Means - Tukey HSD, FWER=0.05
===========================================================================
       group1            group2      meandiff p-adj   lower   upper  reject
---------------------------------------------------------------------------
25/37-year-old Lens 58-year-old Lens  -4.0604 0.0169 -7.5317 -0.5891   True
25/37-year-old Lens          Zonules   3.7315 0.0263   0.349   7.114   True
   58-year-old Lens          Zonules   7.7919 0.0001  3.5258 12.0579   True
---------------------------------------------------------------------------

Pairwise comparisons for RNK(MDP):
           Multiple Comparison of Means - Tukey HSD, FWER=0.05
==========================================================================
       group1            group2      meandiff p-adj  lower   upper  reject
--------------------------------------------------------------------------
25/37-year-old Lens 58-year-old Lens  -0.0261 0.018 -0.0487 -0.0036   True
25/37-year-old Lens          Zonules   0.0415   0.0  0.0195  0.0634   True
   58-year-old Lens          Zonules   0.0676   0.0  0.0399  0.0953   True
--------------------------------------------------------------------------

Pairwise comparisons for dCDF:
            Multiple Comparison of Means - Tukey HSD, FWER=0.05
===========================================================================
       group1            group2      meandiff p-adj   lower   upper  reject
---------------------------------------------------------------------------
25/37-year-old Lens 58-year-old Lens   0.0266 0.0114  0.0049  0.0484   True
25/37-year-old Lens          Zonules  -0.0155 0.1996 -0.0367  0.0057  False
   58-year-old Lens          Zonules  -0.0421 0.0007 -0.0688 -0.0154   True
---------------------------------------------------------------------------

Pairwise comparisons for dCH:
            Multiple Comparison of Means - Tukey HSD, FWER=0.05
===========================================================================
       group1            group2      meandiff p-adj   lower   upper  reject
---------------------------------------------------------------------------
25/37-year-old Lens 58-year-old Lens  -0.0251 0.0039 -0.0434 -0.0067   True
25/37-year-old Lens          Zonules   0.0226 0.0084  0.0048  0.0405   True
   58-year-old Lens          Zonules   0.0477    0.0  0.0252  0.0702   True
---------------------------------------------------------------------------
```
